# Efficacy of personal protective equipment to prevent environmental infection of COVID-19 among healthcare workers: a systematic review

**DOI:** 10.1265/ehpm.22-00131

**Published:** 2023-01-07

**Authors:** Sani Rachman Soleman, Zhaoqing Lyu, Takuya Okada, Mariko Harada Sassa, Yukiko Fujii, Manal A.M. Mahmoud, Daniel K Ebner, Kouji H. Harada

**Affiliations:** 1 Department of Health and Environmental Science, Kyoto University Graduate School of Medicine, Yoshida Konoe, Sakyo, Kyoto 606-8501, Japan; 2 Department of Public Health, Faculty of Medicine, Universitas Islam Indonesia, Yogyakarta 55584, Indonesia; 3 Daiichi University of Pharmacy, Fukuoka 8158511, Japan; 4 Faculty of Veterinary Medicine, Assiut University, 71526 Egypt; 5 Department of Radiation Oncology, Mayo Clinic, Rochester MN 55905, United States of America; 6 QST Hospital, National Institutes of Quantum Science and Technology, Chiba, Japan

**Keywords:** Personal protective equipment, COVID-19, Environmental infection, Healthcare workers, Facial masks

## Abstract

**Background:**

Healthcare workers (HCWs) employed personal protective equipment (PPE) during the COVID-19 pandemic, crucial to protecting themselves from infection. To highlight the efficacy of PPE in preventing environmental infection among HCWs, a systematic review was conducted in line with PRISMA guidance.

**Methods:**

A search of the PubMed and Web of Science databases was conducted from January 2019 to April 2021 using pre-defined search terms. Articles were screened by three researchers. The approved papers were read in full and included in this review if relevance was mutually agreed upon. Data were extracted by study design and types of PPEs.

**Results:**

47 of 108 identified studies met the inclusion criteria, with seven reviews and meta-analyses, seven cohort, nine case-control, fifteen cross-sectional studies, four before and after, four case series, and one modeling studies. Wearing PPE offered COVID-19 protection in HCWs but required adequate training. Wearing surgical masks provided improved protection over cloth masks, while the benefit of powered air-purifying respirators is less clear, as are individual gowns, gloves, and/or face shields.

**Conclusions:**

Wearing PPE, especially facial masks, is necessary among HCWs, while training in proper use of PPE is also important to prevent COVID-19 infection.

**Supplementary information:**

The online version contains supplementary material available at https://doi.org/10.1265/ehpm.22-00131.

## Introduction

COVID-19, the infection caused by the SARS-CoV-2 coronavirus, has caused an unprecedented strain on the global healthcare system, particularly with regard to those healthcare workers (HCWs) responsible for managing the pandemic. HCWs, particularly those operating within hospital systems and with direct patient care, have been at high risk for environmental infection, necessitating systemic protective measures to reduce risk. Bandyopadhyay *et al.* reported that as of 2020, 152,888 HCWs had been infected over the course of the pandemic across the world, while 525 physicians had died from the disease within six months of the start of the pandemic, followed by nurses and allied health professionals at 259 and 125, respectively [1]. Heneghan *et al.* estimated daily infection in HCWs increased 9.1% per day in the United Kingdom between March 21 and April 15, 2020 [2].

High rates of infection among HCWs in the early pandemic are believed to be due to personal protective equipment (PPE) shortages during the first wave of the disease, with subsequent decline in infection rate noted following universal masking implementation [3]. Subsequent rollout of PPE has been correlated with improvement in protection, supported by the work by Chatterje, Boffetta and their colleagues [4, 5]. Nonetheless, the number of infections among HCWs has continued to increase both in the general population and in HCWs.

However, though PPE has been an accepted part of infection transmission prevention for HCWs since the onset of the pandemic, the value of individual pieces of PPE toward overall protection are an evolving area of study. Chatterje *et al.* [4] have demonstrated that while masks, caps, gowns, and gloves offer significant protection, shoe covers and face shields appear not to protect HCWs in the clinical setting. Similarly, Boffetta *et al.*’s evaluation [5] supports the use of medical masks and gloves, but suggests poor value to filtering facepieces, face shields, and gowns. Of significant concern for HCWs, Bartoszko and colleagues have suggested that N95 masking, a key piece of PPE during the pandemic, offers little benefit over standard medical masks on evaluation of past respiratory disease epidemics [6].

As a recommendation of the World Health Organization (WHO), healthcare workers must wear medical masks [7] during the pandemic. The United States Centers for Disease Control and Prevention (US CDC) suggested the use of respirators [8] during routine care of COVID-19 patients. Per analysis by MacIntyre and Chungtai, there is evidence of respirator efficacy if worn frequently during work in a healthcare facility; however, medical masks were found to be ineffective, and cloth masks were comparatively less effective than medical masks [9]. Cloth masks exhibit poor filtration, moisture retention, and are generally reused, increasing the risk of infection in both the community and in healthcare settings. Consequently, the WHO did not recommend cloth masks in clinical settings, particularly for highly infectious procedures. Tian *et al.* reported that wearing surgical masks, N95, gowns, gloves, and face protectors provides robust protection to every respiratory virus, including SARS-CoV-2 among HCW [10]. However, Heinzerling *et al.* have underscored that wearing gloves and face masks during Aerosol-Generating Procedures (AGP) carries a high risk for COVID-19 infection; notably, the study described teams predominantly wearing gloves and face masks, but removing masks while speaking [11]. The limited protective efficacy of gloves is concerning, as regular use of gloves may lead to false sense of personal protection with consequent hand-to-face contact as a source of nosocomial infection.

Given proximity to ill patients, HCWs are a vulnerable population uniquely at risk from the COVID-19 pandemic, and so strong preventative measures are necessary. There have been reviews focusing on healthcare workers involved in a specific position, for example in emergency trauma surgery [12], or investigating only a single type of PPE like face masks [13]. Although those reviews provided evidence that PPE use, especially face masks, could protect HCWs from infection, we here conduct a systematic review comprehensively assessing the efficacy of multiple forms of PPE among various HCWs, with analysis of the personal protective methodologies employed by healthcare workers and healthcare systems during the pandemic.

## Methods

### Data source

This review aims to identify and summarize available scientific studies on the protective effects of PPEs employed during the COVID-19 pandemic. Published, peer-reviewed studies were identified from the MEDLINE (PubMed) and Web of Science (WoS) electronic databases with no language restrictions. Given the timeframe of the COVID-19 pandemic, this encompassed January 2019 to April 23, 2021. In addition to publications identified via database search, references contained in the identified literature were also reviewed. The search terms used in the MEDLINE and WoS searches are shown in Table 1. To avoid the omission of potentially qualified papers, terms likely to result in a wider range of papers were intentionally used.

**Table 1:** Search strategy and terms.

**Database: Pubmed (MEDLINE), Web of Science**
PubMed search terms: Search: (healthcare workers[Title/Abstract] OR healthcare worker[Title/Abstract] OR health personnel[Title/Abstract] OR health personnels[Title/Abstract] OR health professionals[Title/Abstract] OR nosocomial[Title/Abstract]) AND (COVID[Title/Abstract] OR SARS-CoV-2[Title/Abstract]) AND (protection)
Web of Science search terms: (((TI=(healthcare workers) OR TI=(healthcare worker) OR TI=(health personnel) OR TI=(health personnel) OR TI=(health professionals) OR TI=(nosocomial) OR AB=(healthcare workers) OR AB=(healthcare worker) OR AB=(health personnel) OR AB=(health personnel) OR AB=(health professionals) OR AB=(nosocomial)) AND (TI=(COVID) OR AB=(COVID) OR TI=(SARS-CoV-2) OR AB=(SARS-CoV-2)) AND TS=(protection))) AND DOP=(2019-01-01/2021-04-23)

### Eligibility criteria

Original papers reporting on the risk of transmission of COVID-19 in HCWs using PPE were eligible. Expert or editorial opinions and studies without original content were excluded by direct screening of titles and abstracts. Appropriate studies in languages other than English were included. Study designs were not restricted. Case reports and conference proceedings were not included in the review unless their content were accessible to the reviewers.

### Literature screening

The titles and abstracts of papers identified in the initial search were screened by three researchers (ZL, SRS, and TO) independently. To maximize the number of eligible papers, any paper approved by any individual researcher was included. The approved papers were read in full by each of the three researchers, and those papers mutually agreed upon as relevant were included. When unclear, the researchers endeavored to contact the original author.

### Data coding and quality assessment

Data was extracted and coded based on author information, journal name, year of publication, design, target population, type of PPE, intervention or exposure, outcomes, results, author-drawn conclusions and funding sources. The extraction was conducted by three authors, SRS, ZL and TO, using EndNote 20.4.1 software. Data extraction was conducted independently, then each of the three authors reviewed all included studies and formed a consensus on the data to extract. The data were transferred into Microsoft Excel for further assessment. Quality assessment was conducted for cohort studies and case-control studies using A Cochrane Risk Of Bias Assessment Tool for Non-Randomized Studies of Interventions (ACROBAT-NRSI) [14]. Quality of the included systematic reviews was assessed using Grading of Recommendations, Assessment, Development and Evaluations (GRADE) system [15]. The assessment was conducted by two authors (SRS and ZL) independently. The results were reviewed and agreed by three authors SRS, ZL and TO.

## Results

### Literature search and screening

**Fig. 1:**
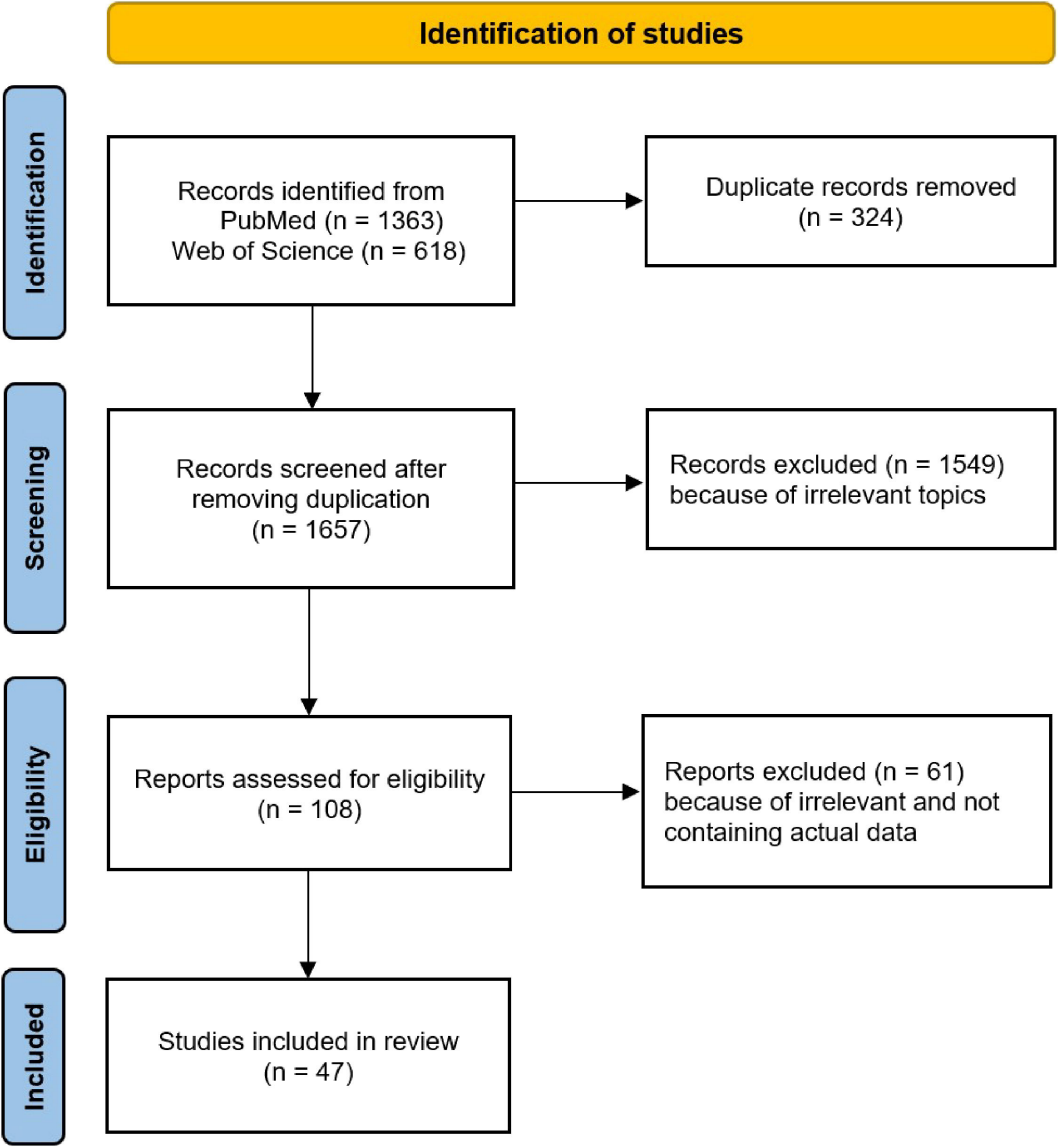
Screening process of evaluated publications.

The approach taken for article screening may be viewed in Fig. 1. In total, 1363 articles were identified via PubMed, and 618 articles were found via Web of Science, using search terms listed in Table 1. All of the identified papers were listed in the Appendix document. On review of titles and abstracts, 1549 papers were excluded, leaving 108 papers for secondary screening. 47 studies met the preset inclusion criteria, containing seven reviews and meta-analyses, seven cohort studies, nine case-control, fifteen cross-sectional studies, four before and after, four case series, and one modeling. A list of the 47 studies and their summary were provided in Table S1.

### Studies related to overall PPE use

Studies on the efficacy of overall PPEs are summarized in Table S2. One systematic review and meta-analysis by Gholami *et al.* focused specifically on evidence in preventing COVID-19 [16]. On analysis, they concluded on review of several observational studies [17–19] that reused PPE (Hazard ratio (HR) = 5.06, 95% confidence interval (CI): 3.9–6.57), inadequate PPE (HR = 5.91, 95% CI = 4.53–7.71), improper PPE use (Relative risk (RR) = 2.82, 95% CI: 1.11–7.18), and suboptimal hand hygiene after contact with patients (RR = 2.43, 95% CI: 1.34–4.39) were all risk factors for COVID-19 infection [16]. A before-after comparing study [20] reported a reduction in cases of COVID-19 in HCWs after implementing comprehensive intervention including adequate provision and proper use of PPEs. The occurrence of COVID-19 infection declined to 0.19% after intervention (*P* < 0.0001) [20].

In contrast, a cohort study by El-Boghdadly *et al.* suggested no significant protective effect of the WHO standard PPE use for COVID-19 infection (HR = 0.97, 95% CI: 0.63–1.51) [21]. However, one case-control study and three cohort studies reinforced the idea that improper or inadequate PPE use could be independent risk factors for infection [17–19, 22]. Wee *et al.* focused on ancillary HCWs in particular, discovering a lower PPE adherence and higher infection rate than other HCWs [23].

A longitudinal point-prevalence study by Fletcher et al. suggested no significant protection from appropriate PPE wearing (*P* = 0.76), particularly wearing N95 (*P* = 0.897) [24]. Other studies regarding non-specific overall PPE use provided less evidence on efficacy, though some further highlighted PPE inadequacy [25], identification of glove contamination in 15.4% of HCWs [26], and challenges related to compliance [27, 28]. One case-control study suggested that appropriate use of PPE during exposure offered protection in HCWs (Odds ratio (OR) = 0.65, 95% CI: 0.55–0.77) [29]. In general, evidence suggests that wearing reused, improper, and inadequate PPE is a risk factor of COVID-19 in HCW.

### Facial masks and other protective respiratory equipment

The individual effectiveness of PPE is summarized in Table S3. Two systematic reviews [30, 31] and three systematic review and meta-analyses [10, 32, 33] demonstrate that wearing facial masks and respiratory protection equipment are protective. Nevertheless, these reviews included limited studies on COVID-19 cases, and generally incorporated evaluated data regarding related other diseases such as Middle East Respiratory Syndrome (MERS), Severe Acute Respiratory Syndrome (SARS, caused by SARS-CoV-1), and other respiratory viruses. Chu *et al.* suggested that N95 or similar respirators were more protective than other masks, such as disposable medical masks or reusable multilayer cotton masks (OR = 0.34, 95% CI: 0.26–0.45) [32]. In a systematic review by Licina *et al.*, powered air-purifying respirators (PAPR) were found to offer no decrease in COVID-19 infection rates in observational studies of airway proceduralists utilizing PAPR versus other protective respiratory equipment [31]. Additionally, combination interventions such as wearing both a face mask and a face shield were found to be more effective than either alone at preventing bioaerosol inhalation [31]. Abboah-Offei *et al.* conducted a rapid review of the literature on face masks, and they also concluded that face masks demonstrate good efficacy in preventing respiratory virus transmission including COVID-19 [30]. These reviews emphasized the efficacy of using face masks in preventing airborne infections including COVID-19 infection, however, the direct evidence for COVID-19 was limited.

Guo *et al.* conducted a case-control study and found facial masks were protective (OR = 0.15, 95% CI: 0.04–0.55) [19]. Another case-control study by Wang X *et al.* specifically evaluating COVID-19 showed that N95 usage might have a protective effect (OR = 0.04) [34]. Sims *et al.* suggested in a retrospective cohort study that wearing an N95 respirator or PAPR had a significantly lower seropositivity rate (10.2%) compared to surgical/other masks (13.1%) or no mask (17.5%) [35].

Two cross-sectional [36, 37], one case series [38], one case-control [34], and one prospective observational study [39] showed no positive PCR results for COVID-19 in HCWs who used facial masks (N95 or FFP2/3). Liu M et al. reported that wearing N95 and surgical masks coupled with hand hygiene and donning and doffing training for AGP was associated with negative testing for COVID-19 infection [36]. Similarly, Oksanen et al. reported that HCWs wearing N95 in concert with gloves, long-sleeved gowns, and eye protection were free from COVID-19 infection, whereas wearing surgical masks alone may have contributed to 9 of 13 HCWs in the study eventually testing positive [39]. Further, one symptom monitoring registry and one multicenter cross-sectional study demonstrated no significant protection from PPEs other than facial masks (OR = 1.396, 95% CI: 0.303–6.423), single-use gloves (OR = 1.013, 95% CI: 0.382–2.682), face-shield/goggles (OR = 0.437; 95% CI: 0.228–0.837), disposable gowns (OR = 1.083, 95% CI: 0.533–2.203), water-proof aprons (*P* = 0.06, OR = 0.498, 95% CI: 0.239–1.034), general use of both reusable and non-reusable gloves (*P* = 0.7, OR = 0.621, 95% CI: 0.133–2.899), and eye protection (*P* = 0.397, OR = 0.701, 95% CI: 0.310–1.593), particularly during AGP [40, 41].

Two studies compared COVID-19 incidence rates before and after the implementation of universal masking [3, 42]. Prior to the intervention phase, the 7-day COVID-19 incidence was increasing with an overlapping slope (0.96, 95% CI: 0.80–1.31), while during the intervention phase the slope was negative (−0.68, 95% CI: −1.06 to −0.31) [42]. Similarly, Wang et al. reported that during the pre-intervention phase, COVID-19 incidence increased sharply from 0% to 21.3%, and during intervention, positivity rate decreased from 14.65% to 11.46% with a net slope change of 1.65% (95% CI: 1.13% to 2.15%; *P* < 0.001) [3]. Both studies suggested that adequate mask use contributed to decreasing incidence rate of COVID-19. Only one cross-sectional study reported that the use of high-level PPE (FFP2 mask or equivalent and eye protection) by emergency department (ED) personnel did not lower the infection rate of ED staff [43].

In summary, facial masks including N95 respirators, surgical masks, or a combination of facial respirators and masks were consistently noted as protective to some degree for COVID-19 in HCWs [4, 5, 11, 19, 29, 34, 40, 41, 44–47].

### Gloves, gowns, face shields and other PPEs

There was a limited number of publications regarding the individual efficacy of gloves, gowns, and face shields. A systematic review and meta-analysis by Tian *et al.* suggested that gloves (OR = 0.48), gowns (OR = 0.46), surgical masks (OR = 0.37), N95 respirators (OR = 0.32), face shields (OR = 0.41), and infection prevention training were found to be individually highly effective in preventing infection, and that PPEs could provide more robust protection in combination with hand hygiene (OR = 0.54) and infection prevention training (OR = 0.24) [10]. Similarly, Chu *et al.* reported that wearing eye protectors was effective at protecting HCW (OR = 0.22, 95% CI: 0.12–0.39) [32]. Lai *et al.* reported in a case-control study that for surgical or operative procedures on patients with confirmed COVID-19 wearing gloves, googles, protective suits, gowns, shoe covers, and hats were protective (*P* < 0.001), while the same worn for performing aerosol-produced operations found no benefit provided by PPE in HCW [44].

In the cross-sectional literature, Zhao *et al.* reported that wearing gloves, isolation gowns, medical protective uniforms, face shields, and goggles resulted in negative testing among 960 HCWs in Wuhan by PCR and antibody test. They concluded that PPE is efficacious in preventing COVID-19 [37]. Khalil *et al.* reported that during usual care for COVID-19 patients, face shields/goggles offered statistically significant protection over other PPE (*P* = 0.012, OR = 0.437, 95% CI: 0.228–0.837), though no particular item was alone statistically significant, other than N95 masking (*P* = 0.021, OR = 0.372, 95% CI: 0.159–0.873) [41]. A case series by Yao *et al.* among anesthesiologists showed that those who wore goggles and face shields for patient intubation demonstrated no evidence of COVID-19 thereafter [38].

Implementation of infection control measures such as wearing PPE in daily practice might also allow HCWs to contain the spread of COVID 19 infection [48, 49] through exclusive use of FFP2/FFP3 (*P* = 0.99) and eye-protective devices (*P* = 0.99) [50]. Similarly, Hou and colleagues proposed a level system of PPE protection for HCWs [51]. Level 1 protection included disposable caps, surgical masks, white coats, and hand hygiene, and N95/FFP (filtering facepiece, FFP), isolation gowns, and disposable gloves were used when necessary. Level 2 protection included goggles and full-face shields, long sleeved, fluid repellent gowns, and shoe covers in addition to PPE for Level 1 protection. Level 3 protection included PPEs for Level 2 protection, as well as an isolation gown on top of the disposable coverall and potential use of a positive pressure helmet. Their study concluded that seropositivity for SARS-CoV-2 antibodies (IgG, IgM, or both IgG/IgM positive) was 3.4% (53 out of 1571) in local healthcare workers from Wuhan with level 2/3 PPE working in isolation areas, and was 5.4% (126 out of 2336) in healthcare staff with level 1 PPE working in non-isolation medical areas. In addition, a before-after comparative study by Cernigliaro *et al.* reinforced that positive rate to SARS-CoV-2 was lower using PPE and preventive measurements (Morbidity rate decreased from 14.3% to 8.6%) in the angiographic suite [52]. Contrarion literature exists as well. Boffetta *et al.* showed that wearing a face shield (OR = 1.22) and gown (OR = 1.39) was associated with COVID-19 infection [5]. Heinzerling and colleagues reported that wearing gloves was associated with high risk of COVID-19 infection (OR = 4.40) [11]. Notably, however, these studies had a non-causal study design and the reason underlying infection is unclear.

Furthermore, a modeling study by Mizukoshi *et al.* assessed risk of COVID-19 morbidity in HCWs during close contact with the patients [53]. They included multiple transmission components of COVID-19 such as hand contact with a contaminated surface, HCW’s fingers touching droplets from patients, and inhalation of aerosols and particles from the saliva of infected patients. They estimated that wearing face masks and shields were considered effective in decreasing the infection risk by between 63% and 99.9% [53].

Calò *et al.*’s scoping review [54] stated that the number of HCWs contaminated from sleeve cuffs, gloves, shoe soles, and patients’ masks were 16.7%, 25%, 50% and 40%, respectively. A cross-sectional study found that suboptimal use of goggles, gloves, and gowns was associated with increased COVID-19 risks in HCW (OR = 1.6, 1.2, and 1.4, respectively) [45]. Early in the pandemic there were notable challenges regarding PPE unavailability, inadequacy, and incorrectly used [55, 56]. Those issues may, in part, account for the heterogeneity of the results from some above-mentioned cross-sectional studies. In general, gloves, gowns, goggles, face shields, shoes, hats, and aprons have been reported to be protective against COVID-19 among HCWs. However, the efficacy of PPE is also related to AGP, donning and doffing, and hand hygiene.

## Discussion

We conducted a qualitative review of articles published during January 2019 to April 2021, including 47 studies regarding PPE use among HCWs worldwide. Since uncontrolled studies and cross-sectional studies provide limited evidence on causal inference, we focused on studies with higher evidence levels, such as reviews, case-control and cohort studies. Our systematic review highlights the efficacy of PPE, with particular attention to individual facial masks and other respiratory devices, gowns, gloves, and facial shields as tools used to protect HCWs during the COVID-19 pandemic. Although all of the identified studies suffer from varying biases, the bulk of the data supports that PPE is essential to protect HCWs from COVID-19 infection, but it must be coupled with adequate training and infection control measures. Facial masks, including N95 and surgical masks offered improved protection over cloth masks. The use of PAPR has been noted in the medical literature, but benefits over masking are difficult to ascertain given limited evidence and potential recall bias. Wearing gloves, gowns, and face shields have debatable efficacy given limited study and mixed results, including evidence that glove usage may increase infection rate, perhaps due to a false perception of protection. Given the likelihood of future pandemics, prospective evaluation of PPE utility in droplet-based and aerosol-based procedures, as well as in common medical treatment environments, is warranted. Further, prospective evaluation of the efficacy of PPE training in HCWs may be warranted, as potential exposure risk reduction may be complicated by concomitant false sense of security. Finally, PPE is effective in preventing nosocomial infection of COVID-19, but it should be noted that supplies of PPE were limited particularly in the early phase of the pandemic. Optimization and reduction of PPE consumption should be further investigated [57].

### Overall PPE use

Several studies have concluded that wearing PPE protects HCWs from COVID-19 infection among HCWs [10, 11, 30, 32, 33]. However, reliable protection is complicated by inadequate, improper, and incorrect usage of PPE, with low adherence in some settings and suboptimal and reused PPE potentially exhibiting lost efficacy, leading to opportunity for infection [16–18, 22, 23, 25, 27, 28, 54–56]. Surveys of infection prevention adherence in HCWs showed inadequate practices, with less than 90% wearing masks [58, 59]. In short, procedural errors and inadequate adherence will increase the actual risk of infection, and the designs of current studies might rise to the level of rigor to constitute actual evidence [58, 59]. Improper use of PPEs and low PPE adherence were shown to be risk factors for COVID-19 infections [16–18, 22, 27, 28, 45, 54]. Gholami *et al.* noted that wearing PPE must be reinforced by several aspects such as training, adequate PPE use, and appropriate hand washing procedures [16]. Although PPE is available for HCW, non-compliance and improper hand washing are risk factors of COVID-19 infection both in and outside of healthcare facilities. Thus, HCW require training on donning, doffing, optimal PPE use, and effective handwashing procedures to reinforce the ability for PPE to prevent nosocomial infection. Even with sufficient PPE, HCW who cared for COVID-19 patients remained at risk of infection, and so similarly ensuring quality and quantity of PPE as well as adequate decontamination procedures following medical procedures is necessary.

Of particular challenge is the consideration that PPE worn as collective bundles confound delineation of the protective effects of each PPE item. These protective effects may be additive when adhering to PPE bundles and thus may not reflect the actual effect estimates of each PPE item. Also, given the recent nature of the pandemic, evidence on COVID-19 included in some meta-analyses was found to be insufficient and with lower certainty [16, 33], and may bias underlying results.

A study by Chu *et al.* demonstrated risk ratio calculations from a meta-analysis for PPE. However, in most published studies, risk estimations for face shields were unadjusted and were not distinguished from other PPE effects [32]. Therefore, as the authors estimated, the integrated risk ratio in a meta-analysis would be too high due to combined PPE effects. Although a meta-analysis including face shields, goggles and visors might underestimate the effect of the face shield, individual study results only for face shields indicated similar risk estimates to goggles [60]. Consequently, the effects of individual PPEs in the real world may be less than estimated in the model analysis (e.g., Mizukoshi *et al.*) [53].

### Facial masks and other protective respiratory equipment

Wearing facial masks provides relatively robust protection for either HCW or non-HCW for respiratory viruses such as influenza and other aerosol-transmitted viruses [30, 33]. A systematic review and meta-analysis study by Liang *et al.* demonstrated that using facial masks by HCWs could reduce the risk of airborne virus infection by 80% (OR = 0.20, 95% CI: 0.11–0.37) [33]. At hand, however, is whether facial masks give strong protection to HCWs. Several types of facial masks were distributed during the pandemic, such as PAPR, N95 and surgical masks. Our finding suggests that these facial masks provide similar protection against respiratory viruses including COVID-19 [3–5, 11, 19, 25, 29, 30, 32–34, 40, 41, 44–47]. Studies focusing on preCOVID-19 data suggest that wearing N95 and surgical masks provide similar protection against respiratory viruses, including SARS-CoV-2 in HCWs, compared to cloth masks [6, 9]. Another systematic review by Samaranayake *et al.* showed that respiratory protective equipment could offer effective protection against aerosolized microbes in HCWs [61]. However, wearing a cloth mask provides a more protective measure than not wearing a mask in HCWs or non-HCWs for respiratory viruses other than SARS-CoV-2 [9]. In this situation, although cloth masks offer less protection than N95 and surgical masks, cloth masks are still recommended, particularly with limited supplies of PPE available to the general population, because they can offer double protection to both one’s self and others from viral infection.

Additionally, mask fit was identified as a key factor in medical mask and respirator efficacy [61]. A simulated model tested the efficacy of N95 and medical masks against viral and bacterial pathogens, finding that if medical masks and N95 were appropriately fitted, risk of infection was lower. Further, poorly fitted N95 were no better than loosely fitted medical masks [62]. MacIntyre *et al.*, in evaluating this, surmised that fitted N95 offered better protection against respiratory pathogens than medical masks [63]. It is thus important to note that it is the proper usage of properly fitted masks that provide protection to HCWs, and improperly used PPE may be ineffective [63].

A study by Mizukoshi *et al.* showed that when HCWs wore surgical masks and face shields, infections reduced by 63% and 99%, respectively [53]. It was estimated that about one-third of the risk could be further reduced if patients wore masks as well. However, the potential effectiveness of the model calculation should be compared with epidemiological evidence in the real world. There are several reports on the effectiveness of these PPE during the early phase of the pandemic. There has been an attempt to extend the wearing of masks for healthcare professionals, patients, and even the general population (universal masking), and during this period a concurrent reduction in the number of COVID-19 patients was noted [3, 42]. This is confounded by variable adherence, local or national lockdowns and stay-at-home orders, as well as development of herd immunity from widespread infection, complicating retrospective evaluation. Within HCWs, infection continued regardless of universal masking [3]. Though universal masking reduces the transmission rate in HCW, masking requires complementary preventative measures to further reduce or eliminate the risk of infection. Potential contamination from hands, droplet, and body fluids may enter the eyes or expose other parts of the body. Consequently, authors have discussed the paradox of universal masking, arguing that it may contribute to an increased risk of COVID-19 spread through other infectious means, and particularly if it distracts attention from other essential infection prevention programs [64].

Data regarding the effectiveness and utility of PAPR in protecting HCWs from viral infections is limited. Licina *et al.* highlighted that wearing PAPR offered no difference in infection rate versus other respiratory devices [31]. Sims *et al.* underscored that seropositive individuals wearing PAPR and N95 demonstrated fewer symptoms or a higher rate of having asymptomatic COVID-19 than individuals wearing surgical masks [35]. Wearing PAPR provided greater protection than other respiratory devices from cross-contamination perhaps due to limited ability for individuals to touch their face, though some published papers showed low-quality evidence [31], with confirmed COVID-19 cases being self-reported [35]. El Boghdadly *et al.* demonstrated no difference in infection rate between utilizing PPE WHO standard including PAPR during airway procedures and not wearing PPE [21].

### Gloves, gowns, and face shields

Our study revealed that the chance of becoming infected were lower among HCW who wore gloves, gowns, and eye protectors [4, 10, 33, 37, 38, 41, 44, 50]. However, none of the mentioned studies demonstrated complete protection if articles were worn individually. It is worth noting that during AGP, risk of infection increased even when wearing adequate gloves, gowns, and goggles [41, 44]. Lai *et al.* stated that AGP was conducted in high-risk departments that a relatively airtight environment and high density of patients might cause aerosol transmission in confined spaces, thus increasing risk of infection even wearing adequate PPE [44].

Several studies have reported that wearing shoes, face shields, gowns, gloves increased probability of COVID-19 infection; however, some data were taken by interview [4, 48], demonstrated low infection rates [11], or were self-reported [5].

It should be noted that all of the identified studies had varying degrees of bias. In particular, studies with non-causal study designs or those with a serious risk of bias provide results with limited reliability. The underlying cause of infection is uncertain due to the inadequate study design. Hence, it might affect the estimation of the effects of PPEs in HCWs.

### Other factors for nosocomial COVID-19 infections

Many studies have shown an increased risk for HCWs who cared for COVID-19 patients. However, working in the intensive care unit (ICU) was not associated with an increased risk of infection [65]. Patients with high salivary viral loads would manifest severe symptoms in the ICU, but the infection risk to HCWs was controlled using PPE and other measures. The most apparent risk to HCWs may be from asymptomatic colleagues or patients in the early stages of infection and without frank symptoms; these could be proven through serial PCR testing and/or contact tracing, measures not universally taken during the pandemic [66, 67]. Potential avenues of exposure included patient saliva, feces, and aerosol generated from using the toilet, or other day to day procedures [36, 68]. Moreover, HCWs can be infected by relatives at home or other people in the community, thereafter introducing the virus to their workplace [50, 69]. Therefore, contact between HCWs in health facilities increases overall infection risk more than what may be simulated in considering a single HCW in a limited setting. Thus, the potential exposure pathways to the virus and modifiers in HCWs are more wide-ranging than accounted for in many studies.

### Risk of bias and certainty of evidence

The result of risk of bias assessment is summarized in Table 2. Given most of the identified studies in our review are observational studies, we evaluated risk of bias on a total of sixteen comparative studies with a cohort or case-control design regarding PPE use among HCWs. Two of the seven studies with a cohort design were assessed as having serious overall risk of bias while investigating PPE use in HCWs [22, 24], mainly because of limited follow-up and no adjustment for potential confounding factors. The other five studies were assessed as having moderate overall risk of bias. Four of the nine other comparative studies with a case-control design were assessed as having serious overall risk of bias. The common reason for this judgement is that those studies failed to identify and adjust the potential confounding factors due to inadequate study design. The overall risk of bias of the other five case-control studies were all considered moderate. Most of the included studies were moderate and several papers were seriously biased.

**Table 2:** Risk of bias assessment on cohort and case-control studies for the investigation on PPE use.

	**Study design**	**Sample size (case/control in case-control studies)**	**Confounding**	**Selection bias**	**Bias in measurement of interventions**	**Bias due to departures from intended interventions**	**Missing data**	**Bias in measurement of outcomes**	**Bias in selection of the reported result**	**Overall risk of bias**
El-Boghdadly et al. [21]	Prospective cohort study	1718	Moderate	Moderate	Moderate	Low	Moderate	Low	Moderate	Moderate
Nguyen et al. [17]	Prospective cohort study	99795	Moderate	Moderate	Serious	Serious	Low	Moderate	Moderate	Moderate
Sims et al. [35]	Prospective cohort study	20614	Serious	Moderate	Serious	Serious	Moderate	Moderate	Low	Moderate
Ran et al. [18]	Retrospective cohort study	72	Moderate	Low	Serious	Serious	Moderate	Moderate	Moderate	Moderate
Wang Q et al. [22]	Retrospective cohort study	5442	Critical	Moderate	Serious	Serious	Serious	Moderate	Moderate	Serious
Oksanen et al. [39]	Cross-sectional and prospective study	866	Serious	Moderate	Moderate	Serious	Moderate	Moderate	Moderate	Moderate
Fletcher et al. [24]	Longitudinal point-prevalence study	around 1400	Critical	Serious	Serious	Serious	Serious	Low	Moderate	Serious
Chatterjee et al. [4]	Case-control study	378/373	Moderate	Low	Moderate	Moderate	Moderate	Low	Moderate	Moderate
Contejean et al. [47]	Case-control study	336/228	Moderate	Moderate	Moderate	Moderate	Moderate	Low	Moderate	Moderate
Dev et al. [29]	Case-control study	506/253	Moderate	Moderate	Moderate	Serious	Moderate	Low	Moderate	Moderate
Guo et al. [19]	Case-control study	24/48	Moderate	Moderate	Moderate	Moderate	Low	Moderate	Moderate	Moderate
Lai et al. [44]	Case-control study	151/174	Moderate	Moderate	Moderate	Serious	Low	Moderate	Moderate	Moderate
Wang X et al. [34]	Case-control study	86/407	Serious	Serious	Moderate	Serious	Low	Moderate	Moderate	Serious
Coppeta et al. [40]	Symptom monitoring	12/994	Serious	Moderate	Serious	Serious	Low	Moderate	Moderate	Serious
Heinzerling et al. [11]	Symptom monitoring	3/34	Critical	Moderate	Serious	Serious	Moderate	Moderate	Moderate	Serious
Farhat et al. [70]	Observational study	13	Serious	Serious	Critical	Critical	Low	Serious	Moderate	Serious

Most of the published papers we identified were observational studies prone to bias. Recall bias and responder bias due to self-report on intervention or exposure history were common in observational studies. It is worth noting that all fifteen observational studies measured PPE use status with questionnaires or interviews. Additionally, it could be difficult to gather information about potential switches in the use of PPE type and compliance on proper use of PPE and other infection prevention measures during the whole study period. Selection bias due to voluntary participation was also possible in most cohort studies and some case-control studies we included. Moreover, some of the studies did not define the PPE types or failed to collect information on all PPEs the participants employed [17, 24, 28, 29, 34, 35, 70]. Regarding outcome measurement, diagnosis based on the presence or absence of symptoms of infection could overlook asymptomatic infected individuals. In general, the risk of bias was generally moderate to serious in the observational studies we identified. Higher quality research on PPE use, such as randomised controlled studies, is limited.

We also assessed the quality of evidence of the systematic reviews we identified according to the GRADE system. The result is summarized in Table 3. The certainty of evidence was classified into four degrees (high, moderate, low, and very low) based on the investigated outcomes regarding PPE use in each review. Five of the seven reviews we found were rated as being of very low to low quality, with the other two reviews rated as having moderate quality of evidence. It should be noted that, among the seven identified reviews, only two [16, 54] provided evidence exclusively on SARS-CoV-2 infection, even though their first outcomes were not investigating PPE use among HCWs. Other reviews combined their investigation of COVID-19 with data from other preCOVID-19 diseases, such as MERS, SARS, and influenza [10, 30–33]. However, in all these reviews, we found limited direct high quality evidence regarding PPE use against COVID-19.

**Table 3:** Certainty assessment on identified reviews for the investigation on PPE use among HCWs.

**Study by**	**Design**	**PPE Intervention**	**Finding**	Study designs of the included studies*	**1. Risk of bias**	**2. Imprecision**	**3. Inconsistency**	**4. Indirectness**	**5. Publication bias**	6. Can Certainty be rated up?**	**GRADE ** **certainty ** **ratings**
Chu et al. [32]	Systematic review and meta-analysis	Face mask, eye protection (goggles, face shield)	Face mask and eye protection protect from viral infection than no use face mask and eye protection	Observational studies (7 comparative studies on COVID-19)	Moderate	Not serious	Not serious	Not serious	Not detected	Large magnitude of effect	Moderate
Gholami et al. [16]	Systematic review and meta-analysis	Wearing N95, mask or respirators. Inadequate PPE, re-using PPE, facial mask, respirators with COVID-19 infection in HCWs	Reused and inadequate PPE associated with COVID-19 infection, suboptimal hand hygiene and improper PPE correlated with COVID-19 infection and mask, N95 protects HCW from infection.	30 observational studies (5 studies investigated PPE)	Moderate	Not serious	Not serious	Not serious	Possible	No	Low
Liang et al. [33]	Systematic review and meta-analysis	N95	N95 protected HCW from COVID-19	13 case-control studies, 6 cluster randomized trials, and 2 cohort studies (1 study investigated SARS-CoV-2)	Low to moderate	Not serious	Not serious	Not serious	Not detected	No	Low
Tian et al. [10]	Systematic review and meta-analysis	Mask, gown, glove, N95, face protection.	Wearing gloves, gown, surgical mask, N95, face protection protected from COVID-19 infection in HCWs	28 retrospective cohort, 10 case-control, 11 prospective cohort, and 5 cross-sectional studies (17 on COVID-19)	Not serious	Not serious	Not serious	Not serious	Possible	Large magnitude of effect	Moderate
Licina et al. [31]	Systematic review	Air-purifying respirator (PAPR)	Field observational studies do not indicate a difference in healthcare worker infection utilizing PAPR devices versus other compliant respiratory equipment	10 studies including 2 RCTs, 3 simulation cross over studies and 5 observational studies (1 cohort study and 1 case series on COVID-19)	Moderate to serious	Serious	Serious	Serious	Not detected	No	Very low
Offei et al. [30]	Systematic Review (rapid review)	Facial masks: N95, cloth masks, surgical masks	Medical masks offered protection in HCW.	Of the 58 papers included in this review, 13 of them were systematic reviews and 45 were quantitative studies (27 of the papers reported studies conducted amongst HCWs, 7 of the studies investigated SARS-CoV-2)	Moderate to serious	Not serious	Unsure due to limited information	Not serious	Possible	No	Very low
Calò et al. [54]	Review (scoping review)	N95 respirator and isolation gown, gloves, PPE contamination	This scoping review summarizes the evidence on the burden, risk assessment, surveillance and management of HWs exposed to SARS-CoV-2.	43 studies, 14 webpages and 5 ongoing trials related HCW	Serious	Serious	Serious	Serious	Not detected	No	Very low

*Evidence from randomised controlled trials starts at high quality and, because of residual confounding, evidence that includes observational data starts at low quality. The certainty in the evidence is increased or decreased for several reasons, described in more detail below (1–6).

**Certainty can be rated up for: 1. Large magnitude of effect 2. Clear dose-response gradient 3. All residual confounding would decrease magnitude of effect rather than increase the magnitude of effect (in situations with an effect)

### Limitations

This study has several limitations. First, this review protocol was not prospectively registered, which may impact the quality of our review or lead to overlap of results. Second, we only searched articles in two databases in a limited search period due to the considerable volume of studies regarding our aim of research. Although we designed the search terms with a wide range of papers, trying to include HCWs in various positions and multiple types of PPE, there may be papers missed due to deliberately unspecific search terms. Our search period was set for 2019 to early 2021, and research published during the review interval is not included. The situation of COVID-19 infection has significantly changed worldwide during 2021 and 2022, reinforcing this approach, given ongoing change in SARS-CoV-2 variants and prolongation of response to less-virulent forms of the disease [71]. The Omicron variant, emerged in November 2021, has been the dominant variant globally since 2022 [71]. According to the US CDC, Omicron can cause more infections and spread more rapidly than the original SARS-CoV-2 strain [72]. Moreover, the universal vaccination in HCWs is an unadjustable variable when evaluating PPE efficacy. Since the COVID-19 vaccine was developed in 2020, vaccination rate was reported higher in HCWs than the general population (over 50% in mid-2021 reported by some US studies) [73, 74]. In August 2022, over 60% of the global population have received at least one dose of COVID-19 vaccine [75]. Since the infection of COVID-19 is set as the outcome in the majority of the studies we focus on, those above-mentioned factors can certainly affect the assessment of PPE efficacy to varying degrees. Those variables are different and difficult to adjust if we investigate the studies throughout 2019 to 2022. However, given the time lag in publication, some papers published after mid-2021 that may have met criteria could have been omitted.

Third, most studies identified are non-randomised observational studies, leading to potential risk of non-adjustable biases, such as selection bias and misclassification, as discussed above. High-quality prospective studies are still lacking. In addition, several reviews included in our study reported only limited researches on PPE against COVID-19 infections, and those papers with low infection rates may not be reflective of HCWs more globally.

Moreover, publication bias may occur in the studies we found. Meta-analysis was not performed in our study due to the difficulty in quantitative assessment, so it is hard to assess potential publication bias in the included studies.

## Conclusion

This systematic review highlights the efficacy of PPE among HCWs in the COVID-19 pandemic. Our findings demonstrate that PPE is necessary but must be accompanied by preventive measure training. In addition, facial masks afford superior protection than cloth masks, while the benefits of PAPR are less clear. The benefit of wearing individual gloves, gowns and face shields is questionable. This study suggests that employment of a full set PPE is recommended, however, more robust evidence is required to inform the efficacy. Controlling COVID-19 infection among HCWs is paramount to ensuring health system resilience during the pandemic.

## Data Availability

All the references evaluated for this systematic review is provided in supplemental table S1.

## Abbreviations

HCW: healthcare worker
PPE: personal protective equipment
WHO: World Health Organization
US CDC: United States Centers for Disease Control and Prevention
AGP: Aerosol-Generating Procedure
ACROBAT-NRSI: A Cochrane Risk Of Bias Assessment Tool for Non-Randomized Studies of Interventions
GRADE: Grading of Recommendations, Assessment, Development and Evaluations
ED: emergency department
MERS: Middle East Respiratory Syndrome
SARS: Severe Acute Respiratory Syndrome
PAPR: powered air-purifying respirators
ICU: intensive care unit

## Supplementary information

**Additional file 1:** **Table S1.** Summary of 47 reviewed publications (7 reviews and meta-analyses, 7 cohort studies, 9 case-control studies, 15 cross-sectional studies, 4 before and after studies, 4 case series and 1 modeling). **Table S2.** Summary of publications related to PPE (1 review, 5 cohort studies, 1 case-control studies, 2 cross-sectional studies, 1 before-after study and 2 case-series studies). **Table S3.** Summary of publications related to facial masks and other protective respiratory equipment (8 reviews, 1 cohort study, 8 case-control studies, 14 cross-sectional studies, 3 before and after comparison studies, 2 case-series studies and 1 modeling study).

**Additional file 2:** **Appendix** Literature search results. List of papers in Pubmed for first screening. List of papers in Web of Science for first screening.
