# Supplementary material for: Efficacy of personal protective equipment to prevent environmental infection of COVID-19 among healthcare workers: a systematic review
Source: Environ Health Prev Med. 2023 Jan 7;28:1. doi: 10.1265/ehpm.22-00131 (PMC9845060; doi:10.1265/ehpm.22-00131)
Supplement: Supplementary file 1 — Additional file 1: Table S1. Summary of 47 reviewed publications (7 reviews and meta-analyses, 7 cohort studies, 9 case-control studies, 15 cross-sectional studies, 4 before and after studies, 4 case series and 1 modeling). Table S2. Summary of publications related to PPE (1 review, 5 cohort studies, 1 case-control studies, 2 cross-sectional studies, 1 before-after study and 2 case-series studies). Table S3. Summary of publications related to facial masks and other protective respiratory equipment (8 reviews, 1 cohort study, 8 case-control studies, 14 cross-sectional studies, 3 before and after comparison studies, 2 case-series studies and 1 modeling study). [file ehpm-28-001-s001.docx]

***Supplementary Material***

Supplementary tables

[Table S1. Summary of 47 reviewed publications (7 reviews and meta-analyses, 7 cohort studies, 9 case-control studies, 15 cross-sectional studies, 4 before and after studies, 4 case series and 1 modeling). 2](#_Toc119922558)

[Table S2. Summary of publications related to PPE (1 review, 5 cohort studies, 1 case-control studies, 2 cross-sectional studies , 1 before-after study and 2 case-series studies). 37](#_Toc119922559)

[Table S3. Summary of publications related to facial masks and other protective respiratory equipment (8 reviews, 1 cohort study, 8 case-control studies, 14 cross-sectional studies, 3 before and after comparison studies, 2 case-series studies and 1 modeling study). 42](#_Toc119922560)

# Table S1. Summary of 47 reviewed publications (7 reviews and meta-analyses, 7 cohort studies, 9 case-control studies, 15 cross-sectional studies, 4 before and after studies, 4 case series and 1 modeling).

| **Study by** | **Date** | **Design** | **Population** | **PPE types** | **PPE Intervention** | **Result** | **Conclusion** | **Outcome** | **Funding sources** |
| --- | --- | --- | --- | --- | --- | --- | --- | --- | --- |
| **Review articles** | | | | | | | |  |  |
| Chu et al. (1) | February to April 2020 | Systematic review and meta-analysis | 44 studies but only 4 studies about COVID-19 in HCW | face mask, eye protection (goggles, face shield) | Comparison between face mask vs no face mask and eye protection vs no eye protection | Face mask vs no face mask (aOR=0.15, unadjusted RR=0.34), eye protection vs no eye protection (unadjusted RR=0.34) | Face mask and eye protection protect from viral infection than no use face mask and eye protection | Criteria infection coronavirus (COVID 19, SARS, MERS) based on WHO defined confirmed or probable. | WHO |
| Gholami et al. (2) | May to July 2020 | Systematic review and meta-analysis | Healthcare workers in 28 studies | N95, mask or respirators | Inadequate PPE, re-using PPE, facial mask, respirators with COVID-19 infection in HCW | There are three studies that associated with PPE use, HCW and COVID-19 infection such as Nguyen et al., Ran et al. and Guo et al. Nguyen et al. stated reused PPE and inadequate PPE in documented COVID-19 patients has unadjusted HR 5.12 and 5.95, respectively. Ran et al. stated suboptimal hand hygiene before, after contact with patients and improper PPE has RR 3.1, 2.4, 2.8. Guo et al. stated that no use N95, wearing respirator or mask, hand hygiene practice has OR 5.20, 0.15, 0.85 | Reused and inadequate PPE associated with COVID-19 infection, suboptimal hand hygiene and improper PPE correlated with COVID-19 infection and mask, N95 protects HCW from infection. | Selected studies of COVID 19 used symptoms, laboratory and imaging finding, | No financial support |
| Liang et al. (3) | 19th May 2020 and 19th June 2020 | Systematic review and meta-analysis | 21 studies, but only one study about COVID-19 in HCW | N95 | N95 vs no face mask | Only one paper by Wang et al. (2020) from this paper by Liang et al. that eligible, OR was 0.04 | N95 protected HCW from COVID-19 | Diagnosis of respiratory virus must have laboratory evidence and local clinical diagnostic criteria are applied during pandemic | No financial support |
| Tian et al. (4) |  | Systematic review and meta-analysis | 54 studies (191,004 Healthcare workers) | Mask, gown, glove, N95, face protection, | Gloves vs no gloves, surgical mask vs no surgical mask, gown vs no gown, N95 vs no N95, face protection vs no face protection | Meta-analysis's OR from this study found wearing gloves, gown, surgical mask, N95, face protection and hand hygiene is 0.48, 0.46, 0.37, 0.32, 0.41, and 0.54. Gloves vs no gloves (Study from Barrett 2020, OR 1.79; Chatterjee 2020, OR 0.38; Heinzerling 2020, OR 4.40; Subtotal OR 1.03), gown vs no gown (study from Barrett 2020, OR 1.79; Chatterjee 2020, OR 0.71; subtotal OR 1.07), surgical mask vs no surgical mask study from Heinzerling 2020, OR 0.02; subtotal OR 0.02; N95 vs no N95 (study from Guo 2020, OR 0.31; Wang Q 2020, OR 0.03; Wang X 2020, OR 0.04; subtotal, OR 0.08), face protection and no face protection (Study from Chatterjee 2020, OR 0.81; subtotal OR 0.81) | Wearing gloves, gown, surgical mask, N95, face protection protect from COVID-19 infection in HCW | Viral respiratory pandemics (SARSCoV-2, MERS, SARS CoV-1, influenza A H1N1, influenza H5N1) | Canadian Anesthesiologists Society Career Scientist Award, as well as the Merit Awards Program from the Department of Anesthesia at the University of Toronto |
| Licina et al. (5) | 8/2020 | Systematic review | Randomized controlled trials, non-randomized controlled trials, and observational studies. 10 full-text studies included | air-purifying respirator (PAPR) | PAPR versus other appropriate respiratory protection. | Two on-field studies reported no difference in the rates of healthcare workers performing airway procedures during the care of critical patients with SARS-CoV-2. A single simulation trial reported a lower level of cross-contamination of participants using PAPR compared to alternative respiratory protection. Field observational studies do not indicate a difference in healthcare worker infection utilizing PAPR devices versus other compliant respiratory equipment. | Field observational studies do not indicate a difference in healthcare worker infection utilizing PAPR devices versus other compliant respiratory equipment | Selected studies used some coronavirus infections such as SARS CoV 2, SARS, Ebola and MERS. | No financial support |
| Offei et al. (6) | 12/2020 | Systematic Review (rapid review) | 27 out 58 studies conducted amongst HCW wearing face masks | Mask, N95, cloth masks, surgical masks | Mask vs no mask, N95 vs surgical mask/medical face mask. | Study from Batozsko et al. medical mask vs N95, OR: 1.06; Chu et al. face mask vs no face mask, aOR: 0.15, unadjusted RR: 0.34; eye protection, unadjusted RR: 0.34; Liang et al., cit Wang et al., wearing mask vs no wearing mask, OR 0.04; Wang et al. A, inadequate protection vs level 2 protection, RR 36.9; Wang et al. B, no mask group vs N95, aOR: 464.82. | Medical masks offered protection in HCW. | The transmission of respiratory viral infection in the selected papers were SARS CoV2, seasonal influenza, H1N1, influenza, MERS etc. | No financial support |
| Calo et al. (7) | 1 January 2020 up to 22 May 2020 | Review (scoping review) | 43 studies, 14 webpages and 5 ongoing trials related HCW | N95 respirator and isolation gown, gloves | PPE contamination | In a study performed in healthcare facilities, the most contaminated objects were self-service printers (20.0%), desktops/keyboards (16.8%) and doorknobs (16.0%); both hand sanitizer dispensers (20.3%) and gloves (15.4%) were the most contaminated PPE. | This scoping review summarizes the evidence on the burden, risk assessment, surveillance and management of HWs exposed to SARS-CoV-2. | Authors confirmed the outcome of published papers only COVID 19 or SARS CoV2. | No financial support |
|  |  |  |  |  |  |  |  |  |  |
| **Cohort study** | | | | | | | |  |  |
| El-Boghdadly et al.  (8) | 23 March and 2 June 2020 | Cohort study (prospective international multicenter study) | Healthcare workers performing or assisting in tracheal intubation of patients with suspected or confirmed COVID-19 across 17 countries. 1718 participants who recorded at least one tracheal intubation and one follow-up | PPE gown; gloves; eye protection; and respirator masks certified to N95 or FFP2 or FFP3 or equivalent standard) N95, FFP3/N100 respirator | PPE conforming to World Health Organization (WHO) recommended minimum standards for aerosol-generating procedures | Univariate analysis PPE WHO standard HR=0.97 (0.63–1.51) | Following WHO recommended minimum standard PPE use in HCWs performing or assisting in tracheal intubation slightly reduced the risk of COVID-19 infection. | Total of 184 participants (10.7%) met the primary endpoint, of whom 144 (8.4%) reported symptomatic self-isolation, 53 (3.1%) reported laboratory-confirmed COVID-19 infection and 2 (0.1%) participants reported hospital admission with COVID-19 symptoms | the Difficult Airway Society (UK), the American Society of Anesthesiologists, the  International Anesthesia Research Society and the Anesthesia Patient Safety Foundation. |
| Nguyen et al. (9) | March 24 (UK) and March 29 (USA) to April 23, 2020 | Cohort study | 99795 front-liner healthcare workers | PPE | Risk reporting of a positive test for COVID-19 according to availability of PPE (Adequate PPE, reuse PPE and inadequate PPE), and exposure of patients (no exposure, suspected and exposure to COVID-19) among HCW | Overall reused PPE and inadequate PPE (unadjusted HR 1.46, 1.32), no exposure to patients COVID-19 in reused PPE and inadequate PPE (unadjusted HR 0.96, 1.53), exposure to patients with suspected COVID-19 in reused and inadequate PPE (unadjusted HR 3.23, 1.87), exposure to patients documented COVID-19 (unadjusted HR 5.2, 5.95) | Reused and inadequate PPE associated with COVID-19 infection in frontline HCW. | Self-reported data from the COVID Symptom Study smartphone application. Total 242 of 2747 cases COVID 19 per 100000 HCWs. | Zoe Global, Wellcome Trust, Engineering and Physical Sciences Research Council, National Institutes of Health Research, UK Research and Innovation, Alzheimer’s Society, National Institutes of Health, National Institute for Occupational Safety and Health, and Massachusetts Consortium on Pathogen Readiness. |
| Oksanen et al. (10) | 15-Jul-20 | Cross-sectional and prospective study | 1072 volunteers: HCWs at the Helsinki University Hospital. 866 (80.8%) of the HCWs completed the questionnaire. | FFP2/3 respirators and surgical masks, etc. | the use of FFP2/3 and masks | In 14 cases (63.6%), occupational infections occurred while using a surgical mask, and all infections originating from patients occurred while using a surgical mask or no mask at all. No occupational infections were found while using an FFP2/3 respirator and following aerosol precautions. In this study, none of the ICU HCWs got infected while using the recommended PPE (a FFP3 respirator [or FFP2 if FFP3 was not available], a pair of gloves, a long-sleeved fluid repellent gown, hair protection and eye protection). | The authors recommended the use of FFP2/3 respirators in all patient contacts with confirmed or suspected COVID-19, along with the use of universal masking | The participants with COVID-19 symptoms were tested with the SARS-CoV-2 real time polymerase chain reaction method. The infection divided into confirmed workplace, likely workplace, unclear and outside workplace | Helsinki University Hospital |
| Ran et al. (11) | Nov 19, 2020 | Cohort study (retrospective) | 72 HCW in Wuhan, 33 general group and 39 high risk group | PPE | Improper PPE (yes/no) between high-risk group and general group. | 18 HCW in high risk got infection and 10 in general groups, while 15 in high risk no infection and 29 in general group. Improper use PPE has RR 2.82. | Improper use of PPE associated infection COVID-19 | The novel coronavirus nucleic acid was detected by real-time fluorescence RT-PCR, and the virus gene was sequenced, which was highly homologous with the known new coronavirus | No financial support |
| Sims et al. (12) | April 13 and May 28, 2020 | Cohort study (prospective) | Blood samples were collected from 20614 of 43000 employees working in hospital | Surgical masks, N95 mask, Powered Air Purifying Respirator (PAPR) | WearingN95/PAPR or other types of masks when exposed to COVID-19 | A total of 1,818 (8.8%) participants were seropositive between April 13 and May 28, 2020. Among the seropositive individuals, 44% reported that they were asymptomatic during the month prior to blood collection. Healthcare roles such as phlebotomy, respiratory therapy, and nursing/nursing support exhibited significantly higher seropositivity. Among participants reporting direct exposure to a COVID-19 positive individual, those wearing an N95/PAPR mask had a significantly lower seropositivity rate (10.2%) compared to surgical/other masks (13.1%) or no mask (17.5%). | Direct contact with COVID-19 patients increased the likelihood of seropositivity among employees but study participants who wore a mask during COVID-19 exposures were less likely to be seropositive. | Detection SARS CoV2 was conducted by qualitative and semi quantitative serology test. Internal validation provided specificity and sensitivity 16-days post PCR at 99.35% and 98.14%. | The Beaumont Health Foundation with philanthropic gifts provided by following: Sidney and Madeline Forbes, Nathan and Catherine Forbes, Edward C Levy and the Linda Dresner Foundation, Stephen and Bobbi Polk, Warren and Carol Ann Rose and Family, Elizabeth Rose, Mickey Shapiro and Family, S.Even and Gwen Weiner and the Hearst Foundation. |
| Wang Q et al. (13) | Final follow up date was March 1, 2020. | Cohort study (retrospective) | 5442 neurosurgery department medical staff (120 cases, in which there were 54 doctor and 66 nurses) | Level 1 protection （including disposable caps, surgical masks, white coats, and hand hygiene. N95/FFP (filtering facepiece, FFP), isolation gowns, and disposable gloves are used when necessary）  Level 2 protection (in addition to PPE for Level 1 protection, goggles and full face shields, long sleeved, fluid repellent gowns, and shoe covers are used)  Level 3 protection (the addition to PPE for Level 2, an isolation gown on top of the disposable coverall and potential use of a positive pressure helmet) | adequate vs inadequate protection | Medical staff members who took inadequate protection had a higher risk of contracting infection than those using level 2 protection (RR=36.9) | Level 2 protection give strong prevention COVID-19 in HCW | Participants with a positive nucleic acid test result on real-time reverse-transcriptase–polymerase chain reaction (RT-PCR) assay of nasal and pharyngeal swab specimens were considered cases | National Natural Science  Foundation of China and the  Fundamental Research Funds for the Central Universities |
| Fletcher et al. (14) | August 17–September 4, 2020 (period 1) and during December 2–23, 2020 (period 2) | Longitudinal point-prevalence study | All HCWs were invited to participate (1385 in the first period and 1445 in the second period) | PPE: hospital policy. Masks including N95/CAPR, surgical masks. | Appropriate PPE use. Use of N95/CAPR or surgical masks. | Wearing appropriate PPE was not associated with seropositivity of COVID 19 infections (P=0.76). Wearing N95 mask or CAPRs was not associate with seropositivity COVID 19 (P=0.897) | Wearing appropriate PPE did not associate with seropositivity of COVID 19 in two periods. | The outcome of COVID-19 disease was diagnosed by either self-reported PCR or antigen test (diagnostic tests) or seropositivity for SARS-CoV-2 antispike protein IgG | No financial support |
|  |  |  |  |  |  |  |  |  |  |
| **Case-control study** | | | | | | | |  |  |
|  |  |  |  |  |  |  |  |  |  |
| Chatterjee et al. (15) | May 8-23, 2020 | Case-control study | 378 case and 373 control HCW in India | PPE, glove, gown, cap, shoe, face shield | PPE usage (never used, always used), mask (any mask, no mask), cap (yes, no), gown (yes, no), shoe (yes, no), face shield (yes, no), gloves (yes, no) | Never used vs used (OR=3.72), any mask vs no mask (OR=0.35), cap yes vs no (OR=0.7), gown yes vs no (0.62), shoes yes vs no (OR=1.05), face shield yes vs no (OR=0.81), gloves yes vs no (OR=0.38) | Never used PPE, using shoes associated with COVID-19 infection; any mask, cap, gown, face shield, glove protect from infection | HCWs testing positive on real-time reverse transcription-polymerase chain reaction (qRT-PCR) for SARS-CoV-2 were defined as cases.  Controls were symptomatic  HCWs who tested negative on qRT-PCR for SARS-CoV-2 under similar considerations. | No financial support |
| Contejean et al. (16) | 24 February until 10 April 2020 | Case-control study | Overall cases are 564, case 336 and control 228 (physician, paramedic staff, administrative staff, other employees) | medical mask. PPE (including gowns, gloves, eye protections, and either medical masks for standard care or FFP2 masks during airway aerosol-generating procedures) | Exposure to patients (close contact with suspected or confirmed case COVID-19 without PPE and with PPE), while exposure to colleagues (wearing medical mask, spending time with colleagues without mask and close contact with colleagues without mask) | Exposure to patients (close contact patients without PPE and with PPE) has OR 1.22[0.64–2.38] and 0.28[0.16–0.51]; while exposure to colleagues by using mask frequently, spending time without mask and close contact without mask has OR 0.45[0.28–0.72], 2.77[1.63–4.77] and 2.8[1.57–5.16] | PPE and medical mask protect HCW from COVID-19 | the RealStar rt-PCR kit, a triplex PCR amplifying the viral genome in the E and S genes and an internal control. The result was considered positive if 3 of the 3 targets were amplified. A control sample was requested if only 1 or 2 target genes were amplified. | There is personal fee and non-funding support from Janssen-Cilag, Sanofi, MSD, Eumedica, Pfizer, Frezenius, Cubist, Correvio, and Astellas. MSD France, ViiV Healthcare, Medtronic SAS etc. |
| Coppeta et al. (17) | Dec 2020 | Case-control study (monitoring study) | Active symptom monitoring in 1006 HCWs identified as contacts of COVID-19 cases. (345 male and 661 female).42 years old (range: 23–69 years) | gloves, masks, eye protection | wearing masks/gloves/eye protection | Regarding the use of personal protective equipment (PPE), only the use of facial masks was inversely related to the chance of becoming infected (p<0.01) | The use of facial masks should be implemented even in settings where known patients with COVID-19 are not present | The Seegene AllplexTM2019-nCoV Assay identifies the virus by multiplex real-time PCR targeting three viral genes (E, RdRP and N) | No financial support |
| Dev et al. (18) | May and July 2020 | Case-control study | HCWs positive for SARS-CoV-2 infection were the cases (n=506) and those negative for SARS-CoV-2 were the controls (HCW: doctor, nurse, sanitarian, technician, security, others) (n=253 | PPE | Appropriate PPE during exposure patients and PPE use | Appropriate PPE during exposure patients (OR 0.65, univariate) and PPE use (OR 0.63, multivariate) | PPE protects HCW from COVID-19 | COVID-19 patient: individuals with laboratory-confirmed SARSCoV2 infection (by reverse transcription polymerase chain reaction [RT-PCR] | No financial support |
| Guo et al. (19) | December 31, 2019, to February 24, 2020, in the urban area of Wuhan. | Case-control study | 26 orthopedic surgeons from 8 hospitals in Wuhan (case 24, control 48) | N95 respirator | Usage of N95 (yes/no), wearing respirator or mask all of time (yes/no) | No use of N95 and wearing mask all of time has OR 5.20 (1.09 to 25.00) and 0.15 (0.04 to 0.55). No mask wearing by patients with suspected COVID-19 has OR 6.05 (1.70 to 21.51) | Respirator N95 can protect orthopedic surgeons from COVID-19 | Of 24 cases, 21 were confirmed cases with positive reverse transcription polymerase chain reaction (RT-PCR) tests or antibody tests, and 3 were clinically diagnosed cases with a history of exposure to COVID-19, fever and respiratory symptoms, a chest CT scan with ground-glass opacity and consolidation, leucopenia and/or lymphopenia, and negative influenza virus tests. | No financial support |
| Heinzerling et al. (20) | April, 2020 | Case-control study (monitoring study) | 37 HCW in California | Gloves, mask | gloves vs no gloves; mask vs no mask | Gloves vs. no gloves (OR=4.40; mask vs no mask (OR=0.02) | Gloves associated with infection SARS-CoV2 and wearing mask protects from SARS-CoV2 infection. | HCWs who developed symptoms during 14 days monitoring were tested for SARS-CoV2 using RT PCR | No financial support |
| Lai et al. (21) | February 11 to February 15, 2020 | Case-control study | 325(infected 151) | mask, glove, goggle, suit,　gowns, shoe covers, hats | mask=: N95/KN95, medical protective mask patient = surgical masks | When performing general operations on confirmed or suspected patients, the use of protective equipment including the effectiveness of masks (p < 0.001), gloves (p < 0.001); and the use of gloves (p < 0.001), suits (p < 0.001), gowns (p < 0.001), shoe covers (p < 0.001), and hats (p < 0.001) were protective factors. The use of protective equipment was a protective factor in most cases. Negative emotions and dissatisfaction to the hospital response were associated with the increased risk of infection | HCWs have an outbreak in concentrated departments in the hospital. The COVID-19 infection is associated with the effectiveness and the use of PPE, emotions and satisfactions to hospital responses. | HCWs were diagnosed as COVID-19 with the positive nucleic acid test or clinical diagnosis. | National Natural Science Foundation of China |
| Wang X a et al. (22) | During March and April 2020 | Case-control study (letter to editor) | 493 medical staff at six departments (Respiratory, Intensive Care Unit (ICU), Infectious Disease, Hepatobiliary Pancreatic Surgery, Trauma and Microsurgery and Urology) from Zhongnan Hospital of Wuhan University | N95 | N95 vs no face mask | Among the 493 medical staff, none of the 278 staff (56 doctors and 222 nurses) in the N95 group became infected, but 10 of 213 staff (77 doctors and 136 nurses) from the no-mask group were confirmed as infected. Infection rate for medical staff was significantly increased in the no-mask group compared with the N95 respirator group (difference: 4.65%, (95% confidence interval: 1.75%-infinite); P<2.2e-16) (adjusted odds ratio: 464.82, (95% confidence interval: 97.73e infinite); P<2.2e-16). | N95 protected HCW from COVID-19 | Cases of 2019-nCoV infection were investigated by chest computed tomography, and confirmed by molecular diagnosis. | Medical Science Advancement Program (Clinical Medicine) of Wuhan University |
| Farhat et al. (23) | 4/2021 | Case-control study | 6 nurses, 3 assistant nurses, 2 residents, 1 fellow, and 1 staff | air impermeable plastic gown with a head-to-toe cover, an N95 mask, gloves, and a face shield. | examined as to whether the cotton gown could be used instead of PPE. After three months, all medical staff were evaluated for clinical signs of COVID-19, such as fever, cough, nausea, and headache. | During these three months, no symptoms or absence due to illness were observed in the staff. | Cotton surgical gown protected the staff against COVID-19. Accordingly, the cotton surgical gown can be used in medical centers to replace PPE sets containing an air-impermeable plastic gown with a head-to-toe cover. | The subjects  were evaluated for clinical signs of COVID-19 diseases, such as fever, dry cough, headache,  dizziness, diarrhea, nausea, and hemoptysis without antibody test | No financial support |
| **Cross-sectional study** | | | | | | | |  |  |
| AranazAndres et al. (24) | 24 February until 10 April 2020 | Cross-sectional study | Of 5223 healthcare workers, 167 included study groups (PCR based) and 156 comparison groups (No PCR) | mask, goggle, gloves, gown | Correct use of mask, goggles, gloves, gown, used hand sanitizer and soap in suboptimal and optimal score in 132 HCW lab confirmed SARS CoV2 vs comparison | Suboptimal use mask, goggles, gloves, gown, hand sanitizer and soap were OR 0.99, 1.6, 1.2, 1.4, 0.93, 0.71 | suboptimal use of goggles, gloves, gown associated with COVID-19 in HCW, while suboptimal wearing mask, hand sanitizer and soap are a protective factor. | RT-PCR assay to detect SARS-CoV-2 | No financial support |
| Boffetta et al. (25) | February 24 to April 10, 2020 | Cross-sectional study | 10,654 Italian HCWs (physician, nurse, health care assistant, technician, others) who were tested for presence of SARS-CoV-2 | Any mask, face shield, gloves, gown, any PPE | Wearing PPE and SARS CoV2 infection in HCW | Any mask OR 0.68; face shield OR 1.22; gloves OR 0.72; gown OR 1.39; and any PPE, OR 0.94. | Wearing mask, gloves, any PPE protects from SARS-CoV2, while face shield and gown associated with SARS-CoV2 | HCWs were tested for SARS-COV-2 infection using either a rhinopharyngeal or an-oro-pharyngeal swab with the criteria based on WHO. Tests were repeated for most subjects with a positive result to monitor the infection; a proportion of subjects with a negative result were re-tested because of repeated contact with a COVID-19 case. | The study was funded with internal resources of the participating institutions Working Group on SARS‑CoV‑2 Infection in Italian Healthcare Workers |
| Moreno-Casbas (26) | 4 to 30 April 2020 | Cross-sectional study (descriptive) | 41239 (analysis for 2230) | Masks, gloves, gowns, goggles, shower etc. | Frequently used of PPE | The perception about the availability of protective measures as «always/frequently» were: FPP1 mask 57.3%, gloves 89.5%, soap 95% and hydro alcoholic solution 91.5%. In PPE, FPP2, FPP3 mask, goggles and disposable gowns at around 50% | Preliminary data are presented, with variability in the response rate by Autonomous Region. Healthcare professionals infected by SARS-CoV-2 identified the management of the chain of infection transmission, the use and adequacy of protective equipment, as well as the effectiveness of handwashing as factors related to the transmission of the virus among professionals. | The diagnosis was made based on a suspicious case and a probable case using a questionnaire. | Instituto de Salud Carlos III |
| Davido et al. (27) | May and July 2020 | Cross-sectional study | 99 hospital staff members (HSM) such as healthcare workers, physicians, nurses, nursing assistants, support staff, administrative employees. Of them, 28 positive PCR tests for COVID-19 and 71 negatives. | Mask | Systematic used mask (yes/no) and meeting without and with mask among 99 HCW | Systematic used mas, OR 21. Meeting without mask, OR 10.3 | Mask protects HCW from COVID-19 infection | A real-time reverse transcriptase polymerase chain reaction (RT-PCR) amplifying the betacoronavirus E gene and the SARS-CoV-2 RdRp gene was performed to  detect the SARS-CoV-2 genome from a nasopharyngeal swab collected by a trained nurse | No financial support |
| Jung et al. (28) |  | Cross-sectional study (Letter to the Editor) | HCWs caring for patients (Nineteen nurses caring for the two patients) | Coveralls PPE | all participants wore PPEs | 15 (11%) revealed positive SARS-CoV-2 PCR results in the following areas: top of the head (26%), foot dorsum (26%), sole (16%), wrist (5%), and abdomen (5%). No SARS-CoV-2 RNA was detected on the neck and back. The top of the head, foot dorsum, and sole were more frequently contaminated than the neck, wrist, abdomen, and back (23% [13/57] vs. 3% [2/76], P < 0.001 by Fishers’ exact test). | Wearing of PPE that fully covers the head and feet, as well as cautious doffing procedures. | Surface swab samples were collected from the outside surface of the PPE at top of the head, neck, wrist, abdomen, back, foot dorsum, and sole by swabbing 15  times using aseptic Dacron swabs that were pre-moistened with viral transport media. The sample then Real-time reverse transcription (RT)-PCR from the patients’ nasopharyngeal swab was performed using Allplex 2019-nCoV | Government wide R&D Fund Project for Infectious Disease Research (GFID), Republic of Korea |
| Khalil et al. (29) | May to June 2020 | Cross-sectional study | 98 COVID-19 positive physicians and 92 COVID-19 negative physicians who work in different healthcare facilities and had known or unknown interactions with COVID-19 patients. | N95 mask, single-use gloves, protective face-shields/goggles, disposable gown, water-proof apron | wearing N95, wearing PPE single-use gloves, protective face-shields/goggles, disposable gown, water-proof apron, proper handling of PPE, proper hand-hygiene during different patient care, and decontamination of the surroundings of the patient | 1. Formal training on PPE use (OR = 1.667; CI: 0.890–3.121) mildly increased the odds of being infected, which was not significant. 2. Wearing the N95 mask was significantly associated with a low probability of COVID-19 infection (OR = 0.373; CI: 0.159–0.873). 3. Single-use gloves, Face-shield/goggles, Disposable gown, Water-proof apron: none of these results were statistically significant | The use of face shields/goggles and N95 masks and decontamination of the patient’s surroundings may give protection against COVID-19. Additionally, reusing medical gowns should be avoided as much as possible. | The case was detected by reverse transcriptase-polymerase chain reaction (RTPCR). The controls were COVID-19 negative (having no symptoms of COVID-19 or tested negative) | No financial support |
| Liu M et al. (30) | 24 January to 7 April 2020 | Cross-sectional study | 420 healthcare professionals (116 doctors and 304 nurses) All healthcare professionals had performed at least one aerosol generating procedure. | Mask: N95 respirator, Surgical mask, medical suit, i solation gown, a pron, g loves, e ye protection, h air cover | Those PPE in ward and during AGP procedure | None of the nasopharyngeal swabs collected from the participants tested positive for nucleic acids on the reverse transcriptase polymerase chain reaction assay for SARS-CoV-2. None of the serum samples of participants tested positive for SARS-CoV-2 specific IgM or IgG antibodies (95% confidence interval 0.0 to 0.7%). | Despite being at high risk of exposure, healthcare professionals who were appropriately protected did not contract infection or develop protective immunity against SARS-COV-2. | Nasopharyngeal swabs were collected three times from each participant during the two week quarantine period. SARS-CoV-2 nucleic acid testing by using reverse transcriptase polymerase chain reaction | The First Affiliated Hospital of Sun Yat-sen University. |
| Schmitz et al. (31) | 1 March and 15 May 2020 | Cross-sectional study | 164 ED staff workers | Glove, surgical hat, eye protection, surgical mask, FFP1, FFP2, N95 | 42 questions about PPE-usage and infection rate of COVID-19. | In 13 hospitals, an FFP2 (filtering facepiece particles >94%  aerosol filtration) mask or equivalent and eye protection was worn for all contacts with patients with suspected or confirmed SARS-CoV-2 during the whole study period. The unadjusted staff infection rate was higher in these hospitals [7.3 (3.4–11.1) vs. 4.0 (1.9–6.1)%, absolute difference+3.3%]. After adjusting for hospital testing policy, type of PPE was not associated with incidence of COVID 19 infections among ED staff (P=0.40) | In this cross-sectional study, the use of high-level PPE (FFP2 or equivalent and eye protection) by ED personnel during all contacts with patients with suspected or confirmed SARS-CoV-2 does not seem to be associated with a lower infection rate of ED staff compared to lower-level PPE use. Attention should be paid to ED layout and social distancing to prevent cross-contamination of ED personnel. | SARS-CoV-2 infections were regarded as confirmed when viral DNA was detected by reverse transcriptase PCR (RT-PCR) in the nasopharynx swab | No financial support |
| Tabah et al. (32) | April 2020. | Cross-sectional study (web-based survey) | 2711 responses from 1797 (67%) physicians, 744 (27%) nurses, and 170 (6%) Allied HCW. | FFP2, N95, PAPR | Wearing PPE during routine care | For routine care, most (1557, 58%) reportedly used FFP2/N95 masks, waterproof long sleeve gowns (1623; 67%), and face shields/visors (1574; 62%). Powered Air-Purifying Respirators were used routinely and for intubation only by 184 (7%) and 254 (13%) respondents, respectively. Surgical masks were used for routine care by 289 (15%) and 47 (2%) for intubations. At least one piece of standard PPE was unavailable for 1402 (52%), and 817 (30%) reported reusing single-use PPE. PPE was worn for a median of 4 h. | PPE practices availability, and confidence in adequacy to provide protection among HCWs at the frontlines of the COVID-19 pandemic. Respondents report widespread shortages and reuse of single-use PPE items. | A web-based survey to HCW reports surrounding PPE related to the COVID-19 pandemic. | Clinical Research Career Development Fellowship from the Wellcome Trust, Post.doc Mobility grant from the  Swiss National Science Foundation |
| Tekalegn et al. (33) | June 3, 2020, to August 11, 2020 | Cross-sectional study (web-based online self-administered questionnaire) | 98 out 368 HCW daily contact directly with patients. | F acemask | C orrect use of facemask: perform hand hygiene before/after wearing masks, reuse masks, etc. | The level of overall correct use of facemask was 10.1% (95% CI: 7.4–13.6). | Health professionals’ practice regarding the correct use of facemask in the context of COVID-19 prevention is very low. | A structured, web-based, and self-administered questionnaire to HCW who caring patients with COVID 19 | No financial support |
| Wee et al. (34) | 1 February 2020 to 30 April 2020 | Cross-sectional survey (letter to editor) | 10 000 HCWs, 1780 of which were ancillary HCWs in Singapore | N95 respirator, disposable gown and gloves, and eye protection and universal masking | Wearing PPE and universal masking between HCWs and ancillary workers | Odd ratio for nurses wore N95, gown, gloves and eye protection is 1.01, 0.74, 0.77, 0.98; allied health is 1, 0.86, 0.86, 0.96 and ancillary HCW is 0.72, 0.64, 0.65, 0.83, respectively. | Although overall rates of infection among HCWs were low, rates of infection were higher in ancillary HCWs compared with medical and nursing staff (0.32%, 5/1548, versus 0.10%, 5/5101; incidence-rate ratio 3.29, p 0.04). | Confirmed case COVID 19 was detected by PCR test | No financial support |
| Zhao et al (35) | July 2020 | Cross sectional study | 960 HCWs who had provided healthcare service in over 37 Hubei hospitals | Gloves, medical mask, N95/FFP2, face shield or goggles, isolation gown, medical protective uniform, positive pressure headgear | wearing PPEs. 98.6% of them showed high levels of adherence to PPE protocols. | positive results in three consecutive RT-PCR tests (7 days apart) | negative results of RT-PCR tests in all participants with a median 40-day exposure duration along with negative results of antibody tests in 70.0% of participants showing that they were never infected indicated that PPE is an efficacious measure to durably contain the nosocomial transmission of SARS-CoV-2. | Before ending quarantine, all participants had three consecutive RT-PCR tests (7 days apart) and some of HCWs were tested for anti-SARS-CoV-2 IgG and IgM | National Natural Science Foundation of China |
| Botti et al. (36) | 2/2021 | cross sectional study | 44 hematology centers | surgical mask, FFP2,　FFP3,　TNT gown, Water resistant gown,　Visor, Safety glasses | Some questions about covid-19 including PPE | Some healthcare professionals were asked to wear PPE in caring hematology patients that found PPE protected HCWs from the infection. | As hematology HCPs apply infection control measures in their daily practice such as wearing PPE, this may have allowed them to contain the spread of the virus in their setting more effectively. | COVID-19 testing was performed on　HCPs in 79.5% of centers, most commonly with both nasopharyngeal swab and blood tests (47.7%); tests were repeated routinely in 68.2% of centers. | No financial support |
| Kindgen-Mills et al. (37) | June 16th to July 2nd 2020 | cross sectional | 516 physicians from all over Germany who attended certified registered training courses for intensive care or emergency medicine in the  city of Arnsberg in Northrhine-Westfalia | FFP2, FFP3, goggles and face shield | Wearing respiratory protective measure and SARS-CoV 2 infection examined by ELISA | No statistically significant correlation  between the availability of FFP2/FFP3 masks and SARS-CoV-2 infection (p=0.99). no statistically significant correlation between the availability of eye protective devices and SARS-CoV-2 (p=0.99)  infection | Wearing masks and eye protectors provided protection to HCWs from CoVID 19 infection. | Serological testing was performed within 3–7 days after sample collection. Antibodies directed against SARS-CoV-2 were detected by automated, CE certified electrochemiluminiscence　immunoassay (ECLIA). Positive samples were further evaluated via semiquantitative, automated anti-SARS-CoV-2 IgG and IgA enzyme-linked immunosorbent assay (ELISA) | No financial support |
| Hou et al. (38) | 9/2020 | cross sectional | 8529 healthcare workers （medical teams aiding Hubei, local healthcare workers in Wuhan and Jingzhou of Hubei Province） | Level 1 protection （including disposable caps, surgical masks, white coats, and hand hygiene. N95/FFP (filtering facepiece, FFP), isolation gowns, and disposable gloves are used when necessary）  Level 2 protection (in addition to PPE for Level 1 protection, goggles and fullface shields, long sleeved, fluid repellent gowns, and shoe covers are used)  Level 3 protection (the addition to PPE for Level 2, an isolation gown on top of the disposable coverall and potential use of a positive pressure helmet) | level 0 PPE protection for HCWs working in the non-medical area  level 1 PPE protection for HCWs working in the non-isolation medical area  level 2/3 PPE protection for HCWs working in the isolation medical area | 1. Serial tests for SARS-CoV-2 RNA and tests for SARS-CoV-2 immunoglobulin M and G after the 6-8 week mission revealed a zero cumulative attack rate in medical teams aiding Hubei.  2. The seropositivity for SARS-CoV-2 antibodies (IgG, IgM, or both IgG/IgM positive) was 3.4% (53 out of 1571) in local healthcare workers from Wuhan with Level 2/3 PPE working in isolation areas and 5.4% (126 out of 2336)　in healthcare staff with Level 1 PPE working in non-isolation medical areas, respectively. | Adequate training PPE can protect medical personnel against SARS-CoV-2 | A serological test for antibodies against SARS-CoV-2 and to submit a self-report of gender, age, division, occupation, history of confirmed COVID-19, and history of working in the isolation area for COVID-19 management | Clinical Innovation Research Program of Guangzhou Regenerative Medicine and Health Guangdong Laboratory, the National Innovation Team Program, Recruitment Program of Leading Talent in Guangdong Province, and a Macao FDCT. |
| **Before-after comparison study** | | | | | | | |  |  |
| Lan et al. (39) | 17 March to 6 May | Before-after study | the Massachusetts statewide population and the HCWs of a Massachusetts community healthcare system | N95s, procedure masks | Public masking implemented: The policy included securing N95s for all direct care staff managing confirmed/suspect COVID-19 patients and providing procedure masks to all other clinical and non-clinical staff. | Pre-intervention, both the healthcare system and the state had strong increasing trends in the 7-day average COVID-19 incidence (Figure 1; Table 1) with overlapping slopes (0.96 (0.80 to 1.13) (standardized beta coefficient (95% CI)) and 0.99 (0.92 to 1.07), respectively). While the temporal trend among Massachusetts residents kept increasing with a similar slope in the intervention phase (0.99 (0.94 to 1.05)), that of the healthcare system decreased and was negative (−0.68 (−1.06 to −0.31)). During epidemic decline, following the states’ pandemic peak, both populations’ incidence showed overlapping negative slopes (−0.90 (−1.19 to −0.60) and −0.99 (−1.07 to −0.92)) | Universal masking was associated with a decreasing COVID-19 incidence trend among HCWs | No specific description detection case of COVID 19 | No financial support |
| Wang X b et al. (40) | Before intervention: March 1-24, 2020; Intervention: April 11-30, 2020 | Before-after study (retrospective) | 9850 tested HCWs | Universal masking | Universal Masking in a Health Care System | Universal masking at Mass General Brigham (MGB) was associated with a significantly lower rate of SARS-CoV-2 positivity among HCWs. During the pre-intervention period, the SARS-CoV-2 positivity rate increased exponentially from 0% to 21.32%, with a weighted mean increase of 1.16% per day and a case doubling time of 3.6 days (95% CI, 3.0-4.5 days). During the intervention period, the positivity rate decreased linearly from 14.65% to 11.46%, with a weighted mean decline of 0.49% per day and a net slope change of 1.65% (95% CI, 1.13%-2.15%; P < .001) more decline per day compared with the pre-intervention period | Universal masking at MGB was associated with a significantly lower rate of SARS-CoV-2 positivity among HCWs | HCWs have symptoms that were tested for SARS-CoV-2 with reverse transcriptase–polymerase chain reaction. | No financial support |
| Cernigliaro et al. (41) | February 2020 and March 2021 | Before- after study | 35 HCWs | FFP2, cap, googles, etc. | Rates of SARS-CoV-2 infection among the healthcare workers (HCW) of the Angiographic Suite. | Positive to SARS-CoV-2 (morbidity tax of 14.3%) was lower by using PPE and preventive measurements | Dedicated routes, elevators, establishing filter areas and a clear demarcation between clean and contaminated areas, dressing and undressing procedures, cleaning procedures and the obligation to always wear a surgical mask during the working shifts are essential to prevent in-hospital infection. | The HCWs are considered positive (molecular or rapid swab) if the monthly screening swab or the swabs for flu-like symptoms found positivity vs COVID-19 antigens | No financial support |
| Liu H et al.(42) | January  1 to February 18, 2020 | Before after study | 196/3120 health care workers were occupationally exposed to  COVID-19 | PPE such as mask, surgical mask, goggle | Provision and　proper use of  personal  protective  equipment (PPE) | Reducing cases of COVID 19 after implementation comprehensive measurement including wearing PPE | After the extensive promotion of comprehensive interventions, the occurrence of COVID-19 occupational exposure declined to 0.19% (P=0.000) | Nasopharyngeal swabs were collected three times from each participant during the two week quarantine period with SARS-CoV-2 nucleic acid testing by using RT PCR | Xiangyang City Guiding Science and Technology Plan Project of COVID-19 in 2020 |
|  | | | | | | | |  |  |
|  |  |  |  |  |  |  |  |  |  |
| **Case series** | | | | | | | |  |  |
| Rubbi et al. (43) | March 25, 2020 to May 05, 2020 | Case series (retrospective observational study) | 93 healthcare professionals tested positive for COVID-19 | Surgical mask, gloves, hair cap, goggles, gowns, water repellent coat, FFP2, shoes, FFP3 | Wearing PPE in health facility | At the time of the infection, most of the staff wore a surgical mask. Cough, asthenia, fever, anosmia, dysgeusia, and rhinitis were common symptoms. Asymptomatic percentage was about 10%. The self-perceived physical condition was high and improved during the observation period. | The diffusion rate of COVID-19 among healthcare workers is relatively low, probably due to the use of personal protective equipment. The distancing, also among colleagues, is a fundamental measure to reduce the possibility of infection. Symptoms are mild and can be controlled by surveillance measures. Constant contact with the organization is an essential strategy for promoting recovery of workers and reducing the spread of the virus within the healthcare organization. | HCW who reported COVID 19 was collected using an on-line questionnaire | No financial support |
| Yao et al. (44) | Tongji Hospital (from February 4 to March 10, 2020) and Union Hospital (from February 13 to March 12, 2020). | Case series (retrospective observational study of two centers) | 2 hospitals in Wuhan, n=202 | All operators wore inner and outer PPE such as Powered air-purifying respirators, N95, etc. | All intubating clinicians wore N95 respirators, surgical masks, eye protection goggles, and a protective coverall with hood and foot covers as inner layer protection. The outer layer of protection comprised a water-resistant full gown and either a face shield, or a full hood, either without a powered air-purifying respirator (PAPR) or with a PAPR with double pairs of gloves used in all intubations. | Adherence using N95, surgical masks, and goggles were lower rates of infections. While lower level adherence used a face shield, full hoods without a PAPR, PAPR, were at high risk of infection (P<0.001). | No evidence of cross infection in the anaesthesiologists who intubated the COVID-19 patients. | SARS-CoV-2 infection confirmed by reverse transcription-polymerase chain reaction (RT-PCR) testing for viral ribonucleic acid in respiratory samples, in combination with pulmonary chest CT findings | One author received funding from Fisher & Paykel |
|  |  |  |  |  |  |  |  |  |  |
| Moey et al. (45) | February to  July 2020 | case series | HCW in Clinic in Singapore | N95, visor, goggles, surgical mask, gown, gloves | Recommendation PPE for various zones in clinics in Singapore. Every zone has different PPE | Provision of adequate personal protection  equipment, zonal segregation of high-risk patients,  reduction in physical patient visits, effective staff  communication, implementation of self-declared  temperature monitoring and the maintenance of  sustainable workload and work hours of HCWs contributed  to the mitigation of COVID-19 infection risk among our staff | The multipronged intervention involving rapid implementation of novel or modification of public health policies, polyclinic layout, staff training, PPE intervention and various infection  control measures have largely mitigated the infection  risks of primary HCWs in this institution. | COVID-19 suspects are swabbed for PCR tests. The results are known within 24–48 hours. | No financial support |
| Alajmi et al. (46) | March 10, 2020 and June 24, 2020. | Case-control study (database research and interview) | Among 16,912 HCWs tested, 10.6% tested positive. | PPE | PPE adherence in COVID-19 designated facility vs non COVID-19 designated facility | Full personal protective equipment (PPE) adherence was 82% at COVID-19-designated facilities but only 68% at non-COVID-19 facilities. PPE use is less stringent in such settings. Risk of exposure and need for strict PPE must be stressed upon all HCWs in all settings. | Risk of exposure and need for strict PPE must be stressed upon all HCWs in all settings. | Testing for COVID-19 was performed using a deep nasopharyngeal and a concomitant throat swab by trained professionals. Validated RTPCR was performed at a single national reference lab to confirm infection. | No financial support |
| Modeling study | | | | | | | |  |  |
| Mizukoshi et al. (47) |  | Modeling study (simulation) |  | face masks and shields | HCW with intervention and without intervention for face mask, face shield and both | the relative risk (RR) of an overall risk for an HCW with an intervention vs. an HCW without intervention was 0.36–0.37, 0.02–0.03, and <4.0 × 10− 4 for a face mask, a face shield, and a face mask plus shield, respectively, in the likely median virus concentration in the saliva (102–104 PFU mL− 1), suggesting that personal protective equipment decreased the infection risk by 63%–>99.9%. In addition, the RR for a face mask worn by the patient, and a face mask worn by the patient plus increase of air change rate from 2 h− 1 to 6 h− 1 was <1.0 × 10− 4 and <5.0 × 10− 5, respectively in the same virus concentration in the saliva. | Importance of the use of a face mask and shield was confirmed. | RR of infection was calculated based on modeling | Kindai University  Support Project Against Covid-19 |

# Table S2. Summary of publications related to PPE (1 review, 5 cohort studies, 1 case-control studies, 2 cross-sectional studies , 1 before-after study and 2 case-series studies).

| **Study by** | **Date** | **Population** | **PPE types** | **PPE Intervention** | **Results** | **Conclusion** |
| --- | --- | --- | --- | --- | --- | --- |
| Gholami et al.  Systematic review and meta-analysis (2) | NA | Healthcare workers in 28 studies | PPE, N95, mask or respirators | Inadequate PPE, re-using PPE, facial mask, respirators with COVID-19 infection in HCW | Nguyen et al. stated reused PPE and inadequate PPE in documented COVID-19 patients has unadjusted HR 5.12 and 5.95, respectively. Ran et al. stated suboptimal hand hygiene before, after contact with patients and improper PPE has RR 3.1, 2.4, 2.8. Guo et al. stated that no use N95, wearing respirator or mask, hand hygiene practice has OR 5.20, 0.15, 0.85 | Reused and inadequate PPE associated with COVID-19 infection, suboptimal hand hygiene and improper PPE correlated with COVID-19 infection and mask, N95 protects HCW from infection. |
| El-Boghdadly et al.  Cohort study (prospective international multicenter study) (8) | 2020 | HCWs performing or assisting in tracheal intubation of COVID-19 patients across 17 countries (1718 participants) | PPE conforming to World Health Organization (WHO) recommended minimum standards for aerosol-generating procedures | PPE use conforming to WHO recommendation | Univariate analysis, PPE WHO standard HR=0.97 (0.63–1.51) | Around 1 in 10 healthcare workers involved in tracheal intubation of patients with suspected or confirmed COVID-19 subsequently reported a COVID-19 outcome. |
| Nguyen et al.  Cohort study (9) | 2020 | 99795 front-liner HCWs | Not specified (Just as PPE) | Availability of PPE (Adequate PPE, reuse PPE and inadequate PPE), and exposure of patients (no exposure, suspected and exposure to COVID-19) among HCW | Overall reused PPE and inadequate PPE (unadjusted HR 1.46, 1.32), no exposure to patients COVID-19 in reused PPE and inadequate PPE (unadjusted HR 0.96, 1.53), exposure to patients with suspected COVID-19 in reused and inadequate PPE (unadjusted HR 3.23, 1.87), exposure to patients documented COVID-19 (unadjusted HR 5.2, 5.95) | Reused and inadequate PPE associated with COVID-19 infection in frontline HCW. |
| Ran et al.  Cohort study (retrospective) (11) | NA | 72 HCW in Wuhan, 33 general group and 39 high risk group | Not specified (Just as PPE) | Improper PPE (yes/no) between high-risk group and general group. | 18 HCW in high risk got infection and 10 in general groups, while 15 in high risk no infection and 29 in general group. Improper use PPE has RR 2.82. | Improper use of PPE associated infection COVID-19 |
| Wang Q et al.  Cohort study (retrospective) (13) | 2014-2020 | 5442 neurosurgery department medical staff (120 cases COVID-19, 54 doctor and 66 nurses) | Not specified (Just as PPE) | adequate vs inadequate protection | Medical staff members who took inadequate protection had a higher risk of contracting infection than those using level 2 protection (RR=36.9) | Level 2 protection give strong prevention COVID-19 in HCW |
| Fletcher et al.  Longitudinal study (14) | August 17–September 4, 2020 (period 1) and during December 2–23, 2020 (period 2) | All HCWs were invited to participate (1385 in the first period and 1445 in the second period) | PPE: hospital policy. Masks including N95/CAPR, surgical masks. | Appropriate PPE use. Use of N95/CAPR or surgical masks. | Wearing appropriate PPE was not associated with seropositivity of COVID 19 infections (P=0.76). Wearing N95 mask or CAPRs was not associate with seropositivity COVID 19 (P=0.897) | Wearing appropriate PPE did not associate with seropositivity of COVID 19 in two periods. |
| Alajmi et al.  Case series (database research and interview) (46) | 2020 | Among 16,912 HCWs tested, 10.6% tested positive. | Not specified (Just as PPE) | PPE adherence in COVID-19 designated facility vs non COVID-19 designated facility | Full personal protective equipment (PPE) adherence was 82% at COVID-19-designated facilities but only 68% at non-COVID-19 facilities. PPE use is less stringent in such settings. Risk of exposure and need for strict PPE must be stressed upon all HCWs in all settings. | Risk of exposure and need for strict PPE must be stressed upon all HCWs in all settings. |
| Dev et al.  Case-control study (18) | 2020 | HCWs positive for SARS-CoV-2 infection (n=506) and those negative (n=253) | PPE (not specific) | Appropriate PPE during exposure patients and PPE use | Appropriate PPE during exposure patients and PPE use (OR 0.65, 0.63, respectively) | PPE protects HCW from COVID-19 |
| Jung et al.  Cross-sectional study (Letter to the Editor) (28) | NA | HCWs caring for patients (19 nurses caring for 2 patients) | Coveralls PPE | all participants wore PPEs | 15 (11%) revealed positive SARS-CoV-2 PCR results in the following areas: top of the head (26%), foot dorsum (26%), sole (16%), wrist (5%), and abdomen (5%). No SARS-CoV-2 RNA was detected on the neck and back. The top of the head, foot dorsum, and sole were more frequently contaminated than the neck, wrist, abdomen, and back (23% [13/57] vs. 3% [2/76], P < 0.001 by Fishers’ exact test). | Wearing of PPE that fully covers the head and feet, as well as cautious doffing procedures. |
| Moreno-Casbas Cross sectional (26) | 2020 | 41239 (analysis for 2230) | Masks, gloves, gowns, alcohol, goggles, shower etc. | Frequently used of PPE | The perception about the availability of protective measures as «always/frequently» were: FPP1 mask 57.3%, gloves 89.5%, soap 95% and hydro alcoholic solution 91.5%. In PPE, FPP2, FPP3 mask, goggles and disposable gowns at around 50% | Preliminary data are presented, with variability in the response rate by Autonomous Region. Healthcare professionals infected by SARS-CoV-2 identified the management of the chain of infection transmission, the use and adequacy of protective equipment, as well as the effectiveness of handwashing as factors related to the transmission of the virus among professionals. |
| Liu H et al. (42)  (Before after study) | January 1 to February 18, 2020 | 196/3120 health care workers were occupationally exposed to COVID-19 | PPE such as mask, surgical mask, goggle | Provision and proper use of personal protective equipment(PPE) | Reducing cases of COVID 19 after implementation comprehensive measurement including wearing PPE | After the extensive promotion of comprehensive interventions, the occurrence of COVID-19 occupational exposure declined to 0.19% (P=0.000) |
| Rubbi et al.  Case series (retrospective observational study) (43) | 2020 | 93 HCWs tested positive for COVID-19 | Surgical mask, gloves, hair cap, goggles, gowns, water repellent coat, FFP2, shoes, FFP3 | Wearing PPE in health facility | At the time of the infection, most of the staff wore a surgical mask. Cough, asthenia, fever, anosmia, dysgeusia, and rhinitis were common symptoms. Asymptomatic percentage was about 10%. The self-perceived physical condition was high and improved during the observation period. | The diffusion rate of COVID-19 among healthcare workers is relatively low, probably due to the use of personal protective equipment. The distancing, also among colleagues, is a fundamental measure to reduce the possibility of infection. |

# Table S3. Summary of publications related to facial masks and other protective respiratory equipment (8 reviews, 1 cohort study, 8 case-control studies, 14 cross-sectional studies, 3 before and after comparison studies, 2 case-series studies and 1 modeling study).

| **Study by** | **Date** | **Population** | **PPE types** | **PPE Intervention** | **Result** | **Conclusion** |
| --- | --- | --- | --- | --- | --- | --- |
| Chu et al.  Systematic review and meta-analysis (1) | 2020 | 44 studies but only 4 studies about COVID-19 in HCW | face mask, eye protection (goggles, face shield) | Comparison between face mask vs no face mask and eye protection vs no eye protection | Face mask vs no face mask (aOR=0.15, unadjusted RR=0.34), eye protection vs no eye protection (unadjusted RR=0.34) | Face mask and eye protection protect from viral infection than no use face mask and eye protection |
| Liang et al.  Systematic review and meta-analysis (3) | 2020 | 21 studies, but only one study about COVID-19 in HCW | N95 | N95 vs no face mask | Only one paper by Wang et al. (2020) from this paper by Liang et al. that eligible, OR was 0.04 | N95 protected HCW from COVID-19 |
| Licina et al.  Systematic review (5) | 2020 | Randomized controlled trials, non-randomized controlled trials, and observational studies. 10 full-text studies included | air-purifying respirator (PAPR) | PAPR versus other appropriate respiratory protection. | Two on-field studies reported no difference in the rates of healthcare workers performing airway procedures during the care of critical patients with SARS-CoV-2. A single simulation trial reported a lower level of cross-contamination of participants using PAPR compared to alternative respiratory protection. Field observational studies do not indicate a difference in healthcare worker infection utilizing PAPR devices versus other compliant respiratory equipment. | Field observational studies do not indicate a difference in healthcare worker infection utilizing PAPR devices versus other compliant respiratory equipment |
| Abboah-Offei et al.  Systematic review (rapid review) (6) | 2020 | 27 out 58 studies conducted amongst HCW wearing face masks | Surgical masks , N95 and surgical masks , all types , paper, cotton and gauze masks , surgical masks and cloth masks , N95, surgical mask and paper mask (n=1) | Mask vs no mask, N95 vs surgical mask/medical face mask. | Study from Batozsko et al. medical mask vs N95, OR: 1.06; Chu et al. face mask vs no face mask, aOR: 0.15, unadjusted RR: 0.34; eye protection, unadjusted RR: 0.34; Liang et al., Wang et al., wearing mask vs no wearing mask, OR 0.04; Wang et al. A, inadequate protection vs level 2 protection, RR 36.9; Wang et al. B, no mask group vs N95, aOR: 464.82. | Medical masks offered protection in HCW. |
| Tian et al.  Systematic review and meta-analysis (4) |  | 54 studies (191,004 Healthcare workers) | Mask, gown, glove, N95, face protection | Gloves vs no gloves, surgical mask vs no surgical mask, gown vs no gown, N95 vs no N95, face protection vs no face protection | Meta-analysis's OR from this study found wearing gloves, gown, surgical mask, N95, face protection and hand hygiene is 0.48, 0.46, 0.37, 0.32, 0.41, and 0.54. Gloves vs no gloves (Study from Barrett 2020, OR 1.79; Chatterjee 2020, OR 0.38; Heinzerling 2020, OR 4.40; Subtotal OR 1.03), gown vs no gown (study from Barrett 2020, OR 1.79; Chatterjee 2020, OR 0.71; subtotal OR 1.07), surgical mask vs no surgical mask study from Heinzerling 2020, OR 0.02; subtotal OR 0.02; N95 vs no N95 (study from Guo 2020, OR 0.31; Wang Q 2020, OR 0.03; Wang X 2020, OR 0.04; subtotal, OR 0.08), face protection and no face protection (Study from Chatterjee 2020, OR 0.81; subtotal OR 0.81) | Wearing gloves, gown, surgical mask, N95, face protection protect from COVID-19 infection in HCW |
| Calo et al.  Review (scoping review) (7) | 2020 | 43 studies, 14 webpages and 5 ongoing trials | N95 respirator and isolation gown, gloves | Survey of PPE contamination | In a study performed in healthcare facilities, the most contaminated objects were self-service printers (20.0%), desktops/keyboards (16.8%) and doorknobs (16.0%); both hand sanitizer dispensers (20.3%) and gloves (15.4%) were the most contaminated PPE. | This scoping review summarizes the evidence on the burden, risk assessment, surveillance and management of HWs exposed to SARS-CoV-2. |
| Sims et al.  Cohort study (prospective) (12) | 2020 | Participants about　43000 blood samples were collected from20614 people working in hospital | Surgical masks, N95 mask, Powered Air Purifying Respirator (PAPR) | HCW who wore N95/PAPR masks when exposed to COVID-19 | A total of 1,818 (8.8%) participants were seropositive between April 13 and May 28, 2020. Among the seropositive individuals, 44% reported that they were asymptomatic during the month prior to blood collection. Healthcare roles such as phlebotomy, respiratory therapy, and nursing/nursing support exhibited significantly higher seropositivity. Among participants reporting direct exposure to a COVID-19 positive individual, those wearing an N95/PAPR mask had a significantly lower seropositivity rate (10.2%) compared to surgical/other masks (13.1%) or no mask (17.5%). | Direct contact with COVID-19 patients increased the likelihood of seropositivity among employees but study participants who wore a mask during COVID-19 exposures were less likely to be seropositive. |
| Chatterjee et al.  Case-control study (15) | 2020 | 378 case and 373 control HCW in India | PPE, glove, gown, cap, shoe, face shield | PPE usage (never used, always used), mask (any mask, no mask), cap (yes, no), gown (yes, no), shoe (yes, no), face shield (yes, no), gloves (yes, no) | Never used vs used (OR=3.72), any mask vs no mask (OR=0.35), cap yes vs no (OR=0.7), gown yes vs no (0.62), shoes yes vs no (OR=1.05), face shield yes vs no (OR=0.81), gloves yes vs no (OR=0.38) | Never used PPE, using shoes associated with COVID-19 infection; any mask, cap, gown, face shield, glove protect from infection |
| Contejean et al.  Case-control study (16) | 2020 | 336 cases and 228 controls (physician, paramedic staff, administrative staff, other employees) | medical mask | Exposure to patients; exposure to colleagues | Exposure to patients (close contact patients without PPE and with PPE) has OR 1.22 and 0.28; while exposure to colleagues by using mask frequently, spending time without mask and close contact without mask has OR 0.45, 2.77 and 2.8 | PPE and medical mask protect HCW from COVID-19 |
| Farhat et al.  Case control study (23) | 4/2021 | 6 nurses, 3 assistant nurses, 2 residents, 1 fellow, and 1 staff | air impermeable plastic gown with a head-to-toe cover, an N95 mask, gloves, and a face shield. | examined as to whether the cotton gown could be used instead of PPE. After three months, all medical staff were evaluated for clinical signs of COVID-19, such as fever, cough, nausea, and headache. | During these three months, no symptoms or absence due to illness were observed in the staff. | Cotton surgical gown protected the staff against COVID-19. Accordingly, the cotton surgical gown can be used in medical centers to replace PPE sets containing an air-impermeable plastic gown with a head-to-toe cover. |
| Guo et al.  Case-control study (19) | 2019-2020 | 26 orthopedic surgeons from 8 hospitals in Wuhan (case 24, control 48) | N95 respirator | Usage of N95 (yes/no), wearing respirator or mask all of time (yes/no) | No use of N95 and wearing mask all of time has OR 5.20 and 0.15 | Respirator N95 can protect orthopedic surgeons from COVID-19 |
| Heinzerling et al.  Case-control study (20) |  | 37 HCW in California | Gloves, mask | gloves vs no gloves; mask vs no mask | Gloves vs. no gloves (OR=4.40; mask vs no mask (OR=0.02) | Gloves associated with infection SARS-CoV2 and wearing mask protects from SARS-CoV2 infection. |
| Lai et al.  Case-control study (21) | 2020 | 325 (infected 151) | mask, glove, goggle, suit, gowns, shoe covers, hats | mask=: N95/KN95, medical protective mask patient = surgical masks | When performing general operations on confirmed or suspected patients, the use of protective equipment including the effectiveness of masks (p < 0.001), gloves (p < 0.001); and the use of gloves (p < 0.001), suits (p < 0.001), gowns (p < 0.001), shoe covers (p < 0.001), and hats (p < 0.001) were protective factors. The use of protective equipment was a protective factor in most cases. Negative emotions and dissatisfaction to the hospital response were associated with the increased risk of infection | HCWs have an outbreak in concentrated departments in the hospital. The COVID-19 infection is associated with the effectiveness and the use of PPE, emotions and satisfactions to hospital responses. |
| Wang X a et al.  Case-control study (letter to editor) (22) | 2020 | 493 medical staff at six departments (Respiratory, ICU, Infectious Disease, Hepatobiliary Pancreatic Surgery, Trauma and Microsurgery and Urology) | N95 | N95 vs no face mask | Among the 493 medical staff, none of the 278 staff (56 doctors and 222 nurses) in the N95 group became infected, but 10 of 213 staff (77 doctors and 136 nurses) from the no-mask group were confirmed as infected. Infection rate for medical staff was significantly increased in the no-mask group compared with the N95 respirator group (difference: 4.65%, (95% confidence interval: 1.75%-infinite); P<2.2e-16) (adjusted odds ratio: 464.82, (95% confidence interval: 97.73e infinite); P<2.2e-16). | N95 protected HCW from COVID-19 |
| Coppeta et al.  Case-control study (monitoring study) (17) | NA | 1006 HCWs identified as contacts of COVID-19 cases | gloves, masks, eye protection | wearing masks/gloves/eye protection | Regarding the use of personal protective equipment (PPE), only the use of facial masks was inversely related to the chance of becoming infected (p<0.01) | The use of facial masks should be implemented even in settings where known patients with COVID-19 are not present |
| Aranaz-Andrés et al.  Cross-sectional study (24) | 2020 | Of 5223 healthcare workers, 167 included study groups (PCR based) and 156 comparison groups (No PCR) | mask, goggle, gloves, gown | Correct use of mask, goggles, gloves, gown, used hand sanitizer and soap in suboptimal and optimal score in 132 HCW lab confirmed SARS CoV2 vs comparison | Suboptimal use mask, goggles, gloves, gown, hand sanitizer and soap were OR 0.99, 1.6, 1.2, 1.4, 0.93, 0.71 | suboptimal use of goggles, gloves, gown associated with COVID-19 in HCW, while suboptimal wearing mask, hand sanitizer and soap are a protective factor. |
| Boffetta et al.  Cross-sectional study (25) | 2020 | 10,654 Italian HCWs (physician, nurse, health care assistant, technician, others) | Any mask, face shield, gloves, gown, any PPE | Wearing PPE and SARS CoV2 infection in HCW | Any mask OR 0.68; face shield OR 1.22; gloves OR 0.72; gown OR 1.39; and any PPE, OR 0.94. | Wearing mask, gloves, any PPE protects from SARS-CoV2, while face shield and gown associated with SARS-CoV2 |
| Botti et al.  Cross sectional (36) | 2/2021 | 44 hematology centers | surgical mask, FFP2, FFP3,TNT gown, Water resistant gown, Visor, Safety glasses | Some questions about covid-19 including PPE | Some healthcare professionals were asked to wear PPE in caring hematology patients that found PPE protected HCWs from the infection. | As hematology HCPs apply COVID 19 infection control measures in their daily practice such as wearing PPE, this may have allowed them to contain the spread of the virus in their setting more effectively. |
| Kindgen-Mills et al.  Cross sectional (37) | June 16th to July 2nd 2020 | 516 physicians from all over Germany who attended certified registered training courses for intensive care or emergency medicine in the  city of Arnsberg in Northrhine-Westfalia | FFP2, FFP3, goggles and face shield | Wearing respiratory protective measure and SARS-CoV 2 infection examined by ELISA | No statistically significant correlation between the availability of FFP2/FFP3 masks and SARS-CoV-2 infection (p=0.99). no statistically significant correlation between the availability of eye protective devices and SARS-CoV-2 (p=0.99) infection | Wearing masks and eye protectors provided protection to HCWs from CoVID 19 infection. |
| Hou et al.  Cross sectional study (38) | 9/2020 | 8529 healthcare workers （medical teams aiding Hubei, local healthcare workers in Wuhan and Jingzhou of Hubei Province） | Level 1 protection （including disposable caps, surgical masks, white coats, and hand hygiene. N95/FFP (filtering facepiece, FFP), isolation gowns, and disposable gloves are used when necessary）  Level 2 protection (in addition to PPE for Level 1 protection, goggles and fullface shields, long sleeved, fluid repellent gowns, and shoe covers are used).  Level 3 protection (the addition to PPE for Level 2, an isolation gown on top of the disposable coverall and potential use of a positive pressure helmet) | level 0 PPE protection for HCWs working in the non-medical area. Level 1 PPE protection for HCWs working in the non-isolation medical area. Level 2/3 PPE protection for HCWs working in the isolation medical area | 1. Serial tests for SARS-CoV-2 RNA and tests for SARS-CoV-2 immunoglobulin M and G after the 6-8 week mission revealed a zero cumulative attack rate in medical teams aiding Hubei. 2. The seropositivity for SARS-CoV-2 antibodies (IgG, IgM, or both IgG/IgM positive) was 3.4% (53 out of 1571) in local healthcare workers from Wuhan with Level 2/3 PPE working in isolation areas and 5.4% (126 out of 2336) in healthcare staff with Level 1 PPE working in non-isolation medical areas, respectively. | Adequate training PPE can protect medical personnel against SARS-CoV-2 |
| Tabah et al.  Cross-sectional study (web-based survey) (32) | 2020 | 2711 responses from 1797 physicians, 744 nurses, and 170 allied HCW. | FFP2, N95, PAPR | Wearing PPE during routine care | For routine care, most (1557, 58%) reportedly used FFP2/N95 masks, waterproof long sleeve gowns (1623; 67%), and face shields/visors (1574; 62%). Powered Air-Purifying Respirators were used routinely and for intubation only by 184 (7%) and 254 (13%) respondents, respectively. Surgical masks were used for routine care by 289 (15%) and 47 (2%) for intubations. At least one piece of standard PPE was unavailable for 1402 (52%), and 817 (30%) reported reusing single-use PPE. PPE was worn for a median of 4 h. | PPE practices availability, and confidence in adequacy to provide protection among HCWs at the frontlines of the COVID-19 pandemic. Respondents report widespread shortages and reuse of single-use PPE items. |
| Tekalegn et al.  Cross-sectional study (33) | 2020 | 98 out 368 HCW daily contact directly with patients. | Ffacemask | Correct use of facemask: perform hand hygiene before/after wearing masks, reuse masks, etc. | The level of overall correct use of facemask was 10.1% (95% CI: 7.4–13.6). | Health professionals’ practice regarding the correct use of facemask in the context of COVID-19 prevention is very low. |
| Davido et al.  Cross-sectional study (27) | 2020 | 99 hospital staff members. Of them, 28 positive PCR test for COVID-19 and 71 negatives. | Mask | Systematic used mask (yes/no) and meeting without and with mask among 99 HCW | Systematic used mas, OR 21. Meeting without mask, OR 10.3 | Mask protects HCW from COVID-19 infection |
| Liu M et al.  Cross-sectional study (30) | 2020 | 420 healthcare professionals (116 doctors and 304 nurses) All healthcare professionals had performed at least one aerosol generating procedure. | Mask: N95 respirator, Surgical mask, medical suit, Isolation gown, Apron, Gloves, Eye protection, Hair cover | Those PPE in ward and during AGP procedure | None of the nasopharyngeal swabs collected from the participants tested positive for nucleic acids on the reverse transcriptase polymerase chain reaction assay for SARS-CoV-2. None of the serum samples of participants tested positive for SARS-CoV-2 specific IgM or IgG antibodies (95% confidence interval 0.0 to 0.7%). | Despite being at high risk of exposure, healthcare professionals who were appropriately protected did not contract infection or develop protective immunity against SARS-COV-2. |
| Oksanen et al.  Cross-sectional and prospective study (10) | 2020 | 1072 volunteers: HCWs at the Helsinki University Hospital. | FFP2/3 respirators and surgical masks, etc. | the use of FFP2/3 and masks | In 14 cases (63.6%), occupational infections occurred while using a surgical mask, and all infections originating from patients occurred while using a surgical mask or no mask at all. No occupational infections were found while using an FFP2/3 respirator and following aerosol precautions. In this study, none of the ICU HCWs got infected while using the recommended PPE (a FFP3 respirator [or FFP2 if FFP3 was not available], a pair of gloves, a long-sleeved fluid repellent gown, hair protection and eye protection). | the use of FFP2/3 respirators in all patient contacts with confirmed or suspected COVID-19, along with the use of universal masking |
| Schmitz et al.  Cross-sectional study (31) | 2020 | 164 ED staff workers | Glove, surgical hat, eye protection, surgical mask, FFP1, FFP2, N95 | 42 questions about PPE-usage and infection rate of COVID-19. | In 13 hospitals, an FFP2 (filtering facepiece particles >94% aerosol filtration) mask or equivalent and eye protection was worn for all contacts with patients with suspected or confirmed SARS-CoV-2 during the whole study period. The unadjusted staff infection rate was higher in these hospitals [7.3 (3.4–11.1) vs. 4.0 (1.9–6.1)%, absolute difference+3.3%]. After adjusting for hospital testing policy, type of PPE was not associated with incidence of COVID 19 infections among ED staff (P=0.40) | The use of high-level PPE (FFP2 or equivalent and eye protection) by ED personnel during all contacts with patients with suspected or confirmed SARS-CoV-2 does not seem to be associated with a lower infection rate of ED staff compared to lower-level PPE use. |
| Khalil et al.  Cross-sectional study (29) | 2020 | 98 COVID-19 positive physicians and 92 negative physicians | N95 mask, single-use gloves, protective face-shields/goggles, disposable gown, water-proof apron | wearing N95, wearing PPE single-use gloves, protective face-shields/goggles, disposable gown, water-proof apron, proper handling of PPE, proper hand-hygiene during different patient care, and decontamination of the surroundings of the patient | Formal training on PPE use (OR = 1.667; CI: 0.890–3.121) mildly increased the odds of being infected, which was not significant. Wearing the N95 mask was significantly associated with a low probability of COVID-19 infection (OR = 0.373; CI: 0.159–0.873). Single-use gloves, Face-shield/goggles, Disposable gown, Water-proof apron: none of these results were statistically significant | The use of face shields/goggles and N95 masks and decontamination of the patient’s surroundings may give protection against COVID-19. Additionally, reusing medical gowns should be avoided as much as possible. |
| Wee et al.  Cross-sectional survey (letter to editor) (34) | 2020 | 10000 HCWs, 1780 of which were ancillary HCWs in Singapore | N95 respirator, disposable gown and gloves, and eye protection and universal masking | Wearing N95 mask, gown, glove, eye protection PPE and universal masking between HCWs and ancillary workers | Although PPE usage was high among all HCWs, ancillary workers had lower odds of PPE adherence during contact episodes with suspected COVID-19 cases, compared with other HCWs Odd ratio for nurses wore N95, gown, gloves and eye protection is 1.01, 0.74, 0.77, 0.98; allied health is 1, 0.86, 0.86, 0.96 and ancillary HCW is 0.72, 0.64, 0.65, 0.83, respectively. | Although overall rates of infection among HCWs were low, rates of infection were higher in ancillary HCWs compared with medical and nursing staff (0.32%, 5/1548, versus 0.10%, 5/5101; incidence-rate ratio 3.29, p 0.04). |
| Zhao et al.  Cross-sectional study (35) | 2020 | 960 HCWs in over 37 Hubei hospitals | Gloves, medical mask, N95/FFP2, face shield or goggles, isolation gown, medical protective uniform, positive pressure headgear | Wearing PPEs. 98.6% of them showed high levels of adherence to PPE protocols. | Negative results of RT-PCR tests in all participants with a median 40-day exposure duration along with negative results of antibody tests in 70.0% of participants showing that they were never infected indicated that PPE is an efficacious measure to durably contain the nosocomial transmission of SARS-CoV-2. | PPE is an efficacious measure SARS CoV2 in HCW. |
| Cernigliaro et al.  Before after study (41) | February 2020 and March 2021 | 35 HCWs | FFP2, cap, googles, etc. | Rates of SARS-CoV-2 infection among the healthcare workers (HCW) of the Angiographic Suite. | Positive to SARS-CoV-2 (morbidity tax of 14.3%) was lower by using PPE and preventive measurements) | Dedicated routes, elevators, establishing filter areas and a clear demarcation between clean and contaminated areas, dressing and undressing procedures, cleaning procedures and the obligation to always wear a surgical mask during the working shifts are essential to prevent in-hospital infection. |
| Lan et al.  Before-after study (39) | 2020 | Massachusetts statewide population and the HCWs of a Massachusetts community healthcare system | N95s, procedure masks | Public masking implemented: The policy included securing N95s for all direct care staff managing confirmed/suspect COVID-19 patients and providing procedure masks to all other clinical and non-clinical staff. | Pre-intervention, both the healthcare system and the state had strong increasing trends in the 7-day average COVID-19 incidence (Figure 1; Table 1) with overlapping slopes (0.96 (0.80 to 1.13) (standardized beta coefficient (95% CI)) and 0.99 (0.92 to 1.07), respectively). While the temporal trend among Massachusetts residents kept increasing with a similar slope in the intervention phase (0.99 (0.94 to 1.05)), that of the healthcare system decreased and was negative (−0.68 (−1.06 to −0.31)). During epidemic decline, following the states’ pandemic peak, both populations’ incidence showed overlapping negative slopes (−0.90 (−1.19 to −0.60) and −0.99 (−1.07 to −0.92)) | Universal masking was associated with a decreasing COVID-19 incidence trend among HCWs |
| Wang X et al. b  (Before-after study) (40) | 2020 | 9850 tested HCWs | Universal masking | Universal Masking in a Health Care System | Universal masking at Mass General Brigham (MGB) was associated with a significantly lower rate of SARS-CoV-2 positivity among HCWs. During the pre-intervention period, the SARS-CoV-2 positivity rate increased exponentially from 0% to 21.32%, with a weighted mean increase of 1.16% per day and a case doubling time of 3.6 days (95% CI, 3.0-4.5 days). During the intervention period, the positivity rate decreased linearly from 14.65% to 11.46%, with a weighted mean decline of 0.49% per day and a net slope change of 1.65% (95% CI, 1.13%-2.15%; P < .001) more decline per day compared with the pre-intervention period | Universal masking at MGB was associated with a significantly lower rate of SARS-CoV-2 positivity among HCWs |
|  |  |  |  |  |  |  |
| Yao et al.  Case series (retrospective observational study of two centers) (44) | 2020 | 2 hospitals in Wuhan (n=202) | All operators wore inner and outer PPE such as Powered air-purifying respirators, N95, etc. | . All intubating clinicians wore N95 respirators, surgical masks, eye protection goggles, and a protective coverall with hood and foot covers as inner layer protection. The outer layer of protection  comprised a water-resistant full gown and either a face shield, or a full hood, either without a powered airpurifying respirator (PAPR) or with a PAPR with double pairs of gloves used in all intubations. | Adherence using N95, surgical masks, and goggles were lower rates of infections. While lower level adherence used a face shield, full hoods without a PAPR, PAPR, were at high risk of infection (P<0.001). | No evidence of cross infection in the anaesthesiologists who intubated the COVID-19 patients. |
| Moey et al.  case series (45) | February to  July 2020 | HCW in Clinic in Singapore | N95, visor, goggles, surgical mask, gown, gloves | Recommendation PPE for various zones in clinics in Singapore. Every zone has different PPE | Provision of adequate personal protection equipment, zonal segregation of high-risk patients, reduction in physical patient visits, effective staff communication, implementation of self-declared temperature monitoring and the maintenance of sustainable workload and work hours of HCWs contributed to the mitigation of COVID-19 infection risk among our staff | The multipronged intervention involving rapid implementation of novel or modification of public health policies, polyclinic layout, staff training, PPE intervention and various infection control measures have largely mitigated the infection risks of primary HCWs in this institution. |
| Mizukoshi et al (modeling study) (47) |  | Modeling study (simulation) | face masks and shields | HCW with intervention and without intervention for face mask, face shield and both | the relative risk (RR) of an overall risk for an HCW with an intervention vs. an HCW without intervention was 0.36–0.37, 0.02–0.03, and <4.0 × 10− 4 for a face mask, a face shield, and a face mask plus shield, respectively, in the likely median virus concentration in the saliva (102–104 PFU mL− 1), suggesting that personal protective equipment decreased the infection risk by 63%–>99.9%. In addition, the RR for a face mask worn by the patient, and a face mask worn by the patient plus increase of air change rate from 2 h− 1 to 6 h− 1 was <1.0 × 10− 4 and <5.0 × 10− 5, respectively in the same virus concentration in the saliva. | Importance of the use of a face mask and shield was confirmed. |

**References**

1. Chu DK, Akl EA, Duda S, Solo K, Yaacoub S, Schünemann HJ. Physical distancing, face masks, and eye protection to prevent person-to-person transmission of SARS-CoV-2 and COVID-19: a systematic review and meta-analysis. Lancet. 2020;395(10242):1973-87.

2. Gholami M, Fawad I, Shadan S, Rowaiee R, Ghanem H, Hassan Khamis A, et al. COVID-19 and healthcare workers: A systematic review and meta-analysis. Int J Infect Dis. 2021;104:335-46.

3. Liang M, Gao L, Cheng C, Zhou Q, Uy JP, Heiner K, et al. Efficacy of face mask in preventing respiratory virus transmission: A systematic review and meta-analysis. Travel Med Infect Dis. 2020;36:101751.

4. Tian C, Lovrics O, Vaisman A, Chin KJ, Tomlinson G, Lee Y, et al. Risk factors and protective measures for healthcare worker infection during highly infectious viral respiratory epidemics: a systematic review and meta-analysis. Infect Control Hosp Epidemiol. 2021:1-102.

5. Licina A, Silvers A, Stuart RL. Use of powered air-purifying respirator (PAPR) by healthcare workers for preventing highly infectious viral diseases-a systematic review of evidence. Syst Rev. 2020;9(1):173.

6. Abboah-Offei M, Salifu Y, Adewale B, Bayuo J, Ofosu-Poku R, Opare-Lokko EBA. A rapid review of the use of face mask in preventing the spread of COVID-19. Int J Nurs Stud Adv. 2021;3:100013.

7. Calò F, Russo A, Camaioni C, De Pascalis S, Coppola N. Burden, risk assessment, surveillance and management of SARS-CoV-2 infection in health workers: a scoping review. Infect Dis Poverty. 2020;9(1):139.

8. El-Boghdadly K, Wong DJN, Owen R, Neuman MD, Pocock S, Carlisle JB, et al. Risks to healthcare workers following tracheal intubation of patients with COVID-19: a prospective international multicentre cohort study. Anaesthesia. 2020;75(11):1437-47.

9. Nguyen LH, Drew DA, Joshi AD, Guo CG, Ma W, Mehta RS, et al. Risk of COVID-19 among frontline healthcare workers and the general community: a prospective cohort study. medRxiv. 2020.

10. Oksanen LAH, Sanmark E, Oksanen SA, Anttila VJ, Paterno JJ, Lappalainen M, et al. Sources of healthcare workers' COVID‑19 infections and related safety guidelines. Int J Occup Med Environ Health. 2021;34(2):239-49.

11. Ran L, Chen X, Wang Y, Wu W, Zhang L, Tan X. Risk Factors of Healthcare Workers With Coronavirus Disease 2019: A Retrospective Cohort Study in a Designated Hospital of Wuhan in China. Clin Infect Dis. 2020;71(16):2218-21.

12. Sims MD, Maine GN, Childers KL, Podolsky RH, Voss DR, Berkiw-Scenna N, et al. COVID-19 seropositivity and asymptomatic rates in healthcare workers are associated with job function and masking. Clin Infect Dis. 2020.

13. Wang Q, Huang X, Bai Y, Wang X, Wang H, Hu X, et al. Epidemiological characteristics of COVID-19 in medical staff members of neurosurgery departments in Hubei province: A multicentre descriptive study. medRxiv. 2020:2020.04.20.20064899.

14. Fletcher JJ, Feucht EC, Hahn PY, McGoff TN, Dehart DJ, Mortada ME, Grifka R. Healthcare-acquired coronavirus disease 2019 (COVID-19) is less symptomatic than community-acquired disease among healthcare workers. Infect Control Hosp Epidemiol. 2022;43(4):490-496.

15. Chatterjee P, Anand T, Singh KJ, Rasaily R, Singh R, Das S, et al. Healthcare workers & SARS-CoV-2 infection in India: A case-control investigation in the time of COVID-19. Indian J Med Res. 2020;151(5):459-67.

16. Contejean A, Leporrier J, Canouï E, et al. Transmission Routes of Severe Acute Respiratory Syndrome Coronavirus 2 Among Healthcare Workers of a French University Hospital in Paris, France. Open Forum Infect Dis. 2021;8(3):ofab054.

17. Coppeta L, Somma G, Ippoliti L, Ferrari C, D'Alessandro I, Pietroiusti A, et al. Contact Screening for Healthcare Workers Exposed to Patients with COVID-19. Int J Environ Res Public Health. 2020;17(23).

18. Dev N, Meena RC, Gupta DK, Gupta N, Sankar J. Risk factors and frequency of COVID-19 among healthcare workers at a tertiary care centre in India: a case-control study. Trans R Soc Trop Med Hyg. 2021.

19. Guo X, Wang J, Hu D, Wu L, Gu L, Wang Y, et al. Survey of COVID-19 Disease Among Orthopaedic Surgeons in Wuhan, People’s Republic of China. JBJS. 2020;102(10):847-54.

20. Heinzerling A, Stuckey MJ, Scheuer T, Xu K, Perkins KM, Resseger H, et al. Transmission of COVID-19 to Health Care Personnel During Exposures to a Hospitalized Patient - Solano County, California, February 2020. MMWR Morb Mortal Wkly Rep. 2020;69(15):472-6.

21. Lai X, Zhou Q, Zhang X, Tan L. What influences the infection of COVID-19 in healthcare workers? J Infect Dev Ctries. 2020;14(11):1231-7.

22. Wang X, Pan Z, Cheng Z. Association between 2019-nCoV transmission and N95 respirator use. J Hosp Infect. 2020 a;105(1):104-5.

23. Farhat AS, Mohamadzadeh A, Saeidi R, Yeganeh Khorasani N. Does the Use of Personal Protection Equipment for the Medical Staff Working with Patients with Coronavirus Disease 2019 Need to be Revised? Iranian Journal of Neonatology. 2021 Apr: 12(2).

24. Aranaz-Andrés JM, McGee-Laso A, Galán JC, Cantón R, Mira J, On Behalf Of The Team Of Work C. Activities and Perceived Risk of Transmission and Spread of SARS-CoV-2 among Specialists and Residents in a Third Level University Hospital in Spain. Int J Environ Res Public Health. 2021;18(6).

25. Boffetta P, Violante F, Durando P, De Palma G, Pira E, Vimercati L, et al. Determinants of SARS-CoV-2 infection in Italian healthcare workers: a multicenter study. Sci Rep. 2021;11(1):5788.

26. Moreno-Casbas MT. Factors related to SARS-CoV-2 infection in healthcare professionals in Spain. The SANICOVI project. Enferm Clin. 2020;30(6):360-70.

27. Davido B, Gautier S, Riom I, Landowski S, Lawrence C, Thiebaut A, et al. The first wave of COVID-19 in hospital staff members of a tertiary care hospital in the greater Paris area: A surveillance and risk factors study. Int J Infect Dis. 2021;105:172-9.

28. Jung J, Kim JY, Bae S, Cha HH, Kim EO, Kim MJ, et al. Contamination of personal protective equipment by SARS-CoV-2 during routine care of patients with mild COVID-19. J Infect. 2020;81(2):e165-e7.

29. Khalil MM, Alam MM, Arefin MK, Chowdhury MR, Huq MR, Chowdhury JA, et al. Role of Personal Protective Measures in Prevention of COVID-19 Spread Among Physicians in Bangladesh: a Multicenter Cross-Sectional Comparative Study. SN Compr Clin Med. 2020:1-7.

30. Liu M, Cheng SZ, Xu KW, Yang Y, Zhu QT, Zhang H, et al. Use of personal protective equipment against coronavirus disease 2019 by healthcare professionals in Wuhan, China: cross sectional study. Bmj. 2020;369:m2195.

31. Schmitz D, Vos M, Stolmeijer R, Lameijer H, Schönberger T, Gaakeer MI, et al. Association between personal protective equipment and SARS-CoV-2 infection risk in emergency department healthcare workers. Eur J Emerg Med. 2020.

32. Tabah A, Ramanan M, Laupland KB, Buetti N, Cortegiani A, Mellinghoff J, et al. Personal protective equipment and intensive care unit healthcare worker safety in the COVID-19 era (PPE-SAFE): An international survey. J Crit Care. 2020;59:70-5.

33. Tekalegn Y, Sahiledengle B, Bekele K, Tesemma A, Aseffa T, Teferu Engida Z, et al. Correct Use of Facemask Among Health Professionals in the Context of Coronavirus Disease (COVID-19). Risk Manag Healthc Policy. 2020;13:3013-9.

34. Wee LE, Sim JXY, Conceicao EP, Aung MK, Ng IM, Ling ML. Re: 'Personal protective equipment protecting healthcare workers in the Chinese epicenter of COVID-19' by Zhao et al. Clin Microbiol Infect. 2020;26(12):1719-21.

35. Zhao Y, Liang W, Luo Y, Chen Y, Liang P, Zhong R, et al. Personal protective equipment protecting healthcare workers in the Chinese epicentre of COVID-19. Clin Microbiol Infect. 2020;26(12):1716-8.

36. Botti S, Serra N, Castagnetti F, et al. Hematology Patient Protection During the COVID-19 Pandemic in Italy: A Nationwide Nursing Survey. Mediterr J Hematol Infect Dis. 2021;13(1):e2021011.

37. Kindgen-Milles D, Brandenburger T, Braun JFW, et al. Prevalence of SARS-COV-2 positivity in 516 German intensive care and emergency physicians studied by seroprevalence of antibodies National Covid Survey Germany (NAT-COV-SURV). PLoS One. 2021;16(4):e0248813.

38. Hou FF, Zhou F, Xu X, et al. Personnel protection strategy for healthcare workers in Wuhan during the COVID-19 epidemic. Precis Clin Med. 2020;3(3):169-174.

39. Lan FY, Christophi CA, Buley J, Iliaki E, Bruno-Murtha LA, Sayah AJ, et al. Effects of universal masking on Massachusetts healthcare workers' COVID-19 incidence. Occup Med (Lond). 2020;70(8):606-9.

40.Wang X, Ferro EG, Zhou G, Hashimoto D, Bhatt DL. Association Between Universal Masking in a Health Care System and SARS-CoV-2 Positivity Among Health Care Workers. Jama. 2020 b;324(7):703-4.

41. Cernigliaro M, Negroni D, Sassone M, et al. Observational study on healthcare workers protection in the angiographic suite during the SARS-CoV-2 pandemic: before and during vax era. J Public Health Res. 2021;10(4):2265.

42. Liu H, Wang Y, He HY, et al. Experience of comprehensive interventions in reducing occupational exposure to COVID-19. J Infect Public Health. 2021;14(2):201-205.

43. Rubbi I, Pasquinelli G, Brighenti A, Fanelli M, Gualandi P, Nanni E, et al. Healthcare personnel exposure to COVID - 19: an observational study on quarantined positive workers. Acta Biomed. 2020;91(12-s):e2020012

44. Yao W, Wang T, Jiang B, Gao F, Wang L, Zheng H, et al. Emergency tracheal intubation in 202 patients with COVID-19 in Wuhan, China: lessons learnt and international expert recommendations. Br J Anaesth. 2020;125(1):e28-e37.

45. Moey PKS, Ang ATW, Ee AGL, et al. What are the measures taken to prevent COVID-19 infection among healthcare workers? A retrospective study in a cluster of primary care clinics in Singapore. BMJ Open. 2021;11(6):e049190.

46. Alajmi J, Jeremijenko AM, Abraham JC, Alishaq M, Concepcion EG, Butt AA, et al. COVID-19 infection among healthcare workers in a national healthcare system: The Qatar experience. Int J Infect Dis. 2020;100:386-9.

47. Mizukoshi A, Nakama C, Okumura J, Azuma K. Assessing the risk of COVID-19 from multiple pathways of exposure to SARS-CoV-2: Modeling in health-care settings and effectiveness of nonpharmaceutical interventions. Environ Int. 2021;147:106338.
