# Supplementary material for: Efficacy of personal protective equipment to prevent environmental infection of COVID-19 among healthcare workers: a systematic review
Source: Environ Health Prev Med. 2023 Jan 7;28:1. doi: 10.1265/ehpm.22-00131 (PMC9845060; doi:10.1265/ehpm.22-00131)
Supplement: Supplementary file 2 — Additional file 2: Appendix Literature search results. List of papers in Pubmed for first screening. List of papers in Web of Science for first screening. [file ehpm-28-001-s002.docx]

*Supplementary Material*

Appendix

Literature search results

[List of papers in Pubmed for first screening 2](#_Toc119922325)

[List of papers in Web of Science for first screening 579](#_Toc119922326)

# List of papers in Pubmed for first screening

(2020). "Covid-19: Indian government vows to protect healthcare workers from violence amid rising cases." Bmj **369**: m1631.

(2020). "[Expert consensus on preventing nosocomial transmission during respiratory care for critically ill patients infected by 2019 novel coronavirus pneumonia]." Zhonghua Jie He He Hu Xi Za Zhi **43**(4): 288-296.

Definite evidence has shown that the novel coronavirus (COVID-19) could be transmitted from person to person, so far more than 1 700 bedside clinicians have been infected. A lot of respiratory treatments for critically ill patients are deemed as high-risk factors for nosocomial transmission, such as intubation, manual ventilation by resuscitator, noninvasive ventilation, high-flow nasal cannula, bronchoscopy examination, suction and patient transportation, etc, due to its high possibility to cause or worsen the spread of the virus. As such, we developed this consensus recommendations on all those high-risk treatments, based on the current evidence as well as the resource limitation in some areas, with the aim to reduce the nosocomial transmission and optimize the treatment for the COVID-19 pneumonia patients. Those recommendations include: (1)Standard prevention and protection, and patient isolation; (2)Patient wearing mask during HFNC treatment; (3)Using dual limb ventilator with filters placed at the ventilator outlets, or using heat-moisture exchanger (HME) instead of heated humidification in single limb ventilator with HME placed between exhalation port and mask; avoid using mask with exhalation port on the mask; (4)Placing filter between resuscitator and mask or artificial airway; (5)For spontaneous breathing patients, placing mask for patients during bronchoscopy examination; for patients receiving noninvasive ventilation, using the special mask with bronchoscopy port to perform bronchoscopy; (6)Using sedation and paralytics during intubation, cuff pressure should be maintained between 25-30 cmH(2)O(1 cmH(2)O=0.098 kPa); (7)In-line suction catheter is recommended and it can be used for one week; (8)Dual-limb heated wire circuits are recommended and only changed with visible soiled; (9)For patients who need breathing support during transportation, placing an HME between ventilator and patient; (10)PSV is recommended for implementing spontaneous breathing trial (SBT), avoid using T-piece to do SBT. When tracheotomy patients are weaned from ventilator, HME should be used, avoid using T-piece or tracheostomy mask. (11)Avoid unnecessary bronchial hygiene therapy; (12) For patients who need aerosol therapy, dry powder inhaler metered dose inhaler with spacer is recommended for spontaneous breathing patients; while vibrating mesh nebulizer is recommended for ventilated patients and additional filter is recommended to be placed at the expiratory port of ventilation during nebulization.

(2020). "[Expert consensus on preventing nosocomial transmission during respiratory care for critically ill patients infected by 2019 novel coronavirus pneumonia]." Zhonghua Jie He He Hu Xi Za Zhi **17**(0): E020.

Definite evidence has shown that the novel coronavirus (COVID-19) could be transmitted from person to person, so far more than 1,700 bedside clinicians have been infected. A lot of respiratory treatments for critically ill patients are deemed as high-risk factors for nosocomial transmission, such as intubation, manual ventilation by resuscitator, noninvasive ventilation, high-flow nasal cannula, bronchoscopy examination, suction and patient transportation, etc, due to its high possibility to cause or worsen the spread of the virus. As such, we developed this consensus recommendations on all those high-risk treatments, based on the current evidence as well as the resource limitation in some areas, with the aim to reduce the nosocomial transmission and optimize the treatment for the COVID-19 pneumonia patients. Those recommendations include: (1) Standard prevention and protection, and patient isolation; (2) Patient wearing mask during HFNC treatment; (3) Using dual limb ventilator with filters placed at the ventilator outlets, or using heat-moisture exchanger (HME) instead of heated humidification in single limb ventilator with HME placed between exhalation port and mask; avoid using mask with exhalation port on the mask; (4) Placing filter between resuscitator and mask or artificial airway; (5) For spontaneous breathing patients, placing mask for patients during bronchoscopy examination; for patients receiving noninvasive ventilation, using the special mask with bronchoscopy port to perform bronchoscopy; (6) Using sedation and paralytics during intubation, cuff pressure should be maintained between 25-30 cmH(2)O; (7) In-line suction catheter is recommended and it can be used for one week; (8) Dual-limb heated wire circuits are recommended and only changed with visible soiled; (9. For patients who need breathing support during transportation, placing an HME between ventilator and patient; (10) PSV is recommended for implementing spontaneous breathing trial (SBT), avoid using T-piece to do SBT. When tracheotomy patients are weaned from ventilator, HME should be used, avoid using T-piece or tracheostomy mask. (11) Avoid unnecessary bronchial hygiene therapy; (12) For patients who need aerosol therapy, dry powder inhaler metered dose inhaler with spacer is recommended for spontaneous breathing patients; while vibrating mesh nebulizer is recommended for ventilated patients and additional filter is recommended to be placed at the expiratory port of ventilation during nebulization.

(2020). "Practitioners specialized in oral health and coronavirus disease 2019: Professional guidelines from the French society of stomatology, maxillofacial surgery and oral surgery, to form a common front against the infectious risk." J Stomatol Oral Maxillofac Surg **121**(2): 155-158.

Medical as well as non-medical practitioners specialized in oral health are at high risk of infection with the Coronavirus-19 (Covid-19) because of the proximity with potentially infected biological fluids. This risk is permanent, especially during examination, care and transfer of patients. Regarding the pandemic progression of Covid-19, efficient protocols of prevention are urgently needed. Based on our experience and on the recently reported guidelines from the French National Agency for Public Health (ARS, March 5, 2020), the French Society of Hospital Hygiene (SFHH, March 4, 2020) and the Department of Infectious Risk Prevention of the Hospitals of Paris-Public Assistance (APHP, March 6, 2020), we provide several recommendations for practitioners specialized in oral health, to protect themselves from nosocomial infections, especially Covid-19.

(2020). "[Technical guidelines for seasonal influenza vaccination in China (2020-2021)]." Zhonghua Liu Xing Bing Xue Za Zhi **41**(10): 1555-1576.

Influenza virus infection is a respiratory infectious disease that can seriously affect human health. Influenza viruses can have frequent antigenic variation and changes, which can result in rapid and widespread transmission resulting in annual epidemics and outbreaks in places of public gathering such as schools, kindergartens and nursing homes. According to WHO estimation, seasonal influenza epidemics have caused an annually 3 to 5 million severe cases and 290 000 to 650 000 deaths globally. Pregnant women, young children, the elderly, and persons with chronic illnesses are at high risk for severe illness and death associated with influenza virus infection. Especially, COVID-19 pandemic might co-circulate with other respiratory infectious diseases such as influenza in the coming winter-spring season. Seasonal influenza vaccination is the most effective way to prevent influenza virus infection and complications from infection. Currently, China has licensed trivalent inactivated influenza vaccine (IIV3) which includes split-virus influenza vaccine and subunit vaccine, quadrivalent inactivated influenza vaccine (IIV4) which is split, and trivalent live attenuated influenza vaccine (LAIV3) which was newly licensed. Except for a few major cities, influenza vaccine is a category Ⅱ vaccine, which means influenza vaccination is voluntary, and recipients must pay for it. In 2018 and 2019, the China CDC issued the "Technical Guidelines for Seasonal Influenza Vaccination in China" . In the past year, new research evidences home and abroad have been published, and new seasonal influenza vaccine has been licensed in China. To strengthen the technical guidance for prevention and control of influenza and operational research on influenza vaccination in China, the National Immunization Advisory Committee (NIAC) Influenza Vaccination Technical Working Group (TWG), updated the 2019-2020 technical guidelines and compiled the "Technical guidelines for seasonal influenza vaccination in China (2020-2021)" . The main updates in this version include the following: First, new research evidences especially studies of China, including disease burden, effectiveness, Vaccine-avoidable disease burden, vaccine safety monitoring, and cost-effectiveness and cost-benefit. Second, policies and measures for influenza prevention and control issued by National Health Commission (PRC) in the past year. Thirdly, new type seasonal influenza vaccine licensed and issued in 2020-2021 in China. Fourth, northern hemisphere influenza vaccination composition for the 2020-2021 season which included trivalent and quadrivalent influenza vaccine. Fifth, Influenza vaccination recommendations for 2020-2021 influenza season. The recommendations described in this report include the following: Points of Vaccination clinics (PoVs) should provide influenza vaccination to all persons aged 6 months and above who are willing to be vaccinated and do not have contraindications. No preferential recommendation is made for one influenza vaccine product over another for persons for whom more than one licensed, recommended, and appropriate product is available. Considering the situation of globally pandemic of COVID-19, to decrease the risk of influenza virus infection and minimize the impact on COVID-19 prevention and control, we recommend the following priority for seasonal influenza vaccination: healthcare workers, including clinical doctors and nurses, public health professionals, quarantine professionals; vulnerable groups living in nursing homes or welfare homes and staffs who take care those vulnerable groups; people work or stay in high population density places, such as teachers and students in kindergartens, primary and secondary school, prisoners and staffs of prisons; other high risk group of influenza, including adults ≥60 years of age, children aged 6-59 months, persons with specific chronic diseases, the family members and caregivers of infants <6 months of age, and pregnant women or women who plan to become pregnant during the influenza season. Children aged 6 months through 8 years require 2 doses of influenza vaccine administered a minimum of 4 weeks apart during their first season of vaccination for optimal protection. People whoever get IIV or LAIV all apply to the principle. If they were vaccinated in 2019-2020 influenza season or a prior season, 1 dose is recommended. People more than 8 years old require 1 dose of influenza vaccine. It is recommended that people receive their influenza vaccination by the end of October. Influenza vaccination should be offered as soon as the vaccination is available. For the people unable to be vaccinated before the end of October, influenza vaccination will continue to be offered for the whole season. Influenza vaccine is also recommended for use in pregnant women during any trimester. These guidelines are intended for use by staff members of the CDCs at all levels who work on influenza control and prevention, PoVs staff members, healthcare workers from the departments of pediatrics, internal medicine, and infectious diseases, and staff members of maternity and child care institutions at all levels. These guidelines will be updated periodically as new evidence becomes available.

(2020). "[Technical guidelines for seasonal influenza vaccination in China (2020-2021)]." Zhonghua Yu Fang Yi Xue Za Zhi **54**(10): 1035-1059.

Influenza virus infection is a respiratory infectious disease that can seriously affect human health. Influenza viruses can have frequent antigenic variation and changes, which can result in rapid and widespread transmission resulting in annual epidemics and outbreaks in places of public gathering such as schools, kindergartens and nursing homes. The World Health Organization (WHO) estimated that seasonal influenza epidemics have caused an annual 3 to 5 million severe cases, and 290000 to 650000 deaths globally. Pregnant women, young children, the elderly, and persons with chronic illnesses are at high risk for severe illness and death associated with influenza virus infection. Especially, COVID-19 pandemic might co-circulate with other respiratory infectious diseases such as influenza in the coming winter-spring season. Seasonal influenza vaccination is the most effective way to prevent influenza virus infection and complications from infection. Currently, China has licensed trivalent inactivated influenza vaccine (IIV3) which includes split-virus influenza vaccine and subunit vaccine, quadrivalent inactivated influenza vaccine (IIV4) which is split, and trivalent live attenuated influenza vaccine(LAIV3) which was newly licensed. Except for a few major cities, influenza vaccine is a category Ⅱ vaccine, which means influenza vaccination is voluntary, and recipients must pay for it. In 2018 and 2019, the Chinese Center for Disease Control and Prevention issued the"Technical Guidelines for Seasonal Influenza Vaccination in China". In the past year, new research evidences home and abroad have been published, and new seasonal influenza vaccine has been licensed in China. To strengthen the technical guidance for prevention and control of influenza and operational research on influenza vaccination in China, the National Immunization Advisory Committee (NIAC) Influenza Vaccination Technical Working Group (TWG), updated the 2019-2020 technical guidelines and compiled the "Technical guidelines for seasonal influenza vaccination in China (2020-2021)". The main updates in this version include the following: First, new research evidences especially studies of China, including disease burden, effectiveness, Vaccine-avoidable disease burden, vaccine safety monitoring, and cost-effectiveness and cost-benefit. Second, policies and measures for influenza prevention and control issued by National Health Commission(PRC)in the past year. Thirdly, new type seasonal influenza vaccine licensed and issued in 2020-2021 in China. Fourth, northern hemisphere influenza vaccination composition for the 2020-2021 season which included trivalent and quadrivalent influenza vaccine. Fifth, Influenza vaccination recommendations for 2020-2021 influenza season. The recommendations described in this report include the following: Points of Vaccination clinics (PoVs) should provide influenza vaccination to all persons aged 6 months and above who are willing to be vaccinated and do not have contraindications. No preferential recommendation is made for one influenza vaccine product over another for persons for whom more than one licensed, recommended, and appropriate product is available. Considering the situation of globally pandemic of COVID-19, to decrease the risk of influenza virus infection and minimize the impact on COVID-19 prevention and control, we recommend the following priority for seasonal influenza vaccination: (1) healthcare workers, including clinical doctors and nurses, public health professionals, quarantine professionals; (2) Vulnerable groups living in nursing homes or welfare homes and staffs who take care those vulnerable groups; (3) People in some key places, such as teachers and students in kindergartens, primary and secondary school, prisoners and staffs of prisons; (4) Other high risk group of influenza, including adults ≥60 years of age, children aged 6-59 months, persons with specific chronic diseases, the family members and caregivers of infants <6 months of age, and pregnant women or women who plan to become pregnant during the influenza season. Children aged 6 months through 8 years require 2 doses of influenza vaccine administered a minimum of 4 weeks apart during their first season of vaccination for optimal protection. People whoever get IIV or LAIV all apply to the principle. If they were vaccinated in 2019-2020 influenza season or a prior season, 1 dose is recommended. People more than 8 years old require 1 dose of influenza vaccine. It is recommended that people receive their influenza vaccination by the end of October. Influenza vaccination should be offered as soon as the vaccination is available. For the people unable to be vaccinated before the end of October, influenza vaccination will continue to be offered for the whole season. Influenza vaccine is also recommended for use in pregnant women during any trimester. These guidelines are intended for use by staff members of the Centers for Disease Control and Prevention at all levels who work on influenza control and prevention, PoVs staff members, healthcare workers from the departments of pediatrics, internal medicine, and infectious diseases, and staff members of maternity and child care institutions at all levels. These guidelines will be updated periodically as new evidence becomes available.

(2021). "CSF Rhinorrhea After Endonasal Intervention to the Skull Base (CRANIAL) - Part 2: Impact of COVID-19." World Neurosurg.

BACKGROUND: During the coronavirus disease 2019 (COVID-19) pandemic, concerns have been raised regarding the increased risk of perioperative mortality for patients with COVID-19, and the transmission risk to healthcare workers, especially during endonasal neurosurgical operations. The Pituitary Society has produced recommendations to guide management during this era. We sought to assess contemporary neurosurgical practice and the effects of COVID-19. METHODS: A multicenter prospective observational cohort study was conducted at 12 tertiary neurosurgical units (United Kingdom and Ireland). Data were collected from March 23 to July 31, 2020, inclusive. The data points collected included patient demographics, preoperative COVID-19 test results, operative modifications, and 30-day COVID-19 infection rates. RESULTS: A total of 124 patients were included. Of the 124 patients, 116 (94%) had undergone COVID-19 testing preoperatively (transsphenoidal approach, 97 of 105 [92%]; expanded endoscopic endonasal approach, 19 of 19 [100%]). One patient (1 of 116 [0.9%]) had tested positive for COVID-19 preoperatively, requiring a delay in surgery until the infection had been confirmed as resolved. Other than transient diabetes insipidus, no other complications were reported for this patient. All operating room staff had worn at least level 2 personal protective equipment. Adaptations to surgical techniques included minimizing drilling, draping modifications, and the use of a nasal iodine wash. At 30 days postoperatively, no evidence of COVID-19 infection (symptoms or positive formal testing results) were found in our cohort and no mortality had occurred. CONCLUSIONS: Preoperative screening protocols and operative modifications have facilitated endonasal neurosurgery during the COVID-19 pandemic, with the Pituitary Society guidelines followed for most of these operations. We found no evidence of COVID-19 infection in our cohort and no mortality, supporting the use of risk mitigation strategies to continue endonasal neurosurgery in subsequent pandemic waves.

Abbas, A., et al. (2021). "Sleep Quality Among Healthcare Workers During the COVID-19 Pandemic and Its Impact on Medical Errors: Kuwait Experience." Turk Thorac J **22**(2): 142-148.

OBJECTIVE: Millions of people suffer from sleep disturbances. In addition, the coronavirus disease 2019 (COVID-19) pandemic created several new challenges-particularly for frontline healthcare workers (HCWs). This study assessed the sleep quality (SQ) among HCWs. MATERIAL AND METHODS: A cross-sectional study was conducted using an English-language online survey. The participants were invited via a web link sent using social network platforms. It included sociodemographic- and profession-related characteristics. COVID-19-associated risks were assessed (e.g., being on the front line, doing swabs, satisfaction about protective equipment, and management protocols). Assessment of SQ was done using the Pittsburgh Sleep Quality Index (PSQI) and various medical errors were recorded. RESULTS: A total of 217 HCWs completed the survey with mean (±standard deviation) age of 35.8 (±7.3) years; 56.2% were male, 18.43% had comorbidities, and 61.75% experienced sleep difficulties before the COVID-19 crisis. This work reports a 78.8% prevalence of poor SQ, with the mean (standard deviation) global PSQI score of 9.36 (±4.4). HCWs with poor sleep experienced more positive comorbid profile (23.64% versus 6.52%, p=0.01). Working on the front lines of COVID-19 was associated with poor sleep (69.59% versus 47.83%, p=0.006). Among the participants, 77.42% performed medical errors, particularly not checking for drug allergies (17.97%), dispensing medication with incomplete instructions (20.74%), providing incorrect doses or overdosing (14.75%), incorrectly explaining the use of medication (9.22%), and prescribing a drug to the wrong patient (10.14%). CONCLUSION: This nationwide survey reported high prevalence of poor SQ among HCWs during the COVID-19 pandemic. Being an HCW on the front lines of COVID-19 and doing swabs with a positive comorbidity was associated with poor sleep.

Abbas, A., et al. (2020). "COVID-19 risk assessments: shortcomings in the protection of Black, Asian and Minority Ethnic healthcare workers." J Hosp Infect **106**(2): 385-386.

Abbas, M., et al. (2021). "Nosocomial transmission and outbreaks of coronavirus disease 2019: the need to protect both patients and healthcare workers." Antimicrob Resist Infect Control **10**(1): 7.

OBJECTIVES: To compile current published reports on nosocomial outbreaks of severe acute respiratory syndrome coronavirus 2 (SARS-CoV-2), evaluate the role of healthcare workers (HCWs) in transmission, and evaluate outbreak management practices. METHODS: Narrative literature review. SHORT CONCLUSION: The coronavirus disease 2019 (COVID-19) pandemic has placed a large burden on hospitals and healthcare providers worldwide, which increases the risk of nosocomial transmission and outbreaks to "non-COVID" patients or residents, who represent the highest-risk population in terms of mortality, as well as HCWs. To date, there are several reports on nosocomial outbreaks of SARS-CoV-2, and although the attack rate is variable, it can be as high as 60%, with high mortality. There is currently little evidence on transmission dynamics, particularly using genomic sequencing, and the role of HCWs in initiating or amplifying nosocomial outbreaks is not elucidated. There has been a paradigm shift in management practices of viral respiratory outbreaks, that includes widespread testing of patients (or residents) and HCWs, including asymptomatic individuals. These expanded testing criteria appear to be crucial in identifying and controlling outbreaks.

Abboah-Offei, M., et al. (2021). "A rapid review of the use of face mask in preventing the spread of COVID-19." Int J Nurs Stud Adv **3**: 100013.

INTRODUCTION: The original use of face masks was to help protect surgical wounds from staff-generated nasal and oral bacteria. Currently governments across the world have instituted the mandatory use of masks and other face coverings so that face masks now find much broader usage in situations where close contact of people is frequent and inevitable, particularly inside public transport facilities, shopping malls and workplaces in response to the COVID-19. OBJECTIVE: We conducted a rapid review to investigate the impact face mask use has had in controlling transmission of respiratory viral infections. METHOD: A rapid review was conducted in line with Preferred Reporting Items for Systematic Reviews and Meta-Analyses guidance. Five electronic databases (CINAHL, Embase, Medline, PsycINFO and Global Health) were searched from database inception to date, using pre-defined search terms. We included all studies of any design and used descriptive analysis to report summary statistics of search results. Data were extracted including sample characteristics, study design, respiratory virus being controlled, type of face masks used and their effectiveness. RESULTS: 58 out of 84 studies met the inclusion criteria, of which 13 were classified as systematic reviews and 45 were quantitative studies (comprising randomised controlled trials, retrospective cohort studies, case control, cross-sectional, surveys, observational and descriptive studies). N = 27 studies were conducted amongst healthcare workers wearing face masks, n = 19 studies among the general population, n = 9 studies among healthcare workers the general population and patients wearing masks, and n = 3 among only patients. Face masks use have shown a great potential for preventing respiratory virus transmission including COVID-19. CONCLUSION: Regardless of the type, setting, or who wears the face mask, it serves primarily a dual preventive purpose; protecting oneself from getting viral infection and protecting others. Therefore, if everyone wears a face mask in public, it offers a double barrier against COVID-19 transmission.

Abd Samat, A. H., et al. (2020). "Knowledge and Confidence Level Among Emergency Healthcare Workers in Airway Management and Resuscitation of Suspected COVID-19 Patients: A Cross Sectional Study in Malaysia." Ann Acad Med Singap **49**(9): 643-651.

INTRODUCTION: This study aims to evaluate the knowledge and confidence of emergency healthcare workers (EHCW) in facing the COVID-19 pandemic. MATERIALS AND METHODS: A cross-sectional online study using a validated questionnaire was distributed to doctors (MD), assistant medical officers (AMO), and staff nurses (SN) at an urban tertiary Emergency Department. It comprised of 40 knowledge and 10 confidence-level questions related to resuscitation and airway management steps. RESULTS: A total of 135 from 167 eligible EHCW were enrolled. 68.9% (n = 93) had high knowledge while 53.3% (n = 72) possessed high confidence level. Overall knowledge mean score was 32.96/40 (SD = 3.63) between MD (33.88±3.09), AMO (32.28±4.03), and SN (32.00±3.60), P= 0.025. EHCWs with a length of service (LOS) between 4-10 years had the highest knowledge compared to those with LOS <4-year (33.71±3.39 versus 31.21±3.19 P = 0.002). Airway-related knowledge was significantly different between the designations and LOS (P = 0.002 and P = 0.003, respectively). Overall, EHCW confidence level against LOS showed significant difference [F (2, 132) = 5.46, P = 0.005] with longer LOS showing better confidence. MD showed the highest confidence compared to AMO and SN (3.67±0.69, 3.53±0.68, 3.26±0.64) P = 0.049. The majority EHCW were confident in performing high-quality chest-compression, and handling of Personal Protective Equipment but less than half were confident in resuscitating, leading the resuscitation, managing the airway or being successful in first intubation attempt. CONCLUSIONS: EHCW possessed good knowledge in airway and resuscitation of COVID-19 patients, but differed between designations and LOS. A longer LOS was associated with better confidence, but there were some aspects in airway management and resuscitation that needed improvement.

Abdel Wahed, W. Y., et al. (2020). "Assessment of Knowledge, Attitudes, and Perception of Health Care Workers Regarding COVID-19, A Cross-Sectional Study from Egypt." J Community Health **45**(6): 1242-1251.

Healthcare workers (HCWs) are at the frontline defense against the coronavirus disease 2019 (COVID-19) pandemic. Inadequate knowledge and incorrect attitudes among HCWs can directly influence practices and lead to delayed diagnosis, poor infection control practice, and spread of disease. This study aimed to assess the knowledge, perception, and attitude of the Egyptian HCWs towards the COVID-19 disease. A descriptive cross-sectional study was conducted in Egypt, among 407 HCWs using a self-administered questionnaire. The mean correct answer rate was 80.4% with a mean knowledge score of 18.5 ± 2.7 out of 24. A positive correlation between knowledge and attitude scores was detected (r = 0.215, p < 0.001). About 83.1% of our participants reported that they were afraid of being infected with COVID-19, and 89.2% stated that they were more susceptible to COVID-19 infection as compared to others. Unavailability of personal protective equipment (PPE), fear of transmitting the disease to their families, and social stigma were the most frequently reported reasons for increased risk perception. The overall knowledge level of HCWs was generally good especially among physicians. A positive attitude was detected among allied health professionals more than physicians. Risk perception was high among HCWs. Causes of increased risk perception need to considered by the government and the Egyptian Ministry of Health.

Abdi, A., et al. (2021). "Preliminary findings of COVID-19 infection in health workers in Somalia: A reason for concern." Int J Infect Dis **104**: 734-736.

BACKGROUND: Somalia, a country with a long history of instability, has a fragile healthcare system that is consistently understaffed. A large number of healthcare workers (HCWs) have become infected during the coronavirus disease-19 (COVID-19) pandemic. OBJECTIVE: This report presents the preliminary findings of COVID-19 infection in Somali HCWs, the first of such information from Somalia. METHODS: This preliminary retrospective study analysed available data on infection rates among Somali HCWs. RESULTS: As of 30 September 2020, 3700 cases of COVID-19, including 98 deaths, had been reported in Somalia; 191 (5%) of these cases were HCWs. During the first 180 days of the outbreak, 311 HCWs were tested for COVID-19 and 191 tested positive (positivity rate: 61%). During the epidemic's peak, HCWs represented at least 5% of cases. Of the 191 infected cases, 52 (27%) were doctors, 63 (33%) were nurses, seven (4%) were laboratory technicians, and 36% were other staff. CONCLUSION: More information must be sought to put measures in place to protect the health and safety of HCWs in Somalia's already understaffed and fragile healthcare system.

Abdi, R., et al. (2020). "Surgical Practice in the Shadow of COVID-19 Outbreak." Arch Bone Jt Surg **8**(Suppl1): 256-261.

BACKGROUND: COVID-19 epidemic rapidly spread all around the world with over 1500 thousand infected cases and 95000 deaths. This rapid pandemic may overwhelm health care capacity and shortage of resources is a major concern. Literature provided guidelines on management of COVID-19 patients but healthcare service to the normal population should be continued meanwhile. Health system should act immediately and wisely to support essential surgical care while fighting against COVID-19. METHODS: We conducted a comprehensive search in the major data bases since 2020, using the combination of MeSH words of "COVID-19 " and "surgery" and finally 34 full texts entered to data extraction phase to define a plan for surgical practice during COVID-19 pandemic. RESULTS: Healthcare workers are at the higher risk of contamination by COVID-19 especially in early stage of outbreak when they were not aware of the different aspects of COVID-19 pandemic. All healthcare staff must be trained to properly use PPE. All patients have to be screened at the hospital triage. All elective surgical interventions must be postponed. Operation room is considered as a place with high risk of cross infection so the highest level of protection should be maintained. Anesthesia, endoscopy and oral surgery are considered as aerosol producing procedures with very high risk of contamination. There is not any evidence to support the risk of infection trough blood products. Postoperative respiratory problems are more common among COVID-19 patients that may increases the estimated risk of morbidity and mortality. CONCLUSION: COVID-19 pandemic is a dynamic challenge for health system to save the healthcare staff and equipment resources by timely decisions. Healthcare workers are at the higher risk of contamination by COVID-19 especially in early phase of epidemic when the protection is sub-optimal.

Abi Karam, K., et al. (2021). "Development of a new aerosol barrier mask for mitigation of spread of SARS-CoV-2 and other infectious pathogens." Respir Med **181**: 106381.

The COVID-19 pandemic has caused huge impact on public health and significantly changed our lifestyle. This is due to the fast airborne oro-nasal transmission of SARS-CoV-2 from the infected individuals. The generation of liquid aerosolized particles occurs when the COVID-19 patients speak, sing, cough, sneeze, or simply breathe. We have developed a novel aerosol barrier mask (ABM) to mitigate the spread of SARS-CoV-2 and other infectious pathogens. This Aerosol Barrier Mask is designed for preventing SARS-CoV-2 transmission while transporting patients within hospital facilities. This mask can constrain aerosol and droplet particles and trap them in a biofilter, while the patient is normally breathing and administrated with medical oxygen. The system can be characterized as an oxygen delivery and mitigation mask which has no unfiltered exhaled air dispersion. The mask helps to prevent the spread of SARS-CoV-2, and potentially other infectious respiratory pathogens and protects everyone in general, especially healthcare professionals.

Abiakam, N., et al. (2021). "Personal protective equipment related skin reactions in healthcare professionals during COVID-19." Int Wound J.

Since the outbreak of COVID-19 pandemic, clinicians have had to use personal protective equipment (PPE) for prolonged periods. This has been associated with detrimental effects, especially in relation to the skin health. The present study describes a comprehensive survey of healthcare workers (HCWs) to describe their experiences using PPE in managing COVID-19 patients, with a particular focus on adverse skin reactions. A 24-hour prevalence study and multi-centre prospective survey were designed to capture the impact of PPE on skin health of hospital staff. Questionnaires incorporated demographics of participants, PPE type, usage time, and removal frequency. Participants reported the nature and location of any corresponding adverse skin reactions. The prevalence study included all staff in intensive care from a single centre, while the prospective study used a convenience sample of staff from three acute care providers in the United Kingdom. A total of 108 staff were recruited into the prevalence study, while 307 HCWs from a variety of professional backgrounds and demographics participated in the prospective study. Various skin adverse reactions were reported for the prevalence study, with the bridge of the nose (69%) and ears (30%) being the most affected. Of the six adverse skin reactions recorded for the prospective study, the most common were redness blanching (33%), itchiness (22%), and pressure damage (12%). These occurred predominantly at the bridge of the nose and the ears. There were significant associations (P < .05) between the adverse skin reactions with both the average daily time of PPE usage and the frequency of PPE relief. The comprehensive study revealed that the use of PPE leads to an array of skin reactions at various facial locations of HCWs. Improvements in guidelines are required for PPE usage to protect skin health. In addition, modifications to PPE designs are required to accommodate a range of face shapes and appropriate materials to improve device safety.

Abolfotouh, M. A., et al. (2020). "Perception and attitude of healthcare workers in Saudi Arabia with regard to Covid-19 pandemic and potential associated predictors." BMC Infect Dis **20**(1): 719.

BACKGROUND: Healthcare workers (HCWs) face considerable mental and physical stress caring for patients with Covid-19. They are at higher risk of acquiring and transmitting this virus. This study aims to assess perception and attitude of HCWs in Saudi Arabia with regard to Covid-19, and to identify potential associated predictors. METHODS: In a cross-sectional study, HCWs at three tertiary hospitals in Saudi Arabia were surveyed via email with an anonymous link, by a concern scale about Covid-19 pandemic during 15-30 April, 2020. Concerns of disease severity, governmental efforts to contain it and disease outcomes were assessed using 32 concern statements in five distinct domains. Multiple regression analysis was used to identify predictors of high concern scores. RESULTS: A total of 844 HCW responded to the survey. Their average age was 40.4 ± 9.5 years, 40.3% were nurses, 58.2% had direct patient contact, and 77.3% were living with others. The majority of participants (72.1%) had overall concern scores of 55 or less out of a maximum score of 96 points, with an overall mean score of 48.5 ± 12.8 reflecting moderate level of concern. Three-fourth of respondents felt at risk of contracting Covid-19 infection at work, 69.1% felt threatened if a colleague contracted Covid-19, 69.9% felt obliged to care for patients infected with Covid-19 while 27.7% did not feel safe at work using the standard precautions available. Nearly all HCWs believed that the government should isolate patients with Covid-19 in specialized hospitals (92.9%), agreed with travel restriction to and/or from areas affected by Covid-19 (94.7%) and felt safe the government implemented curfew and movement restriction periods (93.6%). Predictors of high concern scores were; HCWs of Saudi nationality (p < 0.001), younger age (p = 0.003), undergraduate education (p = 0.044), living with others (p = 0.003) working in the western region (p = 0.003) and direct contact with patients (p = 0.018). CONCLUSIONS: This study highlights the high concern among HCWs about Covid-19 and identifies the predictors of those with highest concern levels. To minimize the potential negative impact of those concerns on the performance of HCWs during pandemics, measures are necessary to enhance their protection and to minimize the psychological effect of the perceived risk of infection.

Abtahi, M., et al. (2021). "An innovative index for assessing vulnerability of employees of different occupations from the COVID-19 pandemic in Iran." Environ Res **197**: 111039.

The vulnerability of employees of different occupations from the Coronavirus disease 2019 (COVID-19) pandemic in Iran was assessed using an innovative index. The vulnerability index was developed in five steps as follows: (1) determining the principles and components of employees' susceptibility and resilience, (2) weighting the principles and components, (3) converting the levels of components to the sub-index values, (4) introducing the aggregation functions, and (5) characterizing the vulnerability index values in five categories as very high (80-100), high (65-79), medium (50-64), low (30-49), and very low (0-29). The average values of susceptibility, resilience, and vulnerability index of the employees were determined to be 35.2 ± 15.0, 73.9 ± 17.0, and 32.9 ± 12.7, respectively. The average resilience of the employees was more desirable than their average susceptibility. The distribution of the employees into the vulnerability index categories was 46.3% for very low, 41.9% for low, 3.6% for medium, and 8.2% for high. The worst cases of susceptibility and resilience principles were exposure to contaminated surfaces (59.1 ± 22.8) and top management commitment (66.6 ± 23.1). The elderly staff (especially over 50 years old), employees with low education levels, and employees in private and self-employment sectors were significantly more vulnerable (p value < 0.01) from the COVID-19 pandemic. The principles with significant incremental effects on the vulnerability index (p value < 0.05) were respectively top management commitment (+1.78), exposure to COVID-19 patients at work (+1.36), exposure to contaminated surfaces (+0.82), installing clear shields and wearing PPE (+0.59), observance of social distancing (+0.48), and just culture (+0.22). An especial plan to support the more vulnerable employees with an emphasis on the principles with the most incremental effects on the vulnerability index can efficiently control the inequality between the employees as well as occupational transmission of the COVID-19 in Iran.

Accardo, D., et al. (2021). "Methods to Reduce the Risk of Exposure to Airborne Pathogens in the Operating Room." Aana j **89**(1): 71-75.

The ongoing coronavirus disease 2019 (COVID-19) pandemic has created many changes and difficulties in healthcare, and the anesthesia specialty is no exception. Both the increased need for personal protective equipment (PPE) and the potential for infection and contamination through respiratory droplets have been sources of much concern. Policies and protocols have been adapted worldwide to help neutralize infection risk and exposure. Transmission of the virus to healthcare workers has been a major concern, and the risk of infection is exceptionally high for Certified Registered Nurse Anesthetists (CRNAs) because of their close contact with infected patients. CRNAs are in a unique position to help decrease exposure for themselves and other members of the healthcare team by taking extra precautions during airway manipulation. A great deal of focus has been placed on reducing risks during intubation, but reports describing methods of reducing contamination and exposure to respiratory droplets during emergence and extubation are scarce. The authors have reviewed techniques to reduce coughing, thereby decreasing the potential of virus exposure through contact with large respiratory droplets and aerosolized particles that may remain suspended in air.

Adams, J. E. and D. J. Ecker (2021). "Telehealth: from the abstract to necessity to competency." FASEB Bioadv.

The COVID-19 pandemic caused significant disruption in medical education. With disruption comes the opportunity for innovation. Telehealth had been growing rapidly in many fields of medicine prior to the pandemic, however, the necessities of social distancing, scarcity of personal protective equipment, and mandates to prevent unnecessary exposures for healthcare workers and patients alike, brought opportunities for the exponential expansion of telehealth. With expansion of telehealth services came the need to expand curriculum in telehealth to prepare medical students to return to vastly transformed clinical settings as well as prepare them for a future clinical landscape likely to incorporate telehealth to a much greater degree. The University of Colorado School of Medicine (CUSOM) rapidly developed a course in telehealth to prepare students for this changing clinical environment. Simultaneously, a faculty development curriculum was created to support clinical faculty new to telehealth in basic skills and teaching in a virtual environment. Lastly, adaptations were made to the summative Clinical Practice Exam administered to students at the completion of clerkships to incorporate telehealth. Recognizing the importance of achieving competence in telehealth, the CUSOM has taken steps to invest in the development of comprehensive and integrated telehealth curricula. Many creative and innovative solutions have been adopted in the wake of this pandemic to allow medical education to continue despite many hurdles and barriers; many of these will not persist past the pandemic. However, we expect telehealth clinical skills and the curricula developed to support them to remain relevant long past the time when the COVID-19 pandemic has faded into history.

Adeniyi, O. V., et al. (2021). "Eastern Cape Healthcare Workers Acquisition of SARS-CoV-2 (ECHAS): Cross-Sectional (Nested Cohort) Study Protocol." Int J Environ Res Public Health **18**(1).

Healthcare workers (HCWs) are at increased risk of infection by the virulent severe acute respiratory syndrome coronavirus-2 (SARS-CoV-2). Though data exist on the positivity rate of the SARS-CoV-2 reverse transcription polymerase chain reaction (RT-PCR) test as well as COVID-19-related deaths amongst HCWs in South Africa, the overall infection rate remains underestimated by these indicators. It is also unclear whether the humoral immune response after SARS-CoV-2 infection offers durable protection against reinfection. This study will assess the SARS-CoV-2 seroprevalence amongst HCWs in the Eastern Cape (EC) and examine the longitudinal changes (rate of decay) in the antibody levels after infection in this cohort. Using a multi-stage cluster sampling of healthcare workers in selected health facilities in the EC, a cross-sectional study of 2250 participants will be recruited. In order to assess the community infection rate, 750 antenatal women in the same settings will be recruited. Relevant demographic and clinical characteristics will be obtained by a self-administered questionnaire. A chemiluminescent microparticle immunoassay (CMIA) will be used for the qualitative detection of IgG antibodies against SARS-CoV-2 nucleocapsid protein. A nested cohort study will be conducted by performing eight-weekly antibody assays (X2) from 201 participants who tested positive for both SARS-CoV-2 RT-PCR and serology. Logistic regression models will be fitted to identify the independent risk factors for SARS-CoV-2 infection. The cumulative SARS-CoV-2 infection rate and infection fatality rate among the frontline HCWs will be estimated. In addition, the study will highlight the overall effectiveness of infection prevention and control measures (IPC) per exposure sites/wards at the selected health facilities. Findings will inform the South African Department of Health's policies on how to protect HCWs better as the country prepares for the second wave of the SARS-CoV pandemic.

Adir, Y., et al. (2020). "COVID-19: minimising risk to healthcare workers during aerosol-producing respiratory therapy using an innovative constant flow canopy." Eur Respir J **55**(5).

An innovative constant flow canopy enables noninvasive respiratory support with minimal risk of healthcare worker infection https://bit.ly/3eqgoVZ

Noninvasive ventilation (NIV), continuous positive airway pressure (CPAP) and high-flow nasal cannula (HFNC) can be used as the first line of treatment in coronavirus disease 2019 (COVID-19) patients with respiratory failure, postponing and maybe even avoiding the need for intubation and mechanical ventilation [1]. Recent systematic review and meta-analysis demonstrated that HFNC reduces the need for intubation compared with conventional oxygen, with no change in the death risk or length of stay in the intensive care unit [2, 3]. No direct evidence supports the use of NIV, due to a high failure rate [4]. However, when resources become limited, with no option of invasive ventilation, the use of NIV may be justified. The major caveat of using noninvasive respiratory support in the face of the COVID-19 pandemic is the generation of aerosols, composed of small virus-containing particles, which may remain suspended in the air, with increased risk for healthcare workers [5, 6]. The risk of aerosolisation depends on many variables, including duration of use, flow velocity, mask leakage and patient coughing and cooperation.

eng

Adrielle Dos Santos, L., et al. (2021). "Recurrent COVID-19 including evidence of reinfection and enhanced severity in thirty Brazilian healthcare workers." J Infect **82**(3): 399-406.

BACKGROUND: There is growing concern about individuals reported to suffer repeat COVID-19 disease episodes, these in a small number of cases characterised as de novo infections with distinct sequences, indicative of insufficient protective immunity even in the short term. METHODS: Observational case series and case-control studies reporting 33 cases of recurrent, symptomatic, qRT-PCR positive COVID-19. Recurrent disease was defined as symptomatic recurrence after symptom-free clinical recovery, with release from isolation >14 days from the beginning of symptoms confirmed by qRT-PCR. The case control study-design compared this group of patients with a control group of 62 patients randomly selected from the same COVID-19 database. RESULTS: Of 33 recurrent COVID-19 patients, 26 were female and 30 were HCW. Mean time to recurrence was 50.5 days which was associated with being a HCW (OR 36.4 (p <0.0001)), and blood type A (OR 4.8 (p = 0.002)). SARS-CoV-2 antibodies were signifcantly lower in recurrent patients after initial COVID-19  (2.4 ± 0.610; p<0.0001) and after recurrence (6.4 ± 11.34; p = 0.007).  Virus genome sequencing identified reinfection by a different isolate in one patient. CONCLUSIONS: This is the first detailed case series showing COVID-19 recurrence with qRT-PCR positivity. For one individual detection of phylogenetically distinct genomic sequences in the first and second episodes confirmed bona fide renfection, but in most cases the data do not formally distinguish between reinfection and re-emergence of a chronic infection reservoir. These episodes were significantly associated with reduced Ab response during initial disease and argue the need for ongoing vigilance without an assumption of protection after a first episode.

Agarwal, A., et al. (2020). "Difficulties Encountered While Using PPE Kits and How to Overcome Them: An Indian Perspective." Cureus **12**(11): e11652.

Background After a slow start due to an effective lockdown, the coronavirus disease 2019 (COVID-19) pandemic in India has been raging at a rapid pace, posing a formidable challenge to the healthcare system in the country. The personal protective equipment (PPE) undoubtedly provides a shield of protection for the healthcare workers (HCWs) fighting the disease as a valuable asset to the nation. However, there have been various problems associated with the PPE, ranging from its shortage to problems arising from heat, dehydration, etc while wearing them. There is a need to assess these problems faced by HCWs both qualitatively and quantitatively for their timely and effective redressal. Methods An electronic questionnaire survey was conducted among a cohort of HCWs who had performed COVID-19 duties and used PPE kits. The cohort consisted of different categories of doctors, nursing personnel, and other paramedical staff. Results The most common problems associated with using PPE kits was excessive sweating (100%), fogging of goggles, spectacles, or face shields (88%), suffocation (83%), breathlessness (61%), fatigue (75%), headache due to prolonged use (28%), and pressure marks on the skin at one or more areas on repeated use (19%). Occasional problems reported were skin allergy/dermatitis caused by the synthetic material of the PPE kit, face shield impinging onto the neck during intubation, and nasal pain, pain at the root of the pinna, and slipperiness of shoe covers. Various ways and means have been employed by the HCWs to actively address and solve these problems. Conclusion These plausible solutions will definitely help the HCWs to deal with and solve the problems arising out of the PPE use.

Agarwal, A., et al. (2021). "Risk of dispersion or aerosol generation and infection transmission with nasopharyngeal and oropharyngeal swabs for detection of COVID-19: a systematic review." BMJ Open **11**(3): e040616.

OBJECTIVES: SARS-CoV-2-related disease, referred to as COVID-19, has emerged as a global pandemic since December 2019. While there is growing recognition regarding possible airborne transmission, particularly in the setting of aerosol-generating procedures and treatments, whether nasopharyngeal and oropharyngeal swabs for SARS-CoV-2 generate aerosols remains unclear. DESIGN: Systematic review. DATA SOURCES: We searched Ovid MEDLINE and EMBASE up to 3 November 2020. We also searched the China National Knowledge Infrastructure, Chinese Medical Journal Network, medRxiv and ClinicalTrials.gov up to 29 March 2020. ELIGIBILITY CRITERIA: All comparative and non-comparative studies that evaluated dispersion or aerosolisation of viable airborne organisms, or transmission of infection associated with nasopharyngeal or oropharyngeal swab testing. RESULTS: Of 7702 citations, only one study was deemed eligible. Using a dedicated sampling room with negative pressure isolation room, personal protective equipment including N95 or higher masks, strict sterilisation protocols, structured training with standardised collection methods and a structured collection and delivery system, a tertiary care hospital proved a 0% healthcare worker infection rate among eight nurses conducting over 11 000 nasopharyngeal swabs. No studies examining transmissibility with other safety protocols, nor any studies quantifying the risk of aerosol generation with nasopharyngeal or oropharyngeal swabs for detection of SARS-CoV-2, were identified. CONCLUSIONS: There is limited to no published data regarding aerosol generation and risk of transmission with nasopharyngeal and oropharyngeal swabs for the detection of SARS-CoV-2. Field experiments to quantify this risk are warranted. Vigilance in adhering to current standards for infection control is suggested.

Agarwal, A., et al. (2020). "Guidance for building a dedicated health facility to contain the spread of the 2019 novel coronavirus outbreak." Indian J Med Res **151**(2 & 3): 177-183.

Preparedness for the ongoing coronavirus disease 2019 (COVID-19) and its spread in India calls for setting up of adequately equipped and dedicated health facilities to manage sick patients while protecting healthcare workers and the environment. In the wake of other emerging dangerous pathogens in recent times, such as Ebola, Nipah and Zika, it is important that such facilities are kept ready during the inter-epidemic period for training of health professionals and for managing cases of multi-drug resistant and difficult-to-treat pathogens. While endemic potential of such critically ill patients is not yet known, the health system should have surge capacity for such critical care units and preferably each tertiary government hospital should have at least one such facility. This article describes elements of design of such unit (e.g., space, infection control, waste disposal, safety of healthcare workers, partners to be involved in design and plan) which can be adapted to the context of either a new construction or makeshift construction on top of an existing structure. In view of a potential epidemic of COVID-19, specific requirements to handle it are also given.

Agarwal, M., et al. (2020). "Hydroxychloroquine as a Chemoprophylactic Agent for COVID-19: A Clinico-Pharmacological Review." Front Pharmacol **11**: 593099.

Hydroxychloroquine has gained much attention as one of the candidate drugs that can be repurposed as a prophylactic agent against SARS-CoV-2, the agent responsible for the COVID-19 pandemic. Due to high transmissibility and presence of asymptomatic carriers and presymptomatic transmission, there is need for a chemoprophylactic agent to protect the high-risk population. In this review, we dissect the currently available evidence on hydroxychloroquine prophylaxis from a clinical and pharmacological point of view. In vitro studies on Vero cells show that hydroxychloroquine effectively inhibits SARS-CoV-2 by affecting viral entry and viral transport via endolysosomes. However, this efficacy has failed to replicate in in vivo animal models as well as in most clinical observational studies and clinical trials assessing pre-exposure prophylaxis and postexposure prophylaxis in healthcare workers. An analysis of the pharmacology of HCQ in COVID-19 reveals certain possible reasons for this failure-a pharmacokinetic failure due to failure to achieve adequate drug concentration at the target site and attenuation of its inhibitory effect due to the presence of TMPRSS2 in airway epithelial cells. Currently, many clinical trials on HCQ prophylaxis in HCW are ongoing; these factors should be taken into account. Using higher doses of HCQ for prophylaxis is likely to be associated with increased safety concerns; thus, it may be worthwhile to focus on other possible interventions.

Agrawal, U., et al. (2020). "Resource husbandry in challenging times." J Pak Med Assoc **70(Suppl 3)**(5): S25-s29.

Coronavirus disease 2019 (COVID-19) pandemic has put a huge pressure on healthcare systems across the globe, more so in developing countries. Not only patients of acute febrile illness and respiratory problems but also patients with other acute and chronic diseases are facing challenges while seeking healthcare, getting laboratory investigations done and obtaining medications. Healthcare workers have their challenges including limited resources, lack of personal protective equipments, and fear of contracting COVID-19. Resource husbandry, which refers to the judicious use of available stocks, is a vital concept that needs to be promoted during such challenging times to combat the shortage of medical resources while simultaneously providing effective treatment to the patients. Some easily implementable concepts of resource husbandry can have a significant impact and result in minimising trouble for many patients during a challenging time.

Agrawal, V. and D. Sharma (2021). ""Comment on the article titled "Nosocomial SARS-CoV-2 transmission in postoperative infection and mortality: analysis of 14 798 procedures" by Elliott JA et al." Br J Surg **108**(1): e53.

Nosocomial SARS-CoV-2 transmission in postoperative infection and mortality assumes great importance given the gradual re-booting of elective surgeries all over the world. It is interesting to see that laparoscopic surgery may be protective for nosocomial transmission of COVID-19 infection owing to reduced duration of hospital stay. For the same reason, the wider application of enhanced recovery after surgery protocols need to be explored. Additionally, now there is a definite need to differentiate COVID-specific deaths (deaths due to complications of COVID-19) from the COVID-related deaths (deaths due to complications of comorbidity or the surgical disease or surgery itself in a COVID-19 positive patient). This is required to identify the real risk of nosocomial infection and the subsequent mortality in elective surgery during this pandemic.

Aguilera, S. B., et al. (2020). "The Impact of COVID-19 on the Faces of Frontline Healthcare Workers." J Drugs Dermatol **19**(9): 858-864.

As the coronavirus epidemic continues, a host of new cutaneous complications is seen on the faces of frontline healthcare workers wearing personal protective equipment on a daily basis. To minimize the risk of COVID-19 infection, healthcare workers wear tight-fitting masks that lead to an excessive amount of pressure on the facial skin. Mechanical pressure, mask materials, and perspiration can all lead to various types of cutaneous lesions such as indentations of the face, skin tears, post-inflammatory hyperpigmentation, ulceration, crusting, erythema, and infection. The objective of this article is to provide effective and straightforward recommendations to those health care providers using facial masks in order to prevent skin-related complications. J Drugs Dermatol. 2020;19(9):858-864. doi:10.36849/JDD.2020.5259.

Ahmed, G. K., et al. (2021). "Comparison of knowledge, attitude, socioeconomic burden, and mental health disorders of COVID-19 pandemic between general population and health care workers in Egypt." Egypt J Neurol Psychiatr Neurosurg **57**(1): 25.

INTRODUCTION: The global devastating effect of COVID-19 has caused anxiety and fear to variable extent among the public. We aimed to evaluate the knowledge, attitude, socioeconomic burden, and the mental health problems regarding anxiety, depression, and obsessive-compulsive disorder during COVID-19 on the general population and HCWs in Egypt. METHODS: This study was conducted using a semi-structured online questionnaire in May 2020. Data on demographic features, socioeconomic scale, knowledge, and attitude regarding COVID-19 and the effect on different aspects of life were collected. Assessment was done using Arabic versions of Beck's Anxiety Inventory, Beck's Depression Inventory-II, and Yale-Brown Obsessive-Compulsive Scale. We divided participants into non-health care workers (non-HCWs) and HCWs groups. RESULTS: There were 524 participants who responded to the survey from 23 governorates. More than half of the participants were females (57.4%), middle age (53%), and middle socioeconomic class (66.6%). Non-HCWs were 402 and HCWs were 122. Most participants had good knowledge about the disease and a positive attitude toward protective measures particularly in HCWs. COVID-19 showed negative impact on different aspects of participants' life. HCWs had higher frequency of anxiety (32%) and OCD (29%) than non-HCWs (30% and 28%, respectively) while non-HCWs had higher depression (69%) than HCWs (66.4%). HCWs had higher rates of severe depression (20.5%) with moderate and severe OCD (4.9%, 1.6% respectively) than non-HCWs. Female gender, young age, urban residence, students, smoking, history of medical illness, and low socioeconomic class were significant associated factors. CONCLUSIONS: Health care workers had good knowledge about COVID-19 and a positive attitude toward the protective measures relative to non-HCWs. COVID-19 had a negative impact on different aspects of life and had a major association with the anxiety, depression, and OCD in both groups. Health professionals are more likely to have these psychological consequences. SUPPLEMENTARY INFORMATION: The online version contains supplementary material available at 10.1186/s41983-021-00280-w.

Ahmed, J., et al. (2020). "Availability of Personal Protective Equipment (PPE) Among US and Pakistani Doctors in COVID-19 Pandemic." Cureus **12**(6): e8550.

Background The coronavirus disease (COVID-19) pandemic has put an excessive strain on healthcare systems across the globe, causing a shortage of personal protective equipment (PPE). PPE is a precious commodity for health personnel to protect them against infections. We investigated the availability of PPE among doctors in the United States (US) and Pakistan. Methods A cross-sectional study, including doctors from the US and Pakistan, was carried out from April 8 to May 5, 2020. An online self-administered questionnaire was distributed to doctors working in hospitals in the US and Pakistan after a small pilot study. All analysis was done using Statistical Package for Social Science (SPSS) version 23.0 (IBM Corp., Armonk, NY). Results After informed consent, 574 doctors (60.6% from Pakistan and 39.4% from the US) were included in the analysis. The majority of the participants were females (53.3%), and the mean age of the participants was 35.3 ± 10.3 years. Most doctors (47.7%) were from medicine and allied fields. Among the participants, 87.6% of doctors from the US reported having access to masks/N95 respirators, 79.6% to gloves, 77.9% to face-shields or goggles, and 50.4% to full-suit/gown. Whereas, doctors in Pakistan reported to have poor availability of PPE with only 37.4% having access to masks/N95 respirator, 34.5% to gloves, 13.8% to face-shields or goggles, and 12.9% to full-suit/gown. The reuse of PPE was reported by 80.5% and 60.3% physicians from the US and Pakistan, respectively. More doctors from Pakistan (50.6%) reported that they had been forced to work without PPE compared to doctors in the US (7.1%). Conclusion There is a lack of different forms of PPE in the US and Pakistan. Doctors from both countries reported that they had been forced to work without PPE. Compared to the US, more doctors from Pakistan reported having faced discrimination in receiving PPE.

Ahmed, M. K., et al. (2020). "Protecting healthcare workers during COVID-19 pandemic with nanotechnology: A protocol for a new device from Egypt." J Infect Public Health **13**(9): 1243-1246.

The outbreak of the coronavirus disease 2019 (COVID-19) caused by severe acute respiratory syndrome coronavirus 2 (SARS-CoV-2) is thought to have occurred first in Wuhan, China in December 2019, before spreading to over 120 countries in the months that followed. It was declared a "public health emergency of international concern" by the World Health Organization on January 31, 2020 and recognized as a pandemic on March 11, 2020. The primary route of SARS-CoV-2 transmission from human to human is through inhalation of respiratory droplets. Devising protective technologies for stopping the spread of the droplets of aerosol containing the viral particles is a vital requirement to curb the ongoing outbreak. However, the current generations of protective respirator masks in use are noted for their imperfect design and there is a need to develop their more advanced analogues, with higher blockage efficiency and the ability to deactivate the trapped bacteria and viruses. It is likely that one such design will be inspired by nanotechnologies. Here we describe a new design from Egypt, utilizing a reusable, recyclable, customizable, antimicrobial and antiviral respirator facial mask feasible for mass production. The novel design is based on the filtration system composed of a nanofibrous matrix of polylactic acid and cellulose acetate containing copper oxide nanoparticles and graphene oxide nanosheets and produced using the electrospinning technique. Simultaneously, the flat pattern fabricated from a thermoplastic composite material is used to provide a solid fit with the facial anatomy. This design illustrates an effort made in a developing setting to provide innovative solutions for combating the SARS-CoV-2 pandemic of potentially global significance.

Ahuja, A. S., et al. (2020). "The Ocular Manifestations of COVID-19 Through Conjunctivitis." Cureus **12**(12): e12218.

There is growing evidence that the novel coronavirus (SARS-CoV-2) is capable of transmission through the eye. Research suggests that infection by SARS-CoV-2 can produce an inflammation of the conjunctiva, which leads to redness and itchiness of the eyes. Furthermore, viral particles have been detected in conjunctival secretions of SARS-CoV-2 patients who present with conjunctivitis and is likely another mode of transmission. A 53-year-old male presented with a complaint of left eye irritation and upper eyelid swelling for the past 24 hours. The right eye had mild irritation but no lid swelling. The left upper eyelid was erythematous, swollen and had crusting along the lashes. There were mild inflammation and injection of the conjunctiva. The initial diagnosis was blepharitis, and it was recommended that he continue with the warm compresses, and doxycycline 100 mg to use if the symptoms worsened or did not improve. The patient underwent SARS-CoV-2 PCR testing as a requirement for travel the next day and was found to be positive for the virus. Over the following days, he developed fatigue and rhinitis but clinically improved within six days of his initial presentation. Physicians and health care workers should be aware of the ocular manifestations of SARS-CoV-2 to make a timely diagnosis of infected individuals. While requirements vary across institutions, it is highly recommended that healthcare workers consistently wear appropriate eye protection when interacting with patients to reduce the spread of disease and potential impact on ocular health from SARS-CoV-2. Additionally, to prevent ocular transmission, all healthcare workers should be immediately educated on the importance of eye protection.

Akram, F. (2021). "Moral Injury and the COVID-19 Pandemic: A Philosophical Viewpoint." Ethics Med Public Health: 100661.

INTRODUCTION: Much has changed in healthcare during the coronavirus disease (COVID)-19 pandemic. Medicine, a profession of traditional principles and virtues, has faced unprecedented challenges in the light of scarce and unequal distribution of ventilators, testing, and personal protective equipment. Healthcare workers have been- and are increasingly likely to be- forced into situations that require difficult decision making under life-and-death conditions. Concepts of "medical necessity" and "maximum benefit" challenge healthcare systems that already struggle to manage unequal treatment and access to services, giving rise to moral distress and moral injury on the front lines. METHOD: This article focuses on moral injury in the context of coronavirus disease (COVID)-19 pandemic. I review recent literature to highlight the psychological impact of many morally-injurious events that have been reported during the COVID-19 pandemic. With the help of a clinical vignette, I point out how healthcare systems adopt many utilitarian policies in times of excessive healthcare burden and offer a viewpoint that many morally injurious events happen when physicians, traditionally practicing Kantian and virtue ethics, are forced to follow utilitarian policies of healthcare system. CONCLUSION: One form of moral injury may arise from inherent conflicts between individual deontological moral judgments and organizational utilitarian moral judgments. More research is needed to validate the philosophical viewpoint as well as to explore whether increased awareness and education of key principles within moral philosophy can better equip healthcare workers in situations when public health takes precedence over individual health.

Akuamoa-Boateng, D., et al. (2020). "Managing patient flows in radiation oncology during the COVID-19 pandemic : Reworking existing treatment designs to prevent infections at a German hot spot area University Hospital." Strahlenther Onkol **196**(12): 1080-1085.

PURPOSE: The described work aimed to avoid cancellations of indispensable treatments by implementing active patient flow management practices and optimizing infrastructure utilization in the radiation oncology department of a large university hospital and regional COVID-19 treatment center close to the first German SARS-CoV‑2 hotspot region Heinsberg in order to prevent nosocomial infections in patients and personnel during the pandemic. PATIENTS AND METHODS: The study comprised year-to-date intervention analyses of in- and outpatient key procedures, machine occupancy, and no-show rates in calendar weeks 12 to 19 of 2019 and 2020 to evaluate effects of active patient flow management while monitoring nosocomial COVID-19 infections. RESULTS: Active patient flow management helped to maintain first-visit appointment compliance above 85.5%. A slight appointment reduction of 10.3% daily (p = 0.004) could still significantly increase downstream planning CT scheduling (p = 0.00001) and performance (p = 0.0001), resulting in an absolute 20.1% (p = 0.009) increment of CT performance while avoiding overbooking practices. Daily treatment start was significantly increased by an absolute value of 18.5% (p = 0.026). Hypofractionation and acceleration were significantly increased (p = 0.0043). Integrating strict testing guidelines, a distancing regimen for staff and patients, hygiene regulations, and precise appointment scheduling, no SARS-CoV‑2 infection in 164 tested radiation oncology service inpatients was observed. CONCLUSION: In times of reduced medical infrastructure capacities and resources, controlling infrastructural time per patient as well as optimizing facility utilization and personnel workload during treatment evaluation, planning, and irradiation can help to improve appointment compliance and quality management. Avoiding recurrent and preventable exposure to healthcare infrastructure has potential health benefits and might avert cross infections during the pandemic. Active patient flow management in high-risk COVID-19 regions can help Radiation Oncologists to continue and initiate treatments safely, instead of cancelling and deferring indicated therapies.

Akudjedu, T. N., et al. (2021). "Impact of the COVID-19 pandemic on clinical radiography practice in low resource settings: The Ghanaian radiographers' perspective." Radiography (Lond) **27**(2): 443-452.

INTRODUCTION: The COVID-19 pandemic has altered the professional practice of all healthcare workers, including radiographers. In the pandemic, clinical practice of radiographers was centred mostly on chest imaging of COVID-19 patients and radiotherapy treatment care delivery to those with cancer. This study aimed to assess the radiographers' perspective on the impact of the pandemic on their wellbeing and imaging service delivery in Ghana. METHODS: A cross-sectional survey of practising radiographers in Ghana was conducted online from March 26th to May 6th, 2020. A previously validated questionnaire that sought information regarding demographics, general perspectives on personal and professional impact of the pandemic was used as the research instrument. Data obtained was analysed using Microsoft Excel® 2016. RESULTS: A response rate of 57.3% (134/234) was obtained. Of the respondents, 75.4% (n = 101) reported to have started experiencing high levels of workplace-related stress after the outbreak. Three-quarters (n = 98, 73.1%) of respondents reported limited access to any form of psychosocial support systems at work during the study period. Half (n = 67, 50%) of the respondents reported a decline in general workload during the study period while only a minority (n = 18, 13.4%) reported an increase in workload due to COVID-19 cases. CONCLUSION: This national survey indicated that majority of the workforce started experiencing coronavirus-specific workplace-related stress after the outbreak. Albeit speculative, low patient confidence and fear of contracting the COVID-19 infection on hospital attendance contributed to the decline in general workload during the study period. IMPLICATIONS FOR PRACTICE: In order to mitigate the burden of workplace-related stress on frontline workers, including radiographers, and in keeping to standard practices for staff mental wellbeing and patient safety, institutional support structures are necessary in similar future pandemics.

Akyala, A. I., et al. (2020). "Severe Acute Respiratory Syndrome Coronavirus 2 (SARS-CoV-2) infection among health care workers in Nasarawa State, Nigeria: implications for infection prevention and control measures." Pan Afr Med J **37**(Suppl 1): 21.

INTRODUCTION: health care workers (HCWs) are on the frontline, waging war against SARS-CoV-2 and have a higher risk of infection with exposure to an infected person with SARS-CoV-2. There is a paucity of information on clinical characteristics and infection risk gradient of HCWs with SARS-CoV-2 with the view to marshal preventive measures. METHODS: we conducted a multi-center case series analysis of 648 HCWs who were randomly selected in private and public hospitals across Nasarawa State, managing cases of SARS-CoV-2. Demographic and epidemiological information, were abstracted from electronic medical records of cases from February to July 2020. Throat and Nasopharyngeal swabs and real-time reverse transcriptase-polymerase chain reaction (RT-PCR) tests for SARS-CoV-2 nucleic acid were performed. RESULTS: overall, 134 of 648 HCWs across health centers in Nasarawa State tested positive for SARS-CoV-2. Eighty male HCWs constituted 30.9% of respondents with a median (interquartile range) age of 36.7 (30.0-47.0) years. Overall, 50 of 134 HCWs (67.5%) with SAR-COV-2 had mild disease. The five most common symptoms amongst cases were fever (67 [90.5%]), myalgia or fatigue (60 [81.1%]), cough (50[67.6%]), sore throat (50 [67.6%]), and muscle ache (50 [67.6%]). Contact with index patients (65 [59.1%]) and colleagues with infection (10 [13.9%]) as well as community-acquired infection (14 [18.9%]) were the main routes of exposure for HCWs. CONCLUSION: HCWs in Nasarawa State face an unprecedented occupational risk of morbidity and mortality as a result of SARS-CoV-2. There is need for rapid development of sustainable infection prevention control measures that protect HCWs from the SARS-CoV-2 ongoing pandemic.

Al Abri, Z. G. H., et al. (2021). "Risk Factors Associated with COVID-19 Infected Healthcare Workers in Muscat Governorate, Oman." J Prim Care Community Health **12**: 2150132721995454.

INTRODUCTION: Coronavirus disease 2019 (COVID-19) has spread rapidly worldwide, causing a global public health crisis. Healthcare workers (HCWs) are vulnerable due to their role in the management of COVID-19 infected patients. As of June 2020, a total of 847 HCWs in Oman had reportedly contracted COVID-19, with an incidence rate of 1.47%. This study therefore aimed to identify factors associated with COVID-19 infection among HCWs in Muscat Governorate, Oman, as well as to evaluate adherence to infection prevention and control (IPC) measures. METHODS: This cross-sectional study involved cases of laboratory-confirmed COVID-19 infection among HCWs working under the Directorate General of Health Services of Muscat Governorate, Ministry of Health, between February and June 2020. Data regarding the participants' sociodemographic characteristics, risk factors, pre-existing medical conditions, and adherence to IPC measures were collected using a self-administered questionnaire distributed via a web-based mobile application. RESULTS: A total of 126 HCWs with confirmed COVID-19 infection participated in the study. Of these, 72.2% were female, 53.2% worked in primary care facilities, and 61.1% were medical doctors or nurses. Only 18.1% were over 45 years of age and 30.2% had pre-existing medical conditions. While 29.4% had never received IPC training, the majority followed recommended hand hygiene practice (96.8%) and social distancing protocols (93.7%) and wore protective facemasks for routine patient care (96.9%). CONCLUSION: While the majority of HCWs followed crucial IPC measures, one-third had never received specific IPC training or faced restrictions on PPE use. HCWs, including those in housekeeping and administrative functions are recommended to undergo rigorous IPC training. In addition, high-risk HCWs could be assigned duties away from active COVID 19 cases. It is recommended to restructure health facilities for better adherence to IPC standards.

Al Kawas, S., et al. (2020). "Post COVID-19 lockdown: measures and practices for dental institutes." BMC Oral Health **20**(1): 291.

Resuming regular clinical activities at dental premises after the COVID-19 lockdown period or post COVID-19 is likely to be a challenge for all dental institutes. When returning to the dental practice or training, staff and students alike should abide by the new rules and regulations. In the process of controlling viral spread, clinical dental facilities face a higher risk of disease transmission among patients as well as clinical and non-clinical staff. Aerosols formation and diffusion into the surrounding air can be a real concern of viral transmission, if no protective measures are established. We aim in this review to present the currently implemented measures and propose changes in clinical dental facilities to minimize the risk of transmission. Dental professionals should be prepared to treat every patient as a suspected COVID-19 carrier and be ready to receive and manage an overwhelming number of patients. We suggest that dental practices establish a sensible workforce shift schedule, improve ventilation levels, reduce dental aerosol generating procedures, and develop a comprehensive guidance to Healthcare Workers to reduce the risk of COVID-19 transmission.

Al Lawati, A., et al. (2021). "Risk of COVID-19 Infection in Healthcare Workers Exposed During Use of Non-invasive Ventilation in a Tertiary Care Hospital in Oman." Oman Med J **36**(2): e236.

OBJECTIVES: Healthcare workers (HCWs), especially those working on the front line, are considered to be at high risk of nosocomial acquisition of the severe acute respiratory syndrome coronavirus 2 (SARS-CoV-2), the virus that causes coronavirus disease 2019 (COVID-19). Little is known about the effectiveness of the recommended protective methods as few reports have described spread of the disease in hospital settings among this high-risk population. We describe the hospital-based transmission of SARS-CoV-2 related to non-invasive ventilation (NIV) in one of the main tertiary care hospitals in Oman. METHODS: All exposed patients and HCWs from Royal Hospital were screened, quarantined, and underwent telephone interviews to stratify their risk factors, clinical symptoms, and exposure risk assessment. RESULTS: A total of 46 HCWs and patients tested positive for SARS-CoV-2 after exposure to an index case who received 48 hours of NIV before diagnosing COVID-19 infection. Over half of the exposed (56.5%; n = 26) were nurses, 26.1% (n = 12) were patients, and 15.2% (n = 7) were doctors. None of the HCWs required hospitalization. Sore throat, fever, and myalgia were the most common symptoms. CONCLUSIONS: NIV poses a significant risk for SARS-CoV-2 transmission within hospital settings if appropriate infection control measures are not taken.

Al Nsour, M., et al. (2020). "The Role of the Global Health Development/Eastern Mediterranean Public Health Network and the Eastern Mediterranean Field Epidemiology Training Programs in Preparedness for COVID-19." JMIR Public Health Surveill **6**(1): e18503.

The World Health Organization (WHO) declared the current COVID-19 a public health emergency of international concern on January 30, 2020. Countries in the Eastern Mediterranean Region (EMR) have a high vulnerability and variable capacity to respond to outbreaks. Many of these countries addressed the need for increasing capacity in the areas of surveillance and rapid response to public health threats. Moreover, countries addressed the need for communication strategies that direct the public to actions for self- and community protection. This viewpoint article aims to highlight the contribution of the Global Health Development (GHD)/Eastern Mediterranean Public Health Network (EMPHNET) and the EMR's Field Epidemiology Training Program (FETPs) to prepare for and respond to the current COVID-19 threat. GHD/EMPHNET has the scientific expertise to contribute to elevating the level of country alert and preparedness in the EMR and to provide technical support through health promotion, training and training materials, guidelines, coordination, and communication. The FETPs are currently actively participating in surveillance and screening at the ports of entry, development of communication materials and guidelines, and sharing information to health professionals and the public. However, some countries remain ill-equipped, have poor diagnostic capacity, and are in need of further capacity development in response to public health threats. It is essential that GHD/EMPHNET and FETPs continue building the capacity to respond to COVID-19 and intensify support for preparedness and response to public health emergencies.

Alabi, O. C., et al. (2020). "Association of gynecological endoscopy surgeons of Nigeria (AGES) advisory on laparoscopic and hysteroscopic procedures during the COVID-19 Pandemic." Niger J Clin Pract **23**(5): 747-749.

Coronavirus 2, or SARS-CoV-2 disease (COVID-19) is a global public health concern. Although there is a paucity of evidence to advise on the best practice, we recommend postponement of elective gynecological endoscopic surgeries until the pandemic is contained. Emergency surgeries should preferably be done through open surgeries than laparoscopy or hysteroscopy approach. However, if or when laparoscopy or hysteroscopy is considered, health personnel in theatre must wear appropriate personal protective equipment (PPE) and all standard precautions should be observed to prevent COVID-19 infection. When COVID-19 is highly suspected or confirmed, the patient should be referred to centers equipped in taking care of such cases.

Alajmi, J., et al. (2020). "COVID-19 infection among healthcare workers in a national healthcare system: The Qatar experience." Int J Infect Dis **100**: 386-389.

BACKGROUND: Our aim was to determine the prevalence of COVID-19 infection in healthcare workers (HCWs) in a national healthcare system and to understand the risk factors for infection. METHODS: The study was conducted at Hamad Medical Corporation (HMC) in Qatar, a national healthcare system with 14 hospitals and >28,000 employees, between March 10 and June 24, 2020. Data on COVID-19+ HCWs were retrieved from the electronic health records and employment records, followed by an email survey and a focused telephone interview. RESULTS: Among 16,912 HCWs tested, 10.6% tested positive. Hospitalization rate was 11.6%, 1.3% required supplemental oxygen, 0.6% needed intensive care unit admission, and 0.3% required mechanical ventilation. There were no deaths. In a follow-up survey of 393 HCWs, 5% reported acquiring infection at a COVID-19-designated facility and 95% at a non-COVID-19 facility having acquired the infection through accidental exposure to a colleague (45%) or a patient (29%). Full personal protective equipment (PPE) adherence was 82% at COVID-19-designated facilities but only 68% at non-COVID-19 facilities. CONCLUSIONS: COVID- 19 infection among HCWs often occurs among those not directly working with COVID-19 patients. PPE use is less stringent in such settings. Risk of exposure and need for strict PPE must be stressed upon all HCWs in all settings.

Alan, H., et al. (2020). ""I'm a hero, but…": An evaluation of depression, anxiety, and stress levels of frontline healthcare professionals during COVID-19 pandemic in Turkey." Perspect Psychiatr Care.

PURPOSE: It was aimed to evaluate depression, anxiety, stress symptoms of health professionals during the COVID-19 pandemic and to reveal the risk factors. DESIGN AND METHODS: Four hundred and sixteen professionals participated in this study. Data were collected online by Depression-Anxiety-Stress Scale. FINDINGS: A statistically significant, positive relationship was determined between professionals' perceptions of COVID-19 risk and scale scores. PRACTICE IMPLICATIONS: The professionals involved in the struggle against the COVID-19 have high levels of depression, anxiety, stress. It is recommended to revise the content to enable individuals to increase skills in coping with similar situations and to take measures to protect their health.

Albacker, T. B., et al. (2020). "Saudi Society for Cardiac Surgeons consensus document on COVID-19, April 1, 2020." Asian Cardiovasc Thorac Ann **28**(6): 307-311.

The COVID-19 pandemic represents an international health crisis that is challenging to all governments. Health practitioners in different fields have a duty to guide people and governments to achieve safe health practices. The Saudi Society for Cardiac Surgeons recognizes that it is difficult to establish evidence-based guidelines for safe cardiac surgery practices in such a crisis because this is an unprecedented health pandemic. So we decided to work with our colleagues in other societies by reviewing different recommendations and safe practices issued by different health organizations and scientific societies. The aim was to come up with recommendations that could guide hospitals, physicians, and other members of the healthcare team on the best available practices that are applicable to our community and that will not only ensure optimum patient care delivery but also protect healthcare workers from the risk of infection and aid the national effort in containing and managing this worldwide pandemic.

Al-Benna, S. (2020). "Protective Measures For Burn Care Professionals During The Coronavirus Disease 2019 Pandemic: Systematic Review." Ann Burns Fire Disasters **33**(3): 182-190.

The emergence of a novel human β coronavirus, severe acute respiratory syndrome coronavirus 2, which causes coronavirus disease 2019 (COVID-19), has developed into a global pandemic and public health emergency. The management of patients with burns must be adapted to this context. The aim of this systematic review is to identify the optimal protection measures during the COVID-19 pandemic and provide guidance of protective measures for burn surgeons. A systematic search of PubMed was performed for articles about COVID-19. "Burn units", "burns", "COVID-19", "health personnel", "protective devices", "severe acute respiratory syndrome coronavirus 2", "surgeons" and "telemedicine" were reviewed during the entire diagnosis and management process of burn patients. Eight articles were included, and five articles emphasized that burn care professionals should pay attention to prevent cross-infection. Only three articles reported in detail how burn care professionals should be protected during surgery in the operating room. These experiences and strategies can help burn care professionals work safely and effectively, and prevent both nosocomial infections and burn care professional infections during the global pandemic of COVID-19.

Alboraie, M., et al. (2020). "The global impact of COVID-19 on gastrointestinal endoscopy units: An international survey of endoscopists." Arab J Gastroenterol **21**(3): 156-161.

BACKGROUND & STUDY AIMS: Corona virus disease-19 (COVID-19) pandemic has markedly impacted routine medical services including gastrointestinal (GI) endoscopy. We aim to report the real-life performance in high volume GI endoscopy units during the pandemic. PATIENTS AND METHODS: A web-based survey covering all aspects of daily performance in GI endoscopy units was sent to endoscopy units worldwide. Responses were collected and data were analyzed to reveal the effect of COVID-19 pandemic on endoscopy practice. RESULTS: Participants from 48 countries (n = 163) responded to the survey with response rate of 67.35%. The majority (85%) decreased procedure volume by over 50%, and four endoscopy units (2.45%) completely stopped. The top three indications for procedures included upper GI bleeding (89.6%), lower GI bleeding (65.6%) and cholangitis (62.6%). The majority (93.9%) triaged patients for COVID-19 prior to procedure. N95 masks were used in (57.1%), isolation gowns in (74.2%) and head covers in (78.5%). Most centers (65%) did not extend use of N95 masks, however 50.9% of centers reused N95 masks. Almost all (91.4%) centers used standard endoscopic decontamination and most (69%) had no negative pressure rooms. Forty-two centers (25.8%) reported positive cases of SARS-CoV-2 infection among patients and 50 (30.7%) centers reported positive cases of SARS-CoV-2 infection among their healthcare workers. CONCLUSIONS: Most GI endoscopy centers had a significant reduction in their volume and most procedures performed were urgent. Most centers used the recommended personal protective equipment (PPE) by GI societies however there is still a possibility of transmission of SARS-CoV-2 infection in GI endoscopy units.

Alcocer-Gamba, M. A., et al. (2020). "Excerpts from the documents of Mexican positions and recommendations in cardiovascular diseases and COVID-19." Arch Cardiol Mex **90**(Supl): 100-110.

The recommendations in which the Mexican Society of Cardiology (SMC) in conjunction with the National Association of Cardiologists of Mexico (ANCAM) as well as different Mexican medical associations linked to cardiology are presented, after a comprehensive and consensual review and analysis of the topics related to cardiovascular diseases in the COVID-19 pandemic. Scientific positions are analyzed and responsible recommendations on general measures are given to patients, with personal care, healthy eating, regular physical activity, actions in case of cardio-respiratory arrest, protection of the patient and health personnel as well as precise indications in the use of non-invasive cardiovascular imaging, prescription of medications, care in specific topics such as systemic arterial hypertension, heart failure, arrhythmias and acute coronary syndromes, in addition to emphasizing electrophysiology, interventionism, cardiac surgery and in cardiac rehabilitation. The main interest is to provide the medical community with a general orientation on what to do in daily practice and patients with cardiovascular diseases in the setting of this unprecedented epidemiological crisis of COVID-19.

Alenazi, T. H., et al. (2020). "Prevalence and predictors of anxiety among healthcare workers in Saudi Arabia during the COVID-19 pandemic." J Infect Public Health **13**(11): 1645-1651.

BACKGROUND: During pandemics, healthcare workers (HCWs) may be prone to higher levels of anxiety than those of the general population. This study aimed to explore the anxiety levels among HCWs in Saudi Arabia during the COVID-19 pandemic and the predictors of increased anxiety levels. METHOD: HCW participants in this cross-section study were solicited by email from the database of registered practitioners of the Saudi Commission for Health Specialties between 15 May and 18 May 2020. Sociodemographic characteristics, work-related factors, and organization-related factors were collected. RESULTS: Four thousand nine hundred and twenty HCWs (3.4%) responded. Reported levels of anxiety were low anxiety (31.5%; n = 1552), medium (36.1%; n = 1778), and high (32.3%; n = 1590). Participants reporting high anxiety levels were more likely to be unmarried (OR = 1.32, 95% CI: 1.14-1.52); nurses (OR = 1.54, 95% 1.24-1.91); workers in radiology (OR = 1.52, 95% CI: 1.01-2.28); or respiratory therapists (OR = 2.28, 95% CI: 1.14-4.54). Social factors associated with high anxiety levels were: living with a person who is elderly (p = 0.01), has a chronic disease (p < 0.0001), has immune deficiency (p < 0.0001), or has a respiratory disease (p-value <0.0001). Organization-related factors associated with a high level of anxiety were: working in an organization that hosts COVID-19 patients and working with such patients (p-value <0.0001). CONCLUSION: Self-reported medium and high levels of anxiety were present in 68.5% of HCWs in the COVID-19 pandemic. This highlights the urgent need to identify high-risk individuals to offer psychological support and provide up to date information on the pandemic. These data should help policymakers drive initiatives forward to protect and prepare HCWs psychological wellbeing.

Alhalaseh, Y. N., et al. (2020). "Allocation of the "Already" Limited Medical Resources Amid the COVID-19 Pandemic, an Iterative Ethical Encounter Including Suggested Solutions From a Real Life Encounter." Front Med (Lausanne) **7**: 616277.

The shortage of healthcare providers is well-documented in low-income countries (LIC) prior to COVID-19, due to various causes including the migration to developed countries, scarcity of supplies, poor healthcare infrastructure, limited ICU facilities, and lack of access to guidelines and protocols. One of the important hitches in LIC is the insufficient testing capacity that precluded accurate assessment of disease burden and subsequently resource allocations. Trying to adhere to the principles of bioethics including respect to others, beneficence, and justice should be applied on the ground in the particular setting of the LIC. Solutions should be tailored to the tangible needs and possibility of implementation in real life in the face of the "already" limited resources by making use of simple, yet plausible, measures. Implementing guidelines and frameworks that were set to work in the better-resourced nations is a call for futility. The adoption of novel solutions to overcome the unique challenges in the LIC is exigent. These include the use of automated screening algorithms and virtual video clinics. Moreover, integrating electronic intensive care unit (e-ICU) software may allow for remote monitoring of multiple patients simultaneously. Telemedicine could help in getting consultations worldwide. It can also enhance healthcare workers' knowledge and introduce new skills through teleconferences, e-workshops, and free webinars. Healthcare workers can be remotely trained to enhance their skills. Agencies, such as the WHO, should develop comprehensive programs to tackle different health issues in LIC in collaboration with major institutions and experts around the world.

Ali, S., et al. (2020). "Psychological impact of the COVID-19 pandemic on healthcare workers at acute hospital settings in the South-East of Ireland: an observational cohort multicentre study." BMJ Open **10**(12): e042930.

OBJECTIVE: Our study aims to understand the psychological impact of the COVID-19 pandemic among healthcare workers (HCWs) at acute hospital settings in the South-East of Ireland, as a crucial step in guiding policies and interventions to maintain their psychological well-being. DESIGN: Observational cohort study. PARTICIPANTS AND SETTING: 472 HCWs participated from two distinct acute hospital settings, A and B, in the South-East of Ireland. PRIMARY AND SECONDARY OUTCOME MEASURES: Measures of psychological distress-depression, anxiety, acute and post-traumatic stress disorder (PTSD)-as dictated by the Depression, Anxiety and Stress Scale (DASS-21) and Impact of Event Scale-Revised (IES-R). An independent sample t-test and a Mann-Whitney U test was used to determine significance of difference in continuous variables between groups. Categorical variables were assessed for significance with a χ(2) test for independence. RESULTS: The DASS-21 provided independent measures of depression (mean 4.57, IQR 2-7), anxiety (mean 3.87, IQR 1-6) and stress (mean 7.41, IQR 4-10). Positive scores were reflected in 201 workers (42.6%) for depression and 213 (45.1%) for both anxiety and stress. The IES-R measured subjective distress on three subscales: intrusion (mean 1.085, IQR 0.375-1.72), avoidance (mean 1.008, IQR 0.375-1.5) and hyperarousal (mean 1.084, IQR 0.5-1.667). Overall, 195 cases (41.3%) were concerning for PTSD. Site B scored significantly higher across all parameters of depression (5.24 vs 4.08, p<0.01), anxiety (4.66 vs 3.3, p<0.01), stress (8.91 vs 6.33, p<0.01) and PTSD (0.058 vs 0.043, p<0.01). Worse outcomes were also noted in HCWs with underlying medical ailments. CONCLUSION: Psychological distress is prevalent among HCWs during the COVID-19 pandemic; screening for adverse mental and emotional outcomes and developing timely tailored preventative measures with effective feedback are vital to protect their psychological well-being, both in the immediate and long-term.

Ali, S., et al. (2020). "Risk Assessment of Healthcare Workers at the Frontline against COVID-19." Pak J Med Sci **36**(Covid19-s4): S99-s103.

The novel coronavirus disease 2019 (COVID-19) is a global pandemic. Healthcare workers (HCWs) are on the frontline of treating patients infected with COVID-19. However, data related to its infection rate among HCWs are limited. The aim was to present evidence associated with the number of HCWs being infected with COVID-19 from most viral affected countries (Italy, China, United States, Spain, and France). Furthermore, we looked into the reasons for HCWs COVID 19 infections and strategies to overcome this problem. Early available evidence suggested that HCWs are being increasingly infected with the novel infection ranging from 15% to 18% and in some cases up to 20% of the infected population. Major factors for infection among HCWs include lack of understanding of the disease, inadequate use and availability of Personal Protective Equipment (PPE), uncertain diagnostic criteria, unavailability of diagnostic tests and psychological stress. Therefore the protection of HCWs by authorities should be prioritized through education and training, the readiness of staff, incentives, availability of PPEs, and psychological support.

Ali, S. M. E. and S. Fatima (2020). "Plan for blood banks to protect blood donors and healthcare workers during COVID-19 pandemic." Hematol Transfus Cell Ther **42**(4): 316-317.

Ali, Z., et al. (2020). "Development of a preoperative Early Warning Scoring System to identify highly suspect COVID-19 patients." J Anaesthesiol Clin Pharmacol **36**(Suppl 1): S62-s74.

BACKGROUND AND AIMS: The coronavirus disease 2019 (COVID-19) is spreading at an unprecedented speed. Lack of resources to test every patient scheduled for surgery and false negative test results contribute to considerable stress to anesthesiologists, along with health risks to both caregivers and other patients. The study aimed to develop an early warning screening tool to rapidly detect 'highly suspect' among the patients scheduled for surgery. METHODS: Review of literature was conducted using terms 'coronavirus' OR 'nCoV 2019' OR 'SARS-CoV-2' OR 'COVID-19' AND 'clinical characteristics' in PUBMED and MedRxiv. Suitable articles were analysed for symptoms and investigations commonly found in COVID-19 patients. Additionally, COVID-19 patient's symptomatology and investigation profiles were obtained through a survey from 20 COVID-19 facilities in India. Based on literature evidence and the survey information, an Early Warning Scoring System was developed. RESULTS: Literature search yielded 3737 publications, of which 195 were considered relevant. Of these 195 studies, those already included in the meta-analyses were not considered for independent assessment. Based on the combined data from meta-analyses and survey, risk factors of COVID-19 disease identified were as follows: history of exposure, fever, cough, myalgias, lymphocytopaenia, elevated C-reactive protein (CRP)/lactate dehydrogenase (LDH) and radiographic infiltrates. CONCLUSION: Development of this Early Warning Scoring System for preoperative screening of patients may help in identifying 'highly suspect' COVID-19 patients, alerting the physician and other healthcare workers on the need for adequate personal protection and also to implement necessary measures to prevent cross infection and contamination during the perioperative period.

Allan, P. J., et al. (2021). "COVID-19 infection in patients with intestinal failure: U.K. experience." JPEN J Parenter Enteral Nutr.

BACKGROUND: The direct effect of the COVID-19 pandemic on patients with intestinal failure (IF) has not been described. METHODS: We conducted a nationwide study of U.K. IF centres to evaluate the infection rates, presentations and outcomes in patients with types 2 and 3 IF. RESULTS: A total of 45 patients with IF contracted COVID-19 between March and August 2020; this included 26 of 2191 (1.2%) Home Parenteral Nutrition (HPN)-dependent adults and 19 of 298 (6.4%) adults hospitalized with type 2 IF. The proportion of patients receiving nursing care for HPN administration was higher in those with community-acquired COVID-19 (66.7%) than the proportion in the entire HPN cohort (26.1%; p<0.01). Two HPN-dependent and 1 hospitalised patient with type 2 IF died as a direct consequence of the virus (6.7% of 45 patients with types 2 or 3 infected). CONCLUSION: This is the first study to describe the outcomes of COVID-19 in a large cohort of patients of requiring long term PN. Methods to reduce hospital and community nosocomial spread would likely be beneficia. This article is protected by copyright. All rights reserved.

Allen, B. D., et al. (2020). "Society for Cardiovascular Magnetic Resonance (SCMR) guidance for re-activation of cardiovascular magnetic resonance practice after peak phase of the COVID-19 pandemic." J Cardiovasc Magn Reson **22**(1): 58.

During the peak phase of the COVID-19 pandemic, alterations of standard operating procedures were necessary for health systems to protect patients and healthcare workers and ensure access to vital hospital resources. As the peak phase passes, re-activation plans are required to safely manage increasing clinical volumes. In the context of cardiovascular magnetic resonance (CMR), re-activation objectives include continued performance of urgent CMR studies and resumption of CMR in patients with semi-urgent and elective indications in an environment that is safe for both patients and health care workers.

Alluhayyan, O. B., et al. (2020). "Occupational-Related Contact Dermatitis: Prevalence and Risk Factors Among Healthcare Workers in the Al'Qassim Region, Saudi Arabia During the COVID-19 Pandemic." Cureus **12**(10): e10975.

Objective This study aimed to estimate and investigate the prevalence and the risk factors implicated in contact dermatitis among healthcare workers in the Al'Qassim region, Saudi Arabia, during the COVID-19 pandemic. Methodology We conducted a cross-sectional survey among healthcare workers at hospitals in the Al'Qassim region. Data was collected using a standardized and validated Nordic Occupational Skin Questionnaire version 2002. We included 408 participants in the analysis. Results The majority of the respondents (66.7%) were females. The mean age of participants was 34 (SD: ±9) years. Most of the participants who reported contact dermatitis were nurses (58.6%). Direct patient care roles represent 78% of participants. Respondents who work 40-50 hours per week represent 61.5% of the sample. The most commonly recorded symptoms were dryness (92.9%), itchiness (50%), and redness (46.4%) of the skin. The most affected site was hand 93.5%. Hand cleanser was the commonest substance implicated in the worsening of the skin changes (59.2%). Protective glove material that worsens contact dermatitis, such as natural rubber/latex, represents 76% of responses. A significant association (p=0.001)was seen in the occurrence of contact dermatitis in those with a history of allergic eye symptoms (33.3%) and those without (58%). Participants with a mean age of 26.47 years were more prone to develop contact dermatitis (CI: 1.19-7.06; p=0.067). Pharmacists and interns had 3.69 and 4.90 times higher odds of having contact dermatitis (CI: 0.95-7.33; CI: 22.1; p=0.027; p=0.038, respectively). Those involved in patient education and research activities at work were 6.48 (p=0.017) and 20.51 (p=0.024) times likely to develop contact dermatitis (CI: 1.38-30.31; CI: 1.49-282.15, respectively). Conclusions We explored the prevalence and risk factors for occupational contact dermatitis among healthcare workers in Saudi Arabia. The prevalence of reported skin changes during the pandemic was 46.4%. Our study also showed that the risk factors of developing contact dermatitis include female gender, history of eye allergies, and young age group.

Almadi, M. A., et al. (2020). "COVID-19 and endoscopy services in intermediately affected countries: a position statement from the saudi gastroenterology association." Saudi J Gastroenterol **26**(5): 240-248.

With the global pandemic due to coronavirus disease 2019 (COVID-19), there has been a significant strain on healthcare facilities. The infectivity rate, as well as the rate of healthcare workers who have fallen ill to the disease, has raised concerns globally on the proper management of patients as well as the role of safe healthcare provision utilizing personal protective equipment (PPE). Furthermore, the limited supply of PPEs has mandated rationing their use to achieve maximum utility and preservation. Multiple gastroenterology associations have issued guidance and statements that would help healthcare providers in navigating these unprecedented and difficult times, and the Saudi Gastroenterology Association has provided this statement in an effort to bring the most up to date information for the management of endoscopy units in terms of resources, manpower planning, scheduling, as well as infection control policies and leadership.

Almaghrabi, R. H., et al. (2020). "Healthcare workers experience in dealing with Coronavirus (COVID-19) pandemic." Saudi Med J **41**(6): 657-660.

To analyze healthcare workers experiences in dealing with the Coronavirus (COVID-19) pandemic. Methods: An anonymous open web-based survey study was conducted among healthcare workers from the March 2020 to April 2020. A total of 24 relevant questions were asked based on participants' characteristics, obligations, and preparedness in healthcare workers in the event of COVID-19 pandemic. Results: Approximately 1036 healthcare workers participated in this study with high response rate. Out of all the participants, 70% were women, 52% belonged to the 26-34 year age range, 50% were nurses, 33.7% were clinicians, 74.3% agreed to work overtime, 93.1% understand why they should stay past their shift end, 97.7% thought that preventing illness among healthcare workers and providing safety to family members, nearly 94% thought that personal protective equipment (PPE) for employees will enhance their willingness to report to work. Approximately 89.3% express a desire for incentives and financial support for family members. Conclusion: We recommend that providing PPE, reducing psychological stress, financial support and safety to family members of healthcare workers will increase the willingness to report to work.

Almhawish, N., et al. (2021). "Protecting healthcare workers in conflict zones during the COVID-19 pandemic: Northwest Syria." J Infect.

Alorjani, M., et al. (2021). "Cellular pathology practice in the era of COVID-19 pandemic-related lockdowns - Experience from a tertiary hospital: A retrospective observational study." Ann Med Surg (Lond) **63**: 102155.

BACKGROUND: The COVID-19 pandemic had many implications on healthcare services, including cellular pathology. The pandemic-related lockdown was applied in Jordan from March to May 2020. King Abdullah University Hospital (KAUH) was chosen to provide care for COVID-19 patients during that period. Since there was no experience in dealing with COVID-19 patients, the hospital maintained some essential services but canceled elective surgeries and procedures. The rationale was to prioritize care for COVID-19 patients and to provide better adherence to infection control policies and protect non-infected patients and healthcare workers. The purpose of the present study is to investigate the impact of COVID-19 pandemic restrictions on cellular pathology practice patterns at KAUH. METHODS: This is a retrospective observational study conducted at KAUH. All cellular pathology reports during the 2020 national lockdown were retrieved. The total numbers of specimens including types and procedures were recorded. Data were compared with the corresponding data in 2019 when there was no pandemic and when hospital and laboratory services were run in full capacity. RESULTS: 2020 lockdown period showed a 57.9% reduction in the total number of specimens received at the cellular pathology laboratory as compared to the corresponding period of 2019 (1400 versus 3322). Emergency procedures have represented 99.1% of the service during the lockdown with a remarkable diversity shift. CONCLUSION: There was a significant drop in the number of specimens dealt with at KAUH cellular pathology laboratory during the COVID-19 pandemic-related national lockdown. We learned from this pandemic how to adapt to such circumstances by adjusting our way of working to reach the best level of staff safety while maintaining highly productive work. Implementing digital pathology platforms, working from home strategies and alternative training methodologies have emerged as an essential need.

Alqahtani, S. A., et al. (2020). "Protected Stroke Mechanical Thrombectomy Code During the Coronavirus (COVID-19) Pandemic: Southwestern Part of Saudi Arabia Stroke Unit Local Protocol." Cureus **12**(4): e7808.

Cerebrovascular diseases are a significant cause of mortality and morbidity worldwide, in particular those with large vessels occlusion (LVO). Coronavirus disease 2019 (COVID-19) has become a global crisis rapidly since its initial outbreak in Wuhan, China, in December, 2019. Stroke due to LVO needs rapid assessment and timely endovascular intervention which can be very challenging during the time of pandemic where you need to deliver proper, safe, and timely care to acute ischemic stroke (AIS) patients with LVO, yet, protecting healthcare workers and existing patients at the medical facility. In this article, we share our local experience in the stroke unit at Aseer Central Hospital which is the main hub of stroke patients in the southwestern part of Saudi Arabia and the primary regional COVID center to provide guidance to perform smooth, safe, and swift mechanical thrombectomy during the coronavirus (COVID-19) pandemic as well as possible similar future situations.

Alreshidi, N. M., et al. (2020). "Assessing healthcare workers' knowledge, emotions and perceived institutional preparedness about COVID-19 pandemic at Saudi hospitals in the early phase of the pandemic." J Public Health Res **9**(4): 1936.

Background: Coronavirus disease 2019 (COVID-19) pandemic extended to reach most countries in the globe during few months. Preparedness of healthcare institutions and healthcare workers (HCWs) are crucial for applying effective prevention and control measures. This study aimed to assess HCWs knowledge, emotions and perception of preparedness of their institutions towards COVID-19 pandemic. Design: A cross-sectional, web-based survey was conducted among hospital HCWs in Saudi Arabia during April 27, 2020 to May 03, 2020. Results: Overall, 1004 completed responses were received. The majority were females (78.8), nurses (84.9%) at middle age 25-39 years (71.8%). Among participants, 95.5% reported receiving training on safely use of personal protective equipment (PPE) and 94.9% did fit the test for N95 respirator. The participants possessed a fair knowledge about COVID-19 disease with a mean knowledge score 6. 61±1.35 points on a scale of 10 points. Most participants (88.7%) were committed to continue work as a professional and ethical duty, however, 27.1% of them scored high on a negative emotional impact scale. Participants appreciated most aspects of institutional preparedness for COVID-19 pandemic; however, they were concerned with the continuous PPE supply. Factors that independently associated with good knowledge and negative emotional response were determined using multivariate logistic regression analysis. Conclusions: Findings revealed fair knowledge about COVID-19 pandemic among HCWs in Saudi hospitals. Concerns and worries were expressed regard working with the highly infectious COVID-19 patients. Participants, appreciated most aspects of institutional preparedness, however they were concerned about the continuous availability and supply of PPE.

Alsayedahmed, H. H. (2020). "COVID-19 Pandemic's precautionary measures had hit the reset button of the quality of life at different aspects." J Infect Dev Ctries **14**(8): 812-816.

COVID-19 is a novel coronavirus that emerged in Wuhan-China during the last quarter in 2019 and caused an infectious respiratory disease pandemic. The strategy of "Attack and Defend" was attempted to mitigate COVID-19 crisis worldwide. A list of precautionary measures were set by healthcare professional experts to protect public from infection, social physical distancing measures being the most efficient. On the other hand, Occupational Safety and Health Administration (OSHA) has established protective guidelines for healthcare workers to guarantee a healthy workplace and safe environment. The physical distancing measures have obviously reshaped the relationship between family members leading to better quality of family life. Meanwhile, travel suspension also led to slightly higher health standards with less air pollution, lower traffic induced stress levels and subsequently a greener footprint. Herein, we intend to highlight the impact of COVID-19 pandemic protective measures on family connections, as well as on environmental health. Moreover, we aimed at pointing out the effect on Saudi Arabian cultural and humanitarian behavior in dealing with the pandemic under the governance care of the custodian of the two holy mosques; King Salman Al-Saud. In fact, a combination of restrictive and protective measures is the most efficient to curb COVID-19 spread. In addition to improving the quality of social life, this pandemic revealed a healthier climate, which will certainly be visible on a large scale. Certainly, all together with solidarity we will be able to restrain COVID-19 pandemic and have a better quality of life.

Al-Tawfiq, J. A., et al. (2020). "Changes in healthcare managing COVID and non-COVID-19 patients during the pandemic: striking the balance." Diagn Microbiol Infect Dis **98**(4): 115147.

Following the emergence of the coronavirus disease 19 (COVID-19) pandemic, healthcare organizations began concentrating on the preparation for and management of the surge of COVID-19 cases while trying to protect the healthcare workers and other patients from getting COVID-19. Changing the way people work requires innovative approaches and questioning some long-held medical practices. There are multiple factors contributing to the apparent reduced utilization of healthcare services to non-COVID-19 patients. The current world crisis is highlighting the need of re-engineering the way we deliver inpatient and outpatient health care, including bolstering social support, in order to be prepared when the next calamity will present itself.

Alterio, D., et al. (2020). "Head and neck cancer radiotherapy amid COVID-19 pandemic: Report from Milan, Italy." Head Neck **42**(7): 1482-1490.

BACKGROUND: Management of head and neck cancers (HNC) in radiation oncology in the coronavirus disease 2019 (COVID-19) era is challenging. Aim of our work is to report organization strategies at a radiation therapy (RT) department in the first European area experiencing the COVID-19 pandemic. METHODS: We focused on (a) dedicated procedures for HNC, (b) RT scheduling, and (c) health care professionals' protection applied during the COVID-19 breakdown (from March 1, 2020 to April 30, 2020). RESULTS: Applied procedures are reported and discussed. Forty-three patients were treated. Image-guided, intensity modulated RT was performed in all cases. Median overall treatment time was 50 (interquartile range: 47-54.25) days. RT was interrupted/delayed in seven patients (16%) for suspected COVID-19 infection. Two health professionals managing HNC patients were proven as COVID-19 positive. CONCLUSION: Adequate and well-timed organization allowed for the optimization of HNC patients balancing at the best of our possibilities patients' care and personnel's safety.

Althwanay, A., et al. (2020). "Medical Education, Pre- and Post-Pandemic Era: A Review Article." Cureus **12**(10): e10775.

A pandemic is the worldwide outbreak and spread of a disease. Although pandemics of influenza have occurred rarely, approximately once every few decades in more than three centuries, the outbreaks of H1N1 and H5N1 influenza, the severe acute respiratory syndrome (SARS), and most recently, the novel coronavirus disease (COVID-19) caused by severe acute respiratory syndrome coronavirus 2 (SARS-CoV-2), have necessitated the institution of protective and preventive measures such as school closure and mandatory quarantine of infected people, as social distancing is considered to be the most effective preventative strategy until the development of a vaccine, treatment, or both. The current pandemic has also resulted in a transformation in medical education for both undergraduate and postgraduate medical students. Clinical rotations for undergraduates have been suspended all over the world; inter-hospital residency rotations and combined teaching sessions have also been curtailed until further notice. During this most recent pandemic, a number of medical schools have immediately converted their whole clinical curriculum into online formats. Similarly, educational and clinical assessments have been converted into online assessments. However, as the pandemic eras tend to recur over time and epidemics will continue to break out, medical students and healthcare workers will remain susceptible to contagion. Hence, we need to adopt a new educational system that would be safe and sustainable in the long run.

Altunisik Toplu, S., et al. (2020). "Relationship between hand hygiene and cutaneous findings during COVID-19 pandemic." J Cosmet Dermatol **19**(10): 2468-2473.

BACKGROUND: In the current situation of the COVID-19 pandemic, healthcare workers (HCWs) have to comply with hygiene conditions and use gloves more frequently and for a longer period of time than they would previously to avoid infection and prevent transmission. AIMS: We aimed to characterize the adverse skin reactions occurring after hand hygiene and glove use in HCWs in a tertiary university hospital to determine the possible causative factors and whether the use of these measures is affected. METHODS: Between April 15 and May 1, 2020, a cross-sectional survey was conducted, using online questionnaire, answered by HCWs in a tertiary university hospital. RESULTS: The increase in general hand-skin problems during the pandemic period was statistically significant (P = .004). The most common symptom was dryness. During the pandemic period, 67 (24.3%) HCWs thought that the conditions were caused by glove use, and 197 (71.4%) thought that they were due to alcohol-based hand antiseptics. The incidence of other hand-skin conditions except for vesicles was statistically higher in women than in men (P < .001). CONCLUSIONS: Increased number of hand-skin conditions during the pandemic should not be ignored, since hand hygiene and glove use are expected to increase.

Ambrosch, A., et al. (2020). "Effect of a strict hygiene bundle for the prevention of nosocomial transmission of SARS-CoV-2 in the hospital: a practical approach from the field." J Infect Public Health **13**(12): 1862-1867.

BACKGROUND: During the novel coronavirus disease (COVID-19) pandemic it is crucial for hospitals to implement infection prevention strategies to reduce nosocomial transmission to the lowest possible number. This is all the more important because molecular tests for identifying SARS-CoV-2 infected patients are uncertain, and the resources available for them are limited. In this view, a monocentric, retrospective study with an interventional character was conducted to investigate the extent to which the introduction of a strict hygiene bundle including a general mask requirement and daily screening for suspicious patients has an impact on the SARS-CoV-2 nosocomial rate in the pandemic environment. METHODS: All inpatients from a maximum care hospital in Regensburg (Bavaria) between March 1st and June 10th 2020 were included. Patient with respiratory symptoms were tested for SARS-CoV-2 at admission, patients were managed according to a standard hygiene protocol. At the end of March a strict hygiene bundle was introduced including a general mask obligation and a daily clinical screening of inpatients for respiratory symptoms. Nosocomial infection rate for COVID-19 and the risk for infection transmission estimated by the nosocomial incidence density before and after introduction the hygiene bundle were compared. The infection pressure for the hospital during the entire observational period was characterized by the infection reports in the region in relation to the number of hospitalized COVID-19 patients and the number of infected employees. RESULTS: In fact, after the introduction of a strict hygiene bundle including a general mouth and nose protection obligation and a daily clinical screening of suspicious patients, a significant reduction of the nosocomial rate from 0.28 to 0.06 (p = 0.026) was observed. Furthermore, the risk of spreading hospital-acquired infections also decreased dramatically from 0.0007 to 0.00018 (p = 0.031; rate ratio after/before 0.25 (95%CI 0.06, 1.07) despite a slow decrease of the hospital COVID 19-prevalence and an increase of infected employees. CONCLUSION: The available data underline that a strict hygiene bundle seem to be associated with a decrease of nosocomial SARS-CoV-2 transmission in the pandemic situation.

Amimo, F., et al. (2021). "A review of prospective pathways and impacts of COVID-19 on the accessibility, safety, quality, and affordability of essential medicines and vaccines for universal health coverage in Africa." Global Health **17**(1): 42.

BACKGROUND: The ongoing pandemic of coronavirus disease 2019 (COVID-19) has the potential to reverse progress towards global targets. This study examines the risks that the COVID-19 pandemic poses to equitable access to essential medicines and vaccines (EMV) for universal health coverage in Africa. METHODS: We searched medical databases and grey literature up to 2 October 2020 for studies reporting data on prospective pathways and innovative strategies relevant for the assessment and management of the emerging risks in accessibility, safety, quality, and affordability of EMV in the context of the COVID-19 pandemic. We used the resulting pool of evidence to support our analysis and to draw policy recommendations to mitigate the emerging risks and improve preparedness for future crises. RESULTS: Of the 310 records screened, 134 were included in the analysis. We found that the disruption of the international system affects more immediately the capability of low- and middle-income countries to acquire the basket of EMV. The COVID-19 pandemic may facilitate dishonesty and fraud, increasing the propensity of patients to take substandard and falsified drugs. Strategic regional cooperation in the form of joint tenders and contract awarding, joint price negotiation and supplier selection, as well as joint market research, monitoring, and evaluation could improve the supply, affordability, quality, and safety of EMV. Sustainable health financing along with international technology transfer and substantial investment in research and development are needed to minimize the vulnerability of African countries arising from their dependence on imported EMV. To ensure equitable access, community-based strategies such as mobile clinics as well as fees exemptions for vulnerable and under-served segments of society might need to be considered. Strategies such as task delegation and telephone triage could help reduce physician workload. This coupled with payments of risk allowance to frontline healthcare workers and health-literate healthcare organization might improve the appropriate use of EMV. CONCLUSIONS: Innovative and sustainable strategies informed by comparative risk assessment are increasingly needed to ensure that local economic, social, demographic, and epidemiological risks and potentials are accounted for in the national COVID-19 responses.

Amorim, M. R., et al. (2021). "Respiratory Viral Shedding in Healthcare Workers Reinfected with SARS-CoV-2, Brazil, 2020." Emerg Infect Dis **27**(6).

We documented 4 cases of severe acute respiratory syndrome coronavirus 2 reinfection by non-variant of concern strains among healthcare workers in Campinas, Brazil. We isolated infectious particles from nasopharyngeal secretions during both infection episodes. Improved and continued protection measures are necessary to mitigate the risk for reinfection among healthcare workers.

Ananth, L. and P. Hosamani (2020). "Management of ENT Surgical Emergencies Amidst COVID-19 Lockdown: Our Experience in a Tertiary Referral Hospital." Indian J Otolaryngol Head Neck Surg: 1-8.

To describe our experience with ENT emergencies during lockdown due to COVID-19 pandemic and provide recommendations for triage, management and protective measures. Retrospective case series. Eleven patients requiring emergency ENT procedures in a tertiary referral hospital during the lockdown period of 24th March to 3rd May 2020 were identified. Clinical profiles, screening and operating room protocols along with the post-operative care and use of personal protective equipment are described. Nine patients were discharged from the hospital and two were in stable condition in the hospital. While lockdowns may be effective in controlling the transmission of COVID-19, they have a negative impact on the routine functioning of healthcare services. Appropriate protocols for screening, triage and management of non-COVID patients with due precautions and infection control strategies can ensure that emergencies get timely and appropriate attention while preventing spread of infection among patients and healthcare workers.

Anderegg, L., et al. (2020). "A scalable method of applying heat and humidity for decontamination of N95 respirators during the COVID-19 crisis." PLoS One **15**(7): e0234851.

A lack of N95 Filtering Facepiece Respirators (FFRs) during the COVID-19 crisis has placed healthcare workers at risk. It is important for any N95 reuse strategy to determine the effects that proposed protocols would have on the physical functioning of the mask, as well as the practical aspects of implementation. Here we propose and implement a method of heating N95 respirators with moisture (85°C, 60-85% humidity). We test both mask filtration efficiency and fit to validate this process. Our tests focus on the 3M 1860, 3M 1870, and 3M 8210 Plus N95 models. After five cycles of the heating procedure, all three respirators pass both quantitative fit testing (score of >100) and show no degradation of mask filtration efficiency. We also test the Chen Heng V9501 KN95 and HKYQ N95 finding no degradation of mask filtration efficiency, however even for unheated masks these scored <50 for every fit test. The heating method presented here is scalable from individual masks to over a thousand a day with a single industrial convection oven, making this method practical for local application inside health-care facilities.

Andresen, M., et al. (2020). "[Expanding ICU facilities in a pandemic scenario]." Rev Med Chil **148**(5): 674-683.

Our country is suffering the effects of the ongoing pandemic of coronavirus disease (COVID-19). Because the vulnerability of healthcare systems, especially the intensive care areas they can rapidly be overloaded. That challenge the ICUs simultaneously on multiple fronts making urgent to increase the number of beds, without lowering the standards of care. The purpose of this article is to discuss some aspects of the national situation and to provide recommendations on the organizational management of intensive care units such as isolation protocols, surge in ICU bed capacity, ensure adequate supplies, protect and train healthcare workers maintaining quality clinical management.

Anelli, F., et al. (2020). "Italian doctors call for protecting healthcare workers and boosting community surveillance during covid-19 outbreak." Bmj **368**: m1254.

Angamuthu, N., et al. (2021). "Transmission of infection among health care personnel performing surgical tracheostomies on COVID-19 patients." Surgeon.

BACKGROUND: Staff and patient safety are of paramount importance while performing a surgical tracheostomy (ST) during the corona virus disease (COVID-19) pandemic. The aim was to assess the incidence of COVID-19 infection among the healthcare personnel (HCP) performing ST on COVID-19 patients. METHODS: One hundred and twenty-two HCP participating in 71 ST procedures performed at our institution between 26th March 2020 and 27th May 2020 were identified. A COVID-19 health questionnaire was distributed among staff with their consent. Data related to the presence of COVID-19 symptoms (new onset continuous cough, fever, loss of taste and/or loss of smell) among HCP involved in ST as well as patient related data were collected. RESULTS: Of the HCP who responded, eleven (15%,11/72) reported key COVID-19 symptoms and went into self-isolation. Ten members from this group underwent a COVID-19 swab test and three tested positive. Only one HCP attended hospital for symptomatic treatment, none required hospitalisation. Sixty percent (43/72) of the responders had a COVID-19 antibody test with a positive rate of 18.6% (8/43). Among the patients undergoing a ST, 67% (37/55) required a direct intensive care unit (ICU) admission; the mean age was 58 years (29-78) with a male preponderance (65.5%). The median time from intubation to ST was 15 days (range 5-33,IQR = 9). The overall mortality was 11% (6/55). CONCLUSIONS: ST can be carried out safely with strict adherence to both, personnel protective equipment and ST protocols which are vital to mitigate the potential transmission of COVID-19 to the HCP.

Aodeng, S., et al. (2020). "Safety and efficacy of tracheotomy for critically ill patients with coronavirus disease 2019 (COVID-19) in Wuhan: a case series of 14 patients." Eur J Cardiothorac Surg **58**(4): 745-751.

OBJECTIVES: Coronavirus disease 2019 (COVID-19) is a global pandemic. Critically ill patients often require prolonged intubation for mechanical ventilation to support breathing; thus, the artificial airway must be managed by tracheotomy. Therefore, studies exploring appropriate and safe methods for tracheotomy that minimize the risks of nosocomial transmission are important. METHODS: A retrospective analysis of the clinical characteristics of 14 critically ill patients with COVID-19, who underwent bedside tracheotomy from March to April 2020 was conducted to summarize the indications for tracheotomy and key points related to personal protective equipment and surgical procedures. RESULTS: All 14 patients were diagnosed with COVID-19 and were critically ill. All tracheotomies were performed in the late phase of the infection course. The interval between the infection and tracheotomy was 33 days, and the median interval between intubation and tracheotomy was 25.5 days. The reverse transcription-polymerase chain reaction results of secretions from the operative incision and inside the tracheotomy tube were negative. Twelve patients improved after tracheotomy, with SpO2 levels maintained above 96%. One patient died of progressive respiratory failure; another patient died of uncontrolled septic shock. No medical staff who participated in the tracheotomy was infected. CONCLUSIONS: Tracheotomy in critically ill patients with COVID-19 who meet the indications for tracheotomy potentially represents a safer approach to manage the airway and help improve the treatment outcomes. A tracheotomy performed in the late phase of the disease has a relatively low risk of infection. Adherence to key steps in the tracheotomy procedure and donning adequate personal protection will help medical staff avoid infection.

Apuzzo, L., et al. (2020). "[SARS-CoV-2: recommendations on nursing care for dialyzed and transplanted patient]." G Ital Nefrol **37**(5).

Coronavirus disease 2019 is an infectious respiratory syndrome caused by the virus called SARS-CoV-2, belonging to the family of coronaviruses. The first ever cases were detected during the 2019-2020 pandemic. Coronaviruses can cause a common cold or more serious diseases such as Middle Eastern Respiratory Syndromes (MERS) and Severe Acute Respiratory Syndrome (SARS). They can cause respiratory, lung and gastrointestinal infections with a mild to severe course, sometimes causing the death of the infected person. This new strain has no previous identifiers and its epidemic potential is strongly associated with the absence of immune response/reactivity and immunological memory in the world population, which has never been in contact with this strain before. Most at risk are the elderly, people with pre-existing diseases and/or immunodepressed, dialyzed and transplanted patients, pregnant women, people with debilitating chronic diseases. They are advised to avoid contacts with other people, unless strictly necessary, and to stay away from crowded places, also observing scrupulously the recommendations of the Istituto Superiore di Sanità. In this article we detail the recommendations that must be followed by the nursing care staff when dealing with chronic kidney disease patients in dialysis or with kidney transplant patients. We delve into the procedures that are absolutely essential in this context: social distancing of at least one meter, use of PPI, proper dressing and undressing procedures, frequent hand washing and use of gloves, and finally the increase of dedicated and appropriately trained health personnel on ward.

Arabi, Y. M., et al. (2021). "How the COVID-19 pandemic will change the future of critical care." Intensive Care Med **47**(3): 282-291.

Coronavirus disease 19 (COVID-19) has posed unprecedented healthcare system challenges, some of which will lead to transformative change. It is obvious to healthcare workers and policymakers alike that an effective critical care surge response must be nested within the overall care delivery model. The COVID-19 pandemic has highlighted key elements of emergency preparedness. These include having national or regional strategic reserves of personal protective equipment, intensive care unit (ICU) devices, consumables and pharmaceuticals, as well as effective supply chains and efficient utilization protocols. ICUs must also be prepared to accommodate surges of patients and ICU staffing models should allow for fluctuations in demand. Pre-existing ICU triage and end-of-life care principles should be established, implemented and updated. Daily workflow processes should be restructured to include remote connection with multidisciplinary healthcare workers and frequent communication with relatives. The pandemic has also demonstrated the benefits of digital transformation and the value of remote monitoring technologies, such as wireless monitoring. Finally, the pandemic has highlighted the value of pre-existing epidemiological registries and agile randomized controlled platform trials in generating fast, reliable data. The COVID-19 pandemic is a reminder that besides our duty to care, we are committed to improve. By meeting these challenges today, we will be able to provide better care to future patients.

Aranaz Andrés, J. M., et al. (2020). "[Masks as personal protective equipment in the COVID-19 pandemic: How, when and which should be used]." J Healthc Qual Res **35**(4): 245-252.

BACKGROUND AND OBJECTIVE: In the COVID-19 pandemic, the demand of masks has been increased by health professionals and the general population. In this context, it is necessary to summarize the features and indications of the different types of masks. MATERIAL AND METHODS: To consult and to compile the different recommendations disseminated by prestigious institutions such as the World Health Organization, the European Center for Disease Prevention, the Center for Evidence-Based Medicine, or the Ministry of Health of the Government of Spain has been reviewed. RESULTS: The institutions consulted recommend reserving FFP respirators for healthcare workers, especially when carrying out aerosol-generating procedures (AGPs) (minimum FFP2 protection) and consider some reutilization systems during times of scarcity. The use of surgical masks is recommended to professionals who do not perform AGPs and to the symptomatic population but exist variations in its indications intended for the general healthy population. CONCLUSION: In the context of shortage of personal protective equipment due to the COVID-19 pandemic, a prioritization and rationalization of the use of each type of mask should be established according to the user and the activity performed.

Publisher: Abstract available from the publisher.

spa

Aranaz-Andrés, J. M., et al. (2021). "Activities and Perceived Risk of Transmission and Spread of SARS-CoV-2 among Specialists and Residents in a Third Level University Hospital in Spain." Int J Environ Res Public Health **18**(6).

This study aims to identify factors related with SARS-CoV-2 infection in physicians and internal residents during the SARS-CoV-2 pandemic at a tertiary hospital in Spain, through a cross- sectional descriptive perception study with analytical components through two questionnaires directed at professionals working at the Ramon y Cajal University Hospital between February and April 2020. In total, 167 professionals formed the study group, and 156 professionals comprised the comparison group. Seventy percent of the professionals perceived a shortage of personal protective equipment (PPE), while 40% perceived a shortage of hand sanitiser, although more than 70% said they used it properly. Soap was more available and had a higher percentage of correct use (73.6-79.5%) (p > 0.05). Hand hygiene was optimal in >70% of professionals according to all five WHO measurements. In the adjusted model (OR; CI95%), belonging to a high-risk specialty (4.45; 1.66-11.91) and the use of public transportation (3.27; 1.87-5.73) remained risk factors. Protective factors were changes of uniform (0.53; 0.32-0.90), sanitation of personal objects before the workday (0.55; 0.31-0.97), and the disinfection of shared material (0.34; 0.19-0.58). We cannot confirm that a shortage or misuse of PPE is a factor in the spread of SARS-CoV-2. Fears and assessments are similar in both groups, but we cannot causally relate them to the spread of infection. The perception of the area of risk is different in both groups, suggesting that more information and education for healthcare workers is needed.

Arefin, M. K. (2021). "Povidone Iodine (PVP-I) Oro-Nasal Spray: An Effective Shield for COVID-19 Protection for Health Care Worker (HCW), for all." Indian J Otolaryngol Head Neck Surg: 1-6.

SARS- CoV-2 or novel coronavirus enters in human body through nose and mouth, stays there for a while. Then binds with ACE2 receptor, enters inside cell, multiply there and manifests. Again, Polyvinyl Pyrrolidone or Povidone Iodine (PVP-I) is a strong microbicidal agent having 99.99% virucidal efficacy in its only 0.23% concentration, irrespective of all known viruses, even in SARS- CoV-2 (in vitro). An oro-nasal spray is designed to apply the PVP-I in nose and oral cavity to gain a protective layer or coating over nasal and oral mucosa, so that SARS-CoV-2 can't bind with the ACE-2 receptor and prevent their entry inside. So, it will be effective for prevention of COVID-19. Moreover, as PVP-I has the ability for destruction of SARS-CoV-2, transmission of SARS- CoV-2 from patient will be reduced also. Thus PVP-I oro-nasal spray can act as an effective shield for COVID-19 protection for healthcare workers, for all.

Arenas, M. D., et al. (2020). "Protection of nephrology health professionals during the COVID-19 pandemic." Nefrologia **40**(4): 395-402.

The COVID-19 epidemic represents a special risk for kidney patients due to their comorbidities and advanced age, and the need for hemodialysis treatment in group rooms. It also represents a risk for professionals responsible for their attention. This manuscript contains a proposal for action to prevent infection of professionals in the Nephrology Services, one of the most valuable assets at the present time.

eng

Arenliu, A., et al. (2020). "Building Online and Telephone Psychological First Aid Services in a Low Resource Setting During COVID-19: The Case of Kosovo." Psychiatr Danub **32**(3-4): 570-576.

The COVID-19 pandemic and the measures brought for prevention of infections are associated with considerable psychosocial and psychological morbidity in the general population. Providing continuous mental health services during the pandemics is a challenge worldwide, especially in low- and middle-income countries (LMICs). This paper reports on the strategies and activities taken to protect public mental health during the COVID-19 pandemic in Kosovo. This included establishing online and phone psychological first aid services and developing psychoeducational videos and webinars. The paper concludes with several lessons learned during the process of establishing and maintaining these interventions, such as: initial political endorsement is crucial to gain and maintain the momentum of services; continuous training of volunteers is crucial for both addressing the needs/problems, which arise during the process of work and maintain the motivation of volunteers; promotion of the services is crucial; academic curricula training mental health professionals should include digital mental health related courses and manualization on how to establish this kind of services in midst of crisis is crucial in order to ensure quick operationalization when needed again especially in LMIC settings. The interventions developed provide opportunity for further research especially by evaluating the impact of the services and exploring how online and provision of mental health and psychoeducation services online could help to cover services gap in times of isolation, limited movement and situations similar to pandemics in settings with limited mental health services and resources. The situation with pandemics with COVID 19 is still far from ending. Future waves of infections and restriction could again spike the mental health and psychosocial and psychological strain of the general population, therefore being prepared in provisions of psychological first aid and other mental health services online is crucial.

Arif, M. and M. Maaref Doost (2020). "Prevalence of COVID-19 Among Typical Ambulatory Care Patients in a District General Hospital in the United Kingdom." Cureus **12**(11): e11398.

Objectives This observational retrospective study was undertaken to ascertain the prevalence of coronavirus disease 2019 (COVID-19) among typical ambulatory care patients. In our hospital, ambulatory care unit (ACU) was supposed to be a COVID-19 free area, and, hence, as per the guidelines, even basic personal protection equipment (PPE) was not provided during the early phase of pandemic. Methods We identified 443 patients who presented to our ACU between March and June 2020 with chest pain or shortness of breath suspected of pulmonary embolism or acute coronary syndrome, which normally makes the bulk of referrals to ACU. As per protocol, patients with COVID-19-like symptoms, e.g., fever, cough, sore throat, and loss of taste and smell, were excluded from ACU. We then, reviewed computed tomography (CT) scans for radiological evidence of COVID-19, and lab data for COVID-19 polymerase chain reaction (PCR) or antibody tests, to find out if any of our patients turned out to be suffering from COVID-19 unexpectedly. Results We found 13 patients with radiological or serological evidence of COVID-19, which equates to a prevalence of 2.93% in this cohort of our ambulatory care patients. Four in our patient cohort showed radiological features that were highly suggestive of COVID-19 pneumonia; 47 chest CT scans were performed, which may suggest a prevalence of around 8.5% (4/47) on radiological ground if everyone was offered a CT scan. Conclusions Due to limited access to data, our result is likely an underestimation of the actual prevalence of COVID-19 among our ACU patients, highlighting the need to review the safety and PPE guidelines for the ambulatory clinic and any similar out-patient areas.

Arifi, A. A. and A. A. Alghamdi (2020). "Cardiac Surgery Services During the COVID-19 Outbreak: Surgeons' Thoughts." J Saudi Heart Assoc **32**(5): 16-19.

Cardiac surgeons during this pandemic crisis have a responsibility to ensure that essential elective cardiac operations are provided at their centers to the public, at the same time, they have to face administrative demands as well as the infection prevention guidelines and restrictions to protect themselves and their patients. Here, we describe the patient and procedures characteristics that we recommend to protect our patients and the healthcare workers.

Arora, P., et al. (2020). "Real-world assessment, relevance, and problems in use of personal protective equipment in clinical dermatology practice in a COVID referral tertiary hospital." J Cosmet Dermatol **19**(12): 3189-3198.

BACKGROUND: Doctors and healthcare workers (HCW) are at frontline in control of the pandemic caused by the novel coronavirus infection (COVID-19). The virus is transmitted by contact, droplet, and airborne transmission; hence, hand hygiene, social distancing, environmental disinfection, and use of appropriate personal protective equipment (PPE) form important components to protect HCWs from cross-infection. Appropriate use of PPE is of paramount importance not only to reduce the risk of transmission but also to maintain adequate stock for those who are dealing directly with COVID-19 patients. AIMS: In this article, we aim to provide the rationale for appropriate use of PPE in the dermatology setting in the current scenario. We have also discussed the scientific evidence for use of each component of protection and the practical problems faced in our COVID referral tertiary hospital. METHODS: Our review was based on articles that have studied or analyzed the efficacy of various protective measures being utilized by health workers against spread of COVID-19. This was done by carrying out a PUBMED search with terms "coronavirus, COVID-19, personal protective equipment (PPE), transmission, mask, face shields, goggles, gloves." We also scrutinized the various pragmatic issues being faced by doctors in our setup while using PPE. RESULTS: In order to maximize the appropriate use of PPE, the rationale for use needs to be understood and problems encountered in daily practice need to be addressed. CONCLUSION: Adherence to protective measures and use of PPE is of utmost importance for HCWs to prevent cross-infection in this pandemic. The use of PPE can limit transmission to a great extent, but appropriate use and avoiding misuse is equally important in the dermatology setting in order to avoid depletion of stock. It is also essential to consider various practical issues with use of PPE and device measures to avoid them so that breach in protocols can be prevented and spread of infection minimized.

Arora, S., et al. (2020). "Evaluation of Knowledge and Preparedness Among Indian Dentists During the Current COVID-19 Pandemic: A Cross-Sectional Study." J Multidiscip Healthc **13**: 841-854.

PURPOSE: The current scenario of the COVID-19 pandemic has forced dentists to prepare themselves by updating their knowledge and receive training to face the present and after effects of COVID-19. The present survey was thus conducted to assess the knowledge, risk perception, attitude, and preparedness of the dentists in India about COVID-19. MATERIALS AND METHODS: A cross-sectional online survey was carried out among registered dentists in India. A self-administered, anonymous, questionnaire comprising of 25 close-ended questions was circulated to gather the relevant information. A total of 765 dentists submitted a response, out of which 646 complete responses were included in the statistical analysis. Pearson's Chi-square test was used for inter-group statistical comparison. RESULTS: The majority (80.8%) of the dentists had a fair knowledge about the characteristics of COVID-19. Nearly 60.7% and 49.7% of the dentists had taken infection control training and special training for COVID-19, respectively. Nearly 50% of the dentists had the perception that COVID-19 is very dangerous. Only 41.8% of the dentists were willing to provide emergency services to patients. Most of the dentists had an opinion that there is a need to enhance personal protective measures. During the survey, it was found that varied technological advances are offered to dentists to ensure good clinical practice. They also concurred that there was a need to standardize the tools and measures available to carry out dental practices. CONCLUSION: In the present study, Indian dentists have presented satisfactory knowledge with adequate preparedness as the majority of them had a fair level of knowledge with significantly higher knowledge among female respondents and those with post-graduation studies. Dentists should make judicious utilization of all the precautionary measures to safely practice dentistry. As we are going through an evolutionary phase where new advances are expected to evolve, dentists will definitely emerge successfully out from the crisis of the COVID-19 pandemic.

Arosio, A. D., et al. (2021). "Performing otolaryngological outpatient consultation during the Covid-19 pandemic." Am J Otolaryngol **42**(2): 102873.

BACKGROUND: The Covid-19 pandemic has had a profound impact on the Otolaryngology outpatient clinical practice, which is at high risk of respiratory viral transmission due to the close contact between the examiner and the patient's airway secretions [1]. Moreover, most otolaryngological procedures, including oropharyngoscopy, generate droplets or aerosols from high viral shedding areas [1]. Thus, only non-deferrable consultations were performed in the outbreak's acute phase. Along with the re-opening of elective clinical services and the impending second wave of the outbreak, a reorganization is necessary to minimize the risk of nosocomial transmission [1]. METHODS: This video (Video 1) shows how to safely conduct an outpatient Otorhinolaryngological consultation, focusing on complete ear, nose and throat examination, according to evidences from the published literature and Otolaryngological societies guidelines [2,3]. RESULTS: After telephonic screening, patients reporting Covid-19 symptoms or closecontact with a Covid-19 case within the last 14 days are referred to telehealth services [1-3]. To avoid crowding, the patient is admitted alone, after body temperature control, except for underage or disabled people [1]. The waiting room assessment must guarantee a social distance of 6 ft [1-3]. The consultation room is reorganized into two separate areas (Fig. 1): 1) a clean desk area, where an assistant wearing a surgical mask and gloves, handles the patient's documentation and writes the medical report, keeping proper distance from the patient, and 2) a separate consultation area, where the examiner, equipped with proper personal protective equipment (Fig. 2) [3,4], carries out the medical interview and physical examination. Endoscopic-assisted ear, nose and throat inspection using a dedicated monitor allows the examiner to maintain an adequate distance from the patient throughout the procedure while providing an optimal view (Figs. 3-6) [3]. Recent evidence shows that nasal endoscopy does not increase droplet production compared to traditional otolaryngological examination [5]. When necessary, nasal topic decongestion and anesthesia must be performed using cottonoids rather than sprays [3]. The patient keeps the nose and mouth covered throughout the consultation, lowering the surgical mask on the mouth for nasal endoscopy and removing it only for oropharyngoscopy. After the consultation, the doffing procedure must be carried out carefully to avoid contamination [4]. All the equipment and surfaces must undergo high-level disinfection with 70% alcohol or 0.1% bleach solutions [3]. Proper room ventilation must precede the next consultation [3]. CONCLUSIONS: The hints provided in this video are useful to ensure both patient and examiner safety during Otolaryngological outpatient consultations and to reduce SARS-CoV-2 transmission.

Aruru, M., et al. (2021). "Pharmacy Emergency Preparedness and Response (PEPR): a proposed framework for expanding pharmacy professionals' roles and contributions to emergency preparedness and response during the COVID-19 pandemic and beyond." Res Social Adm Pharm **17**(1): 1967-1977.

BACKGROUND: Pharmacists have long been involved in public health and emergency preparedness and response (EP&R), including through preventive measures such as screening, vaccinations, testing, medical and pharmaceutical countermeasures, as well as ensuring medication safety and access during natural disasters and pandemics. Pharmacy professionals are considered essential partners in response to the ongoing COVID-19 pandemic. Community and hospital pharmacies are expanding services and hours to provide essential services, putting pharmacists and their co-workers at the frontlines for patient care and safety to improve public health. In addition, pharmacy professionals are increasingly integrating into global, national, state and local EP&R efforts, including into interprofessional teams, such as Medical Reserve Corps (MRCs). However, lacunae exist for further integration of pharmacists into public health and safety initiatives. There are increasing opportunities and recommendations that should be expanded upon to provide improved patient care and population health interventions, and to ensure healthcare worker and public health safety. OBJECTIVE: Develop a Pharmacy Emergency Preparedness and Response (PEPR) Framework and recommendations for pharmacy professional pathways towards full integration within public health EP&R efforts (such as the COVID-19 pandemic), and enhanced recognition of pharmacists' skills, roles and contributions as integral members of the interprofessional healthcare team. METHODS: This paper draws on the American Society of Health-System Pharmacists (ASHP) 2003 Statement on the Role of Health-System Pharmacists in Emergency Preparedness and lessons learned from previous and current public health emergencies, such as the 2009 H1N1 pandemic and the current COVID-19 pandemic, to provide expanded guidance for pharmacists and pharmacy professionals across all practice settings in EP&R. The proposed PEPR framework also incorporates information and recommendations from The Pharmacy Organizations' Joint Policy Recommendations to Combat the COVID-19 Pandemic (March 2020), CDC-NIOSH, International Pharmaceutical Federation (FIP) Guidance, health departments and emergency preparedness guidance and resources, Boards of Pharmacy, and other pharmacy professional organizations and educational institutions. RESULTS: Based on methods and resources utilized in developing this proposed PEPR Framework, five key focus areas were identified, as follows:1)Emergency preparedness and response2)Operations management3)Patient care and population health interventions4)Public health pharmacy education and continuing professional education5)Evaluation, research, and dissemination for impact and outcomes. CONCLUSION: Pharmacists and pharmacy professionals have been at the frontlines in responding to the COVID-19 pandemic. Yet, challenges remain, such as limited availability of personal protection equipment, high risk of infectious exposures inherent in healthcare professions, and legislative hurdles resulting in lack of provider status and related reimbursements. Recommendations to enhance pharmacy's scope as public health professionals involved in EP&R include targeted training and education on key framework areas and policymaking. Pharmacy professionals should further integrate with interdisciplinary public health teams. Additional research and dissemination on impacts and outcomes of EP&R can enhance recognition of pharmacy professionals' contribution and value during public health emergencies. The proposed PEPR Framework can be utilized to develop, implement, evaluate, and disseminate results in order to strengthen existing efforts and to establish new initiatives in EP&R.

Ascott, A., et al. (2021). "Respiratory personal protective equipment for healthcare workers: impact of sex differences on respirator fit test results." Br J Anaesth **126**(1): e48-e49.

Asemahagn, M. A. (2020). "Factors determining the knowledge and prevention practice of healthcare workers towards COVID-19 in Amhara region, Ethiopia: a cross-sectional survey." Trop Med Health **48**: 72.

BACKGROUND: Healthcare workers (HWs) are at the highest risk of getting CIVID-19. This study aimed to assess factors determining the knowledge and prevention of HWs towards COVID-19 in the Amhara Region, Ethiopia. METHODS: A cross-sectional online survey was conducted among 442 HWs using email and telegram addresses. The knowledge and practice of HWs were estimated using 16 knowledge and 11 practice questions. A multivariable logistic regression analysis was used on SPSS version 25 to identify factors related to the knowledge and prevention practice of HWs on COVID-19. Significance was determined at a p value of < 0.05 and association was described by using odds ratio at 95% CI. RESULTS: Of 442 HWs, 398 (90% response rate) responded to the online interview questionnaire. From 398 HWs, 231(58%), 225(56%), 207(53%), and 191(48%) were males, from rural area, aged ≥ 34 years and nurses, respectively. About 279(70%) HWs had good knowledge of COVID-19 followed by 247(62%) good prevention practices. Age < 34 years (AOR = 2.14, 95% CI = 1.25-3.62), rural residence (AOR = 0.44, 95% CI = 0.26-0.70), access to infection prevention (IP) training (AOR = 2.4, 95% CI = 1.36-4.21), presence of IP guideline (AOR = 2.82, 95% CI = 1.64-4.62), and using social media (AOR = 2.51, 95% CI = 1.42-4.53) were factors of knowledge about COVID-19. Whereas, rural residence (AOR = 0.45, 95% CI = 0.31-0.75), facility type (AOR = 0.40, 95% CI = 0.28-0.89), access to IP training (AOR = 2.32, 95% CI = 1.35-4.16), presence of IP guidelines (AOR = 2.10, 95% CI = 1.21-3.45), knowledge about COVID-19 (AOR = 2.98, 95% CI = 2.15-5.27), having chronic illnesses (AOR = 2.0, 95% CI = 1.15-3.75), lack of protective equipment (PPE) (AOR = 0.42, 95% CI = 0.32-0.74), and high workload (AOR = 0.40, 95% CI = 0.36-0.87) were factors of COVID-19 prevention. CONCLUSION: In this study, most of the HWs had good knowledge but had lower prevention practice of COVID-19. Socio-demographic and access to information sources were factors of knowledge on COVID-19. Similarly, residence, shortage of PPE, high workload, comorbidities, knowledge, and access to IP training and guideline were factors limiting prevention practices. Thus, a consistent supply of PPE and improving health workers' knowledge, making IP guidelines and information sources available, and managing chronic illnesses are crucial to prevent COVID-19 among HWs.

Ashari, M. A., et al. (2020). "Strategies for radiology departments in handling the COVID-19 pandemic." Diagn Interv Radiol **26**(4): 296-300.

The world is facing an unprecedented global pandemic in the form of the coronavirus disease 2019 (COVID-19) which has ravaged all aspects of life, especially health systems. Radiology services, in particular, are under threat of being overwhelmed by the sheer number of patients affected, unless drastic efforts are taken to contain and mitigate the spread of the virus. Proactive measures, therefore, must be taken to ensure the continuation of diagnostic and interventional support to clinicians, while minimizing the risk of nosocomial transmission among staff and other patients. This article aims to highlight several strategies to improve preparedness, readiness and response towards this pandemic, specific to the radiology department.

Ashinyo, M. E., et al. (2021). "Infection prevention and control compliance among exposed healthcare workers in COVID-19 treatment centers in Ghana: A descriptive cross-sectional study." PLoS One **16**(3): e0248282.

Compliance with infection prevention and control (IPC) protocols is critical in minimizing the risk of coronavirus disease (COVID-19) infection among healthcare workers. However, data on IPC compliance among healthcare workers in COVID-19 treatment centers are unknown in Ghana. This study aims to assess IPC compliance among healthcare workers in Ghana's COVID-19 treatment centers. The study was a secondary analysis of data, which was initially collected to determine the level of risk of COVID-19 virus infection among healthcare workers in Ghana. Quantitative data were conveniently collected using the WHO COVID-19 risk assessment tool. We analyzed the data using descriptive statistics and logistic regression analyses. We observed that IPC compliance during healthcare interactions was 88.4% for hand hygiene and 90.6% for Personal Protective Equipment (PPE) usage; IPC compliance while performing aerosol-generating procedures (AGPs), was 97.5% for hand hygiene and 97.5% for PPE usage. For hand hygiene during healthcare interactions, lower compliance was seen among nonclinical staff [OR (odds ratio): 0.43; 95% CI (Confidence interval): 0.21-0.89], and healthcare workers with secondary level qualification (OR: 0.24; 95% CI: 0.08-0.71). Midwives (OR: 0.29; 95% CI: 0.09-0.93) and Pharmacists (OR: 0.15; 95% CI: 0.02-0.92) compliance with hand hygiene was significantly lower than registered nurses. For PPE usage during healthcare interactions, lower compliance was seen among healthcare workers who were separated/divorced/widowed (OR: 0.08; 95% CI: 0.01-0.43), those with secondary level qualifications (OR 0.08; 95% CI 0.01-0.43), non-clinical staff (OR 0.16 95% CI 0.07-0.35), cleaners (OR: 0.16; 95% CI: 0.05-0.52), pharmacists (OR: 0.07; 95% CI: 0.01-0.49) and among healthcare workers who reported of insufficiency of PPEs (OR: 0.33; 95% CI: 0.14-0.77). Generally, healthcare workers' infection prevention and control compliance were high, but this compliance differs across the different groups of health professionals in the treatment centers. Ensuring an adequate supply of IPC logistics coupled with behavior change interventions and paying particular attention to nonclinical staff is critical in minimizing the risk of COVID-19 transmission in the treatment centers.

Ashinyo, M. E., et al. (2020). "Healthcare Workers Exposure Risk Assessment: A Survey among Frontline Workers in Designated COVID-19 Treatment Centers in Ghana." J Prim Care Community Health **11**: 2150132720969483.

BACKGROUND: Healthcare workers (HCWs) are faced with an elevated risk of exposure to SARS-COV-2 due to the clinical procedures they perform on COVID-19 patients. However, data for frontline HCWs level of exposure and risk of COVID-19 virus infection are limited. OBJECTIVE: We investigated the level of exposure and risk of COVID-19 virus infection among HCWs in COVID-19 treatment centers in Ghana. METHODS: A cross-sectional study was utilized in this study and HCWs were invited by convenience to participate in the study, 408 HCWs in 4 COVID-19 treatment centers participated in the study. Adherence to infection prevention and control (IPC) measures were used to categorized HCWs as low or high risk of COVID-19 virus infection. The WHO COVID-19 risk assessment tool was used to collect quantitative data from the study participants. RESULTS: There was a high (N = 328, 80.4%) level of occupational exposure to the COVID-19 virus. However, only 14.0% of the exposed HCWs were at high risk of COVID-19 virus infection. Healthcare workers who performed or were present during any aerosol-generating procedures (AGP) were 23.8 times more likely to be exposed compared to HCWs who did not perform or were absent during any AGP (AOR 23.83; 95% CI: 18.45, 39.20). High risk of COVID-19 virus infection was less likely among registered nurses (AOR = 0.09; 95% CI: 0.02, 0.60), HCWs who performed or were present during any AGP (AOR = 0.05; 95% CI: 0.01, 0.50) and HCWs with a master's degree qualification (AOR 0.06; 95% CI: 0.01, 0.63). CONCLUSION: Despite the high level of exposure to the COVID-19 virus among HCWs in the treatment centers, only 14.0% were at high risk of COVID-19 virus infection. To protect this group of HCWs, treatment centers and HCWs should continue to adhere to WHO and national IPC protocols in managing of COVID-19 cases.

Ashokka, B., et al. (2020). "Care of the pregnant woman with coronavirus disease 2019 in labor and delivery: anesthesia, emergency cesarean delivery, differential diagnosis in the acutely ill parturient, care of the newborn, and protection of the healthcare personnel." Am J Obstet Gynecol **223**(1): 66-74.e63.

Coronavirus disease 2019, caused by the severe acute respiratory syndrome coronavirus 2, has been declared a pandemic by the World Health Organization. As the pandemic evolves rapidly, there are data emerging to suggest that pregnant women diagnosed as having coronavirus disease 2019 can have severe morbidities (up to 9%). This is in contrast to earlier data that showed good maternal and neonatal outcomes. Clinical manifestations of coronavirus disease 2019 include features of acute respiratory illnesses. Typical radiologic findings consists of patchy infiltrates on chest radiograph and ground glass opacities on computed tomography scan of the chest. Patients who are pregnant may present with atypical features such as the absence of fever as well as leukocytosis. Confirmation of coronavirus disease 2019 is by reverse transcriptase-polymerized chain reaction from upper airway swabs. When the reverse transcriptase-polymerized chain reaction test result is negative in suspect cases, chest imaging should be considered. A pregnant woman with coronavirus disease 2019 is at the greatest risk when she is in labor, especially if she is acutely ill. We present an algorithm of care for the acutely ill parturient and guidelines for the protection of the healthcare team who is caring for the patient. Key decisions are made based on the presence of maternal and/or fetal compromise, adequacy of maternal oxygenation (SpO(2) >93%) and stability of maternal blood pressure. Although vertical transmission is unlikely, there must be measures in place to prevent neonatal infections. Routine birth processes such as delayed cord clamping and skin-to-skin bonding between mother and newborn need to be revised. Considerations can be made to allow the use of screened donated breast milk from mothers who are free of coronavirus disease 2019. We present management strategies derived from best available evidence to provide guidance in caring for the high-risk and acutely ill parturient. These include protection of the healthcare workers caring for the coronavirus disease 2019 gravida, establishing a diagnosis in symptomatic cases, deciding between reverse transcriptase-polymerized chain reaction and chest imaging, and management of the unwell parturient.

Assadian, O., et al. (2021). "Surgical site infections: guidance for elective surgery during the SARS-CoV-2 pandemic - international recommendations and clinical experience." J Hosp Infect.

BACKGROUND: The COVID-19 pandemic not only had an impact on public life and healthcare facilities in general, but also affected established surgical workflows for elective procedures. The strategy to protect patients and healthcare workers from infection by SARS-CoV-2 in surgical departments has needed step-by-step development. Based on the evaluation of international recommendations and guidelines, as well as personal experiences in a clinical 'hot spot' and in a 450-bed surgical clinic, an adapted surgical site infection (SSI) prevention checklist was needed to develop concise instructions, which described roles and responsibilities of healthcare professionals that could be used for wider guidance in pandemic conditions. METHOD: Publications of COVID-19-related recommendations and guidelines, produced by health authorities and organizations, such as WHO, US-CDC, ECDC, the American College of Surgery and the Robert Koch Institute, were retrieved, assessed and referenced up to 31(st) January 2020. Additionally, clinical personal experiences in Germany were evaluated and considered. RESULTS: Part 1 of this guidance summarizes the experience of a tertiary care, surgical centre which utilized redundant hospital buildings for immediate spatial separation in a 'hot spot' COVID-19 area. Part 2 outlines the successful screening and isolation strategy in a surgical clinic in a region of Germany with outbreaks in surrounding medical centres. Part 3 provides the synopsis of personal experiences and international recommendations suggested for implementation during the COVID-19 pandemic. CONCLUSION: Understanding of COVID-19, and SARS-CoV-2-related epidemiology, is constantly and rapidly changing, requiring continuous adaptation and re-evaluation of recommendations. Established national and local guidelines for continuation of surgical services and prevention of SSI require ongoing scrutiny and focused implementation. This manuscript presents a core facility checklist to support medical institutions to continue their clinical and surgical work during the COVID-19 pandemic.

Ata, F., et al. (2020). "COVID-19 and Heart Failure: The Big Challenge." Heart Views **21**(3): 187-192.

Patients with chronic heart failure (HF) are among the most vulnerable populations in the COVID era. HF patients infected with COVID-19 are at a significant risk of severe illness and death. They usually present with shortness of breath and radiologic signs of an acute decompensation, which can mask the manifestations of COVID-19. Delay in the diagnosis increases the risk of individual poor outcomes and jeopardizes healthcare workers if protective and isolation measures are not established promptly. Furthermore, the COVID-19 pandemic is forcing health-care systems to modify the delivery of care to patients. Outpatient services are being done virtually, and elective procedures postponed. These may have an impact on the quality of life and survival of chronic HF patients. We present two cases of patients with the previous history of HF who developed an acute exacerbation secondary to COVID-19 infection. In this review, we focused on the main challenges physicians face when dealing with COVID-19 in chronic HF patients at the individual and system levels.

Ather, B., et al. (2021). Airborne Precautions. StatPearls. Treasure Island (FL), StatPearls Publishing

Copyright © 2021, StatPearls Publishing LLC.

An airborne disorder is any disease that is caused by a microorganism that is transmitted through the air. Many clinically important airborne diseases are caused by a variety of pathogens, including bacteria, viruses, and fungi. These organisms may be transmitted through sneezing, coughing, spraying of liquids, the spread of dust, talking, or any activity that results in the generation of aerosolized particles. It is important to be aware that airborne diseases, in general, do not include disorders caused by air pollution, poisons, smog, and dust. According to the World Health Organization, “Airborne transmission of infectious agents refers to the transmission of disease caused by the dissemination of droplet nuclei that remain infectious when suspended in air over long distance and time.” Airborne transmission can be characterized as obligate or preferential depending on whether it is only transmitted via droplet nuclei or if it has multiple other routes of transmission. The microorganisms transmitted by an airborne route may be spread via fine mist, dust, aerosols, or liquids. The aerosolized particles are generated from a source of infection, such as an infected patient or animal. In addition, aerosols may be generated from biological waste products that accumulate in garbage cans, caves, and dry arid containers. In aerosolization, the microorganisms that are less than 100 micrometers in size float in the air. These microorganisms, contained in droplets, are then dispersed via air currents to varying distances and can be inhaled by susceptible hosts. Although a majority of the particles will drop off within the vicinity, the infected aerosolized particles often remain suspended in the air and may even travel considerable distances. As the distance between the source and susceptible individuals increases, the rate of transmission decreases. Airborne transmission necessitates the use of available interventions in healthcare facilities to break the transmission of airborne particles from patient to patient and patient to healthcare workers. Airborne particles are considered highly infectious as they often remain suspended in the air and travel by air currents to different parts of the hospital, where there is a potential of them being inhaled by others. In some cases where there is inadequate ventilation, the airborne particle may remain in the hospital room for extended periods and may even be inhaled by a newly admitted patient. The control and prevention of airborne transmission of infections are not simple; it requires the control of airflow with the use of specially designed ventilation systems, the practice of antiseptic techniques, wearing personalized protective equipment (PPE), and performing basic infection prevention measures like hand washing. Airborne Organisms In almost all cases, airborne pathogens cause an inflammatory reaction of the upper airways affecting the nose, sinuses, throat, and lungs. The involvement of these structures may result in sinus congestion, sore throat, and lower respiratory tract symptoms. Any coughing or sneezing activity may then generate aerosolized particles leading to airborne transmission. Some of the common pathogens that may spread via airborne transmission are: Anthrax. Aspergillosis. Blastomycosis. Chickenpox. Adenovirus. Enteroviruses. Rotavirus. Influenza. Rhinovirus. Neisseria meningitidis. Streptococcus pneumoniae. Legionellosis. Measles. Mumps. Smallpox. Cryptococcosis. Tuberculosis. Bordetella pertussis. Severe acute respiratory syndrome (SARS). Middle East Respiratory Syndrome (MERS). Coronavirus Disease 2019 (COVID-19). This is a non-exhaustive list that only encompasses some of the common diseases that have been implicated in airborne transmission. A special note to be made is regarding COVID-19, the 21st-century pandemic which is thought to spread through airborne routes (among other routes). Active measures to prevent airborne transmission have been shown to curb its spread. Airborne diseases are not exclusive to humans and can also infect animals. A notable example is poultry that is often affected by an avian disorder (Newcastle disease), which is also transmitted via an airborne route. However, it is important to understand that exposure to an animal or a patient with an airborne disease does not automatically ensure disease transmission. The infection also depends on the host's immunity, the amount of exposure, and the duration of exposure to the infected patient.  Airborne Particles Generated from Medical Equipment Besides patients, several medical and surgical procedures may also generate aerosolized infectious particles. In most cases, these airborne particles are generated during the manipulation of the lung airways. These include: Manual ventilation with a bag and mask. Intubation. Open endotracheal suctioning. Bronchoscopy. Cardiopulmonary resuscitation. Sputum induction. Chest physiotherapy. Lung surgery. Nebulizer therapy and steam inhalation. Non-invasive positive pressure ventilation (BIPAP, CPAP). An autopsy of the lungs. Care must be taken while performing the above medical procedures on patients known to have diseases with high transmissibility through airborne routes.

Atici, S., et al. (2021). "Symptomatic recurrence of SARS-CoV-2 infection in healthcare workers recovered from COVID-19." J Infect Dev Ctries **15**(1): 69-72.

There is rising concern that patients who recover from COVID-19 may be at risk of recurrence. Increased rates of infection and recurrence in healthcare workers could cause the healthcare system collapse and a further worsening of the COVID-19 pandemic. Herein, we reported the clinically symptomatic recurrent COVID-19 cases in the two healthcare workers who treated and recovered from symptomatic and laboratory confirmed COVID-19. We discuss important questions in the COVID-19 pandemic waiting to be answered, such as the protection period of the acquired immunity, the severity of recurrence and how long after the first infection occurs. We aimed to emphasize that healthcare workers should continue to pay maximum attention to the measures without compromising.

Atkinson, P., et al. (2020). "Understanding the policy dynamics of COVID-19 in the UK: Early findings from interviews with policy makers and health care professionals." Soc Sci Med **266**: 113423.

The UK government response to COVID-19 has been heavily criticised. We report witnesses' perceptions of what has shaped UK policies and how these policies have been received by healthcare workers. Such studies are usually affected by hindsight. Here we deploy a novel prospective approach to capture real-time information. We are historians, social scientists and biomedical researchers who study how societies cope with infectious disease. In February 2020 we began regular semi-structured calls with prominent members of policy communities, and health care professionals, to elicit their roles in, and reactions to, the pandemic response. We report witnesses' perceptions that personal protective equipment (PPE) stocks were too small, early warnings have not led to sufficiently rapid policy decisions, and a lack of transparency is sapping public trust. Significant successes include research mobilisation. The early experiences and reactions of our witnesses suggest important issues for investigation, notably a perception of delay in decision making.

Augusto, J. B., et al. (2020). "Healthcare Workers Bioresource: Study outline and baseline characteristics of a prospective healthcare worker cohort to study immune protection and pathogenesis in COVID-19." Wellcome Open Res **5**: 179.

Background: Most biomedical research has focused on sampling COVID-19 patients presenting to hospital with advanced disease, with less focus on the asymptomatic or paucisymptomatic. We established a bioresource with serial sampling of health care workers (HCWs) designed to obtain samples before and during mainly mild disease, with follow-up sampling to evaluate the quality and duration of immune memory. Methods: We conducted a prospective study on HCWs from three hospital sites in London, initially at a single centre (recruited just prior to first peak community transmission in London), but then extended to multiple sites 3 weeks later (recruitment still ongoing, target n=1,000). Asymptomatic participants attending work complete a health questionnaire, and provide a nasal swab (for SARS-CoV-2 RNA by RT-PCR tests) and blood samples (mononuclear cells, serum, plasma, RNA and DNA are biobanked) at 16 weekly study visits, and at 6 and 12 months. Results: Preliminary baseline results for the first 731 HCWs (400 single-centre, 331 multicentre extension) are presented. Mean age was 38±11 years; 67% are female, 31% nurses, 20% doctors, and 19% work in intensive care units. COVID-19-associated risk factors were: 37% black, Asian or minority ethnicities; 18% smokers; 13% obesity; 11% asthma; 7% hypertension and 2% diabetes mellitus. At baseline, 41% reported symptoms in the preceding 2 weeks. Preliminary test results from the initial cohort (n=400) are available: PCR at baseline for SARS-CoV-2 was positive in 28 of 396 (7.1%, 95% CI 4.9-10.0%) and 15 of 385 (3.9%, 2.4-6.3%) had circulating IgG antibodies. Conclusions: This COVID-19 bioresource established just before the peak of infections in the UK will provide longitudinal assessments of incident infection and immune responses in HCWs through the natural time course of disease and convalescence. The samples and data from this bioresource are available to academic collaborators by application  https://covid-consortium.com/application-for-samples/.

Au-Yong, P. A., et al. (2021). "Perceptions of healthcare workers in high-risk areas of a Singapore hospital during COVID-19: a cross-sectional study." Singapore Med J.

INTRODUCTION: There is worldwide concern over the psycho-emotional impact of COVID-19 on healthcare workers (HCWs). This study aimed to elicit HCWs' perceptions of the adequacy of protective measures in high-risk clinical areas and the factors associated with these perceptions. METHODS: This was a cross-sectional study conducted in April 2020. An anonymous electronic survey was sent via email to operating theatre (OT) and intensive care unit (ICU) staff of Sengkang General Hospital, Singapore. RESULTS: Of the 358 eligible participants, 292 (81.6%) responded to the survey. 93.2% of the participants felt that precautionary measures at work were sufficient and 94.9% acknowledged that adequate training was provided. More than 60% of the participants opined that their chances of contracting COVID-19 were moderate to high. Female gender, nursing occupation and duration of service < 10 years were significantly associated with increased fear of contracting COVID-19, less control over occupational exposure and lower perceived need to care for COVID-19 patients. Having young children at home did not significantly affect these perceptions. The most important ICU precautions were availability of personal protective equipment outside the rooms of COVID-19 positive patients (95.3%) and having visitor restrictions (95.3%). The most important OT measures were having a dedicated OT for COVID-19 positive patients (91.2%) and having simulation as part of protocol familiarisation (91.7%). CONCLUSION: Overall, there was high confidence in the adequacy of COVID-19 protective measures to prevent healthcare transmission in Singapore. The pandemic had a lower degree of psycho-emotional impact on HCWs here as compared to other countries.

Awais, S. B., et al. (2021). "Paramedics in pandemics: protecting the mental wellness of those behind enemy lines." Br J Psychiatry **218**(2): 75-76.

Besides a global health crisis, the COVID-19 pandemic has potential to have a severe and long-lasting psychological impact on frontline healthcare workers such as paramedics. It is imperative to shed light on these mental health issues and employ interventions to protect the mental wellness of this vulnerable group of healthcare workers.

Awano, N., et al. (2020). "Anxiety, Depression, and Resilience of Healthcare Workers in Japan During the Coronavirus Disease 2019 Outbreak." Intern Med **59**(21): 2693-2699.

Objective Coronavirus disease 2019 (COVID-19) is spreading around the world. The aim of this study was to assess the degree of anxiety, depression, resilience, and other psychiatric symptoms among healthcare workers in Japan during the COVID-19 pandemic. Methods This survey involved medical healthcare workers at the Japanese Red Cross Medical Center (Tokyo, Japan) between April 22 and May 15, 2020. The degree of symptoms of anxiety, depression, and resilience was assessed using the Japanese versions of the 7-item Generalized Anxiety Disorder Scale (GAD-7), Center for Epidemiologic Studies Depression Scale (CES-D), and 10-item Connor-Davidson Resilience Scale. Furthermore, we added original questionnaires comprising three factors: (i) anxiety and fear of infection and death; (ii) isolation and unreasonable treatment; and (iii) motivation and escape behavior at work. Results In total, 848 healthcare workers participated in this survey: 104 doctors, 461 nurses, 184 other co-medical staff, and 99 office workers. Among all participants, 85 (10.0%) developed moderate-to-severe anxiety disorder, and 237 (27.9%) developed depression. Problems with anxiety and fear of infection and death, isolation and unreasonable treatment, and motivation and escape from work were higher in the depression group than in the non-depression group (total CES-D score ≥ 16 points). Being a nurse and high total GAD-7 scores were risk factors of depression. Older workers and those with higher resilience were less likely to develop depression than others. Conclusion During the COVID-19 epidemic, many healthcare workers suffered from psychiatric symptoms. Psychological support and interventions for protecting the mental health of them are needed.

Aygun, N., et al. (2020). "Endocrine Surgery during the COVID-19 Pandemic: Recommendations from the Turkish Association of Endocrine Surgery." Sisli Etfal Hastan Tip Bul **54**(2): 117-131.

The 2019 novel coronavirus disease (COVID-19) was initially seen in Wuhan, China, in December 2019. World Health Organization classified COVID-19 as a pandemic after its rapid spread worldwide in a few months. With the pandemic, all elective surgeries and non-emergency procedures have been postponed in our country, as in others. Most of the endocrine operations can be postponed for a certain period. However, it must be kept in mind that these patients also need surgical treatment, and the delay time should not cause a negative effect on the surgical outcome or disease process. It has recently been suggested that elective surgical interventions can be described as medically necessary, time-sensitive (MeNTS) procedures. Some guidelines have been published on proper and safe surgery for both the healthcare providers and the patients after the immediate onset of the COVID-19 pandemic. We should know that these guidelines and recommendations are not meant to constitute a position statement, the standard of care, or evidence-based/best practice. However, these are mostly the opinions of a selected group of surgeons. Generally, only life-threatening emergency operations should be performed in the stage where the epidemic exceeds the capacity of the hospitals (first stage), cancer and transplantation surgery should be initiated when the outbreak begins to be controlled (second stage), and surgery for elective cases should be performed in a controlled manner with suppression of the outbreak (third stage). In this rapidly developing pandemic period, the plans and recommendations to be made on this subject are based on expert opinions by considering factors, such as the course and biology of the disease, rather than being evidence-based. In the recent reports of many endocrine surgery associations and in various reviews, it has been stated that most of the cases can be postponed to the third stage of the epidemic. We aimed to evaluate the risk reduction strategies and recommendations that can help plan the surgery, prepare for surgery, protect both patients and healthcare workers during the operation and care for the patients in the postoperative period in endocrine surgery.

Ayub, M., et al. (2020). "Physicians' Attitudes Towards Treating Patients in the Context of COVID-19 Pandemic in Pakistan." Cureus **12**(9): e10331.

Background and objectives Severe acute respiratory syndrome coronavirus 2 (SARS-CoV-2) has been the cause of a worldwide outbreak of respiratory illness, which has been declared as coronavirus disease 2019 (COVID-19) pandemic by the World Health Organization (WHO). The outbreak has posed a huge challenge to countries around the world and has resulted in a global lockdown. The pandemic has especially overburdened the healthcare sector, resulting in a shortage of personnel and equipment. Along with many other manifestations, it has resulted in stress and anxiety for the physicians as well. Furthermore, many healthcare workers have been reluctant in treating COVID-19 patients. This study aimed to explore the concerns of physicians in the context of the COVID-19 pandemic and to evaluate the reasons for their reluctance to treat the patients. Methodology This descriptive cross-sectional study included 235 physicians from seven hospitals of Pakistan who were actively working amid the COVID-19 pandemic. Data were collected from March 1, 2020, to May 30, 2020, using a structured online questionnaire. Participants were approached via non-probability convenient sampling. Two hundred and eight respondents were included in the data analysis. SPSS Statistics version 23.0 (IBM Corp., Armonk, NY) was used for data entry and analysis. Results A striking 83.7% (n=174) of the respondents expressed their reluctance to treat patients with COVID-19. Concerns they raised included one or more of the following four reasons; lack of proper personal protective equipment (PPE), fear of self-infection, excessive workload, and fear of transmitting the infection to their family members. Of note, 92% (n=161) of the respondents reported a lack of PPE while 74.1% (n=129) reported fear of transmitting the infection to their family members as reasons for their reluctance. The vast majority of the participants reported the need for psychological training to treat the patients' anxiety (95.2%, n=198). Many participants were afraid that their own anxiety might be affecting the quality of care patients were receiving (67.3%, n=140). Hence, most of the participants reported that psychological counseling should be provided (93.3%, n=194). Participants with family members older than 60 years were found to be reluctant to treat patients due to the risk of transmitting the infection to them (69.7%, n=145, p=0.001). Therefore, a major proportion of the participants (96.2%, n=200) felt that the hospitals should provide a place for them to rest and temporarily isolate themselves to avoid coming into contact with their family members. Conclusions We conclude that a major proportion of physicians is reluctant to treat their patients due to multiple factors. The grave situation of the pandemic has taken a toll on their mental health, which could be affecting the quality of care that the patients receive. Their concerns should be addressed to not only provide them with support and improve their working environment but also to ensure that they are fully equipped to provide state-of-the-art care to the patients in these grave times.

Azar, A., et al. (2020). "Fractured aluminum nasopharyngeal swab during drive-through testing for COVID-19: radiographic detection of a retained foreign body." Skeletal Radiol **49**(11): 1873-1877.

The ongoing coronavirus disease 2019 (COVID-19) pandemic has increased the need for safe and efficient testing as a key containment strategy. Drive-through testing with nasopharyngeal swab has been implemented in many places in the USA as it allows for expeditious testing of large numbers of patients, limits healthcare workers' risk of exposure, and minimizes the use of personal protective equipment. We present a case where the aluminum shaft of the nasopharyngeal swab fractured during specimen collection at a drive-through testing facility and was suspected to have remained in the asymptomatic patient. Initial evaluation with a series of radiographs covering the skull base, neck, chest, and abdomen did not reveal the swab. On further clinical evaluation, the swab was found endoscopically, lodged between the left inferior turbinate and nasal floor, and was removed by an otorhinolaryngologist. Using a phantom model, we aimed to delineate an imaging technique to better visualize the aluminum shaft of the nasopharyngeal swab on radiographs to help in identification. A technique using lower tube voltage (kVp) with tight collimation centered at the nasal bones area produced the best visualization of the aluminum shaft of the swab. Recognition that aluminum foreign bodies may be difficult to visualize radiographically and  optimization of radiograph acquisition technique may help guide clinical management in unusual cases. Further evaluation with computed tomography or endoscopy should be considered in suspected cases where radiographs are negative.

Aziminia, N., et al. (2021). "Hospital trainees' worries, perceived sufficiency of information and reported psychological health during the COVID-19 pandemic." Acute Med **20**(1): 25-36.

INTRODUCTION: The COVID-19 pandemic has been unsurpassed in clinical severity or infectivity since the 1918 Spanish influenza pandemic and continues to impact the world. During the A/H1N1 pandemic, healthcare workers presented concerns regarding their own and their families' health, as well as high levels of psychological distress. We aim to assess hospital trainees' concerns, perceived sufficiency of information, behaviour and reported psychological health during the COVID-19 pandemic. DESIGN: Single 39-point questionnaire. SETTING: A large NHS foundation trust in London. PARTICIPANTS: 204 hospital trainee doctors. RESULTS: 204 trainees participated, of whom 91.7% (n=187) looked after COVID-19 patients. 91.6% (n=164) were worried about COVID-19; the most frequent concern was that of family and friends dying from COVID-19 (74.6%, n=126). 22.2% (n=36) reported being infected with COVID-19. 6.8% (n=11) of trainees considered avoiding going to work. Perceived sufficiency of information about COVID-19 was moderately high. 25.9% (n=42) reported social distancing at work compared with 94.4% (n=152) outside work. 98.2% (n=159) reported using PPE and 24.7% (n=40) were confident the provided PPE protected them. 41.9% (n=67) reported their psychological health had been adversely affected. 95.6% (n=153) supported provision of psychological support services and 62.5% (n=100) stated they would consider using them. CONCLUSIONS: A significant proportion of hospital trainees expressed worries about COVID-19, above all with regards to the wellbeing of their loved ones over their own. Confidence in sufficiency of provided information was high and in utilised infection control measures low. A larger proportion of trainees reported psychological as compared with physical health concerns, with a smaller proportion confirming having been infected with COVID-19 although most perceived their risk of infection as high. Seeking solutions to support hospital trainees in their duties and their wellbeing with their input would help to empower them and improve their health and morale while working during pandemics.

Baggiani, A., et al. (2020). "Preparedness and Response to the COVID-19 Emergency: Experience from the Teaching Hospital of Pisa, Italy." Int J Environ Res Public Health **17**(20).

In Italy, the coronavirus disease 2019 (COVID-19) emergency took hold in Lombardy and Veneto at the end of February 2020 and spread unevenly among the other regions in the following weeks. In Tuscany, the progressive increase of hospitalized COVID-19 patients required the set-up of a regional task force to prepare for and effectively respond to the emergency. In this case report, we aim to describe the key elements that have been identified and implemented in our center, a 1082-bed hospital located in the Pisa district, to rapidly respond to the COVID-19 outbreak in order to guarantee safety of patients and healthcare workers.

Bahans, C., et al. (2021). "Ethical considerations of the dynamics of clinical trials in an epidemic context: Studies on COVID-19." Ethics Med Public Health **16**: 100621.

The COVID-19 epidemic has led to the intense mobilization of all health professionals, including those involved in research. From the very beginning, research ethics committees (RECs) have been called upon and mobilized to carry out the scientific and ethical evaluations of research projects to achieve a sound analysis of their risk/benefit balance. The aim of this article is to present an ethical reflection on the challenges and consequences of the fast-track procedure for the evaluation of COVID-19 research projects in the context of a public health emergency. Indeed, a large number of protocols of reduced rigor were hastily prepared without collaboration between researchers and in the absence of national regulation. As a result, a number of ethical dilemmas have emerged concerning the opposing needs of pragmatism imposed by the emergency context and the ethical principles that should govern the conduct of research. Moreover, the dispersion of these individual projects, aggravated by excessive media coverage of specific treatments, has resulted in a weakened impact of the research in the epidemic context. This article provides suggestions for the ethical management of ongoing and upcoming research, giving RECs the opportunity to adapt their evaluations to avoid allowing the pragmatism of the emergency context to subvert the inviolability of the epistemological and ethical principles of research on humans. This reflection may strengthen the ethical basis for the formulation of their decisions.

Bahl, P., et al. (2020). "Airborne or droplet precautions for health workers treating COVID-19?" J Infect Dis.

Cases of COVID-19 have been reported in over 200 countries. Thousands of health workers have been infected and outbreaks have occurred in hospitals, aged care facilities and prisons. World Health Organization (WHO) has issued guidelines for contact and droplet precautions for Healthcare Workers (HCWs) caring for suspected COVID-19 patients, whilst the US Centre for Disease Control (CDC) has recommended airborne precautions. The 1 - 2 m (≈3 - 6 ft) rule of spatial separation is central to droplet precautions and assumes large droplets do not travel further than 2 m (≈6 ft). We aimed to review the evidence for horizontal distance travelled by droplets and the guidelines issued by the World Health Organization (WHO), US Center for Diseases Control (CDC) and European Centre for Disease Prevention and Control (ECDC) on respiratory protection for COVID-19. We found that the evidence base for current guidelines is sparse, and the available data do not support the 1 - 2 m (≈3 - 6 ft) rule of spatial separation. Of ten studies on horizontal droplet distance, eight showed droplets travel more than 2 m (≈6 ft), in some cases more than 8 meters (≈26 ft). Several studies of SARS-CoV-2 support aerosol transmission and one study documented virus at a distance of 4 meters (≈13 ft) from the patient. Moreover, evidence suggests infections cannot neatly be separated into the dichotomy of droplet versus airborne transmission routes. Available studies also show that SARS-CoV-2 can be detected in the air, 3 hours after aeroslisation. The weight of combined evidence supports airborne precautions for the occupational health and safety of health workers treating patients with COVID-19.

Bahrs, C., et al. (2021). "Prospective surveillance study in a 1,400-bed university hospital: COVID-19 exposure at home was the main risk factor for SARS-CoV-2 point seroprevalence among hospital staff." Transbound Emerg Dis.

The Co-HCW study is a prospective cohort study among hospital staff, including healthcare workers (HCWs) and administration staff, at the Jena University Hospital (JUH), Germany. The objectives of this study were to assess SARS-CoV-2 IgG seroprevalence, individual exposure risk factors and compliance of HCWs to wear personal protective equipment (PPE). After the first nosocomial COVID-19 outbreak at JUH, mandatory masking was implemented on 20th March 2020. We evaluated point seroprevalence using two IgG detecting immunoassays and issued a questionnaire to assess COVID-19 exposure, clinical symptoms and compliance to wear PPE. Antibody retesting was offered to participants with a divergent result of both immunoassays 5-10 weeks after the first test. Between 19th May and 19th June 2020, we analysed 660 participants [out of 3,228; 20.4%]. Among them, 212 participants (32.1%) had received a previous COVID-19 test. Four of them (1.9%) reported a positive test result. After recruitment, 18 participants (2.7%) had SARS-CoV-2 antibodies in at least one immunoassay. Overall, 21 participants (3.2%) had any evidence of a past or current SARS-CoV-2 infection. Among them, 13 (61.9%) were not aware of direct COVID-19 exposure and 9 (42.9%) did not report any clinical symptoms. COVID-19 exposure at home (adjusted OR (aOR) with 95% CI: 47.82 (5.49, 416.62)) was associated with SARS-CoV-2 seroprevalence. We observed no evidence for an association between seroprevalence and exposure at work (aOR 0.48 (0.13, 1.70)) or with COVID-19 risk area according to the working place (aOR for intermediate-risk vs. high-risk: 1.97 (0.42, 9.22), aOR for low-risk versus high-risk: 2.10 (0.40, 11.06); p = .655). Reported compliance of HCWs to wear PPE differed (p < .001) between working in high-risk (98.3%) and in intermediate-risk areas (69.8%). In conclusion, compared to administration staff, we observed no additional risk to acquire SARS-CoV-2 infections by patient care, probably due to high compliance to wear PPE.

Bajaj, J. S. and S. L. Solanki (2020). "Study of risk factors and psychological impact in physicians diagnosed with COVID-19: An online, postexposure, cross-sectional survey." J Anaesthesiol Clin Pharmacol **36**(3): 345-349.

BACKGROUND AND AIMS: Healthcare workers (HCW's) who have contracted the disease while working in the hospital may have a deep psychological impact in the form of depression and anxiety, along with the stress of passing the infection to their family members. This study attempts to highlight the risk factors and psychological impact on doctors who have tested positive for coronavirus disease-2019 (COVID-19). MATERIAL AND METHODS: This online cross-sectional survey-based study enrolled all level doctors of all specialities of in Mumbai, India, who had tested positive for novel coronavirus. The survey questionnaire was a 26-item self-administered anonymous set of questions in English language only. This validated questionnaire was distributed by a link via Google forms for 1 week. RESULTS: We received 51 completed responses. Most of them were (67%) postgraduate students. Forty-three (85%) respondents were working in a COVID-19 center. Twenty-three (45%) had a history of contact with these patients without proper personal protective equipment (PPE) with unknown COVID-19 status of the patient at the time of contact. Ten percent regretted their decision of becoming a doctor. Only half (51%) had family support during their illness and 45% relied on friends and colleagues in the hospital. Twenty-six percent mentioned having a low mood throughout the illness. A major portion of people (32%) having fear of severe illness and death. CONCLUSION: Medical doctors working in direct contact with confirmed cases of COVID-19 are at increased risk of contracting the infection and it has a major impact on physical and mental health of medical doctors.

Bajaj, N., et al. (2020). "Salivary detection of SARS-CoV-2 (COVID-19) and implications for oral health-care providers." Head Neck **42**(7): 1543-1547.

The coronavirus disease 2019 (COVID-19) pandemic has become a major public health crisis. The diagnostic and containment efforts for the disease have presented significant challenges for the global health-care community. In this brief report, we provide perspective on the potential use of salivary specimens for detection and serial monitoring of severe acute respiratory syndrome coronavirus 2 (SARS-CoV-2), based on current literature. Oral health-care providers are at an elevated risk of exposure to COVID-19 due to their proximity to nasopharynx of patients, and the practice involving the use of aerosol-generating equipment. Here, we summarize the general guidelines for oral health-care specialists for prevention of nosocomial transmission of COVID-19, and provide specific recommendations for clinical care management.

Baker, J. M., et al. (2020). "Quantification of occupational and community risk factors for SARS-CoV-2 seropositivity among healthcare workers in a large U.S. healthcare system." medRxiv.

BACKGROUND: Quantifying occupational risk factors for SARS-CoV-2 infection among healthcare workers can inform efforts to improve healthcare worker and patient safety and reduce transmission. This study aimed to quantify demographic, occupational, and community risk factors for SARS-CoV-2 seropositivity among healthcare workers in a large metropolitan healthcare system. METHODS: We analyzed data from a cross-sectional survey conducted from April through June of 2020 linking risk factors for occupational and community exposure to COVID-19 with SARS-CoV-2 seropositivity. A multivariable logistic regression model was fit to quantify risk factors for infection. Participants were employees and medical staff members who elected to participate in SARS-CoV-2 serology testing offered to all healthcare workers as part of a quality initiative, and who completed a survey on exposure to COVID-19 and use of personal protective equipment. Exposures of interest included known demographic risk factors for COVID-19, residential zip code incidence of COVID-19, occupational exposure to PCR test-positive healthcare workers or patients, and use of personal protective equipment. The primary outcome of interest was SARS-CoV-2 seropositivity. RESULTS: SARS-CoV-2 seropositivity was estimated to be 5.7% (95% CI: 5.2%-6.1%) among 10,275 healthcare workers. Community contact with a person known or suspected to have COVID-19 (aOR=1.9, 95% CI:1.4-2.5) and zip code level COVID-19 incidence (aOR: 1.4, 95% CI: 1.0-2.0) increased the odds of infection. Black individuals were at high risk (aOR=2.0, 95% CI:1.6-2.4). Overall, occupational risk factors accounted for 27% (95% CI: 25%-30%) of the risk among healthcare workers and included contact with a PCR test-positive healthcare worker (aOR=1.2, 95% CI:1.0-1.6). CONCLUSIONS: Community risk factors, including contact with a COVID-19 positive individual and residential COVID-19 incidence, are more strongly associated with SARS-CoV-2 seropositivity among healthcare workers than exposure in the workplace.

Baker, J. R., et al. (2020). "American Neurogastroenterology and Motility Society Task Force Recommendations for Resumption of Motility Laboratory Operations During the COVID-19 Pandemic." Am J Gastroenterol **115**(10): 1575-1583.

The American Neurogastroenterology and Motility Society Task Force recommends that gastrointestinal motility procedures should be performed in motility laboratories adhering to the strict recommendations and personal protective equipment (PPE) measures to protect patients, ancillary staff, and motility allied health professionals. When available and within constraints of institutional guidelines, it is preferable for patients scheduled for motility procedures to complete a coronavirus disease 2019 (COVID-19) test within 48 hours before their procedure, similar to the recommendations before endoscopy made by gastroenterology societies. COVID-19 test results must be documented before performing procedures. If procedures are to be performed without a COVID-19 test, full PPE use is recommended, along with all social distancing and infection control measures. Because patients with suspected motility disorders may require multiple procedures, sequential scheduling of procedures should be considered to minimize need for repeat COVID-19 testing. The strategies for and timing of procedure(s) should be adapted, taking into consideration local institutional standards, with the provision for screening without testing in low prevalence areas. If tested positive for COVID-19, subsequent negative testing may be required before scheduling a motility procedure (timing is variable). Specific recommendations for each motility procedure including triaging, indications, PPE use, and alternatives to motility procedures are detailed in the document. These recommendations may evolve as understanding of virus transmission and prevalence of COVID-19 infection in the community changes over the upcoming months.

Balachandar, V., et al. (2020). "COVID-19: emerging protective measures." Eur Rev Med Pharmacol Sci **24**(6): 3422-3425.

The COVID-19 (Coronavirus disease 2019) spreads primarily through droplets of saliva or discharge from the nose. COVID-19 is predominantly considered as an unavoidable pandemic, and scientists are very curious about how to provide the best protection to the public before a vaccine can be made available. There is an urge to manufacture a greater number of masks to prevent any aerosol with microbes. Hence, we aim to develop an efficient viral inactivation system by exploiting active compounds from naturally occurring medicinal plants and infusing them into nanofiber-based respiratory masks. Our strategy is to develop fibrous filtration with three-layered masks using the compounds from medicinal plants for viral deactivation. These masks will be beneficial not just to healthcare workers but common citizens as well. In the absence of vaccination, productive masks can be worn to prevent transmission of airborne pathogenic aerosols and control diseases.

Ballard, M., et al. (2020). "Prioritising the role of community health workers in the COVID-19 response." BMJ Glob Health **5**(6).

COVID-19 disproportionately affects the poor and vulnerable. Community health workers are poised to play a pivotal role in fighting the pandemic, especially in countries with less resilient health systems. Drawing from practitioner expertise across four WHO regions, this article outlines the targeted actions needed at different stages of the pandemic to achieve the following goals: (1) PROTECT healthcare workers, (2) INTERRUPT the virus, (3) MAINTAIN existing healthcare services while surging their capacity, and (4) SHIELD the most vulnerable from socioeconomic shocks. While decisive action must be taken now to blunt the impact of the pandemic in countries likely to be hit the hardest, many of the investments in the supply chain, compensation, dedicated supervision, continuous training and performance management necessary for rapid community response in a pandemic are the same as those required to achieve universal healthcare and prevent the next epidemic.

Ballestero, M. F. M., et al. (2020). "Pediatric neurosurgery during the COVID-19 pandemic: update and recommendations from the Brazilian Society of Pediatric Neurosurgery." Neurosurg Focus **49**(6): E2.

OBJECTIVES: Coronavirus disease (COVID-19) is a potentially severe respiratory illness that has threatened humanity globally. The pediatric neurosurgery practice differs from that of adults in that it treats children in various stages of physical and psychological development and contemplates diseases that do not exist in other areas. The aim of this study was to identify the level of knowledge and readiness of the healthcare providers, as well as to evaluate new preventive practices that have been introduced, psychological concerns, and the impact of the COVID-19 pandemic on pediatric neurosurgical units in Brazil. METHODS: Pediatric neurosurgeons were given an online questionnaire developed by the Brazilian Society of Pediatric Neurosurgery to evaluate the impact of the COVID-19 pandemic on their clinical practice. RESULTS: Of a cohort of 110 active members of the Brazilian Society of Pediatric Neurosurgery, 76 completed the survey (69%). Ninety-six percent were aware of the correct use of and indication for the types of personal protective equipment in clinical and surgical practices, but only 73.7% of them had unrestricted access to this equipment. Ninety-eight percent of participants agreed or strongly agreed that the pandemic had affected their pediatric neurosurgical practice. The COVID-19 pandemic interfered with outpatient care in 88% of the centers, it affected neurosurgical activity in 90.7%, and it led to the cancellation of elective neurosurgical procedures in 57.3%. Concerning the impact of COVID-19 on surgical activity, 9.2% of the centers had less than 25% of the clinical practice affected, 46.1% had 26%-50% of their activity reduced, 35.5% had a 51%-75% reduction, and 9.2% had more than 75% of their surgical work cancelled or postponed. Sixty-three percent affirmed that patients had been tested for COVID-19 before surgery. Regarding the impact of the COVID-19 pandemic on the mental health of those interviewed, 3.9% reported fear and anxiety with panic episodes, 7.9% had worsening of previous anxiety symptoms, 60.5% reported occasional fear, 10.5% had sadness and some depressive symptoms, and 2.6% reported depressive symptoms. CONCLUSIONS: The COVID-19 pandemic has posed unprecedented challenges to healthcare services worldwide, including neurosurgical units. Medical workers, pediatric neurosurgeons included, should be aware of safety measures and follow the recommendations of local healthcare organizations, preventing and controlling the disease. Attention should be given to the psychological burden of exposure to SARS-CoV-2 in healthcare workers, which carries a high risk of anxiety and depression.

Balmaks, R., et al. (2020). "A Simulation-Based Failure Mode Analysis of SARS-CoV-2 Infection Control and Prevention in Emergency Departments." Simul Healthc.

BACKGROUND: Severe acute respiratory syndrome coronavirus 2 [SARS-CoV-2, the causative agent of coronavirus disease 2019 (COVID-19)] outbreak has been declared a global pandemic by the World Health Organization. The COVID-19 pandemic has highlighted problems of sustainable infection prevention and control measures worldwide, particularly the emerging issues with an insufficient supply of personal protective equipment. The aim of this study was to provide an action plan for mitigation of occupational hazards and nosocomial spread of SARS-CoV-2 through a failure mode analysis based on observations during in situ simulations. METHODS: A multicenter, cross-sectional, observational, simulation-based study was performed in Latvia from March 2 to 26, 2020. This study was conducted at 7 hospitals affiliated with Riga Stradiņš University. The presentation of a COVID-19 patient was simulated with an in situ simulations, followed by a structured debrief. Healthcare Failure Modes and Effects Analysis is a tool for conducting a systematic, proactive analysis of a process in which harm may occur. We used Healthcare Failure Modes and Effects Analysis to analyze performance gaps and systemic issues. RESULTS: A total of 67 healthcare workers from 7 hospitals participated in the study (range = 4-17). A total of 32 observed failure modes were rated using a risk matrix. Twenty-seven failure modes (84.4%) were classified as either medium or high risk or were single-point weaknesses, hence evaluated for action type and action; 11 (40.7%) were related to organizational, 11 (40.7%) to individual, and 5 (18.5%) to environmental factors. CONCLUSIONS: Simulation-based failure mode analysis helped us identify the risks related to the preparedness of the healthcare workers and emergency departments for the COVID-19 pandemic in Latvia. We believe that this approach can be implemented to assess and maintain readiness for the outbreaks of emerging infectious diseases in the future.

Banerjee, D., et al. (2021). "Psychosocial Framework of Resilience: Navigating Needs and Adversities During the Pandemic, A Qualitative Exploration in the Indian Frontline Physicians." Front Psychol **12**: 622132.

INTRODUCTION: Frontline healthcare workers (HCW) have faced significant plight during the ongoing Coronavirus disease 2019 (COVID-19) pandemic. Studies have shown their vulnerabilities to depression, anxiety disorders, post-traumatic stress, and insomnia. In a developing country like India, with a rising caseload, resource limitations, and stigma, the adversities faced by the physicians are more significant. We attempted to hear their "voices" to understand their adversities and conceptualize their resilience framework. METHODS: A qualitative approach was used with a constructivist paradigm. After an initial pilot, a socio-demographically heterogeneous population of 172 physicians working in COVID-designated centers were purposively sampled from all over India. Following in-depth virtual interviews using a pre-formed semi-structured guide, the data was transcribed and translated verbatim. The interview was focused on their challenges, needs, and processes of coping and support. Charmaz's grounded theory was used for analysis supplemented by NVivo 10 software. RESULTS: Fear of infection, uncertainty, stigma, guilt, and social isolation emerged as the main challenges. Simultaneously, their "unmet needs" were flexible work policies, administrative measures for better medical protection, the sensitivity of media toward the image of HCW, effective risk communication for their health, and finally, social inclusion. Their resilience "framework" emerged as a process while navigating these adversities and consisted of three facets: forming a "resilient identity," managing the resilience, and working through the socio-occupational distress. The role of mental well-being, social network, peer support, problem negotiation, and self-care emerged as the key coping strategies. CONCLUSION: The study findings support the global call for better psychosocial health and quality of life of the frontline HCWs. Their "unheard voices" explored in the study can anchor subsequent resilience-enhancing interventions and policies. Guidelines focusing on the psychological wellbeing of frontline HCWs need to be grounded in their unmet needs and lived experiences.

Barallat, J., et al. (2020). "Seroprevalence of SARS-CoV-2 IgG specific antibodies among healthcare workers in the Northern Metropolitan Area of Barcelona, Spain, after the first pandemic wave." PLoS One **15**(12): e0244348.

BACKGROUND: The rapid spread of Severe Acute Respiratory Syndrome Coronavirus 2 (SARS-CoV-2) around the world has caused a global pandemic, infecting millions of individuals, with an unprecedented impact in health care systems worldwide. Healthcare workers are one of the risk groups that need to be well protected, due to their strategic role in patient management, presently and in prevention of healthcare needs for future outbreaks. Here, we present the results of the first SARS-CoV-2 seroprevalence study in the Northern Metropolitan Area of Barcelona, Spain. METHODS: IgG SARS-CoV-2 antibodies were analyzed in serum samples from 7563 healthcare workers of the Northern Metropolitan Area of Barcelona. Samples were collected after the first pandemic wave (from May 4th to May 22nd, 2020) and were analyzed by automated chemiluminescence assays. All samples were tested for IgG anti-S1/S2. Participant samples with negative or equivocal results but with analytical signals above the limit of detection and/or previously confirmed COVID-19 diagnosis were also tested for IgG anti-Nucleocapsid. RESULTS: A total of 779 of 7563 (10.3%) healthcare workers were positive for anti-SARS-CoV-2 IgG (specific for either S1/S2 or N antigens). No significant differences were observed between those working at primary care or at the reference hospital. Interestingly, among 341 participants with a confirmed COVID-19 diagnosis, 36 (10.55%) tested negative for SARS-CoV-2 IgG (both S1/S2 and recombinant N antigen). CONCLUSION: Seroprevalence of anti-SARS-CoV-2 IgG in the healthcare workers of the North Metropolitan Area of Barcelona was higher than in the general population in the same geographical area. Safety measures have to be stressed in order to protect these essential workers from future pandemic waves.

Barello, S., et al. (2020). "The psychosocial impact of flu influenza pandemics on healthcare workers and lessons learnt for the COVID-19 emergency: a rapid review." Int J Public Health **65**(7): 1205-1216.

OBJECTIVES: During a pandemic, healthcare workers (HCWs) are essential to the health system response. Based on our knowledge, little information is available regarding the psychosocial impact on HCWs or interventions for supporting them during pandemics. Therefore, the study aimed to assess available literature on perceived stress and psychological responses to influenza pandemics in HCWs and identify implications for healthcare practice and future research. METHODS: This is a rapid review of the literature. The review was conducted according to the Preferred Reporting Items for Systematic Review and Meta-Analysis. RESULTS: Across all the studies-both qualitative and quantitative-HCWs working during the epidemic reported frequent concerns regarding their own health and the fear of infecting their families, friends and colleagues. Moreover, social isolation, uncertainty, fears of stigmatization and reluctance to work or considering absenteeism were frequently reported. Moreover, many studies highlighted a high prevalence of high levels of stress, anxiety and depression symptoms, which could have long-term psychological implications in HCWs. CONCLUSIONS: This rapid review offers an overview of the major concerns regarding HCWs' psychosocial well-being and possible preventive strategies, which could be useful for the current COVID-19 outbreak and similar future pandemics. Studies suggested to invest on preventive psychological, social, family and physical support and to guaranteeing reasonable work conditions and others in order to protect HCWs from the long-lasting psychological effect of the COVID-19 pandemic.

Barlow, B., et al. (2020). "Minimizing pharmacotherapy-related healthcare worker exposure to SARS-CoV-2." Am J Health Syst Pharm **77**(18): 1529-1531.

Barnabas, R. V., et al. (2020). "Efficacy of hydroxychloroquine for post-exposure prophylaxis to prevent severe acute respiratory syndrome coronavirus 2 (SARS-CoV-2) infection among adults exposed to coronavirus disease (COVID-19): a structured summary of a study protocol for a randomised controlled trial." Trials **21**(1): 475.

OBJECTIVES: Primary Objective • To test the efficacy of Hydroxychloroquine (HCQ) (400 mg orally daily for 3 days then 200 mg orally daily for an additional 11 days, to complete 14 days) to prevent incident SARS-CoV-2 infection, compared to ascorbic acid among contacts of persons with SARS-CoV-2 infection Secondary objectives • To determine the safety and tolerability of HCQ as SARS-CoV-2 Post-exposure Prophylaxis (PEP) in adults • To test the efficacy of HCQ (400 mg orally daily for 3 days then 200 mg orally daily for an additional 11 days, to complete 14 days) to prevent incident SARS-CoV-2 infection 2 weeks after completing therapy, compared to ascorbic acid among contacts of persons with SARS-CoV-2 infection • To test the efficacy of HCQ to shorten the duration of SARS-CoV-2 shedding among those with SARS-CoV-2 infection in the HCQ PEP group • To test the efficacy of HCQ to prevent incident COVID-19 TRIAL DESIGN: This is a randomized, multi-center, placebo-equivalent (ascorbic acid) controlled, blinded study of HCQ PEP for the prevention of SARS-CoV-2 infection in adults exposed to the virus. PARTICIPANTS: This study will enroll up to 2000 asymptomatic adults 18 to 80 years of age (inclusive) at baseline who are close contacts of persons with polymerase chain reaction (PCR)-confirmed SARS-CoV-2 or clinically suspected COVID-19 and a pending SARS-CoV-2 PCR test. This multisite trial will be conducted at seven sites in Seattle (UW), Los Angeles (UCLA), New Orleans (Tulane), Baltimore (UMB), New York City (NYU), Syracuse (SUNY-Upstate), and Boston (BMC). Inclusion criteria Participants are eligible to be included in the study only if all of the following criteria apply: 1.Men or women 18 to 80 years of age inclusive, at the time of signing the informed consent2.Willing and able to provide informed consent3.Had a close contact of a person (index) with known PCR-confirmed SARS-CoV-2 infection or index who is currently being assessed for COVID-19 Close contact is defined as: a.Household contact (i.e., residing with the index case in the 14 days prior to index diagnosis or prolonged exposure within a residence/vehicle/enclosed space without maintaining social distance)b.Medical staff, first responders, or other care persons who cared for the index case without personal protection (mask and gloves)4.Less than 4 days since last exposure (close contact with a person with SARS-CoV-2 infection) to the index case5.Access to device and internet for Telehealth visits6.Not planning to take HCQ in addition to the study medication Exclusion criteria Participants are excluded from the study if any of the following criteria apply: 1.Known hypersensitivity to HCQ or other 4-aminoquinoline compounds2.Currently hospitalized3.Symptomatic with subjective fever, cough, or shortness of breath4.Current medications exclude concomitant use of HCQ5.Concomitant use of other anti-malarial treatment or chemoprophylaxis, including chloroquine, mefloquine, artemether, or lumefantrine.6.History of retinopathy of any etiology7.Psoriasis8.Porphyria9.Known bone marrow disorders with significant neutropenia (polymorphonuclear leukocytes <1500) or thrombocytopenia (<100 K)10.Concomitant use of digoxin, cyclosporin, cimetidine, amiodarone, or tamoxifen11.Known moderate or severe liver disease12.Known long QT syndrome13.Severe renal impairment14.Use of any investigational or non-registered drug or vaccine within 30 days preceding the first dose of the study drugs or planned use during the study period INTERVENTION AND COMPARATOR: Households will be randomized 1:1 (at the level of household), with close contact participants receiving one of the following therapies: •HCQ 400 mg orally daily for 3 days then 200 mg orally daily for an additional 11 days •Placebo-like control (ascorbic acid) 500 mg orally daily for 3 days then 250 mg orally daily for 11 days MAIN OUTCOMES: The primary outcome of the study is the incidence of SARS-CoV-2 infection through day 14 among participants who are SARS-CoV-2 negative at baseline by randomization group. RANDOMISATION: Participants will be randomized in a 1:1 ratio to HCQ or ascorbic acid at the level of the household (all eligible participants in 1 household will receive the same intervention). The randomization code and resulting allocation list will be generated and maintained by the Study Statistician. The list will be blocked and stratified by site and contact type (household versus healthcare worker). BLINDING (MASKING): This is a blinded study. HCQ and ascorbic acid will appear similar, and taste will be partially masked as HCQ can be bitter and ascorbic acid will be sour. The participants will be blinded to their randomization group once assigned. Study team members, apart from the Study Pharmacist and the unblinded statistical staff, will be blinded. Laboratory staff are blinded to the group allocation. NUMBERS TO BE RANDOMISED (SAMPLE SIZE): The sample size for the study is N=2 000 participants randomized 1:1 to either HCZ (n=1 000) and ascorbic acid (n=1 000). TRIAL STATUS: Protocol version: 1.2 05 April 2020 Recruitment is ongoing, started March 31 and anticipated end date is September 30, 2020. TRIAL REGISTRATION: ClinicalTrials.gov, Protocol Registry Number: NCT04328961 Date of registration: April 1, 2020, retrospectively registered FULL PROTOCOL: The full protocol is attached as an additional file, accessible from the Trials website (Additional file 1). In the interest in expediting dissemination of this material, the familiar formatting has been eliminated; this Letter serves as a summary of the key elements of the full protocol.

Barratt, R., et al. (2020). "Characteristics of personal protective equipment training programs in Australia and New Zealand hospitals: A survey." Infect Dis Health **25**(4): 253-261.

BACKGROUND: Using personal protective equipment (PPE) is one of several fundamental measures to prevent the transmission of infection and infectious diseases and is particularly pertinent in the current COVID-19 pandemic. Appropriate use of PPE by healthcare workers is, however, often suboptimal. Training and monitoring of PPE competency are essential components of an infection prevention and control program but there is a paucity of research and data on the content of such training programs across Australasia. This paper reports the results of a survey that characterised the nature of PPE training in Australian and New Zealand hospitals. METHODS: A population-based online survey was distributed to members of three major Australasian colleges representing infection prevention and control. RESULTS: Results indicate that, although training is frequently provided at orientation, many healthcare workers do not receive regular updates. Training programmes combine online and classroom sessions, but over a third do not include a practical component. The frequency of monitoring PPE competency is variable with one third of respondents indicating that no auditing occurs. PPE items used for high-level training are variable, with use of powered air purifying respirators (PAPRs) uncommon. CONCLUSION: The results of this study suggest that HCWs' confidence, competence and familiarity with PPE are a concern, which in the context of the current global COVID-19 pandemic is problematic. More research is needed into how PPE training programs could be better designed, to prepare HCWs for practice using PPE safely and confidently.

Barreiro, D., et al. (2020). "[Are health personnel well protected against SARS-CoV-2?]." Medicina (B Aires) **80**(5): 583-584.

Barrett, E. S., et al. (2020). "Prevalence of SARS-CoV-2 infection in previously undiagnosed health care workers at the onset of the U.S. COVID-19 epidemic." medRxiv.

IMPORTANCE: Healthcare workers are presumed to be at increased risk of severe acute respiratory syndrome coronavirus-2 (SARS-CoV-2) infection due to occupational exposure to infected patients. However, no epidemiological study has examined the prevalence of SARS-CoV-2 infection in a cohort of healthcare workers during the early phase of community transmission. OBJECTIVE: To determine the baseline prevalence of SARS-CoV-2 infection in a cohort of previously undiagnosed healthcare workers and a comparison group of non-healthcare workers. DESIGN: Prospective cohort study Setting: A large U.S. university and two affiliated university hospitals Participants: 546 health care workers and 283 non-health care workers with no known prior SARS-CoV-2 infection Exposure: Healthcare worker status and role Main outcome(s) and measure(s): SARS-CoV-2 infection status as determined by presence of SARS-CoV-2 RNA in oropharyngeal swabs. RESULTS: At baseline, 41 (5.0%) of participants tested positive for SARS-CoV-2 infection, of whom 14 (34.2%) reported symptoms. The prevalence of SARS-CoV-2 infection was higher among healthcare workers (7.3%) than in non-healthcare workers (0.4%), representing a 7.0% greater absolute risk (95% confidence interval for risk difference 4.7%, 9.3%). The majority of infected healthcare workers (62.5%) worked as nurses. Positive tests increased across the two weeks of cohort recruitment in line with rising confirmed cases in the hospitals and surrounding counties. CONCLUSIONS AND RELEVANCE: In a prospective cohort conducted in the early phases of community transmission, healthcare workers had a higher prevalence of SARS-CoV-2 infection than non-healthcare workers, attesting to the occupational hazards of caring for patients in this crisis. Baseline data reported here will enable us to monitor the spread of infection and examine risk factors for transmission among healthcare workers. These results will inform optimal strategies for protecting the healthcare workforce, their families, and their patients.

Barry, T. W. L., et al. (2020). "In the Extraordinary Times of Coronavirus Disease 2019: Clinical Strategies for Performing Spinal Surgery." Asian Spine J **14**(5): 721-729.

The coronavirus disease 2019 (COVID-19) pandemic has caused pronounced strain on global healthcare systems, forcing the streamlining of clinical activities and conservation of health resources. There is a pressing need for institutions to present discipline-specific strategies for the management of COVID-19 patients. We present the comprehensive considerations at the National University Hospital, Singapore from the surgeon's and anesthetist's perspectives in the performance of spinal surgery in COVID-19 patients. These are based on national guidelines and overarching principles of protection for the healthcare workers (HCWs) and efficiency in surgical planning. The workflow begins with the emergency department screening that has been adapted to the local epidemiology of COVID-19 in order to identify suspected/confirmed cases. If patient history cannot be obtained, demographic, clinical, and imaging data are used. Designated orthopedic "contaminated teams" are available 24/7 with an activation time of <30 minutes for review. In cases where sub-specialty spine surgeons were required, these professionals were inducted into the "contaminated team" and quarantined until cleared to return to work. Indications for emergency spine surgery were determined pre-emptively. Preoperative surgical considerations included the minimization of manpower, limited dissection, reduced operative time, and judicious use of equipment, leading to reduced aerosolization. Anesthesia considerations include preoperative screening for COVID-19-related concerns that influence surgery, operating room process planning and induction, intraoperative, reversal, recovery, and resuscitation considerations. Focused multi-disciplinary preoperative briefing facilitates familiarization. Surgical, anesthetic, and postoperative workflows were designed to reduce the risk of transmission and protect HCWs while effectively performing spinal surgery. The COVID-19 pandemic has necessitated paradigm shifts in healthcare planning, hospital workflows, and operative protocols. The viral burden does not discriminate between surgeons and physicians, and it is crucial that we, as medical professionals, adapt practices to be malleable and fluid to address the ever-changing developments.

Bartoszko, J. J., et al. (2020). "Medical masks vs N95 respirators for preventing COVID-19 in healthcare workers: A systematic review and meta-analysis of randomized trials." Influenza Other Respir Viruses **14**(4): 365-373.

BACKGROUND: Respiratory protective devices are critical in protecting against infection in healthcare workers at high risk of novel 2019 coronavirus disease (COVID-19); however, recommendations are conflicting and epidemiological data on their relative effectiveness against COVID-19 are limited. PURPOSE: To compare medical masks to N95 respirators in preventing laboratory-confirmed viral infection and respiratory illness including coronavirus specifically in healthcare workers. DATA SOURCES: MEDLINE, Embase, and CENTRAL from January 1, 2014, to March 9, 2020. Update of published search conducted from January 1, 1990, to December 9, 2014. STUDY SELECTION: Randomized controlled trials (RCTs) comparing the protective effect of medical masks to N95 respirators in healthcare workers. DATA EXTRACTION: Reviewer pair independently screened, extracted data, and assessed risk of bias and the certainty of the evidence. DATA SYNTHESIS: Four RCTs were meta-analyzed adjusting for clustering. Compared with N95 respirators; the use of medical masks did not increase laboratory-confirmed viral (including coronaviruses) respiratory infection (OR 1.06; 95% CI 0.90-1.25; I(2)  = 0%; low certainty in the evidence) or clinical respiratory illness (OR 1.49; 95% CI: 0.98-2.28; I(2)  = 78%; very low certainty in the evidence). Only one trial evaluated coronaviruses separately and found no difference between the two groups (P = .49). LIMITATIONS: Indirectness and imprecision of available evidence. CONCLUSIONS: Low certainty evidence suggests that medical masks and N95 respirators offer similar protection against viral respiratory infection including coronavirus in healthcare workers during non-aerosol-generating care. Preservation of N95 respirators for high-risk, aerosol-generating procedures in this pandemic should be considered when in short supply.

Barycka, K., et al. (2020). "Should emergency medical service stuff use respirators with filtered valve in COVID-19 pandemic?" Adv Respir Med **88**(6): 638-639.

Performing medical procedures with the use of personal protective equipment may reduce the efficiency of medical procedures performed, for example, as with the current use of respiratory protection devices, including N95 or surgical masks. Healthcare workers (HCWs) using N95 respirators or medical masks may experience discomfort associated with wearing a mask when performing medical procedures, in particular those associated with increased physical activity, causing increased respiratory effort.

Barycka, K., et al. (2020). "Risk of self-contamination among healthcare workers in the COVID-19 pandemic." Am J Emerg Med.

Bashir, S., et al. (2021). "Healthcare workers' knowledge and attitudes towards COVID-19 in Saudi Arabia." Eur Rev Med Pharmacol Sci **25**(2): 1060-1069.

OBJECTIVE: The world is facing a devastating challenge in the COVID-19 (coronavirus disease 19) outbreak.  Healthcare workers (HCWs) provide the first line of defense against any disease outbreak. Thus, the present study is designed to assess HCWs' attitudes towards, knowledge of, and awareness of COVID-19. MATERIALS AND METHODS: A cross-sectional observational study was conducted using an online survey on social media; the participants were 597 adults. The study was conducted from March to April 2020. The questionnaire was randomly administered on Arabic-language social media applications; the 26-item survey assessed knowledge levels, attitudes, and practices. RESULTS: The analysis includes 597 complete responses from HCWs; participants included physicians, nurses, medical students, and pharmacists. Most participants were females aged 18 to 25 years. Most participants agreed that the virus can spread via direct and indirect contact. Most participants indicated that they wash their hands; avoid touching their eyes, nose, or mouth; and avoid crowded places to protect themselves against infection. Most indicated that a bat was the original source of the virus. Most participating HCWs indicated that the symptoms of COVID-19 include fever, cough, and shortness of breath. Most of the participants also indicated that there is no specific treatment for patients infected with COVID-19. CONCLUSIONS: The findings of this work can support the design of effective measures to prevent and control COVID-19 infections during the pandemic. The results also highlight where improvement is needed to HCWs' knowledge of and attitudes towards COVID-19; the findings also highlight the best healthcare practices regarding this illness.

Bashirian, S., et al. (2020). "Factors associated with preventive behaviours of COVID-19 among hospital staff in Iran in 2020: an application of the Protection Motivation Theory." J Hosp Infect **105**(3): 430-433.

This study was conducted to predict the preventive behaviours of healthcare workers (HCWs) towards COVID-19 based on the Protection Motivation Theory (PMT). This cross-sectional and analytical study was conducted on 761 HCWs in Hamadan, Iran, using multi-stage random sampling. The preventive behaviours against COVID-19 among HCWs were assessed at a relatively desirable level. Based on the PMT, threat and coping appraisal were predictors of protection motivation to conduct COVID-19 preventive behaviours (P<0.001). The intention was also predictive of COVID-19 preventive behaviours (P<0.001). Consideration of personnel's self-efficacy and their knowledge regarding the effectiveness of protective behaviours in designing staff training programmes are recommended.

Basseal, J. M., et al. (2020). "COVID-19: Infection prevention and control guidance for all ultrasound practitioners." Australas J Ultrasound Med **23**(2): 90-95.

The severe acute respiratory syndrome coronavirus (SARS-CoV-2), an enveloped virus, is the causative agent of the disease known as COVID-19 (coronavirus disease-2019). Proper infection prevention and control measures and good hygiene practices are essential to prevent spread of COVID-19 and protect both patients and the healthcare worker. These guidelines are relevant to all ultrasound practitioners and provides guidance on cleaning and disinfection of ultrasound equipment, the environment and PPE (protective personal equipment) during the COVID-19 outbreak in the Australasian region.

Basso, C., et al. (2020). "Feasibility of postmortem examination in the era of COVID-19 pandemic: the experience of a Northeast Italy University Hospital." Virchows Arch **477**(3): 341-347.

With the continuous spreading of SARS-CoV-2 and increasing number of deaths worldwide, the need and appropriateness for autopsy in patients with COVID-19 became a matter of discussion. In fact, in the COVID-19 era protection of healthcare workers is a priority besides patient management. No evidence is currently available about the real risk related to the procedure as well as to the subsequent management of the samples. We herein describe the procedure that has been used to perform the first series of postmortem examinations in the COVID center of the Padua University Hospital, Padua, Italy, after the implementation of an ad hoc operating procedure, to minimize the risk of infection for pathologists and technicians. Provided that the procedure is performed in an adequate environment respecting strict biosafety rules, our data indicate that complete postmortem examination appears to be safe and will be highly informative providing useful insights into the complex disease pathogenesis.

Basso, T., et al. (2020). "Virus transmission during orthopedic surgery on patients with COVID-19 - a brief narrative review." Acta Orthop **91**(5): 534-537.

Background and purpose - COVID-19 is among the most impactful pandemics that the society has experienced. Orthopedic surgery involves procedures generating droplets and aerosols and there is concern amongst surgeons that otherwise rational precautionary principles are being set aside due to lack of scientific evidence and a shortage of personal protective equipment (PPE). This narrative review attempts to translate relevant knowledge into practical recommendations for healthcare workers involved in orthopedic surgery on patients with known or suspected COVID-19.Patients and methods - We unsystematically searched in PubMed, reference lists, and the WHO's web page for relevant publications concerning problems associated with the PPE used in perioperative practice when a patient is COVID-19 positive or suspected to be. A specific search for literature regarding COVID-19 was extended to include publications from the SARS epidemic in 2002/3.Results - Transmission of infectious viruses from patient to surgeon during surgery is possible, but does not appear to be a considerable problem in clinical practice. Seal-leakage is a problem with surgical masks. Due to the lack of studies and reports, the possibility of transmission of SARS-CoV-2 from patient to surgeon during droplet- and aerosol-generating procedures is unknown.Interpretation - Surgical masks should be used only in combination with a widely covering visor and when a respirator (N95, FFP2, P3) is not made available. Furthermore, basic measures to reduce shedding of droplets and aerosols during surgery and correct and consistent use of personal protective equipment is important.

Basso, T., et al. (2020). "Transmission of infection from non-isolated patients with COVID-19 to healthcare workers." J Hosp Infect **106**(4): 639-642.

Insufficiently protected healthcare workers (HCWs), defined as high-risk contacts of patients with coronavirus disease 2019 (COVID-19), are routinely quarantined. This study evaluated the transmission of infection from a symptomatic patient with COVID-19 to 60 HCWs exposed at ≤2 m for ≥15 min or during aerosol-generating procedures. Following ≥106 unique high-risk contacts, none of the HCWs tested positive for severe acute respiratory syndrome coronavirus-2 RNA or developed antibodies. The HCWs reported adherence to basic infection control procedures. These results are in accordance with other reports, and should reassure HCWs and further stimulate broader evaluation of the foundation for the current practice of home quarantining non-symptomatic HCWs.

Bastani, P. and M. A. Bahrami (2020). "COVID-19 Related Misinformation on Social Media: A Qualitative Study from Iran." J Med Internet Res.

BACKGROUND: Background: During outbreaks of diseases a great amount of health threatening misinformation is produced and released. In the web-2 era much of this misinformation is disseminated via social media where information could spread easily and quickly. Monitoring social media content provides crucial insights for health managers to manage the crisis. OBJECTIVE: Objective: Given the misinformation surrounding COVID-19 outbreak, this study was aimed to analyze contents of the most commonly used social networks in Iran that is among the affected countries. METHODS: Methods: A social media monitoring conducted through a qualitative design to analyze the discussions of social media users about the content related to COVID-19 transferred via Iranian medical faculty members` groups in Telegram and Whats App during Feb 20 to March 20, 2020 emphasizing the misinformation. Discourse analysis was applied and the written dialogues and discussions regarding misinformation about different aspects of the outbreak between medical faculty members all over the country were analyzed. RESULTS: Results: Cultural factors, demand pressure for information during the crisis, the easiness of information dissemination via social networks, marketing incentives and the poor legal supervision of online contents are the main reasons of misinformation dissemination. Disease statistics; treatments, vaccines and medicines; prevention and protection methods; dietary recommendations and disease transmission ways are the main subjective categories of releasing misinformation regarding novel coronavirus outbreak. Consequences of misinformation dissemination regarding disease include psychosocial; economic; health status; health system and ethical ones. Active and effective presence of health professionals and authorities on social media during the crisis and the improvement of public health literacy in the long term are the most recommended strategies for dealing with issues related to misinformation. CONCLUSIONS: Conclusion: This study contributes the management of COVID-19 outbreak trough providing applicable insights for health managers to manage public information in this challenging time.

Batistaki, C., et al. (2020). "Lessons learned from first case of Cesarean delivery in a COVID-19 positive parturient in Greek region." J Anaesthesiol Clin Pharmacol **36**(Suppl 1): S121-s124.

We report the successful anesthetic management of a 24-year-old patient, with an active COVID-19 viral infection, scheduled for elective Cesarean section at 40(th) week of pregnancy. This was the first case in Greek region, and we report and discuss the difficulties and safety issues regarding a COVID-19 positive patient during an elective cesarean delivery. Regional anesthesia with full protective equipment for health personnel involved, along with careful planning and adherence to guidelines achieved safe completion of the operation.

Battista, R. A., et al. (2021). "Personal Protective Equipment (PPE) in COVID 19 Pandemic: Related Symptoms and Adverse Reactions in Healthcare Workers and General Population." J Occup Environ Med **63**(2): e80-e85.

OBJECTIVES: To assess prevalence of Personal Protective Equipment (PPE)-related symptoms and adverse reactions during Coronavirus Disease 2019 pandemics. METHODS: We conducted an observational study among people exposed to various degree of infectious risk. Data were collected with a self-administered online questionnaire. RESULTS: The entire cohort complained about a wide range of adverse reactions: respiratory symptoms affected 80.3% of respondents, 68.5% referred pressure-related skin lesions, fewer manifested a dermatosis of different grade or ocular symptoms. Most of the affected individuals belonged to healthcare staff and manifestations were predicted by wearing time (more than 6 h/d). Moreover, symptoms were higher in the healthcare staff wearing N95/FFP2 respirator mask. CONCLUSIONS: Given the crucial role of PPE to contain the pandemic infection, more attention has to be paid to exposed categories, establishing preventive measure of side effects to ensure total safety.

Bays, D. J., et al. (2020). "Investigation of Nosocomial SARS-CoV-2 Transmission from Two Patients to Health Care Workers Identifies Close Contact but not Airborne Transmission Events." Infect Control Hosp Epidemiol: 1-22.

OBJECTIVE: To describe the pattern of transmission of SARS-CoV-2 during 2 nosocomial outbreaks of COVID-19 with regard to the possibility of airborne transmission. DESIGN: Contact investigations with active case finding were used to assess the pattern of spread from 2 COVID-19 index patients. SETTING: A community hospital and university medical center in the United States, in February and March, 2020, early in the COVID-19 pandemic. PATIENTS: Two index patients and 421 exposed health care workers. METHODS: Exposed staff were identified by analyzing the EMR and conducting active case finding in combination with structured interviews. Staff were tested for COVID-19 by obtaining oropharyngeal/nasopharyngeal specimens, with RT-PCR testing to detect SARS-CoV-2. RESULTS: Two separate index patients were admitted in February and March 2020, without initial suspicion for COVID-19 and without contact or droplet precautions in place; both patients underwent several aerosol generating procedures in this context. A total of 421 health care workers were exposed in total, and the results of the case contact investigations identified 8 secondary infections in health care workers. In all 8 cases, the staff had close contact with the index patients without sufficient personal protective equipment. Importantly, despite multiple aerosol generating procedures, there was no evidence of airborne transmission. CONCLUSION: These observations suggest that, at least in a healthcare setting, a majority of SARS-CoV-2 transmission is likely to take place during close contact with infected patients through respiratory droplets, rather than by long-distance airborne transmission.

Behera, P., et al. (2021). "Role of ivermectin in the prevention of SARS-CoV-2 infection among healthcare workers in India: A matched case-control study." PLoS One **16**(2): e0247163.

BACKGROUND: Ivermectin is one among several potential drugs explored for its therapeutic and preventive role in SARS-CoV-2 infection. The study was aimed to explore the association between ivermectin prophylaxis and the development of SARS-CoV-2 infection among healthcare workers. METHODS: A hospital-based matched case-control study was conducted among healthcare workers of AIIMS Bhubaneswar, India, from September to October 2020. Profession, gender, age and date of diagnosis were matched for 186 case-control pairs. Cases and controls were healthcare workers who tested positive and negative, respectively, for COVID-19 by RT-PCR. Exposure was defined as the intake of ivermectin and/or hydroxychloroquine and/or vitamin-C and/or other prophylaxis for COVID-19. Data collection and entry was done in Epicollect5, and analysis was performed using STATA version 13. Conditional logistic regression models were used to describe the associated factors for SARS-CoV-2 infection. RESULTS: Ivermectin prophylaxis was taken by 76 controls and 41 cases. Two-dose ivermectin prophylaxis (AOR 0.27, 95% CI, 0.15-0.51) was associated with a 73% reduction of SARS-CoV-2 infection among healthcare workers for the following month. Those involved in physical activity (AOR 3.06 95% CI, 1.18-7.93) for more than an hour/day were more likely to contract SARS-CoV-2 infection. Type of household, COVID duty, single-dose ivermectin prophylaxis, vitamin-C prophylaxis and hydroxychloroquine prophylaxis were not associated with SARS-CoV-2 infection. CONCLUSION: Two-dose ivermectin prophylaxis at a dose of 300 μg/kg with a gap of 72 hours was associated with a 73% reduction of SARS-CoV-2 infection among healthcare workers for the following month. Chemoprophylaxis has relevance in the containment of pandemic.

Bein, B., et al. (2020). "SARS-CoV-2/COVID-19: Evidence-Based Recommendations on Diagnosis and Therapy." Geburtshilfe Frauenheilkd **80**(5): 491-498.

COVID-19, a new viral disease affecting primarily the respiratory system and the lung, has caused a pandemic posing serious challenges to healthcare systems around the world. In about 20% of patients, severe symptoms occur after a mean incubation period of 5 - 6 days; 5% of patients need intensive care therapy. Mortality is about 1 - 2%. Protecting healthcare workers is of paramount importance in order to prevent hospital-acquired infections. Therefore, during all procedures associated with aerosol production, personal protective equipment consisting of a FFP2/FFP3 (N95) respiratory mask, gloves, safety glasses and a waterproof overall should be used. Therapy is based on established recommendations issued for patients with acute lung injury (ARDS). Lung protective ventilation, prone position, restrictive fluid management and adequate management of organ failure are the mainstays of therapy. In case of fulminant lung failure, veno-venous extracorporeal membrane oxygenation may be used as a rescue in experienced centres. New, experimental therapies are evolving with ever increasing frequency; currently, however, no evidence-based recommendation is possible. If off-label and compassionate use of these drugs is considered, an individual benefit-risk assessment is necessary, since serious side effects have been reported.

Bekker, L. G., et al. (2020). "Protecting healthcare workers: A critical part of the COVID-19 response." S Afr Med J **110**(12): 1154-1155.

Belingheri, M., et al. (2020). "Beyond the assistance: additional exposure situations to COVID-19 for healthcare workers." J Hosp Infect **105**(2): 353.

Ben Amar, W., et al. (2020). "Criminal medical liability in the context of Covid-19 pandemic." Tunis Med **98**(5): 334-342.

The COVID-19 infection causes to medical community many difficulties worldwide. In addition to its therapeutic problems, it can generate situations with high medico-legal risk to doctor who can see his criminal medical liability engaged. In fact, in Tunisia, this new infection imposes many specific legal obligations. Some of these obligations have recently been introduced, therefore still little or not known by doctors, despite the need for them to comply with. In this paper, we propose to analyse the circumstances of medical practice in Covid-19 pandemic period , which risk to engage the doctor's criminal medical liability, and to set out the sanctions incurred, in order to protect health professionals against the specific legal risk of this emerging disease.

Bentivegna, E., et al. (2020). "Impact of COVID-19 prevention measures on risk of health care-associated Clostridium difficile infection." Am J Infect Control.

Clostridium difficile is the most common pathogen between health care-associated infections and its incidence has increased during the last years. lack of enough evidence about effective hygiene interventions to prevent this disease. Due to the coronavirus disease 2019 (COVID‑19) pandemic, several strategies to reduce microorganism spread were adopted in hospital setting. The objective of this study was to establish whether such strategies can reduce health care associated C difficile infection (HA-CDI) incidence. We found that, during the pandemic (2020) HA-CDI incidence was significantly lower with respect to the previous years. This work demonstrates that maintaining this level of attention regarding control activities related to prevention of microorganism transmission significantly reduce HA-CDI and related expenses in terms of health costs and human lives.

Berlit, P., et al. (2020). ""Neurological manifestations of COVID-19" - guideline of the German society of neurology." Neurol Res Pract **2**: 51.

Infection with the new severe acute respiratory syndrome coronavirus 2 (SARS-CoV-2) leads to a previously unknown clinical picture, which is known as COVID-19 (COrona VIrus Disease-2019) and was first described in the Hubei region of China. The SARS-CoV-2 pandemic has implications for all areas of medicine. It directly and indirectly affects the care of neurological diseases. SARS-CoV-2 infection may be associated with an increased incidence of neurological manifestations such as encephalopathy and encephalomyelitis, ischemic stroke and intracerebral hemorrhage, anosmia and neuromuscular diseases. In October 2020, the German Society of Neurology (DGN, Deutsche Gesellschaft für Neurologie) published the first guideline on the neurological manifestations of the new infection. This S1 guideline provides guidance for the care of patients with SARS-CoV-2 infection regarding neurological manifestations, patients with neurological disease with and without SARS-CoV-2 infection, and for the protection of healthcare workers. This is an abbreviated version of the guideline issued by the German Neurological society and published in the Guideline repository of the AWMF (Working Group of Scientific Medical Societies; Arbeitsgemeinschaft wissenschaftlicher Medizinischer Fachgesellschaften).

Bertoli, S., et al. (2021). "Effects of wearing a FFP2 mask on indirect calorimetry measurements: A pilot study." Clin Nutr ESPEN **41**: 443-446.

BACKGROUND & AIMS: During the coronavirus disease 2019 (COVID-19) pandemic the use of Indirect calorimetry (IC) during nutritional rehabilitation program requires special precautions due to possible contagions for patients and health professionals. We evaluated in a sample of healthy subjects the agreement between oxygen consumption (VO(2) mL/min), carbon dioxide production (VCO(2) mL/min), respiratory quotient (RQ) and resting energy expenditure (REE kcal/24 h/day) measured by IC with and without a filtering facepiece mask. MATERIALS: 10 subjects with a mean (SD) age of 43 (10) years and a body mass index of 25.2 (5.8) kg/m(2) underwent indirect calorimetry both with and without a class 2 filtering facepiece mask (FFP2), in random order. The limits of agreement (LOA) and the concordance correlation coefficient (CCC) were used to evaluate the interchangeability of the measurement conditions. RESULTS: The LOA between REE measured with and without FFP2 (-111 to 189 kcal/day) were comparable to those for repeated IC tests without wearing masks and CCC (0.95) showed substantial agreement. CONCLUSIONS: We observed high agreement between REE measured by IC with and without FFP2 mask. These procedures are interchangeable in clinical practice.

Bhandari, P., et al. (2020). "Recovery of endoscopy services in the era of COVID-19: recommendations from an international Delphi consensus." Gut **69**(11): 1915-1924.

The COVID-19 pandemic has had a profound impact on provision of endoscopy services globally as staff and real estate were repurposed. As we begin to recover from the pandemic, a cohesive international approach is needed, and guidance on how to resume endoscopy services safely to avoid unintended harm from diagnostic delays. The aim of these guidelines is to provide consensus recommendations that clinicians can use to facilitate the swift and safe resumption of endoscopy services. An evidence-based literature review was carried out on the various strategies used globally to manage endoscopy during the COVID-19 pandemic and control infection. A modified Delphi process involving international endoscopy experts was used to agree on the consensus statements. A threshold of 80% agreement was used to establish consensus for each statement. 27 of 30 statements achieved consensus after two rounds of voting by 34 experts. The statements were categorised as pre-endoscopy, during endoscopy and postendoscopy addressing relevant areas of practice, such as screening, personal protective equipment, appropriate environments for endoscopy and infection control precautions, particularly in areas of high disease prevalence. Recommendations for testing of patients and for healthcare workers, appropriate locations of donning and doffing areas and social distancing measures before endoscopy are unique and not dealt with by any other guidelines. This international consensus using a modified Delphi method to produce a series of best practice recommendations to aid the safe resumption of endoscopy services globally in the era of COVID-19.

Bharatendu, C., et al. (2020). "Powered Air Purifying Respirator (PAPR) restores the N95 face mask induced cerebral hemodynamic alterations among Healthcare Workers during COVID-19 Outbreak." J Neurol Sci **417**: 117078.

BACKGROUND AND AIM: COVID-19 pandemic has resulted in an unprecedented increased usage of Personal protective equipment (PPE) by healthcare-workers. PPE usage causes headache in majority of users. We evaluated changes in cerebral hemodynamics among healthcare-workers using PPE. METHODS: Frontline healthcare-workers donning PPE at our tertiary center were included. Demographics, co-morbidities and blood-pressure were recorded. Transcranial Doppler (TCD) monitoring of middle cerebral artery was performed with 2-MHz probe. Mean flow velocity (MFV) and pulsatility index (PI) were recorded at baseline, after donning N95 respirator-mask, and after donning powered air-purifying respirator (PAPR), when indicated. End-tidal carbon-dioxide (ET-CO2) pressure was recorded for participants donning PAPR in addition to the N95 respirator-mask. RESULTS: A total of 154 healthcare-workers (mean age 29 ± 12 years, 67% women) were included. Migraine was the commonest co-morbidity in 38 (25%) individuals while 123 (80%) developed de-novo headache due to N95 mask. Donning of N95 respirator-mask resulted in significant increase in MFV (4.4 ± 10.4 cm/s, p < 0.001) and decrease in PI (0.13 ± 0.12; p < 0.001) while ET-CO2 increased by 3.1 ± 1.2 mmHg (p < 0.001). TCD monitoring in 24 (16%) participants donning PAPR and N95 respirator mask together showed normalization of PI, accompanied by normalization of ET-CO2 values within 5-min. Combined use of N95 respirator-mask and PAPR was more comfortable as compared to N95 respirator-mask alone. CONCLUSION: Use of N95 respirator-mask results in significant alterations in cerebral hemodynamics. However, these effects are mitigated by the use of additional PAPR. We recommend the use of PAPR together with the N95 mask for healthcare-workers doing longer duties in the hospital wards.

Bhaskar, S., et al. (2020). "Key Strategies for Clinical Management and Improvement of Healthcare Services for Cardiovascular Disease and Diabetes Patients in the Coronavirus (COVID-19) Settings: Recommendations From the REPROGRAM Consortium." Front Cardiovasc Med **7**: 112.

Patients with cardiovascular disease and diabetes are at potentially higher risk of infection and fatality due to COVID-19. Given the social and economic costs associated with disability due to these conditions, it is imperative that specific considerations for clinical management of these patients be observed. Moreover, the reorganization of health services around the pandemic response further exacerbates the growing crisis around limited access, treatment compliance, acute medical needs, and mental health of patients in this specific subgroup. Existing recommendations and guidelines emanating from respective bodies have addressed some of the pressure points; however, there are variations and limitations vis a vis patient with multiple comorbidities such as obesity. This article will pull together a comprehensive assessment of the association of cardiovascular disease, diabetes, obesity and COVID-19, its impact on the health systems and how best health systems can respond to mitigate current challenges and future needs. We anticipate that in the context of this pandemic, the cardiovascular disease and diabetes patients need a targeted strategy to ensure the harm to this group does not translate to huge costs to society and to the economy. Finally, we propose a triage and management protocol for patients with cardiovascular disease and diabetes in the COVID-19 settings to minimize harm to patients, health systems and healthcare workers alike.

Bhaskar, S., et al. (2020). "Acute Neurological Care in the COVID-19 Era: The Pandemic Health System REsilience PROGRAM (REPROGRAM) Consortium Pathway." Front Neurol **11**: 579.

The management of acute neurological conditions, particularly acute ischemic stroke, in the context of Coronavirus disease 2019 (COVID-19), is of importance, considering the risk of infection to the healthcare workers and patients and emerging evidence of the neuroinvasive potential of the virus. There are variations in expert guidelines further complicating the picture for clinicians in acute settings. In this light, there is a compelling need for further formulation of recommendations that compile these variations seen in the numerous guidelines present. Health system protocols for managing ongoing acute neurological care and intervention need consideration of safety and well-being of the frontline healthcare workers and the patients. We examine existing pathways and their efficacy to mitigate viral exposure to the healthcare workers and patients and synthesize a systemic approach to manage patients with acute neurological conditions in the COVID-19 scenario. Early experiences with a COVID-19 positive stroke patient treated with endovascular thrombectomy is presented to highlight the urgent need for adequate personal protective equipment (PPE) during acute neuro-interventional procedures.

Bhaskar, S., et al. (2020). "At the Epicenter of COVID-19-the Tragic Failure of the Global Supply Chain for Medical Supplies." Front Public Health **8**: 562882.

The tragic failure of the global supply chain in the face of the current coronavirus outbreak has caused acute shortages of essential frontline medical devices and personal protective equipment, crushing fear among frontline health workers and causing fundamental concerns about the sustainability of the health system. Much more coordination, integration, and management of global supply chains will be needed to mitigate the impact of the pandemics. This article describes the pressing need to revisit the governance and resilience of the supply chains that amplified the crisis at pandemic scale. We propose a model that profiles critical stockpiles and improves production efficiency through new technologies such as advanced analytics and blockchain. A new governance system that supports intervention by public-health authorities during critical emergencies is central to our recommendation, both in the face of the current crisis and to be better prepared for potential future crises. These reinforcements offer the potential to minimize the compromise of our healthcare workers and health systems due to infection exposure and build capacity toward preparedness and action for a future outbreak.

Bhogal, T., et al. (2021). "Haematological malignancy and nosocomial transmission are associated with an increased risk of death from COVID-19: results of a multi-center UK cohort." Leuk Lymphoma: 1-10.

The COVID-19 pandemic has been a disruptive event for cancer patients, especially those with haematological malignancies (HM). They may experience a more severe clinical course due to impaired immune responses. This multi-center retrospective UK audit identified cancer patients who had SARS-CoV-2 infection between 1 March and 10 June 2020 and collected data pertaining to cancer history, COVID-19 presentation and outcomes. In total, 179 patients were identified with a median age of 72 (IQR 61, 81) and follow-up of 44 days (IQR 42, 45). Forty-one percent were female and the overall mortality was 37%. Twenty-nine percent had HM and of these, those treated with chemotherapy in the preceding 28 days to COVID-19 diagnosis had worse outcome compared with solid malignancy (SM): 62% versus 19% died [HR 8.33 (95% CI, 2.56-25), p < 0.001]. Definite or probable nosocomial SARS-CoV-2 transmission accounted for 16% of cases and was associated with increased risk of death (HR 2.47, 95% CI 1.43-4.29, p = 0.001). Patients with haematological malignancies and those who acquire nosocomial transmission are at increased risk of death. Therefore, there is an urgent need to reassess shielding advice, reinforce stringent infection control, and ensure regular patient and staff testing to prevent nosocomial transmission.

Bianco, F., et al. (2020). "Preventing transmission among operating room staff during COVID-19 pandemic: the role of the Aerosol Box and other personal protective equipment." Updates Surg **72**(3): 907-910.

The COVID-19 pandemic is highly challenging for the operating room staff and healthcare workers in emergency departments. SARS-CoV-2 is a positive-sense single-stranded RNA beta-coronavirus that primarily targets the human respiratory system, with fever, cough, myalgia, and pneumonia as the most common manifestations. However, since SARS-CoV-2 RNA was detected in stool specimens much more attention has been paid to gastrointestinal symptoms such as loss of appetite, nausea, and diarrhea. Furthermore, the expression of ACE-2 receptors in absorptive enterocytes from ileum and colon suggests that these organs should also be considered as a potential high risk for SARS-CoV-2 infection. During aerosol-generating medical procedures (AGMP; e.g. intubating and extubating patients or any surgical procedures), the production of both airborne particles and droplets may increase the risk of infection. In this situation, the surgical staff is strongly recommended to wear personal protective equipment (PPE). A transparent plastic cube, the so-called "Aerosol Box" (AB), has been recently designed to lend further protection against droplets and aerosol exposure during the AGMP.

Bidkar, P., et al. (2020). "Safety tent for enhanced personal protection from aerosol-generating procedures while handling the COVID-19 patient airway." J Anaesthesiol Clin Pharmacol **36**(Suppl 1): S157-s160.

The world is going through the COVID-19 pandemic, which has high virulence and transmission rate. More significant the viral load during exposure, the greater is the likelihood of contracting a severe disease. Healthcare workers (HCWs) involved in airway care of COVID-19 patients are at high risk of getting exposed to large viral loads during aerosol-generating actions such as coughing or sneezing by the patient or during procedures such as bag-mask ventilation, intubation, extubation, and nebulization. This viral load exposure to airway caregivers decreases considerably with the use of an aerosol box during intubation. The safety tent proposed in this article is useful in limiting the viral load that HCWs are exposed to during airway procedures. Its role can be expanded beyond just intubation to protect against all aerosol-generating actions and procedures involving the patient's airway.

Bidzan, M., et al. (2020). "Does Self-Efficacy and Emotional Control Protect Hospital Staff From COVID-19 Anxiety and PTSD Symptoms? Psychological Functioning of Hospital Staff After the Announcement of COVID-19 Coronavirus Pandemic." Front Psychol **11**: 552583.

OBJECTIVES: The aim of this study was to assess coronavirus disease 2019 (COVID-19) anxiety and posttraumatic stress disorder (PTSD) symptoms in the hospital staff, as well as to identify protective factors of COVID-19 anxiety once the coronavirus pandemic was announced in Poland. METHODS: 90 healthcare workers from the hospital in Poland completed validated self-report questionnaires assessing self-efficacy, emotional control, and PTSD symptoms; a questionnaire assessing COVID-19 anxiety; and a socio-demographic questionnaire. A multiple linear regression was conducted to assess the effects of gender, being directly vs indirectly exposed to patients, and general self-efficacy on COVID-19 anxiety. RESULTS: The analysis showed that female (β = -0.271, p < 0.01) healthcare professionals indirectly exposed to patients (β = -0.336, p < 0.01) and those who reported lower levels of general self-efficacy (β = -0.295, p < 0.01) have a stronger tendency to experience COVID-19 anxiety [R (2) = 0.301, F(3,89) = 12.34, p < 0.01]. CONCLUSION: The findings show the importance of self-efficacy for dealing with COVID-19 anxiety. The internal coping strategies should be introduced to healthcare workers.

Bistoquet, M., et al. (2021). "Increased risks of SARS-CoV-2 nosocomial acquisition in high-risk COVID-19 units justify personal protective equipment: a cross-sectional study." J Hosp Infect **107**: 108-110.

Bitencourt, S. M. and C. B. Andrade (2021). "Female healthcare workers and the Covid-19 pandemic in Brazil: a sociological analysis of healthcare work." Cien Saude Colet **26**(3): 1013-1022.

The article aims to discuss the care provided by female healthcare workers in Brazil during the Covid-19 pandemic, based on a sociological analysis by authors who discuss such care as devalued and poorly paid work performed to a large extent by low-income women. The work involves social constructions of emotions and has used the body as a work instrument in care for others. In addition, the increasingly precarious nature of health work in Brazilian society, aggravated in recent decades, with an increase in temporary contracts, loss of labor rights, overload of tasks, and adverse work conditions, among others, adds to the increase in medical and hospital care in the Covid-19 pandemic. In this context, female healthcare workers experience lack of personal protective equipment, fear of coronavirus infection, concerns with their children and other family members, and illness and death of coworkers and themselves. The article highlights the need for government attention and management of healthcare work and professional societies, analyzing the work conditions female healthcare workers are experiencing in confronting the pandemic.

Blad, T., et al. (2020). "A Rapidly Deployable Test Suite for Respiratory Protective Devices in the COVID-19 Pandemic." Appl Biosaf **25**(3): 161-168.

INTRODUCTION: The current COVID-19 pandemic has caused large shortages in personal protective equipment, leading to hospitals buying their supplies from alternative suppliers or even reusing single-use items. Equipment from these alternative sources first needs to be tested to ensure that they properly protect the clinicians that depend on them. This work demonstrates a test suite for protective face masks that can be realized rapidly and cost effectively, using mainly off-the-shelf as well as 3D printing components. MATERIALS AND METHODS: The proposed test suite was designed and evaluated in order to assess its safety and proper functioning according to the criteria that are stated in the European standard norm EN149:2001+A1 7. These include a breathing resistance test, a CO(2) build-up test, and a penetration test. Measurements were performed for a variety of commercially available protective face masks for validation. RESULTS: The results obtained with the rapidly deployable test suite agree with conventional test methods, demonstrating that this setup can be used to assess the filtering properties of protective masks when conventional equipment is not available. DISCUSSION: The presented test suite can serve as a starting point for the rapid deployment of more testing facilities for respiratory protective equipment. This could greatly increase the testing capacity and ultimately improve the safety of healthcare workers battling the COVID-19 pandemic.

Blair, K. J., et al. (2020). "Protecting healthcare workers in the COVID-19 pandemic: respirator shortages and health policy responses in South America." Cad Saude Publica **36**(12): e00227520.

Blake, H., et al. (2020). "Mitigating the Psychological Impact of COVID-19 on Healthcare Workers: A Digital Learning Package." Int J Environ Res Public Health **17**(9).

The coronavirus pandemic (COVID-19) will undoubtedly have psychological impacts for healthcare workers, which could be sustained; frontline workers will be particularly at risk. Actions are needed to mitigate the impacts of COVID-19 on mental health by protecting and promoting the psychological wellbeing of healthcare workers during and after the outbreak. We developed and evaluated a digital learning package using Agile methodology within the first three weeks of UK outbreak. This e-package includes evidence-based guidance, support and signposting relating to psychological wellbeing for all UK healthcare employees. A three-step rapid development process included public involvement activities (PPIs) (STEP 1), content and technical development with iterative peer review (STEP 2), and delivery and evaluation (STEP 3). The package outlines the actions that team leaders can take to provide psychologically safe spaces for staff, together with guidance on communication and reducing social stigma, peer and family support, signposting others through psychological first aid (PFA), self-care strategies (e.g., rest, work breaks, sleep, shift work, fatigue, healthy lifestyle behaviours), and managing emotions (e.g., moral injury, coping, guilt, grief, fear, anxiety, depression, preventing burnout and psychological trauma). The e-package includes advice from experts in mental wellbeing as well as those with direct pandemic experiences from the frontline, as well as signposting to public mental health guidance. Rapid delivery in STEP 3 was achieved via direct emails through professional networks and social media. Evaluation included assessment of fidelity and implementation qualities. Essential content was identified through PPIs (n = 97) and peer review (n = 10) in STEPS 1 and 2. The most important messages to convey were deemed to be normalisation of psychological responses during a crisis, and encouragement of self-care and help-seeking behaviour. Within 7 days of completion, the package had been accessed 17,633 times, and healthcare providers had confirmed immediate adoption within their health and wellbeing provisions. Evaluation (STEP 3, n = 55) indicated high user satisfaction with content, usability and utility. Assessment of implementation qualities indicated that the package was perceived to be usable, practical, low cost and low burden. Our digital support package on 'psychological wellbeing for healthcare workers' is free to use, has been positively evaluated and was highly accessed within one week of release. It is available here: Supplementary Materials. This package was deemed to be appropriate, meaningful and useful for the needs of UK healthcare workers. We recommend provision of this e-package to healthcare workers alongside wider strategies to support their psychological wellbeing during and after the COVID-19 pandemic.

Boccalatte, L. A., et al. (2020). "Brief guideline for the prevention of COVID-19 infection in head and neck and otolaryngology surgeons." Am J Otolaryngol **41**(3): 102484.

IMPORTANCE: Anatomically, viral density is greater in the nasal cavity and the nasopharynx. It is to be expected that instrumentation in or through those areas will entail a higher risk of transmission. That's why head and neck and otolaryngologist surgeons are among the most vulnerable health professionals. OBSERVATIONS: Surgeons should essentially perform procedures they require. Surgeries should be performed with personal protective equipment suitable for the high risk of aerosolization: goggles, N95 face mask, facial mask, blood-repelling gown and gloves. It is advisable to have the cooperative COVID-19 test in all patients. Telemedicine is a useful resource if resources allow it. CONCLUSIONS AND RELEVANCE: Otolaryngologists and related specialists are among the groups at higher risk when performing surgeries and upper airway examinations. There are no emergencies in a pandemic. The care of health professionals is crucial to combating this health situation.

Boddington, N. L., et al. (2021). "Epidemiological and clinical characteristics of early COVID-19 cases, United Kingdom of Great Britain and Northern Ireland." Bull World Health Organ **99**(3): 178-189.

OBJECTIVE: To describe the clinical presentation, course of disease and health-care seeking behaviour of the first few hundred cases of coronavirus disease 2019 (COVID-19) in the United Kingdom of Great Britain and Northern Ireland. METHODS: We implemented the World Health Organization's First Few X cases and contacts investigation protocol for COVID-19. Trained public health professionals collected information on 381 virologically confirmed COVID-19 cases from 31 January 2020 to 9 April 2020. We actively followed up cases to identify exposure to infection, symptoms and outcomes. We also collected limited data on 752 symptomatic people testing negative for COVID-19, as a control group for analyses of the sensitivity, specificity and predictive value of symptoms. FINDINGS: Approximately half of the COVID-19 cases were imported (196 cases; 51.4%), of whom the majority had recent travel to Italy (140 cases; 71.4%). Of the 94 (24.7%) secondary cases, almost all reported close contact with a confirmed case (93 cases; 98.9%), many through household contact (37 cases; 39.8%). By age, a lower proportion of children had COVID-19. Most cases presented with cough, fever and fatigue. The sensitivity and specificity of symptoms varied by age, with nonlinear relationships with age. Although the proportion of COVID-19 cases with fever increased with age, for those with other respiratory infections the occurrence of fever decreased with age. The occurrence of shortness of breath also increased with age in a greater proportion of COVID-19 cases. CONCLUSION: The study has provided useful evidence for generating case definitions and has informed modelling studies of the likely burden of COVID-19.

Boffa, D. J., et al. (2020). "Pandemic Recovery Using a COVID-Minimal Cancer Surgery Pathway." Ann Thorac Surg **110**(2): 718-724.

The coronavirus disease 2019 (COVID-19) pandemic has created unprecedented disruption in health care delivery around the world. In an effort to prevent hospital-acquired COVID-19 infections, most hospitals have severely curtailed elective surgery, performing only surgeries if the patient's survival or permanent function would be compromised by a delay in surgery. As hospitals emerge from the pandemic, it will be necessary to progressively increase surgical activity at a time when hospitals continue to care for COVID-19 patients. In an attempt to mitigate the risk of nosocomial infection, we have created a patient care pathway designed to minimize risk of exposure of patients coming into the hospital for scheduled procedures. The COVID-minimal surgery pathway is a predetermined patient flow, which dictates the locations, personnel, and materials that come in contact with our cancer surgery population, designed to minimize risk for virus transmission. We outline the approach that allowed a large academic medical center to create a COVID-minimal cancer surgery pathway within 7 days of initiating discussions. Although the pathway represents a combination of recommended practices, there are no data to support its efficacy. We share the pathway concept and our experience so that others wishing to similarly align staff and resources toward the protection of patients may have an easier time navigating the process.

Boffetta, P., et al. (2021). "Determinants of SARS-CoV-2 infection in Italian healthcare workers: a multicenter study." Sci Rep **11**(1): 5788.

Healthcare workers (HCWs) are at increased risk of being infected with SARS-CoV-2, yet limited information is available on risk factors of infection. We pooled data on occupational surveillance of 10,654 HCW who were tested for SARS-CoV-2 infection in six Italian centers. Information was available on demographics, job title, department of employment, source of exposure, use of personal protective equipment (PPEs), and COVID-19-related symptoms. We fitted multivariable logistic regression models to calculate odds ratios and 95% confidence intervals of infection. The prevalence of infection ranged from 3.0 to 22.0%, and was correlated with that of the respective areas. Women were at lower risk of infection compared to men. Fever, cough, dyspnea and malaise were the symptoms most strongly associated with infection, together with anosmia and ageusia. No differences in the risk of infection were detected according to job title, or working in a COVID-19 designated department. Reported contact with a patient inside or outside the workplace was a risk factor. Use of a mask was strongly protective against risk of infection as was use of gloves. The use of a mask by the source of exposure (patient or colleague) had an independent effect in reducing infection risk.

Boilève, A., et al. (2020). "COVID-19 management in a cancer center: the ICU storm." Support Care Cancer **28**(10): 5037-5044.

A novel coronavirus, SARS-CoV-2, was first reported as a respiratory illness in December 2019 in Wuhan, China. Since then, the World Health Organization (WHO) Emergency Committee declared a global health. COVID-19 has now spread worldwide and is responsible of more than 472,216 persons, out of 9,100,090 officially diagnosed worldwide since 23 of June. In the context of cancer patients, COVID-19 has a severe impact, regarding pulmonary infection but also cancer treatments in this fragile and immunocompromised population, and ICU admission for cancer patients in the context of COVID-19 requires ethical and clinical consideration. In our cancer center, intensivists, oncologists, pharmacists, and hospital administrators had to prepare for a substantial increase in critical care bed capacity (from 10 ICU beds, 6 medical intensive care beds, and 12 surgical intensive care beds, bed capacity was increased to 28 medical intensive care beds with ventilating capacity) and to adapt infrastructure (i.e., ICU beds), supplies (i.e., drugs, ventilators, protective materials), and staff (i.e., nurses and medical staff). Overall, thirty-three COVID-19 patients were admitted in our ICU, 17 cancer-free and 16 with cancer, and 23 required mechanical ventilation, resulting in 4 deaths (of them two patients with cancer). We report here management of a dedicated intensive care unit of a cancer center during the COVID-19 infection pandemic, considering resource allocation and redistribution of healthcare workers.

Bongiovanni, M. (2020). "COVID-19 reinfection in a healthcare worker." J Med Virol.

The paper by Kang et al. (1) that has been recently published into your journal evaluated a very important topic for the management of COVID‐19 infection as the clinical meaning of being re‐tested positive in subjects recovered from COVID‐19 pneumonia. This article is protected by copyright. All rights reserved.

Booth, A., et al. (2020). "Analysis of a SARS-CoV-2 daily screening programme for healthcare workers at a district hospital in KwaZulu-Natal, a quality improvement initiativ." Afr J Prim Health Care Fam Med **12**(1): e1-e4.

The severe acute respiratory syndrome coronavirus 2 (SARS-CoV-2) has caused an unprecedented burden on our healthcare systems and workers. Healthcare workers are at risk of contracting and spreading SARS-CoV-2 given their proximity to positive cases, often with a lack of personal protective equipment. The South African Department of Health requires that all employees be screened daily for symptoms and potential persons under investigation identified timeously. This report aims to assesses the efficacy of daily self-screening tools in detecting and managing potential staff cases of SARS-CoV-2. Our hospital, situated in KwaZulu-Natal, South Africa, developed a daily self-screening tool for all healthcare workers to complete, consisting of questions on symptoms and epidemiological risk factors. The screening tools were collected and assessed after four weeks of use. Fifty-four forms were assessed. Twenty-eight (51.9%) forms were not completed, whilst 12 (22.2%) indicated positive symptoms with no documentation that any further medical assessment, testing or isolation was done. We identified that the poor completion of forms was likely because of the lack of education of staff on the importance of the forms, poor oversight by management, staff forgetfulness or lack of awareness of the forms. Screening of staff is vital during this pandemic but requires constant oversight by line managers, staff motivation and adequate education. Ongoing development of efficient screening programmes is required.

Bord, S., et al. (2021). "[WEARING A MASK IS A PERSONAL PROTECTION AGAINST SARS-COV-2 INFECTION EVEN IN A VACCINATION-ON-BOARDING COUNTRY]." Harefuah **160**(3): 132-138.

Even with the advent of the COVID-19 vaccine, masks and social distancing are recommended as a precautionary measure to suppress SARS-CoV-2 infection and disease. In Israel, as in many other countries, despite official regulations and widespread availability and accessibility to affordable effective masks, the use of face masks is not consistent or universal. Physicians and other medical and health professionals have a vital role to play in communicating to the public about the importance of masking and encouraging people to wear face masks correctly and consistently. This review underscores the importance of masking as a protective public health mitigation measure. It describes types of face masks mainly used by the public and their effectiveness. It emphasizes the importance of identifying and addressing barriers (e.g., physical, social, economic) to encourage widespread and sustained appropriate use of masks. The article also proposes strategies to enhance masking, such as changing social norms through targeted local interventions and governmental control of mask quality and price.

Boškoski, I., et al. (2020). "COVID-19 pandemic and personal protective equipment shortage: protective efficacy comparing masks and scientific methods for respirator reuse." Gastrointest Endosc **92**(3): 519-523.

BACKGROUND AND AIMS: The abrupt outbreak of the novel coronavirus disease 2019 and its rapid spread over many healthcare systems throughout the world has led to a shortage in personal protective equipment (PPE), which cannot be solved by reducing their use or by increasing production. It is thus necessary to promote PPE rational use, highlighting possible differences in terms of efficacy and promoting an effective technique to reuse them. METHODS: A literature search was performed on PubMed, Scopus, Cochrane database, and Google Scholar, and from the 25 top cited articles, 15 were selected for relevance and impact. RESULTS: Most studies on previous respiratory virus epidemics to date suggest surgical masks are not inferior compared with N95 respirators in terms of protective efficacy among healthcare workers. Therefore, the use of N95 respirators should be limited to high-risk situations. Concerning respirator reuse, highly energetic, short-wave, ultraviolet germicidal irradiation (UVGI) at 254 nm was determined to decontaminate N95 respirators from viral respiratory agents, but UVGI requires careful consideration of the type of respirator and of the biologic target. CONCLUSIONS: Rational use and successful reuse of respirators can help in the shortage of PPE during a pandemic. Further studies testing UVGI and other decontamination techniques are an unmet need. The definitive answer to pandemic issues can be found in artificial intelligence and deep learning. These groundbreaking modalities could help in identifying high-risk patients and in suggesting appropriate types and use of PPE.

Bouchard, J. P. (2020). "[Covid-19: health care workers between heroism and ostracism]." Rev Infirm **69**(260-261): 31-32.

Originating in China in November 2019, the wave of infection caused by the new coronavirus Sars-CoV-2 is sweeping across the planet through human contact and fast, global, mass travel. Health professionals on every level are fighting relentlessly against Covid-19, this deadly, invisible yet real enemy. The public, increasingly worried, expect them to protect it against the disease and death. Perceived by most people as a line of defence against the pandemic, health professionals are supported and treated as heroes. Some people howeverfear tthat they may be possible contamination agents.

Boujaoude, Z., et al. (2021). "Safety and Feasibility of a Novel Protocol for Percutaneous Dilatational Tracheostomy in Patients with Respiratory Failure due to COVID-19 Infection: A Single Center Experience." Pulm Med **2021**: 8815925.

INTRODUCTION: The rapidly spreading Novel Coronavirus 2019 (COVID-19) appeared to be a highly transmissible pathogen in healthcare environments and had resulted in a significant number of patients with respiratory failure requiring tracheostomy, an aerosol-generating procedure that places healthcare workers at high risk of contracting the infection. Instead of deferring or delaying the procedure, we developed and implemented a novel percutaneous dilatational tracheostomy (PDT) protocol aimed at minimizing the risk of transmission while maintaining favorable procedural outcome. Patients and Methods. All patients who underwent PDT per novel protocol were included in the study. The key element of the protocol was the use of apnea during the critical part of the insertion and upon any opening of the ventilator circuit. This was coupled with the use of enhanced personnel protection equipment (PPE) with a powered air-purifying respirator (PAPR). The operators underwent antibody serology testing and were evaluated for COVID-19 symptoms two weeks from the last procedure included in the study. RESULTS: Between March 12th and June 30th, 2020, a total of 32 patients underwent PDT per novel protocol. The majority (80%) were positive for COVID-19 at the time of the procedure. The success rate was 94%. Only one patient developed minor self-limited bleeding. None of the proceduralists developed positive serology or any symptoms compatible with COVID-19 infection. CONCLUSION: A novel protocol that uses periods of apnea during opening of the ventilator circuit along with PAPR-enhanced PPE for PDT on COVID-19 patients appears to be effective and safe for patients and healthcare providers.

Bourgeault, I. L., et al. (2020). "The COVID-19 pandemic presents an opportunity to develop more sustainable health workforces." Hum Resour Health **18**(1): 83.

This commentary addresses the critically important role of health workers in their countries' more immediate responses to COVID-19 outbreaks and provides policy recommendations for more sustainable health workforces. Paradoxically, pandemic response plans in country after country, often fail to explicitly address health workforce requirements and considerations. We recommend that policy and decision-makers at the facility, regional and country-levels need to: integrate explicit health workforce requirements in pandemic response plans, appropriate to its differentiated levels of care, for the short, medium and longer term; ensure safe working conditions with personal protective equipment (PPE) for all deployed health workers including sufficient training to ensure high hygienic and safety standards; recognise the importance of protecting and promoting the psychological health and safety of all health professionals, with a special focus on workers at the point of care; take an explicit gender and social equity lens, when addressing physical and psychological health and safety, recognising that the health workforce is largely made up of women, and that limited resources lead to priority setting and unequitable access to protection; take a whole of the health workforce approach-using the full skill sets of all health workers-across public health and clinical care roles-including those along the training and retirement pipeline-and ensure adequate supervisory structures and operating procedures are in place to ensure inclusive care of high quality; react with solidarity to support regions and countries requiring more surge capacity, especially those with weak health systems and more severe HRH shortages; and acknowledge the need for transparent, flexible and situational leadership styles building on a different set of management skills.

Bradford Smith, P., et al. (2020). "A scoping review of surgical masks and N95 filtering facepiece respirators: Learning from the past to guide the future of dentistry." Saf Sci **131**: 104920.

With the 2019 emergence of coronavirus disease 19 (colloquially called COVID-19) came renewed public concern about airborne and aerosolized virus transmission. Accompanying this concern were many conflicting dialogues about which forms of personal protective equipment best protect dental health care practitioners and their patients from viral exposure. In this comprehensive review we provide a thorough and critical assessment of face masks and face shields, some of the most frequently recommended personal safeguards against viral infection. We begin by describing the function and practicality of the most common mask types used in dentistry: procedural masks, surgical masks, and filtering respirator facemasks (also called N95s). This is followed by a critical assessment of mask use based on a review of published evidence in three key domains: the degree to which each mask type is shown to protect against airborne and aerosolized disease, the reported likelihood for non-compliance among mask users, and risk factors associated with both proper and improper mask use. We use this information to conclude our review with several practical, evidence-based recommendations for mask use in dental and dental educational clinics.

Brandão, C. F. S., et al. (2020). "Clinical simulation strategies for knowledge integration relating to initial critical recognition and management of COVID-19 for use within continuing education and health-related academia in Brazil: a descriptive study." Sao Paulo Med J **138**(5): 385-392.

BACKGROUND: The COVID-19 pandemic has led to an immense need to develop training on case recognition and management, with a focus on patients' and health professionals' safety at several levels of healthcare settings in Brazil. Different simulation strategies can be included in the diverse clinical care phases for these patients. OBJECTIVE: To suggest a complete simulation-based training program for Brazilian hospitals and/or academic institutions at this moment of the pandemic. DESIGN AND SETTING: Descriptive analysis on possible simulated clinical cases using different methodologies, thereby supporting suspected or confirmed COVID-19 patients. METHODS: This was a reflective theoretical descriptive study on an educational program based on clinical simulation, with four practical phases at different performance and complexity levels. Wearing, handling and adequately disposing of personal protective equipment, along with specific respiratory procedures in different healthcare settings up to intensive care for seriously infected patients were addressed. RESULTS: This program was designed for application at different Brazilian healthcare levels through different clinical simulation strategies. Summaries of expected performance were suggested in order to standardize technical capacity within these simulation settings, so as to serve these levels. CONCLUSIONS: Developing training programs for situations such as the current COVID-19 pandemic promotes safety not only for patients but also for healthcare workers. In the present context, clear definition of which patients need hospital outpatient or inpatient care will avoid collapse of the Brazilian healthcare system. Institutions that do not have simulated environments can, through the examples described, adopt procedures to promote didactic information in order to help healthcare professionals during this time.

Brant-Zawadzki, G. M., et al. (2021). "An Aerosol Containment and Filtration Tent for Intubation During the COVID-19 Pandemic." Surg Innov: 1553350621999976.

Background. Exposure to infectious droplets confers a high risk for infection transmission by the SARS-CoV-2 coronavirus. Aerosolizing procedures pose particular concern for increasing healthcare workers' (HCWs) risks of infection. Multiple creative personal protective equipment solutions have been utilized to minimize exposure to infectious particles; however, the overall benefit of many of these devices is limited by a number of factors. Methods. We designed an intubation tent consisting of a metal frame and a clear plastic sheet. The flexible walls of our tent offer increased maneuverability & access, although the efficacy in reducing risk of transmission to HCWs remained unclear. Using an atomizer, particle generator, and matchstick smoke, we simulated the generation of infectious respiratory droplets and aerosols and tested whether our device effectively decreased the concentration of these particles to which a provider might be exposed. Finally, we tested whether the addition of a vacuum fan fit with a high efficiency particulate air filter designed to evacuate contaminated air would influence particle concentrations inside and outside the tent. Results. Droplet dispersion tests with the tent in place showed that the simulated droplet distribution was limited to surfaces within the tent. Aerosol testing under a variety of circumstances consistently showed only a minor rise in particle concentration in the air outside the tent despite an initial peak of particle concentration during generation within. All testing demonstrated declining inside concentrations over time. Conclusions. Our simulations suggest our device has the potential to effectively decrease HCWs' exposure to infectious droplets and aerosolized viral particles.

Brant-Zawadzki, M., et al. (2020). "SARS-CoV-2 antibody prevalence in health care workers: Preliminary report of a single center study." PLoS One **15**(11): e0240006.

Serological surveys have been conducted to establish prevalence for COVID-19 antibodies in various cohorts and communities, reporting a wide range of outcomes. The prevalence of such antibodies among healthcare workers, presumed at higher risk for infection, has been increasingly investigated, more studies are needed to better understand the risks and infection transmission in different healthcare settings. The present study reports on initial sero-surveillance conducted on healthcare workers at a regional hospital system in Orange County, California, during May and June, 2020. Study subjects were recruited from the entire hospital employee workforce and the independent medical staff. Data were collected for job duties and locations, COVID-19 symptoms, a PCR test history, travel record since January 2020, and existence of household contacts with COVID-19. A blood sample was collected from each subject for serum analysis for IgG antibodies to SARS-CoV-2. Of 2,992 tested individuals, a total 2,924 with complete data were included in the analysis. Observed prevalence of 1.06% (31 antibody positive cases), adjusted prevalence of 1.13% for test sensitivity and specificity were identified. Significant group differences between positive vs. negative were observed for age (z = 2.65, p = .008), race (p = .037), presence of fever (p < .001), and loss of smell (p < .001), but not for occupations (p = .710). Possible explanation for this low prevalence includes a relatively low local geographic community prevalence (~4.4%) at the time of testing, the hospital's timely procurement of personal protective equipment, rigorous employee education, patient triage, and treatment protocol development and implementation. In addition, cross-reactive adaptive T cell mediated immunity, as recently described, may possibly play a greater role in healthcare workers than in the general population.

Bresler, A. M., et al. (2020). "[SARS-CoV-2-How can and must medical personnel protect themselves?]." Gefasschirurgie: 1-10.

The SARS-CoV‑2 pandemic represents a great challenge worldwide not only under the aspect of treatment but also of prevention. The infection curve could be flattened by the rapid implementation of simple distance and hygiene measures. In order to ensure current and future patient care in conjunction with protection of medical personnel working in the healthcare system during this pandemic, adequate protective equipment is essential. Routine care can only be resumed if there is sufficient and adequate personal protective equipment (PPE). If recommended hygiene protective measures including the necessary PPE are seriously taken into account both elective interventions and emergency care can be carried out without an increased risk of infection. This is very important in vascular surgery with a high proportion of urgent and emergency disease management.

Publisher: Abstract available from the publisher.

ger

Brooke, B. D., et al. (2020). "Implementing malaria control in South Africa, Eswatini and southern Mozambique during the COVID-19 pandemic." S Afr Med J **110**(11): 1072-1076.

The COVID-19 pandemic has strained healthcare delivery systems in a number of southern African countries. Despite this, it is imperative that malaria control and elimination activities continue, especially to reduce as far as possible the number and rate of hospitalisations caused by malaria. The implementation of enhanced malaria control/elimination activities in the context of COVID-19 requires measures to protect healthcare workers and the communities they serve. The aim of this review is therefore to present innovative ideas for the timely implementation of malaria control without increasing the risk of COVID-19 to healthcare workers and communities. Specific recommendations for parasite and vector surveillance, diagnosis, case management, mosquito vector control and community outreach and sensitisation are given.

Brophy, J. T., et al. (2021). "Sacrificed: Ontario Healthcare Workers in the Time of COVID-19." New Solut **30**(4): 267-281.

Healthcare workers (HCWs) in Ontario, Canada have faced unprecedented risks during the COVID-19 pandemic. They have been infected at an elevated rate compared to the general public. HCWs have argued for better protections with minimal success. A worldwide shortage of N95s and comparable respirators appears to have influenced guidelines for protection, which stand at odds with increasing scientific evidence. In-depth interviews were conducted with ten frontline HCWs about their concerns. They reported that the risk of contracting COVID-19 and infecting family members has created intense anxiety. This, in conjunction with understaffing and an increased workload, has resulted in exhaustion and burnout. HCWs feel abandoned by their governments, which failed to prepare for an inevitable epidemic, despite recommendations. The knowledge that they are at increased risk of infection due to lack of protection has resulted in anger, frustration, fear, and a sense of violation that may have long-lasting implications.Sacrifié: Le personnel de la santé ontarien à l'époque de la COVID-19RésuméEn Ontario, au Canada, le personnel de la santé a eu à faire face à des risques sans précédent durant la pandémie de COVID-19. On y a constaté un taux d'infection plus élevé chez les travailleuses et travailleurs de la santé (TTS) qu'au sein de la population en général. Les TTS ont revendiqué des moyens de protection améliorés, sans grand succès. Une pénurie mondiale de masques respirateurs de type N95 ou similaires semble avoir joué sur les directives en matière de protection, qui ne cadrent pas avec une accumulation de preuves scientifiques. Lors d'entretiens en profondeur, dix TTS de première ligne ont été invités à donner leur avis sur la situation. à les entendre, le risque de contracter la COVID-19 et d'infecter les membres de leur famille leur cause beaucoup d'anxiété. Associée à un manque de personnel et à une charge de travail accrue, cette anxiété se traduit par un épuisement physique et professionnel. Les TTS se sentent abandonnés par leurs gouvernements, qui ont manqué de se préparer à l'inévitabilité d'une épidémie, malgré ce qui leur avait été recommandé. Leur réalisation d'être exposés à un plus grand risque d'infection par manque d'équipement de protection s'est muée en colère, frustration et peur, et en un sentiment de violation de leurs droits dont on peut craindre qu'il subsiste fort longtemps.

Brown, C., et al. (2020). "Brief Summary of Potential SARS-CoV-2 Prophylactic and Treatment Drugs in the Emergency Department." West J Emerg Med **21**(3): 510-513.

As of March 30(th), 2020 there were 161,807 total cases and 2,953 total deaths of SARS-CoV-2 in the United States, with the number of cases expected to rise. Other than supportive care, there are no SARS-CoV-2 specific treatments available for patients discharged from the emergency department (ED) or those admitted to the hospital. In addition, there are no vaccines available to protect our at-risk healthcare workers. The National Institutes of Health is conducting a Phase 1 clinical trial to evaluate for a potential vaccine and the recipients have started to receive the investigational vaccine.2 We present a brief overview of the potential prophylactic and treatment agents under investigation, some which could be initiated in the ED if proven effective.

Brown, E. and L. M. Chan (2020). "Should chest compressions be considered an aerosol-generating procedure? A literature review in response to recent guidelines on personal protective equipment for patients with suspected COVID-19." Clin Med (Lond) **20**(5): e154-e159.

There is disagreement between international guidelines on the level of personal protective equipment (PPE) required for chest compressions for patients with suspected COVID-19. This discrepancy centres on whether they are considered to be an aerosol-generating procedure (AGP), thus requiring airborne protection to prevent transmission to healthcare workers (HCWs). The need to don higher-level PPE has to be weighed against the resulting delay to emergency treatment.We performed a literature search on this topic which found eight relevant studies. All were observational with low patient numbers and multiple confounding factors, but describe cases of acute respiratory infection transmission during chest compressions. One systematic review concluded that chest compressions were not an AGP. Two simulated studies (released as preprints) potentially demonstrate aerosol generation. Given that there is evidence for infection transmission during chest compressions, we conclude that a precautionary approach with appropriate PPE is necessary to protect HCW from contracting a potentially fatal infection.

Brugnolli, A. and L. Prosperi (2020). "[The use of facial masks: updated information after Covid-19]." Assist Inferm Ric **39**(3): 154-161.

. The use of facial masks: updated information after Covid-19. Preventive spread of infections to and from healthcare workers and patients relies on effective use of personal protective equipment. During the Covid-19 epidemic, different, often conflicting indications were given, based on evolving knowledge on the spread mechanism of the virus. In this contribution the most up to date indications on the use of facial masks by health care workers and general population, for the prevention of contagion are discussed and confronted.

Bruni, M., et al. (2020). "Persistence of Anti-SARS-CoV-2 Antibodies in Non-Hospitalized COVID-19 Convalescent Health Care Workers." J Clin Med **9**(10).

Although antibody response to SARS-CoV-2 can be detected early during the infection, several outstanding questions remain to be addressed regarding the magnitude and persistence of antibody titer against different viral proteins and their correlation with the strength of the immune response. An ELISA assay has been developed by expressing and purifying the recombinant SARS-CoV-2 Spike Receptor Binding Domain (RBD), Soluble Ectodomain (Spike), and full length Nucleocapsid protein (N). Sera from healthcare workers affected by non-severe COVID-19 were longitudinally collected over four weeks, and compared to sera from patients hospitalized in Intensive Care Units (ICU) and SARS-CoV-2-negative subjects for the presence of IgM, IgG and IgA antibodies as well as soluble pro-inflammatory mediators in the sera. Non-hospitalized subjects showed lower antibody titers and blood pro-inflammatory cytokine profiles as compared to patients in Intensive Care Units (ICU), irrespective of the antibodies tested. Noteworthy, in non-severe COVID-19 infections, antibody titers against RBD and Spike, but not against the N protein, as well as pro-inflammatory cytokines decreased within a month after viral clearance. Thus, rapid decline in antibody titers and in pro-inflammatory cytokines may be a common feature of non-severe SARS-CoV-2 infection, suggesting that antibody-mediated protection against re-infection with SARS-CoV-2 is of short duration. These results suggest caution in using serological testing to estimate the prevalence of SARS-CoV-2 infection in the general population.

Bueno Ferrán, M. and S. Barrientos-Trigo (2021). "[Caring for the caregiver: The emotional impact of the coronavirus epidemic on nurses and other health professionals]." Enferm Clin **31**: S35-s39.

The health crisis caused by the COVID-19 virus pandemic has once again highlighted the role of health professionals as a key element for their containment, who suffer from high tension marked by healthcare pressure and the lack of means of protection. Given this tension, it is relevant to analyze the emotional impact on health professionals of the coronavirus pandemic and the coping resources to reduce or mitigate this impact. Stress, sleep disorders and depressive symptoms stand out. Some strategies are recommended that have been used by professionals who have previously been under pressure from COVID-19 and have been helpful to them. Finally, some recommendations whose efficacy is known for managing emotional impact are pointed out.

Publisher: Abstract available from the publisher.

spa

Buhat, C. A. H., et al. (2021). "A mathematical model of COVID-19 transmission between frontliners and the general public." Netw Model Anal Health Inform Bioinform **10**(1): 17.

The number of COVID-19 cases is continuously increasing in different countries including the Philippines. It is estimated that the basic reproduction number of COVID-19 is around 1.5-4 (as of May 2020). The basic reproduction number characterizes the average number of persons that a primary case can directly infect in a population full of susceptible individuals. However, there can be superspreaders that can infect more than this estimated basic reproduction number. In this study, we formulate a conceptual mathematical model on the transmission dynamics of COVID-19 between the frontliners and the general public. We assume that the general public has a reproduction number between 1.5 and 4, and frontliners (e.g. healthcare workers, customer service and retail personnel, food service crews, and transport or delivery workers) have a higher reproduction number. Our simulations show that both the frontliners and the general public should be protected against the disease. Protecting only the frontliners will not result in flattening the epidemic curve. Protecting only the general public may flatten the epidemic curve but the infection risk faced by the frontliners is still high, which may eventually affect their work. The insights from our model remind us of the importance of community effort in controlling the transmission of the disease.

Burgoyne, M. E., et al. (2020). "COVID-19 Barriers to Care for Pregnant Patients in Prolonged Isolation." Case Rep Obstet Gynecol **2020**: 8847859.

Severe acute respiratory syndrome coronavirus 2 (SARS-CoV-2), the agent responsible for coronavirus disease 2019 (COVID-19), continues to have a devastating impact on healthcare systems worldwide, and many questions remain unanswered. The effect of COVID-19 on the pregnant population is widely debated, and the unique risks in pregnancy have not yet been elucidated. What has been established, however, is the recommendation for healthcare workers to use personal protective equipment (PPE) for both contact and airborne precautions to prevent transmission of the pathogen-adding another barrier to care for vulnerable populations. We report a case of a young woman from Haiti during her first pregnancy, who was admitted to the antepartum service at 22 weeks of gestation with preterm premature rupture of membranes (PPROM) and remained admitted in isolation, though asymptomatic, for over six weeks due to persistent positive SARS-CoV-2 testing. Our case highlights the unique barriers to care that COVID-19 poses to antepartum patients, particularly in the setting of pregnant women with persistent positive testing.

Burke, R. M., et al. (2020). "Enhanced contact investigations for nine early travel-related cases of SARS-CoV-2 in the United States." PLoS One **15**(9): e0238342.

Coronavirus disease 2019 (COVID-19), the respiratory disease caused by severe acute respiratory syndrome coronavirus 2 (SARS-CoV-2), was first identified in Wuhan, China and has since become pandemic. In response to the first cases identified in the United States, close contacts of confirmed COVID-19 cases were investigated to enable early identification and isolation of additional cases and to learn more about risk factors for transmission. Close contacts of nine early travel-related cases in the United States were identified and monitored daily for development of symptoms (active monitoring). Selected close contacts (including those with exposures categorized as higher risk) were targeted for collection of additional exposure information and respiratory samples. Respiratory samples were tested for SARS-CoV-2 by real-time reverse transcription polymerase chain reaction at the Centers for Disease Control and Prevention. Four hundred four close contacts were actively monitored in the jurisdictions that managed the travel-related cases. Three hundred thirty-eight of the 404 close contacts provided at least basic exposure information, of whom 159 close contacts had ≥1 set of respiratory samples collected and tested. Across all actively monitored close contacts, two additional symptomatic COVID-19 cases (i.e., secondary cases) were identified; both secondary cases were in spouses of travel-associated case patients. When considering only household members, all of whom had ≥1 respiratory sample tested for SARS-CoV-2, the secondary attack rate (i.e., the number of secondary cases as a proportion of total close contacts) was 13% (95% CI: 4-38%). The results from these contact tracing investigations suggest that household members, especially significant others, of COVID-19 cases are at highest risk of becoming infected. The importance of personal protective equipment for healthcare workers is also underlined. Isolation of persons with COVID-19, in combination with quarantine of exposed close contacts and practice of everyday preventive behaviors, is important to mitigate spread of COVID-19.

Burns, E. S., et al. (2021). "Physical and psychological impacts of handwashing and personal protective equipment usage in the COVID-19 pandemic: A UK based cross-sectional analysis of healthcare workers." Dermatol Ther: e14885.

The COVID-19 pandemic has necessitated intensified handwashing and mask usage for healthcare staff. A retrospective cross-sectional study was performed primarily to investigate the potential skin damage and secondary impacts on wellbeing of staff resulting from these practices. Additionally the availability and uptake of occupational health services and moisturisers in the work place was also assessed. The survey was distributed to NHS staff between April and May 2020 and asked questions regarding skin damage, impact on wellbeing and availability and utilisation of occupational health input and moisturisers. Of the 211 responders, 167 washed their hands more than ten times per shift. Three quarters of these reported cracks or fissures in one or more regions of their hands, most frequently to the back of the hands or web spaces. Amongst the 157 staff who wore FFP3 masks, redness of the nasal area was most frequently reported with 8% reporting facial blisters. 36% of staff reported a substantial impact on one or more aspects of their wellbeing. Only 7% of respondents had received specialist advice, yet a quarter (26%) had made or anticipated needing changes to their occupational duties. The majority (63%) felt they required no specialist input, despite 38% of these reporting a substantial detriment to their wellbeing. Handwashing and face mask use is resulting in skin damage amongst healthcare workers during the COVID-19 pandemic, with associated detriment to wellbeing. Healthcare services need to take action to implement measures to prevent, reduce and treat damage including promotion of available specialist support.

Burns, N. M., et al. (2020). "Emergency Mail-in Voting in Rhode Island: Protecting Civic Participation During COVID-19 and Beyond." R I Med J (2013) **103**(8): 14-17.

The COVID-19 pandemic challenges safe and equitable voting in the United States' 2020 elections, and in response, several states including Rhode Island (RI) have made significant changes to election policy. In addition to increasing accessibility of mail-in voting by mailing applications to all registered voters, RI has suspended their notary/witness requirement for both the primary and general election. However, RI's "emergency" voting process still plays a crucial role in allowing voters who missed the mail-in ballot application deadline, such as those unexpectedly hospitalized in the days leading up to the election, to still cast their ballot. COVID-19 has also forced RI to modify its emergency voting procedures, most notably allowing healthcare workers to serve on bipartisan ballot delivery teams. This commentary highlights these salient updates to voting procedures and serves as a primer as to how interested health care workers may navigate this process alongside patients and lead in the arena of patient voting rights.

Burton, M. J., et al. (2020). "Antimicrobial mouthwashes (gargling) and nasal sprays administered to patients with suspected or confirmed COVID-19 infection to improve patient outcomes and to protect healthcare workers treating them." Cochrane Database Syst Rev **9**: Cd013627.

BACKGROUND: COVID-19 infection poses a serious risk to patients and - due to its contagious nature - to those healthcare workers (HCWs) treating them. If the mouth and nose of patients with infection are irrigated with antimicrobial solutions, this may help the patients by killing any coronavirus present at those sites. It may also reduce the risk of the active infection being passed to HCWs through droplet transmission or direct contact. However, the use of such antimicrobial solutions may be associated with harms related to the toxicity of the solutions themselves or alterations in the natural microbial flora of the mouth or nose. OBJECTIVES: To assess the benefits and harms of antimicrobial mouthwashes and nasal sprays administered to patients with suspected or confirmed COVID-19 infection to both the patients and the HCWs caring for them. SEARCH METHODS: Information Specialists from Cochrane ENT and Cochrane Oral Health searched the Central Register of Controlled Trials (CENTRAL 2020, Issue 6); Ovid MEDLINE; Ovid Embase and additional sources for published and unpublished trials. The date of the search was 1 June 2020.  SELECTION CRITERIA: This is a question that urgently requires evidence, however at the present time we did not anticipate finding many completed RCTs. We therefore planned to include the following types of studies: randomised controlled trials (RCTs); quasi-RCTs; non-randomised controlled trials; prospective cohort studies; retrospective cohort studies; cross-sectional studies; controlled before-and-after studies. We set no minimum duration for the studies.   We sought studies comparing antimicrobial mouthwash and/or nasal spray (alone or in combination) at any concentration, delivered with any frequency or dosage to suspected/confirmed COVID-19 patients. DATA COLLECTION AND ANALYSIS: We used standard Cochrane methodological procedures. Our primary outcomes were: 1) RECOVERY* (www.recoverytrial.net) outcomes in patients (mortality; hospitalisation status; use of ventilation; use of renal dialysis or haemofiltration); 2) incidence of symptomatic or test-positive COVID-19 infection in HCWs; 3) significant adverse event: anosmia (or disturbance in sense of smell). Our secondary outcomes were: 4) change in COVID-19 viral load in patients; 5) COVID-19 viral content of aerosol (when present); 6) other adverse events: changes in microbiome in oral cavity, nasal cavity, oro- or nasopharynx; 7) other adverse events: allergy, irritation/burning of nasal, oral or oropharyngeal mucosa (e.g. erosions, ulcers, bleeding), long-term staining of mucous membranes or teeth, accidental ingestion. We planned to use GRADE to assess the certainty of the evidence for each outcome. MAIN RESULTS: We found no completed studies to include in this review. We identified 16 ongoing studies (including 14 RCTs), which aim to enrol nearly 1250 participants. The interventions included in these trials are ArtemiC (artemisinin, curcumin, frankincense and vitamin C), Citrox (a bioflavonoid), cetylpyridinium chloride, chlorhexidine, chlorine dioxide, essential oils, hydrogen peroxide, hypertonic saline, Kerecis spray (omega 3 viruxide - containing neem oil and St John's wort), neem extract, nitric oxide releasing solution, povidone iodine and saline with baby shampoo.  AUTHORS' CONCLUSIONS: We identified no studies for inclusion in this review. This is not surprising given the relatively recent emergence of COVID-19 infection. It is promising that the question posed in this review is being addressed by a number of RCTs and other studies. We are concerned that few of the ongoing studies specifically state that they will evaluate adverse events such as changes in the sense of smell or to the oral and nasal microbiota, and any consequences thereof. Very few interventions have large and dramatic effect sizes. If a positive treatment effect is demonstrated when studies are available for inclusion in this review, it may not be large. In these circumstances in particular it may be a challenge to weigh up the benefits against the harms if the latter are of uncertain frequency and severity.

Burton, M. J., et al. (2020). "Antimicrobial mouthwashes (gargling) and nasal sprays to protect healthcare workers when undertaking aerosol-generating procedures (AGPs) on patients without suspected or confirmed COVID-19 infection." Cochrane Database Syst Rev **9**: Cd013628.

BACKGROUND: COVID-19 infection poses a serious risk to patients and - due to its contagious nature - to those healthcare workers (HCWs) treating them. The risks of transmission of infection are greater when a patient is undergoing an aerosol-generating procedure (AGP). Not all those with COVID-19 infection are symptomatic, or suspected of harbouring the infection. If a patient who is not known to have or suspected of having COVID-19 infection is to undergo an AGP, it would nonetheless be sensible to minimise the risk to those HCWs treating them. If the mouth and nose of an individual undergoing an AGP are irrigated with antimicrobial solutions, this may be a simple and safe method of reducing the risk of any covert infection being passed to HCWs through droplet transmission or direct contact. Alternatively, the use of antimicrobial solutions by the HCW may decrease the chance of them acquiring COVID-19 infection. However, the use of such antimicrobial solutions may be associated with harms related to the toxicity of the solutions themselves or alterations in the natural microbial flora of the mouth or nose. OBJECTIVES: To assess the benefits and harms of antimicrobial mouthwashes and nasal sprays administered to HCWs and/or patients when undertaking AGPs on patients without suspected or confirmed COVID-19 infection. SEARCH METHODS: Information Specialists from Cochrane ENT and Cochrane Oral Health searched the Central Register of Controlled Trials (CENTRAL 2020, Issue 6); Ovid MEDLINE; Ovid Embase and additional sources for published and unpublished trials. The date of the search was 1 June 2020.  SELECTION CRITERIA: This is a question that urgently requires evidence, however at the present time we did not anticipate finding many completed RCTs. We therefore planned to include the following types of studies: randomised controlled trials (RCTs); quasi-RCTs; non-randomised controlled trials; prospective cohort studies; retrospective cohort studies; cross-sectional studies; controlled before-and-after studies. We set no minimum duration for the studies.   We sought studies comparing any antimicrobial mouthwash and/or nasal spray (alone or in combination) at any concentration, delivered to the patient or HCW before and/or after an AGP. DATA COLLECTION AND ANALYSIS: We used standard Cochrane methodological procedures. Our primary outcomes were: 1) incidence of symptomatic or test-positive COVID-19 infection in HCWs or patients; 2) significant adverse event: anosmia (or disturbance in sense of smell). Our secondary outcomes were: 3) COVID-19 viral content of aerosol (when present); 4) change in COVID-19 viral load at site(s) of irrigation; 5) other adverse events: changes in microbiome in oral cavity, nasal cavity, oro- or nasopharynx; 6) other adverse events: allergy, irritation/burning of nasal, oral or oropharyngeal mucosa (e.g. erosions, ulcers, bleeding), long-term staining of mucous membranes or teeth, accidental ingestion. We planned to use GRADE to assess the certainty of the evidence for each outcome. MAIN RESULTS: We found no completed studies to include in this review.   AUTHORS' CONCLUSIONS: We identified no studies for inclusion in this review, nor any ongoing studies. The absence of completed studies is not surprising given the relatively recent emergence of COVID-19 infection. However, we are disappointed that this important clinical question is not being addressed by ongoing studies.

Burton, M. J., et al. (2020). "Use of antimicrobial mouthwashes (gargling) and nasal sprays by healthcare workers to protect them when treating patients with suspected or confirmed COVID-19 infection." Cochrane Database Syst Rev **9**: Cd013626.

BACKGROUND: COVID-19 infection poses a serious risk to patients and - due to its contagious nature - to those healthcare workers (HCWs) treating them. If the mouth and nose of HCWs are irrigated with antimicrobial solutions, this may help reduce the risk of active infection being passed from infected patients to HCWs through droplet transmission or direct contact. However, the use of such antimicrobial solutions may be associated with harms related to the toxicity of the solutions themselves, or alterations in the natural microbial flora of the mouth or nose. Understanding these possible side effects is particularly important when the HCWs are otherwise fit and well. OBJECTIVES: To assess the benefits and harms of antimicrobial mouthwashes and nasal sprays used by healthcare workers (HCWs) to protect themselves when treating patients with suspected or confirmed COVID-19 infection. SEARCH METHODS: Information Specialists from Cochrane ENT and Cochrane Oral Health searched the Central Register of Controlled Trials (CENTRAL 2020, Issue 6); Ovid MEDLINE; Ovid Embase and additional sources for published and unpublished trials. The date of the search was 1 June 2020.  SELECTION CRITERIA: This is a question that urgently requires evidence, however at the present time we did not anticipate finding many completed randomised controlled trials (RCTs). We therefore planned to include the following types of studies: RCTs; quasi-RCTs; non-randomised controlled trials; prospective cohort studies; retrospective cohort studies; cross-sectional studies; controlled before-and-after studies. We set no minimum duration for the studies.   We sought studies comparing any antimicrobial mouthwash and/or nasal spray (alone or in combination) at any concentration, delivered to HCWs, with or without the same intervention being given to the patients with COVID-19. DATA COLLECTION AND ANALYSIS: We used standard Cochrane methodological procedures. Our primary outcomes were: 1) incidence of symptomatic or test-positive COVID-19 infection in HCWs; 2) significant adverse event: anosmia (or disturbance in sense of smell). Our secondary outcomes were: 3) viral content of aerosol, when present (if intervention administered to patients); 4) other adverse events: changes in microbiome in oral cavity, nasal cavity, oro- or nasopharynx; 5) other adverse events: allergy, irritation/burning of nasal, oral or oropharyngeal mucosa (e.g. erosions, ulcers, bleeding), long-term staining of mucous membranes or teeth, accidental ingestion. We planned to use GRADE to assess the certainty of the evidence for each outcome. MAIN RESULTS: We found no completed studies to include in this review. We identified three ongoing studies (including two RCTs), which aim to enrol nearly 700 participants. The interventions included in these trials are povidone iodine, nitric oxide and GLS-1200 oral spray (the constituent of this spray is unclear and may not be antimicrobial in nature).   AUTHORS' CONCLUSIONS: We identified no studies for inclusion in this review. This is not surprising given the relatively recent emergence of COVID-19 infection. It is promising that the question posed in this review is being addressed by two RCTs and a non-randomised study. We are concerned that only one of the ongoing studies specifically states that it will evaluate adverse events and it is not clear if this will include changes in the sense of smell or to the oral and nasal microbiota, and any consequences thereof. Very few interventions have large and dramatic effect sizes. If a positive treatment effect is demonstrated when studies are available for inclusion in this review, it may not be large. In these circumstances in particular, where those receiving the intervention are otherwise fit and well, it may be a challenge to weigh up the benefits against the harms if the latter are of uncertain frequency and severity.

Bwanga, O. (2020). "What nurses need to know about mobile radiography." Br J Nurs **29**(18): 1064-1067.

Nurses have a vital role in providing nursing care to patients requiring mobile radiography. Mobile radiography is requested when a patient's condition makes it impossible for them to be transported to the radiology department. All health professionals involved in mobile radiography, such as nurses, medical doctors and radiographers should be knowledgeable in this area. This is even more important in current practice, where nurse consultants and nurse advanced practitioners are assessing and referring patients for limited radiological examinations. However, there is little literature to equip nurses with knowledge about mobile radiography. The aim of this article is to raise awareness of this subject at a time when the number of patients requiring mobile radiography has increased globally, due to the outbreak of coronavirus. Critically ill patients with COVID-19 require portable chest X-rays to diagnose complications of the disease, such as pneumonia.

Byrne, J. D., et al. (2020). "Injection Molded Autoclavable, Scalable, Conformable (iMASC) system for aerosol-based protection: a prospective single-arm feasibility study." BMJ Open **10**(7): e039120.

OBJECTIVE: To develop and test a new reusable, sterilisable N95 filtering facepiece respirator (FFR)-comparable face mask, known as the Injection Molded Autoclavable, Scalable, Conformable (iMASC) system, given the dire need for personal protective equipment within healthcare settings during the COVID-19 pandemic. DESIGN: Single-arm feasibility study. SETTING: Emergency department and outpatient oncology clinic. PARTICIPANTS: Healthcare workers who have previously undergone N95 fit testing. INTERVENTIONS: Fit testing of new iMASC system. PRIMARY AND SECONDARY OUTCOME MEASURES: Primary outcome is success of fit testing using an Occupational Safety and Health Administration (OSHA)-approved testing method, and secondary outcomes are user experience with fit, breathability and filter replacement. RESULTS: Twenty-four subjects were recruited to undergo fit testing, and the average age of subjects was 41 years (range of 21-65 years) with an average body mass index of 26.5 kg/m(2). The breakdown of participants by profession was 46% nurses (n=11), 21% attending physicians (n=5), 21% resident physicians (n=5) and 12% technicians (n=3). Of these participants, four did not perform the fit testing due to the inability to detect saccharin solution on premask placement sensitivity test, lack of time and inability to place mask over hair. All participants (n=20) who performed the fit test were successfully fitted for the iMASC system using an OSHA-approved testing method. User experience with the iMASC system, as evaluated using a Likert scale with a score of 1 indicating excellent and a score of 5 indicating very poor, demonstrated an average fit score of 1.75, breathability of 1.6, and ease of replacing the filter on the mask was scored on average as 2.05. CONCLUSIONS: The iMASC system was shown to successfully fit multiple different face sizes and shapes using an OSHA-approved testing method. These data support further certification testing needed for use in the healthcare setting.

Cabas, P., et al. (2021). "Community pharmacists' exposure to COVID-19." Res Social Adm Pharm **17**(1): 1882-1887.

INTRODUCTION: Since the beginning of the 2020 Sars-CoV-2 Italian outbreak, healthcare workers have been among the most exposed categories. There is little information about community pharmacists' on occupational exposure, symptoms development, and testing practices in the community pharmacist cohort. METHODS: Between April 30th and May 10th, a questionnaire was administered through social media to Italian community pharmacists. From 67000 pharmacists currently working in community pharmacies, 1632 answered the survey. RESULTS: The survey population reflected the general Italian community pharmacists population in terms of age, gender, and number of co-workers. Protective measures were adopted in up to 99.9% of pharmacies. 624 pharmacists (38.2%) developed at least one COVID-19 related symptom in the period between February 28th and May 10th. Also, 102 pharmacists (6.2%) were tested for COVID-19 and 15, the 15% of the tested population and 0.92% of the whole survey population, resulted positive on nasopharyngeal swab. However, while the number of symptomatic pharmacists decreased, a higher number of tests were performed, thus COVID-19 prevalence among community pharmacists could have been underestimated and is probably intermediate between other healthcare workers and the general population (0.31%). CONCLUSION: Community pharmacists have probably been one of the first categories to experience increased contact risk to SARS-CoV-2. COVID-19 prevalence among pharmacists could have been underestimated. In addition, the rates of protection measures adoption might have helped to reduce the spread of COVID-19 among co-workers and the community.

Cabello, F., et al. (2020). "Consensus on Recommendations for Safe Sexual Activity during the COVID-19 Coronavirus Pandemic." J Clin Med **9**(7).

Sexual activity offers numerous advantages for physical and mental health but maintains inherent risks in a pandemic situation, such as the current one caused by SARS-CoV-2. A group of experts from the Spanish Association of Sexuality and Mental Health (AESexSAME) has reached a consensus on recommendations to maintain lower-risk sexual activity, depending on one's clinical and partner situations, based on the current knowledge of SARS-CoV-2. Different situations are included in the recommendations: a sexual partner passing quarantine without any symptoms, a sexual partner that has not passed quarantine, a sexual partner with some suspicious symptoms of COVID-19, a positive sexual partner with COVID-19, a pregnant sexual partner, a health professional partner in contact with COVID-19 patients, and people without a sexual partner. The main recommendations include returning to engaging in safe sex after quarantine is over (28 days based on the duration one can carry SARS-CoV-2, or 33 days for those who are >60 years old) and all parties are asymptomatic. In all other cases (for those under quarantine, those with some clinical symptoms, health professionals in contact with COVID-19 patients, and during pregnancy), abstaining from coital/oral/anal sex, substituting it with masturbatory or virtual sexual activity to provide maximum protection from the contagion, and increasing the benefits inherent to sexual activity are recommended. For persons without a partner, not initiating sexual activity with a sporadic partner is strongly recommended.

Cabrera-Tasayco, F. D. P., et al. (2020). "Biosafety Measures at the Dental Office After the Appearance of COVID-19: A Systematic Review." Disaster Med Public Health Prep: 1-5.

The purpose of this research was to determine biosecurity measures at the dental office after the appearance of coronavirus disease 2019 (COVID-19). A search was conducted in the main databases of the scientific literature using the words "COVID-19, coronavirus, SARS-Cov2, biosecurity, disinfection and dentistry." We analyzed biosecurity and disinfection standards at the dental office and dental health personnel to date, and their adaptation to the needs and way of working of each. As a result, according to the information collected the following procedure was identified: a telephone appointment must be made and a questionnaire should be given before dental care; at arrival to the appointment, the temperature of the patient should be taken and proper cleaning and disinfection of the waiting room should be maintained. Panoramic radiography and CBCT are the auxiliary methods of choice. Absolute isolation and atraumatic restorative therapy techniques are a good alternative to decrease fluid exposure. The removal of protective clothing and accessories must follow a specific order and washing hands before and after is essential. In conclusion, the efficient biosecurity for dentists and patients in all dental care processes before, during, and immediately after the appointment reduces the risk of COVID-19 infection and allows healthy dental care environments.

Cai, M., et al. (2020). "The Preventive and Control Recommendations on Patient With Cleft Lip and Palate During COVID-19 Pandemic in Shanghai." J Craniofac Surg.

As Corona Virus Disease 2019 (COVID-19) has been gradually controlled domestically, various industries began to resume production in an orderly way. Attention should be paid to the disease and population characteristics of patients with cleft lip with/without palate during diagnosis and treatment. This article summarized and provided prevention and control recommendations on management strategies during hospitalization and protective measures of patients and healthcare workers, hoping to minimize the spread of disease and create a relatively safe environment for medical work.

Cai, M., et al. (2021). "The Preventive and Control Recommendations on Patient With Cleft Lip and Palate During COVID-19 Pandemic in Shanghai." J Craniofac Surg **32**(2): e223-e226.

As Corona Virus Disease 2019 (COVID-19) has been gradually controlled domestically, various industries began to resume production in an orderly way. Attention should be paid to the disease and population characteristics of patients with cleft lip with/without palate during diagnosis and treatment. This article summarized and provided prevention and control recommendations on management strategies during hospitalization and protective measures of patients and healthcare workers, hoping to minimize the spread of disease and create a relatively safe environment for medical work.

Cai, S. J., et al. (2020). "[Analysis of bronchoscope-guided tracheal intubation in 12 cases with coronavirus disease 2019 under the personal protective equipment with positive pressure protective hood]." Zhonghua Jie He He Hu Xi Za Zhi **43**(4): 332-334.

Endotracheal intubation is an independent risk factor for respiratory infectious diseases. We conducted a retrospective study in 12 cases with COVID-19 who underwent endotracheal intubation at ICU of the Guangzhou eighth hospital from January 20 to February 10, 2020. The intubation procedure, anesthetic regimen, and complication were collected and analyzed. The 9 healthcare workers who involved in intubation received virus nucleic acid test and 14 days temperature monitoring. All 12 patients were successfully intubated under the guidance of bronchoscope, without any complications. Midazolam, Propofol and Morphine or fentanyl were used for sedation and analgesia, avoiding patients cough and agitated during the procedure. The 9 healthcare workers were protected under the Personal Protective Equipment(PPE) with positive pressure protective hood. The detection of oropharyngeal swab virus nucleic acid were negative in all 9 healthcare workers, none of them had fever or any respiratory symptoms. The PPE with positive pressure protective hood should be needed to perform bronchoscope-guided endotracheal intubation in patients with COVID-19, it could strengthen to protect healthcare workers from virus exposure.

Callahan, C., et al. (2020). "Nasal-Swab Testing Misses Patients with Low SARS-CoV-2 Viral Loads." medRxiv.

The urgent need for large-scale diagnostic testing for SARS-CoV-2 has prompted pursuit of sample-collection methods of sufficient sensitivity to replace sampling of the nasopharynx (NP). Among these alternatives is collection of nasal-swab samples, which can be performed by the patient, avoiding the need for healthcare personnel and personal protective equipment. Previous studies have reached opposing conclusions regarding whether nasal sampling is concordant or discordant with NP. To resolve this disagreement, we compared nasal and NP specimens collected by healthcare workers in a cohort consisting of individuals clinically suspected of COVID-19 and outpatients known to be SARS-CoV-2 RT-PCR positive undergoing follow-up. We investigated three different transport conditions, including traditional viral transport media (VTM) and dry swabs, for each of two different nasal-swab collection protocols on a total of 308 study participants, and compared categorical results and Ct values to those from standard NP swabs collected at the same time from the same patients. All testing was performed by RT-PCR on the Abbott SARS-CoV-2 RealTime EUA (limit of detection [LoD], 100 copies viral genomic RNA/mL transport medium). We found high concordance (Cohen's kappa >0.8) only for patients with viral loads above 1,000 copies/mL. Those with viral loads below 1,000 copies/mL, the majority in our cohort, exhibited low concordance (Cohen's kappa = 0.49); most of these would have been missed by nasal testing alone. Previous reports of high concordance may have resulted from use of assays with higher LoD (≥1,000 copies/mL). These findings counsel caution in use of nasal testing in healthcare settings and contact-tracing efforts, as opposed to screening of asymptomatic, low-prevalence, low-risk populations. Nasal testing is an adjunct, not a replacement, for NP.

Calò, F., et al. (2020). "Burden, risk assessment, surveillance and management of SARS-CoV-2 infection in health workers: a scoping review." Infect Dis Poverty **9**(1): 139.

BACKGROUND: Health workers (HWs) are at increased risk for severe acute respiratory syndrome-coronavirus-2 (SARS-CoV-2) infection and a possible source of nosocomial transmission clusters. Despite the increased risk, the best surveillance strategy and management of exposed HWs are not yet well known. The aim of this review was to summarize and critically analyze the existing evidence related to this topic in order to support public health strategies aimed at protecting HWs in the hospital setting. MAIN TEXT: A comprehensive computerized literature research from 1 January 2020 up to 22 May 2020 was made to identify studies analyzing the burden of infection, risk assessment, surveillance and management of HWs exposed to SARS-CoV-2. Among 1623 citation identified using MEDLINE, Embase, Google Scholar and manual search, we included 43 studies, 14 webpages and 5 ongoing trials. Health workers have a high risk of acquiring infection while caring for coronavirus disease 2019 (COVID-19) patients. In particular, some types exposures and their duration, as well as the inadequate or non-use of personal protective equipment (PPE) are associated with increased infection risk. Strict infection prevention and control procedures (IPC), adequate training programs on the appropriate use of PPE and close monitoring of HWs with symptom surveillance and testing are essential to significantly reduce the risk. At the moment there is not enough evidence to provide precise indications regarding pre-exposure prophylaxis (PrEP) and post-exposure prophylaxis (PEP). CONCLUSIONS: During the spread of COVID-19 outbreak, numerous published papers investigated the epidemiology, risk assessment and prevention and control of SARS-CoV-2. However, more high-quality studies are needed to provide valid recommendations for better management and for the clinical and microbiological surveillance of healthcare personnel.

Cameli, M., et al. (2020). "Safe performance of echocardiography during the COVID-19 pandemic: a practical guide." Rev Cardiovasc Med **21**(2): 217-223.

Coronavirus disease-2019 (COVID-19) outbreak has become a worldwide healthcare emergency, with continuously growing number of infected subjects. Considering the easy virus spread through respiratory droplets produced with cough, sneezes or spit or through close contact with infected people or surfaces, healthcare workers are further exposed to COVID-19. Particularly, echocardiography remains an essential diagnostic service which, due to the close contact with patients during the exam, provides echocardiographers high-risk of contagion. Therefore, the common modalities of performing echocardiography should be improved in this scenario, avoiding performing unnecessary exams, using the appropriate personal protective equipment depending on patients' status and location, optimizing time-effectiveness of the echocardiographic study and accurately sanitizing the environment and devices after each exam. This paper aims to provide a simple guide for the clinicians to balance between providing the best care to each patient and protecting themselves and other patients from the spread of the virus. It also proposes the use of the mnemonic PREVENT to resume the crucial indications to be followed for the execution of appropriate echocardiographic examination during the COVID-19 pandemic.

Campbell, K. H., et al. (2020). "Consolidation of obstetric services in a public health emergency." Semin Perinatol **44**(7): 151281.

Though much of routine healthcare pauses in a public health emergency, childbirth continues uninterrupted. Crises like COVID-19 put incredible strains on healthcare systems and require strategic planning, flexible adaptability, clear communication, and judicious resource allocation. Experiences from obstetric units affected by COVID-19 highlight the importance of developing new teams and workflows to ensure patient and healthcare worker safety. Additionally, adapting a strategy that combines units and staff from different areas and hospitals can allow for synergistic opportunities to provision care appropriately to manage a structure and workforce at maximum capacity.

Campbell, R. G. (2020). "SARS-CoV-2 and the nose: Risks and implications for primary care." Aust J Gen Pract **49**(11): 728-732.

BACKGROUND: General practitioners (GPs) have some of the highest rates of mortality from COVID-19 among healthcare workers. SARS-CoV-2 has unique properties that place GPs at particular risk. OBJECTIVE: The aim of this article is to discuss the nose-related features of SARS-CoV-2 that place GPs at risk, and to make recommendations pertinent to the safety and protection of primary healthcare physicians. DISCUSSION: The highest viral load of SARS-CoV-2 is in the nose and nasopharynx. It is often highest early in the illness, before the development of symptoms. Further, SARS-CoV-2 replicates and continues to shed in the nasopharynx long after the virus is no longer detectable in the lower respiratory tract. This places any physician performing examinations on, or procedures involving, the upper respiratory tract at risk for contracting COVID-19. New-onset hyposmia and dysgeusia are indicators for COVID-19 and should be included in screening protocols.

Campos, J., et al. (2021). "Symptoms related to mental disorder in healthcare workers during the COVID-19 pandemic in Brazil." Int Arch Occup Environ Health: 1-10.

BACKGROUND: Studies of previous pandemics indicate that healthcare workers have a high risk of developing symptoms related to mental health, especially depression, anxiety, and stress. OBJECTIVE: To identify mental disorder symptoms among Brazilian healthcare workers during the Sars-Cov-2 pandemic and compare findings in different work categories. METHODS: This was an online cross-sectional study. Information related to the pandemic and mental disorder symptoms was collected. The Depression, Anxiety, and Stress Scale and the Impact of Event Scale-revised were used. Associations were estimated by the chi-square test. The mean scores were compared among work categories with ANOVA (α = 5%) and the prevalence of symptoms was estimated. RESULTS: 1,609 healthcare workers participated in the survey [mean age: 36.9 (SD = 11.6) years, women = 83.6%]. There was no association between work category and changes in mental health during the pandemic (p = 0.288) or prevalence of unsafe feeling (p = 0.218). A significant relationship was observed between maintaining work activities during the pandemic and work category (p < 0.001). Physicians had the lowest out-of-work prevalence (9.5%) while dentists had the highest (32.3%). Physicians and nurses showed the highest prevalence of in-person work routine. Psychologists presented the highest prevalence of remote work (64.0%) while dentists had the lowest (20.2%). A high prevalence of depression (D), anxiety (A), and stress (S) symptoms was observed in all professional categories (D: 57.2, 95% CI 48.3-66.1%; A: 46.20%, 95% CI = 37.2-55.2%; S: 55.80%, 95% CI = 46.8-64.8%), with physicians (D = 38.4%, A = 25.80%, S = 37.90%), psychologists (D = 50.2%, A = 39.0%, S = 43.1%), and nurses (D = 50.0%, A = 40.9%, S = 49.0%) having significantly lower scores. Psychologists had the lowest pandemic-related psychological impact (42.70%, 95% CI 36.8-48.6%). CONCLUSION: Extreme changes in the work routine of dentists and psychologists and an overall high prevalence of mental symptoms due to the pandemic were found. Researchers should focus on gathering information that can identify workers at increased risk of mental illness to guide discussions and develop actions to minimize the harm of the pandemic. In addition, we suggest that healthcare and support systems urgently adopt mental health care measures with specialized professionals to protect the psychological well-being of the healthcare community.

Canaday, D. H., et al. (2021). "Reduced BNT162b2 mRNA vaccine response in SARS-CoV-2-naive nursing home residents." medRxiv.

The SARS-CoV-2 pandemic impact on nursing home (NH) residents prompted their prioritization for early vaccination. To fill the data gap for vaccine immunogenicity in NH residents, we examined antibody levels after BNT162b2 mRNA vaccine to spike, receptor binding domain (RBD) and for virus neutralization in 149 NH residents and 111 health care worker controls. SARS-CoV-2-naive NH residents mount antibody responses with nearly 4-fold lower median neutralization titers and half the anti-spike level compared to SARS-CoV-2-naive healthcare workers. By contrast, SARS-CoV-2-recovered vaccinated NH residents had neutralization, anti-spike and anti-RBD titers similar to SARS-CoV-2-recovered vaccinated healthcare workers. NH residents' blunted antibody responses have important implications regarding the quality and durability of protection afforded by neoantigen vaccines. We urgently need better longitudinal evidence on vaccine effectiveness specific to NH resident populations to inform best practices for NH infection control measures, outbreak prevention and potential indication for a vaccine boost.

Candel, B. G. J., et al. (2021). "Telemedicine in the emergency department to decrease personal protective equipment use: a before-and-after study." Emerg Med J **38**(3): 224-228.

BACKGROUND: Personal protective equipment (PPE) used by healthcare workers was scarce during the COVID-19 pandemic. The aim of this study was to assess whether telemedicine (using iPads) reduced PPE use in emergency department (ED) patients who were treated in contact isolation, and whether telemedicine had a positive effect on patient anxiety and satisfaction. METHODS: We conducted a prospective single centre before-and-after study including ED patients ≥18 years who were treated in contact isolation. PPE use, the Hospital Anxiety Scale and the 15-item Picker Patient Experience Questionnaire were compared between the control period (8 April to 14 April 2020) and intervention period (15 April to 24 April 2020). RESULTS: We included 25 patients in each period. PPE use per patient was higher for physicians in the control period (mean 1.7; 95% CI 1.5 to 1.9) compared with the intervention period (mean 1.2; 95% CI 1.0 to 1.3, p<0.01). Total PPE use per patient contact for ED physicians decreased from 42 out of 42 patient contacts in the control period, to 29 out of 66 patient contacts in the intervention period (difference 54.3%; 95% CI 50.1% to 58.6%, p<0.01). Reported anxiety and satisfaction were not significantly different. CONCLUSION: PPE use by physicians can successfully be reduced by using telemedicine in the ED without increasing anxiety or dissatisfaction. This study was a first step to gain experience with telemedicine in the ED which has the potential to reduce PPE use in future pandemics or other patients with an indication for contact isolation.

Canova, V., et al. (2020). "Transmission risk of SARS-CoV-2 to healthcare workers -observational results of a primary care hospital contact tracing." Swiss Med Wkly **150**: w20257.

BACKGROUND: The coronavirus disease (COVID)-19 epidemic is evolving rapidly. Healthcare workers are at increased risk for infection, and specific requirements for their protection are advisable to ensure the functioning of the basic healthcare system, including the availability of general practitioners (GPs). Understanding the transmission risk is particularly important for guiding evidence-based protective measures in the primary healthcare setting. METHODS: Healthcare worker contacts of an initially undiagnosed COVID-19 case, who were without personal protective equipment, in particular not wearing facemasks, were screened with nasopharyngeal swabs and polymerase chain reaction tests for severe acute respiratory syndrome coronavirus 2 (SARS-CoV-2), irrespective of respiratory symptoms or fever seven days after initial contact. The details of exposure to the index case were obtained during routine contact investigation after unintentional pathogen exposure. RESULTS: Twenty-one healthcare workers reported contacts with the index case. Three healthcare workers reported respiratory symptoms (cough) or low-grade fever within 4 days. None of them tested positive for SARS-CoV-2 at the time of symptom onset. All 21 healthcare workers tested SARS-CoV-2 negative 7 days after initial index case contact, including the three healthcare workers with previous symptoms. Ten of the 21 healthcare workers reported a cumulative exposure time of &gt;15 minutes. Longer cumulative contact times were associated with more individual contacts, reduced contact time per contact and activities with physical patient contact. The closest relative of the index patient tested SARS-CoV-2 positive 2 days after the index case presented at the hospital emergency department. CONCLUSION: We found a low risk of SARS-CoV-2 transmission in a primary care setting. These findings are compatible with previous reports of the highest transmission probability in household settings with prolonged close contacts. The current protective measures for healthcare workers, including strict adherence to basic standard hygiene and facemasks, offer considerable protection during short periods of contact with symptomatic COVID-19 cases by diminishing the risk of direct and indirect transmission.

Caram, C. D. S., et al. (2021). "Moral suffering in health professionals: portrait of the work environment in times of COVID-19." Rev Bras Enferm **74Suppl 1**(Suppl 1): e20200653.

OBJECTIVE: To analyze potential triggers of moral suffering experiences of health professionals, reported in the media, during the COVID-19 pandemic and to propose a theoretical construct of analysis. METHODS: Study with qualitative approach whose data source were 50 reports published online, collected passively and actively, submitted to Content Analysis with the help of ATLAS.ti software. RESULTS: The potential moral problems that trigger moral suffering are related to the threat to moral integrity, infrastructure/logistics and teamwork problems, and emotional aspects, revealing their articulation with damage to the foundations of a healthy work environment, which generated the proposal of a construct. FINAL CONSIDERATIONS: The articulation between the experiences of moral suffering and the commitment of the fundamentals of a healthy work environment has brought important contributions to the adoption of strategies to protect and stimulate moral deliberation by professionals in favor of practice and society.

Carnino, J. M., et al. (2020). "Pretreated household materials carry similar filtration protection against pathogens when compared with surgical masks." Am J Infect Control **48**(8): 883-889.

OBJECTIVE: The past 4 months, the emergence and spread of novel 2019 SARS-Cov-2 (COVID-19) has led to a global pandemic which is rapidly depleting supplies of personal protective equipment worldwide. There are currently over 1.6 million confirmed cases of COVID-19 worldwide which has resulted in more the 100,000 deaths. As these numbers grow daily, hospitals are being forced to reuse surgical masks in hopes of conserving their dwindling supply. Since COVID-19 will most likely have effects that last for many months, our nationwide shortage of masks poses a long term issue that must be addressed immediately. METHODS: Based on a previous study by Quan et al., a salt-based soaking strategy has been reported to enhance the filtration ability of surgical masks. We propose a similar soaking process which uses materials widely available in anyone's household. We tested this method of pretreating a variety of materials with a salt-based solution by a droplet test using fluorescently stained nanoparticles similar in size to the COVID-19 virus. RESULTS: In this study, we found that paper towels and surgical masks pretreated with the salt-based solution showed a noticeable increase in filtration of nanoparticles similar in size to the COVID-19 virus. We also show that the TWEEN20 used by Quan et al. is not a critical component for the solution, and using salt alone in solution still provides a dramatically increased level of protection. CONCLUSIONS: We believe this method will allow for healthcare workers to create a disposable added layer of protection to their surgical masks, N95s, or homemade masks by using household available products. Adoption of this method may play an essential role in ensuring the safety of healthcare workers during the COVID-19 pandemic and any pandemics that may arise in the future.

Carracedo, D., et al. (2020). "[Robotic and laparosocpic urological surgery during COVID-19 pandemia.]." Arch Esp Urol **73**(5): 463-470.

OBJECTIVE: SARS-CoV-2 pandemic hashigh repercussion on urologic minimally invasive surgery (MIS). Controversy about safety of MIS procedures during COVID-19 pandemic has been published. Nowadays, our priority should be create agreement in order to restart and organize MIS with safety conditions for patients and healthcare workers. METHODS: Pubmed and web search was conducted with following terms: "SARS-CoV-2", "COVID19", "COVID19 Urology", COVID19 Surgery", "COVID19 transmission", "SARS-CoV-2 transmission", "COVID19 nd minimally invasive surgery", "SARS-CoV-2 and CO 2insuflation". A narrative review of available literature and scientific evidence summary was done. A modify nominal group technique was used to achieve an expert consensus. First draft was circulated amongst authors. Definitive document was approved in May 26th. RESULTS: Non evidence supports higher risk of SARSCoV-2 healthcare workers infection with MIS compared to open surgery. MIS is associated with shorter hospital stay than open surgery. Modify MIS indications to open surgery, with no scientific evidence, could spend valuable resources in detriment to COVID-19 patients. MIS indications should be prioritized attending to available resources and pandemic intensity. SARS-CoV-2screening 72 hours prior to surgery by clinical and epidemiological questionnaire and nasopharyngeal PCRis recommended, in order to prevent nosocomial transmission, professional infections and to minimize postoperative complications. Intraoperative steps should be established to reduce professional exposure to surgical aerosols, including: surgical room reorganization, adequate personal protective equipment, surgical technique optimization and management of CO2 and surgical smoke. CONCLUSIONS: In COVID-19 pandemic de-escalation, MIS carried out with optimal safety measurements, could contribute to reduce hospital resources utilization. With current evidence, MIS should not be limited or reconverted to open surgery during COVID-19 pandemic.

Carretta, G., et al. (2020). "COVID-19 challenge: proactive management of a Tertiary University Hospital in Veneto Region, Italy." Pathog Glob Health **114**(6): 309-317.

BACKGROUND: The aim of this study is to describe the successful emergency plan implemented by Padova University Hospital (AOUP) during the COVID-19 pandemic. METHODS: The emergency plan included early implementation of procedures aimed at meeting the increasing demand for testing and care while ensuring safe and timely care of all patients and guaranteeing the safety of healthcare workers. RESULTS: From 21 February to 1 May 2020, there were 3,862 confirmed cases of SARS-CoV-2 infection in the Province of Padua. A total of 485 patients were hospitalized in AOUP, of which 91 were admitted to the ICU; 12 .6% of admitted patients died. The average bed occupancy rate in the ICU was 61.1% (IQR 43.6%:77.4%). Inpatient surgery and inpatient admissions were kept for 76% and 74%, respectively, compared to March 2019. A total of 123,077 swabs were performed, 19.3% of which (23,725 swabs) to screen AOUP workers. The screening of all staff showed that 137 of 7,649 (1.8%) hospital workers were positive. No healthcare worker died. DISCUSSION: AOUP strategy demonstrated effective management of the epidemic thanks to the timely implementation of emergency procedures, a well-coordinated effort shared by all hospital Departments, and their continuous adjustment to the ongoing epidemic. Timely screening of all hospital workers proved to be particularly important to defend the hospital, avoiding epidemic clusters due to unknown positive cases.

Carter, C., et al. (2021). "SARS-CoV-2 diagnostics: Towards a more comprehensive approach to routine patient testing." J Immunol Methods **494**: 113044.

The SARS-CoV-2 pandemic has provided the stimulus for the rapid development of a variety of diagnostic testing methods. Initially these were deployed as screening tools to evidence spread of the virus within populations. The recent availability of vaccines against the virus and the need to better understand the parameters of post-infection protective immunity requires development of methods, suitable for use in the routine diagnostic laboratory, capable of characterising the viral immune response in greater detail. Such methods need to consider both cellular and humoral immunity. Toward this aim we have investigated use of a commercial multiplex assay (COVID Plus Assay, One Lambda), providing assessment of the SARS-CoV-2 response at structural level, and developed an in-house cell stimulation assay using commercially available viral peptides (Miltenyi). This paper reports our experience in use of these methods in extended investigation of a cohort of healthcare workers with prior screening results indicative of viral infection. The antibody response generated is shown to be both qualitatively and quantitatively different in different individuals. Similarly a recall response to SARS-CoV-2 antigen involving the T cell compartment can be readily demonstrated in recovered individuals but is of variable magnitude.

Carvalho, E. A. and M. V. B. Oliveira (2020). "Safety model for chest drainage in pandemic by COVID-19." Rev Col Bras Cir **47**: e20202568.

Over one million cases of the SARS-CoV-2 virus have been confirmed worldwide, with the death toll exceeding 50,000 people. An important issue to be addressed concerns the exposure of health professionals to this new virus. The first reports from Wuhan province, China, described infection rates of up to 29% among healthcare professionals before the use of personal protective equipment (PPE) was fully regulated. There are several protocols on the correct use of PPE during aerosol-generating procedures. However, there is no specific guidance on how to proceed in cases of need for chest tubes in patients with positive COVID-19 active air leak. The objective of this work is to assist surgeons of the most diverse specialties during the chest drainage of a patient with COVID-19 and to avoid a risk of contamination to the professional and the environment.

Cattelan, A. M., et al. (2020). "An Integrated Strategy for the Prevention of SARS-CoV-2 Infection in Healthcare Workers: A Prospective Observational Study." Int J Environ Res Public Health **17**(16).

BACKGROUND: Since the beginning of SARS-CoV-2 outbreak, a large number of infections have been reported among healthcare workers (HCWs). The aim of this study was to investigate the occurrence of SARS-CoV-2 infection among HCWs involved in the first management of infected patients and to describe the measures adopted to prevent the transmission in the hospital. METHODS: This prospective observational study was conducted between February 21 and April 16, 2020, in the Padua University Hospital (north-east Italy). The infection control policy adopted consisted of the following: the creation of the "Advanced Triage" area for the evaluation of SARS-CoV-2 cases, and the implementation of an integrated infection control surveillance system directed to all the healthcare personnel involved in the Advance Triage area. HCWs were regularly tested with nasopharyngeal swabs for SARS-CoV-2; body temperature and suggestive symptoms were evaluated at each duty. Demographic and clinical data of both patients and HCWs were collected and analyzed; HCWs' personal protective equipment (PPE) consumption was also recorded. The efficiency of the control strategy among HCWs was evaluated identifying symptomatic infection (primary endpoint) and asymptomatic infection (secondary endpoint) with confirmed detection of SARS-CoV-2. RESULTS: 7595 patients were evaluated in the Advanced Triage area: 5.2% resulted positive and 72.4% was symptomatic. The HCW team was composed of 60 members. A total of 361 nasopharyngeal swabs were performed on HCWs. All the swabs resulted negative and none of the HCWs reached the primary or the secondary endpoint. CONCLUSIONS: An integrated hospital infection control strategy, consisting of dedicated areas for infected patients, strict measures for PPE use and mass surveillance, is successful to prevent infection among HCWs.

Catton, H. (2020). "Nursing and health policy perspectives 2020 - a year to remember or one to forget?" Int Nurs Rev **67**(4): 450-452.

2020 has certainly been a year of challenges for nurses and healthcare workers around the world with the relentless spread of COVID-19. These challenges are many, including countries and health systems not being prepared for a pandemic, shortages of personal protective equipment and acute shortages of nurses, poor communication from governments and the lack of accurate data regarding COVID-19 morbidity and mortality among nurses and health workers. Nurses are suffering from psychological distress and are exhausted and burntout as the pandemic's second wave moves around the world. Many have been subjected to violence and aggression from people in their communities. Against this backdrop, nurses' contributions have been vital in saving lives and the profession has learnt many powerful lessons that will resonate in nursing practice for the future. But governments must do more, including ensuring nurses receive vaccinations early to protect them when a successful vaccine becomes available.

Celik, H. K., et al. (2020). "Design and Additive Manufacturing of Medical Face Shield for Healthcare Workers Battling Coronavirus (COVID-19)." Int J Bioprint **6**(4): 286.

During the coronavirus disease-19 pandemic, the demand for specific medical equipment such as personal protective equipment has rapidly exceeded the available supply around the world. Specifically, simple medical equipment such as medical gloves, aprons, goggles, surgery masks, and medical face shields have become highly in demand in the health-care sector in the face of this rapidly developing pandemic. This difficult period strengthens the social solidarity to an extent parallel to the escalation of this pandemic. Education and government institutions, commercial and noncommercial organizations and individual homemakers have produced specific medical equipment by means of additive manufacturing (AM) technology, which is the fastest way to create a product, providing their support for urgent demands within the health-care services. Medical face shields have become a popular item to produce, and many design variations and prototypes have been forthcoming. Although AM technology can be used to produce several types of noncommercial equipment, this rapid manufacturing approach is limited by its longer production time as compared to conventional serial/mass production and the high demand. However, most of the individual designer/maker-based face shields are designed with little appreciation of clinical needs and nonergonomic. They also lack of professional product design and are not designed according to AM (Design for AM [DfAM]) principles. Consequently, the production time of up to 4 - 5 h for some products of these designs is needed. Therefore, a lighter, more ergonomic, single frame medical face shield without extra components to assemble would be useful, especially for individual designers/makers and noncommercial producers to increase productivity in a shorter timeframe. In this study, a medical face shield that is competitively lighter, relatively more ergonomic, easy to use, and can be assembled without extra components (such as elastic bands, softening materials, and clips) was designed. The face shield was produced by AM with a relatively shorter production time. Subsequently, finite element analysis-based structural design verification was performed, and a three-dimensional (3D) prototype was produced by an original equipment manufacturer 3D printer (Fused Deposition Modeling). This study demonstrated that an original face shield design with <10 g material usage per single frame was produced in under 45 min of fabrication time. This research also provides a useful product DfAM of simple medical equipment such as face shields through advanced engineering design, simulation, and AM applications as an essential approach to battling coronavirus-like viral pandemics.

Ceraulo, S., et al. (2020). "Reduce the spread of COVID-19 within the dental practice: the era of single use." Minerva Stomatol.

The year 2020 will be remembered around the world for the coronavirus pandemic. The better known coronavirus disease COVID-19 is an infectious disease caused by a type of coronavirus that is transmitted between individuals through the droplets produced by infected people when they exhale, cough or sneeze, or by touching a contaminated surface and then touching their eyes the nose or mouth. The pandemic has activated all those measures that provide for a state of emergency with consequent remodeling of health facilities in cases of urgency that cannot be deferred. METHODS: In this work, a path within a public or private medical facility was highlighted, using individual protective devices for the patient, establishing behaviors that can reduce the spread of the virus. RESULTS: The COVID-19 path has allowed the health personnel and the staff of the study to be able to work in peace as there is the perception of the total containment of the spread of the virus. CONCLUSIONS: Coronavirus disease (COVID-19) is an infectious disease caused by a coronavirus type transmitted through droplets produced by infected people when they cough, sneeze or exhale. Our proposal in dictating the sequence and types of protective devices to be worn by patients minimizes the contagion between patients in a professional healthcare office and is applicable for any type of infectious emergency.

Chakladar, A., et al. (2021). "Microbial contamination of powered air purifying respirators (PAPR) used by healthcare staff during the COVID-19 pandemic: an in situ microbiological study." Am J Infect Control.

BACKGROUND: Powered air purifying respirators (PAPR) are an option for healthcare workers requiring respiratory protection during the current COVID-19 pandemic; they are shared between multiple people. PAPR hoods are intended for multiple uses by a single user and may pose an infection risk between wearers. METHODS: Internal components of PAPR hoods and corrugated air supply hoses were swabbed for evidence of bacterial, fungal, common respiratory viruses and severe acute respiratory syndrome-coronavirus-2 (SARS-CoV-2) contamination. RESULTS: Twenty-five PAPR hoods were swabbed; 10 (40%) returned positive results. Bacterial growth was detected on six PAPR; five of the PAPR tested positive for fungal growth; all tested negative for SARS-CoV-2 and common respiratory viruses. CONCLUSIONS: Bacteria and fungi can remain on internal components of PAPR hoods and air supply hoses despite following recommended disinfection procedures. PAPR hoods have the potential to act as fomites, cross-infecting wearers, and patients. Current guidelines for disinfecting PAPR hoods may not be effective for use in high risk healthcare environments.

Chakraborty, U., et al. (2020). "COVID-19-associated acute transverse myelitis: a rare entity." BMJ Case Rep **13**(8).

SARS-CoV-2 has wreaked havoc globally and has claimed innumerable lives all over the world. Apart from the characteristic respiratory illness, this disease has been associated with florid extrapulmonary manifestations and complications. A 59-year-old female healthcare worker presented with features of acute-onset non-compressive myelopathy with a sensory level at T10 segment along with high-grade fever for 4 days. MRI of dorsal spine was suggestive of myelitis at T7 vertebral level. She was initiated on injectable steroids and did show some initial signs of recovery. A day later, she developed an acute-onset respiratory failure but could not be revived despite our best efforts. Her nasopharyngeal and oropharyngeal swab turned out to be positive for SARS-CoV-2 reverse transcriptase polymerase chain reaction (RT-PCR). We hereby report a case of acute transverse myelitis with COVID-19 as a probable aetiology.

Chaluvashetty, S. B., et al. (2021). "Interventional radiology and COVID-19: How to face the challenge?" Indian J Radiol Imaging **31**(Suppl 1): S38-s44.

With the sudden outbreak of Coronavirus disease-19 (COVID-19) in China, and its rapid spread across the continents over a short period of time, healthcare workers are posed with the challenge of managing these patients as well protecting themselves from getting infected. Since interventional radiology deals with both elective and emergency services, wherein close patient contact is a norm, there is a substantial risk of acquiring and transmitting infection. Given the circumstances, it is imperative to develop broadly applicable guidelines to utilize the available resources in an optimal fashion and limit transmission of disease. This brief review deals with infection control measures within the Interventional Radiology department or section and possible recommendations that can be adopted at the institutional level.

Chan, H. Y. (2020). "Hospitals' Liabilities in Times of Pandemic: Recalibrating the Legal Obligation to Provide Personal Protective Equipment to Healthcare Workers." Liverp Law Rev: 1-21.

The Covid-19 pandemic has precipitated the global race for essential personal protective equipment in delivering critical patient care. This has created a dearth of personal protective equipment availability in some countries, which posed particular harm to frontline healthcare workers' health and safety, with undesirable consequences to public health. Substantial discussions have been devoted to the imperative of providing adequate personal protective equipment to frontline healthcare workers. The specific legal obligations of hospitals towards healthcare workers in the pandemic context have so far escaped important scrutiny. This paper endeavours to examine this overlooked aspect in the light of legal actions brought by frontline healthcare workers against their employers arising from a shortage of personal protective equipment. By analysing the potential legal liabilities of hospitals, the paper sheds light on the interlinked attributes and factors in understanding hospitals' obligations towards healthcare workers and how such duty can be justifiably recalibrated in times of pandemic.

Chao, T. N., et al. (2020). "Airway management of angioedema patients during the COVID-19 pandemic." World J Otorhinolaryngol Head Neck Surg **6**(Suppl 1): S36-s39.

IMPORTANCE: The COVID-19 pandemic is characterized by high transmissibility from patients with prolonged minimally- or asymptomatic periods, with a particularly increased risk of spread during aerosol-generating procedures, including endotracheal intubation. OBSERVATIONS: All patients presenting with upper airway obstruction due to angioedema during this time should be carefully managed in a way that is safest for both patient and provider. CONCLUSIONS: For patients requiring emergent airway management during the COVID-19 pandemic, minimization of aerosols while taking the necessary precautions to protect healthcare workers should are critical principles for their management.

Charitos, I. A., et al. (2020). "Special features of SARS-CoV-2 in daily practice." World J Clin Cases **8**(18): 3920-3933.

The severe acute respiratory syndrome-coronavirus-2 (commonly known as SARS-CoV-2) is a novel coronavirus (designated as 2019-nCoV), which was isolated for the first time after the Chinese health authorities reported a cluster of pneumonia cases in Wuhan, China in December 2019. Optimal management of the Coronavirus Disease-2019 disease is evolving quickly and treatment guidelines, based on scientific evidence and experts' opinions with clinical experience, are constantly being updated. On January 30, 2020, the World Health Organization declared the SARS-CoV-2 outbreak as a "Public Health Emergency of International Concern". The total lack of immune protection brought about a severe spread of the contagion all over the world. For this reason, diagnostic tools, patient management and therapeutic approaches have been tested along the way, in the desperate race to break free from the widespread infection and its fatal respiratory complications. Current medical knowledge and research on severe and critical patients' management and experimental treatments are still evolving, but several protocols on minimizing risk of infection among the general population, patients and healthcare workers have been approved and diffused by International Health Authorities.

Chatterjee, P., et al. (2020). "Healthcare workers & SARS-CoV-2 infection in India: A case-control investigation in the time of COVID-19." Indian J Med Res **151**(5): 459-467.

BACKGROUND & OBJECTIVES: Healthcare workers (HCWs) are at an elevated risk of contracting COVID-19. While intense occupational exposure associated with aerosol-generating procedures underlines the necessity of using personal protective equipment (PPE) by HCWs, high-transmission efficiency of the causative agent [severe acute respiratory syndrome coronavirus 2 (SARS-CoV-2)] could also lead to infections beyond such settings. Hydroxychloroquine (HCQ), a repurposed antimalarial drug, was empirically recommended as prophylaxis by the National COVID-19 Task Force in India to cover such added risk. Against this background, the current investigation was carried out to identify the factors associated with SARS-CoV-2 infection among HCWs in the country. METHODS: A case-control design was adopted and participants were randomly drawn from the countrywide COVID-19 testing data portal maintained by the ICMR. The test results and contact details of HCWs, diagnosed as positive (cases) or negative (controls) for SARS-CoV-2 using real-time reverse transcription-polymerase chain reaction (qRT-PCR), were available from this database. A 20-item brief-questionnaire elicited information on place of work, procedures conducted and use of PPE. RESULTS: Compared to controls, cases were slightly older (34.7 vs. 33.5 yr) and had more males (58 vs. 50%). In multivariate analyses, HCWs performing endotracheal intubation had higher odds of being SARS-CoV-2 infected [adjusted odds ratio (AOR): 4.33, 95% confidence interval (CI): 1.16-16.07]. Consumption of four or more maintenance doses of HCQ was associated with a significant decline in the odds of getting infected (AOR: 0.44; 95% CI: 0.22-0.88); a dose-response relationship existed between frequency of exposure to HCQ and such reductions (χ([2]) for trend=48.88; P <0.001). In addition, the use of PPE was independently associated with the reduction in odds of getting infected with SARS-CoV-2. INTERPRETATIONS & CONCLUSIONS: Until results of clinical trials for HCQ prophylaxis become available, this study provides actionable information for policymakers to protect HCWs at the forefront of COVID-19 response. The public health message of sustained intake of HCQ prophylaxis as well as appropriate PPE use need to be considered in conjunction with risk homoeostasis operating at individual levels.

Chaturvedi, S., et al. (2020). "Design, usage and review of a cost effective and innovative face shield in a tertiary care teaching hospital during COVID-19 pandemic." J Orthop **21**: 331-336.

BACKGROUND: A major challenge of the coronavirus pandemic is personal protective equipment (PPE) shortage. The open source community mobilised solutions to combat this using 3D printing technology. One such solution was the face shield, which protects facial areas from droplet contamination when used by orthopaedic and other front line health care workers (HCWs). OBJECTIVES: To assess the efficacy of an in-house developed face shield based on feedback by HCWs and its usage in triaging zones and operation theatre in a tertiary care hospital. METHODS: A protective face shield was developed and distributed among the orthopaedic surgeons and front line HCWs involved in the ICU in our hospital and neighbouring facilities. Feedback was obtained using a questionnaire utilising a Likert scale. RESULTS: 227 face shields were distributed to the HCWs in our hospital (157) and neighbouring facilities (70). Design modifications were done as per the needs of the HCWs. 37 HCWs provided feedback giving the face shields an overall mean score of 7.92 out of 10. The poly vinyl chloride (PVC) film visors were better for airway management procedures as it can be tucked into PPE suit and visors with overhead projector (OHP) sheets were suitable for ICU and operative procedures. CONCLUSION: A locally developed face shield design by an inter disciplinary team in synchrony with HCWs is found to increase its acceptability and efficacy. Face shields can be made more effective in different triaging and treatment situations by varying the device setup.

Chau, S. W. H., et al. (2021). "History for some or lesson for all? A systematic review and meta-analysis on the immediate and long-term mental health impact of the 2002-2003 Severe Acute Respiratory Syndrome (SARS) outbreak." BMC Public Health **21**(1): 670.

BACKGROUND: The aims of this systematic review and meta-analysis are to examine the prevalence of adverse mental health outcomes, both short-term and long-term, among SARS patients, healthcare workers and the general public of SARS-affected regions, and to examine the protective and risk factors associated with these mental health outcomes. METHODS: We conducted a systematic search of the literature using databases such as Medline, Pubmed, Embase, PsycInfo, Web of Science Core Collection, CNKI, the National Central Library Online Catalog and dissertation databases to identify studies in the English or Chinese language published between January 2003 to May 2020 which reported psychological distress and mental health morbidities among SARS patients, healthcare workers, and the general public in regions with major SARS outbreaks. RESULTS: The literature search yielded 6984 titles. Screening resulted in 80 papers for the review, 35 of which were included in the meta-analysis. The prevalence of post-recovery probable or clinician-diagnosed anxiety disorder, depressive disorder, and post-traumatic stress disorder (PTSD) among SARS survivors were 19, 20 and 28%, respectively. The prevalence of these outcomes among studies conducted within and beyond 6 months post-discharge was not significantly different. Certain aspects of mental health-related quality of life measures among SARS survivors remained impaired beyond 6 months post-discharge. The prevalence of probable depressive disorder and PTSD among healthcare workers post-SARS were 12 and 11%, respectively. The general public had increased anxiety levels during SARS, but whether there was a clinically significant population-wide mental health impact remained inconclusive. Narrative synthesis revealed occupational exposure to SARS patients and perceived stigmatisation to be risk factors for adverse mental health outcomes among healthcare workers, although causality could not be determined due to the limitations of the studies. CONCLUSIONS: The chronicity of psychiatric morbidities among SARS survivors should alert us to the potential long-term mental health complications of covid-19 patients. Healthcare workers working in high-risk venues should be given adequate mental health support. Stigmatisation against patients and healthcare workers should be explored and addressed. The significant risk of bias and high degree of heterogeneity among included studies limited the certainty of the body of evidence of the review.

Chauhan, A., et al. (2021). "The interplay of circular economy with industry 4.0 enabled smart city drivers of healthcare waste disposal." J Clean Prod **279**: 123854.

Generation of healthcare waste from different patient care activities in hospitals, pathology labs and research centres has been a matter of great concern for environmental and social bodies across the world. This concern comes from its infectious and hazardous nature which brings life taking disease such as human immunodeficiency virus and Hepatitis-B. Moreover, with the outbreak of corona virus disease 2019 (COVID-19) pandemic across the world, healthcare waste has become even more infectious like never before and showing its potential for claiming lives if not disposed properly. Additionally, the COVID-19 has put up another challenge in terms of exponentially increasing demand for personal protective equipments for healthcare workers such as doctors, nurses, ward boys, and sanitation workers. In this paper, seven criteria related to smart healthcare waste disposal system infused by circular economy aspects to recover value from disposables are identified and analysed using a decision making trial and evaluation laboratory (DEMATEL) method. The criteria have been prioritized by its importance and net cause and effect relationship through a causal diagram. Two criteria, (i) digitally connected healthcare centres, waste disposal firms and pollution control board, and (ii) providing a pollution control board's feedback app to public and other stakeholders, feature as strong reasons for a smart healthcare waste disposal system. Conclusively, this study provides a causal relationship model among the intertwined drivers of industry 4.0 and circular economy for developing a smart healthcare waste disposal system enriched with the benefits of circular economy.

Chawla, G., et al. (2020). "Personal Protective Equipment: A Pandora's Box." Indian J Crit Care Med **24**(5): 371-372.

Countries all over the world writhing under the wrath of coronavirus face not only the challenge of protecting their population but the tougher challenge of protecting their healthcare workers (HCWs) dealing with the patient population. This pandemic has put unprecedented pressure on global healthcare systems, with personal protective equipment (PPE) being the most notable one. Apart from the challenge of procurement of PPE, the major challenge is rationalizing the use of PPE in this war against corona virus disease 19 (COVID19). Using PPE comes with its own set of problems such as extreme exhaustion, rashes, inability to consume food, or use washroom which can result in clouding of judgment and breach of infection barrier. Making PPE user-friendly and limiting the interaction of HCW with COVID19 patients coupled with the use of robotics, telemedicine, and other innovations is the need of the hour. HOW TO CITE THIS ARTICLE: Chawla G, Abrol N, Kakkar K. Personal Protective Equipment: A Pandora's Box. Indian J Crit Care Med 2020;24(5):371-372.

Cheek, C., et al. (2021). "Decommissioning and recommissioning a regional hospital in response to a COVID-19 outbreak." Rural Remote Health **21**(2): 6256.

CONTEXT: The COVID-19 outbreak at the North West Regional Hospital (NWRH) site in Tasmania, Australia in April 2020 was both rapid and tragic. Within 10 days of identification of the first healthcare worker infection, both hospitals had closed, and all patients were discharged or decanted to other facilities within the state. The entire hospital staff (approximately 1300 people) and their households (approximately 3000-4000 people) were furloughed for 14 days to halt the spread of infection. During the furlough period, a decommissioning, terminal clean and recommissioning process was undertaken alongside recovery and reorientation of the workforce to personal protective equipment. Within 4 days of closure, an Australian Defence Force and Australian Medical Assistance Team team opened the prioritised emergency department to provide emergency care for the local community, supported by modified diagnostic services. The decommissioning and cleaning rolled on over the ensuing month, in a predetermined priority order. As staff returned from quarantine, they recommissioned their clinical areas. The final ward, a modified medical isolation wing, reopened on day 29. ISSUE: Disaster management activities may be grouped under four main headings: prevention, preparedness, response and recovery. There are many opportunities for improvement and learning, and this article focuses on the local response and recovery, describing the process undertaken from the perspective of a small management group. Authors CC, HE, TB and MW were on the ground during the decommissioning process, then managed aspects of the cleaning and recommissioning remotely from furlough. Authors TA and TC provided specialist IPC support and developed education remotely. LESSONS LEARNED: Almost 2 months on, no new COVID-19 infections had been reported. The aim of this article is to provide a foundation for site-specific adaptation to include in pandemic escalation plans in other regional and rural settings.

Chen, C. H., et al. (2021). "Experience of 2003 SARS has a negative psychological impact on healthcare workers in the COVID-19 pandemic: a cross-sectional study." Sao Paulo Med J **139**(1): 65-71.

BACKGROUND: The COVID-19 pandemic has instilled fear and stress among healthcare workers. OBJECTIVES: The aim of this study was to assess work stress and associated factors among healthcare workers during the COVID-19 outbreak and to evaluate whether prior experience of treating severe acute respiratory syndrome (SARS) had a positive or negative influence on healthcare workers' stress levels during the COVID-19 pandemic. DESIGN AND SETTING: Cross-sectional survey in a tertiary hospital in Kaohsiung City, in southern Taiwan. METHODS: The survey was conducted using an online self-administered questionnaire to measure the stress levels among healthcare workers from March 20 to April 20, 2020. The stress scales were divided into four subscales: worry of social isolation; discomfort caused by the protective equipment; difficulties and anxiety regarding infection control; and workload of caring for patients. RESULTS: The total stress scores were significantly higher among healthcare workers who were aged 41 or above, female, married, parents and nurses. Those with experience of treating SARS reported having significantly higher stress scores on the subscale measuring the discomfort caused by protective equipment and the workload of caring for patients. During the COVID-19 outbreak, frontline healthcare workers with experience of treating SARS indicated having higher stress levels regarding the workload of caring for patients than did non-frontline healthcare workers with no experience of treating SARS. CONCLUSIONS: Work experience from dealing with the 2003 SARS virus may have had a negative psychological impact on healthcare workers amidst the COVID-19 outbreak.

Chen, C. H., et al. (2021). "Experience of 2003 SARS has a negative psychological impact on healthcare workers in the COVID-19 pandemic: a cross-sectional study." Sao Paulo Med J.

BACKGROUND: The COVID-19 pandemic has instilled fear and stress among healthcare workers. OBJECTIVES: The aim of this study was to assess work stress and associated factors among healthcare workers during the COVID-19 outbreak and to evaluate whether prior experience of treating severe acute respiratory syndrome (SARS) had a positive or negative influence on healthcare workers' stress levels during the COVID-19 pandemic. DESIGN AND SETTING: Cross-sectional survey in a tertiary hospital in Kaohsiung City, in southern Taiwan. METHODS: The survey was conducted using an online self-administered questionnaire to measure the stress levels among healthcare workers from March 20 to April 20, 2020. The stress scales were divided into four subscales: worry of social isolation; discomfort caused by the protective equipment; difficulties and anxiety regarding infection control; and workload of caring for patients. RESULTS: The total stress scores were significantly higher among healthcare workers who were aged 41 or above, female, married, parents and nurses. Those with experience of treating SARS reported having significantly higher stress scores on the subscale measuring the discomfort caused by protective equipment and the workload of caring for patients. During the COVID-19 outbreak, frontline healthcare workers with experience of treating SARS indicated having higher stress levels regarding the workload of caring for patients than did non-frontline healthcare workers with no experience of treating SARS. CONCLUSIONS: Work experience from dealing with the 2003 SARS virus may have had a negative psychological impact on healthcare workers amidst the COVID-19 outbreak.

Chen, M., et al. (2020). "Protecting healthcare workers from SARS-CoV-2 and other infections." Epidemiol Infect **148**: e217.

Coronavirus disease 2019 (COVID-19) has had a tremendous impact in China and abroad since its onset in December 2019 and poses a major threat to human health. Healthcare workers (HCWs) are at the forefront of the response to outbreaks. This study reviewed literature data and found that HCWs were at high risk of infection during the COVID-19 pandemic, especially at the early stage of the epidemic, and many factors greatly affected their occupational safety. Although SARS-CoV-2 transmission was controlled in China, the Chinese experience can help protect HCWs from COVID-19 and other respiratory diseases.

Chen, M. L., et al. (2020). "[Infection prevention and control of bedside blood purification treatment in patients with COVID-19]." Beijing Da Xue Xue Bao Yi Xue Ban **52**(3): 414-419.

OBJECTIVE: To explore the infection prevention and control strategy of bedside blood purification treatment in corona virus disease 2019 (COVID-19) isolation ward, and to evaluate the effect of infection prevention and control management measures. METHODS: We summarized and analyzed the clinical features, infection status, outcome and infection prevention and control measures of bedside blood purification treatment patients in COVID-19 isolation ward from February 8, 2020 to March 31, 2020, analyzed the COVID-19 cross-infection between the patients and medical staffs, and the blood-borne pathogens cross-infection situation between the patients, and analyzed the effect of bundle prevention and control measures in controlling the occurrence and spread of cross-infection. RESULTS: A total of 101 COVID-19 patients were hospitalized in this COVID-19 isolation ward, of whom 10 patients (9.90%) received bedside blood purification treatment and the blood purification treatment method was continuous hemodialysis filtration (CVVHDF), and the 10 patients received 79 times of blood purification treatment in total. The prevention and control management measures adopted included divisional isolation, patient behavior isolation and patient placement, operator personal protection and hand hygiene, dialysis waste fluid disposal, isolation room air purification, object surfaces, medical devices and medical fabrics dis-infection management. There were no occurrence and spread of COVID-19 in the medical healthcare workers and blood-borne pathogens cross-infection in the patients. And all the twice throat swabs (two sampling interval > 1 day) of the medical staffs in COVID-19 virus nucleic acid test were negative. The 2 suspected COVID-19 patients' throat swab virus nucleic acid test and the COVID-19 IgG, IgM were always both negative, the chest CT showed no viral pneumonia. CONCLUSION: Bedside blood purification treatment in the COVID-19 isolation ward, the occurrence and spread of healthcare associated infection can be effectively controlled through effective infection prevention and control management, including divisional isolation, patient behavior isolation and patient placement, operator personal protection and hand hygiene, dialysis waste fluid disposal, isolation room's air purification, object surfaces, medical devices and medical fabrics disinfection, which can provide experience for diagnosis, treatment and prevention and control of patients in the respiratory infectious disease ward.

Publisher: Abstract available from the publisher.

chi

Chen, Q., et al. (2020). "Precautionary Measures: Performing ERCP on a Patient With Juxtapapillary Duodenal Diverticula (JPDD)-Related Biliary Stone After COVID-19 Lockdown Restriction Lifted in Wuhan, China." Front Med (Lausanne) **7**: 564.

On April 8, 2020, after nearly 3 months of battling against the outbreak of COVID-19, Wuhan, where the pandemic began, began easing lockdown restrictions. However, given that asymptomatic carriers could continue to lead to transmission of COVID-19 during the very early stages, the endoscopists have taken precautions and conduct risk assessments to perform endoscopic intervention in this transition stage. Here, we have reported an urgent ERCP in a patient with acute pancreatitis secondary to JPDD-related biliary stone. Based on our experiences, the objective is to provide practical suggestions for the safe resumption of ERCP procedures in the setting of the COVID-19 pandemic with specific focus on patient risk assessment, personal protection equipment (PPE), and dress code modalities, all of which have been implemented in our hospital to reduce the risk of viral transmission.

Chen, W. C., et al. (2020). "Guidelines to Reduce Hospitalization Rates for Patients Receiving Curative-Intent Radiation Therapy During the COVID-19 Pandemic: Report From a Multicenter New York Area Institution." Adv Radiat Oncol **5**(4): 621-627.

As the coronavirus disease 2019 pandemic spreads around the globe, access to radiation therapy remains critical for patients with cancer. The priority for all radiation oncology departments is to protect the staff and to maintain operations in providing access to those patients requiring radiation therapy services. Patients with tumors of the aerodigestive tract and pelvis, among others, often experience toxicity during treatment, and there is a baseline risk that adverse effects may require hospital-based management. Routine care during weekly visits is important to guide patients through treatment and to mitigate against the need for hospitalization. Nevertheless, hospitalizations occur and there is a risk of nosocomial severe acute respiratory syndrome coronavirus-2 spread. During the coronavirus disease 2019 pandemic, typical resources used to help manage patients, such as dental services, interventional radiology, rehabilitation, and others are limited or not at all available. Recognizing the need to provide access to treatment and the anticipated toxicity of such treatment, we have developed and implemented guidelines for clinical care management with the hope of avoiding added risk to our patients. If successful, these concepts may be integrated into our care directives in nonpandemic times.

Chen, X., et al. (2020). "Perioperative Management of Patients Infected with the Novel Coronavirus: Recommendation from the Joint Task Force of the Chinese Society of Anesthesiology and the Chinese Association of Anesthesiologists." Anesthesiology **132**(6): 1307-1316.

The outbreak of the new Coronavirus disease, COVID-19, has been involved in 77,262 cases in China as well as in 27 other countries as of February 24, 2020. Because the virus is novel to human beings, and there is no vaccine yet available, every individual is susceptible and can become infected. Healthcare workers are at high risk, and unfortunately, more than 3,000 healthcare workers in China have been infected. Anesthesiologists are among healthcare workers who are at an even higher risk of becoming infected because of their close contact with infected patients and high potential of exposure to respiratory droplets or aerosol from their patients' airways. In order to provide healthcare workers with updated recommendations on the management of patients in the perioperative setting as well as for emergency airway management outside of the operating room, the two largest anesthesia societies, the Chinese Society of Anesthesiology (CSA) and the Chinese Association of Anesthesiologists (CAA) have formed a task force to produce the recommendations. The task force hopes to help healthcare workers, particularly anesthesiologists, optimize the care of their patients and protect patients, healthcare workers, and the public from becoming infected. The recommendations were created mainly based on the practice and experience of anesthesiologists who provide care to patients in China. Therefore, adoption of these recommendations outside of China must be done with caution, and the local environment, culture, uniqueness of the healthcare system, and patients' needs should be considered. The task force will continuously update the recommendations and incorporate new information in future versions.

Chen, X. C., et al. (2020). "Preventive and Control Measures for the Coronavirus Pandemic in Clinical Dentistry." Chin J Dent Res **23**(2): 99-104.

A severe public health crisis has been declared worldwide since coronavirus disease 2019 (COVID-19) was classified as a pandemic of acute respiratory infectious disease by the World Health Organisation (WHO). China has taken strict measures to curb the spread of the disease to save lives, and has managed to control the outbreak. COVID-19 is mainly transmitted through respiratory droplets and close physical contact, so it is challenging to prevent nosocomial infection and possible spread during dental treatment. Since the initial phase of the COVID-19 outbreak, a disease prevention and control strategy based on the new concept of population risk classification and rational use of personal protective equipment has been implemented by the Peking University Hospital of Stomatology. Nosocomial infection prevention and control concepts and measures relating to dental diagnosis and treatment are critically checked in the hospital. Our experiences in handling this situation are shared here and may have wide-ranging implications for infection prevention and control (IPC) for COVID-19 in dental practices worldwide.

Chen, Y., et al. (2020). "High SARS-CoV-2 antibody prevalence among healthcare workers exposed to COVID-19 patients." J Infect **81**(3): 420-426.

The seroprevalence of severe acute respiratory syndrome coronavirus 2 (SARS-CoV-2) was examined among 105 healthcare workers (HCWs) exposed to four patients who were laboratory confirmed with coronavirus disease 2019 (COVID-19), the disease caused by SARS-CoV-2 infection. These HCWs were immediately under quarantine for 14 days as soon as they were identified as close contacts. The nasopharyngeal swab samples were collected on the first and 14(th) day of the quarantine, while the serum samples were obtained on the 14(th) day of the quarantine. With the assay of enzyme immunoassay (EIA) and microneutralization assay, 17.14% (18/105) of HCWs were seropositive, while their swab samples were found to be SARS-CoV-2 RNA negative. Risk analysis revealed that wearing face mask could reduce the infection risk (odds ratio [OR], 0.127, 95% confidence interval [CI] 0.017, 0.968), while when exposed to COVID-19 patients, doctors might have higher risk of seroconversion (OR, 346.837, 95% CI 8.924, 13479.434), compared with HCWs exposed to colleagues as well as nurses and general service assistants who exposed to patients. Our study revealed that the serological testing is useful for the identification of asymptomatic or subclinical infection of SARS-CoV-2 among close contacts with COVID-19 patients.

Cheng, V. C. C., et al. (2020). "Absence of nosocomial transmission of coronavirus disease 2019 (COVID-19) due to SARS-CoV-2 in the prepandemic phase in Hong Kong." Am J Infect Control **48**(8): 890-896.

BACKGROUND: To describe the infection control strategy to achieve zero nosocomial transmission of symptomatic coronavirus disease (COVID-19) due to SARS-CoV-2 during the prepandemic phase (the first 72 days after announcement of pneumonia cases in Wuhan) in Hong Kong. METHODS: Administrative support with the aim of zero nosocomial transmission by reducing elective clinical services, decanting wards, mobilizing isolation facilities, providing adequate personal protective equipment, coordinating laboratory network for rapid molecular diagnosis under 4-tier active surveillance for hospitalized patients and outpatients, and organizing staff forum and training was implemented under the framework of preparedness plan in Hospital Authority. The trend of SARS-CoV-2 in the first 72 days was compared with that of SARS-CoV 2003. RESULTS: Up to day 72 of the epidemic, 130 (0.40%) of 32,443 patients being screened confirmed to have SARS-CoV-2 by reverse transcription polymerase chain reaction. Compared with SARS outbreak in 2003, the SARS-CoV-2 case load constituted 8.9% (130 SARS-CoV-2/1458 SARS-CoV) of SARS-CoV infected cases at day 72 of the outbreak. The incidences of nosocomial acquisition of SARS-CoV per 1,000 SARS-patient-day and per 100 SARS-patient-admission were 7.9 and 16.9, respectively, which were significantly higher than the corresponding incidences of SARS-CoV-2 (zero infection, P <.001). CONCLUSIONS: Administrative support to infection control could minimize the risk of nosocomial transmission of SARS-CoV-2.

Chernyshov, P. V., et al. (2020). "Position statement of the European Academy of Dermatology and Venereology Task Force on Quality of Life and Patient Oriented Outcomes on quality of life issues in dermatologic patients during the COVID-19 pandemic." J Eur Acad Dermatol Venereol **34**(8): 1666-1671.

The pandemic of COVID-19 is a global challenge for health care, and dermatologists are not standing apart from trying to meet this challenge. The European Academy of Dermatology and Venereology (EADV) has collected recommendations from its Task Forces (TFs) related to COVID-19. The Journal of the EADV has established a COVID-19 Special Forum giving free access to related articles. The psychosocial effects of the pandemic, an increase in contact dermatitis and several other skin diseases because of stress, disinfectants and protective equipment use, especially in healthcare workers, the temporary limited access to dermatologic care, the dilemma whether or not to pause immunosuppressive therapy, and, finally, the occurrence of skin lesions in patients infected by COVID-19 all contribute to significant quality of life (QoL) impairment. Here, we present detailed recommendations of the EADV TF on QoL and patient-oriented outcomes on how to improve QoL in dermatologic patients during the COVID-19 pandemic for several different groups of patients and for the general population.

Cherrie, J. W., et al. (2020). "Protecting healthcare workers from inhaled SARS-CoV-2 virus." Occup Med (Lond) **70**(5): 335-337.

Chersich, M. F., et al. (2020). "COVID-19 in Africa: care and protection for frontline healthcare workers." Global Health **16**(1): 46.

Medical staff caring for COVID-19 patients face mental stress, physical exhaustion, separation from families, stigma, and the pain of losing patients and colleagues. Many of them have acquired SARS-CoV-2 and some have died. In Africa, where the pandemic is escalating, there are major gaps in response capacity, especially in human resources and protective equipment. We examine these challenges and propose interventions to protect healthcare workers on the continent, drawing on articles identified on Medline (Pubmed) in a search on 24 March 2020. Global jostling means that supplies of personal protective equipment are limited in Africa. Even low-cost interventions such as facemasks for patients with a cough and water supplies for handwashing may be challenging, as is 'physical distancing' in overcrowded primary health care clinics. Without adequate protection, COVID-19 mortality may be high among healthcare workers and their family in Africa given limited critical care beds and difficulties in transporting ill healthcare workers from rural to urban care centres. Much can be done to protect healthcare workers, however. The continent has learnt invaluable lessons from Ebola and HIV control. HIV counselors and community healthcare workers are key resources, and could promote social distancing and related interventions, dispel myths, support healthcare workers, perform symptom screening and trace contacts. Staff motivation and retention may be enhanced through carefully managed risk 'allowances' or compensation. International support with personnel and protective equipment, especially from China, could turn the pandemic's trajectory in Africa around. Telemedicine holds promise as it rationalises human resources and reduces patient contact and thus infection risks. Importantly, healthcare workers, using their authoritative voice, can promote effective COVID-19 policies and prioritization of their safety. Prioritizing healthcare workers for SARS-CoV-2 testing, hospital beds and targeted research, as well as ensuring that public figures and the population acknowledge the commitment of healthcare workers may help to maintain morale. Clearly there are multiple ways that international support and national commitment could help safeguard healthcare workers in Africa, essential for limiting the pandemic's potentially devastating heath, socio-economic and security impacts on the continent.

Chhajed, P. N., et al. (2021). "Conventional flexible bronchoscopy during the COVID pandemic: A consensus statement from the Indian Association for Bronchology." Lung India **38**(Supplement): S105-s115.

During the times of the ongoing COVID pandemic, aerosol-generating procedures such as bronchoscopy have the potential of transmission of severe acute respiratory syndrome coronavirus 2 to the healthcare workers. The decision to perform bronchoscopy during the COVID pandemic should be taken judiciously. Over the years, the indications for bronchoscopy in the clinical practice have expanded. Experts at the Indian Association for Bronchology perceived the need to develop a concise statement that would assist a bronchoscopist in performing bronchoscopy during the COVID pandemic safely. The current Indian Association for Bronchology Consensus Statement provides specific guidelines including triaging, indications, bronchoscopy area, use of personal protective equipment, patient preparation, sedation and anesthesia, patient monitoring, bronchoscopy technique, sample collection and handling, bronchoscope disinfection, and environmental disinfection concerning the coronavirus disease-2019 situation. The suggestions provided herewith should be adopted in addition to the national bronchoscopy guidelines that were published recently. This statement summarizes the essential aspects to be considered for the performance of bronchoscopy in COVID pandemic, to ensure safety for both for patients and healthcare personnel.

Chieffo, A., et al. (2020). "EAPCI Position Statement on Invasive Management of Acute Coronary Syndromes during the COVID-19 pandemic." EuroIntervention **16**(3): 233-246.

The coronavirus disease 2019 (COVID-19) pandemic poses an unprecedented challenge to healthcare worldwide. The infection can be life threatening and require intensive care treatment. The transmission of the disease poses a risk to both patients and healthcare workers. The number of patients requiring hospital admission and intensive care may overwhelm health systems and negatively affect standard care for patients presenting with conditions needing emergency interventions. This position statements aims to assist cardiologists in the invasive management of acute coronary syndrome (ACS) patients in the context of the COVID-19 pandemic. To that end, we assembled a panel of interventional cardiologists and acute cardiac care specialists appointed by the European Association of Percutaneous Cardiovascular Interventions (EAPCI) and from the Acute Cardiovascular Care Association (ACVC) and included the experience from the first and worst affected areas in Europe. Modified diagnostic and treatment algorithms are proposed to adapt evidence-based protocols for this unprecedented challenge. Various clinical scenarios, as well as management algorithms for patients with a diagnosed or suspected COVID-19 infection, presenting with ST- and non-ST-segment elevation ACS are described. In addition, we address the need for re-organization of ACS networks, with redistribution of hub and spoke hospitals, as well as for in-hospital reorganization of emergency rooms and cardiac units, with examples coming from multiple European countries. Furthermore, we provide a guidance to reorganization of catheterization laboratories and, importantly, measures for protection of healthcare providers involved with invasive procedures.

Chieffo, A., et al. (2020). "EAPCI Position Statement on Invasive Management of Acute Coronary Syndromes during the COVID-19 pandemic." Eur Heart J **41**(19): 1839-1851.

The coronavirus disease 2019 (COVID-19) pandemic poses an unprecedented challenge to healthcare worldwide. The infection can be life threatening and require intensive care treatment. The transmission of the disease poses a risk to both patients and healthcare workers. The number of patients requiring hospital admission and intensive care may overwhelm health systems and negatively affect standard care for patients presenting with conditions needing emergency interventions. This position statements aims to assist cardiologists in the invasive management of acute coronary syndrome (ACS) patients in the context of the COVID-19 pandemic. To that end, we assembled a panel of interventional cardiologists and acute cardiac care specialists appointed by the European Association of Percutaneous Cardiovascular Interventions (EAPCI) and from the Acute Cardiovascular Care Association (ACVC) and included the experience from the first and worst affected areas in Europe. Modified diagnostic and treatment algorithms are proposed to adapt evidence-based protocols for this unprecedented challenge. Various clinical scenarios, as well as management algorithms for patients with a diagnosed or suspected COVID-19 infection, presenting with ST- and non-ST-segment elevation ACS are described. In addition, we address the need for re-organization of ACS networks, with redistribution of hub and spoke hospitals, as well as for in-hospital reorganization of emergency rooms and cardiac units, with examples coming from multiple European countries. Furthermore, we provide a guidance to reorganization of catheterization laboratories and, importantly, measures for protection of healthcare providers involved with invasive procedures.

Chieffo, A., et al. (2021). "Performing elective cardiac invasive procedures during the COVID-19 outbreak: a position statement from the European Association of Percutaneous Cardiovascular Interventions (EAPCI)." EuroIntervention **16**(14): 1177-1186.

The rearrangement of healthcare services required to face the coronavirus disease 2019 (COVID-19) pandemic led to a drastic reduction in elective cardiac invasive procedures. We are already facing a "second wave" of infections and we might be dealing during the next months with a "third wave" and subsequently new waves. Therefore, during the different waves of the COVID-19 pandemic we have to face the problems of how to perform elective cardiac invasive procedures in non-COVID patients and which patients/procedures should be prioritised. In this context, the interplay between the pandemic stage, the availability of healthcare resources and the priority of specific cardiac disorders is crucial. Clear pathways for "hot" or presumed "hot" patients and "cold" patients are mandatory in each hospital. Depending on the local testing capacity and intensity of transmission in the area, healthcare facilities may test patients for SARS-CoV-2 infection before the interventional procedure, regardless of risk assessment for COVID-19. Pre-hospital testing should always be conducted in the presence of symptoms suggestive of SARS-CoV-2 infection. In cases of confirmed or suspected COVID-19 positive patients, full personal protective equipment using FFP 2/N95 masks, eye protection, gowning and gloves is indicated during cardiac interventions for healthcare workers. When patients have tested negative for COVID-19, medical masks may be sufficient. Indeed, individual patients should themselves wear medical masks during cardiac interventions and outpatient visits.

Chien, L. C., et al. (2020). "A Positive-Pressure Environment Disposable Shield (PEDS) for COVID-19 Health Care Worker Protection." Prehosp Disaster Med **35**(4): 434-437.

The COVID-19 pandemic has strained health care system resources and reduced the availability of life-sustaining and medical-grade personal protective equipment (PPE) though the combination of increased demand and disrupted manufacturing supply chains. As a result of these shortages, many health care providers have temporarily used largely untested, improvised PPE (iPPE). Lack of quality control for makeshift PPE and frequent repurposing of used items to conserve supplies increase both the risk of provider infection and nosocomial spread to uninfected patients. One strategy to reduce risk of infection and preserve existing equipment is the implementation of secondary barrier devices placed directly over patients or providers. The authors describe an inexpensive, disposable, positive-pressure head isolation unit that can be rapidly constructed from materials readily available in nearly all health care settings for under five US dollars. The unit was successfully deployed in Taiwan during the 2003 Severe Acute Respiratory Syndrome (SARS) outbreak, and again during the COVID-19 pandemic. The iPPE worn directly by the health care workers (HCWs) can be donned prior to patient contact in the presence of an air source. This strategy may be more protective than a covering placed over the patient in an aerosol-generating environment, which requires the HCW to be in close contact with the patient prior to securing the protective device.

Chirico, F., et al. (2020). "COVID-19: Protecting Healthcare Workers is a priority." Infect Control Hosp Epidemiol **41**(9): 1117.

Chiu, T. F., et al. (2021). "Facing the Coronavirus Pandemic: An Integrated Continuing Education Program in Taiwan." Int J Environ Res Public Health **18**(5).

This study aimed to identify knowledge gaps regarding coronavirus disease 2019 (COVID-19) and develop an integrated educational program for healthcare workers. First, we designed and validated ten multiple-choice questions to identify knowledge gaps among healthcare workers. Within one month of the online test and curriculum offering, 5533 staff had completed the test, with a completion rate of 84.97%. There were 2618 healthcare workers who answered the pre-test 100% correctly. Those who did not answer the pre-test 100% correctly took multiple tests after learning through the online teaching materials. Eventually, 5214 staff passed the test (pre-test or post-test with 100% correct answers). The result showed that all staff had a low correct rate for personal protective equipment (PPE) use recommendations. The Infection Control Center conducted training sessions for hospital staff on how to wear protective clothing. Information on the selection and use of PPE for infection prevention was provided, and participants were allowed time to practice and familiarize themselves with the correct way to wear PPE. Moreover, the Department of Education and Research continued updating the online learning materials based on the most important updated peer-reviewed published articles. The attending teaching physicians helped to search, translate, and take notes on articles in the local language (traditional Chinese) for other colleagues to read easily. We expect to increase learning opportunities for healthcare workers, even during uncertain times such as the current coronavirus pandemic through (1) the hospital-wide course announcements, (2) the continuous placement of test questions and learning files on the digital learning platform, (3) the placement of journal highlights in cloud folders, and (4) the use of the digital learning platform on mobile phones accessible outside the hospital.

Choi, U. Y., et al. (2021). "Surveillance of the infection prevention and control practices of healthcare workers by an infection control surveillance-working group and a team of infection control coordinators during the COVID-19 pandemic." J Infect Public Health **14**(4): 454-460.

BACKGROUND: During the ongoing coronavirus disease (COVID-19) pandemic, hospitals have strengthened their guidelines on infection prevention and control (IPC), and a rigorous adherence to these guidelines is crucial. An infection control surveillance-working group (ICS-WG) and infection control coordinators (ICCs) team were created to monitor the IPC practices of the healthcare workers (HCWs) in a regional hospital in Korea. This study analyzed the surveillance results and aimed to identify what IPC practices needed improvement. METHODS: During phase 1 (March to April 2020), the ICS-WG performed random audits, recorded incidences of improper IPC practices, and provided advice to the violators. During phase 2 (April to July), the ICCs inspected the hospital units and proposed practical ideas about IPC. The surveillance and proposals targeted the following practices: patient screening, usage of personal protective equipment (PPE), hand and respiratory hygiene, equipment reprocessing, environmental cleaning, management of medical waste, and social distancing. RESULTS: In phase 1, of the 127 violations observed, most (32.3%) corresponded to hand and respiratory hygiene. In phase 2, the highest proportion of violation per category was observed in the management of medical waste (37.8%); among these, a higher proportion of violation (71.4%) was observed in the collection of medical waste. Of the 106 proposals made by the ICCs, the most addressed practice was patient screening (28.3%). No case of nosocomial infection was reported during the study period. CONCLUSION: Adherence to proper hand and respiratory hygiene was inadequate at the early stage of the COVID-19 pandemic. The results indicate that more attention and further training are needed for the management of medical waste, particularly medical waste collection, and that continuous upgrading of the strategies for patient screening is essential. These results will be useful in helping other healthcare facilities to establish their IPC strategies.

Chou, E., et al. (2020). "Onsite telemedicine strategy for coronavirus (COVID-19) screening to limit exposure in ED." Emerg Med J **37**(6): 335-337.

Coronavirus (severe acute respiratory syndrome coronavirus 2) outbreak is a public health emergency and a global pandemic. During the present coronavirus disease (COVID-19) crisis, telemedicine has been recommended to screen suspected patients to limit risk of exposure and maximise medical staff protection. We constructed the protective physical barrier with telemedicine technology to limit COVID-19 exposure in ED. Our hospital is an urban community hospital with annual ED volume of approximately 50 000 patients. We equipped our patient exam room with intercom and iPad for telecommunication. Based on our telemedicine screening protocol, physician can conduct a visual physical examination on stable patients via intercom or videoconference. Telemedicine was initially used to overcome the physical barrier between patients and physicians. However, our protocol is designed to create a protective physical barrier to protect healthcare workers and enhance efficiency in ED. The implementation can be a promising protocol in making ED care more cost-effective and efficient during the COVID-19 pandemic and beyond.

Chou, W. P., et al. (2020). "Risk Perception, Protective Behaviors, and General Anxiety during the Coronavirus Disease 2019 Pandemic among Affiliated Health Care Professionals in Taiwan: Comparisons with Frontline Health Care Professionals and the General Public." Int J Environ Res Public Health **17**(24).

This study aimed to compare risk perception, information sources, adoption of protective behaviors against coronavirus disease 2019 (COVID-19), and levels of general anxiety among affiliated health care professionals, frontline health care professionals, and the general public in Taiwan. We recruited participants via a Facebook advertisement. We determined the risk perception, information sources, adoption of protective behaviors against COVID-19, and levels of general anxiety among 1954 respondents. In total, 269 affiliated healthcare workers, 371 frontline healthcare workers, and 1314 members of the general public were recruited into this study. The results indicated that both affiliated and frontline health care professionals had a higher level of risk perception of COVID-19, and more adopted protective behaviors against COVID-19 than the general public. No significant differences in risk perception or the adoption of protective behaviors were identified between affiliated, and frontline, health care professionals. Affiliated health care professionals had a lower level of general anxiety than the general public, whereas frontline health care professionals exhibited no significant difference in level of general anxiety compared with the general public or affiliated health care professionals. As important members of COVID-19 treatment teams, the need for psychological and educational support in affiliated health care professionals should receive attention.

Choudhury, A., et al. (2020). "Physiological Effects of N95 FFP and PPE in Healthcare Workers in COVID Intensive Care Unit: A Prospective Cohort Study." Indian J Crit Care Med **24**(12): 1169-1173.

BACKGROUND: Healthcare workers (HCWs) are at increased risk of exposure to severe acute respiratory syndrome-coronavirus-2 (SARS-CoV-2). Personal protective equipment (PPE) is mandated for HCWs. However, the physiological effects on the HCWs while working in the protective gear remains unexplored. This study aimed to assess the physiological effects of the prolonged use of PPE on HCWs. MATERIALS AND METHODS: Seventy-five HCWs, aged 18-50 years were enrolled in this prospective, observational, cohort study. The physiological variables [heart rate, oxygen saturation, and perfusion index (PI)] were recorded at the start of duty, 4 hours after wearing N95 filtering facepiece respirator (FFR), pre-donning, and post-doffing. The rating of perceived exertion (RPE) score and modified Borg scale for dyspnea was evaluated. The physiological variables were represented as the mean ± standard deviation. Wilcoxon signed-rank test was used to show any difference in RPE and modified Borg scale for dyspnea. A p value of <0.05 was considered significant. RESULTS: There is a statistically significant difference in the physiological parameters post-doffing compared with baseline: Heart rate (p < 0.001); oxygen saturation (p < 0.001); PI (p < 0.001). RPE score showed increased discomfort with continuous use of N95 FFR. However, exertion increased only marginally. The major adverse effects noted with PPE use were fogging, headache, tiredness, difficulty in breathing, and mask soakage, with a resultant mean duration of donning to be 3.1 hours. CONCLUSION: The use of PPE can result in considerable changes in the physiological variables of healthy HCWs. The side effects may lead to excessive exhaustion and increased tiredness after prolonged shifts in the intensive care unit (ICU) while wearing PPE. HOW TO CITE THIS ARTICLE: Choudhury A, Singh M, Khurana DK, Mustafi SM, Ganapathy U, Kumar A, et al. Physiological Effects of N95 FFP and PPE in Healthcare Workers in COVID Intensive Care Unit: A Prospective Cohort Study. Indian J Crit Care Med 2020;24(12):1169-1173.

Chowdhury, M. M., et al. (2020). "Covid-19: virtual occupational skin health clinics for healthcare workers." Bmj **369**: m2281.

Chughtai, A. A., et al. (2020). "Effectiveness of Cloth Masks for Protection Against Severe Acute Respiratory Syndrome Coronavirus 2." Emerg Infect Dis **26**(10).

Cloth masks have been used in healthcare and community settings to protect the wearer from respiratory infections. The use of cloth masks during the coronavirus disease (COVID-19) pandemic is under debate. The filtration effectiveness of cloth masks is generally lower than that of medical masks and respirators; however, cloth masks may provide some protection if well designed and used correctly. Multilayer cloth masks, designed to fit around the face and made of water-resistant fabric with a high number of threads and finer weave, may provide reasonable protection. Until a cloth mask design is proven to be equally effective as a medical or N95 mask, wearing cloth masks should not be mandated for healthcare workers. In community settings, however, cloth masks may be used to prevent community spread of infections by sick or asymptomatically infected persons, and the public should be educated about their correct use.

Chung, H., et al. (2020). "Risk of COVID-19 Transmission from Infected Outpatients to Healthcare Workers in an Outpatient Clinic." J Korean Med Sci **35**(50): e431.

From January to September 2020, we conducted contact-tracing for confirmed coronavirus disease 2019 (COVID-19) patients who visited our outpatient clinic to evaluate the risk of COVID-19 transmission from infected patients to healthcare workers (HCWs). COVID-19 was diagnosed in 8 outpatients. Per the severe acute respiratory syndrome coronavirus-2 (SARS-CoV-2) polymerase chain reaction test results, none of the 317 HCWs who came in contact had a confirmed COVID-19 diagnosis (0.00%; 95% confidence interval, 0.00%-0.01%). This low rate of transmission from infected outpatients to HCWs suggests that standard precaution such as using surgical mask and hand hygiene protects HCWs from SARS-CoV-2 in a low-risk, outpatient clinic setting.

Cieri, M., et al. (2020). "How Are We Facing It? Dispatches From Pathology Residents in a COVID-19 Lombardy Hospital." Front Public Health **8**: 259.

At the end of February, the Italian National Health Service reported a hot spot of Coronavirus disease in the Lombardy region. COVID-19 is a highly pathogenic viral infection which poses some challenges for healthcare workers. Indeed, Pathology Departments are involved in reorganizing samples' management, from their delivery until their processing, according to National and WHO guidelines. Since Lombardy has been declared COVID-19 hot spot, due to decreasing number of surgical procedures, our Department adopted a policy to reduce personnel, allowing pathologists to work remotely during the outbreak. Lacking clear information about viral load on tissue samples, all human specimens must be considered potentially infectious, as well as patients during post-mortem examinations, and clinical information on COVID-19 status is mandatory. It is also important that Pathology staff receive an adequate training, and adherence to rules should be always accompanied by common sense.

Cihan, F. G. and F. Gökgöz Durmaz (2021). "Evaluation of COVID-19 phobia and the feeling of loneliness in the geriatric age group." Int J Clin Pract: e14089.

OBJECTIVE: Social isolation and quarantine are unusual precautions taken all over the world against the COVID-19 pandemic. Some legal sanctions were made in order to protect vulnerable age groups such as the geriatric population, to make them stay at home. These measures, which are vital for pandemic management, have negative psychological effects on elderly individuals. The aim of this study was to reveal how pandemic process affected individuals over 65 years old psychosocially. MATERIALS AND METHODS: Socio-demographic characteristics questionnaire, the COVID-19 Phobia Scale (C19P-S) and the Loneliness Scale for the Elderly individuals were used. Descriptive analysis, Mann-Whitney U-test, Kruskal-Wallis test, Pearson correlation analysis were performed for data analysis. RESULTS: A total of 130 elderly people (80 women, 50 men) were included in the study. Of the participants, 61.5% were women, 67.7% were married and mean age was 71.53 ± 5.57 (min. 65-max. 87) years old. The C19P-S scores were significantly higher in women, singles, people living alone, those living in an apartment, and those with higher economic and educational level (P = .001). There was a strong positive correlation between age and total loneliness score. Loneliness scores were significantly higher in singles, those with higher levels of education and income, those who live alone, who was a relative of a healthcare worker, and whose relatives were infected with COVID-19 (P < .05). CONCLUSION: Our study revealed that elderly individuals, who have to comply with social isolation rules, have increased fear and loneliness due to coronavirus. Considering this situation, timely psychological treatment and social support measures should be taken.

Cinar, P., et al. (2020). "Safety at the Time of the COVID-19 Pandemic: How to Keep our Oncology Patients and Healthcare Workers Safe." J Natl Compr Canc Netw: 1-6.

The novel coronavirus, SARS-CoV-2, was first detected as a respiratory illness in December 2019 in Wuhan City, China. Since then, coronavirus disease 2019 (COVID-19) has impacted every aspect of our lives worldwide. In a time when terms such as social distancing and flattening the curve have become a part of our vernacular, it is essential that we understand what measures can be implemented to protect our patients and healthcare workers. Undoubtedly, healthcare providers have had to rapidly alter care delivery models while simultaneously acknowledging the crucial unknowns of how these changes may affect clinical outcomes. This special feature reviews strategies on how to mitigate transmission of COVID-19 in an effort to reduce morbidity and mortality associated with the disease for patients with cancer without infection, for patients with cancer with COVID-19 infection, and for the healthcare workers caring for them, while continuing to provide the best possible cancer care. [Editor's Note: This article includes the most current information available at time of publication; however, recommendations regarding public safety and practice may change rapidly in this situation. Individuals should get the most up to date information from the CDC website.].

Ciocan, C., et al. (2020). "Impact of wearing a surgical mask on respiratory function in view of a widespread use during COVID-19 outbreak. A case-series study." Med Lav **111**(5): 354-364.

BACKGROUND: Because of the COVID-19 outbreak, the widespread use of Respiratory Protective Devices (RPD) is recommended to prevent the spread of infection. This recommendation involves not only healthcare workers but other category of workers and the general population as well, in public places, especially where social distancing is difficult to maintain. The use of facemasks should not cause physical impairment to individuals, especially for people suffering from lung and heart diseases. OBJECTIVES: To evaluate the impact of RPDs on the respiratory function in healthy and asthmatic subjects, in order to identify the fitness for use mainly, but not only for, occupational purposes during COVID-19 outbreak. METHODS: Ten individuals were included, three of which affected by asthma and three current smokers. A Respiratory Functional Test (RFT) was performed at three times: at the beginning of the work shift 1) without wearing and 2) wearing surgical masks, and 3) after 4 hours of usual working activities wearing the masks. Arterial Blood Gas (ABG) samples were also tested before the first test and the third test. RESULTS: Observed RFTs and ABG parameters did not suffer significant variations, but for Maximal Voluntary Ventilation (P=0.002). Data on asthmatic subjects and smokers were comparable to healthy subjects. DISCUSSION: Our results suggest that wearing a surgical mask does not produce significant respiratory impairment in healthy subjects nor in subjects with asthma. Four hours of continuing mask-wearing do not cause a reduction in breathing parameters. Fitness for use in subjects with more severe conditions has to be evaluated individually. Our adapted technique for RFTs could be adopted for the individual RPDs fitness evaluation.

Publisher: Abstract available from the publisher.

ita

Cioffi, A. and R. Rinaldi (2020). "Covid-19 and medical liability: A delicate balance." Med Leg J **88**(4): 187-188.

During the Covid-19 pandemic, many countries around the world are considering whether and how to provide liability protection to front-line healthcare staff. The guiding principle of liability protection for physicians and others is to ensure that, in a serious emergency situation, health professionals can devote themselves exclusively to their work and to patient care, without the fear of future claims for unforeseeable, but above all unavoidable, injury, loss and damage caused by their conduct. Great care is needed to balance the interests and rights of all those involved. Liability protection could have risky consequences with the final result that doctors will not be protected, but institutions such as health facilities will be even if they were in fact responsible for foreseeable and avoidable damage.

Clementini, M., et al. (2020). "Aerosol transmission for SARS-CoV-2 in the dental practice. A review by SIdP Covid-19 task-force." Oral Dis.

Current evidence suggests that SARS-CoV-2, the virus that causes COVID-19, is predominantly spread from person to person. Aim of this narrative review is to explore transmission modality of SARS-CoV-2 to provide appropriate advice to stakeholders, in order to support the implementation of effective public health measures and protect healthcare workers that primary face the disease. "In vivo" and "in vitro" studies from laboratories and hospitals confirmed the presence of surface contamination and provided insight of SARS-CoV-2 detection in the air, particularly in indoor settings with poor ventilation where aerosol-generating procedures were performed. Measures for aerosol reduction, in conjunction with other effective infection control strategies, are needed to prevent the spread of SARS-CoV-2 in dental setting.

Climent-Ballester, S. and J. Selva-Otaolaurruchi (2020). "Hospital Pharmacy: Comprehensive management of medical devices during SARS-CoV-2." Farm Hosp **44**(7): 21-23.

Medical devices have become essential to the prevention and control of the  COVID-19 pandemic, being crucial for health professionals and patients in  particular, and the population in general. It is important to be aware of the laws  that regulate the management, distribution, and control of medical devices.  Article 82 of the Spanish Law 29/2006 on Guarantees and Rational Use of  Medicines and Medical Devices establishes that it is the responsibility of Hospital  Pharmacy Services "to participate in and coordinate the purchase of medicines and medical devices in the hospital to ensure an efficient acquisition  and rational use of medical devices". For this reason, working groups of the Spanish Society of Hospital Pharmacy and other scientific societies have issued technical guidelines and consensus statements to provide technical support and updated information on the use of masks, individual  protection equipments and other medical devices. In addition, the shortage of  medical devices caused by the high demand has resulted in the uncontrolled  production and distribution of medical devices. This phenomenon, added to the  fraudulent selling of medical devices, highlights the need for a closer surveillance of the market to guarantee the efficacy and safety of available medical devices. A rational use of medical devices is necessary to ensure the availability and safety of these products, which requires the involvement of  different stakeholders, including hospital pharmacists. Thus, it is essential that  hospital pharmacists receive specific training in technical aspects concerning the possession and use of medical devices. This will help guarantee an effective and safe use of medical products. The acquisition and use of medical  devices requires a keen understanding of the technical and legal aspects  concerning these products, which makes hospital pharmacists essential for the  integral management of medical devices.

Clinkard, D., et al. (2021). "Evaluation of N95 respirators, modified snorkel masks and low-cost powered air-purifying respirators: a prospective observational cohort study in healthcare workers." Anaesthesia **76**(5): 617-622.

Disposable N95 respirator masks are the current standard for healthcare worker respiratory protection in the COVID-19 pandemic. In addition to shortages, qualitative fit testing can have low sensitivity for detecting poor fit, leading to inconsistent protection. Multiple groups have developed alternative solutions such as modified snorkel masks to overcome these limitations, but validation of these solutions has been lacking. We sought to determine if N95s and snorkel masks with attached high-efficiency filters provide consistent protection levels in healthcare workers and if the addition of positive pressure via an inexpensive powered-air purifying respirator to the snorkel mask would provide enhanced protection. Fifty-one healthcare workers who were qualitatively fitted with N95 masks underwent quantitative mask fit testing according to a simulated workplace exercise protocol. N95, snorkel masks with high-efficiency filters and snorkel masks with powered-air purifying respirators were tested. Respiratory filtration ratios were collected for each step and averaged to obtain an overall workplace protocol fit factor. Failure was defined as either an individual filtration ratio or an overall fit factor below 100. N95s and snorkel masks with high-efficiency filters failed one or more testing steps in 59% and 20% of participants, respectively, and 24% and 12% failed overall fit factors, respectively. The snorkel masks with powered-air purifying respirators had zero individual or overall failures. N95 and snorkel masks with high-efficiency filter respirators were found to provide inconsistent respiratory protection in healthcare workers.

Cobb, N., et al. (2021). "Pragmatic Recommendations for Infection Prevention and Control Practices for Healthcare Facilities in Low- and Middle-Income Countries during the COVID-19 Pandemic." Am J Trop Med Hyg **104**(3 Suppl): 25-33.

Infection prevention and control (IPC) strategies are key in preventing nosocomial transmission of COVID-19. Several commonly used IPC practices are resource-intensive and may be challenging to implement in resource-constrained settings. An international group of healthcare professionals from or with experience in low- and middle-income countries (LMICs) searched the literature for relevant evidence. We report on a set of pragmatic recommendations for hospital-based IPC practices in resource-constrained settings of LMICs. For cases of confirmed or suspected COVID-19, we suggest that patients be placed in a single isolation room, whenever possible. When single isolation rooms are unavailable or limited, we recommend cohorting patients with COVID-19 on dedicated wards or in dedicated hospitals. We also recommend that cases of suspected COVID-19 be cohorted separately from those with confirmed disease, whenever possible, to minimize the risk of patient-to-patient transmission in settings where confirmatory testing may be limited. We suggest that healthcare workers be designated to care exclusively for patients with COVID-19, whenever possible, as another approach to minimize nosocomial spread. This approach may also be beneficial in conserving limited supplies of reusable personal protective equipment (PPE). We recommend that visitors be restricted for patients with COVID-19. In settings where family members or visitors are necessary for caregiving, we recommend that the appropriate PPE be used by visitors. We also recommend that education regarding hand hygiene and donning/doffing procedures for PPE be provided. Last, we suggest that all visitors be screened for symptoms before visitation and that visitor logs be maintained.

Coelho, M. M. F., et al. (2020). "Pressure injury related to the use of personal protective equipment in COVID-19 pandemic." Rev Bras Enferm **73**(suppl 2): e20200670.

OBJECTIVE: To describe the prevalence and factors associated with pressure injuries related to the use of personal protective equipment during the COVID-19 pandemic. METHODS: Cross-sectional study conducted using an instrument made available in social networks with 1,106 health professionals. The data were analyzed using descriptive statistics and compared, considering pvalue < 0.05. RESULTS: There was a prevalence of 69.4% for pressure injuries related to the use of personal protective equipment, with an average of 2.4 injuries per professional. The significant factors were: under 35 years of age, working and wearing personal protective equipment for more than six hours a day, in hospital units, and without the use of inputs for protection. CONCLUSION: Pressure injuries related to the use of medical devices showed a high prevalence in this population. The recognition of the damage in these professionals makes it possible to advance in prevention strategies.

Cohen, J. and Y. V. M. Rodgers (2020). "Contributing factors to personal protective equipment shortages during the COVID-19 pandemic." Prev Med **141**: 106263.

This study investigates the forces that contributed to severe shortages in personal protective equipment in the US during the COVID-19 crisis. Problems from a dysfunctional costing model in hospital operating systems were magnified by a very large demand shock triggered by acute need in healthcare and panicked marketplace behavior that depleted domestic PPE inventories. The lack of effective action on the part of the federal government to maintain and distribute domestic inventories, as well as severe disruptions to the PPE global supply chain, amplified the problem. Analysis of trade data shows that the US is the world's largest importer of face masks, eye protection, and medical gloves, making it highly vulnerable to disruptions in exports of medical supplies. We conclude that market prices are not appropriate mechanisms for rationing inputs to health because health is a public good. Removing the profit motive for purchasing PPE in hospital costing models, strengthening government capacity to maintain and distribute stockpiles, developing and enforcing regulations, and pursuing strategic industrial policy to reduce US dependence on imported PPE will help to better protect healthcare workers with adequate supplies of PPE.

Cole, J. and E. Barnard (2020). "The Impact of the COVID-19 Pandemic on Healthcare Acquired Infections with Multidrug Resistant Organisms." Am J Infect Control: 5726.

This retrospective, cross-sectional study was conducted in four community hospitals in Los Angeles County, California. The assumption of this study was, coronavirus disease-19 (COVID-19) contributed to the increase in healthcare workers (HCW) compliance with infection prevention measures. IP initiatives fostered among HCWs have increased awareness of effective hand washing, cleaning equipment after use and appropriate personal protective equipment (PPE) use which has subsequently decreased healthcare acquired infections (HAI) with multidrug-resistant organisms (MDRO).

Collins, R. (2020). "COVID-19: Nurses have responded, now it is time to support them as we move forward." Healthc Manage Forum **33**(5): 190-194.

The inspiration for The American Nurse Project, Dr. Rhonda Collins, DNP, RN, FAAN, is Chief Nursing Officer for Vocera Communications. Every year around Nurses Week, Dr. Collins publishes a report examining important issues that impact the nursing profession worldwide. Her 2020 CNO report looks at many of the challenges impacting nurses before, during, and after COVID-19-highlighting the mental anguish and physical burdens that COVID-19 has placed on nurses and other frontline healthcare workers as they put themselves in harm's way to protect others. Dr. Collins examines the foundation of cognitive science and outlines a three-point strategy to guide hospital and nurse leaders moving forward as they strive to support staff members: lightening clinicians' cognitive load by addressing the difference between documentation and communication, relieving the burden of adapting to multiple systems by giving clinicians control over how they communicate, and providing clinicians with clear, contextual, just-in-time information-using software to enhance workflow, not distract from it. During these unprecedented times, health leaders can honour nurses by providing them with the tools to help strengthen resiliency and healing from this crisis.

Contejean, A., et al. (2021). "Comparing Dynamics and Determinants of Severe Acute Respiratory Syndrome Coronavirus 2 Transmissions Among Healthcare Workers of Adult and Pediatric Settings in Central Paris." Clin Infect Dis **72**(2): 257-264.

BACKGROUND: Healthcare workers (HCWs) have paid a heavy toll during the coronavirus disease 2019 (COVID-19) outbreak. Routes of transmission remain to be fully understood. METHODS: This prospective study compared a 1500-bed adult and 600-bed pediatric setting of a tertiary-care university hospital located in central Paris. From 24 February until 10 April 2020, all symptomatic HCWs were screened for severe acute respiratory syndrome coronavirus 2 (SARS-CoV-2) on a nasopharyngeal swab. HCWs screened positive were questioned on their profession, symptoms, and occupational and nonoccupational exposures to SARS-CoV-2. RESULTS: Among 1344 HCWs tested, 373 were positive (28%) and 336 (90%) corresponding questionnaires were completed. Three hospitalizations and no deaths were reported. Most HCWs (70%) had patient-facing occupational activities (22% in COVID-19 dedicated units). The total number of HCW cases peaked on 23 March, then decreased slowly, concomitantly with a continuous increase of compliance to preventive measures (including universal medical masking and personal protective equipment [PPE] for direct care to COVID-19 patients). Attack rates were of 3.2% and 2.3% in the adult and pediatric settings, respectively (P = .0022). In the adult setting, HCWs more frequently reported exposure to COVID-19 patients without PPE (25% vs 15%, P = .046). Report of contacts with children attending out-of-home care facilities dramatically decreased over the study period. CONCLUSIONS: Universal masking, reinforcement of hand hygiene, and PPE with medical masks for patients' care allowed protection of HCWs and containment of the outbreak. Residual transmissions were related to persistent exposures with undiagnosed patients or colleagues and not to contacts with children attending out-of-home care facilities.

Contejean, A., et al. (2021). "Transmission Routes of Severe Acute Respiratory Syndrome Coronavirus 2 Among Healthcare Workers of a French University Hospital in Paris, France." Open Forum Infect Dis **8**(3): ofab054.

In this case-control study on 564 healthcare workers of a university hospital in Paris (France), contacts without protection with coronavirus disease 2019 (COVID-19) patients or with colleagues were associated with infection with severe acute respiratory syndrome coronavirus 2, whereas working in a COVID-dedicated unit and having children kept in childcare facilities were not.

Convissar, D., et al. (2020). "Personal Protective Equipment N95 Facemask Shortage Quick Fix: The Modified Airway From VEntilatoR Circuit (MAVerIC)." Cureus **12**(5): e7914.

We are in a crisis where healthcare providers on the frontlines are running out of the appropriate personal protective equipment including N95 masks and power air-purifying respirators. Here, we propose a makeshift filter mask that we call the Modified Airway from VEntilatoR Circuit (MAVerIC) that can be assembled within seconds using widely available supplies routinely utilized by anesthesia providers in the operating room to provide practitioners on the frontlines with the high standard of protection of a N95 mask during the coronavirus disease 2019 (COVID-19) pandemic, and can be easily quantitatively "fit tested" to ensure no significant leak to optimize safety and efficacy.

Cook, T. M., et al. (2021). "The safety of anaesthetists and intensivists during the first COVID-19 surge supports extension of use of airborne protection PPE to ward staff." Clin Med (Lond) **21**(2): e137-e139.

A key controversy in the COVID-19 pandemic has been over staff safety in health and social care settings. Anaesthetists and intensivists were anticipated to be at the highest risk of work-related infection due to involvement in airway management and management of critical illness and therefore wear the highest levels of personal protective equipment (PPE) in the hospital. However, the data clearly show that those working in anaesthesia and critical care settings are at lower risk of infection, harm and death from COVID-19 than colleagues working on the wards. The observed safety of anaesthetists and intensivists and increased risk to those in other patient-facing roles has implications for transmission-based infection control precautions. The precautionary principle supports extending training in and use of airborne precaution PPE to all staff working in patient-facing roles who have close contact with coughing patients. This will both reduce their risk of contracting COVID-19, maintain services and reduce nosocomial transmission to vulnerable patients. The emergence of a new variant of the SARS-CoV-2 virus with significantly higher transmissibility creates urgency to addressing this matter.

Cook, T. M., et al. (2020). "Consensus guidelines for managing the airway in patients with COVID-19: Guidelines from the Difficult Airway Society, the Association of Anaesthetists the Intensive Care Society, the Faculty of Intensive Care Medicine and the Royal College of Anaesthetists." Anaesthesia **75**(6): 785-799.

Severe acute respiratory syndrome-corona virus-2, which causes coronavirus disease 2019 (COVID-19), is highly contagious. Airway management of patients with COVID-19 is high risk to staff and patients. We aimed to develop principles for airway management of patients with COVID-19 to encourage safe, accurate and swift performance. This consensus statement has been brought together at short notice to advise on airway management for patients with COVID-19, drawing on published literature and immediately available information from clinicians and experts. Recommendations on the prevention of contamination of healthcare workers, the choice of staff involved in airway management, the training required and the selection of equipment are discussed. The fundamental principles of airway management in these settings are described for: emergency tracheal intubation; predicted or unexpected difficult tracheal intubation; cardiac arrest; anaesthetic care; and tracheal extubation. We provide figures to support clinicians in safe airway management of patients with COVID-19. The advice in this document is designed to be adapted in line with local workplace policies.

Coppeta, L., et al. (2020). "Contact Screening for Healthcare Workers Exposed to Patients with COVID-19." Int J Environ Res Public Health **17**(23).

In China and Italy, many cases of coronavirus disease 2019 (COVID-19) have occurred among healthcare workers (HCWs). Prompt identification, isolation and contact tracing of COVID-19 cases are key elements in controlling the COVID-19 pandemic. The aim of this study was to evaluate the rate of Severe Acute Respiratory Syndrome Coronavirus 2 (SARS-CoV-2) infection among HCWs exposed to patients with COVID-19 in relation to the main determinants of exposure. To assess the risk of exposure, we performed active symptom monitoring in 1006 HCWs identified as contacts of COVID19 cases. The presence of symptoms was statistically associated with a positive nasopharyngeal swab result. Only one subject was asymptomatic at the time of positive test. These data suggest that clinical history may help in the selection of subjects to be investigated by means of reverse transcriptase-polymerase chain reaction (RT-PCR) in the case of a shortage of diagnostic resources. We found that close contact (within 2 m for 15 min or more) was not statistically related to contagion. Regarding the use of personal protective equipment (PPE), only the use of facial masks was inversely related to the chance of becoming infected (p < 0.01). In conclusion, our data show that unprotected contacts between HCWs should be considered a major route of HCW contagion, suggesting that the use of facial masks should be implemented even in settings where known patients with COVID-19 are not present.

Cordina, M., et al. (2021). "Attitudes towards COVID-19 vaccination, vaccine hesitancy and intention to take the vaccine." Pharm Pract (Granada) **19**(1): 2317.

BACKGROUND: The pandemic is at a paradoxical stage, with vaccine roll out initiated but a significantly elevated level of infection and death. Hope for recovery lies in high equitable vaccine uptake. OBJECTIVE: The study aimed to: i) explore attitudes and factors influencing attitudes, towards the COVID-19 vaccine amongst people living in Malta, ii) identify the reasons as to why individuals are unsure or unwilling to take the vaccine. METHODS: Two consecutive, short, anonymous online surveys using social media platforms were used to gather data from adult individuals. The first study was open to residents in Malta, while the second study invited international participation. Study 1 consisted of 17 questions inspired by the Theories of Planned Behaviour and Reasoned Action. Study 2 asked participates whether they were willing, unwilling or unsure of taking the vaccine and their reasons for being unsure or unwilling. RESULTS: A total of 2,529 individuals participated in Study 1 and 834 in Study 2. In both studies respondents were predominantly female having a tertiary education. Over 50% declared that they were willing to take the vaccine, with males being more willing (t=5.83, df=1164.2, p<0.00005). Opinions of significant others- family and friends (r=0.22, p<0.005) and health professionals (r=0.74, p<0.005) were associated with willingness to take the vaccine. Vaccine hesitancy was present in the study population with 32.6% being unsure and 15.6% declaring that they were not willing to take the vaccine. Females were more likely to be unsure (Chi-squared=14.63, df=4, p=0.006). Lack of vaccine safety was the main reason cited for unwillingness to take the vaccine. Predictors for willingness to take the vaccine were: i) The belief that the COVID-19 vaccine will protect the health of the people who take it; ii) Valuing the advice of health professionals regarding the effectiveness of COVID-19 vaccine; iii) Having taken the influenza vaccine last year and; iv) Encouraging their elderly parents to take the vaccine. CONCLUSIONS: COVID-19 vaccination information campaigns should promote group strategies, focusing on emphasising the safety of the vaccine and offer reassurance, especially to women.

Corral, J. E., et al. (2020). "COVID-19 polymerase chain reaction testing before endoscopy: an economic analysis." Gastrointest Endosc **92**(3): 524-534.e526.

BACKGROUND AND AIMS: The novel coronavirus disease 2019 (COVID-19) pandemic has limited endoscopy utilization, causing significant health and economic losses. We aim to model the impact of polymerase chain reaction (PCR) testing into resuming endoscopy practice. METHODS: We performed a retrospective review of endoscopy utilization during the COVID-19 pandemic for a baseline reference. A computer model compared 3 approaches: strategy 1, endoscopy for urgent indications only; strategy 2, testing for semiurgent indications; and strategy 3, testing all patients. Analysis was made under current COVID-19 prevalence and projected prevalence of 5% and 10%. Primary outcomes were number of procedures performed and/or canceled. Secondary outcomes were direct costs, reimbursement, personal protective equipment used, and personnel infected. Disease prevalence, testing accuracy, and costs were obtained from the literature. RESULTS: During the COVID-19 pandemic, endoscopy volume was 12.7% of expected. Strategies 2 and 3 were safe and effective interventions to resume endoscopy in semiurgent and elective cases. Investing 22 U.S. dollars (USD) and 105 USD in testing per patient allowed the completion of 19.4% and 95.3% of baseline endoscopies, respectively. False-negative results were seen after testing 4700 patients (or 3 months of applying strategy 2 in our practice). Implementing PCR testing over 1 week in the United States would require 13 and 64 million USD, with a return of 165 and 767 million USD to providers, leaving 65 and 325 healthcare workers infected. CONCLUSIONS: PCR testing is an effective strategy to restart endoscopic practice in the United States. PCR screening should be implemented during the second phase of the pandemic, once the healthcare system is able to test and isolate all suspected COVID-19 cases.

Costa, J. A., et al. (2020). "Cardiovascular Implications in Patients Infected with Covid-19 and the Importance of Social Isolation to Reduce Dissemination of the Disease." Arq Bras Cardiol **114**(5): 834-838.

Respiratory symptoms, especially the development of severe acute respiratory distress syndrome, dominate the discussion and initial concerns of the population and health professionals. However, the cardiovascular system is greatly affected by these conditions and is often responsible for complications and mortality of these patients. In order to show the cardiovascular implications in patients infected with COVID-19 and the importance of social isolation as an alternative to curb the spread of the disease, a literature review was carried out based on 37 articles, in English, Portuguese and Spanish, available on Scielo and PubMed. The findings showed that cardiac complications associated with COVID-19 infection are similar to those produced by: severe acute respiratory syndrome (SARS), Middle East respiratory syndrome (MERS) and influenza. However, COVID-19 has a much greater and faster contamination and, unlike influenza, there is no vaccine or treatment available yet. In view of this, social isolation becomes a tool that can reduce and flatten the curve of cases and thus protect the people at higher risk, decreasing the chances of serious conditions related to the disease, potential deaths and the collapse of the country's health system.

Coto, J., et al. (2020). "The impact of COVID-19 on allied health professions." PLoS One **15**(10): e0241328.

The purpose of the current study was to examine the impact of Severe Acute Respiratory Syndrome Coronavirus 2 (SARS-CoV-2 or COVID-19) on allied health professionals work environment, access to personal protective equipment (PPE) and COVID-19 testing, and mental health. A 34-question survey was developed and distributed electronically to allied health professionals through listservs of professional organizations and social media groups. A total of 921 responses from allied health professionals in a variety of work settings were analyzed. The majority of allied health professionals had access to medical-grade PPE and agreed with their clinics decisions to stay open or closed. Private practices appeared to be the most negatively impacted with regards to employment in the form of pay reductions, furloughs, lay-offs, or the requirement of using paid time off. Importantly, 86% of all respondents, irrespective of employment status, reported feeling stressed with regards to changes in their work environment and transmission of the virus. However, levels of stress were dependent upon access to PPE and mental health resources. Specifically, those with access to mental health support reported lower stress levels than those without such access. These results highlight the need for continuous monitoring of mental health for allied health professionals in order to inform clinic and hospital policies for PPE and the development of brief interventions to mitigate adverse long-term mental health outcomes.

Cotrin, P., et al. (2020). "Healthcare Workers in Brazil during the COVID-19 Pandemic: A Cross-Sectional Online Survey." Inquiry **57**: 46958020963711.

Brazil is in a critical situation due to the COVID-19 pandemic. Healthcare workers that are in the front line face challenges with a shortage of personal protective equipment, high risk of contamination, low adherence to the social distancing measures by the population, low coronavirus testing with underestimation of cases, and also financial concerns due to the economic crisis in a developing country. This study compared the impact of COVID-19 pandemic among three categories of healthcare workers in Brazil: physicians, nurses, and dentists, about workload, income, protection, training, feelings, behavior, and level of concern and anxiety. The sample was randomly selected and a Google Forms questionnaire was sent by WhatsApp messenger. The survey comprised questions about jobs, income, workload, PPE, training for COVID-19 patient care, behavior and feelings during the pandemic. The number of jobs reduced for all healthcare workers in Brazil during the pandemic, but significantly more for dentists. The workload and income reduced to all healthcare workers. Most healthcare workers did not receive proper training for treating COVID-19 infected patients. Physicians and nurses were feeling more tired than usual. Most of the healthcare workers in all groups reported difficulties in sleeping during the pandemic. The healthcare workers reported a significant impact of COVID-19 pandemic in their income, workload and anxiety, with differences among physicians, nurses and dentists.

Cottrell, J., et al. (2020). "An operative barrier system for skull base and mastoid surgery: creating a safe operative theatre in the era of COVID-19." J Otolaryngol Head Neck Surg **49**(1): 71.

Within Neurotology, special draping systems have been devised for mastoid surgery recognizing that drilling of middle ear mucosa is an aerosol generating medical procedure (AGMP) which can place surgical teams at risk of COVID-19 infection. We provide a thorough description of a barrier system utilized in our practice, along with work completed by our group to better quantify its effectiveness. Utilization of a barrier system can provide near complete bone dust and droplet containment within the surgical field and prevent contamination of other healthcare workers. As this is an early system, further adaptations and national collaborations are required to ultimately arrive at a system that seamlessly integrates into the surgical suite. While these barrier systems are new, they are timely as we face a pandemic, and can play a crucial role in safely resuming surgery.

Coutureau, C., et al. (2021). "Does Copper Prevent Nosocomial Transmission of COVID-19?" J Am Med Dir Assoc **22**(1): 219-220.

Craig, S., et al. (2020). "Management of adult cardiac arrest in the COVID-19 era: consensus statement from the Australasian College for Emergency Medicine." Med J Aust **213**(3): 126-133.

INTRODUCTION: The global pandemic of coronavirus disease 2019 (COVID-19) has caused significant worldwide disruption. Although Australia and New Zealand have not been affected as much as some other countries, resuscitation may still pose a risk to health care workers and necessitates a change to our traditional approach. This consensus statement for adult cardiac arrest in the setting of COVID-19 has been produced by the Australasian College for Emergency Medicine (ACEM) and aligns with national and international recommendations. MAIN RECOMMENDATIONS: In a setting of low community transmission, most cardiac arrests are not due to COVID-19. Early defibrillation saves lives and is not considered an aerosol generating procedure. Compression-only cardiopulmonary resuscitation is thought to be a low risk procedure and can be safely initiated with the patient's mouth and nose covered. All other resuscitative procedures are considered aerosol generating and require the use of airborne personal protective equipment (PPE). It is important to balance the appropriateness of resuscitation against the risk of infection. Methods to reduce nosocomial transmission of COVID-19 include a physical barrier such as a towel or mask over the patient's mouth and nose, appropriate use of PPE, minimising the staff involved in resuscitation, and use of mechanical chest compression devices when available. If COVID-19 significantly affects hospital resource availability, the ethics of resource allocation must be considered. CHANGES IN MANAGEMENT: The changes outlined in this document require a significant adaptation for many doctors, nurses and paramedics. It is critically important that all health care workers have regular PPE and advanced life support training, are able to access in situ simulation sessions, and receive extensive debriefing after actual resuscitations. This will ensure safe, timely and effective management of the patients with cardiac arrest in the COVID-19 era.

Cramer, A. K., et al. (2020). "Analysis of SteraMist ionized hydrogen peroxide technology in the sterilization of N95 respirators and other PPE: a quality improvement study." medRxiv.

OBJECTIVE: The COVID-19 pandemic has led to widespread shortages of personal protective equipment (PPE) for healthcare workers, including filtering facepiece respirators (FFRs) such as N95 masks. These masks are normally intended for single use, but their sterilization and subsequent reuse could substantially mitigate a world-wide shortage. DESIGN: Quality assurance. SETTING: A sealed environment chamber installed in the animal facility of an academic medical center. INTERVENTIONS: One to five sterilization cycles using ionized hydrogen peroxide (iHP), generated by SteraMist equipment (TOMI; Frederick, MD). MAIN OUTCOME MEASURES: Personal protective equipment, including five N95 mask models from three manufacturers, were evaluated for efficacy of sterilization following iHP treatment (measured with bacterial spores in standard biological indicator assemblies). Additionally, N95 masks were assessed for their ability to efficiently filter particles down to 0.3um and for their ability to form an airtight seal using a quantitative fit test. Filtration efficiency was measured using ambient particulate matter at a university lab and an aerosolized NaCl challenge at a National Institute for Occupational Safety and Health (NIOSH) pre-certification laboratory. RESULTS: The data demonstrate that N95 masks sterilized using SteraMist iHP technology retain function up to five cycles, the maximum number tested to date. Some but not all PPE could also be sterilized using an iHP environmental chamber, but pre-treatment with a handheld iHP generator was required for semi-enclosed surfaces such as respirator hoses. CONCLUSIONS: A typical iHP environment chamber with a volume of ~80 m3 can treat ~7000 masks per day, as well as other items of PPE, making this an effective approach for a busy medical center.

Cramer, A. K., et al. (2021). "Analysis of SteraMist ionized hydrogen peroxide technology in the sterilization of N95 respirators and other PPE." Sci Rep **11**(1): 2051.

The COVID-19 pandemic has led to widespread shortages of personal protective equipment (PPE) for healthcare workers, including of N95 masks (filtering facepiece respirators; FFRs). These masks are intended for single use but their sterilization and subsequent reuse has the potential to substantially mitigate shortages. Here we investigate PPE sterilization using ionized hydrogen peroxide (iHP), generated by SteraMist equipment (TOMI; Frederick, MD), in a sealed environment chamber. The efficacy of sterilization by iHP was assessed using bacterial spores in biological indicator assemblies. After one or more iHP treatments, five models of N95 masks from three manufacturers were assessed for retention of function based on their ability to form an airtight seal (measured using a quantitative fit test) and filter aerosolized particles. Filtration testing was performed at a university lab and at a National Institute for Occupational Safety and Health (NIOSH) pre-certification laboratory. The data demonstrate that N95 masks sterilized using SteraMist iHP technology retain filtration efficiency up to ten cycles, the maximum number tested to date. A typical iHP environment chamber with a volume of ~ 80 m(3) can treat ~ 7000 masks and other items (e.g. other PPE, iPADs), making this an effective approach for a busy medical center.

Crespo, J., et al. (2020). "Massive impact of coronavirus disease 2019 pandemic on gastroenterology and hepatology departments and doctors in Spain." J Gastroenterol Hepatol.

BACKGROUND AND AIM: Significant human and material resources have been diverted to coronavirus disease 2019 (COVID-19). Healthcare workers are at high risk of severe acute respiratory syndrome coronavirus 2 (SARS-CoV-2) infection. We assess the impact of the COVID-19 pandemic on gastroenterology and hepatology departments and specialists in Spain. METHODS: This study involves a nationwide survey addressing the impact of COVID-19 on resources, procedures, and physicians of gastroenterology and hepatology departments in 81 hospitals representative of the Spanish National Health Service. RESULTS: Overall, 41.8% of hospital beds and 40.7% of gastroenterology and hepatology beds were allocated to COVID-19 patient care, as well as 24.8% of gastroenterologists and 58.3% of residents. Outpatient visits, abdominal ultrasounds, and endoscopies were reduced by 81.8-91.9%. Nine large university hospitals had 75% and 89% reductions in therapeutic endoscopies and hepatocellular carcinoma surgery, respectively, with cancelation of elective liver transplant and transjugular intrahepatic portosystemic shunt. Prevalence of infected physicians was 10.6% and was dependent on regional population incidence (r = 0.74, P = 0.001), with 11% hospitalized and one physician dying. Up to 63.4% of physicians may have been infected before or shortly after Spain entered lockdown, 57% of them having recently performed endoscopies. Adequate protection was acknowledged in > 80% hospitals, but only 2.9% performed regular SARS-CoV-2 testing. CONCLUSIONS: The impact of the COVID-19 pandemic on healthcare delivery has been massive. A wave of gastroenterology-related complications is expected because of resource diversion. Gastroenterologists have a high prevalence of infection, although they may have been infected during a first phase of lower awareness and protection. Regular SARS-CoV-2 screening, adequate protection, and quick reorganization of healthcare resources are still needed.

Crotty, T. J., et al. (2021). "The psychological impact of COVID-19 on ear, nose and throat (ENT) specialists." Ir J Med Sci: 1-7.

INTRODUCTION: Ear, nose and throat (ENT) specialists are a high-risk group for COVID-19. Although the implications of SARS-CoV-2 infection on physical health are well-documented, the psychological impact is frequently overlooked. AIMS: The aim of this study was to gauge the psychological impact of COVID-19 on ENT specialists in Ireland. METHODS: A national, cross-sectional, web-based survey was distributed to ENT specialists during the peak of the COVID-19 pandemic (21(st) May 2020-21(st) June 2020). The questionnaire collected sociodemographic and COVID-19 related data. The GAD-7 was utilized to measure symptoms of clinically significant anxiety disorder. RESULTS: Thirty-eight ENT specialists (M/F:24/12, median age, 38.7 years (23-60 years)) completed the survey. About 34% (n = 13) of participants screened positive for an anxiety disorder, of which 2 (5%) had moderate symptoms. The majority of participants (n = 32, 84%) felt ENT specialists had increased exposure to SARS-CoV-2 compared with other medical specialties. Additionally, 32% (n = 12) felt incapable of protecting themselves from infection. An encouraging proportion of ENT specialists (n = 22, 58%) were aware of psychological support available from national and institutional organizations. CONCLUSIONS: The long-term psychological sequelae of COVID-19 will be felt as the physical burden eases. As we adjust to new normalities, ENT surgeons must be conscientious of the mental health issues that arise from the working environment. Sources of anxiety emanated from a lack of control over infection risk, increased vulnerability to COVID-19 relative to other specialties and the implications this has for ENT specialists and their families. Future interventions must focus on increasing awareness of the available psychological support services for our healthcare workers.

Cuadrado-Lavín, A., et al. (2020). "Controlled, double-blind, randomized trial to assess the efficacy and safety of hydroxychloroquine chemoprophylaxis in SARS CoV2 infection in healthcare personnel in the hospital setting: A structured summary of a study protocol for a randomised controlled trial." Trials **21**(1): 472.

BACKGROUND: SARS-CoV-2 infection presents a high transmission in the group of health professionals in Spain (12-15% infected). Currently there is no accepted chemoprophylaxis but hydroxychloroquine (HDQ) is known to inhibit the coronavirus in vitro. Our hypothesis is that oral administration of hydroxychloroquine to healthcare professionals can reduce the incidence and prevalence of infection as well as its severity in this group. METHODS: Design: Prospective, single center, double blind, randomised, controlled trial (RCT). PARTICIPANTS: Adult health-care professionals (18-65 years) working in areas of high exposure and high risk of transmission of SARS-COV-2 (COVID areas, Intensive Care Unit -ICUs-, Emergency, Anesthesia and all those performing aerosol-generating procedures) will be included. Exclusion criteria include previous infection with SARS CoV2 (positive SARS-CoV-2 PCR or IgG serology), pregnancy or lactation, any contraindication to hydroxychloroquine or evidence of unstable or clinically significant systemic disease. INTERVENTIONS: Patients will be randomized (1:1) to receive once-daily oral Hydroxychloroquine 200mg for two months (HC group) or placebo (P group) in addition to the protective measures appropriate to the level of exposure established by the hospital. A serological evaluation will be carried out every 15 days with PCR in case of seroconversion, symptoms or risk exposure. Primary outcome is the percentage of subjects presenting infection (seroconversion and/or PCR +ve) by the SARS-Cov-2 virus during the observation period. Additionally, both the percentage of subjects in each group presenting Pneumonia with severity criteria (Curb 65 ≥2) and that of subjects requiring admission to ICU will be determined. DISCUSSION: While awaiting a vaccine, hygiene measures, social distancing and personal protective equipment are the only primary prophylaxis measures against SARS-CoV-2, but they have not been sufficient to protect our healthcare professionals. Some evidence of the in vitro efficacy of hydroxychloroquine against this virus is known, along with some clinical data that would support the study of this drug in the chemoprophylaxis of infection. However, there are still no data from controlled clinical trials in this regard. If our hypothesis is confirmed, hydroxychloroquine can help professionals fight this infection with more guarantees. PARTICIPANTS: This is a single-center study that will be carried out at the Marqués de Valdecilla University Hospital. 450 health professionals working at the Hospital Universitario Marqués de Valdecilla in areas of high exposure and high risk of transmission of SARS COV2 (COVID hospital areas, Intensive Care Unit, Emergency, Anesthesia and all those performing aerosol-generating procedures) will be included. INCLUSION CRITERIA: 1) Health professionals aged between 18 and 65 years (inclusive) at the time of the first screening visit; 2) They must provide signed written informed consent and agree to comply with the study protocol; 3) Active work in high exposure areas during the last two weeks and during the following weeks. EXCLUSION CRITERIA: 1) Previous infection with SARS CoV2 (positive coronavirus PCR or positive serology with SARS Cov2 negative PCR and absence of symptoms); 2) Current treatment with hydroxychloroquine or chloroquine; 3) Hypersensitivity, allergy or any contraindication for taking hydroxychloroquine, in the technical sheet; 4) Previous or current treatment with tamoxifen or raloxifene; 5) Previous eye disease, especially maculopathy; 6) Known heart failure (Grade III to IV of the New York Heart Association classification) or prolonged QTc; 7) Any type of cancer (except basal cell) in the last 5 years; 6) Refusal to give informed consent; 8) Evidence of any other unstable or clinically significant untreated immune, endocrine, hematological, gastrointestinal, neurological, neoplastic or psychiatric illness; 9) Antibodies positive for the human immunodeficiency virus; 10) Significant kidney or liver disease; 11) Pregnancy or lactation. INTERVENTION AND COMPARATOR: Two groups will be analyzed with a 1: 1 randomization rate. 1)Intervention: (n = 225): One 200 mg hydroxychloroquine sulfate coated tablet once daily for two months.2)Comparator (control group) (n = 225): One hydroxychloroquine placebo tablet (identical to that of the drug) once daily for two months MAIN OUTCOMES: The primary outcome of this study will be to evaluate: number and percentage of healthcare personnel presenting symptomatic and asymptomatic infection (see "Diagnosis of SARS CoV2 infection" below) by the SARS-Cov2 virus during the study observation period (8 weeks) in both treatment arms;number and percentage of healthcare personnel in each group presenting with Pneumonia with severity criteria (Curb 65 ≥2) and number and percentage of healthcare personnel requiring admission to the Intensive Care Unit (ICU) in both treatment arms. DIAGNOSIS OF SARS COV2 INFECTION: Determination of IgA, IgM and IgG type antibodies against SARS-CoV-2 using the Anti-SARS-CoV-2 ELISA kit (EUROIMMUN Medizinische Labordiagnostika AG, Germany) every two weeks. In cases of seroconversion, a SARS-CoV-2 PCR will be performed to rule out / confirm an active infection (RT-PCR in One Step: RT performed with mastermix (Takara) and IDT probes, following protocol published and validated by the CDC Evaluation of COVID-19 in case of SARS-CoV-2 infection RANDOMISATION: Participants will be allocated to intervention and comparator groups according to a balanced randomization scheme (1: 1). The assignment will be made through a computer-generated numeric sequence for all participants BLINDING (MASKING): Both participants and investigators responsible for recruiting and monitoring participants will be blind to the assigned arm. NUMBERS TO BE RANDOMISED (SAMPLE SIZE): Taking into account the current high prevalence of infection in healthcare personnel in Spain (up to 15%), to detect a difference equal to or greater than 8% in the percentage estimates through a two-tailed 95% CI, with a statistical power of 80% and a dropout rate of 5%, a total of 450 participants will need to be included (250 in each arm). TRIAL STATUS: The protocol approved by the health authorities in Spain (Spanish Agency for Medicines and Health Products "AEMPS") and the Ethics and Research Committee of Cantabria (CEIm Cantabria) corresponds to version 1.1 of April 2, 2020. Currently, recruitment has not yet started, with the start scheduled for the second week of May 2020. TRIAL REGISTRATION: Eudra CT number: 2020-001704-42 (Registered on 29 March 2020) FULL PROTOCOL: The full protocol is attached as an additional file, accessible from the Trials website (Additional file 1). In the interest in expediting dissemination of this material, the familiar formatting has been eliminated; this Letter serves as a summary of the key elements of the full protocol. The study protocol has been reported in accordance with the Standard Protocol Items: Recommendations for Clinical Interventional Trials (SPIRIT) guidelines (Additional file 2).

da Silva Brandão, E., et al. (2020). "Prevención de lesiones cutáneas causadas por productos y equipos de protección personal durante COVID-19: revisión de alcance." J Wound Care **29**(LatAm sup 3): 6-12.

OBJECTIVE: Identify, summarise and present key recommendations published in the literature to prevent skin damage in health professionals as a result of personal protective equipment (PPE) during the covid-19 pandemic. METHOD: A scoping review, based on the Joanna Briggs Institute guidelines, was carried out between December 2019 and May 2020. The studies included male and female health professionals, who wore PPE during the global pandemic, in any given scenario. Headings and abstracts were analysed. Two independent investigators reviewed the full text. RESULTS: Eight publications were included. All of them focused on preventing pressure ulcers (PU) caused by PPE. Hygiene, hydration and the use of skin barriers were highlighted as preventive measures. There were different views around the type of humectant/skin barrier recommended to avoid skin damage. CONCLUSION: More studies identifying the most suitable strategies to maintain health professionals' skin integrity during the covid-19 pandemic are needed.

da Silva, F. C. T. and C. P. Barbosa (2021). "The impact of the COVID-19 pandemic in an intensive care unit (ICU): Psychiatric symptoms in healthcare professionals." Prog Neuropsychopharmacol Biol Psychiatry **110**: 110299.

INTRODUCTION: During the COVID-19 pandemic period, the structure of the Intensive Care Unit (ICU) activities changed fast. It was observed that the mental and physical health of the frontline workers reached levels of extreme clinical and psychological concern. OBJECTIVE: Understand the impact that COVID-19 is having on the front-line clinical team in the ICU environment, as well as reveal what proposals are being made to mitigate the clinical and psychological impacts that this group experiences. METHOD: A systematic review was made following the PRISMA protocol (Preferred Reporting Items for Systematic Reviews and Meta-Analysis). We included any type of study on health workers during the COVID-19 pandemic, with results about their mental health. We were, therefore, interested in quantitative studies examining the prevalence of problems and effects of interventions, as well as qualitative studies examining experiences. We had no restrictions related to study design, methodological quality or language. RESULTS: Twenty-one studies reported on the urgent need for interventions to prevent or reduce mental health problems caused by COVID-19 among health professionals in ICU. Eleven studies demonstrated possibilities for interventions involving organizational adjustments in the ICU, particularly linked to emotional conflicts in the fight against COVID-19. CONCLUSION: The disproportion between the need for technological supplies of intensive care medicine and their scarcity promotes, among many factors, high rates of psychological distress. Anxiety, irritability, insomnia, fear and anguish were observed during the pandemic, probably related to extremely high workloads and the lack of personal protective equipment.

da Silva, F. C. T. and M. L. R. Neto (2021). "Psychiatric symptomatology associated with depression, anxiety, distress, and insomnia in health professionals working in patients affected by COVID-19: A systematic review with meta-analysis." Prog Neuropsychopharmacol Biol Psychiatry **104**: 110057.

BACKGROUND: Health professionals are key personnel to containing infectious diseases like COVID-19. In the face of long work shifts (that reach 16 h per day on average), the risk of getting infected by a high-infectious disease and the lack of enough biological protection measures, mental suffering among health professionals suddenly became evident. METHOD: We carried out an updated meta-analysis to investigate the psychiatric impacts on health professionals in the face of the physical and psychological conditions to which they are subjected due to the high demands of the COVID-19 pandemic. Papers were researched in four databases from December 2019 to April 2020. In total, eight papers were included in the study. RESULTS: Health professionals working to fight COVID-19 are being more severely affected by psychiatric disorders associated with depression, anxiety, distress and insomnia, stress, and indirect traumatization than other occupational groups. No significant differences were observed in the publication bias. CONCLUSION: There is a strong association between health professionals and COVID-19 in terms of psychiatric repercussions. Our meta-analysis showed that health professionals have a higher level of indirect traumatization, in which the level of damage exceeds psychological and emotional tolerance and indirectly results in psychological abnormalities. The incidence of obsessive-compulsive traces and somatizations was higher in situations involving front-line professionals.

D'Accolti, M., et al. (2020). "SARS-CoV-2 RNA contamination on surfaces of a COVID-19 ward in a hospital of Northern Italy: what risk of transmission?" Eur Rev Med Pharmacol Sci **24**(17): 9202-9207.

OBJECTIVE: SARS-CoV-2 can reportedly exist on inanimate surfaces for a long duration, but there is limited data available from Italian COVID-19 hospital wards, especially for non-intensive care units hosting patients that do not require mechanical ventilation. Identification of the extent of environmental contamination can help in understanding possible virus transmission routes, limit hospital infections and protect healthcare workers. Thus, we investigated virus contamination on surfaces of the acute COVID-19 ward of an Italian hospital. MATERIALS AND METHODS: Ward surfaces, including four points inside and six points outside the patients' rooms were sampled by swabs, seven hours after routine sanitation. To minimize the risk of underestimation of virus detection, two different sensitive molecular methods were used comparatively, and specific internal controls were added to enhance the efficiency of all the analysis steps. RESULTS: SARS-CoV-2 contamination was detected in only three out of all the collected samples, i.e., on two floors and one-bathroom sink, likely reflecting aerosol and saliva contamination, respectively. The overall level of contamination was low, and the floors exhibited a very low level of SARS-CoV-2 presence, evidenced by only one of the two methods used. CONCLUSIONS: The existence of SARS-CoV-2 on hospital surfaces may be limited, although it was reported to persist for a longer duration on surfaces under controlled laboratory conditions. Thus, effective transmission of SARS-CoV-2 by surfaces/fomites within the hospital ward may be a rare event. However, the results highlight the importance of assessing method sensitivity and including controls when investigating low-level virus contamination so as to avoid the risk of underestimation of virus presence.

Dacic, S. D., et al. (2020). "Dental care during the Covid-19 pandemic - To treat or not to treat?" J Infect Dev Ctries **14**(10): 1111-1116.

The recent spread of COVID-19 presents a huge public health concern. Clinical presentations of COVID-19 range from asymptomatic cases to severe pneumonia that can lead to death. Drastic measures were necessary to prevent the disease from spreading and protect the most vulnerable groups in the general population. The rapid reorganization of the healthcare system and great efforts made by medical staff were needed to admit to hospitals and then treat a progressively growing number of patients. The predominant route of virus transmission is through direct contact with an infected individual or respiratory droplets, therefore, all dental procedures with aerosol-formation pose an extremely high risk for the spread of infection. The aim of this article is to provide an overview of the current epidemiological situation, routes of transmission, and specific recommendations for dental practices including patient screening and triage, infection control, and treatment protocols. In this situation, it is essential that all dental healthcare workers make wise clinical decisions and educate themselves and their patients on how to prevent the spread of infection.

Dacosta-Urbieta, A., et al. (2020). "Seroprevalence of SARS-CoV-2 Among Pediatric Healthcare Workers in Spain." Front Pediatr **8**: 547.

Spain is one of the countries most severely affected by the SARS-CoV-2 pandemic, with almost 190,000 cases as of April 18, 2020. As healthcare workers (HCW) are one of the groups hardest hit by the infection, it is important to know the seroprevalence of antibodies against SARS-CoV-2 in pediatric departments. We performed 175 immunoglobulin (Ig)M and IgG immunochromatographic rapid tests in the personnel working at the Pediatric Department of the Hospital Clínico Universitario of Santiago de Compostela (Spain), including pediatricians, residents, nurses, and other staff, on days 31-33 since the lockdown started. Seven out of the 175 tests were positive, including four for IgM and three for IgG, leading to a seroprevalence of 4.0% (95% CI: 1.1-6.9%). Only one of them had symptoms at the time of testing (sore throat). All seropositive cases yielded negative RT-PCR of the upper and lower respiratory tract. This is the first SARS-CoV-2 serological survey among HCWs reported in Spain. Notwithstanding the test limitations, our results reveal that personal protection policy and lockdown measures have been effective to limit population exposure. The low seroprevalence rate poses a significant challenge for the next strategic steps of pandemic control.

Dadlani, S. (2020). "SARS-CoV-2 Transmission in a Dental Practice in Spain: After the Outbreak." Int J Dent **2020**: 8828616.

The World Health Organization declared a pandemic on March 11, 2020, due to a virus named SARS-CoV-2 discovered in Wuhan, China, in December 2019. Many countries have been hit hard including Spain, with the highest number of healthcare workers being infected (>50,000). A lack of personal protective equipment and protocols at the time of the outbreak led to many fatalities. Although few of these healthcare workers are dental professionals, this community required protective measures as well. Fortunately, there are no reported cases of SARS-CoV-2 transmission in dental practices. Dental professionals were advised only to treat dental emergencies, and such cases were screened via telephone to maintain social distancing. Nevertheless, new protocols and measures are needed as dental professionals return to normal practice after weeks of confinement in many countries. Relatively, few articles have discussed the management of dental practice during the SARS-CoV-2 with no known articles on postpandemic outbreak guidelines. Though some protocols and measures are the same, there are also many differences. Here, we describe protocols and measures for dental practice in Spain in accordance with the Spanish Health Ministry.

Daghmouri, M. A., et al. (2020). "Personal Protective Equipment: A Cross-Sectional Study in Frontline Healthcare Workers During COVID-19 Outbreak in Tunisia." Tunis Med **98**(8-9): 633-638.

BACKGROUND: For frontline healthcare workers (FHW) who are working with Coronavirus Disease-19 (COVID-19) patients, PPE is currently considered as one of the most discussed topics. Recommendation from international organizations concerning the use of PPE are broadly consistent but equipment use is not. No previous studies examined the use of PPE in Tunisia. AIM: Evaluate the availability of personal protective equipment(PPE) in addition to the reality and perception regarding personal safety in workplace. METHODS: We carried out a cross-sectional survey from 15th April to 5th May 2020 across public and private institutions in Tunisia. A 33-item structured questionnaire was developed and administered to FHWs. RESULTS: We received 723 responses. We found that there was a likely overuse of PPE in addition to a high rate of side effects caused by PPE. CONCLUSIONS: Additional training in PPE use might be useful, and sessional work should be considered to decrease the side effects associated with PPE use.

Dalu, D., et al. (2021). "A proposal of a "ready to use" COVID-19 control strategy in an Oncology ward: Utopia or reality?" Crit Rev Oncol Hematol **157**: 103168.

The Coronavirus Disease-2019 (COVID-19) pandemic is spreading in Italy and Lombardy is one of the most affected region. Cancer patients are higher risk of complication from COVID-19 complications; therefore they should be protected from contagion while still ensuring access to cancer care. The aim of this article is to suggest a strategy to reorganize hospital spaces and Healthcare Professionals (HCPs) staff in order to avoid COVID-19 nosocomial infection in an Oncology ward. SARS-CoV-2 is primarily transmitted through respiratory droplets and by contact. We speculated that precautions against droplet and contact transmission should be the proper way to preserve ward from COVID-19. The essence of our protocol involves: triage outside of the ward, identification of risk zones, traffic control, surveillance of all the involved subjects. Whoever attends the ward must follow the general risk prevention and mitigation measures. The application of this practical strategy can contribute to breaking the cycle of community-hospital-community transmission.

Damiani, G., et al. (2021). "Mask-induced Koebner phenomenon and its clinical phenotypes: A multicenter, real-life study focusing on 873 dermatological consultations during COVID-19 pandemics." Dermatol Ther **34**(2): e14823.

During COVID-19 pandemic, wearing masks for prevention became mandatory but evidence suggest that is also detrimental for skin. Although facial dermatoses due to masks increase in both healthcare workers and general population, a pathogenetic hypothesis remains still elusive. We aimed to evaluate the prevalence of dermatological consultations due to Koebner triggered dermatoses In this prospective, multicenter, real life study carried out in Italy from March 11th to December 11th 2020 during COVID-19 pandemics, dermatological consultations (in-person and telemedicine) to study the prevalence of Koebner (KB) phenomenon due to masks were evaluated. Boyd and Nelder classification was adopted for Koebner phenomenon and Bizzozero's for KB intensity. A total of 229/873 (26.2%) dermatological consultations were KB triggered dermatoses and lesions were located in mask-covered ear area (76 [33.2%]), malar area (73 [31.8%]), perioral area (53 [23.1%]), and nose (27 [11.8%]). The first KB category grouped 142 patients (psoriasis, vitiligo, maskne, and mask rosacea), the second one 24 (warts, molluscum contagiosum, and impetigo), the third one 46 (atopic dermatitis), and the fourth one 17 (eczema). Among previously KB negative psoriatic patients that became KB positive, 9/13 (69.2%) had discontinued or modified the prescribed antipsoriatic treatment. Mask-related Koebner phenomenon is an important clinical sign to orient clinician's therapeutic protocols during COVID-19 pandemic, especially in patients with psoriasis.

Danet Danet, A. (2021). "Psychological impact of COVID-19 pandemic in Western frontline healthcare professionals. A systematic review." Med Clin (Engl Ed).

The aim of this study was to assess the psychological impact among healthcare workers who stand in the frontline of the SARS-CoV-2 crisis and to compare it with the rest of healthcare professionals, by means of a systematic review of Western publications. The systematic review was carried out in PubMed, Scopus and Web of Science databases and 12 descriptive studies were reviewed. The European and American quantitative studies reported moderate and high levels of stress, anxiety, depression, sleep disturbance and burnout, with diverse coping strategies and more frequent and intense symptoms among women and nurses, without conclusive results by age. In the first line of assistance the psychological impact was greater than in the rest of the health professionals and in the Asian area. It is necessary to go deeper into the emotional experiences and professional needs for emotional support in order to design effective interventions for protection and help.

Danet Danet, A. (2021). "Psychological impact of COVID-19 pandemic in Western frontline healthcare professionals. A systematic review." Med Clin (Barc).

The aim of this study was to assess the psychological impact among healthcare workers who stand in the frontline of the SARS-CoV-2 crisis and to compare it with the rest of healthcare professionals, by means of a systematic review of Western publications. The systematic review was carried out in PubMed, Scopus and Web of Science databases and 12 descriptive studies were reviewed. The European and American quantitative studies reported moderate and high levels of stress, anxiety, depression, sleep disturbance and burnout, with diverse coping strategies and more frequent and intense symptoms among women and nurses, without conclusive results by age. In the first line of assistance the psychological impact was greater than in the rest of the health professionals and in the Asian area. It is necessary to go deeper into the emotional experiences and professional needs for emotional support in order to design effective interventions for protection and help.

eng

Dang, A. K., et al. (2020). "Evidence of COVID-19 Impacts on Occupations During the First Vietnamese National Lockdown." Ann Glob Health **86**(1): 112.

BACKGROUND: Although "social isolation" protects the life and health of Vietnamese citizens from the adverse effects of the COVID-19 pandemic, it also triggers massive reductions in the economic activities of the country. OBJECTIVE: our study aimed to identify negative impacts of COVID-19 on occupations of Vietnamese people during the first national lockdown, including the quality and quantity of jobs as well as adverse problems at work due to COVID-19. METHODS: A cross-sectional study using web-based platforms was conducted during the first time of social isolation in Vietnam at the beginning of April 2020. We utilized a respondent-driven sampling technique to select 1423 respondents from 63 cities and provinces over Vietnam. Exploratory factor analysis (EFA) was used to define sub-domains of perceived impacts of COVID-19 on occupations. FINDINGS: Approximately two-thirds of respondents reported decreases in their income (61.6%), and 28.2% reported that their income deficit was 40% and above. The percentage of female individuals having decreased revenue due to COVID-19 was higher than that of male respondents (65.2% and 54.7%, respectively). "Worry that colleagues exposed to COVID-19 patients" and "Being alienated because employment-related to COVID-19" accounted for the highest score in each factor. Compared to healthcare workers, being self-employed/unemployed/retired were less likely to suffer from "Increased workload and conflicts due to COVID-19" and "Disclosure and discrimination related to COVID-19 work exposure." CONCLUSION: Our study revealed a drastic reduction in both the quality and quantity of working, as well as the increased fear and stigmatization of exposure to COVID-19 at workplaces. Health protection and economic support are immediate targets that should be focused on when implementing policies and regulations.

Darwiche, K., et al. (2020). "[Recommendations for Performing Bronchoscopy in Times of the COVID-19 Pandemic]." Pneumologie **74**(5): 260-262.

COVID-19, caused by coronavirus SARS-CoV-2 is a new and ongoing infectious disease affecting healthcare systems worldwide. Healthcare worker are at high risk for COIVD-19 and many have been infected or even died in countries severely affected by COVID-19 like China or Italy. Bronchoscopy causes cough and aerosol production and has to be considered a significant risk for the staff to get infected. Particular recommendations should guide to prevent spreading COVID-19 and to protect healthcare worker when performing a bronchoscopy.

Das, A., et al. (2020). "Two-drape closed pocket technique: minimizing aerosolization in mastoid exploration during COVID-19 pandemic." Eur Arch Otorhinolaryngol **277**(12): 3529-3532.

BACKGROUND: Mastoidectomy is associated with extensive bone-drilling which makes it a major aerosol generating procedure. Considering the ongoing COVID-19 global pandemic, it is essential to devise methods to minimize aerosolization and hence ensure safety of the healthcare workers during the operative procedure. METHODS: Two disposable surgical drapes are used to create a closed pocket prior to commencement of mastoid bone-drilling. This limits aerosolization of bone-dust in the external operating theatre environment. CONCLUSION: Two-drape closed pocket technique is an easy, cost-effective and safe method to limit aerosolization of tissue particles during mastoidectomy.

Davey, S. L., et al. (2021). "Heat stress and PPE during COVID-19: impact on healthcare workers' performance, safety and well-being in NHS settings." J Hosp Infect **108**: 185-188.

Personal protective equipment (PPE) can potentiate heat stress, which may have a negative impact on the wearer's performance, safety and well-being. In view of this, a survey was distributed to healthcare workers (HCWs) required to wear PPE during the coronavirus disease 2019 pandemic in the UK to evaluate perceived levels of heat stress and its consequences. Respondents reported experiencing several heat-related illness symptoms, and heat stress impaired both cognitive and physical performance. The majority of respondents stated that wearing PPE made their job more difficult. These, and additional, responses suggest that modification to current working practices is required urgently to improve the resilience of HCWs to wearing PPE during pandemics.

Davido, B., et al. (2021). "The first wave of COVID-19 in hospital staff members of a tertiary care hospital in the greater Paris area: A surveillance and risk factors study." Int J Infect Dis **105**: 172-179.

INTRODUCTION: Understanding how hospital staff members (HSMs), including healthcare workers, acquired severe acute respiratory syndrome coronavirus 2 (SARS-CoV-2) during the first wave can guide the control measures in the current second wave in Europe. METHODS: From March 5 to May 10, 2020, the Raymond-Poincaré Hospital held a weekday consultation for HSMs for PCR testing. HSMs were requested to complete a questionnaire on their potential exposure to SARS-CoV-2. RESULTS: Of 200 HSMs screened, 70 tested positive for SARS-CoV-2. Ninety-nine HSMs completed the questionnaire of whom 28 tested positive for SARS-CoV-2. In the multivariable analysis, age of ≥44 years (aOR = 5.2, 95% CI [1.4-22.5]) and not systematically using a facemask when caring for a patient (aOR = 13.9, 95% CI [1.8-293.0]) were significantly associated with SARS-CoV-2 infection. Working in a COVID-19-dedicated ward (aOR = 0.7, 95% CI [0.2-3.2]) was not significantly associated with infection. Community-related exposure in and outside the hospital, hospital meetings without facemasks (aOR = 21.3, 95% CI [4.5-143.9]) and private gatherings (aOR = 10, 95% CI [1.3-91.0]) were significantly associated with infection. CONCLUSIONS: Our results support the effectiveness of barrier precautions and highlight in-hospital infections not related to patient care and infections related to exposure in the community. Protecting HSMs against COVID-19 is crucial in fighting the second wave of the epidemic.

Day, A. T., et al. (2020). "Head and neck oncology during the COVID-19 pandemic: Reconsidering traditional treatment paradigms in light of new surgical and other multilevel risks." Oral Oncol **105**: 104684.

The COVID-19 pandemic demands reassessment of head and neck oncology treatment paradigms. Head and neck cancer (HNC) patients are generally at high-risk for COVID-19 infection and severe adverse outcomes. Further, there are new, multilevel COVID-19-specific risks to patients, surgeons, health care workers (HCWs), institutions and society. Urgent guidance in the delivery of safe, quality head and neck oncologic care is needed. Novel barriers to safe HNC surgery include: (1) imperfect presurgical screening for COVID-19; (2) prolonged SARS-CoV-2 aerosolization; (3) occurrence of multiple, potentially lengthy, aerosol generating procedures (AGPs) within a single surgery; (4) potential incompatibility of enhanced personal protective equipment (PPE) with routine operative equipment; (5) existential or anticipated PPE shortages. Additionally, novel, COVID-19-specific multilevel risks to HNC patients, HCWs and institutions, and society include: use of immunosuppressive therapy, nosocomial COVID-19 transmission, institutional COVID-19 outbreaks, and, at some locations, societal resource deficiencies requiring health care rationing. Traditional head and neck oncology doctrines require reassessment given the extraordinary COVID-19-specific risks of surgery. Emergent, comprehensive management of these novel, multilevel surgical risks are needed. Until these risks are managed, we temporarily favor nonsurgical therapy over surgery for most mucosal squamous cell carcinomas, wherein surgery and nonsurgical therapy are both first-line options. Where surgery is traditionally preferred, we recommend multidisciplinary evaluation of multilevel surgical-risks, discussion of possible alternative nonsurgical therapies and shared-decision-making with the patient. Where surgery remains indicated, we recommend judicious preoperative planning and development of COVID-19-specific perioperative protocols to maximize the safety and quality of surgical and oncologic care.

D'Cruz, L. (2020). "PPE or not PPE - that is the question." Br Dent J **228**(10): 753-754.

The world is in the middle of the COVID-19 pandemic and healthcare workers as well as dentists are having to make every difficult decision about their responsibilities in caring for their patients. Compounding this is the shortage of personal protective equipment (PPE), which makes patient-centred care ethically more challenging. Our first response to these ethical challenges should be to start with ourselves to make sure we are safe before we think about the patients. This is not the approach we would normally adopt when treating our patients outside of a pandemic.

De Georgeo, M. R., et al. (2021). "Containing SARS-CoV-2 in hospitals facing finite PPE, limited testing, and physical space variability: Navigating resource constrained enhanced traffic control bundling." J Microbiol Immunol Infect **54**(1): 4-11.

The COVID-19 outbreak has led to a focus by public health practitioners and scholars on ways to limit spread while facing unprecedented challenges and resource constraints. Recent COVID-19-specific enhanced Traffic Control Bundling (eTCB) recommendations provide a cogent framework for managing patient care pathways and reducing health care worker (HCW) and patient exposure to SARS-CoV-2. eTCB has been applied broadly and has proven to be effective in limiting fomite and droplet transmissions in hospitals and between hospitals and the surrounding community. At the same time, resource constrained conditions involving limited personal protective equipment (PPE), low testing availability, and variability in physical space can require modifications in the way hospitals implement eTCB. While eTCB has come to be viewed as a standard of practice, COVID-19 related resource constraints often require hospital implementation teams to customize eTCB solutions. We provide and describe a cross-functional, collaborative on-the-ground adaptive application of eTCB initially piloted at two hospitals and subsequently reproduced at 16 additional hospitals and health systems in the US to date. By effectively facilitating eTCB deployment, hospital leaders and practitioners can establish clearer 'zones of risk' and related protective practices that prevent transmission to HCWs and patients. We outline key insights and recommendations gained from recent implementation under the aforementioned constraints and a cross-functional team process that can be utilized by hospitals to most effectively adapt eTCB under resource constraints.

De Kock, J. H., et al. (2021). "A rapid review of the impact of COVID-19 on the mental health of healthcare workers: implications for supporting psychological well-being." BMC Public Health **21**(1): 104.

BACKGROUND: Health and social care workers (HSCWs) have carried a heavy burden during the COVID-19 crisis and, in the challenge to control the virus, have directly faced its consequences. Supporting their psychological wellbeing continues, therefore, to be a priority. This rapid review was carried out to establish whether there are any identifiable risk factors for adverse mental health outcomes amongst HSCWs during the COVID-19 crisis. METHODS: We undertook a rapid review of the literature following guidelines by the WHO and the Cochrane Collaboration's recommendations. We searched across 14 databases, executing the search at two different time points. We included published, observational and experimental studies that reported the psychological effects on HSCWs during the COVID-19 pandemic. RESULTS: The 24 studies included in this review reported data predominantly from China (18 out of 24 included studies) and most sampled urban hospital staff. Our study indicates that COVID-19 has a considerable impact on the psychological wellbeing of front-line hospital staff. Results suggest that nurses may be at higher risk of adverse mental health outcomes during this pandemic, but no studies compare this group with the primary care workforce. Furthermore, no studies investigated the psychological impact of the COVID-19 pandemic on social care staff. Other risk factors identified were underlying organic illness, gender (female), concern about family, fear of infection, lack of personal protective equipment (PPE) and close contact with COVID-19. Systemic support, adequate knowledge and resilience were identified as factors protecting against adverse mental health outcomes. CONCLUSIONS: The evidence to date suggests that female nurses with close contact with COVID-19 patients may have the most to gain from efforts aimed at supporting psychological well-being. However, inconsistencies in findings and a lack of data collected outside of hospital settings, suggest that we should not exclude any groups when addressing psychological well-being in health and social care workers. Whilst psychological interventions aimed at enhancing resilience in the individual may be of benefit, it is evident that to build a resilient workforce, occupational and environmental factors must be addressed. Further research including social care workers and analysis of wider societal structural factors is recommended.

de la Reza, M. T., et al. (2020). "Emergency Surgery in Urology during the COVID-19 Pandemic." Int Braz J Urol **46**(suppl.1): 201-206.

Proposal: To highlight the indications for emergency surgery during the 2019 Coronavirus pandemic (COVID-19) that support recommendations published in mid-March 2020 by the American Confederation of Urology on its website. MATERIALS AND METHODS: A bibliographic search was conducted in PubMed and Cochrane Library to perform a non-systematic review, using key words: Urology, Emergency and COVID-19, to determine recommendations for patients that should receive emergency care due to urological pathology. RESULTS: The main recommendations and protocols in the management of different urological emergencies during the COVID-19 pandemic are reviewed and discussed. CONCLUSIONS: We are living a new condition with the COVID-19 pandemic, which obliges urologists to conform to the guidelines that appear on a daily basis formulated by multidisciplinary surgical groups to manage urological emergencies. Consequently, in this time of health crisis, we must adapt to the resources available, implementing all biosecurity measures to protect patients and all health personnel who are in charge of patient management.

de Santiago, J., et al. (2020). "COVID-19: gynecologic cancer surgery at a single center in Madrid." Int J Gynecol Cancer **30**(8): 1108-1112.

OBJECTIVES: While numerous medical facilities have been forced to suspend oncological surgery due to system overload, debate has emerged on using non-surgical options on cancer cases during the pandemic. The goal of our study was to analyze, in a retrospective cohort study, the results of gynecological cancer surgery and evaluate postoperative complications in a single center in one of the most affected areas in Europe. METHODS: We retrospectively analyzed the records of patients who were referred between March 2020 and May 2020 for primary surgical treatment of breast, endometrial, ovarian, cervical, or vulvar cancer. RESULTS: The study included a total of 126 patients. Median age was 60 years (range 29-89). Patients were referred with breast (76/126, 60.3%), endometrial (29/126, 23%), ovarian (14/126, 11.1%), cervical (5/126, 4%), or vulvar cancer (2/126, 1.6%). Polymerase chain reaction (PCR) test for detection of severe acute respiratory syndrome coronavirus 2 (SARS-COV-2) was only conducted in 50% of cases due to the low availability of tests during the first phase of our study, and was indicated only in suspected cases according to the healthcare authorities' protocol. Median hospital stay was 1 day (range 0-18). Excluding breast surgery, laparoscopy was the most used procedure (43/126, 34.1%). 15 patients had a postoperative complication (15/126, 11.9%); only in 2 patients (2/15 13.3%) were there reports of Clavien-Dindo grade 3 or 4 complications. 6 patients tested positive for COVID-19 following a PCR diagnostic test, and these surgeries were cancelled. CONCLUSIONS: Adequate protective measures in the setting of COVID-19 free institutions enabled the continuity of cancer surgery without significant compromise of the safety of patients or healthcare workers.

De Simone, B., et al. (2021). "The management of surgical patients in the emergency setting during COVID-19 pandemic: the WSES position paper." World J Emerg Surg **16**(1): 14.

BACKGROUND: Since the COVID-19 pandemic has occurred, nations showed their unpreparedness to deal with a mass casualty incident of this proportion and severity, which resulted in a tremendous number of deaths even among healthcare workers. The World Society of Emergency Surgery conceived this position paper with the purpose of providing evidence-based recommendations for the management of emergency surgical patients under COVID-19 pandemic for the safety of the patient and healthcare workers. METHOD: A systematic review was conducted in accordance with the Preferred Reporting Items for Systematic Review and Meta-analysis Protocols (PRISMA-P) through the MEDLINE (PubMed), Embase and SCOPUS databases. Synthesis of evidence, statements and recommendations were developed in accordance with the GRADE methodology. RESULTS: Given the limitation of the evidence, the current document represents an effort to join selected high-quality articles and experts' opinion. CONCLUSIONS: The aim of this position paper is to provide an exhaustive guidelines to perform emergency surgery in a safe and protected environment for surgical patients and for healthcare workers under COVID-19 and to offer the best management of COVID-19 patients needing for an emergency surgical treatment. We recommend screening for COVID-19 infection at the emergency department all acute surgical patients who are waiting for hospital admission and urgent surgery. The screening work-up provides a RT-PCR nasopharyngeal swab test and a baseline (non-contrast) chest CT or a chest X-ray or a lungs US, depending on skills and availability. If the COVID-19 screening is not completed we recommend keeping the patient in isolation until RT-PCR swab test result is not available, and to manage him/she such as an overt COVID patient. The management of COVID-19 surgical patients is multidisciplinary. If an immediate surgical procedure is mandatory, whether laparoscopic or via open approach, we recommend doing every effort to protect the operating room staff for the safety of the patient.

Debes, J. D., et al. (2021). "Risk of Healthcare Worker Burnout in Africa during the COVID-19 Pandemic." Ann Glob Health **87**(1): 5.

COVID-19 is now impacting every country in Africa and healthcare workers (HCWs) across the continent remain susceptible to professional burnout. We designed a 43-question survey addressing multiple aspects of the COVID-19 pandemic. The survey was anonymous, distributed via email and phone messaging to 13 countries in Africa. We obtained 489 analyzable responses. 49% off HCWs reported a decrease in income, with the majority experiencing between 1-25% salary reduction. Sixty-six percent reported some access to personal protective equipment (PPE), 20% had no access to PPE and only 14% reported proper access. Strikingly, the percentage reporting never feeling depressed changed from 61% before the pandemic to 31% during the pandemic, with an increase in daily depression from 2% to 20%. We found no association between depression and change in income, household size, availability of PPE or lockdown. Safety concerns related to stigma from being HCWs affected 56% of respondents.

Dedeilia, A., et al. (2020). "Pediatric surgery during the COVID-19 pandemic." World J Clin Pediatr **9**(2): 7-16.

The coronavirus disease 2019 (COVID-19) pandemic has had a major impact on pediatric surgery. The infection is often asymptomatic and atypical in children, while overlapping presentations with other infectious diseases generate additional diagnostic challenges. The high probability of missed pediatric cases and the invasive nature of surgery generate great concern for widespread transmission in this setting. Current guidelines suggest that triage of cases should be made on a case-by-case basis by a multidisciplinary team of experts. Decision-making can be assisted by classifying cases as elective, urgent, or an emergency according to the risks of delaying their surgical management. A workflow diagram should ideally guide the management of all cases from admission to discharge. When surgery is necessary, all staff should use appropriate personal protective equipment, and high-risk practices, such as aerosol-generating tools or procedures, should be avoided if possible. Furthermore, carefully designed organizational protocols should be established to minimize transmission while ensuring the uninterrupted operation of pediatric surgery units. For example, surgical teams can be divided into small weekly rotating groups, and healthcare workers should be continuously monitored for COVID-19 symptoms. Additionally, team protocols in the operating room can optimize communication and improve adherence to personal protective equipment use. Isolated operating rooms, pediatric intensive care units, and surgical wards should be specifically designed for suspected or confirmed COVID-19 cases. Finally, transportation of patients should be minimal and follow designated short routes. All these measures can help mitigate the effects of the COVID-19 pandemic on pediatric surgery units.

Dehghani, F., et al. (2020). "The hierarchy of preventive measures to protect workers against the COVID-19 pandemic: A review." Work **67**(4): 771-777.

BACKGROUND: The high spread rate of coronaviruses, specifically severe acute respiratory syndrome coronavirus 2 (SARS-CoV-2), has raised concerns about the spread of the disease in crowded occupational environments. The risk of occupational exposure to coronavirus depends on the type of industry and the nature of work. Currently, most countries are working for reactivating their economies and need to improve working conditions for a safe situation during the COVID-19 pandemic. OBJECTIVE: The present work aimed to investigate the current engineering and administrative control measures, which are necessary to protect workers against COVID-19 in workplaces. METHODS: The current strategies, including engineering control, administrative control, personal protective equipment, and their efficiencies, were reviewed and discussed. RESULTS: Reviewing the literature indicated that a collection of control approaches should be implemented for an effective control of the virus. Control measures could be selected based on the risk of exposure to COVID-19. The results also revealed that relying solely on a specific control measure could not effectively control the outbreak. CONCLUSION: In conclusion, employers and health professionals must continually monitor international and local guidelines to identify changes in recommendations to make their workplaces safer. Establishment of an expert team in any workplace for the implementation of more effective control measures is warranted, as well.

Dehghani, P., et al. (2020). "North American COVID-19 ST-Segment-Elevation Myocardial Infarction (NACMI) registry: Rationale, design, and implications." Am Heart J **227**: 11-18.

The novel coronavirus, severe acute respiratory syndrome coronavirus 2 (SARS-CoV-2), that causes coronavirus disease 2019 (COVID-19), has resulted in a global pandemic. Patients with cardiovascular risk factors or established cardiovascular disease are more likely to experience severe or critical COVID-19 illness and myocardial injury is a key extra-pulmonary manifestation. These patients frequently present with ST-elevation on an electrocardiogram (ECG) due to multiple etiologies including obstructive, non-obstructive, and/or angiographically normal coronary arteries. The incidence of ST-elevation myocardial infarction (STEMI) mimics in COVID-19-positive hospitalized patients, and the association with morbidity and mortality is unknown. Understanding the natural history and appropriate management of COVID-19 patients presenting with ST elevation is essential to inform patient management decisions and protect healthcare workers. METHODS: The Society for Cardiovascular Angiography and Interventions (SCAI) and The Canadian Association of Interventional Cardiology (CAIC) in conjunction with the American College of Cardiology Interventional Council have collaborated to create a multi-center observational registry, NACMI. This registry will enroll confirmed COVID-19 patients and persons under investigation (PUI) with new ST-segment elevation or new onset left bundle branch block (LBBB) on the ECG with clinical suspicion of myocardial ischemia. We will compare demographics, clinical findings, outcomes and management of these patients with a historical control group of over 15,000 consecutive STEMI activation patients from the Midwest STEMI Consortium using propensity matching. The primary clinical outcome will be in- hospital major adverse cardiovascular events (MACE) defined as composite of all-cause mortality, stroke, recurrent MI, and repeat unplanned revascularization in COVID-19 confirmed or PUI. Secondary outcomes will include the following: reporting of etiologies of ST Elevation; cardiovascular mortality due to myocardial infarction, cardiac arrest and /or shock; individual components of the primary outcome; composite primary outcome at 1 year; as well as ECG and angiographic characteristics. CONCLUSION: The multicenter NACMI registry will collect data regarding ST elevation on ECG in COVID-19 patients to determine the etiology and associated clinical outcomes. The collaboration and speed with which this registry has been created, refined, and promoted serves as a template for future research endeavors.

Delaloye, J. R., et al. (2021). "Distribution of low quality filtering facepiece respirators during the COVID-19 pandemic: an independent analysis of the situation in Switzerland." Swiss Med Wkly **151**: w20459.

BACKGROUND: SARS-CoV-2 is a respiratory virus. Transmission occurs by droplets, contact and aerosols. In medical settings, filtering facepiece (FFP) respirators are recommended for use by personnel exposed to aerosol-generating procedures. During the COVID-19 pandemic, the demand for FFP respirators exceeded their supply worldwide and low-quality products appeared on the market, potentially putting healthcare workers at risk. AIMS: To raise awareness about variations in quality of imported FFP respirators in Switzerland during the COVID-19 pandemic, to draw attention to the current directives regulating the market launch of FFP respirators in Switzerland, to provide practical support in identifying suspicious products or documents and, finally, to offer strategies aimed at reducing the distribution of low-quality FFP respirators in the future. METHODS: Three Swiss laboratories, Spiez Laboratory and Unisant&eacute; in partnership with TOXpro SA individually set up testing procedures to evaluate aerosol penetration and fit testing of FFP respirators imported into Switzerland during COVID-19 pandemic. Additionally, Spiez Laboratory visually inspected the products, examined the certification documents and crosschecked the product information with international databases. RESULTS: Between 31 March and 15 June 2020, 151 FFP respirators were analysed. The initial assessment performed before testing allowed a reduction of up to 35% in the number of FFP respirators sent to Spiez Laboratory for evaluation, for which product information found to be faulty. After filtration efficiency evaluation and fit testing, 52% and 60% of all products tested by Spiez Laboratory and Unisant&eacute;-TOXpro SA, respectively, did not meet the minimum performance requirements established independently by the three Swiss laboratories. CONCLUSION: The demand for FFP respirators exceeded the supply capacity from established suppliers of the Swiss market. New production and import channels emerged, as did the number of poor-quality FFP respirators. FFP respirators remaining in stocks should be checked for conformity before being used, or eliminated and replaced if quality does not meet standards.

Delgado, D., et al. (2020). "Personal Safety during the COVID-19 Pandemic: Realities and Perspectives of Healthcare Workers in Latin America." Int J Environ Res Public Health **17**(8).

Healthcare workers exposed to coronavirus (COVID-19) may not have adequate access to personal protective equipment (PPE), safety procedures, and diagnostic protocols. Our objective was to evaluate the reality and perceptions about personal safety among healthcare workers in Latin America. This is a cross-sectional, online survey-based study administered to 936 healthcare professionals in Latin America from 31 March 2020 to 4 April 2020. A 12-item structured questionnaire was developed. A total of 936 healthcare workers completed the online survey. Of them, 899 (95.1%) were physicians, 28 (2.9%) were nurses, and 18 (1.9%) were allied health professionals. Access to protective equipment was as follows: gel hand sanitizer (n = 889; 95%), disposable gloves (n = 853; 91.1%), disposable gowns (n = 630; 67.3%), disposable surgical masks (785; 83.9%), N95 masks (n = 516; 56.1%), and facial protective shields (n = 305; 32.6%). The vast majority (n = 707; 75.5%) had access to personal safety policies and procedures, and 699 (74.7%) participants had access to diagnostic algorithms. On a 1-to-10 Likert scale, the participants expressed limited human resources support (4.92 ± 0.2; mean ± SD), physical integrity protection in the workplace (5.5 ± 0.1; mean ± SD), and support from public health authorities (5.01 ± 0.12; mean ± SD). Healthcare workers in Latin America had limited access to essential PPE and support from healthcare authorities during the COVID-19 pandemic.

Dellweg, D., et al. (2021). "Bench testing of noninvasive ventilation masks with viral filters for the protection from inhalation of infectious respirable particles." J Occup Environ Hyg **18**(3): 118-127.

During the beginning of the SARS-CoV-2 pandemic, there was a shortage of masks and respirators for the protection of health care professionals. Masks for noninvasive ventilation (NIV) in combination with viral-proof filters, worn by healthcare workers, could serve as an alternative protection measure. We determined the simulated protection factor (SPF) of such devices in comparison to conventional surgical masks, N95, and FFP3 respirators. Masks and respirators were mounted on a ventilated mannequin head in a test-chamber. Isotonic saline containing 150 MBq (99m)TC-DTPA (99mTc-diethylenetriamine pentaacetate (DTPA) was nebulized inside the box. The aerosol had a mass median aerodynamic diameter of 0.6 ± 0.4 µm. SPFs were measured using radioactive DTPA particles in the mannequin test system by calculating the ratio of unfiltered particles (P(u)) and filtered particles (P(f)) for each tested device (SPF = P(u)/P(f)). Simulated protection factors were 15.6 ± 3.6 for a ResMed AcuCare mask plus filter, 3.5 ± 0.2 for a ResMed Mirage Quattro FX mask plus filter, 9.5 ± 0.8 for a Loewenstein JOYCEclinc FF mask plus filter, 1.9 ± 0.2 for a surgical mask with a rubber band, 2.7 ± 0.7 for a surgical mask with ribbons, 2.3 ± 0.3 for an FFP3 respirator, and 3.6 ± 1.3 for an N95 respirator. The ResMed AcuCare and the Loewenstein JOYCEclinic FF mask were more effective than any other of the tested devices (p < 0.001). In conclusion, masks normally used for NIV with viral-proof filters can effectively filter respirable particles.

Demmer, R. T., et al. (2020). "SARS-CoV-2 Infection Among Symptom-Free Healthcare Workers." medRxiv.

IMPORTANCE: Current evidence suggests that transmission of severe acute respiratory syndrome coronavirus 2 (SARS-CoV-2) is possible among symptom-free individuals but limited data are available on this topic in healthcare workers (HCW). The quality and acceptability of self-collected nasopharyngeal swabs (NPS) is unknown. OBJECTIVE: To estimate the prevalence of SARS-CoV-2 infection and to assess the acceptability of self-collected NPS among HCW. DESIGN: Cross-sectional convenience sample enrolled between April 20th and June 24th, 2020. We had >95% power to detect at least one positive test if the true underlying prevalence of SARS-CoV2 was > 1%. SETTING: The metropolitan area surrounding Minneapolis and St. Paul, Minnesota. PARTICIPANTS: HCW free of self-reported upper respiratory symptoms were recruited. Exposures: Participants completed questionnaires regarding demographics, household characteristics, personal protective equipment (PPE) utilization and comorbidities. OUTCOMES: A participant self-collected nasopharyngeal swab (NPS) was obtained. SARS-CoV-2 infection was assessed via polymerase chain reaction. NPS discomfort was assessed on a scale of 1 (no discomfort) - 10 (extreme discomfort). NPS duration and depth into the nasopharynx, and willingness to perform future self-collections were assessed. RESULTS: Among n=489 participants 80% were female and mean age+/-SD was 41+/-11. Participants reported being physicians (14%), nurse practitioners (8%), physicians assistants (4%), nurses (51%), medics (3%), or other which predominantly included laboratory technicians and administrative roles (22%). Exposure to a known/suspected COVID-19 case in the 14 days prior to enrollment was reported in 40% of participants. SARS-CoV-2 was not detected in any participant. The mean+/-SD discomfort level of the NPS was 4.5+/-2.0. 95% of participants reported that their self-swab was longer than or equal to the duration of patient swabs they had previously performed, and 89% reported the depth to be deeper than or equal to the depth of previous patient swabs. Over 95% of participants reported a willingness to repeat a self-collected NP swab in the future. CONCLUSIONS AND RELEVANCE: The point prevalence of SARS-CoV-2 infection was likely very low in symptom-free Minnesota healthcare workers from April 20th and June 24th, 2020. Self-collected NP swabs are well-tolerated and a viable alternative to provider-collected swabs to preserve PPE.

Deng, M. (2020). "The prevention and management of the coronavirus disease 2019 (COVID-19) outbreak in radiology departments in epidemic areas." Jpn J Radiol **38**(6): 483-488.

This review based on the actual results of epidemic prevention management in radiology departments in epidemic areas, and using designated hospital management methods, to summarize the radiology protection system. With the cooperation of the whole hospital and radiology department, a number of effective evaluation methods have been carried out to ensure the optimization of clinical protection. These practical methods provide a basis for the further promotion of management strategies and reduction of nosocomial infection. To our knowledge, the establishment of standardized protection and clear process has opened up a new idea of epidemic prevention management, which can effectively provide timely, efficient, and accurate support for clinical research, and promote the transformation from clinical research type to scientific research management type.

Deng, Z. F., et al. (2020). "[Application of tracheotomy in the treatment of severe cases of COVID-19]." Zhonghua Er Bi Yan Hou Tou Jing Wai Ke Za Zhi **55**(6): 576-579.

Objective: To discuss the the effects, indications and protective measures of tracheotomy for severe cases of coronavirus disease 2019 (COVID-19) patients. Methods: A retrospectively analysis was conducted to explore the clinical data of COVID-19 patients who received tracheotomy in February to March 2020, and descriptive statistics were used to analyze the indication of tracheotomy, particularity of intraoperative treatment and protective measures. Results: A total of 4 cases were included in this article. All patients were successfully operated. One case had postoperative incision continuous bleeding, there were not other complications and nosocomial infection among the medical staff. The patient's condition was relieved in different degrees after the operation, who remained hospitalized. Conclusion: Tracheotomy for severe cases of COVID-19 can achieve certain curative effect, but the occurrence of tracheotomy related complications and nosocomial infection should be effectively controlled, and the risk benefit ratio of tracheotomy should be carefully weighed before surgery.

Deng, Z. F., et al. (2020). "[Application of tracheotomy in the treatment of severe cases of COVID-19]." Zhonghua Er Bi Yan Hou Tou Jing Wai Ke Za Zhi **55**(0): E009.

Objective: Todiscuss the the effects, indications and protective measures of tracheotomy for severe cases of 2019 novel corona virus disease(COVID-19)patients. Methods: A retrospectively analyze was conducted to explore the clinical data of ofCOVID-19 patients who received tracheotomy in February to March 2020,descriptive statistics were used to analyze the indication of tracheotomy, particularity of intraoperative treatment and protective measures. Results: A total of 4 cases were included in this article, 3 cases were successfully operated, 1 case of postoperative incision continuous bleeding, there were not other complications and nosocomial infection among the medical staff.the patient's condition was relieved in different degrees after the operation, who remain hospitalized. Conclusion: Tracheotomy for severe cases of COVID-19 can achieve certain curative effect, but the occurrence of tracheotomy related complicationsand nosocomial infection should be effectively controlled, and the risk benefit ratio of tracheotomy should be carefully weighed before surgery.

Dennerlein, J. T., et al. (2020). "An Integrative Total Worker Health Framework for Keeping Workers Safe and Healthy During the COVID-19 Pandemic." Hum Factors **62**(5): 689-696.

OBJECTIVE: The aim was to recommend an integrated Total Worker Health (TWH) approach which embraces core human factors and ergonomic principles, supporting worker safety, health, and well-being during the COVID-19 pandemic. BACKGROUND: COVID-19 has resulted in unprecedented challenges to workplace safety and health for workers and managers in essential businesses, including healthcare workers, grocery stores, delivery services, warehouses, and distribution centers. Essential workers need protection, accurate information, and a supportive work environment with an unwavering focus on effective infection control. METHOD: The investigators reviewed emerging workplace recommendations for reducing workers' exposures to the novel coronavirus and the challenges to workers in protecting their health. Using a theoretical framework and guidelines for integrating safety and health management systems into an organization for TWH, the investigators adapted the framework's key characteristics to meet the specific worker safety and health issues for effective infection control, providing supports for increasing psychological demands while ensuring a safe work environment. RESULTS: The recommended approach includes six key characteristics: focusing on working conditions for infection control and supportive environments for increased psychological demands; utilizing participatory approaches involving workers in identifying daily challenges and unique solutions; employing comprehensive and collaborative efforts to increase system efficiencies; committing as leaders to supporting workers through action and communications; adhering to ethical and legal standards; and using data to guide actions and evaluate progress. CONCLUSION: Applying an integrative TWH approach for worker safety, health, and well-being provides a framework to help managers systematically organize and protect themselves, essential workers, and the public during the COVID-19 pandemic. APPLICATION: By using the systems approach provided by the six implementation characteristics, employers of essential workers can organize their own efforts to improve system performance and worker well-being during these unprecedented times.

Desborough, J., et al. (2020). "The importance of consistent advice during a pandemic: An analysis of Australian advice regarding personal protective equipment in healthcare settings during COVID-19." Aust J Gen Pract **49**(6): 369-372.

BACKGROUND: Lessons from previous pandemics emphasise the importance of consistent information and advice for healthcare workers (HCWs), particularly in relation to their use of personal protective equipment (PPE). OBJECTIVE: The aim of this article is to review the consistency of advice regarding HCW use of PPE among Australian states and territories and 10 key Australian professional and advocacy organisations, against the Australian Government's national guidelines. DISCUSSION: While some recommendations expand on the national guidelines, the Australian Government's efforts to work in concert with states, territories and peak professional and advocacy organisations are evident in the broadly consistent recommendations for the use of PPE by HCWs and other people working in healthcare settings across the country. The overall consistency of recommendations for the use of PPE shows a nationwide attempt to support and protect the healthcare workforce and the Australian community during the COVID-19 pandemic.

d'Ettorre, G., et al. (2021). "Post-Traumatic Stress Symptoms in Healthcare Workers Dealing with the COVID-19 Pandemic: A Systematic Review." Int J Environ Res Public Health **18**(2).

Prevention of post-traumatic stress symptoms (PTSS) in healthcare workers (HCWs) facing the current COVID-19 pandemic is a challenge worldwide as HCWs are likely to experience acute and chronic, often unpredictable, occupational stressors leading to PTSS. This review aims to analyze the literature to discover which topics have been focused on and what the latest developments are in managing the occupational risk of PTSS in HCWs during the current pandemic. For the purpose of this review, we searched for publications in MEDLINE/Pubmed using selected keywords. The articles were reviewed and categorized into one or more of the following categories based on their subject matter: risk assessment, risk management, occurrence rates. A total of 16 publications matched our inclusion criteria. The topics discussed were: "Risk Assessment", "Occurrence Rates", and "Risk Management". Young age, low work experience, female gender, heavy workload, working in unsafe settings, and lack of training and social support were found to be predictors of PTSS. This review's findings showed the need for urgent interventions aimed at protecting HCWs from the psychological impact of traumatic events related to the pandemic and leading to PTSS; healthcare policies need to consider preventive and management strategies toward PTSS, and the related psychic sequelae, in HCWs.

Dev, N., et al. (2021). "Risk factors and frequency of COVID-19 among healthcare workers at a tertiary care centre in India: a case-control study." Trans R Soc Trop Med Hyg.

BACKGROUND: There is a paucity of data on risk factors for infection among healthcare workers (HCWs) from India. Our objective was to evaluate the risk factors and frequency of severe acute respiratory syndrome coronavirus 2 (SARS-CoV-2) infection among HCWs. METHODS: We conducted this retrospective case-control study of 3100 HCWs between May and July 2020. HCWs positive for SARS-CoV-2 infection were the cases (n=506) and those negative for SARS-CoV-2 were the controls (n=253). Univariate analysis was followed by multivariate analysis of key demographic, clinical and infection control variables. RESULTS: SARS-CoV-2 infection was found in 16.32% of HCWs. Nearly 45% of infected HCWs were asymptomatic. The proportions of sanitation workers (24% vs 8%; p<0.0001) and technicians (10% vs 4%; p=0.0002) were higher and that of doctors was lower among cases as compared with controls (23% vs 43%; p<0.0001). On univariate analysis, the type of HCW, smoking, lack of training, inadequate personal protective equipment (PPE) use and taking no or fewer doses of hydroxychloroquine (HCQ) were found to be significant. On multivariate analysis, the type of HCW (risk ratio [RR] 1.67 [95% confidence interval {CI} 1.34 to 2.08], p<0.0001), inappropriate PPE use (RR 0.63 [95% CI 0.44 to 0.89], p=0.01) and taking fewer doses of HCQ (RR 0.92 [95% CI 0.86 to 0.99], p=0.03) were significant. CONCLUSIONS: The frequency of SARS-CoV-2 infection was 16% among HCWs. Being a sanitation worker, inappropriate PPE use and lack of HCQ prophylaxis predisposed HCWs to SARS-CoV-2 infection.

Devrim, İ. and N. Bayram (2020). "Infection control practices in children during COVID-19 pandemic: Differences from adults." Am J Infect Control **48**(8): 933-939.

BACKGROUND: Limited studies have been published on practices and management of Coronavirus Disease 2019 (COVID-19) in children. Despite the fact that COVID-19 rarely caused any severe disease in children, the asymptomatic children might be playing an important role for spreading COVID-19 in healthcare facilities. This review aimed at sharing our experience of how to handle patients with COVID-19 in a pediatric referral and tertiary care hospital to prevent the possible transmissions to the healthcare workers (HCWs). METHODS: This review sought to identify infection control practices measures during COVID-19 pandemic comes from our daily practice combined with the most recent guidelines with the new experience and information. RESULTS: Prevention the transmission of COVID-19 to the HCWs, 4 primary themes should be taken into consideration; (1) ongoing education and importance of the organization of the healthcare facility, (2) proper clinical triage and isolation of the suspected or confirmed COVID-19 patients in the outpatient clinics and in the emergency departments, (3) necessity of the organization of the COVID-19 wards, and (4) utilization of personal protective equipment. CONCLUSIONS: Infection control precautions to prevent the possible transmissions to HCWs as well as the other patients and their caregivers from children with COVID-19 are very critical. If sufficient precautions are not taken, healthcare settings may serve as additional source of transmission and spread of COVID-19 in the society.

Dhahri, A. A., et al. (2020). "A cross-sectional survey on availability of facilities to healthcare workers in Pakistan during the COVID-19 pandemic." Ann Med Surg (Lond) **59**: 127-130.

INTRODUCTION: COVID-19 pandemic has caused a healthcare crisis across the world. Low-economic countries like Pakistan lag behind in an adequate response including supply of Personal Protective Equipment (PPE), leading to panic among healthcare workers. We aim to evaluate hospital settings and state in Pakistan regarding availability of resources and views of healthcare workers on COVID-19. METHOD: A questionnaire survey was carried out among healthcare workers in public and private sector hospitals across Pakistan for a period of one month. The primary measured outcomes were presence of local Standard Operating Procedures (SOPs), availability and training of PPE, specific isolation wards and staff wellbeing support by the hospital management. RESULTS: There were 337 participants, 307 (91.1%) doctors and 11 nurses (3.3%). About two-third of the participants (n = 199, 59%) reported non-availability of PPE and 40% (n = 136) denied availability of local Standard Operating Procedures. About a quarter of the participants (n = 94, 27.8%) had training in Donning and Doffing. Most of the participants (n = 277, 82.1%) felt that it was necessary to have testing available for frontline workers. CONCLUSION: There is lack of PPE and adequate facilities in hospitals as COVID-19 continue to spread in Pakistan. Local medical governing bodies and societies should come forward with guidelines to ascertain wellbeing of the healthcare workers.

Dhillon, R. S., et al. (2021). "Aerosolisation during tracheal intubation and extubation in an operating theatre setting." Anaesthesia **76**(2): 182-188.

Aerosol-generating procedures such as tracheal intubation and extubation pose a potential risk to healthcare workers because of the possibility of airborne transmission of infection. Detailed characterisation of aerosol quantities, particle size and generating activities has been undertaken in a number of simulations but not in actual clinical practice. The aim of this study was to determine whether the processes of facemask ventilation, tracheal intubation and extubation generate aerosols in clinical practice, and to characterise any aerosols produced. In this observational study, patients scheduled to undergo elective endonasal pituitary surgery without symptoms of COVID-19 were recruited. Airway management including tracheal intubation and extubation was performed in a standard positive pressure operating room with aerosols detected using laser-based particle image velocimetry to detect larger particles, and spectrometry with continuous air sampling to detect smaller particles. A total of 482,960 data points were assessed for complete procedures in three patients. Facemask ventilation, tracheal tube insertion and cuff inflation generated small particles 30-300 times above background noise that remained suspended in airflows and spread from the patient's facial region throughout the confines of the operating theatre. Safe clinical practice of these procedures should reflect these particle profiles. This adds to data that inform decisions regarding the appropriate precautions to take in a real-world setting.

Di Lella, F., et al. (2021). "Bedside Surgical Tracheostomy in the Intensive Care Unit during Covid-19 Pandemic." Ann Otol Rhinol Laryngol **130**(3): 304-306.

OBJECTIVES: To describe Otolaryngologists' perspective in managing COVID-19 patients with acute respiratory distress syndrome (ARDS) requiring tracheostomy in the ICUs during the pandemic peak in a dramatic scenario with limited resources. SETTING: Tertiary referral university hospital, regional hub in northern Italy during SARS CoV 2 pandemic peak (March 9th to April 10th, 2020). METHODS: Technical description of open bedside tracheostomies performed in ICUs on COVID-19 patients during pandemic peak with particular focus on resource allocation and healthcare professionals coordination. A dedicated "airway team" was created in order to avoid transportation of critically ill patients and reduce facility contamination. RESULTS: During the COVID-19 pandemic, bedside minimally invasive tracheostomy in the ICU was selected by the Authors over conventional surgical technique or percutaneous procedures for both technical and operational reasons. Otolaryngologists' experience derived from direct involvement in 24 tracheostomies is reported. CONCLUSIONS: Tracheostomies on COVID-19 patients should be performed in a safe and standardized setting. The limited resources available in the pandemic peak required meticulous organization and optimal allocation of the resources to grant safety of both patients and healthcare workers.

Di Maio, P., et al. (2020). "Performing the nasopharyngeal and oropharyngeal swab for 2019-novel coronavirus (SARS-CoV-2) safely: How to dress, undress, and technical notes." Head Neck **42**(7): 1548-1551.

BACKGROUND: To show how to safely perform nasopharyngeal and/or oropharyngeal swabs for 2019-novel coronavirus. METHODS: The video describes in detail the dressing and undressing procedures of health personnel, with the appropriate personal protective equipment. Technical notes for the execution of the nasopharyngeal and oropharyngeal swab are also provided to avoid sampling errors. RESULTS: The undressing phase is the procedure with the highest risk of self-contamination for the health worker. Following the various steps as shown in the video, there were no cases of contagion among the otolaryngology team appointed to perform the swabs for SARS-CoV-2 testing. CONCLUSIONS: This study demonstrates the technical feasibility of safely performing nasopharyngeal and/or oropharyngeal swabs for identification of SARS-CoV-2 viral RNA.

Di Nardo, M., et al. (2020). "A literature review of 2019 novel coronavirus (SARS-CoV2) infection in neonates and children." Pediatr Res.

At the time of writing, there are already millions of documented infections worldwide by the novel coronavirus 2019 (2019-nCoV or severe acute respiratory syndrome coronavirus 2 (SARS-CoV2)), with hundreds of thousands of deaths. The great majority of fatal events have been recorded in adults older than 70 years; of them, a large proportion had comorbidities. Since data regarding the epidemiologic and clinical characteristics in neonates and children developing coronavirus disease 2019 (COVID-19) are scarce and originate mainly from one country (China), we reviewed all the current literature from 1 December 2019 to 7 May 2020 to provide useful information about SARS-CoV2 viral biology, epidemiology, diagnosis, clinical features, treatment, prevention, and hospital organization for clinicians dealing with this selected population. IMPACT: Children usually develop a mild form of COVID-19, rarely requiring high-intensity medical treatment in pediatric intensive care unit.Vertical transmission is unlikely, but not completely excluded.Children with confirmed or suspected COVID-19 must be isolated and healthcare workers should wear appropriate protective equipment.Some clinical features (higher incidence of fever, vomiting and diarrhea, and a longer incubation period) are more common in children than in adults, as well as some radiologic aspects (more patchy shadow opacities on CT scan images than ground-glass opacities).Supportive and symptomatic treatments (oxygen therapy and antibiotics for preventing/treating bacterial coinfections) are recommended in these patients.

Díaz-Guio, D. A., et al. (2020). "COVID-19: Biosafety in the Intensive Care Unit." Curr Trop Med Rep: 1-8.

PURPOSE OF REVIEW: COVID-19 is a new, highly transmissible disease to which healthcare workers (HCWs) are exposed, especially in the intensive care unit (ICU). Information related to protection mechanisms is heterogeneous, and the infected HCWs' number is increasing. This review intends to summarize the current knowledge and practices to protect ICU personnel during the patient management process in the context of the current pandemic. RECENT FINDINGS: The transmission mechanisms of SARS-CoV-2 are mainly respiratory droplets, aerosols, and contact. The virus can last for a few hours suspended in the air and be viable on surfaces for several days. Some procedures carried out in the ICU can generate aerosols. The shortage of respirators, such as the N95, has generated an increase in the demand for other protective equipment in critical care settings. SUMMARY: The probability of transmission depends on the characteristics of the pathogen, the availability of quality personal protective equipment, and the human factors associated with the performance of health workers. It is necessary to have knowledge of the virus and availability of the best possible personal protection equipment, develop skills for handling equipment, and develop non-technical skills during all intensive care process; this can be achieved through structured training.

Dimeglio, C., et al. (2021). "Protection of healthcare workers against SARS-CoV-2 reinfection." Clin Infect Dis.

Ding, B. T. K., et al. (2020). "Operating in a Pandemic: Lessons and Strategies from an Orthopaedic Unit at the Epicenter of COVID-19 in Singapore." J Bone Joint Surg Am **102**(13): e67.

➤. With severe limitations in manpower, facilities, and equipment, and the concern for nosocomial transmission, operating in a pandemic is fraught with danger from multiple fronts. ➤. Strategies to mitigate nosocomial spread include prioritization of existing patients, triaging and treatment of new patient encounters, infection control protocols, perioperative considerations, manpower management, and novel strategies for interdisciplinary interaction and education. ➤. The decision to proceed with or postpone surgery should be based on the urgency of the surgical procedure and the physiological health of the patient. ➤. When performing an operation on a patient who has suspected or confirmed infection with novel coronavirus disease 2019 (COVID-19), personal protection equipment should include hair covers, face shields or goggles, N95 respirator masks, a blood-borne pathogen-resistant surgical gown, shoe covers, and double-gloving with single-use gloves. ➤. Loose-fitting, powered air-purifying respirators should be considered for prolonged surgeries. ➤. An astutely formulated and comprehensive business continuity plan is an orthopaedic unit’s best strategy for maintaining critical standards, discipline, and morale in severe and prolonged outbreaks.

Do, D., et al. (2020). "Healthcare Worker Attendance During the Early Stages of the COVID-19 Pandemic: A Longitudinal Analysis of Daily Fingerprint-Verified Data from All Public-Sector Secondary and Tertiary Care Facilities in Bangladesh." medRxiv.

BACKGROUND: The COVID-19 pandemic has overwhelmed hospitals in several areas in high-income countries. An effective response to this pandemic requires healthcare workers (HCWs) to be present at work, particularly in low- and middle-income countries (LMICs) where they are already in critically low supply. To inform whether and to what degree policymakers in Bangladesh, and LMICs more broadly, should expect a drop in HCW attendance as COVID-19 continues to spread, this study aims to determine how HCW attendance has changed during the early stages of the COVID-19 pandemic in Bangladesh. METHODS: This study analyzed daily fingerprint-verified attendance data from all 527 public-sector secondary and tertiary care facilities in Bangladesh to describe HCW attendance from January 26, 2019 to March 22, 2020, by cadre, hospital type, and geographic division. We then regressed HCW attendance onto fixed effects for day-of-week, month, and hospital, as well as indicators for each of three pandemic periods: a China-focused period (January 11, 2020 [first confirmed COVID-19 death in China] until January 29, 2020), international-spread period (January 30, 2020 [World Health Organization declared a global emergency] until March 6, 2020), and local-spread period (March 7, 2020 [first confirmed COVID-19 case in Bangladesh] until the end of the study period). FINDINGS: On average between January 26, 2019 and March 22, 2020, 34.1% of doctors, 64.6% of nurses, and 70.6% of other healthcare staff were present for their scheduled shift. Attendance rate increased with time in 2019 among all cadres. Attendance level of nurses dropped by 2.5% points (95% CI; -3.2% to -1.8%) and 3.5% points (95% CI; -4.5% to -2.5%) during the international-spread and the local-spread periods of the COVID-19 pandemic, relative to the China-focused period. Similarly, the attendance level of other healthcare staff declined by 0.3% points (95% CI; -0.8% to 0.2%) and 2.3% points (95% CI; -3.0% to -1.6%) during the international-spread and local-spread periods, respectively. Among doctors, however, the international-spread and local-spread periods were associated with a statistically significant increase in attendance by 3.7% points (95% CI; 2.5% to 4.8%) and 4.9% points (95% CI; 3.5% to 6.4%), respectively. The reduction in attendance levels across all HCWs during the local-spread period was much greater at large hospitals, where the majority of COVID-19 testing and treatment took place, than that at small hospitals. CONCLUSIONS: After a year of significant improvements, attendance levels among nurses and other healthcare staff (who form the majority of the healthcare workforce in Bangladesh) have declined during the early stages of the COVID-19 pandemic. This finding may portend an even greater decrease in attendance if COVID-19 continues to spread in Bangladesh. Policymakers in Bangladesh and similar LMICs should undertake major efforts to achieve high attendance levels among HCWs, particularly nurses, such as by providing sufficient personal protective equipment as well as monetary and non-monetary incentives.

Do, D., et al. (2020). "Healthcare worker attendance during the early stages of the COVID-19 pandemic: A longitudinal analysis of fingerprint-verified data from all public-sector secondary and tertiary care facilities in Bangladesh." J Glob Health **10**(2): 020509.

BACKGROUND: The COVID-19 pandemic has overwhelmed hospitals in several areas in high-income countries. An effective response to this pandemic requires health care workers (HCWs) to be present at work, particularly in low- and middle-income countries (LMICs) where they are already in critically low supply. To inform whether and to what degree policymakers in Bangladesh, and LMICs more broadly, should expect a drop in HCW attendance as COVID-19 continues to spread, this study aims to determine how HCW attendance has changed during the early stages of the COVID-19 pandemic in Bangladesh. METHODS: This study analyzed daily fingerprint-verified attendance data from all 527 public-sector secondary and tertiary care facilities in Bangladesh to describe HCW attendance from January 26, 2019 to March 22, 2020, by cadre, hospital type, and geographic division. We then regressed HCW attendance onto fixed effects for day-of-week, month, and hospital, as well as indicators for each of three pandemic periods: a China-focused period (January 11, 2020 (first confirmed COVID-19 death in China) until January 29, 2020), international-spread period (January 30, 2020 (World Health Organization's declaration of a global emergency) until March 6, 2020), and local-spread period (March 7, 2020 (first confirmed COVID-19 case in Bangladesh) until the end of the study period). FINDINGS: On average between January 26, 2019 and March 22, 2020, 34.1% of doctors, 64.6% of nurses, and 70.6% of other health care staff were present for their scheduled shift. HCWs' attendance rate increased with time in 2019 among all cadres. Nurses' attendance level dropped by 2.5% points (95% confidence interval (CI) = -3.2% to -1.8%) and 3.5% points (95% CI = -4.5% to -2.5%) during the international-spread and the local-spread periods of the COVID-19 pandemic, relative to the China-focused period. Similarly, the attendance level of other health care staff declined by 0.3% points (95% CI = -0.8% to 0.2%) and 2.3% points (95% CI = -3.0% to -1.6%) during the international-spread and local-spread periods, respectively. Among doctors, however, the international-spread and local-spread periods were associated with a statistically significant increase in attendance by 3.7% points (95% CI = 2.5% to 4.8%) and 4.9% points (95% CI = 3.5% to 6.4%), respectively. The reduction in attendance levels across all HCWs during the local-spread period was much greater at large hospitals, where the majority of COVID-19 testing and treatment took place, than that at small hospitals. CONCLUSIONS: After a year of significant improvements, HCWs' attendance levels among nurses and other health care staff (who form the majority of Bangladesh's health care workforce) have declined during the early stages of the COVID-19 pandemic. This finding may portend an even greater decrease in attendance if COVID-19 continues to spread in Bangladesh. Policymakers in Bangladesh and similar LMICs should undertake major efforts to achieve high attendance levels among HCWs, particularly nurses, such as by providing sufficient personal protective equipment as well as monetary and non-monetary incentives.

Dobrocky, T., et al. (2021). "Maintaining high thrombectomy rates during pandemics." Curr Opin Neurol **34**(1): 18-21.

PURPOSE OF REVIEW: The aim of this article is to review the current literature on endovascular treatment of acute ischemic stroke in the aftermath of the coronavirus disease 2019 (COVID-19) lockdown. RECENT FINDINGS: The outbreak of the COVID-19 has had effect of unprecedented magnitude on the social, economic and personal aspects around the globe. Healthcare providers were forced to expand capacity to provide care to the surging number of symptomatic COVID-19 patients, while maintaining a fully operating service for all non-COVID patients. The recent literature suggesting an overall decrease in acute ischemic stroke admissions as well as total number of endovascular treatments will be reviewed. Although the underlying reasons therefore remain the matter of debate, it seems that the imposed restrictions, requiring social distancing, and stopping all nonessential services, have led to a higher threshold for patients to seek medical attention, in particular in those with less severe symptoms. Thus, raising public awareness on the importance of strokes and transient ischemic attacks is even more important in the light of the current situation to avoid serious healthcare, economic consequences, and limit long term morbidity. SUMMARY: The priority remains maintaining a fast and efficient pre and in-hospital work-flow while mitigating nosocomial transmission and protecting the patient and the healthcare workers with appropriate personal protective equipment.

Domínguez-Salas, S., et al. (2021). "Healthcare workers' protection and psychological safety during the COVID-19 pandemic in Spain." J Nurs Manag.

AIMS: to analyse the relationship between work engagement, sense of coherence, and psychological distress levels in Spanish health professionals who were active during the COVID-19 pandemic lockdown. BACKGROUND: Work Engagement and Sense of Coherence can help professionals to cope with work-related psychological distress due to the harsh conditions of the COVID-19 working environment. METHODS: cross-sectional observational study of 1459 healthcare professionals. The Utrecht Work Engagement Scale, the Sense of Coherence Scale and Goldberg's General Health Questionnaire were distributed and analyzed with descriptive and multiple linear regression methods. RESULTS: High levels of work engagement, especially in the dedication dimension, of sense of coherence, in particular in the meaningfulness dimension, and psychological distress were obtained. Significant correlations (p<0.001) were identified between all the variables. CONCLUSIONS: Work engagement and sense of coherence correlated positively with each other, and both negatively with psychological distress. So, healthcare professionals, despite presenting psychological distress, perceive their work satisfactorily and positively despite the severity of the situation and the harsh conditions. IMPLICATIONS FOR NURSING MANAGEMENT: Sense of coherence and work engagement are protective factors against psychological distress. Preventive measures for professionals should go through the dimensionalization of the problem and the adaptation of practical measures for daily management.

Donders, F., et al. (2020). "ISIDOG Recommendations Concerning COVID-19 and Pregnancy." Diagnostics (Basel) **10**(4).

Providing guidelines to health care workers during a period of rapidly evolving viral pandemic infections is not an easy task, but it is extremely necessary in order to coordinate appropriate action so that all patients will get the best possible care given the circumstances they are in. With these International Society of Infectious Disease in Obstetrics and Gynecology (ISIDOG) guidelines we aim to provide detailed information on how to diagnose and manage pregnant women living in a pandemic of COVID-19. Pregnant women need to be considered as a high-risk population for COVID-19 infection, and if suspected or proven to be infected with the virus, they require special care in order to improve their survival rate and the well-being of their babies. Both protection of healthcare workers in such specific care situations and maximal protection of mother and child are envisioned.

Dong, Z. Q., et al. (2020). "The social psychological impact of the COVID-19 pandemic on medical staff in China: A cross-sectional study." Eur Psychiatry **63**(1): e65.

BACKGROUND: The COVID-19 outbreak required the significantly increased working time and intensity for health professionals in China, which may cause stress signs. METHODS: From March 2-13 of 2020, 4,618 health professionals in China were included in an anonymous, self-rated online survey regarding their concerns on exposure to the COVID-19 outbreak. The questionnaires consisted of five parts: basic demographic information and epidemiological exposure; occupational and psychological impact; concerns during the episode; coping strategies; and the Huaxi Emotional-Distress Index (HEI). RESULTS: About 24.2% of respondents experienced high levels of anxiety or/and depressive symptoms since the COVID-19 outbreak. Respondents who worried about their physical health and those who had COVID-19 infected friends or close relatives were more likely to have high HEI levels, than those without these characteristics. Further, family relationship was found to have an independent protective effect against high HEI levels. Their main concerns were that their families would not be cared for and that they would not be able to work properly. Compared to respondents with clear emotional problems, those with somewhat hidden emotional issues adopted more positive coping measures. CONCLUSIONS: About a quarter of medical staff experienced psychological problems during the pandemic of COVID-19. The psychological impact of stressful events was related to worrying about their physical health, having close COVID-19 infected acquaintances and family relationship issues. Therefore, the psychological supprot for medical staff fighting in the COVID-19 pandemic may be needed.

Dooley, S. W. and T. R. Frieden (2020). "We must rigorously follow basic infection control procedures to protect our healthcare workers from SARS-CoV-2." Infect Control Hosp Epidemiol **41**(12): 1438-1440.

Because severe acute respiratory coronavirus virus 2 (SARS-CoV-2) spreads easily and healthcare workers are at increased risk of both acquiring and transmitting infection, all healthcare facilities must rapidly and rigorously implement the full hierarchy of established infection controls: source control (removal or mitigation of infection sources), engineering and environmental controls, administrative controls, and personal protective equipment.

Dorman, C., et al. (2021). "Factors Associated with Willingness to be Vaccinated Against COVID-19 in a Large Convenience Sample." J Community Health: 1-7.

Willingness and reasons to be vaccinated against COVID-19 were examined among 26,324 respondents who completed a survey on willingness and questions related to Confidence in vaccine safety, Complacency about the disease, Convenience of vaccination, tendency to Calculate risks versus benefits, and Concern for protecting others. Willingness to be vaccinated differed by age (p < 0.001), by race and ethnicity (p < 0.001) and by level of education (p < 0.001). Willingness generally increased with age and education. Asians were most willing to be vaccinated, followed by non-Hispanic Whites, Hispanics, and non-Hispanic Blacks (p < 0.001). Occupational groups differed in willingness (p < 0.001). Retired and students were more willing than all others (p < 0.001) followed by disabled or unemployed, healthcare workers, and educators. First Responders were least willing to be vaccinated (p < 0.001) followed by construction, maintenance and landscaping, homemakers, housekeeping, cleaning and janitorial workers, and retail and food service. The strongest predictor of willingness was confidence with the safety of the vaccine (r = 0.723, p < 0.001), followed by concern with protecting others by being vaccinated (r = 0.574, p < 0.001), and believing COVID-19 was serious enough to merit vaccination (r = 0.478, p < 0.00). Using multiple regression, confidence in safety was the strongest predictor for all groups. Protecting others was strongest for 13 of 15 demographic groups and 8 of 11 occupational groups. College educated, non-Hispanic Whites, first responders, construction, maintenance and landscape workers, housekeeping, cleaning and janitorial workers all gave greater weight to complacency about the disease. These results can help in designing programs to combat vaccine hesitancy.

Dos Santos, C. F., et al. (2020). "COVID-19 and Mental Health-What Do We Know So Far?" Front Psychiatry **11**: 565698.

The coronavirus disease 2019 pandemic brought several worldwide health, social, and economic disturbances-particularly associated with the imposed confinement measures-that raised concerns about an emerging public mental health crisis. Studies investigating the early mental health impact of the pandemic on general population and vulnerable groups, such as healthcare workers, revealed a high prevalence of stress, anxiety, and depression symptoms, among others, and found several risk and protective factors. Along with these findings, the risk of substance use, suicide, domestic violence, and complicated grief may increase. We further discuss interventions that can be applied at a governmental, institutional, and individual level to minimize the mental health consequences of the pandemic, such as using telehealth to provide remote support or practicing self-care. These interventions should be maintained after the initial outbreak, as current disturbances may impact long-term well-being. We encourage the development of longitudinal studies to assess long-term adaptive responses.

Dotson, G. S., et al. (2020). "Setting occupational exposure limits for antimicrobial agents: A case study based on a quaternary ammonium compound-based disinfectant." Toxicol Ind Health **36**(9): 619-633.

Antimicrobial agents have become an essential tool in controlling the transmission of severe acute respiratory syndrome coronavirus 2 (SARS-CoV-2), and guidelines on their use have been issued by various public health agencies. Through its Emerging Viral Pathogen Guidance for Antimicrobial Pesticides, the US Environmental Protection Agency has approved numerous surface disinfectant products for use against SARS-CoV-2. Despite their widespread use and range of associated health hazards, the majority of active ingredients in antimicrobial products, such as surface disinfectants, lack established occupational exposure limits (OELs) to assist occupational health professionals in characterizing risks from exposures to these chemicals. Based on established approaches from various organizations, a framework for deriving OELs specific to antimicrobial agents was developed that relies on a weight-of-evidence evaluation of the available data. This framework involves (1) a screening-level toxicological assessment based on a review of the existing literature and recommendations, (2) identification of the critical adverse effect(s) and dose-response relationship(s), (3) identification of alternative health-based exposure limits (HBELs), (4) derivation of potential OELs based on identified points of departure and uncertainty factors and/or modification of existing alternative HBELs, and (5) selection of an appropriate OEL. To demonstrate the use of this framework, a case study is described for selection of an OEL for a disinfectant product containing quaternary ammonium compounds (quats). Three potential OELs were derived for this product based on irritation toxicity data, developmental and reproductive toxicity (DART) data, and modification of an existing HBEL. The final selected OEL for the quats-containing product was 0.1 mg/m(3), derived from modification of an existing HBEL. This value represented the lowest resulting value of the three approaches, and thus, was considered protective of irritation and potential DART.

Dramowski, A., et al. (2020). "South African healthcare workers and COVID-19: A shared responsibility to protect a precious and limited resource." S Afr Med J **110**(7): 567-568.

Drenovska, K., et al. (2020). "Covid-19 pandemic and the skin." Int J Dermatol **59**(11): 1312-1319.

In the beginning of the COVID-19 outbreak, skin manifestations, if present, were not paid enough attention. Then, the focus moved toward the impact of the prolonged use of personal protective measures in both healthcare workers and patients. In the meantime, attention is increasingly paid to dermatology as a result of the concern for certain groups of dermatologic patients, including those whose condition may worsen by the thorough disinfection measures and those treated with immunosuppressants or immunomodulators. Following patients with psoriasis on biological therapy, as well as other inflammatory and autoimmune cutaneous disorders such as atopic dermatitis, pemphigus, pemphigoid diseases, and skin cancer provoked the interest of dermatologists. Finally, an intriguing question to the dermatologic society was whether skin changes during COVID-19 infection exist and what could be their diagnostic or prognostic value. Here, we summarize skin conditions during the COVID-19 pandemic, patient information, and expert recommendations and give an overview about the registries launched to document skin changes during COVID-19, as well as details about certain patient groups infected with SARS-CoV-2, for example, psoriasis, atopic dermatitis, and autoimmune bullous diseases.

Drum, E., et al. (2020). "Anesthesia and potential aerosol generation during magnetic resonance imaging in children with COVID-19." Paediatr Anaesth **30**(8): 944-946.

The American College of Radiology recommends minimizing Magnetic Resonance Imaging (MRI) in COVID‐19 patients, postponing non‐urgent exams, and using alternative imaging.(1)Sedation/anesthesia are aerosol generating procedures (AGP) due to requirement of bag‐mask ventilation, intubation, and extubation with consequent risk of exposure to healthcare workers. This is complicated by limitation in the use of personal protective equipment (PPE) in the magnet zone (Zone IV).We describe our experience for children requiring anesthesia for emergency MRI during the COVID‐19 outbreak in Philadelphia.

Du, Q., et al. (2021). "Nosocomial infection of COVID‑19: A new challenge for healthcare professionals (Review)." Int J Mol Med **47**(4).

Nosocomial infections, also known as hospital-acquired infections, pose a serious challenge to healthcare professionals globally during the Coronavirus disease 2019 (COVID‑19) pandemic. Nosocomial infection of COVID‑19 directly impacts the quality of life of patients, as well as results in extra expenditure to hospitals. It has been shown that COVID‑19 is more likely to transmit via close, unprotected contact with infected patients. Additionally, current preventative and containment measures tend to overlook asymptomatic individuals and superspreading events. Since the mode of transmission and real origin of COVID‑19 in hospitals has not been fully elucidated yet, minimizing nosocomial infection in hospitals remains a difficult but urgent task for healthcare professionals. Healthcare professionals globally should form an alliance against nosocomial COVID‑19 infections. The fight against COVID‑19 may provide valuable lessons for the future prevention and control of nosocomial infections. The present review will discuss some of the key strategies to prevent and control hospital‑based nosocomial COVID‑19 infections.

Ebigbo, A., et al. (2021). "Cost-effectiveness analysis of SARS-CoV-2 infection prevention strategies including pre-endoscopic virus testing and use of high risk personal protective equipment." Endoscopy **53**(2): 156-161.

BACKGROUND: Infection prevention strategies to protect healthcare workers in endoscopy units during the post-peak phase of the COVID-19 pandemic are currently under intense discussion. In this paper, the cost-effectiveness of routine pre-endoscopy testing and high risk personal protective equipment (PPE) is addressed. METHOD: A model based on theoretical assumptions of 10 000 asymptomatic patients presenting to a high volume center was created. Incremental cost-effectiveness ratios (ICERs) and absolute costs per endoscopy were calculated using a Monte Carlo simulation. RESULTS: ICER values for universal testing decreased with increasing prevalence rates. For higher prevalence rates (≥ 1 %), ICER values were lowest for routine pre-endoscopy testing coupled with use of high risk PPE, while cost per endoscopy was lowest for routine use of high risk PPE without universal testing. CONCLUSION: In general, routine pre-endoscopy testing combined with high risk PPE becomes more cost-effective with rising prevalence rates of COVID-19.

Edge, H. M., et al. (2020). "The role of Force Health Protection in the Canadian Armed Forces' response to the COVID-19 pandemic." Can Commun Dis Rep **46**(9): 279-281.

Within the Canadian Armed Forces (CAF), public health is managed by the Directorate of Force Health Protection (DFHP), a branch of the Canadian Forces Health Services Group. Since the emergence of the novel coronavirus (SARS-CoV-19), DFHP has become heavily involved in health surveillance, outbreak monitoring, policy development, providing evidence-based guidance and advice, liaising with other national, provincial and territorial, municipal and international public health agencies, and ensuring environmental safety of CAF members. Some specific activities include supporting operations and deployments, amending policies and training and promoting hand hygiene, physical distancing and personal protective equipment use. In addition to taking measures to protect its members, CAF-Department of National Defence has contributed to Canada's national response to coronavirus disease 2019 (COVID-19). The DFHP will be developing training for allied health professionals to assist with contact tracing and follow-up, and will ensure adequate resources are in place to manage surge capacity for COVID-19. With these ongoing efforts, initiatives and lessons learned, DFHP is well placed to carry on with its mandate to protect and promote the health and well-being of CAF members and National Defence civilian employees, assisting Canadians and ensuring that CAF members are ready to serve their missions at home and abroad.

Edington, M., et al. (2020). "Virucidal benefits of povidone-iodine use on the ocular surface: a review." BMJ Open Ophthalmol **5**(1): e000509.

Povidone-iodine (PVI) preparations are well known for their microbicidal effect. In ophthalmology, PVI is commonly used to sterilise the ocular surface prior to surgical procedures. It is also used uncommonly as treatment for adenoviral conjunctivitis, yet the virucidal benefits of PVI have not been clearly documented in existing clinical management guidelines for ocular surface conditions. The COVID-19 pandemic, caused by the novel severe acute respiratory syndrome coronavirus 2 (SARS-CoV-2) virus, has challenged traditional healthcare systems. The morbidity and mortality of this highly contagious disease have resulted in fatalities among healthcare workers, including ophthalmologists. The SARS-CoV-2 virus has been identified on conjunctival testing, a potential source of contagious infection which may be unrecognised in asymptomatic carriers. Concern has been raised that ocular procedures may be 'aerosol-generating' and the additional wearing of personal protective equipment has been recommended to protect operating theatre staff. This literature review demonstrates that PVI has a broad virucidal activity, including against coronaviruses. It is already used perioperatively as standard of ophthalmic care and has been shown clinically to be effective against adenoviruses on the ocular surface. The current surgical practice of application of 5%-10% PVI applied periocularly for 3 min seems to provide an adequate effective reduction in the patient's ocular surface viral load. The virucidal benefits of routine PVI use should be included in ophthalmology guidelines regarding safe ocular surgery protocols.

Ehrlich, H., et al. (2020). "Protecting our healthcare workers during the COVID-19 pandemic." Am J Emerg Med **38**(7): 1527-1528.

Eijkholt, M., et al. (2021). "Should neurosurgeons continue to work in the absence of personal protective equipment during the COVID-19 era?" Acta Neurochir (Wien) **163**(3): 593-598.

The COVID-19 pandemic has resulted in a widespread shortage of personal protective equipment (PPE). Many healthcare workers, including neurosurgeons, have expressed concern about how to safely and adequately perform their medical responsibilities in these challenging circumstances. One of these concerns revolves around the pressing question: should providers continue to work in the absence of adequate PPE? Although the first peak of the COVID-19 crisis seems to have subsided and supply of PPE has increased, concerns about insufficient PPE availability remain. Inconsistent supply, limited efficacy, and continued high demand for PPE, combined with the continued threat of a second COVID-19 wave, mean that the issues surrounding PPE availability remain unresolved, including a duty to work. This paper offers an ethical investigation of whether neurosurgeons should perform their professional responsibilities with limited availability of PPE. We evaluate ethical considerations and conflicting duties and thereby hope to facilitate providers in making a well-considered personal and moral decision about this challenging issue.

Ejeh, F. E., et al. (2020). "Knowledge, attitude, and practice among healthcare workers towards COVID-19 outbreak in Nigeria." Heliyon **6**(11): e05557.

BACKGROUND: Severe acute respiratory syndrome-coronavirus-2 (SARS-CoV-2) infection is a global pandemic. Healthcare workers' (HCWs) role in patient management is predisposing and can serve as means of hospitals and community transmission. This study evaluated HCWs' knowledge, attitude, and practice towards COVID-19 in Nigeria. METHODS: we carried out a cross-sectional survey among HCWs during the COVID-19 outbreak in Nigeria from March to June 2020. The study assessed 346 HCWs for Knowledge, attitude, and practice by using an online (Google form) self-administered questionnaire, based on a convinience sampling technique Data were retrieved and analyzed using descriptive statistics. Chi-Square and one-way ANOVA were used to measure association and difference among demographic variables. The relationship between knowledge, attitude, and practice was measured using Spearman's rho correlation test. RESULTS: the mean knowledge score of the HCWs was 7.1 on a scale of 0-8. The correct overall rate of the knowledge questionnaire was 88.75%. Knowledge was gained mainly from television (35.0%) and social media (35.0%). The mean attitude score on a scale of 0-6 was 5.31 ± 0.39. Most (92.5%) participants were confident that Nigerian medical scientists would win the war against COVID-19. The majority (92.2%) of the respondents thought that SARS-CoV-2 was not a biological weapon. About 1 out of 5 respondents held that faith healing or prayer is the only cure for COVID-19. A vast majority of the HCWs were taking precautionary measures such as avoiding crowded places (94.2%), washing of hands (96.0%), and the use of personal protective equipment (91.6%) against SARS-CoV-2 infection. Nevertheless, only 3 out of 5 HCWs used a face mask when leaving home. There was a significant (p = 0.046) positive correlation (0.584) between knowledge and attitude. CONCLUSION: our results showed that HCWs in Nigeria had excellent knowledge and possessed a positive attitude and good practice towards COVID-19. However; there were areas where poor knowledge, negative attitudes and unacceptable practices were observed. We recommend continuous public health education of HCWs on SARS-COV-2 infection control and prevention.

El Abdellati, K., et al. (2021). "Hospital-wide SARS-CoV-2 antibody screening of staff in a university psychiatric centre in Belgium." BJPsych Open **7**(1): e40.

In this first serosurvey among psychiatric healthcare providers, only 3.2% of a sample of 431 staff members of a Belgian University Psychiatric Centre, screened 3-17 June 2020, had SARS-CoV-2 immunoglobulin G antibodies, which is considerably lower compared with both the general population and other healthcare workers in Belgium. The low seroprevalence was unexpected, given the limited availability of personal protective equipment and the high amount of COVID-19 symptoms reported by staff members. Importantly, exposure at home predicted the presence of antibodies, but exposure at work did not. Measures to prevent transmission from staff to patients are warranted in psychiatric facilities.

El Boghdady, M. and B. M. Ewalds-Kvist (2021). "Laparoscopic Surgery and the debate on its safety during COVID-19 pandemic: A systematic review of recommendations." Surgeon **19**(2): e29-e39.

INTRODUCTION: The transmission of COVID-19 virus since the outbreak of viral pneumonia due to SARS-CoV-2 gave rise to protective operative measures. Aerosol generating procedures such as laparoscopic surgery are known to be associated with increased risks of viral transmission to the healthcare workers. The safety of laparoscopy during the pandemic was then debated. We aimed to systematically review the literature regarding the safe use of laparoscopy during COVID-19. METHODS: We performed a systematic search using PubMed and ScienceDirect databases from inception to 1st May, 2020. The following search terms were used: ''laparoscopic surgery and COVID-19''; ''minimally invasive surgery and COVID-19''. Search items were considered from the nature of the articles, date of publication, aims and findings in relation to use of laparoscopic surgery during COVID-19. The study protocol was registered with PROSPERO register for systematic reviews (CRD42020183432). RESULTS: Altogether, 174 relevant citations were identified and reviewed for this study, of which 22 articles were included. The analysis of the findings in relation to laparoscopic surgery during the pandemic were presented in tabular form. We completed the common recommendations for performing laparoscopy during the COVID-19 pandemic in forms of pre-, intra- and postoperative phases. CONCLUSION: There is no scientific evidence to date for the transmission of COVID-19 by laparoscopic surgery. Laparoscopy can be used with precautions because of its benefits compared to open surgery. If safe, conservative management is the primary alternative during the pandemic. We concluded that recommended precautions should be respected while performing laparoscopy during the pandemic.

El Kassas, M., et al. (2020). "Liver transplantation in the era of COVID-19." Arab J Gastroenterol **21**(2): 69-75.

Liver transplantation is considered the ultimate solution for patients with end-stage chronic liver disease or acute liver failure. Patients with liver transplant need special care starting from preoperative preparation, surgical intervention ending with postoperative care. Transplanted patients have to receive immunosuppressive therapy to prevent rejection. Such a state of immune suppression could predispose to different types of infections in liver transplant recipients. Currently, the world is suffering a pandemic caused by a new strain of the coronavirus family called COVID-19. Certain infection control precautions are needed to protect immunocompromised and vulnerable patients, including liver transplant candidates and recipients from acquiring COVID-19 infection. Restricting non-transplant elective surgical procedures, managing transplant patients in separate outpatient clinics, and in-patient wards can prevent transmission of infection both to patients and healthcare workers. Telemedicine can help in the triage of patients to screen for symptoms of COVID-19 before their regular appointment. Management of immunosuppressive therapy and drug-drug interactions in liver transplant recipients infected with COVID-19 should be cautiously practiced to prevent rejection and effectively treat the underlying infection. In this report, we are trying to summarize available evidence about different aspects of the management of liver transplant candidates and recipients in the era of COVID-19.

Elbay, R. Y., et al. (2020). "Depression, anxiety, stress levels of physicians and associated factors in Covid-19 pandemics." Psychiatry Res **290**: 113130.

AIM: To investigate anxiety, stress, and depression levels of physicians during the Covid-19 outbreak and explored associated factors in both clinical and general site. METHODS: An online survey is conducted to asses psychological responses of healthcare workers and related factors during Covid-19 outbreak. It is consisted of three subsections covering the following areas: 1) sociodemographic data 2) information on individuals` working condition 3) Depression Anxiety and Stress Scale-21 (DAS-21). RESULTS: Of all 442 participants, 286 (64.7%) had symptoms of depression, 224 (51.6%) anxiety, and 182 (41.2%) stress. Being female, young, and single, having less work experience, working in frontline were associated with higher scores, whereas having a child was associated with lower scores in each subscale. Factors found to be associated with higher DAS-21 total scores in frontline workers were as follows: increased weekly working hours, increased number of Covid-19 patients cared for, lower level of support from peers and supervisors, lower logistic support, and lower feelings of competence during Covid-19 related tasks. CONCLUSIONS: Our findings highlight the factors which need to be taken into consideration to protect the mental wellbeing of doctors while fighting with a disaster that has major impacts on society worldwide.

Elbeddini, A., et al. (2020). "Mental health issues impacting pharmacists during COVID-19." J Pharm Policy Pract **13**: 46.

The coronavirus disease 2019 (COVID-19) impact on the mental health of healthcare workers is extremely detrimental. It is imperative that the psychological health of all healthcare workers be protected. However, an often overlooked member of the healthcare frontline is the pharmacist. Pharmacists provide many types of essential services during the pandemic, which often cannot be done from a remote location. Being frontline healthcare workers, pharmacists have experienced an increase in the number of patients seen, the amount of screening and triage being done, the amount of COVID-19 information being delivered, the number of medication shortages, and the amount of workplace harassment taking place. These activities increase the amount of stress, burden, and frustration felt by pharmacists have a negative impact on their mental health and well-being. This article seeks to address the specific implications of COVID-19 on the mental health of pharmacists.

El-Boghdadly, K., et al. (2021). "SARS-CoV-2 infection, COVID-19 and timing of elective surgery: A multidisciplinary consensus statement on behalf of the Association of Anaesthetists, the Centre for Peri-operative Care, the Federation of Surgical Specialty Associations, the Royal College of Anaesthetists and the Royal College of Surgeons of England." Anaesthesia.

The scale of the COVID-19 pandemic means that a significant number of patients who have previously been infected with SARS-CoV-2 will require surgery. Given the potential for multisystem involvement, timing of surgery needs to be carefully considered to plan for safe surgery. This consensus statement uses evidence from a systematic review and expert opinion to highlight key principles in the timing of surgery. Shared decision-making regarding timing of surgery after SARS-CoV-2 infection must account for severity of the initial infection; ongoing symptoms of COVID-19; comorbid and functional status; clinical priority and risk of disease progression; and complexity of surgery. For the protection of staff, other patients and the public, planned surgery should not be considered during the period that a patient may be infectious. Precautions should be undertaken to prevent pre- and peri-operative infection, especially in higher risk patients. Elective surgery should not be scheduled within 7 weeks of a diagnosis of SARS-CoV-2 infection unless the risks of deferring surgery outweigh the risk of postoperative morbidity or mortality associated with COVID-19. SARS-CoV-2 causes either transient or asymptomatic disease for most patients, who require no additional precautions beyond a 7-week delay, but those who have persistent symptoms or have been hospitalised require special attention. Patients with persistent symptoms of COVID-19 are at increased risk of postoperative morbidity and mortality even after 7 weeks. The time before surgery should be used for functional assessment, prehabilitation and multidisciplinary optimisation. Vaccination several weeks before surgery will reduce risk to patients and might lessen the risk of nosocomial SARS-CoV-2 infection of other patients and staff. National vaccine committees should consider whether such patients can be prioritised for vaccination. As further data emerge, these recommendations may need to be revised, but the principles presented should be considered to ensure safety of patients, the public and staff.

El-Boghdadly, K., et al. (2020). "Risks to healthcare workers following tracheal intubation of patients with COVID-19: a prospective international multicentre cohort study." Anaesthesia **75**(11): 1437-1447.

Healthcare workers involved in aerosol-generating procedures, such as tracheal intubation, may be at elevated risk of acquiring COVID-19. However, the magnitude of this risk is unknown. We conducted a prospective international multicentre cohort study recruiting healthcare workers participating in tracheal intubation of patients with suspected or confirmed COVID-19. Information on tracheal intubation episodes, personal protective equipment use and subsequent provider health status was collected via self-reporting. The primary endpoint was the incidence of laboratory-confirmed COVID-19 diagnosis or new symptoms requiring self-isolation or hospitalisation after a tracheal intubation episode. Cox regression analysis examined associations between the primary endpoint and healthcare worker characteristics, procedure-related factors and personal protective equipment use. Between 23 March and 2 June 2020, 1718 healthcare workers from 503 hospitals in 17 countries reported 5148 tracheal intubation episodes. The overall incidence of the primary endpoint was 10.7% over a median (IQR [range]) follow-up of 32 (18-48 [0-116]) days. The cumulative incidence within 7, 14 and 21 days of the first tracheal intubation episode was 3.6%, 6.1% and 8.5%, respectively. The risk of the primary endpoint varied by country and was higher in women, but was not associated with other factors. Around 1 in 10 healthcare workers involved in tracheal intubation of patients with suspected or confirmed COVID-19 subsequently reported a COVID-19 outcome. This has human resource implications for institutional capacity to deliver essential healthcare services, and wider societal implications for COVID-19 transmission.

Elhadi, M., et al. (2020). "Concerns for low-resource countries, with under-prepared intensive care units, facing the COVID-19 pandemic." Infect Dis Health **25**(4): 227-232.

BACKGROUND: Low-resource countries with fragile healthcare systems lack trained healthcare professionals and specialized resources for COVID-19 patient hospitalization, including mechanical ventilators. Additional socio-economic complications such as civil war and financial crisis in Libya and other low-resource countries further complicate healthcare delivery. METHODS: A cross-sectional survey evaluating hospital and intensive care unit's capacity and readiness was performed from 16 leading Libyan hospitals in March 2020. In addition, a survey was conducted among 400 doctors who worked in these hospitals to evaluate the status of personal protective equipment. RESULTS: Out of 16 hospitals, the highest hospital capacity was 1000 in-patient beds, while the lowest was 25 beds with a median of 200 (IQR 52-417, range 25-1000) hospital beds. However, a median of only eight (IQR 6-14, range 3-37) available functioning ICU beds were reported in these hospitals. Only 9 (IQR 4.5-14, range 2-20) mechanical ventilators were reported and none of the hospitals had a reverse transcription-polymerase chain reaction machine for COVID-19 testing. Moreover, they relied on one of two central laboratories located in major cities. Our PPE survey revealed that 56.7% hospitals lacked PPE and 53% of healthcare workers reported that they did not receive proper PPE training. In addition, 70% reported that they were buying the PPE themselves as hospitals did not provide them. CONCLUSION: This study provides an alarming overview of the unpreparedness of Libyan hospitals for detecting and treating patients with COVID-19 and limiting the spread of the pandemic.

Elhadi, M., et al. (2020). "Assessment of Healthcare Workers' Levels of Preparedness and Awareness Regarding COVID-19 Infection in Low-Resource Settings." Am J Trop Med Hyg **103**(2): 828-833.

COVID-19, caused by the SARS-CoV-2 virus, is spreading rapidly worldwide, with devastating consequences for patients, healthcare workers, health systems, and economies. As it reaches low- and middle-income countries, the pandemic puts healthcare workers at high risk and challenges the abilities of healthcare systems to respond to the crisis. This study measured levels of knowledge and preparedness regarding COVID-19 among physicians and nurses. A cross-sectional survey was conducted among healthcare workers in Libya between February 26 and March 10, 2020. We obtained 1,572 valid responses of a possible 2,000 (78.6%) participants from 21 hospitals, of which 65.1% were from physicians and 34.9% from nurses. The majority of participants (70%) used social media as a source of information. A total of 47.3% of doctors and 54.7% of nurses received adequate training on how to effectively use personal protective equipment. Low confidence in managing suspected COVID-19 patients was reported by 83.8% of participants. Furthermore, 43.2% of healthcare workers were aware of proper hand hygiene techniques. Less than 7% of participants received training on how to manage COVID-19 cases, whereas 20.6% of doctors and 26.3% of nurses felt that they were personally prepared for the outbreak. Awareness and preparedness for the pandemic were low among frontline workers during the study. Therefore, an effective educational training program should be implemented to ensure maintenance of appropriate practices during the COVID-19 pandemic.

El-Hage, W., et al. (2020). "[Health professionals facing the coronavirus disease 2019 (COVID-19) pandemic: What are the mental health risks?]." Encephale **46**(3s): S73-s80.

OBJECTIVES: The coronavirus disease 2019 (COVID-19) pandemic has caused major sanitary crisis worldwide. Half of the world has been placed in quarantine. In France, this large-scale health crisis urgently triggered the restructuring and reorganization of health service delivery to support emergency services, medical intensive care units and continuing care units. Health professionals mobilized all their resources to provide emergency aid in a general climate of uncertainty. Concerns about the mental health, psychological adjustment, and recovery of health care workers treating and caring for patients with COVID-19 are now arising. The goal of the present article is to provide up-to-date information on potential mental health risks associated with exposure of health professionals to the COVID-19 pandemic. METHODS: Authors performed a narrative review identifying relevant results in the scientific and medical literature considering previous epidemics of 2003 (SARS-CoV-1) and 2009 (H1N1) with the more recent data about the COVID-19 pandemic. We highlighted most relevant data concerning the disease characteristics, the organizational factors and personal factors that may contribute to developing psychological distress and other mental health symptoms. RESULTS: The disease characteristics of the current COVID-19 pandemic provoked a generalized climate of wariness and uncertainty, particularly among health professionals, due to a range of causes such as the rapid spread of COVID-19, the severity of symptoms it can cause in a segment of infected individuals, the lack of knowledge of the disease, and deaths among health professionals. Stress may also be caused by organizational factors, such as depletion of personal protection equipment, concerns about not being able to provide competent care if deployed to new area, concerns about rapidly changing information, lack of access to up-to-date information and communication, lack of specific drugs, the shortage of ventilators and intensive care unit beds necessary to care for the surge of critically ill patients, and significant change in their daily social and family life. Further risk factors have been identified, including feelings of being inadequately supported, concerns about health of self, fear of taking home infection to family members or others, and not having rapid access to testing through occupational health if needed, being isolated, feelings of uncertainty and social stigmatization, overwhelming workload, or insecure attachment. Additionally, we discussed positive social and organizational factors that contribute to enhance resilience in the face of the pandemic. There is a consensus in all the relevant literature that health care professionals are at an increased risk of high levels of stress, anxiety, depression, burnout, addiction and post-traumatic stress disorder, which could have long-term psychological implications. CONCLUSIONS: In the long run, this tragic health crisis should significantly enhance our understanding of the mental health risk factors among the health care professionals facing the COVID-19 pandemic. Reporting information such as this is essential to plan future prevention strategies. Protecting health care professionals is indeed an important component of public health measures to address large-scale health crisis. Thus, interventions to promote mental well-being in health care professionals exposed to COVID-19 need to be immediately implemented, and to strengthen prevention and response strategies by training health care professionals on mental help and crisis management.

Publisher: Abstract available from the publisher.

fre

Elliott, J. A., et al. (2020). "Nosocomial SARS-CoV-2 transmission in postoperative infection and mortality: analysis of 14 798 procedures." Br J Surg **107**(13): 1708-1712.

This study used a national administrative database to estimate perioperative SARS-CoV-2 infection risk, and associated mortality, relative to nosocomial transmission rates. The impact of nosocomial transmission was greatest after major emergency surgery, whereas laparoscopic surgery may be protective owing to reduced duration of hospital stay. Procedure-specific risk estimates are provided to facilitate surgical decision-making and informed consent. Estimated risks.

Elmore, R., et al. (2020). "Risk and Protective Factors in the COVID-19 Pandemic: A Rapid Evidence Map." Front Public Health **8**: 582205.

Background: Given the worldwide spread of the 2019 Novel Coronavirus (COVID-19), there is an urgent need to identify risk and protective factors and expose areas of insufficient understanding. Emerging tools, such as the Rapid Evidence Map (rEM), are being developed to systematically characterize large collections of scientific literature. We sought to generate an rEM of risk and protective factors to comprehensively inform areas that impact COVID-19 outcomes for different sub-populations in order to better protect the public. Methods: We developed a protocol that includes a study goal, study questions, a PECO statement, and a process for screening literature by combining semi-automated machine learning with the expertise of our review team. We applied this protocol to reports within the COVID-19 Open Research Dataset (CORD-19) that were published in early 2020. SWIFT-Active Screener was used to prioritize records according to pre-defined inclusion criteria. Relevant studies were categorized by risk and protective status; susceptibility category (Behavioral, Physiological, Demographic, and Environmental); and affected sub-populations. Using tagged studies, we created an rEM for COVID-19 susceptibility that reveals: (1) current lines of evidence; (2) knowledge gaps; and (3) areas that may benefit from systematic review. Results: We imported 4,330 titles and abstracts from CORD-19. After screening 3,521 of these to achieve 99% estimated recall, 217 relevant studies were identified. Most included studies concerned the impact of underlying comorbidities (Physiological); age and gender (Demographic); and social factors (Environmental) on COVID-19 outcomes. Among the relevant studies, older males with comorbidities were commonly reported to have the poorest outcomes. We noted a paucity of COVID-19 studies among children and susceptible sub-groups, including pregnant women, racial minorities, refugees/migrants, and healthcare workers, with few studies examining protective factors. Conclusion: Using rEM analysis, we synthesized the recent body of evidence related to COVID-19 risk and protective factors. The results provide a comprehensive tool for rapidly elucidating COVID-19 susceptibility patterns and identifying resource-rich/resource-poor areas of research that may benefit from future investigation as the pandemic evolves.

Erismis, B., et al. (2021). "Annual influenza vaccination effect on the susceptibility to COVID-19 infection." Cent Eur J Public Health **29**(1): 14-17.

OBJECTIVES: We aimed to study the effect of seasonal influenza (flu) vaccination on the susceptibility to coronavirus disease 2019 (COVID-19). METHODS: A total of 203 healthcare workers of a pandemic centre of Istanbul, Turkey, were included in this retrospective study. According to the presence or absence of flu vaccination, participants were divided into group 1 and group 2. A comparison of the rate of COVID-19 was done between these two groups. Also, the mean age and the sex ratio of females/males were evaluated and compared between these two groups. RESULTS: Group 1 participants (n = 65) were older than participants in group 2 (n = 138) (p < 0.05). Despite of this, interestingly, the COVID-19 infection rate was lower in the 1st group (in comparison to the 2nd group) (p < 0.05). CONCLUSION: Our study results showed that, even if low, the flu vaccination may have a protective effect on the susceptibility to COVID-19 infection. Using this beneficial adjuvant effect of the vaccine may help us in this unpredictable battle with the COVID-19 pandemic. Further studies are needed to confirm this assumption.

Erol, V., et al. (2020). "Recommendations for bariatric and metabolic surgical operations during the COVID-19 pandemic in Turkey." Turk J Surg **36**(2): 132-136.

The world has been struggling with the COVID-19 virus since December 2019. Turkey has also been battling with the virus since March 2019. While struggling with this unknown virus, we have postponed our new bariatric surgeries like most elective surgery. However, curfew and quarantine period (increase in food intake and decreased physical activity) increases risks for morbidity and mortality because of obesity and diabetes. When the pandemic decreases and disappears, many obesity patients will seek treatment for obesity and the workload of surgeons will increase. Before bariatric and metabolic surgery operations, which is the most effective treatment of obesity and related comorbidities, necessary precautions must be determined and implemented to protect patients and healthcare workers before and during surgery. In this review, it was aimed to determine the pre-peri and postoperative periods of bariatric surgical requirements. This review has been written on behalf of the Turkish Society for Metabolic and Bariatric Surgery as an initiative in order to answer some questions about bariatric and metabolic surgery during the COVID-19 pandemic.

Erquicia, J., et al. (2020). "Emotional impact of the Covid-19 pandemic on healthcare workers in one of the most important infection outbreaks in Europe." Med Clin (Engl Ed) **155**(10): 434-440.

BACKGROUND AND OBJECTIVES: The Covid-19 pandemic has put healthcare professionals around the world in an unprecedented challenge. This may cause some emotional difficulties and mental health problems. The aim of the present study was to analyze the emotional status among the health care workers form the Hospital of Igualada (Barcelona), while they were facing with Covid-19 in one of the most affected regions in all of Europe. PATIENTS AND METHODS: A total of 395 participants were included in the study. A cross-sectional assessment was carried out between the months of March and April. Information about anxiety, depression, and stress was gathered. We also collected demographic data and concerning potentially stressful factors. RESULTS: A significant proportion of professionals reported symptoms of anxiety (31.4%) and depression (12.2%) from moderate to severe intensity. Symptoms of acute stress were reported by 14.5% of participants. We performed a regression analysis, which explained the 30% of the variance associated with the degree of emotional distress (R² = 0.30). The final model reveals that females (or young males), who are working in the frontline as nursing assistants, caretakers or radiology technicians, with the uncertainty of a possible infection, the perception of inadequate protection measures and having experienced the death of a close person by Covid-19, showed a heightened risk of experiencing psychological distress. CONCLUSIONS: Coping with the Covid-19 pandemic caused a significant impact on emotional status of healthcare workers involved in this study.

Erquicia, J., et al. (2020). "Emotional impact of the Covid-19 pandemic on healthcare workers in one of the most important infection outbreaks in Europe." Med Clin (Barc) **155**(10): 434-440.

BACKGROUND AND OBJECTIVES: The Covid-19 pandemic has put healthcare professionals around the world in an unprecedented challenge. This may cause some emotional difficulties and mental health problems. The aim of the present study was to analyze the emotional status among the health care workers form the Hospital of Igualada (Barcelona), while they were facing with Covid-19 in one of the most affected regions in all of Europe. PATIENTS AND METHODS: A total of 395 participants were included in the study. A cross-sectional assessment was carried out between the months of March and April. Information about anxiety, depression, and stress was gathered. We also collected demographic data and concerning potentially stressful factors. RESULTS: A significant proportion of professionals reported symptoms of anxiety (31.4%) and depression (12.2%) from moderate to severe intensity. Symptoms of acute stress were reported by 14.5% of participants. We performed a regression analysis, which explained the 30% of the variance associated with the degree of emotional distress (Ry=0.30). The final model reveals that females (or young males), who are working in the frontline as nursing assistants, caretakers or radiology technicians, with the uncertainty of a possible infection, the perception of inadequate protection measures and having experienced the death of a close person by Covid-19, showed a heightened risk of experiencing psychological distress. CONCLUSIONS: Coping with the Covid-19 pandemic caused a significant impact on emotional status of healthcare workers involved in this study.

BACKGROUND AND OBJECTIVES: The Covid-19 pandemic has put healthcare professionals around the world in an unprecedented challenge. This may cause some emotional difficulties and mental health problems. The aim of the present study was to analyze the emotional status among the health care workers form the Hospital of Igualada (Barcelona), while they were facing with Covid-19 in one of the most affected regions in all of Europe. PATIENTS AND METHODS: A total of 395 participants were included in the study. A cross-sectional assessment was carried out between the months of March and April. Information about anxiety, depression, and stress was gathered. We also collected demographic data and concerning potentially stressful factors. RESULTS: A significant proportion of professionals reported symptoms of anxiety (31.4%) and depression (12.2%) from moderate to severe intensity. Symptoms of acute stress were reported by 14.5% of participants. We performed a regression analysis, which explained the 30% of the variance associated with the degree of emotional distress (Ry = 0.30). The final model reveals that females (or young males), who are working in the frontline as nursing assistants, caretakers or radiology technicians, with the uncertainty of a possible infection, the perception of inadequate protection measures and having experienced the death of a close person by Covid-19, showed a heightened risk of experiencing psychological distress. CONCLUSIONS: Coping with the Covid-19 pandemic caused a significant impact on emotional status of healthcare workers involved in this study.

eng

Escudero, D., et al. (2020). "SARS-CoV-2 analysis on environmental surfaces collected in an intensive care unit: keeping Ernest Shackleton's spirit." Intensive Care Med Exp **8**(1): 68.

BACKGROUND: Intensive care unit workers are at high risk of acquiring COVID-19 infection, especially when performing invasive techniques and certain procedures that generate aerosols (< 5 μm). Therefore, one of the objectives of the health systems should implement safety practices to minimize the risk of contagion among these health professionals. Monitoring environmental contamination of SARS-CoV-2 may help to determine the potential of the environment as a transmission medium in an area highly exposed to SARS-CoV-2, such as an intensive care unit. The objective of the study was to analyze the environmental contamination by SARS-CoV-2 on surfaces collected in an intensive care unit, which is dedicated exclusively to the care of patients with COVID-19 and equipped with negative pressure of - 10 Pa and an air change rate of 20 cycles per hour. Furthermore, all ICU workers were tested for COVID-19 by quantitative RT-PCR and ELISA methods. RESULTS: A total of 102 samples (72 collected with pre-moistened swabs used for collection of nasopharyngeal exudates and 30 with moistened wipes used in the environmental microbiological control of the food industry) were obtained from ventilators, monitors, perfusion pumps, bed rails, lab benches, containers of personal protective equipment, computer keyboards and mice, telephones, workers' shoes, floor, and other areas of close contact with COVID-19 patients and healthcare professionals who cared for them. The analysis by quantitative RT-PCR showed no detection of SARS-CoV-2 genome in environmental samples collected by any of the two methods described. Furthermore, none of the 237 ICU workers was infected by the virus. CONCLUSIONS: Presence of SARS-CoV-2 on the ICU surfaces could not be determined supporting that a strict cleaning protocol with sodium hypochlorite, a high air change rate, and a negative pressure in the ICU are effective in preventing environmental contamination. These facts together with the protection measures used could also explain the absence of contagion among staff inside ICUs.

Escudero, X., et al. (2020). "The SARS-CoV-2 (COVID-19) coronavirus pandemic: current situation and implications for Mexico." Arch Cardiol Mex **90**(Supl): 7-14.

The Coronavirus pandemic (COVID-19) is one of the most devastating in this century. It originated in China in December 2019 caused by the SARS-Cov-2 virus, and in less than a month it had been classified as an "International Public Health Emergency". To date there are nearly 3 million people infected and more than 250,000 deaths caused by the disease worldwide. Initially it affects the respiratory tract with atypical pneumonia and in severe cases it produces systemic inflammation with cytokine storm that can cause rapid deterioration with circulatory and respiratory failure, coagulopathy and a lethality rate of approximately 7%. In Mexico, the first case was detected in February 2020, and to date there are 26,616 confirmed cases and 2,961 deaths throughout the country. The low number of diagnostic tests conducted in our country clearly underestimates the real incidence and impact of the disease. The most affected groups are those with risk factors such as age over 60, presence of hypertension, diabetes or cardiovascular disease. Of the confirmed cases, 15% are healthcare workers. There is no specific treatment or vaccine yet, so it is important to have hygiene, social isolation and personal protection measures. Health, social and economic consequences could have great impact in the near future.

Eshetie Adane, D., et al. (2021). "Preparedness level and associated factors of health professionals regarding COVID-19." SAGE Open Med **9**: 20503121211001151.

OBJECTIVE: To assess the level of preparedness of health professionals working in South Gondar Zone public hospital, Debre Tabor Comprehensive specialized Hospital for coronavirus. METHODS: An institutional-based cross-sectional survey was conducted with a self-administered questionnaire from April 20 to May 20. Descriptive statistics, chi-square test, bivariable, and multivariable logistic analysis were done. Bivariable and multivariable regression was held to determine significant predictors for preparedness. For all statistical analyses, a p value ⩽ 0.05 was considered as significant. RESULTS: From a total health professionals, 301 health professionals with a response rate of 86% participated in the study. Among these, 206 were males while 95 participants were females with a mean age of 29.71 ± 5.84 years. To determine the preparedness level, we use the mean preparedness score of the respondents. Around 64.1% of health professionals were well prepared. Among 20 respondents who had a chronic illness, 80% of them think their illness is risky for the pandemic. The majority of the respondents (229/76.1%) responded as they were working without protective equipment. From the multivariable analysis male sex (adjusted odds ratio = 2.07, 95% confidence interval = 1.193-3.590), family living with them (adjusted odds ratio = 4.507, 95% confidence interval = 1.863-10.90), and involvement of other previous outbreak (adjusted odds ratio = 2.245, 95% confidence interval = 1.176-4.286) were the predictors for good preparedness. CONCLUSION: Most of the health professionals were well prepared. Being male, living within a family, and involvement of other outbreaks leads to good preparation. Governmental bodies and hospital managers have to help health professionals to be prepared enough to combat the pandemic.

Ey, S., et al. (2020). "Protecting the Well-Being of Medical Residents and Faculty Physicians During the COVID-19 Pandemic: Making the Case for Accessible, Comprehensive Wellness Resources." Glob Adv Health Med **9**: 2164956120973981.

Accounts of frontline health care workers experiencing distress in the midst of the COVID-19 pandemic highlight the need for accessible psychological support for them. Prior to the pandemic, medical residents and physicians often experienced difficulty receiving counseling due to concerns about confidentiality, stigma, cost, time, and reportability to licensure/credentialing bodies. Since 2004, the OHSU Resident and Faculty Wellness Program (RFWP) team has sought to reduce these barriers by providing on-site free, confidential, individual counseling and medication management. Utilization of this program is high with over 500 physicians a year seeking care; 38% of all OHSU residents/fellows and 7% of all faculty eligible for our services participated in 2019-20. In the present essay, we describe how our model of care for trainees and faculty was a key wellness resource during COVID-19. Similar to other accounts of lower help-seeking by health professionals initially during the pandemic, we experienced a slight downturn in utilization rates during the initial weeks of when the pandemic struck our area, but quickly returned to normal and exceeded prior levels. All appointments shifted to telehealth visits and a number of physicians expressed gratitude for the opportunity to talk through concerns and strengthen coping. A number of physicians requested medication consultations to address severe insomnia, anxiety, and depression. We hope that being present in our physicians' lives when they are exposed to COVID-related stress or trauma will keep them safe, help them cope with difficult experiences and losses, and ultimately facilitate both recovery and post-traumatic growth.

Eyre, D. W., et al. (2020). "Differential occupational risks to healthcare workers from SARS-CoV-2 observed during a prospective observational study." Elife **9**.

We conducted voluntary Covid-19 testing programmes for symptomatic and asymptomatic staff at a UK teaching hospital using naso-/oro-pharyngeal PCR testing and immunoassays for IgG antibodies. 1128/10,034 (11.2%) staff had evidence of Covid-19 at some time. Using questionnaire data provided on potential risk-factors, staff with a confirmed household contact were at greatest risk (adjusted odds ratio [aOR] 4.82 [95%CI 3.45-6.72]). Higher rates of Covid-19 were seen in staff working in Covid-19-facing areas (22.6% vs. 8.6% elsewhere) (aOR 2.47 [1.99-3.08]). Controlling for Covid-19-facing status, risks were heterogenous across the hospital, with higher rates in acute medicine (1.52 [1.07-2.16]) and sporadic outbreaks in areas with few or no Covid-19 patients. Covid-19 intensive care unit staff were relatively protected (0.44 [0.28-0.69]), likely by a bundle of PPE-related measures. Positive results were more likely in Black (1.66 [1.25-2.21]) and Asian (1.51 [1.28-1.77]) staff, independent of role or working location, and in porters and cleaners (2.06 [1.34-3.15]).

Eyre, D. W., et al. (2021). "Stringent thresholds in SARS-CoV-2 IgG assays lead to under-detection of mild infections." BMC Infect Dis **21**(1): 187.

BACKGROUND: Thresholds for SARS-CoV-2 antibody assays have typically been determined using samples from symptomatic, often hospitalised, patients. In this setting the sensitivity and specificity of the best performing assays can both exceed 98%. However, antibody assay performance following mild infection is less clear. METHODS: We assessed quantitative IgG responses in a cohort of healthcare workers in Oxford, UK, with a high pre-test probability of Covid-19, in particular the 991/11,475(8.6%) who reported loss of smell/taste. We use anosmia/ageusia and other risk factors as probes for Covid-19 infection potentially undiagnosed by immunoassays by investigating their relationship with antibody readings either side of assay thresholds. RESULTS: The proportion of healthcare workers reporting anosmia/ageusia increased at antibody readings below diagnostic thresholds using an in-house ELISA (n = 9324) and the Abbott Architect chemiluminescent microparticle immunoassay (CMIA; n = 11,324): 426/906 (47%) reported anosmia/ageusia with a positive ELISA, 59/449 (13.1%) with high-negative and 326/7969 (4.1%) with low-negative readings. Similarly, by CMIA, 518/1093 (47.4%) with a positive result reported anosmia/ageusia, 106/686 (15.5%) with a high-negative and 358/9563 (3.7%) with a low-negative result. Adjusting for the proportion of staff reporting anosmia/ageusia suggests the sensitivity of both assays in mild infection is lower than previously reported: Oxford ELISA 89.8% (95%CI 86.6-92.8%) and Abbott CMIA 79.3% (75.9-82.7%). CONCLUSION: Following mild SARS-CoV-2 infection 10-30% of individuals may have negative immunoassay results. While lowered diagnostic thresholds may result in unacceptable specificity, our findings have implications for epidemiological analyses and result interpretation in individuals with a high pre-test probability. Samples from mild PCR-confirmed infections should be included in SARS-CoV-2 immunoassay evaluations.

Fadaak, R., et al. (2021). "Rapid conversion of an in-patient hospital unit to accommodate COVID-19: An interdisciplinary human factors, ethnography, and infection prevention and control approach." PLoS One **16**(1): e0245212.

BACKGROUND: In response to the Coronavirus disease-19 (COVID-19) pandemic, in-patient units in hospitals around the world have altered their patient care routines and Infection Prevention and Control (IPC) practices. Our interdisciplinary team of applied Human Factors (HF), ethnography, and IPC experts assisted one Unit, normally serving general surgical and orthopedic patients, as it rapidly converted to deliver COVID-19-specific care. This paper describes the conversion experience of the Unit, and outlines broader lessons for other acute care teams faced with similar issues. METHODS: We deployed walkthroughs, simulations, and ethnography to identify important safety gaps in care delivery processes on the Unit. These interventions were undertaken using interdisciplinary theories of implementation that combined systems-level HF perspectives, ethnographic approaches, and individual-level IPC perspectives. Timely recommendations were developed and delivered to Unit staff for feedback and implementation. RESULTS: We describe three interventions on the Unit: 1) the de-cluttering and re-organization of personal protective equipment (PPE); 2) the reconfiguring of designated 'dirty' tray tables and supplies; and 3) the redesign of handling pathways for 'dirty' linens and laundry. Each of these interventions was implemented to varying degrees, but all contributed to discussions of safety and IPC implementation that extended beyond the Unit and into the operations of the broader hospital. CONCLUSIONS: Leveraging our team's interdisciplinary expertise and blended approaches to implementation, the interventions assisted in the Unit's rapid conversion towards providing COVID-19-specific care. The deployment and implementation of the interventions highlight the potential of collaboration between HF, ethnography, and IPC experts to support frontline healthcare delivery under pandemic conditions in an effort to minimize nosocomial transmission potential in the acute healthcare setting.

Falcó-Pegueroles, A., et al. (2020). "Ethical conflict during COVID-19 pandemic: the case of Spanish and Italian intensive care units." Int Nurs Rev.

AIM: To identify factors underlying ethical conflict occurring during the current COVID-19 pandemic in the critical care setting. BACKGROUND: During the first wave of the COVID-19 outbreak, Spanish and Italian intensive care units were overwhelmed by the demand for admissions. This fact revealed a crucial problem of shortage of health resources and rendered that decision-making was highly complex. SOURCES OF EVIDENCE: Applying a nominal group technique this manuscript identifies a series of factors that may have played a role in the emergence of the ethical conflicts in critical care units during the COVID-19 pandemic, considering ethical principles and responsibilities included in the International Council of Nurses Code of Ethics. The five factors identified were the availability of resources; the protection of healthcare workers; the circumstances surrounding decision-making, end-of-life care, and communication. DISCUSSION: The impact of COVID-19 on health care will be long-lasting and nurses are playing a central role in overcoming this crisis. Identifying these five factors and the conflicts that have arisen during the COVID-19 pandemic can help to guide future policies and research. CONCLUSIONS: Understanding these five factors and recognizing the conflicts, they may create can help to focus our efforts on minimizing the impact of the ethical consequences of a crisis of this magnitude and on developing new plans and guidelines for future pandemics. IMPLICATIONS FOR NURSING PRACTICE AND POLICY: Learning more about these factors can help nurses, other health professionals, and policymakers to focus their efforts on minimizing the impact of the ethical consequences of a crisis of this scale. This will enable changes in organizational policies, improvement in clinical competencies, and development of the scope of practice.

Fang, M., et al. (2021). "Drivers and mediators of healthcare workers' anxiety in one of the most affected hospitals by COVID-19: a qualitative analysis." BMJ Open **11**(3): e045048.

OBJECTIVE: To report the driving and mediating factors of healthcare workers' anxiety during the COVID-19 pandemic. DESIGN: Qualitative indepth interview study. SETTING: The Central Hospital of Wuhan, Tongji Medical College, Huazhong University of Science and Technology, Wuhan, China. PARTICIPANTS: 53 healthcare workers who were or were not diagnosed with COVID-19. RESULTS: During the COVID-19 pandemic, healthcare workers were initially not sufficiently psychologically prepared. Then they suffered from severe anxiety and apprehension during the peak stage, regardless of whether they were infected with SARS-CoV-2 or not. These negative emotions were exacerbated by four drivers, namely infection risk, supplies, isolation and media. As the epidemic gradually came under control, healthcare workers experienced less anxiety from these factors, but became concerned about their low financial status. To reduce anxiety, healthcare workers diverted their attention from the risk of infection through personal entertainment and religious beliefs and focused on treating their patients. Furthermore, assistance from other people, including colleagues, families, friends, patients and society, helped protect healthcare workers from anxiety. CONCLUSIONS: Personal and social support can relieve healthcare workers' anxiety to some extent during the different stages of an epidemic. Both protective equipment and financial allowance motivate healthcare workers to focus on patient care, although the latter matters more as the epidemic comes under control.

Faraone, A., et al. (2020). "Effectiveness and safety of noninvasive positive pressure ventilation in the treatment of COVID-19-associated acute hypoxemic respiratory failure: a single center, non-ICU setting experience." Intern Emerg Med: 1-8.

The role of noninvasive positive pressure ventilation (NIPPV) in COVID-19 patients with acute hypoxemic respiratory failure (AHRF) is uncertain, as no direct evidence exists to support NIPPV use in such patients. We retrospectively assessed the effectiveness and safety of NIPPV in a cohort of COVID-19 patients consecutively admitted to the COVID-19 general wards of a medium-size Italian hospital, from March 6 to May 7, 2020. Healthcare workers (HCWs) caring for COVID-19 patients were monitored, undergoing nasopharyngeal swab for SARS-CoV-2 in case of onset of COVID-19 symptoms, and periodic SARS-CoV-2 screening serology. Overall, 50 patients (mean age 74.6 years) received NIPPV, of which 22 (44%) were successfully weaned, avoiding endotracheal intubation (ETI) and AHRF-related death. Due to limited life expectancy, 25 (50%) of 50 NIPPV-treated patients received a "do not intubate" (DNI) order. Among these, only 6 (24%) were weaned from NIPPV. Of the remaining 25 NIPPV-treated patients without treatment limitations, 16 (64%) were successfully weaned, 9 (36%) underwent delayed ETI and, of these, 3 (33.3%) died. NIPPV success was predicted by the use of corticosteroids (OR 15.4, CI 1.79-132.57, p 0.013) and the increase in the PaO(2)/FiO(2) ratio measured 24-48 h after NIPPV initiation (OR 1.02, CI 1-1.03, p 0.015), while it was inversely correlated with the presence of a DNI order (OR 0.03, CI 0.001-0.57, p 0.020). During the study period, 2 of 124 (1.6%) HCWs caring for COVID-19 patients were diagnosed with SARS-CoV-2 infection. Apart from patients with limited life expectancy, NIPPV was effective in a substantially high percentage of patients with COVID-19-associated AHRF. The risk of SARS-CoV-2 infection among HCWs was low.

Farrell, S., et al. (2020). "Recommendations for the Care of Pediatric Orthopaedic Patients During the COVID-19 Pandemic." J Am Acad Orthop Surg **28**(11): e477-e486.

The COVID-19 pandemic has necessitated modifications to pediatric orthopaedic practice to protect patients, families, and healthcare workers and to minimize viral transmission. It is critical to balance the benefits of alterations to current practice to reduce the chances of COVID-19 infection, with the potential long-term impact on patients. Early experiences of the pandemic from orthopaedic surgeons in China, Singapore, and Italy have provided the opportunity to take proactive and preventive measures to protect all involved in pediatric orthopaedic care. These guidelines, based on expert opinion and best available evidence, provide a framework for the management of pediatric orthopaedic patients during the COVID-19 pandemic. General principles include limiting procedures to urgent cases such as traumatic injuries and deferring outpatient visits during the acute phase of the pandemic. Nonsurgical methods should be considered where possible. For patients with developmental or chronic orthopaedic conditions, it may be possible to delay treatment for 2 to 4 months without substantial detrimental long-term impact.

Farronato, M., et al. (2020). "A Scoping Review of Respirator Literature and a Survey among Dental Professionals." Int J Environ Res Public Health **17**(16).

Severe acute respiratory syndrome coronavirus 2 (SARS-CoV-2) virus was discovered in China in late 2019 and subsequently triggered a global pandemic. Dentists, like many other health professionals, are at an increased risk of contracting the virus as they work in close proximity to patients, especially when performing aerosol-generating procedures. Thus, in order for dentists to protect themselves and their patients, it is recommended that practitioners wear filtering facepiece 2 (FFP2) respirators. The prolonged use of these FFP2 respirators has been linked to several side effects. The aim of this paper is to assess the perceived experience associated with N95/FFP2 respirators based on the available literature and data collected through an online survey completed by Italian dental professionals. Articles were included up to May 2020 and literature searches were conducted through The National Library of Medicine, Cochrane Central Register of Controlled Trials, and Embase databases. The search terms included COVID-19, respirators, masks, and discomfort. An online survey was administered to 256 Italian dentists. The results from this survey were in agreement with the available literature. The findings concurred that the prolonged use of respirators was associated with headaches (47.5%), severe exertion and discomfort (50.8%), moderate concentration problems (54.3%), moderate breathing difficulties (63.5%), and consequently, an impaired work ability (85.5%). These findings were not influenced by the number of hours spent wearing the respirator. Despite several side effects, FFP2 respirators are fundamental in protecting dentists and their importance was acknowledged.

Felice, C., et al. (2020). "Impact of COVID-19 Outbreak on Healthcare Workers in Italy: Results from a National E-Survey." J Community Health **45**(4): 675-683.

Italy has been the first-hit European country to face the outbreak of coronavirus disease 2019 (COVID-19). Aim of this survey was to assess in depth the impact of the outbreak on healthcare workers (HCW). A 40-item online survey was disseminated via social media inviting Italian HCW, with questions exploring demographics, health status and work environment of respondents. A total of 527 were invited to take part in March 2020, of whom 74% (n = 388) responded to the survey. Of these, 235 (61%) were women. HCW were mostly physicians (74%), from high-prevalence regions (52%). 25% experienced typical symptoms during the last 14 days prior to survey completion, with only 45% of them being tested for COVID-19. Among the tested population, 18 (18%) resulted positive for COVID-19, with 33% being asymptomatic. Only 22% of HCW considered personal protective equipment adequate for quality and quantity. Females and respondents working in high-risk sectors were more likely to rate psychological support as useful (OR, 1.78 [CI 95% 1.14-2.78] P = 0.012, and 2.02 [1.12-3.65] P = 0.020, respectively) and workload as increased (mean increase, 0.38 [0.06-0.69] P = 0.018; and 0.54 [0.16-0.92] P = 0.005, respectively). The insights from this survey may help authorities in countries where COVID-19 epidemic has not yet broken out. Management strategies should be promptly undertaken in order to enhance safety and optimise resource allocation.

Feng, M., et al. (2020). "Geographical and Epidemiological Characteristics of 3,487 Confirmed Cases With COVID-19 Among Healthcare Workers in China." Front Public Health **8**: 586736.

As the first area to report the outbreak, China used to be the front line of the battle against the novel coronavirus SARS-CoV-2. The present descriptive analysis of 3,487 COVID-19-confirmed cases with health workers reported through April 30, 2020 offers important new information to the international community on the epidemic in China. These data showed that Chinese measures including the high-grade protective gear used, mask wearing, and social distancing, are effective in reducing transmission in hospitals.

Ferioli, M., et al. (2020). "Protecting healthcare workers from SARS-CoV-2 infection: practical indications." Eur Respir Rev **29**(155).

The World Health Organization has recently defined the severe acute respiratory syndrome coronavirus 2 (SARS-CoV-2) infection a pandemic. The infection, that may cause a potentially very severe respiratory disease, now called coronavirus disease 2019 (COVID-19), has airborne transmission via droplets. The rate of transmission is quite high, higher than common influenza. Healthcare workers are at high risk of contracting the infection particularly when applying respiratory devices such as oxygen cannulas or noninvasive ventilation. The aim of this article is to provide evidence-based recommendations for the correct use of "respiratory devices" in the COVID-19 emergency and protect healthcare workers from contracting the SARS-CoV-2 infection.

Fernández-Sánchez, V. E., et al. (2020). "[Recommendations on neurophysiological studies during the COVID-19 pandemic]." Rev Neurol **71**(9): 340-350.

At the end of January, the current outbreak of COVID-19 coronavirus disease was declared an important international public health emergency. In Spain, since the government declared the state of alarm on 14 March 2020, doctors responsible for carrying out neurophysiological tests have been performing them without any consensus criterion or clear safety guidelines for doctors, technicians or patients. The following recommendations, based on current knowledge of the disease and therefore liable to change in the future, are proposed when the pandemic appears to have entered a process of decreasing virulence and, with it, the strict containment measures established to date. However, in view of the possibility of a second wave of the pandemic, it seems necessary to establish basic and minimum recommendations to respect the patient's right to appropriate care, similar to that provided prior to the pandemic, and to maintain minimum safety standards for the patients themselves and for the doctors, technicians and health personnel carrying out these tests. These recommendations concern the constitution of a priority based on the reason for consultation, the establishment of calls to check the patient's clinical situation before going to the outpatient department and the rules for carrying out neurophysiological tests, which are generally based on the preservation of hospital circuits, respect for and observation of the known barriers to contagion of this disease, and the use of disposable material. These recommendations are of particular interest, especially given the uncertainty of not knowing the evolution of the SARS-CoV-2 infection in the coming weeks or months.

Ferrari, F. (2020). "COVID-19: Updated Data and its Relation to the Cardiovascular System." Arq Bras Cardiol **114**(5): 823-826.

In December 2019, a new human coronavirus, called severe acute respiratory syndrome coronavirus 2 (SARS-CoV-2) or coronavirus disease 2019 (COVID-19) by the World Health Organization, emerged in the city of Wuhan, China. Spreading globally, it is now considered pandemic, with approximately 3 million cases worldwide at the end of April. Its symptoms include fever, cough, and headache, but the main one is shortness of breath. In turn, it is believed that there is a relationship between COVID-19 and damage to the heart muscle, and hypertensive and diabetic patients, for example, seem to have worse prognosis. Therefore, COVID-19 may worsen in individuals with underlying adverse conditions, and a not negligible number of patients hospitalized with this virus had cardiovascular or cerebrovascular diseases. Systemic inflammatory response and immune system disorders during disease progression may be behind this association. In addition, the virus uses angiotensin-converting enzyme (ACE) receptors, more precisely ACE2, to penetrate the cell; therefore, the use of ACE inhibitor drugs and angiotensin receptor blockers could cause an increase in these receptors, thus facilitating the entry of the virus into the cell. There is, however, no scientific evidence to support the interruption of these drugs. Since they are fundamental for certain chronic diseases, the risk and benefit of their withdrawal in this scenario should be carefully weighed. Finally, cardiologists and health professionals should be aware of the risks of infection and protect themselves as much as possible, sleeping properly and avoiding long working hours.

Figueiredo-Campos, P., et al. (2020). "Seroprevalence of anti-SARS-CoV-2 antibodies in COVID-19 patients and healthy volunteers up to 6 months post disease onset." Eur J Immunol **50**(12): 2025-2040.

SARS-CoV-2 has emerged as a human pathogen, causing clinical signs, from fever to pneumonia-COVID-19-but may remain mild or asymptomatic. To understand the continuing spread of the virus, to detect those who are and were infected, and to follow the immune response longitudinally, reliable and robust assays for SARS-CoV-2 detection and immunological monitoring are needed. We quantified IgM, IgG, and IgA antibodies recognizing the SARS-CoV-2 receptor-binding domain (RBD) or the Spike (S) protein over a period of 6 months following COVID-19 onset. We report the detailed setup to monitor the humoral immune response from over 300 COVID-19 hospital patients and healthcare workers, 2500 University staff, and 198 post-COVID-19 volunteers. Anti-SARS-CoV-2 antibody responses follow a classic pattern with a rapid increase within the first three weeks after symptoms. Although titres reduce subsequently, the ability to detect anti-SARS-CoV-2 IgG antibodies remained robust with confirmed neutralization activity for up to 6 months in a large proportion of previously virus-positive screened subjects. Our work provides detailed information for the assays used, facilitating further and longitudinal analysis of protective immunity to SARS-CoV-2. Importantly, it highlights a continued level of circulating neutralising antibodies in most people with confirmed SARS-CoV-2.

Finkenzeller, T., et al. (2020). "[SARS-CoV-2 antibodies in ICU and clinic staff : From Germany's region with the highest infection rate]." Med Klin Intensivmed Notfmed **115**(Suppl 3): 139-145.

BACKGROUND: Healthcare workers are a high-risk population for SARS-CoV‑2 infection. For capacity planning of healthcare providers and to optimize protection of healthcare workers (HCW) in SARS-CoV‑2 pandemics, it is essential to know the risk of infection and potential immunity status of staff dealing with COVID-19 patients. MATERIALS AND METHODS: We examined seropravalence of SARS-CoV‑2 IgM/IgG antibodies (AB) in HCW of a region with the highest rate of infection (1570/100,000) during COVID-19 pandemic in Germany, 4 months after its start. Employees of a nonmedical company (MU) served as control group. Demographic data, medical history and working situation were recorded. RESULTS: A total of 1838 HCW and 986 MU volunteered to participate. Seroprevalence for SARS-CoV‑2 in HCW was 15.1% and 3.7% in MU. Among HCWs, nurses had a seropositivity of 20.0%, ICU personnel 20.3%, housekeepers 19.3%, physicians 12.0%, medical services (e.g., radiology, physiotherapy) 11.3%, administration 7.1% and technical services 6%. Symptoms typical for COVID-19 were not experienced by 10% of seropositive HCWs. CONCLUSION: Seroprevalence of SARS-CoV‑2 antibodies in HCW of a region heavily affected by COVID-19 is with 15.1% significantly higher than in a control group of nonmedical staff with 3.7%. Infection rate in HCW was higher in staff with close contact to infected patients. Seropositivity in ICU personnel is higher than in other clinical professions. The occupational risk for housekeepers seems to be underestimated.

Fino, E., et al. (2021). "Harnessing distress to boost growth in frontline healthcare workers during COVID-19 pandemic: the protective role of resilience, emotion regulation and social support." Psychol Med: 1-3.

Firew, T., et al. (2020). "Protecting the front line: a cross-sectional survey analysis of the occupational factors contributing to healthcare workers' infection and psychological distress during the COVID-19 pandemic in the USA." BMJ Open **10**(10): e042752.

OBJECTIVE: The COVID-19 pandemic has been associated with significant occupational stressors and challenges for front-line healthcare workers (HCWs), including COVID-19 exposure risk. Our study sought to assess factors contributing to HCW infection and psychological distress during the COVID-19 pandemic in the USA. DESIGN: We conducted a cross sectional survey of HCWs (physicians, nurses, emergency medical technicians (EMTs), non-clinical staff) during May 2020. Participants completed a 42-item survey assessing disease transmission risk (clinical role, work environment, availability of personal protective equipment) and mental health (anxiety, depression and burn-out). SETTING: The questionnaire was disseminated over various social media platforms. 3083 respondents from 48 states, the District of Columbia and US territories accessed the survey. PARTICIPANTS: Using a convenience sample of HCWs who worked during the pandemic, 3083 respondents accessed the survey and 2040 participants completed at least 80% of the survey. PRIMARY OUTCOME: Prevalence of self-reported COVID-19 infection, in addition to burn-out, depression and anxiety symptoms. RESULTS: Participants were largely from the Northeast and Southern USA, with attending physicians (31.12%), nurses (26.80%), EMTs (13.04%) with emergency medicine department (38.30%) being the most common department and specialty represented. Twenty-nine per cent of respondents met the criteria for being a probable case due to reported COVID-19 symptoms or a positive test. HCWs in the emergency department (31.64%) were more likely to contract COVID-19 compared with HCWs in the ICU (23.17%) and inpatient settings (25.53%). HCWs that contracted COVID-19 also reported higher levels of depressive symptoms (mean diff.=0.31; 95% CI 0.16 to 0.47), anxiety symptoms (mean diff.=0.34; 95% CI 0.17 to 0.52) and burn-out (mean diff.=0.54; 95% CI 0.36 to 0.71). CONCLUSION: HCWs have experienced significant physical and psychological risk while working during the COVID-19 pandemic. These findings highlight the urgent need for increased support for provider physical and mental health well-being.

Fletcher, J. J., et al. (2021). "Health Care Acquired COVID-19 is Less Symptomatic than Community Acquired Disease Among Healthcare Workers." Infect Control Hosp Epidemiol: 1-28.

OBJECTIVE: We hypothesized healthcare workers (HCW) with high-risk exposures outside the healthcare system would have less asymptomatic coronavirus 2019 (COVID-19) disease and more symptoms than those without such exposures. DESIGN: A longitudinal point prevalence study during August 17- September 4 2020 and December 2nd - 23rd. SETTING: Community based teaching health system. PARTICIPANTS: All HCS were invited to participate. Among HCW who acquired COVID-19, logistic regression models were used to evaluate the adjusted odds of asymptomatic disease using high-risk exposure outside the healthcare system as the explanatory variable. The number of symptoms between exposure groups was evaluated with the Wilcoxon rank-sum test. The risk of seropositivity among all HCS by work exposure was evaluated during both periods. INTERVENTIONS: Survey and serological testing. RESULT: Seroprevalence increased from 1.9% (95% CI 1.2% - 2.6%) to 13.7% (95% CI 11.9% - 15.5%) during the study. Only during Period 2 did HCW with the highest work exposure (versus low exposure) have an increased risk of seropositivity (RD 7% [95% CI 1% -13%]). Participants who had a high-risk exposure outside of work (compared to those without) had a decreased probability of asymptomatic disease (OR 0.38 [95% CI 0.16 - 0.86]) and demonstrated more symptoms (median of 3 [IQR 5] vs 1 [IQR 2]; P = 0.001). CONCLUSIONS: Health care acquired COVID-19 increases the probability of asymptomatic or mild COVID-19 disease compared to community acquired disease. This suggests infection prevention strategies (including masks and eye protection) may be mitigating inoculum and supports the variolation theory in COVID-19.

Flynn, A. and C. C. Dickey (2021). "In Their Own Words: What Do Healthcare Workers Want from Their Organization during the COVID-19 Pandemic?" Healthc Q **24**(1): 44-49.

Pandemics are associated with heightened distress among healthcare workers (HCWs). We report qualitative findings from a two-stage survey administered to HCWs at a large acute care hospital in Ontario during the COVID-19 pandemic to identify their concerns and wellness needs. Responses reflected HCWs' desires to be heard, protected, prepared, supported and cared for by the organization. HCWs' concerns were diverse and dynamic, reflecting the specific circumstances of their work and personal lives as well as the shifting landscape of the pandemic. We discuss implications for organizations seeking to promote and protect HCWs' psychological well-being and resilience during pandemics.

Foley, S. J., et al. (2020). "Early experiences of radiographers in Ireland during the COVID-19 crisis." Insights Imaging **11**(1): 104.

BACKGROUND: Imaging is crucial for assessing the severity and progression of COVID-19. Radiographers are amongst the first-line health professionals that may be exposed to infected persons. This work describes the early experience of radiographers in Ireland to the impact of COVID-19 using two electronic surveys distributed 6 weeks apart. Results were analysed using descriptive statistics and thematic analysis. RESULTS: A total of 370 responded to the first survey and 276 the second, with all six Irish health regions represented. Three quarters of radiographers (77%) reported having adequate personal protective equipment (PPE) available to them. However, almost half of the radiographers were inadvertently exposed to COVID-19-positive patients without appropriate PPE, largely attributed to poor communication and testing. Anxiety levels while initially high, reduced substantially 6 weeks into the crisis period. However, obvious distress was noted amongst some respondents. Forty percent of radiographers reported burnout symptoms due to the COVID-19 crisis and 30% reported considering changing jobs or retiring since the COVID-19 outbreak. CONCLUSION: Clear communication regarding changing protocols and importantly patients' infectious status are essential to safeguard healthcare workers and to minimise unnecessary anxiety and distress. Attention is required to staff mental health including the identification of burnout symptoms to prevent long-term negative consequences of the pandemic on radiography services.

Fong, S., et al. (2021). "Impact of aerosol box on intubation during COVID-19: a simulation study of normal and difficult airways." Can J Anaesth **68**(4): 496-504.

PURPOSE: Patients with coronavirus disease (COVID-19) are at risk of requiring mechanical ventilation, and concerns of protecting healthcare workers during aerosol-generating medical procedures has led to the design of the aerosol box. METHODS: We conducted a randomized crossover mannequin-based simulation study to compare airway management with and without the aerosol box. Thirty-five anesthesiology participants and three critical care participants with more than 50 intubations with videolaryngoscopes were recruited. There were four airway simulations with and without the aerosol box (normal, pharyngeal swelling, cervical spine rigidity, and tongue edema). Each participant intubated the mannequin in eight consecutive simulations. The primary outcome of the study was time to intubation. Secondary outcomes included intubation attempts, optimization maneuvers, and personal protective equipment breaches. RESULTS: Mean (standard deviation [SD]) time to intubation overall with the box was 30.9 (23.0) sec, while the time to intubation without the box was 25.1 (12.2) sec (mean difference, 5.8; 95% confidence interval [CI], -2.9 to 14.5). For the normal airway scenario, the mean (SD) time to intubation was 18.6 (3.5) sec for no box and 20.4 (3.3) sec for box (mean difference, 1.8; 95% CI, 0.2 to 3.4). During difficult airway scenarios only, the time to intubation was 34.4 (25.6) sec with the aerosol box and 27.3 (13.2) sec without the aerosol box (mean difference, 7.1; 95% CI, -2.5 to 16.7). There were more intubation attempts, personal protective equipment breaches, and optimization maneuvers during use of the aerosol box. CONCLUSIONS: In this mannequin-based simulation study, the use of the aerosol box increased the time to intubation in some contexts but not others. Further studies in a clinical setting should be conducted to make appropriate modifications to the aerosol box to fully elicit its efficacy and safety prior to implementation in airway guidelines for managing patients with COVID-19.

Foo, D. H. P., et al. (2021). "COVID-19 testing strategy in response to infection among healthcare workers in a large non-COVID-designated hospital." Hosp Pract (1995) **49**(2): 110-118.

Sarawak General Hospital, which is the only public access tertiary referral center in Sarawak State for all clinical specialties, was designated a hybrid hospital to treat both COVID-19 and non-COVID-19 patients. During the initial surge of patients admitted with COVID-19, there was also a corresponding increase in health-care workers (HCWs) detected with COVID-19 infection. The latter being isolated, and the large number of staff members that had come into contact with COVID-19 being quarantined from work, placed further strain on the health-care services. The staff mass screening strategy was a policy decision made by the hospital in response to infection among HCWs, and it aimed to reduce in-hospital transmission (particularly among asymptomatic staff), mitigate workforce depletion due to quarantining, and protect the health-care workforce. In this study, we assessed the detection rate of COVID-19 infection from staff mass testing over a five-week period, and described our experience of adopting this surveillance screening strategy alongside ongoing contact tracing and symptomatic screening strategies. Although it was thought that such periodic staff surveillance might be helpful in protecting the health-care workforce within a short period, the long-term implications, especially in settings with limited resources, is significant and therefore explored in this paper. Our findings might provide an evidence-based reference for the future planning of an optimal strategy with the least compromise in care for a larger proportion of non-COVID-19 patients amid efforts against COVID-19 in a large non-COVID-designated hospital with hybrid status.

Ford, T., et al. (2020). "Optimization of resources and modifications in acute ischemic stroke care in response to the global COVID-19 pandemic." J Stroke Cerebrovasc Dis **29**(8): 104980.

BACKGROUND: The COVID-19 pandemic has presented unprecedented challenges to healthcare organizations worldwide. A steadily rising number of patients requiring intensive care, a large proportion from racial and ethnic minorities, demands creative solutions to provide high-quality care while ensuring healthcare worker safety in the face of limited resources. Boston Medical Center has been particularly affected due to the underserved patient population we care for and the increased risk of ischemic stroke in patients with COVID-19 infection. METHODS: We present protocol modifications developed to manage patients with acute ischemic stroke in a safe and effective manner while prioritizing judicious use of personal protective equipment and intensive care unit resources. CONCLUSION: We feel this information will benefit other organizations facing similar obstacles in caring for the most vulnerable patient populations during this ongoing public health crisis.

Forrester, J. D., et al. (2020). "Precautions for Operating Room Team Members During the COVID-19 Pandemic." J Am Coll Surg **230**(6): 1098-1101.

BACKGROUND: The novel coronavirus SARS-CoV-2 (COVID-19) can infect healthcare workers. We developed an institutional algorithm to protect operating room team members during the COVID-19 pandemic and rationally conserve personal protective equipment (PPE). STUDY DESIGN: An interventional platform (operating room, interventional suite, and endoscopy) PPE taskforce was convened by the hospital and medical school leadership and tasked with developing a common algorithm for PPE use, to be used throughout the interventional platform. In conjunction with our infectious disease experts, we developed our guidelines based on potential patterns of spread, risk of exposure, and conservation of PPE. RESULTS: A decision tree algorithm describing our institutional guidelines for precautions for operating room team members was created. This algorithm is based on urgency of operation, anticipated viral burden at the surgical site, opportunity for a procedure to aerosolize virus, and likelihood a patient could be infected based on symptoms and testing. CONCLUSIONS: Despite COVID-19 being a new threat, we have shown that by developing an easy-to-follow decision tree algorithm for the interventional platform teams, we can ensure optimal health care worker safety.

Fouladi Dehaghi, B., et al. (2020). "Face masks vs. COVID-19: a systematic review." Invest Educ Enferm **38**(2).

The coronavirus disease (COVID-19) spread rapidly around the world. Two types of approaches have been applied to use of face masks as a tool to prevent the spread this disease in society. The aim of the systematic review was to assess the effectiveness of face masks against the novel coronavirus. A literature search was performed using different databases until April 30, 2020. Search terms were ‘facemasks’, ‘novel coronavirus’, and ‘healthcare workers’. Five studies were included in the systematic review. A study stated that no difference between surgical and cotton masks. Also, two studies have emphasized the use of surgical masks or N95 respirators by medical staff, and two other studies emphasized the use of any type of face mask by general public. More studies in controlled contexts and studies of infections in healthcare and community places are needed for better definition of the effectiveness of face masks in preventing coronavirus.

Publisher: Abstract available from the publisher.

spa

por

Fouogue, J. T., et al. (2020). "Poor knowledge of COVID-19 and unfavourable perception of the response to the pandemic by healthcare workers at the Bafoussam Regional Hospital (West Region-Cameroon)." Pan Afr Med J **37**(Suppl 1): 19.

INTRODUCTION: WHO warned against a dramatic impact of COVID-19 in Africa unless adequate response strategies are implemented. Whatever the strategy, the role of health staff is pivotal. Objective: Assess knowledge and perception of the response to COVID-19 among health staff. METHODS: we used a convenience non probabilistic sample to conduct a survey with a self-administered questionnaire from April 14 - 29, 2020 at the Bafoussam Regional Hospital (BRH). All the staff was invited to participate. Analyses were done with Microsoft Excel 2010 and Epi-Info version 7.1.5.2. RESULTS: response rate was 76.1% (464/610). Mean age (SD) was 35.0 (8.9) years. Sex ratio (M/F) was 101/356. Nursing/midwifery staff (56.8%) and in-patients units (49.94%) were predominant. Origin and transmission of SARS-CoV-2 were poorly known while knowledge of clinical signs and the role of laboratory tests were good. For 53.2% of respondents all therapeutic regimens are supportive and only 31.6% trusted state-recommended drugs. For 169 of respondents (36.9%), herbal remedies prevent/cure COVID-19. Seventy percent (70%) felt they were not knowledgeable enough to handle COVID-19 cases. Eighty-five point six percent (85.6%) thought the BRH had insufficient resources to fight COVID-19 and 55.6% were dissatisfied with its response (weaknesses: medicines/technologies (74.5%), service delivery (28.1%), staff (10.9%)). Sixty-eight percent (68%) reported insufficient protection on duty. Seventy-six point five percent (76.5%) reported a drop in non-COVID-19 services. Eighty-five point five (85.5%) percent said they complied with community preventive measures. For 44% of respondents, regulations on COVID-19 corpses should be made more culture-sensitive. Fifty one point two percent (51.2%) of respondents were against vaccine trial in their community. CONCLUSION: knowledge was poor and perception of the response to COVID-19 was unfavourable.

Francisco, C. P., et al. (2021). "Moving Forward: Gradual Return of Gastroenterology Practice during the COVID-19 Pandemic." Dig Dis **39**(2): 140-149.

BACKGROUND: The COVID-19 pandemic has caused disruption of routine gastroenterology practice, which has resulted in the suspension of elective endoscopic procedures and outpatient consults. For the past months, the strategy was to mitigate infection risk for the healthcare team while still providing essential service to patients. Prolonged suspension of the outpatient clinics and endoscopy practice, however, is deemed unsustainable and could even be detrimental. It can compromise patient care and result in poor outcomes; hence, a well-crafted plan is needed for the gradual resumption of clinic operations and endoscopic procedures. SUMMARY: As the world begins to transition to the "new normal," there are new health and safety issues to consider. Adaptive measures like telemedicine and electronic health records should be utilized to facilitate patient care while minimizing exposure. Careful patient screening, adequate supply of personal protective equipment, effective infection-control policies, as well as appropriate administrative modifications are needed for a safe return of gastroenterology practice. Key Messages: Ensuring the safety of patients, caregivers, and healthcare workers should remain as top priority. To help ease the transition as we move forward from this pandemic, we present a review of recommendations to guide gastroenterologists and endoscopy unit administrators in the gradual return to gastroenterology practice.

François, T., et al. (2020). "Simulation-Based Rapid Development and Implementation of a Novel Barrier Enclosure for Use in COVID-19 Patients: The SplashGuard CG." Crit Care Res Pract **2020**: 3842506.

BACKGROUND: The current COVID-19 pandemic has resulted in over 54,800,000 SARS-CoV-2 infections worldwide with a mortality rate of around 2.5%. As observed in other airborne viral infections such as influenza and SARS-CoV-1, healthcare workers are at high risk for infection when performing aerosol-generating medical procedures (AGMP). Additionally, the threats of a global shortage of standard personal protective equipment (PPE) prompted many healthcare workers to explore alternative protective enclosures, such as the "aerosol box" invented by a Taiwanese anesthetist. Our study includes the design process of a protective barrier enclosure and its subsequent clinical implementation in the management of critically ill adults and children infected with SARS-CoV-2. METHODS AND RESULTS: The barrier enclosure was designed for use in our tertiary care facility and named "SplashGuard CG" (CG for Care Givers). The device has been adapted using a multi- and interdisciplinary approach, with collaboration between physicians, respiratory therapists, nurses, and biomechanical engineers. Computer-aided design and simulation sessions throughout the entire process facilitated the rapid and safe implementation of the SplashGuard CG in different settings (intensive care unit, emergency department, and the operating room) during AGMPs such as bag-valve-mask ventilation, nasopharyngeal suctioning, intubation and extubation, and noninvasive ventilation. Indications for use and anticipatory precautions were communicated to all healthcare workers using the SplashGuard CG. The entire process was completed within one month. CONCLUSION: The rapid design, development, and clinical implementation of a new barrier enclosure, the "SplashGuard CG," was feasible in this time of crisis thanks to close collaboration between medical and engineering teams and the use of recurring simulation sessions to test and improve the initial prototypes. Following this accelerated process, it is necessary to maintain team skills, monitor any undesirable effects, and evaluate and continuously improve this new device.

Franklin, P. and A. Gkiouleka (2021). "A Scoping Review of Psychosocial Risks to Health Workers during the Covid-19 Pandemic." Int J Environ Res Public Health **18**(5).

The Covid-19 pandemic has exposed health workers to a diverse set of hazards impacting their physical, psychological and social wellbeing. This review aims to provide an overview of the categories of the psychosocial risk factors and hazards affecting HCWs during the Covid-19 pandemic and the recommendations for prevention. We used the scoping review methodology to collate categories of psychosocial risks, the related health outcomes, interventions, and data gaps. The review was conducted on global peer-reviewed academic and authoritative grey literature, published between 1. January-26. October 2020; in total, 220 articles were included into the review and the subsequent analysis. Analysis of the extracted data found PSRs related to four sources: personal protective equipment (PPE), job content, work organisation, and social context. is. Women health workers and nurses reported worst health outcomes. Majority of the research to date concerns health workers in secondary care, while data on psychosocial risks at primary and community-based settings are scarce. However, the emerging research implies that the pandemic creates psychosocial risks also to non-clinical health workers. The intervention and mitigation measures address individual and organisational levels. Preventative and mitigating measures for social and societal risks-such as staff shortages, intersecting inequalities, and financial stressors require further research.

Franza, F., et al. (2020). "The Role of Fatigue of Compassion, Burnout and Hopelessness in Healthcare: Experience in the Time of COVID-19 Outbreak." Psychiatr Danub **32**(Suppl 1): 10-14.

BACKGROUND: In the times of serious health alarm, as it is happening in the COVID-19 pandemic, burden of healthcare is likely to explode. The current pandemic is having a profound effect on all aspects of society, including mental health and physical health. In a previous study we showed interaction between compassion fatigue, burnout and workload. METHODS: In our study, we have evaluated stress levels in 102 healthcare workers recruited in different rehabilitation departments (psychiatric and multidisciplinary). In particular, we evaluated the fatigue of compassion, vicarious trauma, burnout and hope (hopelessness) in the first two months of lookdown due to the COVID-19. RESULTS: The results obtained after the administration of the following evaluation scales: sCFs, CBI, Pro QOL, HBS, showed an overall increase in the scores in all professional figures. Significant compassion fatigue and burnout percentage is present in several groups. The highest levels of hopelessness in some professional figures, while higher educational levels can protect workers from the risk of developing high levels of work stress. CONCLUSIONS: The data obtained with this study are similar to those of our previous study, although they may indicate that during the period of the coronavirus pandemic the scores of the several scales used are higher.

Franzini, T. A. P., et al. (2020). "Approach to Endoscopic Procedures: A Routine Protocol from a Quaternary University Referral Center Exclusively for Coronavirus Disease 2019 Patients." Clinics (Sao Paulo) **75**: e1989.

OBJECTIVES: The present coronavirus disease (COVID-19) pandemic has ushered in an unprecedented era of quality control that has necessitated advanced safety precautions and the need to ensure the adequate protection of healthcare professionals (HCPs). Endoscopy units, endoscopists, and other HCP may be at a significant risk for transmission of the virus. Given the immense burden on the healthcare system and surge in the number of patients with COVID-19, well-designed protocols and recommendations are needed. We aimed to systematically characterize our approach to endoscopic procedures in a quaternary university hospital setting and provide summary protocol recommendations. METHOD: This descriptive study details a COVID-19-specific protocol designed to minimize infection risks to patients and healthcare workers in the endoscopy unit. RESULTS: Our institution, located in São Paulo, Brazil, includes a 900-bed hospital, with a 200-bed-specific intensive care unit exclusively designed for patients with moderate and severe COVID-19. We highlighted recommendations for infection prevention and control during endoscopic procedures, including appropriate triage and screening, outpatient management and procedural recommendations, role and usage of personal protective equipment (PPE), and role and procedural logistics involving COVID-19-positive patients. We also detailed hospital protocols for reprocessing endoscopes and cleaning rooms and also provided recommendations to minimize severe acute respiratory syndrome coronavirus 2 transmission. CONCLUSION: This COVID-19-specific administrative and clinical protocol can be replicated or adapted in multiple institutions and endoscopy units worldwide. Furthermore, the recommendations and summary protocol may improve patient and HCP safety in these trying times.

Fregatti, P., et al. (2020). "Breast Cancer Surgery During the COVID-19 Pandemic: An Observational Clinical Study of the Breast Surgery Clinic at Ospedale Policlinico San Martino - Genoa, Italy." In Vivo **34**(3 Suppl): 1667-1673.

BACKGROUND: COVID-19 pandemic required a marked re-allocation of healthcare resources, including at Breast Units. A patient-tailored program was developed to assess its efficacy regarding prevention of COVID-19 infection among patients with breast cancer undergoing surgery and healthcare workers (HCWs). PATIENTS AND METHODS: From March 9th to April 9th 2020, 91 patients were selected for elective surgery by means of: i) Pre-hospital screening aimed at avoiding hospitalization of symptomatic or suspicious COVID-19 patients, and ii) prioritisation of surgical procedure according to specific disease features. RESULTS: Eighty-five patients (93.4%) were fit for surgery, while five patients (5.5%) were temporarily excluded through 'telephone triage'; another two patients were excluded at in-hospital triage. A total of 71 out of 85 patients (83.5%) were diagnosed with invasive cancer, most of whom were undergoing breast-conserving surgery (61 out of 85 patients, 71.8%). The mean in-hospital stay was 2.2 days (SD=0.7 days). After hospital discharge, no patient needed re-admission due to post-operative complications; moreover, no COVID-19 infection among patients or HCWs was detected. CONCLUSION: Safe breast cancer surgery was accomplished for both patients and HCWs by means of a careful preoperative selection of patients and in-hospital preventative measures. This screening program can be transferred to high-volume Breast Units and it may be useful in implementing European Community recommendations for prevention of COVID-19 infection.

Freitas, B., et al. (2020). "Prevention and control measures for neonatal COVID-19 infection: a scoping review." Rev Bras Enferm **73 Suppl 2**: e20200467.

OBJECTIVE: to identify with the literature the measures to prevent and control neonatal infection by COVID-19. METHODS: a scope review carried out by searching for studies in databases and institutional health websites. The final sample was 25 articles. RESULTS: among the main measures are the use of masks by suspected or infected people in contact with healthy newborns, hand hygiene before and after each care and feeding as well as the tools used for milking. It is indispensable to use personal protective equipment by health professionals in neonatology services to maintain a private room for infected newborns or to use physical barriers. Early diagnosis and timely case management is essential to reduce virus transmissibility. CONCLUSIONS: the research contributed to elucidate health and nursing actions in preventing and controlling neonatal infection by COVID-19.

Friese, C. R., et al. (2020). "Respiratory Protection Considerations for Healthcare Workers During the COVID-19 Pandemic." Health Secur **18**(3): 237-240.

The COVID-19 pandemic has resulted in a surge of patients that exceeds available human and physical resources in many settings, triggering the implementation of crisis standards of care. High-quality respiratory protection is essential to reduce exposure among healthcare workers, yet dire shortages of personal protective equipment in the United States threaten the health and safety of this essential workforce. In the context of rapidly changing conditions and incomplete data, this article outlines 3 important strategies to improve healthcare workers' respiratory protection. At a minimum, healthcare workers delivering care to patients with confirmed or suspected COVID-19 should wear N95 respirators and full-face shields. Several mechanisms exist to boost and protect the supply of N95 respirators, including rigorous decontamination protocols, invoking the Defense Production Act, expanded use of reusable elastomeric respirators, and repurposing industrial N95 respirators. Finally, homemade facial coverings do not protect healthcare workers and should be avoided. These strategies, coupled with longer-term strategies of investments in protective equipment research, infrastructure, and data systems, provide a framework to protect healthcare workers immediately and enhance preparedness efforts for future pandemics.

Froum, S. H. and S. J. Froum (2020). "Incidence of COVID-19 Virus Transmission in Three Dental Offices: A 6-Month Retrospective Study." Int J Periodontics Restorative Dent **40**(6): 853-859.

The World Health Organization (WHO) declared COVID-19 a pandemic on March 11, 2020. SARS CoV-2, the virus that causes COVID-19, has shown the ability to become aerosolized with a potential airborne route of transmission. Dentists and dental hygienists are listed as two of the occupations in a nonhospital setting with the greatest risk of contracting the SARS-CoV-2 virus, as routine dental procedures involve aerosol generation. In a statement on interim guidance, WHO recommended that all routine dental procedures be delayed until COVID-19 transmission rates decrease from community transmission to cluster cases and until the risk of transmission in a dental office can be studied and evaluated. This prospective study involves 2,810 patients treated over a 6-month period (March 15 to September 15, 2020) in three different dental offices by two dentists and three hygienists during and shortly after the height of the pandemic in New York. By utilizing screening questionnaires, performing enhanced infection control, and having appropriate personal protective equipment, these dental offices were able to record no transmission of COVID-19 to the dental healthcare workers or patients during the study. In addition, 69% of the patients treated in these dental offices were recorded as having one or more high-risk comorbidities related to COVID-19 severity.

Fukuti, P., et al. (2020). "How Institutions Can Protect the Mental Health and Psychosocial Well-Being of Their Healthcare Workers in the Current COVID-19 Pandemic." Clinics (Sao Paulo) **75**: e1963.

Fülesdi, B., et al. (2020). "[Challenges of intensive care and anesthesiology related to COVID-19 pandemic. Practical considerations]." Orv Hetil **161**(17): 652-659.

Due to the coronavirus epidemic, healthcare systems face growing challenges all around the world nowadays. These challenges are the most critical in the field of intensive treatment and anesthesiology. One of the most important prerequisites of effective critical care treatment is preserving the involved healthcare workers from the infection, by providing them with detailed practical advices on the preventive measures and treatment strategies. The aim of the present review is to summarize the most important related knowledge available from previous experiences. Orv Hetil. 2020; 161(17): 652–659.

Fwoloshi, S., et al. (2021). "Prevalence of Severe Acute Respiratory Syndrome Coronavirus 2 (SARS-CoV-2) among Health Care Workers-Zambia, July 2020." Clin Infect Dis.

INTRODUCTION: Healthcare workers (HCWs) in Zambia have become infected with SARS-CoV-2, the virus that causes coronavirus disease (COVID-19). However, SARS-CoV-2 prevalence among HCWs is not known in Zambia. METHODS: We conducted a cross-sectional SARS-CoV-2 prevalence survey among Zambian HCWs in twenty health facilities in six districts in July 2020. Participants were tested for SARS-CoV-2 infection using polymerase chain reaction (PCR) and for SARS-CoV-2 antibodies using enzyme-linked immunosorbent assay (ELISA). Prevalence estimates and 95% confidence intervals (CIs), adjusted for health facility clustering, were calculated for each test separately and a combined measure for those who had PCR and ELISA performed. RESULTS: In total, 660 HCWs participated in the study, with 450 (68.2%) providing nasopharyngeal swab for PCR and 575 (87.1%) providing a blood specimen for ELISA. Sixty-six percent of participants were females and the median age was 31.5 years (interquartile range 26.2-39.8 years). The overall prevalence of the combined measure was 9.3% (95% CI 3.8%-14.7%). PCR-positive prevalence of SARS-CoV-2 was 6.6% (95% CI 2.0%-11.1%) and ELISA-positive prevalence was 2.2% (95% CI 0.5%-3.9%). CONCLUSIONS: SARS-CoV-2 prevalence among HCWs was similar to a population-based estimate (10.6%) during a period of community transmission in Zambia. Public health measures such as establishing COVID-19 treatment centers before the first cases, screening for COVID-19 symptoms among patients accessing health facilities, infection prevention and control trainings, and targeted distribution of personal protective equipment based on exposure risk might have prevented increased SARS-CoV-2 transmission among Zambian HCWs.

Galanis, P., et al. (2021). "Seroprevalence of SARS-CoV-2 antibodies and associated factors in healthcare workers: a systematic review and meta-analysis." J Hosp Infect **108**: 120-134.

BACKGROUND: Healthcare workers (HCWs) represent a high-risk population for infection with severe acute respiratory syndrome coronavirus-2 (SARS-CoV-2). AIM: To determine the seroprevalence of SARS-CoV-2 antibodies among HCWs, and identify the factors associated with this seroprevalence. METHODS: The Preferred Reporting Items for Systematic Reviews and Meta-Analysis guidelines were applied for this systematic review and meta-analysis. Databases including PubMed/MEDLINE and preprint services (medRχiv and bioRχiv) were searched from inception to 24(th) August 2020. FINDINGS: Forty-nine studies including 127,480 HCWs met the inclusion criteria. The estimated overall seroprevalence of SARS-CoV-2 antibodies among HCWs was 8.7% (95% confidence interval 6.7-10.9%). Seroprevalence was higher in studies conducted in North America (12.7%) compared with those conducted in Europe (8.5%), Africa (8.2) and Asia (4%). Meta-regression showed that increased sensitivity of antibody tests was associated with increased seroprevalence. The following factors were associated with seropositivity: male gender; Black, Asian and Hispanic HCWs; work in a coronavirus disease 2019 (COVID-19) unit; patient-related work; front-line HCWs; healthcare assistants; shortage of personal protective equipment; self-reported belief of previous SARS-CoV-2 infection; previous positive polymerase chain reaction test; and household contact with suspected or confirmed cases of COVID-19. CONCLUSION: The seroprevalence of SARS-CoV-2 antibodies among HCWs is high. Excellent adherence to infection prevention and control measures; sufficient and adequate personal protective equipment; and early recognition, identification and isolation of HCWs infected with SARS-CoV-2 are imperative to decrease the risk of SARS-CoV-2 infection.

Gallagher, J. E., et al. (2020). "Relevance and paucity of evidence: a dental perspective on personal protective equipment during the COVID-19 pandemic." Br Dent J **229**(2): 121-124.

The global COVID-19 pandemic, caused by the SARS-CoV-2 virus, has highlighted the importance of personal protective equipment (PPE) for health and social care personnel. This is a really important issue for dentistry, where we place great emphasis on infection control and universal precautions, given the nature of care provided. Cochrane have recently updated their review of PPE for preventing highly infectious diseases due to exposure to contaminated body fluids in healthcare staff. It examined evidence on which type of full body PPE and which method of donning (putting on) or doffing (removing) are most effective, while having the least risk of contamination or infection for healthcare workers, as well as which training methods increase compliance with PPE protocols. The objective of this paper is to raise awareness of the above review of PPE, its findings and their relevance to dentistry as outlined in the Cochrane Oral Health website.The available evidence comes from healthcare generally, mostly involving simulation exercises, and is of low or very low certainty. None of the evidence specifically comes from dentistry.The findings in relation to the nature of PPE, methods of donning and doffing, and the importance of training are all of practical relevance to dentistry. Research is critically important to provide evidence for future decision making in support of patients and staff.

Galloro, G., et al. (2020). "Safety in digestive endoscopy procedures in the covid era recommendations in progres of the italian society of digestive endoscopy." Dig Liver Dis **52**(8): 800-807.

The new corona virus disease has started in Wuhan - China at the end of 2019 and quickly spread with a pandemic trend across the rest of the world. The scientific community is making an extraordinary effort to study and control the situation, but the results are just partial. Based on the most recent scientific literature and strong statements by the most prestigious international health institutions, the Italian Society of Digestive Endoscopy has drawn up some recommendations about the use of personal protective equipment, the correct way of dressing and undressing of endoscopists and nurses, before and after digestive endoscopy procedures. In addition, some other important indications are given to reduce the risk of contamination of healthcare providers during endoscopic activities, in the setting of a pandemic. Nevertheless, because of the very quick evolution of our knowledge on this issue, these recommendations must be considered as evolving, because they could change in a short time.

Gandhi, J. A., et al. (2020). "Surgical Mask-the Saviour: from its Invention to the COVID-19 Era." Indian J Surg: 1-2.

The earliest available evidence attributes the discovery of droplets as a mode of transmission of disease to Carl Flügge, a German bacteriologist, a contemporary of Emil Kocher, in 1897. This finding was instrumental in the development of the gauze mask introduced by Johann von Mikulicz Radecki in the same year. A surgical mask has become an indispensable tool in the armamentarium to fight the COVID 19 pandemic. Surgical masks which were once limited to the confines of healthcare setups are now donned by the members of the general public. It has become imperative that a healthcare worker selects the right kind of respiratory protective equipment to protect himself and his patients. The surgical mask has become essential, in a way, for survival.

Garcia Godoy, L. R., et al. (2020). "Facial protection for healthcare workers during pandemics: a scoping review." BMJ Glob Health **5**(5).

BACKGROUND: The coronavirus disease 2019 (COVID-19) pandemic has led to personal protective equipment (PPE) shortages, requiring mask reuse or improvisation. We provide a review of medical-grade facial protection (surgical masks, N95 respirators and face shields) for healthcare workers, the safety and efficacy of decontamination methods, and the utility of alternative strategies in emergency shortages or resource-scarce settings. METHODS: We conducted a scoping review of PubMed and grey literature related to facial protection and potential adaptation strategies in the setting of PPE shortages (January 2000 to March 2020). Limitations included few COVID-19-specific studies and exclusion of non-English language articles. We conducted a narrative synthesis of the evidence based on relevant healthcare settings to increase practical utility in decision-making. RESULTS: We retrieved 5462 peer-reviewed articles and 41 grey literature records. In total, we included 67 records which met inclusion criteria. Compared with surgical masks, N95 respirators perform better in laboratory testing, may provide superior protection in inpatient settings and perform equivalently in outpatient settings. Surgical mask and N95 respirator conservation strategies include extended use, reuse or decontamination, but these strategies may result in inferior protection. Limited evidence suggests that reused and improvised masks should be used when medical-grade protection is unavailable. CONCLUSION: The COVID-19 pandemic has led to critical shortages of medical-grade PPE. Alternative forms of facial protection offer inferior protection. More robust evidence is required on different types of medical-grade facial protection. As research on COVID-19 advances, investigators should continue to examine the impact on alternatives of medical-grade facial protection.

García, I. G., et al. (2020). "A randomized multicenter clinical trial to evaluate the efficacy of melatonin in the prophylaxis of SARS-CoV-2 infection in high-risk contacts (MeCOVID Trial): A structured summary of a study protocol for a randomised controlled trial." Trials **21**(1): 466.

OBJECTIVES: Primary objective: to evaluate the efficacy of melatonin as a prophylactic treatment on prevention of symptomatic SARS-CoV-2 infection among healthcare workers at high risk of SARS-CoV-2 exposure. Secondary objectives: To evaluate the efficacy of melatonin as a prophylactic treatment on prevention of asymptomatic SARS-CoV-2 infection.To evaluate the efficacy of melatonin to prevent the development of severe COVID-19 in the participants enrolled in this study who develop SARS-CoV-2 infection along the trial.To evaluate the duration of COVID-19 symptoms in participants receiving melatonin before the infection.To evaluate seroconversion timing post-symptom onset. Exploratory objectives:To compare severity of COVID-19 between men and women.To evaluate the influence of sleep and diet on prevention from SARS-CoV-2 infection.To evaluate the effect of melatonin on the incidence and characteristics of lymphopenia and increase of inflammatory cytokines related to COVID-19. TRIAL DESIGN: This is a two-arm parallel randomised double-blind controlled trial to evaluate the efficacy of melatonin versus placebo in the prophylaxis of coronavirus disease 2019 among healthcare workers. PARTICIPANTS: Inclusion Criteria: Male or female participants ≥ 18 and ≤ 80 years of age.Healthcare workers from the public and private Spanish hospital network at risk of SARS-CoV 2 infection.Not having a previous COVID19 diagnosis.Understanding the purpose of the trial and not having taken any pre-exposure prophylaxis (PrEP) including HIV PrEP from March 1(st) 2020 until study enrolment.Having a negative SARS-CoV 2 reverse-transcription PCR (RT-PCR) result or a negative serologic rapid test (IgM/IgG) result before randomization.Premenopausal women must have a negative urinary pregnancy test in the 7 days before starting the trial treatment.Premenopausal women and males with premenopausal couples must commit to using a high efficiency anticonceptive method. EXCLUSION CRITERIA: HIV infection.Active hepatitis B infection.Renal failure (CrCl < 60 mL/min/1.73 m2) or need for hemodialysis.Osteoporosis.Myasthenia gravis.Pre-existent maculopathy.Retinitis pigmentosa.Bradycardia (less than 50 bpm).Weight less than 40 Kg.Participant with any immunosuppressive condition or hematological disease.Treatment with drugs that may prolong QT in the last month before randomization for more than 7 days including: azithromycin, chlorpromazine, cisapride, clarithromycin, domperidone, droperidol, erythromycin, halofantrine, haloperidol, lumefantrine, mefloquine, methadone, pentamidine, procainamide, quinidine, quinine, sotalol, sparfloxacin, thioridazine, amiodarone.Hereditary intolerance to galactose, Lapp lactase deficiency or glucose or galactose malabsorption.Treatment with fluvoxamine.Treatment with benzodiazepines or benzodiazepine analogues such as zolpidem, zopiclone or zaleplon.Pregnancy.Breastfeeding.History of potentially immune derived diseases such as: lupus, Crohn's disease, ulcerative colitis, vasculitis or rheumatoid arthritis.Insulin-dependent diabetes mellitus.Known history of hypersensitivity to the study drug or any of its components.Patients that should not be included in the study at the judgment of the research team. Participants will be recruited from the following eight hospitals in Madrid, Spain: Hospital Universitario La Paz, Hospital Ramón y Cajal, Hospital Infanta Sofía, Hospital 12 de Octubre, Hospital Clínico San Carlos, Hospital Central de la defensa Gómez Ulla,Hospital de La Princesa and Hospital Infanta Leonor. INTERVENTION AND COMPARATOR: Experimental: Melatonin (Circadin®, Exeltis Healthcare, Spain): 2 mg of melatonin orally before bedtime for 12 weeks. Comparator: Identical looking placebo (Laboratorios Liconsa, Spain) orally before bedtime for 12 weeks. MAIN OUTCOMES: Number of SARS-CoV-2 (COVID-19) symptomatic infections confirmed by polymerase chain reaction (PCR) test or serologic test or according to each centre diagnosis protocol. Primary outcome will be measured until the end of treatment for each participant (until the date of the last dose taken by each patient). RANDOMISATION: Patients who meet all inclusion and no exclusion criteria will be randomised, stratified by centres, sex and age (<50 and ≥ 50 years old). The randomisation sequence was created using SAS version 9.4 statistical software (procedure 'PROC PLAN') with a 1:1 allocation. No randomisation seed was specified. The randomisation seed was generated taking the hour of the computer where the program was executed. Randomization will be done centrally through the electronic system RedCAP® in order to conceal the sequence until interventions are assigned BLINDING (MASKING): Participants, caregivers, and those assessing the outcomes are blinded to group assignment. NUMBERS TO BE RANDOMISED (SAMPLE SIZE): A total of 450 participants are planned to be enrolled in this clinical trial, 225 in the experimental arm and 225 in the placebo arm. TRIAL STATUS: Protocol version 3.0, 17th of April 2020. Recruitment ongoing. First participant was recruited on the 21st of April 2020. The final participant is anticipated to be recruited on the 31st of May 2020. As of May 18th, 2020, a total of 312 participants have been enrolled (154 at Hospital La Paz, 85 at Hospital Infanta Sofía and 73 at Hospital 12 de Octubre). TRIAL REGISTRATION: EU Clinical Trials Register: 2020-001530-35; Date of trial registration: 13th of April 2020; https://www.clinicaltrialsregister.eu/ctr-search/trial/2020-001530-35/ES FULL PROTOCOL: The full protocol is attached as an additional file, accessible from the Trials website (Additional file 1). In the interest in expediting dissemination of this material, the familiar formatting has been eliminated; this Letter serves as a summary of the key elements of the full protocol.

Garcia-Castrillo, L., et al. (2020). "European Society For Emergency Medicine position paper on emergency medical systems' response to COVID-19." Eur J Emerg Med **27**(3): 174-177.

The 2019 novel coronavirus acute respiratory epidemic is creating a stressed situation in all the health systems of the affected countries. Emergency medical systems and specifically the emergency departments as the front line of the health systems are suffering from overload and severe working conditions, the risk of contagion and transmission of the health professionals adds a substantial burden to their daily work. Under the perspective of European Society For Emergency Medicine, the recommendations provided by the health authorities are reviewed focus on the emergency department's activity.

Garcia-Portabella, M., et al. (2020). "Patient outcomes after humeral fracture surgery during the COVID-19 outbreak in Spain." J Shoulder Elbow Surg **29**(8): 1513-1521.

BACKGROUND: The global spread of the 2019 novel coronavirus (COVID-19) has profoundly affected the way we conduct our health care practices. The goal of this paper is to report the outcomes of 11 patients who underwent humeral fracture surgery in the first few weeks of the COVID-19 outbreak in Spain. METHODS: This was a retrospective case series of 11 patients who underwent humeral fracture surgery during the first weeks of the COVID-19 outbreak in Spain, between March 10 and April 25, 2020. The clinical outcomes of these operative patients were monitored up to May 12, 2020, the final date of follow-up, a minimum of 15 days after all patients were discharged from the hospital. COVID-19 infection, mortality, demographic, clinical, and laboratory data were analyzed. RESULTS: Thirty-six humeral fractures were recorded between March 10 and April 25, 2020. During this period, humeral fracture fixation was the third most common surgery for fracture in our institution after hip fracture and ankle fracture surgery. Eleven patients underwent surgery (30.5%), of whom 7 were women (63.3%). The mean age was 64.8 years (standard deviation, 13.5). Nine operated cases had a proximal humerus fracture and 2 had a humeral shaft fracture. One of the 11 patients was positive for SARS-CoV-2 on the basis of the quantitative reverse transcription polymerase chain reaction of throat swab samples. The overall median surgical time was 101.2 minutes (standard deviation, 28.4). The overall median hospital length stay for the patient discharged was 2.2 days (range, 1-4 days). No COVID-19 nosocomial intrahospital infection occurred, and no patient reported COVID-19 infection during the 15 days after hospital discharge. No intrahospital mortality was recorded. Furthermore, no COVID-19 infection was reported in the shoulder surgeons who performed the surgeries. CONCLUSIONS: Although humeral fractures were not the most frequent fractures during this outbreak, some required surgery. With good preoperative management that included reverse transcription polymerase chain reaction for COVID-19 and chest radiographs, protective measurements for the surgical team, and rapid discharge of the patients, we were able to operate on 11 humeral fractures with no COVID-19 nosocomial intrahospital infection in the patients or in the shoulder surgeons who performed the surgeries.

Garry, S., et al. (2020). "Considerations for planning COVID-19 treatment services in humanitarian responses." Confl Health **14**(1): 80.

The COVID-19 pandemic has the potential to cause high morbidity and mortality in crisis-affected populations. Delivering COVID-19 treatment services in crisis settings will likely entail complex trade-offs between offering services of clinical benefit and minimising risks of nosocomial infection, while allocating resources appropriately and safeguarding other essential services. This paper outlines considerations for humanitarian actors planning COVID-19 treatment services where vaccination is not yet widely available. We suggest key decision-making considerations: allocation of resources to COVID-19 treatment services and the design of clinical services should be based on community preferences, likely opportunity costs, and a clearly articulated package of care across different health system levels. Moreover, appropriate service planning requires information on the expected COVID-19 burden and the resilience of the health system. We explore COVID-19 treatment service options at the patient level (diagnosis, management, location and level of treatment) and measures to reduce nosocomial transmission (cohorting patients, protecting healthcare workers). Lastly, we propose key indicators for monitoring COVID-19 health services.

Garry, S., et al. (2020). "Considerations for planning COVID-19 treatment services in humanitarian responses." Confl Health **14**: 80.

The COVID-19 pandemic has the potential to cause high morbidity and mortality in crisis-affected populations. Delivering COVID-19 treatment services in crisis settings will likely entail complex trade-offs between offering services of clinical benefit and minimising risks of nosocomial infection, while allocating resources appropriately and safeguarding other essential services. This paper outlines considerations for humanitarian actors planning COVID-19 treatment services where vaccination is not yet widely available. We suggest key decision-making considerations: allocation of resources to COVID-19 treatment services and the design of clinical services should be based on community preferences, likely opportunity costs, and a clearly articulated package of care across different health system levels. Moreover, appropriate service planning requires information on the expected COVID-19 burden and the resilience of the health system. We explore COVID-19 treatment service options at the patient level (diagnosis, management, location and level of treatment) and measures to reduce nosocomial transmission (cohorting patients, protecting healthcare workers). Lastly, we propose key indicators for monitoring COVID-19 health services.

Garzaro, G., et al. (2020). "COVID-19 infection and diffusion among the healthcare workforce in a large university-hospital in northwest Italy." Med Lav **111**(3): 184-194.

Backgroud: Since the beginning of the coronavirus disease 2019 (COVID-19) outbreak, healthcare workers (HCWs) have been the workers most likely to contract the disease. Intensive focus is therefore needed on hospital strategies that minimize exposure and diffusion, confer protection and facilitate early detection and isolation of infected personnel. METHODS: To evaluate the early impact of a structured risk-management for exposed COVID-19 HCWs and describe how their characteristics contributed to infection and diffusion. Socio-demographic and clinical data, aspects of the event-exposure (date, place, length and distance of exposure, use of PPE) and details of the contact person were collected. RESULTS: The 2411 HCWs reported 2924 COVID-19 contacts. Among 830 HCWs who were at 'high or medium risk', 80 tested positive (9.6%). Physicians (OR=2.03), and non-medical services -resulted in an increased risk (OR=4.23). Patient care did not increase the risk but sharing the work environment did (OR=2.63). There was a significant time reduction between exposure and warning, exposure and test, and warning and test since protocol implementation. HCWs with management postitions were the main source of infection due to the high number of interactions. DISCUSSION: A proactive system that includes prompt detection of contagious staff and identification of sources of exposure helps to lower the intra-hospital spread of infection. A speedier return to work of staff who would otherwise have had to self-isolate as a precautionary measure improves staff morale and patient care by reducing the stress imposed by excessive workloads arising from staff shortages.

Publisher: Abstract available from the publisher.

ita

Garzaro, G., et al. (2020). "[COVID-19 and work environment: legislative developments about the role of the occupational physician in the emergency management and in the SARS-CoV-2-related work accidents notification]." G Ital Med Lav Ergon **42**(3): 195-200.

Severe Acute Respiratory Syndrome Coronavirus 2 (SARS-CoV-2) and its associated disease (COVID-19) represent a global health emergency that requires integrated and multidisciplinary intervention by international medical and scientific communities, in support of the national governments. In Italy many public health measures have been adopted to contain the transmission of the disease, which also involved occupational physicians. The regulatory path has had a rapid evolution due to the trend of infections and progressive scientific evidence: the most recent Circular from the Ministry of Labor and Social Policies and the Ministry of Health provides for the termination of the "exceptional health surveillance" activity, the management and protection of fragile individuals by activating the medical examination on request of the employee and excludes the old age, without comorbidities, as a specific condition of risk of serious complication of SARS-CoV-2 infection. For preventive and insurance purposes, COVID-19 usually represents a generic biological risk, for which the same measures must be adopted for the entire population. In the Inail Circular no. 13 of April 3, 2020, however, it is stated that professional categories that perform front office duties are considered exposed to a higher risk, as well as, health professionals, can be considered exposed to a specific risk. From January 1st to July 31st 2020, 51,363 cases of infection from SARS-CoV-2 were reported to INAIL as an accident. In the same period INAIL noticed a decrease in reports of accidents and occupational disease overall, correlated to the effects of lockdown and smart working.

Gasparino, R. C., et al. (2021). "Prophylactic dressings in the prevention of pressure ulcer related to the use of personal protective equipment by health professionals facing the COVID-19 pandemic: A randomized clinical trial." Wound Repair Regen **29**(1): 183-188.

Device-related pressure injury (DRPI) is a serious problem that is affecting professionals working on the front lines against COVID-19 due to the prolonged use of personal protective equipment (PPE). In addition to the physical and psychological integrity of professionals, these injuries can compromise the quality of care. Therefore, using technologies to prevent this adverse effect is an urgent matter. This is a parallel two-arm randomized clinical trial without the use of a control group to compare the use of foam and extra-thin hydrocolloid in preventing DRPI associated with the use of PPE by health professionals working on the front lines against coronavirus. In total, 88 professionals were divided into two groups: foam and hydrocolloid. Data were collected using two instruments and related to demographic and professional characteristics and skin evaluation. Each volunteer received one of the dressings, both with the same dimensions and arranged over similar regions, and data were gathered at baseline and after 6 or 12 hours. Descriptive and inferential analytic statistical methods were used; the significance level adopted was 5%. No participant developed DRPI, but four areas with hyperemia were observed in the foam group (two in the forehead, one in the cheeks, and one in the nose bridge), as well as four areas with hyperemia in the hydrocolloid group (two in the nose bridge, one in the right ear, and one in the left ear). There was no difference between the groups regarding skin conditions and discomfort (P > .05). The average cost obtained was $ 5.8/person and $ 4.4/person in the foam group and the hydrocolloid group, respectively, considering the dressing measurements. The results show that foam and extra-thin hydrocolloid were effective in preventing DRPI associated with the use of PPE.

Ge, X. Y., et al. (2020). "Evaluation of the exposure risk of SARS-CoV-2 in different hospital environment." Sustain Cities Soc **61**: 102413.

The ongoing coronavirus disease 2019 (COVID-19) pandemic caused by the severe acute respiratory syndrome coronavirus 2 (SARS-CoV-2) has imposed a significant impact on social and economic activities. As a high infectious pathogen, the existence of SARS-CoV-2 in public space is very important for its transmission. During the COVID-19 pandemic, hospitals are the main places to deal with the diseases. In this work, we evaluated the exposure risk of SARS-CoV-2 in hospital environment in order to protect healthcare workers (HCWs). Briefly, air and surface samples from 6 different sites of 3 hospitals with different protection levels were collected and tested for the SARS-CoV-2 nucleic acid by reverse transcription real-time fluorescence PCR method during the COVID-19 epidemic. We found that the positive rate of SARS-CoV-2 nucleic acid was 7.7 % in a COVID-19 respiratory investigation wards and 82.6 % in a ICUs with confirmed COVID-19 patients. These results indicated that in some wards of the hospital, such as ICUs occupied by COVID-19 patients, the nucleic acid of SARS-CoV-2 existed in the air and surface, which indicates the potential occupational exposure risk of HCWs. This study has clarified retention of SARS-CoV-2 in different sites of hospital, suggesting that it is necessary to monitor and disinfect the SARS-CoV-2 in hospital environment during COVID-19 pandemic, and will help to prevent the iatrogenic infection and nosocomial transmission of SARS-CoV-2 and to better protect the HCWs.

Gee, S., et al. (2020). "Management of clozapine treatment during the COVID-19 pandemic." Ther Adv Psychopharmacol **10**: 2045125320928167.

Clozapine is the only available treatment for refractory schizophrenia but its use involves frequent physical contact with healthcare workers for the purpose of mandatory blood monitoring. During the COVID-19 pandemic, patients taking clozapine will be self-isolating to reduce the risk of infection, not least because these patients are at high risk of serious illness and fatality because of high rates of diabetes, obesity and pulmonary disease and an increased risk of pneumonia. Problems may also arise because both clozapine-induced myocarditis and neutropenic sepsis share signs and symptoms with COVID-19 (fever, chest pain, dyspnoea, etc.). We recommend decreasing the frequency of physical contacts by extending the blood monitoring interval to 12 weeks in those patients taking clozapine for more than 1 year. To distinguish COVID-19 from clozapine-related physical adverse effects, we suggest an urgent antigen test alongside a full blood count. In those taking clozapine who develop COVID-19, we suggest continuing with clozapine whenever possible (even during ventilation), reducing the dose if necessary in line with blood assay results. Blood monitoring should continue but clozapine should only cease if there is a significant fall in neutrophils (COVID-19 is linked to lymphopenia but not neutropenia). To protect against the likelihood and severity of respiratory infection, we recommend the use of vitamin D in all clozapine patients. Initiation of clozapine is likely to remain problematic while the risk of infection remains, given the degree of physical contact required to assure safety.

Gefen, A. and K. Ousey (2020). "Update to device-related pressure ulcers: SECURE prevention. COVID-19, face masks and skin damage." J Wound Care **29**(5): 245-259.

The 2019 novel coronavirus disease (COVID-19) pandemic has brought the effects of device-related pressure ulcers (DRPU) into sharp focus. With the increased use of personal protective equipment (PPE), including face masks, continuous positive airway pressure (CAPP) masks and other devices, the incidence of DRPUs among health professionals and patients alike has risen starkly. As such, the Journal of Wound Care (JWC) consensus document, Device-related pressure ulcers: SECURE prevention, published in February 2020, is more relevant than ever. To help support patients and frontline health professionals, JWC is republishing the consensus in a digital format, along with a new introductory article outlining the DRPU risks posed by PPE and other medical devices used by patients and health professionals during the pandemic, and how the skin damage can be avoided. The aim is to provide frontline staff with a clear, simple strategy on how to prevent the risk of personal skin damage and/or DRPU during the pandemic, as well as point them in the direction of more indepth guidance on long-term strategies for prevention, for both themselves and patients.

Gelardi, M., et al. (2020). "Surgical mask and N95 in healthcare workers of Covid-19 departments: clinical and social aspects." Acta Biomed **91**(4): e2020171.

Introdution. In order to prevent or slow down the transmission of COVID-19, various public health measures have been introduced, including social distancing, environmental disinfection and the use of personal protective equipment (PPE). In this perspective, the clinical practice of healthcare professionals has changed dramatically. As a matter of fact, the use of surgical masks and N95 has significantly worsened the job performance of workers who deal directly with COVID-19 disease. METHODS: The study included 116 health workers employed in the pulmonology, intensive care and infectious diseases departments of Bari and Foggia Hospital, directly involved in the healthcare of patients affected by COVID-19. Between May 1, 2020 and May 31, 2020, each participant completed an online questionnaire aimed to investigate the impact of the COVID-19 pandemic on workers' lifestyle changes and job performances. We compared the results based on the type of mask used by each participant (surgical mask vs N95). RESULTS: Although disturbances related to the use of the mask arose earlier in subjects who wore the N95 (p = 0.0094), healthcare workers that wore surgical masks reported a statistically higher average score for a greater number of disorders. CONCLUSIONS: This is the first study that compares the effects of the two most used PPE on the quality of life of health workers and which highlights the greater discomfort caused by surgical masks. This result brings to light a serious social problem, being surgical masks widely used in everyday life by ordinary people and non-healthcare workers.

Gellings, P. S., et al. (2020). "Recent Advances in the Pursuit of an Effective Acinetobacter baumannii Vaccine." Pathogens **9**(12).

Acinetobacter baumannii has been a major cause of nosocomial infections for decades. The absence of an available vaccine coupled with emerging multidrug resistance has prevented the medical community from effectively controlling this human pathogen. Furthermore, the ongoing pandemic caused by SARS-CoV-2 has increased the risk of hospitalized patients developing ventilator-associated pneumonia caused by bacterial opportunists including A. baumannii. The shortage of antibiotics in the development pipeline prompted the World Health Organization to designate A. baumannii a top priority for the development of new medical countermeasures, such as a vaccine. There are a number of important considerations associated with the development of an A. baumannii vaccine, including strain characteristics, diverse disease manifestations, and target population. In the past decade, research efforts have revealed a number of promising new immunization strategies that could culminate in a safe and protective vaccine against A. baumannii. In this review, we highlight the recent progress in the development of A. baumannii vaccines, discuss potential challenges, and propose future directions to achieve an effective intervention against this human pathogen.

Geng, S., et al. (2021). "The influence of risk perception for COVID-19 pandemic on posttraumatic stress disorder in healthcare workers: A survey from four designated hospitals." Clin Psychol Psychother.

The aim of current study was to investigate risk perception of COVID-19 pandemic, sleep quality and time change of leisure activity and their correlations with posttraumatic stress disorder (PTSD) in healthcare workers (HCWs) from four designated hospitals in China. Medical staffs (n = 317) from three designated hospitals in Guangdong Province and one designated hospital in Guangxi Province were surveyed on their demographic information, sleep quality and time change of leisure activity, risk perception of pandemic and PTSD symptoms (by using PTSD checklist for DSM-5 (PCL-5)). Hierarchical regression and structural equation model (SEM) were used to examine the correlated factors of PTSD. The prevalence of high level of PTSD symptoms (PCL-5 > =33, a probable diagnosis of PTSD) was 10.7%. Regression analysis found that risk perception (dread: β = 0.142, p < 0.01; familiarity: β = 0.203, p < 0.01), sleep quality (β = 0.250, p < 0.001), time change of leisure activity (β = -0.179, p < 0.01), were independently correlated with PTSD severity, which was further confirmed by SEM. Locations of COVID-19-related hazards were significant different in cognitive map of risk perception between groups with high and low levels of PTSD symptoms. Risk perception of COVID-19 pandemic influenced PTSD symptoms in HCWs. Adequate time for leisure activity and good sleep quality protected some HCWs against PTSD symptoms under the influence of pandemic. More researches were warranted to understand the path from pre-factors of risk perception to its psychological consequences among HCWs.

Gentile, I., et al. (2020). "COVID-19: Time for Post-Exposure Prophylaxis?" Int J Environ Res Public Health **17**(11).

From a healthcare perspective, infection due to the novel coronavirus SARS-CoV-2 (severe acute respiratory syndrome coronavirus 2) and the ensuing syndrome called COVID-19 (coronavirus disease 2019) represents the biggest challenge the world has faced in several decades. Particularly worrisome are the high contagiousness of the virus and the saturation of hospitals' capacity due to overwhelming caseloads. Non-pharmaceutical interventions such as quarantine and inter-personal distancing are crucial to limiting the spread of the virus in the general population, but more tailored interventions may be needed at an individual level on a case-by-case basis. In this perspective, the most insidious situation is when an individual has contact with a contagious subject without adequate protection. If rapidly recognized afterwards, this occurrence may be promptly addressed through a post-exposure chemoprophylaxis (PEP) with antiviral drugs. This strategy has been implemented for other respiratory viruses (influenza above all) and was successfully used in South Korea among healthcare workers against the Middle East respiratory syndrome (MERS) coronavirus, by providing people who were exposed to high-risk contacts with lopinavir-ritonavir plus ribavirin. Initial experiences with the use of hydroxychloroquine to prevent COVID-19 also seem promising. Post-exposure chemoprophylaxis might help mitigate the spread of SARS-CoV-2 in the current phase of the COVID-19 pandemic.

Geoffroy, P. A., et al. (2020). "Psychological Support System for Hospital Workers During the Covid-19 Outbreak: Rapid Design and Implementation of the Covid-Psy Hotline." Front Psychiatry **11**: 511.

BACKGROUND: The worldwide coronavirus outbreak has put hospital workers under extreme stress with possible mental health problems. In this context, we decided to rapidly design and implement a psychological support system for all hospital workers in Paris during the Covid-19 outbreak. METHODS: We built a hotline in 3 days using the following steps: 1) official mandate, 2) request for the creation of hotline numbers, 3) formulation of psychological intervention materials and policies, 4) call for volunteer certified psychologists, 5) call for volunteer certified psychiatrists in case of psychiatric cases, 6) creation of an anonymous and protected database, and 7) communication and regular reminders about the existence of the hotline for hospital workers. RESULTS: After the first 26 days, we received 149 calls with a mean of 5.73 calls/day (SD=3.22). The average call duration was 18.5 min (min=1; max=65min; SD=14.7), and mostly women (86%) called. The mean age was 32.7 years old (SD=11.0). Calls from hospital workers were from all professions; though mostly represented by frontline healthcare workers, non-frontline departments also called (total of 44 departments). Reasons for calling were anxiety symptoms (n=73, 49%), request for hotline information (n=31, 20.8%), worries about Covid-19 (n=23, 15.44%), exhaustion (n=17, 11.41%), trauma reactivation (n=10, 6.11%), insomnia (n=9, 6.0%), anger (n=8, 5.37%), depressive (n=6, 4.02%), and psychotic symptoms (n=3, 2.01%). Regarding referrals, 105 (70.47%) of them were referred to psychosocial, Covid, and general support. CONCLUSIONS: This psychological support system can be easily duplicated and seems to benefit all hospital professions that all appeared psychologically affected.

George, M. P., et al. (2020). "How to Leverage Collaborations Between the BME Community and Local Hospitals to Address Critical Personal Protective Equipment Shortages During the COVID-19 Pandemic." Ann Biomed Eng **48**(9): 2281-2284.

The global COVID-19 pandemic disrupted supply chains across the world, resulting in a critical shortage of personal protective equipment (PPE) for frontline healthcare workers. To preserve PPE for healthcare providers treating COVID-19 positive patients and to reduce asymptomatic transmission, the Department of Bioengineering at the University of Colorado, Denver | Anschutz Medical Campus collaborated with National Jewish Health to design and test patterns for cloth face coverings. A public campaign to sew and donate the final pattern was launched and over 2500 face coverings have been donated as a result. Now that nearly three million cases of COVID-19 have been confirmed in the United States, many state and local governments are requiring cloth face coverings be worn in public. Here, we present the collaborative design and testing process, as well as the final pattern for non-patient facing hospital workers and community members alike.

Georger, F., et al. (2020). "[COV IMPACT: Stress exposure analysis among hospital staff in 2 hospitals in France during the COVID-19 pandemic]." Ann Cardiol Angeiol (Paris) **69**(5): 227-232.

The COVID-19 pandemic has swept through our hospitals which have had to adapt as a matter of urgency. We are aware that a health crisis of this magnitude is likely to generate mental disorders particularly affecting exposed healthcare workers. Being so brutal and global, this one-of the kind pandemic has been impacting the staff in their professional sphere but also within their private circle. The COV IMPACT study is an early assessment survey conducted for 2 weeks in May 2020, of the perception by all hospital workers of the changes induced in their professional activity by the pandemic. The study was carried out by a survey sent to the hospital staff of Béziers and Montfermeil. The readjusted working conditions were source of increased physical fatigue for 62 % of the respondents. Moral exhaustion was reported by 36 %. It was related to the stress of contracting the infection (72 %) but above all of transmitting it to relatives (89 %) with a broad perception of a vital risk (41 %). This stress affected all socio-professional categories (CSP) and was independent of exposure to COVID. Change in organisation, lack of information and protective gear and equipment were major factors of insecurity at the start of the epidemic. Work on supportive measures is necessary. It should focus on the spread of information, particularly towards the youngest, as well as bringing more psychological support and a larger amount of medical equipment, beyond healthcare workers and the COVID sectors.

Publisher: Abstract available from the publisher.

fre

Gesser-Edelsburg, A., et al. (2020). "A mixed-methods sequential explanatory design comparison between COVID-19 infection control guidelines' applicability and their protective value as perceived by Israeli healthcare workers, and healthcare executives' response." Antimicrob Resist Infect Control **9**(1): 148.

BACKGROUND: Healthcare workers (HCWs) are on the front line of the COVID-19 outbreak, and their constant exposure to infected patients and contaminated surfaces puts them at risk of acquiring and transmitting the infection. Therefore, they must employ protective measures. In practice, HCWs in Israel were not fully prepared for this sudden COVID-19 outbreak. This research aimed to identify and compare: (1) Israeli HCWs' perceptions regarding the official COVID-19 guidelines' applicability and their protective value, and (2) HCWs executives' response to HWCs' concern regarding personal protective equipment (PPE) shortage. METHODS: A mixed-methods sequential explanatory design consists of: (1) An online survey of 242 HCWs about the application of the guidelines and PPE, and (2) Personal interviews of 15 HCWs executives regarding PPE shortage and the measures they are taking to address it. RESULTS: A significant difference between the perceived applicability and protective value was found for most of the guidelines. Some of the guidelines were perceived as more applicable than protective (hand hygiene, signage at entrance, alcohol rub sanitizers at entrance, and mask for contact with symptomatic patients). Other were perceived as less applicable than protective (prohibited gathering of over 10 people, maintaining a distance of 2 m', and remote services). CONCLUSIONS: HCWs need the support of the healthcare authorities not only to provide missing equipment, but also to communicate the risk to them. Conveying the information with full transparency, while addressing the uncertainty element and engaging the HCWs in evaluating the guidelines, are critical for establishing trust.

Ghafoor, D., et al. (2021). "Excessive use of disinfectants against COVID-19 posing a potential threat to living beings." Curr Res Toxicol **2**: 159-168.

The severe acute respiratory syndrome coronavirus 2 (SARS-CoV-2) emerged in Wuhan city of China in late December 2019 and identified as a novel coronavirus disease 2019 (COVID-19). On January 30, the World Health Organization (WHO) declared the coronavirus outbreak a global public health emergency. The rapid spread of the pathogen across the communities shock the entire population. As no existing therapy were available during the pandemic. Health professionals recommended frequent washing of hands with soap and alcohol-based sanitizers. Disinfectants were extensively sprayed to minimize the possibility of getting COVID-19. Despite the potential benefits of these germicidal agents against COVID-19. Alcohol-based hand sanitizers lead to dry skin, infection, and alcohol poisoning. Children are considered more prone to alcohol poisoning and other major health concern. Precautionary measures should be ensured to protect the community from the possible risk associated with disinfectants.

Ghai, S. (2020). "Are dental schools adequately preparing dental students to face outbreaks of infectious diseases such as COVID-19?" J Dent Educ **84**(6): 631-633.

The recent 2019-novel coronavirus (2019-nCoV, also known as SARS-CoV-2) has caused >2,622,571 confirmed cases of coronavirus disease 2019 (COVID-19) in >185 countries, and >182,359 deaths globally. More than 9000 healthcare workers have also been infected by 2019-nCoV. Prior to the present pandemic of COVID-19, there have been multiple large-scale epidemics and pandemics of other viral respiratory infections, such as seasonal flu, Spanish flu (H1N1), severe acute respiratory syndrome (SARS), Middle East respiratory syndrome (MERS), and others. Dental professionals are at an increased risk for contracting these viruses from dental patients, as dental practice involves face-to-face communication with the patients and frequent exposure to saliva, blood, and other body fluids. Dental education can play an important role in the training of dentists, helping them to adopt adequate knowledge and attitudes related to infection control measures. The current dental curriculum does not cover infection control adequately, especially from airborne pathogens. Infection control education needs to be included in the dental curriculum itself, and students should be trained adequately to protect them and prevent the infection from disseminating even before they see their first patient.

Ghazali, W., et al. (2020). "Gynecological Endoscopic Society of Malaysia Statement and Recommendations on Gynecological Laparoscopic Surgery during COVID-19 Pandemic." Gynecol Minim Invasive Ther **9**(4): 185-189.

OBJECTIVES: While the issue of aerosolization of virus from the blood occurs during usage of energy sources scare practitioners, there have been no reported instances of healthcare workers (HCWs) being infected. COVID-19 virus is primarily transmitted via respiratory droplets and contact routes. Therefore, the ultimate decision for surgery, should be based on which is the safest, quickest route and concurrently ensuring that HCWs are protected during these surgeries. During the time of crisis, HCWs need to concentrate and channel resources to the care of those affected by the coronavirus hence judicious allocation of resources is mandatory. We present the guidelines and recommendations on gynecological laparoscopic surgery during this COVID-19 outbreak in Malaysia. MATERIALS AND METHODS: Thorough search of articles and recommendations were done to look into the characteristics of the virus in terms of transmission and risks during surgery. Smoke plume characteristics, composition and risk of viral transmission were also studied. Search includes The WHO Library, Cochrane Library and electronic databases (PubMed, Google scholar and Science Direct). CONCLUSION: We concluded that there is no scientific basis of shunning laparoscopic approach in surgical intervention. Ultimately, the guiding principles would be of reducing the anesthetic and surgical duration, the availability of full protective gear for HCWs during the surgery and the status of the patient. It is mandatory for viral swab tests to be done within the shortest window period possible, for all cases planned for surgery.

Gheisari, M., et al. (2020). "Skin reactions to non-glove personal protective equipment: an emerging issue in the COVID-19 pandemic." J Eur Acad Dermatol Venereol **34**(7): e297-e298.

Protecting healthcare workers (HCWs) is crucial during Corona Virus Disease 2019 pandemic and requires wearing personal protective equipment (PPE) [1]. While most of the studies have focused on the skin reactions caused by gloves, other PPE such as gowns, respirator masks, face shields and goggles are also worn by HCWs for long hours during the current epidemic and skin irritations caused by these equipment may cause discouragement of health workers from using them [2]. In this letter we have focused on the reaction caused by non‐glove PPE.

Ghimire, P., et al. (2020). "Knowledge, Attitude and Practice of healthcare workers Towards Coronavirus Disease 2019 (COVID-19) Pandemic." J Nepal Health Res Counc **18**(2): 293-300.

BACKGROUND: The emergence of SARS-CoV 2 pandemic is the greatest public health concern of the century. Healthcare workers are the front liners of pandemic management. Their knowledge, attitudes and practices can influence the outcome of pandemic. The study aims to determine the knowledge, attitude and practices among healthcare workers. METHODS: Knowledge, attitude and practice related questionnaire based online survey through Google forms was conducted from Healthcare workers, following ethical approval from NHRC (ERB protocol registration number: 297/2020 P). Online forms were disseminated via Facebook, Instagram, Whatsapp, Viber and personal messaging. Responses containing anonymized data was collected analyzed in using SPSS-version 26, (Year: 2019). The results were interpreted in terms of percentage response, knowledge score, and practice and discussed on the possible solutions in improving the infection prevention and control practice. RESULTS: 473 HCWs responded to the questionnaire, out of which 426 responses met inclusion criteria which is 90% of the total responses. The mean score for knowledge was 3.20 + 1.15 out of maximum seven; with 45.7% correct answers. 70.4 % (300) participants felt that Nepal will be unable to contain the pandemic, 64 % of the total employed participants felt that there was inadequate institutional preparedness to protect HCWs from exposure. 91% participants reported practicing hand hygiene after every patient encounter; whereas 31.7% (77) and 22.1% (59) participants did not know how to check sealing of the N95 masks and use personal protective equipment's respectively. CONCLUSIONS: The survey findings showed deficiencies in knowledge and appropriate practice among the HCWs, in prevention of SARS-CoV-2 transmission. Attitudes towards COVID19 were a mix of both positive and negative viewpoints. Even in health care workers with access to internet, there is significant gap in universal infection prevention and control practices required for self-protection and limiting untoward transmission.

Gholami, M., et al. (2021). "COVID-19 and healthcare workers: A systematic review and meta-analysis." Int J Infect Dis **104**: 335-346.

BACKGROUND: The COVID-19 pandemic has focused attention on the challenges and risks faced by frontline healthcare workers (HCW). This study aimed to describe the clinical outcomes and risk factors for SARS-CoV-2 infection in HCW. METHODS: Three databases were surveyed and 328 articles were identified. Of these, 225 articles did not meet inclusion criteria; therefore, 97 full-text article were reviewed. Finally, after further revision, 30 articles were included in the systematic review and 28 were used for meta-analysis. RESULTS: Twenty-eight studies were identified involving 119,883 patients. The mean age of the patients was 38.37 years (95% CI 36.72-40.03) and males comprised 21.4% (95% CI 12.4-34.2) of the population of HCW. The percentage of HCW who tested positive for COVID-19 was 51.7% (95% CI 34.7-68.2). The total prevalence of comorbidities in seven studies was 18.4% (95% CI 15.5-21.7). The most prevalent symptoms were fever 27.5% (95% CI 17.6-40.3) and cough 26.1% (95% CI 18.1-36). The prevalence of hospitalisation was 15.1% (95% CI 5.6-35) in 13 studies and of death was 1.5% (95% CI 0.5-3.9) in 12 studies. Comparisons of HCW with and without infection showed an increased relative risk for COVID-19 related to personal protective equipment, workplace setting, profession, exposure, contacts, and testing. CONCLUSION: A significant number of HCW were reported to be infected with COVID-19 during the first 6 months of the COVID-19 pandemic, with a prevalence of hospitalisation of 15.1% and mortality of 1.5%. Further data are needed to track the continued risks in HCW as the pandemic evolves and health systems adapt.

Giacalone, S., et al. (2021). "Facial dermatoses in the general population due to wearing of personal protective masks during the COVID-19 pandemic: first observations after lockdown." Clin Exp Dermatol **46**(2): 368-369.

Since SARS‐COV‐2 pandemic began, frontline healthcare workers demonstrated to develop facial dermatoses, such as acne, rosacea and seborrheic dermatitis, secondary to prolonged use of personal protective equipment (PPE)

Giardino, F. R., et al. (2020). "Taking care of healthcare workers during COVID-19 pandemic: does plastic surgery have a role?" Acta Biomed **91**(4): e2020160.

BACKGROUND: The massive use of personal protective equipment is required by the medical and paramedical staff of the COVID-19 dedicated departments. This often causes painful pressure injuries. The aim of this study is to value the use of anesthetic cream and collagen veil masks for the treatment of pain. We also evaluated the possible psychological impact on healthcare workers involved in the emergency, which is currently a hot topic in the worldwide literature. MATERIALS AND METHODS: We applied lidocaine idrochloride 5% cream and a collagen veil mask respectively  before and after each work shift. We evaluated the improvement of pain symptoms through VAS assessments. We used a modified Maslach burnout inventory as well to evaluate the psychological impact of our treatments on  healthcare workers. RESULTS: A significant reduction of pain was detected within the first 3 days, but this was not significant over the long period (10 days). Otherwise, we saw a significant improvement of the psychological assessment (p<0,05). CONCLUSIONS: Parameters such as pain or psychological stress are not objective, but, anyway, our data show a  reduction of pain due to continuative PPE wearing and a significant improvement of psychological wellness of healthcare workers from two different countries, a finding which should not be undervalued by all the health caregivers not directly involved in COVID-19 pandemic.

Gibani, M. M., et al. (2020). "Assessing a novel, lab-free, point-of-care test for SARS-CoV-2 (CovidNudge): a diagnostic accuracy study." Lancet Microbe **1**(7): e300-e307.

BACKGROUND: Access to rapid diagnosis is key to the control and management of severe acute respiratory syndrome coronavirus 2 (SARS-CoV-2). Laboratory RT-PCR testing is the current standard of care but usually requires a centralised laboratory and significant infrastructure. We describe our diagnostic accuracy assessment of a novel, rapid point-of-care real time RT-PCR CovidNudge test, which requires no laboratory handling or sample pre-processing. METHODS: Between April and May, 2020, we obtained two nasopharyngeal swab samples from individuals in three hospitals in London and Oxford (UK). Samples were collected from three groups: self-referred health-care workers with suspected COVID-19; patients attending emergency departments with suspected COVID-19; and hospital inpatient admissions with or without suspected COVID-19. For the CovidNudge test, nasopharyngeal swabs were inserted directly into a cartridge which contains all reagents and components required for RT-PCR reactions, including multiple technical replicates of seven SARS-CoV-2 gene targets (rdrp1, rdrp2, e-gene, n-gene, n1, n2 and n3) and human ribonuclease P (RNaseP) as sample adequacy control. Swab samples were tested in parallel using the CovidNudge platform, and with standard laboratory RT-PCR using swabs in viral transport medium for processing in a central laboratory. The primary analysis was to compare the sensitivity and specificity of the point-of-care CovidNudge test with laboratory-based testing. FINDINGS: We obtained 386 paired samples: 280 (73%) from self-referred health-care workers, 15 (4%) from patients in the emergency department, and 91 (23%) hospital inpatient admissions. Of the 386 paired samples, 67 tested positive on the CovidNudge point-of-care platform and 71 with standard laboratory RT-PCR. The overall sensitivity of the point-of-care test compared with laboratory-based testing was 94% (95% CI 86-98) with an overall specificity of 100% (99-100). The sensitivity of the test varied by group (self-referred healthcare workers 94% [95% CI 85-98]; patients in the emergency department 100% [48-100]; and hospital inpatient admissions 100% [29-100]). Specificity was consistent between groups (self-referred health-care workers 100% [95% CI 98-100]; patients in the emergency department 100% [69-100]; and hospital inpatient admissions 100% [96-100]). Point of care testing performance was similar during a period of high background prevalence of laboratory positive tests (25% [95% 20-31] in April, 2020) and low prevalence (3% [95% 1-9] in inpatient screening). Amplification of viral nucleocapsid (n1, n2, and n3) and envelope protein gene (e-gene) were most sensitive for detection of spiked SARS-CoV-2 RNA. INTERPRETATION: The CovidNudge platform was a sensitive, specific, and rapid point of care test for the presence of SARS-CoV-2 without laboratory handling or sample pre-processing. The device, which has been implemented in UK hospitals since May, 2020, could enable rapid decisions for clinical care and testing programmes. FUNDING: National Institute of Health Research (NIHR) Imperial Biomedical Research Centre, NIHR Health Protection Research Unit in Healthcare Associated Infections and Antimicrobial Resistance at Oxford University in partnership with Public Health England, NIHR Biomedical Research Centre Oxford, and DnaNudge.

Gibson, R. L., et al. (2021). "Treating COVID-19 Acute Severe Hypoxemic Respiratory Failure: First Case Report Utilizing Dexamethasone, Remdesivir, and Convalescent Plasma in Operation Inherent Resolve." Med J (Ft Sam Houst Tex)(Pb 8-21-01/02/03): 28-33.

Coronavirus 2019 (COVID-19) has spread across the globe with a concerningly high infectivity resulting in the World Health Organization deeming it a pandemic. It has resulted in thousands of deaths and placed enormous strain on communities, healthcare systems and healthcare workers as they battle shortages of ventilators, supplies, and difficulties in protecting patients and hospital staff alike. Challenges in managing the disease have led to new treatment and management strategies as healthcare teams struggle to adapt. We present the first case of COVID-19 managed in the austere deployed environment of Operation Inherent Resolve in which the patient was treated with dexamethasone, remdesivir, COVID-19 convalescent plasma, positive pressure ventilation, and proning. We discuss some of the inherent and unique challenges of caring for a patient in this resource constrained environment with a brief review of the literature on the treatment and management.

Giménez-Espert, M. D. C., et al. (2020). "Psychosocial Risks, Work Engagement, and Job Satisfaction of Nurses During COVID-19 Pandemic." Front Public Health **8**: 566896.

Context: COVID-19 pandemic is a serious health emergency that has affected countries all over the world. Health emergencies are a critical psychosocial risk factor for nurses. In general, psychosocial risks constitute serious problems as they impact workers' health, productivity, and efficiency. Despite their importance, few studies analyze nurses' psychosocial risks during a health emergency caused by a pandemic or analyze their perception of the emergency and its relation to such risks. Objectives: To analyze the perception of COVID-19 by nurses, especially about measures, resources, and impact on their daily work. Also, to analyze these professionals' psychosocial risks and the relationship between perception of COVID-19 and these risks. Methods: A descriptive correlational study was performed in a convenience sample of 92 nurses from two public hospitals in the Valencian Community (Spain), (74 women, 79.1%), aged 24-63 (M = 43.37, SD = 11.58). Data were collected via an online self-completed questionnaire during the rise of the pandemic from March 29 to April 8, when the number of infections went from 78,797 to 146,690. Results: The measures and resources available about COVID-19 are relatively low, and the impact on their work is high. Similarly, the most prominent psychosocial risks appear to be emotional work and workload. In contrast, nurses' work engagement is medium, and their satisfaction is high. Finally, there seems to be a negative and significant relationship between the information available to nurses, the measures implemented, and resources with some of their psychosocial risks, and a positive one with job satisfaction and work engagement. There is also a positive and significant relationship only between the impact of COVID-19 and their work inequality, but not for other risks. Conclusions: The resources, measures, and information can be a protective factor facing nurses' psychosocial risks, especially during a pandemic. Studying the relationships between psychosocial risk and perception of a health emergency would be relevant and fundamental to protecting and caring for nurses, health professionals, and society.

Giorgi, G., et al. (2020). "COVID-19-Related Mental Health Effects in the Workplace: A Narrative Review." Int J Environ Res Public Health **17**(21).

The Coronavirus Disease 2019 (COVID-19) pandemic has deeply altered social and working environments in several ways. Social distancing policies, mandatory lockdowns, isolation periods, and anxiety of getting sick, along with the suspension of productive activity, loss of income, and fear of the future, jointly influence the mental health of citizens and workers. Workplace aspects can play a crucial role on moderating or worsening mental health of people facing this pandemic scenario. The purpose of this literature review is to deepen the psychological aspects linked to workplace factors, following the epidemic rise of COVID-19, in order to address upcoming psychological critical issues in the workplaces. We performed a literature search using Google Scholar, PubMed, and Scopus, selecting papers focusing on workers' psychological problems that can be related to the workplace during the pandemic. Thirty-five articles were included. Mental issues related to the health emergency, such as anxiety, depression, post-traumatic stress disorder (PTSD), and sleep disorders are more likely to affect healthcare workers, especially those on the frontline, migrant workers, and workers in contact with the public. Job insecurity, long periods of isolation, and uncertainty of the future worsen the psychological condition, especially in younger people and in those with a higher educational background. Multiple organizational and work-related interventions can mitigate this scenario, such as the improvement of workplace infrastructures, the adoption of correct and shared anti-contagion measures, including regular personal protective equipment (PPE) supply, and the implementation of resilience training programs. This review sets the basis for a better understanding of the psychological conditions of workers during the pandemic, integrating individual and social perspectives, and providing insight into possible individual, social, and occupational approaches to this "psychological pandemic".

Girma, S., et al. (2020). "Risk perception and precautionary health behavior toward COVID-19 among health professionals working in selected public university hospitals in Ethiopia." PLoS One **15**(10): e0241101.

OBJECTIVE: This study was designed to determine risk perception and precautionary health behavior toward coronavirus disease (COVID-19) among health professionals working in selected public university hospitals of Ethiopia. METHODS: A web-based cross-sectional survey was used with health professionals working in selected public university hospitals of Ethiopia. A structured survey questionnaire consisted of socio-demographic, risk perception, and behavioral response items were used. The survey questions were designed on Google form. All academic and clinical staff invited to participate in the online survey, which was carried out from May 1-14, 2020. Data analysis was done using the Statistical Package for Social Sciences version 24. Descriptive statistics computed and the result is presented by tables and figures. RESULTS: A total of 273 health professionals participated in this study. The mean (± SD) age of participants was 31.03 ± 5.11. Study participants' overall mean score of perceived risk was 23.59 ± 4.75. The study participants' mean score of perceived vulnerability (4.01 ± 1.17) was higher than the human immunodeficiency virus, common cold, malaria, and tuberculosis. Regarding precautionary health behavior, the lowest mean score is for wearing gloves 1.82 ± 1.15. CONCLUSION: Participants mean score of perceived vulnerability of coronavirus disease was higher than some of the prevalent infectious disease in the area. Almost all participants applied recommended protective measures to the acceptable level, except for wearing mask and gloves. Therefore, there is a need to further intensification of more effective ways to support health professionals' adherence to major precautionary measure is important.

Glowacki, E. M., et al. (2021). "Identifying #addiction concerns on twitter during the COVID-19 pandemic: A text mining analysis." Subst Abus **42**(1): 39-46.

The 2019 Novel Coronavirus (COVID-19) is responsible for thousands of deaths and hospitalizations. To curb the spread of this highly transmissible disease, governments enacted protective guidelines for its citizens, including social distancing and stay-at-home orders. These restrictions on social interactions can be especially problematic for individuals managing or recovering from addiction given that treatment often involves access to services and resources that became limited or even unavailable at this time. Social media sites like Twitter serve as a space for users to post questions and concerns about timely topics and allow for researchers to track common themes among the public. The goal of this study was to identify how the public was discussing addiction on Twitter during the COVID pandemic. Methods: We performed a text mining analysis to analyze tweets that contained "addiction" and "covid" to capture posts from the public that illustrated comments and concerns about addiction during the COVID-19 pandemic. We report on 3,301 tweets captured between January 31 and April 23, 2020. The study was conducted in the United States, but contained tweets from multiple countries. Results: The most prevalent topics had to do with services offered by Acadia Healthcare and Serenity Healthcare Centers, attempts to manage time while home, difficulties of coping with alcoholism amidst rising sales of alcohol, and attention to ongoing health crises (e.g.,., opioids, vaping). Additional topics included affordable telehealth services, research from France on the relationship between nicotine and COVID-19, concerns about gambling addiction, and changing patterns in substance misuse as drug availability varies. Conclusions: Analyzing Twitter content enables health professionals to identify the public's concerns about addiction during the COVID-19 pandemic. Findings from text mining studies addressing timely health topics can serve as preliminary analyses for building more comprehensive models, which can then be used to generate recommendations for the larger public and inform policy.

Godderis, L., et al. (2020). "COVID-19: a new work-related disease threatening healthcare workers." Occup Med (Lond) **70**(5): 315-316.

Goel, M., et al. (2021). "Post-lockdown challenges for ophthalmologists during COVID-19 pandemic in India: A survey-based analysis." Indian J Ophthalmol **69**(4): 946-950.

PURPOSE: The purpose of this study is to evaluate the post-lockdown challenges during Coronavirus disease 2019 (COVID-19) pandemic amongst the ophthalmologists in India. METHODS: An online survey was sent to the practicing ophthalmologists across India. Data were collected from the responding ophthalmologists and analysed using Medcalc 16.4 software. RESULTS: A total of 794 responses were obtained. Most respondents (51%) were in the age group 30-50 years and were in independent practice (40.05%). Almost three-fourth of ophthalmologists resumed their surgical services after a gap of more than a month post-lockdown. Almost a third of the respondents had significant reduction in their surgical workload during this period. Significant fear of contracting COVID-19 infection in the operation theatres was reported while moderate difficulty was found in procuring protective gear during immediate post-national lockdown period. CONCLUSION: The pandemic has changed the ophthalmic practice significantly, with patient and staff safety becoming areas of major concern. Both financial and psychological concerns affecting healthcare workers need addressing for continued patient care.

Gogna, A., et al. (2020). "Diagnostic Ultrasound Services During the Coronavirus Disease (COVID-19) Pandemic." AJR Am J Roentgenol **215**(5): 1130-1135.

OBJECTIVE. Nosocomial transmission of coronavirus disease (COVID-19) to frontline health care workers is well known, and health care workers may inadvertently become vectors for onward transmission. Ultrasound (US) service providers are at significant risk because scanning usually requires prolonged close patient contact. The bulky US equipment may also facilitate fomite transmission of disease. With increasing use of point-of-care and portable diagnostic US services, the risk is substantial. CONCLUSION. Our experience and protocols may help service providers with their own effective response against COVID-19.

Goh, K. J., et al. (2020). "Preparing your intensive care unit for the COVID-19 pandemic: practical considerations and strategies." Crit Care **24**(1): 215.

The coronavirus disease 2019 (COVID-19) has rapidly evolved into a worldwide pandemic. Preparing intensive care units (ICU) is an integral part of any pandemic response. In this review, we discuss the key principles and strategies for ICU preparedness. We also describe our initial outbreak measures and share some of the challenges faced. To achieve sustainable ICU services, we propose the need to 1) prepare and implement rapid identification and isolation protocols, and a surge in ICU bed capacity; (2) provide a sustainable workforce with a focus on infection control; (3) ensure adequate supplies to equip ICUs and protect healthcare workers; and (4) maintain quality clinical management, as well as effective communication.

Goh, Y., et al. (2020). "The face mask: How a real protection becomes a psychological symbol during Covid-19?" Brain Behav Immun **88**: 1-5.

'The Mask' has become a byword and a precious possession universally. Except for its use by the medical fraternity, answers to the common questions-whether it provides enough protection, which type is optimal for the general public and who really needs to don it, remain poorly understood. For a frontline healthcare worker, wearing mask is a necessity as an important person protection equipment, it is perhaps the most-powerful psychological symbol for the general public. Surprisingly, it even undermines all other recommended practices of infection control and breaking the transmission chain of Covid-19, like hand washing, personal hygiene and social distancing. 'The mask' has evolved with time and yet there is a need to further improve the design for safety, tolerability and comfort. In this review we present the journey of face mask, originating from the first masks aimed at stopping the bad smell to its industrial use to its all-important place in the medical field. Various types of face masks, their filtration efficiency, reusability and current recommendations for their use are presented.

Goldberg, A. B., et al. (2020). "Expecting the unexpected: Echo laboratory preparedness in the time of COVID-19." Echocardiography **37**(8): 1272-1277.

COVID-19 poses a unique set of challenges to the healthcare system due to its rapid spread, intensive resource utilization, and relatively high morbidity and mortality. Healthcare workers are at especially high risk of exposure given the viruses spread through close contact. Reported cardiac complications of COVID-19 include myocarditis, acute coronary syndrome, cardiomyopathy, pericardial effusion, arrhythmia, and shock. Thus, echocardiography is integral in the timely diagnosis and clinical management of COVID-19 patients. Rush University Medical Center has been at the forefront of the COVID-19 response in Illinois with high numbers of cases reported in Chicago and surrounding areas. The echocardiography laboratory at Rush University Medical Center (RUMC) proactively took numerous steps to balance the imaging needs of a busy, nearly 700-bed academic medical center while maintaining safety.

Golladay, G. J., et al. (2021). "Rationale and process for N95 respirator sanitation and re-use in the COVID-19 pandemic." Infect Control Hosp Epidemiol: 1-20.

OBJECTIVE: The novel SARS-CoV-2 virus was first reported in Wuhan, China in December 2019 and is notable for being highly contagious and potentially lethal and is mainly spread by droplet transmission. The US healthcare system's response to the COVID-19 pandemic has been challenged by a shortage of personal protective equipment (PPE), especially N95 respirators. Restricted use, re-use, and sanitation of PPE have been widely adopted to provide protection for frontline healthcare workers caring for often critically ill and highly contagious patients. This objective of this manuscript is to describe our validated process for N95 respirator sanitation. DESIGN: Process development, validation, and implementation. SETTING: Level-I urban academic medical center. METHODS: A multidisciplinary team developed a novel evidence-based process for N95 respirator re-processing and sanitation using ultraviolet (UV) light. Dose measurement, structural integrity, moisture content, particle filtration, fit testing, and environmental testing were performed for both quality control and validation of the process. RESULTS: The process achieved UV light dosing for sanitation while maintaining the functional and structural integrity of the N95 respirators, with a daily potential throughput capacity of ˜12,000 masks. This process has supported our health system to provide respiratory PPE to all frontline team members. CONCLUSIONS: This novel method of N95 respirator sanitation can safely enable re-use of the N95 respirator essential for healthcare workers caring for patients with COVID-19. Our high-throughput process can extend local supplies of this critical PPE until the national supply is replenished.

Gomes, B. A., et al. (2020). "In-House Three-Dimensional Printing Workflow for Face Shield During COVID-19 Pandemic." J Craniofac Surg **31**(6): e652-e653.

From the beginning of the year 2020, the world was affected by a coronavirus disease (COVID-19) pandemic, caused by SARS-CoV-2, leading to a shortage of personal protective equipment (PPE) at a global level, and thus generating exposure of health professionals to this extremely contagious virus. Within this context, the present work seeks to present an alternative for the production of face shields "face shields," in which it recommends its production "in house" through 3D printing, in principle initiated by Prusa Research, where we download your project of support of facial protectors, proceeding with printing through the 3D printer Gtmax3D Core H5. The authors produced a face shield in ABS, in a total time of 3 hours and 44 minutes. Thus, the model presented proved to be feasible, at a low cost, adding to the list of possibilities to produce inputs necessary to maintain the fight against this epidemic.

Gómez-Beltrán, P. A., et al. (2021). "Recommendations for the management of the patient with chronic renal disease with SARS-CoV-2." Enferm Clin **31 Suppl 1**: S55-s61.

The SARS-CoV-2 pandemic, which affects the entire population in general, poses a special risk for patients with chronic kidney disease because of their immunosuppression status, advanced age and the coexistence of significant comorbidity. Renal patients undergoing hemodialysis run the risk not only of suffering but also of transmitting and spreading COVID-19 due to multiple factors, both procedural and logistical, associated with the process. These include the long duration of the technique, collective transfer to and from the centres, and the waiting time before performing the technique, aspects that make strict isolation difficult and therefore increase not only the risk of contagion between patients but also between specialist health professionals, which makes it more difficult to replace professionals. Prevention, protection, detection, isolation and distribution measures are essential in the management of the epidemic and must be taken from the initial stage. For this to be possible, it is necessary to draw up protocols to guide the actions of both the patient and the professionals in the different stages of chronic renal disease.

eng

Gómez-Salgado, J., et al. (2021). "Work engagement and psychological distress of health professionals during the COVID-19 pandemic." J Nurs Manag.

AIMS: To describe the level of work engagement of active health care professionals during the COVID-19 pandemic, and its relationship with psychological distress according to the professional category. BACKGROUND: Health care professionals working on the front line of the COVID-19 pandemic are at risk of psychological distress, and work engagement could be a positive attitude that could serve as a protective factor. METHODS: Cross-sectional observational study of 1,459 health care professionals. Psychological distress was measured with the General Health Questionnaire and work engagement with the Utrecht Work Engagement Scale. Data were analysed with bivariate analyses and correlations. RESULTS: Psychological distress was reported by 80.6% of health care professionals. Work engagement as high with a total mean score of 5.04 (SD = 1.14). The results showed that distressed professionals showed significantly lower levels of work engagement. CONCLUSIONS: The present study identified psychological distress and work engagement experienced by health care professionals during the COVID-19 pandemic. Most of the variables included in the study revealed a significant relationship with psychological distress and work engagement. IMPLICATIONS FOR NURSING MANAGEMENT: The relationship between the working conditions with psychological distress and work engagement suggests that improvements in the workplace are needed to promote protective measure for the mental health of health care professionals.

Gong, K., et al. (2020). "Internet Hospitals Help Prevent and Control the Epidemic of COVID-19 in China: Multicenter User Profiling Study." J Med Internet Res **22**(4): e18908.

BACKGROUND: During the spread of the novel coronavirus disease (COVID-19), internet hospitals in China were engaged with epidemic prevention and control, offering epidemic-related online services and medical support to the public. OBJECTIVE: The aim of this study is to explore the role of internet hospitals during the prevention and control of the COVID-19 outbreak in China. METHODS: Online epidemic-related consultations from multicenter internet hospitals in China during the COVID-19 epidemic were collected. The counselees were described and classified into seven type groups. Symptoms were recorded and compared with reported patients with COVID-19. Hypochondriacal suspicion and offline visit motivation were detected within each counselees' group to evaluate the social panic of the epidemic along with the consequent medical-seeking behaviors. The counselees' motivation and the doctors' recommendation for an offline visit were compared. Risk factors affecting the counselees' tendency of hypochondriacal suspicion and offline visit motivation were explored by logistic regression models. The epidemic prevention and control measures based on internet hospitals were listed, and the corresponding effects were discussed. RESULTS: A total of 4913 consultations were enrolled for analysis with the median age of the counselees at 28 years (IQR 22-33 years). There were 104 (2.12%) healthy counselees, 147 (2.99%) hypochondriacal counselees, 34 (0.69%) exposed counselees, 853 (17.36%) mildly suspicious counselees, 42 (0.85%) moderately suspicious counselees, 3550 (72.26%) highly suspicious counselees, and 183 (3.72%) severely suspicious counselees. A total of 94.20% (n=4628) of counselees had epidemic-related symptoms with a distribution similar to those of COVID-19. The hypochondriacal suspicion (n=2167, 44.11%) was common. The counselees' motivation and the doctors' recommendation for offline visits were inconsistent (P<.001) with a Cohen kappa score of 0.039, indicating improper medical-seeking behaviors. Adult counselees (odds ratio [OR]=1.816, P<.001) with epidemiological exposure (OR 7.568, P<.001), shortness of breath (OR 1.440, P=.001), diarrhea (OR 1.272, P=.04), and unrelated symptoms (OR 1.509, P<.001) were more likely to have hypochondriacal suspicion. Counselees with severe illnesses (OR 2.303, P<.001), fever (OR 1.660, P<.001), epidemiological exposure history (OR 1.440, P=.01), and hypochondriacal suspicion (OR 4.826, P<.001) were more likely to attempt an offline visit. Reattending counselees (OR 0.545, P=.002) were less motivated to go to the offline clinic. CONCLUSIONS: Internet hospitals can serve different types of epidemic counselees, offer essential medical supports to the public during the COVID-19 outbreak, reduce the social panic, promote social distancing, enhance the public's ability of self-protection, correct improper medical-seeking behaviors, reduce the chance of nosocomial cross-infection, and facilitate epidemiological screening, thus, playing an important role on preventing and controlling COVID-19.

Gonzi, G., et al. (2020). "Trauma surgery at a designated COVID-19-free site during the pandemic: a safe model and a possible way to restart routine elective surgery." Bone Jt Open **1**(6): 302-308.

AIMS: Elective operating was halted during the COVID-19 pandemic to increase the capacity to provide care to an unprecedented volume of critically unwell patients. During the pandemic, the orthopaedic department at the Aneurin Bevan University Health Board restructured the trauma service, relocating semi-urgent ambulatory trauma operating to the isolated clean elective centre (St. Woolos' Hospital) from the main hospital receiving COVID-19 patients (Royal Gwent Hospital). This study presents our experience of providing semi-urgent trauma care in a COVID-19-free surgical unit as a safe way to treat trauma patients during the pandemic and a potential model for restarting an elective orthopaedic service. METHODS: All patients undergoing surgery during the COVID-19 pandemic at the orthopaedic surgical unit (OSU) in St. Woolos' Hospital from 23 March 2020 to 24 April 2020 were included. All patients that were operated on had a telephone follow-up two weeks after surgery to assess if they had experienced COVID-19 symptoms or had been tested for COVID-19. The nature of admission, operative details, and patient demographics were obtained from the health board's electronic record. Staff were assessed for sickness, self-isolation, and COVID-19 status. RESULTS: A total of 58 surgical procedures were undertaken at the OSU during the study period; 93% (n = 54) of patients completed the telephone follow-up. Open reduction and internal fixation of ankle and wrist fractures were the most common procedures. None of the patients nor members of their households had developed symptoms suggestive of COVID-19 or required testing. No staff members reported sick days or were advised by occupational health to undergo viral testing. CONCLUSION: This study provides optimism that orthopaedic patients planned for surgery can be protected from COVID-19 nosocomial transmission at separate COVID-19-free sites.Cite this article: Bone Joint Open 2020;1-6:302-308.

Gou, S. M., et al. (2020). "[Treatment of pancreatic diseases and prevention of infection during outbreak of 2019 coronavirus disease]." Zhonghua Wai Ke Za Zhi **58**(5): 326-330.

Objective: To explore the proper protective measures for pancreatic diseases treatment during the outbreak of 2019 coronavirus disease(COVID-19). Methods: Clinical data of four cases of patients that suffered COVID-19 from February 2(nd) to February 9(th), 2020 at Department of Pancreatic Surgery, Union Hospital, Tongji Medical College, Huazhong University of Science and Technology were reviewed induding 4 males and 1 female, aging of 50, 51, 46, 87 years old, respectively. After the first patients cuffed nosocomial infection of COVID-19, the general protective measures were updated.Only one patient was admitted to each room alone, with no more than one caregiver. The body temperature of care givers was measured twice a day.Primary protections were applied to all staff.The floor was sterilized using disinfectant with an effective chlorine concentration of 1 000 mg/L. The protective measures for interventional procedures were as follow. Primary protection was applied to the operators of central venipuncture catheter, percutaneous abdominal/pleural drainage, percutaneous retroperitoneal drainage, percutaneous transhepatic cholangial drainage and other surgical procedures with local anesthesia and epidural anesthesia. Secondary protection was applied to the operators of endoscopic retrograde cholangiopancreatography and surgical procedures with general anesthesia. Results: There were four patients who were diagnosed as COVID-19, of which one died of COVID-19, two were cured, and one was still in hospital for COVID-19. After the update of protective measures, no more nosocomial infection of COVID-19 occurred. Two central venipuncture catheter, three percutaneous abdominal or pleural drainage, one percutaneous retroperitoneal drainage, one percuteneous transhepatic cholecyst drainage and one open surgery with general anesthesia were performed with no infection of operators. Conclusions: The caregivers of patients are potential infection source of COVID-19. Enhanced protective measures including the management measures of caregivers can decrease the risk of nosocomial infection of COVID-19.

Goulart, C. D. L., et al. (2021). "Lifestle and rehabilitation during the COVID-19 pandemic: guidance for health professionals and support for exercise and rehabilitation programs." Expert Rev Anti Infect Ther.

INTRODUCTION: The coronavirus disease 2019 (COVID-19) is a highly contagious respiratory viral disease for both the general population and health care professionals caring for infected patients. Of particular concern is the potential for significant respiratory, cardiovascular, physical, and psychological dysfunctions. AREAS COVERED: In this context, the current review will focus on the following areas: 1) staying physically active during the COVID-19 pandemic; 2) highlighting the importance of understanding COVID-19 mechanisms; 3) preventing infections for healthcare workers by using personal protective equipment; 4) highlighting importance of respiratory care and physical therapy during hospitalization in patients with COVID-19; and 5) facilitating referral to a rehabilitation program in patients recovering from COVID-19. EXPERT OPINION: We recommend daily physical exercise, outdoors or at home, as physical exercise increases the synthesis of anti-inflammatory cytokines; Patients with COVID-19 may develop severe acute respiratory syndrome, hypoxemia, diffuse alveolar damage, ACE2 reduction in the cardiovascular system and muscle weakness acquired through a prolonged hospital stay; The role of the physiotherapist in the hospital environment is of fundamental importance-early mobilization is highly recommended in severe cases of COVID-19.

Grau-Pujol, B., et al. (2020). "Pre-exposure prophylaxis with hydroxychloroquine for high-risk healthcare workers during the COVID-19 pandemic: A structured summary of a study protocol for a multicentre, double-blind randomized controlled trial." Trials **21**(1): 688.

OBJECTIVES: The aim of this study is to assess the efficacy of the use of pre-exposure prophylaxis (PrEP) with hydroxychloroquine against placebo in healthcare workers with high risk of severe acute respiratory syndrome coronavirus 2 (SARS-CoV-2) infection in reducing their risk of coronavirus disease 2019 (COVID-19) disease during an epidemic period. As secondary objectives, we would like to: i) assess the efficacy of the use of PrEP with hydroxychloroquine against placebo in healthcare workers with high risk of SARS-CoV-2 infection in reducing their risk of exposure to SARS-CoV-2 (defined by seroconversion) during an epidemic period, ii) evaluate the safety of PrEP with hydroxychloroquine in adults, iii) describe the incidence of SARS-CoV-2 infection among healthcare workers at high risk of SARS-CoV-2 infection, iv) identify clinical, analytical and microbiological predictors of COVID-19 among healthcare workers at high risk of SARS-CoV-2 infection, v) set up a repository of serum samples obtained from healthcare workers at high risk of SARS-CoV-2 infection for future research on blood markers to predict SARS-CoV-2 infection. TRIAL DESIGN: Multicentre double-blind parallel design (ratio 1:1) randomized controlled clinical trial. PARTICIPANTS: Approximately 440 healthcare workers of four Spanish hospitals (Hospital Clínic of Barcelona, Hospital de la Santa Creu i Sant Pau of Barcelona, Hospital Plató of Barcelona, Hospital General de Granollers, Barcelona) will be recruited. Participants are considered to be at high-risk of SARS-CoV-2 infection due to their frequent contact with suspected and confirmed cases of COVID-19. For eligibility, healthcare workers with 18 years old or older working at least 3 days a week in a hospital with both negative SARS-CoV-2 polymerase chain reaction (PCR) assays and serological COVID-19 rapid diagnostic tests (RDT) are invited to participate. Participants with any of the following conditions are excluded: pregnancy, breastfeeding, ongoing antiviral, antiretroviral or corticosteroids treatment, chloroquine or hydroxychloroquine uptake the last month or any contraindication to hydroxychloroquine treatment. INTERVENTION AND COMPARATOR: Eligible participants will be allocated to one of the two study groups: Intervention group (PrEP): participants will receive the standard of care and will take 400mg of hydroxychloroquine (2 tablets of 200 mg per Dolquine® tablet) daily the first four consecutive days, followed by 400 mg weekly for a period of 6 months. CONTROL GROUP: participants will receive placebo tablets with identical physical appearance to hydroxychloroquine 200 mg (Dolquine®) tablets following the same treatment schedule of the intervention group. Both groups will be encouraged to use the personal protection equipment (PPE) for COVID-19 prevention according to current hospital guidelines. MAIN OUTCOMES: The primary endpoint will be the number of confirmed cases of a COVID-19 (defined by a positive PCR for SARS-CoV-2 or symptoms compatible with COVID-19 with seroconversion) in the PrEP group compared to the placebo group at any time during the 6 months of the follow-up in healthcare workers with negative SARS-CoV-2 PCR and serology at day 0. As secondary endpoints, we will obtain: i) the SARS-CoV-2 seroconversion in the PrEP group compared to placebo during the 6 months of follow-up in healthcare workers with negative serology at day 0; ii) the occurrence of any adverse event related with hydroxychloroquine treatment; iii) the incidence of SARS-CoV-2 infection and COVID-19 among healthcare workers in the non-PrEP group, among the total of healthcare workers included in the non-PrEP group during the study period; iv) the risk ratio for the different clinical, analytical and microbiological conditions to develop COVID-19; v) a repository of serum samples obtained from healthcare workers confirmed COVID-19 cases for future research on blood markers to predict SARS-CoV-2 infection. RANDOMISATION: Participants meeting all eligibility requirements will be allocated to one of the two study arms (PrEP with hydroxychloroquine or non-PrEP control group) in a 1:1 ratio using simple randomisation with computer generated random numbers. BLINDING (MASKING): Participants, doctors and nurses caring for participants, and investigators assessing the outcomes will be blinded to group assignment. NUMBERS TO BE RANDOMISED (SAMPLE SIZE): Each intervention group will have 220 participants, giving a total of 440 participants. TRIAL STATUS: The current protocol version is 1.5, 2(nd) of June 2020. Two hundred and seventy-fiveparticipants were recruited and completed first month follow-up until date. The estimated sample size could not be reached yet due to the declining national epidemic curve. Thus, 275 is the total number of participants included until date. The study has been suspended (26(th) of June) until new epidemic curve occurs. TRIAL REGISTRATION: This trial was registered on April 2(nd) 2020 at clinicaltrials.gov with the number NCT04331834. FULL PROTOCOL: The full protocol is attached as an additional file, accessible from the Trials website (Additional file 1). In the interest in expediting dissemination of this material, the familiar formatting has been eliminated; this Letter serves as a summary of the key elements of the full protocol.

Greenberg, N., et al. (2020). "Managing mental health challenges faced by healthcare workers during covid-19 pandemic." Bmj **368**: m1211.

Greffe, S., et al. (2020). "[Nasopharyngeal carriage of SARS-CoV-2 among health personnel with symptoms suggestive of COVID-19 in a University Hospital in the Paris suburbs]." Rev Med Interne **41**(8): 510-516.

INTRODUCTION: A consultation dedicated to symptomatic health professionals was opened at the beginning of the COVID-19 epidemic in order to meet the specific needs of this population. The objective of this work was to estimate the frequency of SARS-Cov-2 nasopharyngeal carriage in symptomatic healthcare workers suspected of having COVID-19 and to determine the factors associated with this carriage. METHODS: Of the 522 consultants, 308 worked in the Hospital and 214 outside. They had mild forms of COVID-19 and non-specific clinical signs with the exception of agueusia/anosmia, which was significantly more common in those with positive RT-PCR. The rate of RT-PCR positivity was 38% overall, without significant difference according to profession. It was higher among external consultants (47% versus 31%). In the hospital, this rate was significantly lower for symptomatic staff in the care sectors, compared to staff in the technical platforms and laboratories (24%, versus 45%, p = 0.006 and 54%, respectively, p < 0.001), but did not differ between staff in COVID units and other care sectors (30% versus 28%). Among the external consultants, the positivity rates of nursing home and private practices staff (53% and 55% respectively) were more than double that of acute care hospital staff (24%, p < 0.001). CONCLUSIONS: These data confirm the strong impact of COVID-19 on health professionals. The higher positivity rates among symptomatic professionals working outside the hospital compared to those working in hospital may be explained in part by a shortage of protective equipment and by difficulties in accessing virological diagnosis, which were greater outside the hospital when the epidemic began.

Publisher: Abstract available from the publisher.

fre

Greiner, J., et al. (2021). "Learning from the first wave: Lessons about managing patient flow and resource utilization on medical wards at providence health during the COVID-19 pandemic." Healthc (Amst) **9**(2): 100530.

We report the successful implementation of a modified Traffic Control Bundling (TCB) protocol called "Red, Yellow and Green" on the inpatient medical units at St. Paul's Hospital in Vancouver, Canada during the first wave of the coronavirus disease 2019 (COVID-19) pandemic. The modified TCB protocol demonstrates an important example on how hospitals can rapidly reorganize operational and clinical processes to reallocate existing capacity to minimize exposure, improve traffic flow and reduce nosocomial transmissions of COVID-19 to health care workers (HCWs) and other patients. Preliminary evidence demonstrates the benefits on how an existing facility can be redesigned for adjustable ward capacity to provide disease containment under a context of uncertainty of disease transmission and varying patient load. Important lessons in preparation for the evolution of the pandemic fall into categories of risk management, capacity and demand management.

Griswold, D., et al. (2021). "Chest CT and rapid antigen testing for diagnosis of COVID-19 in emergency trauma surgery patients that require urgent care during the pandemic: an umbrella review protocol." JMIR Res Protoc.

BACKGROUND: Many healthcare facilities in low-and middle-income countries are inadequately resourced. COVID-19 has the potential to decimate surgical healthcare services unless health systems take stringent measures to protect healthcare workers (HCWs) from viral exposure and ensure the continuity of specialized care for the patients. Among these measures, timely diagnosis of COVID-19 is paramount to ensure protective measures use and isolation of patients that prevent spread of the infection to healthcare personnel caring for patients with unknown COVID-19 status or contact during the pandemic. Besides molecular and antibody tests, chest CT has been studied as a potential tool to aid in the screening or diagnosis of COVID-19 and could be valuable in the emergency setting. The purpose of the review is to inform future recommendations regarding emergency care of trauma surgery patients. OBJECTIVE: This umbrella review aims to identify and summarize the available literature regarding the diagnostic accuracy of chest CT for COVID-19 in trauma surgery patients requiring urgent care. METHODS: We will conduct several searches in the L·OVE (Living OVerview of Evidence) platform for COVID-19, a system that performs automated regular searches in PubMed, Embase, Cochrane Central Register of Controlled Trials (CENTRAL), and over thirty other sources. The search results will be presented according to the PRISMA flow diagram. This review will preferentially consider systematic reviews of diagnostic test accuracy studies, as well as individual studies of such design if not included in systematic reviews, that assessed the sensitivity and specificity of chest CT in emergency trauma surgery patients. Critical appraisal of the included studies for risk of bias will be conducted. Data will be extracted using a standardized data extraction tool. Findings will be summarised narratively and the Grading of Recommendations, Assessment, Development, and Evaluation (GRADE) approach for grading the certainty of evidence will be reported. RESULTS: Ethics approval is not required for this systematic review, as there will be no patient involvement. The search for this systematic review commenced in October 2020, and we expect to publish the findings in early 2021. The plan for dissemination is to publish review findings in a peer-reviewed journal and present findings at conferences that engage the most pertinent stakeholders. CONCLUSIONS: During the COVID-19 pandemic, protecting HCWs from infection is essential. Up-to-date information on diagnostic tests efficacy for detecting COVID-19 is essential. This review will serve an important role as a thorough summary to inform evidence-based recommendations regarding with the purpose of setting effective policy and clinical guideline recommendations. CLINICALTRIAL: International Prospective Register of Systematic Reviews (PROSPERO) CRD42020198267; https://www.crd.york.ac.uk/PROSPERO/display_record.php?RecordID=198267. INTERNATIONAL REGISTERED REPORT: PRR1-10.2196/25207.

Griswold, D. P., et al. (2021). "Personal protective equipment for reducing the risk of COVID-19 infection among health care workers involved in emergency trauma surgery during the pandemic: An umbrella review." J Trauma Acute Care Surg **90**(4): e72-e80.

BACKGROUND: Health care facilities in low- and middle-income countries are inadequately resourced to adhere to current COVID-19 prevention recommendations. Recommendations for surgical emergency trauma care measures need to be adequately informed by available evidence and adapt to particular settings. To inform future recommendations, we set to summarize the effects of different personal protective equipment (PPE) on the risk of COVID-19 infection in health personnel caring for trauma surgery patients. METHODS: We conducted an umbrella review using Living Overview of Evidence platform for COVID-19, which performs regular automated searches in MEDLINE, Embase, Cochrane Central Register of Controlled Trials, and more than 30 other sources. Systematic reviews of experimental and observational studies assessing the efficacy of PPE were included. Indirect evidence from other health care settings was also considered. Risk of bias was assessed with the AMSTAR II tool (Assessing the Methodological Quality of Systematic Reviews, Ottawa, ON, Canada), and the Grading of Recommendations, Assessment, Development, and Evaluation approach for grading the certainty of the evidence is reported (registered in International Prospective Register of Systematic Reviews, CRD42020198267). RESULTS: Eighteen studies that fulfilled the selection criteria were included. There is high certainty that the use of N95 respirators and surgical masks is associated with a reduced risk of COVID-19 when compared with no mask use. In moderate- to high-risk environments, N95 respirators are associated with a further reduction in risk of COVID-19 infection compared with surgical masks. Eye protection also reduces the risk of contagion in this setting. Decontamination of masks and respirators with ultraviolet germicidal irradiation, vaporous hydrogen peroxide, or dry heat is effective and does not affect PPE performance or fit. CONCLUSION: The use of PPE drastically reduces the risk of COVID-19 compared with no mask use in health care workers. N95 and equivalent respirators provide more protection than surgical masks. Decontamination and reuse appear feasible to overcome PPE shortages and enhance the allocation of limited resources. These effects are applicable to emergency trauma care and should inform future recommendations. LEVEL OF EVIDENCE: Review, level II.

Griswold, D. P., et al. (2021). "Personal protective equipment for reducing the risk of COVID-19 infection among healthcare workers involved in emergency trauma surgery during the pandemic: an umbrella review protocol." BMJ Open **11**(3): e045598.

INTRODUCTION: Many healthcare facilities in low-income and middle-income countries are inadequately resourced and may lack optimal organisation and governance, especially concerning surgical health systems. COVID-19 has the potential to decimate these already strained surgical healthcare services unless health systems take stringent measures to protect healthcare workers (HCWs) from viral exposure and ensure the continuity of specialised care for patients. The objective of this broad evidence synthesis is to identify and summarise the available literature regarding the efficacy of different personal protective equipment (PPE) in reducing the risk of COVID-19 infection in health personnel caring for patients undergoing trauma surgery in low-resource environments. METHODS: We will conduct several searches in the L·OVE (Living OVerview of Evidence) platform for COVID-19, a system that performs automated regular searches in PubMed, Embase, Cochrane Central Register of Controlled Trials and over 30 other sources. The search results will be presented according to the Preferred Reporting Items for Systematic Reviews and Meta-Analyses flow diagram. This review will preferentially consider systematic reviews of experimental and quasi-experimental studies, as well as individual studies of such designs, evaluating the effect of different PPE on the risk of COVID-19 infection in HCWs involved in emergency trauma surgery. Critical appraisal of eligible studies for methodological quality will be conducted. Data will be extracted using the standardised data extraction tool in Covidence. Studies will, when possible, be pooled in a statistical meta-analysis using JBI SUMARI. The Grading of Recommendations, Assessment, Development and Evaluation approach for grading the certainty of evidence will be followed and a summary of findings will be created. ETHICS AND DISSEMINATION: Ethical approval is not required for this review. The plan for dissemination is to publish review findings in a peer-reviewed journal and present findings at high-level conferences that engage the most pertinent stakeholders. PROSPERO REGISTRATION NUMBER: CRD42020198267.

Gross, J. V., et al. (2021). "COVID-19 and healthcare workers: a rapid systematic review into risks and preventive measures." BMJ Open **11**(1): e042270.

OBJECTIVE: The COVID-19 pandemic is demanding for occupational medicine and for public health. As healthcare workers (HCWs) fight impacts of SARS-CoV-2 on front lines, we must create safe work environments through comprehensive risk assessments, evaluation and effective implementation of counter-measures. We ask: 'What does current literature report on health risks at workplaces regarding COVID-19?' and 'What do current studies report on the effectiveness of enacted preventative recommendations?' METHODS: As a snapshot of early HCW research, on 26 April 2020, we conducted a rapid systematic literature search in three databases (PubMed, Web of Science and PsycInfo) for COVID-19-related health outcomes and preventive measures in healthcare-associated workplaces. RESULTS: 27 studies were identified as relevant for exploring the risk of infection, 11 studies evaluated preventive measures. The studies described that SARS-CoV-2 impacts significantly on HCW's health and well-being, not only through infections (n=6), but also from a mental health perspective (n=16). 4 studies reported indirect risks such as skin injuries, one study described headaches to result from the use of personal protective equipment. Few studies provided information on the effectiveness of prevention strategies. Overall, most studies on health risks as well as on the effectiveness of preventive measures were of a moderate-to-low quality; this was mainly due to limitations in study design, imprecise exposure and outcome assessments. CONCLUSIONS: Due to widespread exposure of HCW to SARS-CoV-2, workplaces in healthcare must be as safe as possible. Information from HCW can provide valuable insights into how infections spread, into direct and indirect health effects and into how effectively counter-measures mitigate adverse health outcomes. However, available research disallows to judge which counter-measure(s) of a current 'mix' should be prioritised for HCW. To arrive at evidence-based cost-effective prevention strategies, more well-conceived studies on the effectiveness of counter-measures are needed.

Grossheim, L., et al. (2021). "Cancer and COVID-19 Experiences at African Cancer Centers: The Silver Lining." JCO Glob Oncol **7**: 410-415.

PURPOSE: The COVID-19 pandemic significantly disrupted cancer care in Africa, further exposing major health disparities. This paper compares and contrasts the experiences of 15 clinicians in six different African cancer centers to highlight the positive aspects (silver linings) in an otherwise negative situation. METHODS: Data are from personal experience of the clinicians working at the six cancer centers blended with what is available in the literature. RESULTS: The impact of COVID-19 on cancer care appeared to vary not only across the continent but also over cancer centers. Different factors such as clinic location, services offered, available resources, and level of restrictions imposed because of COVID-19 were associated with these variations. Collectively, delays in treatment and limited access to cancer care were commonly reported in the different regions. CONCLUSION: There is a lack of data on cancer patients with COVID-19 and online COVID-19 and cancer registries for Africa. Analysis of the available data, however, suggests a higher mortality rate for cancer patients with COVID-19 compared with those without cancer. Positive or silver linings coming out of the pandemic include the adoption of hypofractionated radiation therapy and teleoncology to enhance access to care while protecting patients and staff members. Increasing collaborations using online technology with oncology health professionals across the world are also being seen as a silver lining, with valuable sharing of experiences and expertise to improve care, enhance learning, and reduce disparities. Advanced information and communication technologies are seen as vital for such collaborations and could avail efforts in dealing with the ongoing pandemic and potential future crises.

Grossman, J., et al. (2020). "Institution of a Novel Process for N95 Respirator Disinfection with Vaporized Hydrogen Peroxide in the Setting of the COVID-19 Pandemic at a Large Academic Medical Center." J Am Coll Surg **231**(2): 275-280.

Personal protective equipment (PPE) has been an invaluable yet limited resource when it comes to protecting healthcare workers against infection during the 2019 coronavirus (COVID-19) pandemic. In the US, N95 respirator supply chains are severely strained and conservation strategies are needed. A multidisciplinary team at the Washington University School of Medicine, Barnes Jewish Hospital, and BJC Healthcare was formed to implement a program to disinfect N95 respirators. The process described extends the life of N95 respirators using vaporized hydrogen peroxide (VHP) disinfection and allows healthcare workers to retain their own N95 respirator across a large metropolitan healthcare system.

Grosso, A., et al. (2020). "Suppression of Covid-19 outbreak among healthcare workers at the Treviso Regional Hospital, Italy and lessons for ophthalmologists." Eur J Ophthalmol: 1120672120982520.

PURPOSE: To describe a strategy to reduce Covid-19 spread among healthcare workers and provide ophthalmologists with recommendations useful for a possible second wave of Covid-19 in Autumn. METHODS: Epidemiological surveillance at the Cà Foncello Hospital (Veneto, Italy) since 24 February 2020 to 24 April 2020 when the municipality of Treviso was hit by the Covid-19 outbreak. The number of naso-pharigeal (NP) swabs performed was 7010. RESULTS: The number of infected among healthcare workers was 209/ 3924 (5.32%): medical doctors: 28 cases / 498 (5.6%). None among ophthalmologists; specialized nurses: 86/1294 (6.4%) None in the ophthalmic unit; intermediate care technicians: 68/463 (14.7%). The 46% of the positive tested were asymptomatic. We share key suggested actions for the reorganization in ophthalmological services: be part of a global epidemiological local strategy of containment (Testing, Tracing, Treating); protect your department: Keep on screening patients by telephone interview before entering the hospital; promote continuous and appropriate use of PPE both for doctors and for patients; make any effort to obtain a continuous flow of patients in every line of the ophthalmic service; treat appropriately any single patient with vision threatening condition; avoid unnecessary or futile testings and examinations. CONCLUSION: The Treviso model shows that it is possible and safe to keep on performing high risk hospital activities like ophthalmology, even in the epicenter of covid outbreak, if adequate actions are performed. We discuss about the value of NP swabs and serological tests as a strategy in case of a second wave of infections.

Güler, M. G. and E. Geçici (2020). "A decision support system for scheduling the shifts of physicians during COVID-19 pandemic." Comput Ind Eng **150**: 106874.

The rapidly spreading COVID-19 pandemic has affected many people worldwide. Due to the high infectivity, countries make calls to stay at home or take measures such as lockdowns to ensure that people are least affected by the virus. Meanwhile, infected people are getting treatments: people who are slightly affected are quarantined at home, and those who are heavily affected are treated in hospitals. Hence there is an excessive increase in the hospital workload. This causes physical fatigue in healthcare professionals. Along with the increasing workload, the fear of being infected and infecting the environment causes psychological problems in healthcare professionals. It is important to protect healthcare professionals and provide them with suitable working conditions. For this reason, besides the provision of protective equipment such as gloves, overalls, mask, and glasses that are necessary for the protection of healthcare workers from the virus, healthcare services should also be planned very carefully. One of the critical issues is planning the shift schedules of the physicians. In this study, we handle the preparation of a physician shift schedule of a hospital in Turkey during the COVID-19 pandemic. The hospital has established three new COVID-19 related departments and the aim is to provide continuous service in the new departments while maintaining the workload in the existing departments. We propose a mixed integer programming (MIP) model to address the shift scheduling problem and transform it into a decision support system (DSS). The resulting schedules minimize the exposure of the physicians to the virus with a balanced workload while maintaining the healthcare service in all departments.

Gupta, S., et al. (2020). "Survey of prevalence of anxiety and depressive symptoms among 1124 healthcare workers during the coronavirus disease 2019 pandemic across India." Med J Armed Forces India.

BACKGROUND: A prospective study was conducted during the second phase of the coronavirus disease 2019 (COVID-19) pandemic in India to assess the prevalence of anxiety and depressive symptoms among healthcare workers (HCWs) and factors that influence the outcome. METHODS: A self-administered questionnaire was completed by 1124 HCWs during the COVID-19 pandemic (March 30, 2020, to April 2, 2020). Demographic data, questions on COVID-19 and scores of the Hospital Anxiety and Depression Scale were analysed using the chi-square test (Bonferroni correction) and binary logistic regression. RESULTS: The study consists of 1124 HCWs, including 749 doctors, 207 nurses, 135 paramedics, 23 administrators and ten supporting staff members. The prevalence of anxiety and depressive symptoms were reported as 37.2% and 31.4%, respectively. The risk factors for anxiety were female gender (30.6% vs 45.5%), age group (20-35 years) (50.4% vs 61.2%), unmarried (21.2% vs 30.6%) and job profile (nurse) (14.7% vs 26.4%). The protective factor was having service of more than 20 years (23.4% vs 14.8%). The risk factors for depression were age group (20-35 years) (51.3% vs 61.3%) and employed at a primary care hospital (16.2% vs 23.4%). The protective factors were job profile (doctor) (69.9% vs 59.6%) and having service of more than 20 years (22.3% vs 15.5%). CONCLUSION: Approximately one-third of the HCWs reported anxiety and depressive symptoms. The risk factors for anxiety symptoms were female gender, younger age and job profile (nurse) and for depressive symptoms were younger age and working at a primary care hospital. Future research studies should identify strategies for providing a safer and supportive work environment for HCWs to face epidemics/pandemics.

Gupta, S., et al. (2020). "Proposal for the return to routine endoscopy during the COVID-19 pandemic." Gastrointest Endosc **92**(3): 735-742.

In response to the coronavirus disease 2019 (COVID-19) pandemic, many jurisdictions and gastroenterological societies around the world have suspended nonurgent endoscopy. Subject to country-specific variability, it is projected that with current mitigation measures in place, the peak incidence of active COVID-19 infections may be delayed by over 6 months. Although this aims to prevent the overburdening of healthcare systems, prolonged deferral of elective endoscopy will become unsustainable. Herein, we propose that by incorporating readily available point-of-care tests and conducting accurate clinical risk assessments, a safe and timely return to elective endoscopy is feasible. Our algorithm not only focuses on the safety of patients and healthcare workers, but also assists in rationalizing the use of invaluable resources such as personal protective equipment.

Gurzawska-Comis, K., et al. (2020). "Recommendations for Dental Care during COVID-19 Pandemic." J Clin Med **9**(6).

Dental clinics were suspected to be a hotspot for nosocomial transmission of coronavirus disease 19 (COVID-19), yet there has been no clear recommendation about emergency dental care and appropriate personal protective equipment during pandemics. In this paper, we aim to summarize recommendations for (i) patient risk assessment, (ii) patient triage, and (iii) measures to prevent infection of health professionals and nosocomial transmission in dental clinics. The available evidence was collected by performing searches on PubMed, Embase, and Cochrane databases. We reviewed papers on COVID-19, severe acute respiratory syndrome (SARS), Middle East respiratory syndrome (MERS), influenza, and related respiratory viral diseases. Legal and ethical frameworks, as well as international (e.g., World Health Organization (WHO)) and national (e.g., public health institutes, dental associations) guidelines were screened to summarize recommendations related to dental emergency care. To assess the patient risk, a questionnaire was developed to classify patients at unknown, high, and very high risk. Patient triage recommendations were summarized in a flow chart that graded the emergency level of treatments (i.e., urgent, as soon as possible, and postpone). Measures to prevent disease transmission based on current evidence were grouped for dental health professionals, dental clinics, and patients. The present recommendations may support health professionals implement preventative measures during the pandemic.

Ha, J. F. (2020). "The COVID-19 pandemic, personal protective equipment and respirator: A narrative review." Int J Clin Pract **74**(10): e13578.

INTRODUCTION: The coronavirus disease 2019 pandemic has touched almost every continent. Personal protective equipment (PPE) is the final line of protection of healthcare workers (HCW). There is variation as well as controversy of infection control recommendation with regards to the use of PPE for HCW between institutions. The aim of this narrative review is to of examine and summarise the available evidence to guide recommendation for the safety of HCW. METHOD: A literature search was conducted on the PubMed, MedLine and Embase databases with the keywords "personal protective equipment," "COVID 19," "n95," "health care worker" and "mortality." RESULTS: SARS-nCoV-2 is highly contagious. About 3.5%-20% of HCW has been reported to be infected. The mortality ranges from 0.53% to 1.94%. PPE is part of the measure within a package of prevention and control of pandemic, rather than a replacement of. Respirators are more effective than masks in preventing aerosol transmission to HCWs. Extended use may be considered if guidelines are adhered. Powered air-purifying respirators if available should be used in high-risk procedures. CONCLUSION: Transmission of viruses is multimodal and in the setting of a novel pathogen with high case fatality with no proven effective interventions, PPE that affords the best protection should be available to HCWs.

Hacimusalar, Y., et al. (2020). "Anxiety and hopelessness levels in COVID-19 pandemic: A comparative study of healthcare professionals and other community sample in Turkey." J Psychiatr Res **129**: 181-188.

COVID-19 affected our mental health as well as our physical health. In this study, the anxiety and hopelessness levels of healthcare workers and non-healthcare workers and the factors affecting them were evaluated in Turkey. Beck Hopelessness Scale and State-Trait Anxiety Inventory (STAI) was applied online to participants. Totally 2156 individuals were included in the study and 52.0% (n:1121) of them are healthcare workers. The hopelessness and state anxiety levels of healthcare workers were higher than non-healthcare workers. Nurses' hopelessness levels are higher than doctors, and state anxiety levels are higher than both doctors and other healthcare workers. Anxiety and hopelessness levels were higher in women, those living with a high-risk individual at home during the pandemic, those who had difficulty in caring for their children, and those whose income decreased. Anxiety levels are an important predictor of hopelessness. The increase in anxiety levels explained 28.9% of the increase in hopelessness levels. Increased working hours is one of the important factors affecting anxiety. As a conclusion, healthcare workers were more affected psychologically in the COVID-19 pandemic compared to the society. Nurses were affected more than other healthcare workers. It is important to identify the factors affecting anxiety, hopelessness, and individuals who may be more psychologically affected during the pandemic. An important contribution can be made to the protection of public health by ensuring that psychosocial interventions for high-risk groups are planned in advance.

Haedo, M. F., et al. (2020). "Usefulness of blood cultures in COVID-19 pneumonia." Medicina (B Aires) **80 Suppl 6**: 44-47.

The Coronavirus Disease 2019 (COVID-19) pandemic has strained the world's health systems, highlighting the need to optimize its clinical management and treatment. The usefulness of blood cultures in patients with COVID-19 pneumonia has not been proved. We aim to describe the diagnostic yield of early blood cultures in patients with COVID-19 pneumonia in a public hospital in Buenos Aires City. This descriptive observational study included all adult patients with COVID-19 pneumonia admitted to the Internal Medicine ward of Hospital Durand between April 1, 2020 and July 30, 2020, who had blood cultures drawn within 5 days from hospital admission. Among 267 patients hospitalized with COVID-19 pneumonia, 38 had early blood cultures drawn. No clinically relevant microorganism was isolated from blood and contaminant microorganisms were recovered in 7 (18.4%) patients. This study found no evidence of bacteremia in patients with COVID-19 pneumonia. Furthermore, the rate of contaminated blood cultures nearly doubles the reported in patients with community acquired pneumonia (10%), which may be explained by unfamiliarity of additional personal protective equipment worn by healthcare workers. Our results advocate against the routine indication of blood cultures upon admission to the Internal Medicine Ward of patients with COVID-19 pneumonia. We suggest that blood cultures could only be useful in case of clinical deterioration or suspected hospital-acquired infection.

Hailu, D., et al. (2021). "Occupational Health Safety of Health Professionals and Associated Factors During COVID-19 Pandemics at North Showa Zone, Oromia Regional State, Ethiopia." Risk Manag Healthc Policy **14**: 1299-1310.

BACKGROUND: Coronavirus disease 19 was observed as a pandemic and caused many community health problems that resulted in Global issues. It causes death for many individuals including health professionals. This study aimed to determine the occupational health safety of health professionals and associated factors during COVID-19 pandemic at North Showa. METHODS: Institutions-based Cross-sectional study was conducted using a simple random sampling technique from May 10 to June 15, 2020. Interviewer-administered questioners were used, and data were entered into Epi-data version 3.1 and exported to SPSS 23 for analysis. Bi-variable logistic regression was carried out to select candidate variables with a cutoff point < 0.2. Finally, multivariable logistic regression was conducted to identify significant variables. An adjusted odds ratio with 95% CI at a 5% level of significance was used to measure the strength of association. P-value <0.05 indicated a significant association between variables. RESULTS: A total of 280 health professionals participated with a 92.72% response rate. Of which 57.9% (n=162) were males while 42.1% (n=118) females. Of total 48.9% (n=137) (95% CI: 43.2, 55.0) health professionals had poor occupational health and safety. Availability of soap and bleach (AOR=2.50; 1.439, 4.356), Possibility of isolate COVID-19 suspected clients (AOR=2.525; 1.690, 5.062), Availability of infections prevention and control program standards and policy (AOR=2.329; 1.325, 4.092), Availability of policy and procedure to prevent COVID-19 (AOR= 2.427; 1.389, 4.240) were significantly associated. CONCLUSION: The result suggested that occupational health safety was generally low in the study area. Therefore, a preventive measure such as the use of personal protective equipment and adherence to hand hygiene practice and Infection prevention policy could reduce the spread of COVID-19 and further study should be conducted to generate more evidence on determinants of occupational health safety.

Haines, S., et al. (2020). "Practical Considerations When Performing Neurodiagnostic Studies on Patients with COVID-19 and Other Highly Virulent Diseases." Neurodiagn J **60**(2): 78-95.

The coronavirus disease 2019, SARS-COV-2 (the cause of COVID-19), has led to a worldwide shortage of personal protective equipment (PPE) and an increased stress on hospital resources, which has resulted in a spike in the anxiety of the frontline healthcare workers. News reports and information about the virus are rapidly changing. We present a case of a patient with COVID-19 who had a seizure-like spell for which an EEG was performed. In early to mid-March, there were no clear guidelines or recommendations available from neurodiagnostic-related organizations or hospitals on how to adapt procedure workflow to those with COVID-19. When caring for COVID-19 patients, as when caring for any patient with an infectious disease, it is hospital protocol to follow contact, droplet/airborne precautions by wearing appropriate PPE. However, because we knew very little about the coronavirus, this case was different. In this article, we discuss our experience with our EEG workflow and concerns for staff exposure. We then discuss our adaptations and modifications to our standard procedures and protocols. A time analysis comparing our standard EEG protocol with our modified COVID-19 protocol revealed a significant decrease in technologist exposure time (99 minutes versus 51 minutes), which theoretically would reduce the chance of virus transmission to our technologist. At this critical moment in time, we hope such modifications will allow us to continue delivering high quality patient care while optimizing resource utilization and above all keeping our technologists safe.

Haji, J. Y., et al. (2020). "State of Personal Protective Equipment Practice in Indian Intensive Care Units amidst COVID-19 Pandemic: A Nationwide Survey." Indian J Crit Care Med **24**(9): 809-816.

BACKGROUND: Optimal personal protective equipment (PPE) preparedness is key to minimize healthcare workers (HCW) infection with COVID-19. This two-phase survey evaluated PPE preparedness (adherence to Ministry of Health India (MoH) PPE-recommendations; HCW-training; PPE-inventory; PPE-breach management) in Indian intensive care units (ICU). MATERIALS AND METHODS: The phase 1 survey was distributed electronically to intensivists from 481 Indian hospitals between March 25, 2020, and April 06, 2020, as part of a multinational survey. Phase 2 was repeated in 320 Indian hospitals between April 20, 2020, and April 30, 2020. RESULTS: Response rate was 25% from 22 states. PPE practice varied between states and between private, government, and medical colleges. Between phase 1 and phase 2, all aspects of PPE training improved: donning/doffing 43% vs 66%, respectively; p value <0.01); safe waste disposal practices (38% vs 52%; p value = 0.09); intubation training (18% vs 31%; p value = 0.05); and transport (18% vs 31%; p value = 0.05). Perception of confidence for adequate PPE-training improved from 39 to 53% (p value = 0.26). In all, 47 to 60% ICUs adhered to MoH recommendations. Wearing N95-masks at all times increased from 47 to 60% (p value = 0.89). Very few ICUs provided quantitative/qualitative N95 masks fit testing (12% vs 29%; p value <0.01). Low-cost practices like "buddy-system" for donning-doffing (27% vs 44%; p value = 0.02) and showering after PPE breach (10% vs 8%; p value = 0.63) were underutilized. There was reluctance to PPE reuse. In all, 71% were unaware/diffident about PPE inventory. CONCLUSION: Despite interstate variability, most ICUs conformed to MoH recommendations. This survey conducted during initial pandemic phase demonstrated improved PPE preparedness uniformly across India with scope for further improvement. We suggest implementation of quality improvement measures to improve pandemic preparedness and minimize HCW infection rates, focused on regular PPE training, buddy system, and PPE-breach management. HOW TO CITE THIS ARTICLE: Haji JY, Subramaniam A, Kumar P, Ramanathan K, Rajamani A. State of Personal Protective Equipment Practice in Indian Intensive Care Units amidst COVID-19 Pandemic: A Nationwide Survey. Indian J Crit Care Med 2020;24(9):809-816.

Hajjij, A., et al. (2020). "Personal Protective Equipment and Headaches: Cross-Sectional Study Among Moroccan Healthcare Workers During COVID-19 Pandemic." Cureus **12**(12): e12047.

Background Healthcare workers in frontline during the coronavirus 19 disease (COVID-19) pandemic are mandated to wear specific personal protective equipment (PPE) including high filtrating masks and/or eye protection during extended period of time. Compressive headaches secondary to PPE use including N95 masks have been reported. We aim to describe subtypes of headache related to PPE use in our hospital in Casablanca and working condition factors associated with it. Methods We conducted a cross-sectional study among healthcare workers in frontline at Cheikh khalifa International University Hospital, using an online questionnaire. We collected demographic data, comorbidities and previous headaches history. Data about working conditions during pandemic, type and duration of PPE use were described. We calculated the prevalence of De Novo or an aggravated headache among healthcare workers. We studied correlations between PPE related headaches and working conditions and trends in PPE use during the pandemic. Finally, we described the overall discomfort related to PPE use. Results A total of 155 healthcare workers responded to the questionnaire. The N95 masks were the most used type (95.5%) associated with an eye protection in 61.3%. The overall prevalence of headache related to PPE was 62%. It was experienced De Novo in 32.9%, while it was an aggravation of pre-existing headache in 29%. Working more than 8 hours per shift during the pandemic was correlated to De novo headache (p = 0.008). The profession of doctor and working more than 12 hours per shift were correlated to aggravated headache (p = 0.02, p = 0.023). Healthcare workers experienced moderate discomfort, blurred vision and reduced concentration. They judged their professional performance mildly reduced by the use of PPE. Conclusion The increased use of PPE, especially high filtrating masks during the COVID-19 outbreak is responsible for generating headaches in healthcare workers on frontline either De novo or as an aggravation of pre-existing one. Working conditions have the greater impact on generating these types of headaches more than any pre-existing comorbidity. These findings should be considered to accommodate health care professionals to increase efficacy and adherence to protective measures during pandemic.

Hakim, M., et al. (2021). "Access and Use Experience of Personal Protective Equipment Among Frontline Healthcare Workers in Pakistan During the COVID-19 Emergency: A Cross-Sectional Study." Health Secur **19**(2): 140-149.

Healthcare workers are at the highest risk of contracting novel coronavirus disease 2019 (COVID-19) and, therefore, require constant protection. This study assesses access to personal protective equipment (PPE), availability of adequate information about PPE use, self-reported ability to correctly wear and remove (donning and doffing) PPE, and risk perceptions associated with COVID-19 among frontline healthcare workers in Pakistan. Using a structured and validated questionnaire, an online survey was conducted from May 9 to June 5, 2020. Responses were received from 453 healthcare workers. Of these, 218 (48.12%) were doctors, 183 (40.40%) were nurses, and 52 (11.48%) were paramedical staff. Only 129 (28.48%) healthcare workers reported having adequate access to PPE at all times, whereas 156 (34.44%) never had access to PPE and 168 (37.09%) had access to PPE occasionally. Lack of access to PPE led the majority (71.74%) of healthcare workers to use coping strategies such as reuse of N95 and surgical masks. A total of 312 (68.87%) respondents believed that the risk of contracting COVID-19 in the work environment was high and the majority (62.69%) adopted precautionary measures at home to keep their families safe. A significantly high (n = 233, 51.43%, P = .03) number of respondents reported self-medicating. Of all the respondents, only 136 (30.02%) were tested for COVID-19 at least once, of which 32 (23.53%) ever tested positive. These findings suggest that healthcare workers in Pakistan had limited access to PPE. Adequate provision and training is vital to protect the healthcare workforce during the COVID-19 pandemic.

Haldane, V., et al. (2020). "National primary care responses to COVID-19: a rapid review of the literature." BMJ Open **10**(12): e041622.

OBJECTIVE: The aim of this review, conducted in April 2020, is to examine available national primary care guidelines for COVID-19 and to explore the ways in which these guidelines support primary care facilities in responding to the demands of the COVID-19 pandemic. DESIGN: Rapid review and narrative synthesis. DATA SOURCES: PubMed, Embase and Google, as well as the websites of relevant national health departments, were searched from 1 January 2020 to 24 April 2020. ELIGIBILITY CRITERIA: Documents included must be issued by a national health authority, must be specific to COVID-19 care, directed at healthcare workers or managers, and must refer to the role of primary care in the COVID-19 response. RESULTS: We identified 17 documents from 14 countries. An adapted framework on primary care challenges and responses to pandemic influenza framed our analysis. Guidelines generally reported on COVID-19 service delivery and mostly made specific recommendations for ensuring continued delivery of essential primary care services through telehealth or other virtual care modalities. Few offered guidance to support surveillance as a public health function. All offered guidance on implementing outbreak control measures, largely through flexible and coordinated organisational models with partners from various sectors. There was a lack of guidance to support supply chain management and practice resilience in primary care, and lack of personal protective equipment represents a serious threat to the provision of quality care during the pandemic. CONCLUSIONS: Current national primary care guidelines for COVID-19 provide guidance on infection control and minimising the risk of spread in primary care practices, while supporting the use of new technology and coordinated partnerships. However, to ensure primary care practice resilience and quality of care are upheld, guidelines must offer recommendations on supply chain management and operational continuity, supported by adequate resources.

Hall, H. (2020). "The effect of the COVID-19 pandemic on healthcare workers' mental health." Jaapa **33**(7): 45-48.

The novel coronavirus (SARS-CoV-2) that emerged in late 2019 in Wuhan, China, commonly presents as a severe acute respiratory disease referred to as coronavirus disease-2019 (COVID-19). The rapid spread of the disease created challenges for healthcare systems and forced healthcare workers to grapple with clinical and nonclinical stressors, including shortages of personal protective equipment, mortality and morbidity associated with COVID-19, fear of bringing the virus home to family members, and the reality of losing colleagues to the disease. Evidence from previous outbreaks, along with early evidence from the COVID-19 pandemic, suggests that these events have significant short- and long-term effects on the mental health of healthcare workers. All healthcare stakeholders should create short- and long-term plans to support the mental health of workers during and after the COVID-19 pandemic.

Hamm, J. M., et al. (2020). "Telemedicine in the emergency department in the era of COVID-19: front-line experiences from 2 institutions." J Am Coll Emerg Physicians Open **1**(6): 1630-1636.

During the COVID-19 pandemic, one of the major changes that has occurred in emergency medicine is the evolution of telemedicine. With relaxation of regulatory and administrative barriers, the use of this already available technology has rapidly expanded. Telemedicine provides opportunity to markedly decrease personal protective equipment (PPE) and reduce healthcare worker exposures. Moreover, with the convenience and availability of access to medical care via telemedicine, a more fundamental change in healthcare delivery in the United States is likely. The implementation of telemedicine in the emergency department (ED) in particular has great potential to prevent the iatrogenic spread of COVID-19 and protect health care workers. Challenges to widespread adoption of telemedicine include privacy concerns, limitation of physical examination, and concerns of patient experience. In this clinical review, we discuss ED telemedicine applications, logistics, and challenges in the COVID-19 era as well as recent regulatory and legal changes. In addition, examples of telemedicine use are described from 2 institutions. Examples of future applications of telemedicine within the realm of emergency medicine are also discussed.

Han, A. Y., et al. (2020). "Time for a Paradigm Shift in Head and Neck Cancer Management During the COVID-19 Pandemic." Otolaryngol Head Neck Surg **163**(3): 447-454.

OBJECTIVE: The coronavirus disease 2019 (COVID-19) pandemic has caused physicians and surgeons to consider restructuring traditional cancer management paradigms. We aim to review the current evidence regarding the diagnosis and management of head and neck cancer, with an emphasis on the role of the multidisciplinary team (MDT) during a pandemic. DATA SOURCES: COVID-19 resources from PubMed, Google Scholar, the American Academy of Otolaryngology-Head and Neck Surgery, and the American Head and Neck Society were examined. REVIEW METHODS: Studies and guidelines related to the multidisciplinary management of head and neck cancer in the COVID-19 setting were reviewed. A total of 54 studies were included. Given the continuously evolving body of literature, the sources cited include the latest statements from medical and dental societies. RESULTS: The unpredictable fluctuation of hospital resources and the risk of the nosocomial spread of SARS-CoV-2 have direct effects on head and neck cancer management. Using an MDT approach to help define "essential surgery" for immediately life- or function-threatening disease processes in the context of available hospital resources will help to maximize outcomes. Early enrollment in an MDT is often critical for considering nonsurgical options to protect patients and health care workers. The role of the MDT continues after cancer treatment, if delivered, and the MDT plays an essential role in surveillance and survivorship programs in these challenging times. CONCLUSION: Head and neck cancer management during the COVID-19 pandemic poses a unique challenge for all specialists involved. Early MDT involvement is important to maximize patient outcomes and satisfaction in the context of public and community safety.

Han, T., et al. (2020). "The epidemiological characteristics of cluster transmission of coronavirus disease 2019 (COVID-19): a multi-center study in Jiangsu Province." Am J Transl Res **12**(10): 6434-6444.

Coronavirus Disease 2019 (COVID-19) pandemic has rapidly spread across the globe while little multi-center research about the epidemiological characteristics of cluster transmission is conducted. To provide a more comprehensive description of the epidemiological characteristics of cluster transmission and the virulence of SARS-CoV-2 carried by asymptomatic carriers, we studied the epidemiological characteristics of 70 clusters. 70 clusters including 311 consecutive subjects from January 20, 2020, to March 10, 2020, were enrolled. Of 70 clusters, 5 were infected by asymptomatic or presymptomatic carriers. We gathered and analyzed information about their demographic, epidemiological, clinical, diagnostic classification, and cluster characteristics. Among the 66 asymptomatic carriers in Jiangsu Province, 49 asymptomatic were observed in 311 subjects distributed in 70 clusters. We demonstrated that there is a significance between the severity of cases infected by asymptomatic carriers and cases infected by symptomatic patients (P=0.033) and the former usually presented with milder symptoms. A significant difference was shown regarding the level distribution of age (P=0.006) and the frequency distribution of gender (P=0.014) and disease severity of COVID-19 (P=0.008) among the seven groups classified by the relationship with the index cases. The average age of infected medical staff was the youngest and the majority of infected medical are females while the infected patients were generally oldest and usually accompanied by severest symptoms. We concluded that asymptomatic carriers are mainly screened out of clusters and the patients infected by asymptomatic carriers present with milder symptoms than those infected by symptomatic patients, which indicated that the SARS-CoV-2 shares decreased virulence among asymptomatic carriers. Effective measures should be taken to prevent transmission in hospitals to protect doctors, nurses, and patients.

Hanrahan, J., et al. (2021). "Implementation of SHEA/CDC Outbreak Response Training Program Tools to Develop a Dedicated COVID-19 Hospital in Lucas County, Ohio." Infect Control Hosp Epidemiol: 1-8.

We describe a care delivery model in which one hospital in a larger health system was dedicated exclusively to treatment of COVID-19 patients. This allowed for rapid training, conservation of resources and promoted safety of healthcare workers, demonstrated by no healthcare worker exposures due to improper personal protective equipment use.

Hanrath, A. T., et al. (2021). "Prior SARS-CoV-2 infection is associated with protection against symptomatic reinfection." J Infect **82**(4): e29-e30.

• Whether SARS-CoV-2 infection confers immunity to reinfection is uncertain. • The ‘second wave’ of transmission offered an opportunity to examine this. • We observed no symptomatic reinfections in a cohort of healthcare workers. • This apparent immunity to re-infection was maintained for at least 6 months. • Further studies are required to define immunological mechanism(s) and durability.

[Figure: see text]

eng

Haque, M. S., et al. (2021). "SARS-CoV-2 pandemic-induced PPE and single-use plastic waste generation scenario." Waste Manag Res: 734242x20980828.

The SARS-CoV-2 pandemic has demonstrated both positive and negative effects on the environment. Major concerns over personal hygiene, mandated and ease in lockdown actions and slackening of some policy measures have led to a massive surge in the use of disposable personal protective equipment (PPE) and other single-use plastic items. This generated an enormous amount of plastic waste from both healthcare and household units, and will continue to do so for the foreseeable future. Apart from the healthcare workers, the general public have become accustomed to using PPE. These habits are threatening the land and marine environment with immense loads of plastic waste, due to improper disposal practices across the world, especially in developing nations. Contaminated PPE has already made its way to the oceans which will inevitably produce plastic particles alongside other pathogen-driven diseases. This study provided an estimation-based approach in quantifying the amount of contaminated plastic waste that can be expected daily from the massive usage of PPE (e.g. facemasks) because of the countrywide mandated regulations on PPE usage. The situation of Bangladesh has been analysed and projections revealed that a total of 3.4 billion pieces of single-use facemask, hand sanitizer bottles, hand gloves and disposable polyethylene bags will be produced monthly, which will give rise to 472.30 t of disposable plastic waste per day. The equations provided for the quantification of waste from used single-use plastic and PPE can be used for other countries for rough estimations. Then, the discussed recommendations will help concerned authorities and policy makers to design effective response plans. Sustainable plastic waste management for the current and post-pandemic period can be imagined and acted upon.

Hara, T., et al. (2021). "Infection risk in a gastroenterological ward during a nosocomial COVID-19 infection event." J Med Virol **93**(1): 30-31.

The coronavirus disease (COVID‐19) first emerged in Wuhan, China, in December 2019 and rapidly infected a large number of individuals, and disease clusters have spread worldwide. A case of presumably nosocomial COVID‐19 was detected in the gastroenterological ward; however, appropriate precautions against contact and droplet prevented a subsequent infection cluster. This article is protected by copyright. All rights reserved.

Harding, H., et al. (2020). "Aerosol-generating procedures and infective risk to healthcare workers from SARS-CoV-2: the limits of the evidence." J Hosp Infect **105**(4): 717-725.

The transmission behaviour of severe acute respiratory syndrome coronavirus-2 (SARS-CoV-2) is still being defined. It is likely that it is transmitted predominantly by droplets and direct contact and it is possible that there is at least opportunistic airborne transmission. In order to protect healthcare staff adequately it is necessary that we establish whether aerosol-generating procedures (AGPs) increase the risk of transmission of SARS-CoV-2. Where we do not have evidence relating to SARS-CoV-2, guidelines for safely conducting these procedures should consider the risk of transmitting related pathogens. Currently there is very little evidence detailing the transmission of SARS-CoV-2 associated with any specific procedures. Regarding AGPs and respiratory pathogens in general, there is still a large knowledge gap that will leave clinicians unsure of the risk to themselves when offering these procedures. This review aimed to summarize the evidence (and gaps in evidence) around AGPs and SARS-CoV-2.

Hartmann, S., et al. (2020). "Coronavirus 2019 (COVID-19) Infections Among Healthcare Workers, Los Angeles County, February - May 2020." Clin Infect Dis.

Across the world, healthcare workers (HCW) are at a greater risk of infection by the novel coronavirus 2019 (COVID-19) due to the nature of their work. The Los Angeles County Department of Public Health (LAC DPH) set out to understand the impact of COVID-19 on healthcare facilities and HCWs by tracking and analyzing data from case-patient interviews of HCWs. As of May 31st, over three months into the pandemic, nearly 5,500 positive HCWs were reported to LAC DPH, representing 9.6% of all cases. Cases reported working in 27 different setting types, including outpatient medical offices, correctional facilities, emergency medical services, etc., with the highest proportion from long-term care facilities (46.6%) and hospitals (27.7%). Case-patients included both clinical and non-clinical roles, with nearly half (49.4%) of positive HCWs being nurses. Over two-thirds of HCWs (68.6%) worked at some point during their infectious period and nearly half (47.9%) reported a known exposure to a positive patient and/or co-worker within their facility. Overall, compared to all LAC cases, HCWs reported lower rates of hospitalization (5.3% vs. 12.2%) and death (0.7% vs. 4.3%) from COVID-19. There are many factors that increase HCWs risk of infection, including high risk work environment, limited supply of personal protective equipment, and even pressure to help and work during a pandemic. In response to these data, LAC DPH created resources and provided guidance for healthcare facilities to best protect their patients and staff during the COVID-19 pandemic.

Hasan, S. R., et al. (2020). "Anxiety among Doctors during COVID-19 Pandemic in Secondary and Tertiary Care Hospitals." Pak J Med Sci **36**(6): 1360-1365.

OBJECTIVE: To assess the level of anxiety among doctors during COVID-19 pandemic and the associated risk factors. METHODS: This cross-sectional study was conducted from 30th April to 16th May, 2020 in Karachi, Pakistan. The data was collected via an online web-based questionnaire. Questionnaire was used to assess anxiety level using GAD-7 scale among health-care professionals and the risk factors playing role in it. RESULTS: One hundred and fifty-one doctors participated in our study. Out of these 151 participants, 69 (45.7%) had mild, 22 (14.6%) had moderate, and 5 (3.3%) had severe symptoms of anxiety, whereas the remaining 55 (36.4%) had no anxiety according to GAD-7 scale. The median [interquartile range (IQR)] GAD-7 scale scores are 6.0 [3.00-9.00]. Females showed more severe degrees of measurement of anxiety symptoms than males. Doctors dealing with COVID-19 patients showed higher level of anxiety as compared to the doctors who were not dealing with COVID-19 patients, having a significant difference (U = 9.697, p = 0.008). One hundred and forty-one (93.4%) participants were concerned about being exposed to COVID-19 at work and 112 (74.2 %) thought they have inadequate protective equipment for safety. CONCLUSIONS: During COVID-19 pandemic, doctors exhibited different grades of anxiety. In order for healthcare workers to perform to the best of their capability, certain guidelines and interventions are needed.

Hasan, S. S., et al. (2021). "Social distancing and the use of PPE by community pharmacy personnel: Does evidence support these measures?" Res Social Adm Pharm **17**(2): 456-459.

Community pharmacists are one of the most accessible healthcare professionals and are often served as the first point of contact when it comes to minor ailments and health advice. As such, community pharmacists can play a vital role in a country's response to various preventative and public health measures amid the COVID-19 pandemic. Given the essential nature of community pharmacy as a health service, community pharmacies are unlikely to shut down in any foreseeable lockdown scenario. It is therefore important to assess the preventative measure directives for community pharmacies that are in place to safeguard community pharmacy personnel from SARS-CoV-2 in the various parts of the world. Upon reviewing the recommendations of 15 selected countries across five continents (Asia, Europe, Oceania, North America, and Africa) on social distancing and the use of personal protective equipment (PPE) in community pharmacies, we found inconsistencies in the recommended social distance to be practiced within the community pharmacies. There were also varying recommendations on the use of PPE by the pharmacy personnel. Despite the differences in the recommendations, maintaining recommended social distance and the wearing of appropriate PPE is of utmost importance for healthcare workers, including community pharmacy personnel dealing with day-to-day patient care activities, though full PPE should be worn when dealing with suspected COVID-19 patients.

Hashim, L., et al. (2020). "Physician Preparedness in Response to the Coronavirus Disease 2019 Pandemic: A Cross-Sectional Study From a Developing Country." Cureus **12**(9): e10383.

Background December 2019 marked the inception of a global pandemic, with cases being reported worldwide. In the developing nations with scarce healthcare resources, the reliance on healthcare workers who are amply prepared to withstand the prevailing scenario is indispensable. Our study aimed to assess the level of preparedness of doctors working in various hospitals across Pakistan to combat coronavirus disease 2019 (COVID-19). Methods We conducted an online questionnaire-based survey in May 2020 to estimate the level of preparedness of doctors working in various departments of various private and public hospitals across Pakistan. The survey comprised 36 questions, with items evaluating the provision of adequate protective equipment, training, mental health resources, and sound collaboration between healthcare workers and the hospital management during the COVID-19 crisis. Results A total of 346 doctors responded to the survey, among whom 56.4% were working in public sector hospitals and 46.5% were working more than five days per week. Of those included, 87.6% were being provided with disposable gloves, but 72.8% and 43.4% of respondents professed to having no access to eye protective equipment and gowns, respectively. Only 35.3% of respondents claimed to be trained regarding the use of personal protective equipment and 28.95% were being tested. Of the physicians, 43.4% claimed to have no proper triage system for the suspected patients and 98.3% were concerned about transmitting the disease to their family members. Of the doctors, 53.5% reported that there was sound collaboration between the hospital management and healthcare staff. Conclusion The survey provided evidence of inadequate delivery of personal protective equipment and training to doctors working in various hospitals across Pakistan. A sound collaboration between the hospital management and departments needs to be addressed.

Hasnain, M., et al. (2020). "Protection challenges of pregnant women against vertical transmission during COVID-19 epidemic: A narrative review." Am J Infect Control **48**(12): 1516-1519.

This paper presents a narrative review study of 5 popular data repositories focusing on challenges of pregnant women protection during the COVID-19 pandemic. The study concludes that the likelihood of a vertical transmission of COVID-19 infection from pregnant women to neonates was not observed. Nevertheless, it remains a serious risk for them during their earlier stage of pregnancy, thus, special attention from health professionals has been recommended.

Hassan, M. Z., et al. (2021). "Protecting frontline healthcare workers should be the top priority in low-resource health systems: Bangladesh and COVID-19." Infect Control Hosp Epidemiol **42**(1): 121-122.

Hassan, S. S., et al. (2021). "SARS-CoV-2 infections amongst personnel providing home care services for older persons in Stockholm, Sweden." J Intern Med.

BACKGROUND: In Sweden, home care services is a major external contact for older persons. METHODS: Five home care service companies in Stockholm, Sweden, enrolled 405 employees to a study including serum IgG to SARS-CoV-2 and SARS-CoV-2 virus in throat swabs. RESULTS: 20.1% (81/403) of employees were seropositive, about twice as many as in a simultaneously enrolled reference population (healthcare workers entirely without patient contact, n = 3671; 9.7% seropositivity). 13/379 employees (3.4%) had a current infection (PCR positivity). Amongst these, 5 were also seropositive and 3 were positive with low amounts of virus. High amounts of virus and no antibodies (a characteristic for presymptomatic COVID-19) were present in 5 employees (1.3%). CONCLUSIONS: Personnel providing home services for older persons appear to be a risk group for SARS-CoV-2. Likely presymptomatic employees can be readily identified by screening. Increased protection of employees and of the older persons they serve is warranted.

Hayakawa, S., et al. (2021). "Anti-SARS-CoV-2 vaccination strategy for pregnant women in Japan." J Obstet Gynaecol Res.

The current COVID-19 pandemic is a global concern. The recent introduction of vaccines has provided a reason for hope, but new problems, such as vaccine hesitancy, have arisen. One of the most important of these issues is the safety of vaccines for pregnant women. In this article, we collected worldwide indications for vaccination, including women who are pregnant or who wish to become pregnant, and reports of adverse reactions to COVID-19 vaccination. The Japan Society of Obstetrics and Gynecology and the Japanese Society of Infectious Diseases in Obstetrics and Gynecology have published recommendations for the vaccination of pregnant women with a COVID-19 vaccine. The guidelines are as follows: (1) pregnant women should not be excluded from vaccination; (2) informed consent should be obtained before vaccination; (3) healthcare workers and pregnant women with complications such as diabetes, hypertension, and obesity should be vaccinated preferentially; (4) vaccination should be avoided until 12 weeks of gestation during organogenesis; (5) spouse and family members should be vaccinated actively; and (6) nursing mothers are not particularly affected. This policy has been adopted in government guidelines. Additional efforts should be made to protect pregnant women from infection and severe illness with COVID-19 by eliminating vaccine hesitancy.

Hazarika, M. (2020). "The cacophony of emotions in a mental health professional from the isolation ward during coronavirus disease 2019 (COVID-19) pandemic." Open J Psychiatry Allied Sci **11**(2): 130-132.

This is a narrative of the thoughts of a first-line healthcare worker, and the psychological overview of the same while working in isolation ward and being in quarantine during coronavirus disease 2019 (COVID-19) pandemic.

Heffernan, D. S., et al. (2020). "Surgical Infection Society Guidance for Operative and Peri-Operative Care of Adult Patients Infected by the Severe Acute Respiratory Syndrome Coronavirus-2 (SARS-CoV-2)." Surg Infect (Larchmt) **21**(4): 301-308.

Background: Severe acute respiratory syndrome coronavirus-2 (SARS-CoV-2)-associated viral infection (coronavirus disease 2019, COVID-19) is a virulent, contagious viral pandemic that is affecting populations worldwide. As with any airborne viral respiratory infection, surgical and non-surgical patients may be affected. Methods: Review and synthesis of pertinent English-language literature pertaining to COVID-19 infection among adult patients. Results: COVID-19 disease that requires hospitalization results in critical illness approximately 25% of the time and requires mechanical ventilation with positive airway pressure. Acute kidney injury, a marked hypercoagulable state, and sometimes myocarditis can be features of COVID-19 in addition to the characteristic severe acute lung injury. Even if not among the most seriously afflicted, older patients with medical comorbidities are both predisposed to infection and risk increased morbidity and mortality, however, all persons presenting for surgical intervention should be suspected of infection (and thus transmissibility) even if asymptomatic. Although most elective surgery has been curtailed by administrative or governmental fiat, patients will still need urgent or emergency operative intervention for time-sensitive disease processes such as malignant neoplasia or for true emergencies such as perforated viscus or traumatic injury. It is possible to provide safe surgical care for SARS-CoV-2-positive patients and minimize nosocomial transmission to healthcare workers. Conclusions: This guidance will facilitate appropriate protection of patients and staff, and maintenance of infection control measures to assist surgical personnel and facilities to prepare for COVID-19-infected adult patients requiring urgent or emergent operative intervention and to provide optimal patient care.

Heider, C. A., et al. (2021). "Prevalence of Voice Disorders in Healthcare Workers in the Universal Masking COVID-19 Era." Laryngoscope **131**(4): E1227-e1233.

OBJECTIVES/HYPOTHESIS: To determine the prevalence and associated risk factors of voice disorders in healthcare workers of high-risk hospital care units during the 2019 coronavirus disease (COVID-19) pandemic. STUDY DESIGN: Cross-sectional study. METHODS: Questionnaire survey to healthcare personnel of COVID-19 high-risk hospital units was conducted, regarding demographic data, clinical activity, the pattern of usage of personal protective equipment, medical and vocal history, vocal symptoms, and Spanish validated Voice Handicap Index (VHI)-10 questionnaire. RESULTS: A total of 221 healthcare workers answered the survey. Nearly 33% of them reported having trouble with their voice during the last month, and 26.24% had an abnormal score in the Spanish validated VHI-10 questionnaire. The mean VHI-10 score was 7.92 (95% confidence interval 6.98-8.85). The number of working hours, the number of hours of mask daily use, simultaneous surgical and self-filtering mask use, and working in intermediate or intensive care units were independent variables significantly associated with a higher VHI-10 score. CONCLUSIONS: Healthcare workers of high-risk hospital care units during the universal masking COVID-19 pandemic are at risk of voice disorders. LEVEL OF EVIDENCE: 3 Laryngoscope, 131:E1227-E1233, 2021.

Hemati, S., et al. (2021). "Simultaneous monitoring of SARS-CoV-2, bacteria, and fungi in indoor air of hospital: a study on Hajar Hospital in Shahrekord, Iran." Environ Sci Pollut Res Int: 1-11.

The novel SARS-CoV-2 outbreak was declared as pandemic by the World Health Organization (WHO) on March 11, 2020. Understanding the airborne route of SARS-CoV-2 transmission is essential for infection prevention and control. In this study, a total of 107 indoor air samples (45 SARS-CoV-2, 62 bacteria, and fungi) were collected from different wards of the Hajar Hospital in Shahrekord, Iran. Simultaneously, bacterial and fungal samples were also collected from the ambient air of hospital yard. Overall, 6 positive air samples were detected in the infectious 1 and infectious 2 wards, intensive care unit (ICU), computed tomography (CT) scan, respiratory patients' clinic, and personal protective equipment (PPE) room. Also, airborne bacteria and fungi were simultaneously detected in the various wards of the hospital with concentrations ranging from 14 to 106 CFU m(-3) and 18 to 141 CFU m(-3), respectively. The highest mean concentrations of bacteria and fungi were observed in respiratory patients' clinics and ICU wards, respectively. Significant correlation (p < 0.05) was found between airborne bacterial concentration and the presence of SARS-CoV-2, while no significant correlation was found between fungi concentration and the virus presence. This study provided an additional evidence about the presence of SARS-CoV-2 in the indoor air of a hospital that admitted COVID-19 patients. Moreover, it was revealed that the monitoring of microbial quality of indoor air in such hospitals is very important, especially during the COVID-19 pandemic, for controlling the nosocomial infections.

Heng, A. L., et al. (2020). "The bug stops with me: Prevention of COVID-19 nosocomial transmission during radiographic procedures." J Med Imaging Radiat Sci **51**(4): 540-545.

INTRODUCTION: Nosocomial transmission of Coronavirus Disease 2019 (COVID-19) is a preventable risk to the patient population and radiographer workforce. This article aims to describe the prevention of COVID-19 nosocomial transmission during radiographic procedures by the utilization of stringent infection prevention measures at a leading tertiary hospital in Singapore. METHODS: The implemented measures are the appropriate use of personal protective equipment, staff education, infection controls measures such as equipment disinfection, physical distancing and segregation of staff and patients. RESULTS: Despite the handling of 1637 COVID-19 cases as of July 25, 2020, there has not been a single case of known nosocomial transmission of the disease in the institution. DISCUSSION: The absence of nosocomial transmission suggests that the implemented measures are adequate. These measures will need to be sustainable for the long term in order to ensure continued success. CONCLUSION: Radiographers are a crucial part of the team in the fight against COVID-19 and are at risk of contracting COVID-19. Strict adherence to appropriate infection control measures is essential for the safety of Radiology staff and their patients, and the successful control of this pandemic.

Publisher: Abstract available from the publisher.

fre

Hennein, R., et al. (2021). "Socio-ecological predictors of mental health outcomes among healthcare workers during the COVID-19 pandemic in the United States." PLoS One **16**(2): e0246602.

BACKGROUND: Healthcare workers are at increased risk of adverse mental health outcomes during the COVID-19 pandemic. Studies are warranted that examine socio-ecological factors associated with these outcomes to inform interventions that support healthcare workers during future disease outbreaks. METHODS: We conducted an online cross-sectional study of healthcare workers during May 2020 to assess the socio-ecological predictors of mental health outcomes during the COVID-19 pandemic. We assessed factors at four socio-ecological levels: individual (e.g., gender), interpersonal (e.g., social support), institutional (e.g., personal protective equipment availability), and community (e.g., healthcare worker stigma). The Personal Health Questionnaire-9, Generalized Anxiety Disorder-7, Primary Care Post-Traumatic Stress Disorder, and Alcohol Use Disorders Identification Test-Concise scales assessed probable major depression (MD), generalized anxiety disorder (GAD), posttraumatic stress disorder (PTSD), and alcohol use disorder (AUD), respectively. Multivariable logistic regression models were used to assess unadjusted and adjusted associations between socio-ecological factors and mental health outcomes. RESULTS: Of the 1,092 participants, 72.0% were female, 51.9% were frontline workers, and the mean age was 40.4 years (standard deviation = 11.5). Based on cut-off scores, 13.9%, 15.6%, 22.8%, and 42.8% had probable MD, GAD, PTSD, and AUD, respectively. In the multivariable adjusted models, needing more social support was associated with significantly higher odds of probable MD, GAD, PTSD, and AUD. The significance of other factors varied across the outcomes. For example, at the individual level, female gender was associated with probable PTSD. At the institutional level, lower team cohesion was associated with probable PTSD, and difficulty following hospital policies with probable MD. At the community level, higher healthcare worker stigma was associated with probable PTSD and AUD, decreased satisfaction with the national government response with probable GAD, and higher media exposure with probable GAD and PTSD. CONCLUSIONS: These findings can inform targeted interventions that promote healthcare workers' psychological resilience during disease outbreaks.

Herrero San Martin, A., et al. (2020). "Sleep characteristics in health workers exposed to the COVID-19 pandemic." Sleep Med **75**: 388-394.

INTRODUCTION: The development of sleep disorders, and specifically insomnia, has been linked to the exposure to different stressors. In this line, Coronavirus disease 2019 (COVID-19) outbreak caused by the new coronavirus SARS-CoV-2, has caused a huge impact on our environment, and has exposed healthcare workers to an unprecedented threat. In this study, we try to assess sleep quality and the development of sleep disorders in health personnel directly dedicated to the care of COVID-19 patients at the height of the pandemic, compared to the general population. MATERIALS AND METHODS: A cross-sectional, anonymized, self-reported questionnaire survey was carried out at the "12 de Octubre" Hospital, in Madrid, Spain, during the outbreak of COVID-19, from March 1st to April 30th 2020. We compared two groups, healthcare workers who have treated directly COVID-19 patients versus non-healthcare workers. The questionnaire included demographic data, sleep related aspects, Epworth Sleepiness Scale (ESS), Pittsburgh Sleep Quality Index (PSQI), Insomnia Severity Index (ISI) and 17-items Hamilton Rating Scale (HRS). RESULTS: In total 170 participants completed the questionnaire successfully, 100 healthcare workers and 70 non-healthcare workers. Self-reported insomnia, nightmares, sleepwalking, sleep terrors and PSQI>6 were more frequent in the healthcare group (p < 0,05). Shift work was associated to greater risk when performing multiple logistic regression analysis. CONCLUSIONS: We observed that, during the outbreak of COVID-19, healthcare workers on the front line developed more sleep disturbances than non-healthcare professionals, and they had worse quality of sleep. Special attention should be paid to shift workers. Concrete protection and prevention measures for particularly exposed population should be considered in pandemic situations.

Herzik, K. A. and L. Bethishou (2021). "The impact of COVID-19 on pharmacy transitions of care services." Res Social Adm Pharm **17**(1): 1908-1912.

COVID-19 has necessitated alterations to the delivery of healthcare services. Modifications include those made to improve patient and healthcare worker safety such as the use of personal protective equipment. Pharmacy services, specifically pharmacy transitions of care services have not been immune to change which have brought along their own set of unique challenges to consider. This paper discusses how COVID-19 has impacted the delivery of pharmacy transitions of care services with real world examples from Sharp Grossmont Hospital and Hoag Memorial Hospital Presbyterian. Procedures implemented to minimize the spread and contraction of COVID-19 such as minimized patient contact and altered visitor policies have made it more challenging to obtain a best possible medication list the patient was taking prior to arrival to the hospital which has lead to an increased reliance on secondary sources to complete medication histories. Regarding discharge prescriptions, preference has shifted to the use of electronic vs. hard copy prescriptions, mail order, and utilization of med to bed programs and other hospital medication delivery services to limit patient contact in outpatient pharmacies. An improved effort to resolve medication acquisition issues prior to discharge utilizing patient assistance programs and other hospital programs to cover the cost of medications for COVID positive patients under certain circumstances has been seen. This paper highlights the important role pharmacists can play in providing effective communication, supporting continuity of care, and advocating for patient engagement and empowerment during transitions of care in the COVID-19 pandemic.

Hessling, M., et al. (2020). "Selection of parameters for thermal coronavirus inactivation - a data-based recommendation." GMS Hyg Infect Control **15**: Doc16.

Background: Healthcare workers and large parts of the population are currently using personal protective equipment, such as face masks, to avoid infections with the novel coronavirus SARS-CoV-2. This equipment must be sterilized as gently as possible before reuse. One possibility is thermal inactivation, but professional autoclaves with their high temperatures are often not available or suitable. If the inactivation period is long enough, coronavirus inactivation can also be carried out at relatively low temperatures. The required duration was determined in this study. Material and methods: Data from published thermal inactivation studies on coronaviruses were applied to determine the temperature dependence of the rate constant k(T) for each coronavirus by employing Arrhenius models. Results: The data obtained exhibit large variations, which appear to be at least partially caused by different sample properties. Samples with high protein content or samples in dry air sometimes seem to be more difficult to inactivate. Apart from this, the Arrhenius models describe the thermal inactivation properties well and SARS-CoV and SARS-CoV-2 can even be represented by a combined model. Furthermore, the available data suggest that all samples, including critical ones, can be mathematically included by a worst-case Arrhenius model. Conclusion: Coronaviruses can already be inactivated at relatively low temperatures. For most samples, application times of approximately 32.5, 3.7, and 0.5 minutes will be sufficient at 60°C, 80°C, and 100°C, respectively, for a 5 log-reduction. For difficult conditions, the worst-case model provides significantly longer application times of 490, 55, and 8 minutes for the temperatures mentioned.

Hill, W. C., et al. (2020). "Testing of Commercial Masks and Respirators and Cotton Mask Insert Materials using SARS-CoV-2 Virion-Sized Particulates: Comparison of Ideal Aerosol Filtration Efficiency versus Fitted Filtration Efficiency." Nano Lett **20**(10): 7642-7647.

Shortages in the availability of personal protective face masks during the COVID-19 pandemic required many to fabricate masks and filter inserts from available materials. While the base filtration efficiency of a material is of primary importance when a perfect seal is possible, ideal fit is not likely to be achieved by the average person preparing to enter a public space or even a healthcare worker without fit-testing before each shift. Our findings suggest that parameters including permeability and pliability can play a strong role in the filtration efficiency of a mask fabricated with various filter media, and that the filtration efficiency of loosely fitting masks/respirators against ultrafine particulates can drop by more than 60% when worn compared to the ideal filtration efficiency of the base material. Further, a test method using SARS-CoV-2 virion-sized silica nanoaerosols is demonstrated to assess the filtration efficiency against nanoparticulates that follow air currents associated with mask leakage.

Hines, S. E., et al. (2021). "Trends in Moral Injury, Distress, and Resilience Factors among Healthcare Workers at the Beginning of the COVID-19 Pandemic." Int J Environ Res Public Health **18**(2).

The coronavirus severe acute respiratory syndrome (COVID-19) pandemic has placed increased stress on healthcare workers (HCWs). While anxiety and post-traumatic stress have been evaluated in HCWs during previous pandemics, moral injury, a construct historically evaluated in military populations, has not. We hypothesized that the experience of moral injury and psychiatric distress among HCWs would increase over time during the pandemic and vary with resiliency factors. From a convenience sample, we performed an email-based, longitudinal survey of HCWs at a tertiary care hospital between March and July 2020. Surveys measured occupational and resilience factors and psychiatric distress and moral injury, assessed by the Impact of Events Scale-Revised and the Moral Injury Events Scale, respectively. Responses were assessed at baseline, 1-month, and 3-month time points. Moral injury remained stable over three months, while distress declined. A supportive workplace environment was related to lower moral injury whereas a stressful, less supportive environment was associated with increased moral injury. Distress was not affected by any baseline occupational or resiliency factors, though poor sleep at baseline predicted more distress. Overall, our data suggest that attention to improving workplace support and lowering workplace stress may protect HCWs from adverse emotional outcomes.

Hoernke, K., et al. (2021). "Frontline healthcare workers' experiences with personal protective equipment during the COVID-19 pandemic in the UK: a rapid qualitative appraisal." BMJ Open **11**(1): e046199.

OBJECTIVES: To report frontline healthcare workers' (HCWs) experiences with personal protective equipment (PPE) during the COVID-19 pandemic in the UK. To understand HCWs' fears and concerns surrounding PPE, their experiences following its guidance and how these affected their perceived ability to deliver care during the COVID-19 pandemic. DESIGN: A rapid qualitative appraisal study combining three sources of data: semistructured in-depth telephone interviews with frontline HCWs (n=46), media reports (n=39 newspaper articles and 145 000 social media posts) and government PPE policies (n=25). PARTICIPANTS: Interview participants were HCWs purposively sampled from critical care, emergency and respiratory departments as well as redeployed HCWs from primary, secondary and tertiary care centres across the UK. RESULTS: A major concern was running out of PPE, putting HCWs and patients at risk of infection. Following national level guidance was often not feasible when there were shortages, leading to reuse and improvisation of PPE. Frequently changing guidelines generated confusion and distrust. PPE was reserved for high-risk secondary care settings and this translated into HCWs outside these settings feeling inadequately protected. Participants were concerned about differential access to adequate PPE, particularly for women and Black, Asian and Minority Ethnic HCWs. Participants continued delivering care despite the physical discomfort, practical problems and communication barriers associated with PPE use. CONCLUSION: This study found that frontline HCWs persisted in caring for their patients despite multiple challenges including inappropriate provision of PPE, inadequate training and inconsistent guidance. In order to effectively care for patients during the COVID-19 pandemic, frontline HCWs need appropriate provision of PPE, training in its use as well as comprehensive and consistent guidance. These needs must be addressed in order to protect the health and well-being of the most valuable healthcare resource in the COVID-19 pandemic: our HCWs.

Hossain, F. and A. Clatty (2021). "Self-care strategies in response to nurses' moral injury during COVID-19 pandemic." Nurs Ethics **28**(1): 23-32.

These are strange and unprecedented times in the wake of the COVID-19 pandemic. Most frontline healthcare professionals have never witnessed anything like this before. As a result, staff may experience numerous and continuous traumatic events, which in many instances, will negatively affect their psychological well-being. Particularly, nurses face extraordinary challenges in response to shifting protocols, triage, shortages of resources, and the astonishing numbers of patients who require care in expedited time constraints. As most healthcare workers are passionate nursing professionals, frustration and often a sense of powerlessness occur when they find themselves unable to provide needed care to their patients. The overwhelming number of deaths, patients isolated and dying alone, and the ever-present fear of being infected and then infecting colleagues, family, friends due to the lack of protective gear or known protocols takes its toll on emotional and psychological well-being. For nurses, the experience of this significant (hopefully once-in-a-lifetime) event can inflict on-going moral injury. Nurses affected by this trauma require education, coping tools, and therapy to help avoid or alleviate the adverse effects on their well-being. Institutions must provide these resources to tend to the well-being of their healthcare staff, during and beyond the pandemic. This article aims to investigate moral distress-considering it as a moral injury-and offer tools and recommendations to support healthcare nurses as they respond to this crisis and its aftermath.

Hossain, L., et al. (2021). "Engineering laminated paper for SARS-CoV-2 medical gowns." Polymer (Guildf) **222**: 123643.

The COVID-19 pandemic has highlighted the need for diversity in the market and alternative materials for personal protective equipment (PPE). Paper has high coatability for tunable barrier performance, and an agile production process, making it a potential substitute for polyolefin-derived PPE materials. Bleached and newsprint papers were laminated with polyethylene (PE) coatings of different thicknesses, and characterised for their potential use as medical gowns for healthcare workers and COVID-19 patients. Thicker PE lamination improved coating homogeneity and water vapour resistance. 49 GSM bleached paper with 16 GSM PE coating showed high tensile and seam strength, and low water vapour transmission rate (WVTR). Phi-X174 bacteriophage testing revealed that paper laminated with 15 GSM coating hinders virus penetration. This research demonstrates that PE laminated paper is a promising material for low cost viral protective gowns.

Hossain, M. A., et al. (2021). "Healthcare Workers' Knowledge, Attitude, and Practice Regarding Personal Protective Equipment for the Prevention of COVID-19." J Multidiscip Healthc **14**: 229-238.

PURPOSE: Adequate knowledge, positive attitude, and proper practice of personal protective equipment by healthcare workers are necessary to get protection from COVID-19 infection. But this area is yet to be explored. Hence, we aimed to assess the knowledge, attitude, and practice (KAP) regarding personal protective equipment (PPE) among healthcare workers, along with a survey of the possible determinants. SUBJECTS AND METHODS: For this cross-sectional study, online and offline surveys were conducted among a sample of 393 healthcare workers from five different districts of Bangladesh. A validated self-administered questionnaire comprising five sections (socio-demography, work-related information, knowledge, attitude, and practice) was used for data collection. Multivariate stepwise forward logistic regression was applied to find significant factors associated with good attitude, and practice using SPSS version 25. RESULTS: The average age of the 393 participants was 28.9±5.2 years with a male-female ratio of one. Of them, 99.5% (n=391) had good knowledge, 88.8% (n=349) had positive attitude and 51.7% (n=203) had good practice regarding PPE. Results revealed that being a physician and living at home were significantly associated with a positive attitude. While being a non-physician, having lower education, working in private hospitals, and using office transport were associated with good practice regarding PPE. CONCLUSION: The findings demonstrated that the healthcare workers had an overall good knowledge and a positive attitude but a poor practice regarding PPE. This study also highlighted the factors influencing KAP towards PPE that must be addressed in future education, awareness, and counseling programs.

Hoti, K., et al. (2020). "Provision of community pharmacy services during COVID-19 pandemic: a cross sectional study of community pharmacists' experiences with preventative measures and sources of information." Int J Clin Pharm **42**(4): 1197-1206.

Background An infectious disease caused by a novel coronavirus (later called COVID-19) reached pandemic levels in 2020 and community pharmacists were involved in responding to this pandemic, also in Kosovo. Objectives To explore the experiences of community pharmacists in relation to provision of community pharmacy services during COVID-19 pandemic. Setting Community pharmacists in Kosovo. Methods This was a cross-sectional study where data was collected via a self-administered online questionnaire, from 264 pharmacists actively practicing in Kosovo during the pandemic. The questionnaire consisted of a combination of closed and open-ended questions, optional statements and statements on a five-point Likert scale, derived at least in part from the Transtheoretical Model. One-way analysis of variance was used to analyze differences in responses to Likert-type items whereas categorical variables were analyzed using Chi square testing. Main outcome measures Community pharmacists' perceptions on COVID-19 related preventative measures. Results A response rate of 40.6% was achieved. Sufficient and adequate COVID-19-related preventative measures were being implemented by a majority of pharmacies (n = 232; 87.9%), and over two-thirds of respondents agreed/strongly agreed that their pharmacies were sufficiently prepared with protective equipment for their personnel. Implementation of preventative measures was associated with respondents' perception that pharmacists and the pharmacy profession were valued more by patients during the pandemic and to a lesser degree, by other health professionals. Most commonly stated pros dealt with employee and patient safety, while key cons dealt with increased costs and running out of the necessary protective equipment. Key barriers to pharmacy activities were price increases by wholesalers, and patients' panic and excessive buying, whereas drivers dealt with professional obligation to assist and opportunity to prove inseparable to other health professionals. The most popular means of accessing COVID-19 related information by pharmacists was via mobile devices and information from professional organizations was considered most useful by pharmacists. Conclusions Community pharmacies actively implemented various measures as precautions to mitigate the spread of COVID-19. Our findings highlight the value of continuous provision of information by professional organizations and use of mobile devices as key means to access information by pharmacists.

Hou, X., et al. (2021). "Educational needs in the COVID-19 pandemic: a Delphi study among doctors and nurses in Wuhan, China." BMJ Open **11**(4): e045940.

OBJECTIVE: To identify theoretical and technical aspects regarding treatment, prevention of spread and protection of staff to inform the development of a comprehensive training curriculum on COVID-19 management. DESIGN: Cross-sectional study. SETTING: Nine hospitals caring for patients with COVID-19 in Wuhan, China. PARTICIPANTS: 134 Chinese healthcare professionals (74 doctors and 60 nurses) who were deployed to Wuhan, China during the COVID-19 epidemic were included. A two-round Delphi process was initiated between March and May 2020. In the first round, the participants identified knowledge, technical and behavioural (ie, non-technical) skills that are needed to treat patients, prevent spread of the virus and protect healthcare workers. In round 2, the participants rated each item according to its importance to be included in a training curriculum on COVID-19. Consensus for inclusion in the final list was set at 80%. PRIMARY OUTCOME MEASURES: Knowledge, technical and behavioural (ie, non-technical) skills that could form the basis of a training curriculum for COVID-19 management. RESULTS: In the first round 1398 items were suggested by the doctors and reduced to 67 items after content analysis (treatment of patients: n=47; infection prevention and control: n=20). The nurses suggested 1193 items that were reduced to 70 items (treatment of patients: n=49; infection prevention and control: n=21). In round 2, the response rates were 82% in doctors and 93% in nurses. Fifty-eight items of knowledge, technical and behavioural skills were agreed on by the doctors to include in the final list. For the nurses, 58 items were agreed on. CONCLUSIONS: This needs assessment process resulted in a comprehensive list of knowledge, technical and behavioural skills for COVID-19 management. Educators can use these to guide decisions regarding content of training curricula not only for COVID-19 management but also in preparation for future viral pandemic outbreaks.

Houghton, C., et al. (2020). "Barriers and facilitators to healthcare workers' adherence with infection prevention and control (IPC) guidelines for respiratory infectious diseases: a rapid qualitative evidence synthesis." Cochrane Database Syst Rev **4**(4): Cd013582.

BACKGROUND: This review is one of a series of rapid reviews that Cochrane contributors have prepared to inform the 2020 COVID-19 pandemic. When new respiratory infectious diseases become widespread, such as during the COVID-19 pandemic, healthcare workers' adherence to infection prevention and control (IPC) guidelines becomes even more important. Strategies in these guidelines include the use of personal protective equipment (PPE) such as masks, face shields, gloves and gowns; the separation of patients with respiratory infections from others; and stricter cleaning routines. These strategies can be difficult and time-consuming to adhere to in practice. Authorities and healthcare facilities therefore need to consider how best to support healthcare workers to implement them. OBJECTIVES: To identify barriers and facilitators to healthcare workers' adherence to IPC guidelines for respiratory infectious diseases. SEARCH METHODS: We searched OVID MEDLINE on 26 March 2020. As we searched only one database due to time constraints, we also undertook a rigorous and comprehensive scoping exercise and search of the reference lists of key papers. We did not apply any date limit or language limits. SELECTION CRITERIA: We included qualitative and mixed-methods studies (with a distinct qualitative component) that focused on the experiences and perceptions of healthcare workers towards factors that impact on their ability to adhere to IPC guidelines for respiratory infectious diseases. We included studies of any type of healthcare worker with responsibility for patient care. We included studies that focused on IPC guidelines (local, national or international) for respiratory infectious diseases in any healthcare setting. These selection criteria were framed by an understanding of the needs of health workers during the COVID-19 pandemic. DATA COLLECTION AND ANALYSIS: Four review authors independently assessed the titles, abstracts and full texts identified by our search. We used a prespecified sampling frame to sample from the eligible studies, aiming to capture a range of respiratory infectious disease types, geographical spread and data-rich studies. We extracted data using a data extraction form designed for this synthesis. We assessed methodological limitations using an adapted version of the Critical Skills Appraisal Programme (CASP) tool. We used a 'best fit framework approach' to analyse and synthesise the evidence. This provided upfront analytical categories, with scope for further thematic analysis. We used the GRADE-CERQual (Confidence in the Evidence from Reviews of Qualitative research) approach to assess our confidence in each finding. We examined each review finding to identify factors that may influence intervention implementation and developed implications for practice. MAIN RESULTS: We found 36 relevant studies and sampled 20 of these studies for our analysis. Ten of these studies were from Asia, four from Africa, four from Central and North America and two from Australia. The studies explored the views and experiences of nurses, doctors and other healthcare workers when dealing with severe acute respiratory syndrome (SARS), H1N1, MERS (Middle East respiratory syndrome), tuberculosis (TB), or seasonal influenza. Most of these healthcare workers worked in hospitals; others worked in primary and community care settings. Our review points to several barriers and facilitators that influenced healthcare workers' ability to adhere to IPC guidelines. The following factors are based on findings assessed as of moderate to high confidence. Healthcare workers felt unsure as to how to adhere to local guidelines when they were long and ambiguous or did not reflect national or international guidelines. They could feel overwhelmed because local guidelines were constantly changing. They also described how IPC strategies led to increased workloads and fatigue, for instance because they had to use PPE and take on additional cleaning. Healthcare workers described how their responses to IPC guidelines were influenced by the level of support they felt that they received from their management team. Clear communication about IPC guidelines was seen as vital. But healthcare workers pointed to a lack of training about the infection itself and about how to use PPE. They also thought it was a problem when training was not mandatory. Sufficient space to isolate patients was also seen as vital. A lack of isolation rooms, anterooms and shower facilities was a problem. Other important practical measures described by healthcare workers included minimising overcrowding, fast-tracking infected patients, restricting visitors, and providing easy access to handwashing facilities. A lack of PPE, and equipment that was of poor quality, was a serious concern for healthcare workers and managers. They also pointed to the need to adjust the volume of supplies as infection outbreaks continued. Healthcare workers believed that they followed IPC guidance more closely when they saw the value of it. Some healthcare workers felt motivated to follow the guidance because of fear of infecting themselves or their families, or because they felt responsible for their patients. Some healthcare workers found it difficult to use masks and other equipment when it made patients feel isolated, frightened or stigmatised. Healthcare workers also found masks and other equipment uncomfortable to use. The workplace culture could also influence whether healthcare workers followed IPC guidelines or not. Across many of the findings, healthcare workers pointed to the importance of including all staff, including cleaning staff, porters, kitchen staff and other support staff when implementing IPC guidelines. AUTHORS' CONCLUSIONS: Healthcare workers point to several factors that influence their ability and willingness to follow IPC guidelines when managing respiratory infectious diseases. These include factors tied to the guideline itself and how it is communicated, support from managers, workplace culture, training, physical space, access to and trust in personal protective equipment, and a desire to deliver good patient care. The review also highlights the importance of including all facility staff, including support staff, when implementing IPC guidelines.

Hsu, S. H., et al. (2020). "Aerosol containment box to the rescue: extra protection for the front line." Emerg Med J **37**(7): 400-401.

As the COVID-19 pandemic has created shortages of vital personal protective equipment that threatens healthcare workers' risk of exposure, a need for innovative new ways to protect healthcare workers has emerged. An aerosol containment box that covers the patient's head and neck in bed provides a solution to protect clinicians during aerosol-generating procedures such as intubation. We collaborated with original designer HYL and modified the size to adapt to larger patients and operator mobility. We expand its applicability by allowing the use of different instruments. The container is outfitted with an ultra-low particulate air-equipped filtration vacuum device to create negative pressure within the chamber and actively remove floating droplet nuclei generated during a procedure. This barrier method will be a valuable and economical option to protect healthcare workers on the front line globally during this pandemic and beyond.

Htun, H. L., et al. (2020). "Responding to the COVID-19 Outbreak in Singapore: Staff Protection and Staff Temperature and Sickness Surveillance Systems." Clin Infect Dis **71**(8): 1947-1952.

BACKGROUND: Coronavirus disease 2019 (COVID-19) is an emerging infectious disease caused by a novel coronavirus (SARS-CoV-2) and first reported in Wuhan, China, in December 2019. Since the severe acute respiratory syndrome (SARS) outbreak in 2003, Tan Tock Seng Hospital (TTSH) in Singapore has routinely fit-tested staff for high-filtration N95 respirators and established Web-based staff surveillance systems. The routine systems were enhanced in response to Singapore's first imported COVID-19 case on 23 January 2020. METHODS: We conducted a cross-sectional study from 23 January to 23 February 2020 among healthcare workers to evaluate the effectiveness of the staff protection and surveillance strategy in TTSH, a 1600-bed multidisciplinary acute-care hospital colocated with the 330-bed National Centre for Infectious Diseases (NCID). As of 23 February 2020, TTSH/NCID has managed 76% of confirmed COVID-19 cases in Singapore. The hospital adopted a multipronged approach to protect and monitor staff with potential COVID-19 exposures: (1) risk-based personal protective equipment, (2) staff fever and sickness surveillance, and (3) enhanced medical surveillance of unwell staff. RESULTS: A total of 10 583 staff were placed on hospitalwide fever and sickness surveillance, with 1524 frontline staff working in COVID-19 areas under close surveillance. Among frontline staff, a median of 8 staff illness episodes was seen per day; almost 10% (n = 29) resulted in hospitalization. None of the staff was found to be infected with COVID-19. CONCLUSIONS: A robust staff protection and health surveillance system that is routinely implemented during non-outbreak periods and enhanced during the COVID-19 outbreak is effective in protecting frontline staff from the infection.

Hu, K., et al. (2021). Ophthalmic Manifestations Of Coronavirus (COVID-19). StatPearls. Treasure Island (FL), StatPearls Publishing

Copyright © 2021, StatPearls Publishing LLC.

Since December 2019, coronavirus disease 2019 (COVID-19) has become a global pandemic caused by the highly transmissible severe acute respiratory syndrome coronavirus 2 (SARS-CoV-2). There have been several reports of eye redness and irritation in COVID-19 patients, both anecdotal and published, suggesting that conjunctivitis may be an ocular manifestation of SARS-CoV-2 infection.  A study conducted during the 2003 Severe Acute Respiratory Syndrome (SARS) outbreak detected SARS-CoV in tear samples in SARS patients in Singapore. Lack of eye protection was a primary risk factor of SARS-CoV transmission from SARS patients to healthcare workers in Toronto, prompting a concern that respiratory illness could be transmitted through ocular secretions. Similar concerns have been raised with SARS-CoV-2, especially among eye care providers and those on the front lines triaging what could be initial symptoms of COVID-19. As conjunctivitis is a common eye condition, ophthalmologists may be the first medical professionals to evaluate a patient with COVID-19. Indeed, one of the first providers to voice concerns regarding the spread of Coronavirus in Chinese patients was Li Wenliang, MD, an ophthalmologist. He later died from COVID-19 and was believed to have contracted the virus from an asymptomatic glaucoma patient in his clinic. The authors of this article have attempted to collect the most up-to-date information on ophthalmic manifestations of COVID-19 as a resource for identifying symptoms, providing diagnostic pearls, and mitigating transmission.

Hu, X. and W. Huang (2020). "Protecting the psychological well-being of healthcare workers affected by the COVID-19 outbreak: Perspectives from China." Nurs Health Sci **22**(3): 837-838.

Hua, C. G., et al. (2020). "[Strategy of dental clinics to cope with the epidemic period of infectious diseases based on the experience of corona virus disease outbreak]." Hua Xi Kou Qiang Yi Xue Za Zhi **38**(2): 117-121.

The outbreak of corona virus disease (COVID-19) has raised concerns among dentists to develop strategies to prevent infection of dental equipment, materials, and patients during an epidemic period. Strategies following the National Laws and Standards of China and local standards of several provinces for controlling cross-infection and instituting protective measures for medical staff in dental clinics during an epidemic period are discussed. A proposal is put forth for dental clinics that will face similar situations in the future. Further research is warranted to address potential problems that will be encountered under such dire circumstances.

Hua, W., et al. (2020). "Spinal surgery and related management on patients with COVID-19: experience of a regional medical centre in Wuhan." Bone Jt Open **1**(5): 88-92.

During the pandemic of COVID-19, some patients with COVID-19 may need emergency surgeries. As spine surgeons, it is our responsibility to ensure appropriate treatment to the patients with COVID-19 and spinal diseases. A protocol for spinal surgery and related management on patients with COVID-19 has been reviewed. Patient preparation for emergency surgeries, indications, and contraindications of emergency surgeries, operating room preparation, infection control precautions and personal protective equipments (PPE), anesthesia management, intraoperative procedures, postoperative management, medical waste disposal, and surveillance of healthcare workers were reviewed. It should be safe for surgeons with PPE of protection level 2 to perform spinal surgeries on patients with COVID-19. Standardized and careful surgical procedures should be necessary to reduce the exposure to COVID-19.

Huang, F., et al. (2021). "COVID-19 outbreak and healthcare worker behavioural change toward hand hygiene practices." J Hosp Infect.

BACKGROUND: The coronavirus disease (COVID-19) pandemic has affected healthcare workers (HCWs) in their clinical practice. HCWs were challenged with new guidelines and practices to protect themselves from occupational risks. AIM: To determine whether hand hygiene behaviour by real-time measurement was related to the dynamic of the epidemic, and the type of patient being cared for in France. METHODS: This study used an automated hand hygiene recording system to measure HCW hand hygiene on entry to and exit from patient rooms throughout the COVID-19 pandemic. The correlation between hand hygiene compliance and COVID-19 epidemiological data was analysed. Analysis of variance was performed to compare compliance rate during the different periods of the epidemic. FINDINGS: HCW hand hygiene rate on room entry decreased over time; on room exit, it increased by 13.73% during the first wave of COVID-19, decreased by 9.87% during the post-lockdown period, then rebounded by 2.82% during the second wave of the epidemic. Hand hygiene during patient care and hand hygiene on room exit had a positive relationship with the local COVID-19 epidemic; conversely, hand hygiene on room entry did not depend on the trend of the epidemic, nor on nursing of COVID-19 patients, and it decreased over time. CONCLUSION: HCWs modified their behaviours to face the risk propensity of the pandemic. However, to improve the poor compliance at room entry, reducing confusion between the hand hygiene recommendation and glove recommendation may be necessary; disinfection of gloving hands might solve this issue.

Huang, Q., et al. (2021). "Gender Differences in Psychological and Behavioral Responses of Infected and Uninfected Health-Care Workers During the Early COVID-19 Outbreak." Front Public Health **9**: 638975.

Objective: Understanding gender differences in responses of health-care workers (HCWs) to COVID-19 outbreak is an effective way to promote customized supports. Methods: During February 2020, 103 HCWs infected with COVID-19 (64 females and 39 males) and 535 uninfected HCWs (383 females and 152 males) were recruited in a cross-sectional study. Level of attention, six emotional status, and self-evaluation of eight protective measures were recorded. Multivariable Firth's logistic regressions were applied to explored independent effect of gender. Results: During early outbreak, female HCWs were more likely to give greater attention, adjusted OR:1.92 (95%CI 1.14-3.23) in total HCWs. Higher proportion of anxiety was observed in female HCWs, adjusted OR:3.14 (95%CI 1.98-4.99) for total HCWs, 4.32(95%CI 1.32-14.15) for infected HCWs and 2.97 (1.78, 4.95) for uninfected HCWs. Proportion of pessimism, fear, full of fighting spirit, and optimism were low, and no gender differences were observed. During a later outbreak, a majority of HCWs reported being very familiar with eight protective measures. After training, a proportion of high self-evaluation in hand hygiene, wearing gloves, and surgical masks increased independently in female HCWs, and adjusted ORs were 3.07 (95% CI 1.57-5.99), 2.37 (95% CI 1.26-4.49), and 1.92 (95% CI 1.02-3.62), respectively. Infection status amplified gender difference in anxiety, hand hygiene, and glove wearing. Conclusion: Female HCWs perceived the outbreak seriously, effective emotional and psychological well-ness should be targeted at female HCWs preferentially, and male HCWs should be encouraged to express their feelings and be further trained.

Huang, Z., et al. (2020). "Occupational exposure to SARS-CoV-2 in burns treatment during the COVID-19 epidemic: Specific diagnosis and treatment protocol." Biomed Pharmacother **127**: 110176.

Epidemic prevention and control measures for the new coronavirus disease 2019 (COVID-19) has achieved significant results. As of 8 April 2020, 22,073 infection cases of COVID-19 among healthcare workers from 52 countries had been reported to WHO. COVID-19 has strong infectivity, high transmission speeds, and causes serious infection among healthcare worker. Burns are an acute-care condition, and burn treatment needs to be initiated before COVID-19 infection status can be excluded. The key step to infection prevention is to identify risk points of infection exposure, strengthen the protection against those risk points, and formulate an appropriate diagnosis and treatment protocol. Following an in-depth study of the latest literature on COVID-19 diagnosis and treatment, we reviewed the protocols surrounding hospitalization of patients with extensive burns (area≥50 %) in our hospital from February 2009 to February 2019 and, in accordance with the epidemiological characteristics of COVID-19, developed an algorithm for protection during diagnosis and treatment of burns. Therefore, the aspects of medical protection and the diagnosis and treatment of burns appear to be particularly important during the prevention and control of the COVID-19. This algorithm was followed for 4 patients who received emergency treatment in February 2020 and were hospitalized. All healthcare worker were protected according to the three-tiered protective measures, and there was no nosocomial infection. During the COVID-19 epidemic, the early stages of emergency treatment for patients with extensive burns requiring the establishment of venous access for rehydration, endotracheal intubation or tracheostomy, wound treatment, and surgery are the risk points for exposure to infection. The implementation of effective, appropriate-grade protection and formulation of practical treatment protocols can increase protection of healthcare worke and reduce the risk of COVID-19 infection exposure.

Huber, T., et al. (2021). "Principles and practice for SARS-CoV-2 decontamination of N95 masks with UV-C." Biophys J.

A mainstay of personal protective equipment during the coronavirus disease 2019 pandemic is the N95 filtering facepiece respirator. N95 respirators are commonly used to protect healthcare workers from respiratory pathogens, including the novel coronavirus severe acute respiratory syndrome coronavirus 2, and are increasingly employed by other frontline workers and the general public. Under routine circumstances, these masks are disposable, single-use items, but extended use and reuse practices have been broadly enacted to alleviate critical supply shortages during the coronavirus disease 2019 pandemic. Although extended-time single use presents a low risk of pathogen transfer, repeated donning and doffing of potentially contaminated masks presents increased risk of pathogen transfer. Therefore, efficient and safe decontamination methods for N95 masks are needed to reduce the risk of reuse and mitigate local supply shortages. Here, we review the available literature concerning use of germicidal ultraviolet-C (UV-C) light to decontaminate N95 masks. We propose a practical method for repeated point-of-use decontamination using commercially available UV-C cross-linker boxes from molecular biology laboratories to expose each side of the mask to 800-1200 mJ/cm(2) of UV-C. We measure the dose that penetrated to the interior of the respirators and model the potential germicidal action on coronaviruses. Our experimental results, in combination with modeled data, suggest that such a UV-C treatment cycle should induce a >3-log-order reduction in viral bioburden on the surface of the respirators and a 2-log-order reduction throughout the interior. We find that a dose 50-fold greater does not impair filtration or fit of 3M 8210 N95 masks, indicating that decontamination can be performed repeatedly. As such, UV-C germicidal irradiation is a practical strategy for small-scale point-of-use decontamination of N95s.

Huffman, E. M., et al. (2021). "How resilient is your team? Exploring healthcare providers' well-being during the COVID-19 pandemic." Am J Surg **221**(2): 277-284.

BACKGROUND: The global COVID-19 pandemic has placed tremendous physical and mental strain on the US healthcare system. Studies examining the effects of outbreaks have demonstrated both an increased prevalence and long-term development of Post-Traumatic Stress Disorder (PTSD) symptoms in healthcare providers. We sought to assess the impact of the COVID-19 pandemic on the psychological well-being of medical providers, medical trainees, and administrators at a large academic center to identify stressors and moderators to guide future mental health and hospital-system interventions. METHODS: A 42-item survey examining specific stressors, grit, and resilience was widely distributed to physicians, residents, fellows, and administrators a large academic institution for departmental distribution. Survey results were analyzed using descriptive statistics, ANOVA, and multivariate linear regressions. A p-value <0.05 was considered statistically significant. RESULTS: A total of 785 participants completed the survey. The majority of respondents rated their stress to be significantly increased during the pandemic. Respondents' fear of transmitting the virus to their family members was a significant stressor. Higher resilience was associated with lower stress, anxiety, fatigue, and sleep disturbances. Overall, respondents felt supported by their departments and institution and felt contingency plans and personal protective equipment were adequate. CONCLUSIONS: Healthcare workers have increased resilience in the face of heightened stress during a pandemic. Higher resilience and grit were protective factors in managing personal and system-level stressors at the peak of the COVID-19 pandemic in our institution. Implementing an intervention designed to enhance healthcare workers' resilience in response to the COVID-19 pandemic is warranted.

Huh, S. (2020). "How to train health personnel to protect themselves from SARS-CoV-2 (novel coronavirus) infection when caring for a patient or suspected case." J Educ Eval Health Prof **17**: 10.

Husain, S. A., et al. (2021). "Review of hygiene adaptations among UK doctors in controlling the spread of SARS-CoV-2 infection." Clin Med (Lond) **21**(1): e20-e25.

BACKGROUND: A significant number of healthcare workers around the world have contracted COVID-19 from their workplace, thus there is a need to investigate common hygiene practices. AIM: We aimed to describe the common hygiene adaptations of doctors in the hospital and at home. METHODS: A survey of 110 doctors in UK was carried out to determine their hygiene adaptations and practices. Data were collected on demographics and personal protective equipment compliance. RESULTS: The key findings were frequent hand washing (51%), change of clothing entering and leaving hospital (88% and 92%, respectively), taking a shower upon returning home (85%) and washing work attire at temperatures of 60-80°C (50%). A higher proportion of junior doctors washed their scrubs (p=0.004) and stethoscopes (p=0.014) compared with consultants and seniors. Female doctors cleaned their mobile phones (p=0.022) and work belongings (p=0.01) more frequently. CONCLUSION: This study signified that junior doctors were more meticulous in hygiene adaptations and female doctors were more fastidious in personal hygiene. The observations of this study may be beneficial in preventing transmission of infection to families of healthcare professionals and are important to implement in the case of a second wave of COVID-19.

Hussain, M., et al. (2021). "Healthcare workers during the COVID-19 pandemic: Experiences of doctors and nurses in Bangladesh." Int J Health Plann Manage.

Healthcare workers, who are in low-resource settings, are critically vulnerable during the COVID-19 pandemic. The increasing rate of coronavirus infection in a developing country such as Bangladesh caused the highest death rate of doctors among frontline service providers and resulted in fear and anxiety among healthcare workers. Even with the preliminary measures of hospitals and clinics to protect healthcare workers, the growing casualties are alarming. This research uses case study approach to explore the issues doctors and nurses face in 'priority intervention areas' (PIA) in order to improve the health system quality. Qualitative in-depth semi-structured interviews were conducted from 12 May to 4 June 2020 among doctors and nurses from two different private hospitals in Dhaka city. Data were analysed using thematic content analysis. The two significant areas that required immediate attention were identified from the PIA framework as 'patient and staff safety, infection control' and 'cultural aspects and community engagement'. Each area of the PIA framework showed previously ignored issues in the current health system. The adaptation of the PIA framework helped identify critical health system issues. Possible corrective actions including proper planning and management of isolating the infected patients and provision of adequate personal protective equipment are recommended to management and policymakers to save the lives of healthcare workers and to minimise the spread of infection.

Hussain, Z. B., et al. (2020). "Protecting healthcare workers from COVID-19: learning from variation in practice and policy identified through a global cross-sectional survey." Bone Jt Open **1**(5): 144-151.

AIMS: The COVID-19 pandemic presents an unprecedented burden on global healthcare systems, and existing infrastructures must adapt and evolve to meet the challenge. With health systems reliant on the health of their workforce, the importance of protection against disease transmission in healthcare workers (HCWs) is clear. This study collated responses from several countries, provided by clinicians familiar with practice in each location, to identify areas of best practice and policy so as to build consensus of those measures that might reduce the risk of transmission of COVID-19 to HCWs at work. METHODS: A cross-sectional descriptive survey was designed with ten open and closed questions and sent to a representative sample. The sample was selected on a convenience basis of 27 senior surgeons, members of an international surgical society, who were all frontline workers in the COVID-19 pandemic. This study was reported according to the Standards for Reporting Qualitative Research (SRQR) checklist. RESULTS: Responses were received by all 27 surgeons from 22 countries across six continents. A number of the study respondents reported COVID-19-related infection and mortality in HCWs in their countries. Differing areas of practice and policy were identified and organized into themes including the specification of units receiving COVID-19 patients, availability and usage of personal protective equipment (PPE), other measures to reduce staff exposure, and communicating with and supporting HCWs. Areas more specific to surgery also identified some variation in practice and policy in relation to visitors to the hospital, the outpatient department, and in the operating room for both non-urgent and emergency care. CONCLUSION: COVID-19 presents a disproportionate risk to HCWs, potentially resulting in a diminished health system capacity, and consequently an impairment to population health. Implementation of these recommendations at an international level could provide a framework to reduce this burden.

Hwang, J., et al. (2020). "Responding to the COVID-19 pandemic: The role of occupational health services in a tertiary hospital in Singapore." J Occup Health **62**(1): e12172.

With coronavirus disease 2019 declared a Public Health Emergency of International Concern on 30 January 2020, occupational health services in a tertiary hospital in Singapore stepped up via a three-pronged approach, namely, protection of individual staff, protection of staff workforce, and prevention of nosocomial spread so as to support business continuity plans. Despite the multiple new challenges brought by the COVID-19 pandemic, the hospital's occupational health services were able to adapt and keep all employees and patients safe with strong support from senior management and close collaboration with various departments.

Iannone, P., et al. (2020). "The need of health policy perspective to protect Healthcare Workers during COVID-19 pandemic. A GRADE rapid review on the N95 respirators effectiveness." PLoS One **15**(6): e0234025.

Protecting Health Care Workers (HCWs) during routine care of suspected or confirmed COVID-19 patients is of paramount importance to halt the SARS-CoV-2 (Severe Acute Respiratory Syndrome-Coronavirus-2) pandemic. The WHO, ECDC and CDC have issued conflicting guidelines on the use of respiratory filters (N95) by HCWs. We searched PubMed, Embase and The Cochrane Library from the inception to March 21, 2020 to identify randomized controlled trials (RCTs) comparing N95 respirators versus surgical masks for prevention of COVID-19 or any other respiratory infection among HCWs. The grading of recommendations, assessment, development, and evaluation (GRADE) was used to evaluate the quality of evidence. Four RCTs involving 8736 HCWs were included. We did not find any trial specifically on prevention of COVID-19. However, wearing N95 respirators can prevent 73 more (95% CI 46-91) clinical respiratory infections per 1000 HCWs compared to surgical masks (2 RCTs; 2594 patients; low quality of evidence). A protective effect of N95 respirators in laboratory-confirmed bacterial colonization (RR = 0.41; 95%CI 0.28-0.61) was also found. A trend in favour of N95 respirators was observed in preventing laboratory-confirmed respiratory viral infections, laboratory-confirmed respiratory infection, and influenza like illness. We found no direct high quality evidence on whether N95 respirators are better than surgical masks for HCWs protection from SARS-CoV-2. However, low quality evidence suggests that N95 respirators protect HCWs from clinical respiratory infections. This finding should be contemplated to decide the best strategy to support the resilience of healthcare systems facing the potentially catastrophic SARS-CoV-2 pandemic.

Ibáñez-Cervantes, G., et al. (2020). "Disinfection of N95 masks artificially contaminated with SARS-CoV-2 and ESKAPE bacteria using hydrogen peroxide plasma: Impact on the reutilization of disposable devices." Am J Infect Control **48**(9): 1037-1041.

INTRODUCTION: One of the serious consequences of the SARS-CoV-2 pandemic is the shortage of protective equipment for health personnel. N95 masks are considered one of the essential protective equipment in the management of patients with COVID-19. The shortage of N95 masks implies potential health risks for health personnel and significant economic losses for the health institution. The objective of this work was to investigate the disinfection of N95 masks artificially contaminated with SARS-CoV-2 and ESKAPE bacteria by using hydrogen peroxide plasma. MATERIAL AND METHODS: We examined the disinfection capacity of hydrogen peroxide plasma against the SARS-CoV-2 and 2 members of the ESKAPE bacteria (Acinetobacter baumannii and Staphylococcus aureus) through a study of artificial contamination in situ of N95 masks. Amplification of specific genes by real-time reverse transcription polymerase chain reaction of SARS-CoV-2 and microbiological culture of ESKAPE bacteria was performed before and after the disinfection process. RESULTS: SARS-CoV-2 was not detected in all assays using 5 different concentrations of the virus, and A baumannii and S aureus were not cultivable with inoculums of 10(2) to 10(6) CFU after disinfection tests of N95 masks with hydrogen peroxide plasma. CONCLUSION: Disinfection of N95 masks by using the hydrogen peroxide plasma technology can be an alternative for their reuse in a shortage situation. Implications for the use of disinfection technologies of N95 masks and the safety of health personnel are discussed.

Ibrahim, M., et al. (2020). "Comparison of the Effectiveness of Different Barrier Enclosure Techniques in Protection of Healthcare Workers During Tracheal Intubation and Extubation." A A Pract **14**(8): e01252.

The coronavirus disease 2019 (COVID-19; SARS-CoV-2) pandemic has created serious challenges to anesthesiologists. As hospitalized patients' respiratory function deteriorates, many will require endotracheal intubation. Airway management of infected patients risks aerosolization of viral-loaded droplets that pose serious hazards to the anesthesiologist and all health care personnel present. The addition of an enclosure barrier during airway management minimizes the hazard by entrapping the droplets and possibly the aerosols within an enclosed space adding additional protection for health care workers. The aim of this study was to compare the effectiveness of different barrier enclosure techniques during tracheal intubation and extubation.

Idris, M. and E. Nathaniel (2021). "Protective Modifications in Ophthalmology Practice during COVID-19 Pandemic: Challenges and Lessons Learned." J Coll Physicians Surg Pak **30**(1): S32-s34.

Ophthalmology is a specialty which involves close contact with patients. Personal protective equipment (PPE) along with modifications in examination techniques and equipment are needed to avoid spread of coronavirus infectious disease (COVID-19) to health professionals. This communication aims to highlight and critically analyse the measures suggested to control this spread. We also highlighted our experience with protective gear modifications. As with any practice, triage is cornerstone. Use of disinfectants, good personal hygiene practices and PPE for patients and staff, must be adopted for safe ophthalmology practices. Key Words: COVID-19, Ophthalmology, Personal protective equipment (PPE).

Ierardi, A. M., et al. (2020). "Preparation of a radiology department in an Italian hospital dedicated to COVID-19 patients." Radiol Med **125**(9): 894-901.

Preparedness for the ongoing coronavirus disease 2019 (COVID-19) and its spread in Italy called for setting up of adequately equipped and dedicated health facilities to manage sick patients while protecting healthcare workers, uninfected patients, and the community. In our country, in a short time span, the demand for critical care beds exceeded supply. A new sequestered hospital completely dedicated to intensive care (IC) for isolated COVID-19 patients needed to be designed, constructed, and deployed. Along with this new initiative, the new concept of "Pandemic Radiology Unit" was implemented as a practical solution to the emerging crisis, born out of a critical and urgent acute need. The present article describes logistics, planning, and practical design issues for such a pandemic radiology and critical care unit (e.g., space, infection control, safety of healthcare workers, etc.) adopted in the IC Hospital Unit for the care and management of COVID-19 patients.

Ilesanmi, O. S. and O. F. Fagbule (2020). "REALITIES OF COVID-19 PANDEMIC IN NIGERIA: COPING WITH THE CHALLENGES AND CHARTING A WAY FORWARD." Ann Ib Postgrad Med **18**(1): 78-84.

The impacts of COVID-19 have cut across both developed and developing countries alike. This has prompted different coping mechanisms for survival through these trying times. The reality of stigmatization in this period has negatively affected health-seeking behavior and public response to the outbreak. Challenges exist at both the community and the healthcare providing facilities. We present a review of best practices in coping with COVID-19. Our focus is on how to cope with the challenges and strategies to improve the response to the pandemic in Nigeria. It is required that safety measures be fully adopted and practiced by individuals and groups. Collaborative efforts are required by all stakeholders, government, healthcare workers, and private organizations to mitigate the negative effects of the pandemic, avoid stigmatization, and ease the return journey to normalcy.

Imai, H. (2020). "Trust is a key factor in the willingness of health professionals to work during the COVID-19 outbreak: Experience from the H1N1 pandemic in Japan 2009." Psychiatry Clin Neurosci **74**(5): 329-330.

Imamura, R., et al. (2021). "Impact of the COVID-19 Pandemic on Physicians Working in the Head and Neck Field." Int Arch Otorhinolaryngol **25**(1): e150-e159.

Background  With the COVID-19 pandemic, the clinical practice of physicians who work in the head and neck field in Brazil dropped dramatically. The sustained impact of the pandemic is not known. Methods  An anonymous online survey was distributed to Brazilian otolaryngologists, head and neck surgeons, medical and radiation oncologists, asking about their clinical practice in the third to fourth months of the pandemic. Results  The survey was completed by 446 specialists. About 40% reported reduction of more than 75% in outpatient care. A reduction of 90% to 100% in airway endoscopies was reported by 50% of the responders, and the same rate of reduction regarding surgeries (pediatric or nasosinusal) was reported by 80% of them. Family income decreased by 50%, and the psychological burden on physicians was considerable. The availability of personal protective equipment and safety precautions were limited, especially in the public sector. Conclusion  COVID-19 is still impacting the head and neck field, and safety concerns may hinder the prompt resumption of elective care.

Inglis, R., et al. (2020). "Pragmatic Recommendations for Safety while Caring for Hospitalized Patients with Coronavirus Disease 2019 (COVID-19) in Low- and Middle-Income Countries." Am J Trop Med Hyg **104**(3 Suppl): 12-24.

Infection prevention and control measures to control the spread of COVID-19 are challenging to implement in many low- and middle-income countries (LMICs). This is compounded by the fact that most recommendations are based on evidence that mainly originates in high-income countries. There are often availability, affordability, and feasibility barriers to applying such recommendations in LMICs, and therefore, there is a need for developing recommendations that are achievable in LMICs. We used a modified version of the GRADE method to select important questions, searched the literature for relevant evidence, and formulated pragmatic recommendations for safety while caring for patients with COVID-19 in LMICs. We selected five questions related to safety, covering minimal requirements for personal protective equipment (PPE), recommendations for extended use and reuse of PPE, restriction on the number of times healthcare workers enter patients' rooms, hand hygiene, and environmental ventilation. We formulated 21 recommendations that are feasible and affordable in LMICs.

Inglis, T. J. J. and K. Mathee (2021). "JMM Profile: Severe acute respiratory syndrome coronavirus 2 (SARS-CoV-2)." J Med Microbiol **70**(3).

The severe acute respiratory syndrome coronavirus 2 (SARS-CoV-2) virus is the cause of an infection known as coronavirus infectious disease 2019 (COVID-19). COVID-19 has become a global source of morbidity, mortality and social disruption since its emergence in East Asia in late 2019 and subsequent pandemic spread. Typical symptoms include cough, sore throat, fever, and sudden loss of taste and smell. Persistent, post-infection sequelae have been noted in a minority of cases. Severe complications and deaths occur mostly in older adults. Laboratory confirmation can be performed by viral RNA and antigen detection in nasal swabs or by detecting specific neutralizing antibodies. There is no effective and approved antiviral treatment, but several vaccines with favourable safety and efficacy profiles are being used in mass vaccination programmes. Vaccine-based COVID control should be seen as an addition to existing hygiene measures such as physical distancing, increased hand hygiene, cough etiquette, and barrier protection with personal protective equipment for frontline healthcare workers and other high-risk professions.

Ingrassia, P. L., et al. (2020). "COVID-19 crisis, safe reopening of simulation centres and the new normal: food for thought." Adv Simul (Lond) **5**: 13.

BACKGROUND: The world is facing a massive burden from the coronavirus disease 2019 (COVID-19) pandemic. Governments took the extraordinary step of locking down their own countries to curb the spread of the coronavirus. After weeks of severe restrictions, countries have begun to relax their strict lockdown measures. However, reopening will not be back to normal.Simulation facilities (SF) are training spaces that enable health professionals and students to learn skills and procedures in a safe and protected environment. Today's clinicians and students have an expectation that simulation laboratories are part of lifelong healthcare education. There is great uncertainty about how COVID-19 will impact future training in SF. In particular, the delivery of training activities will benefit of adequate safety measures implemented for all individuals involved.This paper discusses how to safely reopen SF in the post-lockdown phase. MAIN BODY: The paper outlines 10 focus points and provides operational tips and recommendations consistent with current international guidelines to reopen SF safely in the post-lockdown phase. Considering a variety of national advices and regulations which describe initial measures for the reopening of workplaces as well as international public health recommendations, we provide points of reflection that can guide decision-makers and SF leaders on how to develop local approaches to specific challenges. The tips have been laid out taking also into account two main factors: (a) the SF audience, mainly consisting of undergraduate and postgraduate healthcare professionals, who might face exposure to COVID-19 infection, and (b) for many simulation-based activities, such as teamwork training, adequate physical distancing cannot be maintained. CONCLUSIONS: The planning of future activities will have to be based not only on safety but also on flexibility principles.Sharing common methods consistent with national and international health guidelines, while taking into account the specific characteristics of the different contexts and centres, will ultimately foster dissemination of good practices.This article seeks to further the conversation. It is our hope that this manuscript will prompt research about the impact of such mitigation procedures and measures in different countries.

Ioannidis, J. P. A. (2020). "Global perspective of COVID-19 epidemiology for a full-cycle pandemic." Eur J Clin Invest **50**(12): e13423.

As of October 2020, there are >1 million documented deaths with COVID-19. Excess deaths can be caused by both COVID-19 and the measures taken. COVID-19 shows extremely strong risk stratification across age, socioeconomic factors, and clinical factors. Calculation of years-of-life-lost from COVID-19 is methodologically challenging and can yield misleading over-estimates. Many early deaths may have been due to suboptimal management, malfunctional health systems, hydroxychloroquine, sending COVID-19 patients to nursing homes, and nosocomial infections; such deaths are partially avoidable moving forward. About 10% of the global population may be infected by October 2020. Global infection fatality rate is 0.15-0.20% (0.03-0.04% in those <70 years), with large variability across locations with different age-structure, institutionalization rates, socioeconomic inequalities, population-level clinical risk profile, public health measures, and health care. There is debate on whether at least 60% of the global population must be infected for herd immunity, or, conversely, mixing heterogeneity and pre-existing cross-immunity may allow substantially lower thresholds. Simulations are presented with a total of 1.58-8.76 million COVID-19 deaths over 5-years (1/2020-12/2024) globally (0.5-2.9% of total global deaths). The most favorable figures in that range would be feasible if high risk groups can be preferentially protected with lower infection rates than the remaining population. Death toll may also be further affected by potential availability of effective vaccines and treatments, optimal management and measures taken, COVID-19 interplay with influenza and other health problems, reinfection potential, and any chronic COVID-19 consequences. Targeted, precise management of the pandemic and avoiding past mistakes would help minimize mortality.

Ippolito, M., et al. (2021). "Personal protective equipment use by healthcare workers in intensive care unit during the early phase of COVID-19 pandemic in Italy: a secondary analysis of the PPE-SAFE survey." Ther Adv Infect Dis **8**: 2049936121998562.

BACKGROUND: Italy was the first Western country to be heavily affected by COVID-19. Healthcare workers (HCWs) were exposed to a high risk of occupational infection, partially due to insufficient personal protective equipment (PPE) supplies. This study aimed to describe the practices, availability, training, confidence in PPE use and the adverse effects due to extended PPE use, as reported by HCWs in Italy. We also aimed to provide a comparison between Italian data and those from other countries. METHODS: This study was a secondary analysis of a previously published international study, the PPE-SAFE Survey, conducted in April 2020. Data were analysed from the original study database. RESULTS: We analysed the responses from 380 healthcare workers based in Italy, out of the 2711 respondents to the international survey. Among the Italian respondents, FFP2 and FFP3 respirators or equivalent were the most used masks for routine tasks (respectively 188/380, 50%; and 163/380, 43%). The median time of wearing PPE without taking a break was 5 h [interquartile range (IQR) 4-6], with statistically significant difference from other countries [median 4 h (IQR 2-5) p < 0.0001]. In Italy, 249 out of 380 (65%) HCWs had never performed a formal fit test for a N95 mask or equivalent and 91/380 (24%) never had a partner for donning and doffing procedures. Most of the respondents (299/380, 79%) had received formal training in PPE use at any time. CONCLUSION: Most of the surveyed Italian HCWs reported working at above usual capacity, long shifts with PPE without breaks and routine use in intensive care unit of aerosol protection (e.g. FFP2/FFP3), hazmat suits and face shields/visors. The correct adherence to safety procedures (e.g. donning/doffing in pairs, performing fit test) has substantial scope for improvement in the future.

Ippolito, M., et al. (2020). "Medical masks and Respirators for the Protection of Healthcare Workers from SARS-CoV-2 and other viruses." Pulmonology **26**(4): 204-212.

The use of medical masks and respirators as personal protective equipment is pivotal to reducing the level of biological hazard to which healthcare workers are exposed during the outbreak of highly diffusible pathogens, such as the recent novel coronavirus SARS-CoV-2. Unfortunately, during this pandemic, supplies are rapidly running out worldwide, with potential consequences for the rate of occupational infections. Also, knowledge about specific characteristics of respirators is of utmost importance to select the proper type according to the clinical setting. A wide variety of literature is available on the topic, but mostly based on Influenza viruses infection models. Clinical evidence on the use of respirators is poor and interest in the topic has not been constant over time. A better understanding of SARS-CoV-2 transmission is needed, together with high-quality clinical data on the use of respirators or alternative devices. Moreover, healthcare workers, regardless of their level of experience, should receive specific training. This review aims to summarize the available evidence on the use of medical masks and respirators in the context of viral infections, especially the current coronavirus disease 2019 (COVID-19).

Iserson, K. V. (2020). "Healthcare Ethics During a Pandemic." West J Emerg Med **21**(3): 477-483.

As clinicians and support personnel struggle with their responsibilities to treat during the current COVID-19 pandemic, several ethical issues have emerged. Will healthcare workers and support staff fulfill their duty to treat in the face of high risks? Will institutional and government leaders at all levels do the right things to help alleviate healthcare workers risks and fears? Will physicians be willing to make hard, resource-allocation decisions if they cannot first husband or improvise alternatives?With our healthcare facilities and governments unprepared for this inevitable disaster, front-line doctors, advanced providers, nurses, EMS, and support personnel struggle with acute shortages of equipment-both to treat patients and protect themselves. With their personal and possibly their family's lives and health at risk, they must weigh the option of continuing to work or retreat to safety. This decision, made daily, is based on professional and personal values, how they perceive existing risks-including available protective measures, and their perception of the level and transparency of information they receive. Often, while clinicians get this information, support personnel do not, leading to absenteeism and deteriorating healthcare services. Leadership can use good risk communication (complete, widely transmitted, and transparent) to align healthcare workers' risk perceptions with reality. They also can address the common problems healthcare workers must overcome to continue working (ie, risk mitigation techniques). Physicians, if they cannot sufficiently husband or improvise lifesaving resources, will have to face difficult triage decisions. Ideally, they will use a predetermined plan, probably based on the principles of Utilitarianism (maximizing the greatest good) and derived from professional and community input. Unfortunately, none of these plans is optimal.

Iskanderian, R. R., et al. (2020). "Outcomes and Impact of a Universal COVID-19 Screening Protocol for Asymptomatic Oncology Patients." Gulf J Oncolog **1**(34): 7-12.

INTRODUCTION: COVID-19 has become a serious hazard worldwide in a relatively short time. Scientific evidence supports that cancer patients infected with COVID-19 had a higher risk of developing severe complications. COVID-19 patients can be asymptomatic during part or all of their disease course, therefore it is a compelling need to develop universal pre-interventional COVID-19 screening guidelines. The aim of this study is to is review COVID-19 positive rate among asymptomatic cancer patients since the implementation of universal policy at our institution, and assess the impact of diagnosing COVID-19 on delay of oncologic interventions. MATERIALS AND METHODS: The study population comprised of all cancer patients planned for high risk interventions between April 1, 2020 - May 14, 2020 at Cleveland Clinic Abu Dhabi [CCAD] after implementing universal COVID-19 screening policy. DISCUSSION: Nosocomial transmission of COVID-19 among cancer patients could result in poor outcomes. Universal screening for high-risk populations may facilitate earlier diagnosis of COVID-19 and implementation of control strategies. Our review demonstrated that [7.5%] of asymptomatic cancer patients tested positive for COVID-19 after implementation of universal prospective screening policy. The overall evidence supporting universal COVID-19 screening of cancer patients is limited, yet as tests become more widely available, it may be reasonable to screen all cancer patients for COVID-19 before anticancer therapies. While such policy may delay and affect the timing of anticancer therapy as shown in this report, it should improve the safety of care for oncology patients and help protect healthcare workers. CONCLUSION: COVID-19 infection rate is higher in cancer patients than general population and can present without symptoms. Universal COVID-19 screening of cancer patients before high risk interventions is supported by the present findings.

Islam, M. S., et al. (2020). "Current knowledge of COVID-19 and infection prevention and control strategies in healthcare settings: A global analysis." Infect Control Hosp Epidemiol **41**(10): 1196-1206.

OBJECTIVE: In the current absence of a vaccine for COVID-19, public health responses aim to break the chain of infection by focusing on the mode of transmission. We reviewed the current evidence on the transmission dynamics and on pathogenic and clinical features of COVID-19 to critically identify any gaps in the current infection prevention and control (IPC) guidelines. METHODS: In this study, we reviewed global COVID-19 IPC guidelines by organizations such as the World Health Organization (WHO), the US Centers for Disease Control and Prevention (CDC), and the European Centre for Disease Prevention and Control (ECDC). Guidelines from 2 high-income countries (Australia and United Kingdom) and from 1 middle-income country (China) were also reviewed. We searched publications in English on 'PubMed' and Google Scholar. We extracted information related to COVID-19 transmission dynamics, clinical presentations, and exposures that may facilitate transmission. We then compared these findings with the recommended IPC measures. RESULTS: Nosocomial transmission of SARS-CoV-2 in healthcare settings occurs through droplets, aerosols, and the oral-fecal or fecal-droplet route. However, the IPC guidelines fail to cover all transmission modes, and the recommendations also conflict with each other. Most guidelines recommend surgical masks for healthcare providers during routine care and N95 respirators for aerosol-generating procedures. However, recommendations regarding the type of face mask varied, and the CDC recommends cloth masks when surgical masks are unavailable. CONCLUSION: IPC strategies should consider all the possible routes of transmission and should target all patient care activities involving risk of person-to-person transmission. This review may assist international health agencies in updating their guidelines.

Ivanyi, P., et al. (2021). "Protective measures for patients with advanced cancer during the Sars-CoV-2 pandemic: Quo vadis?" Clin Exp Metastasis: 1-5.

Cancer patients represent a vulnerable cohort during the Sars-CoV-2 pandemic. Oncological societies have generated a plethora of recommendations, but precise instructions about routine oncological procedures remain scarce. Here, we report on local COVID-19 protection measures established in an interdisciplinary approach at a tertiary care center during the first wave of the pandemia in Germany. Following these measures, no additional morbidity or mortality during oncological procedures was observed, and no nosocomial infections were registered. However, Validation of our measures is outstanding and regional SARS-CoV-2 prevalence was low. However, specific oncological measures might be important to ensure optimal oncological results, especially for advanced cancer stages during this and future pandemia. In the future, communication about these measures might be crucial to a cancer patient´s assigned network to reduce the danger of excess mortality within the second wave of the COVID-19 pandemic.

Ivy, C. C., et al. (2021). "The impact of COVID-19 on hand therapy practice." J Hand Ther.

BACKGROUND: Hand therapists and health care providers across the spectrum have been profoundly impacted by COVID-19. Greater insight and information regarding how practitioners have been affected by this unparalleled pandemic is important. PURPOSE: Survey research was performed to examine the impact of the COVID-19 pandemic on hand therapy practice. STUDY DESIGN: Online survey research. METHODS: Four constructs guided the development of the survey: psychosocial and financial impact; safety practice patterns; changes in current practice patterns; use of telehealth. The survey was distributed to members of the American Society of Hand Therapists from April 14, 2020 through May 4, 2020. Descriptive demographic data were obtained. Frequencies were examined using ChiSquare, correlations were examined using Spearman Correlation Coefficient, and means were compared via independent t-test. RESULTS: A total of 719 members responded to the survey. Eighty-six percent of therapists reported feeling more stress than they did prior to the COVID-19 pandemic. This level of stress was similar across ages, practice settings, financial stability or instability, and geographical settings. Older therapists (r(s) = 0.04) and those that practiced longer (r(s) = 0.009) felt more comfortable with in-person treatment. Ninety-eight percent of therapists reported a decrease in caseload. Postoperative cases (P= .0001) and patients ages 19-49 were more likely to receive in-person treatment (P= .002). 46% of therapists reported providing telehealth services. Nontraumatic, nonoperative cases (P= .0001) and patients aged 65 or older were more likely to receive telehealth services (P= .0001). Younger therapists (r(s) = 0.03) and therapists working in outpatient therapist owned, outpatient corporate owned, and outpatient academic medical centers (X(2) [4, N = 637] = 15.9463, P= .003) were more likely to utilize telehealth. CONCLUSION: Stress was felt globally among hand therapy clinicians regardless of financial security or insecurity, age, practice area, or geographical setting. Therapists saw a drastic decrease in caseloads. In-person caseloads shifted primarily to postoperative cases. STUDY DESIGN: Web based survey.

Iwu, C. J., et al. (2020). "Cochrane corner: personal protective equipment for preventing highly infectious diseases such as COVID-19 in healthcare staff." Pan Afr Med J **37**: 148.

As coronavirus disease (COVID-19) cases continue to increase in Africa, healthcare workers (HCWs) have a high risk of being infected and the risks may be higher among those who work closely with patients. The risks of HCW infections can be mitigated with adequate precautions within healthcare facilities, especially with the use of personal protective equipment (PPE). We highlight and contextualise the findings of a Cochrane review on the type of PPE that protects best, the best way to put PPE on (donning) or to remove PPE (doffing) and how to train HCWs to use PPE. The review found low-certainty of evidence that full body PPE offer more protection, but HCWs may be faced with difficulty during donning and doffing. Following standard guidelines may be helpful in reducing infection and increasing compliance among HCWs. Video training and simulations may be better methods for training on the correct use of PPE than traditional methods of teaching. Countries must, therefore, ensure that HCWs undergo compulsory training on the correct use of PPE; regardless of their professional category. Of the 24 studies included in this review, none was conducted on the African continent. There is thus an urgent need for well conducted studies on the experiences of HCWs using full-body covering PPE within the African context. Such studies could lead to tailored interventions that will improve the proper use of PPE among HCWs.

Iyengar, K. P., et al. (2020). "COVID-19 and mortality in doctors." Diabetes Metab Syndr **14**(6): 1743-1746.

BACKGROUND AND AIMS: COVID-19 disease appear to have been associated with significant mortality amongst doctors and health care workers globally. We explore the various risk factors associated with this occupational risk, especially focusing on India. This may elucidate lessons to protect these frontline workers during the COVID-19 pandemic. METHODS: We carried out a comprehensive review of the literature using suitable keywords such as 'COVID-19', 'pandemics', 'physicians' 'mortality' and 'health personnel' on the search engines of PubMed, SCOPUS, Google Scholar and ResearchGate in the month of July 2020 during the current COVID-19 pandemic and assessed mortality data. RESULTS: Mortality in health care professionals has been on the rise. The countries which faced the pandemic in the early months of 2020 have had a huge surge in mortality amongst doctors due to COVID-19. India continues to show a rising trend in COVID-19 cases, however although compared to the western world India has seen a comparatively favourable statistic. Male gender, elderly doctors and those belonging to Black, Asian, and Minority Ethnic (BAME) community seem to be predisposing factors in the western world. CONCLUSION: COVID-19 has been associated with an increased mortality in doctors and health care workers. Until an effective cure/vaccine is developed, risk assessments at work, mitigating confounding factors, adequate supply of personal protective equipment (PPE) and enhanced protection against infection are necessary to protect health care professionals on the coronavirus frontline. Otherwise this occupational risk can lead to further untimely mortality and become another unintended consequence of the COVID-19 pandemic.

Jain, K., et al. (2020). "Protocolized approach to a COVID-19 parturient undergoing a cesarean section - A case report." J Anaesthesiol Clin Pharmacol **36**(3): 407-410.

The health care workers working in the operation theater (OT) face many unique challenges to deal with the corona virus disease (COVID-19) patients undergoing surgery. We hereby report the management of a COVID-19 parturient undergoing cesarean section in the dedicated COVID health center. Patient care of this special and vulnerable subset of population with least exposure of COVID-19 to health care worker was the cornerstone of the management. We have summarized some of the important precautionary measures which were taken during cesarean section to minimize exposure and genuine use of resources in this pandemic. To conclude, despite Personal Protective Equipment (PPE), precautionary measures and strategies are of utmost importance and should be opted so as to lower the virus contagion risk.

Jarmoliński, T., et al. (2020). "SARS-CoV-2 viral clearance during bone marrow aplasia after allogeneic hematopoietic stem cell transplantation-A case report." Pediatr Transplant: e13875.

Respiratory viral infections are known causes of mortality after allogeneic hematopoietic stem cell transplantation (HSCT). Here, we report a unique case of a child with viral pneumonia caused by coinfection with human metapneumovirus (MPV), respiratory syncytial virus (RSV), and SARS-CoV-2 after HSCT. A 9-year-old girl with acute lymphoblastic leukemia underwent allogeneic HSCT from a matched, unrelated donor. During the post-transplant period, in profound leukopenia (below 10 leukocytes/µL), she was diagnosed with SARS-CoV-2, MPV, and RSV pneumonia and was treated with ribavirin and chloroquine. Before leukocyte recovery, the girl became asymptomatic, and SARS-CoV-2 and RSV clearance was achieved. The shedding of SARS-CoV-2 stopped before immune system recovery, and one may hypothesize that the lack of an inflammatory response might have been a contributing factor to the mild clinical course. Post-transplant care in HSCT recipients with COVID-19 infection is feasible in regular transplant units, provided the patient does not present with respiratory failure. Early and repeated testing for SARS-CoV-2 in post-transplant patients with concomitant infection mitigation strategies should be considered in children after HSCT who develop fever, respiratory symptoms, and perhaps gastrointestinal symptoms to control the spread of COVID-19 both in patients and in healthcare workers in hospital environments. Training of staff and the availability of personal protective equipment are crucial for containing SARS-CoV-2 infection.

Jazuli, F., et al. (2020). "Endotracheal intubation with barrier protection." Emerg Med J **37**(7): 398-399.

Given the high risk of healthcare worker (HCW) infection with COVID-19 during aerosol-generating medical procedures, the use of a box barrier during intubation for protection of HCWs has been examined. Previous simulation work has demonstrated its efficacy in protecting HCWs from cough-expelled droplets. Our objective was to assess its ability to protect HCWs against aerosols generated during aerosol-generating medical procedures. We used a battery-powered vapouriser to assess movement of vapour with: (1) no barrier; (2) a box barrier; and (3) a box barrier and a plastic sheet covering the box and patient's body. We visualised the trajectory of vapour and saw that the vapour remained within the barrier space when the box barrier and plastic sheet were used. This is in contrast to the box barrier alone, where vapour diffused towards the feet of the patient and throughout the room, and to no barrier where the vapour immediately diffused to the laryngoscopist. This demonstrates that the box with the plastic sheet has the potential to limit the spread of aerosols towards the laryngoscopist, and thus may play a role in protecting HCWs during aerosol-generating medical procedures. This is of particular importance in the care of patients with suspected COVID-19.

Jefferson, T., et al. (2020). "Physical interventions to interrupt or reduce the spread of respiratory viruses." Cochrane Database Syst Rev **11**: Cd006207.

BACKGROUND: Viral epidemics or pandemics of acute respiratory infections (ARIs) pose a global threat. Examples are influenza (H1N1) caused by the H1N1pdm09 virus in 2009, severe acute respiratory syndrome (SARS) in 2003, and coronavirus disease 2019 (COVID-19) caused by SARS-CoV-2 in 2019. Antiviral drugs and vaccines may be insufficient to prevent their spread. This is an update of a Cochrane Review published in 2007, 2009, 2010, and 2011. The evidence summarised in this review does not include results from studies from the current COVID-19 pandemic. OBJECTIVES: To assess the effectiveness of physical interventions to interrupt or reduce the spread of acute respiratory viruses. SEARCH METHODS: We searched CENTRAL, PubMed, Embase, CINAHL on 1 April 2020. We searched ClinicalTrials.gov, and the WHO ICTRP on 16 March 2020. We conducted a backwards and forwards citation analysis on the newly included studies. SELECTION CRITERIA: We included randomised controlled trials (RCTs) and cluster-RCTs of trials investigating physical interventions (screening at entry ports, isolation, quarantine, physical distancing, personal protection, hand hygiene, face masks, and gargling) to prevent respiratory virus transmission. In previous versions of this review we also included observational studies. However, for this update, there were sufficient RCTs to address our study aims.   DATA COLLECTION AND ANALYSIS: We used standard methodological procedures expected by Cochrane. We used GRADE to assess the certainty of the evidence. Three pairs of review authors independently extracted data using a standard template applied in previous versions of this review, but which was revised to reflect our focus on RCTs and cluster-RCTs for this update. We did not contact trialists for missing data due to the urgency in completing the review. We extracted data on adverse events (harms) associated with the interventions. MAIN RESULTS: We included 44 new RCTs and cluster-RCTs in this update, bringing the total number of randomised trials to 67. There were no included studies conducted during the COVID-19 pandemic. Six ongoing studies were identified, of which three evaluating masks are being conducted concurrent with the COVID pandemic, and one is completed. Many studies were conducted during non-epidemic influenza periods, but several studies were conducted during the global H1N1 influenza pandemic in 2009, and others in epidemic influenza seasons up to 2016. Thus, studies were conducted in the context of lower respiratory viral circulation and transmission compared to COVID-19. The included studies were conducted in heterogeneous settings, ranging from suburban schools to hospital wards in high-income countries; crowded inner city settings in low-income countries; and an immigrant neighbourhood in a high-income country. Compliance with interventions was low in many studies. The risk of bias for the RCTs and cluster-RCTs was mostly high or unclear. Medical/surgical masks compared to no masks We included nine trials (of which eight were cluster-RCTs) comparing medical/surgical masks versus no masks to prevent the spread of viral respiratory illness (two trials with healthcare workers and seven in the community). There is low certainty evidence from nine trials (3507 participants) that wearing a mask may make little or no difference to the outcome of influenza-like illness (ILI) compared to not wearing a mask (risk ratio (RR) 0.99, 95% confidence interval (CI) 0.82 to 1.18. There is moderate certainty evidence that wearing a mask probably makes little or no difference to the outcome of laboratory-confirmed influenza compared to not wearing a mask (RR 0.91, 95% CI 0.66 to 1.26; 6 trials; 3005 participants). Harms were rarely measured and poorly reported. Two studies during COVID-19 plan to recruit a total of 72,000 people. One evaluates medical/surgical masks (N = 6000) (published Annals of Internal Medicine, 18 Nov 2020), and one evaluates cloth masks (N = 66,000). N95/P2 respirators compared to medical/surgical masks We pooled trials comparing N95/P2 respirators with medical/surgical masks (four in healthcare settings and one in a household setting). There is uncertainty over the effects of N95/P2 respirators when compared with medical/surgical masks on the outcomes of clinical respiratory illness (RR 0.70, 95% CI 0.45 to 1.10; very low-certainty evidence; 3 trials; 7779 participants) and ILI (RR 0.82, 95% CI 0.66 to 1.03; low-certainty evidence; 5 trials; 8407 participants). The evidence is limited by imprecision and heterogeneity for these subjective outcomes. The use of a N95/P2 respirator compared to a medical/surgical mask probably makes little or no difference for the objective and more precise outcome of laboratory-confirmed influenza infection (RR 1.10, 95% CI 0.90 to 1.34; moderate-certainty evidence; 5 trials; 8407 participants). Restricting the pooling to healthcare workers made no difference to the overall findings. Harms were poorly measured and reported, but discomfort wearing medical/surgical masks or N95/P2 respirators was mentioned in several studies. One ongoing study recruiting 576 people compares N95/P2 respirators with medical surgical masks for healthcare workers during COVID-19. Hand hygiene compared to control Settings included schools, childcare centres, homes, and offices. In a comparison of hand hygiene interventions with control (no intervention), there was a 16% relative reduction in the number of people with ARIs in the hand hygiene group (RR 0.84, 95% CI 0.82 to 0.86; 7 trials; 44,129 participants; moderate-certainty evidence), suggesting a probable benefit. When considering the more strictly defined outcomes of ILI and laboratory-confirmed influenza, the estimates of effect for ILI (RR 0.98, 95% CI 0.85 to 1.13; 10 trials; 32,641 participants; low-certainty evidence) and laboratory-confirmed influenza (RR 0.91, 95% CI 0.63 to 1.30; 8 trials; 8332 participants; low-certainty evidence) suggest the intervention made little or no difference. We pooled all 16 trials (61,372 participants) for the composite outcome of ARI or ILI or influenza, with each study only contributing once and the most comprehensive outcome reported. The pooled data showed that hand hygiene may offer a benefit with an 11% relative reduction of respiratory illness (RR 0.89, 95% CI 0.84 to 0.95; low-certainty evidence), but with high heterogeneity. Few trials measured and reported harms. There are two ongoing studies of handwashing interventions in 395 children outside of COVID-19. We identified one RCT on quarantine/physical distancing. Company employees in Japan were asked to stay at home if household members had ILI symptoms. Overall fewer people in the intervention group contracted influenza compared with workers in the control group (2.75% versus 3.18%; hazard ratio 0.80, 95% CI 0.66 to 0.97). However, those who stayed at home with their infected family members were 2.17 times more likely to be infected. We found no RCTs on eye protection, gowns and gloves, or screening at entry ports. AUTHORS' CONCLUSIONS: The high risk of bias in the trials, variation in outcome measurement, and relatively low compliance with the interventions during the studies hamper drawing firm conclusions and generalising the findings to the current COVID-19 pandemic. There is uncertainty about the effects of face masks. The low-moderate certainty of the evidence means our confidence in the effect estimate is limited, and that the true effect may be different from the observed estimate of the effect. The pooled results of randomised trials did not show a clear reduction in respiratory viral infection with the use of medical/surgical masks during seasonal influenza. There were no clear differences between the use of medical/surgical masks compared with N95/P2 respirators in healthcare workers when used in routine care to reduce respiratory viral infection. Hand hygiene is likely to modestly reduce the burden of respiratory illness. Harms associated with physical interventions were under-investigated. There is a need for large, well-designed RCTs addressing the effectiveness of many of these interventions in multiple settings and populations, especially in those most at risk of ARIs.

Jeon, Y. W., et al. (2020). "Protection of Healthcare Workers Against COVID-19 at a Large Teaching Hospital in Seoul, Korea." Yonsei Med J **61**(7): 631-634.

Thirteen patients with coronavirus disease 2019 (COVID-19) visited a university hospital in Seoul before recognizing their disease infections, causing contact with 184 hospital workers. We classified the patients into four risk levels and provided corresponding management measures. At 31 days after the last event, all screening laboratory results were negative, and no symptoms/signs were reported.

Jereczek-Fossa, B. A., et al. (2020). "COVID-19 outbreak and cancer radiotherapy disruption in Italy: Survey endorsed by the Italian Association of Radiotherapy and Clinical Oncology (AIRO)." Radiother Oncol **149**: 89-93.

Italy experienced one of the world's deadliest COVID-19 outbreaks and healthcare systems had to instantly reorganise activity. The Italian Radiation Oncology Departments adapted numerous solutions to minimize the disruptions. Information technologies, treatment prioritization and implementation of hypofractionation and protection procedures allowed balancing between cancer patient care and patient/healthcare workers safety.

Jewkes, S. V., et al. (2020). "Nosocomial spread of COVID-19: lessons learned from an audit on a stroke/neurology ward in a UK district general hospital." Clin Med (Lond) **20**(5): e173-e177.

We describe the details of a COVID-19 outbreak in a 25-bedded Birmingham neurology/stroke ward in the early phase of the pandemic (March to May 2020). Twenty-one of 133 admissions (16%) tested positive for COVID-19 and of those, 8 (6% of all admissions to the ward) were determined to be nosocomial. Thus 38% (8/21) of COVID-19 infections were hospital-acquired. Ten of the patients that contracted COVID-19 died; of these three were hospital-acquired cases. Five of the 21 patients had negative swabs prior to receiving a positive test result. This study highlights the importance of appropriate use of personal protective equipment (PPE) with high-risk patients (including those with stroke and complex brain injury with tracheostomies) and the difficulties of COVID-19 management in a high-risk patient population.

Jha, S. K. (2020). "Physiological Effects of N95 FFP and Personal Protective Equipment in Healthcare Workers in COVID ICU: A Prospective Cohort Study." Indian J Crit Care Med **24**(12): 1156-1157.

How to cite this article: Jha SK. Physiological Effects of N95 FFP and Personal Protective Equipment in Healthcare Workers in COVID ICU: A Prospective Cohort Study. Indian J Crit Care Med 2020;24(12):1156-1157.

Jia, J., et al. (2021). "Application of an optimized pharyngeal swab-assisted device in pharyngeal sampling for COVID-19 patients." Ann Transl Med **9**(4): 319.

BACKGROUND: Coronavirus disease 2019 (COVID-19), which is caused by severe acute respiratory syndrome coronavirus 2 (SARS-CoV-2), has triggered a global pandemic. Healthcare workers are placed at an elevated risk of nosocomial cross-infection from clinical exposure. One diagnostic criterion for COVID-19 is a positive result from a real-time reverse transcriptase-polymerase chain reaction (qRT-PCR) assay of pharyngeal swab specimens, which has been a routine procedure for healthcare workers during the outbreak. In the context of a global shortage of personal protective equipment (PPE), we aimed to lower the probability of clinical cross-infection without impacting the results of pharynx sampling through an optimized pharyngeal swab assisted device (OPAD). METHODS: To evaluate the efficacy and feasibility of an OPAD for the detection of SARS-CoV-2, 22 confirmed COVID-19 cases were enrolled in our self-controlled study. The results of two pharyngeal sampling qRT-PCR tests using the OPAD or the traditional method were recorded each. Clinical data including baseline characteristics, laboratory tests, and computed tomography (CT) results were also collected. The procedure duration and levels of pharynx exposure with the OPAD, and the diagnostic consistency between the OPAD and the traditional method for pharyngeal sampling qRT-PCR, were evaluated individually. Additionally, a questionnaire was designed for healthcare workers who had performed the pharyngeal swab to deepen our understanding of their attitude during their service on the frontline. RESULTS: In all 44 samplings (22 samples with each method), the qRT-PCR results of 18 pairs (81.82%) were consistent, while 3 (13.64%) were single positive with the OPAD. The positive rate was slightly higher with the OPAD (54.55%, 12/22) than with the traditional method (45.45%, 10/22). Using the OPAD, the average procedure duration of sampling was 30 s (30±13 s). Pharynx exposure was excellent in 21 subjects (95.45%, 21/22), which meant that the operator could acquire the swabs without difficulty. CONCLUSIONS: As the COVID-19 pandemic escalates, our OPAD has identical efficacy compared to the traditional method for pharyngeal swabs, and it can also contribute to protecting the safety of healthcare workers.

Jian, M., et al. (2021). "Changes in Neuroanesthesia Practice During the Early Stages of the COVID-19 Pandemic: Experiences From a Single Center in China." J Neurosurg Anesthesiol **33**(1): 73-76.

BACKGROUND: Coronavirus disease 2019 (COVID-19), caused by a novel coronavirus, is highly contagious. Global medical systems have been heavily impacted by the COVID-19 pandemic. Although the majority of patients with intracranial disease require time-sensitive surgery, how to conduct neurosurgery and prevent and control nosocomial infection during a pandemic is challenging. MATERIALS AND METHODS: We retrospectively reviewed the clinical data of patients undergoing neurosurgical and neurointerventional procedures at Beijing Tiantan Hospital, China during the early stages of the COVID-19 pandemic between January 21 and July 31, 2020. A 3-level system of COVID-19 risk was established based on medical conditions, epidemiologic, and symptom inquiry and the results of triage. A transitional unit was established for patients in whom COVID-19 had not been ruled out on admission to hospital. RESULTS: A total of 4025 patients underwent neurosurgery during the study period, including 768 emergent and 3257 nonemergent procedures. Of these patients, 3722 were low-risk for COVID-19, 303 were moderate-risk, and none were high-risk. In addition, 1419 patients underwent neurointerventional procedures, including 114 emergent and 1305 nonemergent interventions, of which 1339 were low-risk patients, 80 were moderate-risk and none were high-risk. A total of 895 patients (neurosurgical and neurointerventional) were admitted to the transitional unit. Forty-five patients were diagnosed with COVID-19 and transferred to the COVID-19 designated hospital. There were no cases of COVID-19 nosocomial infections among surgical patients or health care workers. CONCLUSION: On the basis of our single-center experience, developing a full screening protocol for COVID-19, establishing a risk level, and using a transitional unit for those with unknown COVID-19 status are effective measures to provide a safe environment for patients and health care workers.

Jiang, H., et al. (2020). "Emergency management in fever clinic during the outbreak of COVID-19: an experience from Zhuhai." Epidemiol Infect **148**: e174.

Coronavirus disease 2019 (COVID-19) is a global health threat. A hospital in Zhuhai adopted several measures in Fever Clinic Management (FCM) to respond to the outbreak of COVID-19. FCM has been proved to be effective in preventing nosocomial cross infection. Faced with the emergency, the hospital undertook creative operational steps in relation to the control and spread of COVID-19, with special focuses on physical and administrative layout of buildings, staff training and preventative procedures. The first operational step was to set up triaging stations at all entrances and then complete a standard and qualified fever clinic, which was isolated from the other buildings within our hospital complex. Secondly, the hospital established its human resource reservation for emergency response and the allocation of human resources to ensure strict and standardised training methods through the hospital for all medical staff and ancillary employees. Thirdly, the hospital divided the fever clinic into partitioned areas and adapted a three-level triaging system. The experiences shared in this paper would be of practical help for the facilities that are encountering or will encounter the challenges of COVID-19, i.e. to prevent nosocomial cross infection among patients and physicians.

Jin, S., et al. (2021). "The Resumption of Sports Medicine During the COVID-19 Post-Epidemic Period: Experiences from Wuhan, People's Republic of China." J Bone Joint Surg Am **103**(1): 10-14.

Coronavirus disease 2019 (COVID-19) is spreading worldwide, with its outlook not looking optimistic. Simultaneously, the epidemic is currently under control in many areas. The resumption of work and production in areas that have achieved control of outbreaks is a problem. Considering the extremely transmissible nature of COVID-19, and the presence of asymptomatic infected people, avoiding nosocomial infection and protecting medical staff and patients during the post-epidemic period remain difficult problems that need to be solved. At present, few articles have examined relevant experiences in the field of sports medicine. Wuhan, the People's Republic of China, was the original epicenter of COVID-19, with physicians as the initial frontline workers. Wuhan is now gradually returning to a more normal state after a series of urgent, strict, and effective measures were utilized to combat the epidemic. During this time, we collected first-hand experiences of sports medicine work resumption in the initial 2-month period, including preparations before resuming work, outpatient management methods, online outpatient services, inpatient ward management, principles for determination of the examination results, and preparations for operations. The strict and feasible management strategies that we conducted were useful in avoiding hospital-based infections. We intend to share our own experiences and thoughts in this area. We hope that this will be helpful and inspiring to our sports medicine colleagues around the world.

Jin, Y. H., et al. (2020). "Perceived infection transmission routes, infection control practices, psychosocial changes, and management of COVID-19 infected healthcare workers in a tertiary acute care hospital in Wuhan: a cross-sectional survey." Mil Med Res **7**(1): 24.

BACKGROUND: Many healthcare workers were infected by coronavirus disease 2019 (COVID-19) early in the epidemic posing a big challenge for epidemic control. Hence, this study aims to explore perceived infection routes, influencing factors, psychosocial changes, and management procedures for COVID-19 infected healthcare workers. METHODS: This is a cross-sectional, single hospital-based study. We recruited all 105 confirmed COVID-19 healthcare workers in the Zhongnan Hospital of Wuhan University from February 15 to 29, 2020. All participants completed a validated questionnaire. Electronic consent was obtained from all participants. Perceived causes of infection, infection prevention, control knowledge and behaviour, psychological changes, symptoms and treatment were measured. RESULTS: Finally, 103 professional staff with COVID-19 finished the questionnaire and was included (response rate: 98.1%). Of them, 87 cases (84.5%) thought they were infected in working environment in hospital, one (1.0%) thought their infection was due to the laboratory environment, and 5 (4.9%) thought they were infected in daily life or community environment. Swab of throat collection and physical examination were the procedures perceived as most likely causing their infection by nurses and doctors respectively. Forty-three (41.8%) thought their infection was related to protective equipment, utilization of common equipment (masks and gloves). The top three first symptoms displayed before diagnosis were fever (41.8%), lethargy (33.0%) and muscle aches (30.1%). After diagnosis, 88.3% staff experienced psychological stress or emotional changes during their isolation period, only 11.7% had almost no emotional changes. Arbidol (Umifenovir; an anti-influza drug; 69.2%) was the drug most commonly used to target infection in mild and moderate symptoms. CONCLUSION: The main perceived mode of transmission was not maintaining protection when working at a close distance and having intimate contact with infected cases. Positive psychological intervention is necessary.

John, A. R., et al. (2020). "Scalable in-hospital decontamination of N95 filtering face-piece respirator with a peracetic acid room disinfection system." Infect Control Hosp Epidemiol: 1-10.

BACKGROUND: Critical shortages of personal protective equipment, especially N95 respirators, during the coronavirus disease 2019 (COVID-19) pandemic continues to be a source of concern. Novel methods of N95 filtering face-piece respirator decontamination that can be scaled-up for in-hospital use can help address this concern and keep healthcare workers (HCWs) safe. METHODS: A multidisciplinary pragmatic study was conducted to evaluate the use of an ultrasonic room high-level disinfection system (HLDS) that generates aerosolized peracetic acid (PAA) and hydrogen peroxide for decontamination of large numbers of N95 respirators. A cycle duration that consistently achieved disinfection of N95 respirators (defined as ≥6 log10 reductions in bacteriophage MS2 and Geobacillus stearothermophilus spores inoculated onto respirators) was identified. The treated masks were assessed for changes to their hydrophobicity, material structure, strap elasticity, and filtration efficiency. PAA and hydrogen peroxide off-gassing from treated masks were also assessed. RESULTS: The PAA room HLDS was effective for disinfection of bacteriophage MS2 and G. stearothermophilus spores on respirators in a 2,447 cubic-foot (69.6 cubic-meter) room with an aerosol deployment time of 16 minutes and a dwell time of 32 minutes. The total cycle time was 1 hour and 16 minutes. After 5 treatment cycles, no adverse effects were detected on filtration efficiency, structural integrity, or strap elasticity. There was no detectable off-gassing of PAA and hydrogen peroxide from the treated masks at 20 and 60 minutes after the disinfection cycle, respectively. CONCLUSION: The PAA room disinfection system provides a rapidly scalable solution for in-hospital decontamination of large numbers of N95 respirators during the COVID-19 pandemic.

Johnson, H., et al. (2021). "COVID-19 (SARS-CoV-2) in Non-Airborne body fluids: A systematic review & Meta-analysis." Turk J Urol **47**(2): 87-97.

Severe acute respiratory syndrome coronavirus 2 (SARS-CoV-2) pandemic has been predominantly respiratory. This study aimed to evaluate the presence of virus in non-airborne body fluids as transmission vehicles. Medline, EMBASE, and Cochrane Library databases were searched from December 01, 2019, to July 01, 2020, using terms relating to SARS-CoV-2 and non-airborne clinical sample sources (feces, urine, blood, serum, serum, and peritoneum). Studies in humans, of any design, were included. Risk of bias assessment was performed using the Quality Assessment of Diagnostic Accuracy 2 tool. Preferred Reporting Items for Systematic Reviews & Meta-Analyses) guidelines were used for abstracting data. If ≥5 studies reported proportions for the same non-respiratory site, a meta-analysis was conducted using either a fixed or random-effects model, depending on the presence of heterogeneity. A total of 22 studies with 648 patients were included. Most were cross-sectional and cohort studies. The SARS-CoV-2 RNA was most frequently detected in feces. Detectable RNA was reported in 17% of the blood samples, 8% of the serum, 16% in the semen, but rarely in urine. Prevalence of SARS-CoV-2 in non-airborne sites varies widely with a third of non-airborne fluids. Patients with bowel and non-specific symptoms have persistence of virus in feces for upto 2 weeks after symptom resolution. Although there was a very low detection rate in urine, given the more frequent prevalence in blood samples, the presence of SARS-CoV-2 in patients with disrupted urothelium or undergoing urinary tract procedures, is likely to be higher. Healthcare providers need to consider non-airborne transmission and persistence of SARS-CoV-2 in body fluids to enable appropriate precautions to protect healthcare workers and carers.

Jones, B., et al. (2020). "COVID-19 pandemic: The impact on vulnerable children and young people in Australia." J Paediatr Child Health **56**(12): 1851-1855.

The COVID-19 pandemic and associated system disruptions are impacting all children and young people (CYP) in Australia. For vulnerable groups of CYP, who already experience poorer health and well-being, these impacts are amplified. Challenges include reduced access to usual services, reduced community supports, financial instability, unemployment and other life circumstances that threaten to widen pre-existing inequities. This article aims to present the reasons for vulnerability of CYP during the pandemic, and to focus on actions by health professionals that mitigate additional challenges to their health and well-being. Using a rapid review of the literature and team-based discussions, eight vulnerable groups were identified: CYP with disabilities, mental health conditions and chronic diseases; CYP facing financial hardship; within the child protection system; Aboriginal; migrant and refugee; in residential care; rural; and isolated CYP. Recommendations for action are required at the level of governments, health professionals and researchers and include enhancing access to health and social supports, prioritising vulnerable CYP in resuming health activity and elevating the voice of CYP in designing the response. The pandemic can be conceptualised as an opportunity to create a more equitable society as we document the inequities that have been exacerbated. Vulnerable groups of CYP must be recognised and heard, and targeted actions must focus on improving their health outcomes during the pandemic and beyond.

Jones, M. S., et al. (2020). "Inpatient Transition to Virtual Care During COVID-19 Pandemic." Diabetes Technol Ther **22**(6): 444-448.

Introduction: During the coronavirus disease 2019 (COVID-19) outbreak, novel approaches to diabetes care have been employed. Care in both the inpatient and outpatient setting has transformed considerably. Driven by the need to reduce the use of personal protective equipment and exposure for patients and providers alike, we transitioned inpatient diabetes management services to largely "virtual" or remotely provided care at our hospital. Methods: Implementation of a diabetes co-management service under the direction of the University of North Carolina division of endocrinology was initiated in July 2019. In response to the COVID-19 pandemic, the diabetes service was largely transitioned to a virtual care model in March 2020. Automatic consults for COVID-19 patients were implemented. Glycemic outcomes from before and after transition to virtual care were evaluated. Results: Data over a 15-week period suggest that using virtual care for diabetes management in the hospital is feasible and can provide similar outcomes to traditional face-to-face care. Conclusion: Automatic consults for COVID-19 patients ensure that patients with serious illness receive specialized diabetes care. Transitioning to virtual care models does not limit the glycemic outcomes of inpatient diabetes care and should be employed to reduce patient and provider exposure in the setting of COVID-19. These findings may have implications for reducing nosocomial infection in less challenging times and might address shortage of health care providers, especially in the remote areas.

Jones, N. K., et al. (2021). "Single-dose BNT162b2 vaccine protects against asymptomatic SARS-CoV-2 infection." Elife **10**.

The BNT162b2 mRNA COVID-19 vaccine (Pfizer-BioNTech) is being utilised internationally for mass COVID-19 vaccination. Evidence of single-dose protection against symptomatic disease has encouraged some countries to opt for delayed booster doses of BNT162b2, but the effect of this strategy on rates of asymptomatic SARS-CoV-2 infection remains unknown. We previously demonstrated frequent pauci- and asymptomatic SARS-CoV-2 infection amongst healthcare workers (HCWs) during the UK's first wave of the COVID-19 pandemic, using a comprehensive PCR-based HCW screening programme (Rivett et al., 2020; Jones et al., 2020). Here, we evaluate the effect of first-dose BNT162b2 vaccination on test positivity rates, and find a four-fold reduction in asymptomatic infection amongst HCWs ≥12 days post-vaccination. These data provide real-world evidence of short-term protection against asymptomatic SARS-CoV-2 infection following a single dose of BNT162b2 vaccine, suggesting that mass first-dose vaccination will reduce SARS-CoV-2 transmission, as well as the burden of COVID-19 disease.

Jose, S., et al. (2021). "Health Problems and Skin Damages Caused by Personal Protective Equipment: Experience of Frontline Nurses Caring for Critical COVID-19 Patients in Intensive Care Units." Indian J Crit Care Med **25**(2): 134-139.

BACKGROUND: In the event of coronavirus disease-2019 (COVID-19) spread worldwide, frontline healthcare workers play a key role in the containment of this devastating pandemic, and to prevent the cross-transmission and gain confidence in battle with the pandemic, they are wearing personal protective equipment (PPE). AIM AND OBJECTIVE: To explore the adverse health problems and skin reactions caused by the use of PPEs among the frontline nurses in the ICUs of COVID hospital. MATERIALS AND METHODS: A cross-sectional study was conducted using an online-based questionnaire assessing the physical problems, and adverse skin reactions of PPEs were sent among the 150 frontline nurses in ICUs of COVID hospital. The collected data were analyzed using descriptive statistics. RESULTS: We got 137 valid responses from frontline nurses, and the most common adverse health effects expressed by them were headache (73.4%), extreme sweating (59.6%), and difficulty in breathing (36.7%); 91.7% complained about the fogging of the goggle. Majority of frontline nurses expressed nasal bridge scarring (76.64%) and indentation and pain on the back of the ears (66.42%) as the adverse skin reactions after wearing N95 masks. The common skin problems identified due to double gloving of latex gloves were excessive skin soakage with sweat (70.07%) and skin chapping (19%). The protective clothing caused minimal adverse reactions, and excessive sweating (71.53%) was the most reported. CONCLUSION: The healthcare workers wearing PPE for a prolonged period show significant adverse effects, so appropriate strategies should be taken to prevent the adverse effects by designing effective PPEs and education of preventive measures among healthcare workers. HOW TO CITE THIS ARTICLE: Jose S, Cyriac MC, Dhandapani M. Health Problems and Skin Damages Caused by Personal Protective Equipment: Experience of Frontline Nurses Caring for Critical COVID-19 Patients in Intensive Care Units. Health Problems and Skin Damages Caused by Personal Protective Equipment: Experience of Frontline Nurses Caring for Critical COVID-19 Patients in Intensive Care Units. Indian J Crit Care Med 2021;25(2):134-139.

Jumlongkul, A. and P. Chutivongse (2020). "Robotic-Assisted Surgery for Cadaveric Skull Opening: A New Method of Autopsy Procedure." Front Robot AI **7**: 622083.

Background: Sawing of bone is an essential part of an autopsy procedure. An oscillating saw always generates noise, fine infectious dust particles, and the possibility of traumatic injuries, all of which can induce occupational hazard risks to autopsy workers, especially during the COVID-19 pandemic. Objectives: The first goal of this study was to explore the production of noise and bone dust emission, comparing an oscillating saw and a robotic autopsy saw during an autopsy. The second goal was to evaluate the performance of a new robotic autopsy method, used during skull opening. The third goal was to encourage mortuary workers to use robotic technology during the autopsy procedure to protect us away from occupational injuries as well as airborne infections. Materials and Methods: The experiments involved a comparison of noise levels and aerosol production during skull cutting between the oscillating saw and the robotic autopsy saw. Results: The results confirmed that noise production from the robotic autopsy saw was lower than the oscillating saw. However, the bone dust levels, produced by the robotic autopsy saw, were greater than the oscillating saw, but were not greater than the dust concentrations which were present before opening the skull. Conclusions: The use of a new robotic system might be an alternative choice for protecting against occupational damage among the healthcare workers. Further research might attempt to consider other healthcare problems which occur in the autopsy workplace and apply the robotic-assisted technology in autopsy surgery.

Jung, J., et al. (2020). "Contamination of personal protective equipment by SARS-CoV-2 during routine care of patients with mild COVID-19." J Infect **81**(2): e165-e167.

Kadali, R. A. K., et al. (2021). "Adverse effects of COVID-19 mRNA-1273 vaccine: A randomized, cross-sectional study on healthcare workers with detailed self-reported symptoms." J Med Virol.

There are concerns regarding the side effects of the new COVID-19 mRNA-1273 vaccine among healthcare workers (HCWs) in the United States OBJECTIVE: To investigate the side effects of the mRNA-1273 vaccine with detailed review of organ systems METHODS: A randomized, cross-sectional study using an independent online survey questionnaire with responses from HCWs RESULTS: Of all participants, 87.8% (1116/1271) provided complete responses. Of them, 38.7 % (432/1116) received the mRNA-1273 vaccine, among which, 89.35% were females. 425 of these 432 mRNA-1273 vaccine recipients (98.34%) reported at least one or more symptoms. The results were classified based on the frequency of symptoms reported post vaccination. Of these, 254/432 (58.8%) were able to continue their daily routine activities. 108/432 (25%) were temporarily unable to perform daily activities, 120/432 (27.78%) required transient time off from work, 17/432 (3.94%) required help from an outpatient provider, 1/432 (0.23%) required help from emergency department, and none of them were hospitalized. Despite the wide array of self-reported symptoms, 97.02% of the HCWs did not intend to skip the second dose of vaccine CONCLUSIONS: Among all the symptoms reported, localized pain, generalized weakness, headache, myalgia, chills, fever, nausea, joint pains, sweating, localized swelling at the injection site, dizziness, itching, rash, decreased appetite, muscle spasm, decreased sleep quality, and brain fogging/confusion are the most commonly reported symptoms (in descending order of occurrence). Most of the symptoms reported were non-life threatening. Despite the wide array of self-reported symptoms, there appears to be a higher acceptance for the vaccine This article is protected by copyright. All rights reserved.

Kadoya, Y., et al. (2021). "Knowledge, perception, and level of confidence regarding COVID-19 care among healthcare workers involved in cardiovascular medicine: a web-based cross-sectional survey in Japan." J Cardiol **77**(3): 239-244.

BACKGROUND: The pandemic of coronavirus disease 2019 (COVID-19) has a significant impact on daily practice in cardiovascular medicine. The preparedness of healthcare workers (HCWs) can affect the spread of infection and the maintenance of the healthcare system. This study aimed to investigate the knowledge, perception, and level of confidence regarding COVID-19 care among HCWs involved in cardiovascular medicine. METHODS: A cross-sectional, web-based study about COVID-19 was performed between April 22 and May 7, 2020, among 311 HCWs in cardiovascular departments. The demographic information, COVID-19-related knowledge, and perception and level of confidence toward COVID-19 care were assessed. RESULTS: The median age of the participants was 38 years, and 215 (69.8%) were male. There were 134 (43.1%) physicians and 177 (56.9%) non-physician HCWs. The HCWs, especially non-physician HCWs, had insufficient knowledge about infection-prevention measures for COVID-19, such as how to isolate patients with COVID-19, how to use personal protective equipment, and how to prevent infection during aerosol-generating procedures. Most HCWs showed a low level of confidence toward COVID-19 care, and such poor confidence was associated with the lack of knowledge on optimal infection-prevention measures. CONCLUSIONS: This survey revealed the lack of knowledge about adequate infection-prevention measures for COVID-19. More attention should be paid to the preparedness of HCWs, and educating and supporting HCWs involved in cardiovascular medicine is an urgent need.

Kalaitzaki, A. (2021). "Posttraumatic symptoms, posttraumatic growth, and internal resources among the general population in Greece: A nation-wide survey amid the first COVID-19 lockdown." Int J Psychol.

Scarce and inconclusive evidence exists on the mental health consequences of the COVID-19 lockdown. This study examined the psychological impact of the lockdown in Greece, resilience levels, use of coping strategies, and identified high-risk groups. A sample of 1661 participants (mean age = 39.5, SD = 12.2; 75.5% females) completed a web-based survey, which was distributed through social networking sites, webpages, and personal contacts. Posttraumatic symptoms, posttraumatic growth, resilience, and coping strategies were assessed. Different population subgroups suffered the impact of lockdown disproportionately. Healthcare workers, females, younger, less educated, and those living alone reported higher rates of posttraumatic stress symptoms. Females achieved more posttraumatic growth and were using coping strategies more frequently than men. Men, older, healthcare workers, and those with a partner were more resilient. Interventions need to be developed to target personal resources, protect vulnerable populations, facilitate posttraumatic growth, and ameliorate wellbeing and quality of life.

Kalimuddin, S., et al. (2021). "Early T cell and binding antibody responses are associated with Covid-19 RNA vaccine efficacy onset." Med (N Y).

BACKGROUND: RNA vaccines against Covid-19 have demonstrated ∼95% efficacy in Phase III clinical trials. Although complete vaccination consisted of two-doses, the onset of protection for both licensed RNA vaccines was observed as early as 12 days after a single dose. The adaptive immune response that coincides with this onset of protection could represent the necessary elements of immunity against Covid-19. METHODS: Serological and T cell analysis was performed in a cohort of 20 healthcare workers after receiving the first dose of Pfizer/BioNTech BNT162b2 vaccine. The primary endpoint was the adaptive immune responses detectable at Days 7 and 10 after dosing. RESULTS: Spike-specific T cells and binding antibodies were detectable 10 days after the first dose of vaccine, in contrast to receptor-blocking and SARS-CoV-2 neutralising antibodies which were mostly undetectable at this early time-point. CONCLUSIONS: Our findings suggest that early T cell and binding antibody responses, rather than either receptor-blocking or virus neutralizing activity, induced early protection against Covid-19. FUNDING: The study was funded by a generous donation from The Hour Glass to support Covid-19 research.

Kampf, G., et al. (2020). "COVID-19-associated shortage of alcohol-based hand rubs, face masks, medical gloves, and gowns: proposal for a risk-adapted approach to ensure patient and healthcare worker safety." J Hosp Infect **105**(3): 424-427.

The coronavirus disease 2019 (COVID-19) pandemic has caused a huge demand for alcohol-based hand rubs, medical gloves, face masks, and gowns in healthcare and from the public. More and more hospitals face a serious shortage of these articles. We propose a risk-adapted approach to ensure adequate patient and healthcare worker safety for as long as possible.

Kanu, S., et al. (2021). "Healthcare Workers' Knowledge, Attitude, Practice and Perceived Health Facility Preparedness Regarding COVID-19 in Sierra Leone." J Multidiscip Healthc **14**: 67-80.

BACKGROUND: Healthcare workers (HCWs) are known to spearhead the fight against the COVID-19 pandemic. As such, their knowledge, attitude, and practice (KAP) toward coronavirus disease 2019 (COVID-19) are considered critical to the success of the current COVID-19 response efforts. This study aims to determine HCWs' KAP toward COVID-19 and assesses their perception of their healthcare facilities preparedness to respond appropriately to the ongoing COVID-19 pandemic in Sierra Leone. METHODS: We conducted an online cross-sectional study among HCWs (n=516) between 1st May 2020 and 30th June 2020. We collected our data using a self-administered structured questionnaire via email and online social media platforms. We analyzed our data using descriptive statistics and regression analysis (p<0.05). RESULTS: Close to three-fourth of HCWs (n=375, 72.7%) were knowledgeable regarding COVID-19. Doctors were more knowledgeable than community health workers and laboratory technicians (AOR= 2.48, 95% CI: 1.16-5.31, p=0.019) regarding COVID-19. Close to two-thirds of HCWs (n=301, 58.3%) HCWs show positive attitudes toward COVID-19. Being male (AOR=2.08, 95% CI: 1.36-3.20, p=0.001) and directly involved in COVID-19 patient care (AOR=3.21, 95% CI: 1.88-5.48, p<0.001) were identified as predictors of positive attitude towards COVID-19. HCWs are generally adhering to COVID-19 safe practices with majority indicating that they regularly wash or sanitize their hands (n=510, 98.8%) and used facemask at point of care (n=499, 96.7%). Majority of HCWs are of the view that their healthcare facilities are ill-prepared to adequately respond to COVID-19 with majority (n= 400, 77.5%) of them stating that their facilities lack enough personal protective equipment. CONCLUSION: HCWs in Sierra Leone showed good knowledge, positive attitude and practice regarding COVID-19. However, HCWs are of the view that their healthcare facilities are ill-prepared to respond adequately to the COVID-19 outbreak. Health authorities and policymakers need to provide the necessary resources to allow HCWs to work in a safe environment.

Kapoor, A., et al. (2021). "Breathability and Safety Testing of Personal Protective Equipment: "Human-comfort" Factor Remains Undefined." Indian J Crit Care Med **25**(1): 12-15.

Healthcare systems all over the world have been enormously affected by the COVID-19 pandemic. Healthcare workers (HCWs) taking care of these patients need personal protective equipments (PPEs) standardized for full protection from droplets and aerosols carrying viral load to variable distances. There has been a surge of manufacturers supplying these protective gears in India and regulatory agencies have issued technical specifications pertaining to PPEs focusing solely on synthetic blood penetration tests (SBPTs) and keeping the upper limit of non-woven fabric to 95 g/m(2) (GSM). These PPE specifications are silent on air permeability (AP) and water/moisture vapor transmission rate (WVTR/MVTR) of the fabric. As a result, most of the PPE kits, despite having appropriate SBPT certifications from regulatory agencies, have extremely poor permeability and breathability. The acceptability of PPEs by HCWs can be vastly improved when the end-users are proactively invited to participate in "comfort testing" of PPEs before getting issuance of certification for marketing. "Field testing" or "end-user trials" in which HCWs don the PPE and assess it for comfort while performing different types of clinical work, e.g., in intensive care units (ICUs), operation theaters, cath labs, etc., also takes into account a hitherto often ignored "human-comfort-factor" that not only enhances the understanding of HCWs about the need for the PPEs but can also motivate them to use it without worrying about discomfort. We hereby propose that comfort fit testing (COmfort and Material Fit is an Obviously Required Test) should be a part of the mandatory testing and certification process for PPE, so that the industry invests wisely in manufacturing PPE kits that are not only certified for fabric but are also tested for comfort factors. How to cite this article: Kapoor A, Baronia AK, Azim A, Agarwal G, Prasad N, Mishra R, et al. Breathability and Safety Testing of Personal Protective Equipment: "Human-comfort" Factor Remains Undefined. Indian J Crit Care Med 2021;25(1):12-15.

Kapoor, M. C. (2020). "Respiratory and cardiovascular effects of COVID-19 infection and their management." J Anaesthesiol Clin Pharmacol **36**(Suppl 1): S21-s28.

The COVID-19 epidemic has put an enormous burden on the health-care system and the economy. The virus has very high infectivity and is crippling in patients developing severe disease. The disease caused by this infective agent, a novel RNA coronavirus (SARS-CoV-2), was named by the World Health Organization as COVID-19. SARS-CoV-2 usually enters the human body from the respiratory tract and gradually causes systemic disease. The disease is mild in 81% and severe in the balance. The virus causes multiorgan damage and primarily damages airway epithelium, small intestine epithelium, and vascular endothelium, which are organs with high angiotensin-converting enzyme (angiotensin-converting enzyme-2 [ACE2] expression). The most affected organ is the lungs, and the cardiovascular system follows it closely. Symptomatic hypoxic patients are initially treated with oxygen supplementation, but those with severe hypoxia need mechanical ventilation support. Patients with COVID-19 infection present as two phenotypes. The ventilation strategy should be based on the phenotype. The disease causes major hemodynamic disturbances in its invasion of the cardiovascular system. Strict personal protection protocols are needed to ensure the safety of health-care workers and nosocomial spread.

Kara, E., et al. (2020). "Knowledge and Attitudes Among Hospital Pharmacists About COVID-19." Turk J Pharm Sci **17**(3): 242-248.

OBJECTIVES: Coronavirus (COVID-19) cases and deaths related to the virus have been reported all over the world. Pharmacists play an important role in conveying accurate information about COVID-19 to the community. The aim of this study was to evaluate the knowledge and attitudes among hospital pharmacists about COVID-19. MATERIALS AND METHODS: A questionnaire was distributed to pharmacists participating in the 7th National Hospital and Institution Pharmacists Congress. The questions included in this questionnaire were created using the Turkish COVID-19 Scientific Committee guideline (COVID-19 Guideline). RESULTS: Analysis of 237 questionnaires (72.6% completed by women) showed that the media (television, newspaper), internet (nonscientific resources), internet (scientific resources), and social media were the most popular sources of information (60.3%, 53.6%, 53.2%, and 41.4%, respectively). The participants' age and the source of information that they used had an important influence on their knowledge of and attitudes towards COVID-19 infection. The majority of the participants (72.6%) stated that they were not wearing any kind of mask. Transmission of the disease by airborne route was well known by the participants (91.1%), as well as the main symptoms such as fever (92.4%), cough (84.4%), and dyspnea (60.3%). The participants were aware of the risk groups for COVID-19 infection such as advanced age (84.8%) and having comorbidities (80.2%). Washing hands with soap (92.0%), using hand disinfectants (80.6%), and avoiding contact with sick people (81.9%) were popular answers for protection from the disease, but wearing an N95 mask was also mentioned by 59.1% of the participants. Prevention of the disease by rinsing the nose with saline solution was believed in by 43.9% of the participants. CONCLUSION: Classical media and social media affect the attitudes of both the public and health professionals. Using media tools for accurate information is one of the basic conditions for preventing and controlling the spread of the disease.

Karampelias, V., et al. (2021). "Precaution Measures for the Safety of the Personnel in the Surgical Wards during the COVID-19 Pandemic." J Hand Surg Asian Pac Vol **26**(1): 127-129.

Coronavirus disease-19 has affected million of people worldwide, constituting the biggest social, economic, and health crisis since World War 2. During this pandemic, the hospitals have become hot zones for the treatment of patients. Therefore, it is important to take the appropriate protective measures and ensure the physician's health and, especially, those who work in the intensive care units and in operating rooms. In this letter, we are trying to make a discussion regarding the measures that should be considered by the healthcare workers who are facing this invisible enemy during their effort to provide their services in the surgery rooms.

Karim, N., et al. (2020). "Sustainable Personal Protective Clothing for Healthcare Applications: A Review." ACS Nano **14**(10): 12313-12340.

Personal protective equipment (PPE) is critical to protect healthcare workers (HCWs) from highly infectious diseases such as COVID-19. However, hospitals have been at risk of running out of the safe and effective PPE including personal protective clothing needed to treat patients with COVID-19, due to unprecedented global demand. In addition, there are only limited manufacturing facilities of such clothing available worldwide, due to a lack of available knowledge about relevant technologies, ineffective supply chains, and stringent regulatory requirements. Therefore, there remains a clear unmet need for coordinating the actions and efforts from scientists, engineers, manufacturers, suppliers, and regulatory bodies to develop and produce safe and effective protective clothing using the technologies that are locally available around the world. In this review, we discuss currently used PPE, their quality, and the associated regulatory standards. We survey the current state-of-the-art antimicrobial functional finishes on fabrics to protect the wearer against viruses and bacteria and provide an overview of protective medical fabric manufacturing techniques, their supply chains, and the environmental impacts of current single-use synthetic fiber-based protective clothing. Finally, we discuss future research directions, which include increasing efficiency, safety, and availability of personal protective clothing worldwide without conferring environmental problems.

Kasloff, S. B., et al. (2021). "Stability of SARS-CoV-2 on critical personal protective equipment." Sci Rep **11**(1): 984.

The spread of COVID-19 in healthcare settings is concerning, with healthcare workers representing a disproportionately high percentage of confirmed cases. Although SARS-CoV-2 virus has been found to persist on surfaces for a number of days, the extent and duration of fomites as a mode of transmission, particularly in healthcare settings, has not been fully characterized. To shed light on this critical matter, the present study provides the first comprehensive assessment of SARS-CoV-2 stability on experimentally contaminated personal protective equipment (PPE) widely used by healthcare workers and the general public. Persistence of viable virus was monitored over 21 days on eight different materials, including nitrile medical examination gloves, reinforced chemical resistant gloves, N-95 and N-100 particulate respirator masks, Tyvek, plastic, cotton, and stainless steel. Unlike previous reports, viable SARS-CoV-2 in the presence of a soil load persisted for up to 21 days on experimentally inoculated PPE, including materials from filtering facepiece respirators (N-95 and N-100 masks) and a plastic visor. Conversely, when applied to 100% cotton fabric, the virus underwent rapid degradation and became undetectable by TCID(50) assay within 24 h. These findings underline the importance of appropriate handling of contaminated PPE during and following use in high-risk settings and provide interesting insight into the potential utility of cotton in limiting COVID-19 transmission.

Kassir, G., et al. (2021). "Psychological distress experienced by self-quarantined undergraduate university students in Lebanon during the COVID-19 outbreak." Int J Psychiatry Clin Pract: 1-8.

INTRODUCTION: Quarantine, although essential during contagious outbreaks, has been correlated with poor psychological outcomes in the general population. Such outcomes include low mood, suicide, and post-traumatic stress symptoms. Studies have mostly looked at the mental health of general citizens, healthcare workers, or infected survivors, with limited research targeting university students. This study aimed to understand the psychological distress experienced by self-quarantined undergraduate university students in Lebanon during the COVID-19 outbreak. METHODS: Undergraduate students enrolled at the American University of Beirut were invited to participate in a 47-item online questionnaire. 73 participants completed the questionnaire. Demographic data, data about COVID-19 exposure, stressors during quarantine, understanding the rationale, compliance, and difficulties associated with quarantine, and levels of psychological distress were analysed. RESULTS: 75.3% of the participants were considered as having a high risk of developing acute stress. Undergoing quarantine for more than 14 days, having a chronic medical illness, inadequate access to supplies, and fear of infection were all significantly associated with an increased risk of acute stress. CONCLUSION: Despite being a necessary preventive measure during infectious disease outbreaks, quarantine can be associated with negative psychological effects, particularly in undergraduate students. Providing preventive and effective interventions is of utmost necessity.KEY POINTSDuring COVID-19, three-quarter of students had high risk for acute stress.Females had higher odds of high-risk acute stress compared to males.Having a chronic medical condition was associated with high risk acute stress.Long quarantine and lack of supplies were associated with high risk acute stress.Providing interventions to protect the mental health of students is necessary.

Kasztelewicz, B., et al. (2021). "Prevalence of IgG antibodies against SARS-CoV-2 among healthcare workers in a tertiary pediatric hospital in Poland." PLoS One **16**(4): e0249550.

Data on the prevalence of the SARS-CoV-2 antibody in healthcare workers (HCWs) is scarce, especially in pediatric settings. The purpose of this study was to evaluate SARS-CoV-2 IgG-positivity among HCWs of a tertiary pediatric hospital. In addition, follow-up of the serological response in the subgroup of seropositive HCWs was analysed, to gain some insight on the persistence of IgG antibodies to SARS-CoV-2. We performed a retrospective analysis of voluntary SARS-CoV-2 IgG testing, which was made available free of charge to HCWs of the Children's Memorial Health Institute in Warsaw (Poland). Plasma samples were collected between July 1 and August 9, 2020, and tested using the Abbott SARS-CoV-2 IgG assay. Of 2,282 eligible participants, 1,879 (82.3%) HCWs volunteered to undergo testing. Sixteen HCWs tested positive for SARS-CoV-2 IgG, corresponding to a seroprevalence of 0.85%. Among seropositive HCWs, three HCWs had confirmed COVID-19. Nine (56.3%) of the seropositive HCWs reported neither symptoms nor unprotected contact with confirmed SARS-CoV-2 cases in the previous months. A decline in the IgG index was observed at a median time of 86.5 days (range:84‒128 days) after symptom onset or RT-PCR testing. Further studies are necessary to elucidate the duration of persistence of anti-SARS-CoV-2 antibodies, as well as the correlation between seropositivity and protective immunity against reinfection. Regardless of the persistence of antibodies and their protective properties, such low prevalence indicates that this population is vulnerable to a second wave of the COVID-19 pandemic.

Kato, S., et al. (2020). "Maximizing Health-Care Capacity in Response to COVID-19 Outbreak: Rapid Expansion Through Education by Health Emergency and Disaster Experts." Disaster Med Public Health Prep: 1-3.

Delivering adequate health care in the setting of the ongoing pandemic is challenging. Due to coronavirus disease 2019 (COVID-19), the Tokyo Metropolitan government has been forced to expand their acute health-care capacity corresponding to infectious diseases within a short period. Responding to this situation, health emergency and disaster experts of the Tokyo Disaster Medical Assistance Team took the initiative in creating a brief education course. We established the course for expanding infectious disease care capacity by a dedicated hands-on lecture for health professionals who are unfamiliar with infectious disease care in ordinary circumstances. Our lecture included the typical course of COVID-19, use of personal protective equipment, environmental sterilization, medical-ward zoning, and safe caregiving. Hospitals that received customized lectures reported by means of a questionnaire that the lectures were well suited to their needs. Currently, the health-care system in Tokyo has increased its capacity to meet the demand and has not been affected by COVID-19. Our experience shows that health emergency and disaster experts can assist hospitals in crisis by providing educational materials.

Kaur, S. P. and V. Gupta (2020). "COVID-19 Vaccine: A comprehensive status report." Virus Res **288**: 198114.

The current COVID-19 pandemic has urged the scientific community internationally to find answers in terms of therapeutics and vaccines to control SARS-CoV-2. Published investigations mostly on SARS-CoV and to some extent on MERS has taught lessons on vaccination strategies to this novel coronavirus. This is attributed to the fact that SARS-CoV-2 uses the same receptor as SARS-CoV on the host cell i.e. human Angiotensin Converting Enzyme 2 (hACE2) and is approximately 79% similar genetically to SARS-CoV. Though the efforts on COVID-19 vaccines started very early, initially in China, as soon as the outbreak of novel coronavirus erupted and then world-over as the disease was declared a pandemic by WHO. But we will not be having an effective COVID-19 vaccine before September, 2020 as per very optimistic estimates. This is because a successful COVID-19 vaccine will require a cautious validation of efficacy and adverse reactivity as the target vaccinee population include high-risk individuals over the age of 60, particularly those with chronic co-morbid conditions, frontline healthcare workers and those involved in essentials industries. Various platforms for vaccine development are available namely: virus vectored vaccines, protein subunit vaccines, genetic vaccines, and monoclonal antibodies for passive immunization which are under evaluations for SARS-CoV-2, with each having discrete benefits and hindrances. The COVID-19 pandemic which probably is the most devastating one in the last 100 years after Spanish flu mandates the speedy evaluation of the multiple approaches for competence to elicit protective immunity and safety to curtail unwanted immune-potentiation which plays an important role in the pathogenesis of this virus. This review is aimed at providing an overview of the efforts dedicated to an effective vaccine for this novel coronavirus which has crippled the world in terms of economy, human health and life.

Kavanagh, F. G., et al. (2021). "Risk of disease transmission from flexible nasoendoscopy during the coronavirus disease 2019 pandemic." J Laryngol Otol **135**(3): 246-249.

BACKGROUND: Concerns have emerged regarding infection transmission during flexible nasoendoscopy. METHODS: Information was gathered prospectively on flexible nasoendoscopy procedures performed between March and June 2020. Patients and healthcare workers were followed up to assess for coronavirus disease 2019 development. One-sided 97.5 per cent Poisson confidence intervals were calculated for upper limits of risk where zero events were observed. RESULTS: A total of 286 patients were recruited. The most common indication for flexible nasoendoscopy was investigation of 'red flag' symptoms (67 per cent). Forty-seven patients (16 per cent, 95 per cent confidence interval = 13-21 per cent) had suspicious findings on flexible nasoendoscopy requiring further investigation. Twenty patients (7.1 per cent, 95 per cent confidence interval = 4.4-11 per cent) had new cancer diagnoses. Zero coronavirus disease 2019 infections were recorded in the 273 patients. No. 27 endoscopists (the doctors and nurses who carried out the procedures) were followed up.The risk of developing coronavirus disease 2019 after flexible nasoendoscopy was determined to be 0-1.3 per cent. CONCLUSION: The risk of coronavirus disease 2019 transmission associated with performing flexible nasoendoscopy in asymptomatic patients, while using appropriate personal protective equipment, is very low. Additional data are required to confirm these findings in the setting of further disease surges.

Kaye, K., et al. (2020). "Elective, Non-urgent Procedures and Aesthetic Surgery in the Wake of SARS-COVID-19: Considerations Regarding Safety, Feasibility and Impact on Clinical Management." Aesthetic Plast Surg **44**(3): 1014-1042.

BACKGROUND: The worldwide spread of a novel coronavirus disease (COVID-19) has led to a near total stop of non-urgent, elective surgeries across all specialties in most affected countries. In the field of aesthetic surgery, the self-imposed moratorium for all aesthetic surgery procedures recommended by most international scientific societies has been adopted by many surgeons worldwide and resulted in a huge socioeconomic impact for most private practices and clinics. An important question still unanswered in most countries is when and how should elective/aesthetic procedures be scheduled again and what kind of organizational changes are necessary to protect patients and healthcare workers when clinics and practices reopen. Defining manageable, evidence-based protocols for testing, surgical/procedural risk mitigation and clinical flow management/contamination management will be paramount for the safety of non-urgent surgical procedures. METHODS: We conducted a MEDLINE/PubMed research for all available publications on COVID-19 and surgery and COVID-19 and anesthesia. Articles and referenced literature describing possible procedural impact factors leading to exacerbation of the clinical evolution of COVID-19-positive patients were identified to perform risk stratification for elective surgery. Based on these impact factors, considerations for patient selection, choice of procedural complexity, duration of procedure, type of anesthesia, etc., are discussed in this article and translated into algorithms for surgical/anesthesia risk management and clinical management. Current recommendations and published protocols on contamination control, avoidance of cross-contamination and procedural patient flow are reviewed. A COVID-19 testing guideline protocol for patients planning to undergo elective aesthetic surgery is presented and recommendations are made regarding adaptation of current patient information/informed consent forms and patient health questionnaires. CONCLUSION: The COVID-19 crisis has led to unprecedented challenges in the acute management of the crisis, and the wave only recently seems to flatten out in some countries. The adaptation of surgical and procedural steps for a risk-minimizing management of potential COVID-19-positive patients seeking to undergo elective aesthetic procedures in the wake of that wave will present the next big challenge for the aesthetic surgery community. We propose a clinical algorithm to enhance patient safety in elective surgery in the context of COVID-19 and to minimize cross-contamination between healthcare workers and patients. New evidence-based guidelines regarding surgical risk stratification, testing, and clinical flow management/contamination management are proposed. We believe that only the continuous development and broad implementation of guidelines like the ones proposed in this paper will allow an early reintegration of all aesthetic procedures into the scope of surgical care currently performed and to prepare the elective surgical specialties better for a possible second wave of the pandemic. LEVEL OF EVIDENCE V: This journal requires that authors assign a level of evidence to each article. For a full description of these Evidence-Based Medicine ratings, please refer to the Table of Contents or the online Instructions to Authors www.springer.com/00266.

Kea, B., et al. (2021). "An international survey of healthcare workers use of personal protective equipment during the early stages of the COVID-19 pandemic." J Am Coll Emerg Physicians Open **2**(2): e12392.

OBJECTIVE: Little is known regarding the specific ways personal protective equipment (PPE) has been used and reused during the coronavirus disease 2019 (COVID-19) pandemic. The objective of this study was to evaluate the patterns of PPE use and the impact of PPE availability on the attitudes and well-being of an international population of healthcare workers. METHODS: This was an online, cross-sectional survey of healthcare workers. The survey was disseminated internationally using social media, specialty society list-serves, and email augmented by snowball sampling to healthcare workers who provided direct care to patients with suspected or confirmed COVID-19. The survey was conducted between April 13 and May 1, 2020. The primary outcome was self-reported PPE use during aerosol-generating medical procedures. Other outcomes included PPE use during care for respiratory patients in general, PPE reuse, PPE decontamination, and healthcare worker impressions related to their work and the pandemic. RESULTS: A total of 2227 healthcare workers from 23 countries completed the survey. The N95 was the most common respirator among the 1451 respondents who performed aerosol-generating procedures (n = 1050, 72.3%). Overall, 1783 (80.1%) of providers reported general reuse of PPE, which was similar across US regions but less common in Canada, Italy, and Spain. The most commonly reused item of PPE was the N95 respirator, with the majority of respondents who reused PPE reporting N95 reuse (n = 1157, 64.9%). Of the 1050 individuals who wore an N95 mask while performing an aerosol-generating medical procedure, 756 (72%) reported re-using an N95, and 344 (45.5%) reported reuse for >3 days. Qualitative results identified several common themes, including (1) lack of availability of PPE, (2) fear and anxiety as a result of inadequate PPE, (3) potential exposure to family members, and (4) concerns regarding workload and pay. CONCLUSIONS: This international survey of healthcare workers found that N95 respirators were commonly used to care for patients with respiratory symptoms with and without aerosol-generating medical procedures. Healthcare workers reported an unprecedented need to reuse PPE that was designed for single-use, specifically the N95 respirator. The reuse of PPE increased the perceived risk for COVID-19 infection and harmed mental health.

Kearney, M., et al. (2021). "Future-proof Radiation therapist (RTT) practice in a pandemic - Lessons learnt from COVID-19." Tech Innov Patient Support Radiat Oncol **17**: 18-24.

BACKGROUND AND PURPOSE: The European SocieTy for Radiotherapy and Oncology Radiation Therapist Committee (ESTRO RTTC) published a guidance document and infographic providing recommendations to minimise risk of COVID-19 transmission in radiotherapy (RT) departments. The purpose of this study was to investigate the changes embedded in RT practice in the COVID-19 era and to recommend proactive measures to protect RT practice in future pandemics. MATERIALS AND METHODS: The study was initiated by the ESTRO Radiation Oncology Safety and Quality Committee (ROSQC). A survey consisting of multiple choice, open ended and Likert scale questions was created to analyse the extent of changes embedded in RT practice in response to the COVID-19 pandemic under the four domains: patient care, RTT workflow, remote working and RT practice. This online survey was distributed globally in May 2020. RESULTS: 229 respondents across 27 countries completed the survey. 60% of respondents reported continuing/commencing RT in COVID-19 patients. Routine testing of patients and RTTs was not common. Split teams' procedures, hot linacs and separate entrances were implemented by 50% of respondents. Remote working was implemented for RT team members where face to face patient contact was not essential. Lack of staff, connectivity issues and lack of confirmed positive cases in the department were the main reasons cited for not implementing recommended measures. CONCLUSION: It is suggested that RT departments have responded to the COVID-19 pandemic and implemented certain changes in RT practice. RT departments should act now to implement recommended proactive measures to protect patients and RTTs - frontline healthcare workers.

Keske, Ş., et al. (2020). "Influenza vaccination among infection control teams: A EUCIC survey prior to COVID-19 pandemic." Vaccine **38**(52): 8357-8361.

We aimed to describe the influenza vaccination rate and its determinants among infection control team (ICT) across different countries. Online multilingual survey consisting of 23 items, between 17 May -15 July of 2019 targeting the opinions and practices of ICTs regarding the 2018-2019 influenza season was employed. Participants were reached via European Society of Clinical Microbiology and Infectious Diseases (ESCMID) and European Union Certificate for Infection Control (EUCIC) newsletters,social media, and national societies. In total, 899 participants from 56 countries responded to the survey. The overall vaccination rate was 76%, being the highest in Finland, Portugal, Norway, and Israel (100%), whereas the lowest in Italy (68%) and Turkey (39%). Influenza vaccination rate was 86% among IC physicians and 52% among IC nurses. The most significant factors affecting participants' decision were personal influenza vaccine experience (49%) and attitude of the scientific authorities (48%). In multivariate analysis, vaccination of the ICT head (OR: 16.04, 95%CI: 8.4-30.8, p < 0.001) and having free vaccine (OR: 7.56, 95%CI: 2.1-27.4, p = 0.02) were found to be the strongest predictors for influenza vaccination, whereas working in Turkey (OR: 0.41, 95%CI: 0.22-0.77, p = 0.006) and being an IC nurse (OR:0.43, 95%CI: 0.24-0.80, p = 0.007) were significantly associated with not having been vaccinated. In conclusion, COVID-19 pandemic increased the importance of protection against respiratory viruses including influenza. Vaccination strategies should have a special emphasis on IC nurses, who have a relatively lower vaccination rate, should enhance the vaccination of the ICT leaders, and put effort to provide free availability of the influenza vaccine.

Ketas, T. J., et al. (2021). "Antibody responses to SARS-CoV-2 mRNA vaccines are detectable in saliva." bioRxiv.

Vaccines are critical for curtailing the COVID-19 pandemic (1, 2). In the USA, two highly protective mRNA vaccines are available: BNT162b2 from Pfizer/BioNTech and mRNA-1273 from Moderna (3, 4). These vaccines induce antibodies to the SARS-CoV-2 S-protein, including neutralizing antibodies (NAbs) predominantly directed against the Receptor Binding Domain (RBD) (1-4). Serum NAbs are induced at modest levels within ∼1 week of the first dose, but their titers are strongly boosted by a second dose at 3 (BNT162b2) or 4 weeks (mRNA-1273) (3, 4). SARS-CoV-2 is most commonly transmitted nasally or orally and infects cells in the mucosae of the respiratory and to some extent also the gastrointestinal tract (5). Although serum NAbs may be a correlate of protection against COVID-19, mucosal antibodies might directly prevent or limit virus acquisition by the nasal, oral and conjunctival routes (5). Whether the mRNA vaccines induce mucosal immunity has not been studied. Here, we report that antibodies to the S-protein and its RBD are present in saliva samples from mRNA-vaccinated healthcare workers (HCW). Within 1-2 weeks after their second dose, 37/37 and 8/8 recipients of the Pfizer and Moderna vaccines, respectively, had S-protein IgG antibodies in their saliva, while IgA was detected in a substantial proportion. These observations may be relevant to vaccine-mediated protection from SARS-CoV-2 infection and disease.

Key, T., et al. (2020). "Personal protective equipment during the COVID-19 crisis: a snapshot and recommendations from the frontline of a university teaching hospital." Bone Jt Open **1**(5): 131-136.

AIMS: The adequate provision of personal protective equipment (PPE) for healthcare workers has come under considerable scrutiny during the COVID-19 pandemic. This study aimed to evaluate staff awareness of PPE guidance, perceptions of PPE measures, and concerns regarding PPE use while caring for COVID-19 patients. In addition, responses of doctors, nurses, and other healthcare professionals (OHCPs) were compared. METHODS: The inclusion criteria were all staff working in clinical areas of the hospital. Staff were invited to take part using a link to an online questionnaire advertised by email, posters displayed in clinical areas, and social media. Questions grouped into the three key themes - staff awareness, perceptions, and concerns - were answered using a five-point Likert scale. The Kruskal-Wallis test was used to compare results across all three groups of staff. RESULTS: Overall, 315 staff took part in our study. There was a high awareness of PPE guidance at 84.4%, but only 52.4% of staff reported adequate PPE provision. 67.9% were still keen to come to work, despite very high levels of anxiety relating to contracting COVID-19 despite wearing PPE. Doctors had significantly higher ratings for questions relating to PPE awareness compared to other staff groups, while nursing staff and OHCPs had significantly higher levels of anxiety compared to doctors in relation to PPE and contracting COVID-19 (p < 0.05 using a Kruskal-Wallis test). CONCLUSION: We believe four recommendations are key to improve PPE measures and decrease anxiety: 1) nominated ward/department PPE champions; 2) anonymized reporting for PPE concerns; 3) formal PPE education sessions; and 4) drop-in counselling sessions for staff. We hope the insight and recommendations from this study can improve the PPE situation and maintain the health and wellbeing of the clinical work force, in order to care for COVID-19 patients safely and effectively.

Khajuria, A., et al. (2021). "Workplace factors associated with mental health of healthcare workers during the COVID-19 pandemic: an international cross-sectional study." BMC Health Serv Res **21**(1): 262.

BACKGROUND: The association of workplace factors on mental health of healthcare workers (HCWs) during the COVID-19 pandemic needs to be urgently established. This will enable governments and policy-makers to make evidence-based decisions. This international study reports the association between workplace factors and the mental health of HCWs during the pandemic. METHODS: An international, cross-sectional study was conducted in 41 countries. The primary outcome was depressive symptoms, derived from the validated Patient Health Questionnaire-2 (PHQ-2). Multivariable logistic regression identified factors associated with mental health outcomes. Inter-country differences were also evaluated. RESULTS: A total of 2527 responses were received, from 41 countries, including China (n = 1213; 48.0%), UK (n = 891; 35.3%), and USA (n = 252; 10.0%). Of all participants, 1343 (57.1%) were aged 26 to 40 years, and 2021 (80.0%) were female; 874 (34.6%) were doctors, and 1367 (54.1%) were nurses. Factors associated with an increased likelihood of depressive symptoms were: working in the UK (OR = 3.63; CI = [2.90-4.54]; p < 0.001) and USA (OR = 4.10; CI = [3.03-5.54]), p < 0.001); being female (OR = 1.74; CI = [1.42-2.13]; p < 0.001); being a nurse (OR = 1.64; CI = [1.34-2.01]; p < 0.001); and caring for a COVID-19 positive patient who subsequently died (OR = 1.20; CI = [1.01-1.43]; p = 0.040). Workplace factors associated with depressive symptoms were: redeployment to Intensive Care Unit (ICU) (OR = 1.67; CI = [1.14-2.46]; p = 0.009); redeployment with perceived unsatisfactory training (OR = 1.67; CI = [1.32-2.11]; p < 0.001); not being issued with appropriate personal protective equipment (PPE) (OR = 2.49; CI = [2.03-3.04]; p < 0.001); perceived poor workplace support within area/specialty (OR = 2.49; CI = [2.03-3.04]; p < 0.001); and perceived poor mental health support (OR = 1.63; CI = [1.38-1.92]; p < 0.001). CONCLUSION: This is the first international study, demonstrating that workplace factors, including PPE availability, staff training pre-redeployment, and provision of mental health support, are significantly associated with mental health during COVID-19. Governments, policy-makers and other stakeholders need to ensure provision of these to safeguard HCWs' mental health, for future waves and other pandemics.

Khalefa, M. A., et al. (2021). "Evidence-based review of safe theatre practice during the COVID-19 pandemic beyond personal protective equipment." Ann R Coll Surg Engl **103**(2): 88-95.

INTRODUCTION: COVID-19 remains a threat for a fear of a second pandemic. Emergency orthopaedic operations are still among the most commonly performed procedures with increased risk of transmission of SARS CoV-2 to the patients and the healthcare workers. The aim of this study was to present the evidence available into best practices limiting the spread of COVID-19 in healthcare setting during current and future pandemics. METHODS: A review of the literature was performed in multiple databases (PubMed, the Cochrane Library, Google Scholar, World Health Organization and Centers for Disease Control), using 'COVID-19' with other relevant keywords in different combinations. Owing to the limited and heterogenous evidence available, data were presented in a narrative manner. FINDINGS: From the evidence gathered it was noted that a multimodal approach to minimising pathogen transmission is required. This primarily comprises the wider engineering and administrative controls to reduce the concentration of the pathogen and to separate staff and patients from it. Theatre isolation and traffic control bundling, theatre flow and logistics, ventilation and waste management form a pivotal role in the environmental/engineering controls. Administrative measures include policies for both patients and staff. For patients, isolation and preoperative screening are of utmost value. For staff, testing for COVID-19, risk assessment, redeployment and provision of persona; protective equipment, together with the necessary training are important administrative controls. CONCLUSION: We believe these measures are likely to improve the sustainability of resources and can be carried to elective settings in order to return to some form of normality and help to mitigate the effects of future pandemics.

Khalil, M. M., et al. (2020). "Role of Personal Protective Measures in Prevention of COVID-19 Spread Among Physicians in Bangladesh: a Multicenter Cross-Sectional Comparative Study." SN Compr Clin Med: 1-7.

This study aims to determine the role of personal protective measures in the prevention of COVID-19 spread among the physicians working at different health facilities in Bangladesh. This hospital-based cross-sectional comparative study was conducted from May to June 2020. A total of 98 COVID-19 positive physicians and 92 COVID-19 negative physicians (physicians with no symptoms of COVID-19 or who tested negative) were enrolled. The questionnaire was adapted from a tool developed by the World Health Organization (WHO) for risk assessment and management of exposure of healthcare workers in the context of COVID-19. Data were collected from the respondents online using Google forms. There was no significant difference in baseline information between COVID-19 positive and COVID-19 negative physicians. The physicians, who were unaware of direct participation in COVID-19 patient care, had higher odds of being COVID-19 positive (OR = 4.018; CI: 1.532-10.535). Additionally, the physicians, who were unaware of the COVID-19 status while performing the aerosol-generated procedure (AGP), had a higher chance of being COVID-19 positive (OR = 2.522; CI: 1.020-6.233). Using face shields/goggles (OR = 0.437; CI:0.228-0.837) and regular decontamination of the patient's surroundings (OR = 0.392; CI:0.176-0.873) while usual take care of patients and use of N95 masks while performing AGP (OR = 0.372; CI:0.159-0.873) had protective roles against COVID-19 among the physicians. The physicians who had reused the medical gown had two times more chances of being tested positive for COVID-19 than those who had not reused it (OR = 2.3; CI:1.251-4.259). The use of face shields/goggles and N95 masks and decontamination of the patient's surroundings may give protection against COVID-19. Additionally, reusing medical gowns should be avoided as much as possible.

Khera, R., et al. (2020). "An Evaluation of the Vulnerable Physician Workforce in the United States During the Coronavirus Disease-19 Pandemic." medRxiv.

BACKGROUND: The coronavirus disease-19 (COVID-19) pandemic threatens to overwhelm the healthcare resources of the country, but also poses a personal hazard to healthcare workers, including physicians. To address the potential impact of excluding physicians with a high risk of adverse outcomes based on age, we evaluated the current patterns of age of licensed physicians across the United States. METHODS: We compiled information from the 2018 database of actively licensed physicians in the Federation of State Medical Boards (FSMB) across the US. Both at a national- and the state-level, we assessed the number and proportion of physicians who would be at an elevated risk due to age over 60 years. RESULTS: Of the 985,026 licensed physicians in the US, 235857 or 23.9% were aged 25-40 years, 447052 or 45.4% are 40-60 years, 191794 or 19.5% were 60-70 years, and 106121 or 10.8% were 70 years or older. Age was not reported in 4202 or 0.4% of physicians. Overall, 297915 or 30.2% of physicians were 60 years of age or older, 246167 (25.0%) 65 years and older, and 106121 (10.8%) 70 years or older. States in the US reported that a median 5470 licensed physicians (interquartile range [IQR], 2394 to 10108) were 60 years of age or older. Notably, states of North Dakota (n=1180) and Vermont (n = 1215) had the lowest and California (n=50786) and New York (n=31582) the highest number of physicians over the age of 60 years (Figure 1). Across states, the median proportion of physicians aged 60 years and older was 28.9% (IQR, 27.2%, 31.4%), and ranged between 25.9% for Nebraska to 32.6% for New Mexico (Figure 2). DISCUSSION: Older physicians represent a large proportion of the US physician workforce, particularly in states with the worst COVID-19 outbreak. Therefore, their exclusion from patient care will be impractical. Optimizing care practices by limiting direct patient contact of physicians vulnerable to adverse outcomes from COVID-19, potentially by expanding their participation in telehealth may be a strategy to protect them.

Khorasanee, R., et al. (2021). "The effects of COVID-19 on sickness of medical staff across departments: A single centre experience." Clin Med (Lond) **21**(2): e150-e154.

INTRODUCTION: COVID-19 presents a risk to healthcare workers, incurring harm to staff physical and mental wellbeing and difficulties in provision of care and service planning. METHODOLOGY: Doctors' anonymised demographic and staff sickness data were collected between 16 March and 26 April 2020, corresponding with the single centre's greatest COVID-19 caseload. FINDINGS: 128 (39%) of doctors experienced at least one sickness episode. Episodes totalled 1,240 days, equating to a sickness absence rate of 9.1%. Rates varied between departments and grades. High levels of sickness were seen in medicine and both adult and paediatric emergency departments with the lowest levels seen in intensive care. DISCUSSION: COVID-19 caused a burden of sickness on the medical workforce which must be accounted for in future workforce planning. The disparity in sickness rates across departments is likely to be multi-factorial. Further study is needed to investigate these factors to protect healthcare staff and their patients.

Kicska, G., et al. (2020). "Statement from the North American Society for Cardiovascular Imaging on imaging strategies to reduce the scarcity of healthcare resources during the COVID-19 outbreak." Int J Cardiovasc Imaging **36**(8): 1387-1393.

Coronavirus disease 2019 (COVID-19), caused by severe acute respiratory syndrome coronavirus 2 (SARS-CoV-2), is an evolving global pandemic that is predicted to strain healthcare resources at multiple locations throughout North America and the World. As of April 6, 2020, the apex of infection rates is predicted to occur within 1 to 5 weeks at various locations. Widespread reports of personal protective equipment (PPE) shortages, and healthcare worker exposure to disease have become commonplace. To mitigate this crisis, we are suggesting imaging strategies that aim to use the least PPE, require the smallest number of potential staff exposures, and streamlines utilization of imaging. They are broadly organized by (1) substituting a noninvasive diagnostic test in place of a semi-invasive or invasive diagnostic tests, and (2) consolidating diagnostic imaging.

Kiely, L. F., et al. (2021). "Irritant contact dermatitis in healthcare workers as a result of the COVID-19 pandemic: a cross-sectional study." Clin Exp Dermatol **46**(1): 142-144.

COVID-19 healthcare workers (HCWs) require frequent handwashing and use of personal protective equipment (PPE) to prevent infection. However, evidence is emerging that these practices are causing adverse effects on their skin integrity. A single-centre, cross-sectional study of HCWs from an Irish hospital was undertaken to evaluate the degree of COVID-19-related irritant contact dermatitis (ICD) between April and May 2020. Of 270 participants surveyed, 223 (82.6%) reported symptoms of ICD. The hands were the most commonly affected site (76.47%) and the most frequently reported symptom was dry skin (75.37%). Nearly all (268; 99.26%) HCWs had increased hand-washing frequency, but 122 (45.35%) did not use emollients. In the ICD group, 24.7% cited a history of dermatitis compared with 4.3% of unaffected staff (P < 0.001). The ICD group recorded PPE usage for an average of 3.15 h compared with the non-ICD group at 1.97 h (P = 0.21). Promoting awareness of COVID-19-related ICD is vital to highlight prevention and treatment for frontline staff.

Kim, H., et al. (2021). "Access to personal protective equipment in exposed healthcare workers and COVID-19 illness, severity, symptoms and duration: a population-based case-control study in six countries." BMJ Glob Health **6**(1).

BACKGROUND: Despite the widespread implementation of personal protective equipment (PPE) in the COVID-19 pandemic, there are surprisingly few studies of its impact. To assess the risk, severity and duration of COVID-19 in relation to access to PPE in at-risk healthcare workers (HCWs). METHODS: From 17 July to 25 September 2020, at-risk physicians and nurses registered as a provider in the Survey Healthcare Globus network in six countries (the UK, Germany, France, Italy, Spain and USA) were identified based on adult medical specialties with frequent and close contact with patients with COVID-19. Exposed HCWs completed a detailed questionnaire including demographics, medical, social and lifestyle factors. COVID-19 cases were defined as COVID-19 symptoms (fever, cough, fatigue, loss of taste or smell) and asymptomatic COVID-19 test positive cases. RESULTS: Among 2884 exposed HCWs (94% medical doctors and 6% nurses or physician assistants), there were 514 reports of COVID-19 illness and 54 asymptomatic COVID-19 test positive cases. COVID-19 risk was significantly associated with close contact with COVID-19 cases both inside and outside the workplace, number of work shifts and hours worked per week. Limited access to PPE compared with access to a fresh mask, gown and gloves and face shield with each patient encounter was associated with a 2.2-fold to 22-fold increased risk of reporting COVID-19 symptoms (p<0.0001), a pattern consistent across all six countries. Further, limited access to PPE was associated with symptom duration greater than 2 weeks and the presence of moderate to severe symptoms such as difficulty breathing, abnormal chest X-ray, low oxygen saturations, respiratory distress and acute lung injury. CONCLUSION: In six countries, less access to PPE was strongly associated with both increased risk of reporting COVID-19 illness as well as more prolonged and severe disease course in frontline HCWs.

Kim, H., et al. (2020). "The experience of infection prevention for coronavirus disease 2019 (COVID-19) during general anesthesia in an epidemic of COVID-19: including unexpected exposure case - Two cases report." Anesth Pain Med (Seoul) **15**(3): 388-396.

BACKGROUND: Coronavirus disease 2019 (COVID-19) has been an epidemic in the Republic of Korea since the first patient who came in China was confirmed as having the disease on January 19, 2020. Therefore, surgery for COVID-19-related patients is expected to increase. CASE: We experienced two cases of COVID-19-related patients who underwent surgery for femur fracture under general anesthesia. In one case, infection control to prevent transmission was achieved, while in the second case, healthcare workers were exposed to severe acute respiratory syndrome coronavirus-2 (SARS-CoV-2). CONCLUSIONS: In areas where there is COVID-19 epidemic, SARS-CoV-2 real-time polymerase chain reaction should be performed before surgery in patients with symptoms of respiratory infection such as fever, cough, and sputum, or for those in whom the possibility of COVID-19 cannot be completely excluded. Efforts should be made to prevent healthcare worker's exposure and the contamination of the operating room.

Kim, H. J., et al. (2021). "Importance of Preoperative Screening Strategies for Coronavirus Disease 2019 in Patients Undergoing Cesarean Sections: A Retrospective, Large Single-Center, Observational Cohort Study." J Clin Med **10**(4).

During the coronavirus disease 2019 (COVID-19) pandemic, many guidelines have recommended postponing non-emergency operations. However, cesarean sections cannot be indefinitely delayed. Our institution has established a COVID-19 screening strategy for patients undergoing cesarean section. We evaluated the usefulness of this screening strategy. Parturients undergoing cesarean section at our center during the first peak of the COVID-19 outbreak were retrospectively analyzed. Each parturient underwent a questionnaire survey evaluating epidemiological correlation and symptoms at admission. Reverse transcriptase-polymerase chain reaction (RT-PCR) testing and/or chest radiography were performed. In total, 296 parturients underwent cesarean section. All elective and 37 emergency cesarean sections were performed in general operating rooms because they were considered to have a low risk of COVID-19 infection through the screening process. However, 42 emergency cases were performed in negative-pressure operating rooms with full personal protective equipment (PPE) because the RT-PCR results could not be confirmed in a timely manner. None of them were positive for RT-PCR, and there were no cases of nosocomial infection. A comprehensive preoperative screening strategy, including symptomatic and epidemiological correlation, PCR, and/or imaging test, should be performed in patients undergoing cesarian section. Further, cesarean sections in parturients with unconfirmed COVID-19 status should be performed in a negative-pressure operating room with appropriate PPE.

Kim, Y. J., et al. (2021). "How to keep patients and staff safe from accidental SARS-CoV-2 exposure in the emergency room: Lessons from South Korea's explosive COVID-19 outbreak." Infect Control Hosp Epidemiol **42**(1): 18-24.

OBJECTIVES: We report our experience with an emergency room (ER) shutdown related to an accidental exposure to a patient with coronavirus disease 2019 (COVID-19) who had not been isolated. SETTING: A 635-bed, tertiary-care hospital in Daegu, South Korea. METHODS: To prevent nosocomial transmission of the disease, we subsequently isolated patients with suspected symptoms, relevant radiographic findings, or epidemiology. Severe acute respiratory coronavirus 2 (SARS-CoV-2) reverse-transcriptase polymerase chain reaction assays (RT-PCR) were performed for most patients requiring hospitalization. A universal mask policy and comprehensive use of personal protective equipment (PPE) were implemented. We analyzed effects of these interventions. RESULTS: From the pre-shutdown period (February 10-25, 2020) to the post-shutdown period (February 28 to March 16, 2020), the mean hourly turnaround time decreased from 23:31 ±6:43 hours to 9:27 ±3:41 hours (P < .001). As a result, the proportion of the patients tested increased from 5.8% (N=1,037) to 64.6% (N=690) (P < .001) and the average number of tests per day increased from 3.8±4.3 to 24.7±5.0 (P < .001). All 23 patients with COVID-19 in the post-shutdown period were isolated in the ER without any problematic accidental exposure or nosocomial transmission. After the shutdown, several metrics increased. The median duration of stay in the ER among hospitalized patients increased from 4:30 hours (interquartile range [IQR], 2:17-9:48) to 14:33 hours (IQR, 6:55-24:50) (P < .001). Rates of intensive care unit admissions increased from 1.4% to 2.9% (P = .023), and mortality increased from 0.9% to 3.0% (P = .001). CONCLUSIONS: Problematic accidental exposure and nosocomial transmission of COVID-19 can be successfully prevented through active isolation and surveillance policies and comprehensive PPE use despite longer ER stays and the presence of more severely ill patients during a severe COVID-19 outbreak.

Kimura, Y., et al. (2020). "Society of swallowing and dysphagia of Japan: Position statement on dysphagia management during the COVID-19 outbreak." Auris Nasus Larynx **47**(5): 715-726.

On April 14, the Society of Swallowing and Dysphagia of Japan (SSDJ) proposed its position statement on dysphagia treatment considering the ongoing spread of severe acute respiratory syndrome coronavirus 2 (SARS-CoV-2). The main routes of transmission of SARS-CoV-2 are physical contact with infected persons and exposure to respiratory droplets. In cases of infection, the nasal cavity and nasopharynx have the highest viral load in the body. Swallowing occurs in the oral cavity and pharynx, which correspond to the sites of viral proliferation. In addition, the possibility of infection by aerosol transmission is also concerning. Dysphagia treatment includes a broad range of clinical assessments and examinations, dysphagia rehabilitation, oral care, nursing care, and surgical treatments. Any of these can lead to the production of droplets and aerosols, as well as contact with viral particles. In terms of proper infection control measures, all healthcare professionals involved in dysphagia treatment must be fully briefed and must appropriately implement all measures. In addition, most patients with dysphagia should be considered to be at a higher risk for severe illness from COVID-19 because they are elderly and have complications including heart diseases, diabetes, respiratory diseases, and cerebrovascular diseases. This statement establishes three regional categories according to the status of SARS-CoV-2 infection. Accordingly, the SSDJ proposes specific infection countermeasures that should be implemented considering 1) the current status of SARS-CoV-2 infection in the region, 2) the patient status of SARS-CoV-2 infection, and 3) whether the examinations or procedures conducted correspond to aerosol-generating procedures, depending on the status of dysphagia treatment. This statement is arranged into separate sections providing information and advice in consideration of the COVID-19 outbreak, including "terminology", "clinical swallowing assessment and examination", "swallowing therapy", "oral care", "surgical procedure for dysphagia", "tracheotomy care", and "nursing care". In areas where SARS-CoV-2 infection is widespread, sufficient personal protective equipment should be used when performing aerosol generation procedures. The current set of statements on dysphagia management in the COVID-19 outbreak is not an evidence-based clinical practice guideline, but a guide for all healthcare workers involved in the treatment of dysphagia during the COVID-19 epidemic to prevent SARS-CoV-2 infection.

Kirk, A. H. P., et al. (2021). "Psychosocial impact of the COVID-19 pandemic on paediatric healthcare workers." Ann Acad Med Singap **50**(3): 203-211.

INTRODUCTION: Frontline healthcare workers (HCWs) exposed to coronavirus disease 2019 (COVID-19) are at risk of psychological distress. This study evaluates the psychological impact of COVID-19 pandemic on HCWs in a national paediatric referral centre. METHODS: This was a survey-based study that collected demographic, work environment and mental health data from paediatric HCWs in the emergency, intensive care and infectious disease units. Psychological impact was measured using the Depression, Anxiety, Stress Scale-21. Multivariate regression analysis was performed to identify risk factors associated with psychological distress. RESULTS: The survey achieved a response rate of 93.9% (430 of 458). Of the 430 respondents, symptoms of depression, anxiety and stress were reported in 168 (39.1%), 205 (47.7%) and 106 (24.7%), respectively. Depression was reported in the mild (47, 10.9%), moderate (76, 17.7%), severe (23, 5.3%) and extremely severe (22, 5.1%) categories. Anxiety (205, 47.7%) and stress (106, 24.7%) were reported in the mild category only. Collectively, regression analysis identified female sex, a perceived lack of choice in work scope/environment, lack of protection from COVID-19, lack of access to physical activities and rest, the need to perform additional tasks, and the experience of stigma from the community as risk factors for poor psychological outcome. CONCLUSION: A high prevalence of depression, anxiety and stress was reported among frontline paediatric HCWs during the COVID-19 pandemic. Personal psychoneuroimmunity and organisational prevention measures can be implemented to lessen psychiatric symptoms. At the national level, involving mental health professionals to plan and coordinate psychological intervention for the country should be considered.

Kisely, S., et al. (2020). "Occurrence, prevention, and management of the psychological effects of emerging virus outbreaks on healthcare workers: rapid review and meta-analysis." Bmj **369**: m1642.

OBJECTIVE: To examine the psychological effects on clinicians of working to manage novel viral outbreaks, and successful measures to manage stress and psychological distress. DESIGN: Rapid review and meta-analysis. DATA SOURCES: Cochrane Central Register of Controlled Trials, PubMed/Medline, PsycInfo, Scopus, Web of Science, Embase, and Google Scholar, searched up to late March 2020. ELIGIBILITY CRITERIA FOR STUDY SELECTION: Any study that described the psychological reactions of healthcare staff working with patients in an outbreak of any emerging virus in any clinical setting, irrespective of any comparison with other clinicians or the general population. RESULTS: 59 papers met the inclusion criteria: 37 were of severe acute respiratory syndrome (SARS), eight of coronavirus disease 2019 (covid-19), seven of Middle East respiratory syndrome (MERS), three each of Ebola virus disease and influenza A virus subtype H1N1, and one of influenza A virus subtype H7N9. Of the 38 studies that compared psychological outcomes of healthcare workers in direct contact with affected patients, 25 contained data that could be combined in a pairwise meta-analysis comparing healthcare workers at high and low risk of exposure. Compared with lower risk controls, staff in contact with affected patients had greater levels of both acute or post-traumatic stress (odds ratio 1.71, 95% confidence interval 1.28 to 2.29) and psychological distress (1.74, 1.50 to 2.03), with similar results for continuous outcomes. These findings were the same as in the other studies not included in the meta-analysis. Risk factors for psychological distress included being younger, being more junior, being the parents of dependent children, or having an infected family member. Longer quarantine, lack of practical support, and stigma also contributed. Clear communication, access to adequate personal protection, adequate rest, and both practical and psychological support were associated with reduced morbidity. CONCLUSIONS: Effective interventions are available to help mitigate the psychological distress experienced by staff caring for patients in an emerging disease outbreak. These interventions were similar despite the wide range of settings and types of outbreaks covered in this review, and thus could be applicable to the current covid-19 outbreak.

Kishi, T., et al. (2020). "Recommendations for Maintaining the Cardiovascular Care System Under the Conditions of the COVID-19 Pandemic　- 1st Edition, April 2020." Circ J **84**(11): 2023-2026.

BACKGROUND: The Japanese Circulation Society proposes recommendations for all healthcare professionals involved in cardiovascular medicine to protect them from infection and ensure that seriously ill patients requiring urgent care receive proper treatment.Methods and Results:Patients are divided into "Positive or suspected coronavirus disease 2019 (COVID-19)" and "All others". Furthermore, tests and treatments are divided into emergency or standby. For each category, we propose recommendations. CONCLUSIONS: To maintain the cardiovascular care system, The Japanese Circulation Society recommends completely preventing nosocomial COVID-19 infections, ensuring adequate PPE necessary for healthcare personnel, and learning and implementing standard precautions.

Kline, J. D. and A. E. Donovan (2020). "Sentinel Case of COVID-19 at Fort Stewart, GA in a National Guard Soldier Participating in Annual Training: A Case Report." Mil Med **185**(11-12): e2158-e2161.

For healthcare providers, specifically military and federal public health personnel, prompt and accurate diagnosis and isolation of SARS-CoV-2 novel coronavirus patients provide a two-fold benefit: (1) directing appropriate treatment to the infected patient as early as possible in the progression of the disease to increase survival rates and minimize the devastating sequelae following recovery and remission of symptoms; (2) provide critical information requirements that enable commanders and public health officials to best synchronize policy, regulations, and troop movement restrictions while best allocating scarce resources in the delicate balance of risk mitigation versus mission readiness. Simple personal protective measures and robust testing and quarantine procedures, instituted and enforced aggressively by senior leaders, physicians, and healthcare professionals at all levels are an essential aspect of the battle against the COVID-19 pandemic that will determine the success or failure of the overall effort. As consideration, the authors respectfully submit this vignette of the first confirmed positive COVID-19 case presenting to the Emergency Department at Winn Army Community Hospital, Fort Stewart, Georgia.

Knights, H., et al. (2020). "Characteristics and outcomes of patients with COVID-19 at a district general hospital in Surrey, UK." Clin Med (Lond) **20**(5): e148-e153.

BACKGROUND: This retrospective cohort study aims to define the clinical findings and outcomes of every patient admitted to a district general hospital in Surrey with COVID-19 in March 2020, providing a snapshot of the first wave of infection in the UK. This study is the first detailed insight into the impact of frailty markers on patient outcomes and provides the infection rate among healthcare workers. METHODS: Data were obtained from medical records. Outcome measures were level of oxygen therapy, discharge and death. Patients were followed up until 21 April 2020. RESULTS: 108 patients were included. 34 (31%) died in hospital or were discharged for palliative care. 43% of patients aged over 65 died. The commonest comorbidities were hypertension (49; 45%) and diabetes (25; 23%). Patients who died were older (mean difference ±SEM, 13.76±3.12 years; p<0.0001) with a higher NEWS2 score (median 6, IQR 2.5-7.5 vs median 2, IQR 2-6) and worse renal function (median differences: urea 2.7 mmol/L, p<0.01; creatinine 4 μmol/L, p<0.05; eGFR 14 mL/min, p<0.05) on admission compared with survivors. Frailty markers were identified as risk factors for death. Clinical Frailty Scale (CFS) was higher in patients over 65 who died than in survivors (median 5, IQR 4-6 vs 3.5, IQR 2-5; p<0.01). Troponin and creatine kinase levels were higher in patients who died than in those who recovered (p<0.0001). Lymphopenia was common (median 0.8, IQR 0.6-1.2; p<0.005). Every patient with heart failure died (8). 26 (24%) were treated with continuous positive airway pressure (CPAP; median 3 days, IQR 2-7.3) and 9 (8%) were intubated (median 14 days, IQR 7-21). All patients who died after discharge (4; 6%) were care home residents. 276 of 699 hospital staff tested were positive for COVID-19. CONCLUSIONS: This study identifies older patients with frailty as being particularly vulnerable and reinforces government policy to protect this group at all costs.

Knoll, R. L., et al. (2020). "Containment of a Large SARS-CoV-2 Outbreak Among Healthcare Workers in a Pediatric Intensive Care Unit." Pediatr Infect Dis J **39**(11): e336-e339.

OBJECTIVE: Healthcare workers (HCWs) are particularly exposed SARS-CoV-2 because they are critical in preventing viral transmission and treating COVID-19 patients. Within HCWs, personnel of intensive care units (ICUs) are at the forefront of treating patients with a severe course of COVID-19 infection and therefore represent an extremely vulnerable group. Thus, our objective is to contribute to establish means of infection control protecting HCWs in the frontline of the current pandemic. DESIGN: An outbreak of SARS-CoV-2 was detected and contained in a pediatric ICU (PICU). The first positive case was identified with a point-of-care diagnostic system on site. Real-time PCR-based testing systems from self-collected nasopharyngeal samples swabs were used to test for viral RNA of SARS-CoV-2 in the follow-up. SETTING: PICU within a tertiary university hospital in Germany. PARTICIPANTS: Healthcare workers of the PICU. INTERVENTIONS: Positive HCWs were sent into quarantine. Containment measures were implemented including wearing of surgical-masks, physical distancing and systematic testing. RESULTS: Among 432 HCWs, 91 (25%) were tested. Forty-five percent reported symptoms corresponding to characteristics of COVID-19. Of those, only 19,5% (8 HCWs) were tested positive for SARS-CoV-2. No infection occurred outside the PICU. After the implementation of containment measures, viral transmission was stopped. CONCLUSIONS: In the present study, a large outbreak within a team of healthcare workers of a PICU, affecting almost one fifth of the entire personnel is documented, along with detailed insights about how the outbreak was contained and how operability of the unit was maintained.

Ko, J. H., et al. (2020). "Serologic Evaluation of Healthcare Workers Caring for COVID-19 Patients in the Republic of Korea." Front Microbiol **11**: 587613.

The safety of healthcare workers (HCWs) against severe acute respiratory syndrome virus 2 (SARS-CoV-2) transmission is an important aspect of managing the coronavirus disease 2019 (COVID-19) pandemic. In the South Korea, highly stringent infection prevention and control (IPC) guidelines are implemented, and reports of healthcare-associated SARS-CoV-2 transmission among HCWs are limited. However, subclinical infections may have been missed by the current symptom-based screening strategy. To evaluate the risk of undetected SARS-CoV-2 transmissions from COVID-19 patients to HCWs, we conducted a multicenter seroprevalence study after the first surge of the COVID-19 outbreak. A total of 432 HCWs were evaluated, comprising 309 HCWs designated to laboratory-confirmed COVID-19 patient care and 123 non-designated HCWs. Designated HCWs wore personal protective equipment including an N95 respirator, eye protection, hooded overalls, shoe covers, and inner and outer gloves. Use of a powered air-purifying respirator was recommended for aerosol-generating procedures or long-duration care activities. A high-sensitivity (99.1%) fluorescence immunoassay immunoglobulin G (IgG) kit was used as the initial screening test, and two enzyme-linked immunosorbent assay kits for total and IgG antibodies were used to confirm the test results. A microneutralization test was additionally performed to evaluate the neutralizing activity of positive specimens. Among the evaluated HCWs, none of the non-designated HCWs had a positive result, while one of the HCWs designated for COVID-19 patient care (1/309, 0.3%) was seropositive for SARS-CoV-2 with confirmed neutralizing activity (1:40). This finding suggests that subclinical seroconversion may occur among HCWs caring for COVID-19 patients, although the risk is low under strict IPC guidance.

Kobayashi, M., et al. (2020). "Should COVID-19 patients be taken to an airborne infection isolation room without cardiopulmonary resuscitation?" Am J Emerg Med.

Koehler, P., et al. (2021). "Bronchoscopy safety precautions for diagnosing COVID-19 associated pulmonary aspergillosis-A simulation study." Mycoses **64**(1): 55-59.

OBJECTIVES: With the outbreak of coronavirus disease 2019 (COVID-19), clinicians have used personal protective equipment to avoid transmission of severe acute respiratory syndrome coronavirus 2. However, they still face occupational risk of infection, when treating COVID-19 patients. This may be highest during invasive diagnostic procedures releasing aerosols and droplets. Thereby, the use of diagnostic procedures for Covid-19 associated aspergillosis may be delayed or impeded, as use of bronchoscopy has been discouraged. This leads to avoidance of a crucial procedure for diagnosing invasive aspergillosis. We intent to visualise aerosol and droplet spread and surface contamination during bronchoscopy and address which measures can avoid exposure of health-care workers. METHODS: We created a simulation model to visualise aerosol and droplet generation as well as surface contamination by nebulising fluorescent solution detected by using ultraviolet light- and slow-motion capture. We repurposed covers for ultrasound transducers or endoscopic cameras to prevent surface and ambient air contamination. RESULTS: In our bronchoscopy simulation model, we noticed extensive aerosol generation, droplet spread and surface contamination. Exposure of health-care workers and contamination of surfaces can be efficiently reduced by repurposing covers for ultrasound transducers or endoscopic cameras to seal the tube opening during bronchoscopy in mechanically ventilated patients. CONCLUSION: Adequate personal protective equipment and safety strategies allow to minimise contamination during bronchoscopy in mechanically ventilated COVID-19 patients.

Koh, F. H., et al. (2020). "Full-Dress Rehearsals with Actual Non-Infected Patients Builds Confidence and Familiarity - An Experience from Singapore during the COVID-19 Pandemic." J Invest Surg: 1-3.

PURPOSE: COVID-19 has spread throughout the world with merciless pace. Personal protective equipment (PPE) is required to protect healthcare workers from contracting the virus. The authors evaluated the use of full-dress rehearsals (FDR) to familiarize staff with protocols and equipment that are not often use otherwise. METHODS: A single institution in Singapore performed FDR with noninfected patients for endoscopy after refresher courses with PPE were conducted. FDRs were conducted between February and March 2020, during the initial phase of the COVID-19 outbreak. The FDRs involved all phases of endoscopy. Statistics from the FDRs were compared against baseline procedure statistics. A self-administered survey was conducted for the procedurists after the FDRs. RESULTS: A total of 26 FDRs were conducted and 20 (77%) completed the survey. Among all the FDRs conducted, there was a slight increase in duration for colonoscopy FDRs (21.5 min vs 20.0 min, p = 0.22). There were no complications. All respondents had a good experience (score rating of 4-5/5). On qualitative analysis, the themes identified were: FDRs provided a good opportunity to have hands-on familiarization of the equipment on top of routine refresher training. Participants were cognizant of the ongoing outbreak and the evolving threat. Safety measures were reinforced. Procedurists who acknowledged weaknesses, requested for more training. CONCLUSIONS: Simulation training with patients provides participants with a level of realism that is critical for rapid learning. In crisis management, the most effective protocol familiarization is important to instill confidence in the PPE, workflows and healthcare partners.

Kokudo, N. and H. Sugiyama (2020). "Call for international cooperation and collaboration to effectively tackle the COVID-19 pandemic." Glob Health Med **2**(2): 60-62.

The world is facing an unprecedented challenge in every place that is affected by the spreading COVID-19 pandemic. With China recording fewer and fewer cases, Europe and the Americas have become the epicenter of the pandemic since mid-March 2020, respectively accounting for 54.8% (621,407) and 27.8% (315,714) of 1,133,758 confirmed cases globally as of April 5. Moreover, the number of confirmed cases in the US (273,808), Spain (124,736), Italy (124,632), and Germany (91,714) has exceeded the number in China (82,930) so far. International cooperation and coordination are essential to tackling this pandemic in terms of both assistance with emergency medical supplies and medical technical assistance. Coordinated global action has been called for by the World Health Organization (WHO), G7, G20, the World Trade Organization (WTO), and other bodies. More effective actions are urgently needed to protect the most vulnerable, including older people and people with an underlying medical condition, as well as healthcare workers, who are most frequently exposed and who are vital to the response.

Kong, Q. and L. Yan (2020). "Prevention and Control Strategies for Coronavirus Disease-2019 in a Tertiary Hospital in the Middle East of China." Risk Manag Healthc Policy **13**: 1563-1569.

BACKGROUND: Coronavirus disease-2019 (COVID-19) is a new infectious disease. The COVID-19 outbreak has quickly spread globally and now is having remarkable effects on public health and national economies. PURPOSE: Effective measures for prevention and control of nosocomial infection are urgently needed to control COVID-19 infection among medical staff. METHODS: Herein, we described COVID-19 prevention and control strategies that have been implemented in a tertiary hospital in the middle east of China. Hospital layout was adjusted to prevent and control transmission. Other procedures were then implemented to prevent the spread of the virus, including specialized training and evaluation of medical staff, pre-testing and triage, measures to promote early identification of patients, environmental cleaning and disinfection, new medical waste processing procedures, new patient management procedures, timely disinfection of potential sources of nosocomial infection, and strict protective measures. RESULTS: Since the new measures were implemented, to date there have only been three new confirmed cases of COVID-19 infection, no cases of medical staff infection, and no COVID-19-related deaths at the hospital. CONCLUSION: The current report provides a protocol that could be implemented in medical departments in similar cities worldwide.

Köseoğlu Toksoy, C., et al. (2021). "Headache related to mask use of healthcare workers in COVID-19 pandemic." Korean J Pain **34**(2): 241-245.

BACKGROUND: It has been reported that a new type of headache may develop as a result of face mask use during the COVID-19 pandemic. The aim of this study is to investigate the clinical features of face mask-related headache during the COVID-19 pandemic. METHODS: This is a cross-sectional study carried out on healthcare workers at the Afyonkarahisar Health Sciences University. The number of workers at our university was established. Sample size was calculated using the G*Power program. A questionnaire consisting of questions relating to pre-existing headache, an aggravation in headache, and de-novo headache was filled out by 3 neurologists with all participants. RESULTS: Data was collected from a total of 375 participants, after the exclusion of 5 individuals who refused to participate. Out of all participants, 26 (6.9%) used a filtering mask, 274 (73.1%) used a surgical mask, 75 (20.0%) participants used a combination of both masks. The number of participants with preexisting headache was 114 (30.4%) had pre-existing headache. Of those with pre-existing headache, 77 (67.5%) healthcare workers had reported an aggravation in their headache after mask use. De-novo headache was observed in 116 (30.9%) of participants. De-novo headache characteristics included throbbing in 17 (14.7%) participants and pressing in 99 (85.3%) participants. In addition, symptoms such as tachypnea, sleep disturbance, and fatigue were found to be significantly higher. CONCLUSIONS: This study indicates that healthcare workers develop headaches due to use of masks during the COVID-19 outbreak.

Kotfis, K., et al. (2020). "COVID-19: What do we need to know about ICU delirium during the SARS-CoV-2 pandemic?" Anaesthesiol Intensive Ther **52**(2): 132-138.

In March 2020, the World Health Organisation announced the COVID-19 pandemic caused by the SARS-CoV-2 virus. As well as respiratory failure, the SARS-CoV-2 may cause central nervous system (CNS) involvement, including delirium occurring in critically ill patients (ICU delirium). Due attention must be paid to this subject in the face of the COVID-19 pandemic. Delirium, the detection of which takes less than two minutes, is frequently underestimated during daily routine ICU care, but it may be a prodromal symptom of infection or hypoxia associated with severe respiratory failure. During the COVID-19 pandemic, systematic delirium monitoring using validated tests (CAM-ICU or ICDSC) may be sacrificed. This is likely to be due to the fact that the main emphasis is placed on organisational issues, i.e. the lack of ventilators, setting priorities for limited mechanical ventilation options, and a shortage of personal protective equipment. Early identification of patients with delirium is critical in patients with COVID-19 because the occurrence of delirium may be an early symptom of worsening respiratory failure or of infectious spread to the CNS mediated by potential neuroinvasive mechanisms of the coronavirus. The purpose of this review is to identify problems related to the development of delirium during the COVID-19 epidemic, which are presented in three areas: i) factors contributing to delirium in COVID-19, ii) potential pathophysiological factors of delirium in COVID-19, and iii) long-term consequences of delirium in COVID-19. This article discusses how healthcare workers can reduce the burden of delirium by identifying potential risk factors and difficulties during challenges associated with SARS-CoV-2 infection.

Kothekar, A. T. and A. P. Kulkarni (2020). "Basic Principles of Disinfection and Sterilization in Intensive Care and Anesthesia and Their Applications during COVID-19 Pandemic." Indian J Crit Care Med **24**(11): 1114-1124.

Understanding the concepts of disinfection, sterilization, cleaning and asepsis is of prime importance to prevent transmission of infection to patients and to protect healthcare workers (HCWs). Proper disinfection of surfaces after cleaning, an important consideration at all times, has assumed special significance during the current pandemic. The global shortage of disposable equipment such as personal protective equipment (PPE), specifically N95 masks and surgical 3 ply masks, and other items makes the HCWs vulnerable to transmission of infection while caring for these patients. Therefore, resterilization of such items has assumed equal importance. Cleaning, the first step in the process of sterilization, is of vital importance to reduce bioburden. The type of disinfection required depends on the nature of the equipment and its intended use. For example, critical items need high-level decontamination. In this narrative review, we elaborate on the methods of decontamination and sterilization. Many chemicals can be used for both sterilization and disinfection, and the difference lies in the concentration of the chemical and exposure time. We have also summarized strategies which can be used for resterilization of single-use items, in view of the shortages caused by the current pandemic. HOW TO CITE THIS ARTICLE: Kothekar AT, Kulkarni AP. Basic Principles of Disinfection and Sterilization in Intensive Care and Anesthesia and Their Applications during COVID-19 Pandemic. Indian J Crit Care Med 2020;24(11):1114-1124.

Koti, M., et al. (2020). "BCG vaccine and COVID-19: implications for infection prophylaxis and cancer immunotherapy." J Immunother Cancer **8**(2).

The COVID-19 pandemic has killed over 400 000 people globally. Ecological evidence indicates that countries with national universal BCG vaccination programs for tuberculosis (TB) prevention have a much lower incidence of severe COVID-19 and mortality compared with those that do not have such programs. BCG is a century old vaccine used for TB prevention via infant/childhood vaccination in lowto middle-income countries with high infection prevalence rate and is known to reduce all-cause neonatal mortality. BCG remains the standard immunotherapy treatment for patients with high-risk non-muscle invasive bladder cancer globally for more than 44 years. Several trials are, therefore, investigating BCG as a prophylactic against COVID-19 in healthcare workers and the elderly. In this commentary, we discuss the potential mechanisms that may underlie BCG associated heterologous protection with a focus on tertiary lymphoid structure (TLS) organogenesis. Given the significance of TLSs in mucosal immunity, their association with positive prognosis and response to immune checkpoint blockade with a critical role of Type I interferon (IFN-1) in inducing these, we also discuss potentiating TLS formation as a promising approach to enhance anti-tumor immunity. We propose that lessons learned from BCG immunotherapy success could be applied to not only augment such microbe-based therapeutics but also lead to similar adjunctive IFN-1 activating approaches to improve response to immune checkpoint blockade therapy in cancer.

Kowalski, L. P., et al. (2020). "Effect of the COVID-19 Pandemic on the Activity of Physicians Working in the Areas of Head and Neck Surgery and Otorhinolaryngology." Int Arch Otorhinolaryngol **24**(3): e258-e266.

Introduction  Coronavirus disease 2019 (COVID-19) is an acute infection caused by the new coronavirus (SARS-CoV-2) and it is highly transmissible, especially through respiratory droplets. To prepare the health system for the care of these patients also led to a restriction in the activity of several medical specialties. Physicians who work with patients affected by diseases of the head and neck region constitute one of the populations most vulnerable to COVID-19 and also most affected by the interruption of their professional activities. Objective  The aim of the present study was to assess the impact of the COVID-19 pandemic on the practice of head and neck surgeons and otorhinolaryngologists in Brazil. Methods  An anonymous online survey of voluntary participation was applied, containing 30 questions regarding demographic aspects, availability of personal protective equipment (PPE), and impact on the routine of head and neck surgeons and otorhinolaryngologists, as well as clinical oncologists and radiation oncologists who work with head and neck diseases. Results  Seven hundred and twenty-nine answers were received in a period of 4 days, ∼ 40 days after the 1 (st) confirmed case in Brazil. With professionals working in public and private services, there was a high level of concerns with the disease and its consequences, limited availability of PPE and a significant decrease in the volume of specialized medical care. Conclusion  The study demonstrated a direct impact of the COVID-19 pandemic on the clinical practice of specialties related to the treatment of patients with diseases of the head and neck region already in the beginning of the illness management in Brazil.

Kozanhan, B., et al. (2020). "Can "YouTube" help healthcare workers for learning accurate donning and doffing of personal protective equipments?" Enferm Infecc Microbiol Clin.

INTRODUCTION: Healthcare workers have a high risk of cross-infection during the care of Covid-19 cases. Personal protective equipment can reduce the risk. However, healthcare workers must be trained for the proper use of personal protective equipment to decrease exposure risk. This study aimed to investigate whether videos available on YouTube, presenting procedures of donning and doffing personal protective equipment, can be a useful learning resource for healthcare workers. METHODS: A search of YouTube was conducted using the keywords "Covid-19, personal protective equipment, donning, doffing". Two investigators reviewed each video and collected the basic video information. Total videos were assessed independently as educationally useful and non-useful categories using a valid tool. The relationship of each video's usefulness with viewers' preferences and the upload source were analyzed. RESULTS: A total of 300 videos were assessed; 66 (22%) fulfilled the inclusion criteria. Total video scores of educationally useful videos were higher than non-useful ones; the differences were significant. Healthcare/government agencies and hospitals mostly created educationally useful videos, e-learning platforms, and individuals mainly created non-useful videos. Significant correlations were observed between the video's usefulness and the total view and views per day. CONCLUSIONS: During a pandemic, YouTube might be a resource for learning donning and doffing of personal protective equipment for healthcare workers if an appropriate selection process applied for determining educationally useful videos.

Publisher: Abstract available from the publisher.

spa

Kraus, A., et al. (2020). "Practical Solutions for Healthcare Worker Protection During the COVID-19 Pandemic Response in the Ambulatory, Emergency, and Inpatient Settings." J Occup Environ Med **62**(11): e616-e624.

OBJECTIVE: Protecting healthcare workers is an essential component of a successful response to the COVID-19 pandemic. The resource intensive nature of infectious disease protection, budgetary constraints, and global shortages of personal protective equipment (PPE) make this a daunting task. Practical, easily implemented strategies for healthcare workers (HCW) protection are needed. METHODS: We cross-reference the "Systems, Space, Staff, and Stuff" paradigm from disaster management and the "Hierarchy of Controls" approach to infection prevention from the Center for Disease Control and Prevention (CDC) to generate a narrative overview of worker protection strategies relevant to COVID-19. RESULTS: Alternative types of PPE, management of hazards, and reorganizing how people work can optimize HCWs protection. CONCLUSIONS: A comprehensive PPE strategy can utilize the "systems, space, staff, stuff" paradigm of disaster management to identify new or underutilized solutions to HCWs protection.

Krok, D., et al. (2021). "Risk of Contracting COVID-19, Personal Resources and Subjective Well-Being among Healthcare Workers: The Mediating Role of Stress and Meaning-Making." J Clin Med **10**(1).

The latest research suggests that the relationships between the risk of contracting COVID-19, personal resources and subjective well-being have rather an indirect character and can include the occurrence of mediating factors related to meaning-making processes and stress experiences. Protection motivation theory offers a theoretical paradigm that enables these associations to be thoroughly investigated and understood. The current study aimed to examine the mediating roles of meaning-making and stress in the relationship of risk of contracting COVID-19 and personal resources (self-efficacy and meaning in life) with subjective well-being among healthcare workers. A total of 225 healthcare workers from hospitals, medical centres and diagnostic units completed a set of questionnaires during the first few months of the COVID-19 lockdown period (March-May 2020). The results revealed that greater self-efficacy and meaning in life were associated with higher cognitive and affective dimensions of subjective well-being, whereas a lesser risk of contracting COVID-19 was only associated with the higher affective dimension. The central finding demonstrated different mediating roles of stress and meaning-making in the relationship of risk of contracting COVID-19 and personal resources with the cognitive and affective dimensions of subjective well-being. This confirmed the applicability of meaning-oriented and stress management processes for understanding how healthcare workers' well-being is affected during the COVID-19 pandemic.

Król, Z., et al. (2020). "Transformation of a large multi-speciality hospital into a dedicated COVID-19 centre during the coronavirus pandemic." Ann Agric Environ Med **27**(2): 201-206.

INTRODUCTION: The article describes the process of converting a large multi-specialized hospital into one dedicated to COVID-19 patients, and present established standards of work organization in all the wards and training system of the medical and supporting staff. The several weeks pandemic of the COVID-19 disease has forced the healthcare systems of numerous countries to adjust their resources to the care of the growing number of COVID-19 patients. Managers were presented with the challenge of protecting the healthcare workers from transmission of the disease within medical institutions, and issues concerning the physical and psychological depletion of personnel. MATERIAL AND METHODS: Based on analyses of the structure and work processes in Central Clinical Hospital (CCH) reconstructive strategic plan was developed. It included: division of existing wards into observation and isolation wards; installing locks; weekly plan for supplying personal protection equipment (PPE); designating new access to the hospital and communication routes; training of medical and supporting staff. The plan was implemented from the first days of conversion of the hospital. RESULTS: The wards of the CCH were converted for observation and isolation, and each one was fitted with sanitary locks. There was a big improvement in the supply of PPE for the medical staff. Separation of the 'dirty' and 'clean' parts of the CCH were attained, and widespread intensive training not only protected personnel against infections, but also diminished unrest which was discernable at the beginning of conversion. CONCLUSIONS: The transformation efforts will ultimately be appraised at the end of the epidemic, but the data looks encouraging. Two weeks after conversion, the testing of hospital Staff was started and by the end of April, 459 tests were had been conducted, of which only 11 were positive.

Ku, M., et al. (2020). "Provision of a consistent national approach to radiation therapy workforce protection measures in Australia during the COVID-19 pandemic." Aust Health Rev **44**(4): 535-539.

Healthcare workers are at the frontline managing COVID-19 patients with transmission of the COVID-19 virus to healthcare workers evident in many Australian states. Minimisation of this spread is vital to protecting the healthcare workforce with individual organisations detailing best practice for infection and control. However, interpretation and implementation of infection control guidelines is varied across Australian Radiation Therapy Departments, highlighting inconsistencies. Strong leadership, quality communication and clear direction is required during this crisis to ensure that radiation therapists receive all necessary support and resources required to maintain safety and well-being during the COVID-19 pandemic.

Kua, J., et al. (2021). "healthcareCOVID: a national cross-sectional observational study identifying risk factors for developing suspected or confirmed COVID-19 in UK healthcare workers." PeerJ **9**: e10891.

OBJECTIVE: To establish the prevalence, risk factors and implications of suspected or confirmed coronavirus disease 2019 (COVID-19) infection among healthcare workers in the United Kingdom (UK). DESIGN: Cross-sectional observational study. SETTING: UK-based primary and secondary care. PARTICIPANTS: Healthcare workers aged ≥18 years working between 1 February and 25 May 2020. MAIN OUTCOME MEASURES: A composite endpoint of laboratory-confirmed diagnosis of SARS-CoV-2, or self-isolation or hospitalisation due to suspected or confirmed COVID-19. RESULTS: Of 6,152 eligible responses, the composite endpoint was present in 1,806 (29.4%) healthcare workers, of whom 49 (0.8%) were hospitalised, 459 (7.5%) tested positive for SARS-CoV-2, and 1,776 (28.9%) reported self-isolation. Overall, between 11,870 and 21,158 days of self-isolation were required by the cohort, equalling approximately 71 to 127 working days lost per 1,000 working days. The strongest risk factor associated with the presence of the primary composite endpoint was increasing frequency of contact with suspected or confirmed COVID-19 cases without adequate personal protective equipment (PPE): 'Never' (reference), 'Rarely' (adjusted odds ratio 1.06, (95% confidence interval: [0.87-1.29])), 'Sometimes' (1.7 [1.37-2.10]), 'Often' (1.84 [1.28-2.63]), 'Always' (2.93, [1.75-5.06]). Additionally, several comorbidities (cancer, respiratory disease, and obesity); working in a 'doctors' role; using public transportation for work; regular contact with suspected or confirmed COVID-19 patients; and lack of PPE were also associated with the presence of the primary endpoint. A total of 1,382 (22.5%) healthcare workers reported lacking access to PPE items while having clinical contact with suspected or confirmed COVID-19 cases. CONCLUSIONS: Suspected or confirmed COVID-19 was more common in healthcare workers than in the general population and is associated with significant workforce implications. Risk factors included inadequate PPE, which was reported by nearly a quarter of healthcare workers. Governments and policymakers must ensure adequate PPE is available as well as developing strategies to mitigate risk for high-risk healthcare workers during future COVID-19 waves.

Kulkarni, A. P., et al. (2020). "Cardiopulmonary Resuscitation during COVID-19 Pandemic: Outcomes, Risks, and Protective Strategies for the Healthcare Workers and Ethical Considerations." Indian J Crit Care Med **24**(9): 868-872.

The crisis caused by Coronavirus disease-2019 (COVID-19) pandemic has led us to safeguard ourselves and our colleagues against transmission of this highly contagious infection, while aiming for the same goals of care. In spite of the stringent measures adopted by affected countries, rising number of healthcare workers (HCWs) are getting infected, dwindling the scarce manpower at our disposal. In the pre-COVID-19 times, cardiopulmonary resuscitation (CPR) was offered unhesitantly to all patients, who had even a slim chance of achieving return of spontaneous circulation. In COVID-19 era, CPR, due to some components being high aerosol-generating procedures (AGPs), has become high-risk procedure for the HCWs. Instead of "Primum non nocere" (first do no harm), we are forced to change to "Primum non nocere ad te" (first do no harm to yourself). The challenge is therefore to provide best possible chance of survival to deserving patients, whose COVID-19 status might be unknown, without causing harm to the HCWs. In this review, we discuss the current data regarding infected HCWs, outcomes of inhospital and out-of-hospital cardiac arrests, components of CPR which are high-risk AGPs, how to safeguard the HCWs while offering CPR, and the ethical considerations when CPR is considered, in this COVID-19 era. We wish to emphasize here that there is NO EMERGENCY in a pandemic, and time must be made for donning appropriate PPE. We feel that clear policies need to be developed by the institutions to deliver CPR to correct population, in this challenging period. How to cite this article: Kulkarni AP, Singh Y, Garg H, Jha S. Cardiopulmonary Resuscitation during COVID-19 Pandemic: Outcomes, Risks, and Protective Strategies for the Healthcare Workers and Ethical Considerations. Indian J Crit Care Med 2020;24(9):868-872.

Kumar, K. S., et al. (2021). "Deployable Telescopic Tubular Mechanisms With a Steerable Tongue Depressor Towards Self-Administered Oral Swab." Front Robot AI **8**: 612959.

Swabbing tests have proved to be an effective method of diagnosis for a wide range of diseases. Potential occupational health hazards and reliance on healthcare workers during traditional swabbing procedures can be mitigated by self-administered swabs. Hence, we report possible methods to apply closed kinematic chain theory to develop a self-administered viral swab to collect respiratory specimens. The proposed sensorized swab models utilizing hollow polypropylene tubes possess mechanical compliance, simple construction, and inexpensive components. In detail, the adaptation of the slider-crank mechanism combined with concepts of a deployable telescopic tubular mechanical system is explored through four different oral swab designs. A closed kinematic chain on suitable material to create a developable surface allows the translation of simple two-dimensional motion into more complex multi-dimensional motion. These foldable telescopic straws with multiple kirigami cuts minimize components involved in the system as the characteristics are built directly into the material. Further, it offers a possibility to include soft stretchable sensors for realtime performance monitoring. A variety of features were constructed and tested using the concepts above, including 1) tongue depressor and cough/gag reflex deflector; 2) changing the position and orientation of the oral swab when sample collection is in the process; 3) protective cover for the swabbing bud; 4) a combination of the features mentioned above.

Kumar, P., et al. (2020). "Ultraviolet Box: An Innovative In-house Use of Ultraviolet Irradiation for Conservation of Respirators in COVID-19 Pandemic." Indian J Crit Care Med **24**(8): 713-715.

AIM: To use ultraviolet (UV) radiations in an indigenous method for sterilization of respirators for reuse during COVID-19 outbreak. BACKGROUND: COVID-19 outbreak has infected more than 200 countries. In India, till now, more than 100,000 cases have been reported. Healthcare workers are at high risk of developing infections being in the frontline of taking care of COVDI-19 cases. The demands of personal protective equipment (PPE) are increasing, but the same is not matched with supply due to various reasons. In such scenarios, reusing respirators and face shields is an alternative. UV radiations have quick action and are able to preserve the quality of respirators. We have developed a UV box for surface sterilization of respirators with an intention to reuse. TECHNIQUE: A thermocol box was taken from the central drug store and was fitted with two UV tubes of 254 nm wavelength procured from local service center of water purifiers. The position of the two tubes was such that one was near the base while other was fixed at the top. An aluminum mesh frame was placed in the middle of the box to act as a platform. The roof of the box was converted into a lid. The effectiveness of assembly was tested using culture of Escherichia coli and Staphylococcus aureus. In addition, a biological indicator tube containing test strip with spores of Bacillus atrophaeus was also exposed to UV light for a predefined duration, which did not show any color change after incubation for 48 hours. CONCLUSION: Our prototype assembly with supported efficacy from microbiological tests is an option for use of UV light within available resources for disinfection and reuse of scarce supplies of personal protective equipment. CLINICAL SIGNIFICANCE: UV box can help in meeting the demand supply deficit for respirators, face shields, and goggles that are paramount for the protection of HCW. HOW TO CITE THIS ARTICLE: Kumar P, Chaudhry D, Parmar A, Tyagi D, BG Manjunath, Singh PK. Ultraviolet Box: An Innovative In-house Use of Ultraviolet Irradiation for Conservation of Respirators in COVID-19 Pandemic. Indian J Crit Care Med 2020;24(8):713-715.

Kumar, S. and H. P. Lee (2020). "The perspective of fluid flow behavior of respiratory droplets and aerosols through the facemasks in context of SARS-CoV-2." Phys Fluids (1994) **32**(11): 111301.

In the unfortunate event of the current ongoing pandemic COVID-19, where vaccination development is still in the trial phase, several preventive control measures such as social distancing, hand-hygiene, and personal protective equipment have been recommended by health professionals and organizations. Among them, the safe wearing of facemasks has played a vital role in reducing the likelihood and severity of infectious respiratory disease transmission. The reported research in facemasks has covered many of their material types, fabrication techniques, mechanism characterization, and application aspects. However, in more recent times, the focus has shifted toward the theoretical investigations of fluid flow mechanisms involved in the virus-laden particles' prevention by using facemasks. This exciting research domain aims to address the complex fluid transport that led to designing a facemask with a better performance. This Review discusses the recent updates on fluid flow dynamics through the facemasks. Key design aspects such as thermal comfort and flow resistance are discussed. Furthermore, the recent progress in the investigations on the efficacy of facemasks for the prevention of COVID-19 spread and the impact of wearing facemasks is presented.

Kumar, S. S., et al. (2020). "Risk factors and outcome among COVID-19 exposed and quarantined healthcare workers: A study on the status of existing practices of standard precautions." J Family Med Prim Care **9**(10): 5355-5359.

CONTEXT: Health care workers (HCWs) are at high risk of COVID-19 infection but data on the risk factors for exposure and infection rate among Indian HCWs are limited. AIMS: Our study aims to identify the risk factors and behavior of HCWs which make them high risk for COVID-19 infection and the infection rate among them. SETTINGS AND DESIGN: This is a retrospective study conducted at All India Institute of Medical Sciences, New Delhi. METHODS AND MATERIAL: Fifty HCWs quarantined at our institute in April and May 2020 following exposure to confirmed or suspected COVID-19 cases, or due to development of Influenza-Like Illness (ILI) were included. Data was collected from medical records in a predesigned proforma and analyzed. RESULTS: Thirty-eight (76%) of the 50 quarantined HCWs had high-risk exposure and there was a significant breach in personal protective measures. N-95 masks were worn by 59.6%, gloves by 61.7%, and goggles or face shields by 2%. Exposures were more common in non-COVID areas of the hospital. Hydroxychloroquine pre-exposure prophylaxis was taken by 7 (14%). 3 (6%) were confirmed to be COVID-19 positive during the quarantine period. CONCLUSIONS: Our study has shown leniency among HCWs in adhering to infection control and personal protective measures resulting in an increased quarantine and infection rate and loss of manpower. The safety of our HCWs must be given paramount importance during this pandemic and should be ensured by educating them about infection control, and persistently reinforcing and strictly adhering to standard precautions.

Kunz, Y., et al. (2020). "[What should urologists know about SARS-CoV-2? Risk analysis for urological operations and recommendations for action in clinical routine]." Urologe A **59**(11): 1361-1370.

BACKGROUND: COVID-19 poses a challenge to healthcare systems worldwide. Due to the increasing number of cases, surgeons in urology have also been confronted with SARS-CoV‑2 infections. Thus, there is an urgent need for clinical guidance and recommendations. AIM: Our work aims to create a widespread assessment of a possible risk for infection with SARS-CoV‑2 during surgical procedures. Based on current data and current national and international guidelines, we try to assess the risk of infection when handling human tissue and the necessary hygienic measures that are needed. Finally, recommendations for daily urologic work are derived and explained. MATERIALS AND METHODS: The current literature in PubMed, bioRxiv and medRxiv and data available from the WHO and Robert-Koch-Institut on SARS-CoV‑2 and surgical procedures in (potentially) infected patients are reviewed. The endpoint of our research was 21 April 2020. CONCLUSION: Based on our research, general and specific recommendations for clinical urologic praxis can be derived. Although it remains unclear whether SARS-CoV‑2 is transmitted via the aerosols produced, current PPE in operating rooms probably does not offer sufficient protection during surgical interventions during the SARS-CoV‑2 pandemic. Use of FFP‑2 masks, safety goggles and full-body protective suits is crucial. To contain viral spread on surfaces and personnel, complex filter systems (HEPA) should be used as well as closed suction devices during surgery. Combined with consequent disinfection of surfaces and behavioral measures, a safe environment for healthcare workers in urology can be created. Thus, according to current knowledge, we believe that emergency and urgent surgical procedures are not contraindicated, provided that appropriate precautionary safety measures are followed.

Publisher: Abstract available from the publisher.

ger

Kunzler, A. M., et al. (2021). "Mental burden and its risk and protective factors during the early phase of the SARS-CoV-2 pandemic: systematic review and meta-analyses." Global Health **17**(1): 34.

BACKGROUND: Mental burden due to the SARS-CoV-2 pandemic has been widely reported for the general public and specific risk groups like healthcare workers and different patient populations. We aimed to assess its impact on mental health during the early phase by comparing pandemic with prepandemic data and to identify potential risk and protective factors. METHODS: For this systematic review and meta-analyses, we systematically searched PubMed, PsycINFO, and Web of Science from January 1, 2019 to May 29, 2020, and screened reference lists of included studies. In addition, we searched PubMed and PsycINFO for prepandemic comparative data. Survey studies assessing mental burden by the SARS-CoV-2 pandemic in the general population, healthcare workers, or any patients (eg, COVID-19 patients), with a broad range of eligible mental health outcomes, and matching studies evaluating prepandemic comparative data in the same population (if available) were included. We used multilevel meta-analyses for main, subgroup, and sensitivity analyses, focusing on (perceived) stress, symptoms of anxiety and depression, and sleep-related symptoms as primary outcomes. RESULTS: Of 2429 records retrieved, 104 were included in the review (n = 208,261 participants), 43 in the meta-analysis (n = 71,613 participants). While symptoms of anxiety (standardized mean difference [SMD] 0.40; 95% CI 0.15-0.65) and depression (SMD 0.67; 95% CI 0.07-1.27) were increased in the general population during the early phase of the pandemic compared with prepandemic conditions, mental burden was not increased in patients as well as healthcare workers, irrespective of COVID-19 patient contact. Specific outcome measures (eg, Patient Health Questionnaire) and older comparative data (published ≥5 years ago) were associated with increased mental burden. Across the three population groups, existing mental disorders, female sex, and concerns about getting infected were repeatedly reported as risk factors, while older age, a good economic situation, and education were protective. CONCLUSIONS: This meta-analysis paints a more differentiated picture of the mental health consequences in pandemic situations than previous reviews. High-quality, representative surveys, high granular longitudinal studies, and more research on protective factors are required to better understand the psychological impacts of the SARS-CoV-2 pandemic and to help design effective preventive measures and interventions that are tailored to the needs of specific population groups.

Kuo, F. L., et al. (2020). "Survey on perceived work stress and its influencing factors among hospital staff during the COVID-19 pandemic in Taiwan." Kaohsiung J Med Sci **36**(11): 944-952.

This study aimed to investigate the perceived work stress and its influencing factors among hospital staff during the novel coronavirus (COVID-19) pandemic in Taiwan. A web-based survey was conducted at one medical center and two regional hospitals in southern Taiwan, targeting physicians, nurses, medical examiners, and administrators. The questionnaire included items on the demographic characteristics of hospital staff and a scale to assess stress among healthcare workers caring for patients with a highly infectious disease. A total of 752 valid questionnaires were collected. The hospital staff reported a moderate level of stress and nurses had a highest level of stress compared to staff in the other three occupational categories. The five highest stress scores were observed for the items "rough and cracked hands due to frequent hand washing and disinfectant use," "inconvenience in using the toilet at work," "restrictions on eating and drinking at work," "fear of transmitting the disease to relatives and friends," and "fear of being infected with COVID-19." Discomfort caused by protective equipment was the major stressor for the participants, followed by burden of caring for patients. Among participants who experienced severe stress (n = 129), work stress was higher among those with rather than without minor children. The present findings may serve as a reference for future monitoring of hospital staff's workload, and may aid the provision of support and interventions.

Kutti-Sridharan, G., et al. (2020). "SARS-CoV2 in Different Body Fluids, Risks of Transmission, and Preventing COVID-19: A Comprehensive Evidence-Based Review." Int J Prev Med **11**: 97.

The world is combating a common and invisible enemy severe acute respiratory syndrome coronavirus 2 (SARS-CoV2), a highly transmissible virus responsible for serious respiratory illness coronavirus disease-2019 (COVID-19). As with all respiratory viruses, public health measures are focused on contact tracing, isolation, and treatment of affected individuals, who have respiratory symptoms. However, it is spreading efficiently, and it can be explained from its stealth transmission from presymptomatic and asymptomatic individuals. Droplet and contact precautions are followed universally. Healthcare workers are at higher risk of acquiring infection and they are additionally required to follow airborne and eye protection. Recent studies indicate viral particles can be isolated from many body fluids including feces, saliva, semen, and tears, suggesting transmission could be possibly occurring through some of these routes as well. We have done an evidence-based review of all potential modes of transmission and discussed preventive measures to stop the spread. There is an urgent need for educating the healthcare professionals, governments, and public regarding other potential modes of transmission. Strict preventive measures need to be used to stop the spread.

Kwan, W. M., et al. (2020). "Bundled interventions for consumption management and monitoring of personal protective equipment in COVID-19 pandemic in Hong Kong local hospitals." BMJ Open Qual **9**(4).

Since the outbreak of COVID-19 in December 2019, there had been global shortage of personal protective equipment (PPE) supply due to the breakage of supply chain and also the forbidding of PPE exported by various countries. This situation had greatly affected the healthcare services in local hospitals of Hong Kong. To maintain the availability of PPE for healthcare workers in high-risk clinical settings, the cluster management of New Territories West Cluster, Hospital Authority, had implemented a bundle of interventions in controlling and managing the PPE consumption and ensuring its proper use. A Taskforce on Management of PPE was set up in February 2020 with the aim to monitor and manage the use of PPE in five local hospitals and eight general outpatient clinics of New Territories West Cluster, which were governed in a cluster basis, under the COVID-19 epidemic. Interventions including cutting down non-essential services, implementing telecare, monitoring PPE consumption at unit level and PPE stock at the Cluster Central Distribution Centre and forming mobile infection teams were implemented. The updated PPE standards and usage guidelines to clinical staff were promulgated through forums, newsletters and unit visits. The PPE consumption rates of individual unit were reviewed. Significant decrease in PPE consumption rates was noted when comparing with the baseline data. Comparing the data between 20 February and 1 June 2020, the overall PPE consumption rates were reduced by 64% (r=-0.841; p<0.001) while the PPE consumption rates in anaesthesia and operating theatres, and isolation and surveillance wards were reduced by 47% (r=-0.506; p=0.023) and 49% (r=-0.810; p<0.001), respectively. A bundled approach, including both administrative measures and staff education, is effective in managing PPE consumption during major infection outbreaks especially when PPE supply is at risk.

Kwek, J. W., et al. (2021). "Early Outcomes of a National Cancer Center's Strategy Against COVID-19 Executed Through a Disease Outbreak Response Taskforce." JCO Oncol Pract **17**(3): e343-e354.

PURPOSE: We present the strategy of a comprehensive cancer center organized to make operations pandemic proof and achieve continuity of cancer care during the COVID-19 pandemic. METHODS: Disease Outbreak Response (DORS) measures implemented at our center and its satellite clinics included strict infection prevention, manpower preservation, prudent resource allocation, and adaptation of standard-of-care treatments. Critical day-to-day clinical operations, number of persons screened before entry, staff temperature monitoring, and personal protection equipment stockpile were reviewed as a dashboard at daily DORS taskforce huddles. Polymerase chain reaction swab tests performed for patients and staff who met defined criteria for testing of severe acute respiratory syndrome coronavirus 2 (SARS-CoV-2) infection were tracked. Descriptive statistics of outpatient attendances and treatment caseloads from February 3 to May 23, 2020, were compared with the corresponding period in 2019. RESULTS: We performed COVID-19 swabs for 80 patients and 93 staff, detecting three cancer patients with community-acquired COVID-19 infections with no nosocomial transmission. Patients who required chemotherapy, radiotherapy, or surgery and patients who are on maintenance treatment continued to receive timely treatment without disruption. The number of intravenous chemotherapy treatments was maintained at 97.8% compared with 2019, whereas that of weekly radiotherapy treatments remained stable since December 2019. All cancer-related surgeries proceeded without delay, with a 0.3% increase in workload. Surveillance follow-ups were conducted via teleconsultation, accounting for a 30.7% decrease in total face-to-face clinic consultations. CONCLUSION: Through the coordinated efforts of a DORS taskforce, it is possible to avoid nosocomial SARS-CoV-2 transmissions among patients and staff without compromising on care delivery at a national cancer center.

Kwok, K. O., et al. (2021). "Editor's Choice: Influenza vaccine uptake, COVID-19 vaccination intention and vaccine hesitancy among nurses: A survey." Int J Nurs Stud **114**: 103854.

BACKGROUND: A healthy healthcare system requires healthy healthcare workers. Protecting healthcare workers including nurses against COVID-19 is crucial, and vaccination could be a viable future option. However, vaccine hesitancy remains a global challenge. Nurses, as a trustworthy and creditable source of vaccine-related information, may build public confidence in vaccination. Hence, research on vaccine hesitancy among nurses is warranted. OBJECTIVES: This study estimated nurses' influenza vaccination behaviors and intention to receive COVID-19 vaccine when available, and examined their corresponding 5C psychological antecedents (confidence, complacency, constraints, calculation, and collective responsibility). To investigate the impact of COVID-19-related work demands, the mediation effects of work stress on the association between work demands and COVID-19 vaccination intention were also examined. DESIGN: Cross-sectional online survey SETTINGS: Nurses were invited to participate via the promotion of a professional nursing organization and by personal referrals during the COVID-19 outbreak in Hong Kong between mid-March and late April 2020. PARTICIPANTS: 1,205 eligible nurses (mean age = 40.79, SD = 10.47; 90% being female) were included in the analyses. METHODS: Demographics, influenza vaccination, intention to have COVID-19 vaccine, the 5C vaccine hesitancy components, work stress and COVID-19-related work demands (insufficient supply of personal protective equipment, involvement in isolation rooms, and unfavorable attitudes towards workplace infection control policies) were reported in the survey. RESULTS: The influenza vaccine uptake rate and the proportion intending to take COVID-19 vaccine were 49% and 63%, respectively. Influenza vaccination was associated with working in public hospitals and all 5C constructs (more confidence, more collective responsibility and less complacency, constraints, and calculation), whereas stronger COVID-19 vaccination intention was associated with younger age, more confidence, less complacency and more collective responsibility. COVID-19-related demands were associated with greater work stress, and hence stronger COVID-19 vaccination intention. CONCLUSION: The potential uptake rate of COVID-19 vaccine among nurses was suboptimal to achieve herd immunity. The 5C constructs were useful in predicting influenza vaccination and, to a lesser extent, the intention to take COVID-19 vaccine. The uncertain attributes such as effectiveness, side effects, and effective duration of the COVID-19 vaccine may contribute to this discrepancy. With less work stress among nurses in the post-pandemic period, the intention to take COVID-19 vaccine will likely drop. The 5C constructs should be infused in vaccination campaigns. While a COVID-19 vaccine could be ready soon, the nursing profession may not be ready to accept it. More research work is needed to boost the uptake rate. TWEETABLE ABSTRACT: Less than two-third of nurses intended to take COVID-19 vaccine when available. While a COVID-19 vaccine could be ready soon, nursing profession is not ready to accept it.

Laamrani, F. Z., et al. (2020). "Reorganization of the imaging units in the context of the COVID-19 pandemic: experience of the Ibn Sina University Hospital in Rabat." Pan Afr Med J **35**(Suppl 2): 30.

The global health system is currently facing the new SARS-COV 2 pandemy. This exceptional situation requires, from our African health systems, to reorganize and readapt the usual protocols when they were carried out before the crisis and/or their urgent implementation otherwise. As imaging is one of the pillars of the diagnosis of infection with this emerging virus, it was essential to rethink the imaging department organization so as to dedicate a unit to COVID-19 activity while maintaining the usual emergency activity within the Ibn Sina university hospital in Rabat. The protection of exposed personnel and the bio-cleaning of radiology equipment and rooms also became an evidence. The active involvement of the administration, the Clinical Pharmacy Department and the Nosocomial Infections Control Committee is a key to the success of this reorganization.

Ladoire, S., et al. (2021). "Seroprevalence of SARS-CoV-2 among the staff and patients of a French cancer centre after first lockdown: The canSEROcov study." Eur J Cancer **148**: 359-370.

BACKGROUND: In view of the potential gravity of severe acute respiratory syndrome coronavirus-2 (SARS-CoV-2) infection for patients with cancer, epidemiological data are vital to assess virus circulation among patients and staff of cancer centres. We performed a prospective study to investigate seroprevalence of SARS-CoV-2 antibodies among staff and patients with cancer at a large cancer centre, at the end of the period of first national lockdown in France and to determine factors associated with the risk of SARS-CoV-2 infection. METHODS: After the first lockdown, all medical and non-medical staff, as well as all patients attending the medical oncology department were invited to undergo serological testing for SARS-CoV-2 between 11 May and 30 June 2020. All participants were also invited to complete a questionnaire collecting data about their living and working conditions, and for patients, medical management during lockdown. FINDINGS: A total of 1,674 subjects (663 staff members, 1011 patients) were included. Seroprevalence was low in both staff (1.8%) and patients (1.7%), despite more features of high risk for severe forms among patients. None of the risk factors tested in our analysis (working or living conditions, comorbidities, management characteristics during lockdown) was found to be statistically associated with seroprevalence in either staff or patients. There was no significant difference in the proportion of symptomatic and asymptomatic subjects between staff and patients. Only fever, loss of smell, and loss of taste were significantly more frequent among seropositive patients, in both staff and patients. INTERPRETATION: We report very low seroprevalence of antibodies against SARS-CoV-2 in the staff (caregiving and non-caregiving) and patients of a large cancer care centre in which strict hygiene, personal protection, and social distancing measures were implemented.

Lago, P., et al. (2020). "[COVID-19 emergency management activities promoted by an university hospital in Northern Italy]." G Ital Med Lav Ergon **42**(3): 187-194.

Background. In December 2019, a Coronavirus 2019 epidemic (COVID-19) was reported, caused by a severe acute respiratory syndrome coronavirus 2 (SARS-CoV-2), which occurred in the city of Wuhan, Hubei province, China. Perceived risk of contracting diseases has led many Governments and Healthcare Organizations to implement a variety of control and protection measures for the population, in particular for health professionals who have made contact with positive Covid-19 patients. In this publication, we have carried out a review of the information available, in order to share the prevention and protection measures for health and safety at work, which a University Hospital of Pavia, in Northern Italy, has remodulated, according to the changed scenario in which professionals finds themselves carrying out their profession in the post lockdown, in account to the specificity of processes and methods of work organizing, which overall, they serve to characterize risks, in order to be able to prevent them in the best possible way for patients, visitors and healthcare professionals.

Lai, C. C., et al. (2020). "Population-based seroprevalence surveys of anti-SARS-CoV-2 antibody: An up-to-date review." Int J Infect Dis **101**: 314-322.

Severe acute respiratory syndrome coronavirus 2 (SARS-CoV-2), the causative agent of coronavirus disease 2019 (COVID-19), has led to a global pandemic. However, the majority of currently available data are restricted to laboratory-confirmed cases for symptomatic patients, and the SARS-CoV-2 infection can manifest as an asymptomatic or mild disease. Therefore, the true extent of the burden of COVID-19 may be underestimated. Improved serological detection of specific antibodies against SARS-CoV-2 could help estimate the true numbers of infections. This article comprehensively reviews the associated literature and provides updated information regarding the seroprevalence of the anti-SARS-CoV-2 antibody. The seroprevalence can vary across different sites and the seroprevalence can increase with time during longitudinal follow-up. Although healthcare workers (HCWs), especially those caring for COVID-19 patients, are considered as a high-risk group, the seroprevalence in HCWs wearing adequate personal protective equipment is thought to be no higher than that in other groups. With regard to sex, no statistically significant difference has been found between male and female subjects. Some, but not all, studies have shown that children have a lower risk than other age groups. Finally, seroprevalence can vary according to different populations, such as pregnant women and hemodialysis patients; however, limited studies have examined these associations. Furthermore, the continued surveillance of seroprevalence is warranted to estimate and monitor the growing burden of COVID-19.

Lai, T. H. T., et al. (2020). "Stepping up infection control measures in ophthalmology during the novel coronavirus outbreak: an experience from Hong Kong." Graefes Arch Clin Exp Ophthalmol **258**(5): 1049-1055.

PURPOSE: Coronavirus disease (COVID-19) has rapidly emerged as a global health threat. The purpose of this article is to share our local experience of stepping up infection control measures in ophthalmology to minimise COVID-19 infection of both healthcare workers and patients. METHODS: Infection control measures implemented in our ophthalmology clinic are discussed. The measures are based on detailed risk assessment by both local ophthalmologists and infection control experts. RESULTS: A three-level hierarchy of control measures was adopted. First, for administrative control, in order to lower patient attendance, text messages with an enquiry phone number were sent to patients to reschedule appointments or arrange drug refill. In order to minimise cross-infection of COVID-19, a triage system was set up to identify patients with fever, respiratory symptoms, acute conjunctivitis or recent travel to outbreak areas and to encourage these individuals to postpone their appointments for at least 14 days. Micro-aerosol generating procedures, such as non-contact tonometry and operations under general anaesthesia were avoided. Nasal endoscopy was avoided as it may provoke sneezing and cause generation of droplets. All elective clinical services were suspended. Infection control training was provided to all clinical staff. Second, for environmental control, to reduce droplet transmission of COVID-19, installation of protective shields on slit lamps, frequent disinfection of equipment, and provision of eye protection to staff were implemented. All staff were advised to measure their own body temperatures before work and promptly report any symptoms of upper respiratory tract infection, vomiting or diarrhoea. Third, universal masking, hand hygiene, and appropriate use of personal protective equipment (PPE) were promoted. CONCLUSION: We hope our initial experience in stepping up infection control measures for COVID-19 infection in ophthalmology can help ophthalmologists globally to prepare for the potential community outbreak or pandemic. In order to minimise transmission of COVID-19, ophthalmologists should work closely with local infection control teams to implement infection control measures that are appropriate for their own clinical settings.

Lai, X., et al. (2020). "What influences the infection of COVID-19 in healthcare workers?" J Infect Dev Ctries **14**(11): 1231-1237.

INTRODUCTION: The outbreak of COVID-19 has spread worldwide. The evidence about risk factors of healthcare workers who infected COVID-19 is limited. This study aims to describe characteristics and influencing factors of the COVID-19 infection in healthcare workers. METHODOLOGY: The study was performed among COVID-19 infected and uninfected healthcare workers in three hospitals in Wuhan. A total of 325 healthcare workers participated; among them 151 COVID-19-infected healthcare workers were included. Characteristics of infected healthcare workers, and influencing factors including exposure histories, the use of protective equipment in different risk conditions and areas, perceptions, emotions, satisfactions and educations were described and analyzed. RESULTS: Healthcare workers got infected clustered mostly in the physical examination center. When performing general operations on confirmed or suspected patients, the use of protective equipment including the effectiveness of masks (p < 0.001), gloves (p < 0.001); and the use of gloves (p < 0.001), suits (p < 0.001), gowns (p < 0.001), shoe covers (p < 0.001), and hats (p < 0.001) were protective factors. The use of protective equipment was a protective factor in most cases. Negative emotions and dissatisfaction to the hospital response were associated with the increased risk of infection. CONCLUSIONS: The use of protective equipment, emotions and satisfactions to hospital responses are key COVID-19-infected factors. The awareness, the supply and the use of protective equipment, the layout of departments and other environmental and management factors should be strictly equipped. In addition, hospitals should also pay attention to emotions and satisfaction of healthcare workers.

Lake, E. A., et al. (2021). "Knowledge, attitude and practice towards COVID-19 among health professionals in Ethiopia: A systematic review and meta-analysis." PLoS One **16**(2): e0247204.

BACKGROUND: The World Health Organization (WHO) declared coronavirus disease 2019 (COVID-19) a global pandemic on 11th March, 2020. In Ethiopia, more than 90,490 and 1,300 confirmed cases and deaths were reported by the Federal Ministry of Health at the time of writing up this project. As health care providers are frontline workers managing the COVID-19 pandemic, this systematic review and meta-analysis aimed to assess the pooled level of knowledge, attitude, and practice towards COVID-19 among health professionals in Ethiopia. METHODS: PubMed, Google Scholar, Excerpta Medica database (EMBASE), Cochrane Library, Web of Science, and African Journal of Online (AJOL) were searched. The data were extracted using Microsoft Excel and analyzed using STATA version 14. Publication bias was checked by funnel plot and more objectively through Egger's regression test, with P < 0.05 considered to indicate potential publication bias. The heterogeneity of studies was checked using I2 statistics. Pooled analysis was conducted using a weighted inverse variance random-effects model. Subgroup analysis was done related to geographic region and time. A leave-one-out sensitivity analysis was also employed. RESULT: A total of 11 studies with 3,843 study participants for knowledge, eight studies with 2,842 study participants for attitude and 10 studies with 3, 435 study participants for practice were used to estimate the pooled level of good knowledge, positive attitude and poor practice among health professionals. The overall estimated good level of knowledge, positive attitude and poor practice towards COVID-19 was found to be 79.4% (95% CI: 73.5%-85.2%; I2 = 96%), 73.7% (95%CI: 63.09%-84.4%; I2 = 98.3%) and 40.3% (95%CI: 31.1%-49.6%; I2 = 97.1%) respectively. CONCLUSION: Study findings showed that there were significant gaps in COVID-19 related knowledge, attitude and practice with respect to World Health Organization recommendations on COVID-19 management and personal protection practices. This study therefore recommends that institutions provide with immediate effect accurate and up-to-date information on COVID-19 and training that encourages improved knowledge, attitude and practice to mitigate this pandemic.

Lakhani, A., et al. (2020). "Corona Virus (COVID-19) and its Impact on Health Care Workers." J Assoc Physicians India **68**(9): 66-69.

Pandemics always come up with various life-threatening issues. COVID-19 outbreak came up with the same issues along with certain other problems involving public, administrative and healthcare sector concerns. It resembled the SARS outbreak but posed such challenges against the world that are uneasy to handle. The disease which started from Wuhan, China has now affected almost every country in a ruthless manner. Healthcare workers are working day and night just to protect the citizens despite being at high-risk exposure and they are being aimed by the virus due to shortage of Personal Protection Equipment kits. Not only this, but they are being brutally harassed by the patients themselves. Social, economic, psychiatric and many other factors are responsible for deteriorating the health of these frontline healthcare workers who are now being allegedly regarded as "Healthcare Warriors".

Lam, D. S. C., et al. (2020). "COVID-19: Special Precautions in Ophthalmic Practice and FAQs on Personal Protection and Mask Selection." Asia Pac J Ophthalmol (Phila) **9**(2): 67-77.

The Coronavirus Disease 2019 (COVID-19), caused by severe acute respiratory coronavirus-2, was first reported in December 2019. The World Health Organization declared COVID-19 a pandemic on March 11, 2020 and as of April 17, 2020, 210 countries are affected with >2,000,000 infected and 140,000 deaths. The estimated case fatality rate is around 6.7%. We need to step up our infection control measures immediately or else it may be too late to contain or control the spread of COVID-19. In case of local outbreaks, the risk of infection to healthcare workers and patients is high. Ophthalmic practice carries some unique risks and therefore high vigilance and special precautions are needed. We share our protocols and experiences in the prevention of infection in the current COVID-19 outbreak and the previous severe acute respiratory syndrome epidemic in Hong Kong. We also endeavor to answer the key frequently asked questions in areas of the coronaviruses, COVID-19, disease transmission, personal protection, mask selection, and special measures in ophthalmic practices. COVID-19 is highly infectious and could be life-threatening. Using our protocol and measures, we have achieved zero infection in our ophthalmic practices in Hong Kong and China. Preventing spread of COVID-19 is possible and achievable.

Lam, S. C., et al. (2020). "Perceived Risk and Protection From Infection and Depressive Symptoms Among Healthcare Workers in Mainland China and Hong Kong During COVID-19." Front Psychiatry **11**: 686.

Psychological health among healthcare workers (HCWs) has become a major concern since the COVID-19 outbreak. HCWs perceived risks of contracting COVID-19, in relation to depression were investigated. It was hypothesized that perceived high risk of contracting COVID-19 (close contact with cases, inadequate provision of personal protective equipment, insufficient infection control training, and presence of symptoms) would be significant predictors of depression. Our cross-sectional survey was completed by HCWs across three regions (Hubei, Guangdong, Hong Kong) between March 9 to April 9 2020 using convenience sampling. Depression was assessed using the 9-item Patient Health Questionnaire (PHQ-9). Prevalence of depression was 50.4% (95% CI: 44.5-56.2), 15.1% (10.1-21.9) and 12.9% (10.3-16.2) for HCWs in Hong Kong, Hubei and Guangdong, respectively. The strongest significant risk factors for depression, after adjustment, were HCWs who reported the greatest extent of feeling susceptible to contracting COVID-19 and those who reported the greatest difficulty obtaining face masks. HCWs whose family/peers greatly encouraged face mask use had lower prevalence of depression. Access to adequate supplies of personal protective equipment is essential for the psychological health of HCWs working in stressful environments, through potentially easing their perceptions of vulnerability to COVID-19.

LaMontagne, A. D., et al. (2020). "Strengthening respiratory protection from SARS-CoV-2 in certain general practice contexts." Aust J Gen Pract **49**.

We recommend a precautionary approach to respiratory protection for healthcare workers potentially exposed to SARS-CoV-2 until the efficacy of surgical masks can be proven.

Lan, F. Y., et al. (2020). "Effects of universal masking on Massachusetts healthcare workers' COVID-19 incidence." Occup Med (Lond) **70**(8): 606-609.

BACKGROUND: Healthcare workers (HCWs) and other essential workers are at risk of occupational infection during the COVID-19 pandemic. Several infection control strategies have been implemented. Evidence shows that universal masking can mitigate COVID-19 infection, though existing research is limited by secular trend bias. AIMS: To investigate the effect of hospital universal masking on COVID-19 incidence among HCWs compared to the general population. METHODS: We compared the 7-day average incidence rates between a Massachusetts (USA) healthcare system and Massachusetts residents statewide. The study period was from 17 March (the date of first incident case in the healthcare system) to 6 May (the date Massachusetts implemented public masking). The healthcare system implemented universal masking on 26 March, we allotted a 5-day lag for effect onset and peak COVID-19 incidence in Massachusetts was 20 April. Thus, we categorized 17-31 March as the pre-intervention phase, 1-20 April the intervention phase and 21 April to 6 May the epidemic decline phase. Temporal incidence trends (i.e. 7-day average slopes) were compared using standardized coefficients from linear regression models. RESULTS: The standardized coefficients were similar between the healthcare system and the state in both the pre-intervention and epidemic decline phases. During the intervention phase, the healthcare system's epidemic slope became negative (standardized β: -0.68, 95% CI: -1.06 to -0.31), while Massachusetts' slope remained positive (standardized β: 0.99, 95% CI: 0.94 to 1.05). CONCLUSIONS: Universal masking was associated with a decreasing COVID-19 incidence trend among HCWs, while the infection rate continued to rise in the surrounding community.

Lan, F. Y., et al. (2020). "COVID-19 symptoms predictive of healthcare workers' SARS-CoV-2 PCR results." PLoS One **15**(6): e0235460.

BACKGROUND: Coronavirus 2019 disease (COVID-19) is caused by the virus SARS-CoV-2, transmissible both person-to-person and from contaminated surfaces. Early COVID-19 detection among healthcare workers (HCWs) is crucial for protecting patients and the healthcare workforce. Because of limited testing capacity, symptom-based screening may prioritize testing and increase diagnostic accuracy. METHODS AND FINDINGS: We performed a retrospective study of HCWs undergoing both COVID-19 telephonic symptom screening and nasopharyngeal SARS-CoV-2 assays during the period, March 9-April 15, 2020. HCWs with negative assays but progressive symptoms were re-tested for SARS-CoV-2. Among 592 HCWs tested, 83 (14%) had an initial positive SARS-CoV-2 assay. Fifty-nine of 61 HCWs (97%) who were asymptomatic or reported only sore throat/nasal congestion had negative SARS-CoV-2 assays (P = 0.006). HCWs reporting three or more symptoms had an increased multivariate-adjusted odds of having positive assays, 1.95 (95% CI: 1.10-3.64), which increased to 2.61 (95% CI: 1.50-4.45) for six or more symptoms. The multivariate-adjusted odds of a positive assay were also increased for HCWs reporting fever and a measured temperature ≥ 37.5°C (3.49 (95% CI: 1.95-6.21)), and those with myalgias (1.83 (95% CI: 1.04-3.23)). Anosmia/ageusia (i.e. loss of smell/loss of taste) was reported less frequently (16%) than other symptoms by HCWs with positive assays, but was associated with more than a seven-fold multivariate-adjusted odds of a positive test: OR = 7.21 (95% CI: 2.95-17.67). Of 509 HCWs with initial negative SARS-CoV-2 assays, nine had symptom progression and positive re-tests, yielding an estimated negative predictive value of 98.2% (95% CI: 96.8-99.0%) for the exclusion of clinically relevant COVID-19. CONCLUSIONS: Symptom and temperature reports are useful screening tools for predicting SARS-CoV-2 assay results in HCWs. Anosmia/ageusia, fever, and myalgia were the strongest independent predictors of positive assays. The absence of symptoms or symptoms limited to nasal congestion/sore throat were associated with negative assays.

Lan, K. P., et al. (2021). "Experience of Resources Management on COVID-19 Epidemic at a Tertiary Medical Center in Northern Taiwan." J Chin Med Assoc.

BACKGROUND: Coronavirus disease (COVID-19) is a respiratory infection caused by severe acute respiratory syndrome coronavirus 2 (SARS-CoV-2) that causes infection symptoms including fever, cough, respiratory and gastrointestinal symptoms, and even loss of smell/taste, so far had caused 489,000 people to be infected with 32,000 deaths. This article aims to develop some strategies in dealing with COVID-19 epidemic to prevent nosocomial infection and ensure the safety of health care workforce and employees. METHODS: This is a prospectively registered and retrospective descriptive study investigating the clinical characteristics, results of diagnostic tests, and patients' disposition from February 1, 2020, to April 30, 2020, at a tertiary medical center in Northern Taiwan. RESULTS: There is no nosocomial spreading of SARS-CoV-2 in our facility. The following strategies were followed: information transparency, epidemic prevention resources planning by authorities, multidisciplinary cooperation, informative technologies, immigration quarantine policies, travel restrictions, management of diversion/subdivision, self-health monitoring, social distancing, screening of travel, occupation, contact, and cluster (TOCC) history, traffic control bundling (TCB), training of using personal protective equipment, real-name visiting management, and employee care. The patients' basic characteristics and diagnostic results were gathered. Of the 3,832 cases, about 25.9% had travel history. Most of them were traveling to Asia (419 persons/time, 10.9%) and from China (256 persons/time, 6.7%). Meanwhile, health care personnel accounted for 316 persons/time (8.3%) and cleaning personnel, 6 persons/time (0.16%). The 36 cases who care or contact with confirmed cases have negative results from the COVID-19 test. The most frequent symptoms were fever and upper respiratory infection followed by gastrointestinal symptoms. CONCLUSION: The above strategies were followed. Patients were stratified based on the risk of TOCC history assessment to ensure safety of health care personnel and patients' appropriate and timely medical services.

Lapolla, P., et al. (2020). "First-in-man craniectomy and asportation of solitary cerebellar metastasis in COVID-19 patient: A case report." Int J Surg Case Rep **77**: 753-758.

INTRODUCTION: Severe acute respiratory syndrome coronavirus 2 (SARS-CoV-2) outbreak has an impact on the delivery of neurosurgical care, and it is changing the perioperative practice worldwide. We present the first case in the literature of craniectomy procedure and asportation of a solitary cerebellar metastasis of the oesophagus squamous carcinoma in a 77 years old woman COVID-19 positive. In these particular circumstances, we show that adequate healthcare resources and risk assessments are essential in the management of COVID-19 patients referred to emergency surgery. PRESENTATION OF CASE: The case here presented was treated in 2019 for squamous carcinoma of the oesophagus. In April 2020, she presented a deterioration of her clinical picture consisting of dysphagia, abdominal pain, hyposthenia and ataxia. A Head CT scan was performed, which showed the presence of a solitary cerebellar metastasis. Her associated SARS-CoV-2 positivity status represented the principal clinical concern throughout her hospitalisation. DISCUSSION: The patient underwent a suboccipital craniectomy procedure with metastasis asportation. She tested positive for SARS-CoV-2 in the pre- and post-operative phases, but she was not admitted to the intensive care unit because she did not present any respiratory complications. Her vital parameters and inflammation indexes fell within the reference ranges, and she was kept in isolation for 16 days in our neurosurgical unit following strict COVID-19 measures. She was asymptomatic and not treated for any of the specific and non-specific symptoms of COVID-19. CONCLUSION: This is the first case reported of solitary cerebellar metastasis of oesophagus carcinoma operated on a COVID-19 positive patient. It shows that asymptomatic COVID-19 positive patients can undergo major emergency surgeries without the risk of infecting the operating team if adequate Personal Protection Equipment (PPE) is used. The patient remained asymptomatic and did not develop the disease's active phase despite undergoing a stressful event such as a major emergency neurosurgical procedure. In the current crisis, a prophylactic COVID-19 screening test can identify asymptomatic patients undergoing major emergency surgery and adequate resource planning and Personal Protective Equipment (PPE) for healthcare workers can minimise the effect of the COVID-19 pandemic.

Lastrucci, V., et al. (2020). "SARS-CoV-2 Seroprevalence Survey in People Involved in Different Essential Activities during the General Lock-Down Phase in the Province of Prato (Tuscany, Italy)." Vaccines (Basel) **8**(4).

Serosurveys may help to assess the transmission dynamics in high-risk groups. The aim of the study was to assess the SARS-CoV-2 antibody seroprevalence in people who had performed essential activities during the lock-down period in the Province of Prato (Italy), and to evaluate the risk of exposure to SARS-CoV-2 according to the type of service. All the workers and volunteers of the Civil Protection, employees of the municipalities, and all the staff of the Health Authority of the Province of Prato were invited to be tested with a rapid serological test. A total of 4656 participants were tested. SARS-CoV-2 antibodies were found in 138 (2.96%) cases. The seroprevalence in health care workers, in participants involved in essential support services and in those who worked from home were 4.1%, 1.4% and 1.0%, respectively. Health care workers experienced higher odds of seropositivity (OR 4.38, 95%CI 2.19-10.41) than participants who were assigned to work-from-home; no significant seropositivity differences were observed between support services and work-from-home groups. A low circulation of SARS-CoV-2 was observed among participants performing different essential activities. Findings highlighted the risk of in-hospital transmission in healthcare workers and that community support services may increase the risk of seropositivity to a limited extent in low incidence areas.

Lauer, J. K., et al. (2020). "PPE during a pandemic: The experience of obtaining PPE and lessons learned from a department of obstetrics and gynecology in New York city." Semin Perinatol **44**(6): 151293.

The COVID-19 Pandemic is an ongoing crisis that has strained hospitals and health systems around the globe. The provision of personal protective equipment (PPE) for frontline healthcare workers is of utmost importance in sustaining an effective response to this crisis. New York City has experienced one of the most devastating outbreaks of the SARS-CoV-2 virus. In this article we report the experience of the Department of Obstetrics and Gynecology at Columbia University in New York City in managing the supply of PPE for providers and staff during the height of the outbreak. We describe the types of equipment used and aspects of PPE regulation and certification. We also describe our practices in extended use and reuse of PPE in light of the current understanding of the virus characteristics and modes of transmission.

Lavinsky, J., et al. (2020). "An update on COVID-19 for the otorhinolaryngologist - a Brazilian Association of Otolaryngology and Cervicofacial Surgery (ABORL-CCF) Position Statement." Braz J Otorhinolaryngol **86**(3): 273-280.

INTRODUCTION: We are facing a pandemic with a great impact worldwide, as a result of the rapid spread of the novel coronavirus (COVID-19). The medical community is still getting to know behavior of this virus and the consequences from a population point of view. All this knowledge is extremely dynamic, so some behaviors are still not well established. Otorhinolaryngologists have a central role in the management of this situation, in which they must assess the patient, avoid contamination to and by health professionals and other patients. Thus, the recommendations of the Brazilian Association of Otorhinolaryngology and Cervical-Facial Surgery (ABORL-CCF) have the main objective of reducing the spread of the new coronavirus during otorhinolaryngological care and assisting in the management of these patients. METHODS: Review of the main recommendations of national and international scientific societies, decisions by government agencies and class councils. The topics will be related to the general aspects of COVID-19, personal protective equipment, care in patient assistance, endoscopic exam routines and the management of sinonasal, otological and pediatric evaluations related to COVID-19. RESULTS: The use of personal protective equipment is considered crucial in routine ENT care. We recommend postponing appointments, exams and elective surgeries to reduce the spread of COVID-19. Similarly, we recommend changing routines in several areas of otolaryngology. Additionally, guidance is provided on the use of telemedicine resources during the pandemic period. CONCLUSIONS: We are still at the beginning of the COVID-19 pandemic and scientific evidence is still scarce and incomplete, so these ABORL-CCF recommendations for otorhinolaryngologists may be updated based on new knowledge and the pattern of the new coronavirus spread.

Publisher: Abstract available from the publisher.

por

Lázaro-Pérez, C., et al. (2020). "Anxiety About the Risk of Death of Their Patients in Health Professionals in Spain: Analysis at the Peak of the COVID-19 Pandemic." Int J Environ Res Public Health **17**(16).

The COVID-19 health crisis has had a global effect, but the consequences in the different countries affected have been very different. In Spain, in a short period of time, health professionals went from a situation of stability to living with a working environment characterized by overcrowded hospitals, lack of individual protection equipment, non-existent or contradictory work protocols, as well as an unknown increase in mortality. Although in their professional activity health workers are closely linked to death processes, in recent months, working conditions and health emergencies have drawn an unheard of working scenario, with the stress and anxiety they may suffer when faced with the death of their patients. The present quantitative research was carried out in different hospitals in Spain on health professionals during the month of April 2020. Through the subscale of anxiety in the face of the death of others, developed by Collett-Lester, it has been verified that health professionals have had to develop their work in a context of precariousness, putting at risk both their individual and collective health, notably increasing anxiety in the face of the death of their patients. The predictive variables of this anxiety have been the absence of individual protection equipment, as well as high levels in the burnout subscales of emotional exhaustion and depersonalization.

Ledda, C., et al. (2021). "SARS-CoV-2 Seroprevalence Post-First Wave among Primary Care Physicians in Catania (Italy)." Trop Med Infect Dis **6**(1).

Family physicians or pediatricians and general practitioners (GPs) work in non-hospital settings. GPs usually visit many patients, frequently at their homes, with low potential, if any, to control the work setting. Particularly during the initial phases of the COVID-19 outbreak, they were not informed about the occurrence of SARS-CoV-2-infected patients, with inadequate information regarding the risk, a lack of suitable protective measures and, in some cases, deficient or poor accessibility to personal protective equipment (PPE). During the first wave of COVID-19, primary care physicians were on the front line and isolated the first cases of the disease. The present study aims to estimate the seroprevalence of SARS-CoV-2 in a cohort of 133 GPs working in Catania (Italy) after the first wave of COVID-19. Serological analysis revealed a low seroprevalence (3%) among GPs. The low seroprevalence highlighted in the results can be attributed to correct management of patients by GPs in the first wave. It is now hoped that mass vaccination, combined with appropriate behavior and use of PPE, can help further reduce the risk of COVID-19 disease.

Lee, E. B., et al. (2021). "What Is New in Occupational Allergic Contact Dermatitis in the Year of the COVID Pandemic?" Curr Allergy Asthma Rep **21**(4): 26.

PURPOSE OF REVIEW: This article aims to summarize some recent trends in occupational allergic contact dermatitis (ACD), including dermatitis related to pandemic-level personal protective equipment in healthcare workers, hazards patients may experience when working from home, and occupational perspectives on the recent American Contact Dermatitis Society (ACDS) allergens of the year and ACDS Core Allergen Series updates. RECENT FINDINGS: Recent ACDS Allergens of the Year may be particularly relevant to healthcare workers, including isobornyl acrylate, which is present in glucose sensors and propylene glycol present in hand cleansers and disinfectants. Lavender, limonene, and linalool, all of which are new additions to the ACDS Core Allergen Series, have been reported as causes for occupational ACD in massage therapists and aromatherapists. Isothiazolinone allergy continues to rise in both consumer and occupational settings. Finally, the COVID-19 pandemic has resulted in a wave of occupational ACD in healthcare workers to personal protective equipment, and revealed new potential allergens for individuals working from home. Occupational allergic contact dermatitis continues to exert a significant occupational disease burden. Remaining aware of the current trends in allergens may allow for earlier recognition, diagnosis, and treatment, subsequently helping our patients to work in healthier and safer environments.

Lee, E. S., et al. (2020). "Perceived stress and associated factors among healthcare workers in a primary healthcare setting: the Psychological Readiness and Occupational Training Enhancement during COVID-19 Time (PROTECT) study." Singapore Med J.

INTRODUCTION: Disease outbreaks such as the coronavirus disease 2019 (COVID-19) pandemic significantly heighten the psychological stress of healthcare workers (HCWs). The objective of this study was to understand the factors contributing to the perceived stress levels of HCWs in a public primary care setting during the COVID-19 pandemic, including their training, protection and support (TPS), job stress (JS), and perceived stigma and interpersonal avoidance. METHODS: This cross-sectional study using an electronic self-administered questionnaire was conducted at the National Healthcare Group Polyclinics in March 2020. Data was collected anonymously. Analysis was performed using regression modelling. RESULTS: The response rate was 69.7% (n = 1,040). The mean perceived stress level of HCWs in various departments ranged from 17.2 to 20.3. Respondents who reported higher perceived stress were those who made alternative living arrangements, were more affected by the current pandemic, reported higher JS and were Muslims. Respondents who reported lower perceived stress were those who had been through the severe acute respiratory syndrome epidemic in 2003 and H1N1 pandemic in 2009 as HCWs, and those who had higher confidence in the organisation's TPS. CONCLUSION: All HCWs, regardless of scope of work, were similarly stressed by the current pandemic compared to the general population. Improving the confidence of HCWs in their training, protection and the support of personal protective equipment, and retaining experienced HCWs who can provide advice and emotional support to younger colleagues are important. Adequate psychological support for HCWs in the pandemic can be transformed into reserves of psychological resilience for future disease outbreaks.

Lee, H. C. and C. L. Goh (2021). "'Occupational dermatoses from Personal Protective Equipment during the COVID-19 pandemic in the tropics - A Review'." J Eur Acad Dermatol Venereol **35**(3): 589-596.

The COVID-19 pandemic has enveloped the world and there has been a high incidence of occupational dermatoses related to Personal Protective Equipment (PPE) amongst healthcare workers (HCWs) during this period. Prevention and management of these conditions will not only improve staff morale and quality of life, but will also minimize the risk of breaching PPE protocol due to such symptoms. The tropical climate in Singapore predisposes HCWs to more skin damage and pruritus due to intense heat, high humidity and sun exposure. The effects of friction, occlusion, hyperhidrosis and overheating on the skin in the tropics should not be neglected. Preventive measures can be taken based on our recommendations, and the working environment can be made more conducive for frontline HCWs. We review the literature and discuss various preventive and management strategies for these occupational skin diseases for our frontline HCWs, especially those working in less controlled working environments beyond the hospital in Singapore. Shorter shifts and frequent breaks from PPE are recommended. Duration of continuous PPE-usage should not exceed 6 h, with breaks in non-contaminated areas every 2-3 h to hydrate and mitigate the risk of skin reactions. Other strategies, such as teledermatology, should be considered so that consultations can remain accessible, while ensuring the safety and well-being of our clinical staff.

Lee, J., et al. (2020). "Heat Stress and Thermal Perception amongst Healthcare Workers during the COVID-19 Pandemic in India and Singapore." Int J Environ Res Public Health **17**(21).

The need for healthcare workers (HCWs) to wear personal protective equipment (PPE) during the coronavirus disease 2019 (COVID-19) pandemic heightens their risk of thermal stress. We assessed the knowledge, attitudes, and practices of HCWs from India and Singapore regarding PPE usage and heat stress when performing treatment and care activities. One hundred sixty-five HCWs from India (n = 110) and Singapore (n = 55) participated in a survey. Thirty-seven HCWs from Singapore provided thermal comfort ratings before and after ice slurry ingestion. Differences in responses between India and Singapore HCWs were compared. A p-value cut-off of 0.05 depicted statistical significance. Median wet-bulb globe temperature was higher in India (30.2 °C (interquartile range [IQR] 29.1-31.8 °C)) than in Singapore (22.0 °C (IQR 18.8-24.8 °C)) (p < 0.001). Respondents from both countries reported thirst (n = 144, 87%), excessive sweating (n = 145, 88%), exhaustion (n = 128, 78%), and desire to go to comfort zones (n = 136, 84%). In Singapore, reports of air-conditioning at worksites (n = 34, 62%), dedicated rest area availability (n = 55, 100%), and PPE removal during breaks (n = 54, 98.2%) were higher than in India (n = 27, 25%; n = 46, 42%; and n = 66, 60%, respectively) (p < 0.001). Median thermal comfort rating improved from 2 (IQR 1-2) to 0 (IQR 0-1) after ice slurry ingestion in Singapore (p < 0.001). HCWs are cognizant of the effects of heat stress but might not adopt best practices due to various constraints. Thermal stress management is better in Singapore than in India. Ice slurry ingestion is shown to be practical and effective in promoting thermal comfort. Adverse effects of heat stress on productivity and judgment of HCWs warrant further investigation.

Lee, K. Y., et al. (2020). "Consensus statement on coronary intervention during the coronavirus disease 19 pandemic: from the Korean Society of Interventional Cardiology." Korean J Intern Med **35**(4): 749-757.

Coronavirus disease 2019 (COVID-19) is a highly contagious disease caused by the novel virus severe acute respiratory syndrome coronavirus 2. The first case developed in December, 2019 in Wuhan, China; several months later, COVID-19 has become pandemic, and there is no end in sight. This disaster is also causing serious health problems in the area of cardiovascular intervention. In response, the Korean Society of Interventional Cardiology formed a COVID-19 task force to develop practice guidelines. This special article introduces clinical practice guidelines to prevent secondary transmission of COVID-19 within facilities; the guidelines were developed to protect patients and healthcare workers from this highly contagious virus. We hope these guidelines help healthcare workers and cardiovascular disease patients around the world cope with the COVID-19 pandemic.

Lee, K. Y., et al. (2020). "Consensus Statement on Coronary Intervention during the Coronavirus Disease 2019 (COVID-19) Pandemic: from the Korean Society of Interventional Cardiology (KSIC)." Korean Circ J **50**(11): 974-983.

Coronavirus disease 2019 (COVID-19) is a highly contagious disease caused by the novel virus severe acute respiratory syndrome coronavirus-2. The first case developed in December, 2019 in Wuhan, China; several months later, COVID-19 has become pandemic, and there is no end in sight. This disaster is also causing serious health problems in the area of cardiovascular intervention. In response, the Korean Society of Interventional Cardiology formed a COVID-19 task force to develop practice guidelines. This special article introduces clinical practice guidelines to prevent secondary transmission of COVID-19 within facilities; the guidelines were developed to protect patients and healthcare workers from this highly contagious virus. We hope these guidelines help healthcare workers and cardiovascular disease patients around the world cope with the COVID-19 pandemic.

Lee, S., et al. (2020). "Asymptomatic carriage and transmission of SARS-CoV-2: What do we know?" Can J Anaesth **67**(10): 1424-1430.

PURPOSE: Risk to healthcare workers treating asymptomatic patients infected with severe acute respiratory syndrome coronavirus 2 (SARS-CoV-2) in the operating room depends on multiple factors. This review examines the evidence for asymptomatic or pre-symptomatic carriage of SARS-CoV-2, the risk of transmission from asymptomatic patients, and the specific risks associated with aerosol-generating procedures. Protective measures, such as minimization of aerosols and use of personal protective equipment in the setting of treating asymptomatic patients, are also reviewed. SOURCE: We examined the published literature as well as Societal guidelines. PRINCIPAL FINDINGS: There is evidence that a proportion of those infected with SARS-CoV-2 have detectable viral loads prior to exhibiting symptoms, or without ever developing symptoms. The degree of risk of transmission from asymptomatic patients to healthcare providers will depend on the prevalence of disease in the population, which is difficult to assess without widespread population screening. Aerosol-generating procedures increase the odds of viral transmission from infected symptomatic patients to healthcare providers, but transmission from asymptomatic patients has not been reported. Techniques to minimize aerosolization and appropriate personal protective equipment may help reduce the risk to healthcare workers in the operating room. Some societal guidelines recommend the use of airborne precautions during aerosol-generating procedures on asymptomatic patients during the coronavirus disease pandemic, although evidence supporting this practice is limited. CONCLUSION: Viral transmission from patients exhibiting no symptoms in the operating room is plausible and efforts to reduce risk to healthcare providers include reducing aerosolization and wearing appropriate personal protective equipment, the feasibility of which will vary based on geographic risk and equipment availability.

Publisher: Abstract available from the publisher.

fre

Lee, Y., et al. (2020). "Impact of hospital lockdown secondary to COVID-19 and past pandemics on surgical practice: A living rapid systematic review." Am J Surg.

BACKGROUND: The COVID-19 pandemic has disrupted surgical practice worldwide. There is widespread concern for surgeon and provider safety, and the implications of hospital lockdown on patient care during epidemics. METHODS: Medline, EMBASE, CENTRAL, and PubMed were systematically searched from database inception to July 1, 2020 and ongoing monthly surveillance will be conducted. We included studies that assessed postoperative patient outcomes or protection measures for surgical personnel during epidemics. RESULTS: We included 61 studies relevant to the COVID-19 pandemic and past epidemics. Lockdown measures were noted globally including cancellation of elective surgeries and outpatient clinics. The pooled postoperative complication rate during epidemics was 21.0% among 2095 surgeries. 31 studies followed the health of surgical workers with the majority noting no adverse outcomes with proper safety measures. CONCLUSIONS: This review highlights postoperative patient outcomes during worldwide epidemics including the COVID-19 pandemic and identifies specific safety measures to minimize infection of healthcare workers.

Lee, Y. S., et al. (2021). "Women's Health in Times of Emergency: We Must Take Action." J Womens Health (Larchmt) **30**(3): 289-292.

Women have historically faced gendered patterns of disadvantage during times of emergency. Evidence demonstrates differences in gendered exposures and inequities during acute crises such as natural disasters and pandemics, including Covid-19, and longer-term emergencies such as climate change. These patterns, without intervention, may be perpetuated in future crises. Threats to women's health in times of emergency can arise from restricted access to health care, economic disadvantages, and harmful social norms. During crises, women face additional barriers to accessing maternal, contraceptive, and abortion care, likely exacerbating existing inequities in reproductive health outcomes. Gendered inequalities in financial and economic stability can become even more stark. Globally, women perform the majority of health care and unpaid caregiving work, but face barriers to affording costs of living and obtaining health insurance due to over-representation in low-wage jobs. Finally, gendered expectations of social roles contribute to increased vulnerabilities, such as displacement and poverty. Violence against women rises in times of emergency and pathways to escaping trauma can be limited. In addition to directly addressing women's unique barriers and providing support in times of emergency through bolstering health care access, economic, and social support systems, thoughtful solutions such as trauma-informed care, increasing the number of women in leadership roles, educational initiatives, and advocacy from health professionals are needed to protect and advance women's health.

Lee-Archer, P., et al. (2021). "A comparison of anesthetic protective barriers for the management of COVID-19 pediatric patients." Paediatr Anaesth **31**(3): 323-329.

BACKGROUND: Barrier techniques, such as plastic sheets or intubation boxes, are purported to offer additional protection for healthcare workers. AIMS: To assess the functionality, perceived safety, droplet protection, and aerosol protection of several barrier techniques. METHODS: Firstly, a simulation study with 12 different laryngoscopists was conducted to assess the time taken to perform an intubation (via direct laryngoscopy, via video laryngoscopy, and via a bougie) with four different barrier techniques (personal protective equipment only, a plastic sheet, a tented plastic sheet, and an intubation box). Secondly, a cough at the time of intubation was simulated using ultraviolet dye to assess the spread of droplets; and thirdly, smoke was used to assess the spread of aerosols. RESULTS: Intubation time using the box was noninferior to using no barrier. Based on subjective ratings by the laryngoscopists, the most functional technique was no barrier followed by the intubation box, then the tented sheet, and then the plastic sheet. The technique that conferred the highest feeling of safety to the laryngoscopists was the intubation box, followed by the tented sheet, then no barrier, and then the plastic sheet. All the barriers prevented the ultraviolet dye contaminating the head and torso of the laryngoscopist. Smoke remained within the intubation box if plastics sheets were used to cover the openings and suction was ineffective at clearing it. With no barrier in place, smoke was effectively cleared away from the patient in a theater with laminar flow but tended to spread up toward the laryngoscopist in a room without laminar flow. CONCLUSIONS: A well-designed intubation box is an effective barrier against droplets and is noninferior to no barrier in relation to intubation time. However, a box interferes with laminar flow in theaters with formal ventilation systems and may result in accumulation of aerosols if it is completely enclosed.

Legler, S., et al. (2021). "Mixed Methods Evaluation of an Intra-Hospital Telemedicine Program for Patients Admitted with COVID-19." J Med Internet Res.

BACKGROUND: Increasing incidence of coronavirus 2019 (COVID-19) infection has challenged healthcare systems to increase capacity while needing to conserve personal protective equipment (PPE) supplies and minimize nosocomial spread. Telemedicine shows promise to address these challenges but lacks comprehensive evaluation in the inpatient environment. OBJECTIVE: To evaluate an intra-hospital telemedicine program (virtual care), along with its impact on exposure risk and communication. METHODS: We conducted a natural experiment of virtual care on patients admitted for COVID-19. The primary exposure variable was documented use of virtual care. Patient characteristics, PPE use rates and their association with virtual care use were assessed. In parallel, we conducted surveys with patients and clinicians to capture satisfaction with virtual care along the domains of communication, medical treatment, and exposure risk. RESULTS: Of 137 total patients in our primary analysis, 43 patients used virtual care. In total, there were 82 inpatient days of use and 401 inpatient days without use. Hospital utilization and illness severity was similar in patients who opted-in vs opted-out. Virtual care was associated with a significant reduction in PPE use and physical exam rate. Surveys of 41 patients and clinicians showed high rates of recommendation for further use, and subjective improvements in communication. However, providers and patients expressed limitations in usability, medical assessment and empathetic communication. CONCLUSIONS: In this pilot natural experiment, only a subset of patients used inpatient virtual care. When used, virtual care was associated with reductions in PPE use, reductions in exposure risk, and patient and provider satisfaction.

Leira-Sanmartín, M., et al. (2021). "Psychological Impact of COVID-19 Pandemic and Related Variables: A Cross-Sectional Study in a Sample of Workers in a Spanish Tertiary Hospital." Int J Environ Res Public Health **18**(7).

Introduction: We intend to objectify the psychological impact of the COVID-19 pandemic on the workers of a tertiary hospital. Methods: All the workers were invited to an online survey. In total, 657 workers were recruited, including 536 healthcare workers (HCWs) and 121 non-healthcare workers (nHCWs). General Health Questionnaire-12 items (GHQ-12) was used as a screening tool. Sociodemographic data, working environmental conditions, and health behaviors were also analyzed. Results: inadequate sleep, poor nutritional and social interaction habits, misuse of psychotropics, female gender, COVID-19 clinical diagnosis, and losing a relative by COVID-19 were variables associated with higher probability of GHQ-12 positive screening. Significant differences between "frontline workers" and the rest were not found, nor was higher the probability of psychological distress in healthcare workers compared to non-healthcare workers. After 3 months from the peak of the pandemic, 63.6% of participants screening positive in GHQ-12 reported remaining "the same or worse." Limitations: Causal inferences cannot be established. Retrieval and selection biases must be considered as the survey was not conducted during the peak of the outbreak. Conclusions: psychological impact of COVID-19 has been broad, heavy, and persistent in our institution. Proper assessment and treatment must be offered to all hospital workers.

Leite, K. F. S., et al. (2021). "Máscaras do Bem: An analysis of face-mask performance from a volunteer mask-making initiative in Ribeirão Preto, Brazil." Public Health Pract (Oxf) **2**: 100094.

OBJECTIVES: This study examines the response of a group of volunteers in Ribeirão Preto, Brazil, as the city faced an unprecedented demand for face masks during the onset of the COVID-19 crisis in 2020. The performance of artisanal-produced masks was compared with industry equivalents. STUDY DESIGN: Case report with comparative testing. METHODS: A comparison was made between two parallel projects that produced single-use masks for healthcare workers and reusable masks for the community. Mask samples were tested for filtration efficiency (FE) and breathability (pressure drop). RESULTS: Results for FE averaged 40-60% for healthcare masks and 10% for community masks; both types of masks were tested for particle sizes of 0.3 μm. CONCLUSIONS: While performance was inferior to standard comparators, the masks investigated in this study afforded a level of protection in the absence of alternatives, especially in non-aerosol generating contexts. The findings of this study are useful for communities with limited resources in other developing countries. In addition, insights can be gained from the experiences in Ribeirão Preto in terms of how to respond to future health emergencies.

Lembo, M., et al. (2021). "Thermal discomfort in healthcare workers during the COVID-19 pandemic." Med Lav **112**(2): 123-129.

INTRODUCTION: Due to the COVID-19 pandemic, healthcare workers are now required to use additional personal protective equipment (PPEs) to protect themselves against the virus. That led to an increased clothing insulation which is negatively affecting the perceived healthcare workers' thermal sensation. OBJECTIVES: While demonstrating through software simulations the potential level of thermal discomfort healthcare workers involved in the COVID-19 emergency can be subjected to, this work aims at identifying measures to improve thermal sensation perception and acceptable thermal conditions for medical personnel. METHODS: After having obtained the insulation values of individual clothing used by staff during COVID-19 emergency through the use of a thermal well-being evaluation software, the Fanger indexes (PMV - Predicted Mean Vote and PPD - Predicted Percentage of Dissatisfied) were calculated in order to estimate staff satisfaction to microclimatic conditions. RESULTS: The use of COVID-19 additional PPEs with an air temperature equal to 22 °C (normally considered optimal) brings the PMV index equal to 0.6, which corresponds to 11.8 % being unsatisfied (PPD) due to perceived heat. DISCUSSION: The use of additional protective devices significantly increases the clothing insulation level, facilitating the onset of conditions of thermal discomfort in the health workers. Workers engaged in the execution of nasopharyngeal swabs were most affected by the summer weather conditions and certainly represent the most critical category, for which it would be recommended to implement a higher turnover of service to reduce individual exposure time and consequent discomfort.

Lepelletier, D., et al. (2020). "What face mask for what use in the context of COVID-19 pandemic? The French guidelines." J Hosp Infect **105**(3): 414-418.

In the context of the COVID-19 pandemic, wearing a face mask has become usual and ubiquitous, in both hospitals and community. However, the general public is consuming surgical or filtering face piece (FFP) masks irrespective of their specificity, leading to global supply shortage for the most exposed persons, which are healthcare workers. This underlines the urgent need to clarify the indications of the different categories of mask, in order to rationalize their use. The study herein specifies the French position for the rational use of respiratory protective equipment for healthcare workers.

Leti Acciaro, A., et al. (2021). "Retrospective study in clinical governance and financing system impacts of the COVID-19 pandemic in the hand surgery and microsurgery HUB center." Musculoskelet Surg: 1-6.

INTRODUCTION: The authors presented a retrospective study in the surgical activity of the HUB center for Hand Surgery and Microsurgery in Emilia-Romagna comparing the data between March and April 2020, in the peak of Covid pandemic, with the same period in 2019. MATERIALS AND METHODS: During the two months period of March-April 2020 versus 2019 the authors analyzed the surgical procedures performed in elective and emergency surgery with hospitalization and Day or Outpatient surgery regime. Surgical treatments with no hospitalization were planned in the Day-Surgery Service. The financing system impacts were analyzed according to the Diagnosis Related Groups (DRG), the costs accounting method mostly used in European countries. RESULTS: An overall reduction of 68.5% was recorded in surgical procedures, with a more relevant reduction of 92.3% in elective surgery and a significantly less relevant reduction of 37.2% in urgent one. Replantation did not present a reduction in number of cases, while cutting lesions of tendons at the hand and fingers increased such as the bone and ligament injuries during domestic accidents. The negative impact in the financial system recorded a reduction of 32.5%. DISCUSSION: The epidemiology of hand trauma looks not only at the artisanal and industrial injuries, but also mostly at the accidents in daily life activities. The data of the study evidenced the significantly increase in the injuries occurring in the domestic environment. Elective surgery was canceled. The 86% of surgical procedures performed were urgent ones and the 72.8% of these were possible in Day and Outpatient surgery with significantly reduction in hospitalization. All procedures followed a rigid process for patient and healthcare workers with regard for personal protection and safety. Telemedicine was arranged in emergencies, and economic damage was analyzed also in the following rebound effect during summer period. CONCLUSIONS: The significantly less reduction recorded in urgent surgery vs the more relevant reduction in elective one showed how the hand injuries remained a major issue also during the lockdown. The data highlighted the relevant role of the organizational aspects of the surgical procedures and planning in hand trauma. Despite the financial impact of the elective surgery, the presence of a functional and skill Emergency Service and Day-Surgery Service resulted fundamental in the efficacy and efficiency of the patient management and in containment of economic damage. The telemedicine was significantly limited by liability and risk management issues.

Leung, K. K. Y., et al. (2021). "Airway management in children with COVID-19." Hong Kong Med J.

The novel coronavirus disease (COVID-19) may result in acute respiratory distress syndrome and respiratory failure, necessitating mechanical respiratory support. Healthcare professionals are exposed to a particularly high risk of contracting the virus while providing resuscitation and respiratory support, which may in turn result in grave consequences and even death. Although COVID-19 has been shown to cause milder disease in children, paediatricians and intensivists who provide care for children must be prepared to provide optimal respiratory support without putting themselves or other medical, nursing, and paramedical staff at undue risk. We propose an airway management approach that is especially relevant in the current COVID-19 pandemic and provides instructions for: (1) Elective intubation for respiratory failure; and (2) Emergency intubation during cardiopulmonary resuscitation. To minimise risk, intubation methods must be kept as straightforward as possible and should include the provision of appropriate personal protection and equipment to healthcare workers. We identify two key considerations: that bag-mask ventilation should be avoided if possible and that bacterial and viral filters should be placed in the respiratory circuit. Our novel approach provides a framework for airway management that could benefit paediatric critical care practitioners who provide care for any children with a novel viral illness, with a focus on infection prevention during high-risk airway management procedures.

Leung, M. S. T., et al. (2020). "COVID-19 and Oncology: Service transformation during pandemic." Cancer Med **9**(19): 7161-7171.

BACKGROUND: The COVID-19 pandemic has challenged healthcare systems around the world, where resources have refocused on increasing critical bed capacity to prepare for the peak in incidence of COVID-19. Oncology faces an unprecedented challenge as patients require multidisciplinary care and are more likely to be immunosuppressed. Services in oncology have been transformed using minimal resources over a short period of time. This transformation continues and telemedicine is playing a key role. AIMS: We explore how services in oncology have transformed to deliver services including consultations, systemic anticancer therapy, and surgery for patients, while shielding them from contracting COVID-19. We assess the risks and benefits of the service transformation in the immediate, interim, and long term, and how telemedicine supports the process. METHODS: We performed a comprehensive review of the literature using suitable keywords on the search engines of PubMed, SCOPUS, Google Scholar, and latest official data from May to June 2020. RESULTS: Through the published literature on this topic, we discuss the transformations in oncology and the impact on patients and healthcare workers due to the COVID-19 pandemic. We reflect on the lessions from COVID-19 and assess the role of telemedicine in the future of oncology services. CONCLUSION: Transformation of services in oncology effectively shields patients from COVID-19 infections, and telemedicine plays a role in virtual consultations. The long-term effects are yet to be seen, such as safety of home-based treatment, and effectiveness of virtual communication on patient care. As oncology requires a multidisciplinary approach, telemedicine will play a key role to improve patient-centered cancer care in the future.

Levin, M., et al. (2020). "Acute asthma management during SARS-CoV2-pandemic 2020." World Allergy Organ J **13**(5): 100125.

BACKGROUND: The current COVID-19 pandemic has changed many medical practices in order to provide additional protection to both our patients and healthcare providers. In many cases this includes seeing patients through electronic means such as telehealth or telephone rather than seeing them in person. Asthma exacerbations cannot always be treated in this way. PROBLEM: Current emergency unit asthma guidelines recommend bronchodilators be administered by metered dose inhaler (MDI) and spacer for mild-moderate asthma and include it as a choice even in severe asthma, but many emergency units continue to prefer nebulised therapy for patients who urgently require beta-agonists. The utilization of nebulised therapy potentially increases the risk of aerosolization of the coronavirus. Since nosocomial transmission of respiratory pathogens is a major threat in the context of the SARS-CoV-2 pandemic, use of nebulised therapy is of even greater concern due to the potential increased risk of infection spread to nearby patients and healthcare workers. PRACTICAL IMPLICATIONS: We propose a risk stratification plan that aims to avoid nebulised therapy, when possible, by providing an algorithm to help better delineate those who require nebulised therapy. Protocols that include strategies to allow flexibility in using MDIs rather than nebulisers in all but the most severe patients should help mitigate this risk of aerosolised infection transmission to patients and health care providers. Furthermore, expedient treatment of patients with high dose MDI therapy augmented with more rapid initiation of systemic therapy may help ensure patients are less likely to deteriorate to the stage where nebulisers are required.

Lewin, I. J. S., et al. (2020). "Preparing a deployed role 3 medical treatment facility for COVID-19 in Afghanistan." BMJ Mil Health.

The Craig Joint Theater Hospital at Bagram Airfield is the coalition role 3 facility for the North Atlantic Treaty Organization-led Operation RESOLUTE SUPPORT in northern Afghanistan. The onset of the global COVID-19 pandemic in early 2020 presented the challenges of limiting viral transmission, disease force protection, specific protection of healthcare workers and management of patients with COVID-19, all while continuing to provide high-quality care for battlefield trauma. The estimated COVID-19 threat led to the introduction of enhanced force protection measures across the Combined Joint Operations Area-Afghanistan. Surveillance testing of high-risk groups at coalition locations was established. Non-essential movements were restricted and quarantine periods instigated. Protection of healthcare workers and patients was improved with enhanced personal protective equipment as well as structural, procedural and personnel changes across the role 3 facility in order to limit viral exposure and transmission. This occurred in a resource-limited environment without degrading overall clinical capability.

Li, D. T. S., et al. (2021). "Facial protection in the era of COVID-19: A narrative review." Oral Dis **27 Suppl 3**: 665-673.

We live in extraordinary times, where COVID-19 pandemic has brought the whole world to a screeching halt. Tensions and contradictions that surround the pandemic ridden world include the availability, and the lack thereof, various facial protection measures to mitigate the viral spread. Here, we comprehensively explore the different types of facial protection measures, including masks, needed both for the public and the healthcare workers (HCW). We discuss the anatomy, the critical issues of disinfection and reusability of masks, the alternative equipment available for the protection of the facial region from airborne diseases, such as face shields and powered air-purifying respirators (PAPR), and the skin health impact of prolonged wearing of facial protection by HCW. Clearly, facial protection, either in the form of masks or alternates, appears to have mitigated the pandemic as seen from the minimal COVID-19 spread in countries where public mask wearing is strictly enforced. On the contrary, the healthcare systems, that appear to have been unprepared for emergencies of this nature, should be appropriately geared to handle the imbalance of supply and demand of personal protective equipment including face masks. These are two crucial lessons we can learn from this tragic experience.

Li, F., et al. (2021). "Factors that affect the duration of wearing disposable personal protective equipment by healthcare professionals in Wuhan during treatment of COVID-19 patients: An epidemiological study." Nurs Health Sci **23**(1): 245-254.

The purpose of this study of healthcare workers who cared for COVID-19 patients was to identify factors that affected the duration of wearing personal protective equipment (PPE). The results of this study will provide initial guidance to practicing clinicians and a foundation for further research on this topic. This cross-sectional study examined 139 frontline healthcare professionals who worked at a single hospital in Wuhan, China, from March 16 to April 1, 2020. General and demographic data, physical and mental status, use of personal protective equipment, type of hospital work, and duration of wearing personal protective equipment were recorded. The mean duration of wearing personal protective equipment was 194.17 min (standard deviation: 3.71). Multiple linear regression analysis indicated that the duration of wearing personal protective equipment was significantly associated with the presence of a chronic disease, working hours when feeling discomfort, lack of patient cooperation and subsequent psychological pressure, prolonged continuous wearing of personal protective equipment, feeling anxious about physical strength, and the presence of fatigue when wearing personal protective equipment. These factors should be considered by practicing healthcare professionals and in future studies that examine the optimal duration of wearing personal protective equipment.

Li, J., et al. (2020). "Mild manifestations of COVID-19 in healthcare workers." PLoS Negl Trop Dis **14**(12): e0008950.

Medical staff treating Coronavirus Disease 2019 (COVID-19) patients are at high risk for exposure to Severe Acute Respiratory Syndrome Coronavirus 2 (SARS-CoV-2), and many have been infected, which may cause panic among medical workers, their relatives, health professionals, and government leaders. We report the epidemiologic and clinical characteristics of healthcare workers and that the majority of infected medical staff had milder symptoms/conditions with a better prognosis than admitted patients. Timely improvement to medical staff's working conditions such as allowing adequate rest and providing sufficient medical protection is extremely important.

Li, Q., et al. (2020). "Airway management strategies under the situation of prevention and control of nosocomial infections for coronavirus disease 2019." Zhong Nan Da Xue Xue Bao Yi Xue Ban **45**(5): 603-608.

Since the outbreak of COVID-19, the prevention and control of nosocomial infections has been highly valued. Airway management, including endotracheal intubation, extubation, and suction, has been considered as the high-risk virus-spreading procedures, which can put the health providers at a high risk of nosocomial infections. As hospitals at all levels will gradually resume their routine medical work, effective managements for the airway of the silent asymptomatic carriers and patients with delayed symptoms, treatment for severe patients, and prevention of cross infection in hospital have become the focus for the current prevention and control of nosocomial infections. Under the guidance of partitioned and graded prevention and differential control strategies at this stage, we comprehensively analyzed four main intubation methods used in the current clinical work including rapid sequence intubation, laryngeal mask insertion, intubation guided by video flexible intubating scope and awake tracheal intubation. Furthermore, we discussed and summarized intubation and protection strategies for 3 categories of patients during the COVID-19 pandemic, providing evidence for protecting medical stuff in operating room and ICU against severe acute respiratory syndrome coronavirus 2 infection.

Li, W., et al. (2020). "Progression of Mental Health Services during the COVID-19 Outbreak in China." Int J Biol Sci **16**(10): 1732-1738.

The novel coronavirus disease (COVID-19) has been rapidly transmitted in China, Macau, Hong Kong, and other Asian and European counterparts. This COVID-19 epidemic has aroused increasing attention nationwide. Patients, health professionals, and the general public are under insurmountable psychological pressure which may lead to various psychological problems, such as anxiety, fear, depression, and insomnia. Psychological crisis intervention plays a pivotal role in the overall deployment of the disease control. The National Health Commission of China has summoned a call for emergency psychological crisis intervention and thus, various mental health associations and organizations have established expert teams to compile guidelines and public health educational articles/videos for mental health professionals and the general public alongside with online mental health services. In addition, mental health professionals and expert groups are stationed in designated isolation hospitals to provide on-site services. Experts have reached a consensus on the admission of patients with severe mental illness during the COVID-19 outbreak in mental health institutions. Nevertheless, the rapid transmission of the COVID-19 has emerged to mount a serious challenge to the mental health service in China.

Li, X., et al. (2021). "Review of the risk factors for SARS-CoV-2 transmission." World J Clin Cases **9**(7): 1499-1512.

The severe acute respiratory syndrome coronavirus 2 (SARS-CoV-2) pandemic, which has lasted for nearly a year, has made people deeply aware of the strong transmissibility and pathogenicity of SARS-CoV-2 since its outbreak in December 2019. By December 2020, SARS-CoV-2 had infected over 65 million people globally, resulting in more than 1 million deaths. At present, the exact animal origin of SARS-CoV-2 remains unclear and antiviral vaccines are now undergoing clinical trials. Although the social order of human life is gradually returning to normal, new confirmed cases continue to appear worldwide, and the majority of cases are sporadic due to environmental factors and lax self-protective consciousness. This article provides the latest understanding of the epidemiology and risk factors of nosocomial and community transmission of SARS-CoV-2, as well as strategies to diminish the risk of transmission. We believe that our review will help the public correctly understand and cope with SARS-CoV-2.

Li, Y., et al. (2020). "Face masks to prevent transmission of COVID-19: A systematic review and meta-analysis." Am J Infect Control.

BACKGROUND: Based on the status of the COVID-19 global pandemic, there is an urgent need to systematically evaluate the effectiveness of wearing masks to protect public health from COVID-19 infection. METHODS: The Preferred Reporting Items for Systematic Reviews and Meta-Analysis statement was consulted to report this systematic review. We conducted a systematic review and meta-analysis to evaluate the effectiveness of using face masks to prevent the spread of SARS-CoV-2. Relevant articles were retrieved from PubMed, Web of Science, ScienceDirect, Cochrane Library, and Chinese National Knowledge Infrastructure, VIP (Chinese) database. There were no language restrictions. This study was registered with PROSPERO under the number CRD42020211862. RESULTS: A total of 6 studies were included, involving 4 countries, after a total of 5,178 eligible articles were searched in databases and references. In general, wearing a mask was associated with a significantly reduced risk of COVID-19 infection (OR = 0.38, 95% CI: 0.21-0.69, I(2) = 54.1%). For the healthcare workers group, masks were shown to have a reduced risk of infection by nearly 70%. Sensitivity analysis showed that the results were robust. CONCLUSIONS: The results of this systematic review and meta-analysis support the conclusion that wearing a mask could reduce the risk of COVID-19 infection. Robust randomized trials are needed in the future to better provide evidence for these interventions.

Li, Y. K., et al. (2020). "Clinical and Transmission Characteristics of Covid-19 - A Retrospective Study of 25 Cases from a Single Thoracic Surgery Department." Curr Med Sci **40**(2): 295-300.

The outbreak of corona virus disease 2019 (Covid-19) imposes a major challenge in managing patients undergoing surgical operation. In this study, we analyzed clinical and transmission features of 25 cases of Covid-19 from a single thoracic department, including 13 patients and 12 health care staff. There were 13 males and 12 females. The median age of the patients was 61 (range: 51 to 69) years. The median age of the health care staff was 35 (range: 22 to 51) years. By the end of follow-up date (Mar. 3, 2020), there were 16 non-severe cases (64%) and 9 severe cases (36%), 5 cases were dead (20%). Nineteen (76%) of the infected cases were confirmed by SARS-CoV-2 nucleic acid test, the rest were clinically diagnosed as suspected Covid-19 cases, and 19 (76%) of the infected cases had positive exposure history. We found that COPD was significantly associated with severity and death (P=0.040, and P=0.038, respectively), and chest operation was significantly associated with death for Covid-19 patients (P=0.039). A potential "super spreader" may be the source of the transmission before the implementation of quarantine and comprehensive protection. It was concluded that Covid-19 is associated with poor prognosis for patients undergoing thoracic operation, especially for those with COPD. Implementation of comprehensive protective measures is important to control nosocomial infection.

Liang, M., et al. (2020). "Efficacy of face mask in preventing respiratory virus transmission: A systematic review and meta-analysis." Travel Med Infect Dis **36**: 101751.

BACKGROUND: Conflicting recommendations exist related to whether masks have a protective effect on the spread of respiratory viruses. METHODS: The Preferred Reporting Items for Systematic Reviews and Meta-Analysis (PRISMA) statement was consulted to report this systematic review. Relevant articles were retrieved from PubMed, Web of Science, ScienceDirect, Cochrane Library, and Chinese National Knowledge Infrastructure (CNKI), VIP (Chinese) database. RESULTS: A total of 21 studies met our inclusion criteria. Meta-analyses suggest that mask use provided a significant protective effect (OR = 0.35 and 95% CI = 0.24-0.51). Use of masks by healthcare workers (HCWs) and non-healthcare workers (Non-HCWs) can reduce the risk of respiratory virus infection by 80% (OR = 0.20, 95% CI = 0.11-0.37) and 47% (OR = 0.53, 95% CI = 0.36-0.79). The protective effect of wearing masks in Asia (OR = 0.31) appeared to be higher than that of Western countries (OR = 0.45). Masks had a protective effect against influenza viruses (OR = 0.55), SARS (OR = 0.26), and SARS-CoV-2 (OR = 0.04). In the subgroups based on different study designs, protective effects of wearing mask were significant in cluster randomized trials and observational studies. CONCLUSIONS: This study adds additional evidence of the enhanced protective value of masks, we stress that the use masks serve as an adjunctive method regarding the COVID-19 outbreak.

Liang, X. Y., et al. (2021). "Management of acute coronary syndrome in the context of coronavirus disease 2019." Medicine (Baltimore) **100**(1): e24151.

Coronavirus disease 2019 (COVID-19) is still developing worldwide. The prognosis of the disease will become worse and mortality will be even higher when it is combined with cardiovascular disease. Furthermore, COVID-19 is highly infectious and requires strict isolation measures. For acute coronary syndromes (ACS), a common cardiovascular disease, infection may aggravate the occurrence and development of ACS, making the management of more difficult. It will be an enormous challenge for clinical practice to deal with ACS in this setting of COVID-19.Aim to reduce the mortality of ACS patients during the epidemic of COVID-19 by standardizing procedures as much as possible.Pubmed and other relevant databases were searched to retrieve articles on COVID-19 and articles on ACS management strategies during previous influenza epidemics. The data was described and synthesized to summarize the diagnosis and management strategy of ACS, the preparation of catheter laboratory, and the protection of the medical staff in the context of COVID-19. Ethical approval is not required in this study, because it is a review with no recourse to patient identifiable information.Standardized diagnosis and treatment advice can help reduce the mortality of COVID-19 patients with ACS. In the absence of contraindications, the third generation of thrombolytic drugs should be the first choice for thrombolytic treatment in the isolation ward. For patients who have to receive PCI, this article provides detailed protective measures to avoid nosocomial infection.

Licina, A. and A. Silvers (2021). "Use of powered air-purifying respirator(PAPR) as part of protective equipment against SARS-CoV-2-a narrative review and critical appraisal of evidence." Am J Infect Control **49**(4): 492-499.

BACKGROUND: The last 2 decades have seen an increasing frequency of zoonotic origin viral diseases leaping from animal to human hosts including Severe Acute Respiratory Syndrome Coronaviruses (SARS-CoV-2). Respiratory component of the infectious disease program against SARS-CoV-2 incorporates use of protective airborne respiratory equipment. METHODS: In this narrative review, we explore the features of Powered Air Purifying Respirators (PAPR) as well as logistical and evidence-based advantages and disadvantages. RESULTS: Simulation study findings support increased heat tolerance and wearer comfort with a PAPR, versus decreased communication ability, mobility, and dexterity. Although PAPRs have been recommended for high-risk procedures on suspected or confirmed COVID-19 patients, this recommendation remains controversial due to lack of evidence. Guidelines for appropriate use of PAPR during the current pandemic are sparse. International regulatory bodies do not mandate the use of PAPR for high-risk aerosol generating procedures in patients with SARS-CoV-2. Current reports of the choice of protective respiratory technology during the SARS-CoV-2 pandemic are disparate. Patterns of use appear to be related to geographical locations. DISCUSSION: Field observational studies do not indicate a difference in healthcare worker infection utilizing PAPR devices versus other compliant respiratory equipment in healthcare workers performing AGPs in patients with SARS-CoV-2. Whether a higher PAPR filtration factor translates to decreased infection rates of HCWs remains to be elucidated. Utilization of PAPR with high filtration efficiency may represent an example of "precautionary principle" wherein action taken to reduce risk is guided by logistical advantages of PAPR system.

Licina, A., et al. (2020). "Use of powered air-purifying respirator (PAPR) by healthcare workers for preventing highly infectious viral diseases-a systematic review of evidence." Syst Rev **9**(1): 173.

BACKGROUND: Healthcare workers (HCWs) are at particular risk during pandemics and epidemics of highly virulent diseases with significant morbidity and case fatality rate. These diseases include severe acute respiratory syndrome coronaviruses, SARS-CoV-1 and SARS-CoV-2, Middle Eastern Respiratory Syndrome (MERS), and Ebola. With the current (SARS-CoV-2) global pandemic, it is critical to delineate appropriate contextual respiratory protection for HCWs. The aim of this systematic review was to evaluate the effect of powered air-purifying respirators (PAPRs) as part of respiratory protection versus another device (egN95/FFP2) on HCW infection rates and contamination. METHODS: Our primary outcomes included HCW infection rates with SARS-CoV-2, SARS-CoV-1, Ebola, or MERS when utilizing PAPR. We included randomized controlled trials, non-randomized controlled trials, and observational studies. We searched the following databases: MEDLINE, EMBASE, and Cochrane Library (Cochrane Database of Systematic Reviews and CENTRAL). Two reviewers independently screened all citations, full-text articles, and abstracted data. Due to clinical and methodological heterogeneity, we did not conduct a meta-analysis. Where applicable, we constructed evidence profile (EP) tables for each individual outcome. Confidence in cumulative evidence for each outcome was classified according to the GRADE system. RESULTS: We identified 689 studies during literature searches. We included 10 full-text studies. A narrative synthesis was provided. Two on-field studies reported no difference in the rates of healthcare workers performing airway procedures during the care of critical patients with SARS-CoV-2. A single simulation trial reported a lower level of cross-contamination of participants using PAPR compared to alternative respiratory protection. There is moderate quality evidence that PAPR use is associated with greater heat tolerance but lower scores for mobility and communication ability. We identified a trend towards greater self-reported wearer comfort with PAPR technology in low-quality observational simulation studies. CONCLUSION: Field observational studies do not indicate a difference in healthcare worker infection utilizing PAPR devices versus other compliant respiratory equipment. Greater heat tolerance accompanied by lower scores of mobility and audibility in PAPR was identified. Further pragmatic studies are needed in order to delineate actual effectiveness and provider satisfaction with PAPR technology. SYSTEMATIC REVIEW REGISTRATION: The protocol for this review was prospectively registered with the International Register of Systematic Reviews identification number CRD42020184724 .

Lidström, A. K., et al. (2020). "Work at inpatient care units is associated with an increased risk of SARS-CoV-2 infection; a cross-sectional study of 8679 healthcare workers in Sweden." Ups J Med Sci **125**(4): 305-310.

BACKGROUND: During the Covid-19 pandemic, the protection of healthcare workers has been in focus throughout the world, but the availability and quality of personal protective equipment has at times and in some settings been suboptimal. MATERIALS AND METHODS: A total of 8679 healthcare workers and healthcare support staff in the county of Uppsala, north of Stockholm, were included in this cross-sectional study. All subjects were analysed for IgG anti-SARS-CoV-2, and predictors for positive serostatus were analysed in a logistic regression model including demographic parameters and self-reported employment characteristics. RESULTS: Overall, 577 (6.6%) were classified as seropositive, with no statistically significant differences between healthcare workers and support staff. Among healthcare workers, age (OR 0.987 per year, 95% CI 0.980-0.995), time to sampling (OR 1.019 per day, 95% CI 1.004-1.035), and employment at an outpatient care unit (OR 0.620, 95% CI 0.487-0.788) were statistically significantly associated with risk of infection. Covid-19 specific units were not at particular risk, compared to other units with comparable characteristics and staff demography. CONCLUSION: Our findings indicate that SARS-CoV-2 transmission is related to inpatient healthcare work, and illustrate the need for a high standard of basic hygiene routines in all inpatient care settings.

Lie, S. A., et al. (2020). "Practical considerations for performing regional anesthesia: lessons learned from the COVID-19 pandemic." Can J Anaesth **67**(7): 885-892.

Coronavirus disease (COVID-19) was declared a pandemic by the World Health Organization on 11 March 2020 because of its rapid worldwide spread. In the operating room, as part of hospital outbreak response measures, anesthesiologists are required to have heightened precautions and tailor anesthetic practices to individual patients. In particular, by minimizing the many aerosol-generating procedures performed during general anesthesia, anesthesiologists can reduce exposure to patients' respiratory secretions and the risk of perioperative viral transmission to healthcare workers and other patients. To avoid any airway manipulation, regional anesthesia should be considered whenever surgery is planned for a suspect or confirmed COVID-19 patient or any patient who poses an infection risk. Regional anesthesia has benefits of preservation of respiratory function, avoidance of aerosolization and hence viral transmission. This article explores the practical considerations and recommended measures for performing regional anesthesia in this group of patients, focusing on control measures geared towards ensuring patient and staff safety, equipment protection, and infection prevention. By doing so, we hope to address an issue that may have downstream implications in the way we practice infection control in anesthesia, with particular relevance to this new era of emerging infectious diseases and novel pathogens. The severe acute respiratory syndrome coronavirus 2 (SARS-CoV-2) is not the first, and certainly will not be the last novel virus that will lead to worldwide outbreaks. Having a well thought out regional anesthesia plan to manage these patients in this new normal will ensure the best possible outcome for both the patient and the perioperative management team.

Lim, C. Y., et al. (2020). "Staff rostering, split team arrangement, social distancing (physical distancing) and use of personal protective equipment to minimize risk of workplace transmission during the COVID-19 pandemic: A simulation study." Clin Biochem **86**: 15-22.

BACKGROUND: The recent global survey promoted by the International Federation of Clinical Chemistry and Laboratory Medicine (IFCC) Taskforce on COVID-19 (coronavirus disease 2019) described staff rostering and organization as significant operational challenges during the COVID-19 pandemic. METHOD: A discrete event simulation was used to explore the impact of different permutations of staff roster, including the number of shifts per day, the number of staff on duty per shift, overall number of staff accessible to work in the laboratory (i.e. overall staff pool), the frequency of shift changes (i.e. number of consecutive days worked), fixed work-rest days and split team arrangement on workplace transmission of COVID-19 by a simulated index staff who acquired the infection from the community over 21 days. Additionally, the impact of workplace social distancing (physical distancing) and use of personal protective equipment (PPE) were investigated. RESULTS: A higher rate of transmission was associated with smaller overall staff pool (expressed as multiples of the number of staff per shift), higher number of shifts per day, higher number of staff per shift, and longer consecutive days worked. Having fixed work-rest arrangement did not significantly reduce the transmission rate unless the workplace outbreak was prolonged. Social distancing and PPE use significantly reduced the transmission rate. CONCLUSION: Laboratories should consider organizing the staff into smaller teams/shift and reduce the number of consecutive days worked. Additionally, our observation aligns with the IFCC biosafety recommendation of monitoring staff health (to detect early infection), split team arrangement, workplace social distancing and use of PPE.

Lim, L. W., et al. (2020). "Sustainable practice of ophthalmology during COVID-19: challenges and solutions." Graefes Arch Clin Exp Ophthalmol **258**(7): 1427-1436.

PURPOSE: The Coronavirus (COVID-19) outbreak is rapidly emerging as a global health threat. With no proven vaccination or treatment, infection control measures are paramount. In this article, we aim to describe the impact of COVID-19 on our practice and share our strategies and guidelines to maintain a sustainable ophthalmology practice. METHODS: Tan Tock Seng Hospital (TTSH) Eye Centre is the only ophthalmology department supporting the National Centre for Infectious Diseases (NCID), which is the national screening center and the main center for management of COVID-19 patients in Singapore. Our guidelines during this outbreak are discussed. RESULTS: Challenges in different care settings in our ophthalmology practice have been identified and analyzed with practical solutions and guidelines implemented in anticipation of these challenges. First, to minimize cross-infection of COVID-19, stringent infection control measures were set up. These include personal protective equipment (PPE) for healthcare workers and routine cleaning of "high-touch" surfaces. Second, for outpatient care, a stringent dual screening and triaging process were carried out to identify high-risk patients, with proper isolation for such patients. Administrative measures to lower patient attendance and reschedule appointments were carried out. Third, inpatient and outpatient care were separated to minimize interactions. Last but not least, logistics and manpower plans were drawn up in anticipation of resource demands and measures to improve the mental well-being of staff were implemented. CONCLUSION: We hope our measures during this COVID-19 pandemic can help ophthalmologists globally and serve to guide and maintain safe access in ophthalmology clinics when faced with similar disease outbreaks.

Limbu, D. K., et al. (2020). "Healthcare workers' knowledge, attitude and practices during the COVID-19 pandemic response in a tertiary care hospital of Nepal." PLoS One **15**(11): e0242126.

BACKGROUND: COVID-19 is an ongoing pandemic, for which appropriate infection prevention and control measures need to be adopted. Healthcare workers' adherence to prevention and control measures is affected by their knowledge, attitudes, and practices (KAP) towards COVID-19. In this study, we assessed the KAP among healthcare workers towards the COVID-19 during the ongoing pandemic. METHOD: A self-developed piloted KAP questionnaire was administered to the recruited healthcare workers involved in the COVID-19 response at the Universal College of Medical Sciences Teaching Hospital (UCMSTH), in Bhairahawa, Nepal. The knowledge questionnaire consisted of questions regarding the clinical characteristics, prevention, and management of COVID-19. Assessment on attitudes and practices towards COVID-19 included questions on behaviour and change in practices made towards COVID-19 response. Knowledge scores were calculated and compared by demographic characteristics and their attitude and practices towards COVID-19. Data were analysed using bivariate statistics. RESULTS: A total of 103 healthcare workers participated in the study. The mean age of the participants was 28.24±6.11 years (range: 20-56); 60.2% were females; 61.2% were unmarried; 60.2% had a medical degree, and 39.8% were the nursing staff. The mean knowledge score was 10.59±1.12 (range: 7-13), and it did not vary significantly when adjusted for demographic characteristics. The attitude was positive for 53.4% of the participants with a mean knowledge score of 10.35±1.19 and negative for 46.6% participants with a mean knowledge score of 10.88±0.98 (p = 0.02). The practice was good (≥3 score) for 81.5% participants with a mean knowledge score of 10.73±1.12 and poor for 18.5% participants with a mean knowledge score of 10.46±1.13 (p = 0.24). The attitude of the participants improved with increasing age (29.55±7.17, p = 0.02). CONCLUSION: There is comparably better knowledge regarding COVID-19 among healthcare workers. Appropriate practice correlates with better knowledge and positive attitude towards COVID-19 infection is seen with increasing age. Hence, training on protection and protective measures for having a positive attitude among healthcare workers is necessary against the fight with COVID-19 infection.

Lin, L., et al. (2020). "Management strategies in a thoracic surgery ward during COVID-19 pandemic: Experience from West China Hospital." World J Virol **9**(4): 47-53.

The coronavirus disease 2019 was first reported in Wuhan in December 2019 and then spread rapidly throughout the world. On March 11, 2020, the World Health Organization declared coronavirus disease 2019 a pandemic. In response to the pandemic, the management division of West China Hospital oversaw the implementation of hospital-wide emergency measures. In accordance with these measures, the hospital's thoracic surgery ward implemented a new management system by reformulating staff training plans, patient admission procedures, and other systems for managing the ward and protecting perioperative patients. Overall, the ward was successful in restoring normal working order, protecting all staff from occupational exposures, and ensuring the safety of inpatients and their families.

Lin, Z., et al. (2020). "Ward renovation and PPE use procedures to protect medical staff from COVID-19 infection." J Infect Dev Ctries **14**(6): 554-558.

In the early stages of the coronavirus disease 2019 (COVID-19) outbreak in Wuhan, many cross-infections occurred due to the limited number of wards and insufficient medical staff, which could not cope with the large number of patients visiting the hospital. A series of new infection control measures were implemented in our institution and a Wuhan hospital supported by our medical team, mainly including temporarily transforming the general ward into a passage for the staff to enter the infectious ward and standardizing the procedure for the wearing and removal of personal protection equipment (PPE). These measures significantly improved the situation, and no member of our medical staff was infected with severe acute respiratory syndrome coronavirus 2 (SARS-CoV-2) during the middle and late stages of the disease epidemic. We hope that these experiences can provide references for medical institutions that may face an outbreak of COVID-19, especially those in underdeveloped countries and regions.

Lincango-Naranjo, E., et al. (2021). "Paradigms about the COVID-19 pandemic: knowledge, attitudes and practices from medical students." BMC Med Educ **21**(1): 128.

BACKGROUND: As the disease caused by the novel coronavirus has spread globally, there has been significant economic instability in the healthcare systems. This reality was especially accentuated in Ecuador where, the shortage of healthcare workers combined with cultural and macroeconomic factors has led Ecuador to face the most aggressive outbreak in Latin America. In this context, the participation of final-year medical students on the front line is indispensable. Appropriate training on COVID-19 is an urgent requirement that universities and health systems must guarantee. We aimed to describe the knowledge, attitudes, and practices of Ecuadorian final-year medical students that could potentially guide the design of better medical education curricula regarding COVID-19. METHODS: This was a cross-sectional 33-item online survey conducted between April 6 to April 2020 assessing the knowledge, attitudes, and practices toward the diagnosis, treatment, prevention, and prognosis toward COVID-19 in Ecuadorian final-year medical students. It was sent by email, Facebook, and WhatsApp. RESULTS: A total of 309 students responded to the survey. Out of which 88% of students scored high (≥ 70% correct) for knowledge of the disease. The majority of students were pessimistic about possible government actions, which is reflected in the negative attitude towards the control of COVID-19 and volunteering during the outbreak in Ecuador (77%, and 58% of the students, respectively). Moreover, 91% of students said they did not have adequate protective equipment. The latter finding was significantly associated with negative attitudes. CONCLUSIONS: Although a large number of students displayed negative attitudes, the non-depreciable percentage of students who were willing to volunteer and the coexisting high level of knowledge displayed by students, suggests that Ecuador has a capable upcoming workforce that could benefit from an opportunity to strengthen, improve and advance their training in preparation for COVID-19. Not having personal protective equipment was significantly associated to negative attitudes. Providing the necessary tools and creating a national curriculum may be one of the most effective ways to ensure all students are trained, whilst simultaneously focusing on the students' most pressing concerns. With this additional training, negative attitudes will improve and students will be better qualified.

Liné, C., et al. (2020). "[Not Available]." Rech Soins Infirm(142): 77-85.

Introduction&#160;: The COVID-19 pandemic has warranted the implementation of barrier actions and the wearing of personal protective equipment (PPE). These behavioral adaptations counteract spontaneous self-touching reflex gestures. I propose to explore the mind-body experience generated by these gesture changes in this health context.Method&#160;: I conducted a qualitative study with nine nurses who volunteered as reinforcements. The semistructured interviews I carried out were analyzed using the interpretative phenomenological analysis approach.Results&#160;: The perception of the risk of self-inoculation is not related to the level of virus exposure. Facial PPE is perceived both as reassuring and difficult to put up with. The experience of self-touches is influenced by the level of attention the subject pays to his or her face and by the medical background related to this. Three kinds of mind-body adaptations are used to control self-touching&#160;: control by avoidance, control by keeping one&#8217;s hands busy, and compensatory bodily strategies. The representations of the bodily self are modified in terms of gestures and of the feeling of humanness. This generates a form of bodily abandonment and a lived experience of soiled bodies.Conclusion&#160;: The results highlight the cognitive load involved in refraining from self-touching and with the wearing of facial PPE. They emphasize the importance of questioning the implications of these sensorimotor modifications. Supporting health professionals in assessing the mind-body repercussions in a pandemic situation appears key.

Liow, M. H. L., et al. (2020). "From "business continuity" to "back to business" for orthopaedic surgeons during the COVID-19 pandemic." Bone Jt Open **1**(6): 222-228.

The coronavirus disease 2019 (COVID-19) pandemic has led to unprecedented challenges to healthcare systems worldwide. Orthopaedic departments have adopted business continuity models and guidelines for essential and non-essential surgeries to preserve hospital resources as well as protect patients and staff. These guidelines broadly encompass reduction of ambulatory care with a move towards telemedicine, redeployment of orthopaedic surgeons/residents to the frontline battle against COVID-19, continuation of education and research through web-based means, and cancellation of non-essential elective procedures. However, if containment of COVID-19 community spread is achieved, resumption of elective orthopaedic procedures and transition plans to return to normalcy must be considered for orthopaedic departments. The COVID-19 pandemic also presents a moral dilemma to the orthopaedic surgeon considering elective procedures. What is the best treatment for our patients and how does the fear of COVID-19 influence the risk-benefit discussion during a pandemic? Surgeons must deliberate the fine balance between elective surgery for a patient's wellbeing versus risks to the operating team and utilization of precious hospital resources. Attrition of healthcare workers or Orthopaedic surgeons from restarting elective procedures prematurely or in an unsafe manner may render us ill-equipped to handle the second wave of infections. This highlights the need to develop effective screening protocols or preoperative COVID-19 testing before elective procedures in high-risk, elderly individuals with comorbidities. Alternatively, high-risk individuals should be postponed until the risk of nosocomial COVID-19 infection is minimal. In addition, given the higher mortality and perioperative morbidity of patients with COVID-19 undergoing surgery, the decision to operate must be carefully deliberated. As we ramp-up elective services and get "back to business" as orthopaedic surgeons, we have to be constantly mindful to proceed in a cautious and calibrated fashion, delivering the best care, while maintaining utmost vigilance to prevent the resurgence of COVID-19 during this critical transition period. Cite this article: Bone Joint Open 2020;1-6:222-228.

Liu, H., et al. (2021). "A survey of laboratory biosafety and protective measures in blood transfusion departments during the COVID-19 pandemic." Vox Sang.

BACKGROUND AND OBJECTIVES: Thousands of healthcare workers (HCWs) have been infected with 2019 novel coronavirus pneumonia (COVID-19) during the COVID-19 pandemic. Laboratory personnel in blood transfusion departments may be infected with severe acute respiratory syndrome coronavirus 2 (SARS-CoV-2) if laboratory biosafety protection is insufficient. Therefore, we investigated the current situation of laboratory biosafety protection in blood transfusion departments to determine how to improve the safety of laboratory processes. MATERIALS AND METHODS: An online survey was conducted in blood transfusion departments from 1st to 6th May 2020 in China. A total of 653 individuals completed the questionnaire. The questionnaire was designed with reference to COVID-19 laboratory biosafety summarized in Annex II. All responses were summarized using only descriptive statistics and expressed as frequencies and ratios [n (%)]. RESULTS: Most participants were concerned about COVID-19. Some participants had inadequate knowledge of COVID-19. Two participants stated that there were laboratory personnel infected with SARS-CoV-2 in their departments. A total of 31 (4.7%) participants did not receive any safety and security training. In terms of laboratory biosafety protection practices, the major challenges were suboptimal laboratory safety practices and insufficient laboratory conditions. CONCLUSION: The major deficiencies were insufficient security and safety training, and a lack of personal protective equipment, automatic cap removal centrifuges and biosafety cabinets. Consequently, we should enhance the security and safety training of laboratory personnel to improve their laboratory biosafety protection practices and ensure that laboratory conditions are sufficient to improve the safety of laboratory processes.

Liu, J., et al. (2021). "Midwifery and Nursing Strategies to protect against COVID-19 During the Third Trimester of Pregnancy." Midwifery **92**: 102876.

OBJECTIVE: The rapid pace of the severe acute respiratory syndrome coronavirus 2 (SARS-CoV-2) pandemic (COVID-19) presents significant challenges to midwives and nurses. This study aimed to explore midwifery and nursing interventions to limit the transmission of COVID-19 among women in their third trimester of pregnancy, to reduce the incidence of nosocomial infection and promote safety of care for women and their infants. METHOD: We completed a retrospective review of medical records from 35 women in their third trimester of pregnancy with SARS-CoV-2, admitted to one hospital in Wuhan, China in January and February 2020. We investigated the clinical characteristics of the COVID-19 infection in pregnancy, and the individualized midwifery and nursing care offered, including environmental protection, prevention of nosocomial infection, maternal observations, monitoring of signs and symptoms of COVID-19, and psychological care. RESULT: Thirty-one women had a caesarean section, and four had vaginal births. Retrospective analysis of midwifery and nursing strategies implemented to care for these women showed no maternal complications or nosocomial infections. CONCLUSIONS AND IMPLICATIONS FOR PRACTICE: The care strategies we implemented could prevent complications and nosocomial infection in the third trimester of pregnancy, thus ensuring the safety of women and their infants. Further research needs to determine treatment priorities for women infected with COVID-19 during pregnancy and the postnatal period.

Liu, J., et al. (2020). "Gynecological prevention and control model based on ward rearrangement and zoning management in pandemic period of COVID-19." Panminerva Med.

By 25th April 2020, the outbreak of COVID-19 caused 2719897 confirmed cases and 187705 deaths globally, remarkably more than Severe Acute Respiratory Syndrome (SARS) (8273 cases, 775 deaths) and Middle East Respiratory Syndrome (MERS) (1139 cases, 431 deaths) in 2003 and 2013, respectively. Gynecology is a specialty department with a large number of critical and severe patients. Consequently, it is of preeminent importance to formulate the in-patient management process. Rearranging the gynecological wards and managing ward partition, as well as the medical protection measures in specialized areas, are suitable for the current prevention and control for COVID-19 pandemic and the therapeutic requirements of patients. To effectively minimize nosocomial infections during the COVID- 19 pandemic period, our department implemented a novel prevention strategy based on the ward redesign and partition management. With this model, our department effectively protected the safety and health of patients and medical care staff from cross and nosocomial infection in the hospital. Now we would like to share the experience and strategies we implemented as following.

Liu, M., et al. (2020). "Media exposure to COVID-19 information, risk perception, social and geographical proximity, and self-rated anxiety in China." BMC Public Health **20**(1): 1649.

BACKGROUND: The coronavirus disease 2019 (COVID-19) is an emerging infectious disease that spreads around the world. The lack of effective antiviral drugs and vaccines, along with the relatively high mortality rate and high contagiousness, has raised strong public concerns over COVID-19, especially for people living in the most severely affected areas. This study aimed to clarify the influencing factors for the anxiety level among the Chinese people during the COVID-19 pandemic, with a particular focus on the media exposure to different COVID-19 information. METHODS: A total of 4991 respondents were randomly recruited from a national online panel from February 12th, 2020 to February 14th, 2020, a period when the number of COVID-19 cases surpassed 10,000 in a single day, with the total cases in China reaching up to 90,000. The relationships between media exposure of COVID-19 information, social and geographical proximity to COVID-19, risk perceptions were assessed using hierarchical ordinary least squares regression analysis. RESULTS: The media exposure to COVID-19 information was differently associated with anxiety. Meanwhile, the anxiety level was found to be high in respondents who personally knew someone infected with COVID-19 or those who living in an area with reported cases. Respondents who perceived more risks also reported a higher level of anxiety. CONCLUSIONS: This study highlights the role of media exposure in affecting individuals' anxiety level during the COVID-19 pandemic. Besides, it is recommended that government and health professionals are recommended to adopt effective risk communication strategies to protect citizens' mental health during the pandemic.

Liu, S. Y., et al. (2021). "Protection procedures and preventions against the spread of coronavirus disease 2019 in healthcare settings for nursing personnel: Lessons from Taiwan." Aust Crit Care **34**(2): 182-190.

BACKGROUND: Since coronavirus disease 2019 was first discovered, at the time of writing this article, the number of people infected globally has exceeded 1 million. Its high transmission rate has resulted in nosocomial infections in healthcare facilities all over the world. Nursing personnel account for nearly 50% of the global health workforce and are the primary provider of direct care in hospitals and long-term care facilities. Nurses stand on the front line against the spread of this pandemic, and proper protection procedures are vital. OBJECTIVES: The present study aims to share the procedures and measures used by Taiwan nursing personnel to help reduce global transmission. REVIEW METHODS: Compared with other regions, where large-scale epidemics have overwhelmed the health systems, Taiwan has maintained the number of confirmed cases within a manageable scope. A review of various national and international policies and guidelines was carried out to present proper procedures and preventions for nursing personnel in healthcare settings. RESULTS: This study shows how Taiwan's health system rapidly identified suspected cases as well as the prevention policies and strategies, key protection points for nursing personnel in implementing high-risk nursing tasks, and lessons from a nursing perspective. CONCLUSIONS: Various world media have affirmed the rapid response and effective epidemic prevention strategies of Taiwan's health system. Educating nurses on procedures for infection control, reporting cases, and implementing protective measures to prevent nosocomial infections are critical to prevent further outbreaks.

Liu, Y., et al. (2020). "Protecting Healthcare Workers Amid the COVID-19 Crisis: A Safety Protocol in Wuhan." Front Public Health **8**: 577499.

Coronavirus disease 2019 (COVID-19), which is caused by a distinct coronavirus, is an acute infectious disease that spreads mainly via the respiratory route. During the COVID-19 outbreak, many healthcare systems faced a severe burden when a large number of healthcare workers (HCWs) became infected due to the lack of adequate protection. Consequently, it was apparent that it is important to ensure the health and safety of HCWs in order to control the outbreak throughout society. In this article, we share our successful protocol for protecting the safety of HCWs in the course of their daily work in an orthopedics department with the aim of eventually reducing the risk of nosocomial infection. None of our HCWs or their families contracted the infection during the COVID-19 pandemic.

Liu, Y., et al. (2021). "Corrigendum: Protecting Healthcare Workers Amid the COVID-19 Crisis: A Safety Protocol in Wuhan." Front Public Health **9**: 660323.

[This corrects the article DOI: 10.3389/fpubh.2020.577499.].

Lo, S. Y., et al. (2021). "Perioperative challenges in managing a patient with COVID-19 undergoing debridement for massive scalp myiasis." BMJ Case Rep **14**(2).

A young man presented to our centre needing an urgent debridement of his postcraniotomy wound due to massive myiasis during the COVID-19 pandemic in October 2020. Prior to the surgery, his nasopharyngeal swab real-time PCR test result was unknown. One day later, it returned as SARS-CoV-2 positive. All healthcare workers who were involved in the patient management avoided cross infection as they wore appropriate personal protective equipment. This article depicts the importance of adequate preparations when handling potentially infectious patients and the perioperative issues associated with it.

Lo Vecchio, A., et al. (2021). "Risk of SARS-CoV-2 Transmission in Health Care Personnel Working in a Pediatric COVID-19 Unit." Hosp Pediatr **11**(3): e42-e47.

OBJECTIVES: The paucity of symptoms and the difficulties in wearing personal protective equipment make children a potential source of severe acute respiratory syndrome coronavirus 2 (SARS-CoV-2) infection for health care workers (HCWs). Previous experience in pediatric settings reported high rate of intrahospital SARS-CoV-2 transmission in HCWs caring for children. We aimed at investigating the rate and determinants of SARS-CoV-2 infection among HCWs working in a regional reference center in the Southern Italy. METHODS: A prospective observational study was conducted to monitor the occurrence of SARS-CoV-2 infections among HCWs and investigate the relation between the infection rate and hours of exposure or number and characteristics of procedures, including nasopharyngeal swab, high-flow oxygen delivery, suctioning of airway secretions, sputum induction, and nebulizer administration. RESULTS: After 5 months of monitoring, 425.6 hours of SARS-CoV-2 exposure (18.5 hours per person), and 920 hospital procedures, no case of nosocomial transmission was reported among the 23 HCWs enrolled in the study. CONCLUSIONS: The application of stringent preventive measures, also outside the area dedicated to patients' care, can effectively control infection spreading also in pediatric settings.

Londoño-Ramírez, A. C., et al. (2021). "Impact of COVID-19 on the Anxiety Perceived by Healthcare Professionals: Differences between Primary Care and Hospital Care." Int J Environ Res Public Health **18**(6).

The COVID-19 pandemic has had an emotional impact on healthcare professionals at different levels of care, and it is important to understand the levels of anxiety of hospital personnel (HP) compared to those of primary care personnel (PCP). The objectives herein were to assess the differences in anxiety levels between these populations and to detect factors that may influence them. The anxiety levels (measured using the Hospital Anxiety and Depression (HAD) scale) of the HP and PCP groups were compared using data collected from a cross-sectional study. The secondary variables included demographic and health data, confinement factors, contact with COVID-19 patients, having suffered from COVID-19, perceptions of protection, caregiver overload, threat, and satisfaction with management. We found anxiety "case" (35.6%) and "at-risk" (21%), with statistically significant differences in the group "at risk", and higher scores in the PCP group. The factors associated with the perception of threat and protection were significant determinants of an increase in anxiety, with all of them showing statistically significant differences. There were greater symptoms of anxiety in the PCP group than the HP group (32% vs. 18%). The factors associated with the prevalence of anxiety symptoms were the perceptions of threat, protection, management, caregiver overload, and perceived degree of threat associated with COVID-19.

Lotta, G., et al. (2021). "The vulnerabilities of the Brazilian health workforce during health emergencies: Analysing personal feelings, access to resources and work dynamics during the COVID-19 pandemic." Int J Health Plann Manage.

Public health emergencies are a test of resilience for health systems, which depend on health workforces that are well managed and cared for. The COVID-19 pandemic exposed the weakness of many health systems in preparing their health workforces. The crisis also exacerbated the unequal conditions between different professions, an issue that is still understudied in the workforce literature. This paper analyzes the consequences of the COVID-19 pandemic for different health professionals, considering the ways in which Brazil's the health system does or does not protect them. We also analyse the role of pre-existing inequalities between different professions and social groups within the workforce in shaping their different experiences of the pandemic. We present data comparing the perceptions of different health professionals facing the pandemic in Brazil: physicians, nurses, and community health workers. Data were collected in an online survey in Brazil with 1630 health care workers between June 15th and July 1st. Findings suggest that none of the professions felt well prepared to work under emergencies. However, differences relating to professional background were exacerbated during the pandemic, creating unequal conditions for different health workers. These inequalities may pose new challenges for the post-pandemic scenario.

Lubansu, A., et al. (2020). "COVID-19 Impact on Neurosurgical Practice: Lockdown Attitude and Experience of a European Academic Center." World Neurosurg **144**: e380-e388.

BACKGROUND: The coronavirus disease 2019 (COVID-19) pandemic is an unprecedented challenge. Different models of reorganization have been described aiming to preserve resources and ensure optimal medical care. Limited clinical neurosurgical experience with patients with COVID-19 has been reported. We share organizational experience, attitudes, and preliminary data of patients treated at our institution. METHODS: Institutional guidelines and patient workflow are described and visualized. A cohort of all neurosurgical patients managed during the lockdown period is presented and analyzed, assessing suspected nosocomial infection risk factors. A comparative surgical subcohort from the previous year was used to investigate the impact on surgical activity. RESULTS: A total of 176 patients were admitted in 66 days, 20 of whom tested positive for COVID-19. Patients initially admitted to the neurosurgical ward were less likely to be suspected for a COVID-19 infection compared with patients admitted for critical emergencies, particularly with neurovascular and stroke-related diseases. The mortality of patients with COVID-19 was remarkably high (45%), and even higher in patients who underwent surgical intervention (77%). In addition to the expected decrease in surgical activity (-53%), a decrease in traumatic emergencies was noted. CONCLUSIONS: By applying infection prevention and resource-sparing logistics measures shared by the international medical community, we were able to maintain essential neurosurgical care in a pandemic with controlled nosocomial infection risk. Special consideration should be given to medical management and surgical indications in patients infected with severe acute respiratory syndrome coronavirus 2, because they seem to show a problematic hemostatic profile that might result in an unfavorable clinical and surgical outcome.

Luceño-Moreno, L., et al. (2020). "Symptoms of Posttraumatic Stress, Anxiety, Depression, Levels of Resilience and Burnout in Spanish Health Personnel during the COVID-19 Pandemic." Int J Environ Res Public Health **17**(15).

The number of health workers infected with COVID-19 in Spain is one of the highest in the world. The aim of this study is to analyse posttraumatic stress, anxiety and depression during the COVID-19 pandemic. Associations between burnout, resilience, demographic, work and COVID-19 variables are analysed. Cross-sectional data on 1422 health workers were analysed. A total of 56.6% of health workers present symptoms of posttraumatic stress disorder, 58.6% anxiety disorder, 46% depressive disorder and 41.1% feel emotionally drained. The profile of a health worker with greater posttraumatic stress symptoms would be a person who works in the Autonomous Community of Madrid, in a hospital, is a woman, is concerned that a person he/she lives with may be infected, and thinks that he/she is very likely to be infected. The risk variables for anxiety and depression would be a person that is a woman, working 12- or 24-h shifts, and being worried that a family member could be infected. High scores on emotional exhaustion and depersonalization are risk factors for mental health, with resilience and personal fulfilment being protective variables. Data are provided to improve preventive measures for occupational health workers.

Lui, R. N. (2020). "Safety in endoscopy for patients and healthcare workers during the COVID-19 pandemic." Tech Innov Gastrointest Endosc.

The coronavirus disease 2019 (COVID-19), caused by the Severe Acute Respiratory Syndrome Coronavirus 2 (SARS-CoV-2), is still wreaking havoc in many parts of the world and poses a great burden to healthcare systems worldwide. Mitigation and suppression strategies have been implemented globally but the disease has proven to be difficult to contain. Initially many elective gastrointestinal (GI) endoscopies were cancelled to reduce the risk of infection and conserve personal protective equipment (PPE), but many endoscopy units are now faced with the dilemma of resuming endoscopy service during the pandemic as indefinitely postponing diagnostic procedures may lead to a delay in the diagnosis and treatment of malignancies. Further concerns are surfacing as COVID-19 is now known to affect the GI tract and may potentially be spread via the fecal-oral route. Until more effective drugs and vaccines are available, it is unlikely that the pandemic will wind down in the near future. Maintaining a balance between protecting healthcare workers (HCWs) and patients from being infected on the one hand and providing timely and effective clinical care on the other will become increasingly important as the pandemic persists. In this narrative review, the risk of COVID-19 infection for HCWs and patients undergoing endoscopy, and recommendations on maintaining safe, high-quality endoscopy practice will be discussed.

Lui, R. N., et al. (2020). "Overview of guidance for endoscopy during the coronavirus disease 2019 pandemic." J Gastroenterol Hepatol **35**(5): 749-759.

From its beginning in December 2019, the coronavirus disease 2019 outbreak has spread globally from Wuhan and is now declared a pandemic by the World Health Organization. The sheer scale and severity of this pandemic is unprecedented in the modern era. Although primarily a respiratory tract infection transmitted by direct contact and droplets, during aerosol-generating procedures, there is a possibility of airborne transmission. In addition, emerging evidence suggests possible fecal-oral spread of the virus. Clinical departments that perform endoscopy are faced with daunting challenges during this pandemic. To date, multiple position statements and guidelines have been issued by various professional organizations to recommend practices in endoscopic procedures. This article aims to summarize and discuss available evidence for these practices, to provide guidance for endoscopy to enhance patient safety, avoid nosocomial outbreaks, protect healthcare personnel, and ensure rational use of personal protective equipment. Responses adapted to national recommendations and local infection control guidelines and tailored to the availability of medical resources are imminently needed to fight the coronavirus disease 2019 pandemic.

Luk, H. N., et al. (2021). "Application of Plastic Sheet Barrier and Video Intubating Stylet to Protect Tracheal Intubators During Coronavirus Disease 2019 Pandemic: A Taiwan Experience." Cell Transplant **30**: 963689720987527.

Since its outbreak in China, the Coronavirus disease 2019 (COVID-19) pandemic has caused worldwide disaster. Globally, there have been 71,581,532 confirmed cases of COVID-19, including 1,618,374 deaths, reported to World Health Organization (data retrieved on December 16, 2020). Currently, no treatment modalities for COVID-19 (e.g., vaccines or antiviral drugs) with confirmed efficacy and safety are available. Although the possibilities and relevant challenges of some alternatives (e.g., use of stem cells as immunomodulators) have been proposed, the personal protective equipment is still the only way to protect and lower infection rates of COVID-19 among healthcare workers and airway managers (intubators). In this article, we described the combined use of a plastic sheet as a barrier with the intubating stylet for tracheal intubation in patients needing mechanical ventilation. Although conventional or video-assisted laryngoscopy is more popular and familiar to other groups around the world, we believe that the video-assisted intubating stylet technique is much easier to learn and master. Advantages of the video stylet include the creation of greater working distance between intubator and patient, less airway stimulation, and less pharyngeal space needed for endotracheal tube advancement. All the above features make this technique reliable and superior to other devices, especially when a difficult airway is encountered in COVID scenario. Meanwhile, we proposed the use of a flexible and transparent plastic sheet to serve as a barrier against aerosol and droplet spread during airway management. We demonstrated that the use of a plastic sheet would not interfere or hinder the intubator's maneuvers during endotracheal intubation. Moreover, we demonstrated that the plastic sheet was effective in preventing the spread of mist and water spray in simulation models with a mannequin. In our experience, we found that this technique most effectively protected the intubator and other operating room personnel from infection during the COVID-19 pandemic.

Lumlertgul, S. and P. C. Inboriboon (2021). "How Clean Are You Really? A Simulation Study of Droplet Splash Using an Acrylic Box During Cardiopulmonary Resuscitation." Simul Healthc **16**(2): 153-154.

In light of the COVID-19 pandemic, protocols to reduce risk of exposure to healthcare teams have been implemented. The use of an acrylic box during intubation and cardiopulmonary resuscitation has been adopted worldwide. A study was conducted to assess the effectiveness of an acrylic box in limiting contamination. The findings indicate that healthcare workers are still vulnerable to exposure despite the use of personal protective equipment (PPE) and an acrylic box. The causes of contamination were related to improper PPE donning and doffing and incomplete coverage by the acrylic box. Institutions should focus on ensuring proper donning and doffing of PPE and incorporating high-fidelity simulation training to prepare teams to resuscitate COVID-19 patients.

Lumley, S. F., et al. (2021). "The duration, dynamics and determinants of SARS-CoV-2 antibody responses in individual healthcare workers." Clin Infect Dis.

BACKGROUND: SARS-CoV-2 IgG antibody measurements can be used to estimate the proportion of a population exposed or infected and may be informative about the risk of future infection. Previous estimates of the duration of antibody responses vary. METHODS: We present 6 months of data from a longitudinal seroprevalence study of 3276 UK healthcare workers (HCWs). Serial measurements of SARS-CoV-2 anti-nucleocapsid and anti-spike IgG were obtained. Interval censored survival analysis was used to investigate the duration of detectable responses. Additionally, Bayesian mixed linear models were used to investigate anti-nucleocapsid waning. RESULTS: Anti-spike IgG levels remained stably detected after a positive result, e.g., in 94% (95% credibility interval, CrI, 91-96%) of HCWs at 180 days. Anti-nucleocapsid IgG levels rose to a peak at 24 (95% credibility interval, CrI 19-31) days post first PCR-positive test, before beginning to fall. Considering 452 anti-nucleocapsid seropositive HCWs over a median of 121 days from their maximum positive IgG titre, the mean estimated antibody half-life was 85 (95%CrI, 81-90) days. Higher maximum observed anti-nucleocapsid titres were associated with longer estimated antibody half-lives. Increasing age, Asian ethnicity and prior self-reported symptoms were independently associated with higher maximum anti-nucleocapsid levels and increasing age and a positive PCR test undertaken for symptoms with longer anti-nucleocapsid half-lives. CONCLUSION: SARS-CoV-2 anti-nucleocapsid antibodies wane within months, and faster in younger adults and those without symptoms. However, anti-spike IgG remains stably detected. Ongoing longitudinal studies are required to track the long-term duration of antibody levels and their association with immunity to SARS-CoV-2 reinfection.

Luo, L. S., et al. (2020). "COVID-19: Presumed Infection Routes and Psychological Impact on Staff in Administrative and Logistics Departments in a Designated Hospital in Wuhan, China." Front Psychol **11**: 1501.

Objective: Our aim was to explore the presumed infection routes and psychological impact of COVID-19 on staff in administrative and logistics departments (ALDs). Methods: We gathered data from all 18 staff members with COVID-19 in ALDs in Zhongnan Hospital of Wuhan University, China. The baseline, job before diagnosis, presumed infection environment, use of protective equipment, and psychological status before and after diagnosis were collected and analyzed. A total of 18 uninfected staff members working alongside them in the same environment and 18 random matched infected doctors and nurses formed two control groups; the psychological impact of these three groups was then compared. Results: Of the 18 members of staff, 88.89% were infected due to the working environment (hospital), and nine had face-to-face conversations with doctors and nurses in their daily work. Many staff members did not take any protective measures in their routine work. Before they were diagnosed, 12 staff members were aware of the seriousness of the epidemic, and most of the staff maintained a neutral attitude to the COVID-19 outbreak. A total of 77.78% of the staff experienced psychological stress or emotional changes after diagnosis, which were mainly caused by family health and disease related issues. Most of them managed their emotions by self-control and video calls with their families. There was no significant difference in psychological impact among the three groups, but uninfected staff members were fully aware of the seriousness of the epidemic. Conclusions: Effective protective measures should be taken for staff members in ALDs. Psychological interventions are very important to help infected staff members in ALDs cope with psychological distress.

Luo, M., et al. (2020). "The psychological and mental impact of coronavirus disease 2019 (COVID-19) on medical staff and general public - A systematic review and meta-analysis." Psychiatry Res **291**: 113190.

The coronavirus disease 2019 (COVID-19) pandemic has caused enormous psychological impact worldwide. We conducted a systematic review and meta-analysis on the psychological and mental impact of COVID-19 among healthcare workers, the general population, and patients with higher COVID-19 risk published between 1 Nov 2019 to 25 May 2020. We conducted literature research using Embase, PubMed, Google scholar and WHO COVID-19 databases. Among the initial search of 9207 studies, 62 studies with 162,639 participants from 17 countries were included in the review. The pooled prevalence of anxiety and depression was 33% (95% confidence interval: 28%-38%) and 28% (23%-32%), respectively. The prevalence of anxiety and depression was the highest among patients with pre-existing conditions and COVID-19 infection (56% [39%-73%] and 55% [48%-62%]), and it was similar between healthcare workers and the general public. Studies from China, Italy, Turkey, Spain and Iran reported higher-than-pooled prevalence among healthcare workers and the general public. Common risk factors included being women, being nurses, having lower socioeconomic status, having high risks of contracting COVID-19, and social isolation. Protective factors included having sufficient medical resources, up-to-date and accurate information, and taking precautionary measures. In conclusion, psychological interventions targeting high-risk populations with heavy psychological distress are in urgent need.

Luo, Y., et al. (2021). "Chinese Expert Consensus on Protection for Ultrasound Healthcare Workers against COVID-19." Ultrasound Med Biol **47**(2): 222-229.

Severe acute respiratory syndrome coronavirus 2 (SARS-CoV-2) infection has caused a worldwide pandemic and poses a serious public health risk. It has been proven that lung ultrasound can be extremely valuable in the diagnosis and treatment of the disease, which could also minimize the number of exposed healthcare workers and equipment. Because healthcare workers in ultrasound departments are in close contact with patients who might be infected or virus carriers, it is extremely important that they be provided sufficient protection. Extremely aggressive protection should be avoided because it might lead to a lack of protection equipment for the hospital. Guidance on proper protection management should be provided in detail, for example, how to choose personal protective equipment, how to disinfect the environment. To address these problems, on behalf of the Chinese Ultrasound Doctors Association, Chinese PLA Professional Committee of Ultrasound in Medicine, Beijing Institute of Ultrasound in Medicine and Chinese Research Hospital Association Ultrasound Professional Committee, the authors have summarized the recommendations for effective protection according to existing hygienic standards, their experience and available literature. After the recommendations were completed, two online conferences were held on January 31, 2020 and February 7, 2020, at which the recommendations were discussed in detail. A modified version of the work was circulated and finally approved by all authors, and is the present Chinese Expert Consensus on Protection for Ultrasound Healthcare Workers against COVID-19.

Luong-Nguyen, M., et al. (2020). "[Nosocomial infection with SARS-Cov-2 within departments of digestive surgery]." J Chir Visc **157**(3): S13-s19.

INTRODUCTION: The COVID-19 pandemic imposed a drastic reduction in surgical activity in order to respond to the influx of hospital patients and to protect uninfected patients by avoiding hospitalization. However, little is known about the risk of infection during hospitalization or its consequences. The aim of this work was to report a series of patients hospitalized on digestive surgery services who developed a nosocomial infection with SARS-Cov-2 virus. METHODS: This is a non-interventional retrospective study carried out within three departments of digestive surgery. The clinical, biological and radiological data of the patients who developed a nosocomial infection with SARS-Cov-2 were collected from the computerized medical record. RESULTS: From March 1, 2020 to April 5, 2020, among 305 patients admitted to digestive surgery departments, 15 (4.9 %) developed evident nosocomial infection with SARS-Cov-2. There were nine men and six women, with a median age of 62 years (35-68 years). All patients had co-morbidities. The reasons for hospitalization were: surgical treatment of cancer (n = 5), complex emergencies (n = 5), treatment of complications linked to cancer or its treatment (n = 3), gastroplasty (n = 1), and stoma closure (n = 1). The median time from admission to diagnosis of SARS-Cov-2 infection was 34 days (5-61 days). In 12 patients (80%), the diagnosis was made after a hospital stay of more than 14 days (15-63 days). At the end of the follow-up, two patients had died, seven were still hospitalized with two of them on respiratory assistance, and six patients were discharged post-hospitalization. CONCLUSIONS: The risk of SARS-Cov-2 infection during hospitalization or following digestive surgery is a real and potentially serious risk. Measures are necessary to minimize this risk in order to return to safe surgical activity.

Publisher: Abstract available from the publisher.

fre

Luong-Nguyen, M., et al. (2020). "Nosocomial infection with SARS-Cov-2 within Departments of Digestive Surgery." J Visc Surg **157**(3s1): S13-s18.

INTRODUCTION: The COVID-19 pandemic imposed a drastic reduction in surgical activity in order to respond to the influx of hospital patients and to protect uninfected patients by avoiding hospitalization. However, little is known about the risk of infection during hospitalization or its consequences. The aim of this work was to report a series of patients hospitalized on digestive surgery services who developed a nosocomial infection with SARS-Cov-2 virus. METHODS: This is a non-interventional retrospective study carried out within three departments of digestive surgery. The clinical, biological and radiological data of the patients who developed a nosocomial infection with SARS-Cov-2 were collected from the computerized medical record. RESULTS: From March 1, 2020 to April 5, 2020, among 305 patients admitted to digestive surgery departments, 15 (4.9%) developed evident nosocomial infection with SARS-Cov-2. There were nine men and six women, with a median age of 62 years (35-68 years). All patients had co-morbidities. The reasons for hospitalization were: surgical treatment of cancer (n=5), complex emergencies (n=5), treatment of complications linked to cancer or its treatment (n=3), gastroplasty (n=1), and stoma closure (n=1). The median time from admission to diagnosis of SARS-Cov-2 infection was 34 days (5-61 days). In 12 patients (80%), the diagnosis was made after a hospital stay of more than 14 days (15-63 days). At the end of the follow-up, two patients had died, seven were still hospitalized with two of them on respiratory assistance, and six patients were discharged post-hospitalization. CONCLUSIONS: The risk of SARS-Cov-2 infection during hospitalization or following digestive surgery is a real and potentially serious risk. Measures are necessary to minimize this risk in order to return to safe surgical activity.

Luvira, V., et al. (2020). "Temporal Change of SARS-CoV-2 in Clinical Specimens of COVID-19 Pneumonia Patients." Am J Trop Med Hyg **103**(3): 1204-1206.

The quality and type of specimen collection affect the sensitivity of real-time reverse transcriptase-PCR (rRT-PCR) for the diagnosis of SARS-CoV-2. In this report, the course over time of rRT-PCR for SARS-CoV-2 in 26 clinical specimens collected from the upper (nasopharyngeal and throat swabs) and lower (sputum) respiratory tracts of COVID-19 cases with pneumonia was investigated along with the clinical course. The preliminary results revealed that higher SARS-CoV-2 RNA concentration and longer time for detection make self-collected sputum a preferable specimen for the diagnosis and follow-up of COVID-19 pneumonia. Self-collection of sputum can minimize the risk of unnecessary exposure to healthcare workers, preserve the shortage of personal protective equipment, and limit viral transmission to the environment.

Luze, H., et al. (2020). "Personal protective equipment in the COVID-19 pandemic and the use of cooling-wear as alleviator of thermal stress : A pilot study in plastic surgery staff members." Wien Klin Wochenschr: 1-9.

BACKGROUND: High temperatures at workplaces lead to health-related risks and premature exhaustion. The coronavirus disease 2019 (COVID-19) pandemic requires many health professionals to perform under unfavorable conditions. Personal protective equipment (PPE) causes thermal stress and negatively affects performance. PATIENTS, MATERIALS AND METHODS: This pilot project investigated the effects of PPE and additional cooling wear on physiological parameters and concentration of six healthy staff members of the Plastic Surgery Department of the Medical University of Graz, Austria during simulated patient care. In this study two 1‑hour cycles with patient care-related tasks with PPE and PPE + cooling-wear, respectively, were conducted. A third cycle with scrubs exclusively served as baseline/negative control. The assessment occurred immediately pre-cycles and post-cycles. RESULTS: Pre-cycle assessments showed no significant differences between the cycles. After PPE cycle, increased physical stress levels and decrements in concentration capacity were observed. Physiological parameters were significantly less affected in the cooling cycle, while concentration capacity slightly increased. CONCLUSION: COVID-19 PPE causes considerable thermal stress, ultimately affecting human performance. As opportunity to withstand thermal stress, and improve patients' and professionals' safety, cooling-wear can be considered relevant. Medical personnel performing in exceptional situations may particularly benefit from further development and investigation of cooling strategies.

Lyons, A. B., et al. (2021). "Skin and eye protection against ultraviolet C from ultraviolet germicidal irradiation devices during the COVID-19 pandemic." Int J Dermatol **60**(4): 391-393.

With the COVID-19 pandemic depleting personal protective equipment worldwide, various methods including ultraviolet C (UVC) germicidal irradiation (UVGI) have been implemented to decontaminate N95 filtering facepiece respirators. These devices pose a risk for UVC exposure to the operator with reported adverse effects generally limited to the eyes and skin. Our hospitals are currently using UVC devices for N95 decontamination with a few reported cases of face and neck erythema from exposure. Because sunscreens are designed and tested for UVA and UVB protection only, their effects on blocking UVC are largely unknown. Therefore, our objective was to determine if various sunscreens, UV goggles, and surgical mask face shields minimize UVC exposure from UVGI devices. Our study clearly demonstrated that healthcare workers responsible for the disinfection of PPE using UVGI devices should always at least utilize clear face shields or UV goggles and sunscreen to protect against side effects of UVC exposure.

Ma, L., et al. (2020). "[Management protocol and outcome assessment of ruptured intracranial aneurysm emergency surgery during coronavirus disease 2019 pandemic]." Zhonghua Yi Xue Za Zhi **100**(47): 3768-3774.

Objective: To explore the improvement of emergency admission screening and perioperative management protocols in the scenario of the coronavirus disease 2019 (COVID-19) pandemic and its regular prevention and control for patients with ruptured intracranial aneurysms, which are the most common emergency cases with the most urgent needs for emergent surgery. Methods: The response protocol of the emergency surgical management of ruptured intracranial aneurysm during the epidemic period (from January to March, 2020) at Beijing Tiantan Hospital, Capital Medical University was reviewed. The prognosis of emergent aneurysm surgery under different levels of protection or during the same period of 2019 and 2020 was further compared to describe the operation and prognosis under the new management protocol. Results: A total of 127 emergency cases with aneurysmal subarachnoid hemorrhage were referred to Beijing Tiantan Hospital, Capital Medical University from January 20 to March 25, 2020, and 42 cases(33.1%) underwent emergent aneurysm clipping after multi-desciplinary consultation. Admissions of emergency cases required epidemiological, laboratory, and imaging screenings for COVID-19, with additional throat swab virus nucleic acid screening afterwards. During the same period, 9 cases of COVID-19 were confirmed in the emergency screening, and no false negative cases were found. Compared with the same period in 2019, the interval between emergency visits and emergency craniotomy did not increase significantly due to the preoperative screening ((37±17) hours during the epidemic period versus (29±12) hours at the same period in 2019, P=0.058). There was no significant difference in the incidence of perioperative adverse events and postoperative neurological dysfunction (P=0.779). According to the screening results, the corresponding operative and postoperative management protocol and protection standards were adopted. There was no significant difference in the prognosis of emergent surgery between patients with a negative initial screening and those who were to be excluded or suspected in the initial screening (P=0.678). Although viral nucleic acid screening tended to prolong the time interval before surgical intervention ((36±15) hours before nucleic acid screening versus (40±20) hours after nucleic acid screening, P=0.453), there was no statistically significant difference in the preoperative adverse events and postoperative neurological function (P=0.653). Conclusion: The current protocol of COVID-19 screening and stratified emergent surgery management based on screening results can effectively identify suspected and confirmed COVID-19 cases, thereby ensuring timely, safe and effective emergent surgery and prohibiting nosocomial spread.

Ma, Y., et al. (2020). "[Perioperative infection prevention strategies for double-lung transplantation in elderly patients with COVID-19]." Zhejiang Da Xue Xue Bao Yi Xue Ban **49**(5): 618-622.

OBJECTIVE: To summarize the experience of perioperative prevention during double-lung transplantation for elderly patients with coronavirus disease 2019 (COVID-19). METHODS: Clinical data of 2 elderly patients with COVID-19 who underwent double-lung transplantation in the First Affiliated Hospital of Zhejiang University School of Medicine in March 2020 were retrospectively reviewed. Perioperative protective measures were introduced in terms of medical staffing, respiratory tract, pressure injuries, air in operating room, instruments and equipment, pathological specimens, and information management. RESULTS: Two cases of double-lung transplantation were successfully completed, and the patients had no operation-related complications. Extracorporeal membrane oxygenator was successfully removed 2 to 4 days after surgery and the patients recovered well. There was no infection among medical staff. CONCLUSIONS: Adequate preoperative preparation, complete patient transfer procedures, proper placement of instruments and equipment, strengthening of intraoperative care management, and attention to prevention of pressure injury complications can maximize the safety of COVID-19 patients and medical staff.

MacInnes, E. G., et al. (2020). "Breast Cancer Surgery During the COVID-19 Pandemic Peak in the UK: Operative Outcomes." Cureus **12**(7): e9280.

Introduction  The COVID-19 pandemic caused widespread changes in delivery of breast cancer care, aiming to protect vulnerable patients whilst minimising compromise to oncological outcomes. This multicentre observational study aimed to establish early surgical outcomes from breast cancer surgery performed during the peak of the COVID-19 pandemic.  Materials and methods  Data were collected on consecutive patients that underwent breast surgery in four units between 16 March and 24 April 2020. Outcome data at 30 days post-operation were collected, including documented COVID-19 cases in patients and reported cases in healthcare workers directly involved in their care. Recommended modifications to practice to reduce COVID-19 transmission risk, both to patients and healthcare workers in each centre, are described.  Results  A total of 202 patients underwent surgery in four hospitals delivering breast services in the West Yorkshire region over the six-week period at the peak of the pandemic. The age ranged from 28 to 91 years (median 57, interquartile range, 48-65) with 22% having co-morbidities linked to COVID-19, e.g. diabetes or respiratory disease. No patients presented post-operatively with COVID-19 symptoms and at 30 days there had not been any identified COVID-19 cases. There were no unexpected critical care admissions or deaths. One healthcare worker involved in the delivery of breast surgery was diagnosed with COVID-19 during this time and made an uneventful recovery.  Conclusion  Breast cancer surgery, in selected groups and with meticulous adherence to measures designed to reduce COVID-19 transmission, does not appear to be associated with elevated risk to patients or healthcare workers.

MacIntyre, C. R. and A. A. Chughtai (2020). "A rapid systematic review of the efficacy of face masks and respirators against coronaviruses and other respiratory transmissible viruses for the community, healthcare workers and sick patients." Int J Nurs Stud **108**: 103629.

BACKGROUND: The pandemic of COVID-19 is growing, and a shortage of masks and respirators has been reported globally. Policies of health organizations for healthcare workers are inconsistent, with a change in policy in the US for universal face mask use. The aim of this study was to review the evidence around the efficacy of masks and respirators for healthcare workers, sick patients and the general public. METHODS: A systematic review of randomized controlled clinical trials on use of respiratory protection by healthcare workers, sick patients and community members was conducted. Articles were searched on Medline and Embase using key search terms. RESULTS: A total of 19 randomised controlled trials were included in this study - 8 in community settings, 6 in healthcare settings and 5 as source control. Most of these randomised controlled trials used different interventions and outcome measures. In the community, masks appeared to be effective with and without hand hygiene, and both together are more protective. Randomised controlled trials in health care workers showed that respirators, if worn continually during a shift, were effective but not if worn intermittently. Medical masks were not effective, and cloth masks even less effective. When used by sick patients randomised controlled trials suggested protection of well contacts. CONCLUSION: The study suggests that community mask use by well people could be beneficial, particularly for COVID-19, where transmission may be pre-symptomatic. The studies of masks as source control also suggest a benefit, and may be important during the COVID-19 pandemic in universal community face mask use as well as in health care settings. Trials in healthcare workers support the use of respirators continuously during a shift. This may prevent health worker infections and deaths from COVID-19, as aerosolisation in the hospital setting has been documented.

MacIntyre, C. R., et al. (2020). "Contamination and washing of cloth masks and risk of infection among hospital health workers in Vietnam: a post hoc analysis of a randomised controlled trial." BMJ Open **10**(9): e042045.

BACKGROUND: In a previous randomised controlled trial (RCT) in hospital healthcare workers (HCWs), cloth masks resulted in a higher risk of respiratory infections compared with medical masks. This was the only published RCT of cloth masks at the time of the COVID-19 pandemic. OBJECTIVE: To do a post hoc analysis of unpublished data on mask washing and mask contamination from the original RCT to further understand poor performance of the two-layered cotton cloth mask used by HCWs in that RCT. SETTING: 14 secondary-level/tertiary-level hospitals in Hanoi, Vietnam. PARTICIPANTS: A subgroup of 607 HCWs aged ≥18 years working full time in selected high-risk wards, who used a two-layered cloth mask and were part of a randomised controlled clinical trial comparing medical masks and cloth masks. INTERVENTION: Washing method for cloth masks (self-washing or hospital laundry). A substudy of contamination of a sample of 15 cloth and medical masks was also conducted. OUTCOME MEASURE: Infection rate over 4 weeks of follow up and viral contamination of masks tested by multiplex PCR. RESULTS: Viral contamination with rhinovirus was identified on both used medical and cloth masks. Most HCW (77% of daily washing) self-washed their masks by hand. The risk of infection was more than double among HCW self-washing their masks compared with the hospital laundry (HR 2.04 (95% CI 1.03 to 4.00); p=0.04). There was no significant difference in infection between HCW who wore cloth masks washed in the hospital laundry compared with medical masks (p=0.5). CONCLUSIONS: Using self-reported method of washing, we showed double the risk of infection with seasonal respiratory viruses if masks were self-washed by hand by HCWs. The majority of HCWs in the study reported hand-washing their mask themselves. This could explain the poor performance of two layered cloth masks, if the self-washing was inadequate. Cloth masks washed in the hospital laundry were as protective as medical masks. Both cloth and medical masks were contaminated, but only cloth masks were reused in the study, reiterating the importance of daily washing of reusable cloth masks using proper method. A well-washed cloth mask can be as protective as a medical mask. TRIAL RESGISTRATION NUMBER: ACTRN12610000887077.

MacKenzie, M., et al. (2021). "The Impact of the Coronavirus Disease 2019 (COVID-19) Pandemic on Healthcare Workers with Pre-Existing Psychiatric Conditions." Psychiatr Q: 1-10.

Both healthcare workers (HCWs) and psychiatric patients during the COVID-19 pandemic appear to have elevated prevalence of psychiatric symptoms, but little is known about HCWs with psychiatric diagnoses. To examine their response to the pandemic, we analyzed their perspective, and association with psychiatric symptoms and stress among HCW with psychiatric diagnosis. Using an online survey of HCW, we analyzed demographics, work information, health factors, open-ended question, sources of stress and standardized mental health scales (Perceived Stress Scale (PSS), Generalized Anxiety Disorder-7 (GAD-7), Patient Health Questionnaire-8 (PHQ-8), and Posttraumatic Stress Disorder Checklist (PCL)). Sixteen out of 129 HCWs reported a pre-existing psychiatric diagnosis (median age 32 years, 8 were females, 8 work in the emergency department). Their perception of the impact severity on symptoms was significantly correlated with all the mental health scales and with stress from avoiding physical contact. In multivariate analysis, PSS score and PCL score were associated with self-rated impact of the COVID-19 pandemic on symptoms (standardized beta = .51 for PCL and standardized beta = .55 for PSS). GAD-7 score was significantly related to both impact (standardized beta = .44) and stress from avoiding physical contact (standardized beta = .53). HCWs with psychiatric diagnoses reported a range of perception of the impact of the pandemic on their symptoms with increased severity associated with worse psychiatric outcomes and more stress from avoiding physical contact with others. There is a growing importance to protect HCWs mental health, including those with pre-existing psychiatric diagnosis, and proactively counter psychosocial consequences of healthcare crises.

Mahmood, S. U., et al. (2020). "Strategies for Rational Use of Personal Protective Equipment (PPE) Among Healthcare Providers During the COVID-19 Crisis." Cureus **12**(5): e8248.

As the coronavirus 2019 (COVID-19) began spreading globally with no clear treatment in sight, prevention became a major part of controlling the disease and its effects. COVID-19 spreads from the aerosols of an infected individual whether they are showing any symptoms or not. Therefore, it becomes nearly impossible to point exactly where the patient is. This is where personal protective equipment (PPE) comes in. These are masks, respirators, gloves, and in hospitals where the contact with the infected and confirmed patient is direct, also gowns or body covers. The PPEs play a major role in the prevention and control of the COVID-19. The PPE is able to prevent any invasion of the virus particles into the system of an individual which is why it is an essential item to have for healthcare workers. Due to the high demand for PPEs all around the world, it is important to optimize the use of protective gear and ration the supplies so that the demand are met. However, there are guidelines recommended by the World Health Organization (WHO) and the Centers for Disease Control and Prevention (CDC) to maintain the supply in the wake of this increased demand of PPE, how the manufacturers should track their supplies, and how the recipients should manage them. Various strategies can be used to increase the re-use of PPEs during the COVID-19 pandemic that has modified the donning and doffing procedure.

Mahto, M., et al. (2021). "Seroprevalence of IgG against SARS-CoV-2 and its determinants among healthcare workers of a COVID-19 dedicated hospital of India." Am J Blood Res **11**(1): 44-52.

Healthcare workers (HCWs) due to their job profile are at utmost risk of contracting severe acute respiratory syndrome coronavirus-2 (SARS-CoV-2) infection. Serological survey is an useful tool for vulnerability mapping in an infectious disease pandemic. The aim of the current study was to assess seroprevalence of IgG against SARS-CoV-2 and its determinants among HCWs of a tertiary healthcare facility of India. It was an observational study, cross-sectional in design conducted among 919 HCWs of All India Institute of Medical Sciences, Patna, Bihar, India during September, 2020. In results, IgG seroprevalence for SARS-CoV-2 among the study subjects was 13.3% [95% confidence interval (CI): 11.2-15.6%]. In univariate logistic regression analysis; gender, occupation, place of posting, use of full personal protective equipment (PPE), prior corona virus disease (COVID)-19 infection, influenza like illness (ILI), use of steam inhalation, consumption of azithromycin, zinc and vitamin C were the significant attributes which affected the IgG seropositivity for SARS-CoV-2. In the multivariable logistic regression model; occupation, place of posting, prior COVID-19 infection and ILI were significant determinants of IgG seropositivity for SARS-CoV-2. To conclude, majority of the HCWs were found to be IgG seronegative for SARS-CoV-2. Till availability of effective vaccine all of the HCWs should abide by infection prevention and control (IPC) measures to keep themselves and their contacts protected from SARS-CoV-2.

Maida, M., et al. (2020). "Impact of the COVID-19 pandemic on Gastroenterology Divisions in Italy: A national survey." Dig Liver Dis **52**(8): 808-815.

BACKGROUND: In Italy, the spread of the COVID-19 pandemic has stressed the entire healthcare system and required a huge re-organization of many Divisions, including those of Gastroenterology. AIMS: to assess the impact of COVID-19 pandemic on Gastroenterology Divisions across Italy. METHODS: All members of the Italian Society of Gastroenterology (SIGE) were invited to answer a web-based survey. RESULTS: Data of 121 hospitals from all 20 Italian regions were analyzed. Overall, 10.7% Gastroenterology Divisions have been converted to Covid Units. Outpatients consultations, endoscopic and ultrasound procedures were limited to urgencies and oncology indications in 85.1%, 96.2% and 72.2% of Units, respectively, and 46.7% of them suspended the screening for colorectal cancer. Moreover, 72.2% of the staff received a training for use of personal protective equipment, although 45.5% did not have sufficient devices for adequate replacement. Overall, 132 healthcare workers in 41 Gastroenterology Divisions were found to be infected. CONCLUSION: This is the first study to evaluate, at a country level, the impact of COVID-19 outbreak on Gastroenterology Divisions. Substantial changes of practice and reduction of procedures have been recorded in the entire country. The long-term impact of such modifications is difficult to estimate but potentially very risky for many digestive diseases.

Majumder, M. A. A., et al. (2020). "Antimicrobial Stewardship: Fighting Antimicrobial Resistance and Protecting Global Public Health." Infect Drug Resist **13**: 4713-4738.

Antimicrobial resistance (AMR) is a serious threat to global public health. It increases morbidity and mortality, and is associated with high economic costs due to its health care burden. Infections with multidrug-resistant (MDR) bacteria also have substantial implications on clinical and economic outcomes. Moreover, increased indiscriminate use of antibiotics during the COVID-19 pandemic will heighten bacterial resistance and ultimately lead to more deaths. This review highlights AMR's scale and consequences, the importance, and implications of an antimicrobial stewardship program (ASP) to fight resistance and protect global health. Antimicrobial stewardship (AMS), an organizational or system-wide health-care strategy, is designed to promote, improve, monitor, and evaluate the rational use of antimicrobials to preserve their future effectiveness, along with the promotion and protection of public health. ASP has been very successful in promoting antimicrobials' appropriate use by implementing evidence-based interventions. The "One Health" approach, a holistic and multisectoral approach, is also needed to address AMR's rising threat. AMS practices, principles, and interventions are critical steps towards containing and mitigating AMR. Evidence-based policies must guide the "One Health" approach, vaccination protocols, health professionals' education, and the public's awareness about AMR.

Mak, S. T. and H. K. Yuen (2020). "Oculoplastic surgery practice during the COVID-19 novel coronavirus pandemic: experience sharing from Hong Kong." Orbit **39**(4): 316-318.

At present, all parts of the world are hit hard by COVID-19. The first confirmed case of COVID-19 in the territory of Hong Kong was announced on January 23, 2020. Since then, oculoplastic surgeons in Hong Kong have been taking every measure to protect all healthcare workers and patients from contracting the disease. This paper aims to share the experiences of and measures taken by local oculoplastic surgeons in combating COVID-19. Three main aspects are discussed, namely clinical, administrative, and training and educational. We hope our experiences would provide reference to fellow oculoplastic colleagues in other parts of the world in fighting this COVID-19 pandemic.

Malik, F. T., et al. (2020). "Clinical Presentation, Management and In-Hospital Outcome of Healthcare Personnel With COVID-19 Disease." Cureus **12**(8): e10004.

Objective Healthcare personnel (HCP) are undoubtedly one of the major frontline fighters in the coronavirus disease 2019 (COVID-19) pandemic. Therefore, it comes as no surprise that many HCP have become infected by COVID-19 globally. The infection of HCP has received great attention in social media and is frequently reported from different parts of the world. However, there are few scientific reports addressing this aspect of the COVID-19 pandemic. The aim of this study was to evaluate the characteristics of clinical presentation, treatment, and outcome of COVID-19 infection among the HCP of our setting. Methods This cross-sectional study was performed in the National Heart Foundation Hospital & Research Institute of Bangladesh from April 29 to July 20, 2020. HCP employed in this hospital who experienced fever or respiratory symptoms or came in close contact with COVID-19 patients at home or their workplace were included in this study. The presence of COVID-19 disease was confirmed by real-time reverse transcriptase-polymerase chain reaction on nasopharyngeal samples. A total of 394 HCP were sampled and 139 had a positive corona test. Structured interviews were conducted to document symptoms for all HCP with confirmed COVID-19. Data analysis was performed in July 2020. Results Out of 1,409 HCP, 139 subjects tested positive for COVID-19. Among the HCP, infection rate was 9.86%. The mean age of the study population was 34.08±11.11 years (range: 20-69 yrs), of whom 82 (59%) were female. Most of this cohort were nurses (56 [40.3%]) and physicians (25 [18%]), and the remaining 58 (41.7%) were other staff. The mean duration of onset of symptoms to test was 2.89±2.07 days. The most common symptoms were fever (84.2%), fatigue (56.1%), cough (54%), body ache (39.6%), headache, and anosmia (38.8%). Most subjects had mild disease (125 [93%]), three (2.1%) of the HCP had moderate disease and one (0.7%) had severe disease. Ten of the HCP (7.2%) were asymptomatic. Most of them were treated either by ivermectin plus azithromycin or ivermectin plus doxycycline. Only 20 (14.4%) of the HCP were hospitalized, while others were treated either in home isolation (59.7%) or in institutional isolation (25.9%). Recovery was almost uneventful except one healthcare worker who died. Conclusion Most HCP had mild symptoms and a few of them were asymptomatic also. HCP with mild COVID-19 symptoms may be treated in home or institutional isolation. As they are a vulnerable group for infection, providing adequate protection to HCP is absolutely mandatory to safeguard them from this pandemic.

Malik, J. S., et al. (2020). "Maximising application of the aerosol box in protecting healthcare workers during the COVID-19 pandemic." Anaesthesia **75**(7): 974-975.

Maltezou, H. C., et al. (2021). "Hospital factors associated with SARS-CoV-2 infection among healthcare personnel in Greece." J Hosp Infect **109**: 40-43.

Healthcare workers (HCWs) have been recognized as a high-risk group for infection with severe acute respiratory syndrome coronavirus-2 (SARS-CoV-2). This study estimated their risk of infection based on hospital characteristics. Factors significantly associated with increased risk for SARS-CoV-2 infection were: working in a non-referral hospital compared with a coronavirus disease 2019 (COVID-19) referral hospital, working in a hospital with a high number of employees, and working in a hospital with an increased number of patients with COVID-19. This study revealed gaps in infection control in the non-referral hospitals. There is an urgent need for continuous training in infection control practices. Compliance of HCWs with the use of personal protective equipment should also be addressed.

Manaye, G., et al. (2021). "Evaluation of the Efficacy of Alcohol-Based Hand Sanitizers Sold in Southwest Ethiopia." Infect Drug Resist **14**: 547-554.

BACKGROUND: Regarding the protection of community and health professionals suffering from a COVID-19 outbreak, currently different alcohol-based hand sanitizers have been distributed. Even though for effective protection effective alcohol-based hand sanitizers are mandatory. Their efficacy was not evaluated. This is the reason why this research was designed to assess the antimicrobial efficacy of hand sanitizers that have been sold in southern parts of Ethiopia. METHODS: Six test organisms (Staphylococcus aureus, Escherichia coli, Pseudomonas aeruginosa, Klebsiella pneumoniae, Salmonella typhimurium and Shigella boydii) were selected from different clinical specimens. Then seven locally made products of alcohol-based hand sanitizers (MTU, Folium Fine, Epharm, Harego, Taflen and Sheba) were purchased and the disc diffusion, minimum inhibition concentration and minimum bactericidal concentration test were done against selected test organisms. Quality control measures throughout the whole process of the laboratory work were implemented and descriptive parameters were analyzed. RESULTS: The zones of inhibition of the hand sanitizers at their claim concentration were ranged from noninhibition zone (Folium) to 27mm (Sheba). The minimum inhibitory concentration against all selected test organisms was observed at 45%, 55%, 65% and Taflen on undiluted form. The growth of test organisms was decreased across increasing the concentration gradient of different hand sanitizers. MTU except against E. coli, Fine, Hargo, Ephra and Sheba hand sanitizers were showed growth below 60% concentrations for all test micro-organisms. But a Folium product against all selected test micro-organisms and MTU product against E. coli were not bactericidal. CONCLUSION: Fine, Hargo, Ephra, Sheba and Taflen sanitizers were the products that were effective in inhibiting the growth of all the selected test organisms, and they were having effective bactericidal activity in vitro at their claim concentration. But MTU product against E. coli and Folium product against all test microorganisms were not having an effective bactericidal activity. So, regulatory authorities and manufacturers should implement strict quality control measures and regular observations throughout the production to ensure the efficacy of hand sanitizers.

Manh Than, H., et al. (2020). "Mental Health and Health-Related Quality-of-Life Outcomes Among Frontline Health Workers During the Peak of COVID-19 Outbreak in Vietnam: A Cross-Sectional Study." Risk Manag Healthc Policy **13**: 2927-2936.

PURPOSE: Mental health is an important component of the protection strategy for healthcare workers (HCWs). However, it has not been well described in Vietnam during the COVID-19 outbreak. This study aims to measure the psychological distress and health-related quality-of-life among frontline healthcare workers during the peak of the outbreak in Vietnam. PATIENTS AND METHODS: We conducted a cross-sectional survey on 173 health workers at two national tertiary hospitals in Hanoi, Vietnam from March to April 2020. The psychological distress was measured by the Depression, Anxiety, and Stress Scale - 21 Items (DASS-21), Impact of Event Scale - Revised (IES-R), and the Insomnia Severity Index (ISI). EQ-5D-5L was used to determine the health-related quality-of-life (HRQoL) outcomes. RESULTS: Among 173 HCWs, the proportion of reported depression symptoms, anxiety symptoms, and stress was 20.2%, 33.5%, and 12.7%, respectively. The median EQ-5D-5L index score was 0.93 (IQR=0.85-0.94), and the anxiety/depression aspect had the highest reported problems. The most COVID-19-specific concerns among frontline HCWs were the reduction of income (59%) and the increase of living costs (54.3%). HCWs working in the COVID-19-designated hospital had a significantly higher rate of mental health problems and had a lower HRQoL outcome than those working in non-COVID-19-designated hospitals. Other factors associated with psychological distress and sleep problems include age, job title, income, chronic diseases status, and years of working in healthcare settings. HCWs who were ≥30 years old, had higher working years, had higher incomes, and had mental health and sleep problems were more likely to have lower HRQoL scores. CONCLUSION: We reported a moderate rate of psychological distress and lower HRQoL outcomes among frontline HCWs during the COVID-19 outbreak in Vietnam. Various factors were found to be associated with mental health and HRQoL that might be useful for implementing appropriate interventions for HCWs in low-resource settings.

Maniaci, A., et al. (2021). "Comfort rules for face masks among healthcare workers during COVID-19 spread." Ann Ig.

BACKGROUND: The outbreak of Coronavirus disease 2019 (COVID-19) made imperative the use of protective devices as a source control tool. As there is no definite antiviral treatment and effective vaccine, the only efficient means of protecting and mitigating infectious contagion has been the use of personal protective equipment, especially by healthcare workers. However, masks affect the humidification process of inhaled air, possibly leading to a basal inflammatory state of the upper airways. STUDY DESIGN: This is a single-center observational study conducted at the University Hospital of Catania from April 1, 2020, to June 31, 2020. METHODS: We analyzed the role of protective masks on the elimination of upper airways complaints in healthcare workers of the University Hospital of Catania. We evaluated 277 subjects through a selfadministered 17 item questionnaire based on respiratory, work performance and health-related quality of life domains. RESULTS: A higher prevalence of nasal and ocular symptoms, perceived reduced work performance, difficulty in concentrating, and sleep disorders were found. After two weeks adhering to a list of good practices that we recommended, significant reversibility of the symptoms investigated and work performance enhancement were observed. CONCLUSIONS: Despite clinical complaints related to personal protective equipment, effective amelioration through usage rules is easily obtained. Given the essential use of protective masks, healthcare workers have to adhere to appropriate work and safety prevention rules.

Manisty, C., et al. (2021). "Time series analysis and mechanistic modelling of heterogeneity and sero-reversion in antibody responses to mild SARS‑CoV-2 infection." EBioMedicine **65**: 103259.

BACKGROUND: SARS-CoV-2 serology is used to identify prior infection at individual and at population level. Extended longitudinal studies with multi-timepoint sampling to evaluate dynamic changes in antibody levels are required to identify the time horizon in which these applications of serology are valid, and to explore the longevity of protective humoral immunity. METHODS: Healthcare workers were recruited to a prospective cohort study from the first SARS-CoV-2 epidemic peak in London, undergoing weekly symptom screen, viral PCR and blood sampling over 16-21 weeks. Serological analysis (n =12,990) was performed using semi-quantitative Euroimmun IgG to viral spike S1 domain and Roche total antibody to viral nucleocapsid protein (NP) assays. Comparisons were made to pseudovirus neutralizing antibody measurements. FINDINGS: A total of 157/729 (21.5%) participants developed positive SARS-CoV-2 serology by one or other assay, of whom 31.0% were asymptomatic and there were no deaths. Peak Euroimmun anti-S1 and Roche anti-NP measurements correlated (r = 0.57, p<0.0001) but only anti-S1 measurements correlated with near-contemporary pseudovirus neutralising antibody titres (measured at 16-18 weeks, r = 0.57, p<0.0001). By 21 weeks' follow-up, 31/143 (21.7%) anti-S1 and 6/150 (4.0%) anti-NP measurements reverted to negative. Mathematical modelling revealed faster clearance of anti-S1 compared to anti-NP (median half-life of 2.5 weeks versus 4.0 weeks), earlier transition to lower levels of antibody production (median of 8 versus 13 weeks), and greater reductions in relative antibody production rate after the transition (median of 35% versus 50%). INTERPRETATION: Mild SARS-CoV-2 infection is associated with heterogeneous serological responses in Euroimmun anti-S1 and Roche anti-NP assays. Anti-S1 responses showed faster rates of clearance, more rapid transition from high to low level production rate and greater reduction in production rate after this transition. In mild infection, anti-S1 serology alone may underestimate incident infections. The mechanisms that underpin faster clearance and lower rates of sustained anti-S1 production may impact on the longevity of humoral immunity. FUNDING: Charitable donations via Barts Charity, Wellcome Trust, NIHR.

Mann, A., et al. (2020). "Considerations for Pregnant Dental and Health Care Workers amid COVID-19." JDR Clin Trans Res **5**(4): 300-306.

INTRODUCTION: Coronavirus disease 2019 (COVID-19) is a highly contagious disease that quickly reached pandemic levels. Over 5 million COVID-19 cases and approximately 330,000 deaths have been recorded worldwide. Transmission is primarily spread through direct, indirect (through contaminated objects or surfaces), or close contact with infected people via respiratory droplets, the mouth, and/or nose secretions. Health care professionals (HCPs), including dental HCPs, are recognized to be at considerably high risk for infection due to the close proximity to patients and aerosol-generating procedures. During pregnancy, HCPs may be at even higher risk since pregnancy substantially increases the susceptibility to infectious diseases. OBJECTIVES: Here, we present the posed risks and potential effects of COVID-19 on maternal and fetal health. Current prevention and management strategies for COVID-19 on pregnant dental and HCPs are also discussed. RESULTS: Significant progress is being made in understanding the pathogenesis and clinical consequences of COVID-19. Pregnant women are affected more adversely with viral illnesses, although evidence of vertical transmission of COVID-19 is controversial. Based on the presence of atypical symptoms, the significant numbers of asymptomatic individuals who are COVID-19 positive, and the high susceptibility to viral diseases observed in pregnant women, recommendations have been put forth to limit the exposure of COVID-19-positive or even suspected cases to pregnant HCPs, and these are likely to evolve as new information becomes available. CONCLUSION: Pregnant HCPs require extra caution: not only are they considered a high-risk population, but their work at the frontline in a pandemic may expose them to additional risks. Complete awareness of the effects of COVID-19 on maternal and fetal/infant health, as well as prevention and management guidelines for pregnant HCPs, will allow for a safer work environment. Health care institutional policies aimed at protecting pregnant HCPs should consider avoiding their assignment as first responders, especially if equally trained staff are available. KNOWLEDGE TRANSFER STATEMENT: Dental and health care professionals can use the information in this review to improve their awareness of COVID-19 risks, signs, and symptoms and the associated effects on the health of pregnant health care professionals and their unborn/newborn children.

Manomaipiboon, A., et al. (2020). "The new silicone elastometric half-piece respirator, VJR-NMU: A novel and effective tool to prevent COVID-19." PLoS One **15**(12): e0237206.

Filter facepiece respirators (FFRs) are critical for preventing the transmission of respiratory tract infection disease, especially the dreadful coronavirus 2 (SARs-CoV-2). The N95 mask is a prototype, high-efficiency protective device that can effectively protect against airborne pathogens of less than 0.3 μm. The N95 mask is tightly fitting and has high filtration capacity. The ongoing COVID-19 pandemic has led to a greater requirement for FFR. This rising demand greatly exceeds current production capabilities and stockpiles, resulting in shortages. To address this, our team has invented a new type of half-piece respirator made from silicone and assembled with HEPA or elastostatic filter. A variety of methods have been used to evaluate this new device, including a qualitative fit test with the Bitrex® test kit and filtration test. The preliminary results showed that the new elastometric respirators pass the fit test. The filtration tests also confirmed the superiority of the new respirator over traditional N95 masks, with a mean performance of protection greater than 95%. For the filters, we used two types: SafeStar, which is a kind of HEPA filter; and CareStar, which is considered an elastostatic filler. CareStar was developed to filter virus and bacteria in the operating room, with a limit duration of use up to 24 h, while the safe star was designed for 72 h use and has the quality equivalent to a HEPA filter. Our study demonstrated superior filtration efficacy of both filters, more than 98% even after 24 h of use. CareStar has significantly more filtration efficacy than a safe star (p < 0.001). In conclusion, the development of our new N99 half-piece respirator should ultimately be applicable to healthcare workers with at least non-inferiority to the previously used N 95 respirators. As a universal masking policy is generally implemented, health care workers who are at risk must be protected with appropriate devices. Currently, the adequate supply of such equipment is not feasible. The advent of the new protective device will help protect healthcare workers and replenish the shortage of N95 respirators during the COVID-19 pandemic.

Maple, P. A. C. and K. Sikora (2021). "How Useful is COVID-19 Antibody Testing - A Current Assessment for Oncologists." Clin Oncol (R Coll Radiol) **33**(1): e73-e81.

The coronavirus disease 2019 (COVID-19) pandemic due to infection by a new human coronavirus, severe acute respiratory syndrome coronavirus 2 (SARS-CoV-2), has seriously disrupted the provision of oncology services and their uptake. Antibody testing, both at an individual level and of populations, has been widely viewed to be a key activity for guiding the options for treatment of high-risk individuals, as well as the implementation of safe control of infection measures. Ideally, the detection of a specific antibody should signify that all individuals tested have been infected by SARS-CoV-2 and that in the case of specific IgG that they are immune to further infection. This would enable SARS-CoV-2-infected individuals to be appropriately managed and healthcare workers shown to be immune to return to work where they would no longer pose a risk to their patients or be at risk themselves. Unfortunately, this is not the case for COVID-19, where it has been shown that immunity may not be protective, and seroconversion delayed or absent. The variability in antibody test performance, particularly that of lateral flow assays, has caused confusion for the public and healthcare professions alike. Many antibody test devices have been made available without independent evaluations and these may lack both adequate sensitivity and specificity. This review seeks to educate healthcare workers, particularly those working in oncology, of the current benefits and limitations of SARS-CoV-2 antibody testing.

Marchetti, D., et al. (2020). "Parenting-Related Exhaustion During the Italian COVID-19 Lockdown." J Pediatr Psychol **45**(10): 1114-1123.

OBJECTIVE: Worldwide, the coronavirus disease 2019 (COVID-19) pandemic has generated significant worry, uncertainty, anxiety, sadness, and loneliness. In Italy, these effects have been particularly pronounced. While research on the COVID-19 outbreak has mainly focused on the clinical features of infected patients and the psychological impact on the general population and health professionals, no investigation has yet assessed the psychological impact of the pandemic on parents. In the present research, we conducted a web-based survey of Italian parents to examine the prevalence of parenting-related exhaustion-and to identify its associated risk and protective factors-4 weeks into the lockdown. METHODS: A total of 1,226 parents provided their consent to participate in the study and completed a demographic questionnaire, information relating to particular COVID-19 experiences, and measures of emotional exhaustion, parental resilience, social connections, and psychological distress during the lockdown. RESULTS: Seventeen percent of our sample experienced significant parenting-related exhaustion, with mothers more severely affected. Multiple regression analyses showed that greater parenting-related exhaustion was predicted by psychological distress, lower parental resilience, motherhood, fewer perceived social connections, and being single, as well as having a child with special needs, having a large number of children, and having younger children. CONCLUSION: The findings add further support to the call for preventive programs to support parents throughout the COVID-19 pandemic. Mental health professionals and social workers should be warned of the effects of lockdown and social distancing on parenting and, consequently, the well-being of children.

Marco, C. A., et al. (2020). "Post-traumatic stress and stress disorders during the COVID-19 pandemic: Survey of emergency physicians." J Am Coll Emerg Physicians Open **1**(6): 1594-1601.

OBJECTIVE: Emergency physicians routinely encounter stressful clinical situations, including treating victims of crime, violence, and trauma; facing the deaths of patients; and delivering bad news. During a pandemic, stress may be increased for healthcare workers. This study was undertaken to identify symptoms of post-traumatic stress disorder (PTSD) among emergency physicians during the coronavirus disease 2019 (COVID-19) pandemic. METHODS: This cross-sectional survey was developed using the Life Events Checklist for Diagnostic and Statistical Manual of Mental Disorders, 5th Edition: DSM-5 (DSM-5) and the PTSD Checklist for DSM-5 (PCL-5). The survey was distributed to members of the American College of Emergency Physicians from May 21, 2020, through June 22, 2020. RESULTS: Among 1300 emergency physicians, a significant number of participants (22.3%; 95% confidence interval, 20.3-24.3%) reported symptoms of stress consistent with PTSD (PCL score ≥ 33). Higher PCL-5 scores were associated with age younger than 50 years (P < 0.05) and <10 years in practice (P < 0.05). The major sources of stress identified by participants included disinformation about COVID-19, computer work/electronic medical record, personal protective equipment concerns, and workload. The most common consequences of workplace stress were feeling distant or cut off from other people and sleep disturbance, such as trouble falling or staying asleep. CONCLUSIONS: A significant number of emergency physicians reported symptoms of stress consistent with PTSD. Higher PCL-5 scores were associated with age younger than 50 years and <10 years in practice.

Marco, D., et al. (2021). "Risk factors of SARS-CoV-2 seroprevalence among hospital employees in Italy - a single center study." Intern Med J.

BACKGROUND: The SARS-CoV-2 outbreak early in 2020 overwhelmed the Italian national health system, and hospitals were considered places at high risk of spreading the infection. We explored specific antibody seroprevalence of all employees at a single hospital in the epicenter of the outbreak, to identify areas of risk in nosocomial setting and to evaluate the usefulness of antibody testing. METHODS: All hospital workers were invited to fill in a questionnaire and undergo a blood test for SARS-CoV-2 IgG, using two commercial tests (DiaSorin and Abbott). Seropositivity was determined overall and according to demographic and occupations characteristics, for both tests singly and combined. RESULTS: The study enrolled 1562 hospital workers (95% of the eligible population). Overall, 153 participants (9.8%) were positive for SARS-CoV-2 IgG on DiaSorin test, and 150 (9.6%) were positive on Abbott test; both tests were positive in 123 cases (7.9%) and at least one was positive in 180 cases (11.5%). Factors associated with SARS-CoV-2 seropositivity included: being a smoker, working in Emergency or Medicine Departments, being a healthe care practitioner, self-reporting a relative with COVID-19 or symptoms suggestive of COVID-19, and having undergone a nasopharyngeal swab test. The tests were accurate in discriminating infected cases, with an area under the ROC curve of 0.867 using manufacturer-suggested cut-offs and 0.929 using optimized cut-offs. For discriminating symptomatic subjects, this value was 0.915 using optimized cut-offs. CONCLUSIONS: Seroprevalence for SARS-CoV-2 in this population of hospital workers was overall about 10%, with an excess prevalence in roles and departments associated with contacts with COVID-19 patients. This article is protected by copyright. All rights reserved.

Mariani, A., et al. (2021). "Factors associated with risk of COVID-19 contagion for endoscopy healthcare workers: A survey from the Italian society of digestive endoscopy." Dig Liver Dis.

BACKGROUND AND AIMS: The present study was aimed to assess the risk of SARS-CoV-2 infection and associated factors among HCWs in endoscopy centers in Italy. METHODS: All members of the Italian Society of Digestive Endoscopy (SIED) were invited to participate to a questionnaire-based survey during the first months of the COVID-19 outbreak in Italy. RESULTS: 314/1306 (24%) SIED members accounting for 201/502 (40%) endoscopic centers completed the survey. Personal Protection Equipment (PPE) were available in most centers, but filtering face-piece masks (FFP2 or FFP3) and negative pressure room were not in 10.9 and 75.1%. Training courses on PPE use were provided in 57.2% of centers only; there was at least one positive HCW in 17.4% of centers globally, 107/3308 (3.2%) HCWs were diagnosed with COVID-19 with similar rates of physicians (2.9%), nurses (3.5%) and other health operators (3.5%). Involvement in a COVID-19 care team (OR: 4.96) and the lack of training courses for PPE, (OR: 2.65) were associated with increased risk. CONCLUSIONS: The risk of COVID-19 among endoscopy HCWs was not negligible and was associated with work in a COVID-19 care team and lack of education on proper PPE use. These data deserve attention during the subsequent waves.

Marín-Gabriel, J. C. and E. R. Santiago (2020). "AEG-SEED position paper for the resumption of endoscopic activity after the peak phase of the COVID-19 pandemic." Gastroenterol Hepatol **43**(7): 389-407.

INTRODUCTION: The COVID-19 pandemic has led to the suspension of programmed activity in most of the Endoscopy Units in our environment. The aim of this document is to facilitate the resumption of elective endoscopic activity in an efficient and safe manner. MATERIAL AND METHODS: A series of questions considered to be of clinical and logistical relevance were formulated. In order to elaborate the answers, a structured bibliographic search was carried out in the main databases and the recommendations of the main Public Health and Digestive Endoscopy institutions were reviewed. The final recommendations were agreed upon through telematic means. RESULTS: A total of 33 recommendations were made. The main aspects discussed are: 1) Reassessment and prioritization of the indication, 2) Restructuring of spaces, schedules and health personnel, 3) Screening for infection, 4) Hygiene measures and personal protective equipment. CONCLUSION: The AEG and SEED recommend restarting endoscopic activity in a phased, safe manner, adapted to local resources and the epidemiological situation of SARS-CoV-2 infection.

eng

Markus, H. S. and S. Martins (2021). "COVID-19 and stroke-Understanding the relationship and adapting services. A global World Stroke Organisation perspective." Int J Stroke **16**(3): 241-247.

A year ago the World Stroke Organisation (WSO) highlighted the enormous global impact of the COVID-19 pandemic on stroke care. In this review, we consider a year later where we are now, what the future holds, and what the long-term effects of the pandemic will be on stroke. Stroke occurs in about 1.4% of patients hospitalized with COVID-19 infection, who show an excess of large vessel occlusion and increased mortality. Despite this association, stroke presentations fell dramatically during the pandemic, although emerging data suggest that total stroke mortality may have risen with increased stroke deaths at home and in care homes. Strategies and guidelines have been developed to adapt stroke services worldwide, and protect healthcare workers. Adaptations include increasing use of telemedicine for all aspects of stroke care. The pandemic is exacerbating already marked global inequalities in stroke incidence and mortality. Lastly, the pandemic has had a major impact on stroke research and funding, although it has also emphasized the importance of large scale collaborative research initiatives.

Markwell, A., et al. (2020). "Clinical and ethical challenges for emergency departments during communicable disease outbreaks: Can lessons from Ebola Virus Disease be applied to the COVID-19 pandemic?" Emerg Med Australas **32**(3): 520-524.

EDs fulfil a frontline function during public health emergencies (PHEs) and will play a pivotal role during the COVID-19 pandemic. This perspective article draws on qualitative data from a longitudinal, ethnographic study of an Australian tertiary ED to illustrate the clinical and ethical challenges faced by EDs during PHEs. Interview data collected during the 2014 Ebola Virus Disease PHE of International Concern suggest that ED clinicians have a strong sense of professional responsibility, but this can be compromised by increased visibility of risk and sub-optimal engagement from hospital managers and public health authorities. The study exposes the tension between a healthcare worker's right to protection and a duty to provide treatment. Given the narrow window of opportunity to prepare for a surge of COVID-19 presentations, there is an immediate need to reflect and learn from previous experiences. To maintain the confidence of ED clinicians, and minimise the risk of moral injury, hospital and public health authorities must urgently develop processes to support ethical healthcare delivery and ensure adequate resourcing of EDs.

Marler, H. and A. Ditton (2021). ""I'm smiling back at you": Exploring the impact of mask wearing on communication in healthcare." Int J Lang Commun Disord **56**(1): 205-214.

BACKGROUND: Surgical and respirator masks are worn to reduce the risk of droplet and airborne transmission of viral respiratory disease. As a result of the novel coronavirus (COVID-19) pandemic, mask wearing has been designated mandatory for healthcare professionals working in UK hospitals for the foreseeable future. It is thus timely to consider the long-term implications of mask wearing on communication within healthcare settings, from both a patient and a clinician perspective. AIMS: The primary objective is to identify research evidence that corresponds to the mask-wearing experiences of healthcare professionals working on the ground. By drawing together a summary of the literature illustrating the potential challenges associated with mask wearing, it is possible to make an application to various clinical cohorts and to formulate a set of preliminary, evidence-based support strategies. The paper additionally explores the role for the Speech and Language Therapist (SLT) in supporting communication in the context of mask wearing. METHODS & PROCEDURES: Through a scoping review of the relevant literature, this paper reflects holistically on the prospective challenges associated with mask wearing across a variety of healthcare settings and patient populations. The subsequent conclusions have been used to inform the proposed clinical guidelines for safe and effective practice. OUTCOMES & RESULTS: There is a current research gap with regards to mask wearing in non-medical and non-clinical healthcare workers, and the impact this may have on both a professional and a personal basis. In the absence of preliminary data, the development of associated communication support strategies is hindered. This paper draws upon a variety of clinically conceivable issues faced by healthcare professionals, outlines important practical and ethical considerations, and proposes evidence-based solutions to some of the challenges identified. Future research is required to gather evidence with regards to actual clinical experiences of mask wearing to substantiate hypotheses. CONCLUSIONS & IMPLICATIONS: Although undoubtedly essential in protecting the health of staff and patients, there are numerous logistical, physiological, psychological, social and economic complications associated with the wearing of masks. The ability of healthcare staff to successfully communicate with patients and with colleagues is jeopardized, which may adversely affect the efficiency, effectiveness, equitability and, most notably, safety of therapeutic intervention. The SLT has a distinct role in facilitating communication in order to safeguard the provision, accessibility and efficacy of services. What this paper adds What is already known on the subject Existing research explores the impact of mask wearing on medical doctors, surgeons and dentists, and upon the corresponding patient cohorts. Little is known about how mask wearing may affect Allied Health Professionals and their ability to deliver therapeutic interventions safely and effectively. With mandatory face covering potentially a long-term requirement for UK healthcare staff, it is both timely and relevant to consider the consequences of mask wearing on communication across acute and community settings. What this paper adds to existing knowledge This paper identifies a range of prospective key issues associated with mask wearing across a variety of clinical and non-clinical populations, with application specifically to vulnerable patient cohorts. Through evidence synthesis, this paper provides a summary of fundamental issues supported by relevant literature, and draws upon these in order to propose a preliminary set of evidence-based clinical guidelines setting out potential solutions to the challenges faced. This review additionally assists in quantifying the role of the SLT within these extraordinary circumstances, with the aim of prompting unified practice, building upon professional guidance and increasing skill recognition. What are the potential or actual clinical implications of this work? In addition to their role in facilitating the development of individualised communication strategies for patients, SLTs should actively seek to provide widely accessible multidisciplinary education opportunities focusing on supporting communication; with specific reference to mask wearing and the associated communicative challenges. At a commissioning and managerial level, leaders within healthcare should acknowledge mask wearing as just one of the complexities associated with frontline working in the context of the COVID-19 pandemic, and aim to support their workforce by delivering resources and protocols which maximize and promote staff safety, efficiency, resilience and well-being in concurrence with positive patient outcomes.

Marmor, M., et al. (2020). "Quality improvement tool for rapid identification of risk factors for SARS-CoV-2 infection among healthcare workers." J Hosp Infect **105**(4): 710-716.

The rapid growth of the coronavirus disease 2019 (COVID-19) pandemic, limited availability of personal protective equipment, and uncertainties regarding transmission modes of severe acute respiratory syndrome coronavirus-2 have heightened concerns for the safety of healthcare workers (HCWs). Systematic studies of occupational risks for COVID-19 in the context of community risks are difficult and have only recently started to be reported. Ongoing quality improvement studies in various locales and within many affected healthcare institutions are needed. A template design for small-scale quality improvement surveys is proposed. Such surveys have the potential for rapid implementation and completion, are cost-effective, impose little administrative or workforce burden, can reveal occupational risks while taking community risks into account, and can be repeated easily with short time intervals between repetitions. This article describes a template design and proposes a survey instrument that is easily modifiable to fit the particular needs of various healthcare institutions in the hope of beginning a collaborative effort to refine the design and instrument. These methods, along with data management and analytic techniques, can be widely useful and shared globally. The authors' goal is to facilitate quality improvement surveys aimed at reducing the risk of occupational infection of HCWs during the COVID-19 pandemic.

Marot, S., et al. (2021). "Rapid decline of neutralizing antibodies against SARS-CoV-2 among infected healthcare workers." Nat Commun **12**(1): 844.

There are only few data concerning persistence of neutralizing antibodies (NAbs) among SARS-CoV-2-infected healthcare workers (HCW). These individuals are particularly exposed to SARS-CoV-2 infection and at potential risk of reinfection. We followed 26 HCW with mild COVID-19 three weeks (D21), two months (M2) and three months (M3) after the onset of symptoms. All the HCW had anti-receptor binding domain (RBD) IgA at D21, decreasing to 38.5% at M3 (p < 0.0001). Concomitantly a significant decrease in NAb titers was observed between D21 and M2 (p = 0.03) and between D21 and M3 (p < 0.0001). Here, we report that SARS-CoV-2 can elicit a NAb response correlated with anti-RBD antibody levels. However, this neutralizing activity declines, and may even be lost, in association with a decrease in systemic IgA antibody levels, from two months after disease onset. This short-lasting humoral protection supports strong recommendations to maintain infection prevention and control measures in HCW, and suggests that periodic boosts of SARS-CoV-2 vaccination may be required.

Márquez-González, H., et al. (2021). "Risk conditions in healthcare workers of a pediatric coronavirus disease center in Mexico City." Bol Med Hosp Infant Mex **78**(2): 110-115.

BACKGROUND: The new evere acute respiratory syndrome coronavirus 2 (SARS-CoV-2) is characterized by its high capacity to transmit. Health-care personnel is highly susceptible to becoming infected. This study aimed to determine the characteristics and known risk factors for contagion and severe outcomes of SARS-CoV-2 disease in health-care personnel of a pediatric coronavirus disease (COVID) center in Mexico City. METHODS: In the last week of March 2020 (at the beginning of phase 2 of the Ministry of Health's national campaign in Mexico), a study was conducted on healthcare workers of a pediatric COVID hospital in Mexico City. Using a virtual interview, we evaluated comorbidities, mobility, areas and functions where they carry out the activities, protection measures, contact history, and vaccination. According to their activities, healthcare workers were classified into the following areas: medical, nursing, other health-care personnel (researchers, nutritionists, rehabilitation, imaging, and laboratory), administrative, and other services. We compared the variables between the groups of healthcare workers with the X2 test. RESULTS: We included 812 participants. The mean age was 41 ± 11 years, and 33% were overweight or obese, 18% were over 60 years old, and 19% had high blood pressure. Medical and nursing personnel presented a higher proportion in the use of standard protection measures. CONCLUSIONS: Among healthcare workers, there are risk conditions for the development of complications in case of SARS-CoV-2 infection. Most medical and nursing personnel use standard protective measures.

Marraha, F., et al. (2021). "Skin Reactions to Personal Protective Equipment among First-Line COVID-19 Healthcare Workers: A Survey in Northern Morocco." Ann Work Expo Health.

OBJECTIVES: Health care workers (HCWs) adopted several protective measures, including hand hygiene and wearing personal protective equipment (PPE) during the COVID-19 outbreak. However, the frequent use of these preventive measures can lead to skin reactions. Our study aimed to determine the frequency of these reactions in Northern Morocco. In addition, we also looked at the risk factors and the consequences of these injuries on work efficiency and performance. MATERIALS AND METHODS: An anonymous online survey was used to collect data, which was sent to 500 health workers in the study region. Descriptive and inferential statistics were used to analyze the data on IBM SPSS software. RESULTS: In total, 273/500 responded to the questionnaire (55%). For the participants' profession, 41% were doctors, 32% were nursing staff, and 26% held other jobs. The general prevalence rate of adverse reactions for all health workers was (80%), including skin problems: after wearing goggles (58%), after wearing surgical masks and respirators (57%), after handwashing and wearing gloves (45%), after wearing a face shield (23%), and after wearing protective clothing (11%). Bleach immersion was highly significantly associated with hand reaction (OR: 2.9, 95% CI: 1.77-4.90; P < 0.001). Moreover, we found a statistically significant association between hand cream use more than twice daily and fewer reactions (OR: 1.9, 95% CI: 0.98-3.77; P = 0.038). The skin reactions related to goggles use were also significantly associated with use duration (OR: 1.7, 95% CI: 0.988-3.12; P = 0.05). Similarly, wearing masks and N95 respirators and their related adverse reactions were significantly associated with use duration (OR: 0.5, 95% CI: 0.20-0.7; P = 0.02). In addition, adverse reactions of regular use of protective clothing were related to the frequency of its use per shift (OR: 3.5, 95% CI: 1.47-8.54; P = 0.05). CONCLUSIONS: Our survey-based study showed that the prevalence of these skin reactions in our context should not be neglected. The length of daily wearing time and the frequency of PPE uses were the most implicated factors. More attention must be paid to these reactions for better care of HCWs during these critical times.

Martí, M., et al. (2021). "Protective Face Mask Filter Capable of Inactivating SARS-CoV-2, and Methicillin-Resistant Staphylococcus aureus and Staphylococcus epidermidis." Polymers (Basel) **13**(2).

Face masks have globally been accepted to be an effective protective tool to prevent bacterial and viral transmission, especially against indoor aerosol transmission. However, commercial face masks contain filters that are made of materials that are not capable of inactivating either SARS-CoV-2 or multidrug-resistant bacteria. Therefore, symptomatic and asymptomatic individuals can infect other people even if they wear them because some viable viral or bacterial loads can escape from the masks. Furthermore, viral or bacterial contact transmission can occur after touching the mask, which constitutes an increasing source of contaminated biological waste. Additionally, bacterial pathogens contribute to the SARS-CoV-2-mediated pneumonia disease complex, and their resistance to antibiotics in pneumonia treatment is increasing at an alarming rate. In this regard, herein, we report the development of a non-woven face mask filter fabricated with a biofunctional coating of benzalkonium chloride that is capable of inactivating more than 99% of SARS-CoV-2 particles in one minute of contact, and the life-threatening methicillin-resistant Staphylococcus aureus and Staphylococcus epidermidis (normalized antibacterial halos of 0.52 ± 0.04 and 0.72 ± 0.04, respectively). Nonetheless, despite the results obtained, further studies are needed to ensure the safety and correct use of this technology for the mass production and commercialization of this broad-spectrum antimicrobial face mask filter. Our novel protective non-woven face mask filter would be useful for many healthcare workers and researchers working in this urgent and challenging field.

Martin, G., et al. (2020). "Use of the HoloLens2 Mixed Reality Headset for Protecting Health Care Workers During the COVID-19 Pandemic: Prospective, Observational Evaluation." J Med Internet Res **22**(8): e21486.

BACKGROUND: The coronavirus disease (COVID-19) pandemic has led to rapid acceleration in the deployment of new digital technologies to improve both accessibility to and quality of care, and to protect staff. Mixed-reality (MR) technology is the latest iteration of telemedicine innovation; it is a logical next step in the move toward the provision of digitally supported clinical care and medical education. This technology has the potential to revolutionize care both during and after the COVID-19 pandemic. OBJECTIVE: This pilot project sought to deploy the HoloLens2 MR device to support the delivery of remote care in COVID-19 hospital environments. METHODS: A prospective, observational, nested cohort evaluation of the HoloLens2 was undertaken across three distinct clinical clusters in a teaching hospital in the United Kingdom. Data pertaining to staff exposure to high-risk COVID-19 environments and personal protective equipment (PPE) use by clinical staff (N=28) were collected, and assessments of acceptability and feasibility were conducted. RESULTS: The deployment of the HoloLens2 led to a 51.5% reduction in time exposed to harm for staff looking after COVID-19 patients (3.32 vs 1.63 hours/day/staff member; P=.002), and an 83.1% reduction in the amount of PPE used (178 vs 30 items/round/day; P=.02). This represents 222.98 hours of reduced staff exposure to COVID-19, and 3100 fewer PPE items used each week across the three clusters evaluated. The majority of staff using the device agreed it was easy to set up and comfortable to wear, improved the quality of care and decision making, and led to better teamwork and communication. In total, 89.3% (25/28) of users felt that their clinical team was safer when using the HoloLens2. CONCLUSIONS: New technologies have a role in minimizing exposure to nosocomial infection, optimizing the use of PPE, and enhancing aspects of care. Deploying such technologies at pace requires context-specific information security, infection control, user experience, and workflow integration to be addressed at the outset and led by clinical end-users. The deployment of new telemedicine technology must be supported with objective evidence for its safety and effectiveness to ensure maximum impact.

Martin-Delgado, J., et al. (2020). "Availability of personal protective equipment and diagnostic and treatment facilities for healthcare workers involved in COVID-19 care: A cross-sectional study in Brazil, Colombia, and Ecuador." PLoS One **15**(11): e0242185.

Many affected counties have had experienced a shortage of personal protective equipment (PPE) during the coronavirus disease (COVID-19) pandemic. We aimed to investigate the needs of healthcare professionals and the technical difficulties faced by them during the initial outbreak. A cross-sectional web-based survey was conducted among the healthcare workforce in the most populous cities from three Latin American countries in April 2020. In total, 1,082 participants were included. Of these, 534 (49.4%), 263 (24.3%), and 114 (10.5%) were physicians, nurses, and other professionals, respectively. At least 70% of participants reported a lack of PPE. The most common shortages were shortages in gown coverall suits (643, 59.4%), N95 masks (600, 55.5%), and face shields (569, 52.6%). Professionals who performed procedures that generated aerosols reported shortages more frequently (p<0.05). Professionals working in the emergency department and primary care units reported more shortages than those working in intensive care units and hospital-based wards (p<0.001). Up to 556 (51.4%) participants reported the lack of sufficient knowledge about using PPE. Professionals working in public institutions felt less prepared, received less training, and had no protocols compared with their peers in working private institutions (p<0.001). Although the study sample corresponded to different hospital centers in different cities from the participating countries, sampling was non-random. Healthcare professionals in Latin America may face more difficulties than those from other countries, with 7 out of 10 professionals reporting that they did not have the necessary resources to care for patients with COVID-19. Technical and logistical difficulties should be addressed in the event of a future outbreak, as they have a negative impact on healthcare workers. Clinical trial registration: NCT04486404.

Martínez-Beneyto, Y., et al. (2021). "Spanish Dentists' Awareness, Knowledge, and Practice Regarding COVID-19: A Multiple Regression Analysis." Int Dent J.

INTRODUCTION: During the first months of the coronavirus disease 2019 (COVID-19) pandemic, Spain had the highest mortality rate and the second-highest infection rate in the world. OBJECTIVE: To analyze the occupational situation of dentists, hygienists, and dental auxiliary staff during the peak of the pandemic, after the state of alarm was declared in Spain, and when the state of alarm was declared. In addition, a possible relationship between the geographical distribution of infected people and the availability of individual protection systems was investigated. MATERIAL AND METHODS: A cross-sectional questionnaire was answered by 6470 dentists and dental staff via WhatsApp and social media. RESULTS: A total of 1 in 4 dental professionals ceased working completely. Of those that kept working, 25.28% of dentists and 19.61% of hygienist-auxiliary were equipped with filtering face piece (FFP) 2 masks (P < .05), and 61.8% complied with the official protection recommendations set by the General Council of Dentists of Spain. Nearly 59.4% of respondents had symptoms, but only 1.5% of dentists were tested, with 14% of dentists in isolation at the time of response. Overall, it is suggested that 10% of dental professionals may have been in direct contact with the coronavirus. CONCLUSIONS: Direct contact of Spanish dental health professionals with severe acute respiratory syndrome coronavirus disease 2 (SARS CoV-2) has been high during the most active phase of the pandemic. Dental professionals did not have personal protective equipment (PPE) necessary to care for patients, a situation that justified the reduction in scheduled dental care and only emergencies being treated. The Spanish geographical regions with the highest number of contagions had the least amount of individual protective resources (FFP2 and FFP3 masks).

Martínez-López, J., et al. (2020). "Psychological Impact of COVID-19 Emergency on Health Professionals: Burnout Incidence at the Most Critical Period in Spain." J Clin Med **9**(9).

BACKGROUND: The health profession is a burnout producer due to the continuous contact with pain and suffering. In addition, excessive workloads can generate stress and psychological distress. OBJECTIVES: The aim of this study was to determine the degree of burnout and its main triggers in health professionals in Spain at the most critical period of the COVID-19 emergency. METHOD: A quantitative research was developed through a simple random sampling in different Spanish hospitals through the period of greatest impact of the pandemic (N = 157). Data were collected using a standardized questionnaire from Maslach burnout inventory (MBI) containing 22 items, which measures three subscales: emotional burnout, depersonalization, and self-fulfillment. RESULTS: depersonalization values reached 38.9%. A total of 90.4% of the health professionals considered that psychological care should be provided from the work centers. Furthermore, 43.3% of the health professionals estimated that they might need psychological treatment in the future. Finally, 85.4% stated that the lack of personal protective equipment (PPE) generated an increase in stress and anxiety. CONCLUSION: This study demonstrates the need to consider specific mental health care services and training in crises to avoid possible psychological disorders. The information obtained is also valuable for the development of future prevention protocols and training of health personnel to face pandemics of these characteristics or emergency scenarios. Having the necessary physical means for their protection, as well to updated regular and accurate information, is essential to avoid feelings of fear and uncertainty. This would promote the health of these professionals.

Martín-Rodríguez, F., et al. (2021). "Blood Biomarkers for Assessing Headaches in Healthcare Workers after Wearing Biological Personal Protective Equipment in a COVID-19 Field Hospital." J Pers Med **11**(1).

The coronavirus disease 2019 (COVID-19) has led to a pandemic, which among other things, has highlighted biosafety as a key cornerstone in the management of disease transmission. The aim of this work was to analyze the role played by different blood biomarkers in predicting the appearance of headaches in healthcare workers wearing personal protective equipment (PPE) in a COVID-19 treatment unit. A prospective cohort study of 38 healthcare workers was performed during April 2020. Blood analysis, performed just before the start of a 4 hour shift, was carried out on all volunteers equipped with PPE. At the end of their shifts and after decontamination, they were asked if they had suffered from headache in order to obtain a binary outcome. The baseline creatinine value reflected a specific odds ratio of 241.36 (95% CI: 2.50-23,295.43; p = 0.019) and an area under the curve (AUC) value of 0.737 (95%CI: 0.57-0.90; p < 0.01). Blood creatinine is a good candidate for predicting the appearance of a de novo headache in healthcare workers after wearing PPE for four hours in a COVID-19 unit.

Martín-Vaquero, Y., et al. (2021). "Safe handling of clothing and hygiene of patients and health professionals: Scoping review." Enferm Clin **31 Suppl 1**: S89-s93.

INTRODUCTION: In January 2020, an outbreak of pneumonia caused by a zoonotic virus (SARS-CoV-2) appeared in China. The main route of transmission is considered to be the inhalation of large respiratory drops, by deposition in the mucosa and hands or contaminated fomites. The objective is to identify the interventions to be performed during hospitalization for the correct and safe handling of clothing and hygiene of patients and health professionals. METHOD: Scoping review carried out without chronological or language delimitation in the PUBMED and Cochrane databases. Tracking standards and recommendations of national and international government entities to answer the research question on the safe handling of clothing and skin hygiene in patients and in health professionals to avoid Covid-19 infection. The data analysis was carried out in 2stages: in the first, identification and categorization of the studies, and in the second, content analysis as an informative and classifying criterion. RESULTS: 14 documents have been selected, mainly from government entities. The recommendations are structured in 5 sections on the management of clothing and skin hygiene of infected patients and health professionals in the hospital setting. CONCLUSION: The clothing of patients and healthcare personnel are transmitting vehicles of the disease. Its correct treatment helps to improve the control of the same and the correct use of the resources available at the moment. Proper skin hygiene, especially hand cleansing, is one of the basic pillars for infection prevention and control. We highlight the similarity of some of the guidelines collected and provided by the different agencies consulted.

INTRODUCTION: In January 2020, an outbreak of pneumonia caused by a zoonotic virus (SARS-CoV-2) appeared in China. The main route of transmission is considered to be the inhalation of large respiratory drops, by deposition in the mucosa and hands or contaminated fomites. The objective is to identify the interventions to be performed during hospitalization for the correct and safe handling of clothing and hygiene of patients and health professionals. METHOD: Scoping review carried out without chronological or language delimitation in the PUBMED and Cochrane databases. Tracking standards and recommendations of national and international government entities to answer the research question on the safe handling of clothing and skin hygiene in patients and in health professionals to avoid Covid-19 infection. The data analysis was carried out in 2 stages: in the first, identification and categorization of the studies, and in the second, content analysis as an informative and classifying criterion. RESULTS: 14 documents have been selected, mainly from government entities. The recommendations are structured in 5 sections on the management of clothing and skin hygiene of infected patients and health professionals in the hospital setting. CONCLUSION: The clothing of patients and healthcare personnel are transmitting vehicles of the disease. Its correct treatment helps to improve the control of the same and the correct use of the resources available at the moment. Proper skin hygiene, especially hand cleansing, is one of the basic pillars for infection prevention and control. We highlight the similarity of some of the guidelines collected and provided by the different agencies consulted.

eng

Martischang, R., et al. (2021). "SARS-CoV-2 seroconversion and occupational exposure of employees at a Swiss university hospital: a large longitudinal cohort study." Infect Control Hosp Epidemiol: 1-53.

BACKGROUND: The dynamics of coronavirus disease 2019 (COVID-19) seroconversion of hospital employees are understudied. Our aim was to measure the proportion of seroconverted employees and evaluate risk factors for seroconversion. METHODS: This prospective cohort study recruited Geneva University Hospitals employees sampled 3 times every 3 weeks from March 30 to June 12, 2020. We measured the proportion of seroconverted employees, and determined prevalence ratios of risk factors for seroconversion using mixed-effects multivariable Poisson regression models. FINDINGS: Overall, 3'421 participants (29% of all employees) were included with 92% follow-up. The proportion of seroconverted employees increased from 4·4% [95%CI, 3·7-5·1] at baseline to 8·5% [95%CI, 7·6-9·5] at the last visit. The proportions of seroconverted employees working in COVID-19 (32·3%) and non COVID-19 geriatrics and rehabilitation (G&R) wards (12·3%) were higher compared to office workers (4·9%) at the last visit. Only nursing assistants had a significantly higher risk of seroconversion when compared to office workers (11·7% vs 4·9%, p = 0·006). Significant risk factors for seroconversion included use of public transportation (adjusted prevalence ratio, 1·59 [95%CI 1·25-2·03]), community exposure to SARS-CoV-2 (2·80 [95%CI 2·22-3·54]), working in a ward with a nosocomial COVID outbreak (2·93 [95%CI 2·27-3·79]), and working in COVID-19 (3·47 [95%CI 2·45-4·91]) or non-COVID-19 G&R wards (1·96 [95%CI 1·46-2·63]). An association was observed between reported use of respirators and lower risk of seroconversion (0·73 [95%CI 0·55-0·96]). INTERPRETATION: Additional efforts are needed to protect employees in elderly care wards. There is an urgent need for randomized trials on the protective effect of respirators.

Masen, M. A., et al. (2020). "Evaluating lubricant performance to reduce COVID-19 PPE-related skin injury." PLoS One **15**(9): e0239363.

BACKGROUND: Healthcare workers around the world are experiencing skin injury due to the extended use of personal protective equipment (PPE) during the COVID-19 pandemic. These injuries are the result of high shear stresses acting on the skin, caused by friction with the PPE. This study aims to provide a practical lubricating solution for frontline medical staff working a 4+ hours shift wearing PPE. METHODS: A literature review into skin friction and skin lubrication was conducted to identify products and substances that can reduce friction. We evaluated the lubricating performance of commercially available products in vivo using a custom-built tribometer. FINDINGS: Most lubricants provide a strong initial friction reduction, but only few products provide lubrication that lasts for four hours. The response of skin to friction is a complex interplay between the lubricating properties and durability of the film deposited on the surface and the response of skin to the lubricating substance, which include epidermal absorption, occlusion, and water retention. INTERPRETATION: Talcum powder, a petrolatum-lanolin mixture, and a coconut oil-cocoa butter-beeswax mixture showed excellent long-lasting low friction. Moisturising the skin results in excessive friction, and the use of products that are aimed at 'moisturising without leaving a non-greasy feel' should be prevented. Most investigated dressings also demonstrate excellent performance.

Massoudi, N. and B. Mohit (2021). "A Case-Control Study of the 2019 Influenza Vaccine and Incidence of COVID-19 Among Healthcare Workers." J Clin Immunol **41**(2): 324-334.

PURPOSE: The influenza vaccine is essential in reducing the influenza burden, especially among healthcare workers (HCW). Experimental studies suggest both coronaviruses and influenza viruses engage with the angiotensin-converting enzyme 2 (ACE 2) and tetraspanin antibodies, and that ACE 2 tetraspanin antibodies in turn may inhibit both coronavirus and low-pathogenicity influenza A viruses (LP IAV) infections. This study aims to investigate the potential clinical association between receiving the 2019 influenza vaccine and the incidence of COVID-19 among HCW. METHODS: We designed a case-control study within a hospital setting in Iran when it became a center for treating COVID-19 patients. We collected data and calculated relevant incidence and associative measures among HCW who had received the 2019 influenza vaccine as compared to HCW who had not received the vaccine. RESULTS: Our total sample size was 261 HCW. Of 80 COVID-19 incident cases, three cases had received the influenza vaccine, while 87 of 181 controls had received the vaccine. The odds ratio (OR) and confidence interval (CI) of being vaccinated were 0.04 (95% CI: 0.01 to 0.14) among COVID-19 cases as compared to controls. CONCLUSIONS: Significant findings suggest that the 2019 influenza vaccine may have a protective association against COVID-19 among HCW.

Mathur, S., et al. (2020). "Development of an enhanced acute care surgery service in response to the COVID-19 global pandemic." Injury **51**(10): 2135-2141.

BACKGROUND: As the COVID-19 pandemic sweeps across the world, healthcare departments must adapt to meet the challenges of service provision and staff/patient protection. Unlike elective surgery, acute care surgery (ACS) workloads cannot be artificially reduced providing a unique challenge for administrators to balance healthcare resources between the COVID-19 surge and regular patient admissions. METHODS: An enhanced ACS (eACS) model of care is described with the aim of limiting COVID-19 healthcare worker and patient cross-infection as well as providing 24/7 management of emergency general surgical (GS) and trauma patients. The eACS service comprised 5 independent teams covering a rolling 1:5 24-hr call. Attempts to completely separate eACS teams and patients from the elective side were made. The service was compared to the existing ACS service in terms of clinical and efficiency outcomes. Finally, a survey of staff attitudes towards these changes, concerns regarding COVID-19 and psychological well-being was assessed. RESULTS: There were no staff/patient COVID-19 cross-infections. Compared to the ACS service, eACS patients had reduced overall length of stay (2-days), time spent in the Emergency Room (46 min) and time from surgery to discharge (2.4-hours). The eACS model of care saved financial resources and bed-days for the organisation. The changes were well received by team-members who also felt that their safety was prioritised. CONCLUSION: In healthcare systems not overwhelmed by COVID-19, an eACS model may assist in preserving psychological well-being for healthcare staff whilst providing 24/7 care for emergency GS and trauma patients.

Maude, R. R., et al. (2021). "Improving knowledge, attitudes and practice to prevent COVID-19 transmission in healthcare workers and the public in Thailand." BMC Public Health **21**(1): 749.

BACKGROUND: Key infection prevention and control measures to limit transmission of COVID-19 include social distancing, hand hygiene, use of facemasks and personal protective equipment. However, these have limited or no impact if not applied correctly through lack of knowledge, inappropriate attitude or incorrect practice. In order to maximise the impact of infection prevention and control measures on COVID-19 spread, we undertook a study to assess and improve knowledge, attitudes and practice among 119 healthcare workers and 100 general public in Thailand. The study setting was two inpatient hospitals providing COVID-19 testing and treatment. Detailed information on knowledge, attitudes and practice among the general public and healthcare workers regarding COVID-19 transmission and its prevention were obtained from a combination of questionnaires and observations. RESULTS: Knowledge of the main transmission routes, commonest symptoms and recommended prevention methods was mostly very high (> 80%) in both groups. There was lower awareness of aerosols, food and drink and pets as sources of transmission; of the correct duration for handwashing; recommended distance for social/physical distancing; and about recommended types of face coverings. Information sources most used and most trusted were the workplace, work colleagues, health workers and television. The results were used to produce a set of targeted educational videos which addressed many of these gaps with subsequent improvements on retesting in a number of areas. This included improvements in handwashing practice with an increase in the number of areas correctly washed in 65.5% of the public, and 57.9% of healthcare workers. The videos were then further optimized with feedback from participants followed by another round of retesting. CONCLUSIONS: Detailed information on gaps in knowledge, attitudes and practice among the general public and healthcare workers regarding COVID-19 transmission and its prevention were obtained from a combination of questionnaires and observations. This was used to produce targeted educational videos which addressed these gaps with subsequent improvements on retesting. The resulting videos were then disseminated as a resource to aid in efforts to fight COVID-19 in Thailand and worldwide.

Mayr, N. P., et al. (2020). "[Occupational safety in the treatment of COVID-19 patients : Relevant laws and regulations for the treating personnel]." Anaesthesist **69**(10): 712-716.

The intensive medical care of COVID-19 patients presents the deployed personnel with as yet unknown challenges. For example, protective equipment is now being extensively used, which was otherwise only used in selected situations. Working in such an environment is to be evaluated differently under the aspect of occupational safety than other patient care, especially as more than 1900 suspected cases of a SARS-CoV-19 occupational disease were reported among healthcare workers in Germany. Even in a pandemic, the legal requirements remain valid and personal protective equipment (PPE) has to comply with given standards. The use of FFP3 masks is required in aerosol-forming situations, such as endotracheal intubation or bronchoscopy. In contrast to surgical face masks, there is a maximum wearing time for FFPs masks. Furthermore, in a pandemic there is a basic danger of PPE shortage and recycling of face masks is under discussion. Therefore, usage of non-EU certified PPE may come into effect but this has to follow the requirements defined by European regulations. The aim of this article is to provide an overview of the currently relevant rules and regulations in Germany.

Mbachu, C. N. P., et al. (2020). "COVID-19 infection: Knowledge, attitude, practices, and impact among healthcare workers in a South-Eastern Nigerian state." J Infect Dev Ctries **14**(9): 943-952.

INTRODUCTION: The coronavirus disease (COVID-19) infection rate and mortality among Nigerian health care workers appear to be on the increase. This study determined the level of knowledge, attitude, practices, and impact of COVID-19 infection on healthcare workers (HCWs) working in a South-Eastern Nigerian state. METHODOLOGY: This was a web-based, cross-sectional study conducted among healthcare workers in South-eastern, Nigeria during the lockdown period. Socio-demographic profile, knowledge of COVID-19, fears and impact concerning COVID-19, attitude of health workers to work, preventive practices during this pandemic period were obtained. Data were analysed using STATA 16.0. Chi-square and Fisher's exact tests of association were used to determine the association between variables, with the significance level set at p < 0.05. RESULTS: A total of 403 health care workers participated in the study. Majority of participants (n = 357, 88.59%) had good knowledge and good preventive practices (n = 328, 81.39%) of COVID-19. A significant proportion of respondents had a poor attitude to work (n = 101, 25.06%) and an attitude of indifference (n = 233, 57.82%). Almost half (48.64%) of participants had been negatively affected by COVID-19. Knowledge significantly influenced practice (p = 0.029). Lack of Personal protective equipment, fear of dying and going to common places, had a significant impact on the attitude of workers. CONCLUSION: Good knowledge which influenced practice, high use of preventive practices, with associated poor and indifferent attitude was noted among healthcare workers. Fear of death and lack of personal protective equipment had a strong impact on attitude. Female HCWs had poorer attitude to work than males.

McCarthy, R., et al. (2020). "The Importance of Personal Protective Equipment Design and Donning and Doffing Technique in Mitigating Infectious Disease Spread: A Technical Report." Cureus **12**(12): e12084.

During the current coronavirus pandemic, significant emphasis has been placed on the importance of mitigating nosocomial spread of coronavirus disease 2019 (COVID-19). One important consideration involves the appropriate use of effective personal protective equipment (PPE), which may reduce a healthcare provider's likelihood of becoming infected while simultaneously minimizing exposure to other patients that they care for. This may reduce demands placed on the healthcare system and help to preserve the workforce. First, the importance of PPE design cannot be underestimated, as the manufacturing process must strive to maximize protection of the user while ensuring adequate comfort. Second, it has been demonstrated that inadequate education and training can significantly impact compliance with PPE recommendations. Technique regarding donning and doffing of PPE is crucial to the protection of those who don it. The purpose of this technical report is two-fold: first, to describe some important considerations in the manufacturing and design process of face shields to maximize protection for healthcare providers, and second, to describe a simulation scenario that may be used to train healthcare workers in the appropriate donning and doffing of PPE.

McConnell, D. (2020). "Balancing the duty to treat with the duty to family in the context of the COVID-19 pandemic." J Med Ethics **46**(6): 360-363.

Healthcare systems around the world are struggling to maintain a sufficient workforce to provide adequate care during the COVID-19 pandemic. Staffing problems have been exacerbated by healthcare workers (HCWs) refusing to work out of concern for their families. I sketch a deontological framework for assessing when it is morally permissible for HCWs to abstain from work to protect their families from infection and when it is a dereliction of duty to patients. I argue that it is morally permissible for HCWs to abstain from work when their duty to treat is outweighed by the combined risks and burdens of that work. For HCWs who live with their families, the obligation to protect one's family from infection contributes significantly to those burdens. There are, however, a range of complicating factors including the strength of duty to treat which varies according to the HCW's role, the vulnerability of family members to the disease, the willingness of family members to risk infection and the resources available to the HCW to protect their family. In many cases, HCWs in 'frontline' roles with a weak duty to treat and families at home will be morally permitted to abstain from work given the risks posed by COVID-19; therefore, society should provide additional incentives to maintain sufficient staff in these roles.

McCormick, W. L., et al. (2021). "Level of respiratory protection for healthcare workers caring for coronavirus disease 2019 (COVID-19) patients: A survey of hospital epidemiologists." Infect Control Hosp Epidemiol: 1-2.

McDermott, C., et al. (2021). "Sonographic Diagnosis of COVID-19: A Review of Image Processing for Lung Ultrasound." Front Big Data **4**: 612561.

The sustained increase in new cases of COVID-19 across the world and potential for subsequent outbreaks call for new tools to assist health professionals with early diagnosis and patient monitoring. Growing evidence around the world is showing that lung ultrasound examination can detect manifestations of COVID-19 infection. Ultrasound imaging has several characteristics that make it ideally suited for routine use: small hand-held systems can be contained inside a protective sheath, making it easier to disinfect than X-ray or computed tomography equipment; lung ultrasound allows triage of patients in long term care homes, tents or other areas outside of the hospital where other imaging modalities are not available; and it can determine lung involvement during the early phases of the disease and monitor affected patients at bedside on a daily basis. However, some challenges still remain with routine use of lung ultrasound. Namely, current examination practices and image interpretation are quite challenging, especially for unspecialized personnel. This paper reviews how lung ultrasound (LUS) imaging can be used for COVID-19 diagnosis and explores different image processing methods that have the potential to detect manifestations of COVID-19 in LUS images. Then, the paper reviews how general lung ultrasound examinations are performed before addressing how COVID-19 manifests itself in the images. This will provide the basis to study contemporary methods for both segmentation and classification of lung ultrasound images. The paper concludes with a discussion regarding practical considerations of lung ultrasound image processing use and draws parallels between different methods to allow researchers to decide which particular method may be best considering their needs. With the deficit of trained sonographers who are working to diagnose the thousands of people afflicted by COVID-19, a partially or totally automated lung ultrasound detection and diagnosis tool would be a major asset to fight the pandemic at the front lines.

McDougall, R. J., et al. (2020). "Balancing health worker well-being and duty to care: an ethical approach to staff safety in COVID-19 and beyond." J Med Ethics.

The COVID-19 pandemic has highlighted the risks that can be involved in healthcare work. In this paper, we explore the issue of staff safety in clinical work using the example of personal protective equipment (PPE) in the COVID-19 crisis. We articulate some of the specific ethical challenges around PPE currently being faced by front-line clinicians, and develop an approach to staff safety that involves balancing duty to care and personal well-being. We describe each of these values, and present a decision-making framework that integrates the two. The aim of the framework is to guide the process of balancing these two values when staff safety is at stake, by facilitating ethical reflection and/or decision-making that is systematic, specific and transparent. It provides a structure for individual reflection, collaborative staff discussion, and decision-making by those responsible for teams, departments and other groups of healthcare staff. Overall the framework guides the decision maker to characterise the degree of risk to staff, articulate feasible options for staff protection in that specific setting and identify the option that ensures any decrease in patient care is proportionate to the increase in staff well-being. It applies specifically to issues of PPE in COVID-19, and also has potential to assist decision makers in other situations involving protection of healthcare staff.

McEllistrem, M. C., et al. (2021). "SARS-CoV-2 is associated with high viral loads in asymptomatic and recently symptomatic healthcare workers." PLoS One **16**(3): e0248347.

BACKGROUND: Healthcare workers (HCW) are at increased risk of SARS-CoV-2 infection from both patients and other HCW with coronavirus disease 2019 (COVID-19). RT-PCR cycle threshold (Ct) values of SARS-CoV-2 ≤ 34 and the first 7-9 days of symptoms are associated with enhanced infectivity. We determined Ct values and duration of symptoms of HCW with a positive SARS-CoV-2 test. As HCW often assume their greatest risk of acquiring SARS-CoV-2 is working on a COVID-19 unit, we also determined Ct values and symptom duration of inpatients with a positive SARS-CoV-2 test. METHODS: From 6/24/2020-8/23/2020, Ct values and duration of symptoms from 13 HCW, 12 outpatients, and 28 inpatients who had a positive nasopharyngeal swab for SARS-CoV-2 were analyzed. RESULTS: Among HCW with a positive SARS-CoV-2 test, 46.2% (6/13) were asymptomatic and requested testing due to an exposure to someone with COVID-19; 83.3% (5/6) of those exposures occurred in the community rather than in the hospital. The median Ct value of HCW was 23.2, and 84.6% (11/13) had a Ct value ≤ 34. The median Ct value of 29.0 among outpatients with COVID-19 did not significantly differ from HCW. In contrast, inpatients with a positive SARS-CoV-2 test had a median Ct value of 34.0 (p = 0.003), which translated into a median ~1,000-fold lower viral load than observed in HCW. Among those with symptoms related to COVID-19, no (0/6) HCW compared to 50% (6/12) of inpatients had symptoms for at least one week (p = 0.04). CONCLUSIONS: At our institution, asymptomatic COVID-19 accounted for nearly half of the cases among HCW. Symptomatic HCW had high viral loads and short duration of symptoms, both of which are associated with peak infectivity. Infection prevention programs should educate HCW on these findings in an effort to increase adherence to the requirement to maintain six feet separation in workspaces and breakrooms, in addition to consistently wearing personal protection equipment.

McEniery, C. M., et al. (2020). "ChemoPROphyLaxIs with hydroxychloroquine For covId-19 infeCtious disease (PROLIFIC) to prevent covid-19 infection in frontline healthcare workers: A structured summary of a study protocol for a randomised controlled trial." Trials **21**(1): 604.

OBJECTIVES: PRIMARY OBJECTIVE: To determine whether chemoprophylaxis with hydroxychloroquine versus placebo increases time to contracting coronavirus disease 2019 (COVID-19) in frontline healthcare workers. SECONDARY OBJECTIVES: 1) To determine whether chemoprophylaxis with daily versus weekly dosing of hydroxychloroquine increases time to contracting COVID-19 disease in frontline healthcare workers. 2) To compare the number of COVID-19 cases between each trial arm on the basis of positive tests (as per current clinical testing methods and/or serology) 3) To compare the percentage of COVID-19 positive individuals with current testing methods versus serologically-proven COVID-19 in each trial arm 4) To compare COVID-19 disease severity in each trial arm 5) To compare recovery time from COVID-19 infection in each trial arm EXPLORATORY OBJECTIVES: 1) To determine compliance (as measured by trough pharmacokinetic hydroxychloroquine levels) on COVID-19 positive tests 2) To determine if genetic factors determine susceptibility to COVID-19 disease or response to treatment 3) To determine if blood group determines susceptibility to COVID-19 disease 4) To compare serum biomarkers of COVID-19 disease in each arm TRIAL DESIGN: Double-blind, multi-centre, 2-arm (3:3:2 ratio) randomised placebo-controlled trial PARTICIPANTS: National Health Service (NHS) workers who have direct patient contact delivering care to patients with COVID-19. Participants in the trial will be recruited from a number of NHS hospitals directly caring for patients with COVID-19. INCLUSION CRITERIA: To be included in the trial the participant MUST: 1) Have given written informed consent to participate 2) Be aged 18 years to 70 years 3) Not previously have been diagnosed with COVID-19 4) Work in a high-risk secondary or tertiary healthcare setting (hospitals accepting COVID-19 patients) with direct patient-facing care EXCLUSION CRITERIA: The presence of any of the following will mean participants are ineligible: 1) Known COVID-19 positive test at baseline (if available) 2) Symptomatic for possible COVID-19 at baseline 3) Known hypersensitivity reaction to hydroxychloroquine, chloroquine or 4-aminoquinolines 4) Known retinal disease 5) Known porphyria 6) Known chronic kidney disease (CKD; eGFR<30ml/min) 7) Known epilepsy 8) Known heart failure or conduction problems 9) Known significant liver disease (Gilbert's syndrome is permitted) 10) Known glucose-6-phosphate dehydrogenase (G6PD) deficiency 11) Currently taking any of the following contraindicated medications: Digoxin, Chloroquine, Halofantrine, Amiodarone, Moxifloxacin, Cyclosporin, Mefloquine, Praziquantel, Ciprofloxacin, Clarithromycin, Prochlorperazine, Fluconazole 12) Currently taking hydroxychloroquine or having a clinical indication for taking hydroxychloroquine 13) Currently breastfeeding 14) Unable to be followed-up during the trial 15) Current or future involvement in the active treatment phase of other interventional research studies (excluding observational/non-interventional studies) before study follow-up visit 16) Not able to use or have access to a modern phone device/web-based technology 17) Any other clinical reason which may preclude entry in the opinion of the investigator INTERVENTION AND COMPARATOR: Interventions being evaluated are: A) Daily hydroxychloroquine or B) Weekly hydroxychloroquine or C) Placebo The maximum treatment period is approximately 13 weeks per participant. Hydroxychloroquine-identical matched placebo tablets will ensure that all participants are taking the same number and dosing regimen of tablets across the three trial arms. There is no variation in the dose of hydroxychloroquine by weight. The dosing regimen for the three arms of the study (A, B, C) are described in further detail below. Arm A: Active Hydroxychloroquine (- daily dosing and placebo-matched hydroxychloroquine - weekly dosing). Form: Tablets Route: Oral. Dose and Frequency: Active hydroxychloroquine: Days 1-2: Loading phase - 400mg (2 x 200mg tablets) taken twice a day for 2 days Days 3 onwards: Maintenance Phase - 200mg (1 x 200mg tablet) taken once daily, every day for 90 days (~3 months) Matched Placebo hydroxychloroquine: Days 3 onwards: Maintenance Phase - 2 tablets taken once a week on the same day each week (every 7(th) day) for 90 days (~3 months) Arm B: Active Hydroxychloroquine (- weekly dosing and placebo matched hydroxychloroquine - daily dosing.) Form: Tablets Route: Oral. Dose and Frequency: Active hydroxychloroquine: Days 1-2: Loading Phase - 400mg (2 x 200mg tablets) taken twice daily for 2 days Days 3 onwards: Maintenance Phase - 400mg (2 x 200mg tablets) taken once a week on the same day each week (every 7(th) day) for 90 days (~3 months) Matched Placebo hydroxychloroquine: Days 3 onwards: Maintenance Phase - 1 tablet taken once daily for 90 days (~3 months) Arm C: Matched placebo Hydroxychloroquine (- daily dosing and matched placebo hydroxychloroquine - weekly dosing.) Form: Table. Route: Oral. Frequency: Matched placebo hydroxychloroquine - daily dosing: Days 1-2: Loading Phase - 2 tablets taken twice daily for 2 days Days 3 onwards: Maintenance Phase - 1 tablet taken once daily for 90 days (~3 months) Matched placebo hydroxychloroquine - weekly dosing: Days 3 onwards: Maintenance Phase - 2 tablets taken once a week on the same day each week (every 7th day) for 90 days (~3 months) A schematic of the dosing schedule can be found in the full study protocol (Additional File 1). MAIN OUTCOMES: Time to diagnosis of positive COVID-19 disease (defined by record of date of symptoms onset and confirmed by laboratory test) RANDOMISATION: Participants will be randomised to either hydroxychloroquine dosed daily with weekly placebo, HCQ dosed weekly with daily placebo, or placebo dosed daily and weekly. Randomisation will be in a 3:3:2 ratio [hydroxychloroquine-(daily), hydroxychloroquine-(weekly), placebo], using stratified block randomisation. Random block sizes will be used, and stratification will be by study site. BLINDING (MASKING): Participants and trial investigators consenting participants, delivering trial assessments and procedures will be blinded to intervention. NUMBERS TO BE RANDOMISED (SAMPLE SIZE): A sufficient number of participants will be enrolled so that approximately 1000 participants in total will have data suitable for the primary statistical analysis. It is anticipated that approximately 1,200 participants will need to be enrolled in total, to allow for a 20% dropout over the period of the trial. This would result in approximately 450:450:300 participants randomised to hydroxychloroquine daily, hydroxychloroquine weekly+daily matched placebo or matched-placebo daily and weekly. TRIAL STATUS: V 1.0, 7(th) April 2020 EU Clinical Trials Register EudraCT Number: 2020-001331-26 Date of registration: 14(th) April 2020 Trial registered before first participant enrolment. Trial site is Cambridge University Hospitals NHS Foundation Trust. Recruitment started on 11(th) May 2020. It is anticipated that the trial will run for 12 months. The recruitment end date cannot yet be accurately predicted. FULL PROTOCOL: The full protocol is attached as an additional file, accessible from the Trials website (Additional file 1). In the interest of expediting dissemination of this material, the familiar formatting has been eliminated; this Letter serves as a summary of the key elements of the full protocol. The study protocol has been reported in accordance with the Standard Protocol Items: Recommendations for Clinical Interventional Trials (SPIRIT) guidelines (Additional file 2).

McGriff, J. A. and L. Denny (2020). "What COVID-19 Reveals about the Neglect of WASH within Infection Prevention in Low-Resource Healthcare Facilities." Am J Trop Med Hyg **103**(5): 1762-1764.

The highly infectious nature of the SARS-CoV-2 virus requires rigorous infection prevention and control (IPC) to reduce the transmission of COVID-19 within healthcare facilities, but in low-resource settings, the lack of water access creates a perfect storm for low-handwashing adherence, ineffective surface decontamination, and other environmental cleaning functions that are critical for IPC compliance. Data from the WHO/UNICEF Joint Monitoring Programme show that one in four healthcare facilities globally lacks a functional water source on premises (i.e., basic water service); in sub-Saharan Africa, half of all healthcare facilities have no basic water services. But even these data do not tell the whole story, other water, sanitation, and hygiene (WASH) assessments in low-resource healthcare facilities have shown the detrimental effects of seasonal or temporary water shortages, nonfunctional water infrastructure, and fluctuating water quality. The rapid spread of COVID-19 forces us to reexamine prevailing norms within national health systems around the importance of WASH for quality of health care, the prioritization of WASH in healthcare facility investments, and the need for focused, cross-sector leadership and collaboration between WASH and health professionals. What COVID-19 reveals about infection prevention in low-resource healthcare facilities is that we can no longer afford to "work around" WASH deficiencies. Basic WASH services are a fundamental prerequisite to compliance with the principles of IPC that are necessary to protect patients and healthcare workers in every setting.

McKinney, E. L., et al. (2020). "COVID-19, disability and the context of healthcare triage in South Africa: Notes in a time of pandemic." Afr J Disabil **9**: 766.

During disasters, when resources and care are scarce, healthcare workers are required to make decisions and prioritise which patients receive life-saving resources over others. To assist healthcare workers in standardising resources and care, triage policies have been developed. However, the current COVID-19 triage policies and practices in South Africa may exclude or disadvantage many disabled people, especially people with physical and intellectual impairments, from gaining intensive care unit (ICU) access and receiving ventilators if becoming ill. The exclusion of disabled people goes against the principles established in South Africa's Constitution, in which all people are regarded as equal, have the right to life and inherent dignity, the right to access healthcare, as well as the protection of dignity. In addition, the triage policy contravenes the United Nations Convention on the Rights of Persons with Disabilities, which the South African government has signed and ratified. This article raises debates about whose lives matter and whose lives are 'worth' saving over others, and although the focus is on South Africa, the issues may be relevant to other countries where life-saving resources are being rationed.

Mecham, J. C., et al. (2020). "Utility of Tracheostomy in Patients With COVID-19 and Other Special Considerations." Laryngoscope **130**(11): 2546-2549.

INTRODUCTION: Patients who become severely ill from coronavirus disease 2019 (COVID-19) have a high likelihood of needing prolonged intubation, making tracheostomy a likely consideration. The infectious nature of COVID-19 poses an additional risk of transmission to healthcare workers that should be taken into consideration. METHODS: We explore current literature and recommendations for tracheostomy in patients with COVID-19 and look back at previous data from severe acute respiratory syndrome coronavirus 1 (SARS-CoV-1), the virus responsible for the SARS outbreak of 2003. RESULTS: Given the severity and clinical uncertainty of patients with COVID-19 and the increased risk of transmission to clinicians, careful consideration should be taken prior to performing tracheostomy. If tracheostomy is performed, we recommend a bedside approach to limit exposure time and number of exposed personnel. Bronchoscopy use with a percutaneous approach should be limited in order to decrease viral exposure. CONCLUSION: Thorough preprocedural planning, use of experienced personnel, enhanced personal protective equipment where available, and a thoughtful anesthesia approach are instrumental in maximizing positive patient outcomes while successfully protecting the safety of healthcare personnel. Laryngoscope, 130:2546-2549, 2020.

Meda, M., et al. (2020). "Unintended consequences of long-sleeved gowns in a critical care setting during the COVID-19 pandemic." J Hosp Infect **106**(3): 605-609.

At the peak of the coronavirus disease 2019 (COVID-19) pandemic, hand hygiene audits indicated decreased compliance in a 12-bed critical care (CC) area with ventilated COVID-19 patients, where staff used personal protective equipment (PPE), including sessional use of long-sleeved gowns in accordance with the recommendations of Public Health England. There was also a cluster of three central venous catheter (CVC) infections along with increases in the number of patients from whom enteric Gram-negative bacteria (GNB) were isolated from sterile sites. Environmental sampling of near-patient surfaces and frequently touched sites demonstrated that 11.5% of areas were contaminated with enteric GNB in the COVID-19 CC area, compared with 2.6% and 2.7% in COVID-19 and non-COVID-19 general wards, respectively. Following a risk assessment, hospital policy was changed to replace long-sleeved gowns with short-sleeved gowns. The CC unit underwent enhanced cleaning with hypochlorite-based disinfectant and was resampled 8 days later. On resampling, no GNB were isolated from the CC unit. Following this change in PPE, hand hygiene compliance returned to baseline standards and no further CVC infections were identified. Staff reported a preference for short-sleeved gowns. No evidence currently exists that PPE beyond that recommended for pandemic influenza (respiratory protection plus standard PPE) adds to the protection of healthcare workers (HCWs) from severe acute respiratory syndrome coronavirus-2. Long-sleeved gowns prevent HCWs performing hand hygiene effectively. While it is imperative that HCWs are adequately protected, protection of patients from infection hazards is equally important. Further studies are necessary to establish risks from PPE to inform a review of current guidance.

Mekhemar, M., et al. (2021). "The Psychological Impact of the COVID-19 Pandemic on Dentists in Germany." J Clin Med **10**(5).

Since the announcement of the coronavirus 2019 (COVID-19) outbreak as a pandemic, several studies reported increased psychological distress among healthcare workers. In this investigation, we examined the association between psychological outcomes and various factors among German dentists. Dentists from all German federal states were invited to participate in this study through a self-administered online questionnaire between July and November 2020. This questionnaire collected information on demographics, Depression Anxiety Stress Scales (DASS-21), and the Impact of Events Scale-Revised (IES-R) instrument. The associations displayed between demographic and psychological outcomes of depression, anxiety, stress, intrusion, avoidance, and hyperarousal were evaluated. Seven-hundred-and-thirty-two dentists participated in the survey and reported overall scores of (4.88 ± 4.85), (2.88 ± 3.57), (7.08 ± 5.04), (9.12 ± 8.44), (10.68 ± 8.88) and (10.35 ± 8.68) for depression, anxiety, stress, intrusion, avoidance, and hyperarousal, respectively. For females, being between 50-59 years of age, being immune deficient or chronically ill, working at a dental practice, and considering the COVID-19 pandemic a financial hazard were reported as significant associated factors (p < 0.05) with higher DASS-21 and IES-R scores. These findings underline the aspects which need to be taken into attention to protect the mental wellbeing of dentists in Germany during the crisis.

Mekonen, E., et al. (2020). "The Psychological Impact of COVID-19 Outbreak on Nurses Working in the Northwest of Amhara Regional State Referral Hospitals, Northwest Ethiopia." Psychol Res Behav Manag **13**: 1353-1364.

INTRODUCTION: The psychosocial consequences of the coronavirus pandemic are serious for health professionals including nurses because of a higher level of exposure. Nurses often face huge psychological pressure as a result of workload, long hours, and working in a high-risk environment. This study aimed to assess the prevalence and associated factors of anxiety, depression, and stress among nurses working in northwest Amhara referral hospitals. METHODS: Institution-based cross-sectional study was conducted from 25th September to 20th October 2020. A total of 302 nurses were selected using a simple random sampling technique. A structured pretested self-administered questionnaire was used to collect data. The data were entered into EpiData version 3 and analyzed using SPSS version 21. A binary logistic regression model was used to compute bivariable and multivariable analyses. RESULTS: The prevalence of anxiety, depression, and stress among nurses was 69.6%, 55.3%, and 20.5%, respectively. Unavailability of a guideline, fear of infecting family, and having chronic diseases increase the risk of developing anxiety. Nurses who did not have a guideline, received negative feedback from families, had a history of mental disorders, and chronic diseases have a higher odds of depression. Working in the night shift, lack of training, fear of infecting family, negative feedback from families, presence of confirmed/suspected cases in the family, and having chronic diseases increase the risk of developing stress. CONCLUSION: More than two-thirds, more than half, and nearly one-fifth of the nurses had anxiety, depression, and stress, respectively. It is better to create awareness for the community, avail a guideline, train nurses, and give special attention to nurses with chronic disease and a history of mental disorders to minimize the psychological impact of the COVID-19 pandemic on nurses and protect their mental health. The government shall develop and implement national programs for occupational health and safety, prevent violence in the workplace, improve psychological well-being, and protect from physical and biological hazards to take care of the mental health of healthcare professionals during this pandemic.

Melidis, C. and M. Vantsos (2020). "[Comment] Ethical and practical considerations on cancer recommendations during COVID-19 pandemic." Mol Clin Oncol **13**(3): 5.

National and international authorities and societies have recently published important cancer treatment recommendations in order to propose extra measures that should be taken during the COVID-19 pandemic, such as prioritisation of intend-to-cure treatments and younger patients, omission of non-urgent cases, and reduction of personnel present. These measures raise important ethical considerations, since they prioritise protection of Health Systems and Professionals without seemingly taking cancer patient feelings of stress into consideration. This could lead to an erosion of the physician-patient relationship, which is considered the core element of medical ethics. Moreover, they raise practical concerns about the continuous education of Health Professionals, the status of reference centres and the evaluation of the hitherto cancer treatments.

Mendelson, D., et al. (2020). "Legal Implications of Personal Protective Equipment Use When Treating Patients for COVID-19 (SARS-CoV-2)." J Law Med **27**(4): 856-864.

Front-line health care personnel, including anaesthetists, otolaryngologists, and other health professionals dealing with acute cases of coronavirus, face a high risk of infection and thus mortality. The scientific evidence establishes that to protect them, hospital protocols should require that wearing of the highest levels of personal protective equipment (PPE) be available for doctors and nurses performing aerosol-generating procedures, such as intubation, sputum induction, open suctioning of airways, bronchoscopy, etc. of COVID-19 patients. Although several international bodies have issued recommendations for a very high-level PPE to be used when these procedures are undertaken, the current PPE guidelines in Australia have tended to be more relaxed, and hospital authorities relying on them might not comply with legal obligations to their employee health care workers. Failure to provide high-level PPE in many hospitals is of concern for a large number of health care workers; this article examines the scientific literature on the topic and provides a legal perspective on hospital authorities' possible liability in negligence.

Mermel, L. A. (2020). "Respiratory protection for healthcare workers caring for COVID-19 patients." Infect Control Hosp Epidemiol **41**(9): 1064-1065.

Mersha, A., et al. (2021). "Health professionals practice and associated factors towards precautionary measures for COVID-19 pandemic in public health facilities of Gamo zone, southern Ethiopia: A cross-sectional study." PLoS One **16**(3): e0248272.

INTRODUCTION: Coronavirus disease-2019 (COVID-19) is a highly contagious acute respiratory disease, which caused by a novel coronavirus. The disease disrupts health systems and resulting in social, political, and economic crises. Health professionals are in front of this pandemic and always work in a high-risk environment. The best prevention for COVID-19 is avoiding exposure to the virus. Some studies reported health professional's practice of precautionary measures for COVID-19. Nevertheless, a few have identified factors affecting. As such, this study aimed to fill those research gaps in the study setting. METHODS: In this cross-sectional study, 428 health professionals involved from the public health facilities of the Gamo zone, southern Ethiopia. A simple random sampling method employed, and the data collected by the interviewer-administered Open Data Kit survey tool and observational checklist. The data analyzed in Stata version 15, and a binary logistic regression model used to identify factors. In this study, a statistically significant association declared at P< 0.05. RESULTS: In this study, 35.3% (95%CI: 30.7%, 39.8%) of health professionals' had a good practice on precautionary measures for the COVID-19 pandemic. Use hand sanitizer or wash hands continuously with soap and water (68.9%), cover nose and mouth with a tissue during sneezing or coughing (67.3%), and use facemask in crowds (56.8%) were the most common practice reported by study participants. Marital status, being married (AOR = 1.84, 95%CI: 1.06, 3.18), good knowledge on the COVID-19 pandemic (AOR = 2.02, 95%CI: 1.02, 3.18), and positive attitude towards precautionary measures for the COVID-19 were factors showed significant association with the practice. CONCLUSIONS: The magnitude of good practice of precautionary measures for the COVID-19 pandemic among health professionals was low. As such, different interventions to improve the knowledge and attitude of health professionals in the health care system are highly needed to boost the practice and to advance service delivery.

Mersha, A., et al. (2021). "Perceived barriers to the practice of preventive measures for COVID-19 pandemic among health professionals in public health facilities of the Gamo zone, southern Ethiopia: a phenomenological study." BMC Public Health **21**(1): 199.

BACKGROUND: Novel coronavirus is a global pandemic and killed many individuals, including health care professionals. It caused stress on the health care system of all countries. Presently, studies are emerging regarding the COVID-19 pandemic in different aspects. However, a few have explored barriers that affecting the practice of preventive measures for the COVID-19. As such, the study aimed to fill these research gaps in the study setting. METHODS: A semi-structured interview guide was used to conduct this phenomenological study among 16 key informants. Key informants were recruited by the purposive sampling method. To analyze that data, thematic content analysis was employed by using an inductive approach in NVivo 12 Pro software. RESULTS: In this study, six main themes were identified with the sub-themes. Overview of COVID-19 pandemic (with the six sub-themes), consequences (with the two sub-themes), perceived practice (with four sub-themes), perceived barriers (with four sub-themes), newfangled activities (with three sub-themes), and suggestion for improvement (with seven sub-themes) were the major themes. The participants perceived the influence of shortage of personal protective equipment and solutions for hand hygiene, negligence and ignorance, inadequate infrastructure, lack of training, and lack of attention and recognition for the staff on the practice of preventive measures. CONCLUSIONS: This study showed a gap in preventive measure practices for the COVID-19 in the health care system. Community influences, health care provider related barriers, institutional barriers, and lack of communication and support affect the practice. Hence, attention should give to fulfill the necessary supplies in the health facilities, improve the infrastructures, and equip health professionals by providing capacity-building activities. Besides, health care workers must recognize, and attention is needed.

Metelmann, I. B. and A. Busemann (2020). "Elective surgery in times of COVID-19: A two-centre analysis of postponed operations and disease-related morbidity and mortality." Z Evid Fortbild Qual Gesundhwes **158-159**: 62-65.

INTRODUCTION: COVID-19 had an impact on the whole range of worldwide medical services. Due to the high risk of in-hospital transmission and disproportionate perioperative rates of morbidity and mortality in occult COVID-19 patients surgeons were faced with the challenging triage of surgeries into emergency, urgent and elective. The present study investigates postponed elective surgery and its impact on the medical condition of patients in two high-volume departments of general, visceral, thoracic, transplant and vascular surgery. METHODS: Operations that have been postponed due to COVID-19 were recorded in the Departments of General-, Visceral-, Thoracic- and Vascular Surgery at the University Hospitals of Leipzig and Greifs-wald. Data was analysed descriptively concerning patient outcomes as well as emergency admissions and surgeries. RESULTS: In the Leipzig and Greifswald University Hospitals 89 and 92 elective surgeries were postponed, respectively. No patient needed an extension of surgical procedure when eventually operated. One patient with extensive obesity died early during the suspension period due to cardiac complications. Four patients needed emergency admission to hospital one of whom required urgent surgery. In neither of the two surgical departments did a patient acquire a nosocomial infection with COVID-19. DISCUSSION: While medical consequences of COVID-19 seem multidimensional and severe, our data indicate that the short-term postponement of elective surgery did not cause an unproportional increase of morbidity and mortality. Although the restrictions may have been fear-driven, given no confirmed cases and thus no concrete risk of infection, the early and well-coordinated action may have provided protection from uncontrolled interruption of medical services by loss of medical workforce or capacity. CONCLUSION: Well-organized and early suspension of elective surgery had no disproportionate impact on patient outcomes while averting nosocomial transmission of COVID-19.

Publisher: Abstract available from the publisher.

ger

Michel-Kabamba, N., et al. (2020). "Occupational COVID-19 Prevention among Congolese Healthcare Workers: Knowledge, Practices, PPE Compliance, and Safety Imperatives." Trop Med Infect Dis **6**(1).

The SARS-CoV-2 (COVID-19) pandemic has had a tremendous impact on the functionality of health systems and world affairs. We assessed knowledge, attitudes, and practices (KAPs) of healthcare workers (HCWs) in the Democratic Republic of the Congo (DRC). This was a cross-sectional study conducted in 23 referral hospitals located in three towns of the DRC (Lubumbashi, Kamina, Mbuji-Mayi). In total, 613 HCWs were surveyed using the World Health Organization's (WHO's) "Exposure Risk Assessment in the Context of COVID-19" questionnaire. Participants included medical doctors (27.2%) and other categories of HCWs (72.8%). The mean age was 40.3 ± 11.7 years. Over 80% (range: 83-96%) of respondents had sufficient knowledge on each of the three domains: COVID-19 symptoms, disease transmission, and patient care approach. However, attitudes and practices scores were relatively low. Only 27.7% of HCWs were willing to receive a COVID-19 vaccine when it is available, whereas 55% of HCWs complied with good practices; 49.4% wore masks consistently and, surprisingly, only 54.9% used personal protective equipment (PPE) consistently at work and during contact with patients. Knowledge level was positively associated with the use of social media as a primary source of COVID-19-related information and the category of residence, with HCWs from towns already affected by the COVID-19 epidemic being more likely to have positive attitudes (adjusted OR, 1.64; 95%CI, 1.32-2.20) and comply with good practices (aOR, 2.79; 95%CI, 1.93-4.06). This study showed that most Congolese HCWs had sufficient knowledge on COVID-19, whereas the majority did not comply with consistent PPE use. The government of the DRC should urgently take major steps in capacity building for HCWs in outbreak preparedness and supplying hospitals with PPE.

Michno, D. A., et al. (2021). "How can we help? Medical students' views on their role in the COVID-19 pandemic." J Public Health (Oxf).

BACKGROUND: The ongoing COVID-19 pandemic has resulted in a sharp rise in demand for healthcare workers worldwide. This has been coupled with reduced numbers of available medical professionals due to confirmed or suspected infections with SARS-CoV-2. To counteract these shortages, governments of several countries have considered the enrolment of medical students into the workforce in order to help to tackle the ongoing crisis. METHODS: Questionnaire-based study assessing the perceived role of medical students in assisting in the COVID-19 pandemic. The primary aim was to determine factors contributing to the willingness of medical students to actively assist in the pandemic. The secondary aim was to evaluate their perspectives regarding the associated changes in medical education. RESULTS: Out of 760 responses, 71.18% of medical students were willing to assist the medical workforce during the pandemic. Clinical year students were more likely to help in a medical capacity than pre-clinical students (OR = 0.384, 95%CI [0.167, 0.883, P < 0.05)]. Respondents concerned about their own well-being were less likely to engage in clinical work (OR = 0.680, 95%CI [0.491, 0.941], P < 0.020). Students who agreed that online lectures will negatively impact their education were inclined to think that the academic year should be extended (95%CI [0.065, 0.271], P = 0.001). CONCLUSIONS: Most students are willing to help in both a medical and non-medical capacity. Their primary concerns when working in a medical setting are the risk of infecting their relatives and patients, lack of protective equipment and necessary knowledge as well as legal uncertainty whilst working without a medical qualification.

Milazzo, L., et al. (2021). "Dynamics of the seroprevalence of SARS-CoV-2 antibodies among healthcare workers at a COVID-19 referral hospital in Milan, Italy." Occup Environ Med.

OBJECTIVES: Healthcare workers (HCWs) are at high risk of developing SARS-CoV-2 infection. The aim of this single-centre prospective study was to evaluate the trend of SARS-CoV-2 seroprevalence in HCWs working at the primary referral centre for infectious diseases and bioemergencies (eg, COVID-19) in Northern Italy and investigate the factors associated with seroconversion. METHODS: Six hundred and seventy-nine HCW volunteers were tested for anti-SARS-CoV-2 antibodies three times between 4 March and 27 May 2020 and completed a questionnaire covering COVID-19 exposure, symptoms and personal protective equipment (PPE) training and confidence at each time. RESULTS: SARS-CoV-2 seroprevalence rose from 3/679 to 26/608 (adjusted prevalence: 0.5%, 95% CI 0.1 to 1.7% and 5.4%, 95% CI 3.6 to 7.9, respectively) between the first two time points and then stabilised, in line with the curve of the COVID-19 epidemic in Milan. From the first time point, 61.6% of the HCWs had received training in the use of PPE and 17 (61.5%) of those who proved to be seropositive reported symptoms compatible with SARS-CoV-2 infection. Contacts with ill relatives or friends and self-reported symptoms were independently associated with an increased likelihood of seroconversion (p<0.0001 for both), whereas there was no significant association with professional exposure. CONCLUSION: The seroprevalence of SARS-CoV-2 among the HCWs at our COVID-19 referral hospital was low at the time of the peak of the epidemic. The seroconversions were mainly attributable to extrahospital contacts, probably because the hospital readily adopted effective infection control measures. The relatively high number of asymptomatic seropositive HCWs highlights the need to promptly identify and isolate potentially infectious HCWs.

Miles, A., et al. (2021). "Dysphagia Care Across the Continuum: A Multidisciplinary Dysphagia Research Society Taskforce Report of Service-Delivery During the COVID-19 Global Pandemic." Dysphagia **36**(2): 170-182.

At the time of writing this paper, there are over 11 million reported cases of COVID-19 worldwide. Health professionals involved in dysphagia care are impacted by the COVID-19 pandemic in their day-to-day practices. Otolaryngologists, gastroenterologists, rehabilitation specialists, and speech-language pathologists are subject to virus exposure due to their proximity to the aerodigestive tract and reliance on aerosol-generating procedures in swallow assessments and interventions. Across the globe, professional societies and specialty associations are issuing recommendations about which procedures to use, when to use them, and how to reduce the risk of COVID-19 transmission during their use. Balancing safety for self, patients, and the public while maintaining adequate evidence-based dysphagia practices has become a significant challenge. This paper provides current evidence on COVID-19 transmission during commonly used dysphagia practices and provides recommendations for protection while conducting these procedures. The paper summarizes current understanding of dysphagia in patients with COVID-19 and draws on evidence for dysphagia interventions that can be provided without in-person consults and close proximity procedures including dysphagia screening and telehealth.

Miles, L. F., et al. (2020). "The design and manufacture of 3D-printed adjuncts for powered air-purifying respirators." Anaesth Rep **8**(2): e12055.

Spurred in part by literature published in the immediate aftermath of the severe acute respiratory syndrome epidemic in 2003, powered air-purifying respirators have seen increased use worldwide during the COVID-19 pandemic. Whereas these devices provide excellent protection of the user, there is an added element of risk during doffing and cleaning of the device. An additional layer of barrier protection, in the form of a polypropylene gown, to be worn over the hood and motor belt, can be used to minimise this risk. However, the device entrains air perpendicular to the lie of the gown, resulting in the impermeable material being sucked into the air intake, and partial occlusion of flow. In this report, we describe a clinical-academic partnership whereby a bespoke filter guard was designed to disrupt airflow and prevent gown entrainment, thereby enabling full barrier protection of both the device and user. This intervention was simple, cheap, scalable and able to be mass produced.

Miller, L. E., et al. (2020). "Considerations for the otolaryngologist in the era of COVID-19: a review of the literature." Curr Opin Otolaryngol Head Neck Surg **28**(4): 228-234.

PURPOSE OF REVIEW: The SARS-CoV-2 coronavirus pandemic, referred to as COVID-19, has spread throughout the globe since its first case in China in December 2019, leaving a significant number of people infected and clinically ill. The purpose of this review is to provide the current known clinical characteristics of and management for COVID-19 as it relates to otolaryngology. RECENT FINDINGS: COVID-19 is a highly transmissible respiratory disease with common presenting symptoms of fever, cough, and fatigue. In the absence of available vaccines or antiviral therapies, symptomatic and respiratory support is the current standard of therapy. Measures to prevent further transmission have been enacted globally including social distancing and cancellation of public events. Given elevated viral load in the upper aerodigestive tract, extra precautions in patients with otolaryngology needs have been recommended for protection of both healthcare workers and patients. SUMMARY: Otolaryngologists face unique risk from COVID-19. Maintaining appropriate preventive health measures and remaining updated on institutional clinical guidelines is paramount for both caretaker safety and patient care.

Mira, J. J., et al. (2020). "Preventing and Addressing the Stress Reactions of Health Care Workers Caring for Patients With COVID-19: Development of a Digital Platform (Be + Against COVID)." JMIR Mhealth Uhealth **8**(10): e21692.

BACKGROUND: COVID-19 became a major public health concern in March 2020. Due to the high rate of hospitalizations for COVID-19 in a short time, health care workers and other involved staff are subjected to a large workload and high emotional distress. OBJECTIVE: The objective of this study is to develop a digital tool to provide support resources that might prevent and consider acute stress reactions in health care workers and other support staff due to the COVID-19 pandemic. METHODS: The contents of the digital platform were created through an evidence-based review and consensus conference. The website was built using the Google Blogger tool. The Android version of the app was developed in the Java and XML languages using Android Studio version 3.6, and the iOS version was developed in the Swift language using Xcode version 11.5. The app was evaluated externally by the Andalusian Agency for Healthcare Quality. RESULTS: We detected the needs and pressing situations of frontline health care workers, and then, we proposed a serial of recommendations and support resources to address them. These resources were redesigned using the feedback received. A website in three different languages (Spanish, English, and Portuguese) and a mobile app were developed with these contents, and the AppSaludable Quality Seal was granted to the app. A specific self-report scale to measure acute stress and additional tools were included to support the health care workforce. This instrument has been used in several Latin American countries and has been adapted considering cultural differences. The resources section of the website was the most visited with 18,516 out of 68,913 (26.9%) visits, and the "Self-Report Acute Stress Scale" was the most visited resource with 6468 out of 18,516 (34.9%) visits. CONCLUSIONS: The Be + against COVID platform (website and app) was developed and launched to offer a pool of recommendations and support resources, which were specifically designed to protect the psychological well-being and the work morale of health care workers. This is an original initiative different from the usual psychological assistance hotlines.

Missoni, E., et al. (2021). "Face Masks for All and All for Face Masks in the COVID-19 Pandemic: Community Level Production to Face the Global Shortage and Shorten the Epidemic." Disaster Med Public Health Prep **15**(1): e29-e33.

The current coronavirus disease 2019 (COVID-19) pandemic caused a global shortage of medical masks, leaving most exposed health personnel without appropriate protection.Since the beginning of the outbreak, the World Health Organization WHO) has revised several times the recommendations on general use of facemasks. Until recently, WHO recommended to limit the use of facemasks to symptomatic people and advised against off-standard solutions. Moreover, recommendations differ among and within countries, causing public confusion and individual initiative.There is wide consensus that universal appropriate use of masks may contribute both to contain the epidemic and to reduce the burden on national procurement, if a community production approach is followed. Especially in low-middle income countries, due to the scarce capacity of national industrial production or import, the use of masks produced at community level may become the only viable option. For the purpose ad hoc guidelines will be needed.Current knowledge and experience call for further and updated review of global and national guidelines to provide clear and consistent criteria to ensure the widest availability and appropriate use of facial protection, bearing in mind populations in socio-economic disadvantaged settings.

Mittal, A., et al. (2020). "Gargle lavage as a viable alternative to swab for detection of SARS-CoV-2." Indian J Med Res **152**(1 & 2): 77-81.

BACKGROUND & OBJECTIVES: Nasopharyngeal and oropharyngeal swab (NPS and OPS) collection is widely accepted as the preferred method for obtaining respiratory samples. However, it has certain disadvantages which may be overcome by gargling. The primary objective of this study was to assess agreement between gargle lavage and swab as an appropriate respiratory sample for the detection of SARS-CoV-2. The secondary objective was to assess the patient acceptability of the two sampling methods. METHODS: It was a cross-sectional study done at a tertiary care hospital in New Delhi, India, on 50 confirmed COVID-19 patients. Paired swab (NPS and OPS) and gargle samples were taken within 72 h of their diagnosis. Samples were processed by reverse transcription-polymerase chain reaction (RT-PCR) for detection of SARS-CoV-2. Post-sample collection, a 10-point scale was administered to assess the level of discomfort with either of the collection methods. RESULTS: All gargle samples were positive and comparable to their corresponding swab samples irrespective of the symptoms and duration of illness. The cycle threshold (C(t)) values for gargle samples were slightly higher but comparable to those of swabs. Bland-Altman plot showed good agreement between the two methods. Majority (72%) of the patients reported moderate-to-severe discomfort with swab collection in comparison to 24 per cent reporting only mild discomfort with gargle collection. INTERPRETATION & CONCLUSIONS: Our preliminary results show that the gargle lavage may be a viable alternative to swabs for sample collection for the detection of SARS-CoV-2. Adoption of gargle lavage for sample collection will have a significant impact as it will enable easy self-collection, relieve healthcare workers and also lead to substantial cost savings by reducing the need for swabs and personal protective equipment.

Mizukoshi, A., et al. (2021). "Assessing the risk of COVID-19 from multiple pathways of exposure to SARS-CoV-2: Modeling in health-care settings and effectiveness of nonpharmaceutical interventions." Environ Int **147**: 106338.

We assessed the risk of COVID-19 infection in a healthcare worker (HCW) from multiple pathways of exposure to SARS-CoV-2 in a health-care setting of short distance of 0.6 m between the HCW and a patient while caring, and evaluated the effectiveness of a face mask and a face shield using a model that combined previous infection-risk models. The multiple pathways of exposure included hand contact via contaminated surfaces and an HCW's fingers with droplets, droplet spray, and inhalation of inspirable and respirable particles. We assumed a scenario of medium contact time (MCT) and long contact time (LCT) over 1 day of care by an HCW. SARS-CoV-2 in the particles emitted by coughing, breathing, and vocalization (only in the LCT scenario) by the patient were considered. The contribution of the risk of infection of an HCW by SARS-CoV-2 from each pathway to the sum of the risks from all pathways depended on virus concentration in the saliva of the patient. At a virus concentration in the saliva of 10(1)-10(5) PFU mL(-1) concentration in the MCT scenario and 10(1)-10(4) PFU mL(-1) concentration in the LCT scenario, droplet spraying was the major pathway (60%-86%) of infection, followed by hand contact via contaminated surfaces (9%-32%). At a high virus concentration in the saliva (10(6)-10(8) PFU mL(-1) in the MCT scenario and 10(5)-10(8) PFU mL(-1) in the LCT scenario), hand contact via contaminated surfaces was the main contributor (41%-83%) to infection. The contribution of inhalation of inspirable particles was 4%-10% in all assumed cases. The contribution of inhalation of respirable particles increased as the virus concentration in the saliva increased, and reached 5%-27% at the high saliva concentration (10(7) and 10(8) PFU mL(-1)) in the assumed scenarios using higher dose-response function parameter (0.246) and comparable to other pathways, although these were worst and rare cases. Regarding the effectiveness of nonpharmaceutical interventions, the relative risk (RR) of an overall risk for an HCW with an intervention vs. an HCW without intervention was 0.36-0.37, 0.02-0.03, and <4.0 × 10(-4) for a face mask, a face shield, and a face mask plus shield, respectively, in the likely median virus concentration in the saliva (10(2)-10(4) PFU mL(-1)), suggesting that personal protective equipment decreased the infection risk by 63%->99.9%. In addition, the RR for a face mask worn by the patient, and a face mask worn by the patient plus increase of air change rate from 2 h(-1) to 6 h(-1) was <1.0 × 10(-4) and <5.0 × 10(-5), respectively in the same virus concentration in the saliva. Therefore, by modeling multiple pathways of exposure, the contribution of the infection risk from each pathway and the effectiveness of nonpharmaceutical interventions for COVID-19 were indicated quantitatively, and the importance of the use of a face mask and shield was confirmed.

Moghadas, S. M., et al. (2021). "The impact of vaccination on COVID-19 outbreaks in the United States." Clin Infect Dis.

BACKGROUND: Global vaccine development efforts have been accelerated in response to the devastating COVID-19 pandemic. We evaluated the impact of a 2-dose COVID-19 vaccination campaign on reducing incidence, hospitalizations, and deaths in the United States (US). METHODS: We developed an agent-based model of SARS-CoV-2 transmission and parameterized it with US demographics and age-specific COVID-19 outcomes. Healthcare workers and high-risk individuals were prioritized for vaccination, while children under 18 years of age were not vaccinated. We considered a vaccine efficacy of 95% against disease following 2 doses administered 21 days apart achieving 40% vaccine coverage of the overall population within 284 days. We varied vaccine efficacy against infection, and specified 10% pre-existing population immunity for the base-case scenario. The model was calibrated to an effective reproduction number of 1.2, accounting for current non-pharmaceutical interventions in the US. RESULTS: Vaccination reduced the overall attack rate to 4.6% (95% CrI: 4.3% - 5.0%) from 9.0% (95% CrI: 8.4% - 9.4%) without vaccination, over 300 days. The highest relative reduction (54-62%) was observed among individuals aged 65 and older. Vaccination markedly reduced adverse outcomes, with non-ICU hospitalizations, ICU hospitalizations, and deaths decreasing by 63.5% (95% CrI: 60.3% - 66.7%), 65.6% (95% CrI: 62.2% - 68.6%), and 69.3% (95% CrI: 65.5% - 73.1%), respectively, across the same period. CONCLUSIONS: Our results indicate that vaccination can have a substantial impact on mitigating COVID-19 outbreaks, even with limited protection against infection. However, continued compliance with non-pharmaceutical interventions is essential to achieve this impact.

Mohammadi, F., et al. (2020). "Health Professionals' Perception of Psychological Safety in Patients with Coronavirus (COVID-19)." Risk Manag Healthc Policy **13**: 785-794.

BACKGROUND: Medical personnel act as the protectors of people's health by preventing, controlling, and treating emerging diseases, including the coronavirus infection. COVID-19 is a highly infectious and contagious disease which has presented the Iranian healthcare system with a variety of clinical challenges. There is a lack of research on clinical challenges in health crises especially those caused by emerging diseases, hence a need for more exploration of these clinical challenges and dilemmas. The present study aims to determine the different aspects of health professionals' perception of the psychological safety of patients infected with the coronavirus (COVID-19). METHODS: The present study is a qualitative work which uses conventional content analysis. The participants were 17 health professionals who were responsible for COVID-19 patients and met the inclusion criteria. They were selected via purposeful sampling. The study lasted from February to March 2020. Data were collected through semi-structured, individual interviews which were conducted via video call. The collection of data was kept up to the point of saturation. The collected data were analyzed using the conventional content analysis method. RESULTS: Three themes such as "respect for dignity", "comprehensive support" and "peaceful environment" with 11 categories were extracted from the findings of the study. CONCLUSION: According to the results of the present study, patients infected with the coronavirus should be given care in a peaceful environment where they receive comprehensive support and have their dignity respected. Such conditions can guarantee the psychological safety of these patients and produce positive behavioral results on their part. Thus, it is essential that the cultural, professional, and organizational prerequisites of protecting all the dimensions of the psychological safety of these patients be provided.

Mohseni, M., et al. (2020). "Diagnosis of a Case of Suspected COVID-19 Using Telemedicine Technology in the Emergency Department." Am J Case Rep **21**: e926251.

BACKGROUND Coronavirus disease 2019 (COVID-19) is an ongoing worldwide pandemic infection. Healthcare workers must utilize appropriate personal protective equipment (PPE) and infection control prevention techniques given the high risk of transmission and potential morbidity associated with COVID-19. We present a case report highlighting the strengths and potential applications of telemedicine technology in a patient's evaluation during an ongoing emerging, novel infectious disease. CASE REPORT A 67-year-old male presented to the Emergency Department (ED) with complaints of fever. His presentation coincided with a recent declaration of a pandemic caused by COVID-19 and a known exposure. Telemedicine evaluation was performed using InTouch Provider® software (InTouch Health, Goleta, CA, USA). The treating clinician was able to interact with the patient entirely through observations via web camera. COVID-19 nasopharyngeal swab polymerase chain reaction testing was ordered and was performed by a dedicated triage nurse. The patient was deemed stable for discharge given his normal vital signs and well appearance. Approximately 72 hours after discharge from the ED, the patient was contacted with positive COVID-19 swab results. CONCLUSIONS We present the first known case report highlighting use of telemedicine to diagnosis COVID-19 in a patient present in the ED. With the appropriate systems in place, this method of evaluating the patient helped to limit clinician exposure, decrease risk of transmission to key personnel, and assisted with preserving PPE supplies. Use of telemedicine affords multiple benefits in the effective diagnosis, evaluation, and potential prevention of spread of COVID-19.

Moni, M., et al. (2020). "Waiting for Godot: A cross sectional survey based analysis of the hydroxychloroquine prophylaxis strategy against COVID-19 in India." J Public Health Res **9**(4): 1888.

Background: India currently has the second largest burden of infections due to COVID-19. Health Care Worker (HCW) shortages are endemic to Indian healthcare. It should therefore be a huge priority to protect this precious resource as a critical component of the systemic response to this pandemic. Advisories from the Indian Council of Medical Research (ICMR) have focused on using hydroxychloroquine prophylaxis against COVID-19 in at risk HCW. This prophylaxis strategy has no evidence. In further jeopardy there appear to insubstantial attempts to build this evidence as well. In this connection, we commissioned a survey within our Institution to estimate the penetration of hydroxychloroquine (HCQ) use and use this to statistically model the impact of current ongoing studies in India. We also briefly review the literature on HCQ prophylaxis for COVID-19. Design and methods: A structured survey designed using RedCAP application was disseminated among healthcare professionals employed at an academic referral tertiary care centre via online social media platforms. The survey was kept open for the entire month of June 2020. The survey was additionally used to statistically model the size of studies required to comprehensively address the efficacy of HCQ in this setting. Results: 522 responses were received, of which 4 were incomplete. The ICMR strategy of 4 or more doses of HCQ was complete only in 15% of HCW in our survey. The majority of respondents were doctors (238, 46%). Amongst all category of responders, only 12% (n=63) received the full course. A majority of those who initiated the chemoprophylaxis with HCQ turned out to be medical professionals (59/63) with neither nurse nor other categories of healthcare workers accessing the medication. The respondents of our institutional survey did not report any life-threatening side effects. Presuming efficacy as per ICMR modelling for new registry trial on the lines of the published case control study, equal allocation between cases and controls and assuming a RR of 1.3.6, the power of such a study would be very low for n=2000 for event rates from 2.5-12.5%. Conclusion: We report the low penetration of HCQ chemoprophylaxis among the healthcare workers of our institution. We highlight the inherent drawbacks in the study design of current national COVID related trial based on the statistical modelling of our survey results and published literature, and thereby emphasis the need of evidence-based strategies contributing to research policy at national level.

Mont'alverne, F. J. A., et al. (2020). "Management of acute stroke and urgent neurointerventional procedures during COVID-19 pandemic: recommendations on the Scientific Department on Cerebrovascular Diseases of the Brazilian Academy of Neurology, Brazilian Society of Cerebrovascular Diseases and Brazilian Society of Neuroradiology." Arq Neuropsiquiatr **78**(7): 440-449.

INTRODUCTION: Although the 2019 severe acute respiratory syndrome coronavirus 2 infection (SARS-CoV-2, COVID-19) pandemic poses new challenges to the healthcare system to provide support for thousands of patients, there is special concern about common medical emergencies, such as stroke, that will continue to occur and will require adequate treatment. The allocation of both material and human resources to fight the pandemic cannot overshadow the care for acute stroke, a time-sensitive emergency that with an inefficient treatment will further increase mortality and long-term disability. OBJECTIVE: This paper summarizes the recommendations from the Scientific Department on Cerebrovascular Diseases of the Brazilian Academy of Neurology, the Brazilian Society of Cerebrovascular Diseases and the Brazilian Society of Neuroradiology for management of acute stroke and urgent neuro-interventional procedures during the COVID-19 pandemic, including proper use of screening tools, personal protective equipment (for patients and health professionals), and patient allocation.

Montanari Vergallo, G., et al. (2021). "The COVID-19 pandemic and contact tracing technologies, between upholding the right to health and personal data protection." Eur Rev Med Pharmacol Sci **25**(5): 2449-2456.

Countries responded to the COVID-19 pandemic with various levels of restrictions and lockdown in an effort to save lives and prevent the saturation and collapse of national health systems. Unfortunately, the blockades have entailed hefty socioeconomic costs. In order to contrast the spread of the virus, states have used contact tracing technology, in the form of mobile phone applications designed to track close contacts of those infected with COVID-19. Recent research has shown the effectiveness of this solution, particularly when used in conjunction with manual tracking. Nonetheless, the contact tracing app raises concerns due to the potential privacy implications. The authors have delved into the European legislation that protects privacy through the principles of proportionality and minimization, arguing that in order to quickly resolve the pandemic caused by COVID-19, one cannot blindly trust the exclusive help of technology. Instead, we need the involvement of health personnel, scientists, and no less importantly, the citizenry's sense of solidarity and the duty to abide by the rules of social distancing, the use of protective devices and hygiene rules to protect public health.

Montrief, T., et al. (2020). "COVID-19 respiratory support in the emergency department setting." Am J Emerg Med **38**(10): 2160-2168.

INTRODUCTION: Severe acute respiratory syndrome-related coronavirus 2 (SARS-CoV-2), which causes the coronavirus disease 2019 (COVID-19), may result in severe complications, multiorgan dysfunction, acute respiratory failure, and death. SARS-CoV-2 is highly contagious and places healthcare workers at significant risk, especially during aerosol-generating procedures, including airway management. OBJECTIVE: This narrative review outlines the underlying respiratory pathophysiology of patients with COVID-19 and discusses approaches to airway management in the emergency department (ED) based on current literature. DISCUSSION: Patients presenting with SARS-CoV-2 infection are at high risk for acute respiratory failure requiring airway management. Among hospitalized patients, 10-20% require intensive care unit admission, and 3-10% require intubation and mechanical ventilation. While providing respiratory support for these patients, proper infection control measures, including adherence to personal protective equipment policies, are necessary to prevent nosocomial transmission to healthcare workers. A structured approach to respiratory failure in these patients includes the use of exogenous oxygen via nasal cannula or non-rebreather, as well as titrated high-flow nasal cannula and non-invasive ventilation. This review offers several guiding principles and resources designed to be adapted in conjunction with local workplace policies for patients requiring respiratory support. CONCLUSIONS: While the fundamental principles of acute respiratory failure management are similar between COVID-19 and non-COVID-19 patients, there are some notable differences, including a focus on provider safety. This review provides an approach to airway management and respiratory support in the patient with COVID-19.

Moore, G., et al. (2021). "Detection of SARS-CoV-2 within the healthcare environment: a multi-centre study conducted during the first wave of the COVID-19 outbreak in England." J Hosp Infect **108**: 189-196.

BACKGROUND: Understanding how severe acute respiratory syndrome coronavirus-2 (SARS-CoV-2) is spread within the hospital setting is essential in order to protect staff, implement effective infection control measures, and prevent nosocomial transmission. METHODS: The presence of SARS-CoV-2 in the air and on environmental surfaces around hospitalized patients, with and without respiratory symptoms, was investigated. Environmental sampling was undertaken within eight hospitals in England during the first wave of the coronavirus disease 2019 outbreak. Samples were analysed using reverse transcription polymerase chain reaction (PCR) and virus isolation assays. FINDINGS: SARS-CoV-2 RNA was detected on 30 (8.9%) of 336 environmental surfaces. Cycle threshold values ranged from 28.8 to 39.1, equating to 2.2 x 10(5) to 59 genomic copies/swab. Concomitant bacterial counts were low, suggesting that the cleaning performed by nursing and domestic staff across all eight hospitals was effective. SARS-CoV-2 RNA was detected in four of 55 air samples taken <1 m from four different patients. In all cases, the concentration of viral RNA was low and ranged from <10 to 460 genomic copies/m(3) air. Infectious virus was not recovered from any of the PCR-positive samples analysed. CONCLUSIONS: Effective cleaning can reduce the risk of fomite (contact) transmission, but some surface types may facilitate the survival, persistence and/or dispersal of SARS-CoV-2. The presence of low or undetectable concentrations of viral RNA in the air supports current guidance on the use of specific personal protective equipment for aerosol-generating and non-aerosol-generating procedures.

Moore, Z., et al. (2021). "Facial pressure injuries and the COVID-19 pandemic: skin protection care to enhance staff safety in an acute hospital setting." J Wound Care **30**(3): 162-170.

OBJECTIVES: The aim of this study was to determine the impact of a specially designed care bundle on the development of facial pressure injuries (PI) among frontline healthcare workers wearing personal protective equipment (PPE) during the COVID-19 pandemic. The primary outcome of interest was the incidence of facial PIs. The secondary outcomes of interest were facial pain while wearing PPE and ease of use of the care bundle. METHODS: This study used a voluntary survey by questionnaire, supplemented by a qualitative analysis of interviews from a small purposive sample that took place in one large Irish hospital over a two-month period in 2020. The hospital was a city-based public university teaching hospital with 800 inpatient beds. The intervention was a care bundle consisting of skin protection, face mask selection, material use, skin inspection, cleansing and hydration developed in line with international best practice guidelines. All staff working in COVID-19 wards, intensive care units and the emergency department in the hospital were given a kitbag containing the elements of the care bundle plus an information pamphlet. Data were collected via a survey and interviews. RESULTS: A total of 114 staff provided feedback on the use of the care bundle. Before using the care bundle 29% (n=33) of the respondents reported developing a facial PI, whereas after using the care bundle only 8% (n=9) of the respondents reported developing a facial PI. The odds ratio (OR) of skin injury development was 4.75 (95% confidence interval (CI): 2.15-10.49; p=0.0001), suggesting that after the care bundle was issued, those who responded to the survey were almost five times less likely to develop a skin injury. Interviews with 14 staff determined that the bundle was easy to use and safe. CONCLUSION: Among those who responded to the survey, the use of the bundle was associated with a reduction in the incidence of skin injury from 29% to 8%, and respondents found the bundle easy to use, safe and effective. As with evidence from the international literature, this study has identified that when skincare is prioritised, and a systematic preventative care bundle approach is adopted, there are clear benefits for the individuals involved.

Moorthy, A. and T. K. Sankar (2020). "Emerging public health challenge in UK: perception and belief on increased COVID19 death among BAME healthcare workers." J Public Health (Oxf) **42**(3): 486-492.

BACKGROUND: Coronavirus infection Disease 19 impacted every part of the world and routine life. Recent report from the Office of national statistics in UK reported disproportionate death among Black Asian and minority ethnic (BAME) population. NHS is heavily relied on the BAME work force both in front line and in the community. We attempted to explore the beliefs and perception about reported worrying issue among BAME health work force in a Diverse city of Leicester. METHODS: This is a cross-sectional survey using 20 questions in an electronic format. The target population was identified through Leicester Asian Doctors Society and Leicester Asian Nurses Society. The questionnaire was then distributed electronically to the members. Survey questionnaire was accessed by 372, incomplete response (172) were excluded and 200 completed responses were analysed. RESULTS: Majority of BAME workforce are routinely involved in front line duties. More than 70% were anxious about their role during this pandemic. The Personal Protective Equipment (PPE) supply was adequate, and the support received from the local healthcare providers was more than satisfactory. The work force perceived co-morbidity, lack of PPE and testing were one of the few reasons for increased death in BAME. BAME group felt adequate provision of PPE, increased testing and improving mental health well-being is required to alleviate concerns and improve BAME working life in NHS. CONCLUSION: BAME workforce are routinely involved in front line work and current anxiety level is very high. Adequate provision of mental health support with clear risk stratification for return to work is required urgently.

Morawska, L., et al. (2020). "How can airborne transmission of COVID-19 indoors be minimised?" Environ Int **142**: 105832.

During the rapid rise in COVID-19 illnesses and deaths globally, and notwithstanding recommended precautions, questions are voiced about routes of transmission for this pandemic disease. Inhaling small airborne droplets is probable as a third route of infection, in addition to more widely recognized transmission via larger respiratory droplets and direct contact with infected people or contaminated surfaces. While uncertainties remain regarding the relative contributions of the different transmission pathways, we argue that existing evidence is sufficiently strong to warrant engineering controls targeting airborne transmission as part of an overall strategy to limit infection risk indoors. Appropriate building engineering controls include sufficient and effective ventilation, possibly enhanced by particle filtration and air disinfection, avoiding air recirculation and avoiding overcrowding. Often, such measures can be easily implemented and without much cost, but if only they are recognised as significant in contributing to infection control goals. We believe that the use of engineering controls in public buildings, including hospitals, shops, offices, schools, kindergartens, libraries, restaurants, cruise ships, elevators, conference rooms or public transport, in parallel with effective application of other controls (including isolation and quarantine, social distancing and hand hygiene), would be an additional important measure globally to reduce the likelihood of transmission and thereby protect healthcare workers, patients and the general public.

Moreno-Casbas, M. T. (2020). "Factors related to SARS-CoV-2 infection in healthcare professionals in Spain. The SANICOVI project." Enferm Clin **30**(6): 360-370.

OBJECTIVE: To describe the factors related to the situation of SARS-CoV-2 transmission identified by health professionals in Spain and to propose prevention strategies. METHOD: Cross-sectional descriptive study. The population were healthcare professionals working in institutions caring for COVID-19 patients and also confirmed cases of SARS-CoV-2 infection. A questionnaire with sociodemographic, occupational and epidemiological variables was used. Descriptive and bivariate analysis was performed according to the nature of the variables. RESULTS: A total of 2.230 questionnaires were analysed on a potential population of 41,239 (5.47%). The diagnosis was made based on a suspicious case (63.4%) and a probable case (12.3%). A study of contacts was carried out at 50.3%. The perception about the availability of protective measures as «always/frequently» were: FPP1 mask 57.3%, gloves 89.5%, soap 95% and hydroalcoholic solution 91.5%. In PPE, FPP2, FPP3 mask, goggles and disposable gowns at around 50%. The availability of protective measures, by field of work, presented significant differences. The average number of patients attended related to the performance of hand hygiene at moment4 and the perception of performing it correctly at moments4 and5. CONCLUSIONS: Preliminary data are presented, with variability in the response rate by Autonomous Region. Healthcare professionals infected by SARS-CoV-2 identified the management of the chain of infection transmission, the use and adequacy of protective equipment, as well as the effectiveness of handwashing as factors related to the transmission of the virus among professionals.

OBJECTIVE: To describe the factors related to the situation of SARS-CoV-2 transmission identified by health professionals in Spain and to propose prevention strategies. METHOD: Cross-sectional descriptive study. The population were healthcare professionals working in institutions caring for COVID-19 patients and also confirmed cases of SARS-CoV-2 infection. A questionnaire with sociodemographic, occupational and epidemiological variables was used. Descriptive and bivariate analysis was performed according to the nature of the variables. RESULTS: A total of 2.230 questionnaires were analysed on a potential population of 41,239 (5.47%). The diagnosis was made based on a suspicious case (63.4%) and a probable case (12.3%). A study of contacts was carried out at 50.3%. The perception about the availability of protective measures as «always/frequently» were: FPP1 mask 57.3%, gloves 89.5%, soap 95% and hydroalcoholic solution 91.5%. In PPE, FPP2, FPP3 mask, goggles and disposable gowns at around 50%. The availability of protective measures, by field of work, presented significant differences. The average number of patients attended related to the performance of hand hygiene at moment 4 and the perception of performing it correctly at moments 4 and 5. CONCLUSIONS: Preliminary data are presented, with variability in the response rate by Autonomous Region. Healthcare professionals infected by SARS-CoV-2 identified the management of the chain of infection transmission, the use and adequacy of protective equipment, as well as the effectiveness of handwashing as factors related to the transmission of the virus among professionals.

eng

Moreno-Jiménez, J. E., et al. (2021). "The Job Demands and Resources Related to COVID-19 in Predicting Emotional Exhaustion and Secondary Traumatic Stress Among Health Professionals in Spain." Front Psychol **12**: 564036.

The current COVID-19 crisis may have an impact on the mental health of professionals working on the frontline, especially healthcare workers due to the increase of occupational psychosocial risks, such as emotional exhaustion and secondary traumatic stress (STS). This study explored job demands and resources during the COVID-19 crisis in predicting emotional exhaustion and STS among health professionals. The present study is a descriptive and correlational cross-sectional design, conducted in different hospitals and health centers in Spain. The sample consisted of 221 health professionals with direct involvement in treating COVID-19. An online survey was created and distributed nationwide from March 20 to April 15 which assessed: sociodemographic and occupational data, fear of contagion, contact with death/suffering, lack of material and human protection resources (MHRP), challenge, emotional exhaustion, and STS. Descriptive findings show high levels of workload, contact with death/suffering, lack of MHPR and challenge, and are moderately high for fear of contagion, emotional exhaustion, and STS. We found an indirect significant effect of lack of MHPR on predicting (1) emotional exhaustion through the workload and (2) on STS through fear of contagion, contact with death/suffering, and workload. To conclude, this study examines the immediate consequences of the crisis on health professionals' well-being in Spain, emphasizing the job demands related to COVID-19 that health professionals are facing, and the resources available in these health contexts. These findings may boost follow-up of this crisis among health professionals to prevent them from long-term consequences.

Morgan, R. C., Jr. and T. N. Reid (2020). "On Answering the Call to Action for COVID-19: Continuing a Bold Legacy of Health Advocacy." J Natl Med Assoc **112**(6): 675-680.

The indelible impacts on our nation from the Coronavirus pandemic along with high fatality rates that disproportionately burden racial and ethnic minorities necessitate long-term coordinated federal, state and local action to improve critical determinants of population health, specifically important health and public health infrastructures as well as emergency and disaster preparedness systems. While our purview as the new pandemic epicenter should be a sufficient driver, coordinated health professionals bringing thoughtful attention to our historical context may be warranted. Prompting our advocacy should be the reality that our collective ability to rebound from such crises may ultimately hinge on protecting and equipping our most vulnerable racial-ethnic minority groups and any susceptible individuals within those populations. Recent historic firsts on behalf of racial and ethnic minorities taken by U.S. Department of Health and Human Services, through the Health Resources and Services Administration, the Office of Minority Health and the Centers for Disease Control and Prevention in response to COVID-19, if proven effective, should be considered for permanency within policy, practice and funding. In addition, given the complex history of Black Americans in this country and persistent and substantial Black-white disparities on health and economic measures across the board, the ultimate solution for improving the health and status Black Americans may look slightly different. Influenced by the 400th year anniversary of the first documented arrival of unfree Africans in North America in 1619, as well as the introduction of bills S.1080 and H.R.40 into Congress (The Commission to Study and Develop Reparation Proposals for African-Americans Act), some kind of reparations for Black Americans might serve as the logical starting point for further advocacy. Nevertheless, we remain supportive allies of all organizations concerned with communities who suffer the weight of this pandemic and any future world health disasters. What is additionally needed is a thoughtful unification of efforts and a commitment to sustained progress with measurable results for as long as the need exists and certainly for the foreseeable future. Let us as humane clinicians and public health professionals capture this moment of challenge and follow through on this urgent call to action.

Morgan, R. C., Jr. and T. N. Reid (2020). "On Answering the Call to Action For COVID-19: Continuing a Bold Legacy of Health Advocacy." J Natl Med Assoc **112**(3): 324-328.

The disproportionately high burden of death and disability observed for racial and ethnic minorities under the Coronavirus pandemic necessitates sustained advocacy by the medical and public health communities around critical determinants of population health. Prompting our advocacy should be the understanding that our collective ability to rebound from such crises may ultimately hinge on protecting and equipping our most vulnerable racial-ethnic minority groups and any susceptible individuals within those populations. If proven effective, recent historic firsts by the U.S. Department of Health and Human Services (HHS), through the Health Resources and Services Administration (HRSA), the Office of Minority Health (OMH) and the Centers for Disease Control and Prevention (CDC) in response to COVID-19 should be championed for permanency within policy, practice and funding. In addition, given the complex history of Black Americans in this country and persistent and substantial Black-white disparities on health and economic measures across the board, some kind of reparations for this group may serve as a logical starting point for further advocacy. Nevertheless, we remain supportive allies of all organizations concerned with communities who suffer the weight of this pandemic and any future world health disasters. Let us as human clinicians and public health professionals capture this moment of challenge and engage in thoughtful unification of effort and commit to measurable progress for as long as the need exists and certainly for the foreseeable future.

Mori, M., et al. (2020). "COVID-19: clinical issues from the Japan Surgical Society." Surg Today **50**(8): 794-808.

In this unprecedented COVID-19 pandemic, several key issues must be addressed to ensure safe treatment and prevent rapid spread of the virus and a consequential medical crisis. Careful evaluation of a patient's condition is crucial for deciding the triage plan, based on the status of the disease and comorbidities. As functionality of the medical care system is greatly affected by the environmental situation, the treatment may differ according to the medical and infectious disease circumstances of the institution. Importantly, all medical staff must prevent nosocomial COVID-19 by minimizing the effects of aerosol spread and developing diagnostic and surgical procedures. Polymerase chain reaction (PCR) screening for COVID-19 infection, particularly in asymptomatic patients, should be encouraged as these patients are prone to postoperative respiratory failure. In this article, the Japan Surgical Society addresses the general principles of surgical treatment in relation to COVID-19 infection and advocates preventive measures against viral transmission during this unimaginable COVID-19 pandemic.

Morice, A. H. (2020). "Editorial comment on: Global burden of COVID-19 pandemic on healthcare workers." ERJ Open Res **6**(2).

This is an important paper but from my personal observations of how we are handling the pandemic in hospitals, it is perhaps not the risk of dealing with the patients that is the problem. In my institution, we have been fortunate to have sufficient personal protective equipment (PPE) and we are well protected when entering the ward environment. However, wearing PPE is uncomfortable and it is disposed of on leaving the patient's room. Staff then congregate at the nurses' station and in the offices, where it is impossible to observe social isolation. I believe that the main threat to healthcare workers its other healthcare workers! We have had ward-based outbreaks with staff all contracting the virus simultaneously, presumably from an asymptomatic carrier in the workplace. This issue needs to be urgently addressed.

Morishita, K., et al. (2021). "Impact of incentives for health-care workers wearing personal protective equipment while dealing with coronavirus disease in Japan." J Occup Health **63**(1): e12213.

Morris, C. R., et al. (2021). "Prevalence of SARS-CoV-2 antibodies in pediatric healthcare workers." Int J Infect Dis **105**: 474-481.

OBJECTIVES: To determine SARS-CoV-2-antibody prevalence in pediatric healthcare workers (pHCWs). DESIGN: Baseline prevalence of anti-SARS-CoV-2-IgG was assessed in a prospective cohort study from a large pediatric healthcare facility. Prior SARS-CoV-2 testing history, potential risk factors and anxiety level about COVID-19 were determined. Prevalence difference between emergency department (ED)-based and non-ED-pHCWs was modeled controlling for those covariates. Chi-square test-for-trend was used to examine prevalence by month of enrollment. RESULTS: Most of 642 pHCWs enrolled were 31-40years, female and had no comorbidities. Half had children in their home, 49% had traveled, 42% reported an illness since January, 31% had a known COVID-19 exposure, and 8% had SARS-CoV-2 PCR testing. High COVID-19 pandemic anxiety was reported by 71%. Anti-SARS-CoV-2-IgG prevalence was 4.1%; 8.4% among ED versus 2.0% among non-ED pHCWs (p < 0.001). ED-work location and known COVID-19 exposure were independent risk factors. 31% of antibody-positive pHCWs reported no symptoms. Prevalence significantly (p < 0.001) increased from 3.0% in April-June to 12.7% in July-August. CONCLUSIONS: Anti-SARS-CoV-2-IgG prevalence was low in pHCWs but increased rapidly over time. Both working in the ED and exposure to a COVID-19-positive contact were associated with antibody-seropositivity. Ongoing universal PPE utilization is essential. These data may guide vaccination policies to protect front-line workers.

Morrison, E. H., et al. (2021). "How the COVID-19 Pandemic has changed multiple sclerosis clinical practice: Results of a nationwide provider survey." Mult Scler Relat Disord **51**: 102913.

BACKGROUND: The COVID-19 crisis has created unanticipated changes in health care delivery for people living with multiple sclerosis (MS). The pandemic's rapid evolution has resulted in a knowledge gap in how COVID-19 has affected MS clinical practice. Our objective was to understand how the COVID-19 pandemic has affected clinical practice patterns in a nationwide cohort of MS clinicians across the United States. METHODS: In collaboration with the National Multiple Sclerosis Society (NMSS), we developed a 28-item SurveyMonkey(TM) electronic questionnaire exploring MS specialists' perceptions of how COVID-19 has altered how they prescribe MS disease-modifying therapies (DMTs), provide telehealth and other services, and view issues affecting their own well-being including re-deployment to the front lines of COVID-19 care and availability of personal protective equipment (PPE). NMSS staff sent a recruitment email containing the electronic survey link to 188 clinicians who serve on regional NMSS Healthcare Provider Councils across the US, 86 (45.7%) of whom were MS specialist physicians. RESULTS: Eighty-six of 188 potential respondents (45.7%) from 32 US states completed the survey including 45 physicians (41 neurologists, 3 physiatrists and 1 family physician), 18 rehabilitation therapists, 7 psychologists, 6 nurse practitioners, 4 social workers, 2 physician assistants, 2 nurses and 2 health professionals from other disciplines. More than 80% of all respondents working on-site in a health care setting believed they had adequate PPE. More than 41% were able to distance safely from others at work. Nearly 10% of respondents reported they had been re-deployed to the front lines of COVID-19 patient care, and an additional 16.9% anticipated being re-deployed. Among the MS specialist physician subgroup, nearly one-third reported using telemedicine to provide over 75% of their clinical care. Only 16.7% believed COVID-19 had not changed how they prescribe DMTs. Therapies prescribed more often during the pandemic included β-IFNs (28.6% of prescribers), natalizumab (23.8%), glatiramer acetate (21.4%) and teriflunomide (19%). DMTs prescribed less often included alemtuzumab (64.3% of prescribers), cladribine (54.8%), ocrelizumab and rituximab (50%), and fingolimod and siponimod (40.5%). For at least some of their patients during the pandemic, some MS specialists reported suspending certain DMTs including alemtuzumab (21.4% of prescribers), ocrelizumab and rituximab (16.7%) and cladribine (11.9%). Others reported extending DMT dosing intervals for natalizumab (38.1%), fingolimod and siponimod (11.9%). CONCLUSIONS: In this nationwide survey, MS specialist physicians and other clinicians serving on regional NMSS Healthcare Provider Councils across the US reported profound changes in how they are delivering MS care during the COVID-19 pandemic.

Morse, J. M., et al. (2021). "Developing the Resilience Framework for Nursing and Healthcare." Glob Qual Nurs Res **8**: 23333936211005475.

Despite four decades of resilience research, resilience remains a poor fit for practice as a scientific construct. Using the literature, we explored the concepts attributed to the development of resilience, identifying those that mitigate symptoms of distress caused by adversity and facilitate coping in seven classes of illness: transplants, cancer, mental illness, episodic illness, chronic and painful illness, unexpected events, and illness within a dyadic relationship. We identified protective, compensatory, and challenge-related coping-concept strategies that healthcare workers and patients use during the adversity experience. Healthcare-worker assessment and selection of appropriate coping concepts enable the individual to control their distress, resulting in attainment of equanimity and the state of resilience, permitting the resilient individual to work toward recovery, recalibration, and readjustment. We inductively developed and linked these conceptual components into a dynamic framework, The Resilience Framework for Nursing and Healthcare, making it widely applicable for healthcare across a variety of patients.

Mosheva, M., et al. (2021). "The association between witnessing patient death and mental health outcomes in frontline COVID-19 healthcare workers." Depress Anxiety **38**(4): 468-479.

BACKGROUND: Healthcare workers (HCW) treating coronavirus disease 2019 (COVID-19) patients face high levels of psychological stress. We aimed to compare mental health outcomes, risk and protective factors for posttraumatic stress symptoms (PTSS), probable depression, and anxiety between HCW working in COVID-19 and non-COVID-19 wards. METHODS: A self-report survey, administered in a large tertiary hospital in Israel during the peak of the COVID-19 outbreak was completed by 828 HCW (42.2% physicians, 57.8% nurses. Patient-Reported Outcomes Measurement Information System; the Patient Health Questionnaire-9; the Primary Care-Post Traumatic Stress Disorder Screen for DSM-5 (PC-PTSD-5) were used for assessing anxiety, depression, and PTSS, respectively. Pandemic-related stress factors, negative experiences, and potential protective factors were also assessed. RESULTS: Median PC-PTSD scores differed significantly between study teams (χ(2) [5] = 17.24; p = .004). Prevalence of probable depression and anxiety were similar in both groups. Risk factors for mental health outcomes included mental exhaustion, anxiety about being infected and infecting family. Overall, higher proportion of the COVID-19 team witnessed patient deaths as compared to the non-COVID-19 team (50.2% vs. 24.7%). Witnessing patient death at the COVID-19 wards was associated with a four-fold increased likelihood of PTSS (odds ratio [OR] = 3.97; 95% confidence interval [CI], 1.58-9.99; p = .0007), compared with the non-COVID-19 wards (OR 0.91; 95% CI, 0.51-1.61; p = .43). CONCLUSIONS: Witnessing patient death appears to be a risk factor for PTSS unique to HCW directly engaged in treating patients with COVID-19. Our findings suggest that helping HCW cope with COVID-19 related deaths might reduce their risk of posttraumatic stress.

Motara, F., et al. (2020). "The "Intubox": Enhancing Frontline Healthcare Worker Safety During Coronavirus Disease 2019 (COVID-19)." Cureus **12**(6): e8530.

There has been a substantial burden of healthcare worker infection during the current coronavirus (COVID-19) pandemic, likely due to a lack of adequate preparedness, suboptimal institutional infection control measures, atypical patient presentation, poor compliance with personal protective equipment (PPE) and exposure to high-risk aerosol generating procedures, such as endotracheal intubation. There is significant concern that developing countries will face heightened levels of staff exposure during the COVID-19 pandemic. To mitigate this exposure risk during procedures, such as endotracheal intubation, various "aerosol boxes" have been designed by frontline healthcare workers. However, in practice these boxes were found to hamper endotracheal intubation and other procedures due to the limited space and manoeuvrability they allow. To further reduce particle dispersion and to improve on the practicality and ergonomic design of the prototype "aerosol box", the Intubox was developed by staff at the Charlotte Maxeke Johannesburg Academic Hospital after instituting several changes to the prototype design.

Mottay, L., et al. (2020). "KN95 filtering facepiece respirators distributed in South Africa fail safety testing protocols." S Afr Med J **0**(0): 13162.

BACKGROUND: Given the global shortage of N95 filtering facepiece respirators (FFP2 in Europe) during the COVID-19 pandemic, KN95 masks (Chinese equivalent of the N95 and FFP2) were imported and distributed in South Africa (SA). However, there are hardly any published independent safety data on KN95 masks. OBJECTIVES: To evaluate the seal, fit and filtration efficiency of several brands of KN95 masks marketed for widespread use in SA healthcare facilities, using standardised testing protocols. METHODS: The verifiability of manufacturer and technical details was first ascertained, followed by evaluation of the number of layers comprising the mask material. The testing protocol involved a directly observed positive and negative pressure user seal check, which if passed was followed by qualitative fit testing (sodium saccharin) in healthy laboratory or healthcare workers. Quantitative fit testing (3M) was used to validate the qualitative fit testing method. The filtration efficacy and integrity of the mask filter material were evaluated using a particle counter-based testing rig utilising aerosolised saline (expressed as filtration efficacy of 0.3 µm particles). Halyard FLUIDSHIELD 3 N95 and 3M 1860 N95 masks were used as controls. RESULTS: Twelve KN95 mask brands (total of 36 masks) were evaluated in 7 participants. The mask type and manufacturing details were printed on only 2/12 brands (17%) as per National Institute of Occupational Safety and Health and European Union regulatory requirements. There was considerable variability in the number of KN95 mask layers (between 3 and 6 layers in the 12 brands evaluated). The seal check pass rate was significantly lower in KN95 compared with N95 masks (1/36 (3%) v. 12/12 (100%); p&lt;0.0001). Modification of the KN95 ear-loop tension using head straps or staples, or improving the facial seal using Micropore 3M tape, enhanced seal test performance in 15/36 KN95 masks evaluated (42%). However, none of these 15 passed downstream qualitative fit testing compared with the control N95 masks (0/15 v. 12/12; p&lt;0.0001). Only 4/8 (50%) of the KN95 brands tested passed the minimum filtration requirements for an N95 mask (suboptimal KN95 filtration efficacy varied from 12% to 78%, compared with 56% for a surgical mask and &gt;99% for the N95 masks at the 0.3 µm particle size). CONCLUSIONS: The KN95 masks tested failed the stipulated safety thresholds associated with protection of healthcare workers against airborne pathogens such as SARS-CoV-2. These preliminary data have implications for the regulation of masks and their distribution to healthcare workers and facilities in SA.

Moul, J. W., et al. (2020). "Managing Prostate Cancer Surgical Patients during the COVID-19 Pandemic: A Brief Report of the Duke Cancer Institute's Initial Experience." Oncology (Williston Park) **34**(5): 156-162.

The coronavirus disease 2019 pandemic has rapidly placed tremendous stress on health systems around the world. In response, multiple health systems have postponed elective surgeries in order to conserve hospital beds and personal protective equipment, minimize patient traffic, and prevent unnecessary utilization and exposure of healthcare workers. The American College of Surgeons released the following statement on March 13, 2020: "Each hospital, health system and surgeon should thoughtfully review all scheduled elective procedures with a plan to minimize, postpone, or cancel electively scheduled operations, endoscopes, or other invasive procedures until we have passed the predicted inflection point in the exposure graph and can be confident that our health care infrastructure can support a potentially rapid and overwhelming uptick in critical patient care needs." In our state, North Carolina, Governor Roy Cooper requested that all hospitals postpone elective and non-urgent procedures and surgeries effective March 23, 2020.

Mousing, C. A. and D. Sørensen (2021). "Living with the risk of being infected: COPD patients' experiences during the coronavirus pandemic." J Clin Nurs.

AIMS: To explore the existential significance of living with the risk of being infected with coronavirus in patients with COPD. BACKGROUND: Distancing measures aim to break the coronavirus transmission chains. Physical separation from social networks and social isolation are correlated with anxiety and depression. People with a chronic obstructive lung disease are particularly vulnerable due to the increased risk of a serious course of illness, so therefore many of them choose self-isolation to protect themselves from COVID-19. DESIGN: A qualitative exploratory study using individual semi-structured interviews. METHODS: From June-September 2020, 13 participants were recruited through advertisements on Facebook as a convenience sample for semi-structured individual interviews. The interviews took place through virtual platforms or in physical meetings. Data were analysed using Ricoeur's phenomenological approach, involving naïve reading, a structural analysis and a critical interpretation strategy. The study has been reported in line with COREQ guidelines. FINDINGS: Living with the threat of being infected with coronavirus has greatly affected everyday life for patients with COPD. The nagging fear of coronavirus as a death threat was a dominant feeling, together with anxiety, loneliness and hope. With self-isolation, followed concerns of being forgotten and thoughts of the future, balancing between fearing the worst, and hoping the best. CONCLUSIONS: Patients with moderate to severe COPD feel compelled to self-isolate, as they fear dying from COVID-19. The study revealed a need for proactive contact with health professionals to calm the patients' feelings of deprivation, loneliness, hopelessness and anxiety. RELEVANCE TO CLINICAL PRACTICE: Information about the patient's perspective may be used to develop targeted interventions aimed at giving adequate information, supporting hope, implementing digital or virtual solutions to keep in contact and avoid the feeling of being alone and forgotten during a pandemic crisis.

Mulchandani, R., et al. (2021). "Association between self-reported signs and symptoms and SARS-CoV-2 antibody detection in UK key workers." J Infect.

BACKGROUND: Screening for SARS-CoV-2 antibodies is under way in some key worker groups; how this adds to self-reported COVID-19 illness is unclear. In this study, we investigate the association between self-reported belief of COVID-19 illness and seropositivity. METHODS: Cross-sectional study of three key worker streams comprising (A) Police and Fire & Rescue (2 sites) (B) healthcare workers (1 site) and (C) healthcare workers with previously positive PCR result (5 sites). We collected self-reported signs and symptoms of COVID-19 and compared this with serology results from two SARS-CoV-2 immunoassays (Roche Elecsys® and EUROIMMUN). RESULTS: Between 01 and 26 June, we recruited 2847 individuals (Stream A: 1,247, Stream B: 1,546 and Stream C: 154). Amongst those without previous positive PCR tests, 687/2,579 (26%) reported belief they had COVID-19, having experienced compatible symptoms; however, only 208 (30.3%) of these were seropositive on both immunoassays. Both immunoassays had high sensitivities relative to previous PCR positivity (>93%); there was also limited decline in antibody titres up to 110 days post symptom onset. Symptomatic but seronegative individuals had differing symptom profiles and shorter illnesses than seropositive individuals. CONCLUSION: Non-COVID-19 respiratory illness may have been mistaken for COVID-19 during the outbreak; laboratory testing is more specific than self-reported key worker beliefs in ascertaining past COVID-19 disease.

Mulu, G. B., et al. (2020). "Preparedness and Responses of Healthcare Providers to Combat the Spread of COVID-19 Among North Shewa Zone Hospitals, Amhara, Ethiopia, 2020." Infect Drug Resist **13**: 3171-3178.

BACKGROUND: Coronavirus disease 2019 (COVID-19) is an emerging respiratory disease that is caused by a novel coronavirus and was first detected in December 2019 in Wuhan, China. The disease is highly infectious, and its main clinical symptoms include fever, dry cough, fatigue, myalgia, and dyspnea. Healthcare providers are in front in fighting the coronavirus spread by making themselves the risk of contracting the disease. OBJECTIVE: To assess the preparedness and responses of healthcare providers to combat the spread of COVID-19 among North Shewa Zone Hospitals, Amhara, Ethiopia. METHODS: Facility-based cross-sectional study was conducted from April to May 2020 among 422 healthcare providers in the North Shewa Zone, Amhara, Ethiopia using a self-administered questionnaire. Study subjects were selected through systematic random sampling based on their proportional distribution of sample size to each hospital. A structured questionnaire was used to collect the data. The data were coded and entered into the Epi data 4.2.1 version and the analysis was carried out in Statistical Package for Social Science 25 versions. RESULTS: Four hundred four participants involved in the study have been given a response rate of 95.7%. The self-satisfaction of healthcare providers revealed 301 (74.5%) of study participants feel unsafe in their workplace. Two-third, 260 (64.4%), of them responded that they feel anxious while working with febrile patients. Nearly one-third (31%), 27.4%, 15.9%, 14.5%, 14.2% of HCPs had access to gloves, facemask, goggle, shoe, and apron respectively in hospitals. CONCLUSION: Protecting healthcare workers is a public health priority. Access to essential personal protective equipment during the COVID-19 pandemic was limited. The poor perception of healthcare professionals about not having enough support from medical institutions and public health authorities raises the need to urgently implement strategies to protect healthcare workers in the time of the COVID-19 pandemic.

Mumtaz, N., et al. (2021). "COVID-19 Rehab Fright Management." Pak J Med Sci **37**(1): 277-282.

Being diagnosed as positive for SARS-2 CoV RNA on PCR generates anxiety and stress as well as depression due to the prospects of being isolated. With genetically varied forms of virus on the rise the widely adopted manner to protect oneself is social distancing. This is frightening for rehabilitation professionals who at times are exposed at close quarters to the patients as rehabilitation is essential. Data in this backdrop is lacking, as this is a recent and ongoing pandemic. As such the current study was conducted with an attempt to review and highlight the causes of fright among rehabilitation professionals and possible management options in the wake of current pandemic of COVID-19 in the perspective of Pakistan. For this purpose literature was searched from major electronic databases including PubMed, Google, Google Scholar and Web-of-science, with keywords "Covid-19, mental health, telehealth, telemedicine, tele-rehabilitation and combination of words". Eighty English, full text articles were studied out of which 36 were used for the literature review. With this literature review we conclude that COVID-19 has resulted in fear of contracting and transmitting this disease among health professionals and can be reduced and managed by provision of tele-rehabilitation and telehealth facilities. Patients emerging from prolonged mechanical ventilations require extensive rehabilitation to restore routine body functions. The role of the otolaryngologist and speech language pathologist (SLP) is formal and direct to ensure appropriate and timely long term intervention and rehabilitation to ensure that these individuals re-enter mainstream activities.

Munawar, K. and F. R. Choudhry (2021). "Exploring stress coping strategies of frontline emergency health workers dealing Covid-19 in Pakistan: A qualitative inquiry." Am J Infect Control **49**(3): 286-292.

BACKGROUND: The COVID-19 outbreak has gravely impacted the physical and psychological health of people. As the outbreak is ongoing, it is crucial to equip the emergency healthcare workers (HCWs) to be medically and psychologically prepared. OBJECTIVE: To examine the psychological impact of COVID-19 on emergency HCWs and to understand how they are dealing with COVID-19 pandemic, their stress coping strategies or protective factors, and challenges while dealing with COVID-19 patients. METHODS: Using a framework thematic analysis approach, 15 frontline emergency HCWs directly dealing with COVID-19 patients from April 2, 2020 to April 25, 2020. The semi-structured interviews were conducted face-to-face or by telephone. Data were analyzed using thematic analysis. RESULTS: Findings highlighted first major theme of stress coping, including, limiting media exposure, limited sharing of Covid-19 duty details, religious coping, just another emergency approach, altruism, and second major theme of Challenges includes, psychological response and noncompliance of public/denial by religious scholar. CONCLUSIONS: Participants practiced and recommended various coping strategies to deal with stress and anxiety emerging from COVID-19 pandemic. Media was reported to be a principal source of raising stress and anxiety among the public. Religious coping as well as their passion to serve humanity and country were the commonly employed coping strategies.

Munzer, B. W., et al. (2020). "In-situ Simulation Use for Rapid Implementation and Process Improvement of COVID-19 Airway Management." West J Emerg Med **21**(6): 99-106.

INTRODUCTION: The coronavirus disease 2019 (COVID-19) pandemic presents unique challenges to frontline healthcare workers. In order to safely care for patients new processes, such as a plan for the airway management of a patient with COVID-19, must be implemented and disseminated in a rapid fashion. The use of in-situ simulation has been used to assist in latent problem identification as part of a Plan-Do-Study-Act cycle. Additionally, simulation is an effective means for training teams to perform high-risk procedures before engaging in the actual procedure. This educational advance seeks to use and study in-situ simulation as a means to rapidly implement a process for airway management in patients with COVID-19. METHODS: Using an airway algorithm developed by the authors, we designed an in-situ simulation scenario to train physicians, nurses, and respiratory therapists in best practices for airway management of patients with COVID-19. Physician participants were surveyed using a five-point Likert scale with regard to their comfort level with various aspects of the airway algorithm both before and after the simulation in a retrospective fashion. Additionally, we obtained feedback from all participants and used it to refine the airway algorithm. RESULTS: Over a two-week period, 93 physicians participated in the simulation. We received 81 responses to the survey (87%), which showed that the average level of comfort with personal protective equipment procedures increased significantly from 2.94 (95% confidence interval, 2.71-3.17) to 4.36 (4.24-4.48), a difference of 1.42 (1.20-1.63, p < 0.001). There was a significant increase in average comfort level in understanding the physician role with scores increasing from 3.51 (3.26-3.77) to 4.55 (2.71-3.17), a difference of 1.04 (0.82-1.25, p < 0.001). There was also increased comfort in performing procedural tasks such as intubation, from 3.08 (2.80-3.35) to 4.38 (4.23-4.52) after the simulation, a difference of 1.30 points (1.06-1.54, p < 0.001). Feedback from the participants also led to refinement of the airway algorithm. CONCLUSION: We successfully implemented a new airway management guideline for patients with suspected COVID-19. In-situ simulation is an essential tool for both dissemination and onboarding, as well as process improvement, in the context of an epidemic or pandemic.

Mureddu, G. F., et al. (2020). "Cardiac rehabilitation activities during the COVID-19 pandemic in Italy. Position Paper of the AICPR (Italian Association of Clinical Cardiology, Prevention and Rehabilitation)." Monaldi Arch Chest Dis **90**(2).

The COVID-19 outbreak is having a significant impact on both cardiac rehabilitation (CR) inpatient and outpatient healthcare organization. The variety of clinical and care scenarios we are observing in Italy depends on the region, the organization of local services and the hospital involved. Some hospital wards have been closed to make room to dedicated beds or to quarantine the exposed health personnel. In other cases, CR units have been converted or transformed into COVID-19 units.  The present document aims at defining the state of the art of CR during COVID-19 pandemic, through the description of the clinical and management scenarios frequently observed during this period and the exploration of the future frontiers in the management of cardiac rehabilitation programs after the COVID-19 outbreak.

Murewanhema, G. and R. Makurumidze (2020). "Essential health services delivery in Zimbabwe during the COVID-19 pandemic: perspectives and recommendations." Pan Afr Med J **35**(Suppl 2): 143.

Zimbabwe reported its first case of COVID-19 on 20 March 2020, and since then the number has increased to over 4000. To contain the spread of the causative SARS-CoV-2 and prepare the healthcare system, public health interventions, including lockdowns, were imposed on 30 March 2020. These resulted in disruptions in healthcare provision, and movement of people and supply chains. There have been resultant delays in seeking and accessing healthcare by the patients. Additionally, disruption of essential health services in the areas of maternal and child health, sexual and reproductive health services, care for chronic conditions and access to oncological and other specialist services has occurred. Thus, there may be avoidable excess morbidity and mortality from non-COVID-19 causes that is not justifiable by the current local COVID-19 burden. Measures to restore normalcy to essential health services provision as guided by the World Health Organisation and other bodies needs to be considered and implemented urgently, to avoid preventable loss of life and excess morbidity. Adequate infection prevention and control measures must be put in place to ensure continuity of essential services whilst protecting healthcare workers and patients from contracting COVID-19.

Murewanhema, G., et al. (2020). "Restoring and maintaining robust maternity services in the COVID-19 era: a public health dilemma in Zimbabwe." Pan Afr Med J **37**(Suppl 1): 32.

Lockdown policies, travel restrictions and reduced provision of healthcare in Zimbabwe in response to the COVID-19 pandemic have brought unprecedented challenges for healthcare delivery. Maternity services, including antenatal care, labour and delivery as well as postnatal care have been affected directly and indirectly by the pandemic and resultant control interventions, with delays introduced at several points across the continuum of care. Unfortunately, maternity conditions are time-sensitive, and delays can negatively impact feto-maternal outcomes, with increased maternal, fetal or neonatal morbidity and mortality. An audit at central hospitals revealed reduced utilisation of maternity services and a trend towards an increase in maternal mortality. A formal evaluation is required; however, mitigating public health interventions are required, especially as the burden of COVID-19 in the country has considerably come down. The World Health Organisation offers useful technical guidance for maintaining essential health services in pandemic times in low-resources settings, and rationalising the use of personal protective equipment, which can be contextualised and adopted to restore and maintain essential health services. Restoration of essential maternity services is urgently required in an environment that protects healthcare workers and their clients, minimising their risk of contracting COVID-19 whilst optimising fetomaternal outcomes. Thus, the various stakeholders involved in maternity care must urgently come together and find ways of achieving this goal.

Mushtaq, S., et al. (2021). "Cutaneous adverse effects due to personal protective measures during COVID-19 pandemic: a study of 101 patients." Int J Dermatol **60**(3): 327-331.

BACKGROUND: Coronavirus Disease 2019 (COVID-19) is a viral illness caused by the novel coronavirus SARS-CoV-2 which spreads via droplets from an infected person. There has been an unprecedented rise in the use of personal protective equipment and practice of personal hygiene measures against COVID-19. The extended use of protective measures (PM) can lead to ill effects on the skin. Our aim was to investigate PM-induced dermatoses amongst healthcare workers and the general population during the COVID-19 pandemic. METHODS: A cross-sectional study was conducted over a period of 2 months. The study subjects were patients who presented to dermatology outpatient clinics or sought teleconsultation for skin problems related to the use of PMs against COVID-19. A detailed history was obtained and cutaneous examination was documented for all the patients in a pre-set proforma. Diagnoses of the adverse skin effects were formulated based upon history and clinical examination. RESULTS: A total of 101 cases with cutaneous adverse effects due to the use of PMs against COVID-19 were included in the study. The general population and healthcare workers were affected similarly, comprising of 54.5% and 45.5%, respectively. The mean age of the study participants was 36.71 ± 15.72 years. The most common culprit material was soap and water (56.4%). Contact dermatitis was found to be the most common adverse effect in the majority of our patients (72.3%). The most common symptom reported was pruritus (45.5%). The wearing of personal protective equipment for a longer duration was significantly associated with multiple symptoms (P = 0.026). CONCLUSION: The enhanced use of different PMs against COVID-19 can result in a variety of adverse skin effects. In our study, the use of soap and water was the most common culprit PM, and contact dermatitis was the most common adverse effect noted.

Mutambudzi, M., et al. (2020). "Occupation and risk of severe COVID-19: prospective cohort study of 120 075 UK Biobank participants." Occup Environ Med.

OBJECTIVES: To investigate severe COVID-19 risk by occupational group. METHODS: Baseline UK Biobank data (2006-10) for England were linked to SARS-CoV-2 test results from Public Health England (16 March to 26 July 2020). Included participants were employed or self-employed at baseline, alive and aged <65 years in 2020. Poisson regression models were adjusted sequentially for baseline demographic, socioeconomic, work-related, health, and lifestyle-related risk factors to assess risk ratios (RRs) for testing positive in hospital or death due to COVID-19 by three occupational classification schemes (including Standard Occupation Classification (SOC) 2000). RESULTS: Of 120 075 participants, 271 had severe COVID-19. Relative to non-essential workers, healthcare workers (RR 7.43, 95% CI 5.52 to 10.00), social and education workers (RR 1.84, 95% CI 1.21 to 2.82) and other essential workers (RR 1.60, 95% CI 1.05 to 2.45) had a higher risk of severe COVID-19. Using more detailed groupings, medical support staff (RR 8.70, 95% CI 4.87 to 15.55), social care (RR 2.46, 95% CI 1.47 to 4.14) and transport workers (RR 2.20, 95% CI 1.21 to 4.00) had the highest risk within the broader groups. Compared with white non-essential workers, non-white non-essential workers had a higher risk (RR 3.27, 95% CI 1.90 to 5.62) and non-white essential workers had the highest risk (RR 8.34, 95% CI 5.17 to 13.47). Using SOC 2000 major groups, associate professional and technical occupations, personal service occupations and plant and machine operatives had a higher risk, compared with managers and senior officials. CONCLUSIONS: Essential workers have a higher risk of severe COVID-19. These findings underscore the need for national and organisational policies and practices that protect and support workers with an elevated risk of severe COVID-19.

Muzzi, E., et al. (2021). "Short report on the effects of SARS-CoV-2 face protective equipment on verbal communication." Eur Arch Otorhinolaryngol: 1-6.

OBJECTIVE: To predict the impact of face personal protective equipment on verbal communication during the SARS-CoV-2 pandemic. DESIGN: We assessed the effect of common types and combinations of face personal protective equipment on speech intelligibility in quiet and in a simulated noisy environment. RESULTS: Wearing face personal protective equipment impairs transmission of middle-to-high voice frequencies and affects speech intelligibility. Surgical masks are responsible for up to 23.3% loss of speech intelligibility in noisy environments. The effects are larger in the condition of advanced face personal protective equipment, accounting for up to 69.0% reduction of speech intelligibility. CONCLUSION: The use of face personal protective equipment causes significant verbal communication issues. Healthcare workers, school-aged children, and people affected by voice and hearing disorders may represent specific at-risk groups for impaired speech intelligibility.

Myatra, S. N., et al. (2020). "Anesthesia for oral surgeries during the COVID-19 pandemic." J Anaesthesiol Clin Pharmacol **36**(Suppl 1): S96-s104.

The severe acute respiratory syndrome corona virus 2(SARS-Cov2) virus replicates in the nasal cavity, nasopharynx, and the oropharynx. During oral surgery, the risk of viral transmission is high during instrumentation in these areas, while performing airway management procedures, the oral surgery itself, and related procedures. During the corona virus disease 2019 (COVID-19) pandemic, patients with an oral pathology usually present for emergency procedures. However, patients with oral cancer, being a semi-emergency, may also present for diagnostic and therapeutic procedures. When elective surgeries are resumed, these patients will come to the operating room. In asymptomatic patients, the false-negative rate can be as high as 30%. These patients are a source of infection to the healthcare workers and other patients. This mandates universal precautions to be taken for all patients presenting for surgery. Lesions along the airway, distorted anatomy secondary to cancer therapy, shared airway with the surgeon, surgical handling of the airway and the risk of bleeding, make airway management challenging in these patients, especially while wearing personal protective equipment. Airway management procedures, oral surgery, use of cautery, and other powered surgical instruments in the aero digestive tract, along with constant suctioning are a source of significant aerosol generation, further adding to the risk of viral transmission. Maintaining patient safety, while protecting the healthcare workers from getting infected during oral surgery is paramount. Meticulous advance planning and team preparation are essential. In this review, we discuss the challenges and recommendations for safe anesthesia practice for oral surgery during the COVID-19 pandemic, with special emphasis on risk mitigation.

Nachega, J. B., et al. (2021). "Contact Tracing and the COVID-19 Response in Africa: Best Practices, Key Challenges, and Lessons Learned from Nigeria, Rwanda, South Africa, and Uganda." Am J Trop Med Hyg **104**(4): 1179-1187.

Most African countries have recorded relatively lower COVID-19 burdens than Western countries. This has been attributed to early and strong political commitment and robust implementation of public health measures, such as nationwide lockdowns, travel restrictions, face mask wearing, testing, contact tracing, and isolation, along with community education and engagement. Other factors include the younger population age strata and hypothesized but yet-to-be confirmed partially protective cross-immunity from parasitic diseases and/or other circulating coronaviruses. However, the true burden may also be underestimated due to operational and resource issues for COVID-19 case identification and reporting. In this perspective article, we discuss selected best practices and challenges with COVID-19 contact tracing in Nigeria, Rwanda, South Africa, and Uganda. Best practices from these country case studies include sustained, multi-platform public communications; leveraging of technology innovations; applied public health expertise; deployment of community health workers; and robust community engagement. Challenges include an overwhelming workload of contact tracing and case detection for healthcare workers, misinformation and stigma, and poorly sustained adherence to isolation and quarantine. Important lessons learned include the need for decentralization of contact tracing to the lowest geographic levels of surveillance, rigorous use of data and technology to improve decision-making, and sustainment of both community sensitization and political commitment. Further research is needed to understand the role and importance of contact tracing in controlling community transmission dynamics in African countries, including among children. Also, implementation science will be critically needed to evaluate innovative, accessible, and cost-effective digital solutions to accommodate the contact tracing workload.

Nagler, A. R., et al. (2021). "Early Results from Severe Acute Respiratory Syndrome Coronavirus 2 Polymerase Chain Reaction Testing of Healthcare Workers at an Academic Medical Center in New York City." Clin Infect Dis **72**(7): 1241-1243.

Coronavirus disease 2019 (COVID-19) reverse-transcription polymerase chain reaction employee testing was implemented across New York University Langone Health. Over 8 weeks, 14 764 employees were tested; 33% of symptomatic employees, 8% of asymptomatic employees reporting COVID-19 exposure, and 3% of employees returning to work were positive. Positivity rates declined over time, possibly reflecting the importance of community transmission and efficacy of personal protective equipment.

Nakamura, K., et al. (2020). "A sporadic COVID-19 pneumonia treated with extracorporeal membrane oxygenation in Tokyo, Japan: A case report." J Infect Chemother **26**(7): 756-761.

INTRODUCTION: An ongoing outbreak of a novel coronavirus disease (coronavirus disease 2019, COVID-19) has become a global threat. While clinical reports from China to date demonstrate that the majority of cases remain relatively mild and recover with supportive care, it is also crucial to be well prepared for severe cases warranting intensive care. Initiating appropriate infection control measures may not always be achievable in primary care or in acute-care settings. CASE: A 45-year-old man was admitted to the intensive care unit due to severe pneumonia, later confirmed as COVID-19. His initial evaluation in the resuscitation room and treatments in the intensive care unit was performed under droplet and contact precaution with additional airborne protection using the N95 respirator mask. He was successfully treated in the intensive care unit with mechanical ventilation and extracorporeal membrane oxygenation for respiratory support; and antiretroviral treatment with lopinavir/ritonavir. His total intensive care unit stay was 15 days and was discharged on hospital day 24. CONCLUSIONS: Strict infection control precautions are not always an easy task, especially under urgent care in an intensive care unit. However, severe cases of COVID-19 pneumonia, or another novel infectious disease, could present at any moment and would be a continuing challenge to pursue appropriate measures. We need to be well prepared to secure healthcare workers from exposure to infectious diseases and nosocomial spread, as well as to provide necessary intensive care.

Nam, S. H. and J. C. Yang (2021). "COVID-19 Pandemic and Mental Health of Vulnerable Two Groups: Developmental Trauma of the Child-Adolescents and Work Disaster of Health Care Workers." Chonnam Med J **57**(1): 7-12.

COVID-19 has spread worldwide. People are struggling to adjust to a new normal, but changes in their daily routines are also causing stress. A person may feel depressed, uneasy, or suicidal and may complain of symptoms such as panic attacks, post-traumatic stress disease (PTSD), psychosis, obsessive-compulsive disorder, or paranoia when personal resilience cannot effectively process the stress. Children, adolescents, and health care workers are especially psychologically vulnerable groups in the pandemic calamity situation; therefore, a long-term intervention plan is necessary for them. When intervening with children and adolescents, it should be considered that each individual has different ways of expressing stress according to the developmental level of cognition, language, and emotion, and taking into account these developmental levels, it is necessary to help them achieve developmental tasks appropriate for their age. Health care workers feel psychological pain from problems such as the risk of becoming infected, the risk of passing the virus to their families, overwork, isolation, and stigma. Therefore, it is necessary to help them recover themselves by supplying personal protective equipment and providing the most basic resources necessary for adequate rest, work-life balance, and childcare.

Naqvi, S. H. S., et al. (2020). "Povidone-iodine solution as SARS-CoV-2 prophylaxis for procedures of the upper aerodigestive tract a theoretical framework." J Otolaryngol Head Neck Surg **49**(1): 77.

BACKGROUND: The COVID-19 pandemic has raised concerns of inadvertent SARS-CoV-2 transmission to healthcare workers during routine procedures of the aerodigestive tract in asymptomatic COVID-19 patients. Current efforts to mitigate this risk focus on Personal Protective Equipment, including high-efficiency filtration as well as other measures. Because the reservoir for SARS-CoV-2 shedding is in the nasopharynx and nasal and oral cavities, the application of viricidal agents to these surfaces may reduce virus burden. Numerous studies have confirmed that povidone-iodine inactivates many common respiratory viruses, including SARS-CoV-1. Povidone-iodine also has good profile for mucosal tolerance. Thus, we propose a prophylactic treatment protocol for the application of topical povidone-iodine to the upper aerodigestive tract. CONCLUSION: Such an approach represents a low-cost, low-morbidity measure that may reduce the risks associated with aerosol-generating procedures performed commonly in otorhinolaryngology operating rooms.

Nasrabadi, A. N., et al. (2021). "Challenges with healthcare workers' protection during the COVID-19 pandemic in Iran." Br J Oral Maxillofac Surg **59**(3): e114-e117.

During the COVID-19 pandemic, healthcare workers stand in the frontline of the fight against the disease. This study aimed to assess the challenges with personal protection during the pandemic in Tehran, Iran. Seven-hundred healthcare workers were surveyed between April and May 2020, and the study was performed in surgical care wards. Questionnaires, which were sent to 1000 workers via online platforms, social networks, and emails to different associations, were used to assess the use of personal protective equipment (PPE) and the reasons for not using it. A total of 700 replied. Face masks and face shields had the highest and lowest use, respectively. Equipment shortage, time constraint, low supervision, and lack of information were the reasons for personnel not using PPE. To reduce the burden of COVID-19 and accelerate the process of patient care, it is important to protect and maintain the well-being of staff. Organised national and international arrangements to protect them are critical.

Naveed, H., et al. (2020). "Ophthalmic Workplace Modifications for the Post-COVID Era." J Ophthalmic Vis Res **15**(3): 400-407.

The COVID-19 pandemic necessitates implementation of exposure control measures in all facets of the healthcare sector. Healthcare professionals who work in busy ophthalmology clinics and theaters are amidst the highest at-risk of contracting COVID-19. The authors review the up-to-date scientific evidence of SARS-CoV-2 transmission to demystify and explain the exposure control options available for ophthalmic workplace and offer insights from an industrial hygiene standpoint. As the we enter the post-COVID world, these measures will be critical to enhance workplace safety, and thus protect patients and staff alike.

Nayak, S., et al. (2021). "Virus containment box for retinopathy of prematurity screening and laser." Indian J Ophthalmol **69**(2): 406-408.

PURPOSE: Health care workers are susceptible to contracting infection with COVID-19 by aerosol transmission. This is a risk while examining and/or treating an un-sedated neonate in retinopathy of prematurity (ROP) screening and treatment. But screening for neonates for ROP and treating with laser, when required, should not be delayed to avoid the blindness. We describe a cost-effective method of containing aerosols generated during such a procedure in an un-sedated baby. METHODS: An acrylic transparent containment box was prepared to accommodate an average-sized infant. The box had four walls and a roof. The floor was open to place the container box over the baby. The walls have two types of openings, large ones to allow passage of hands to examine the baby, small ones to enable passage of oxygen tubing when required. A simulation was created to examine the impact of aerosol spray on examining healthcare personnel. RESULTS: The cost of the acrylic box was negligible. It could be assembled locally with available acrylic sheets and craftsmen. It was not difficult to examine the baby inside the box, and the simulation demonstrated that it protected the health personnel from the aerosol contamination. CONCLUSION: The described method is likely to increase healthcare personnel's confidence not to delay or deny ROP screening and laser treatment and save the babies from blindness.

Ndakwah, G. S., et al. (2021). "Infection Control in Interventional Radiology During the COVID-19 Era." Curr Probl Diagn Radiol **50**(3): 284-287.

The COVID-19 pandemic has challenged the capacity of interventional radiology departments worldwide to effectively treat COVID-19 and non-COVID-19 patients while preventing disease transmission among patients and healthcare workers. In this review, we describe the various data driven infection control measures implemented by the interventional radiology department of a large tertiary care center in the United States including the use and novel re-use of personal protective equipment, COVID-19 testing strategies, modifications in procedural workflows and the leveraging of telehealth visits. Herein, we provide effective triage, procedural, and management algorithms that may guide other interventional radiology departments during the ongoing COVID-19 pandemic and in future infectious disease outbreaks.

Nemat, A., et al. (2020). "A Survey of the Healthcare Workers in Afghanistan during the COVID-19 Pandemic." Am J Trop Med Hyg **104**(2): 537-539.

Healthcare workers (HCWs) in Afghanistan faced many challenges during the COVID-19 pandemic. A cross-sectional, online survey was conducted from July 4, 2020 to July 12, 2020 to evaluate the working conditions and health situation of HCWs in Afghanistan during the pandemic. Healthcare workers from 34 provinces, who were actively working in Afghan hospitals, were invited to participate in this study; 925 HCWs completed the survey. The results showed that 85% of the HCWs participated in the study were tested positive for COVID-19. This highlights the critical need of HCWs for personal protective equipment when caring for suspected and/or confirmed cases of the COVID-19.

Nemeth, O., et al. (2021). "An Experience of Public Dental Care during the COVID-19 Pandemic: Reflection and Analysis." Int J Environ Res Public Health **18**(4).

Since its emergence in China, the COVID-19 pandemic has become the number 1 health challenge in the world with all affected countries trying to learn from each other's experiences. When it comes to health services, dental care does not seem to be a priority area, despite the fact that it is among the highest risk medical specialisations in terms of spreading the infection. Using the Department of Community Dentistry of Semmelweis University as a case study, the objective of this paper is to introduce and analyze the system and organizational level measures, which have been implemented in dental care in Hungary during the first months of the COVID-19 outbreak. The system level measures to promote social distancing, to reduce the use of health services and to protect high risk health professionals, together with the deployment of protective equipment and the reorganization of patient pathways at the organizational level proved to be effective in keeping the outbreak in control. There are two, less frequently mentioned ingredients of successful coping with the COVID-19 challenge. First, mental health support is at least as important as physical protection. Second, most of the interventions do not require big financial investments, but behavioural change, which in turn requires leadership and change management skills.

Nepal, R. and B. Bhattarai (2020). "The Grim Reality of Health System Uncovered with COVID-19 Pandemic in Nepal." J Nepal Health Res Counc **18**(3): 569-571.

With advent of community transmission of COVID-19 in Nepal, the number of cases continues to rise and poses threat to the fragile health system of our country. 'Trace, isolate, test and treat' is the strategy advocated by World Health Organization to fight against COVID-19. Despite the efforts for last nine months, Nepal lacks in some aspect of this strategy. Lack of prompt testing facilities and substandard quarantine and isolation centers, have led to mismanagement of cases. The panic regarding COVID-19, lack of adequate protective measures to healthcare workers in early stage of the pandemic, and nation-wise lockdown, has led to collateral damage in the form of increased morbidity and mortality due to non-COVID related illnesses. COVID-19 pandemic has uncovered the grim reality of the debilitated health system of our country. With mass influx of Nepali migrant workers, the epidemic is expected to grow exponentially. We need to understand that the health system of Nepal must be prepared to function to its maximum capacity in the coming days. Keywords: COVID-19; health; Nepal; pandemic.

Neuwirth, M. M., et al. (2020). "Adherence to personal protective equipment use among healthcare workers caring for confirmed COVID-19 and alleged non-COVID-19 patients." Antimicrob Resist Infect Control **9**(1): 199.

Adherence observations of health care workers (HCW) revealed deficiencies in the use of recommended personal protective equipment (PPE) among HCW caring in COVID-19 and non-COVID-19 wards during the first period of the SARS-CoV-2 pandemic in a university hospital in Germany. The adherence to wearing surgical face or FFP2-masks and disinfecting hands prior to donning and after doffing the PPE was significantly higher in COVID-19 wards However, there was no total adherence of 100% in COVID-19 wards.

Newman, K. L., et al. (2021). "Experiences and emotional strain of NHS frontline workers during the peak of the COVID19 pandemic." Int J Soc Psychiatry: 207640211006153.

BACKGROUND: The mental health of the population has been negatively affected due to the pandemic. Frontline healthcare workers with increased exposure to COVID diagnosis, treatment and care were especially likely to report psychological burden, fear, anxiety and depression. AIM: To elicit how working as a health professional during the pandemic is impacting on the psychological wellbeing of frontline staff. METHOD: United Kingdom population of healthcare workers were approached by advertising the survey via social media, NHS trusts and other organisations. Open-ended survey answers were qualitatively explored using content analysis. RESULTS: Survey collected data from 395 NHS staff was developed into three themes; (1) Despair and uncertainty: feeling overwhelmed trying to protect everyone, (2) Behavioural and psychological impact: affecting wellbeing and functioning and (3) Coping and employer support: getting the right help. CONCLUSION: NHS staff felt enormous burden to adequately complete their professional, personal and civil responsibility to keep everyone safe leading to negative psychological and behavioural consequences and desire for NHS employers to offer better support. As the pandemic progresses, the results of this study may inform NHS employers on how optimum support can be offered to help them cope with negative psychological consequences of the pandemic.

Nguyen, L. H., et al. (2020). "Risk of COVID-19 among frontline healthcare workers and the general community: a prospective cohort study." medRxiv.

BACKGROUND: Data for frontline healthcare workers (HCWs) and risk of SARS-CoV-2 infection are limited and whether personal protective equipment (PPE) mitigates this risk is unknown. We evaluated risk for COVID-19 among frontline HCWs compared to the general community and the influence of PPE. METHODS: We performed a prospective cohort study of the general community, including frontline HCWs, who reported information through the COVID Symptom Study smartphone application beginning on March 24 (United Kingdom, U.K.) and March 29 (United States, U.S.) through April 23, 2020. We used Cox proportional hazards modeling to estimate multivariate-adjusted hazard ratios (aHRs) of a positive COVID-19 test. FINDINGS: Among 2,035,395 community individuals and 99,795 frontline HCWs, we documented 5,545 incident reports of a positive COVID-19 test over 34,435,272 person-days. Compared with the general community, frontline HCWs had an aHR of 11·6 (95% CI: 10·9 to 12·3) for reporting a positive test. The corresponding aHR was 3·40 (95% CI: 3·37 to 3·43) using an inverse probability weighted Cox model adjusting for the likelihood of receiving a test. A symptom-based classifier of predicted COVID-19 yielded similar risk estimates. Compared with HCWs reporting adequate PPE, the aHRs for reporting a positive test were 1·46 (95% CI: 1·21 to 1·76) for those reporting PPE reuse and 1·31 (95% CI: 1·10 to 1·56) for reporting inadequate PPE. Compared with HCWs reporting adequate PPE who did not care for COVID-19 patients, HCWs caring for patients with documented COVID-19 had aHRs for a positive test of 4·83 (95% CI: 3·99 to 5·85) if they had adequate PPE, 5·06 (95% CI: 3·90 to 6·57) for reused PPE, and 5·91 (95% CI: 4·53 to 7·71) for inadequate PPE. INTERPRETATION: Frontline HCWs had a significantly increased risk of COVID-19 infection, highest among HCWs who reused PPE or had inadequate access to PPE. However, adequate supplies of PPE did not completely mitigate high-risk exposures. FUNDING: Zoe Global Ltd., Wellcome Trust, EPSRC, NIHR, UK Research and Innovation, Alzheimer's Society, NIH, NIOSH, Massachusetts Consortium on Pathogen Readiness.

Nicastro, E., et al. (2020). "A Pediatric Emergency Department Protocol to Avoid Intrahospital Spread of SARS-CoV-2 during the Outbreak in Bergamo, Italy." J Pediatr **222**: 231-235.

The pandemic of coronavirus SARS-CoV-2 disease affected Northern Italy, spreading from the Bergamo province to the entire country. During reorganization of our emergency department to support patients presenting with coronavirus SARS-CoV-2 disease, we aimed to evaluate whether children play a role in intrahospital spread of the infection.

Nichetti, F., et al. (2020). "COVID-19 risk for patients undergoing anticancer treatment at the outpatient clinic of the National Cancer Institute of Milan: the COVINT study." ESMO Open **5**(Suppl 3).

BACKGROUND: In the midst of the COVID-19 pandemic, patients with cancer are regarded as a highly vulnerable population. Overall, those requiring hospital admission for treatment administration are potentially exposed to a higher risk of infection and worse outcome given the multiple in-hospital exposures and the treatment immunosuppressive effects. METHODS: COVINT is an observational study assessing COVID-19 incidence among patients receiving anticancer treatment in the outpatient clinic of the Istituto Nazionale dei Tumori di Milano. All consecutive patients with non-haematological malignancies treated with intravenous or subcutaneous/intramuscular anticancer therapy in the outpatient clinic were enrolled. The primary endpoint is the rate of occurrence of COVID-19. Secondary endpoints included the rate of COVID-19-related deaths and treatment interruptions. The association between clinical and biological characteristics and COVID-19 occurrence is also evaluated. COVID-19 diagnosis is defined as (1) certain if confirmed by reverse transcriptase PCR assay of nasopharyngeal swabs (NPS); (2) suspected in case of new symptoms or CT scan evidence of interstitial pneumonia with negative/not performed NPS; (3) negative in case of neither symptoms nor radiological evidence. RESULTS: In the first 2 months (16 February-10 April 2020) of observation, 1081 patients were included. Of these, 11 (1%) were confirmed and 73 (6.7%) suspected for COVID-19. No significant differences in terms of cancer and treatment type emerged between the three subgroups. Prophylactic use of myeloid growth factors was adopted in 5.3%, 2.7% and 0% of COVID-19-free, COVID-19-suspected and COVID-19-confirmed patients (p=0.003). Overall, 96 (8.9%) patients delayed treatment as a precaution for the pandemic. Among the 11 confirmed cases, 6 (55%) died of COVID-19 complications, and anticancer treatment was restarted in only one. CONCLUSIONS: During the pandemic peak, accurate protective measures successfully resulted in low rates of COVID-19 diagnosis, although with high lethality. Prospective patients' surveillance will continue with NPS and serology testing to provide a more comprehensive epidemiological picture, a biological insight on the impact of cytotoxic treatments on the immune response, and to protect patients and healthcare workers.

Nichols, B. E., et al. (2021). "CPR during COVID-19: Use of Expert-driven Rapid Cycle Deliberate Practice to Implement PALS Guidelines." Pediatr Qual Saf **6**(1): e374.

The American Heart Association (AHA) and other national institutions have endorsed modifications to resuscitation guidelines given the risk of healthcare workers' (HCWs) exposure to COVID-19. Institutional implementation of the COVID-19-focused guidelines requires both proof of feasibility and education of HCW. Pediatric critical care medical directors at The University of Texas Southwestern/Children's Health System of Texas (UTSW/CHST) created a guideline for the resuscitation of COVID-19 patients. The simulation team used in situ simulation to demonstrate guideline feasibility and to create educational materials. METHODS: A UTSW/CHST guideline incorporated COVID-19-focused AHA and other national organizational recommendations to fit the institutional needs. A high-fidelity in situ simulation helped test the feasibility and optimize the UTSW/CHST guideline. We developed a novel form of rapid cycle deliberate practice (RCDP), expert-driven RCDP, in which all simulation participants are experts, to debrief the simulation. RESULTS: In situ simulation with expert-driven RCDP demonstrated guideline feasibility in the resuscitation of a COVID-19 patient while balancing the protection of HCW. Expert-driven RCDP allowed for real-time alterations to the guideline during the simulation event. Video recording and dissemination of the simulation allowed for the education of over 300 staff on the new recommendations. CONCLUSIONS: High-fidelity in situ simulation with expert-driven RCDP created a rapid consensus among expert critical care providers to develop the UTSW/CHST guideline and quickly adopt the new AHA recommendations. This debriefing method helped minimize the risk of HCW exposure by minimizing the number of required participants and time for simulation. We recommend using this distinctive, expert-driven RCDP debriefing method for expeditious testing of COVID-19-focused processes at other institutions.Video Abstract available at: [link forthcoming].

Nicholson, P. J. and D. Sen (2021). "Healthcare workers and protection against inhalable SARS-CoV-2 aerosols." Occup Med (Lond).

Niikura, R., et al. (2021). "International Observational Survey of the Effectiveness of Personal Protective Equipment during Endoscopic Procedures Performed in Patients with COVID-19." Digestion: 1-9.

INTRODUCTION AND AIMS: This international survey was performed to evaluate the cumulative incidence of nosocomial novel coronavirus disease 2019 (COVID-19) among healthcare professionals during endoscopic procedures. METHODS: We performed an international web-based self-reported questionnaire survey. Participants completed the questionnaires every week for 12 weeks. The questionnaire elicited responses regarding the development of COVID-19 and details of the personal protective equipment (PPE) used. RESULTS: All 483 participants were included in the analysis. Participants had a mean age of 42.3 years and comprised 68.3% males. The geographic distribution of the study population was Asia (89.2%), Europe (2.9%), North and South America (4.8%), Oceania (0.6%), and Africa (1.5%). The most common endoscopy-related role of the participants was endoscopist (78.7%), and 74.5% had >10 years of experience. Fourteen participants had performed 83 endoscopic procedures in patients positive for COVID-19. During the mean follow-up period of 4.95 weeks, there were no cases of COVID-19 when treating COVID-19 positive patients. The most common PPE used by participants treating patients with COVID-19 was a surgical mask plus N95 mask plus face shield, goggles, cap, long-sleeved isolation gown, and single pair of gloves. The most common PPE used by participants treating patients without COVID-19 was a surgical mask, no face shield but goggles, cap, long-sleeved isolation gown, and single pair of gloves during all endoscopic procedures. CONCLUSIONS: The risk of COVID-19 transmission during any endoscopic procedure was low in clinical practice.

Ning, X., et al. (2020). "The mental health of neurological doctors and nurses in Hunan Province, China during the initial stages of the COVID-19 outbreak." BMC Psychiatry **20**(1): 436.

BACKGROUND: Neurological symptoms are increasingly being noted among COVID-19 patients. Currently, there is little data on the mental health of neurological healthcare workers. The aim of this study was to identify the prevalence and influencing factors on anxiety and depression in neurological healthcare workers in Hunan Province, China during the early stage of the Coronavirus Disease 2019 (COVID-19) outbreak. METHODS: An online cross-sectional study was conducted among neurological doctors and nurses in early February 2020 in Hunan Province. Symptoms of anxiety and depression were assessed by the Chinese version of the Self-Rating Anxiety Scale (SAS) (defined as a total score ≥ 50) and Self-Rating Depression Scale (SDS) (defined as a total score ≥ 53). The prevalences of probable anxiety and depression were compared between different groups, and multivariate logistic regression analysis was used to understand the independent influencing factors on anxiety and depression. RESULTS: The prevalence of probable anxiety and depression in neurological nurses (20.3 and 30.2%, respectively) was higher than that in doctors (12.6 and 20.2%, respectively). Female healthcare workers (18.4%) had a higher proportion of anxiety than males (10.8%). Probable anxiety and depression were more prevalent among nurses, younger workers (≤ 40 years), and medical staff with junior titles. Logistic regression analysis showed that a shortage of protective equipment was independently associated with probable anxiety (OR = 1.980, 95% CI: 1.241-3.160, P = 0.004), while young age was a risk factor for probable depression (OR = 2.293, 95% CI: 1.137-4.623, P = 0.020) among neurological healthcare workers. CONCLUSIONS: Probable anxiety and depression were more prevalent among neurological nurses than doctors in Hunan Province. The shortage of protective equipment led to probable anxiety, and young age led to probable depression in healthcare workers in neurology departments, which merits attention during the battle against COVID-19.

Nioi, M., et al. (2020). "COVID-19 and Italian Healthcare Workers From the Initial Sacrifice to the mRNA Vaccine: Pandemic Chrono-History, Epidemiological Data, Ethical Dilemmas, and Future Challenges." Front Public Health **8**: 591900.

On March 11, 2020, the World Health Organization (WHO) declared the coronavirus disease 2019 (COVID-19) outbreak a pandemic. Simultaneously, in Italy, in which the first case had occurred on February 18, the rigid phase of the lockdown began. The country has attracted worldwide attention, becoming at the same time a field of study both concerning the spread of the pandemic and advanced assessments of the effectiveness of political, public health, and therapeutic measures. The protagonists of the Italian crisis were the healthcare workers (HCWs) who were exposed to severe acute respiratory syndrome coronavirus 2 (SARS-CoV-2) without having any perception of what they were facing, courageously contributing to the containment of the epidemic to be defined by the media as "heroes." However, in the first phase of the pandemic (March-May 2020), the price that the Italian Public Health System had to pay both in terms of the number of positive virus cases and deaths among the HCWs was beyond and represented a peculiarity compared to what happened in other countries. In the current study, after a summary of the evolution of the pandemic in Italy, we offer an analysis of the statistical data concerning contagions and deaths among healthcare workers (physicians in particular). In conclusion, we describe the critical issues that still need to be resolved and the future challenges facing healthcare workers and the general population.

Nishimura, H., et al. (2020). "New applications of a portable isolation hood for use in several settings and as a clean hood." J Thorac Dis **12**(7): 3500-3506.

BACKGROUND: We previously reported that we developed a compact and portable isolation hood that covers the top half of a patient sitting or lying in bed. The negative pressure inside the hood is generated by a fan-filter-unit (FFU) through which infectious aerosols from a patient are filtered. The outside area is kept clean which decreases the risk of nosocomial infections in hospital wards. We tried new applications of the hood. METHODS: The negative pressure hood was newly applied in an intensive care unit (ICU) as a place where a staff performs the practice of suctioning that generates much aerosol from the patient, as well as a waiting space for patients. Furthermore, the possibility that the hood can be converted to a positive pressure hood as a clean hood by switching the airflow direction of FFU was assessed. The cleaning efficacy of the inside of the hood was tested using an aerosolized cultured influenza virus tracer and an optimal airflow rate was determined according to the test results. RESULTS: The hood, named Barrihood, was found to be competent to be used (I) for tracheal suctioning in ICU, (II) as a waiting space for a child in a nursery who suddenly showed symptoms of the disease and waiting to be picked-up by the guardian, and (III) as a waiting space in a special outpatient clinic in a hospital for COVID-19 suspected cases to prevent dissemination of airborne pathogens. The positive pressure hood was also competent in keeping clean air quality that meets the standard class 100 of NASA's bio-clean room category. CONCLUSIONS: The proposed new applications will broaden the range of the hood's usage. The isolation hood could be useful in many settings to protect people outside the hood from a patient inside, or to protect an individual inside from air particles outside the hood, such as airborne pathogens, allergens, or hazardous particulate matter like PM2.5.

Nishio, N., et al. (2021). "Surgical strategy and optimal timing of tracheostomy in patients with COVID-19: Early experiences in Japan." Auris Nasus Larynx **48**(3): 518-524.

OBJECTIVE: Tracheostomy is an important surgical procedure for coronavirus disease-2019 (COVID-19) patients who underwent prolonged tracheal intubation. Surgical indication of tracheostomy is greatly affected by the general condition of the patient, comorbidity, prognosis, hospital resources, and staff experience. Thus, the optimal timing of tracheostomy remains controversial. METHODS: We reviewed our early experience with COVID-19 patients who underwent tracheostomy at one tertiary hospital in Japan from February to September 2020 and analyzed the timing of tracheostomy, operative results, and occupational infection in healthcare workers (HCWs). RESULTS: Of 16 patients received tracheal intubation with confirmed severe acute respiratory syndrome coronavirus 2 (SARS-CoV-2) infection, five patients (31%) received surgical tracheostomy in our hospital. The average consultation time for surgical tracheostomy was 7.4 days (range, 6 - 9 days) from the COVID-19 team to the otolaryngologist. The duration from tracheal intubation to tracheostomy ranged from 14 to 27 days (average, 20 days). The average time of tracheostomy was 27 min (range, 17 - 39 min), and post-wound bleeding occurred in only one patient. No significant differences in hemoglobin (Hb) levels were found between the pre- and postoperative periods (mean: 10.2 vs. 10.2 g/dl, p = 0.93). Similarly, no difference was found in white blood cell (WBC) count (mean: 12,200 vs. 9,900 cells /µl, p = 0.25). After the tracheostomy, there was no occupational infection among the HCWs who assisted the tracheostomy patients during the perioperative period. CONCLUSION: We proposed a modified weaning protocol and surgical indications of tracheostomy for COVID-19 patients and recommend that an optimal timing for tracheostomy in COVID-19 patients of 2 - 3 weeks after tracheal intubation, from our early experiences in Japan. An experienced multi-disciplinary tracheostomy team is essential to perform a safe tracheostomy in patients with COVID-19 and to minimize the risk of occupational infection in HCWs.

Nogee, D. and A. J. Tomassoni (2020). "Covid-19 and the N95 respirator shortage: Closing the gap." Infect Control Hosp Epidemiol **41**(8): 958.

Due to extreme shortages of personal protective equipment caused by the COVID-19 pandemic, many healthcare workers will be forced to recycle protective masks intended for disposal after a single use. We propose investigating the use of ultraviolet germicidal irradiation to sterilize masks of SARS-CoV-2 for safer reuse.

Nogueira, M. S. (2020). "Ultraviolet-based biophotonic technologies for control and prevention of COVID-19, SARS and related disorders." Photodiagnosis Photodyn Ther **31**: 101890.

• The COVID-19 pandemic led to a global shortage of personal protective equipment (PPE), physicians, negative pressure rooms, and hospital beds. • UV strategies have been developed to extend the lifespan of medical equipment as well as disinfect environmental surfaces. • Regulatory mechanisms to speed up development and approval of technologies and medicines against COVID-19 have been issued. • Opportunities to scale the production of UV devices for microbial inactivation are vast. • Development of reliable and safe technology to ensure the safety of healthcare workers and contain the virus spreading is the main priority.

Nohuz, E., et al. (2020). "Technics to put on and remove personal protective equipment before surgical or obstetrical procedure in suspected or infected COVID-19 patients (with video)." J Gynecol Obstet Hum Reprod **49**(9): 101859.

Coronavirus pandemic is widely changing our professional daily practice and preventive measures must be taken and taught. Before any planned gynaecological or obstetric surgery, specific technics to put on and safely remove personal protective equipment should be implemented in order to avoid any contamination for both patients and healthcare workers.

Noor Azhar, M., et al. (2020). "COVID-19 aerosol box as protection from droplet and aerosol contaminations in healthcare workers performing airway intubation: a randomised cross-over simulation study." Emerg Med J **38**(2): 111-117.

BACKGROUND: Concerns over high transmission risk of SARS-CoV-2 have led to innovation and usage of an aerosol box to protect healthcare workers during airway intubation in patients with COVID-19. Its efficacy as a barrier protection in addition to the use of a standard personal protective equipment (PPE) is not fully known. We performed a simulated study to investigate the relationship between aerosol box usage during intubation and contaminations on healthcare workers pre-doffing and post-doffing of PPE. METHODS: This was a randomised cross-over study conducted between 9 April to 5 May 2020 in the ED of University Malaya Medical Centre. Postgraduate Emergency Medicine trainees performed video laryngoscope-assisted intubation on an airway manikin with and without an aerosol box in a random order. Contamination was simulated by nebulised Glo Germ. Primary outcome was number of contaminated front and back body regions pre-doffing and post-doffing of PPE of the intubator and assistant. Secondary outcomes were intubation time, Cormack-Lehane score, number of intubation attempts and participants' feedback. RESULTS: Thirty-six trainees completed the study interventions. The number of contaminated front and back body regions pre-doffing of PPE was significantly higher without the aerosol box (all p values<0.001). However, there was no significant difference in the number of contaminations post-doffing of PPE between using and not using the aerosol box, with a median contamination of zero. Intubation time was longer with the aerosol box (42.5 s vs 35.5 s, p<0.001). Cormack-Lehane scores were similar with and without the aerosol box. First-pass intubation success rate was 94.4% and 100% with and without the aerosol box, respectively. More participants reported reduced mobility and visibility when intubating with the aerosol box. CONCLUSIONS: An aerosol box may significantly reduce exposure to contaminations but with increased intubation time and reduced operator's mobility and visibility. Furthermore, the difference in degree of contamination between using and not using an aerosol box could be offset by proper doffing of PPE.

Noor, M., et al. (2020). "Does Working in a COVID-19 Receiving Health Facility Influence Seroprevalence to SARS-CoV-2?" Cureus **12**(11): e11389.

Objective In this study, we aimed at comparing the seroprevalence of severe acute respiratory syndrome coronavirus 2 (SARS-CoV-2) antibodies in healthcare workers (HCWs) in coronavirus disease 2019 (COVID-19) receiving and non-COVID-19 receiving hospitals in Peshawar, Pakistan. Methods This cross-sectional analytical study was conducted in a COVID-19 receiving hospital (hospital 'A') and a non-COVID-19 receiving hospital (hospital 'B'). Using stratified random sampling, 1,011 HCWs (439 from hospital 'A' and 572 from hospital 'B') were recruited to participate in the study. Immunoglobulin G/immunoglobulin M (IgG/IgM) antibodies were checked using Elecsys® (Roche, Basel, Switzerland) Anti-SARS-CoV-2 immunoassay. The chi-squared test was used to compare frequencies, and the binary logistic regression model was used to predict the association between study variables' seropositivity to SARS-CoV-2. A p-value of <0.05 was considered statistically significant. Results The overall seroprevalence to SARS-CoV-2 antibodies in the two hospitals was 30.76%. It was 28.2% in hospital 'A' and 32.7% in hospital 'B' (p=0.129). The seroprevalence in HCWs having direct contact with COVID-19 patients was higher (33.1%) in non-COVID-19 receiving hospital versus 23.8% in COVID-19 receiving hospital (p=0.034). Seroprevalence was highest among administrative staff (44.0%), followed by nurses (30.8%), residents (19.8%), and consultants (17.8%) (p=0.001). As compared to consultants, the administrative and nursing staff were 3.398 and 3.116 times more likely to have positive antibodies, respectively. There were no significant differences in the seroprevalence between the respective categories of staff of the two hospitals. Conclusions The non-COVID-19 receiving hospital had a higher proportion of seropositive HCWs than the COVID-19 receiving hospital. The HCWs in the non-COVID-19 receiving hospital who had direct contact with patients had significantly higher seroprevalence. Seroprevalence was highest for administrative staff followed by nursing staff, residents, and consultants. Regardless of the COVID-19 status of the healthcare facility, all HCWs shall be trained on, and consistently follow, the proper protocols for donning and doffing of personal protective equipment (PPE).

Noor, R. and S. M. Maniha (2020). "A brief outline of respiratory viral disease outbreaks: 1889-till date on the public health perspectives." Virusdisease **31**(4): 1-9.

Severe acute respiratory syndrome coronavirus 2 (SARS-CoV-2) is currently causing the respiratory illness termed as the coronavirus disease 2019 or the COVID-19 pandemic. Indeed, the significant increase in deaths in the current days due to influenza around the world started in 1889 is a continued public health threat because of its intermittent style of pandemic outbreaks. An array of research on the influenza viruses has been conducted especially pointing on (1) the development of the anti-viral drugs and the design of probable vaccines on trial basis, (2) the biochemical and genetic aspects underlying the viral pathogenicity, (3) the viral epidemiology, and on (4) the protective immunity against the influenza viruses. Current review briefly discussed the epidemic/ pandemic history of influenza and correlated with the current epidemiology, the possible preventive measures that may be taken by the public health professionals as well as to increase the protective awareness among the general people. The viral reassortments during the initiation of pandemics have also been focused based on the previous literatures.

Noorimotlagh, Z., et al. (2021). "A systematic review of possible airborne transmission of the COVID-19 virus (SARS-CoV-2) in the indoor air environment." Environ Res **193**: 110612.

At the end of December 2019, the rapid spread of the COVID-19 (SARS-CoV-2) disease and, subsequently, deaths around the world, lead to the declaration of the pandemic situation in the world. At the beginning of the epidemic, much attention is paid to person-to-person transmission, disinfection of virus-contaminated surfaces, and social distancing. However, there is much debate about the routes of disease transmission, including airborne transmission, so it is important to elucidate the exact route of transmission of the COVID-19 disease. To this end, the first systematic review study was conducted to comprehensively search all databases to collect studies on airborne transmission of SARS-CoV-2 in indoor air environments. In total, 14 relevant and eligible studies were included. Based on the findings, there is a great possibility of airborne transmission of SARS-CoV-2 in indoor air environments. Therefore, some procedures are presented such as improving ventilation, especially in hospitals and crowded places, and observing the interpersonal distance of more than 2 m so that experts in indoor air quality consider them to improve the indoor air environments. Finally, in addition to the recommendations of the centers and official authorities such as hand washing and observing social distancing, the route of air transmission should also be considered to further protect health personnel, patients in hospitals, and the public in other Public Buildings.

Nordio, M., et al. (2021). "Risk factors and action thresholds for the novel coronavirus pandemic. Insights from the Italian Society of Nephrology COVID-19 Survey." J Nephrol **34**(2): 325-335.

BACKGROUND AND AIM: Over 80% (365/454) of the nation's centers participated in the Italian Society of Nephrology COVID-19 Survey. Out of 60,441 surveyed patients, 1368 were infected as of April 23rd, 2020. However, center-specific proportions showed substantial heterogeneity. We therefore undertook new analyses to identify explanatory factors, contextual effects, and decision rules for infection containment. METHODS: We investigated fixed factors and contextual effects by multilevel modeling. Classification and Regression Tree (CART) analysis was used to develop decision rules. RESULTS: Increased positivity among hemodialysis patients was predicted by center location [incidence rate ratio (IRR) 1.34, 95% confidence interval (CI) 1.20-1.51], positive healthcare workers (IRR 1.09, 95% CI 1.02-1.17), test-all policy (IRR 5.94, 95% CI 3.36-10.45), and infected proportion in the general population (IRR 1.002, 95% CI 1.001-1.003) (all p < 0.01). Conversely, lockdown duration exerted a protective effect (IRR 0.95, 95% CI 0.94-0.98) (p < 0.01). The province-contextual effects accounted for 10% of the total variability. Predictive factors for peritoneal dialysis and transplant cases were center location and infected proportion in the general population. Using recursive partitioning, we identified decision thresholds at general population incidence ≥ 229 per 100,000 and at ≥ 3 positive healthcare workers. CONCLUSIONS: Beyond fixed risk factors, shared with the general population, the increased and heterogeneous proportion of positive patients is related to the center's testing policy, the number of positive patients and healthcare workers, and to contextual effects at the province level. Nephrology centers may adopt simple decision rules to strengthen containment measures timely.

Norful, A. A., et al. (2021). "Primary drivers and psychological manifestations of stress in frontline healthcare workforce during the initial COVID-19 outbreak in the United States." Gen Hosp Psychiatry **69**: 20-26.

OBJECTIVE: The purpose of this study was to understand the physical and psychological impact of high stress clinical environments and contributory factors of burnout in multidisciplinary healthcare workforce during the initial outbreak of COVID-19. METHOD: In-person qualitative interviews informed by an adaptation of Karasek's Job Demand-control model were conducted with a convenience sample of healthcare workforce from March to April 2020. RESULTS: Themes emerging from interviews coalesced around three main areas: fear of uncertainty, physical and psychological manifestations of stress, and resilience building. Shifting information, a lack of PPE, and fear of infecting others prompted worry for those working with Covid-infected patients. Participants reported that stress manifested more psychologically than physically. Individualized stress mitigation efforts, social media and organizational transparency were reported by healthcare workers to be effective against rising stressors. CONCLUSION: COVID-19 has presented healthcare workforce with unprecedented challenges in their work environment. With attention to understanding stressors and supporting clinicians during healthcare emergencies, more research is necessary in order to effectively promote healthcare workforce well-being.

Norkiene, I., et al. (2021). "'Should I Stay, or Should I Go?' Psychological Distress Predicts Career Change Ideation among Intensive Care Staff in Lithuania and the UK Amid COVID-19 Pandemic." Int J Environ Res Public Health **18**(5).

The COVID-19 pandemic had a significant effect on healthcare globally. Additional pressure created by coronavirus adversely affected the mental health and psychological well-being of healthcare workers, leading many to question their desire and willingness to continue working in healthcare. This study aimed to identify predictors for career change ideation among healthcare professionals in two countries; Lithuania and the United Kingdom amid the coronavirus pandemic. In total, 610 healthcare professionals from Lithuania and the UK (285 and 325, respectively) participated in a survey from May to August 2020. Psychological distress and psychological well-being were measured using the self-report scales "DASS-21" and "WHO-5". Almost half of the sample (49.2%), 59.6% and 40.0% in Lithuanian and the UK, respectively, exhibited career change ideation, the country effect was significant (AOR = 2.21, p < 0.001). Stronger ideation to leave healthcare was predicted by higher levels of depression (AOR = 1.10, p = 0.005), stress (AOR = 1.10, p = 0.007), anxiety surrounding inadequate personal protective equipment (AOR = 2.27, p = 0.009), and lower psychological well-being scores (AOR = 1.10, p = 0.007). We conclude that psychosocial support must be provided for healthcare professionals to prevent burnout and loss of staff amid the pandemic.

Ntshalintshali, S. and C. Mnqwazi (2020). "Affordable digital innovation to reduce SARS-CoV-2 transmission among healthcare workers." S Afr Med J **110**(7): 605-606.

In the South African public healthcare sector, patient medical records are still written on paper and stored in filing rooms. There has been an attempt to move towards a paperless electronic system in many public healthcare facilities, but owing to lack of funding, this has been a challenge to achieve. During the current COVID-19 pandemic, the virus could be transmitted through the physical manipulation of patient records by various categories of staff who handle the records with or without gloves for protection. We discuss a digital option that has been partially used at Tygerberg Hospital (TBH), Cape Town, to avoid SARS-CoV-2 patient hard-copy record manipulation. It includes assignment of a QR code to every patient admitted as a person under investigation or confirmed COVID-19 case. The QR code is synced to one of the many free online medical notes smartphone applications (apps), which are password-protected with patient information privacy regulations (Trello is used at TBH), for daily medical notes review and editing. Upon discharge, all notes made during the patient's hospital stay, together with the discharge summary, are printed to generate a hard copy of notes for filing to avoid violation of the current national and provincial patient records policy. Doing this means that a patient will have a virtual online file through the designated app until discharge, when a physical file will be made for storage and safekeeping. It will keep physical manipulation of patient records to the minimum, and potentially assist in reducing transmission of the SARS-CoV-2 virus among healthcare workers.

Nuñez, J. H., et al. (2020). "Rethinking Trauma Hospital Services in one of Spain's Largest University Hospitals during the COVID-19 pandemic. How can we organize and help? Our experience." Injury **51**(12): 2827-2833.

INTRODUCTION: The severe disruptions caused by the SARS-CoV-2 coronavirus have necessitated a redistribution of resources to meet hospitals' current service needs during this pandemic. The aim is to share our experiences and outcomes during the first month of the Covid-19 pandemic, based on the strategies recommended and strategies we have implemented. METHODS: Our experience comes from our work at a referral hospital within the Spanish National Health System. Changes to clinical practice have largely been guided by the current evidence and four main principles: (1) patient and health-care worker protection, (2) uninterrupted necessary care, (3) conservation of health-care resources, (4) uninterrupted formation for residents. Based on these principles, changes in the service organization, elective clinical visits, emergency visits, surgical procedures, and inpatient and outpatient care were made. RESULTS: Using the guidance of experts, we were able to help the hospital address the demands of the Covid-19 outbreak. We reduced to a third of our orthopaedics and trauma hospital beds, provided coverage for general emergency services, and five ICUs, all continuing to provide care for our patients, in the form of 102 trauma surgeries, 6413 phone interviews and 520 emergency clinic visits. Also in the third week, we were able to restart morning meetings via telematics, and teaching sessions for our residents. On the other hand, eight of the healthcare personnel on our service (10.8%) became infected with Covid-19. CONCLUSIONS: As priorities and resources increasingly shift towards the COVID-19 pandemic, it is possible to maintain the high standard and quality of care necessary for trauma and orthopaedics patients while the pandemic persists. We must be prepared to organize our healthcare workers in such a way that the needs of both inpatients and outpatients are met. It is still possible to operate on those patients who need it. Unfortunately, some healthcare workers will become infected. It is essential that we protect those most susceptible to severer consequences of Covid-19. Also crucial are optimized protective measures.

Núñez-Mujica, G., et al. (2020). "Data Analysis of Infection Rates Among Exposed Healthcare Workers Could Reveal Effective Prophylactics Against SARS-CoV-2." Adv Exp Med Biol **1288**: 1-3.

The global crisis provoked by the SARS-CoV-2 pandemic, and the economic and social consequences associated to the essential policies applied to contain it, necessitates the expedited development of therapeutic solutions. It is a priority to produce data both rapidly and accurately in order to identify current therapies that can be repurposed to offer protection from SARS-CoV-2 infection. As healthcare workers are both at high risk for infection and able to be readily diagnosed, they offer a potential wealth of data to be analyzed. A systematic data analysis of exposure and infection rates among healthcare workers could yield patterns identifying common protective factors, such as medications with prophylactic potential against SARS-CoV-2, that can be fast-tracked into available therapies. With results suggesting their activity against other coronaviruses and their widespread adoption, Antiretroviral cocktails could be a promising initial target for such large-scale data analysis approach.

Obaseki, D. E., et al. (2020). "Staff risks stratification in preparation for COVID-19 in a tertiary healthcare facility in Nigeria." Pan Afr Med J **35**(Suppl 2): 124.

INTRODUCTION: this report is a documentation of a staff risk stratification programme, undertaken in University of Benin Teaching Hospital, with outcomes, and the actions taken to protect staff. METHODS: an adapted risk stratification tool was circulated to all staff through their respective heads of departments/units. Staff were expected to voluntary assess their health and risk status in the context of COVID-19, using the tool. A central multi-disciplinary screening committee assessed submissions and invited staff who required further evaluation for physical interviews. Respondents were categorized into three risk/exposure groups from lowest to highest - A, B, and C, based on their individual health assessments, occupational exposures, and information obtained from direct interviews. RESULTS: the committee received submissions from 746 staff, representing 19.4% (about a fifth) of the hospital's 3,840 staff. One hundred and twenty two of these were invited for physical interviews, of whom 88 (72.1%) were categorized as high risk (Category C): pregnancy (53.4%); bronchial asthma (19.3%); hypertension (11.4%); cancer (3.4%) and sickle cell disease (2.3%); fractures and pulmonary tuberculosis (1%, respectively). These staff were recommended for redeployment from areas of high risk exposure to COVID-19. CONCLUSION: a management-driven risk assessment of hospital staff in preparation for the COVID-19 pandemic revealed that a fifth of staff assessed themselves as being vulnerable to adverse outcomes from exposure. It is our hope that similar risk stratification programmes will become standard practice in healthcare facilities during disease outbreaks, especially in Africa.

O'Byrne, C., et al. (2021). "Knowledge of face masks and attitudes to re-sterilisation among healthcare workers." Ir J Med Sci: 1-6.

INTRODUCTION: COVID-19 was declared a pandemic in March 2020. With the sudden surge in demand for personal protective equipment (PPE), significant concerns regarding the ongoing availability emerged. One solution proposed is re-sterilisation of respirator masks and this has been commenced in some parts of the world. On review of the literature, very little is documented regarding the knowledge of masks and the attitudes of healthcare workers towards using re-sterilised masks. METHODS: A comprehensive questionnaire was used to assess general knowledge and attitudes around facemasks and respirators. RESULTS: There were 190 respondents. There were significant gaps in knowledge and understanding of when particular face masks should be worn. One-third had significant concerns about ongoing availability. One-third had concerns about the quality of the masks as the pandemic continued. Only 10% of respondents underwent formal face-fitting. Eighty percent of respondents stated they would wear a re-sterilised mask. A further 15% would use a re-sterilised mask but required certain reassurances. Five percent of our respondents would not use a re-sterilised mask under any circumstances. DISCUSSION: Ensuring an adequate understanding of face masks is crucial among healthcare workers (HCWs) and this study highlights a need for further education. It also demonstrates a general acceptability among HCWs towards the use of re-sterilised face masks.

Odor, P. M., et al. (2020). "Anaesthesia and COVID-19: infection control." Br J Anaesth **125**(1): 16-24.

The world is currently facing an unprecedented healthcare crisis caused by a pandemic novel beta coronavirus, severe acute respiratory syndrome coronavirus 2 (SARS-CoV-2). The pathogen is spread by human-to-human transmission via droplets exposure and contact transfer, causing mild symptoms in the majority of cases, but critical illness, bilateral viral pneumonia, and acute respiratory distress syndrome (ARDS) in a minority. Currently, controlling infection to prevent the spread of SARS-CoV-2 is the primary public healthcare intervention used. The pace of transmission and global scale of SARS-CoV-2 infections has implications for strategic oversight, resource management, and responsiveness in infection control. This article presents a summary of learning points in epidemiological infection control from the SARS epidemic, alongside a review of evidence connecting current understanding of the virologic and environmental contamination properties of SARS-CoV-2. We present suggestions for how personal protective equipment policies relate to the viral pandemic context and how the risk of transmission by and to anaesthetists, intensivists, and other healthcare workers can be minimised.

Ogbe, A., et al. (2021). "T cell assays differentiate clinical and subclinical SARS-CoV-2 infections from cross-reactive antiviral responses." Nat Commun **12**(1): 2055.

Identification of protective T cell responses against SARS-CoV-2 requires distinguishing people infected with SARS-CoV-2 from those with cross-reactive immunity to other coronaviruses. Here we show a range of T cell assays that differentially capture immune function to characterise SARS-CoV-2 responses. Strong ex vivo ELISpot and proliferation responses to multiple antigens (including M, NP and ORF3) are found in 168 PCR-confirmed SARS-CoV-2 infected volunteers, but are rare in 119 uninfected volunteers. Highly exposed seronegative healthcare workers with recent COVID-19-compatible illness show T cell response patterns characteristic of infection. By contrast, >90% of convalescent or unexposed people show proliferation and cellular lactate responses to spike subunits S1/S2, indicating pre-existing cross-reactive T cell populations. The detection of T cell responses to SARS-CoV-2 is therefore critically dependent on assay and antigen selection. Memory responses to specific non-spike proteins provide a method to distinguish recent infection from pre-existing immunity in exposed populations.

O'Hearn, K., et al. (2020). "Efficacy and safety of disinfectants for decontamination of N95 and SN95 filtering facepiece respirators: a systematic review." J Hosp Infect **106**(3): 504-521.

BACKGROUND: Decontaminating and reusing filtering facepiece respirators (FFRs) for healthcare workers is a potential solution to address inadequate FFR supply during a global pandemic. AIM: The objective of this review was to synthesize existing data on the effectiveness and safety of using chemical disinfectants to decontaminate N95 FFRs. METHODS: A systematic review was conducted on disinfectants to decontaminate N95 FFRs using Embase, Medline, Global Health, Google Scholar, WHO feed, and MedRxiv. Two reviewers independently determined study eligibility and extracted predefined data fields. Original research reporting on N95 FFR function, decontamination, safety, or FFR fit following decontamination with a disinfectant was included. FINDINGS AND CONCLUSION: A single cycle of vaporized hydrogen peroxide (H(2)O(2)) successfully removes viral pathogens without affecting airflow resistance or fit, and maintains an initial filter penetration of <5%, with little change in FFR appearance. Residual hydrogen peroxide levels following decontamination were within safe limits. More than one decontamination cycle of vaporized H(2)O(2) may be possible but further information is required on how multiple cycles would affect FFR fit in a real-world setting before the upper limit can be established. Although immersion in liquid H(2)O(2) does not appear to adversely affect FFR function, there is no available data on its ability to remove infectious pathogens from FFRs or its impact on FFR fit. Sodium hypochlorite, ethanol, isopropyl alcohol, and ethylene oxide are not recommended due to safety concerns or negative effects on FFR function.

Ojha, S., et al. (2020). "Knowledge of Handling the Personal protective equipment (PPE) by frontline Allied Health professionals in COVID-19 outbreak - A Web-based Survey Study." J Radiol Nurs.

Ever since the COVID-19 has emerged the number of affected individuals has been increasing exponentially. The frontline health workers are in constant risk due to the etiology and mode of transmission of the disease. Thus, the proper use of personal protective equipment (PPE) is very crucial during this pandemic. This study is to assess the knowledge regarding the handling of personal protective equipment among frontline allied health professionals who are directly in contact with COVID-19 patients. A standard questionnaire was prepared in Google forms and circulated within the network of healthcare workers via online platforms. A total of 143 frontline health workers participated in the questionnaire Survey. It was found that only 44.1% of the total participants had previously undergone training regarding the handling of PPE. About 86.0% of participants gave correct response to the safety etiquette questions. For another knowledge-based question about using the N95 respirator in conjunction with a Face shield, 86.7% of participants responded correctly. Only 67.8% of participants gave the right response to the question regarding the sequence of donning and doffing of PPE. The last question was regarding knowledge about the disposal of PPE, where 95.8% of PPE responded correctly. Despite the safety precautions and procedures set forth by the Centers for Disease Control and Prevention (CDC) for handling COVID-19 patients, the healthcare workers are still prone to occupational hazards. The only barrier standing between the healthcare workers and COVID-19 is the personal protective equipment. Thus, in-depth training and education should be imparted upon the healthcare workers in the present pandemic situation.

O'Kelly, E., et al. (2021). "Comparing the fit of N95, KN95, surgical, and cloth face masks and assessing the accuracy of fit checking." PLoS One **16**(1): e0245688.

INTRODUCTION: The COVID-19 pandemic has made well-fitting face masks a critical piece of protective equipment for healthcare workers and civilians. While the importance of wearing face masks has been acknowledged, there remains a lack of understanding about the role of good fit in rendering protective equipment useful. In addition, supply chain constraints have caused some organizations to abandon traditional quantitative or/and qualitative fit testing, and instead, have implemented subjective fit checking. Our study seeks to quantitatively evaluate the level of fit offered by various types of masks, and most importantly, assess the accuracy of implementing fit checks by comparing fit check results to quantitative fit testing results. METHODS: Seven participants first evaluated N95 and KN95 respirators by performing a fit check. Participants then underwent quantitative fit testing wearing five N95 respirators, a KN95 respirator, a surgical mask, and fabric masks. RESULTS: N95 respirators offered higher degrees of protection than the other categories of masks tested; however, it should be noted that most N95 respirators failed to fit the participants adequately. Fit check responses had poor correlation with quantitative fit factor scores. KN95, surgical, and fabric masks achieved low fit factor scores, with little protective difference recorded between respiratory protection options. In addition, small facial differences were observed to have a significant impact on quantitative fit. CONCLUSION: Fit is critical to the level of protection offered by respirators. For an N95 respirator to provide the promised protection, it must fit the participant. Performing a fit check via NHS self-assessment guidelines was an unreliable way of determining fit.

Oksanen, L. A. H., et al. (2021). "Sources of healthcare workers' COVID‑19 infections and related safety guidelines." Int J Occup Med Environ Health.

OBJECTIVES: To evaluate the effectiveness of safety guidelines in the workplace, the authors analyzed the work-related exposure to SARS-CoV-2 and the source of COVID‑19 infections among healthcare workers (HCWs), together with the use of personal protective equipment (PPE). MATERIAL AND METHODS: A cross-sectional prospective study was conducted in tertiary hospitals in the Uusimaa region, Finland, with 1072 volunteers being enrolled in the study from among the HCWs at the Helsinki University Hospital. Overall, 866 (80.8%) HCWs (including 588 nurses, 170 doctors, and 108 laboratory and medical imaging nurses) completed the questionnaire by July 15, 2020, with 52% of the participants taking care of COVID‑19 patients. The participants answered a structured questionnaire regarding their use of PPE, the ability to follow safety guidelines, exposure to COVID‑19, and the source of potential COVID‑19 infections. The participants with COVID‑19 symptoms were tested with the SARS-CoV-2 realtime polymerase chain reaction method. All infected participants were contacted, and their answers were confirmed regarding COVID‑19 exposure. RESULTS: In total, 41 (4.7%) participants tested positive for SARS-CoV-2, with 22 (53.6%) of infections being confirmed or likely occupational, and 12 (29.3%) originating from colleagues. In 14 cases (63.6%), occupational infections occurred while using a surgical mask, and all infections originating from patients occurred while using a surgical mask or no mask at all. No occupational infections were found while using an FFP2/3 respirator and following aerosol precautions. The combined odds ratio for working at an intensive care unit, an emergency department, or a ward was 3.4 (95% CI: 1.2-9.2, p = 0.016). CONCLUSIONS: A high infection rate was found among HCWs despite safety guidelines. Based on these findings, the authors recommend the use of FFP2/3 respirators in all patient contacts with confirmed or suspected COVID‑19, along with the use of universal masking, also in personnel rooms.

Okuhama, A., et al. (2020). "Detection of SARS-CoV-2 in Hemodialysis Effluent of Patient with COVID-19 Pneumonia, Japan." Emerg Infect Dis **26**(11): 2758-2761.

We report detection of severe acute respiratory syndrome coronavirus 2 RNA in hemodialysis effluent from a patient in Japan with coronavirus disease and prolonged inflammation. Healthcare workers should observe strict standard and contact precautions and use appropriate personal protective equipment when handling hemodialysis circuitry from patients with diagnosed coronavirus disease.

Okuhara, T., et al. (2020). "Examining persuasive message type to encourage staying at home during the COVID-19 pandemic and social lockdown: A randomized controlled study in Japan." Patient Educ Couns **103**(12): 2588-2593.

OBJECTIVE: Behavioral change is the only prevention against the COVID-19 pandemic until vaccines become available. This is the first study to examine the most persuasive message type in terms of narrator difference in encouraging people to stay at home during the COVID-19 pandemic and social lockdown. METHODS: Participants (n = 1,980) were randomly assigned to five intervention messages (from a governor, a public health expert, a physician, a patient, and a resident of an outbreak area) and a control message. Intention to stay at home before and after reading messages was assessed. A one-way ANOVA with Tukey's or Games-Howell test was conducted. RESULTS: Compared with other messages, the message from a physician significantly increased participants' intention to stay at home in areas with high numbers of people infected (versus a governor, p = .002; an expert, p = .023; a resident, p = .004). CONCLUSION: The message from a physician-which conveyed the crisis of overwhelmed hospitals and consequent risk of people being unable to receive treatment-increased the intent to stay at home the most. PRACTICE IMPLICATIONS: Health professionals and media operatives may be able to encourage people to stay at home by disseminating the physicians' messages through media and the internet.

Oladeru, O. T., et al. (2020). "A call to protect patients, correctional staff and healthcare professionals in jails and prisons during the COVID-19 pandemic." Health Justice **8**(1): 17.

This editorial describes why surge planning in the community must account for potential infection outbreaks in jails and prisons, and why incarcerated people and those in contact with them, including over 450,000 correctional officers and thousands of healthcare staff working in prisons, are at significant risk of COVID-19 exposure. We then explain how our nation's jails and prisons will continue to serve as breeding grounds for devastating COVID-19 outcomes and offer specific guidance and a call to action for the immediate development of correctional healthcare strategies designed to protect the health and safety of patients and correctional and healthcare staff and the communities in which they are situated. Correctional officers and correctional healthcare professionals need the nation's reassurance during this dire time that they will not be abandoned and further stigmatized for responding to the needs of incarcerated people. Our collective health depends on it.

Oladipo, E. K., et al. (2020). "A critical appraisal of COVID-19 as a nosocomial infection: an African perspective." Pan Afr Med J **36**: 310.

The pandemic of Coronavirus disease 19 is not abating since the outbreak began in December 2019. Africa is currently experiencing a surge after an initial low incidence and nosocomial infections could be contributing to this. A dominant factor responsible for this is a weak healthcare system because of many years of neglect due to abysmal budgetary allocation to the sector. The testing capacity for COVID-19 diagnosis in Africa is grossly inadequate coupled with a severe shortage of personal protective equipment and inadequate infectious diseases expert. These factors exposed the frontline health workers and patients to the hazard of nosocomial infection with the attendants´ morbidity and mortality. Deliberate efforts need to be made toward reducing nosocomial COVID-19 infection.

Olmos, C., et al. (2021). "SARS-CoV-2 infection in asymptomatic healthcare workers at a clinic in Chile." PLoS One **16**(1): e0245913.

Asymptomatic SARS-CoV-2 infection of healthcare workers (HCWs) has been reported as a key player in the nosocomial spreading of COVID-19. Early detection of infected HCWs can prevent spreading of the virus in hospitals among HCWs and patients. We conducted a cross-sectional study to determine the asymptomatic infection of HCWs in a private clinic in the city of Santiago, Chile. Our study was conducted during a period of 5 weeks at the peak of transmission of SARS-CoV-2 in Chile. Nasopharyngeal samples were obtained from 413 HCWs and tested for the presence of SARS-CoV-2 using RT-qPCR. We found that a 3.14% of HCWs were positive for the presence of SARS-CoV-2 (14/413). Out of these, 7/14 were completely asymptomatic and did not develop symptoms within 3 weeks of testing. Sequencing of viral genomes showed the predominance of the GR clade; however, sequence comparison demonstrated numerous genetic differences among them suggesting community infection as the main focus of transmission among HCWs. Our study demonstrates that the protocols applied to protect HCWs and patients have been effective as no infection clusters due to asymptomatic carriers were found in the clinic. Together, these data suggest that infection with SARS-CoV-2 among HCWs of this health center is not nosocomial.

Omura, M., et al. (2020). "Nurses' health beliefs about paper face masks in Japan, Australia and China: a qualitative descriptive study." Int Nurs Rev **67**(3): 341-351.

AIM: To explore the health beliefs of clinical and academic nurses from Japan, Australia and China regarding wearing paper masks to protect themselves and others, and to identify differences in participants' health beliefs regarding masks. BACKGROUND: The correct use of face masks and consensus among health professionals across the globe is essential for containing pandemics, and nurses need to act according to policy to protect themselves, educate the public and preserve resources for frontline health workers. Paper masks are worn by health professionals and the general public to avoid the transmission of respiratory infections, such as COVID-19, but there appear to be differences in health beliefs of nurses within and between countries regarding these. METHODS: This qualitative descriptive study used content analysis with a framework approach. FINDINGS: There were major differences in nurse participants' beliefs between and within countries, including how nurses use paper masks and their understanding of their efficacy. In addition, there were cultural differences in the way that nurses use masks in their daily lives and nursing practice contexts. CONCLUSION: Nurses from different working environments, countries and areas of practice hold a variety of health beliefs about mask wearing at the personal and professional level. IMPLICATIONS FOR NURSING POLICY AND HEALTH POLICY: The COVID-19 pandemic has sparked much discussion about the critical importance of masks for the safety of health professionals, and there has been considerable discussion and disagreement about health policies regarding mask use by the general public. Improper use of masks may have a role in creating mask shortages or transmitting infections. An evidence-based global policy on mask use for respiratory illnesses for health professionals, including nurses, and the general public needs to be adopted and supported by a wide-reaching education campaign.

Onaizah, O., et al. (2021). "Guidelines for Robotic Flexible Endoscopy at the Time of COVID-19." Front Robot AI **8**: 612852.

Flexible endoscopy involves the insertion of a long narrow flexible tube into the body for diagnostic and therapeutic procedures. In the gastrointestinal (GI) tract, flexible endoscopy plays a major role in cancer screening, surveillance, and treatment programs. As a result of gas insufflation during the procedure, both upper and lower GI endoscopy procedures have been classified as aerosol generating by the guidelines issued by the respective societies during the COVID-19 pandemic-although no quantifiable data on aerosol generation currently exists. Due to the risk of COVID-19 transmission to healthcare workers, most societies halted non-emergency and diagnostic procedures during the lockdown. The long-term implications of stoppage in cancer diagnoses and treatment is predicted to lead to a large increase in preventable deaths. Robotics may play a major role in this field by allowing healthcare operators to control the flexible endoscope from a safe distance and pave a path for protecting healthcare workers through minimizing the risk of virus transmission without reducing diagnostic and therapeutic capacities. This review focuses on the needs and challenges associated with the design of robotic flexible endoscopes for use during a pandemic. The authors propose that a few minor changes to existing platforms or considerations for platforms in development could lead to significant benefits for use during infection control scenarios.

Onchonga, D., et al. (2021). "Anxiety and depression due to 2019 SARS-CoV-2 among frontier healthcare workers in Kenya." Heliyon **7**(3): e06351.

BACKGROUND: The novel coronavirus disease continues to spread across the globe, causing anxiety and depression among healthcare workers. OBJECTIVES: The current study aimed to determine the levels of anxiety and depression due to the coronavirus pandemic among healthcare workers in Kenya. METHODS: A total sample of 476 respondents participated. The 7-item Generalized Anxiety Disorder Scale (GAD-7) and Patient-Health Questionnaire (PHQ-9), together with a socio-demographic questionnaire, were applied. Stratified sampling was used. Data was analysed using the Statistical Package Programme for Social Science Version 23.0.0. Kruskal Wallis test and Mann-Whitney U test were employed to establish the differences in levels of anxiety and depression across socio-demographic characteristics. Ordinal logistic regression analysis was used to establish the predictors of levels of anxiety and depression, and associations were considered significant at p < 0.05. RESULTS: A total of 35.1% (n = 167) of the participants had mild anxiety, and 13.4% (n = 64) severe anxiety. Approximately 53.6% (n = 255) had mild depression while 9.2% (n = 44) had severe depression. The univariate analysis illustrated a statistical difference in anxiety levels in gender (p > 0.027), years of work experience (p = 0.005), and the cadre of respondents (p = 0.0028). Gender was statistically significant with the level of depression (p = 0.045). About 62.6% (n = 298) of healthcare workers had been trained, and only 9% (n = 43) were confident in managing COVID-19 cases. A large proportion, 98% (n = 458) had concerns about the availability of personal protective equipment. CONCLUSION: The study findings indicated that the majority of healthcare workers had mild anxiety and depression. Female healthcare workers were more likely to experience severe anxiety and depression. Also, levels of anxiety and depression differed across different cadres of healthcare workers.

Ong, J. J. Y., et al. (2020). "Headaches Associated With Personal Protective Equipment - A Cross-Sectional Study Among Frontline Healthcare Workers During COVID-19." Headache **60**(5): 864-877.

BACKGROUND: Coronavirus disease 2019 (COVID-19) is an emerging infectious disease of pandemic proportions. Healthcare workers in Singapore working in high-risk areas were mandated to wear personal protective equipment (PPE) such as N95 face mask and protective eyewear while attending to patients. OBJECTIVES: We sought to determine the risk factors associated with the development of de novo PPE-associated headaches as well as the perceived impact of these headaches on their personal health and work performance. The impact of COVID-19 on pre-existing headache disorders was also investigated. METHODS: This is a cross-sectional study among healthcare workers at our tertiary institution who were working in high-risk hospital areas during COVID-19. All respondents completed a self-administered questionnaire. RESULTS: A total of 158 healthcare workers participated in the study. Majority [126/158 (77.8%)] were aged 21-35 years. Participants included nurses [102/158 (64.6%)], doctors [51/158 (32.3%)], and paramedical staff [5/158 (3.2%)]. Pre-existing primary headache diagnosis was present in about a third [46/158 (29.1%)] of respondents. Those based at the emergency department had higher average daily duration of combined PPE exposure compared to those working in isolation wards [7.0 (SD 2.2) vs 5.2 (SD 2.4) hours, P < .0001] or medical ICU [7.0 (SD 2.2) vs 2.2 (SD 0.41) hours, P < .0001]. Out of 158 respondents, 128 (81.0%) respondents developed de novo PPE-associated headaches. A pre-existing primary headache diagnosis (OR = 4.20, 95% CI 1.48-15.40; P = .030) and combined PPE usage for >4 hours per day (OR 3.91, 95% CI 1.35-11.31; P = .012) were independently associated with de novo PPE-associated headaches. Since COVID-19 outbreak, 42/46 (91.3%) of respondents with pre-existing headache diagnosis either "agreed" or "strongly agreed" that the increased PPE usage had affected the control of their background headaches, which affected their level of work performance. CONCLUSION: Most healthcare workers develop de novo PPE-associated headaches or exacerbation of their pre-existing headache disorders.

Ong, K. Y., et al. (2020). "Managing asthma in the COVID-19 pandemic and current recommendations from professional bodies: a review." J Asthma: 1-8.

OBJECTIVE: To provide a summary of the management of asthma in the current COVID-19 pandemic by examining and comparing the recommendations from various professional bodies. DATA SOURCES AND STUDY SELECTION: Websites of known respiratory professional bodies were searched for COVID-19 guidance related to asthma. Subject matter experts were also consulted for useful resources. Resources that were targeted at healthcare professionals were included, while those targeting patients and the general public were excluded. RESULTS: There is currently little data to suggest that asthma protects from or increases the risk of COVID-19, nor is there any data to support strong recommendations for or against specific asthma treatments. Physicians should continue to manage asthma according to existing accepted asthma guidelines and recommendations. All prescribed medications, especially inhaled corticosteroids, should be continued, and, where indicated, oral corticosteroids and biologic therapies should still be used. Nebulizers and spirometry should be avoided where possible to reduce the risk of viral transmission. A detailed history should be taken to differentiate asthma exacerbations from COVID-19. CONCLUSION: Understanding similarities and differences among the recommendations of the various professional bodies will aid in medical decision-making in managing asthma in the COVID-19 pandemic. Health professionals should also consider the individual needs, preferences and values of their patients and the unique characteristics of their local healthcare systems.

Ong, S., et al. (2020). "Anesthesia guidelines for COVID-19 patients: a narrative review and appraisal." Korean J Anesthesiol **73**(6): 486-502.

The coronavirus disease 2019 (COVID-19) pandemic has challenged health systems globally and prompted the publication of several guidelines. The experiences of our international colleagues should be utilized to protect patients and healthcare workers. The primary aim of this article is to appraise national guidelines for the perioperative anesthetic management of patients with COVID-19 so that they can be enhanced for the management of any resurgence of the epidemic. PubMed and EMBASE databases were systematically searched for guidelines related to SARS-CoV and SARS-CoV-2. Additionally, the World Federation Society of Anesthesiologists COVID-19 resource webpage was searched for national guidelines; the search was expanded to include countries with a high incidence of SARS-CoV. The guidelines were evaluated using the Appraisal of Guidelines for Research and Evaluation II tool. Guidelines from Australia, Canada, China, India, Italy, South Africa, South Korea, Taiwan, the United Kingdom, and the United States of America were evaluated. All the guidelines focused predominantly on intubation and infection control. The scope and purpose of guidelines from China were the most comprehensive. The UK and South Africa provided the best clarity. Editorial independence, the rigor of development, and applicability scored poorly. Heterogeneity and gaps pertaining to preoperative screening, anesthesia technique, subspecialty anesthesia, and the lack of auditing of guidelines were identified. Evidence supporting the recommendations was weak. Early guidelines for the anesthetic management of COVID-19 patients lacked quality and a robust reporting framework. As new evidence emerges, national guidelines should be updated to enhance rigor, clarity, and applicability.

Oropallo, A., et al. (2021). "Wound care during the COVID-19 pandemic: improving outcomes through the integration of telemedicine." J Wound Care **30**(Sup2): S12-s17.

COVID-19 is highly contagious and its rapid spread burdens the healthcare system. As the number of confirmed cases goes up, the shortage of medical resources has become a challenge. To avoid the collapse of the healthcare system during the fight with COVID-19, all healthcare workers, including wound care practitioners, should adapt to new roles and use any appropriate methods available to slow the spread of the virus. Integrating telemedicine into wound care during the outbreak helps maintain social distancing, preserve personal protective equipment and medical resources, and eliminate unnecessary exposure for both vulnerable patients and high-risk healthcare workers.

Orrù, G., et al. (2021). "Secondary Traumatic Stress and Burnout in Healthcare Workers during COVID-19 Outbreak." Int J Environ Res Public Health **18**(1).

(1) Background: The present study aims to assess the level of professional burnout and secondary traumatic stress (STS), and to identify potential risk or protective factors among health care workers (HCWs) during the coronavirus disease 2019 (COVID-19) outbreak.; (2) Methods: This cross-sectional study, based on an online survey, collected demographic data and mental distress outcomes from 184 HCWs from 1 May 2020, to 15 June 2020, from 45 different countries. The degree of STS, perceived stress and burnout was assessed using the Secondary Traumatic Stress Scale (STSS), the Perceived Stress Scale (PSS) and Maslach Burnout Inventory Human Service Survey (MBI-HSS) respectively. Stepwise multiple regression analysis was performed to identify potential risk and protective factors for STS; (3) Results: 184 HCWs (M = 90; Age mean: 46.45; SD: 11.02) completed the survey. A considerable proportion of HCWs had symptoms of STS (41.3%), emotional exhaustion (56.0%), and depersonalization (48.9%). The prevalence of STS was 47.5% in frontline HCWs while in HCWs working in other units it was 30.3% (p < 0.023); 67.1% for the HCWs exposed to patients' death and 32.9% for those HCWs which were not exposed to the same condition (p < 0.001). In stepwise multiple regression analysis, perceived stress, emotional exhaustion, and exposure to patients' death remained as significant predictors in the final model for STS (adjusted R2 = 0.537, p < 0.001); (4) Conclusions: During the current COVID-19 pandemic, HCWs facing patients' physical pain, psychological suffering, and death are more likely to develop STS.

Ortiz, Z., et al. (2020). "[Concerns and demands regarding COVID-19. Survey of health personnel]." Medicina (B Aires) **80 Suppl 3**: 16-24.

The COVID-19 pandemic affected the organization of health services and had consequences for health teams, according to the pre-existing safety and working conditions. During the first week of April 2020, a cross sectional study was carried out with a qualitative-quantitative approach. The aim was to explore the conditions determining the organizational climate: leadership, communication, institutional resources, cohesion/conflict management, and training; and how these were perceived by health personnel to deal with the pandemic. A total of 5670 healthcare workers participated in an online survey and 50 were interviewed, from all subsectors of the Argentinean health system (public, private and union-health insurance); 72.9% were women, 51.4% were physicians, and the predominant age group was under 40 years. In the qualitative sample (interviews), 52% were men, 62% were physicians, and the average age was 44.8 years. The dimensions of the organizational climate were stratified and five independent predictors of perception of conditions were identified: age, gender, tasks performed, health system subsector, and jurisdiction. The condition most frequently perceived as inadequate were the inaccessibility of institutional resources and the access to personal protective equipment was a major concern. Claims included the need of institutional strategies to support healthcare workers and of a clear and uniform communication. In conclusion, at the time of the study, the health personnel perceived serious deficits in their organizations regarding the conditions necessary to confront COVID-19, with differences among subsectors of the health system.

Ostrc, T., et al. (2020). "Urgent dental care on a national level during the COVID-19 epidemic." Clin Exp Dent Res.

OBJECTIVE: This paper aims to report and discuss the organization and statistics of dental care during the COVID-19 epidemics on the national level in Slovenia, providing helpful information to health policy planners worldwide. MATERIAL AND METHODS: During an eight-week lockdown, Emergency Dental Centers (EDCs) were established and coordinated on the national level to treat patients' urgent dental conditions. Telemedicine was used on the first level of triage to reduce contacts between healthcare workers and patients. Weekly coordination between EDCs was supported by real-time data acquisition on the number of patient visits, prescribed medicine, the number and type of dental procedures, and the usage of personal protective equipment (PPE). RESULTS: In EDCs, 27,468 patients were serviced, on average 235 patients per day/million people. The care was provided by 4798 man days of dental health care workers. Except for the first week, treatment and triage visits showed a slight increase. The number of incisions was nearly constant, while the number of extractions increased. A nearly threefold increase was found for emergency endodontic treatments (EET). The number of antibiotic prescriptions demonstrated an increasing trend. Analgesic prescriptions showed a decreasing trend from the beginning of lockdown. CONCLUSIONS: The reorganization and centralization of dental care proved to be an efficient model in Slovenia for the provision of urgent dental care, and the management of the healthcare workforce and PPE. Data from this study may provide helpful information for planning the needs and corresponding resources for the next waves of epidemics of COVID-19.

Özdemir, L., et al. (2020). "Respirators used by healthcare workers due to the COVID-19 outbreak increase end-tidal carbon dioxide and fractional inspired carbon dioxide pressure." J Clin Anesth **66**: 109901.

• Due to COVID-19, compliance of manufactured respirators with standards seems to be placed in the second plan. • Personal protective equipments (PPE), such as respirators, causes carbon dioxide re-breathing in healthcare workers. • Even when resting, both expiratory and inspiratory carbon dioxide levels increase significantly with the use of PPE. • It should be kept in mind that healthcare workers may experience symptoms related to hypercapnia during their use of PPE.

Pacis, M., et al. (2020). "Prophylactic Dressings for Maintaining Skin Integrity of Healthcare Workers When Using N95 Respirators While Preventing Contamination Due to the Novel Coronavirus: A Quality Improvement Project." J Wound Ostomy Continence Nurs **47**(6): 551-557.

PURPOSE: Extended use of N95 respirator masks is far more prevalent during the coronavirus disease 2019 (COVID-19) pandemic. As WOC nurses, we were tasked with formulating procedures for protecting the facial skin integrity of healthcare workers (HCWs) using personal protective devices when caring for patients with suspected or active COVID-19, while avoiding contamination when the masks are donned or doffed. This quality improvement project describes how we approached this project within the limited time frame available as we cared for patients with established and suspected COVID-19. PARTICIPANTS AND SETTING: This project focused on HCW use of N95 respirator masks and dressings currently available in our facility. The 4 WOC nurses acted as quality improvement project directors and as participants. The setting for our project was our facility's simulation laboratory. APPROACH: We evaluated 6 topical products (an alcohol-free liquid acrylate, thin film dressing, thin hydrocolloid dressing, hydrocolloid blister care cushion, thin foam transfer dressing, and thick foam dressing) applied to skin in contact with 3 N95 respirators; all are available on our facility's formulary and all are in widespread clinical use. After the product was applied to the face and nose, the N95 respirator was donned and evaluated for fit. Participants then wore the devices for 10 hours and doffed the mask using established facility procedures. In order to evaluate for potential contamination including possible aerosolization, we applied a commercially available fluorescent lotion to simulate the presence of infectious particles. Contamination was assessed using an ultraviolet light for all dressings except for the alcohol-free liquid acrylate. We also evaluated cutaneous responses (skin integrity, irritation, comfort) during this period. OUTCOMES: We found that contamination of the simulated pathogen did not occur with removal of any of the protective products. No skin irritation was noted with any of the tested products after a 10-hour wear time underneath the N95 respirator masks, but mild discomfort was experienced with 3 of the dressings (thin film dressing and both hydrocolloid dressings). CONCLUSION: Based on these experiences, we recommend application of an alcohol-free liquid acrylate film to prevent facial skin injury associated with friction from the extended use of an N95 respirator mask. We further recommend performing a fit test and user-performed seal check with the use of any topical dressing and especially those that add cushion. For the duration of the COVID-19 pandemic, we recommend use of protective dressings to maintain skin integrity and protection from coronavirus infection as HCWs continue to provide care to all of patients under their care.

Packwood, R., et al. (2020). "An Unusual Case Report of COVID-19 Presenting with Meningitis Symptoms and Shingles." Clin Pract Cases Emerg Med **4**(3): 316-320.

INTRODUCTION: As severe acute respiratory syndrome coronavirus 2 (SARS-CoV-2) spreads across the globe, physicians face the challenges of a contagious pandemic including which patients to isolate, how to conserve personal protective equipment, and who to test. The current protocol at our hospital is to place anyone with new cough, dyspnea, or fever into airborne and contact precautions and consider them for testing. Unfortunately, the symptomatic presentations of coronavirus disease 2019 (COVID-19) are proving more variable than previously thought. CASE REPORT: Our case of COVID-19 presented with headache and then progressed to a meningitis-like illness with co-existing shingles rash. CONCLUSION: COVID-19 can have a variety of initial presentations that are not the classic respiratory symptoms and fever. These presenting symptoms of COVID-19 can include a meningitis-like illness, as our case report indicates. The wide variety of presentations of COVID-19 may warrant widespread testing to identify cases, protect healthcare workers, and prevent the spread of this pandemic.

Padula, W. V., et al. (2021). "Best-Practices for Preventing Skin Injury Beneath Personal Protective Equipment During the COVID-19 Pandemic: A Position Paper from the National Pressure Injury Advisory Panel (NPIAP)." J Clin Nurs.

COVID-19 has infected millions of patients and impacted healthcare workers worldwide. Personal Protective Equipment (PPE) is a key component of protecting frontline clinicians against infection. The benefits of PPE far outweigh the risks, nonetheless, many clinicians are exhibiting skin injury caused by PPE worn incorrectly. These skin injuries, ranging from lesions to open wounds are concerning because they increase the susceptibility of viral infection and transmission to other individuals. Early into the COVID-19 pandemic (April 2020), the U.S. National Pressure Injury Advisory Panel (NPIAP) developed a series of position statements to improve wear-ability of PPE and protect healthcare professionals and their patients as safe from harm as possible under the circumstances. The NPIAP positions, which were formed by conducting a systematic review of what was known at the time, include: (1) Prepare skin before and after wearing PPE with skin sealants, barrier creams and moisturizers; (2) Frequent PPE offloading to relieve pressure and shear applied to skin; (3) treat visible skin injuries immediately caused by PPE to minimize future infection; (4) non-porous dressings may provide additional skin protection, but lack evidence; (5) health systems should take care to educate clinicians about placement and personal hygiene related to handling PPE. Throughout all of these practices, handwashing remains a top priority to handle PPE. These NPIAP positions provided early guidance to reduce the risk of skin injury caused by PPE based on available research regarding PPE injuries, a cautious application of evidence-based recommendations on prevention of device related pressure injuries in patients and the expert opinion of the NPIAP Board of Directors. Clinicians who adhere to these recommendations reduce the prospects of skin damage and long-term effects (e.g. scarring). These simple steps to minimize the risk of skin injury and reduce the risk of coronavirus infection from PPE can help.

Paffenholz, P., et al. (2020). "Perception of the 2020 SARS-CoV-2 pandemic among medical professionals in Germany: results from a nationwide online survey." Emerg Microbes Infect **9**(1): 1590-1599.

Background: The COVID-19 pandemic represents an unprecedented global challenge and implicates a wide range of burden on medical professionals. Here, we evaluated the perception of the COVID-19 pandemic among medical professionals in Germany. Methods: A total of n = 2827 medical professionals participated in an online survey between 27 March and 11 April. Results: While most participants stated that Germany was well prepared and rated the measures taken by their employer as positive, subgroup analyses revealed decisive differences. The preventive measures were rated significantly worse by nurses compared to doctors (p < 0.001) and by participants from ambulatory healthcare centres compared to participants from maximum-care hospitals (p < 0.001). Importantly, shortage of protective medical equipment was reported more commonly in the ambulatory sector (p < 0.001) and in East German federal states (p = 0.004). Moreover, the majority of health care professionals (72.4%) reported significant restrictions of daily work routine. Finally, over 60% of medical professionals had concerns regarding their own health, which were more pronounced among female participants (p = 0.024). Conclusion: This survey may indicate starting points on how medical professionals could be supported in carrying out their important activities during the ongoing and future healthcare challenges.

Paiano, M., et al. (2020). "Mental health of healthcare professionals in China during the new coronavirus pandemic: an integrative review." Rev Bras Enferm **73**(suppl 2): e20200338.

OBJECTIVE: to identify publishing related to the mental health of health professionals working in the front line of the COVID-19 pandemic. METHODS: an integrative review that included primary articles indexed in the Latin American and Caribbean Literature in Health Sciences, Medical Literature Analysis and Retrieval System Online, Cumulative Index to Nursing and Allied Health Literature, Scopus, Embase, Web of Science, Science Direct databases and US National Library of Medicine databases. The result analysis was performed descriptively, in four analytical categories. RESULTS: The publishing involved aspects related to insufficient personal protective equipment, feelings of fear and stigma, the need for psychological and psychiatric support and the possibility of post-outbreak mental disorders. CONCLUSION: All mentioned aspects have a direct impact on the mental health of professionals, demanding the creation of strategies that minimize the emotional burnout of workers, considering that each country and culture reacts differently to the disease.

Pajević, I., et al. (2020). "Organization of mental healthcare in Bosnia and Herzegovina during coronavirus disease 2019 pandemic." Indian J Psychiatry **62**(Suppl 3): S479-s491.

BACKGROUND: Coronavirus disease 2019 (COVID-19), like any other pandemic, has imposed an unprecedented threat to physical and mental health to all nations, worldwide. There is no enough evidence in the literature in this area. The present study has been done to explore the organization of psychiatric services in Bosnia and Herzegovina (BH) to meet mental health needs of BH citizens during the particular restrictive measures caused by COVID-19 pandemic. MATERIALS: This online survey has been done for BH psychiatric institutions. Data were collected from psychiatric institutions in the mental health network of BH. A total of 38 complete responses have been received. RESULTS: Of 38 study participants, three were the departments of psychiatry in university clinical centers, two were psychiatric hospitals, four were psychiatric wards in general hospitals, 27 were community mental health centers, and two were institutes for alcoholism and drug addiction. During the pandemic, all services functioned on a reduced scale, adhering to measures to protect and self-protect both staff and service users. Protective equipment was provided to staff in some institutions in a timely and complete manner and in some in an untimely and incomplete manner. Consultative psychiatric examinations were mainly performed through telephone and online, where it exists as a standard patient monitoring protocol. The application of long-acting antipsychotics was continuous with adherence to restricted and protective measures. In opiate addiction replacement therapy services, substitution therapy was provided for a longer period to reduce frequent contacts between staff and patients. Individual and group psychotherapy continued in reduced number using online technologies, although this type of service was not administratively regulated. An initiative has been given to regulate and administratively recognize telepsychiatry by health insurance funds in the country. A number of psychological problems associated with restrictive measures and fear of illness have been reported by patients as well as by the professionals in mental healthcare teams. There were no COVID-19-positive patients seeking help from institutions that responded to the questionnaire. In one center, infected people with COVID-19 from abroad sought help through the phone. Only one involuntary hospitalization was reported. The involvement of mental health professionals in the work of crisis headquarters during the design of the COVID-19 pandemic control measures varies from satisfactory to insufficient. Education of staff, patients, and citizens was regular with direct instructions through meetings, press, and electronic media. CONCLUSIONS: During the COVID-19 pandemic in BH, all psychiatric services functioned on a reduced scale, adhering to measures to protect and self-protect staff and service users. All patients who asked for help have been adequately treated in direct inpatient or outpatient mental healthcare or online, despite telepsychiatric services not being recognized in health system in BH. There were neither infected patients nor staff with COVID-19 in the psychiatric institutions who responded in this research. A large-scale, multicenter study needs to be performed to get a broader picture and to guide us for future better service planning and delivery.

Palacios, R., et al. (2020). "Double-Blind, Randomized, Placebo-Controlled Phase III Clinical Trial to Evaluate the Efficacy and Safety of treating Healthcare Professionals with the Adsorbed COVID-19 (Inactivated) Vaccine Manufactured by Sinovac - PROFISCOV: A structured summary of a study protocol for a randomised controlled trial." Trials **21**(1): 853.

OBJECTIVES: To evaluate the efficacy of two doses of the adsorbed vaccine COVID-19 (inactivated) produced by Sinovac in symptomatic individuals, with virological confirmation of COVID-19, two weeks after the completion of the two-dose vaccination regimen, aged 18 years or older who work as health professionals providing care to patients with possible or confirmed COVID-19. To describe the occurrence of adverse reactions associated with the administration of each of two doses of the adsorbed vaccine COVID-19 (inactivated) produced by Sinovac up to one week after vaccination in Adults (18-59 years of age) and Elderly (60 years of age or more). TRIAL DESIGN: This is a Phase III, randomized, multicenter, endpoint driven, double-blind, placebo-controlled clinical trial to assess the efficacy and safety of the adsorbed vaccine COVID-19 (inactivated) produced by Sinovac. The adsorbed vaccine COVID-19 (inactivated) produced by Sinovac (product under investigation) will be compared to placebo. Voluntary participants will be randomized to receive two intramuscular doses of the investigational product or the placebo, in a 1: 1 ratio, stratified by age group (18 to 59 years and 60 years or more) and will be monitored for one year by active surveillance of COVID-19. Two databases will be established according to the age groups: one for adults (18-59 years) and one for the elderly (60 years of age or older). The threshold to consider the vaccine efficacious will be to reach a protection level of at least 50%, as proposed by the World Health Organization and the FDA. Success in this criterion will be defined by sequential monitoring with adjustment of the lower limit of the 95% confidence interval above 30% for the primary efficacy endpoint. PARTICIPANTS: Healthy participants and / or participants with clinically controlled disease, of both genders, 18 years of age or older, working as health professionals performing care in units specialized in direct contact with people with possible or confirmed cases of COVID-19. Participation of pregnant women and those who are breastfeeding, as well as those intending to become pregnant within three months after vaccination will not be allowed. Participants will only be included after signing the voluntary Informed Consent Form and ensuring they undergo screening evaluation and conform to all the inclusion and exclusion criteria. All the clinical sites are located in Brazil. INTERVENTION AND COMPARATOR: Experimental intervention: The vaccine was manufactured by Sinovac Life Sciences (Beijing, China) and contains 3 μg/0.5 mL (equivalent to 600 SU per dose) of inactivated SARS-CoV-2 virus, and aluminium hydroxide as adjuvant. Control comparator: The placebo contains aluminium hydroxide in a 0.5 mL solution The schedule of both, experimental intervention and placebo is two 0.5 mL doses IM (deltoid) with a two week interval. MAIN OUTCOMES: The primary efficacy endpoint is the incidence of symptomatic cases of virologically confirmed COVID-19 two weeks after the second vaccination. The virological diagnosis will be confirmed by detection of SARS-CoV-2 nucleic acid in a clinical sample. The primary safety endpoint is the frequency of solicited and unsolicited local and systemic adverse reactions during the period of one week after vaccination according to age group in adult (18-59 years old) and elder (60 years of age or older) subjects. Adverse reactions are defined as adverse events that have a reasonable causal relationship to vaccination. RANDOMISATION: There will be two randomization lists, one for each age group, based on the investigational products to be administered, i.e., vaccine or placebo at a 1: 1 ratio. Each randomization list will be made to include up to 11,800 (18-59 year-old) adults, and 1,260 elderly (60 y-o and older) participants, the maximum number of participants needed per age group. An electronic central randomization system will be used to designate the investigational product that each participant must receive. BLINDING (MASKING): This trial is designed as a double-blind study to avoid introducing bias in the evaluation of efficacy, safety and immunogenicity. The clinical care team, the professionals responsible for the vaccination and the participants will not know which investigational product will be administered. Only pharmacists or nurses in the study who are responsible for the randomization, separation and blinding of the investigational product will have access to unblinded information. The sponsor's operational team will also remain blind. NUMBERS TO BE RANDOMISED (SAMPLE SIZE): The total number of participants needed to evaluate efficacy, 13,060 participants, satisfies the needed sample size calculated to evaluate safety. Therefore, the total number obtained for efficacy will be the number retained for the study. Up to 13,060 participants are expected to enter the study, with up to 11,800 participants aged 18 to 59 years and 1,260 elderly participants aged 60 and over. Half of the participants of each group will receive the experimental vaccine and half of them will receive the placebo. The recruitment of participants may be modified as recommended by the Data Safety Monitoring Committee at time of the interim unblinded analysis or blind assessment of the COVID-19 attack rate during the study. TRIAL STATUS: Protocol version 2.0 - 24-Aug-2020. Recruitment started on July 21(st), 2020. The recruitment is expected to conclude in October 2020. TRIAL REGISTRATION: ClinicalTrials.gov Identifier: NCT0445659 . Registry on 2 July 2020 FULL PROTOCOL: The full protocol is attached as an additional file, accessible from the Trials website (Additional file 1). In the interest in expediting dissemination of this material, the familiar formatting has been eliminated; this Letter serves as a summary of the key elements of the full protocol.

Palermo, N. E., et al. (2020). "Diabetic Ketoacidosis in COVID-19: Unique Concerns and Considerations." J Clin Endocrinol Metab **105**(8).

CONTEXT: While individuals with diabetes appear to be at similar risk for SARS-CoV-2 infection to those without diabetes, they are more likely to suffer severe consequences, including death. Diabetic ketoacidosis (DKA) is a common and potentially lethal acute complication of diabetes arising from a relative insulin deficiency, which occurs more often in those with type 1 diabetes and in the setting of moderate to severe illness. Early reports indicate that among patients with pre-existing diabetes, DKA may be a common complication of severe COVID-19 and a poor prognostic sign. CASE DESCRIPTION: This clinical perspective explores the key elements of caring for individuals with DKA during the COVID-19 pandemic through 2 cases. Topics addressed include diagnosis, triage, and the fundamental principles of treatment with a focus on the importance of characterizing DKA severity and medical complexity to determine the best approach. CONCLUSIONS: As discussed, some tenets of DKA management may require flexibility in the setting of COVID-19 due to important public health goals, such as preventing transmission to highest risk individuals, reducing healthcare worker exposure to infected patients, and preserving personal protective equipment. Evidence for alternative treatment strategies is explored, with special attention placed on treatment options that may be more relevant during the pandemic, including use of subcutaneous insulin therapy. Finally, DKA is often a preventable condition. We include evidence-based strategies and guidance designed to empower clinicians and patients to avoid this serious complication when possible.

Pan, D. and K. Rajwani (2021). "Implementation of Simulation Training During the COVID-19 Pandemic: A New York Hospital Experience." Simul Healthc **16**(1): 46-51.

Simulation played a critical role in our institution's response to the COVID-19 pandemic in New York City. With the rapid influx of critically ill patients, resource limitations, and presented safety concerns, simulation became a vital tool that provided solutions to the many challenges we faced. In this article, we describe how simulation training was deployed at our institution throughout the course of the pandemic, which included the period of our medical surge. Simulation helped refine protocols, facilitate practice changes, uncover safety gaps, and train redeployed healthcare workers in unfamiliar roles. We also discuss the obstacles we encountered with implementing simulations during the pandemic, the measures we took to adapt to our limitations, and the simulation strategies and end products that were derived from these adaptations.

Pan, L., et al. (2021). "How to optimize the Radiology protocol during the global COVID-19 epidemic: Keypoints from Sichuan Provincial People's Hospital." Clin Imaging **69**: 324-327.

Currently, the COVID-19 pneumonia epidemic is spreading worldwide. Pulmonary imaging plays an important role. The pulmonary imaging (chest computed tomography and Digital radiography) are indispensable for definitive diagnosis and reexamination. It should be noted that nosocomial infection is not uncommon. Many cases including health workers are infected. This is the experience of our radiology department's protocols during the outbreak, we used this protocol to cope with the COVID-19 in Sichuan Province， besides，there is zero infection for health workers during the whole epidemic. So, we would like to share our experience to other radiologists to avoid the nosocomial infection as low as possible. We have six key points for updating the protocol in the epidemic period of COVID-19: 1. Triage system: three-level triage, 2. Maximum Protection Principle, 3. Technical operation principle: careful, fast and stable, 4. Radiologist's Responsibility and Notice, 5. Disinfection measures of machine room, 6. Hospital information construction, network office, accelerate the sharing of imaging, and carry out MDT consultation.

Pan, S. C., et al. (2021). "A cross-sectional seroprevalence for COVID-19 among healthcare workers in a tertially care hospital in Taiwan." J Formos Med Assoc.

BACKGROUND: Healthcare workers (HCWs) are at the frontline during the pandemic of COVID-19 globally. According to the WHO situation report at April 17, there were 22, 073 HCWs contracted the infection. Whether the infection control policy and practice in the hospital setting can protect the HCWs is an important issue. METHODS: We performed a cross-sectional serology study in a tertiary care hospital in Taiwan to explore the sero-prevalence rate among HCWs. The participants are enrolled on a voluntary basis. A structured questionnaire was collected to gather the epidemiology character and risk factors for potential exposure. ELISA tests as Architect SARS-CoV-2 IgG (Abbott) and Elecsys Anti-SARS-CoV-2 assay (Roche) were used to detect antibody responses. If any of the tests was positive, a western blot assay was used for confirmation. RESULTS: There were 194 HCWs participated during July 1 to Aug. 31, 2020. The mean age was 36.3 ± 10.4. More than half of the participants had possible hospital associated risk for COVID-19 exposure (110/192, 57.3%) and 64 had possible community risk for COVID-19 exposure (64/194, 33.0%). There was only one participant had positive test by Architect IgG test and confirmed to be negative for seasonal coronavirus and SARS-CoV-2 antibody. (Mikrogen Diagnostik, Germany). CONCLUSION: The cross-sectional serology study in a tertiary care hospital in Taiwan revealed no HCWs had positive serology response to SARS-CoV-2. We believe that the infection control policy and practice in the hospital and in the community are both important to prevent the disease transmission.

Pan, Y., et al. (2020). "Transmission routes of SARS-CoV-2 and protective measures in dental clinics during the COVID-19 pandemic." Am J Dent **33**(3): 129-134.

RESULTS: The outbreak of coronavirus disease 2019 (COVID-19) has become a primary challenging public health issue for not only China but also the world. On March 11, 2020, the World Health Organization declared that the pandemic of COVID-19 had become a public health emergency of global concern. As of May 12, 2020 COVID-19 has been reported in over 180 countries/regions, with a total of 4,178,156 confirmed cases and over 280,000 deaths. The risk of cross-infection by severe acute respiratory syndrome coronavirus 2 (SARS-CoV-2) might be elevated between dental specialists and patients due to the peculiarity of dental practice. Therefore, detailed and effective infection control measures are imminently needed to prevent nosocomial coronavirus infection. This paper, based on proven effective experience, relevant guidelines, and research, not only presents the fundamental knowledge about the name, etiologic characteristics, epidemiologic characteristics and clinical manifestations of COVID-19, transmission routes and risk points of SARS-CoV-2 in dental clinics but also provides recommended protective measures for dental professionals to interdict the transmission of SARS-CoV-2 in dental clinics. Classification schemes as well as color identification according to the results of the questionnaire survey and temperature measurement in precheck and triages are innovations proposed in this paper. CLINICAL SIGNIFICANCE: This paper, based on proven effective experience, relevant guidelines, and research, not only presents the fundamental knowledge about the name, etiologic characteristics, epidemiologic characteristics and clinical manifestations of COVID-19, transmission routes and risk points of SARS-CoV-2 in dental clinics but also provides recommended protective measures for dental professionals to interdict the transmission of SARS-CoV-2 in dental clinics.

Panayiotou, A., et al. (2020). "Escalation and de-escalation of the radiology response to COVID-19 in a tertiary hospital in South London: The King's College Hospital experience." Br J Radiol **93**(1116): 20201034.

The pandemic of COVID-19 presented an enormous challenge to the medical world in terms of diagnosis, treatment and health-care management as well as service organisation and provision. This novel virus and its spread affected every aspect of modern medical practice, ranging from investigating transmission of this new pathogen, antigen testing of symptomatic patients, imaging, assessing different treatment regimens and the production of a new vaccine. Imaging played a crucial role in the diagnosis of COVID-19-related lung disease, with plain radiography and CT being the main diagnostic modalities, with ultrasound a useful bedside imaging tool. The accurate and early diagnosis of the disease was not the only issue faced by Radiology Departments across the world; prevention of nosocomial infection, creating capacity with elective imaging suspension, management and protection of the workforce being few of the numerous challenges. The purpose of this manuscript is to present the steps that the Radiology Department of a large urban tertiary facility with a local vulnerable population, undertook to adapt the imaging service and structure, both initially escalating and then de-escalating a response to the COVID-19 pandemic. A step-by-step management strategy, effective and sustained staff deployment, imaging management are presented and discussed, to provide a guide for managing a major incident in a radiology department.

Panigrahi, S. K., et al. (2020). "Covid-19 chemoprophylaxis: Ethics of prevention based on anecdotal evidence." Indian J Med Ethics **-**(-): 1-6.

The ongoing pandemic of Covid-19 caused by the SARS-CoV-2 virus has infected more than 6 million all over the world and has caused more than 3.8 lakh fatalities till date(1) Health workers are the frontline responders and are exposed to a plethora of health hazards. Recently, an advisory by the Indian Council of Medical Research for the use of hydroxychloroquine as post-exposure prophylaxis was hailed as an outstanding initiative for the protection of healthcare workers and high risk contacts of patients. But the evidence of effectiveness available is only from in vitro studies and non-randomised control trials of insufficient sample size. Several ongoing large scale clinical trials are focused on the same research questions, the preliminary results of which are still awaited. The present study discusses the ethics of the introduction of therapeutic or preventive interventions based on limited available evidence during the ongoing pandemic of Covid-19.<br><br>.

Pankajakshan, A., et al. (2020). "COVID-19: What have we learnt, and plan for the future." J Pak Med Assoc **70(Suppl 3)**(5): S30-s33.

Coronavirus disease (COVID-19) has spread like wildfire and affected almost every stratum of society. It has brought humans together and taught us to have compassion and collaboration among individuals, governments and organisations. Adequate protection of healthcare workers who are at the frontline must be taken care of. Good hygiene and social distancing go a long way in controlling an epidemic. We must strive towards building more balanced and sustainable models and healthcare systems in the future as we try to successfully emerge from this outbreak.

Panzeri, A., et al. (2021). "Psychological Differences Among Healthcare Workers of a Rehabilitation Institute During the COVID-19 Pandemic: A Two-Step Study." Front Psychol **12**: 636129.

Introduction: Healthcare workers facing the threatening COVID-19 can experience severe difficulties. Despite the need to evaluate both the psychological distress and positive protective resources, brief and reliable assessment tools are lacking. Aim: Study 1 aimed at developing a new assessment tool to measure psychological distress and esteem in healthcare workers during the COVID-19 pandemic. Study 2 aimed to explore and compare the psychological reactions of healthcare workers of the COVID-19 and the non-COVID-19 wards. Methods: In Study 1, psychologists created 25 items based on their clinical experience. A preliminary qualitative evaluation selected the best 15 items for the new tool (CPI-HP) assessing the COVID-19 psychological impact with 2 scales: psychological distress and esteem. The CPI-HP was administered to 110 healthcare professionals to study its psychometric properties and the internal structure with exploratory graph analysis and confirmatory factor analysis. Study 2 compared two groups of healthcare professionals of the COVID-19 and non-COVID-19 departments. Results: In Study 1, the CPI-HP showed satisfying psychometric properties, and the two-factor structure was confirmed with good fit indices. In Study 2, the two groups of healthcare workers showed comparable levels of psychological distress and resilient coping, but the COVID-19 group displayed significantly higher esteem and appreciation of the experience. Discussion: All operators showed high psychological distress during the emergency, but the COVID-19 group reported higher resources, probably due to stronger group cohesion and greater esteem, perceived meaning, and own work value. Conclusion: Assessing the psychological distress and resources of healthcare professionals with specific tools is important. Psychological interventions should promote their psychological health.

Papaefstathiou, E., et al. (2020). "Urology during a Crisis: A Management Algorithm." Urol J **17**(5): 536-539.

COVID-19 pandemic has affected more than a million people worldwide causing a public health crisis. Under these unique circumstances, urologists continue to provide essential healthcare services and support healthcare systems, by participating in the treatment of COVID-19(+) patients and sparing vital equipment and hospital beds. However, delivering medical care during the pandemic requires strategic planning for all surgical and outpatient activities. Proposed measures include rescheduling elective non-oncological surgeries and using a prioritization protocol for oncological surgeries according to hospital capacity. Following that, outpatient clinics could be partly replaced by telemedicine. Additionally, urologists should be trained in screening and treating patients with COVID-19 during their daily routine.  In order to efficiently provide their services, a management protocol for suspected or known COVID-19 urological patients should be implemented. Furthermore, preventive measures for the nosocomial dispersion of the virus and training on self-protective equipment is mandatory for all physicians. Finally, organizational planning for the best utilization of the staff is of utmost importance. Implementation and adaptation of the protocols according to local requirements and guidelines will ameliorate the quality of services and population's health status. Finally, enhancement of current practices will prepare health systems for future crisis.

Papageorgiou, N., et al. (2020). "Ethnicity and COVID-19 cardiovascular complications: a multi-center UK cohort." Am J Cardiovasc Dis **10**(4): 455-462.

BACKGROUND: Recent reports suggest an association between ethnicity and COVID-19 mortality. In the present multi-center study, we aimed to assess the differences underlying this association, and ascertain whether ethnicity also mediates other aspects of COVID-19 like cardiovascular complications. METHODS: Data were collected from a mixed-ethnicity UK cohort of 613 patients admitted and diagnosed COVID-19 positive, across six hospitals in London during the second half of March 2020: 292 were White Caucasian ethnicity, 203 were Asian and 118 were of Afro-Caribbean ethnicity. RESULTS: Caucasian patients were older (P<0.001) and less likely to have hypertension (P=0.038), while Afro-Caribbean patients had higher prevalence of diabetes mellitus (P<0.001). Asian patients were more likely to present with venous thromboembolic disease (adj.OR=4.10, 95% CI 1.49-11.27, P=0.006). On the other hand, Afro-Caribbean had more heart failure (adj.OR=3.64, 95% CI 1.50-8.84, P=0.004) and myocardial injury (adj.OR=2.64, 95% CI 1.10-6.35, P=0.030). Importantly, our adjusted multi-variate Cox regression analysis revealed significantly higher all-cause mortality both for Asian (adj.HR=1.89, 95% CI 1.23-2.91, P=0.004) and Afro-Caribbean ethnicity (adj.HR=2.09, 95% CI 1.30-3.37, P=0.002). CONCLUSIONS: Our data show that COVID-19 may have different presentations and follow different clinical trajectories depending on the ethnicity of the affected subject. Awareness of complications more likely to arise in specific ethnicities will allow a more timely diagnosis and preventive measures for patients at risk. Due to increased mortality, individuals of Afro-Caribbean and Asian ethnicity should be considered as high-risk groups. This may have an impact on health-resource allocation and planning, definition of vulnerable groups, disease management, and the protection of healthcare workers at the frontline.

Papoutsi, E., et al. (2020). "Global burden of COVID-19 pandemic on healthcare workers." ERJ Open Res **6**(2).

Provision of PPE, placing experienced HCWs mostly in organisational positions, increasing testing, minimising exposure by adjusting shift schedules, and providing food and sleep facilities could be some restorative measures to protect HCWs from #COVID19 https://bit.ly/2zcVARW.

Pappa, S., et al. (2021). "From Recession to Depression? Prevalence and Correlates of Depression, Anxiety, Traumatic Stress and Burnout in Healthcare Workers during the COVID-19 Pandemic in Greece: A Multi-Center, Cross-Sectional Study." Int J Environ Res Public Health **18**(5).

The COVID-19 pandemic has the potential to adversely affect the mental health of healthcare workers (HCWs). The public healthcare system in Greece was already facing serious challenges at the outset of the outbreak following years of austerity and an escalating refugee crisis. This multi-center, cross-sectional study aims to assess the levels and associated risk factors of anxiety, depression, traumatic stress and burnout of frontline staff in Greece. A total of 464 self-selected HCWs in six reference hospitals completed a questionnaire comprising sociodemographic and work-related information and validated psychometric scales. The proportion of HCWs with symptoms of moderate/severe depression, anxiety and traumatic stress were 30%, 25% and 33%, respectively. Burnout levels were particularly high with 65% of respondents scoring moderate/severe in emotional exhaustion, 92% severe in depersonalization and 51% low/moderate in personal accomplishment. Predictive factors of adverse psychological outcomes included fear, perceived stress, risk of infection, lack of protective equipment and low social support. The psychological burden associated with COVID-19 in healthcare professionals in Greece is considerable, with more than half experiencing at least mild mental health difficulties. Findings signal the need for immediate organizational and individually tailored interventions to enhance resilience and support wellbeing under pandemic conditions.

Parekh, U. and T. Kanchan (2020). "COVID-19 instigates resurgence of 'needless autopsies' issue in India." J Forensic Leg Med **74**: 102028.

COVID-19 has swamped the entire world and turned into a pandemic. Its high contagiousness compelled authorities to categorize all autopsies as 'high risk' considering the risk of exposure to the healthcare workers. In India, the Criminal Procedure Code authorizes investigating police officer to hold an inquest into suspicious deaths. The present article draw attention towards the 'needless autopsies' in times of COVID-19 and emphasizes on causes and recommendations.

Park, H. C., et al. (2020). "Korean clinical practice guidelines for preventing transmission of coronavirus disease 2019 (COVID-19) in hemodialysis facilities." Kidney Res Clin Pract **39**(2): 145-150.

Coronavirus disease 2019 (COVID-19) is a highly contagious viral disease that is caused by the novel virus Severe Acute Respiratory Syndrome Coronavirus-2 (SARS-CoV-2). COVID-19 has become pandemic since December 2019, when the first case developed in Wuhan, China. Patients receiving hemodialysis are more vulnerable to viral transmission because their immune functions are impaired and they receive treatment within a narrow space. Calling on previous experience with Middle East Respiratory Syndrome during the 2015 outbreak, the joint committee of the Korean Society of Nephrology and the Korean Society of Dialysis Therapy quickly formed a COVID-19 task force team to develop a manual before the first index case was diagnosed in the hemodialysis unit. This special article introduces clinical practice guidelines to prevent secondary transmission of COVID-19 within hemodialysis facilities, which were developed to protect patients, healthcare workers, and caregivers from this highly transmissible virus. The areas of infection control covered by these guidelines include standard precautions, performing dialysis therapy for confirmed or suspected cases, performing cohort isolation for contact patients, and disease monitoring and contact surveillance. We hope these guidelines help healthcare workers and hemodialysis patients around the world cope with the COVID-19 pandemic.

Park, S. H. (2020). "Personal Protective Equipment for Healthcare Workers during the COVID-19 Pandemic." Infect Chemother **52**(2): 165-182.

The coronavirus disease (COVID-19) pandemic has posed a challenge for healthcare systems, and healthcare workers (HCWs) are at high risk of exposure. Protecting HCWs is of paramount importance to maintain continuous patient care and keep healthcare systems functioning. Used alongside administrative and engineering control measures, personal protective equipment (PPE) is the last line of defense and the core component of protection. Current data suggest that severe acute respiratory syndrome coronavirus 2 (SARS-CoV-2) is mainly transmitted through respiratory droplets and close contact. Airborne transmission may occur during aerosol-generating procedures. However, the modes of transmission still remain uncertain, especially regarding the possibility of airborne transmission when aerosol-generating procedures are not performed. Thus, there are some inconsistencies in the respiratory protective equipment recommended by international and national organizations. In Korea, there have been several modifications to PPE recommendations offering options in choosing PPE for respiratory and body protection, which confuses HCWs; they are often unsure what to wear and when to wear it. The choice of PPE is based on the risk of exposure and possible modes of transmission. The level of protection provided by PPE differs based on standards and test methods. Thus, understanding them is the key in selecting the proper PPE. This article reviews evidence on the mode of SARS-CoV-2 transmission, compares the current PPE recommendations of the World Health Organization with those in Korea, and discusses standard requirements and the proper selection of PPE.

Park, S. R., et al. (2020). "Effect of face mask on skin characteristics changes during the COVID-19 pandemic." Skin Res Technol.

BACKGROUND: Previous studies have demonstrated the possibility of adverse effects of prolonged wearing of personal protective equipment in healthcare workers. However, there are a few studies about the effects on skin characteristics after wearing a mask for non-healthcare workers. In this study, we evaluated the dermatologic effects of wearing a mask on the skin over time. MATERIALS AND METHOD: Twenty-one healthy men and women participated in the study. All participants wore masks for 6 hours consecutively. Three measurements were taken (a) before wearing the mask, (b) after wearing the mask for 1 hour, and (c) after wearing the mask for 6 hours. Skin temperature, skin redness, sebum secretion, skin hydration, trans-epidermal water loss, and skin elasticity were measured. RESULTS: The skin temperature, redness, hydration, and sebum secretion were changed significantly after 1 and 6 hours of wearing a mask. Skin temperature, redness, and hydration showed significant differences between the mask-wearing area and the non-mask-wearing area. CONCLUSION: Mask-wearing conditions and time can change several skin characteristics. In particular, it is revealed that the perioral area could be most affected.

Parravano, M., et al. (2020). "Protect Healthcare Workers and Patients from COVID-19: The Experience of Two Tertiary Ophthalmology Care Referral Centers in Italy." Ophthalmol Ther **9**(2): 231-234.

Pasquel, F. J. and G. E. Umpierrez (2020). "Individualizing Inpatient Diabetes Management During the Coronavirus Disease 2019 Pandemic." J Diabetes Sci Technol **14**(4): 705-707.

Diabetes is associated with poor clinical outcomes in hospitalized patients with coronavirus disease 2019 (COVID-19). During this pandemic, many hospitals have already become overwhelmed around the world and are rapidly entering crisis mode. While there are global efforts to boost personal protective equipment (PPE) production, many centers are improvising care strategies, including the implementation of technology to prevent healthcare workers' exposures and reduce the waste of invaluable PPE. Not optimizing glycemic control due to clinical inertia driven by fear or lack of supplies may lead to poor outcomes in patients with diabetes and COVID-19. Individualized care strategies, novel therapeutic regimens, and the use of diabetes technology may reduce these barriers. However, systematic evaluation of these changes in care is necessary to evaluate both patient- and community-centered outcomes.

Passarelli, V. C., et al. (2021). "Asymptomatic COVID-19 in hospital visitors: The underestimated potential of viral shedding." Int J Infect Dis **102**: 412-414.

In a single day, six of 150 (4%) asymptomatic visitors were diagnosed with COVID-19 at a hospital with a universal masking policy. Two inpatients (contacts) subsequently developed symptoms. More rigorous protective measures during visitation periods may need to be included in infection control practices to reduce nosocomial transmissions.

Patel, A. J., et al. (2020). "Minimising risk to thoracic surgical teams in an era of COVID-19: exploring possible preventative measures." Indian J Thorac Cardiovasc Surg **37**(2): 1-5.

The demands of curtailing the impact of the coronavirus disease 2019 (COVID-19) global pandemic have disrupted the world's ability to care for patients with thoracic pathologies. Those who undergo thoracic surgical therapeutic procedures are a high-risk category, likely to have impaired lung function but also high risk for exposing clinical teams to aerosolised viral loads. In light of this global pandemic, thorough pre-procedural planning, adequate personal protective equipment (PPE), experienced personnel and judicious anaesthetic and intra-operative measures will serve to be instrumental in ensuring positive patient outcomes whilst still protecting the safety of healthcare workers.

Patel, B., et al. (2021). "Self-contained system for mitigation of contaminated aerosol sources of SARS-CoV-2&nbsp." Res Sq.

Contaminated aerosols and micro droplets are easily generated by infected hosts through sneezing, coughing, speaking and breathing (1-3) and harm humansâ€™ health and the global economy. While most of the efforts are usually targeted towards protecting individuals from getting infected, (4) eliminating transmissions from infection sources is also important to prevent disease transmission. Supportive therapies for Severe Acute Respiratory Syndrome Coronavirus 2 (SARS CoV-2) pneumonia such as oxygen supplementation, nebulizers and non-invasive mechanical ventilation all carry an increased risk for viral transmission via aerosol to healthcare workers. (5-9) In this work, we study the efficacy of five methods for self-containing aerosols emitted from infected subjects undergoing nebulization therapies with a diverse spectrum on oxygen delivery therapies. The work includes five study cases: Case I: Use of a Full-Face Mask with biofilter in bilevel positive airway pressure device (BPAP) therapy, Case II: Use of surgical mask in High Flow Nasal Cannula (HFNC) therapy, Case III: Use of a modified silicone disposable mask in a HFNC therapy, Case IV: Use of a modified silicone disposable mask with a regular nebulizer and normal breathing, Case V: Use of a mitigation box with biofilter in a Non-Invasive Positive Pressure Ventilator (NIPPV). We demonstrate that while cases I, III and IV showed efficacies of 98-100%; cases II and V , which are the most commonly used, resulted with significantly lower efficacies of 10-24% to mitigate the dispersion of nebulization aerosols. Therefore, implementing cases I, III and IV in health care facilities may help battle the contaminations and infections via aerosol transmission during a pandemic.

Patel, V., et al. (2020). "Cardiac Surgery During the Coronavirus Disease 2019 Pandemic: Perioperative Considerations and Triage Recommendations." J Am Heart Assoc **9**(13): e017042.

The coronavirus disease 2019 pandemic, caused by severe acute respiratory syndrome coronavirus-2, represents the third human affliction attributed to the highly pathogenic coronavirus in the current century. Because of its highly contagious nature and unprecedented global spread, its aggressive clinical presentation, and the lack of effective treatment, severe acute respiratory syndrome coronavirus-2 infection is causing the loss of thousands of lives and imparting unparalleled strain on healthcare systems around the world. In the current report, we discuss perioperative considerations for patients undergoing cardiac surgery and provide clinicians with recommendations to effectively triage and plan these procedures during the coronavirus disease 2019 outbreak. This will help reduce the risk of exposure to patients and healthcare workers and allocate resources appropriately to those in greatest need. We include an algorithm for preoperative testing for coronavirus disease 2019, personal protective equipment recommendations, and a classification system to categorize and prioritize common cardiac surgery procedures.

Patella, V., et al. (2020). "Management of the patient with allergic and immunological disorders in the pandemic COVID-19 era." Clin Mol Allergy **18**: 18.

The pandemic COVID-19 abruptly exploded, taking most health professionals around the world unprepared. Italy, the first European country to be hit violently, was forced to activate the lockdown in mid-February 2020. At the time of the spread, a high number of victims were quickly registered, especially in the regions of Northern Italy which have a high rate of highly-polluting production activities. The need to hospitalize the large number of patients with severe forms of COVID-19 led the National Health System to move a large number of specialists from their disciplines to the emergency hospital departments for the treatment of COVID-19. Furthermore, the lockdown itself has limited the possibility for general practitioners and pediatricians to be able to make outpatient visits and/or home care for patients with chronic diseases. Among them, the patient with atopic diseases, such as asthma, rhinitis and atopic dermatitis, is worthy of particular attention as she/he is immersed in a studded negative scenario with the onset of spring, a factor that should not be underestimated for those who suffer from pollen allergy. The Italian Society of Asthma Allergology and Clinical Immunology, to quickly deal with the lack of references and specialist medical procedures, has produced a series of indications for immunologic patient care that are reported in this paper, and can be used as guidelines by specialists of our discipline.

Paul, L. A., et al. (2021). "Characteristics associated with household transmission of SARS-CoV-2 in Ontario, Canada: A cohort study." Clin Infect Dis.

BACKGROUND: Within-household transmission of SARS-CoV-2 infection has been identified as one of the main sources of spread of COVID-19 after lockdown restrictions and self-isolation guidelines are implemented. Secondary attack rates among household contacts are estimated to be five to ten times higher than among non-household contacts, but it is unclear which individuals are more prone to transmit infection within their households. METHODS: Using address matching, a cohort was assembled of all laboratory-confirmed cases of COVID-19 residing in private households in Ontario, Canada. Descriptive analyses were performed to compare characteristics of cases in households that experienced secondary transmission versus those that did not. Logistic regression models were fit to determine index case characteristics and neighbourhood characteristics associated with transmission. RESULTS: Between January and July, 2020, there were 26,714 cases of COVID-19 residing in 21,226 households. Longer testing delays (≥5 days versus 0 days OR=3.02, 95% CI: 2.53 - 3.60) and male gender (OR=1.28, 95% CI: 1.18 - 1.38) were associated with greater odds of household secondary transmission, while being a healthcare worker (OR=0.56, 95% CI: 0.50 - 0.62) was associated with lower odds of transmission. Neighbourhoods with larger average family size and a higher proportion of households with multiple persons per room were also associated with greater odds of transmission. CONCLUSIONS: It is important for individuals to get tested for SARS-CoV-2 infection as soon as symptoms appear, and to isolate away from household contacts; this is particularly important in neighbourhoods with large family sizes and/or crowded households.

Payne, D. and M. Peache (2021). "Aerosol-generating procedures in home care." Br J Community Nurs **26**(2): 76-80.

COVID-19 has changed the landscape of healthcare in the UK since the first confirmed case in January 2020. Most of the resources have been directed towards reducing transmission in the hospital and clinical environment, but little is known about what community nurses can do to reduce the risk when they nurse people in their own homes? This article looks at what COVID-19 is, how it is spread and how health professionals are at an increased risk from aerosol-generating procedures (AGPs). There is also a discussion on the benefit of mask usage. It defines what AGPs are, which clinical procedures are AGPs, including ones performed in the community setting, and which identified clinical practices that have been mistaken for AGPs. There is also a discussion on the suitability of performing cardiopulmonary resuscitation (CPR). It also describes how to reduce the risk by the use of full personal protective equipment (PPE) and other strategies when AGPs are performed in a patient's home. It ends with general advice about managing the risk of COVID-19 transmission with patients in their homes.

Pazmiño Erazo, E. E., et al. (2021). "Factors Associated With Psychiatric Adverse Effects in Healthcare Personnel During the COVID-19 Pandemic in Ecuador." Rev Colomb Psiquiatr.

INTRODUCTION: Since the emergence of the novel coronavirus disease (COVID-19), the world has faced a pandemic with consequences at all levels. In many countries, the health systems collapsed and healthcare professionals had to be on the front line of this crisis. The adverse effects on the mental health of healthcare professionals have been widely reported. This research focuses on identifying the main factors associated with adverse psychological outcomes. METHODS: Descriptive, cross-sectional study based on surveys, applying the PHQ-9, GAD-7, ISI and EIE-R tests to healthcare professionals from Ecuador during the COVID-19 pandemic. RESULTS: 1,028 participants, distributed in: 557 physicians (54.18%), 349 nurses (33.94%), 29 laboratory workers (2.82%), 27 paramedics (2.62%), 52 psychologists (5.05%) and 14 respiratory therapists (1.36%), from 16 of the 24 provinces of Ecuador. Of these, 27.3% presented symptoms of depression, 39.2% anxiety symptoms, 16.3% insomnia and 43.8% symptoms of PTSD, with the 4 types of symptoms ranging from moderate to severe. The most relevant associated factors were: working in Guayas (the most affected province) (OR = 2.18 for depressive symptoms and OR = 2.59 for PTSD symptoms); being a postgraduate doctor (OR = 1.52 for depressive symptoms and OR = 1.57 for insomnia), perception of not having the proper protective equipment (OR = 1.71 for symptoms of depression and OR = 1.57 for symptoms of anxiety) and being a woman (OR = 1.39 for anxiety). CONCLUSIONS: Healthcare professionals can suffer a significant mental condition that may require psychiatric and psychological intervention. The main associated factors are primarily related to living and working in cities with a higher number of cases and the characteristics of the job, such as being a postgraduate doctor, as well as the perception of security. The main risk factors are primarily related to geographical distribution and job characteristics, such as being a resident physician and self-perception of safety. Further studies are required as the pandemic evolves.

eng

Pecchia, L., et al. (2020). "The Inadequacy of Regulatory Frameworks in Time of Crisis and in Low-Resource Settings: Personal Protective Equipment and COVID-19." Health Technol (Berl): 1-9.

COVID-19 pandemic is plaguing the world and representing the most significant stress test for many national healthcare systems and services, since their foundation. The supply-chain disruption and the unprecedented request for intensive care unit (ICU) beds have created in Europe conditions typical of low-resources settings. This generated a remarkable race to find solutions for the prevention, treatment and management of this disease which is involving a large amount of people. Every day, new Do-It-Yourself (DIY) solutions regarding personal protective equipment and medical devices populate social media feeds. Many companies (e.g., automotive or textile) are converting their traditional production to manufacture the most needed equipment (e.g., respirators, face shields, ventilators etc.). In this chaotic scenario, policy makers, international and national standards bodies, along with the World Health Organization (WHO) and scientific societies are making a joint effort to increase global awareness and knowledge about the importance of respecting the relevant requirements to guarantee appropriate quality and safety for patients and healthcare workers. Nonetheless, ordinary procedures for testing and certification are currently questioned and empowered with fast-track pathways in order to speed-up the deployment of new solutions for COVID-19. This paper shares critical reflections on the current regulatory framework for the certification of personal protective equipment. We hope that these reflections may help readers in navigating the framework of regulations, norms and international standards relevant for key personal protective equipment, sharing a subset of tests that should be deemed essential even in a period of crisis.

Pediconi, F., et al. (2020). "Breast imaging and cancer diagnosis during the COVID-19 pandemic: recommendations from the Italian College of Breast Radiologists by SIRM." Radiol Med **125**(10): 926-930.

The Italian College of Breast Radiologists by the Italian Society of Medical Radiology (SIRM) provides recommendations for breast care provision and procedural prioritization during COVID-19 pandemic, being aware that medical decisions must be currently taken balancing patient's individual and community safety: (1) patients having a scheduled or to-be-scheduled appointment for in-depth diagnostic breast imaging or needle biopsy should confirm the appointment or obtain a new one; (2) patients who have suspicious symptoms of breast cancer (in particular: new onset palpable nodule; skin or nipple retraction; orange peel skin; unilateral secretion from the nipple) should request non-deferrable tests at radiology services; (3) asymptomatic women performing annual mammographic follow-up after breast cancer treatment should preferably schedule the appointment within 1 year and 3 months from the previous check, compatibly with the local organizational conditions; (4) asymptomatic women who have not responded to the invitation for screening mammography after the onset of the pandemic or have been informed of the suspension of the screening activity should schedule the check preferably within 3 months from the date of the not performed check, compatibly with local organizational conditions. The Italian College of Breast Radiologists by SIRM recommends precautions to protect both patients and healthcare workers (radiologists, radiographers, nurses, and reception staff) from infection or disease spread on the occasion of breast imaging procedures, particularly mammography, breast ultrasound, breast magnetic resonance imaging, and breast intervention procedures.

Peine, A., et al. (2021). "[Perception of the COVID-19 Pandemic among Pneumology Professionals in Germany]." Pneumologie **75**(4): 284-292.

The COVID-19 pandemic represents a huge burden on global health systems. Although far-reaching prevention measures such as the increase of intensive care capacities and drastic restrictions of public life have so far been able to avert an overload of the German health care system, the current situation implies an exceptionally high burden on medical professionals. The current study presents the results of an opinion evaluation among 513 pneumology specialists in Germany in the period from March 27(th) to April 11(th), 2020. While the majority of respondents stated that Germany was "well" prepared for the pandemic, this assessment was significantly worse among participants from the outpatient sector compared to the hospital sector (p < 0.001). Furthermore, a lack of medical protective equipment was reported significantly more frequently by respondents from the outpatient sector (p < 0.001). The importance of telemedicine approaches during the COVID-19 pandemic was rated "high" (35.2 %) or "very high" (17.2 %) by most pneumology professionals, with participants from the hospital sector giving a higher rating (p < 0.001). Finally, 45.8 % of the respondents expressed a "negative" influence of the COVID-19 pandemic on their personal mood and 58.3 % expressed "strong" or "very strong" concerns about the health of their fellow human beings. This assessment was significantly stronger among female participants and participants from the nursing sector (p < 0.001). In summary, the current study analyses for the first time the professional and personal impact of the COVID-19 pandemic on pneumology professionals in Germany. The results could help to identify first starting points to better support health professionals during the current and future challenges.

Pellini, F., et al. (2021). "Effects of Lean Thinking and Emerging Technologies on Breast Cancer Patients' Therapeutic Process During COVID-19 Pandemic: A Case-Control Matched Study." Front Surg **8**: 582980.

Introduction: The advent of the COVID-19 pandemic has led to the sudden disruption of routine medical care, and the subsequent reorganization of hospital structures and therapeutic algorithms, aiming at protecting patients and health professionals. This was inevitably bound to affect our Breast Unit, dilating both pre- and post-operative times. The aim of this study was to evaluate the effect on patients' flow of organizational and logistic changes (key interventions) based on lean thinking implemented after the COVID-19 outbreak. Materials and Methods: Clinical and demographic data were retrospectively collected from patients undergoing sentinel lymph node biopsy for breast cancer at the Verona University Hospital from January 2018 to June 2020. Patients enrolled (n = 341) were divided into two groups according to date of admission: before (Group A; n = 294) and after (Group B; n = 47) the implementation of key interventions. Each case in Group B was subsequently matched 1:1 by means of case-control matching with cases from Group A for age, comorbidities, and type of surgery (Subgroup A1; N = 47). Pre-admission time (T0) and length of stay (T1) were compared between the three groups. Results: Median T0 was 312 h, whereas median T1 was 24 h. Patients in Group B had a higher frequency of comorbidities (57.4 vs. 25.2%, p = 0.001) and underwent mastectomy more often than patients in Group A (61.7 vs. 36.7%, p = 0.001). Both median T0 and T1 were higher in group B than in group A (384 vs. 300 h, p = 0.001, 48 vs. 24 h, p = 0.001, respectively). Median T0 and T1 did not significantly differ between Group B and Subgroup A1 (all p > 0.05). Conclusions: Lean thinking and new technologies could prove useful to the optimization of preoperative and postoperative times during the current pandemic, minimizing healthcare personnel and patients' exposure to SARS-CoV-2, and promoting a rational use of limited resources, while complying with oncological principles.

Perella, P., et al. (2021). "Minimising exposure to droplet and aerosolised pathogens: a computational fluid dynamics study." Br J Anaesth **126**(2): 544-549.

BACKGROUND: Hazardous pathogens are spread in either droplets or aerosols produced during aerosol-generating procedures (AGP). Adjuncts minimising exposure of healthcare workers to hazardous pathogens released during AGP may be beneficial. We used state-of-the-art computational fluid dynamics (CFD) modelling to optimise the performance of a custom-designed shield. METHODS: We modelled airflow patterns and trajectories of particles (size range 1-500 μm) emitted during a typical cough using CFD (ANSYS Fluent software, Canonsburg, PA, USA), in the presence and absence of a protective shield enclosing the head of a patient. We modelled the effect of different shield designs, suction tube position, and suction flow rate on particle escape from the shield. RESULTS: Use of the shield prevented escape of 99.1-100% of particles, which were either trapped on the shield walls (16-21%) or extracted via suction (79-82%). At most, 0.9% particles remained floating inside the shield. Suction flow rates (40-160 L min(-1)) had no effect on the final location of particles in a closed system. Particle removal from within the shield was optimal when a suction catheter was placed vertically next to the head of the patient. Addition of multiple openings in the shield reduced the purging performance from 99% at 160 L min(-1) to 67% at 40 L min(-1). CONCLUSION: CFD modelling provides information to guide optimisation of the efficient removal of hazardous pathogens released during AGP from a custom-designed shield. These data are essential to establish before clinical use, pragmatic clinical trials, or both.

Peres, D., et al. (2020). "[Respiratory and Facial Protection: Current Perspectives in the Context of the COVID-19 Pandemic]." Acta Med Port **33**(9): 583-592.

The new SARS-CoV-2 pandemic is an example of an international public health emergency, which is associated with considerable social and economic challenges. At the healthcare level there is the risk that nosocomial outbreaks can be local amplifiers. Adequate infection control practices are of critical importance, which include proper use of personal protective equipment. This equipment must be appropriate to the pathogen transmission route that, in the case of SARS-CoV-2, occurs through droplet and contact routes. The infected individual, when talking, coughing or sneezing, spreads droplets containing the virus, directly contaminating other individuals within one to two meters of distance, as well as the surrounding environment. Airborne transmission may occur when aerosol-generating procedures are performed. Concerning respiratory protection, there is currently weak evidence that the use of respirators provides better protection than surgical masks for SARS-CoV-2 or other viruses (with the exception of aerosol-generating procedures, in which case the use of a respirator is recommended). Eye protection should be guaranteed whenever there is a risk of splashes, droplets or aerosols. The use of different, or higher than necessary, level of personal protective equipment, for the transmission route of the agent, is a form of misuse and can affect its supply for situations when it is clearly indicated. The adequate provision of protective equipment, as well as training of healthcare professionals in its correct use, is highly recommended to ensure safety of care.

Pérez de la Serna, Y. B. J., et al. (2021). "Risk of COVID-19 transmission in esophageal, anorectal manometry, and 24-hour impedance-pH monitoring." Rev Esp Enferm Dig.

Background The impact of the COVID-19 pandemic has led to the interruption of most manometry or impedance-pH monitoring studies. The risk to restarting activities is unknown. Objective Assess the risk of SARS-Cov-2 virus contagion, both to patients and healthcare workers in relation to esophageal and anorectal functional tests during the pandemic without protective measures. Method A questionnaire was designed to find out whether patients and healthcare workers had COVID-19, confirmed either by a test or only compatible symptoms, after functional studies were performed from January until March 2020. Results 263 (92.9%) patients answered the survey. In the two weeks after functional test, four (1.52%) patients had confirmed COVID-19 (adjusted rate 8.34 cases per 1,000 (95% CI -0.06-16.74), OR 0.84 (95% CI: 0.83-0.85), p<0.001), no one after anorectal manometry. Other five had only compatible symptoms, total 9 (3.42%) (adjusted rate 27.50 cases / 1,000 (95% CI: 7.27-47.74), OR 2.84 (95% CI: 2.81-2.87). In the total study period 18.25% had COVID-19 confirmed or with compatible symptoms. The average of days between the procedure and the first day of symptoms was progressively shortened: January 56 days, February 33 days, March 10.5 days. Two out of 10 healthcare workers (20%) were confirmed with COVID-19. Conclusions The risk of COVID-19 contagion when performing functional tests is low and more related to the evolution of the pandemic than to the procedures themselves. The small number of healthcare workers included in the study does not allow a definitive conclusion to be drawn on their risk of contagion.

Pérez-Chacón, M., et al. (2021). "Sensory Processing Sensitivity and Compassion Satisfaction as Risk/Protective Factors from Burnout and Compassion Fatigue in Healthcare and Education Professionals." Int J Environ Res Public Health **18**(2).

The study analyzes sensory processing sensitivity and the compassion satisfaction as risk/protective factors against burnout and compassion fatigue, during the first period of the COVID-19 health emergency. A sample of 1566 Spanish adult healthcare (n = 694) and education (n = 872) professionals was evaluated. An ad hoc questionnaire for sociodemographic data, and the highly sensitive person scale (HSPS), Maslach burnout inventory (MBI) and professional quality of life scale (ProQOL-vIV) were administered. Burnout and compassion fatigue were observed in the healthcare and education professionals, where personal realization and depersonalization were higher in healthcare and compassion fatigue in education. The protective role of compassion satisfaction was confirmed, as was sensory processing sensitivity as a risk factor, except for its low sensory threshold dimension, which positively influenced personal realization. The findings of this study demonstrate the presence of burnout and compassion fatigue in healthcare and education professionals, displaying compassion fatigue as an emerging psychosocial risk in education, which was made more severe under the conditions of study, which is at the beginning of the COVID-19 pandemic. The importance of incorporating adequate management strategies for high sensitivity, empathy and compassion satisfaction in prevention programs is emphasized.

Pérez-García, F., et al. (2020). "Severe acute respiratory coronavirus virus 2 (SARS-CoV-2) infection among hospital workers in a severely affected institution in Madrid, Spain: A surveillance cross-sectional study." Infect Control Hosp Epidemiol: 1-7.

OBJECTIVE: To analyze the impact of the coronavirus disease 2019 (COVID-19) pandemic in workers of a hospital located in one of the most affected areas in Spain. DESIGN, SETTINGS, AND PATIENTS: Cross-sectional study performed between March and May 2020 over all workers of a secondary hospital in Madrid, Spain. METHODS: We employed polymerase chain reaction (PCR, for symptomatic individuals) and serology (for both PCR-negative symptomatic workers and asymptomatic workers) as diagnostic tests for severe acute respiratory coronavirus virus 2 (SARS-CoV-2). We analyzed the prevalence of the virus in healthcare workers (HCWs) and nonhealthcare workers (nHCWs). We also collected information about the use of personal protective equipment (PPEs) and possible contacts prior to infection. RESULTS: In total, 2,963 workers were included: 1,092 were symptomatic, and of these, 539 were positive by PCR (49.4% of symptomatic workers). From the remaining symptomatic workers, 197 (35.6%) were positive by serology. Regarding asymptomatic workers, 345 were positive by serology (31.9% of infected workers). In total, 1,081 (36.5%) presented a positive diagnostic test for SARS-CoV-2. Infection rates were different between HCWs (37.4%) and nHCWs (29.8%) (P = .006). In the multivariate logistic regression analysis, the use of PPE (protective: OR, 0.56; 95% CI, 0.44-0.72; P < .001) and previous contact with COVID-19 patients (risk factor: OR, 1.69; 95% CI, 1.28-2.24; P < .001) were independent factors that were associated with SAS-CoV-2 infection. CONCLUSIONS: Overall, >36% of our workers became infected with SARS-CoV-2, and the rate of asymptomatic infections accounted for almost 32% of all SARS-CoV-2 infections. We detected differences in the rates of infection between HCWs and nHCWs. The use of PPE and previous contact with COVID-19 patients were associated with SARS-CoV-2 infection.

Permana, H. and N. N. M. Soetedjo (2020). "Is subcutaneous insulin administration adequate for the management of hyperglycemic crisis in COVID-19?" Diabetes Metab Syndr **14**(5): 947-948.

There is a desperate need to explore different insulin administration strategies, particularly in coronavirus disease 2019 (COVID-19) patients with hyperglycemic crisis. Noteworthily, diabetes mellitus (DM) and poorly controlled blood glucose increase the risk of mortality and severity of COVID-19. Intravenous (IV) insulin administration with hourly monitoring of blood glucose is the ideal approach in managing patients with hyperglycemic crisis, but it is not judicious to be applied in developing countries where shortage of personal protective equipment (PPE) is a major issue. Furthermore, increasing the probability of "already greater risks" for doctors or other healthcare workers contracting COVID-19 seems inappropriate. Thus, an alternative administration strategy and more moderate glucose monitoring to reduce the contact exposure of healthcare workers with COVID-19 patients, by ensuring appropriate blood glucose levels, needs to be performed in this critical pandemic era. Subcutaneous (SC) rapid-acting insulin analog administration could presumably be a solution to this contentious issue.

Persaud, E. and A. Mitchell (2021). "Needlestick Injuries Among Healthcare Workers Administering COVID-19 Vaccinations in the United States." New Solut **31**(1): 16-19.

As mass COVID-19 vaccination programs roll out across the country, we are potentially faced with compromising workers' health for the sake of the broader public health, as it relates to occupational exposure to contaminated needles and syringes. We have the opportunity to provide recommendations that advance protection of workers through the industrial hygiene hierarchy of controls, especially in light of the twentieth anniversary of the Needlestick Safety and Prevention Act. Specifically, greater focus on institutional controls that can dictate the safety culture and climate of institutions that roll out COVID-19 vaccination programs, while maintaining careful focus on preventing sharps injuries and blood exposure. In addition, we provide suggestions for the role that engineering controls, such as devices with sharps injury prevention features play in protecting workers from exposure to bloodborne pathogens, as well as the importance of ongoing injury incident surveillance.

Petrosino, F., et al. (2021). "Transmission of SARS-Cov-2 and other enveloped viruses to the environment through protective gear: a brief review." EuroMediterr J Environ Integr **6**(2): 48.

Over the past two decades, several deadly viral epidemics have emerged, which have placed humanity in danger. Previous investigations have suggested that viral diseases can spread through contaminants or contaminated surfaces. The transmission of viruses via polluted surfaces relies upon their capacity to maintain their infectivity while they are in the environment. Here, a range of materials that are widely used to manufacture personal protective equipment (PPE) are summarized, as these offer effective disinfection solutions and are the environmental variables that influence virus survival. Infection modes and prevention as well as disinfection and PPE disposal strategies are discussed. A coronavirus-like enveloped virus can live in the environment after being discharged from a host organism until it infects another healthy individual. Transmission of enveloped viruses such as SARS-CoV-2 can occur even without direct contact, although detailed knowledge of airborne routes and other indirect transmission paths is still lacking. Ground transmission of viruses is also possible via wastewater discharges. While enveloped viruses can contaminate potable water and wastewater through human excretions such as feces and droplets, careless PPE disposal can also lead to their transmission into our environment. This paper also highlights the possibility that viruses can be transmitted into the environment from PPE kits used by healthcare and emergency service personnel. A simulation-based approach was developed to understand the transport mechanism for coronavirus and similar enveloped viruses in the environment through porous media, and preliminary results from this model are presented here. Those results indicate that viruses can move through porous soil and eventually contaminate groundwater. This paper therefore underlines the importance of proper PPE disposal by healthcare workers in the Mediterranean region and around the world.

Pfenninger, E. G., et al. (2021). "[Assessment of the risk of infection from SARS-CoV-2 for healthcare workers-findings from practice]." Bundesgesundheitsblatt Gesundheitsforschung Gesundheitsschutz **64**(3): 304-313.

SARS-CoV‑2 has rapidly spread over the world in a pandemic manner causing an infection of predominantly pulmonary manifestation named the COVID-19 disease. Currently, there is neither an effective vaccination nor a specific therapy available. At least two vaccines will be available at the time of publication. In the international press, the risk for medical personnel of SARS-CoV‑2 is rated as high. The Robert Koch Institute, Germany's leading epidemiological authority, regards the risk of infection for the general population to be high. The aim of this article is to discuss and reassess the risk of infection and disease for healthcare workers based on practical experience, national regulations and guidelines, and the number of infections. Both unprotected healthcare workers and healthcare workers equipped with personal protective equipment (PPE) are considered. A corresponding risk matrix is created.The risk of infection with SAR-CoV‑2 for healthcare workers is comparable to the general population and rated as high. Proper use of PPE reduces this risk to medium. PPE consists of liquid-proof gowns, gloves, and filtering face pieces (FFP; FFP 2 as a standard, FFP 3 for aerosol-releasing interventions), a hair cover, and protective goggles. Improper use of PPE, inadequate hygienic measures, and long working shifts increase the risk of infection.

Pfenninger, E. G. and U. X. Kaisers (2020). "[Provisioning of personal protective equipment in hospitals in preparation for a pandemic]." Anaesthesist **69**(12): 909-918.

BACKGROUND: At the beginning of the SARS-CoV‑2 outbreak, personal protective equipment (PPE) was scarce worldwide, leading to the treatment of patients partially without sufficient protection for the medical personnel. In order to be prepared for a new epidemic or pandemic or a "second wave" of COVID-19 outbreak and to meet a renewed deficiency of PPE, considerations were made on how personnel and patients can be better protected by appropriate provisioning. OBJECTIVE: The aim of this study was to develop a tool to predict the necessary amount of PPE to be in stock at a transregional university hospital for a certain period of time during a pandemic. MATERIAL AND METHODS: The consumption of PPE needed for every patient was calculated based on the following data of the Ulm University Hospital: the total consumption of healthcare workers' PPE for April 2020 recorded by the materials management department and the number of patients suffering from COVID-19 and their treatment days. From the amount of PPE necessary for every patient in the intensive care unit (ICU) or in an infection ward, a PPE calculator was created in which the estimated amount of PPE can be calculated with the input variables "patients in intensive care unit", "patients in infection ward" and "treatment days". To validate the PPE calculator, the actual consumption of PPE for May 2020 at the Ulm University hospital was compared to the theoretically calculated demand by the PPE calculator. RESULTS: In April 2020 PPE consisting of 18 different items were kept in stock at Ulm University Hospital and in total 1,995,500 individual items were used. 22 intensive care patients with 257 nursing days and in the infection ward 39 patients with 357 nursing days were treated for COVID-19 disease, leading to a total of 603.2 man-days. A total of 34,550 KN95 masks, 1,558,780 gloves and 1100 goggles or protective visors were used, with a daily average of 49 NK95 masks and 2216 gloves required per ICU patient. In May 2020, 6 ICU patients and 19 patients in infection wards were treated for COVID-19 with 34 nursing days in intensive care and 201 nursing days in infection wards. The use of PPE material was 39% lower than in the previous month but in absolute terms 82% and on average 39% higher than calculated. CONCLUSION: The developed tool allows our hospital to estimate the necessary amount of PPE to be kept in stock for future pandemics. By taking local conditions into account this tool can also be helpful for other hospitals.

Phu, H. T., et al. (2020). "Design and evaluation of a portable negative pressure hood with HEPA filtration to protect health care workers treating patients with transmissible respiratory infections." Am J Infect Control **48**(10): 1237-1243.

BACKGROUND: To mitigate potential exposure of healthcare workers (HCWs) to SARS-CoV-2 via aerosol routes, we have developed a portable hood which not only creates a barrier between HCW and patient, but also utilizes negative pressure with filtration of aerosols by a high-efficiency particulate air filter. MATERIAL AND METHODS: The hood has iris-port openings for access to the patient, and an opening large enough for a patient's head and upper torso. The top of the hood is a high-efficiency particulate air filter connected to a blower to apply negative pressure. We determined the aerosol penetration from outside to inside in laboratory experiments. RESULTS: The penetration of particles from within the hood to the breathing zones of HCWs outside the hood was near 10(-4) (0.01%) in the 200-400 nm size range, and near 10(-3) (0.1%) for smaller particles. Penetration values for particles in the 500 nm-5 μm range were below 10(-2) (1%). Fluorometric analysis of deposited fluorescein particles on the personal protective equipment of an HCW revealed that negative pressure reduces particle deposition both outside and inside the hood. CONCLUSIONS: We find that negative pressure hoods can be effective controls to mitigate aerosol exposure to HCWs, while simultaneously allowing access to patients.

Picard, J., et al. (2020). "Wearing of face masks by healthcare workers during COVID-19 lockdown: what did the public observe through the French media?" J Hosp Infect **106**(3): 617-620.

Wearing a face mask is a major issue in the fight against the spread of the COVID-19 pandemic. The French general population widely started to wear this personal protective equipment usually dedicated to healthcare workers, without being educated to its correct use. People base their behaviour on what they see in the media. However, we observed that mask wearing of healthcare workers published in the media during the pandemic only conformed to good practice guidelines in 70.8% of the photographs collected on some of the main French information websites. Health authorities should communicate widely regarding the good practices for mask wearing in the general population.

Piché-Renaud, P. P., et al. (2021). "Healthcare worker perception of a global outbreak of novel coronavirus (COVID-19) and personal protective equipment: Survey of a pediatric tertiary-care hospital." Infect Control Hosp Epidemiol **42**(3): 261-267.

OBJECTIVE: In this study, we aimed to capture perspectives of healthcare workers (HCWs) on coronavirus disease 2019 (COVID-19) and infection prevention and control (IPAC) measures implemented during the early phase of the COVID-19 pandemic. DESIGN: A cross-sectional survey of HCWs. PARTICIPANTS: HCWs from the Hospital for Sick Children, Toronto, Canada. INTERVENTION: A self-administered survey was distributed to HCWs. We analyzed factors influencing HCW knowledge and self-reported use of personal protective equipment (PPE), concerns about contracting COVID-19 and acceptance of the recommended IPAC precautions for COVID-19. RESULTS: In total, 175 HCWs completed the survey between March 6 and March 10: 35 staff physicians (20%), 24 residents or fellows (14%), 72 nurses (41%), 14 respiratory therapists (8%), 14 administration staff (8%), and 14 other employees (8%). Most of the respondents were from the emergency department (n = 58, 33%) and the intensive care unit (n = 58, 33%). Only 86 respondents (50%) identified the correct donning order; only 60 (35%) identified the correct doffing order; but the majority (n = 113, 70%) indicated the need to wash their hands immediately prior to removal of their mask and eye protection. Also, 91 (54%) respondents felt comfortable with recommendations for droplet and/or contact precautions for routine care of patients with COVID-19. HCW occupation and concerns about contracting COVID-19 outside work were associated with nonacceptance of the recommendations (P = .016 and P = .036 respectively). CONCLUSION: As part of their pandemic response plans, healthcare institutions should have ongoing training for HCWs that focus on appropriate PPE doffing and discussions around modes of transmission of COVID-19.

Piombino, P., et al. (2021). "Facing COVID-19 pandemic: development of custom-made face mask with rapid prototyping system." J Infect Dev Ctries **15**(1): 51-57.

BACKGROUND: COVID-19 is a global pandemic. The virus spreads through respiratory droplets and close contact. Therefore, the availability of personal protective equipment (PPE) for healthcare professionals is essential. 3D printing technology could represent a valid option to ameliorate PPE shortages. METHODOLOGY: Custom-made face mask were designed on the basis of facial scan and then 3D-printed. The whole protocol is executed with freeware software and only required a 3D printer. Six healthcare workers wore the device weekly thus expressing a judgment regarding quality of work, respiratory and skin comfort. RESULTS: The estimated total cost of a single mask is approximately 5 USD. The virtual design of a complete mask lasted 68 minutes on average. Most healthcare workers rated comfort as very good. CONCLUSIONS: Based on the encouraging results obtained, we can confidently confirm that custom-made masks are novel and useful devices that may be used in the fight against COVID-19.

Piombino, P., et al. (2020). "Guidelines and Recommendations for Patient Management, Surgical Activities, and Safety in the Maxillofacial Unit and Head and Neck Department During COVID-19 Pandemic." J Craniofac Surg **31**(6): e630-e633.

The recent pandemic has led to an unprecedented overload of sanitary systems around the world. Despite that a maxillofacial department is not a frontline specialty in the treatment of coronavirus disease 2019 infections, our department has found itself faced with numerous problems in keeping the care system active and efficient while ensuring safety for patients and healthcare professionals. Massive redistribution of health personnel was needed to improve prevention and personal safety measures. The education and training system has been kept active, giving residents a decisive role in managing the state of emergency response. This article outlines new guidelines for infection prevention: from clinical control, treatment processes, clinical management, protection, and disinfection of healthcare professionals.

Pires, C. (2021). "A pre-systematic review on use of masks as protection material for SARS-COV-2 during COVID-19 pandemic." Int J Clin Pract: e14215.

BACKGROUND: Coronavirus disease 2019 (COVID-19) was first reported in Wuhan, China on 31 December 2019. AIMS: To review, analyse and discuss all works about the wearing and development of facemasks as potential protection against SARS-COV-2 during the COVID-19 pandemic. METHODS: PubMed, SciELO, Google Scholar and DOAJ were browsed. KEYWORDS: 'mask and (SARS or Cov)' (1 August 2019 to 12 April 2020). INCLUSION CRITERIA: original research. PRISMA criteria were followed. RESULTS: 21 works were selected. Identified topics: effectiveness of protective equipment/devices (PE) (n=3), universal wearing of facemasks plus other protective measures in the community (n=3), impact of infection control measures on hospitals and epidemiological tracing (n=3), development of new PE (n=2), sanitisation of facemasks (n=4), clinical guidance (n=4) and questionnaires (n=2). DISCUSSION: It seems that the effectiveness of PE, namely facemasks, is not fully known. Universal wearing of facemasks may be simultaneously recommended with other protective measures. Nosocomial and community infections seem to be preventable. New PE, methods of facemask sanitisation and clinical guidance are emerging, but caution is recommended regarding their adoption, revision and monitoring by international boards and institutions. Questionnaires are useful tools for collecting citizens' opinions on implementing public health measures, and thus contribute to mitigating the COVID-19 pandemic. CONCLUSIONS: Universal wearing of facemasks in the community is likely to be recommend during the COVID-19pandemic. Since SARS-COV-2 is highly contagious, a set of measures should be considered. Facemask sanitisation is possible, but standardised procedures are lacking. New PE is emerging and requires detailed regulatory approval. It is advisable to follow popular opinion.

Plasek, J. M., et al. (2020). "Following data as it crosses borders during the COVID-19 pandemic." J Am Med Inform Assoc **27**(7): 1139-1141.

Data change the game in terms of how we respond to pandemics. Global data on disease trajectories and the effectiveness and economic impact of different social distancing measures are essential to facilitate effective local responses to pandemics. COVID-19 data flowing across geographic borders are extremely useful to public health professionals for many purposes such as accelerating the pharmaceutical development pipeline, and for making vital decisions about intensive care unit rooms, where to build temporary hospitals, or where to boost supplies of personal protection equipment, ventilators, or diagnostic tests. Sharing data enables quicker dissemination and validation of pharmaceutical innovations, as well as improved knowledge of what prevention and mitigation measures work. Even if physical borders around the globe are closed, it is crucial that data continues to transparently flow across borders to enable a data economy to thrive, which will promote global public health through global cooperation and solidarity.

Ploumpidis, D. (2020). "Living with covid-19." Psychiatriki **31**(3): 197-200.

From the beginning of 2020, the alarming news from Italy and the first known cases arrived in Greece, along with travelers from the Holy Land. Spain, France and all other countries followed. From the first week of March, restrictive measures began in Greece and then confinement in order to limit the spread of the pandemic and not drown the National Health System by serious cases. The policy of restrictive measures to stop the pandemic was the internationally accepted response1 and the generalized adherence proved effective, despite the shock and the various reactions from the unprecedented, generalized state of restriction, different scale from epidemics of other times.2-4 In other countries, the loose restrictive measures have cost thousands of deaths. The general restrictive measures, however, have serious consequences for people's mental equilibrium, economy and employment, and for this reason they can only be of limited duration.5 The gradual return to normal life rhythms began gradually from May. But the test of the holiday time and the gradual opening of the tourist season, that is, the open communication of moving populations with limited, random checks for the virus and only local restrictions of the gathering of citizens. We are experiencing now the gradual and severe increase in cases, with an uncertain spreading, with asymptomatic and younger in age likely playing a central role in the spread of the virus, while the fear of a potential large increase in serious cases remains. At the time of this writing, the only means of coping is to maintain and locally strengthen the protective measures, while we gradually realize that these measures came to stay for many more months.6,7 A major problem is that the initial small number of cases favored the underestimation of risk by part of the population, and the serious consequences on people's jobs and lives, along with the deregulation of employment and social security relations legislated in recent months, have provided substantial material to conspiracy theories. We carefully monitor the research on the epidemiological behavior of covid-19 and the clinical data, the discussion on the drugs that make the symptoms milder, the effect of the virus on the CNS and the expected vaccine or vaccines. Our role from the beginning concerned the enormous psychological burden of a global health crisis, with serious consequences on people's working and social lives and equally avoiding the deregulation of the management of our patients and of the units that provide it. At the time of confinement, the telephone contact, familiar to all generations, offered valuable information and support, the lines 10306 and 1110, as well as many local or voluntary help-lines. Telepsychiatry was used more widely - being also an initiative of the Hellenic Psychiatric Association - and seems to have entered impetuously in our work, as well as in education. Its central or complementary role and its safe use are issues that are widely discussed at the moment.8 Protection measures against the virus seriously affect the normal functioning of health and mental health services. The solution should be sought in the urgent operational upgrade, the very necessary reinforcement of the staff and its effective protection from the virus and stress from exposure to danger, which has emerged as important factors for the normal operation of the mental health units.9 The use of telepsychiatry has solved a number of operational problems, but in many cases, it remains complementary, as it cannot replace physical presence in more specific care operations. The need not only to normalize, but to improve the follow up of our patients, the need to strengthen the units that provide basic, community mental health services, is shown by the increase in cases of involuntary hospitalization in the psychiatric hospitals of Athens and Thessaloniki during the last two months. A health crisis that affects almost the entire planet and the working and social life of most is a major social and political problem that concerns all of us,10 while mental health professionals are called to offer their scientific tools for people to face a multifaceted threat and in particular to claim and ensure the continuation of the treatment of our patients and the normal operation of our units.

Pokrajac, N., et al. (2020). "Mastery Learning Ensures Correct Personal Protective Equipment Use in Simulated Clinical Encounters of COVID-19." West J Emerg Med **21**(5): 1089-1094.

INTRODUCTION: The correct use of personal protective equipment (PPE) limits transmission of serious communicable diseases to healthcare workers, which is critically important in the era of coronavirus disease 2019 (COVID-19). However, prior studies illustrated that healthcare workers frequently err during application and removal of PPE. The goal of this study was to determine whether a simulation-based, mastery learning intervention with deliberate practice improves correct use of PPE by physicians during a simulated clinical encounter with a COVID-19 patient. METHODS: This was a pretest-posttest study performed in the emergency department at a large, academic tertiary care hospital between March 31-April 8, 2020. A total of 117 subjects participated, including 56 faculty members and 61 resident physicians. Prior to the intervention, all participants received institution-mandated education on PPE use via an online video and supplemental materials. Participants completed a pretest skills assessment using a 21-item checklist of steps to correctly don and doff PPE. Participants were expected to meet a minimum passing score (MPS) of 100%, determined by an expert panel using the Mastery Angoff and Patient Safety standard-setting techniques. Participants that met the MPS on pretest were exempt from the educational intervention. Testing occurred before and after an in-person demonstration of proper donning and doffing techniques and 20 minutes of deliberate practice. The primary outcome was a change in assessment scores of correct PPE use following our educational intervention. Secondary outcomes included differences in performance scores between faculty members and resident physicians, and differences in performance during donning vs doffing sequences. RESULTS: All participants had a mean pretest score of 73.1% (95% confidence interval [CI], 70.9-75.3%). Faculty member and resident pretest scores were similar (75.1% vs 71.3%, p = 0.082). Mean pretest doffing scores were lower than donning scores across all participants (65.8% vs 82.8%, p<0.001). Participant scores increased 26.9% (95% CI of the difference 24.7-29.1%, p<0.001) following our educational intervention resulting in all participants meeting the MPS of 100%. CONCLUSION: A mastery learning intervention with deliberate practice ensured the correct use of PPE by physician subjects in a simulated clinical encounter of a COVID-19 patient. Further study of translational outcomes is needed.

Polkinghorne, A. and J. Branley (2020). "Evidence for decontamination of single-use filtering facepiece respirators." J Hosp Infect **105**(4): 663-669.

Single-use filtering face respirators (FFRs) are critical pieces of personal protective equipment for healthcare workers treating patients with suspected upper respiratory tract pathogens. Experiences during pandemics in the 2000s, as well as the ongoing COVID-19 pandemic caused by the SARS-2-CoV-2, have highlighted concerns over the pressures that sustained respiratory virus pandemics may have on supplies of FFRs globally. Decontamination of FFRs has been posited as one solution to support the re-use of FFRs with a growing body of literature over the last 10+ years beginning to examine both the efficacy of disinfection of contaminated FFRs but also the impact of the decontamination process on the FFR's performance. Physical and chemical methods of decontamination have been tested for treatment of FFRs with ultraviolet germicidal irradiation, sterilization by steam, ethylene oxide and vaporous hydrogen peroxide, demonstrating the most promising results thus far. Many of these methods utilize existing equipment that may already be available in hospitals and could be re-purposed for FFR decontamination. Importantly, some methods may also be replicated on household equipment, broadening the utility of FFR decontamination across a range of healthcare settings. Utilizing techniques to experimentally contaminate FFRs with a range of microorganisms, most decontamination methods appear to reduce the risk of the mask as a source of infection to the wearer and others to negligible levels. The performance of the filter, especially the efficiency of particle penetration following treatment, varied greatly depending on the processing method as well as the model of the filter itself, however. Urgent regulatory body-supported research is required to endorse the routine decontamination of FFRs. In emergency settings, these methods should nevertheless be carefully considered as one strategy to address potential shortfalls in supplies of FFRs for healthcare workers.

Pombo, A. A. M., et al. (2021). "Endoscopy infection control strategy during the COVID-19 pandemic: experience from a tertiary cancer center in Brazil." Clinics (Sao Paulo) **76**: e2280.

OBJECTIVES: Strategic planning for coronavirus disease (COVID-19) care has dominated the agenda of medical services, which have been further restricted by the need for minimizing viral transmission. Risk is particularly relevant in relation to endoscopy procedures. This study aimed to describe a contingency plan for a tertiary academic cancer center, define a strategy to prioritize and postpone examinations, and evaluate the infection rate among healthcare workers (HCWs) in the endoscopy unit of the Cancer Institute of the State of São Paulo (ICESP). METHODS: We created a strategy to balance the risk of acute respiratory syndrome coronavirus 2 (SARS-CoV-2) infection and to mitigate the effects of postponing endoscopic procedures in oncological patients. A retrospective analysis of prospectively collected data on all endoscopies between March and June 2020 compared with those during the same period in 2019 was carried out. All HCWs were interviewed to obtain clinical data and SARS-CoV-2 test results. RESULTS: During the COVID-19 outbreak, there was a reduction of 55% in endoscopy cases in total. Colonoscopy was the most affected modality. The total infection rate among all HCWs was 38%. None of the senior digestive endoscopists had COVID-19. However, all bronchoscopists had been infected. One of three fellows had a serological diagnosis of COVID-19. Two-thirds of all nurses were infected, whereas half of all technicians were infected. CONCLUSIONS: In this pandemic scenario, all endoscopy services must prioritize the procedures that will be performed. It was possible to maintain some endoscopic procedures, including those meant to provide nutritional access, tissue diagnosis, and endoscopic resection. Personal protective equipment (PPE) seems effective in preventing transmission of COVID-19 from patients to digestive endoscopists. These measures can be useful in planning, even for pandemics in the future.

Pouralizadeh, M., et al. (2020). "Anxiety and depression and the related factors in nurses of Guilan University of Medical Sciences hospitals during COVID-19: A web-based cross-sectional study." Int J Afr Nurs Sci **13**: 100233.

BACKGROUND: The outbreak of COVID-19 is an unprecedented challenge to the health systems in Iran. We aimed to assess the psychological impact of this outbreak on nurses in the hospitals of Guilan University of Medical Sciences that is one of the top provinces of incidence of COVID-19. METHODS: In a web-based cross-sectional study, 441 nurses working were selected from the hospitals, from 7 to 12 April 2020. Anxiety and depression were measured using the Generalized Anxiety Disorder-7 and the Patient Health Questionnaire-9, respectively. Simple and multiple logistic regression models were used to identify the factors related to anxiety and depression. RESULTS: The majority were in contact with suspected or confirmed COVID-19 cases (93.4%) and their relatives had been infected with COVID-19 (42%). The mean of anxiety-7 and depression total scores were 8.64 ± 5.60 and 8.48 ± 6.19, respectively. Female (OR = 3.27, 95% CI = 1.01-10.64), working in COVID-19 designated hospital (OR = 1.82, 95% CI = 1.13-2.93), being suspected with COVID-19 infection (OR = 2.01, 95% CI = 1.25-3.26), and insufficient personal protective equipment (OR = 2.61, 95% CI = 1.68-4.06) were associated with anxiety. Depression was significantly associated with female sex (OR = 4.62, %95 CI = 1.24-17.16), having chronic disease (OR = 2.12, 95% CI = 1.20-3.74), being suspected or confirmed with COVID-19 infection (OR = 3.44, 95% CI = 2.11-5.59, and OR = 2.21, 95% CI = 1.04-4.70, respectively), and insufficient personal protective equipment (OR = 1.86, 95% CI = 1.19-2.91). CONCLUSION: The finding declares healthcare workers are at high risk for mental illness. Continuous supervision of the psychological consequences following infectious diseases outbreaks should be a part of the preparedness efforts of health care systems.

Pourani, M. R., et al. (2020). "Prevalence of hand contact urticaria and related risk factors among healthcare workers during the COVID-19 pandemic: A self-reported assessment." Dermatol Ther **33**(6): e14367.

Poyiadji, N., et al. (2021). "Impact of the COVID-19 pandemic on radiology physician work RVUs at a large subspecialized radiology practice." Clin Imaging **73**: 38-42.

PURPOSE: As the COVID-19 pandemic continues, efforts by radiology departments to protect patients and healthcare workers and mitigate disease spread have reduced imaging volumes. This study aims to quantify the pandemic's impact on physician productivity across radiology practice areas as measured by physician work Relative Value Units (wRVUs). MATERIALS AND METHODS: All signed diagnostic and procedural radiology reports were curated from January 1st to July 1st of 2019 and 2020. Physician work RVUs were assigned to each study type based on the Medicare Physician Fee Schedule. Utilizing divisional assignments, radiologist schedules were mapped to each report to generate a sum of wRVUs credited to that division for each week. Differential impact on divisions were calculated relative to a matched timeframe in 2019 and a same length pre-pandemic time period in 2020. RESULTS: All practice areas saw a substantial decrease in wRVUs from the 2020 pre- to intra-pandemic time period with a mean decrease of 51.5% (range 15.4%-76.9%). The largest declines were in Breast imaging, Musculoskeletal, and Neuroradiology, which had decreases of 76.9%, 75.3%, and 67.5%, respectively. The modalities with the greatest percentage decrease were mammography, MRI, and non-PET nuclear medicine. CONCLUSION: All radiology practice areas and modalities experienced a substantial decrease in wRVUs. The greatest decline was in Breast imaging, Neuroradiology, and Musculoskeletal radiology. Understanding the differential impact of the pandemic on practice areas will help radiology departments prepare for the potential depth and duration of the pandemic by better understanding staffing needs and the financial effects.

Prakash, O., et al. (2021). "Assessing seropositivity for IgG antibodies against SARS-CoV-2 in Ahmedabad city of India: a cross-sectional study." BMJ Open **11**(1): e044101.

OBJECTIVES: To study the percentage seropositivity for SARS-CoV-2 to understand the pandemic status and predict the future situations in Ahmedabad. STUDY DESIGN: Cross-sectional study. SETTINGS: Field area of Ahmedabad Municipal Corporation. PARTICIPANTS: More than 30 000 individuals irrespective of their age, sex, acute/past COVID-19 infection participated in the serosurvey which covered all the 75 Urban Primary Health Centres (UPHCs) across 48 wards and 7 zones of the city. Study also involved healthcare workers (HCWs) from COVID-19/non-COVID-19 hospitals. INTERVENTIONS: Seropositivity of IgG antibodies against SARS-CoV-2 was measured as a mark of COVID-19 infection. PRIMARY AND SECONDARY OUTCOMES: Seropositivity was used to calculate cumulative incidence. Correlation of seropositivity with available demographic detail was used for valid and precise assessment of the pandemic situation. RESULTS: From 30 054 samples, the results were available for 29 891 samples and the crude seropositivity is 17.61%. For all the various age groups, the seropositivity calculated between 15% and 20%. The difference in seropositivity for both the sex group is statistically not significant. The seropositivity is significantly lower (13.64%) for HCWs as compared with non-HCWs (18.71%). Seropositivity shows increasing trend with time. Zone with maximum initial cases has high positivity as compared with other zones. UPHCs with recent rise in cases are leading in seropositivity as compared with earlier and widely affected UPHCs. CONCLUSIONS: The results of serosurveillance suggest that the population of Ahmedabad is still largely susceptible. People still need to follow preventive measures to protect themselves till an effective vaccine is available to the people at large. The data indicate the possibility of vanishing immunity over time and need further research to cross verify with scientific evidences.

Prasitsirikul, W., et al. (2020). "Nurse infected with Covid-19 from a provisional dengue patient." Emerg Microbes Infect **9**(1): 1354-1355.

We report a 35-year-old female nurse who possibly received the SARS-CoV-2 virus during the blood sampling of a 35-year-old male patient initially suspected as a dengue infection. The patient had mild thrombocytopenia and positive dengue IgG and IgM whereas the clinicians were not aware of the possibility of false-positive dengue serology revealed in the published case report from Singapore. The nurse put on a pair of gloves but did not wear a mask during the only encounter with this patient. This nosocomial transmission raised a safety concern among healthcare professionals in an area with a relatively low Covid-19 prevalence, especially when the clinical and laboratory characteristics could be confused with other viral infections.

Preti, E., et al. (2020). "The Psychological Impact of Epidemic and Pandemic Outbreaks on Healthcare Workers: Rapid Review of the Evidence." Curr Psychiatry Rep **22**(8): 43.

PURPOSE OF REVIEW: We aim to provide quantitative evidence on the psychological impact of epidemic/pandemic outbreaks (i.e., SARS, MERS, COVID-19, ebola, and influenza A) on healthcare workers (HCWs). RECENT FINDINGS: Forty-four studies are included in this review. Between 11 and 73.4% of HCWs, mainly including physicians, nurses, and auxiliary staff, reported post-traumatic stress symptoms during outbreaks, with symptoms lasting after 1-3 years in 10-40%. Depressive symptoms are reported in 27.5-50.7%, insomnia symptoms in 34-36.1%, and severe anxiety symptoms in 45%. General psychiatric symptoms during outbreaks have a range comprised between 17.3 and 75.3%; high levels of stress related to working are reported in 18.1 to 80.1%. Several individual and work-related features can be considered risk or protective factors, such as personality characteristics, the level of exposure to affected patients, and organizational support. Empirical evidence underlines the need to address the detrimental effects of epidemic/pandemic outbreaks on HCWs' mental health. Recommendations should include the assessment and promotion of coping strategies and resilience, special attention to frontline HCWs, provision of adequate protective supplies, and organization of online support services.

Price, J. R., et al. (2021). "Development and Delivery of a Real-time Hospital-onset COVID-19 Surveillance System Using Network Analysis." Clin Infect Dis **72**(1): 82-89.

BACKGROUND: Understanding nosocomial acquisition, outbreaks, and transmission chains in real time will be fundamental to ensuring infection-prevention measures are effective in controlling coronavirus disease 2019 (COVID-19) in healthcare. We report the design and implementation of a hospital-onset COVID-19 infection (HOCI) surveillance system for an acute healthcare setting to target prevention interventions. METHODS: The study took place in a large teaching hospital group in London, United Kingdom. All patients tested for SARS-CoV-2 between 4 March and 14 April 2020 were included. Utilizing data routinely collected through electronic healthcare systems we developed a novel surveillance system for determining and reporting HOCI incidence and providing real-time network analysis. We provided daily reports on incidence and trends over time to support HOCI investigation and generated geotemporal reports using network analysis to interrogate admission pathways for common epidemiological links to infer transmission chains. By working with stakeholders the reports were co-designed for end users. RESULTS: Real-time surveillance reports revealed changing rates of HOCI throughout the course of the COVID-19 epidemic, key wards fueling probable transmission events, HOCIs overrepresented in particular specialties managing high-risk patients, the importance of integrating analysis of individual prior pathways, and the value of co-design in producing data visualization. Our surveillance system can effectively support national surveillance. CONCLUSIONS: Through early analysis of the novel surveillance system we have provided a description of HOCI rates and trends over time using real-time shifting denominator data. We demonstrate the importance of including the analysis of patient pathways and networks in characterizing risk of transmission and targeting infection-control interventions.

Priede, A., et al. (2021). "Mental health interventions for healthcare workers during the first wave of COVID-19 pandemic in Spain." Rev Psiquiatr Salud Ment.

OBJECTIVE: In order to reduce distress associated with working with COVID-19 patients, several psychological intervention programmes for healthcare workers have been developed in Spain. We aimed to describe the main characteristics and components of these programmes for healthcare workers treating COVID-19 patients in Spanish hospitals. MATERIAL AND METHODS: An online survey was designed to evaluate the main characteristics of psychological intervention programmes for healthcare workers during the first wave of COVID-19 pandemic. RESULTS: Valid responses were received from 36 hospitals. Most of these programmes offered both in-person and online therapy. The most common aim of these interventions was emotional regulation, which was treated by psychoeducational and cognitive-behavioural techniques in individual interventions. Group interventions mainly used psychoeducation and mindfulness. Only half of the teams that offered in-person interventions received training in the proper use of personal protective equipment. CONCLUSIONS: Several hospitals in Spain have developed mental health interventions for healthcare workers during the COVID-19 pandemic, deploying a wide range of therapeutic modalities and techniques. The rapid implementation of these programmes during the pandemic suggests that safety may not have received sufficient attention. The planning and development of interventions for healthcare workers during pandemics merits greater attention by national and regional authorities and institutions.

Publisher: Abstract available from the publisher.

spa

Profili, J., et al. (2021). "Overview of the User Experience for Snorkeling Mask Designs during the COVID-19 Pandemic." Healthcare (Basel) **9**(2).

During the first wave of the COVID-19 pandemic, industries and academic institutes have collaborated to resolve the worldwide medical supply shortage issues. Innovative designs of 3D-printed items were proposed and developed by the maker community as a temporary solution to address the lack of personal protective equipment. An overview of global ongoing and past initiatives during the COVID-19 pandemic along with their challenges on retrofitting full-face snorkeling masks for healthcare applications such as splash-proof face shields, respirator masks and non-invasive ventilation systems are reported in this contribution. This study reviews these global initiatives and challenges. From our analysis, the present situation highlights the need to build solid networks between healthcare institutes and the different rapid prototyping initiatives. A clear feedback system needs to be implemented to facilitate effective collaboration between engineering (maker) and healthcare teams, to optimize the available human resources, and to achieve adequate product developments responding to the needs of healthcare workers.

Prokopenko, M., et al. (2020). "Evaluating Healthcare Worker Competence in the Correct Use of Personal Protective Equipment in the COVID Era: A Quality Improvement Project." Cureus **12**(12): e11954.

INTRODUCTION: Throughout the coronavirus disease 2019 pandemic, personal protective equipment (PPE) guidance has rapidly evolved. Healthcare workers (HCWs) should use PPE correctly to reduce the risk of nosocomial transmission of the coronavirus. We predict a lack of training regarding correct PPE usage amongst HCWs and introduce a low-resource method of training. METHODS: HCWs from various disciplines at a District General Hospital self-rated their ability in utilising PPE using uncontrolled pre- and post-session 16-item questionnaires following a single PPE training session. Participant responses were analysed using Student's t-test for independent (unpaired) samples. RESULTS: Of 64 participants, 37 participants (59%) received any prior PPE training. Six participants (9%) previously received specific severe acute respiratory syndrome coronavirus 2 PPE training. Survey scores were higher in the post-test than the pre-test group. CONCLUSION: This study highlights the lack of formal PPE training amongst HCWs and the need for establishing PPE training as part of the mandatory training of HCWs.

Psichogiou, M., et al. (2020). "Antibodies against SARS-CoV-2 among health care workers in a country with low burden of COVID-19." PLoS One **15**(12): e0243025.

INTRODUCTION: Greece is a country with limited spread of SARS-CoV-2 and cumulative infection attack rate of 0.12% (95% CI 0.06-0.26). Health care workers (HCWs) are a well-recognized risk group for COVID-19. The study aimed to estimate the seroprevalence of antibodies to SARS-CoV-2 in a nosocomial setting and assess potential risk factors. METHODS: HCWs from two hospitals participated in the study. Hospital-1 was a tertiary university affiliated center, involved in the care of COVID-19 patients while hospital-2 was a tertiary specialized cardiac surgery center not involved in the care of these patients. A validated, CE, rapid, IgM/IgG antibody point-of-care test was used. Comparative performance with a reference globally available assay was assessed. RESULTS: 1,495 individuals consented to participate (response rate 77%). The anti-SARS-CoV-2 weighted prevalence was 1.26% (95% CI 0.43, 3.26) overall and 0.53% (95% CI 0.06, 2.78) and 2.70% (95% CI 0.57, 9.19) in hospital-1 and hospital-2, respectively although the study was underpowered to detect statistically significant differences. The overall, hospital-1, and hospital-2 seroprevalence was 10, 4 and 22 times higher than the estimated infection attack rate in general population, respectively. Suboptimal use of personal protective equipment was noted in both hospitals. CONCLUSIONS: These data have implications for the preparedness of a second wave of COVID-19 epidemic, given the low burden of SARS-CoV-2 infection rate, in concordance with national projections.

Puca, E., et al. (2020). "Short epidemiological overview of the current situation on COVID-19 pandemic in Southeast European (SEE) countries." J Infect Dev Ctries **14**(5): 433-437.

We are living in times where a viral disease has brought normal life in much of the world to a halt. The novel coronavirus known as severe acute respiratory syndrome coronavirus 2 (SARS-CoV-2) causing coronavirus disease 2019 (COVID-19) started in December 2019 in Wuhan, China initially and in a short time crossed the European borders. After mitigating the epidemic in China, Italy became one of the most COVID-19 affected countries worldwide. International travelers are important sources of infectious diseases and a possible source of epidemic. Due to its political, geographic, and cultural similarities, Italy is one of the main economic partners of Southeast European (SEE) countries. Our data show that infection in index cases in all 11 SEE countries was travel-related with Italy being a source country for 8/11 countries. After the first case identifications on February 25, the number of cases in SEE countries is continually rising reaching the total number of 15,612 with 565 fatal cases and overall case fatality ratio (CFR) of 3.6 (median 3.8, range 0.8-5.5) by April 10, 2020. At a time when the COVID-19 pandemic is approaching its peak, apart from the problems with treatment of the disease and care for critically ill patients, there are other equally important problems, such as organization of outbreak response, provision of health care, lack of hospital personnel, disruption of personal protective equipment supply chains and health care workers (HCWs) protection. But what is more important is the heroic behavior of the HCWs who are showing their humanity by disregarding their lives.

Pudszuhn, A., et al. (2020). "[Elective tracheostomy in COVID-19 patients: experience with a standardized interdisciplinary approach]." Hno **68**(11): 838-846.

Experience with an interdisciplinary SOP (standard operating procedure) for tracheostomy (TS) in COVID-19 patients, taking into account the general national and international recommendations, is reported. The operative timing of TS due to prolonged invasive ventilation and frustrating weaning attempts was determined on an interdisciplinary level and involved phases of both high and low disease activity. Five TS were performed in patients with an average age of 70.6 years. In addition to the standard COVID-19 protective measures for medical staff to avoid nosocomial COVID-19 infection, SOP-supported communication during the TS leads to periprocedural safety for all involved. COVID-19 infections among medical staff in the departments involved are not yet known.

Puliatti, S., et al. (2020). "COVID-19 and urology: a comprehensive review of the literature." BJU Int **125**(6): E7-e14.

OBJECTIVE: To discuss the impact of COVID-19 on global health, particularly on urological practice and to review some of the available recommendations reported in the literature. MATERIAL AND METHODS: In the current narrative review the PubMed database was searched to identify all the related reports discussing the impact of COVID-19 on the urological field. RESULTS: The COVID-19 pandemic is the latest and biggest global health threat. Medical and surgical priorities have changed dramatically to cope with the current challenge. These changes include postponements of all elective outpatient visits and surgical procedures to save facilities and resources for urgent cases and patients with COVID-19 patients. This review discuss some of the related changes in urology. CONCLUSIONS: Over the coming weeks, healthcare workers including urologists will be facing increasingly difficult challenges, and consequently, they should adopt triage strategy to avoid wasting of medical resources and they should endorse sufficient protection policies to guard against infection when dealing with COVID-19 patients.

Qaseem, A., et al. (2021). "What Is the Antibody Response and Role in Conferring Natural Immunity After SARS-CoV-2 Infection? Rapid, Living Practice Points From the American College of Physicians (Version 1)." Ann Intern Med.

Description: The widespread availability of SARS-CoV-2 antibody tests raises important questions for clinicians, patients, and public health professionals related to the appropriate use and interpretation of these tests. The Scientific Medical Policy Committee (SMPC) of the American College of Physicians developed these rapid, living practice points to summarize the current and best available evidence on the antibody response to SARS-CoV-2 infection, antibody durability after initial infection with SARS-CoV-2, and antibody protection against reinfection with SARS-CoV-2. Methods: The SMPC developed these rapid, living practice points based on a rapid and living systematic evidence review done by the Portland VA Research Foundation and funded by the Agency for Healthcare Research and Quality. Ongoing literature surveillance is planned through December 2021. When new studies are identified and a full update of the evidence review is published, the SMPC will assess the new evidence and any effect on the practice points. Practice Points: Practice Point 1: Do not use SARS-CoV-2 antibody tests for the diagnosis of SARS-CoV-2 infection. Practice Point 2: Antibody tests can be useful for the purpose of estimating community prevalence of SARS-CoV-2 infection. Practice Point 3: Current evidence is uncertain to predict presence, level, or durability of natural immunity conferred by SARS-CoV-2 antibodies against reinfection (after SARS-CoV-2 infection).

Qi, B., et al. (2020). "Protecting Healthcare Professionals during the COVID-19 Pandemic." Biomed Res Int **2020**: 8469560.

OBJECTIVE: To understand how to implement proactive prevention measures among healthcare professionals for preventing potential nosocomial infection. METHODS: 91 healthcare professionals confirmed with the COVID-19 infection were collected, and clinical characteristics and epidemiological data were evaluated. RESULTS: Among the cases, 77 cases (84.6%) were confirmed by the viral nucleic acid test, and the other 14 cases were diagnosed by the clinical investigation. Ground glass opacity and bilateral shadows distribution were observed in 78 cases (85.6%). 56 cases (61.5%) were admitted into Zhongnan Hospital and subjected to antiviral treatment. 73 of a total of 91 cases (80.2%) with a median incubation period of 3 days (IQR, 2 to 6) reported close contact history with patients with the COVID-19 infection. The most common symptoms at the onset of illness were fever (66 cases, 72.5%) and cough (54 cases, 59.3%). The initial positive rate of the CT scan and RT-PCR assay were 84.6% and 48.4%, respectively (P < 0.01). There were 50 cases occurred during the early stage (before Jan 20, 2020), whereas 41 cases occurred at a late stage (after Jan 20, 2020). In the early stage, the most common route of exposure to COVID-19 was via direct care in the absence of any invasive procedure. By contrast, 37 healthcare professionals infected with COVID-19 in the late stage were confirmed to have been exposed via aerosol-generating procedures. CONCLUSION: Identification of the asymptomatic individuals in healthcare settings and prompt response when a suspicious case is considered may render effective control of the nosocomial infection during this pandemic.

Qiu, X., et al. (2021). "Evaluating the contributions of strategies to prevent SARS-CoV-2 transmission in the healthcare setting: a modelling study." BMJ Open **11**(3): e044644.

INTRODUCTION: Since its onset, the COVID-19 pandemic has caused significant morbidity and mortality worldwide, with particularly severe outcomes in healthcare institutions and congregate settings. To mitigate spread, healthcare systems have been cohorting patients to limit contacts between uninfected patients and potentially infected patients or healthcare workers (HCWs). A major challenge in managing the pandemic is the presence of currently asymptomatic/presymptomatic individuals capable of transmitting the virus, who could introduce COVID-19 into uninfected cohorts. The optimal combination of personal protective equipment (PPE), testing and other approaches to prevent these events is unclear, especially in light of ongoing limited resources. METHODS: Using stochastic simulations with a susceptible-exposed-infected-recovered dynamic model, we quantified and compared the impacts of PPE use, patient and HCWs surveillance testing and subcohorting strategies. RESULTS: In the base case without testing or PPE, the healthcare system was rapidly overwhelmed, and became a net contributor to the force of infection. We found that effective use of PPE by both HCWs and patients could prevent this scenario, while random testing of apparently asymptomatic/presymptomatic individuals on a weekly basis was less effective. We also found that even imperfect use of PPE could provide substantial protection by decreasing the force of infection. Importantly, we found that creating smaller patient/HCW-interaction subcohorts can provide additional resilience to outbreak development with limited resources. CONCLUSION: These findings reinforce the importance of ensuring adequate PPE supplies even in the absence of testing and provide support for strict subcohorting regimens to reduce outbreak potential in healthcare institutions.

Que, J., et al. (2020). "Psychological impact of the COVID-19 pandemic on healthcare workers: a cross-sectional study in China." Gen Psychiatr **33**(3): e100259.

BACKGROUND: Healthcare workers fighting against the coronavirus disease 2019 (COVID-19) pandemic are under tremendous pressure, which puts them at an increased risk of developing psychological problems. AIMS: This study aimed to investigate the prevalence of psychological problems in different healthcare workers (ie, physicians, medical residents, nurses, technicians and public health professionals) during the COVID-19 pandemic in China and explore factors that are associated with the onset of psychological problems in this population during this public health crisis. METHODS: A cross-sectional, web-based survey was conducted in February 2020 among healthcare workers during the COVID-19 pandemic. Psychological problems were assessed using the Generalized Anxiety Disorder Scale, Patient Health Questionnaire and Insomnia Severity Index. Logistic regression analyses were used to explore the factors that were associated with psychological problems. RESULTS: The prevalence of symptoms of anxiety, depression, insomnia and the overall psychological problems in healthcare workers during the COVID-19 pandemic in China was 46.04%, 44.37%, 28.75% and 56.59%, respectively. The prevalence of the overall psychological problems in physicians, medical residents, nurses, technicians and public health professionals was 60.35%, 50.82%, 62.02%, 57.54% and 62.40%, respectively. Compared with healthcare workers who did not participate in front-line work, front-line healthcare workers had a higher risk of anxiety, insomnia and overall psychological problems. In addition, attention to negative or neutral information about the pandemic, receiving negative feedback from families and friends who joined front-line work, and unwillingness to join front-line work if given a free choice were three major factors for these psychological problems. CONCLUSIONS: Psychological problems are pervasive among healthcare workers during the COVID-19 pandemic. Receiving negative information and participating in front-line work appear to be important risk factors for psychological problems. The psychological health of different healthcare workers should be protected during the COVID-19 pandemic with timely interventions and proper information feedback.

Quigley, A. L., et al. (2021). "Estimating the burden of COVID-19 on the Australian healthcare workers and health system during the first six months of the pandemic." Int J Nurs Stud **114**: 103811.

INTRODUCTION: There are no publicly available national data on healthcare worker infections in Australia. It has been documented in many countries that healthcare workers (HCW) are at increased occupational risk of COVID-19. We aimed to estimate the burden of COVID-19 on Australia HCW and the health system by obtaining and organizing data on HCW infections, analyzing national HCW cases in regards to occupational risk and analyzing healthcare outbreak. METHODS: We searched government reports and websites and media reports to create a comprehensive line listing of Australian HCW infections and nosocomial outbreaks between January 25(th) and July 8(th), 2020. A line list of HCW related COVID-19 reported cases was created and enhanced by matching data extracted from media reports of healthcare related COVID-19 relevant outbreaks and reports, using matching criteria. Rates of infections and odds ratios (ORs) for HCW were calculated per state, by comparing overall cases to HCW cases. To investigate the sources of infection amongst HCW, transmission data were collated and graphed to show distribution of sources. RESULTS: We identified 36 hospital outbreaks or HCW infection reports between January 25(th) and July 8(th), 2020. According to our estimates, at least 536 HCW in Australia had been infected with COVID-19, comprising 6.03% of all reported infections. The rate of HCW infection was 90/100000 and of community infection 34/100,000. HCW were 2.69 times more likely to contract COVID-19 (95% CI 2.48 to 2.93; P < 0.001). The timing of hospital outbreaks did not always correspond to community peaks. Where data were available, a total of 131 HCW across 21 outbreaks led to 1656 HCW being furloughed for quarantine. In one outbreak, one hospital was closed and 1200 HCW quarantined. CONCLUSION: The study shows that HCW were at nearly 3 times the risk of infection. Of concern, this nearly tripling of risk occurred during a period of low community prevalence suggesting failures at multiple hazard levels including PPE policies within the work environment. Even in a country with relatively good control of COVID-19, HCW are at greater risk of infection than the general community and nosocomial outbreaks can have substantial effects on workforce capacity by the quarantine of numerous HCW during an outbreak. The occurrence of hospital outbreaks even when community incidence was low highlights the high risk setting that hospitals present. Australia faced a resurgence of COVID-19 after the study period, with multiple hospital outbreaks. We recommend formal reporting of HCW infections, testing protocols for nosocomial outbreaks, cohorting of workforce to minimize the impact, and improved PPE guidelines to provide precautionary and optimal protection for HCW.

Quiroga, B., et al. (2021). "Suboptimal personal protective equipment and SARS-CoV-2 infection in Nephrologists: a Spanish national survey." Clin Kidney J **14**(4): 1216-1221.

BACKGROUND: Healthcare workers have been overexposed to severe acute respiratory syndrome coronavirus 2 (SARS-CoV-2) during the current pandemic, but there is little information on the impact of SARS-CoV-2 on nephrologists. The aim of this study was to assess SARS-CoV-2 infections in nephrologists in the first and second pandemic waves, describing risk factors and clinical features. METHODS: This national survey was sent to Spanish nephrologists. Epidemiological data, comorbidities and medications were collected and compared between infected and non-infected nephrologists. Symptoms, prescribed treatments and outcomes are described for infected nephrologists. RESULTS: Three hundred and twenty-seven nephrologists (66% female, age 46 ± 11 years) completed the survey. Of them, 62 (19%) were infected by SARS-CoV-2. Infection was detected by real-time reverse transcription-polymerase chain reaction in the 37 symptomatic patients (62%) and by serological tests in 25 (38%) asymptomatic individuals. Five (8%) of the infected nephrologists were hospitalized. Contrary to the general population, most infections occurred during the first pandemic wave and, specifically, during the first month, when personal protective equipment (PPE) shortages were more severe. Factors associated with infection in univariate analysis were younger age (P = 0.004), work in non-nephrology departments (P = 0.045), higher exposure to coronavirus disease 2019 patients (P < 0.001), lack of appropriate PPE (P < 0.001) and non-O ABO blood group. In an adjusted multivariate model, only lack of appropriate PPE remained predictive of infection [hazard ratio 3.5 (95% confidence interval 1.9-6.8), P < 0.0001]. CONCLUSIONS: SARS-CoV-2 infection was frequent among nephrologists, was frequently diagnosed late and was associated with working conditions.

Radhakrishnan, N., et al. (2021). "Clinical Audit on Symptomatology of Covid-19 Healthcare Workers and Impact on Quality-of-Life (QOL) Due to Continuous Facemask Usage: A Prospective Study." Indian J Otolaryngol Head Neck Surg: 1-8.

Covid-19 due to Sars-Cov-2 infection has reached pandemic proportion. Many healthcare workers are involved in managing both COVID-suspected and confirmed cases. It is mandatory for healthcare workers to have droplet and contact precautions by means of Personal protective equipment (PPE), facemask, face shield or eye protection. Prolonged usage of medical mask results in various adverse effects. This study is an attempt to know the common effects of prolonged face mask in healthcare workers and its resultant quality-of-life (QOL). To study the common effects of prolonged face mask and its impact on QOL of healthcare workers during the COVID 19 crisis. This was a prospective cross-sectional study conducted over 6 months among 2750 healthcare workers. A questionnaire requesting demographic details and most common side effects after prolonged usage of face mask was circulated. We also attached a short form-12 (sf-12) questionnaire to assess its impact on QOL. Out of 2750 personnel, 299 were excluded. Male preponderance was noted. Study was conducted on candidates using 3ply mask or above. Age range was between 18 and 65 years with mean age being 37.61 ± 15.23 in mask users < 5 h per day, 32.2 ± 10.02 in 5-10 h group and 30.19 ± 8.15 in 10 h group. 8.48% (n = 174) had comorbidities. QOL impacted. The complaints with face mask use definitely are troublesome with increase in severity with duration of mask usage. This definitely has a proportional impact on the healthcare workers' QOL.

Radulesco, T., et al. (2020). "COVID-19 and rhinology, from the consultation room to the operating theatre." Eur Ann Otorhinolaryngol Head Neck Dis **137**(4): 309-314.

The purpose of this article is to give rhinologists advice on how to adapt their standard practice during the COVID-19 pandemic. The main goal of these recommendations is to protect healthcare workers against COVID-19 while continuing to provide emergency care so as to prevent loss of chance for patients. We reviewed our recommendations concerning consultations, medical prescriptions and surgical activity in rhinology.

Ragó, Z., et al. (2020). "[Opportunity of periodic monitoring of COVID-19 patients, asymptomatic virus carriers, and postinfectious individuals with IgM/IgG rapid antibody tests among healthcare workers during SARS-CoV-2 pandemic]." Orv Hetil **161**(21): 854-860.

The first Hungarian COVID-19 case was reported on March 4, 2020 by Hungarian officials. Healthcare workers (HCWs) are at the highest risk of contracting the novel coronavirus (SARS-CoV-2), with 12% of total coronavirus cases confirmed among them recently. 80% of the infected persons show only mild, moderate symptoms or stay asymptomatic. The single-stranded viral RNA can be detected by RT-PCR from the respiratory tract, urine, blood and, particulary in children, from stool samples for 30-40 days. We have no valid data of how many HCWs have been infected since the Hungarian SARS-CoV-2 outbreak, due to the lack of the systematic screening. HCWs could play a critical role in transmission and might jeopardize the health of both their patients and their own family members. Both cross-sectional and longitudinal sudies are recommended to evaluate the ratio of the recovered, i.e., "already protected", the ones in the acute phase, i.e., "the infectious", and the virus-naive, i.e., "at risk" workers. Of the available molecular diagnostic options, in addition to RT-PCR it would be advisable to introduce the novel rapid antibody tests which can give quick results, reveal the timeline of the infection, are easy to handle, inexpensive and can be used periodically to monitor HCWs' viral status during the still unkown duration of the SARS-CoV-2 pandemic. Orv Hetil. 2020; 161(21): 854-860.

Rahman, M., et al. (2020). "Mental Distress and Human Rights Violations During COVID-19: A Rapid Review of the Evidence Informing Rights, Mental Health Needs, and Public Policy Around Vulnerable Populations." Front Psychiatry **11**: 603875.

Background: COVID-19 prevention and mitigation efforts were abrupt and challenging for most countries with the protracted lockdown straining socioeconomic activities. Marginalized groups and individuals are particularly vulnerable to adverse effects of the pandemic such as human rights abuses and violations which can lead to psychological distress. In this review, we focus on mental distress and disturbances that have emanated due to human rights restrictions and violations amidst the pandemic. We underscore how mental health is both directly impacted by the force of pandemic and by prevention and mitigation structures put in place to combat the disease. Methods: We conducted a review of relevant studies examining human rights violations in COVID-19 response, with a focus on vulnerable populations, and its association with mental health and psychological well-being. We searched PubMed and Embase databases for studies between December 2019 to July 2020. Three reviewers evaluated the eligibility criteria and extracted data. Results: Twenty-four studies were included in the systematic inquiry reporting on distress due to human rights violations. Unanimously, the studies found vulnerable populations to be at a high risk for mental distress. Limited mobility rights disproportionately harmed psychiatric patients, low-income individuals, and minorities who were at higher risk for self-harm and worsening mental health. Healthcare workers suffered negative mental health consequences due to stigma and lack of personal protective equipment and stigma. Other vulnerable groups such as the elderly, children, and refugees also experienced negative consequences. Conclusions: This review emphasizes the need to uphold human rights and address long term mental health needs of populations that have suffered disproportionately during the pandemic. Countries can embed a proactive psychosocial response to medical management as well as in existing prevention strategies. International human rights guidelines are useful in this direction but an emphasis should be placed on strengthening rights informed psychosocial response with specific strategies to enhance mental health in the long-term. We underscore that various fundamental human rights are interdependent and therefore undermining one leads to a poor impact on the others. We strongly recommend global efforts toward focusing both on minimizing fatalities, protecting human rights, and promoting long term mental well-being.

Rajasekaran, R. B., et al. (2020). "Service delivery during the COVID-19 pandemic: Experience from The Oxford Bone Tumour and Soft Tissue Sarcoma service." J Clin Orthop Trauma **11**(Suppl 4): S419-s422.

The COVID-19 (Coronavirus disease 2019) pandemic has caused an unprecedented strain on healthcare systems across the globe. Apart from being a major hurdle to delivery of basic healthcare services, this may be associated with potential harm for cancer patients. Usually being immunocompromised, cancer patients are at a higher risk of contracting the disease and with hospitals being a potential source of the infection, an urgent need to reorganise the structure of delivery of cancer care is essential. Cancer departments must balance patient care whilst also minimising transmission among patients and healthcare professionals. The Oxford Sarcoma Service was re-structured based on the guidelines issued by the National Health Service (NHS) and the British Orthopaedic Oncology Society (BOOS) to deliver unhindered care to patients. Prioritising patients who needed urgent surgery, weighing the risk-benefit ratio while delivering adjuvant treatments and conducting regular virtual multi-disciplinary team (MDT) meetings combined with personal protection equipment (PPE) usage by all involved healthcare workers were salient features in terms of ensuring the delivery of effective care during the COVID-19 pandemic. Our new model of modus operandi during this global crisis was effective in delivering high standard of care to patients and might serve as a guide to similar units managing bone and soft tissue tumours.

Rajkumar, R. P. (2021). "Harnessing the Neurobiology of Resilience to Protect the Mental Well-Being of Healthcare Workers During the COVID-19 Pandemic." Front Psychol **12**: 621853.

Healthcare workers are at a high risk of psychological morbidity in the face of the COVID-19 pandemic. However, there is significant variability in the impact of this crisis on individual healthcare workers, which can be best explained through an appreciation of the construct of resilience. Broadly speaking, resilience refers to the ability to successfully adapt to stressful or traumatic events, and thus plays a key role in determining mental health outcomes following exposure to such events. A proper understanding of resilience is vital in enabling a shift from a reactive to a proactive approach for protecting and promoting the mental well-being of healthcare workers. Research in the past decade has identified six areas that provide promising leads in understanding the biological basis of individual variations in resilience. These are: (1) the key role played by the monoamines noradrenaline and serotonin, (2) the centrality of the hypothalamic-pituitary-adrenal axis in influencing stress vulnerability and resilience, (3) the intimate links between the immune system and stress sensitivity, (4) the role of epigenetic modulation of gene expression in influencing the stress response, (5) the role played by certain neuropeptides as a natural "brake" mechanism in the face of stress, and (6) the neurobiological mechanisms by which environmental factors, such as exercise, diet, and social support, influence resilience to subsequent life events. Though much of this research is still in its early stages, it has already provided valuable information on which strategies - including dietary changes, lifestyle modification, environmental modification, psychosocial interventions, and even pharmacological treatments - may prove to be useful in fostering resilience in individuals and groups. This paper examines the above evidence more closely, with a specific focus on the challenges faced by healthcare workers during the COVID-19 pandemic, and provides suggestions regarding how it may be translated into real-world interventions, as well as how the more tentative hypotheses advanced in this field may be tested during this critical period.

Ramaraj, P., et al. (2020). "Triaging of respiratory protective equipment on the assumed risk of SARS-CoV-2 aerosol exposure in patient-facing healthcare workers delivering secondary care: a rapid review." BMJ Open **10**(10): e040321.

OBJECTIVES: In patient-facing healthcare workers delivering secondary care, what is the evidence behind UK Government personal protective equipment (PPE) guidance on surgical masks versus respirators for SARS-CoV-2 protection? DESIGN: Two independent reviewers performed a rapid review. Appraisal was performed using Critical Appraisal Skills Programme checklists and Grading of Recommendations, Assessment, Development and Evaluations methodology. Results were synthesised by comparison of findings and appraisals. DATA SOURCES: MEDLINE, Google Scholar, UK Government COVID-19 website and grey literature. ELIGIBILITY CRITERIA: Studies published on any date containing primary data comparing surgical facemasks and respirators specific to SARS-CoV-2, and studies underpinning UK Government PPE guidance, were included. RESULTS: Of 30 identified, only 3 laboratory studies of 14 different respirators and 12 surgical facemasks were found. In all three, respirators were significantly more effective than facemasks when comparing protection factors, reduction factors, filter penetrations, total inspiratory leakages at differing particle sizes, mean inspiratory flows and breathing rates. Tests included live viruses and inert particles on dummies and humans. In the six clinical studies (6502 participants) included the only statistically significant result found continuous use of respirators more effective in clinical respiratory illness compared with targeted use or surgical facemasks. There was no consistent definition of 'exposure' to determine the efficacy of respiratory protective equipment (RPE). It is difficult to define 'safe'. CONCLUSIONS: There is a paucity of evidence on the comparison of facemasks and respirators specific to SARS-CoV-2, and poor-quality evidence in other contexts. The use of surrogates results in extrapolation of non-SARS-CoV-2 specific data to guide UK Government PPE guidance. The appropriateness of this is unknown given the uncertainty over the transmission of SARS-CoV-2.This means that the evidence base for UK Government PPE guidelines is not based on SARS-CoV-2 and requires generalisation from low-quality evidence of other pathogens/particles. There is a paucity of high-quality evidence regarding the efficacy of RPE specific to SARS-CoV-2. UK Government PPE guidelines are underpinned by the assumption of droplet transmission of SARS-CoV-2.These factors suggest that the triaging of filtering face piece class 3 respirators might increase the risk of COVID-19 faced by some.

Ramzy, M., et al. (2020). "COVID-19 cardiac arrest management: A review for emergency clinicians." Am J Emerg Med **38**(12): 2693-2702.

INTRODUCTION: A great deal of literature has recently discussed the evaluation and management of the coronavirus disease of 2019 (COVID-19) patient in the emergency department (ED) setting, but there remains a dearth of literature providing guidance on cardiac arrest management in this population. OBJECTIVE: This narrative review outlines the underlying pathophysiology of patients with COVID-19 and discusses approaches to cardiac arrest management in the ED based on the current literature as well as extrapolations from experience with other pathogens. DISCUSSION: Patients with COVID-19 may experience cardiovascular manifestations that place them at risk for acute myocardial injury, arrhythmias, and cardiac arrest. The mortality for these critically ill patients is high and increases with age and comorbidities. While providing resuscitative interventions and performing procedures on these patients, healthcare providers must adhere to strict infection control measures and prioritize their own safety through the appropriate use of personal protective equipment. A novel approach must be implemented in combination with national guidelines. The changes in these guidelines emphasize early placement of an advanced airway to limit nosocomial viral transmission and encourage healthcare providers to determine the effectiveness of their efforts prior to placing staff at risk for exposure. CONCLUSIONS: While treatment priorities and goals are identical to pre-pandemic approaches, the management of COVID-19 patients in cardiac arrest has distinct differences from cardiac arrest patients without COVID-19. We provide a review of the current literature on the changes in cardiac arrest management as well as details outlining team composition.

Randremanana, R., et al. (2021). "Institut Pasteur International Network's efforts to guide control measures against the coronavirus disease 2019 (COVID-19) epidemic among healthcare workers in Africa." Int J Infect Dis **103**: 525-526.

During epidemic periods, HCW are vulnerable. In Africa, cohort studies implemented by the Institut Pasteur International Network in five countries showed after 3-month follow-up around 40% of the HCW have been infected by the SARS-CoV-2. So advocacy for HCW protection strategy need to be fostered and sustained by the health authorities all over the African continent.

Rangachari, P. and L. W. J (2020). "Preserving Organizational Resilience, Patient Safety, and Staff Retention during COVID-19 Requires a Holistic Consideration of the Psychological Safety of Healthcare Workers." Int J Environ Res Public Health **17**(12).

During the COVID-19 pandemic, healthcare workers are fighting a lethal virus with acute shortages of Personal Protective Equipment (PPE). These unprecedented circumstances have amplified the sources of emotional distress and worker burnout. However, many healthcare organizations (HCOs) in the United States, have opted for a "stoic approach" to healthcare worker support, i.e., no additional support beyond federal and state policy protections for the licensing and liability of healthcare workers. In this scenario, a key public health concern is sustaining an adequate healthcare workforce, both by way of quantity (adequate numbers) and quality (maximizing clinician resilience to provide safe care to large volumes of patients under challenging conditions). Therefore, it is imperative for HCO leaders to recognize that a limited view of worker psychological safety, without due consideration for the broader emotional distress created by the pandemic, could have the effect of restricting organizational resilience and adversely impacting patient safety and staff retention during and beyond the pandemic. This paper uses the organizational resilience framework to discuss the potential impact of a stoic approach to healthcare worker support on patient safety and staff retention in a hospital intensive care unit (ICU) during COVID-19. The discussion in turn, helps to develop recommendations for HCOs to overcome these challenges.

Ranjan, P., et al. (2020). "Association Between Self-Reported Adherence to Preventive Practices and Probability of Turning COVID-19 Positive: A Cross-Sectional Analytical Study." Cureus **12**(12): e11815.

Background Preventive practices are the mainstay to mitigate the spread of the COVID-19 pandemic. We tried to assess the self-reported adherence of our participants to the already known preventive practices. Furthermore, we tried to determine whether the non-compliance to specific preventive practices was associated with the acquisition of the infection or not. Methods We enrolled 379 healthcare workers, hospital staff, and their family members who were tested for COVID-19 by reverse transcription-polymerase chain reaction (RT-PCR) in an outpatient clinic. Socio-demography and the infection prevention practices of the individuals were recorded in a preformed questionnaire. Statistical analysis was performed to find out the statistical association between these factors and the RT-PCR results. Adjusted and unadjusted odds ratios were determined to find out the degree of protection provided by each of the preventive practices concerning the development of the disease. Results Social distancing (p<0.001), hand hygiene (p<0.001), ensuring N-95 mask fit check (p<0.001), and the use of alternative medications (p=0.002) were found to be protective. Resident doctors were at a lower risk of developing the disease as compared to the other healthcare workers (odds ratio: 0.39). Conclusion The failure to practice the already known preventive practices is probably one of the most important factors in the progression of the COVID-19 pandemic. Adherence to these practices is the intervention of choice to reduce disease transmission in the current scenario.

Rao, S. P. N., et al. (2021). "Small and sick newborn care during the COVID-19 pandemic: global survey and thematic analysis of healthcare providers' voices and experiences." BMJ Glob Health **6**(3).

INTRODUCTION: The COVID-19 pandemic is disrupting health systems globally. Maternity care disruptions have been surveyed, but not those related to vulnerable small newborns. We aimed to survey reported disruptions to small and sick newborn care worldwide and undertake thematic analysis of healthcare providers' experiences and proposed mitigation strategies. METHODS: Using a widely disseminated online survey in three languages, we reached out to neonatal healthcare providers. We collected data on COVID-19 preparedness, effects on health personnel and on newborn care services, including kangaroo mother care (KMC), as well as disruptors and solutions. RESULTS: We analysed 1120 responses from 62 countries, mainly low and middle-income countries (LMICs). Preparedness for COVID-19 was suboptimal in terms of guidelines and availability of personal protective equipment. One-third reported routine testing of all pregnant women, but 13% had no testing capacity at all. More than 85% of health personnel feared for their own health and 89% had increased stress. Newborn care practices were disrupted both due to reduced care-seeking and a compromised workforce. More than half reported that evidence-based interventions such as KMC were discontinued or discouraged. Separation of the mother-baby dyad was reported for both COVID-positive mothers (50%) and those with unknown status (16%). Follow-up care was disrupted primarily due to families' fear of visiting hospitals (~73%). CONCLUSION: Newborn care providers are stressed and there is lack clarity and guidelines regarding care of small newborns during the pandemic. There is an urgent need to protect life-saving interventions, such as KMC, threatened by the pandemic, and to be ready to recover and build back better.

Rapisarda, L., et al. (2021). "Facemask headache: a new nosographic entity among healthcare providers in COVID-19 era." Neurol Sci **42**(4): 1267-1276.

BACKGROUND: SARS-CoV-2 is a novel infectious agent causing coronavirus disease 2019, which has been declared as pandemic in March 2020. Personal protective equipment has been mandatory for healthcare workers in order to contain the outbreak of pandemic disease. Mild neurological disturbances such as headache have been related to the extensive utilization of facemask. This study aims to examine headache variations related to the intensive utilization of facemask among a cohort of healthcare professionals in a setting of low-medium risk of exposure to SARS-CoV-2. METHODS: This is a cross-sectional study among healthcare providers from different hospital and clinics in Italy. Each participant completed a specifically designed self-administered questionnaire. Headache features and outcome measures' change from baseline were evaluated over a 4-month period, in which wearing facemask has become mandatory for Italian healthcare workers. RESULTS: A total of 400 healthcare providers completed the questionnaire, 383 of them met the inclusion criteria. The majority were doctors, with a mean age of 33.4 ± 9.2 years old. Among 166/383 subjects, who were headache free at baseline, 44 (26.5%) developed de novo headache. Furthermore, 217/383 reported a previous diagnosis of primary headache disorder: 137 were affected by migraine and 80 had tension-type headache. A proportion (31.3%) of these primary headache sufferers experienced worsening of their pre-existing headache disorder, mainly for migraine frequency and attack mean duration. CONCLUSIONS: Our data showed the appearance of de novo associated facemask headache in previous headache-free subjects and an exacerbation of pre-existing primary headache disorders, mostly experienced by people with migraine disease.

Rasmussen, K. M. B., et al. (2021). "COVID-19 infection rate among tertiary referral center otorhinolaryngology healthcare workers." Eur Arch Otorhinolaryngol: 1-8.

IMPORTANCE: Otorhinolaryngology is considered one of the medical specialties with a high risk for exposure to corona disease 2019 (COVID-19). Uncontrolled transmission in a hospital department poses a risk to both healthcare workers (HCWs) and patients. OBJECTIVE: To monitor SARS-CoV-2 incidence, transmission, and antibody development among HCWs to identify high risk procedures, pathways, and work areas within the department. METHODS: Prospective cohort study of HCWs using repetitive oro- and nasopharygeal swab samples, antibody tests, and self-reported symptoms questionnaires at a tertiary referral center in Copenhagen, Denmark. RESULTS: 347/361 (96%) HCWs participated. Seven (1.9%) were positive on swab tests and none had symptoms. Fifteen (4.2%) developed antibodies. Only one case of potential transmission between HCWs was identified. Infection rates were low and no procedures or areas within the department were identified as exposing HCWs to a higher risk. CONCLUSIONS AND RELEVANCE: Adherence to the surveillance program was high. The low incidence among HCW during the first wave of the COVID-19 pandemic may reflect local transmission and infection control precautions, as well as a low infectious burden in the Danish society.

Ratwani, R. M., et al. (2020). "A descriptive analysis of an on-demand telehealth approach for remote COVID-19 patient screening." J Telemed Telecare: 1357633x20943339.

Introduction: COVID-19 requires methods for screening patients that adhere to physical distancing and other Centers for Disease Control and Prevention guidelines. There is little data on the use of on-demand telehealth to meet this need.Methods: The functional performance of on-demand telehealth as a COVID-19 remote patient screening approach was conducted by analysing 9270 patient requests.Results: Most on-demand telehealth requests (5712 of 9270 total requests; 61.6%) had a visit reason that was likely COVID-19 related. Of these, 79.1% (4518 of 5712) resulted in a completed encounter and 20.9% (1194 of 5712) resulted in left without being seen. Of the 4518 completed encounters, 19.1% were referred to an urgent care centre, emergency department or COVID-19 testing centre. The average completed encounter wait time was 26.5 min and the mean visit length was 8.8 min. For patients that completed an encounter 42.8% (1935 of 4518) stated they would have sought in-person care and 9.1% stated they would have done nothing if on-demand telehealth was unavailable.Discussion: On-demand telehealth can serve as a low-barrier approach to screen patients for COVID-19. This approach can prevent patients from visiting healthcare facilities, which reduces physical contact and reduces healthcare worker use of personal protective equipment.

Rawaf, S., et al. (2020). "Lessons on the COVID-19 pandemic, for and by primary care professionals worldwide." Eur J Gen Pract **26**(1): 129-133.

The COVID-19 pandemic has modified organisation and processes of primary care. In this paper, we aim to summarise experiences of international primary care systems. We explored personal accounts and findings in reporting on the early experiences from primary care during the pandemic, through the online Global Forum on Universal Health Coverage and Primary Health Care. During the early stage of the pandemic, primary care continued as the first point of contact to the health system but was poorly informed by policy makers on how to fulfil its role and ill equipped to provide care while protecting staff and patients against further spread of the infection. In many countries, the creativity and initiatives of local health professionals led to the introduction or extension of the use of telephone, e-mail and virtual consulting, and introduced triaging to separate 'suspected' COVID-19 from non-COVID-19 care. There were substantial concerns of collateral damage to the health of the population due to abandoned or postponed routine care. The pandemic presents important lessons to strengthen health systems through better connection between public health, primary care, and secondary care to cope better with future waves of this and other pandemics.

Razvi, S., et al. (2020). "Exposure of hospital healthcare workers to the novel coronavirus (SARS-CoV-2)." Clin Med (Lond) **20**(6): e238-e240.

Healthcare workers (HCWs) are at higher risk of infection with the coronavirus disease 2019 (COVID-19) and can also amplify outbreaks within healthcare facilities if they become ill. Certain groups are known to be at higher risk of contracting severe COVID-19 infection, such as men and people from Black, Asian and minority ethnic (BAME) backgrounds. Identifying and managing HCWs who have been exposed to COVID-19 is of utmost importance in preventing healthcare transmission and protecting staff and vulnerable patients in healthcare settings. Recently, antibody testing to diagnose previous COVID-19 exposure among HCW has commenced in the UK. This provided an opportunity to assess exposure to COVID-19 among the various subgroups within the HCW community, based on their roles and ethnic background. We found that HCWs working in patient-facing roles were twice as likely to have been exposed to COVID-19 than their colleagues in non-patient-facing roles. Reassuringly, workers from BAME backgrounds had a similar risk of previous COVID-19 exposure to their white colleagues. More research is required to assess how frontline staff, especially those working in patient facing roles, can reduce their risk of exposure to COVID-19.

Razzak, J. A., et al. (2020). "Initial estimates of COVID-19 infections in hospital workers in the United States during the first wave of pandemic." PLoS One **15**(12): e0242589.

OBJECTIVE: We estimated the number of hospital workers in the United States (US) that might be infected or die during the COVID-19 pandemic based on the data in the early phases of the pandemic. METHODS: We calculated infection and death rates amongst US hospital workers per 100 COVID-19-related deaths in the general population based on observed numbers in Hubei, China, and Italy. We used Monte Carlo simulations to compute point estimates with 95% confidence intervals for hospital worker (HW) infections in the US based on each of these two scenarios. We also assessed the impact of restricting hospital workers aged ≥ 60 years from performing patient care activities on these estimates. RESULTS: We estimated that about 53,000 hospital workers in the US could get infected, and 1579 could die due to COVID19. The availability of PPE for high-risk workers alone could reduce this number to about 28,000 infections and 850 deaths. Restricting high-risk hospital workers such as those aged ≥ 60 years from direct patient care could reduce counts to 2,000 healthcare worker infections and 60 deaths. CONCLUSION: We estimate that US hospital workers will bear a significant burden of illness due to COVID-19. Making PPE available to all hospital workers and reducing the exposure of hospital workers above the age of 60 could mitigate these risks.

Razzini, K., et al. (2020). "SARS-CoV-2 RNA detection in the air and on surfaces in the COVID-19 ward of a hospital in Milan, Italy." Sci Total Environ **742**: 140540.

The COVID-19 outbreak has rapidly progressed worldwide finding the health system, scientists and society unprepared to face a little-known, fast spreading, and extremely deadly virus. Italy is one of the countries hardest hit by the pandemic, resulting in healthcare facilities bearing heavy burdens and severe restrictive measures. Despite efforts to clarify the virus transmission, especially in indoor scenarios, several aspects of SARS-CoV-2 spread are still rudimentary. This study evaluated the contamination of the air and surfaces by SARS-CoV-2 RNA in the COVID-19 isolation ward of a hospital in Milan, Italy. A total of 42 air and surface samples were collected inside five different zones of the ward including contaminated (COVID-19 patients' area), semi-contaminated (undressing room), and clean areas. SARS-CoV-2 RNA detection was performed using real time reverse transcription polymerase chain reaction. Overall, 24.3% of swab samples were positive, but none of these were collected in the clean area. Thus, the positivity rate was higher in contaminated (35.0%) and semi-contaminated (50.0%) areas than in clean areas (0.0%; P<0.05). The most contaminated surfaces were hand sanitizer dispensers (100.0%), medical equipment (50.0%), medical equipment touch screens (50.0%), shelves for medical equipment (40.0%), bedrails (33.3%), and door handles (25.0%). All the air samples collected from the contaminated area, namely the intensive care unit and corridor, were positive while viral RNA was not detected in either semi-contaminated or clean areas. These results showed that environmental contamination did not involve clean areas, but the results also support the need for strict disinfection, hand hygiene and protective measures for healthcare workers as well as the need for airborne isolation precautions.

Regli, A., et al. (2021). "The role of fit testing N95/FFP2/FFP3 masks: a narrative review." Anaesthesia **76**(1): 91-100.

For healthcare workers performing aerosol-generating procedures during the COVID-19 pandemic, well fitted filtering facepiece respirators, for example, N95/FFP2 or N99/FFP3 masks, are recommended as part of personal protective equipment. In this review, we evaluate the role of fit checking and fit testing of respirators, in addition to airborne protection provided by respirators. Filtering facepiece respirators are made of material with sufficient high filter capacity to protect against airborne respiratory viruses. Adequate viral protection can only be provided by respirators that properly fit the wearer's facial characteristics. Initial fit pass rates vary between 40% and 90% and are especially low in female and in Asian healthcare workers. Fit testing is recommended to ensure a proper fit of respirators for the individual healthcare worker so that alternative respirators can be selected if required. Although fit testing is required to comply with respirator standards, it is not performed consistently within all healthcare settings. Fit checking (a self-test) is recommended every time a healthcare worker dons a respirator, but is unreliable in detecting proper fit or leak. Additionally, fit testing has a high educational value and as such is best performed as part of a hospital respiratory protection programme. Whether fit checking alone, as opposed to fit tested and fit checked respirators, provides adequate airborne protection against aerosols containing the SARS-CoV-2 virus and other respiratory viruses remains unknown. While fit testing undoubtedly incurs additional costs, it is still recommended, not only to protect healthcare workers but also as it may reduce overall healthcare cost when considering the potential costs of sickness leave and the associated legal costs of compensation.

Reiter, T., et al. (2021). "COVID-19 serology in nephrology healthcare workers." Wien Klin Wochenschr: 1-8.

BACKGROUND: Chronic kidney disease patients show a high mortality in cases of a severe acute respiratory syndrome coronavirus-2 (SARS-CoV‑2) infection. Thus, information on the sero-status of nephrology personnel might be crucial for patient protection; however, limited information exists about the presence of SARS-CoV‑2 antibodies in asymptomatic individuals. METHODS: We examined the seroprevalence of SARS-CoV‑2 IgG and IgM antibodies among healthcare workers of a tertiary care kidney center during the the first peak phase of the corona virus disease 2019 (COVID-19) crisis in Austria using an orthogonal test strategy and a total of 12 commercial nucleocapsid protein or spike glycoprotein-based assays as well as Western blotting and a neutralization assay. RESULTS: At baseline 60 of 235 study participants (25.5%, 95% confidence interval, CI 20.4-31.5%) were judged to be borderline positive or positive for IgM or IgG using a high sensitivity/low specificity threshold in one test system. Follow-up analysis after about 2 weeks revealed IgG positivity in 12 (5.1%, 95% CI: 2.9-8.8%) and IgM positivity in 6 (2.6%, 95% CI: 1.1-5.6) in at least one assay. Of the healthcare workers 2.1% (95% CI: 0.8-5.0%) showed IgG nucleocapsid antibodies in at least 2 assays. By contrast, positive controls with proven COVID-19 showed antibody positivity among almost all test systems. Moreover, serum samples obtained from healthcare workers did not show SARS-CoV‑2 neutralizing capacity, in contrast to positive controls. CONCLUSION: Using a broad spectrum of antibody tests the present study revealed inconsistent results for SARS-CoV‑2 seroprevalence among asymptomatic individuals, while this was not the case among COVID-19 patients. TRIAL REGISTRATION NUMBER: CONEC, ClinicalTrials.gov number NCT04347694.

Rekatsina, M., et al. (2020). "Healthcare at the time of COVID-19: A review of the current situation with emphasis on anesthesia providers." Best Pract Res Clin Anaesthesiol **34**(3): 539-551.

Coronavirus disease 2019 (COVID-19) is spreading rapidly around the world with devastating consequences on patients, healthcare workers, health systems, as well as economies. While, healthcare systems are globally operating at maximum capacity, healthcare workers and especially anesthesia providers are facing extreme pressures, something that is also leading to declining availability and increasing stress. In this regard, it is extremely concerning the fact that some regions worldwide have reported up to 20% of their cases to be healthcare workers. When considering that the global case fatality rate may be as much as 5.4%, these numbers are concerning and unacceptable. As this pandemic accelerates, access to personal protective equipment for health workers is a key concern since at present, healthcare workers are every country's most valuable resource in the fight against COVID-19. Governments and heath organizations should take care of their staff and support them in any way possible. This review aims to describe the current situation anesthesia providers are facing in the setting of COVID-19 and provide solutions and evidence on important concerns, including which guidance to follow, the level of equipment that is adequate, and the level of protection they need for every patient being administered an anesthetic.

Ren, J., et al. (2020). "Prevention of nosocomial COVID-19 infections in otorhinolaryngology-head and neck surgery." World J Otorhinolaryngol Head Neck Surg **6**(Suppl 1): S6-s10.

Coronavirus disease 2019 (COVID-19) has rapidly evolved into a pandemic, causing a global public health crisis. Many frontline healthcare workers providing ear, nose and throat services have been reported to contract COVID-19 at work. Early during the COVID-19 outbreak, several medical professionals in Otolaryngology-Head and Neck Surgery were infected in Wuhan, China. A series of measures were then taken immediately, which successfully halted the spread of the disease. Here we would like to share the lessons we have learned, and our experience to protect our health care workers during the COVID-19 pandemic.

Ren, S. Y., et al. (2020). "Stability and infectivity of coronaviruses in inanimate environments." World J Clin Cases **8**(8): 1391-1399.

Severe acute respiratory syndrome coronavirus 2 (SARS-CoV-2) is a highly contagious virus that can transmit through respiratory droplets, aerosols, or contacts. Frequent touching of contaminated surfaces in public areas is therefore a potential route of SARS-CoV-2 transmission. The inanimate surfaces have often been described as a source of nosocomial infections. However, summaries on the transmissibility of coronaviruses from contaminated surfaces to induce the coronavirus disease 2019 are rare at present. This review aims to summarize data on the persistence of different coronaviruses on inanimate surfaces. The literature was systematically searched on Medline without language restrictions. All reports with experimental evidence on the duration persistence of coronaviruses on any type of surface were included. Most viruses from the respiratory tract, such as coronaviruses, influenza, SARS-CoV, or rhinovirus, can persist on surfaces for a few days. Persistence time on inanimate surfaces varied from minutes to up to one month, depending on the environmental conditions. SARS-CoV-2 can be sustained in air in closed unventilated buses for at least 30 min without losing infectivity. The most common coronaviruses may well survive or persist on surfaces for up to one month. Viruses in respiratory or fecal specimens can maintain infectivity for quite a long time at room temperature. Absorbent materials like cotton are safer than unabsorbent materials for protection from virus infection. The risk of transmission via touching contaminated paper is low. Preventive strategies such as washing hands and wearing masks are critical to the control of coronavirus disease 2019.

Ren, Y., et al. (2021). "Practice and exploration of infection prevention and control measures based on risk management of surgical patients during the epidemic of corona virus disease 2019 (COVID-19)." Am J Infect Control **49**(2): 151-157.

BACKGROUND: An outbreak of corona virus disease 2019 (COVID-19) in Wuhan, China has spread quickly across the world, the World Health Organization (WHO) has declared this a pandemic. COVID-19 can be transmitted from human to human and cause nosocomial infection that has brought great challenges to infection control in medical institutions. Due to the professional characteristics, the research hospital still received a large number of trauma emergency tasks during the outbreak. It is urgent to establish a graded prevention and control guidance of surgery. METHODS: Review the implementation of surgical grading control measures in this hospital during the epidemic of COVID-19. RESULTS: The surgical prevention measures based on patients with different risks included prescreening and preoperative risk assessment, preparation of operating room, medical staff protection and environmental disinfection measures, etc. From January 20 to March 5, 2020, a total of 4,720 operations had been performed in this hospital, of which 1,565 were emergency operations and 22 for medium-risk and high-risk patients who may have the 2019 severe acute respiratory syndrome coronavirus 2 infection. And there is no medical staff exposed during the implementation of protective measures. CONCLUSIONS: Through the risk assessment of surgical patients and adopting surgical grading control measures, the risk of severe acute respiratory syndrome coronavirus 2 spread during the surgical process can be reduced greatly.

Reszke, R., et al. (2021). "The Utilization of Protective Face Masks among Polish Healthcare Workers during COVID-19 Pandemic: Do We Pass the Exam?" Int J Environ Res Public Health **18**(2).

Relevant personal protective measures during the COVID-19 pandemic include face masks, possibly decreasing the risk of infection among the general population and healthcare workers (HCW) if utilized properly. The aim of the study was to assess whether different Polish HCW utilize face masks according to the 2020 World Health Organization guidance (WHO) criteria. This cross-sectional study included 1156 respondents who participated in an internet survey evaluating mask-related behaviors. All the WHO criteria were complied with by 1.4% of participants, regardless of medical profession, specialty or place of employment. HCW mostly adhered to criterion 1 (C1; strict covering of the face and mouth with the mask; 90.8%), C4 (washing/disinfecting the hands after touching/taking off the mask; 49%) and C3 (taking off the mask properly without touching the anterior surface; 43.4%), whereas C2 (avoidance of touching the mask with hands) was complied with least commonly (6.8%). HCW with mask-induced itch (31.6%) complied to C2 less often (odds ratio 0.53; p = 0.01). The study reveals that Polish HCW rarely adhere to all the 2020 WHO guidance criteria on the use of masks, whereas the adherence to particular criteria is variable and may be associated with the presence of skin-related conditions and other factors. Better compliance with the recommendations in the future is necessary to increase personal safety of HCW and prevent the transmission of SARS-CoV-2.

Rhee, C., et al. (2020). "Incidence of Nosocomial COVID-19 in Patients Hospitalized at a Large US Academic Medical Center." JAMA Netw Open **3**(9): e2020498.

IMPORTANCE: Some patients are avoiding essential care for fear of contracting coronavirus disease 2019 (COVID-19) in hospitals. There are few data, however, on the risk of acquiring COVID-19 in US hospitals. OBJECTIVE: To assess the incidence of COVID-19 among patients hospitalized at a large US academic medical center in the 12 weeks after the first inpatient case was identified. DESIGN, SETTING, AND PARTICIPANTS: This cohort study included all patients admitted to Brigham and Women's Hospital (Boston, Massachusetts) between March 7 and May 30, 2020. Follow-up occurred through June 17, 2020. Medical records for all patients who first tested positive for severe acute respiratory syndrome coronavirus 2 (SARS-CoV-2) by reverse-transcription polymerase chain reaction (RT-PCR) on hospital day 3 or later or within 14 days of discharge were reviewed. EXPOSURES: A comprehensive infection control program was implemented that included dedicated COVID-19 units with airborne infection isolation rooms, personal protective equipment in accordance with US Centers for Disease Control and Prevention recommendations, personal protective equipment donning and doffing monitors, universal masking, restriction of visitors, and liberal RT-PCR testing of symptomatic and asymptomatic patients. MAIN OUTCOMES AND MEASURES: Whether infection was community or hospital acquired based on timing of tests, clinical course, and exposures. RESULTS: Over the 12-week period, 9149 patients (mean [SD] age, 46.1 [26.4] years; median [IQR] age, 51 years [30-67 years]; 5243 female [57.3%]) were admitted to the hospital, for whom 7394 SARS-CoV-2 RT-PCR tests were performed; 697 COVID-19 cases were confirmed, translating into 8656 days of COVID-19-related care. Twelve of the 697 hospitalized patients with COVID-19 (1.7%) first tested positive on hospital day 3 or later (median, 4 days; range, 3-15 days). Of these, only 1 case was deemed to be hospital acquired, most likely from a presymptomatic spouse who was visiting daily and diagnosed with COVID-19 before visitor restrictions and masking were implemented. Among 8370 patients with non-COVID-19-related hospitalizations discharged through June 17, 11 (0.1%) tested positive within 14 days (median time to diagnosis, 6 days; range, 1-14 days). Only 1 case was deemed likely to be hospital acquired, albeit with no known exposures. CONCLUSIONS AND RELEVANCE: In this cohort study of patients in a large academic medical center with rigorous infection control measures, nosocomial COVID-19 was rare during the height of the pandemic in the region. These findings may inform practices in other institutions and provide reassurance to patients concerned about contracting COVID-19 in hospitals.

Rhee, M. S. M., et al. (2021). "Carbon dioxide increases with face masks but remains below short-term NIOSH limits." BMC Infect Dis **21**(1): 354.

BACKGROUND AND PURPOSE: COVID-19 pandemic led to wide-spread use of face-masks, respirators and other personal protective equipment (PPE) by healthcare workers. Various symptoms attributed to the use of PPE are believed to be, at least in part, due to elevated carbon-dioxide (CO2) levels. We evaluated concentrations of CO2 under various PPE. METHODS: In a prospective observational study on healthy volunteers, CO2 levels were measured during regular breathing while donning 1) no mask, 2) JustAir® powered air purifying respirator (PAPR), 3) KN95 respirator, and 4) valved-respirator. Serial CO2 measurements were taken with a nasal canula at a frequency of 1-Hz for 15-min for each PPE configuration to evaluate whether National Institute for Occupational Safety and Health (NIOSH) limits were breached. RESULTS: The study included 11 healthy volunteers, median age 32 years (range 16-54) and 6 (55%) men. Percent mean (SD) changes in CO2 values for no mask, JustAir® PAPR, KN95 respirator and valve respirator were 0.26 (0.12), 0.59 (0.097), 2.6 (0.14) and 2.4 (0.59), respectively. Use of face masks (KN95 and valved-respirator) resulted in significant increases in CO2 concentrations, which exceeded the 8-h NIOSH exposure threshold limit value-weighted average (TLV-TWA). However, the increases in CO2 concentrations did not breach short-term (15-min) limits. Importantly, these levels were considerably lower than the long-term (8-h) NIOSH limits during donning JustAir® PAPR. There was a statistically significant difference between all pairs (p < 0.0001, except KN95 and valved-respirator (p = 0.25). However, whether increase in CO2 levels are clinically significant remains debatable. CONCLUSION: Although, significant increase in CO2 concentrations are noted with routinely used face-masks, the levels still remain within the NIOSH limits for short-term use. Therefore, there should not be a concern in their regular day-to-day use for healthcare providers. The clinical implications of elevated CO2 levels with long-term use of face masks needs further studies. Use of PAPR prevents relative hypercapnoea. However, whether PAPR should be advocated for healthcare workers requiring PPE for extended hours needs to evaluated in further studies.

Riccò, M., et al. (2020). "Inappropriate risk perception for SARS-CoV-2 infection among Italian HCWs in the eve of COVID-19 pandemic." Acta Biomed **91**(3): e2020040.

Sir, Italy has been recently involved in the outbreak of severe interstitial pneumonia associated with the previously unknown Coronavirus SARS-CoV-2 (1,2). Even before the notification of the first autochthonous cases, the SARS-CoV-2 associated syndrome (COVID-19) had raised an intense attention in the public opinion (3), with a counterproductive over-abundance of mixed quality information. As even Italian healthcare workers (HCWs) were not spared by subsequent misunderstandings and knowledge gaps during the previous influenza pandemic of 2009 (4), we performed a web-based survey (Google® Modules), specifically aimed to characterize knowledge status and risk perceptions in a sample from participating to 6 Facebook discussion groups (181,684 total unique members at the time of the study). The questionnaire was made available between February 1st and 7th, 2020, i.e. around 2 weeks before the first COVID-19 was officially diagnosed in Italian residents.   Overall, the sampled population included 2106 respondents (Table 1), and 39.3% were HCWs. Even though HCWs were more likely to exhibit a better understanding of SARS-CoV-2/COVID-19 related issues (aOR 2.195, 95%CI 1.809 to 2.664), they were not exempt for misunderstandings, particularly on actual incidence and lethality of COVID-19. Interestingly, most of respondents were aware of the main clinical features of COVID-19, with HCWs more frequently acknowledging that the COVID-19 may run pauci- or even asymptomatic (86.3% vs. 79.1%), resembling an Influenza-Like Illness (i.e. fever, cough, headache, etc.), with a potential latency up to 14 days (85·9% vs· 80·3%), eventually spreading by droplets (98.5% vs. 92.7%) rather through running water (92.3% vs· 79.8%), or blood/body fluids (88.0% vs. 70.4%).   Retrospectively, the assessment of preventive measures and risk perception appears somewhat worrisome. For instance, while HCWs were more likely to acknowledge as an appropriate preventive measure wearing a filtering mask (i.e. N95/FFP2/3 mask; aOR 2.296, 95%CI 1.507 to 3.946), around ¼ of HCWs failed to recognize the importance of such personal protective equipment, while 7.4% felt as appropriate the wearing of a surgical mask. Moreover, not only COVID-19 was appropriately acknowledged as a severe disease by only 62.0% of respondents, with no differences between HCWs and non-HCWs, but an even smaller share (i.e. 8.0%) reported any concern for being infected by SARS-CoV-2 in Italy. In fact, at the time of the survey SARS-CoV-2 was more properly associated with international travelers (26.7%).   Our results are therefore of certain interests for several reasons. First at all, early epidemiological reports on the Italian cases of COVID-19 hint towards some failures in the initial management of incident cases (5-6). In fact, in our survey a large share of respondents substantially overlooked the risk to interact with SARS-CoV-2 positive subjects, that was otherwise perceived as a not-so-severe disease (i.e. "nothing more than a seasonal flu", as often described in some social media) (7). Moreover, around a 1/3 of HCWs participating to the study presumptively did not use proper personal protective equipment for the airways interacting with possible COVID-19 cases, either underestimating the infection risk or being unable to recognize early symptoms. Actually, the base of evidence shared by participants at the time of the study substantially ignored that COVID-19 may be characterized by dermatologic and gastro-intestinal symptoms (8-9). As most of infections may be actually pauci- or asymptomatic, such early exposure in the healthcare settings may have contributed to the quick spreading of SARS-CoV-2 epidemic in Northern Italy.   Therefore, despite the intrinsic limits of a convenience sampling, web-based survey (10), our study stresses the importance to improve the overall quality of information on COVID-19 conveyed not only in HCWs, but also in the general population. Moreover, our data may contribute to clarify the early stages of SARS-CoV-2 pandemic in Italy.

Ridder, M. P., et al. (2021). "A Practical Approach to Filtering Facepiece Respirator Decontamination and Reuse: Ultraviolet Germicidal Irradiation." Curr Treat Options Infect Dis: 1-12.

PURPOSE OF REVIEW: Prior outbreaks of respiratory viruses have demonstrated the need for adequate personal protective equipment (PPE) for healthcare workers, particularly filtering facepiece respirators (FFR). Due to shortfalls of PPE during the SARS CoV-2 pandemic, the need for FFR decontamination and reuse (FFR-DR) strategies is paramount. This paper aims to discuss primary decontamination strategies, with an in-depth analysis of ultraviolet germicidal irradiation (UVGI), arriving at the decontamination strategy utilized at the Nebraska Medical Center (NMC). METHODS: Review of the primary literature in regard to FFR-DR as well as a synopsis of the current protocol for FFR-DR at NMC. RECENT FINDINGS: UVGI demonstrates effective decontamination of multiple pathogens-including several human respiratory viruses-while maintaining mask integrity and filtering capacity. UVGI was associated with degradation of strap integrity at higher doses than that utilized for decontamination or with reuse beyond 20 times. SUMMARY: UVGI effectively decontaminates N95 FFRs without significant reduction to fit or strap integrity and can be employed as a strategy for FFR-DR in times of emergency.

Riguzzi, M. and S. Gashi (2021). "Lessons From the First Wave of COVID-19: Work-Related Consequences, Clinical Knowledge, Emotional Distress, and Safety-Conscious Behavior in Healthcare Workers in Switzerland." Front Psychol **12**: 628033.

The coronavirus disease (COVID-19) imposes an unusual risk to the physical and mental health of healthcare workers and thereby to the functioning of healthcare systems during the crisis. This study investigates the clinical knowledge of healthcare workers about COVID-19, their ways of acquiring information, their emotional distress and risk perception, their adherence to preventive guidelines, their changed work situation due to the pandemic, and their perception of how the healthcare system has coped with the pandemic. It is based on a quantitative cross-sectional survey of 185 Swiss healthcare workers directly attending to patients during the pandemic, with 22% (n = 40) of them being assigned to COVID-19-infected patients. The participants answered between 16th June and 15th July 2020, shortly after the first wave of COVID-19 had been overcome and the national government had relaxed its preventive regulations to a great extent. The questionnaire incorporated parts of the "Standard questionnaire on risk perception of an infectious disease outbreak" (version 2015), which were adapted to the case of COVID-19. Clinical knowledge was lowest regarding the effectiveness of standard hygiene (p < 0.05). Knowledge of infectiousness, incubation time, and life-threatening disease progression was higher, however still significantly lower than regarding asymptomatic cases and transmission without physical contact (p < 0.001). 70% (95%-confidence interval: 64-77%) of the healthcare workers reported considerable emotional distress on at least one of the measured dimensions. They worried significantly more strongly about patients, elderly people, and family members, than about their own health (p < 0.001). Adherence to (not legally binding) preventive guidelines by the government displayed patterns such that not all guidelines were followed equally. Most of the participants were faced with a lack of protective materials, personnel, structures, processes, and contingency plans. An increase in stress level was the most prevalent among the diverse effects the pandemic had on their work situation. Better medical equipment (including drugs), better protection for their own mental and physical health, more (assigned) personnel, more comprehensive information about the symptoms of the disease, and a system of earlier warning were the primary lessons to be learned in view of upcoming waves of the pandemic.

Rincón Sánchez, R. A., et al. (2020). "Quality of vision in endoscopy in the midst of a pandemic: Does PPE influence quality of vision during gastrointestinal endoscopy?" Gastroenterol Hepatol.

INTRODUCTION: In the midst of the SARS-CoV-2 virus (COVID-19) pandemic, health professionals, specifically gastroenterologists, have had to use personal protective equipment (PPE) to reduce contact with droplets and aerosols generated during gastrointestinal endoscopy. OBJECTIVE: To evaluate the impact of the use of two types of PPE on quality of vision during gastrointestinal endoscopy. METHODS: A cross-sectional observational pilot study in gastroenterologists who undergo an ophthalmological examination of visual acuity and quality of vision when using two types of PPE. Type #1: 3M N95 1860 green respirator + 3M mono safety glasses + protective screen. Type #2: 3M 6800 full facepiece + 3M NIOSH 7093C HF/P100 filters. RESULTS: Visual acuity and quality of vison parameters while using the PPE that is routinely used when performing gastrointestinal endoscopy during the pandemic were evaluated. It was found that Modality #1 was associated with decreases of up to 37% in visual acuity, 25% in colour visualisation and 75% in contrast sensitivity among digestive endoscopists within minutes of placement. These figures worsened over the course of the procedure, rising to 75%, 60% and 100%, respectively. Modality #2 was not associated with any deterioration in quality of vision. CONCLUSIONS: The different PPE modalities used during gastrointestinal endoscopy could have an impact on the quality of endoscopy studies performed during the SARS-CoV-2 (COVID-19) pandemic.

eng

Riou, C., et al. (2020). "Rapid, simplified whole blood-based multiparameter assay to quantify and phenotype SARS-CoV-2 specific T cells." medRxiv.

Rapid tests to evaluate SARS-CoV-2-specific T cell responses are urgently needed to decipher protective immunity and aid monitoring vaccine-induced immunity. Using a rapid whole blood assay requiring minimal amount of blood, we measured qualitatively and quantitatively SARS-CoV-2-specific CD4 T cell responses in 31 healthcare workers, using flow cytometry. 100% of COVID-19 convalescent participants displayed a detectable SARS-CoV-2-specific CD4 T cell response. SARS-CoV-2-responding cells were also detected in 40.9% of participants with no COVID-19-associated symptoms or who tested PCR negative. Phenotypic assessment indicated that, in COVID-19 convalescent participants, SARS-CoV-2 CD4 responses displayed an early differentiated memory phenotype with limited capacity to produce IFNγ. Conversely, in participants with no reported symptoms, SARS-CoV-2 CD4 responses were enriched in late differentiated cells, co-expressing IFNγ and TNFα and also Granzyme B. This proof of concept study presents a scalable alternative to PBMC-based assays to enumerate and phenotype SARS-CoV-2-responding T cells, thus representing a practical tool to monitor adaptive immunity in vaccine trials. SUMMARY: In this proof of concept study, we show that SARS-CoV-2 T cell responses are easily detectable using a rapid whole blood assay requiring minimal blood volume. Such assay could represent a suitable tool to monitor adaptive immunity in vaccine trials.

Rivett, L., et al. (2020). "Screening of healthcare workers for SARS-CoV-2 highlights the role of asymptomatic carriage in COVID-19 transmission." Elife **9**.

Significant differences exist in the availability of healthcare worker (HCW) SARS-CoV-2 testing between countries, and existing programmes focus on screening symptomatic rather than asymptomatic staff. Over a 3 week period (April 2020), 1032 asymptomatic HCWs were screened for SARS-CoV-2 in a large UK teaching hospital. Symptomatic staff and symptomatic household contacts were additionally tested. Real-time RT-PCR was used to detect viral RNA from a throat+nose self-swab. 3% of HCWs in the asymptomatic screening group tested positive for SARS-CoV-2. 17/30 (57%) were truly asymptomatic/pauci-symptomatic. 12/30 (40%) had experienced symptoms compatible with coronavirus disease 2019 (COVID-19)>7 days prior to testing, most self-isolating, returning well. Clusters of HCW infection were discovered on two independent wards. Viral genome sequencing showed that the majority of HCWs had the dominant lineage B∙1. Our data demonstrates the utility of comprehensive screening of HCWs with minimal or no symptoms. This approach will be critical for protecting patients and hospital staff.

Patients admitted to NHS hospitals are now routinely screened for SARS-CoV-2 (the virus that causes COVID-19), and isolated from other patients if necessary. Yet healthcare workers, including frontline patient-facing staff such as doctors, nurses and physiotherapists, are only tested and excluded from work if they develop symptoms of the illness. However, there is emerging evidence that many people infected with SARS-CoV-2 never develop significant symptoms: these people will therefore be missed by ‘symptomatic-only’ testing. There is also important data showing that around half of all transmissions of SARS-CoV-2 happen before the infected individual even develops symptoms. This means that much broader testing programs are required to spot people when they are most infectious. Rivett, Sridhar, Sparkes, Routledge et al. set out to determine what proportion of healthcare workers was infected with SARS-CoV-2 while also feeling generally healthy at the time of testing. Over 1,000 staff members at a large UK hospital who felt they were well enough to work, and did not fit the government criteria for COVID-19 infection, were tested. Amongst these, 3% were positive for SARS-CoV-2. On closer questioning, around one in five reported no symptoms, two in five very mild symptoms that they had dismissed as inconsequential, and a further two in five reported COVID-19 symptoms that had stopped more than a week previously. In parallel, healthcare workers with symptoms of COVID-19 (and their household contacts) who were self-isolating were also tested, in order to allow those without the virus to quickly return to work and bolster a stretched workforce. Finally, the rates of infection were examined to probe how the virus could have spread through the hospital and among staff – and in particular, to understand whether rates of infection were greater among staff working in areas devoted to COVID-19 patients. Despite wearing appropriate personal protective equipment, healthcare workers in these areas were almost three times more likely to test positive than those working in areas without COVID-19 patients. However, it is not clear whether this genuinely reflects greater rates of patients passing the infection to staff. Staff may give the virus to each other, or even acquire it at home. Overall, this work implies that hospitals need to be vigilant and introduce broad screening programmes across their workforces. It will be vital to establish such approaches before ‘lockdown’ is fully lifted, so healthcare institutions are prepared for any second peak of infections.

eng

Robbins, T., et al. (2020). "Healthcare staff perceptions and misconceptions regarding antibody testing in the United Kingdom: implications for the next steps for antibody screening." J Hosp Infect.

BACKGROUND: Healthcare workers have been at increased risk of exposure, infection and serious complications from COVID-19. Antibody testing has been used to identify staff members who have been previously infected by SARS-CoV-2, and has been rolled out rapidly in the United Kingdom. A number of comment and editorial articles have been published that raise concerns about antibody testing in this context. We present perceptions of National Health Service (NHS) healthcare workers in relation to SARS-CoV-2 antibody testing. METHODS: An electronic survey regarding perceptions towards SARS-CoV-2 antibody testing was distributed to all healthcare workers at a major NHS tertiary hospital following implementation of antibody testing. RESULTS: In total, 560 healthcare workers completed the survey (80% female; 25% of Black and Minority Ethnic background; 58% from frontline clinical staff). Exploring whether they previously had COVID-19 was the primary reported reason for choosing to undergo antibody testing (85.2%). In case of a positive antibody test, 72% reported that they would feel relieved, whilst 48% felt that they would be happier to work in a patient-facing area. Moreover, 12% responded that a positive test would mean "social distancing is less important", with 34% of the responders indicating that in this case they would be both less likely to catch COVID-19 and happier to visit friends/relatives. CONCLUSIONS: NHS staff members primarily seek out SARS-CoV-2 antibody testing for an appropriate reason. Based on our findings and given the lack of definite data regarding the extent of immunity protection from a positive SARS-CoV-2 antibody test, significant concerns may be raised regarding the reported interpretation by healthcare workers of positive antibody test results. This needs to be further explored and addressed to protect NHS staff and patients.

Rodríguez-Rey, R., et al. (2020). "Working in the Times of COVID-19. Psychological Impact of the Pandemic in Frontline Workers in Spain." Int J Environ Res Public Health **17**(21).

This study evaluates the psychological impact (PI) of the COVID-19 pandemic in frontline workers in Spain. Participants were 546 workers (296 healthcare workers, 105 media professionals, 89 grocery workers, and 83 protective service workers). They all completed online questionnaires assessing PI, sadness, concerns related to the COVID-19 pandemic, and demographic and work-related variables. All groups but protective services workers showed higher PI levels than the general population. Healthcare and grocery workers were the most affected, with 73.6% and 65.2% of the participants, respectively, showing a severe PI. Women showed a higher PI level. Healthcare workers in the regions with higher COVID-19 incidences reported greater PI levels. The main concerns were being infected by COVID-19 or infecting others. Levels of concern correlated with higher PI levels. The protection equipment was generally reported as insufficient, which correlated with higher PI levels. Professionals reporting to overwork during the crisis (60% mass-media, 38% of healthcare and grocery and 21.7% of protective service) showed higher PI levels. In the healthcare group, taking care of patients with COVID-19 (77%) or of dying patients with COVID-19 (43.9%) was associated with higher PI levels. The perceived social recognition of their work was inversely related to PI. Most of the sample had not received psychological support. We suggest some organizational measures for frontline institutions, such as the periodical monitoring or inclusion of psychologists specialized in crisis-management to prevent negative symptoms and provide timely support.

Rogado, J., et al. (2020). "Covid-19 and lung cancer: A greater fatality rate?" Lung Cancer **146**: 19-22.

BACKGROUND: Currently there are no reported series determining the Covid-19 infected lung cancer patient´s characteristics and outcome that allow us to clarify strategies to protect our patients. In our study we determine whether exists differences in cumulative incidence and severity of Covid-19 infection between lung cancer patients visiting our Medical Oncology department and the reference population of our center (320,000 people), in the current epicenter of the pandemic in Europe (Madrid, Spain). We also describe clinical and demographic factors associated with poor prognosis and Covid-19 treatment outcomes. PATIENTS AND METHODS: We retrospectively reviewed 1878 medical records of all Covid-19 patients who were admitted at Hospital Universitario Infanta Leonor of Madrid between March 5, 2020 and April 7, 2020, in order to detect cumulative incidence of Covid-19 in lung cancer patients. We also described Covid-19 treatment outcome, mortality and associated risk factors using univariate and multivariate logistic regression analysis. RESULTS: 17/1878 total diagnosis in our center had lung cancer (0.9 %) versus 1878/320,000 of the total reference population (p = 0.09). 9/17 lung cancer patients with Covid-19 diagnosis died (52.3 %) versus 192/1878 Covid-19 patients in our center (p < 0.0001). Dead lung cancer patients were elderly compared to survivors: 72 versus 64.5 years old (p = 0.12). Combined treatment with hydroxychloroquine and azithromycin improves the outcome of Covid-19 in lung cancer patients, detecting only 1/6 deaths between patients under this treatment versus others treatment, with statistical significance in the univariate and multivariate logistic regression (OR 0.04, p = 0.018). CONCLUSIONS: Lung cancer patients have a higher mortality rate than general population. Combined hydroxychloroquine and azithromycin treatment seems like a good treatment option. It is important to try to minimize visits to hospitals (without removing their active treatments) in order to decrease nosocomial transmission.

Romagnani, P., et al. (2020). "The COVID-19 infection: lessons from the Italian experience." J Public Health Policy **41**(3): 238-244.

The first case of the new coronavirus, COVID-19, was reported in China on 17 November 2019. By the end of March 2020, the rapid global spread of infection affected over 1 million people. Italy is one of the countries most impacted, with over 100,000 positive cases identified. The first detected cases were reported on 21 February 2020 in two Italian towns: Vo' Euganeo in the Province of Padua, Veneto region, and Codogno, in the Province of Lodi, Lombardy. In the next weeks the epidemic spread quickly across the country but mainly in the north of Italy. The two regions: Veneto and Lombardy, implemented different strategies to control the viral spread. In Veneto, health personnel tested both symptomatic and asymptomatic subjects, while in Lombardy only symptomatic cases were investigated. We analyzed the evolution of the epidemic in these regions and showed that testing both symptomatic and asymptomatic cases is a more effective strategy to mitigate the epidemic impact. We strongly recommend that decision-makers: ensure early isolation of symptomatic patients and rapid identification of their contacts; maximize testing rapidly, especially among people with multiple daily contacts with infected populations, high exposure to the public in essential services; rapidly increase diagnostic capacity by mobilizing trained personnel capable of performing rRT-PCR on respiratory samples; equip the population with protective masks.

Roupa, Z., et al. (2021). "Assessment of Knowledge and Perceptions of Health Workers Regarding COVID-19: A Cross-Sectional Study from Cyprus." J Community Health **46**(2): 251-258.

The COVID-19 disease is presently a matter of global public health concern as it could be potentially fatal. Health workers have at the moment, inadequate knowledge of prevention measures, and their erroneous practices may directly increase the risk of spread. As a result, matters are complicated further as far as the chain of infection is concerned. The present cross-sectional study was conducted with 494 Health Worker participants in Cyprus between the 1st and 20th of May and the data were collected via an online questionnaire. The survey was created using information from a previous study as well as current directives published on the WHO website. The statistical software SPSS 22.0 was used for data analysis. The ANOVA test was used for the comparison of quantitative variables, while the Kruskal-Wallis test was used in cases of non-parametric distribution. The Pearson or Spearman coefficients were used to test correlation and the levels of materiality were set to 0,05. Women constituted 66,7% of the participants and 44,8% of all participants were between the age of 30-39 years old. The majority (75,4%) of the participants were Nursing Officers and 44,5% held postgraduate qualifications. HWs appeared to have a positive perception (x̅ = 3.58, SD = 0.53, MR = 1-5) regarding the course of the pandemic and their knowledge of the virus was found to be generally satisfactory. The findings of the current study indicate that in case of a similar global public health crisis, certain educational interventions should be implemented. This could be achieved with the use of an appropriate strategy. As a result, all demographic-working strata of health professionals would be well-educated and informed.

Roux, C. L. and A. Dramowski (2020). "Personal protective equipment (PPE) in a pandemic: Approaches to PPE preservation for South African healthcare facilities." S Afr Med J **110**(6): 466-468.

Personal protective equipment (PPE) is key to protecting healthcare workers from COVID-19 infection, but the pandemic has disrupted supply chains globally and necessitated rapid review of the scientific evidence for PPE re-use. In South Africa, where the COVID-19 epidemic is still developing, healthcare facilities have a short window of opportunity to improve PPE supply chains, train staff on prudent PPE use, and devise plans to track and manage the inevitable increases in PPE demand. This article discusses the available PPE preservation strategies and addresses the issue of decontamination and re-use of N95 respirators as a last-resort strategy for critical shortages during the pandemic.

Rowan, N. J. and J. G. Laffey (2020). "Challenges and solutions for addressing critical shortage of supply chain for personal and protective equipment (PPE) arising from Coronavirus disease (COVID19) pandemic - Case study from the Republic of Ireland." Sci Total Environ **725**: 138532.

Coronavirus (COVID-19) is highly infectious agent that causes fatal respiratory illnesses, which is of great global public health concern. Currently, there is no effective vaccine for tackling this COVID19 pandemic where disease countermeasures rely upon preventing or slowing person-to-person transmission. Specifically, there is increasing efforts to prevent or reduce transmission to front-line healthcare workers (HCW). However, there is growing international concern regarding the shortage in supply chain of critical one-time-use personal and protective equipment (PPE). PPE are heat sensitive and are not, by their manufacturer's design, intended for reprocessing. Most conventional sterilization technologies used in hospitals, or in terminal medical device sterilization providers, cannot effectively reprocess PPE due to the nature and severity of sterilization modalities. Contingency planning for PPE stock shortage is important. Solutions in the Republic of Ireland include use of smart communication channels to improve supply chain, bespoke production of PPE to meets gaps, along with least preferred option, use of sterilization or high-level disinfection for PPE reprocessing. Reprocessing PPE must consider material composition, functionality post treatment, along with appropriate disinfection. Following original manufacturer of PPE and regulatory guidance is important. Technologies deployed in the US, and for deployment in the Republic of Ireland, are eco-friendly, namely vaporised hydrogen peroxide (VH2O2), such as for filtering facepiece respirators and UV irradiation and High-level liquid disinfection (Actichlor+) is also been pursed in Ireland. Safeguarding supply chain of PPE will sustain vital healthcare provision and will help reduce mortality.

Rowan, N. J. and J. G. Laffey (2021). "Unlocking the surge in demand for personal and protective equipment (PPE) and improvised face coverings arising from coronavirus disease (COVID-19) pandemic - Implications for efficacy, re-use and sustainable waste management." Sci Total Environ **752**: 142259.

Currently, there is no effective vaccine for tackling the ongoing COVID-19 pandemic caused by SARS-CoV-2 with the occurrence of repeat waves of infection frequently stretching hospital resources beyond capacity. Disease countermeasures rely upon preventing person-to-person transmission of SARS-CoV2 so as to protect front-line healthcare workers (HCWs). COVID-19 brings enormous challenges in terms of sustaining the supply chain for single-use-plastic personal and protective equipment (PPE). Post-COVID-19, the changes in medical practice will drive high demand for PPE. Important countermeasures for preventing COVID-19 transmission include mitigating potential high risk aerosol transmission in healthcare setting using medical PPE (such as filtering facepiece respirators (FFRs)) and the appropriate use of face coverings by the general public that carries a lower transmission risk. PPE reuse is a potential short term solution during COVID-19 pandemic where there is increased evidence for effective deployment of reprocessing methods such as vaporized hydrogen peroxide (30 to 35% VH2O2) used alone or combined with ozone, ultraviolet light at 254 nm (2000 mJ/cm(2)) and moist heat (60 °C at high humidity for 60 min). Barriers to PPE reuse include potentially trust and acceptance by HCWs. Efficacy of face coverings are influenced by the appropriate wearing to cover the nose and mouth, type of material used, number of layers, duration of wearing, and potentially superior use of ties over ear loops. Insertion of a nose clip into cloth coverings may help with maintaining fit. Use of 60 °C for 60 min (such as, use of domestic washing machine and spin dryer) has been advocated for face covering decontamination. Risk of virus infiltration in improvised face coverings is potentially increased by duration of wearing due to humidity, liquid diffusion and virus retention. Future sustained use of PPE will be influenced by the availability of recyclable PPE and by innovative biomedical waste management.

Rubbi, I., et al. (2020). "Healthcare personnel exposure to COVID - 19: an observational study on quarantined positive workers." Acta Biomed **91**(12-s): e2020012.

BACKGROUND AND AIM OF THE STUDY: COVID-19 is characterized by super spread events occurring in communities, e.g., hospitals. To limit virus diffusion among healthcare workers the use of personal protective equipment and screening tests are highly advised; also, isolation of virus positive professionals while monitoring their health condition is recommended. This study aims to assess, in a cohort of COVID-19 positive quarantined healthcare workers, the perceived source of infection and exposure risk as well as the clinical evolution of the disease through a surveillance interview. METHODS: A retrospective observational study accounting 896 observations on 93 healthcare professionals tested positive for COVID-19. Data were collected from the Nursing and Technical Directorate of Romagna, Ravenna, Local Health Company, Italy. RESULTS: 99.5% of the positive workers accepted phone interviews with management staff. 2.6% of workers were positive with increasing records in the specialist medical area. Nurses and social health professionals were mostly affected.  Patient exposure at a distance <1 m and a contact time > 2 hours was the first cause of positivity. In COVID-19 and territorial emergency departments, the first cause was the contact with colleagues. At the time of the infection, most of the staff wore a surgical mask. Cough, asthenia, fever, anosmia, dysgeusia, and rhinitis were common symptoms. Asymptomatic percentage was about 10%. The self-perceived physical condition was high (>7) and improved during the observation period. CONCLUSIONS: The diffusion rate of COVID-19 among healthcare workers is relatively low, probably due to the use of personal protective equipment. The distancing, also among colleagues, is a fundamental measure to reduce the possibility of infection. Symptoms are mild and can be controlled by surveillance measures. Constant contact with the organization is an essential strategy for promoting recovering of workers and reducing the spread of the virus within the healthcare organization.

Ruetzler, K., et al. (2021). "Respiratory protection among healthcare workers during cardiopulmonary resuscitation in COVID-19 patients." Am J Emerg Med **39**: 233.

Russell, L., et al. (2021). "Fluid-repellent surgical mask (FRSM) fit - one size does not fit all." Clin Med (Lond).

BACKGROUND: Fluid-repellent surgical masks (FRSMs) are recommended by the UK government for healthcare workers as personal protective equipment (PPE) against SARS-COV-2. UK Infection Prevention and Control (IPC) national guidelines states that 'masks must be well-fitting and fit for purpose, fully covering the mouth and nose'. AIM: To review the fit of the FRSM supplied to the NHS front line workers against the national IPC guidelines and, through re-audit, assess for improvements in fit with FRSM worn with a plastic strap (intervention A) and FFP3 mask (intervention B). METHOD: A three-part closed-loop audit was carried out comprising controlled observation, observation in the clinical area and questionnaire. Re-audit was carried out following interventions A and B. RESULTS: FRSMs slipped below the nose in 43% and below the mouth of 10% of participants during the controlled observation and below the nose (above or below the mouth) in 30% of staff in the clinical area. No masks slipped below the nose or mouth with intervention A or B. 86% of participants reported touching the FRSM to keep it in the correct position and 66% reported touching the FFP3. CONCLUSION: The current supply of FRSMs are poorly fitting for many users and do not meet the UK IPC guideline standard. These issues were not evident when worn with a plastic strap or with FFP3 masks.

Rutkowski, J. L., et al. (2020). "AAID White Paper: Management of the Dental Implant Patient During the COVID-19 Pandemic and Beyond." J Oral Implantol **46**(5): 454-466.

The scientific community's understanding of how the SARS-CoV-2 virus is transmitted and how to best mitigate its spread is improving daily. To help protect patients from acquiring COVID-19 from a dental office nosocomial infection, many state or local governments have classified dental treatments as "nonessential" and have paused routine dental care. Dentists have been instructed to perform only procedures designated as emergencies. Unfortunately, there is not a good understanding of what a dental emergency is among governmental leaders. What a government agency may perceive as an elective procedure may be seen as "essential" by the dental clinician responsible for maintaining the oral health of the patient. Each dental specialty understands the effects delayed care has on a patient's oral and systemic health. Dentistry has made extensive progress in improving oral health through prevention of the dental emergency. The dental profession must work together to prevent the reversal of the progress dentistry and patients have made. This American Academy of Implant Dentistry (AAID) White Paper discusses what COVID-19 is and how it impacts dental treatments, presents guidelines for dentistry in general and for dental implant related treatments, specifically. Recommendations for implant dentistry include the following: (1) what constitutes a dental implant related emergency, (2) how patients should be screened and triaged, (3) what personal protective equipment is necessary, (4) how operatories should be equipped, (5) what equipment should be used, and (6) what, when, and how procedures can be performed. This paper is intended to provide guidance for the dental implant practice so patients and dental health care providers can be safe, and offices can remain open and viable during the pandemic.

Rymarowicz, J., et al. (2020). "General surgeons' attitudes towards COVID-19: A national survey during the SARS-CoV-2 virus outbreak." Eur Surg: 1-6.

BACKGROUND: The COVID-19 global pandemic left the unprepared health care systems struggling to mount a measured response. This gave rise to important questions about surgeons' attitude towards surgical practice and the level of preparation at work. METHODS: Cross-sectional web-based national survey distributed to general surgeons by e‑mail over a period of 7 days. RESULTS: Among 304 responders, 42.6% were working in the hospital with COVID-19 patients. Three quarters of all surgeons (74.5%) were afraid of contracting the disease. While 42% expressed a fear for their own life while caring for COVID-19 patients, 90.1% were afraid of transmitting the disease to family members. The average reported level of PPE provided at the workplace was significantly higher among the group which was not afraid of contracting COVID-19 than among the group afraid of contracting COVID-19 (4.0 vs. 3.12, p = 0.02). Nearly all surgeons (93.8%) agreed that cancer surgeries should be continued during the pandemic and 49% perceived laparoscopy as a safe approach when operating on COVID-19 positive patients. CONCLUSION: A high proportion of surgeons admitted being afraid of working during the COVID-19 pandemic, which had various implications for their attitude towards surgical practice. Protecting health care workers is an important component of public health measures for addressing the epidemic.

Saad, N. and S. Moussa (2021). "Immune response to COVID-19 infection: a double-edged sword." Immunol Med: 1-10.

COVID-19 global pandemic has not ceased to spread worldwide since December 2019. Today, scientists and healthcare workers are urgently working to stop this viral invasion and protect the world community. Deciphering the specific cellular and molecular immune response to the new coronavirus 2019 is an essential step in order to develop effective treatment and vaccine. Recovery from COVID-19 infection was linked to appropriate immune responses. However, disease severity was correlated to impaired immune reactions. This review summarized the latest research findings on the role of immune system in fighting and also in the pathogenesis of COVID-19. In addition, it highlighted the immunological basis for the new coronavirus 2019 prevention, therapy and diagnosis.

Saadatian-Elahi, M., et al. (2020). "Protocol for a prospective, observational, hospital-based multicentre study of nosocomial SARS-CoV-2 transmission: NOSO-COR Project." BMJ Open **10**(10): e039088.

INTRODUCTION: The newly identified SARS-CoV-2 can cause serious acute respiratory infections such as pneumonia. In France, mortality rate in the general population was approximately 10% and could reach higher levels at the hospital. In the current context of high incidence rates of SARS-CoV-2 in the community, a significant increase in the rate of nosocomial transmission is expected. The risk of nosocomial transmission could even be higher in low-income countries that have fragile healthcare systems. This protocol is intended to estimate the prevalence and incidence of suspected or confirmed cases of nosocomial SARS-CoV-2 infection, the clinical spectrum and the determinants (risk factors/protective) at participating hospitals. METHODS AND ANALYSIS: This will be an international multicentre prospective, observational, hospital-based study in adults and children. It will include volunteer patients and healthcare professionals in France and hospitals affiliated with the GABRIEL network. Demographic and clinical data will be collected using case report forms designed especially for the purpose of the project. A nasopharyngeal swab will be collected and tested for SARS-CoV-2 by reverse-transcriptase PCR. Characteristics of the study participants, the proportion of confirmed nosocomial SARS-CoV-2 infections relative to all patients with syndromes suggestive of SARS-CoV-2 infection, will be analysed. Appropriate multivariate modelling will be used to identify the determinants associated with nosocomial onset. ETHICS AND DISSEMINATION: This study was approved by the clinical research and committee of all participating countries. The findings will be submitted to peer-reviewed journal for publication and shared with national health authorities. TRIAL REGISTRATION NUMBER: NCT04290780.

Sabetian, G., et al. (2021). "COVID-19 infection among healthcare workers: a cross-sectional study in southwest Iran." Virol J **18**(1): 58.

OBJECTIVE: With the novel coronavirus pandemic, the impact on the healthcare system and workers cannot be overlooked. However, studies on the infection status of medical personnel are still lacking. It is imperative to ensure the safety of health-care workers (HCWs) not only to safeguard continuous patient care but also to ensure they do not transmit the virus, therefore evaluation of infection rates in these groups are indicated. METHODS: Demographic and clinical data regarding infected cases among HCWs of Fars, Iran with positive SARS-CoV-2 PCR tests were obtained from 10th March to 17th May 2020. RESULTS: Our data demonstrated a rate of 5.62% (273 out of 4854 cases) infection among HCW, with a mean age of 35 years and a dominance of female cases (146 cases: 53.5%). The majority of infected cases were among nurses (51.3%), while the most case infection rate (CIR) was among physicians (27 positive cases out of 842 performed test (3.2%)). Also, the highest rate of infection was in the emergency rooms (30.6%). Also, 35.5% of the patients were asymptomatic and the most frequent clinical features among symptomatic patients were myalgia (46%) and cough (45.5%). Although 5.5% were admitted to hospitals, there were no reports of ICU admission. Furthermore, 10.3% of the cases reported transmitting the infection to family and friends. Regarding safety precautions, 1.6% didn't wear masks and 18.7% didn't use gloves in work environments. CONCLUSION: HCWs are among the highest groups at risk of infection during the COVID-19 pandemic; therefore, evaluating infection rates and associated features is necessary to improve and adjust protective measures of these vulnerable, yet highly essential group.

Sadhu, S., et al. (2020). "COVID-19: Limiting the Risks for Eye Care Professionals." Ocul Immunol Inflamm **28**(5): 714-720.

After the outbreak of the disease COVID-19, it has reached pandemic proportions within a very short time. It is mainly transmitted human-to-human through direct contact with secretions from an infected person or through inhalation of droplets containing SARS-CoV-2. It is controversial whether the virus may be transmitted via tears. Exposed ocular surface can serve as a gateway in transmission and acquiring respiratory diseases. Considering the reported cases on healthcare workers indicating nosocomial transmission and the anatomical and physiological aspects it is perceived that ophthalmic healthcare professionals are at higher risk of contracting the virus by virtue of their job. In this narrative review we discuss current evidence around detection of SARS-CoV-2 in human tears and forms of transmissions reported to date. We also provide a comprehensive approach that may be implemented in an ophthalmic care facility to protect healthcare personnel, as well as patients, from contracting the virus.

Saedon, H., et al. (2020). "Video Conferencing in the Intravitreal Injection Clinic in Response to the COVID-19 Pandemic." Ophthalmol Ther **9**(3): 1-6.

PURPOSE: To share a useful intervention to minimize risk of COVID-19 infection to both healthcare workers and patients in the eye clinic. METHODS: We present our experience of virtual, within-clinic remote visual acuity assessment to reduce the risk of infection with COVID-19. RESULTS: Along with standard recommendations for personal protective equipment and hand hygiene to contain viral spread and treating only urgent cases, remote within-clinic visual acuity testing and consultations can be undertaken with minimal specialist equipment and appears to provide useful information whilst being acceptable to patients. CONCLUSION: Ophthalmology practice must adapt in order to combat COVID-19. This measure can easily be incorporated into daily practice to reduce both patient footfall within the department and close contact between patient and healthcare practitioners.

Saeed, R., et al. (2021). "COVID-19 Pandemic Prevalence and Risk Factors for Depression Among Health Care Workers in South Asia." Asia Pac J Public Health: 10105395211002324.

Health care workers (HCW) are especially vulnerable to developing mental health problems in pandemic situations. The impact may be much devastating in developing countries due to fragmented health care system. Our study aims to explore the underlying burden of depression and its related factors among health care workers in South Asian countries. Data was collected through an online survey. A total of 476 health care workers including doctors, nurses, and paramedical staff participated from Pakistan, India, and Sri Lanka. A validated WHO Self Reporting Questionnaire (SRQ-20) was used to screen for Depression. A 25.7% prevalence of depression was reported among health care workers. Almost 70% of them were either not satisfied or partially satisfied with the provided personal protective equipment. On multivariable analysis, females gender (aOR=1.80, 95% CI: 1.11 - 2.90), fear of unprotection (aOR=1.80, 95% CI: 1.11 - 2.90), confirmed COVID-19 case in workplace (aOR=1.98, 95% CI= 1.18 - 3.33) and family (aOR=2.98, 95% CI: 1.02 - 8.70) were found to be independent predictors of depression among healthcare workers. Depression among HCW in South-Asian countries is quite alarming. This may jeopardize attention to other non-COVID health problems which are still on a priority in developing countries.

Saenz, L. C., et al. (2020). "Recommendations for the organization of electrophysiology and cardiac pacing services during the COVID-19 pandemic : Latin American Heart Rhythm Society (LAHRS) in collaboration with: Colombian College Of Electrophysiology, Argentinian Society of Cardiac Electrophysiology (SADEC), Brazilian Society Of Cardiac Arrhythmias (SOBRAC), Mexican Society Of Cardiac Electrophysiology (SOMEEC)." J Interv Card Electrophysiol **59**(2): 307-313.

COVID-19 is a rapidly evolving public health emergency that has largely impacted the provision of healthcare services around the world. The challenge for electrophysiology teams is double; on one side preventing disease spread by limiting all nonessential face-to-face interactions, but at the same time ensuring continued care for patients who need it. These guidelines contain recommendations regarding triaging in order to define what procedures, device checks and clinic visits can be postponed during the pandemic. We also discuss best practices to protect patients and healthcare workers and provide guidance for the management of COVID-19 patients with arrhythmic conditions.

Safdar, N., et al. (2020). "Determining the source of transmission of SARS-CoV-2 infection in a healthcare worker." medRxiv.

Healthcare workers (HCWs) are at the frontlines of the COVID-19 pandemic and are at risk of exposure to SARS-CoV-2 infection from their interactions with patients and in the community. Limited availability of recommended personal protective equipment (PPE), in particular N95 respirators, has fueled concerns about whether HCWs are adequately protected from exposure while caring for patients. Understanding the source of SARS-CoV-2 infection in a HCW - the community or the healthcare system - is critical for understanding the effectiveness of hospital infection control and PPE practices. In Dane County, Wisconsin, community prevalence of SARS-CoV-2 is relatively low (cumulative prevalence of ~0.06% - positive cases / total population in Dane county as of April 17). Although SARS-CoV-2 infections in HCWs are often presumed to be acquired during the course of patient care, there are few reports unambiguously identifying the source of acquisition. The objective of this brief report was to determine the source of transmission of SARS-CoV-2 in a healthcare worker.

Safdar, N., et al. (2020). "Using Virus Sequencing to Determine Source of SARS-CoV-2 Transmission for Healthcare Worker." Emerg Infect Dis **26**(10): 2489-2491.

Whether a healthcare worker's severe acute respiratory syndrome coronavirus 2 (SARS-CoV-2) infection is community or hospital acquired affects prevention practices. We used virus sequencing to determine that infection of a healthcare worker who cared for 2 SARS-CoV-2-infected patients was probably community acquired. Appropriate personal protective equipment may have protected against hospital-acquired infection.

Sagaon-Teyssier, L., et al. (2020). "Assessment of mental health outcomes and associated factors among workers in community-based HIV care centers in the early stage of the COVID-19 outbreak in Mali." Health Policy Open **1**: 100017.

BACKGROUND AND OBJECTIVES: In Mali, the non-governmental association (NGO) ARCAD Santé PLUS launched the CovidPrev program in response to the COVID-19 outbreak to ensure continuity of HIV care-related activities. This study aimed to identify individual and structural factors associated with mental health disorders (MHD) in the NGO's healthcare workers (HCW) in the early stage of the outbreak. METHODS: Data were collected between April 6 and 11, 2020 for 135 HCW in ARCAD Santé PLUS's 18 community-based HIV care centers. Outcomes corresponded to the PHQ-9, GAD-7 and ISI instruments for depression, anxiety and insomnia, respectively. A general mixture model with a negative binomial distribution was implemented. RESULTS: Most HCW were men (60.7%) and median age was 40 years IQR[33-46]. Symptoms of depression, anxiety and insomnia were declared by 71.9, 73.3, and 77% participants, respectively. Women were at greater risk of MHD. A lack of personal protection equipment and human resources, especially nurses, was associated with a high risk of MHD. CONCLUSIONS: Health policy must place non-front line HCW, including those in NGOs, at the core of the healthcare system response to the COVID-19 outbreak, as they ensure continuity of care for many diseases including HIV. The efficacy of public health strategies depends on the capacity of HCW to fully with and competently perform their duties.

Sahu, D. K., et al. (2020). "Smart polymeric eye gear: A possible preventive measure against ocular transmission of COVID-19." Med Hypotheses **144**: 110288.

The angiotensin-converting enzyme 2(ACE-2) receptors with approx. 0.8% congestion in conjunctival surface, leads to increase susceptibility of Covid-19 transmission through ocular surface. It has been observed that prophylactic measures such as goggle or face shield are unable to offer complete protection against ocular transmission of SRS-CoV-2. Hence, it is hypothesized that topical ocular prophylaxis using biocompatible polymers with reported in-vitro and in-vivo evidence of ACE inhibition and antiviral activity appears to be a promising strategy for preventing ocular transmission of Covid-19 to healthcare workers. They are capable of binding to ACE-2 receptors which may provide highly potential trails to block virus entry to host cells. Further biopolymers imparting antiviral activities greatly improve their protective performance. They not only provide prolong protection but also are safe for long-term use. This article discusses the description of structural and functional attributes of ACE-2 to identify appropriate polymer with better binding affinity. Furthermore, potential polymers with appropriate concentration are suggested for evaluation through a hypothesis to consider them for Covid-19 implication.

Saibene, A. M., et al. (2021). "Appropriateness for SARS-CoV-2 vaccination for otolaryngologist and head and neck surgeons in case of pregnancy, breastfeeding, or childbearing potential: Yo-IFOS and CEORL-HNS joint clinical consensus statement." Eur Arch Otorhinolaryngol: 1-9.

PURPOSE: SARS-CoV-2 vaccines are a key step in fighting the pandemic. Nevertheless, their rapid development did not allow for testing among specific population subgroups such as pregnant and breastfeeding women, or elaborating specific guidelines for healthcare personnel working in high infection risk specialties, such as otolaryngology (ORL). This clinical consensus statement (CCS) aims to offer guidance for SARS-CoV-2 vaccination to this high-risk population based on the best evidence available. METHODS: A multidisciplinary international panel of 33 specialists judged statements through a two-round modified Delphi method survey. Statements were designed to encompass the following topics: risk of SARS-Cov-2 infection and use of protective equipment in ORL; SARS-Cov-2 infection and vaccines and respective risks for the mother/child dyad; and counseling for SARS-CoV-2 vaccination in pregnant, breastfeeding, or fertile healthcare workers (PBFHW). All ORL PBFHW were considered as the target audience. RESULTS: Of the 13 statements, 7 reached consensus or strong consensus, 2 reached no consensus, and 2 reached near-consensus. According to the statements with strong consensus otorhinolaryngologists-head and neck surgeons who are pregnant, breastfeeding, or with childbearing potential should have the opportunity to receive SARS-Cov-2 vaccination. Moreover, personal protective equipment (PPE) should still be used even after the vaccination. CONCLUSION: Until prospective evaluations on these topics are available, ORL-HNS must be considered a high infection risk specialty. While the use of PPE remains pivotal, ORL PBFHW should be allowed access to SARS-CoV-2 vaccination provided they receive up-to-date information.

Saito, T. and T. Asai (2020). "Aerosol containment device for airway management of patients with COVID-19: a narrative review." J Anesth: 1-6.

Severe acute respiratory syndrome-coronavirus-2 (SARS-CoV-2), which causes coronavirus disease 2019 (COVID-19), is highly contagious. To protect healthcare workers from infection during airway management, some expert recommendations and guidelines recommended wearing P2/N95 masks, goggles or glasses, glove, face-shields, and gowns as standard personal protective equipment (PPE). Nevertheless, several simulation studies have suggested that the standard PPE may not fully protect healthcare workers. Dr. Hsien Yung Lai introduced an acrylic box ("aerosol box") as a part of PPE during airway management. Since then, several companies and healthcare workers have made their own modified devices ("aerosol containment device"), and the use of such a device has spread worldwide, without being formally assessed for its effectiveness, efficacy and safety. Several simulation studies have indicated that "aerosol containment device" would make tracheal intubation more difficult. In addition, the device would prevent the spread of droplets from a patient, but may increase the risk of healthcare workers being exposed to a higher concentration of viral aerosols. Therefore, the current state of knowledge indicates that an "aerosol containment device" without vacuum mechanism has only limited efficacy in protecting healthcare workers from viral transmission.

Salter, S. (2020). "Reinventing Cloth Masks in the Face of Pandemics." Risk Anal.

Because asymptomatic carriers of COVID-19 produce respiratory droplets that can remain suspended in air for several hours, social distancing may not be a reliable physical barrier to transmission. During the COVID-19 pandemic, however, some governments were reluctant to mandate public mask use out of concern this would worsen shortages of respirators for healthcare workers. Cloth masks with a filtering effectiveness of 70-90% can be made from widely available materials, and are a better option than respirators for the public. Countries could rapidly implement Effective Fiber Mask Programs (EFMPs) to use local resources to mass produce effective and affordable cloth masks, and to engage the public in their correct use. EFMPs could be a cost-effective measure to ease isolation while limiting new infections during pandemics. EFMPs could also protect healthcare workers by increasing the supply of respirators for their use, reducing their risk of acquiring the illness from their communities, and by reducing the number of patients they must treat.

Samaranayake, L. P., et al. (2020). "The effectiveness and efficacy of respiratory protective equipment (RPE) in dentistry and other health care settings: a systematic review." Acta Odontol Scand **78**(8): 626-639.

OBJECTIVE: The global pandemic of coronavirus disease-19, caused by the Severe Acute Respiratory Syndrome Coronavirus 2 (SARS-CoV-2), is the latest hazard facing healthcare workers (HCW) including dental care workers (DCW). It is clear that the major mode of SARS-CoV-2 transmission is the airborne route, through inhalation of virus-infested aerosols and droplets. Several respiratory protection equipment (RPE), including masks, face shields/visors, and respirators, are available to obviate facial and conjunctival contamination by microbes. However, as their barrier value against microbial inhalation has not been evaluated, we systematically reviewed the data on the effectiveness and efficacy of facemasks and respirators, including protective eyewear, with particular emphasis on dental healthcare. MATERIAL AND METHODS: PubMed, MEDLINE, the Cochrane Library, and Embase databases were searched between 01January 1990 and 15 May 2020. RESULTS: Of 310 identified English language records, 21 were included as per eligibility criteria. In clinical terms, wearing layered, face-fitting masks/respirators and protective-eyewear can limit the spread of infection among HCWs. Specifically, combined interventions such as a face mask and a face shield, better resist bioaerosol inhalation than either alone. The prolonged and over-extended use of surgical masks compromise their effectiveness. CONCLUSIONS: In general, RPE is effective as a barrier protection against aerosolized microbes in healthcare settings. But their filtration efficacy is compromised by the (i) inhalant particle size, (ii) airflow dynamics, (iii) mask-fit factor, (iv) period of wear, (v) 'wetness' of the masks, and (vi) their fabrication quality. The macro-data presented here should inform policy formulation on RPE wear amongst HCWs.

Samavedam, S. (2021). ""Physical" Aspect of COVID Nursing." Indian J Crit Care Med **25**(2): 109-110.

The use of personal protection equipment (PPE) is associated with physical and physiological derangements. Healthcare workers are at the forefront of the fight against COVID-19 and are continuously exposed to PPE. This editorial discusses a survey carried out by Jose et al. capturing the physical problems associated with PPE use. How to cite this article: Samavedam S. "Physical" Aspect of COVID Nursing. Indian J Crit Care Med 2021;25(2):109-110.

Sampson, C. S. and A. Beckett (2020). "Novel, Inexpensive Portable Respiratory Protection Unit (PRPU) for Healthcare Workers." Clin Pract Cases Emerg Med **4**(2): 126-128.

INTRODUCTION: Given concern for increased aerosolization during intubation of patients with severe acute respiratory syndrome coronavirus, we sought to create a portable, inexpensive, and easily constructed device to help protect healthcare workers. METHODS: A respiratory protection unit can be constructed in approximately 30 minutes and for less than 50 United States dollars in materials, using polyvinylchloride pipe and automobile collision wrap. CONCLUSION: This device provides possible increased protection during video laryngoscopy and can easily be replicated.

Sanchez-Betancourt, A., et al. (2020). "First surgical experience with a COVID-19 positive patient in Costa Rica: case report, staff safety protocol and brief review of literature." Surg Case Rep **6**(1): 281.

BACKGROUND: The safe management of patients with COVID-19 has been a challenge during the current pandemic, leading to healthcare workers being disproportionately affected by the virus. In Costa Rica, 20% of all infections and 27% of all ICU cases during the initial weeks of the outbreak were healthcare workers. The existing recommendations and protocols on how to care for an infected patient that requires acute surgical management have been applied successfully in various scenarios. We look to describe the first case of a COVID-19 patient that required surgical management in Costa Rica and present a summary of the protection measures utilized in a tertiary care hospital. MATERIALS AND METHODS: A review of literature utilizing Embase, Medline Complete and Google Scholar was performed. A surgical case report using the SCARE statement guidelines was drafted and a brief summary of the 54 items contained in the hospital's COVID-19 surgical protocol is described. CASE PRESENTATION: We present the case of a 29 year old obese male who had acute appendicitis with perforation that contracted SARS-CoV-2 and became symptomatic at home on pod#3, who later required multiple surgeries to address an infected abdominal hematoma both while having an active COVID-19 infection and afterwards. CONCLUSIONS: Safety measures for both staff and patients are of the utmost importance during the current coronavirus pandemic. Limitations in the availability of personal protection equipment as well a lack of knowledge and experience with handling surgical patients with this condition have led to various safety and attention protocols being drafted. The successful management of this patient is the first experience in Costa Rica on how to properly address staff safety during a surgical procedure. None of the workers involved in care of this patient were diagnosed with SARS-CoV-2.

Sánchez-Taltavull, D., et al. (2021). "Modelling strategies to organize healthcare workforce during pandemics: application to COVID-19." J Theor Biol: 110718.

Protection of the healthcare workforce is of paramount importance for the care of patients in the setting of a pandemic such as coronavirus disease 2019 (COVID-19). Healthcare workers are at increased risk of becoming infected. The ideal organisational strategy to protect the workforce in a situation in which social distancing cannot be maintained remains to be determined. In this study, we have mathematically modelled strategies for the employment of the hospital workforce with the goal of simulating the health and productivity of the workers. The models were designed to determine if desynchronization of medical teams by dichotomizing the workers may protect the workforce. Our studies model workforce productivity and the efficiency of home office applied to the case of COVID-19. The results reveal that a desynchronization strategy in which two medical teams work alternating for 7 days increases the available workforce.

Santana López, B. N., et al. (2021). "Attitudes and beliefs of Spanish healthcare professionals during the COVID-19 pandemic." Sci Prog **104**(2): 368504211003775.

During the COVID-19 pandemic, healthcare professionals are taking the risk of becoming infected or infecting their families. Spain is the country with the highest number of infected healthcare professionals worldwide. Our aim was to study the attitudes and beliefs of these professionals during the current pandemic. Descriptive study conducted by using an online questionnaire-based on an earlier one-which was sent to healthcare professionals at the national level, during the week March 20-27, 2020. Healthcare professionals returned 971 completed questionnaires. A total of 803 (82.7%) participants thought that they did not have suitable PPEs (Personal Protective Equipment) to protect them from infection with COVID-19. In addition, even 229 (23.58%) agreed to go on working even if they were not. In spite of this, 606 (62.4%) of them were ready to work, even with a higher-than-usual risk of becoming infected at work and getting ill. Remarkably high professional commitment has been observed among Spanish healthcare workers in the current pandemic. They were ready to work even when many of them considered that they did not have suitable PPEs, and were thus taking a higher than usual infection risk. However, they put the health of their relatives before their duties at work.

Santana-López, B. N., et al. (2020). "[Commitment and responsibility of health professionals in a region of Spain during the Covid-19 pandemic]." Salud Publica Mex **62**(6): 868-869.

Santos, K. O. B., et al. (2020). "Labor, health and vulnerability in the COVID-19 pandemic." Cad Saude Publica **36**(12): e00178320.

This essay discusses the repercussions of the COVID-19 pandemic on the relation between labor and health, from the perspective of the workers' risk and vulnerability. The pandemic has represented a humanitarian crisis, since both the disease and the measures to contain it lead to persistent socioeconomic effects. In this context, the labor category has an important role, either because of the feasibility of maintaining the social distancing and living conditions allowed by the employment relationship, or because of the impossibility of adopting protection strategies due to job insecurity. The essay was initially built on the basis of a literature review on the interface between COVID-19 and workers' health, carried out from December 2019 to April 2020, on the PubMed, BIREME, Cochrane Library, medRxiv and LitCovid bases, as well as using gray literature. Health professionals are more affected, but they also have greater access to diagnosis; however, data are still scarce on other professional categories, as well as on the social determinants that lead to greater labor-related vulnerability. In Brazil, the pandemic coincides with a situation in which workers accumulate significant losses of labor and social security rights, in addition to pre-existing social inequalities, such as precarious housing, with greater exposure and risk. Although the pandemic is still evolving, social inequalities are expected to intensify, with the deep retraction of the economy, and workers must be a priority target of attention in the control and spread of the disease, in addition to being the axis for planning public social and health protection policies.

Sarfraz, A., et al. (2020). "Availability, Use, and Satisfaction of Personal Protective Equipment Among Healthcare Workers: A Cross-Sectional Assessment of Low- and Middle-Income Countries." J Occup Environ Med **62**(11): e657-e664.

OBJECTIVE: To assess the discrepancy among and within low- and middle-income countries (LMICs) regarding PPE availability, use, and satisfaction. METHODS: The study population consisted of healthcare workers from LMICs who partook in the questionnaire survey from March 1, 2020, until April 15, 2020. RESULTS: In the bivariate analysis, gender (P = 0.05), HCWs (P < 0.01), and level of care (P < 0.01) were associated with the public or private sector (P < 0.05). Using multivariate analysis, PPE factors were associated with the health sector (p < 0.05). The multivariate logistic regression model determined a Pearson's χ value of 706.736 (df = 726, P = -0.689) and a c-statistic of 0.592, indicating a good model. CONCLUSION: In LMICs, huge discrepancies are present in PPE provision to HCWs, especially among the public healthcare sectors. Efforts at national and international levels ought to be addressed to protect frontline HCWs at higher risk of contracting COVID-19.

Saseedharan, S., et al. (2020). "CT Scan Findings of Five Mildly Symptomatic COVID Patients from India." J Coll Physicians Surg Pak **30**(6): 48-49.

The outbreak of the SARS-Cov-2 (Severe Acute Respiratory Syndrome - Coronavirus 2) viral pandemic which started at Wuhan China has spread to 200 countries and have resulted in a huge loss to mankind and the global economy. Computerised Tomographic (CT) scan may play an important role in the diagnosis and management of the COVID -19-the disease caused by the SARS-Cov-2. This modality might help in triaging this extremely contagious disease and thus help serve in reducing the outbreak. We present five cases that presented to the hospital with mild symptoms, where the CT scan findings were disproportionate to the symptoms thus leading to effective triage. This resulted in effective disposition in isolation ward and thus helped to protect the healthcare workers from contraction of the disease. This is especially important as the definitive diagnosis via RT-PCR (Reverse Transcriptase - Polymerase Chain Reaction) would take almost 24 hours in the present time in India. Key Words: CT scan, COVID- 19, Triage, SARS-Cov-2.

Sathiamurthy, N., et al. (2020). "Aerosol-generating procedures in thoracic surgery in the COVID-19 era in Malaysia." Asian Cardiovasc Thorac Ann **28**(8): 495-499.

BACKGROUND: The Covid-19 pandemic has caused changes in the surgical treatment of non-Covid patients, especially in thoracic surgery because most procedures are aerosol generating. Hospital Kuala Lumpur, where thoracic procedures are performed, was badly affected. We describe our experience in performing aerosol generating procedures safely in thoracic surgery during the Covid-19 era. METHODS: Medical records of patients who underwent thoracic surgery from March 18, 2020 to May 17, 2020 were reviewed retrospectively. All patients undergoing thoracic surgery were tested for Covid-19 using the reverse transcriptase polymerase chain reaction method. Patients with malignancy were observed for 10 to 14 days in the ward after testing negative. The healthcare workers donned personal protective equipment for all the cases, and the number of healthcare workers in the operating room was limited to the minimum required. RESULTS: A total of 44 procedures were performed in 26 thoracic surgeries. All of these procedures were classified as aerosol generating, and the mean duration of the surgery was 130 ± 43 minutes. None of the healthcare workers involved in the surgery were exposed or infected by Covid-19. CONCLUSION: Covid-19 will be a threat for a long time and thoracic surgeons must continue to provide their services, despite having to deal with aerosol generating procedures, in the new normal. Covid-19 testing of all surgical candidates, using the reverse transcriptase polymerase chain reaction, donning full personal protective equipment for healthcare workers, and carefully planned procedures are among the measures suggested to prevent unnecessary Covid-19 exposure in thoracic surgery.

Savoia, E., et al. (2020). "Factors associated with access and use of PPE during COVID-19: A cross-sectional study of Italian physicians." PLoS One **15**(10): e0239024.

OBJECTIVES: During the course of the Novel Coronavirus (SARS-CoV-2) pandemic, Italy has reported one of the highest number of infections. Nearly ten percent of reported coronavirus infections in Italy occurred in healthcare workers. This study aimed to understand physicians' access to personal protective equipment (PPE) and to information about their use, risk perception and strategies adopted to prevent contracting the infection. METHODS: We undertook a cross-sectional, online self-reported survey implemented between March 31 and April 5 2020 of Italian physicians. RESULTS: Responses were received from 516 physicians, only 13% of which reported to have access to PPE every time they need them. Approximately half of the physicians reported that the information received about the use of PPE was either clear (47%) or complete (54%). Risk perception about contracting the infection was influenced by receiving adequate information on the use of PPE. Access to adequate information on the use of PPE was associated with better ability to perform donning and doffing procedures [OR = 2.2 95% C.I. 1.7-2.8] and reduced perception of risk [OR = 0.5, 95% C.I. 0.4-0.6]. CONCLUSIONS: Results from this rapid survey indicate that while ramping up supplies on PPE for healthcare workers is certainly of mandatory importance, adequate training and clear instructions are just as important.

Scaggs Huang, F. and J. K. Schaffzin (2021). "Rewriting the playbook: infection prevention practices to mitigate nosocomial severe acute respiratory syndrome coronavirus 2 transmission." Curr Opin Pediatr **33**(1): 136-143.

PURPOSE OF REVIEW: Given the limited evidence and experience with severe acute respiratory syndrome coronavirus 2 (SARS-CoV-2), this novel pathogen has challenged the field of infection prevention. Despite uncertainty, infection prevention principles and experience with similar diseases have helped guide how to best protect providers and patients against disease acquisition. RECENT FINDINGS: Guidance to date has relied on data from SARS-CoV-1 and MERS-CoV to guide practices on patient isolation and personal protective equipment (PPE) use. Although a face mask and eye protection are likely adequate for most clinical scenarios, published guidelines for PPE can be confusing and conflicting. Consensus for what constitutes a high-risk aerosol-generating procedure (AGP) is lacking, but most agree providers performing procedures such as bronchoscopy, intubation, and cardiopulmonary resuscitation would likely benefit from the use of an N95 respirator and eye protection. SUMMARY: Needed research to elucidate the predominant SARS-CoV-2 mode of transmission is not likely to be completed in the immediate future. Recommendations for PPE to mitigate procedure-associated risk remain controversial. Nonetheless, implementation of existing measures based on basic infection prevention principles is likely to prevent transmission significantly.

Schilling, K., et al. (2020). "An accessible method for screening aerosol filtration identifies poor-performing commercial masks and respirators." J Expo Sci Environ Epidemiol: 1-10.

BACKGROUND: The COVID-19 pandemic has presented an acute shortage of regulation-tested masks. Many of the alternatives available to hospitals have not been certified, leaving uncertainty about their ability to properly protect healthcare workers from SARS-CoV-2 transmission. OBJECTIVE: For situations where regulatory methods are not accessible, we present experimental methods to evaluate mask filtration and breathability quickly via cost-effective approaches (e.g., ~$2000 USD) that could be replicated in communities of need without extensive infrastructure. We demonstrate the need for screening by evaluating an existing diverse inventory of masks/respirators from a local hospital. METHODS: Two experimental approaches are presented to examine both aerosol filtration and flow impedance (i.e., breathability). For one of the approaches ("quick assessment"), screening for appropriate filtration could be performed under 10 min per mask, on average. Mask fit tests were conducted in tandem but are not the focus of this study. RESULTS: Tests conducted of 47 nonregulation masks reveal variable performance. A number of commercially available masks in hospital inventories perform similarly to N95 masks for aerosol filtration of 0.2 μm and above, but there is a range of masks with relatively lower filtration efficiencies (e.g., <90%) and a subset with poorer filtration (e.g., <70%). All masks functioned acceptably for breathability, and impedance was not correlated with filtration efficiency. SIGNIFICANCE: With simplified tests, organizations with mask/respirator shortages and uncertain inventories can make informed decisions about use and procurement.

Schmitt, J., et al. (2021). "Protection Level and Reusability of a Modified Full-Face Snorkel Mask as Alternative Personal Protective Equipment for Healthcare Workers during the COVID-19 Pandemic." Chem Res Toxicol **34**(1): 110-118.

The worldwide outbreak of COVID-19 has drastically increased pressure on medical resources and highlighted the need for rapidly available, large-scale, and low-cost personal protective equipment (PPE). In this work, an alternative full-face mask is adapted from a modified snorkel mask to be used as PPE with two medical-grade filters and a 3D-printed adapter. Since the mask covers the eyes, mouth, and nose, it acts as a full-face shield, providing additional protection to healthcare workers. The SARS-CoV-2 has a size between 60 nm and 140 nm, and airborne viral particles can be carried by larger droplets with sizes up to several millimeters. The minimum filtration efficiency of mechanical and electrostatic filters is usually reached between 30 nm and 300 nm. The filtration efficiency of different medical filters is measured for particles below 300 nm to cover the size of the SARS-CoV-2 and small virus-laden droplets, and determine the minimum efficiency. The filtration performance of the adapted full-face mask is characterized using NaCl particles below 500 nm and different fitting scenarios to determine the minimum protection efficiency. The mask is compared to a commercial respirator and characterized according to the EN 149 standard, demonstrating that the protection fulfills the requirements for the FFP2 level (filtering face-piece 2, stopping at least 94% of airborne particles). The device shows a good resistance to several cycles of decontamination (autoclaving and ethanol immersion), is easy to be produced locally at low cost, and helps to address the shortage in FFP2 masks and face shields by providing adequate protection to healthcare workers against particles <500 nm in size.

Schmitz, D., et al. (2020). "Association between personal protective equipment and SARS-CoV-2 infection risk in emergency department healthcare workers." Eur J Emerg Med.

BACKGROUND AND IMPORTANCE: Healthcare personnel working in the emergency department (ED) is at risk of acquiring severe acute respiratory syndrome coronavirus-2 (SARS-Cov-2). So far, it is unknown if the reported variety in infection rates among healthcare personnel is related to the use of personal protective equipment (PPE) or other factors. OBJECTIVE: The aim of this study was to investigate the association between PPE use and SARS-CoV-2 infections among ED personnel in the Netherlands. DESIGN, SETTING AND PARTICIPANTS: A nationwide survey, consisting of 42 questions about PPE-usage, ED layout - and workflow and SARS-CoV-2 infection rates of permanent ED staff, was sent to members of the Dutch Society of Emergency Physicians. Members were asked to fill out one survey on behalf of the ED of their hospital. The association between PPE use and the infection rate was investigated using univariable and multivariable regression analyses, adjusting for potential confounders. OUTCOME MEASURES: Primary outcome was the incidence of confirmed SARS-CoV-2 infections among permanent ED staff between 1 March and 15 May 2020. RESULTS: Surveys were sent to 64 EDs of which 45 responded (70.3%). In total, 164 ED staff workers [5.1 (3.2-7.0)%] tested positive for COVID-19 during the study period compared to 0.087% of the general population. There was significant clustering of infected ED staff in some hospitals (range: 0-23 infection). In 13 hospitals, an FFP2 (filtering facepiece particles >94% aerosol filtration) mask or equivalent and eye protection was worn for all contacts with patients with suspected or confirmed SARS-CoV-2 during the whole study period. The unadjusted staff infection rate was higher in these hospitals [7.3 (3.4-11.1) vs. 4.0 (1.9-6.1)%, absolute difference + 3.3%]. Hospital staff testing policy was identified as a potential confounder of the relation between PPE use and confirmed SARS-CoV-2 infections (collinearity statistic 0.95). After adjusting for hospital testing policy, type of PPE was not associated with incidence of COVID 19 infections among ED staff (P = 0.40). CONCLUSION: In this cross-sectional study, the use of high-level PPE (FFP2 or equivalent and eye protection) by ED personnel during all contacts with patients with suspected or confirmed SARS-CoV-2 does not seem to be associated with a lower infection rate of ED staff compared to lower level PPE use. Attention should be paid to ED layout and social distancing to prevent cross-contamination of ED personnel.

Schneider, S., et al. (2020). "SARS-Coronavirus-2 cases in healthcare workers may not regularly originate from patient care: lessons from a university hospital on the underestimated risk of healthcare worker to healthcare worker transmission." Antimicrob Resist Infect Control **9**(1): 192.

BACKGROUND: Coronavirus disease 2019 (COVID-19) caused by severe acute respiratory syndrome coronavirus 2 (SARS-CoV-2) represents an unprecedented healthcare challenge. Various SARS-CoV-2 outbreaks in healthcare facilities have been reported. Healthcare workers (HCWs) may play a critical role in the spread of the virus, particularly when asymptomatic. We examined four healthcare-associated outbreaks of SARS-CoV-2 infections that occurred at a university hospital in Berlin, Germany. We aimed to describe and analyze the spread of the virus in order to draw conclusions for effective containment of SARS-CoV-2 in healthcare facilities. METHODS: Healthcare-associated outbreaks of SARS-CoV-2 infections were defined as two or more laboratory confirmed infections with SARS-CoV-2 where an epidemiological link within the healthcare setting appeared likely. We focused our analysis on one of three sites of the Charité-University Medicine hospital within a 2 month period (March and April 2020). RESULTS: We observed four healthcare-associated outbreaks of SARS-CoV-2 infections, with a total of 24 infected persons (23 HCWs and one patient). The outbreaks were detected in the departments of nephrology and dialysis (n = 9), anesthesiology (n = 8), surgical pediatrics (n = 4), and neurology (n = 3). Each outbreak showed multiple unprotected contacts between infected HCWs. A combination of contact tracing, testing, physical distancing and mandatory continuous wearing of face masks by all HCWs was able to contain all four outbreaks. CONCLUSIONS: HCW to HCW transmission represented the likely source of the four outbreaks. Ensuring proper physical distancing measures and wearing of protective equipment, also when interacting with colleagues, must be a key aspect of fighting COVID-19 in healthcare facilities.

Schnell, E., et al. (2020). "Homegrown Ultraviolet Germicidal Irradiation for Hospital-Based N95 Decontamination during the COVID-19 Pandemic." medRxiv.

Coronavirus disease (COVID-19), the disease caused by the severe acute respiratory syndrome coronavirus 2 (SARS-CoV-2) virus, is responsible for the 2020 global pandemic and characterized by high transmissibility and morbidity. Healthcare workers (HCWs) are at risk of contracting COVID-19, and this risk is mitigated through the use of personal protective equipment such as N95 Filtering Facepiece Respirators (FFRs). The high demand for FFRs is not currently met by global supply chains, potentially placing HCWs at increased exposure risk. Effective FFR decontamination modalities exist, which could maintain respiratory protection for HCWs in the midst of the current pandemic, through the decontamination and re-use of FFRs. Here, we present a locally-implemented ultraviolet-C germicidal irradiation (UVGI)-based FFR decontamination pathway, utilizing a home-built UVGI array assembled entirely with previously existing components available at our institution. We provide recommendations on the construction of similar systems, as well as guidance and strategies towards successful institutional implementation of FFR decontamination.

Schöppenthau, D., et al. (2020). "Preventing SARS-CoV-2 In-Hospital Infections in Cardiovascular Patients and Medical Staff: An Observational Study From the German Heart Center Berlin." Front Med (Lausanne) **7**: 616648.

Objective: COVID-19 is a highly contagious disease caused by severe acute respiratory syndrome coronavirus 2 (SARS-CoV-2). Preventing in-hospital infections is crucial to protect patients and hospital staff. Methods: At the very beginning of the COVID-19 pandemic, the German Heart Center initiated obligatory wearing of surgical face masks for patients and employees, SARS-CoV-2 screening for all patients, and symptom-based testing for employees. In addition, access restriction, closure of outpatient departments, and postponing non-urgent procedures were implemented with community-initiated regulations. Results: During the observation period (03/16/2020-04/27/2020), 1,128 SARS-CoV-2 tests were performed in 983 persons (1.1 tests/person; 589 in patients and 394 in hospital employees). Up to 60% of the clinical workforce was tested based on symptoms and risk (62.5% symptoms, 19.3% direct or indirect contact to known COVID-19, 4.5% returnee from risk area, 13.7% without specific reason). Patient testing for SARS-CoV-2 was obligatory (100% tested). The overall prevalence of positive tests during the observation period was 0.4% (n = 5 out of 1,128 tests performed). The incidence of new infections with SARS-CoV-2 was 0.5% (n = 5 out of 983 individuals; three healthcare workers, two patients). No nosocominal infections occurred, despite a mean number of 14.8 in-hospital contacts. Conclusion: Comprehensive SARS-CoV-2 testing and surgical face masks for patients and hospital staff, in addition to others measures, are key factors for the early detection of COVID-19 and to prevent spreading in the vulnerable hospital population.

Schultz, K., et al. (2021). "Implementation of a virtual ward as a response to the COVID-19 pandemic." Aust Health Rev.

ObjectiveThe aim of this study was to describe and evaluate the implementation of a virtual ward as a COVID-19 hospital avoidance response strategy and identify opportunities for improvement and future applicability.MethodsA mixed-method observational study was conducted of a centralised virtual ward, which operated in a large metropolitan Australian health service from 23 March to 1 June 2020.ResultsIn total, 238 unique patients were admitted to the virtual ward, accounting for 264 individual admission episodes and 2451 virtual bed days. Twenty (7.6%) episodes resulted in transfer to hospital and 136 patients provided responses to feedback surveys and reported their experience as very good (61.7%, n=87) or good (34.8%, n=49). Implementation success was high, with the model widely accepted and adopted across the health service. The service delivery model was considered to be low-cost in comparison to inpatient hospital-based care.ConclusionsOverall, as a rapidly developed and implemented low-tech model of care, the virtual ward was found to provide an effective, accessible and low-cost solution to managing low-acuity COVID-19-positive patients in the community. This model should be considered in future pandemics as a hospital-avoidance response, with the ability to minimise patient-to-healthcare worker transmission, reduce personal protective equipment use and enhance patient adherence with isolation requirements. Targeted remote telemonitoring should be considered as a future modification to improve patient care.What is known about this topic?Virtual wards aim to reduce hospital demand by providing hospital-level care in community settings such as the patients' home. The COVID-19 pandemic has seen a rapid increase in the utilisation of virtual wards as an acute healthcare response that facilitates contactless care of infectious patients. Despite this rapid adoption, there is limited literature on the effectiveness of virtual ward models of care in a pandemic context.What does this paper add?This study provides a detailed description of the implementation of a virtual ward in a large metropolitan health service. It evaluates the effectiveness of the virtual ward as a COVID-19 response strategy and identifies opportunities for improvement and future applicability. This study contributes to the growing body of literature on the COVID-19 healthcare response and virtual wards.What are the implications for practitioners?This study details the implementation of a virtual ward and highlights potential facilitators and barriers to successful implementation and sustained applicability. Findings provide a comparative benchmark for other health services implementing virtual wards as a pandemic response strategy.

Schutzer-Weissmann, J., et al. (2020). "Severe acute respiratory syndrome coronavirus 2 infection risk during elective peri-operative care: a narrative review." Anaesthesia **75**(12): 1648-1658.

The protection of healthcare workers from the risk of nosocomial severe acute respiratory syndrome coronavirus 2 (SARS-CoV-2) infection is a paramount concern. SARS-CoV-2 is likely to remain endemic and measures to protect healthcare workers against nosocomial infection will need to be maintained. This review aims to inform the assessment and management of the risk of SARS-CoV-2 transmission to healthcare workers involved in elective peri-operative care. In the absence of data specifically related to the risk of SARS-CoV-2 transmission in the peri-operative setting, we explore the evidence-base that exists regarding modes of viral transmission, historical evidence for the risk associated with aerosol-generating procedures and contemporaneous data from the COVID-19 pandemic. We identify a significant lack of data regarding the risk of transmission in the management of elective surgical patients, highlighting the urgent need for further research.

Schwartz, J., et al. (2020). "Protecting Healthcare Workers During the Coronavirus Disease 2019 (COVID-19) Outbreak: Lessons From Taiwan's Severe Acute Respiratory Syndrome Response." Clin Infect Dis **71**(15): 858-860.

During major epidemic outbreaks, demand for healthcare workers (HCWs) grows even as the extreme pressures they face cause declining availability. We draw on Taiwan's severe acute respiratory syndrome (SARS) experience to argue that a modified form of traffic control bundling (TCB) protects HCW safety and by extension strengthens overall coronavirus disease 2019 (COVID-19) epidemic control.

Schwartz, K. L., et al. (2020). "Epidemiology, clinical characteristics, household transmission, and lethality of severe acute respiratory syndrome coronavirus-2 infection among healthcare workers in Ontario, Canada." PLoS One **15**(12): e0244477.

INTRODUCTION: Protecting healthcare workers (HCWs) from Severe Acute Respiratory Syndrome Coronavirus-2 (SARS-CoV-2) is a priority to maintain a safe and functioning healthcare system. Our objective was to describe and compare the epidemiology, clinical characteristics, and lethality of SARS-CoV-2 infections among HCWs compared to non-HCWs. METHODS: Using reportable disease data at Public Health Ontario, we conducted a population-based cross-sectional study comparing demographic, exposure, and clinical variables between HCWs and non-HCWs with SARS-CoV-2 infections as of 30 September 2020. We calculated rates of infections over time and determined the frequency of within household transmissions using natural language processing based on residential address. We evaluated the risk of death using a multivariable logistic regression model adjusting for age, sex, comorbidities, symptoms, and long-term care home exposure. RESULTS: There were 7,050 (12.5%) HCW SARS-CoV-2 infections in Ontario, Canada, of whom 24.9% were nurses, 2.3% were physicians, and the remaining 72.8% other specialties, including personal support workers. Overall HCWs had an infection rate of 1,276 per 100,000 compared to non-HCWs of 346 per 100,000 (3.7 times higher). This difference decreased from a 7 times higher rate in April to no difference in September 2020. Twenty-six percent of HCWs had a household member with SARS-CoV-2 infection; 6.8% were probable acquisitions, 12.3% secondary transmissions, and 6.9% unknown direction of transmission. Death among HCWs was 0.2% compared to 6.1% of non-HCWs. The risk of death in HCWs remained significantly lower than non-HCWs after adjustment (adjusted odds ratio 0.09; 95%CI 0.05-0.17). CONCLUSION: HCWs represent a disproportionate number of diagnosed SARS-CoV-2 infections in Ontario, however this discrepancy is at least partially explained by limitations in testing earlier in the pandemic for non-HCWs. We observed a low risk of death in HCWs which could not be completely explained by other factors.

Seale, H., et al. (2020). "Improving the impact of non-pharmaceutical interventions during COVID-19: examining the factors that influence engagement and the impact on individuals." BMC Infect Dis **20**(1): 607.

BACKGROUND: During an evolving outbreak or pandemic, non-pharmaceutical interventions (NPIs) including physical distancing, isolation, and mask use may flatten the peak in communities. However, these strategies rely on community understanding and motivation to engage to ensure appropriate compliance and impact. To support current activities for COVID-19, the objectives of this narrative review was to identify the key determinants impacting on engagement. METHODS: An integrative narrative literature review focused on NPIs. We aimed to identify published peer-reviewed articles that focused on the general community (excluding healthcare workers), NPIs (including school closure, quarantine, isolation, physical distancing and hygiene behaviours), and factors/characteristics (including social, physical, psychological, capacity, motivation, economic and demographic) that impact on engagement. RESULTS: The results revealed that there are a range of demographic, social and psychological factors underpinning engagement with quarantine, school closures, and personal protective behaviours. Aside from the factors impacting on acceptance and compliance, there are several key community concerns about their use that need to be addressed including the potential for economic consequences. CONCLUSION: It is important that we acknowledge that these strategies will have an impact on an individual and the community. By understanding the barriers, we can identify what strategies need to be adopted to motivate individuals and improve community compliance. Using a behavioural framework to plan interventions based on these key barriers, will also ensure countries implement appropriate and targeted responses.

See, A., et al. (2021). "Adaptations of a Tertiary Otorhinolaryngology Head and Neck Surgery Department in Singapore during the COVID-19 Outbreak." Ann Otol Rhinol Laryngol **130**(2): 177-181.

PURPOSE: The novel coronavirus 2019 (COVID-19) outbreak which was first reported in Wuhan, China has been declared a pandemic by the World Health Organization on March 11, 2020. Otorhinolaryngologists deal intimately with pathologies of the head and neck region and upper respiratory tract and have been reported as a vulnerable group of healthcare workers who may be more susceptible to COVID-19 nosocomial infection. METHODS: In this article, we provide a comprehensive overview of the adaptations of Singapore's largest tertiary Otorhinolaryngology department during the COVID-19 outbreak. This was undertaken via an evidence-based approach. The relevant medical literature and evidence underlying our adaptations are highlighted. RESULTS: A four-pronged strategy including (1) personnel segregation, (2) triaging and decantment, (3) use of personal protective equipment and (4) changes in clinical practice was employed. The strategy was bolstered by drawing upon a collective learnt experience from the 2003 Severe Acute Respiratory Syndrome (SARS) outbreak. CONCLUSION: A rigorous framework which can preserve operationality while navigating the heightened risks during this outbreak is critical for every Otorhinolaryngology department. As the pandemic continues to evolve and more scientific reports of this disease are made available, approaches will need to be morphed.

Selhorst, P., et al. (2020). "Symptomatic SARS-CoV-2 reinfection of a health care worker in a Belgian nosocomial outbreak despite primary neutralizing antibody response." Clin Infect Dis.

BACKGROUND: It is currently unclear whether SARS-CoV-2 reinfection will remain a rare event, only occurring in individuals who fail to mount an effective immune response, or whether it will occur more frequently when humoral immunity wanes following primary infection. METHODS: A case of reinfection was observed in a Belgian nosocomial outbreak involving 3 patients and 2 health care workers. To distinguish reinfection from persistent infection and detect potential transmission clusters, whole genome sequencing was performed on nasopharyngeal swabs of all individuals including the reinfection case's first episode. IgA, IgM, and IgG and neutralizing antibody responses were quantified in serum of all individuals, and viral infectiousness was measured in the swabs of the reinfection case. RESULTS: Reinfection was confirmed in a young, immunocompetent health care worker as viral genomes derived from the first and second episode belonged to different SARS-CoV-2 clades. The symptomatic reinfection occurred after an interval of 185 days, despite the development of an effective humoral immune response following symptomatic primary infection. The second episode, however, was milder and characterized by a fast rise in serum IgG and neutralizing antibodies. Although contact tracing and virus culture remained inconclusive, the health care worker formed a transmission cluster with 3 patients and showed evidence of virus replication but not of neutralizing antibodies in her nasopharyngeal swabs. CONCLUSION: If this case is representative of most Covid-19 patients, long-lived protective immunity against SARS-CoV-2 after primary infection might not be likely.

Seligman, H., et al. (2020). "Management of Acute Coronary Syndromes During the Coronavirus Disease 2019 Pandemic: Deviations from Guidelines and Pragmatic Considerations for Patients and Healthcare Workers." Interv Cardiol **15**: e16.

Coronavirus disease 2019 (COVID-19) is forcing cardiology departments to rapidly adapt existing clinical guidelines to a new reality and this is especially the case for acute coronary syndrome pathways. In this focused review, the authors discuss how COVID-19 is affecting acute cardiology care and propose pragmatic guideline modifications for the diagnosis and management of acute coronary syndrome patients, particularly around the appropriateness of invasive strategies as well as length of hospital stay. The authors also discuss the use of personal protective equipment for healthcare workers in cardiology. Based on shared global experiences and growing peer-reviewed literature, it is possible to put in place modified acute coronary syndrome treatment pathways to offer safe pragmatic decisions to patients and staff.

Seligman, H., et al. (2021). "Reusable snorkel masks adapted as particulate respirators." PLoS One **16**(4): e0249201.

INTRODUCTION: During viral pandemics, filtering facepiece (FFP) masks together with eye protection form the essential components of personal protective equipment (PPE) for healthcare workers. There remain concerns regarding insufficient global supply and imperfect protection offered by currently available PPE strategies. A range of full-face snorkel masks were adapted to accept high grade medical respiratory filters using bespoke-designed 3D-printed connectors. We compared the protection offered by the snorkel to that of standard PPE using a placebo-controlled respirator filtering test as well as a fluorescent droplet deposition experiment. Out of the 56 subjects tested, 42 (75%) passed filtering testing with the snorkel mask compared to 31 (55%) with a FFP3 respirator mask (p = 0.003). Amongst the 43 subjects who were not excluded following a placebo control, 85% passed filtering testing with the snorkel versus to 68% with a FFP3 mask (p = 0.008). Following front and lateral spray of fluorescence liquid particles, the snorkel mask also provided superior protection against droplet deposition within the subject's face, when compared to a standard PPE combination of FFP3 masks and eye protection (3.19x108 versus 6.81x108 fluorescence units, p<0.001). The 3D printable adaptors are available for free download online at https://www.ImperialHackspace.com/COVID-19-Snorkel-Respirator-Project/. CONCLUSION: Full-face snorkel masks adapted as particulate respirators performed better than a standard PPE combination of FFP3 mask and eye protection against aerosol inhalation and droplet deposition. This adaptation is therefore a promising PPE solution for healthcare workers during highly contagious viral outbreaks.

Selvakumar, K. and S. Lokesh (2021). "The prediction of the lifetime of the new coronavirus in the USA using mathematical models." Soft comput: 1-20.

The World Health Organization (WHO) on December 31, 2019, was informed of several cases of respiratory diseases of unknown origin in the city of Wuhan in the Chinese Province of Hubei, the clinical manifestations of which were similar to those of viral pneumonia and manifested as fever, cough, and shortness of breath. And, the disease caused by the virus is named the new coronavirus disease 2019 and it will be abbreviated as 2019-nCoV and COVID-19. As of January 30, 2020, the WHO classified this epidemic as a global health emergency (Chung et al. in Radiology 295(1):202-207, 2020). It is an international real-life problem. Due to deaths, globally everyone is under fear. Now, it is the responsibility of researchers to give hope to the people. In this article, we aim to better protect people and general pandemic preparedness by predicting the lifetime of the disease-causing virus using three mathematical models. This article deals with a complex real-life problem people face all over the world, an international real-life problem. The main focus is on the USA due to large infection and death due to coronavirus and thereby the life of every individual is uncertain. The death counts of the USA from February 29 to April 22, 2020, are used in this article as a data set. The death counts of the USA are fitted by the solutions of three mathematical models and a solution to an international problem is achieved. Based on the death rate, the lifetime of the coronavirus COVID-19 is predicted as 1464.76 days from February 29, 2020. That is, after March 2024 there will be no death in the USA due to COVID-19 if everyone follows the guidelines of WHO and the advice of healthcare workers. People and government can get prepared for this situation and many lives can be saved. It is the contribution of soft computing. Finally, this article suggests several steps to control the spread and severity of the disease. The research work, the lifetime prediction presented in this article is entirely new and differs from all other articles in the literature.

Senent-Boza, A., et al. (2020). "Early implementation of protective measures defines surgical outcomes in the COVID-19 pandemic." Surg Today **50**(9): 1107-1112.

Quick implementation of specific protocols and protective measures in a tertiary hospital in Spain allowed for the early diagnosis and optimal management of patients with SARS-CoV-2 infection and proper protection of staff and inpatients. From the COVID-19 outbreak in this country until the time of writing, 14 patients in our hospital underwent surgery with COVID-19, or COVID-19 developed postoperatively. Their postoperative outcomes did not differ from those in our routine clinical practice, with a 0% respiratory failure rate and a 7.14% mortality rate, in contrast with other published series. COVID-19 did not develop in any of the healthcare workers present in the operating room during these procedures or in those who cared for these patients on the ward.

Seow, J., et al. (2020). "Longitudinal observation and decline of neutralizing antibody responses in the three months following SARS-CoV-2 infection in humans." Nat Microbiol **5**(12): 1598-1607.

Antibody responses to SARS-CoV-2 can be detected in most infected individuals 10-15 d after the onset of COVID-19 symptoms. However, due to the recent emergence of SARS-CoV-2 in the human population, it is not known how long antibody responses will be maintained or whether they will provide protection from reinfection. Using sequential serum samples collected up to 94 d post onset of symptoms (POS) from 65 individuals with real-time quantitative PCR-confirmed SARS-CoV-2 infection, we show seroconversion (immunoglobulin (Ig)M, IgA, IgG) in >95% of cases and neutralizing antibody responses when sampled beyond 8 d POS. We show that the kinetics of the neutralizing antibody response is typical of an acute viral infection, with declining neutralizing antibody titres observed after an initial peak, and that the magnitude of this peak is dependent on disease severity. Although some individuals with high peak infective dose (ID(50) > 10,000) maintained neutralizing antibody titres >1,000 at >60 d POS, some with lower peak ID(50) had neutralizing antibody titres approaching baseline within the follow-up period. A similar decline in neutralizing antibody titres was observed in a cohort of 31 seropositive healthcare workers. The present study has important implications when considering widespread serological testing and antibody protection against reinfection with SARS-CoV-2, and may suggest that vaccine boosters are required to provide long-lasting protection.

Serrão, C., et al. (2021). "Burnout and Depression in Portuguese Healthcare Workers during the COVID-19 Pandemic-The Mediating Role of Psychological Resilience." Int J Environ Res Public Health **18**(2).

During the COVID-19 pandemic, healthcare workers (HCW) have been exposed to multiple psychosocial stressors. Resilience might protect employees from the negative consequences of chronic stress. The aim of this study was to explore the mediating role of resilience in the relationship between depression and burnout (personal, work-related, and client-related). A cross-sectional study was performed using an online questionnaire distributed via social networks. A survey was conducted comprising standardized measures of resilience (Resilience Scale-25 items), depression (subscale of Depression Anxiety Stress Scales-21 items), and burnout (Copenhagen Burnout Inventory Scale-19 items). A total of 2008 subjects completed the survey, and a hierarchical regression model was estimated for each burnout dimension. The results revealed that depression had not only a directed effect on personal, work- and client-related burnout, but also an indirect small effect on it through resilience. Psychological resilience played a partial mediating role between depression and all burnout dimensions. This partial mediation suggests that there may be other possible variables (e.g., social connection, self-compassion, gratitude, sense of purpose) that further explain the associations.

Setiawati, Y., et al. (2021). "Anxiety and Resilience of Healthcare Workers During COVID-19 Pandemic in Indonesia." J Multidiscip Healthc **14**: 1-8.

INTRODUCTION: The COVID-19 pandemic has an impact on the physical health and mental health of the community, including healthcare workers. Several studies have shown symptoms of depression, anxiety, and sleep disorders in healthcare workers during this pandemic. However, not many studies have examined the resilience of healthcare workers during this pandemic. Resilience is a person's ability to rise and adapt when times are difficult and is considered to have a protective effect on mental problems. PURPOSE: This study aims to determine the correlation between resilience and anxiety in healthcare workers during COVID-19 pandemic. MATERIALS AND METHODS: This research was a cross-sectional study with observational analytic methods. The respondents were healthcare workers at Dr. Soetomo Hospital as the COVID-19 referral hospital in Surabaya, East Java, Indonesia. Data were collected from 10 to 16 June 2020 by distributing online questionnaires through the Google form application. There were three questionnaires used: demographic data, the State-Trait Anxiety Inventory (STAI) questionnaire, and the Connor-Davidson Resilience Scale (CR-RISC) questionnaire. RESULTS: The 227 respondents had filled out the questionnaire online with 33% had high state anxiety and 26.9% had high trait anxiety. The mean score of the respondents' resilience was 69 ± 15.823. The Spearman correlation test showed a significant relationship between anxiety and resilience (p <0.05), both S-Anxiety and T-Anxiety. CONCLUSION: A significant correlation was found between the level of resilience and anxiety experienced by healthcare workers during the COVID-19 pandemic. The lower the resilience, the higher the anxiety experienced.

Shah, V., et al. (2021). "Extra Precautions while Caring for a Suspected COVID-19 Patient in an ICU beyond PPE and Hand Hygiene." Indian J Crit Care Med **25**(3): 331-336.

Since the diagnosis of the first case of COVID-19 in December 2019, there have been reports of several healthcare workers infected with COVID-19. It has changed the infection control practices of most ICUs all over the world. Prevention is better than cure is definitely proven true as there is no definite cure for COVID-19 yet. Personal protective equipment and hand hygiene are a must while handling any suspected COVID-19 patient. Apart from that, there are several other things, which should be followed in ICU and specifically while caring for a patient on ventilator. There are a large number of interventions done while treating any critically ill patient in ICU, which can generate aerosols and exaggerate spread of COVID-19, which include high-flow nasal cannula, NIV, nebulization, suctioning, bag and mask ventilation, endotracheal intubation, and bronchoscopy. We will be reviewing those things beyond PPE and hand hygiene along with the rationale of each of them, which can help to minimize the risk of exposure to healthcare workers and other patients in the surrounding. It will help not only to prevent COVID-19 transmission but also to reduce overall nosocomial infection rate. As per our knowledge, this will be the first paper reviewing innovative ideas to minimize the risk of infection in a comprehensive manner. How to cite this article: Shah V, Tyagi N, Trivedi D. Extra Precautions while Caring for a Suspected COVID-19 Patient in an ICU beyond PPE and Hand Hygiene. Indian J Crit Care Med 2021;25(3):331-336.

Shaheen, S., et al. (2020). "Rapid guide to the management of cardiac patients during the COVID-19 pandemic in Egypt: "a position statement of the Egyptian Society of Cardiology"." Egypt Heart J **72**(1): 30.

COVID-19 pandemic poses an enormous challenge to healthcare system in Egypt. This document is a position statement from the Egyptian Society of Cardiology. It aims to provide information to cardiovascular healthcare providers in Egypt to guarantee delivery of quality patient care and ensure adequate levels of protection against infection during the COVID-19 pandemic. Older patients and those with cardiovascular disease are at higher risk of mortality. The current situation requires unusual allocation of resources which may negatively impact the care of patients with cardiovascular disease. Cardiologists should be prepared in the COVID-19 pandemic. The challenge is in providing the best quality of care despite limited resources while keeping all medical staff as safe as possible. Consider deferring elective procedures whenever possible. All medical staff should undergo rigorous training on infection control and the use of high-quality personal protection equipment. Cardiologists should promote telemedicine in the outpatient setting, prioritize outpatient contacts, and avoid nosocomial dissemination of the virus to patients and healthcare providers. A much conservative approach for emergent cardiac patients is recommended, and invasive interventions are reserved for high risk hemodynamically unstable patients. During the pandemic, the most important principles of treatment should be controlling the spread of infection as the first priority, prompt assessment of patient risk, recommending conservative medical therapy rather than invasive interventions, and strict infection control measures to limit infection spread within the hospital and to healthcare workers.

Shahid, H., et al. (2020). "COVID-19 and Its Psychological Impacts on Healthcare Staff - A Multi-Centric Comparative Cross-Sectional Study." Cureus **12**(11): e11753.

Background Since the first case of coronavirus disease-19 (COVID-19) in Pakistan was reported in February 2020, the medical and paramedical staff has been working on the frontlines to deal with this disease. They have been facing significant strain and stress due to the pandemic, affecting their social, mental, and personal life. The purpose of this study is to investigate the psychological effects of the COVID-19 pandemic, etiology, personal coping mechanisms, and the strategies that are being adopted to reduce stress by the healthcare workers (HCWs) working in COVID-19 dedicated wards (group 2) and compare it with staff working in other departments but not in COVID-19 wards amid this pandemic (group 1) in various hospitals of Lahore, Pakistan. Methods The comparative cross-sectional study was designed which included doctors, nurses, and allied health professionals from various hospitals of Lahore, Pakistan. A questionnaire was designed which consisted of five sections, and 51 questions. A Chi-square test was used to compare the responses between these two groups. Results The study questionnaire was submitted by 200 participants, 100 responses for each group (see the Appendix). In group 1, HCWs not working in COVID-19 dedicated floors were afraid of getting infected, transmitting the infection to their families and concerned about using personal protective equipment (PPE) improperly. They reported a lack of concentration and tense muscles. The coping mechanisms of this group were exercise, strict precautions at work, and social distancing measures. While HCWs serving in COVID-19 dedicated wards were concerned and afraid of putting their families at risk by working in the high-risk environment; the major stresses in this group were: lack of knowledge about proper strategies for treatment, they faced insecurity due to physical and verbal violence by caretakers of COVID-19 patients, and lack of concentration. The coping mechanism was the support of their families and taking strict precautions, with self-isolation if required, to avoid any disease transmission to their families. The proposed strategies to be implemented included teaching skills for self-rescue as well as the implementation of policies at the administrative level to reduce working hours and frequent shift rotation. Conclusion The COVID-19 outbreak posed a great deal of mental stress among HCWs working on the COVID-19 floor as well as those serving in other departments of the hospital. The HCWs from group 1 were most afraid of getting infected and putting family members at risk, experienced tense muscles and lack of concentration, coped their stress by exercise and being more vigilant, and suggested the strategies of teaching skills for self-rescue and better community awareness. While the staff from the second group were most afraid of being the source of infection and violence from the caretakers of patients, experienced tense muscles, used family support, and strict isolation measures as coping mechanisms and suggested the strategies of self-rescue and increase in wages of directly exposed healthcare workers to deal with such pandemics in future in a better way.

Shahriarirad, R., et al. (2021). "The mental health impact of COVID-19 outbreak: a Nationwide Survey in Iran." Int J Ment Health Syst **15**(1): 19.

BACKGROUND: Disease outbreak not only carries the risk of death to the public due to the infection, but it also can lead to unbearable psychological impact on the mental health of the individuals. This study aims to explore and evaluate the burden of psychological problems on the Iranian general population during the outbreak of COVID-19. METHOD: A cross-sectional web-based survey was conducted among the general population of Iran age 15 and above. Demographic variables, depression, and anxiety symptoms were evaluated using the Patient Health Questionnaire-9 and General Anxiety Disorder-7 questionnaires. RESULTS: Among the 8591 participants, the mean age was 34.37 (± 11.25) years and 66.4% were female while 33.6% were male. Based on our results, 1295 (15.1%) and 1733 (20.1%) of the general population had clinically significant depressive and anxiety symptoms, respectively. Based on the demographic variables, female gender was associated with a higher risk for developing depression and anxiety symptoms, whereas getting information about the disease from medical journals and articles, being older, and being married were considered as associated protective factors. In terms of depression, being a healthcare worker was an associated risk factor. On the other hand, for anxiety, having higher education was a protective factor while a higher number of individuals in a household was considered as a risk factor. CONCLUSIONS: This study identified a major mental health problem in the Iranian population during the time of the COVID-19 outbreak. Therefore, establishing a targeted mental health support program during the time of public emergencies, such as the disease outbreak, is advised.

Shang, J., et al. (2021). "The state of infection prevention and control at home health agencies in the United States prior to COVID-19: A cross-sectional study." Int J Nurs Stud **115**: 103841.

BACKGROUND: Home health care is a rapidly growing healthcare sector worldwide. Home health professionals face unique challenges related to preventing and controlling infections, which are likely to amplify during an infectious disease outbreak (e.g. SARS-CoV-2). Little is known about the current state of infection prevention and control-related policies and outbreak preparedness at U.S. home health agencies. OBJECTIVES: In this study, we conducted a national survey to assess infection prevention and control-related policies, infrastructure, and procedures prior to the SARS-CoV-2 pandemic. DESIGN: Cross-sectional study. SETTING/PARTICIPANTS: Using a stratified random sample of 1506 U.S. home health agencies, we conducted a 61-item survey (paper and online) from November 9, 2018 to December 31, 2019. METHODS: Survey data were linked to publicly-available data on the quality of patient care, patient satisfaction, and other agency characteristics. Probability weights were developed to account for sample design and nonresponse; Pearson's χ2, Fisher's exact, t-tests or linear regression were used to compare the universe of agencies/respondents and urban/rural agencies. RESULTS: 35.6% of agencies responded (n = 536). Most home health personnel in charge of infection prevention and control have other responsibilities; one-third have no formal infection prevention and control training. Rural agencies are more likely to not have anyone in charge of infection prevention and control compared to those in urban areas. About 22% of agencies implement recommended guidelines when administering antibiotics. Less than a third (26.4%) report that their staff vaccination rates were higher than 95% during the last flu season. Only 48.1% of agencies accept patients requiring ventilation, and of those, 40.9% located in rural areas do not have specific infection prevention and control policies for ventilated patients, compared to 20.8% in urban areas (p < 0.001). Only 39.7% of agencies provide N95 respirators to their clinical staff; rural agencies are significantly more likely to provide those supplies than urban agencies (50.7% vs. 37.7%, p = 0.004). Lastly, agencies report their greatest challenges with infection prevention and control are collecting/reporting infection data and adherence to/monitoring of nursing bag technique. CONCLUSIONS: Prior to the SARS-CoV-2 pandemic, we found that infection prevention and control was suboptimal among U.S. home health care agencies. Consequently, most agencies have limited capacity to respond to infectious disease outbreaks. Staff and personal protective equipment shortages remain major concerns, and agencies will need to quickly adjust their existing infection prevention and control policies and potentially create new ones. In the long-term, agencies also need to improve influenza vaccination coverage among their staff. Tweetable abstract: Infection prevention and control infrastructure, policies and procedures and outbreak preparedness at U.S. home health agencies was found to be suboptimal in nationally-representative survey conducted just prior to the COVID-19 pandemic.

Sharaf, R. F. and N. Kabel (2021). "Awareness and knowledge of undergraduate dental students about the signs and symptoms of Corona viral infection (COVID-19), and the required infection control measures to prevent its spread." Bull Natl Res Cent **45**(1): 32.

BACKGROUND: Coronavirus disease (COVID-19) is considered a highly contagious disease with flu-like symptoms and causing relatively high level of death. It can be transmitted from a person to another through droplets and that makes the dentists at high risk of infection. Therefore, the aim of the current study was to assess the awareness and knowledge of dental students about the signs and symptoms of Coronavirus disease (COVID-19) and to evaluate their awareness about the required infection control measures during the dental treatment to control the spread of the disease. A questionnaire was formed using Google forms and distributed among dental students and interns in different universities in Cairo, Egypt. Questions were about signs and symptoms of COVID-19, attitude of the dentists toward dental treatment of suspected patients and the required personal protective equipment (PPE) and infection control measures at the dental clinic. RESULTS: The majority of the participants strongly agreed/agreed that COVID-19 is a highly dangerous disease, Participants reported that the most common symptom is difficulty in breathing (89.4%) followed by fever (84.4%). Face shield was the most recommended personal protective equipment (PPE) during dental treatment (98.6%) followed by disposable gown (96.3%). The majority of participants (84.8%) recommended using 70% ethyl alcohol as the first method to disinfect surfaces in between dental visits, followed by sodium hypochlorite. CONCLUSIONS: Dental students and interns in Cairo, Egypt, have good knowledge and awareness about COVID-19 and the necessary precautions required to provide adequate dental treatment for the patients during the pandemic COVID-19; however, the importance of infection control should be highlighted for both clinical and preclinical dental students, to provide safe dental treatment to the patients as well as protection of the dentists and healthcare workers.

Sharma, A. A., et al. (2021). "A call to action: why medical education curriculum needs to encourage young physicians to innovate." Arch Dermatol Res: 1-3.

Patient-centered and physician-led innovations are key to promoting physicians as visionary leaders in the healthcare system especially during times of crises. COVID-19 has inspired some promising recent advancements within medicine worth noting, including improvements in telemedicine, 3-D printed personal protective equipment (PPE) and ventilators, drug and vaccine development, sterilization of PPE allowing for reuse, and point of care testing; they highlight a broader lesson for how we might innovate better within medicine, even after the crisis has passed. As such, with the complexities of modern-day medicine, to continue to foster this culture of innovation, it is paramount that going forward, medical education adapt and embrace an innovation curriculum that prepares physicians and healthcare workers to work with their communities and researchers to confidently tackle any challenges that may present. Integrating innovation into our careers and medical training is important for advancement of the field and to be able to handle challenges that may present to the healthcare system.

Sharma, K., et al. (2021). "Impact of COVID-19 Pandemic and Pattern of Patient Care in Otorhinolaryngology Practice in a Tertiary Referral Centre." Indian J Otolaryngol Head Neck Surg: 1-8.

To study the effect of COVID-19 pandemic on patient load in a tertiary care centre and the innovations and methods used to improve the safety of the healthcare workers, to provide adequate treatment in the department of Otorhinolaryngology. This study was conducted in the Department of Otorhinolaryngology and Head and Neck Surgery, at a tertiary care hospital centre in North-East India. This study included data collected from the patient registers maintained in our department, and included data over a 4-month period, from April, 2020 to July, 2020. Age, gender, place of residence, clinical diagnosis and the operative procedure performed were included in the data profile for analysis. The above-mentioned registers were also reviewed to retrieve details about the rate of admission during the study period in the previous year. Data was collected and represented, in both descriptive and tabular forms, after proper statistical analysis. We found out that there is a drastic reduction in number of patients attending in our department of Otorhinolaryngology during this COVID-19 pandemic. Certain innovative methods for protecting healthcare workers from viral transmission were put into our practice based on the peer reviewed articles, from June, 2020 and the rate of elective procedures and in-patient admissions were thus increased. Knowledge of new innovative methods in Otorhinolaryngology will help overcome the difficulties faced during the current COVID-19 pandemic.

Shavit, D., et al. (2020). "Assessment of Alternative Personal Protective Equipment by Emergency Department Personnel During the SARS-CoV-2 Pandemic: A Simulation-Based Pilot Study." Simul Healthc **15**(6): 445-446.

Shortage of personal protective equipment (PPE) for frontline healthcare workers managing the current severe acute respiratory syndrome coronavirus 2 (SARS-CoV-2) pandemic is a major, global challenge. In this pilot study, we describe a simulation-based method for evaluating the suitability and acceptability of an alternative biological isolation garment (BIG, a gown or a suit) for clinical use by emergency department (ED) personnel. Using a high-fidelity simulator, participants provided airway management according to the SARS-CoV-2 protocol. A nonvisible fluorescent marker was used as a surrogate marker of contamination. We assessed ultraviolet light visualization of the fluorescent marker after doffing and satisfaction with donning, use during simulation, and doffing. We found that after doffing, markers were not visualized on any of the participants and that the median satisfaction scores of the alternative and standard BIG (sBIG) were 4 [interquartile range (IQR) = 1-5] and 4 (IQR = 2-4), respectively. The results suggest the suitability and acceptability of the alternative BIG (aBIG) for use by ED personnel.

Shen, B., et al. (2020). "Wuchang Fangcang Shelter Hospital: Practices, Experiences, and Lessons Learned in Controlling COVID-19." SN Compr Clin Med: 1-6.

In early January 2020, the outbreak of the new corona virus pneumonia (Corona Virus Disease 2019, COVID-19) occurred. Wuhan, the capital city of Hubei province, became the epicenter of the disease in China. The rapid growth of patients had exceeded the maximum affordability of local medical resources. A large comprehensive gymnasium was converted into Wuchang Fangcang Shelter Hospital in order to provide adequate medical beds and appropriate care for the confirmed patients with mild to moderate symptoms. For these hospitalized patients with COVID-19, medication became the mainstay of therapy. From 5th February to 10th March, a team of pharmacists successfully completed drug supplies and pharmaceutical services for 1124 patients and approximately 800 medical staff, and, while doing so, received zero complaint, and experienced zero disputes and zero pharmacist infection. This paper summarizes the development and construction of the pharmacy, human resource allocation of pharmacists, pharmacy administration, and pharmaceutical services. It aims to review a 34-day period of pharmaceutical practice and serve as a reference for other health professionals working on COVID-19 prevention and treatment in other regions.

Sier, R. S. and O. I. Onugha (2021). "Tracheostomy and Improvement in Utilization of Hospital Resources During SARS-CoV-2 Pandemic Surge." Surg Technol Int **38**.

The SARS-CoV-2 pandemic has affected millions across the world. Significant patient surges have caused severe resource allocation challenges in personal protective equipment, medications, and staffing. The virus produces bilateral lung infiltrates causing significant oxygen depletion and respiratory failure thus increasing the need for ventilators. The patients who require ventilation are often requiring prolonged ventilation and depleting hospital resources. Tracheostomy is often utilized in patients requiring prolonged ventilation, and early tracheostomy in critical care patients has been shown in some studies to improve a variety of factors including intensive care unit (ICU) length of stay, ventilation weaning, and decreased sedation medication utilization. In a patient surge setting, as long as adequate personal protective equipment (PPE) is available to minimize spread to healthcare workers, early tracheostomy may be a beneficial management of these patients. Decreasing sedative medication utilization may help prevent shortages in future waves of infection and improve patient-provider communication as patients are more alert. Tracheostomy care is easier than endotracheal intubation and may have decreased viral aerosolization risk, particularly if repeat intubation is necessary after a weaning trial. Additionally, tracheostomy patients can be monitored with less staff, decreasing total healthcare worker exposure to infection. To manage risk of exposure, coordination of ventilation controlled by an anesthesiologist or a critical care physician with a surgeon during the procedure can minimize aerosolization to the team. Risk management and resource allocation is of the utmost importance in any global crisis and procedures must be appropriately planned and benefits to patients, as well as minimized exposure to healthcare providers, must be considered. Early tracheostomy could be a beneficial procedure for severe SARS-CoV-2 patients to minimize long-term virus aerosolization and exposure for healthcare workers while decreasing sedation, allowing for earlier transfer out of the ICU, and improving hospital resource utilization.

Sigahi, T., et al. (2021). "A systematic review on the impacts of Covid-19 on work: Contributions and a path forward from the perspectives of ergonomics and psychodynamics of work." Hum Factors Ergon Manuf.

Work is a major issue in the discussions on the Covid-19 pandemic, whose consequences impair the capacities of workers at all organizational levels and impose unexpected challenges on managers. Nevertheless, the scientific literature still lacks an overview of how researchers have been covering the issue. This article presents a systematic review to investigate the impact of Covid-19 on work and workers of all occupations, reveal research gaps, and help managers to adapt to organizations amid the pandemic. This review is based on a sample of 258 papers from Web of Science and Scopus databases. Quantitative and qualitative analysis indicated a vast majority of studies focused on frontline healthcare workers and a lack of empirical investigation on managers' work. We show how literature has been addressing different aspects of work (e.g., workers' health, working conditions, procedures, protection equipment, remote working, etc.) in the various economic sectors. We discuss the results in light of ergonomics and psychodynamics of work, two disciplines that conceive work and organization as intimately connected, and that can assist managers in meeting the difficulties brought by the pandemic. We highlight that considering the construction of health, interindividual variability, subjective relation to work, supportive workplace environment, and organizational and individual capabilities can play a key role in successfully adapting and transforming organizations in the current scenario.

Signorelli, C. and G. M. Fara (2020). "COVID-19: Hygiene and Public Health to the front." Acta Biomed **91**(3-s): 7-8.

Public Health professionals and academics have been on the frontline of Italian history during the COVID-19 response like they never did before. Ancient professors of Hygiene such as Celli, Pagliani, Sclavo, Petragnani, Seppilli and Giovanardi flanked politicians in other critical moments. They helped them to manage healthcare reforms, earthquakes response, Seveso Dioxin disaster, cholera and poliomielitis epidemics and other health threats.   The ongoing COVID-19 epidemic has highlighted the paramount importance of the practical application of basic concepts of public health, which were considered so far became obsolete, such as personal hygiene, quarantine, individual protective devices or basic epidemiological measures. Hygiene and Public Health used to have a targeted audience in professionals and lecturers. Nowadays, these topics are critical and of concern of a much larger audience. Public Health women and men are now asked to act in task forces, media broadcasts, webinars and consulting activities.   In phase 2 of this epidemic - which is about to begin when this volume is to be published - the role of Public Health professionals could become even more relevant. However, this unexpected season must be managed with seriousness and intelligence, capitalizing it also for the future. If our Post-Graduate Schools of Public Health ("Igiene e Medicina Preventiva"), our scientific associations, our academic lecturers and our officers do not prove to be up to the situation, a dull future for the discipline might very well be. On the contrary, if as we all hope, we will be able to ride the wave of a dramatic health crisis, transforming it into a relevant scientific and professional opportunity, then we will be able to build on the post-COVID-19 a cutting-edge, a more attractive, relevant and modern discipline. This Supplement of Acta Biomedica, planned before the onset of the epidemic emergency in Italy, but already successful in presenting two papers on COVID-19, is further proof of how current and lively our discipline is.

Silich, B. A. (2020). "Method to Reduce Aerosolized Contaminant Concentration Exposure to Healthcare Workers During the COVID-19 Pandemic when Temporary Isolation Systems Are Required." West J Emerg Med **21**(6): 93-98.

The COVID-19 pandemic has strained the healthcare system. It has led to the use of temporary isolation systems and less-then-optimum patient placement configurations because of inadequate number of isolation rooms, both of which can compromise provider safety. Three key elements require special attention to reduce the maximum and average aerosolized contaminant concentration exposure to a healthcare worker in any isolation system: flow rate; air changes per hour; and patient placement. This is important because concentration exposures of aerosolized contaminants to healthcare workers in hospitals using temporary isolation systems can reach levels 21-30 times greater than a properly engineered negative pressure isolation room. A working knowledge of these three elements can help create a safer environment for healthcare workers when isolation rooms are not available.

Silva, R. N., et al. (2021). "Cardiorespiratory and skeletal muscle damage due to COVID-19: making the urgent case for rehabilitation." Expert Rev Respir Med: 1-14.

INTRODUCTION: It has become increasingly evident that COVID-19 contributes to multiorgan pathophysiology. The systemic inflammatory response increases both pro-inflammatory cytokine and chemokine levels, leading to immune dysregulation and increasing the likelihood of incurring cardiac and pulmonary injuries. AREAS COVERED: Longer periods of hospitalization (~20 days) increase susceptibility to ICU-acquired muscle weakness and deconditioning, which decreases muscle function and functional capacity. These conditions affect the quality of life in the post-COVID-19 period and require multi-disciplinary approaches to rehabilitate the cardiopulmonary and musculoskeletal systems of these patients. In this context, this narrative review, which included articles published in the Embase, PEDro and PubMed databases up to December 2020, is focused on discussing the essential role of exercise and rehabilitation health professionals in the COVID-19 recovery process, from hospitalization to hospital discharge, addressing strategies for professionals to mitigate the cardiac and pulmonary impairments associated with hospitalization to home or ambulatory rehabilitation, purposing ways to conduct rehabilitation programs to restore their functional status and quality of life after the infection. EXPERT OPINION: In the current environment, these findings further point to the vital role of rehabilitation health professionals in the coming years and the urgent need to develop strategies to assist COVID-19 survivors.

Sim, W. S., et al. (2020). "DORSCON Orange: An Approach to Challenges in a Busy Antenatal Diagnostic Centre in the Midst of a Global Pandemic." Ann Acad Med Singap **49**(9): 677-683.

The WHO declared the coronavirus disease 2019 (COVID-19) a global pandemic on 11 March 2020. Lessons from SARS epidemic led Singapore to develop stringent infection control protocols in preparation for future pandemics. However, unlike SARS, COVID-19 appears to be more transmissible and is predicted to continue for longer. As of 14 June 2020, there have been 40,197 positive cases with 26 deaths in Singapore, and KK Women's and Children's Hospital (KKH) has managed a total of 73 cases. Obstetrics ultrasound is an indispensable medical service and must continue to operate during a pandemic. A key balance must be struck between keeping patients and healthcare workers safe while being able to provide quality and prompt obstetric care. Our Antenatal Diagnostic Centre (ADC) in KKH developed new strategies to adapt to the pandemic when the national Disease Outbreak Response System Condition (DORSCON) was raised from yellow to orange on 7 February 2020. In this paper, we discuss our clinical workflow to reduce the risk of transmission amongst patients and staff while minimising disruption to our services.

Simmons, S. E., et al. (2021). "Deactivation of SARS-CoV-2 with pulsed-xenon ultraviolet light: Implications for environmental COVID-19 control." Infect Control Hosp Epidemiol **42**(2): 127-130.

OBJECTIVES: Prolonged survival of severe acute respiratory syndrome coronavirus 2 (SARS-CoV-2) on environmental surfaces and personal protective equipment may lead to these surfaces transmitting this pathogen to others. We sought to determine the effectiveness of a pulsed-xenon ultraviolet (PX-UV) disinfection system in reducing the load of SARS-CoV-2 on hard surfaces and N95 respirators. METHODS: Chamber slides and N95 respirator material were directly inoculated with SARS-CoV-2 and were exposed to different durations of PX-UV. RESULTS: For hard surfaces, disinfection for 1, 2, and 5 minutes resulted in 3.53 log10, >4.54 log10, and >4.12 log10 reductions in viral load, respectively. For N95 respirators, disinfection for 5 minutes resulted in >4.79 log10 reduction in viral load. PX-UV significantly reduced SARS-CoV-2 on hard surfaces and N95 respirators. CONCLUSION: With the potential to rapidly disinfectant environmental surfaces and N95 respirators, PX-UV devices are a promising technology to reduce environmental and personal protective equipment bioburden and to enhance both healthcare worker and patient safety by reducing the risk of exposure to SARS-CoV-2.

Simms, A., et al. (2020). "The impact of having inadequate safety equipment on mental health." Occup Med (Lond) **70**(4): 278-281.

BACKGROUND: Concerns are being raised about the impact of inadequate safety equipment on the mental health of healthcare workers during the COVID-19 medical response. AIMS: To assess the impact of inadequate safety equipment on the mental health of service personnel deployed on operations in order to better understand the impact on those working under the similarly demanding conditions of the COVID-19 medical response. METHODS: Self-report surveys were conducted in four operational environments with 3435 personnel providing data. Surveys recorded data on socio-demographic, military and operational characteristics, mental health measures and specific occupational stressors. Analysis through logistic regression explored the association between inadequate equipment and all other factors. RESULTS: A total of 3401 personnel provided data on their perceptions of the adequacy of their equipment, of which 532 (15%) stated that they had a lot of concerns that they did not have the right equipment in working order. Analysis found significantly greater odds of reporting symptoms of common mental health disorders (CMD), 2.49 (2.03-3.06), post-traumatic stress disorder (PTSD), 2.99 (2.11-4.24), poorer global health 2.09 (1.62-2.70) and emotional problems 1.69 (1.38-2.06) when individuals reported working with inadequate equipment. Analyses remained significant when adjusted for confounding factors such as rank, sex and operational environment. CONCLUSIONS: An individual's perception of having inadequate equipment is significantly associated with symptoms of CMD, probable PTSD, poorer global health and increased reporting of emotional problems. This in turn may impact on their ability to safely carry out their duties and may have longer-term mental health consequences.

Sims, M. D., et al. (2020). "COVID-19 seropositivity and asymptomatic rates in healthcare workers are associated with job function and masking." Clin Infect Dis.

BACKGROUND: Although the risk of exposure to SARS-CoV-2 is higher for frontline healthcare workers, not all personnel have similar risks. Determining infection rate is difficult due to the limits on testing and the high rate of asymptomatic individuals. Detection of antibodies against SARS-CoV-2 may be useful for determining prior exposure to the virus and assessing mitigation strategies, such as isolation, masks, and other protective equipment. METHODS: An online assessment that included demographic, clinical, and exposure information and a blood sample was collected from 20,614 participants out of ~43,000 total employees at Beaumont Health, which includes eight hospitals distributed across the Detroit metropolitan area in southeast Michigan. The presence of anti-SARS-CoV-2 IgG was determined using the EUROIMMUN assay. RESULTS: A total of 1,818 (8.8%) participants were seropositive between April 13 and May 28, 2020. Among the seropositive individuals, 44% reported that they were asymptomatic during the month prior to blood collection. Healthcare roles such as phlebotomy, respiratory therapy, and nursing/nursing support exhibited significantly higher seropositivity. Among participants reporting direct exposure to a COVID-19 positive individual, those wearing an N95/PAPR mask had a significantly lower seropositivity rate (10.2%) compared to surgical/other masks (13.1%) or no mask (17.5%). CONCLUSIONS: Direct contact with COVID-19 patients increased the likelihood of seropositivity among employees but study participants who wore a mask during COVID-19 exposures were less likely to be seropositive. Additionally, a large proportion of seropositive employees self-reported as asymptomatic. (Funded by Beaumont Health and by major donors through the Beaumont Health Foundation)ClinicalTrials.gov number NCT04349202.

Singh, B., et al. (2021). "Chloroquine or hydroxychloroquine for prevention and treatment of COVID-19." Cochrane Database Syst Rev **2**: Cd013587.

BACKGROUND: The coronavirus disease 2019 (COVID-19) pandemic has resulted in substantial mortality. Some specialists proposed chloroquine (CQ) and hydroxychloroquine (HCQ) for treating or preventing the disease. The efficacy and safety of these drugs have been assessed in randomized controlled trials. OBJECTIVES: To evaluate the effects of chloroquine (CQ) or hydroxychloroquine (HCQ) for 1) treating people with COVID-19 on death and time to clearance of the virus; 2) preventing infection in people at risk of SARS-CoV-2 exposure; 3) preventing infection in people exposed to SARS-CoV-2. SEARCH METHODS: We searched the Cochrane Central Register of Controlled Trials (CENTRAL), MEDLINE, Embase, Current Controlled Trials (www.controlled-trials.com), and the COVID-19-specific resources www.covid-nma.com and covid-19.cochrane.org, for studies of any publication status and in any language. We performed all searches up to 15 September 2020. We contacted researchers to identify unpublished and ongoing studies. SELECTION CRITERIA: We included randomized controlled trials (RCTs) testing chloroquine or hydroxychloroquine in people with COVID-19, people at risk of COVID-19 exposure, and people exposed to COVID-19. Adverse events (any, serious, and QT-interval prolongation on electrocardiogram) were also extracted. DATA COLLECTION AND ANALYSIS: Two review authors independently assessed eligibility of search results, extracted data from the included studies, and assessed risk of bias using the Cochrane 'Risk of bias' tool. We contacted study authors for clarification and additional data for some studies. We used risk ratios (RR) for dichotomous outcomes and mean differences (MD) for continuous outcomes, with 95% confidence intervals (CIs). We performed meta-analysis using a random-effects model for outcomes where pooling of effect estimates was appropriate. MAIN RESULTS: 1. Treatment of COVID-19 disease We included 12 trials involving 8569 participants, all of whom were adults. Studies were from China (4); Brazil, Egypt, Iran, Spain, Taiwan, the UK, and North America (each 1 study); and a global study in 30 countries (1 study). Nine were in hospitalized patients, and three from ambulatory care. Disease severity, prevalence of comorbidities, and use of co-interventions varied substantially between trials. We found potential risks of bias across all domains for several trials. Nine trials compared HCQ with standard care (7779 participants), and one compared HCQ with placebo (491 participants); dosing schedules varied. HCQ makes little or no difference to death due to any cause (RR 1.09, 95% CI 0.99 to 1.19; 8208 participants; 9 trials; high-certainty evidence). A sensitivity analysis using modified intention-to-treat results from three trials did not influence the pooled effect estimate.  HCQ may make little or no difference to the proportion of people having negative PCR for SARS-CoV-2 on respiratory samples at day 14 from enrolment (RR 1.00, 95% CI 0.91 to 1.10; 213 participants; 3 trials; low-certainty evidence). HCQ probably results in little to no difference in progression to mechanical ventilation (RR 1.11, 95% CI 0.91 to 1.37; 4521 participants; 3 trials; moderate-certainty evidence). HCQ probably results in an almost three-fold increased risk of adverse events (RR 2.90, 95% CI 1.49 to 5.64; 1394 participants; 6 trials; moderate-certainty evidence), but may make little or no difference to the risk of serious adverse events (RR 0.82, 95% CI 0.37 to 1.79; 1004 participants; 6 trials; low-certainty evidence). We are very uncertain about the effect of HCQ on time to clinical improvement or risk of prolongation of QT-interval on electrocardiogram (very low-certainty evidence). One trial (22 participants) randomized patients to CQ versus lopinavir/ritonavir, a drug with unknown efficacy against SARS-CoV-2, and did not report any difference for clinical recovery or adverse events. One trial compared HCQ combined with azithromycin against standard care (444 participants). This trial did not detect a difference in death, requirement for mechanical ventilation, length of hospital admission, or serious adverse events. A higher risk of adverse events was reported in the HCQ-and-azithromycin arm; this included QT-interval prolongation, when measured. One trial compared HCQ with febuxostat, another drug with unknown efficacy against SARS-CoV-2 (60 participants). There was no difference detected in risk of hospitalization or change in computed tomography (CT) scan appearance of the lungs; no deaths were reported. 2. Preventing COVID-19 disease in people at risk of exposure to SARS-CoV-2 Ongoing trials are yet to report results for this objective. 3. Preventing COVID-19 disease in people who have been exposed to SARS-CoV-2 One trial (821 participants) compared HCQ with placebo as a prophylactic agent in the USA (around 90% of participants) and Canada. Asymptomatic adults (66% healthcare workers; mean age 40 years; 73% without comorbidity) with a history of exposure to people with confirmed COVID-19 were recruited. We are very uncertain about the effect of HCQ on the primary outcomes, for which few events were reported: 20/821 (2.4%) developed confirmed COVID-19 at 14 days from enrolment, and 2/821 (0.2%) were hospitalized due to COVID-19 (very low-certainty evidence). HCQ probably increases the risk of adverse events compared with placebo (RR 2.39, 95% CI 1.83 to 3.11; 700 participants; 1 trial; moderate-certainty evidence). HCQ may result in little or no difference in serious adverse events (no RR: no participants experienced serious adverse events; low-certainty evidence). One cluster-randomized trial (2525 participants) compared HCQ with standard care for the prevention of COVID-19 in people with a history of exposure to SARS-CoV-2 in Spain. Most participants were working or residing in nursing homes; mean age was 49 years. There was no difference in the risk of symptomatic confirmed COVID-19 or production of antibodies to SARS-CoV-2 between the two study arms. AUTHORS' CONCLUSIONS: HCQ for people infected with COVID-19 has little or no effect on the risk of death and probably no effect on progression to mechanical ventilation. Adverse events are tripled compared to placebo, but very few serious adverse events were found. No further trials of hydroxychloroquine or chloroquine for treatment should be carried out. These results make it less likely that the drug is effective in protecting people from infection, although this is not excluded entirely. It is probably sensible to complete trials examining prevention of infection, and ensure these are carried out to a high standard to provide unambiguous results.

Singh Gambhir, R., et al. (2020). "Covid-19: a survey on knowledge, awareness and hygiene practices among dental health professionals in an Indian scenario." Rocz Panstw Zakl Hig **71**(2): 223-229.

BACKGROUND: Severe acute respiratory syndrome coronavirus 2 (SARS-CoV-2 or COVID-19) continues to spread globally. It has become a major cause of concern for health care professionals all over the world. OBJECTIVE: The aim of this study was to assess knowledge, awareness and hygiene practices regarding COVID-19 among private dental practitioners practicing in Tricity (Chandigarh, Panchkula and Mohali) in India during these critical times. MATERIALS AND METHODS: A total of 245 private dentists participated in this cross-sectional survey and finally 215 constituted the final sample size. A self-administered, multiple choice type questionnaire (verified by a specialist) was administered to obtain information from the subjects. The questionnaire was divided into two parts and included 15 questions on knowledge and awareness regarding COVID-19. Statistical analysis was done using ANOVA and Student’s t-test. RESULTS: Percentage of subjects who answered correctly regarding main symptoms of COVID-19 and primary mode of transmission was 87% and 82.5% respectively. One-third of the subjects were not aware regarding Personal Protective Equipment (PPE) to be used while rendering dental treatment. 75% of subjects were of the opinion that supportive care is the current treatment regime for COVID-19. Less than one-third of subjects (30.2%) reported high scores. Education level (p=0.018) and health sector profile (p=0.024) of the subjects were significantly associated with mean knowledge scores. CONCLUSION: The findings of the present study showed that some notable deficiencies in knowledge existed among dental professionals regarding some vital aspects of COVID-19. Therefore, there is an urgent need for improving dentists’knowledge via health education and training programs. Further studies on the subject are also warranted once the situation normalizes.

Singh, P., et al. (2021). "Pharmacological Prophylaxis and Personal Protective Equipment (PPE) Practices in Gynecological Cancer Surgery During COVID-19 Pandemic." Indian J Gynecol Oncol **19**(1): 19.

PURPOSE: In the absence of vaccine, proper use of personal protective equipment (PPE) is the most important strategy to protect healthcare workers against COVID-19 infection. The recommendations on pharmacological prophylaxis against COVID-19 infection are controversial. The aim of current study was to assess PPE practices during surgery on COVID-19 negative gynecological cancer patients and use of pharmacologic prophylaxis by clinicians practicing gynecologic oncology. METHODS: We disbursed a survey questionnaire through various social media platforms among clinicians practicing gynecologic oncology. The survey consisted of 37 questions divided into five subgroups evaluating demographic details, use of pharmacological prophylaxis against COVID-19, preoperative COVID-19 screening protocol, details on PPE usage and associated discomfort, if any. RESULTS: Two hundred twenty oncologists from 13 countries responded to the survey. Pharmacological prophylaxis was being used by 85 (38.6%) respondents; most common agent was hydroxychloroquin (HCQ) by 24.5% respondents. Routine preoperative screening for COVID-19 was performed by 214 (97.3%) respondents. Some degree of discomfort during surgery due to PPE use was reported by 170 (77.3%) respondents, which was moderate to severe in 73 (33.2%) respondents. Most common difficulties associated with face mask/shield were problems in communication (69.5%) and breathing (58.1%). Eye protection was associated with poor visibility, fogging and headache. Unusual fatigue attributed to PPE use was experienced by 143(65%) respondents. CONCLUSION: Use of pharmacological prophylaxis against COVID-19 is controversial and the same is reflected in our survey. Most respondents adhered to PPE use despite experiencing some physical discomfort. SUPPLEMENTARY INFORMATION: The online version contains supplementary material available at 10.1007/s40944-021-00500-4.

Singh, S. (2020). "Disability ethics in the coronavirus crisis." J Family Med Prim Care **9**(5): 2167-2171.

The disability viewpoint is fundamental for understanding and advancing social justice for everyone in the population. Despite this fact, it is regularly dismissed by public health experts and policymakers. Understanding of disability rights is central in an all-inclusive COVID-19 preparedness. This paper attempts to explore disability ethics in understanding structural discrimination, equitable practices, respect for disability culture and ways to safeguard health care professionals with disabilities in the coronavirus pandemic. In crisis standards of care, resource allocations must not be solely based on a disabled person's subjective quality of life. Health professionals should avoid stereotypes about an individual's disability to ration care. Triage protocol committees and disaster risk reduction working groups should explicitly recruit people with disabilities and chronic illnesses in their response strategies. Disability ethics can reform medical rationing by removing prejudices and safeguarding fair protection of the interests of all patients, including those with a disability.

Sinnige, J. S., et al. (2021). "Protection of healthcare workers during aerosol-generating procedures with local exhaust ventilation." Br J Anaesth.

Skokauskas, N., et al. (2020). "Supporting children of healthcare workers during the COVID-19 pandemic." Eur Child Adolesc Psychiatry: 1-2.

Slidell, M. B., et al. (2020). "Pediatric Modification of the Medically Necessary, Time-Sensitive Scoring System for Operating Room Procedure Prioritization During the COVID-19 Pandemic." J Am Coll Surg **231**(2): 205-215.

BACKGROUND: The COVID-19 pandemic forced surgeons to reconsider concepts of "elective" operations. Perceptions about the time sensitivity and medical necessity of a procedure have taken on greater significance during the pandemic. The evolving ethical and clinical environment requires reappraisal of perioperative factors, such as personal protective equipment conservation; limiting the risk of exposure to COVID-19 for patients, families, and healthcare workers; preservation of hospital beds and ICU resources; and minimizing COVID-19-related perioperative risk to patients. STUDY DESIGN: A scaffold for the complex decision-making required for prioritization of medically necessary, time-sensitive (MeNTS) operations was developed for adult patients by colleagues at the University of Chicago. Although adult MeNTS scoring can be applied across adult surgical specialties, some variables were irrelevant in a pediatric population. Pediatric manifestations of chronic diseases and congenital anomalies were not accounted for. To account for the unique challenges children face, we modified the adult MeNTS system for use across pediatric subspecialties. RESULTS: This pediatric MeNTS scoring system was applied to 101 cases both performed and deferred between March 23 and April 19, 2020 at the University of Chicago Comer Children's Hospital. The pediatric MeNTS scores provide a safe, equitable, transparent, and ethical strategy to prioritize children's surgical procedures. CONCLUSIONS: This process is adaptable to individual institutions and we project it will be useful during the acute phase of the pandemic (maximal limitations), as well as the anticipated recovery phase.

Smith, A. A., et al. (2020). "COVID-19 drive-through testing survey: Measuring the burden on healthcare workers." J Am Coll Emerg Physicians Open **1**(6): 1444-1449.

OBJECTIVE: To survey individuals who tested positive for severe acute respiratory syndrome coronavirus 2 (SARS-CoV-2) at 1 of 4 Trinity Health of New England drive-through testing centers to assess their demographic information, hospitalization rate, preexisting conditions, possible routes of exposures, duration of symptoms, and subsequent household infections of healthcare workers (HCWs) when compared to non-HCWs. METHODS: Data were collected via a telephone survey using a standardized script. Between March 1, 2020 and June 17, 2020, 28,903 people were tested at 4 Connecticut drive-through testing centers. Individuals who tested positive between March 16 and April 21, 2020 were randomly contacted. Of those individuals, 100 people agreed to complete the survey. Bivariate analysis and logistic regression were performed. RESULTS: HCWs comprised 46% of the 100 survey respondents during the study period. Similarly, HCWs comprised 42.1% of all individuals who tested positive and listed an employer between March 1 and June 17, 2020. HCWs reported a longer duration of symptoms (17.39 vs 13.44 days) and were more likely to report work as their route of exposure (80.4% vs 27.8%) than non-HCWs. CONCLUSIONS: HCWs may face a disproportionate risk of contracting COVID-19 and self-report a longer duration of symptoms than the general public. The data suggest a need for an increased recovery time away from work than is currently recommended by the Centers for Disease Control and Prevention, as well as an increase in infection precautions for HCWs.

Smith, A. A., et al. (2020). "Identifying Patients at Greatest Risk of Mortality due to COVID-19: A New England Perspective." West J Emerg Med **21**(4): 785-789.

INTRODUCTION: Severe acute respiratory syndrome coronavirus 2 (SARS-CoV-2) has spread rapidly since December 2019, resulting in a pandemic that has, as of May 24, 2020, yielded over 5.3 million confirmed cases and over 340,000 deaths. As businesses move to safely reopen and frontline healthcare workers (HCW) continue to face this crisis, it is essential that health officials know who in the population is at the greatest risk of mortality if hospitalized and, therefore, has the greatest need to protect themselves from being infected. We examined the factors that increase the risk of mortality among hospitalized COVID-19 patients. METHODS: This was a retrospective cohort study including confirmed COVID-19 patients admitted to the four Trinity Health of New England hospitals (THONE) in Connecticut and Massachusetts who either died or were discharged between March 1-April 22, 2020. Demographics, comorbidities, and outcomes of care were extracted from the electronic health record. A model of in-hospital mortality was made using a generalized linear model with binomial distribution and log link. RESULTS: The analysis included 346 patients: 229 discharged and 117 deceased. The likelihood of in-hospital mortality was increased for patients who were aged 60 or older (relative risk [RR] = 2.873; 95% confidence interval [CI], 1.733-4.764; p = <0.001), had diabetes (RR = 1.432; 95% CI,1.068-1.921; p = 0.016), or had chronic obstructive pulmonary disease (COPD) (RR = 1.410; 95% CI, 1.058-1.878; p = 0.019). Hyperlipidemia had a protective effect, reducing the likelihood of mortality (RR = 0.745; 95% CI, 0.568-0.975; p = 0.032). Sensitivity and specificity of the model were 51.4% and 88.4%, respectively. CONCLUSIONS: Being age 60 or older or having a history of diabetes or COPD are the most useful risk factors associated with mortality in hospitalized COVID-19 patients. As states ease stay-at-home orders, risk factors of severe disease can be used to identify those more likely to have worse outcomes if infected and hospitalized and, therefore, who in particular should continue to follow public health guidelines for avoiding infection: stay home if possible; practice physical distancing; and wear a facemask.

Smith, C. (2020). "The structural vulnerability of healthcare workers during COVID-19: Observations on the social context of risk and the equitable distribution of resources." Soc Sci Med **258**: 113119.

Healthcare workers have emerged as a vulnerable population group during COVID-19, and securing supply chains of personal protective equipment (PPE) has been identified as a critical issue to protect healthcare workers and to prevent health system overwhelm. While securing PPE is a complex logistical challenge facing many countries, it is vital to recognise the social and health systems issues that structure the differential degrees of risk faced by various subgroups of healthcare workers. As an illustrative case study, the author identifies two key social factors that are likely to face the degrees of risk faced by midwives in the Special Region of Yogyakarta, Indonesia, if and when COVID-19 takes hold in Indonesia. Healthcare workers in both high and low resource-settings globally are likely to face particular risks and vulnerabilities that are shaped by localized social and health systems factors. Qualitative social and health systems research can and should be utilized proactively in order to protect healthcare workers, to inform more equitable program design, and to create a foundation for health equity within the future of global health that emerges from the pandemic.

Smith, C. R., et al. (2020). "A Flexible Enclosure to Protect Respiratory Therapists During Aerosol-Generating Procedures." Respir Care **65**(12): 1923-1932.

BACKGROUND: Exposure of respiratory therapists (RTs) during aerosol-generating procedures such as endotracheal intubation is an occupational hazard. Depending on the hospital, RTs may serve as laryngoscopist or in a role providing ventilation support and initiating mechanical ventilation. This study aimed to evaluate the potential exposure of RTs serving in either of these roles. METHODS: We set up a simulated patient with severe acute respiratory syndrome coronavirus-2 (SARS-CoV-2) infection in an ICU setting requiring endotracheal intubation involving a laryngoscopist, a nurse, and an RT supporting the laryngoscopist. All participants wore appropriate personal protective equipment (PPE). A fluorescent marker was sprayed by an atomizer during the procedure using 3 different methods for endotracheal intubation. The 3 techniques included PPE alone, a polycarbonate intubating box, or a coronavirus flexible enclosure, which consisted of a Mayo stand with plastic covering. The laryngoscopist and the supporting RT were assessed with a black light for contamination with the fluorescent marker. All simulations were recorded. RESULTS: When using only PPE, both the laryngoscopist and the RT were grossly contaminated. When using the intubating box, the laryngoscopist's contamination was detectable only on the gloves: the gown and face shield remained uncontaminated; the RT was still grossly contaminated on the gloves, gown, neck, and face shield. When using the coronavirus flexible enclosure system, both the laryngoscopist and the RT were better protected, with contamination detected only on the gloves of the laryngoscopist and the RT. CONCLUSIONS: Of the 3 techniques, the coronavirus flexible enclosure contained the fluorescent marker more effectively during endotracheal intubation than PPE alone or the intubating box based on exposure of the laryngoscopist and supporting RT. Optimizing containment during aerosol-generating procedures like endotracheal intubation is a critical component of minimizing occupational and nosocomial spread of SARS-CoV-2 to RTs who may serve as either the laryngoscopist or a support role.

Smith, D., et al. (2020). "Tracheostomy in the intensive care unit: Guidelines during COVID-19 worldwide pandemic." Am J Otolaryngol **41**(5): 102578.

PURPOSE: COVID-19 has become a pandemic with significant consequences worldwide. About 3.2% of patients with COVID-19 will require intubation and invasive ventilation. Moreover, there will be an increase in the number of critically ill patients, hospitalized and intubated due to unrelated acute pathology, who will present underlying asymptomatic or mild forms of COVID-19. Tracheostomy is one of the procedures associated with an increased production of aerosols and higher risk of transmission of the virus to the health personnel. The aim of this paper is to describe indications and recommended technique of tracheostomy in COVID-19 patients, emphasizing the safety of the patient but also the medical team involved. MATERIALS AND METHODS: A multidisciplinary group made up of surgeons with privileges to perform tracheostomies, intensive care physicians, infectious diseases specialists and intensive pulmonologists was created to update previous knowledge on performing a tracheostomy in critically ill adult patients (>18 years) amidst the SARS-CoV-2 pandemic in a high-volume referral center. Published evidence was collected using a systematic search and review of published studies. RESULTS: A guideline comprising indications, surgical technique, ventilator settings, personal protective equipment and timing of tracheostomy in COVID-19 patients was developed. CONCLUSIONS: A safe approach to performing percutaneous dilational bedside tracheostomy with bronchoscopic guidance is feasible in COVID-19 patients of appropriate security measures are taken and a strict protocol is followed. Instruction of all the health care personnel involves is key to ensure their safety and the patient's favorable recovery.

Smith, D. R. M., et al. (2020). "Optimizing COVID-19 surveillance in long-term care facilities: a modelling study." BMC Med **18**(1): 386.

BACKGROUND: Long-term care facilities (LTCFs) are vulnerable to outbreaks of coronavirus disease 2019 (COVID-19). Timely epidemiological surveillance is essential for outbreak response, but is complicated by a high proportion of silent (non-symptomatic) infections and limited testing resources. METHODS: We used a stochastic, individual-based model to simulate transmission of severe acute respiratory syndrome coronavirus 2 (SARS-CoV-2) along detailed inter-individual contact networks describing patient-staff interactions in a real LTCF setting. We simulated distribution of nasopharyngeal swabs and reverse transcriptase polymerase chain reaction (RT-PCR) tests using clinical and demographic indications and evaluated the efficacy and resource-efficiency of a range of surveillance strategies, including group testing (sample pooling) and testing cascades, which couple (i) testing for multiple indications (symptoms, admission) with (ii) random daily testing. RESULTS: In the baseline scenario, randomly introducing a silent SARS-CoV-2 infection into a 170-bed LTCF led to large outbreaks, with a cumulative 86 (95% uncertainty interval 6-224) infections after 3 weeks of unmitigated transmission. Efficacy of symptom-based screening was limited by lags to symptom onset and silent asymptomatic and pre-symptomatic transmission. Across scenarios, testing upon admission detected just 34-66% of patients infected upon LTCF entry, and also missed potential introductions from staff. Random daily testing was more effective when targeting patients than staff, but was overall an inefficient use of limited resources. At high testing capacity (> 10 tests/100 beds/day), cascades were most effective, with a 19-36% probability of detecting outbreaks prior to any nosocomial transmission, and 26-46% prior to first onset of COVID-19 symptoms. Conversely, at low capacity (< 2 tests/100 beds/day), group testing strategies detected outbreaks earliest. Pooling randomly selected patients in a daily group test was most likely to detect outbreaks prior to first symptom onset (16-27%), while pooling patients and staff expressing any COVID-like symptoms was the most efficient means to improve surveillance given resource limitations, compared to the reference requiring only 6-9 additional tests and 11-28 additional swabs to detect outbreaks 1-6 days earlier, prior to an additional 11-22 infections. CONCLUSIONS: COVID-19 surveillance is challenged by delayed or absent clinical symptoms and imperfect diagnostic sensitivity of standard RT-PCR tests. In our analysis, group testing was the most effective and efficient COVID-19 surveillance strategy for resource-limited LTCFs. Testing cascades were even more effective given ample testing resources. Increasing testing capacity and updating surveillance protocols accordingly could facilitate earlier detection of emerging outbreaks, informing a need for urgent intervention in settings with ongoing nosocomial transmission.

Smith, P. M., et al. (2020). "Labour Market Attachment, Workplace Infection Control Procedures and Mental Health: A Cross-Sectional Survey of Canadian Non-healthcare Workers during the COVID-19 Pandemic." Ann Work Expo Health.

BACKGROUND: The COVID-19 pandemic has led to large proportions of the labour market moving to remote work, while others have become unemployed. Those still at their physical workplace likely face increased risk of infection, compared to other workers. The objective of this paper is to understand the relationship between working arrangements, infection control programs (ICP), and symptoms of anxiety and depression among Canadian workers, not specifically working in healthcare. METHODS: A convenience-based internet survey of Canadian non-healthcare workers was facilitated through various labour organizations between April 26 and June 6, 2020. A total of 5180 respondents started the survey, of which 3779 were assessed as employed in a full-time or part-time capacity on 2 March 2020 (prior to large-scale COVID-19 pandemic responses in Canada). Of this sample, 3305 (87.5%) had complete information on main exposures and outcomes. Anxiety symptoms were measured using the Generalised Anxiety Disorder screener (GAD-2), and depressive symptoms using the Patient Health Questionnaire screener (PHQ-2). For workers at their physical workplace (site-based workers) we asked questions about the adequacy and implementation of 11 different types of ICP, and the adequacy and supply of eight different types of personal protective equipment (PPE). Respondents were classified as either: working remotely; site-based workers with 100% of their ICP/PPE needs met; site-based workers with 50-99% of ICP/PPE needs met; site-based workers with 1-49% of ICP/PPE needs met; site-based workers with none of ICP/PPE needs met; or no longer employed. Regression analyses examined the association between working arrangements and ICP/PPE adequacy and having GAD-2 and PHQ-2 scores of three and higher (a common screening point in both scales). Models were adjusted for a range of demographic, occupation, workplace, and COVID-19-specific factors. RESULTS: A total of 42.3% (95% CI: 40.6-44.0%) of the sample had GAD-2 scores of 3 and higher, and 34.6% (95% CI: 32.-36.2%) had PHQ-2 scores of 3 and higher. In initial analyses, symptoms of anxiety and depression were lowest among those working remotely (35.4 and 27.5%), compared to site-based workers (43.5 and 34.7%) and those who had lost their jobs (44.1 and 35.9%). When adequacy of ICP and PPE was taken into account, the lowest prevalence of anxiety and depressive symptoms was observed among site-based workers with all of their ICP needs being met (29.8% prevalence for GAD-2 scores of 3 and higher, and 23.0% prevalence for PHQ-2 scores of 3 and higher), while the highest prevalence was observed among site-based workers with none of their ICP needs being met (52.3% for GAD-2 scores of 3 and higher, and 45.8% for PHQ-2 scores of 3 and higher). CONCLUSION: Our results suggest that the adequate design and implementation of employer-based ICP have implications for the mental health of site-based workers. As economies re-open the ongoing assessment of ICP and associated mental health outcomes among the workforce is warranted.

Sokunbi, O. J., et al. (2020). "Maintaining paediatric cardiac services during the COVID-19 pandemic in a developing country in sub-Saharan Africa: guidelines for a "scale up" in the face of a global "scale down"." Cardiol Young **30**(11): 1588-1594.

The COVID-19 pandemic is currently ravaging the globe and the African continent is not left out. While the direct effects of the pandemic in regard to morbidity and mortality appear to be more significant in the developed world, the indirect harmful effects on already insufficient healthcare infrastructure on the African continent would in the long term be more detrimental to the populace. Women and children form a significant vulnerable population in underserved areas such as the sub-Saharan region, and expectedly will experience the disadvantages of limited healthcare coverage which is a major fall out of the pandemic. Paediatric cardiac services that are already sparse in various sub-Saharan countries are not left out of this downsizing. Restrictions on international travel for patients out of the continent to seek medical care and for international experts into the continent for regular mission programmes leave few options for children with cardiac defects to get the much-needed care.There is a need for a region-adapted guideline to scale-up services to cater for more children with congenital heart disease (CHD) while providing a safe environment for healthcare workers, patients, and their caregivers. This article outlines measures adapted to maintain paediatric cardiac care in a sub-Saharan tertiary centre in Nigeria during the COVID-19 pandemic and will serve as a guide for other institutions in the region who will inadvertently need to provide these services as the demand increases.

Solia, E., et al. (2020). "The Role of Surgical Masks during the COVID-19 Pandemic. A Mini-Review." J Long Term Eff Med Implants **30**(4): 241-246.

Introduction - The coronavirus disease 2019 (COVID-19) is considered to be highly contagious and restriction of transmission requires the utilization of protective equipment like surgical masks from both healthcare workers and public. The aim of this review is to investigate the role of surgical masks during the COVID-19 pandemic. Materials and Methods - A literature research was conducted via PubMed to detect articles featuring the potential protective role of surgical masks when they were worn by healthcare workers or by the general public. Results - Among 114 articles, only 31 met the inclusion criteria. Thirteen additional articles provided useful information according to the aim of this review. Existing literature supports the positive effect of surgical masks on COVID-19 con-lamination. Debate on the efficacy of surgical masks compared with other forms of facial protective devices exists. There seems to be a tendency to favor the use of particulate-filtering facepiece respirators in healthcare professionals who face higher risk of infection. However, surgical masks primarily and cloth masks secondarily seem to be adequate means of protection for the general public and for healthcare workers during procedures where respiratory droplets are not generated. Rational use of surgical masks is imperative; however, reuse after decontamination is not ideal. Conclusions - For optimum protection from COVID-19, the use of surgical masks should be combined with other infection control measures like hand hygiene and social distancing, since the level of their effectiveness is still being investigated. Shortage of surgical masks should be prevented; therefore, rational use plays a crucial role in this direction.

Somashekhar, S. P., et al. (2020). "Adaptations and Safety Modifications to Perform Safe Minimal Access Surgery (MIS: Laparoscopy and Robotic) During the COVID-19 Pandemic: Practice Modifications Expert Panel Consensus Guidelines from Academia of Minimal Access Surgical Oncology (AMASO)." Indian J Surg Oncol: 1-11.

The pandemic of COVID-19 across the globe triggered national lockdowns hampering normal working for all the essential services including healthcare. In order to reduce transmission and safety of patients and healthcare workers, the elective surgeries have been differed. The visits to the hospitals for follow-ups and consultations received temporary halt. However, we cannot halt the treatment for cancer patients who may or may not be COVID-19 positives. These are emergencies and should be treated ASAP. Conducting emergency surgeries during pandemic like COVID-19 is challenge for surgeons and the entire hospital infrastructure. The available information about COVID-19 and its propensity of contamination through droplets and aerosol need some modifications for conducting surgeries successfully without contaminating the hospital buildings, protecting healthcare teams and the patient. With these objectives, some modifications in the operating theater including surgical techniques for minimal access, laparoscopy, and robotic surgery are proposed in this review article. This review article also discusses the safety measures to be followed for the suspected or confirmed COVID-19 patient and the guidelines and recommendations for healthcare teams while treating these patients. Although there is little evidence of viral transmission through laparoscopic or open approaches, modifications to surgical practice such as the use of safe smoke evacuation and minimizing energy device used to reduce the risk of exposure to aerosolized particles to healthcare team are proposed in this review article.

Somashekhar, S. P., et al. (2020). "Minimally Invasive Surgery and Surgical Smoke, Decoding Fear and Ensuring Safety: Adaptations and Safety Modifications During COVID Pandemic." Indian J Gynecol Oncol **18**(3): 96.

The most fearful word starting from C, Cancer has now been replaced with COVID-19 owing to its associated physical, emotional and financial hardships as well as its social stigma. Never before we as medical fraternity been challenged to take care of patients and at the same time consider the safety of ourselves, family members and our fellow healthcare workers. Emotions and fear-driven treatments that are otherwise inefficacious may contribute to a false sense of security, unwarranted side-effects, divert resources and delay research into treatments that may actually work. Decoding fear with available evidence i.e. practicing evidence-based medicine will guide us in better handling of situations in this pandemic. The objective of this review is to discuss the modifications required in the operating theatre during COVID-19 times for minimal access, laparoscopy and robotic surgery, especially with regard to the handling of surgical smoke, minimally invasive surgical instruments, trocars with smoke evacuator and special personal protection equipment. Although there is no evidence of viral transmission through laparoscopic or open approaches, we recommend modifications to surgical practice such as the use of safe smoke evacuation and minimizing energy device use. We have come up with Rule of 20 for 2020 pandemic in operation theatres and modification of trocar for safe handling of surgical smoke in MIS which can be used in resource-limited settings. Hospitals must follow specific protocols and arrange suitable training of the healthcare workers. We believe that "Fears are educated into us, and can, if we wish, be educated out".

Sommerstein, R., et al. (2020). "Risk of SARS-CoV-2 transmission by aerosols, the rational use of masks, and protection of healthcare workers from COVID-19." Antimicrob Resist Infect Control **9**(1): 100.

OBJECTIVES: To determine the risk of SARS-CoV-2 transmission by aerosols, to provide evidence on the rational use of masks, and to discuss additional measures important for the protection of healthcare workers from COVID-19. METHODS: Literature review and expert opinion. SHORT CONCLUSION: SARS-CoV-2, the pathogen causing COVID-19, is considered to be transmitted via droplets rather than aerosols, but droplets with strong directional airflow support may spread further than 2 m. High rates of COVID-19 infections in healthcare-workers (HCWs) have been reported from several countries. Respirators such as filtering face piece (FFP) 2 masks were designed to protect HCWs, while surgical masks were originally intended to protect patients (e.g., during surgery). Nevertheless, high quality standard surgical masks (type II/IIR according to European Norm EN 14683) appear to be as effective as FFP2 masks in preventing droplet-associated viral infections of HCWs as reported from influenza or SARS. So far, no head-to-head trials with these masks have been published for COVID-19. Neither mask type completely prevents transmission, which may be due to inappropriate handling and alternative transmission pathways. Therefore, compliance with a bundle of infection control measures including thorough hand hygiene is key. During high-risk procedures, both droplets and aerosols may be produced, reason why respirators are indicated for these interventions.

Song, P. and T. Karako (2020). "Scientific solidarity in the face of the COVID-19 pandemic: researchers, publishers, and medical associations." Glob Health Med **2**(2): 56-59.

In the face of COVID-19, the scientific community has rapidly come together to address this outbreak in an open and collaborative manner to support the global response to this outbreak by rapidly sharing and highlighting research data and relevant findings. COVID-19 research is being published at a furious pace. Over 6,000 articles have been published as of 20 April 2020, and at least 15 online resource centers/websites for COVID-19 have been created by publishers to enable fast and free access to the latest research, evidence, and data available. Moreover, many evidence-based guidelines for COVID-19 have been issued based on academic articles and summaries of the experiences of frontline medical personnel. Various academic medical associations are also actively sharing information and providing technical support. As an example, 93 guides/proposals/responses to COVID-19 have been issued so far by 50 medical associations in Japan. However, few publications and national situation reports have provided information on the number of infected healthcare workers (HCWs). More publications and national situation reports are urgently needed to provide scientific information to devise specific infection prevention and control measures in order to protect HCWs from infection.

Sougou, N. M., et al. (2020). "[Risk perception of COVID-19 pandemic among health care providers: qualitative study conducted at the King Baudoin Hospital in Guédiawaye, the first hospital faced with managing a community-acquired COVID-19 case in Senegal]." Pan Afr Med J **37**(Suppl 1): 23.

INTRODUCTION: health care systems in West Africa have been under strain since the beginning of the COVID-19 pandemic. The exposure of health personnel to infection during the COVID-19 pandemic has been reported in several studies. The purpose of this study was to analyze health workers' actual and perceived risk in the first hospital faced with managing a community-acquired COVID-19 case in Senegal. METHODS: we conducted an exploratory descriptive study of health care providers' perception about their own risk during the COVID-19 pandemic. Forty-seven health-care providers were interviewed in personal, extensive interviews in this hospital. RESULTS: the fear of disease was present among the health staff. This fear was caused by several exogenous and endogenous factors, including the lack of knowledge of the virus and COVID-19 disease, the feeling of vulnerability due to insufficient availability of personal protective equipment, healthcare professionals' position in relation to the priesthood from the medical profession and the real and perceived risk of putting their family and their entourage in potentially dangerous situations. CONCLUSION: this study highlights the need for psycho-affective management of health care workers during this pandemic by taking the gender dimension into account. The provision of personal adequate protective equipment and stress management measures could enable front-line workers to cope with this pandemic in complete serenity.

Publisher: Abstract available from the publisher.

fre

Sowerby, L. J., et al. (2020). "International registry of otolaryngologist-head and neck surgeons with COVID-19." Int Forum Allergy Rhinol **10**(11): 1201-1208.

BACKGROUND: It has become clear that healthcare workers are at high risk, and otolaryngology has been theorized to be among the highest risk specialties for coronavirus disease 2019 (COVID-19). The purpose of this study was to detail the international impact of COVID-19 among otolaryngologists, and to identify instructional cases. METHODS: Country representatives of the Young Otolaryngologists-International Federation of Otolaryngologic Societies (YO-IFOS) surveyed otolaryngologists through various channels. Nationwide surveys were distributed in 19 countries. The gray literature and social media channels were searched to identify reported deaths of otolaryngologists from COVID-19. RESULTS: A total of 361 otolaryngologists were identified to have had COVID-19, and data for 325 surgeons was available for analysis. The age range was 25 to 84 years, with one-half under the age of 44 years. There were 24 deaths in the study period, with 83% over age 55 years. Source of infection was likely clinical activity in 175 (54%) cases. Prolonged exposure to a colleague was the source for 37 (11%) surgeons. Six instructional cases were identified where infections occurred during the performance of aerosol-generating operations (tracheostomy, mastoidectomy, epistaxis control, dacryocystorhinostomy, and translabyrinthine resection). In 3 of these cases, multiple operating room attendees were infected, and in 2, the surgeon succumbed to complications of COVID-19. CONCLUSION: The etiology of reported cases within the otolaryngology community appear to stem equally from clinical activity and community spread. Multiple procedures performed by otolaryngologists are aerosol-generating procedures (AGPs) and great care should be taken to protect the surgical team before, during, and after these operations.

Sri Santosh, T., et al. (2020). "A Review of Salivary Diagnostics and Its Potential Implication in Detection of Covid-19." Cureus **12**(4): e7708.

Saliva is an exocrine secretion produced from the salivary glands and has numerous functions, such as cleansing and protection of the oral cavity, antimicrobial effects and aids in digestion. Due to the speedy development in the field of salivaomics, saliva is now well accepted as a pool of biological markers that vary from changes in biochemicals, nucleic acids and proteins to the microflora. Saliva has an immense potential as a diagnostic fluid and offers an edge over other biological fluids as its collection method does not require invasive procedure, economical and is useful for monitoring systemic health. Development of sensitive and precise salivary diagnostic tools and the formulation of defined guidelines following meticulous testing will allow salivary diagnostics to be utilised as chair side tests for various oral and systemic diseases in the near future. The coronavirus disease (Covid-19) pandemic is the biggest challenge and global health crisis for the world since World War Two. Rapid and accurate diagnosis of Covid-19 is crucial in controlling the outbreak in the community and in hospitals. Nasopharyngeal and oropharyngeal swabs are the recommended specimen types for Covid-19 diagnostic testing. The collection of these specimen types requires close contact between healthcare workers and patients and poses a risk of transmission of the virus, causes discomfort and may cause bleeding, especially in patients with condition such as thrombocytopenia. Hence, nasopharyngeal or oropharyngeal swabs are not desirable for sequential monitoring of viral load. Saliva specimens can be obtained easily as the patient is asked to spit into a sterile bottle. The collection of saliva is non-invasive and greatly minimizes the exposure of healthcare workers to Covid-19. Saliva has a high consistency rate of greater than 90% with nasopharyngeal specimens in the detection of respiratory viruses, including coronaviruses. Saliva has also been used in screening respiratory viruses among hospitalized patients without pyrexia or respiratory symptoms. SARS-CoV can be detected in saliva at high titers. Salivary diagnostics is a dynamic field that is being incorporated as part of disease diagnosis, clinical monitoring of systemic health and to make significant clinical decisions for patient care. More research is required to analyze the potential diagnostic of Covid-19 in saliva to develop rapid chair side tests for the detection of Covid-19 and it is also pivotal to improve and develop successful strategies for prevention, especially for dentists and healthcare professionals who are involved in performing aerosol-generating procedures.

Srinivasan, S. and W. Peh (2020). "N95 Filtering Facepiece Respirators during the COVID-19 Pandemic: Basics, Types, and Shortage Solutions." Malays Orthop J **14**(2): 16-22.

The coronavirus disease 2019 (COVID-19) is highly infectious, with the current pandemic causing significant morbidity and mortality worldwide. As large numbers of frontline healthcare workers (HCWs) have also been infected and several have died, there is much global concern about protective measures for them, particularly those performing surgery or other procedures with close patient contact. Since the beginning of the pandemic, there has been and there remains a shortage in the supply of personal protective equipment (PPE), including the N95 filtering facepiece (FFP) respirator, for HCWs. N95 respirators have filtration efficiency of 95% of aerosol particles. Surgical N95 respirators are used where fluid resistance is also required together with respiratory protection, e.g. during surgery or interventional procedures. The shortage of N95 respirators may be overcome by extended use and reuse - comprising rotation and decontamination by approved techniques. The additional role of powered air-purifying respirators (PAPR) is also discussed.

Srivastava, A., et al. (2020). "Surgical practice recommendations for minimal access surgeons during COVID 19 pandemic - Indian inter-society directives." J Minim Access Surg **16**(3): 195-200.

These are inter-society guidelines for performance of laparoscopic surgery during COVID-19 pandemic that has affected the way of surgical practice. The safety of healthcare workers and patients is being challenged. It is prudent that our surgical practice should adapt to this rapidly changing health environment. The guidance issued is based on global practices and national governmental directives. The Inter-Society Group urges you to be updated with the developing situation and evolving changes.

Stawicki, S. P., et al. (2020). "The 2019-2020 Novel Coronavirus (Severe Acute Respiratory Syndrome Coronavirus 2) Pandemic: A Joint American College of Academic International Medicine-World Academic Council of Emergency Medicine Multidisciplinary COVID-19 Working Group Consensus Paper." J Glob Infect Dis **12**(2): 47-93.

What started as a cluster of patients with a mysterious respiratory illness in Wuhan, China, in December 2019, was later determined to be coronavirus disease 2019 (COVID-19). The pathogen severe acute respiratory syndrome coronavirus 2 (SARS-CoV-2), a novel Betacoronavirus, was subsequently isolated as the causative agent. SARS-CoV-2 is transmitted by respiratory droplets and fomites and presents clinically with fever, fatigue, myalgias, conjunctivitis, anosmia, dysgeusia, sore throat, nasal congestion, cough, dyspnea, nausea, vomiting, and/or diarrhea. In most critical cases, symptoms can escalate into acute respiratory distress syndrome accompanied by a runaway inflammatory cytokine response and multiorgan failure. As of this article's publication date, COVID-19 has spread to approximately 200 countries and territories, with over 4.3 million infections and more than 290,000 deaths as it has escalated into a global pandemic. Public health concerns mount as the situation evolves with an increasing number of infection hotspots around the globe. New information about the virus is emerging just as rapidly. This has led to the prompt development of clinical patient risk stratification tools to aid in determining the need for testing, isolation, monitoring, ventilator support, and disposition. COVID-19 spread is rapid, including imported cases in travelers, cases among close contacts of known infected individuals, and community-acquired cases without a readily identifiable source of infection. Critical shortages of personal protective equipment and ventilators are compounding the stress on overburdened healthcare systems. The continued challenges of social distancing, containment, isolation, and surge capacity in already stressed hospitals, clinics, and emergency departments have led to a swell in technologically-assisted care delivery strategies, such as telemedicine and web-based triage. As the race to develop an effective vaccine intensifies, several clinical trials of antivirals and immune modulators are underway, though no reliable COVID-19-specific therapeutics (inclusive of some potentially effective single and multi-drug regimens) have been identified as of yet. With many nations and regions declaring a state of emergency, unprecedented quarantine, social distancing, and border closing efforts are underway. Implementation of social and physical isolation measures has caused sudden and profound economic hardship, with marked decreases in global trade and local small business activity alike, and full ramifications likely yet to be felt. Current state-of-science, mitigation strategies, possible therapies, ethical considerations for healthcare workers and policymakers, as well as lessons learned for this evolving global threat and the eventual return to a "new normal" are discussed in this article.

Steinberg, B. E., et al. (2020). "Efficacy and safety of decontamination for N95 respirator reuse: a systematic literature search and narrative synthesis." Can J Anaesth **67**(12): 1814-1823.

PURPOSE: Under times of supply chain stress, the availability of some medical equipment and supplies may become limited. The current pandemic involving severe acute respiratory syndrome coronavirus 2 has highlighted limitations to the ordinary provision of personal protective equipment (PPE). For perioperative healthcare workers, N95 masks provide a stark example of PPE in short supply necessitating the creation of scientifically valid protocols for their decontamination and reuse. METHODS: We performed a systematic literature search of MEDLINE, Embase, Cochrane CENTRAL databases, and ClinicalTrials.gov to identify peer-reviewed articles related to N95 mask decontamination and subsequent testing for the integrity of mask filtration and facial seal. To expand this search, we additionally surveyed the official statements from key health agencies, organizations, and societies for relevant citations. RESULTS: Our initial database search resulted in five articles that met inclusion criteria, with 26 articles added from the expanded search. Our search did not reveal any relevant randomized clinical trials or cohort studies. We found that moist mask heating (65-80°C at 50-85% relative humidity for 20-30 min) and vaporous hydrogen peroxide treatment were supported by the literature to provide consistent viral decontamination without compromising mask seal and filtration efficiency. Other investigated decontamination methods lacked comprehensive scientific evidence for all three of these key criteria. CONCLUSIONS: N95 mask reprocessing using either moist heat or vaporous hydrogen peroxide is recommended to ensure healthcare worker safety.

Štěpánek, L., et al. (2021). "Demand and motivation for influenza vaccination among healthcare workers before and during the COVID-19 era: a cross-sectional survey." Hum Vaccin Immunother: 1-6.

Protection of healthcare workers (HCWs) against influenza is essential for patient health and a functional health system. The study aimed to analyze the demand for seasonal influenza vaccination (SIV) among various groups of HCWs in a tertiary care hospital before and during the COVID-19 pandemic and to identify their motives for this season's SIV. Before this influenza season (2020/21), the hospital management offered free SIV to all HCWs and promoted it on the internal network. Out of 4,167 HCWs, 630 HCWs expressed interest in SIV and were vaccinated in the hospital. They filled in a total of 603 self-administered pen-and-paper questionnaires. The mean age of the respondents (374 females and 229 males) was 45 ± 12 years. Physicians accounted for 48% of the vaccinated persons but for only 24% (p < .001) of all HCWs to whom SIV was offered. Only 16% of respondents vaccinated this year also received SIV before the last season (2019/20), with the proportion of physicians (19%) being statistically significantly higher than that of non-physicians (13%, p = .045) and the proportion of chronically ill HCWs (22%) being higher than that of healthy individuals (13%, p = .004). Most frequently, respondents' motivation to get vaccinated this year was self-protection (61%), that is concerns about contracting influenza together with COVID-19 or alone, followed by family protection (58%) and patient protection (53%). In conclusion, COVID-19 contributed to an increased demand for SIV among HCWs and the threat of contracting it together with influenza was the most frequent motive.

Sterman, Y., et al. (2021). "Safety on demand: A case study for the design and manufacturing-on-demand of personal protective equipment for healthcare workers during the COVID-19 pandemic." Saf Sci **136**: 105162.

The COVID-19 pandemic has highlighted the difficulties of countries and healthcare systems in preparing for major emergency situations. In the first month of the pandemic there was a global shortage of personal protective equipment (PPE), thereby causing a rise in infection cases and deaths among healthcare workers (HCWs) in some countries. Moreover, the PPE used in hospitals today is not designed for prolonged use, and causes problems such as headaches and vision difficulties. To address these problems, a case study of an active PPE system for HCWs that is based on 3D printing and the use of ready-made parts is presented here. This case study demonstrates a new approach toward the design and manufacturing of PPE in emergency situations that relies on rapid development and domestic manufacturing of products through 3D printing technologies. The system was developed during the first months of the pandemic under restrictive quarantine conditions, and was fabricated and then tested by HCWs at several hospitals. Apart from sterilization difficulties, the system received positive feedback in user testing.

Stock, A. D., et al. (2020). "COVID-19 Infection Among Healthcare Workers: Serological Findings Supporting Routine Testing." Front Med (Lausanne) **7**: 471.

A growing body of evidence demonstrates that asymptomatic and pre-symptomatic transmission of SARS-CoV-2 is a major contributor to the COVID-19 pandemic. Frontline healthcare workers in COVID-19 hotspots have faced numerous challenges, including shortages of personal protective equipment (PPE) and difficulties acquiring clinical testing. The magnitude of the exposure of healthcare workers and the potential for asymptomatic transmission makes it critical to understand the incidence of infection in this population. To determine the prevalence of asymptomatic SARS-CoV-2 infection amongst healthcare workers, we studied frontline staff working in the Montefiore Health System in New York City. All participants were asymptomatic at the time of testing and were tested by RT-qPCR and for anti-SARS-CoV-2 antibodies. The medical, occupational, and COVID-19 exposure histories of participants were recorded via questionnaires. Of the 98 asymptomatic healthcare workers tested, 19 (19.4%) tested positive by RT-qPCR and/or ELISA. Within this group, four (4.1%) were RT-qPCR positive, and four (4.1%) were PCR and IgG positive. Notably, an additional 11 (11.2%) individuals were IgG positive without a positive PCR. Two PCR positive individuals subsequently developed COVID-19 symptoms, while all others remained asymptomatic at 2-week follow-up. These results indicate that there is considerable asymptomatic infection with SARS-CoV-2 within the healthcare workforce, despite current mitigation policies. Furthermore, presuming that asymptomatic staff are not carrying SARS-CoV-2 is inconsistent with our results, and this could result in amplified transmission within healthcare settings. Consequently, aggressive testing regiments, such as testing frontline healthcare workers on a regular, multi-modal basis, may be required to prevent further spread within the workforce and to patients.

Sturrock, B. R., et al. (2020). "Should nasogastric tube insertion during the COVID-19 pandemic be considered as an aerosol-generating procedure?" Br J Hosp Med (Lond) **81**(6): 1-6.

Nasogastric tubes are used frequently in surgical patients for bowel decompression, provision of enteral nutritional support and preventing aspiration of gastric contents. There is no conclusive research into the risk of COVID-19 transmission associated with nasogastric tube insertion, although evidence from the severe acute respiratory syndrome outbreak appears to suggest that there is no increased risk of transmission. However, close contact with a COVID-19 patient, especially those displaying respiratory symptoms, is likely to increase the risk of transmission. Nasogastric tube insertion requires increased time spent at a patient's bedside and can also cause pharyngeal irritation, resulting in coughing. In addition, the nasogastric tube can expose the healthcare worker to potentially infectious saliva. Therefore, there is a clear need for increased evidence regarding the risk of transmission associated with nasogastric tube insertion, to ensure that such risks can be mitigated.

Su, J. Y., et al. (2020). "[The Readiness and Mental Construction of Nurses Caring for Patients With COVID-19: An Example From a Medical Center in Northern Taiwan]." Hu Li Za Zhi **67**(6): 18-24.

The COVID-19 epidemic continues to spread, and frontline health professionals have unfortunately contracted this virus because of exposure while providing clinical care. Based on the painful experience of the previous SARS epidemic in Taiwan, nurses have adopted a standard of care for infection protection that incorporates early prevention and detection. However, international public health experts remain unable to control the infectivity and variability of the COVID19 virus, which increases the mental stress on frontline nurses when performing care. In this paper, which uses a medical center in the north of Taiwan as an example, JBI clinical guidelines, infection control experience, and nurses' awareness, perceived support, and self-efficacy are applied to suggest ways to improve epidemic prevention and mental construction, enhance nurses' psychological empowerment and refection, promote role identity in nursing and self-value, establish health team awareness, strengthen infection protection concepts, and integrate infection control concepts into the nursing models to develop effective standard operating procedures for epidemic prevention management and effectively prevent the spread of the virus and maintain public health.

Subramaniam, A., et al. (2020). "Noninvasive Oxygen Strategies to Manage Confirmed COVID-19 Patients in Indian Intensive Care Units: A Survey." Indian J Crit Care Med **24**(10): 926-931.

BACKGROUND: About 5% of hospitalized coronavirus disease 2019 (COVID-19) patients will need intensive care unit (ICU) admission for hypoxemic respiratory failure requiring oxygen support. The choice between early mechanical ventilation and noninvasive oxygen therapies, such as, high-flow nasal oxygen (HFNO) and/or noninvasive positive-pressure ventilation (NPPV) has to balance the contradictory priorities of protecting healthcare workers by minimizing aerosol-generation and optimizing resource management. This survey over two timeframes aimed to explore the controversial issue of location and noninvasive oxygen therapy in non-intubated ICU patients using a clinical vignette. MATERIALS AND METHODS: An online survey was designed, piloted, and distributed electronically to Indian intensivists/anesthetists, from private hospitals, government hospitals, and medical college hospitals (the latter two referred to as first-responder hospitals), who are directly responsible for admitting/managing patients in ICU. RESULTS: Of the 204 responses (125/481 in phase 1 and 79/320 in phase 2), 183 responses were included. Respondents from first-responder hospitals were more willing to manage non-intubated hypoxemic patients in neutral pressure rooms, while respondents from private hospitals preferred negative-pressure rooms (p < 0.001). In both the phases, private hospital doctors were less comfortable to use any form of noninvasive oxygen therapies in neutral-pressure rooms compared to first-responder hospitals (low-flow oxygen therapy: 72 vs 50%, p < 0.01; HFNO: 47 vs 24%, p < 0.01 and NPPV: 38 vs 28%, p = 0.20). INTERPRETATION: Variations existed in practices among first-responder and private intensivists/anesthetists. The resource optimal private hospital intensivists/anesthetists were less comfortable using noninvasive oxygen therapies in managing COVID-19 patients. This may reflect differential resource availability necessitating resolution at national, state, and local levels. HOW TO CITE THIS ARTICLE: Subramaniam A, Haji JY, Kumar P, Ramanathan K, Rajamani A. Noninvasive Oxygen Strategies to Manage Confirmed COVID-19 Patients in Indian Intensive Care Units: A Survey. Indian J Crit Care Med 2020;24(10):926-931.

Sun, Q., et al. (2021). "Saliva as a testing specimen with or without pooling for SARS-CoV-2 detection by multiplex RT-PCR test." PLoS One **16**(2): e0243183.

BACKGROUND: Sensitive and high throughput molecular detection assays are essential during the coronavirus disease 2019 (COVID-19) pandemic, caused by the severe acute respiratory syndrome coronavirus 2 (SARS-CoV-2). The vast majority of the SARS-CoV-2 molecular assays use nasopharyngeal swab (NPS) or oropharyngeal swab (OPS) specimens collected from suspected individuals. However, using NPS or OPS as specimens has apparent drawbacks, e.g. the collection procedures for NPS or OPS specimens can be uncomfortable to some people and may cause sneezing and coughing which in turn generate droplets and/or aerosol particles that are of risk to healthcare workers, requiring heavy use of personal protective equipment. There have been recent studies indicating that self-collected saliva specimens can be used for molecular detection of SARS-CoV-2 and provides more comfort and ease of use for the patients. Here we report the performance of QuantiVirus™ SARS-CoV-2 test using saliva as the testing specimens with or without pooling. METHODS: Development and validation studies were conducted following FDA-EUA and molecular assay validation guidelines. Using SeraCare Accuplex SARS-CoV-2 reference panel, the limit of detection (LOD) and clinical performance studies were performed with the QuantiVirus™ SARS-CoV-2 test. For clinical evaluation, 85 known positive and 90 known negative clinical NPS samples were tested. Additionally, twenty paired NPS and saliva samples collected from recovering COVID-19 patients were tested and the results were further compared to that of the Abbott m2000 SARS-CoV-2 PCR assay. Results of community collected 389 saliva samples for COVID-19 screening by QuantiVirus™ SARS-CoV-2 test were also obtained and analyzed. Additionally, testing of pooled saliva samples was evaluated. RESULTS: The LOD for the QuantiVirus™ SARS-CoV-2 test was confirmed to be 100-200 copies/mL. The clinical performance studies using contrived saliva samples indicated that the positive percentage agreement (PPA) of the QuantiVirus™ SARS-CoV-2 test is 100% at 1xLOD, 1.5xLOD and 2.5xLOD. No cross-reactivity was observed for the QuantiVirus™ SARS-CoV-2 test with common respiratory pathogens. Testing of clinical samples showed a positive percentage agreement (PPA) of 100% (95% CI: 94.6% to 100%) and a negative percentage agreement (NPA) of 98.9% (95% CI: 93.1% to 99.9%). QuantiVirus™ SARS CoV-2 test had 80% concordance rate and no significant difference (p = 0.13) between paired saliva and NPS specimens by Wilcoxon matched pairs signed rank test. Positive test rate was 1.79% for 389 saliva specimens collected from local communities for COVID-19 screening. Preliminary data showed that saliva sample pooling up to 6 samples (1:6 pooling) for SARS-CoV-2 detection is feasible (sensitivity 94.8% and specificity 100%). CONCLUSION: The studies demonstrated that the QuantiVirus™ SARS-CoV-2 test has a LOD of 200 copies/mL in contrived saliva samples. The clinical performance of saliva-based testing is comparable to that of NPS-based testing. Pooling of saliva specimens for SARS-CoV-2 detection is feasible. Saliva based and high-throughput QuantiVirus™ SARS-CoV-2 test offers a highly desirable testing platform during the ongoing COVID-19 pandemic.

Sun, S., et al. (2021). "COVID-19 and healthcare system in China: challenges and progression for a sustainable future." Global Health **17**(1): 14.

With the ongoing COVID-19 outbreak, healthcare systems across the world have been pushed to the brink. The approach of traditional healthcare systems to disaster preparedness and prevention has demonstrated intrinsic problems, such as failure to detect early the spread of the virus, public hospitals being overwhelmed, a dire shortage of personal protective equipment, and exhaustion of healthcare workers. Consequently, this situation resulted in manpower and resource costs, leading to the widespread and exponential rise of infected cases at the early stage of the epidemic. To limit the spread of infection, the Chinese government adopted innovative, specialized, and advanced systems, including empowered Fangcang and Internet hospitals, as well as high technologies such as 5G, big data analysis, cloud computing, and artificial intelligence. The efficient use of these new forces helped China win its fight against the virus. As the rampant spread of the virus continues outside China, these new forces need to be integrated into the global healthcare system to combat the disease. Global healthcare system integrated with new forces is essential not only for COVID-19 but also for unknown infections in the future.

Supady, A., et al. (2021). "Ethical obligations for supporting healthcare workers during the COVID-19 pandemic." Eur Respir J **57**(2).

Based on considerations of justice, healthcare workers must be able to rely on support and protection from the societies in which they work. Prioritisation of healthcare workers for vaccines may be a way to maintain a functioning healthcare system. https://bit.ly/3pouzQu

During the coronavirus disease 2019 (COVID-19) pandemic, some healthcare facilities have, at times, reached the limits of their capacity to handle the surge in patient volume. Hospital beds and other medical resources became scarce as a consequence. Healthcare workers (HCWs), both clinical and non-clinical, were required to increase their workload, under extremely stressful circumstances.

eng

Super, J., et al. (2020). "Letter to the Editor: Impact of enhanced personal protective equipment on the physical and mental well-being of healthcare workers during COVID-19." Postgrad Med J.

Suzuki, T., et al. (2021). "Effectiveness of personal protective equipment in preventing severe acute respiratory syndrome coronavirus 2 infection among healthcare workers." J Infect Chemother **27**(1): 120-122.

INTRODUCTION: Information on the effectiveness of personal protective equipment (PPE) for preventing severe acute respiratory syndrome coronavirus 2 (SARS-CoV-2) infection among healthcare workers (HCWs), especially among HCWs with frequent contact with patients with SARS-CoV-2, is limited. METHODS: We conducted a prospective cohort study on 49 HCWs who worked in close contact with patients with SARS-CoV-2 infection. HCWs had blood samples taken every 2 weeks to test for SARS-CoV-2 antibodies using two different types of assay. RESULTS: Forty-nine participants (31 nurses, 15 doctors, 3 other workers) were enrolled. In total, 112 blood samples are obtained from participants. The median work days in 2 weeks was 9 (interquartile range (IQR): 5-10) days. In a single work day, 30 of the 49 participants (61.5%) had contact with patients with suspected or conformed SARS-CoV-2 at least 8 times, and approximately 60% of participants had more than 10 min of contact with a single patient. The median self-reported compliance to PPE was 90% (IQR: 80-100%). Seven participants tested positive for SARS-CoV-2 antibody using enzyme-linked immunosorbent assay (ELISA); however, none were seropositive for SARS-CoV-2 neutralizing antibody, so the positive ELISA results were assumed to be false-positive. CONCLUSIONS: The study provides evidence that appropriate PPE is sufficient to prevent infection amongHCWs. It is necessary to establish a system that provides a stable supply of PPE for HCWs to perform their duties.

Swaminathan, R., et al. (2020). "Impact of enhanced personal protective equipment on the physical and mental well-being of healthcare workers during COVID-19." Postgrad Med J.

BACKGROUND: The COVID-19 pandemic has necessitated the use of enhanced personal protective equipment (PPE) in healthcare workers in patient-facing roles. We describe the impact on the physical and mental well-being of healthcare professionals who use enhanced PPE consistently. METHODS: We conducted a single-centre, cross-sectional study among healthcare professionals who use enhanced PPE. A web-based questionnaire was disseminated to evaluate the effects on individuals' physical and mental well-being. Physical and mental impact was assessed through a visual analogue scale. RESULTS: Prospective analysis of the views of 72 respondents is reported. 63.9% were women and 36.1% were men. Physical impact included exhaustion, headache, skin changes, breathlessness and a negative impact on vision. Communication difficulties, somnolence, negative impact on overall performance and difficulties in using surgical instrumentation were reported. CONCLUSION: Our study demonstrates the undeniable negative impact on the front-line healthcare workers using enhanced PPE and lays the ground for larger multicentric assessments given for it to potentially be the norm for the foreseeable future.

Szabo, R. A., et al. (2021). "Covid-19 changes to maternity care: Experiences of Australian doctors." Aust N Z J Obstet Gynaecol.

BACKGROUND: The COVID-19 pandemic meant rapid changes to Australian maternity services. All maternity services have undertaken significant changes in relation to policies, service delivery and practices and increased use of personal protective equipment. AIMS: The aim of this study was to explore and describe doctors' experiences of providing maternity care during the COVID-19 pandemic in Australia. METHODS: A national online survey followed by semi-structured interviews with a cohort of participants was conducted during the first wave of the COVID-19 pandemic in Australia (May-June 2020). Participants were recruited through social media networks. Eighty-six doctors completed the survey, and eight were interviewed. RESULTS: Almost all doctors reported rapid development of new guidelines and major changes to health service delivery. Professional colleges were the main source of new information about COVID-19. Most (89%) doctors felt sufficiently informed to care for women with COVID-19. Less than half of doctors felt changes would be temporary. Doctors described workforce disruptions with associated personal and professional impacts. The ability to access and process up-to-date, evidence-based information was perceived as important. Doctors acknowledged that altered models of care had increased pregnant women's anxiety and uncertainty. All doctors described silver linings from sector changes. CONCLUSIONS: This study provides unique insights into doctors' experiences of providing maternity care during the COVID-19 pandemic in Australia. Findings have immediate relevance to the maternity sector now and into the future. Lessons learnt provide an opportunity to reshape the maternity sector to better prepare for future public health crises.

Tabah, A., et al. (2020). "Personal protective equipment and intensive care unit healthcare worker safety in the COVID-19 era (PPE-SAFE): An international survey." J Crit Care **59**: 70-75.

PURPOSE: To survey healthcare workers (HCW) on availability and use of personal protective equipment (PPE) caring for COVID-19 patients in the intensive care unit (ICU). MATERIALS AND METHOD: A web-based survey distributed worldwide in April 2020. RESULTS: We received 2711 responses from 1797 (67%) physicians, 744 (27%) nurses, and 170 (6%) Allied HCW. For routine care, most (1557, 58%) reportedly used FFP2/N95 masks, waterproof long sleeve gowns (1623; 67%), and face shields/visors (1574; 62%). Powered Air-Purifying Respirators were used routinely and for intubation only by 184 (7%) and 254 (13%) respondents, respectively. Surgical masks were used for routine care by 289 (15%) and 47 (2%) for intubations. At least one piece of standard PPE was unavailable for 1402 (52%), and 817 (30%) reported reusing single-use PPE. PPE was worn for a median of 4 h (IQR 2, 5). Adverse effects of PPE were associated with longer shift durations and included heat (1266, 51%), thirst (1174, 47%), pressure areas (1088, 44%), headaches (696, 28%), Inability to use the bathroom (661, 27%) and extreme exhaustion (492, 20%). CONCLUSIONS: HCWs reported widespread shortages, frequent reuse of, and adverse effects related to PPE. Urgent action by healthcare administrators, policymakers, governments and industry is warranted.

Tabah, A., et al. (2020). "Corrigendum to "Personal protective equipment and intensive care unit healthcare worker safety in the COVID-19 Era (PPE-SAFE): An international survey" [Journal of Critical Care, Volume 59, October 2020, Pages 70-75]." J Crit Care **63**: 280-281.

Tack, J., et al. (2020). "A survey on the impact of the COVID-19 pandemic on motility and functional investigations in Europe and considerations for recommencing activities in the early recovery phase." Neurogastroenterol Motil **32**(7): e13926.

BACKGROUND: The COVID-19 pandemic, declared by WHO on March 13, 2020, had a major global impact on the healthcare system and services. In the acute phase, the presence of the SARS-CoV-2 virus in the aerodigestive tract limited activities in the gastroenterology clinic and procedures to emergencies only. Motility and function testing was interrupted and as we enter the recovery phase, restarting these procedures requires a safety-focused approach with adequate infection prevention for patients and healthcare professionals. METHODS: We summarized knowledge on the presence of the SARS-CoV-2 virus in the aerodigestive tract and the risk of spread with motility and functional testing. We surveyed 39 European centers documenting how the pandemic affected activities and which measures they are considering for restarting these measurements. We propose recommendations based on current knowledge as applied in our center. RESULTS: Positioning of catheters for gastrointestinal motility tests carries a concern for aerosol-borne infection of healthcare workers. The risk is low with breath tests. The surveyed centers stopped almost all motility and function tests from the second half of March. The speed of restarting and the safety measures taken varied highly. CONCLUSIONS AND INFERENCES: Based on these findings, we provided recommendations and practical relevant information for motility and function test procedures in the COVID-19 pandemic era, to guarantee a high-quality patient care with adequate infection prevention.

Takhar, A., et al. (2020). "Recommendation of a practical guideline for safe tracheostomy during the COVID-19 pandemic." Eur Arch Otorhinolaryngol **277**(8): 2173-2184.

PURPOSE: The COVID-19 pandemic is placing unprecedented demand upon critical care services for invasive mechanical ventilation. There is current uncertainty regarding the role of tracheostomy for weaning ventilated patients with COVID-19 pneumonia. This is due to a number of factors including prognosis, optimal healthcare resource utilisation, and safety of healthcare workers when performing such a high-risk aerosol-generating procedure. METHODS: Literature review and proposed practical guideline based on the experience of a tertiary healthcare institution with 195 critical care admissions for COVID-19 up until 4th April 2020. RESULTS: A synthesis of the current international literature and reported experience is presented with respect to prognosis, viral load and staff safety, thus leading to a pragmatic recommendation that tracheostomy is not performed until at least 14 days after endotracheal intubation in COVID-19 patients. Practical steps to minimise aerosol generation in percutaneous tracheostomy are outlined and we describe the process and framework for setting up a dedicated tracheostomy team. CONCLUSION: In selected COVID-19 patients, there is a role for tracheostomy to aid in weaning and optimise healthcare resource utilisation. Both percutaneous and open techniques can be performed safely with careful modifications to technique and appropriate enhanced personal protective equipment. ORL-HNS surgeons can play a valuable role in forming tracheostomy teams to support critical care teams during this global pandemic.

Talarico, V., et al. (2021). "Impact of novel coronavirus Disease-19 (COVID-19) pandemic in Italian pediatric emergency departments: a national survey." Ital J Pediatr **47**(1): 47.

BACKGROUND: Coronavirus Disease-19 (COVID-19) has rapidly become a pandemic emergency, distressing health systems in each affected country. Preparation strategies for managing this pandemic have been keys to face the COVID-19 surge all over the world and all levels of care. MATERIALS AND METHODS: During the epidemic, the Italian society of pediatric emergency-urgency (SIMEUP) promoted a national survey aiming to evaluate preparedness and response of pediatric emergency departments (PED) critical in ensuring optimal management of COVID-19 cases. RESULTS: Our results suggest that Italian PED have promptly set a proactive approach to the present emergency. 98.9% of the hospitals have defined special pathways and assistive protocols concerning the management of pediatric COVID-19 cases. The highest percentage of application of the measures for preventive and protective for COVID-19 concerned the use of personal protective equipments. CONCLUSIONS: Results show that the following measures for pediatric patients, admitted in PED, have been promptly implemented throughout the whole country: eg. use of protective devices, pre-triage of patients accessing the hospital. Despite COVID-19 being a new threat, we have shown that by developing an easy-to-follow decision algorithm and clear plans for the interventional platform teams, we can ensure optimal health care workers and patients' safety.

Tan Chor Lip, H., et al. (2020). "Critical adjustments and trauma surgery trends in adaptation to COVID-19 pandemic in Malaysia." Chin J Traumatol **23**(4): 207-210.

Malaysia has one of the highest total numbers of COVID-19 infections amongst the Southeast Asian nations, which led to the enforcements of the Malaysian "Movement Control Order" to prohibit disease transmission. The overwhelming increasing amount of infections has led to a major strain on major healthcare services. This leads to shortages in hospital beds, ventilators and critical personnel protective equipment. This article focuses on the critical adaptations from a general surgery department in Malaysia which is part of a Malaysian tertiary hospital that treats COVID-19 cases. The core highlights of these strategies enforced during this pandemic are: (1) surgery ward and clinic decongestions; (2) deferment of elective surgeries; (3) restructuring of medical personnel work force; (4) utilization of online applications for tele-communication; (5) operating room (OR) adjustments and patient screening; and (6) continuing medical education and updating practices in context to COVID-19. These adaptations were important for the continuation of emergency surgery services, preventing transmission of COVID-19 amongst healthcare workers and optimization of medical personnel work force in times of a global pandemic. In addition, an early analysis on the impact of COVID-19 pandemic and lockdown measures in Malaysia towards the reduction in total number of elective/emergent/trauma surgeries performed is described in this article.

Tan, L., et al. (2020). "Personal protective equipment and evidence-based advice for surgical departments during COVID-19." ANZ J Surg **90**(9): 1566-1572.

BACKGROUND: Inconsistencies regarding the use of appropriate personal protective equipment (PPE) have raised concerns for the safety of surgical staff during the coronavirus disease 2019 (COVID-19) pandemic. This rapid review synthesizes the literature and includes input from clinical experts to provide evidence-based guidance for surgical services. METHODS: The rapid review comprised of targeted searches in PubMed and grey literature. Pertinent findings were discussed by a working group of clinical experts, and consensus recommendations, consistent with Australian and New Zealand Government guidelines, were formulated. RESULTS: There was a paucity of high-quality primary studies specifically investigating appropriate surgical PPE for healthcare workers treating patients possibly infected with COVID-19. SARS-CoV-2 is capable of aerosol, droplet and fomite transmission, making it essential to augment standard infection control measures with appropriate PPE, especially during surgical emergencies and aerosol-generating procedures. All biological material should be treated a potential source of SARS-COV-2. Staff must have formal training in the use of PPE and should be supervised by a colleague during donning and doffing. Patients with suspected or confirmed COVID-19 should wear a surgical mask during transfer to and from theatre. Potential solutions exist in the literature to extend the use of surgical P2/N95 respirators in situations of limited supply. CONCLUSION: PPE is advised for all high-risk procedures and when a patient's COVID-19 status is unknown. Surgical departments should facilitate staggered rostering, remote meeting attendance, and self-isolation of symptomatic staff. Vulnerable surgical staff should be identified and excluded from operations with a high risk of COVID-19 infection.

Tan, S. Y., et al. (2020). "The accuracy of healthcare worker versus self collected (2-in-1) Oropharyngeal and Bilateral Mid-Turbinate (OPMT) swabs and saliva samples for SARS-CoV-2." PLoS One **15**(12): e0244417.

BACKGROUND: Self-sampling for SARS-CoV-2 would significantly raise testing capacity and reduce healthcare worker (HCW) exposure to infectious droplets personal, and protective equipment (PPE) use. METHODS: We conducted a diagnostic accuracy study where subjects with a confirmed diagnosis of COVID-19 (n = 401) and healthy volunteers (n = 100) were asked to self-swab from their oropharynx and mid-turbinate (OPMT), and self-collect saliva. The results of these samples were compared to an OPMT performed by a HCW in the same patient at the same session. RESULTS: In subjects confirmed to have COVID-19, the sensitivities of the HCW-swab, self-swab, saliva, and combined self-swab plus saliva samples were 82.8%, 75.1%, 74.3% and 86.5% respectively. All samples obtained from healthy volunteers were tested negative. Compared to HCW-swab, the sensitivities of a self-swab sample and saliva sample were inferior by 8.7% (95%CI: 2.4% to 15.0%, p = 0.006) and 9.5% (95%CI: 3.1% to 15.8%, p = 0.003) respectively. The combined detection rate of self-swab and saliva had a sensitivity of 2.7% (95%CI: -2.6% to 8.0%, p = 0.321). The sensitivity of both the self-collection methods are higher when the Ct value of the HCW swab is less than 30. The specificity of both the self-swab and saliva testing was 100% (95% CI 96.4% to 100%). CONCLUSION: Our study provides evidence that sensitivities of self-collected OPMT swab and saliva samples were inferior to a HCW swab, but they could still be useful testing tools in the appropriate clinical settings.

Tan, T. W., et al. (2020). "Intra-hospital preventive principles to protect frontline healthcare workers to overcome pandemic COVID-19 in Taiwan." Crit Care **24**(1): 328.

Tan, W., et al. (2020). "Whole-Process Emergency Training of Personal Protective Equipment Helps Healthcare Workers Against COVID-19: Design and Effect." J Occup Environ Med **62**(6): 420-423.

OBJECTIVE: To develop an emergency training program of personal protective equipment (PPE) for general healthcare workers (HCWs) who may be under the threat of Corona Virus Disease 2019 (COVID-19) and evaluate the effect of the program. METHODS: A three-stage training program was designed. The complete clinical workflow together with infectious disease ward was simulated. To verify the effect of the program, an experimental training with pre- and post-test was conducted before large-scale training. RESULTS: Post-test scores were significantly improved when compared with the pre-test scores. Among all PPE, N95 respirator and protective coverall needed training most. Meanwhile, "proficiency level" and "mutual check & help" also needed to be strengthened as independent scoring points. CONCLUSION: This training program significantly improved the performances of participants. It may therefore be applied for general HCWs on a larger scale.

Tang, J., et al. (2020). "Risk factors for facial pressure sore of healthcare workers during the outbreak of COVID-19." Int Wound J **17**(6): 2028-2030.

Tangcharoensathien, V., et al. (2020). "Framework for Managing the COVID-19 Infodemic: Methods and Results of an Online, Crowdsourced WHO Technical Consultation." J Med Internet Res **22**(6): e19659.

BACKGROUND: An infodemic is an overabundance of information-some accurate and some not-that occurs during an epidemic. In a similar manner to an epidemic, it spreads between humans via digital and physical information systems. It makes it hard for people to find trustworthy sources and reliable guidance when they need it. OBJECTIVE: A World Health Organization (WHO) technical consultation on responding to the infodemic related to the coronavirus disease (COVID-19) pandemic was held, entirely online, to crowdsource suggested actions for a framework for infodemic management. METHODS: A group of policy makers, public health professionals, researchers, students, and other concerned stakeholders was joined by representatives of the media, social media platforms, various private sector organizations, and civil society to suggest and discuss actions for all parts of society, and multiple related professional and scientific disciplines, methods, and technologies. A total of 594 ideas for actions were crowdsourced online during the discussions and consolidated into suggestions for an infodemic management framework. RESULTS: The analysis team distilled the suggestions into a set of 50 proposed actions for a framework for managing infodemics in health emergencies. The consultation revealed six policy implications to consider. First, interventions and messages must be based on science and evidence, and must reach citizens and enable them to make informed decisions on how to protect themselves and their communities in a health emergency. Second, knowledge should be translated into actionable behavior-change messages, presented in ways that are understood by and accessible to all individuals in all parts of all societies. Third, governments should reach out to key communities to ensure their concerns and information needs are understood, tailoring advice and messages to address the audiences they represent. Fourth, to strengthen the analysis and amplification of information impact, strategic partnerships should be formed across all sectors, including but not limited to the social media and technology sectors, academia, and civil society. Fifth, health authorities should ensure that these actions are informed by reliable information that helps them understand the circulating narratives and changes in the flow of information, questions, and misinformation in communities. Sixth, following experiences to date in responding to the COVID-19 infodemic and the lessons from other disease outbreaks, infodemic management approaches should be further developed to support preparedness and response, and to inform risk mitigation, and be enhanced through data science and sociobehavioral and other research. CONCLUSIONS: The first version of this framework proposes five action areas in which WHO Member States and actors within society can apply, according to their mandate, an infodemic management approach adapted to national contexts and practices. Responses to the COVID-19 pandemic and the related infodemic require swift, regular, systematic, and coordinated action from multiple sectors of society and government. It remains crucial that we promote trusted information and fight misinformation, thereby helping save lives.

Tan-Loh, J. and B. M. K. Cheong (2021). "A descriptive analysis of clinical characteristics of COVID-19 among healthcare workers in a district specialist hospital." Med J Malaysia **76**(1): 24-28.

INTRODUCTION: COVID-19 is a highly transmissible respiratory virus that has affected millions of people worldwide in the span of months. The burden of disease among healthcare workers (HCW) has not been well studied despite reports of infectivity and transmission around the world. Two HCW in Hospital Teluk Intan (HTI) contracted COVID-19 while attending a social event. They were in close proximity with colleagues upon returning to work, resulting in the spread of infection among other HCW in HTI. OBJECTIVE: The objectives of this paper are to gain a better understanding of the key presenting symptoms of COVID-19 in HCWs in a district specialist hospital, to establish the proportion of symptomatic COVID-19 cases among HCWs and its severity and to determine the time taken from onset of symptoms or perceived exposure to diagnostic testing. METHODOLOGY: This is a retrospective descriptive analysis of clinical characteristics of subjects infected with COVID-19 among HCW in HTI. Their demography and clinical characteristics were recorded. RESULTS: There were 47 HCW in HTI who tested positive for COVID-19. The mean age of the patients was 37.5 years old. 7 patients (15.2%) had at least more than one comorbidity. Average duration of time from perceived close contact to onset of symptom was 4.5 days, while the mean duration of time from symptoms to first positive RT-PCR result was 3.4 days. Six patients (13.0%) were asymptomatic throughout, whereas 40 (87.0%) had at least one symptom prior to hospitalization. The most commonly reported symptoms were fever (65.2%), sore throat (39.1%) and cough (37.0%). In terms of severity of symptoms, the majority of patients experienced mild symptoms (Group 2, 52.2%). Two patients (4.3%) with multiple comorbidities had severe disease requiring ICU admission and mechanical ventilation. There were no mortalities, and the longest staying patient was hospitalized for 18 days. The high rates of infectivity among HCW in HTI can be attributed to working in close proximity while in the asymptomatic incubation phase, while no HCW directly involved in the care of COVID-19 positive patients were tested positive. CONCLUSION: We report that HCW share similar clinical characteristics of COVID-19 infection as those of non HCW patients in earlier studies. The infection can spread rapidly within healthcare settings via close contacts among infected HCWs. As such, we advocate distancing when working and usage of personal protective equipment when treating patients with respiratory illness to reduce transmission of COVID-19.

Tao, J., et al. (2020). "Emergency management of 2019 novel coronavirus: implications for the dermatology department." Br J Dermatol **182**(6): e195.

Since cases first emerged in December 2019, COVID-19 (a type of coronavirus) has rapidly become pandemic. This fast-tracked paper (published quickly) from China on COVID-19 is written by dermatologists at the epicentre of the outbreak in Wuhan. Dermatology clinic staff may be at risk because protective equipment is not routinely available, and skin lesions might possibly transmit the virus indirectly. These authors suggest preventive measures based on experience in this and previous coronavirus outbreaks. Online consultation for non-urgent patients reduces the numbers of patients attending clinics. Nurse-led triage, to identify patients with possible COVID-19, at the entrances of hospital and skin clinics directs patients with a cough or fever to a specific COVID-19 area and a dermatologist is consulted if the fever might be related to skin disease. Clinic staff wear N95 masks and observe hand hygiene during consultations. Patients are admitted to a ward only if routine blood tests and chest CT scans exclude COVID-19. Triage will not detect patients who are showing no symptoms but who are developing the disease, so the hospital should provide an on-call expert team to discuss inpatients suspected or diagnosed with COVID-19 and refer them to radiology, respiratory or intensive care colleagues as required. Confirmed cases are managed following local policies. Skin disorders in COVID-19 inpatients can usually be managed remotely using photographs, email and teleconferencing. If necessary a multidisciplinary team (a team of medical staff from different specialties) can meet in the clean area of the isolation ward. If the dermatologist must see the patient, all records should be provided in advance to minimise exposure time. With these precautions, as of 20th February 2020 no infected patients were detected in the Wuhan Dermatology Department. This is a summary of the study: Emergency management for preventing and controlling nosocomial infection of 2019 novel coronavirus: implications for the dermatology department.

Tapia, P. M., et al. (2020). "[Adherence to COVID-19 trasmission prevention measures among health care workers]." Rev Med Chil **148**(11): 1589-1597.

BACKGROUND: COVID-19 is highly transmissible, thus requiring strict measures to prevent its propagation. AIM: To report a survey about self-reported adherence to recommendations aimed to reduce the transmission of COVID-19, among health care personnel. MATERIAL AND METHODS: A cross-sectional survey among health professionals about adherence to recommendations to prevent COVID-19 transmission was carried out in a public hospital in Chile. The survey had 11 questions and was developed using Delphi methodology, according to the recommendations of the World Health Organization and the Centers for Disease Control. RESULTS: The survey was answered by 137 of 155 invited workers. Hand washing, use of personal protection equipment, use of the elbow or tissue to sneeze, out-of-hospital hand washing and exclusive use of the uniform in the hospital, had an adherence of over 90%. The adherence to face touching avoidance during working hours, and face mask use in the public areas, was over 50%. No statistical differences were observed between gender or professionals. CONCLUSIONS: The adherence reported by health care workers was adequate in most of the evaluated recommendations.

Tarfaoui, M., et al. (2020). "Additive manufacturing in fighting against novel coronavirus COVID-19." Int J Adv Manuf Technol: 1-15.

Nowadays, COVID-19 also known as novel coronavirus has become a global pandemic by causing severe respiratory tract infections in humans without any definite treatment or vaccine. Therefore, disease control measures include slowing down or averting the transfer of this viral infection from person to person. Continuous efforts are carried out to avoid the transmission of this disease to frontline healthcare personnel using single-use personal protective equipment (PPE). However, a critical shortage in this equipment around the world is becoming an alarming concern. Therefore, it is vital to present a possible alternative to overcome the acute shortage of protective gear such as face masks against this infectious disease which can have universal accessibility and is easily available. Additive manufacturing (AM), also known as 3D printing, is a possible solution to overcome the shortage of protective gear and can play a vital role in supporting their conventional production supplies during this global pandemic situation. In this context, this paper provides a brief background study of COVID-19, its conventional preventive measure, and a detailed overview regarding the latest AM efforts including designers' providers and makers in the 3D printing community. Moreover, numerous inquiries and questions such as technical factors, testing recommendations and characterization methods and biological concerns such as biocompatibility and sterilization for the AM manufactured medical devices are addressed in this paper. In the end, two examples of AM medical devices, i.e., face mask and Ambu bag ventilator, are presented and studied through numerical simulations.

Tchouaket Nguemeleu, E., et al. (2020). "Development and validation of a time and motion guide to assess the costs of prevention and control interventions for nosocomial infections: A Delphi method among experts." PLoS One **15**(11): e0242212.

BACKGROUND: Nosocomial infections place a heavy burden on patients and healthcare providers and impact health care institutions financially. Reducing nosocomial infections requires an integrated program of prevention and control using key clinical best care practices. No instrument currently exists that measures these practices in terms of personnel time and material costs. OBJECTIVE: To develop and validate an instrument that would measure nosocomial infection control and prevention best care practice costs, including estimates of human and material resources. METHODS: An evaluation of the literature identified four practices essential for the control of pathogens: hand hygiene, hygiene and sanitation, screening and additional precaution. To reflect time, materials and products used in these practices, our team developed a time and motion guide. Iterations of the guide were assessed in a Delphi technique; content validity was established using the content validity index and reliability was assessed using Kruskall Wallis one-way ANOVA of rank test. RESULTS: Two rounds of Delphi review were required; 88% of invited experts completed the assessment. The final version of the guide contains eight dimensions: Identification [83 items]; Personnel [5 items]; Additional Precautions [1 item]; Hand Hygiene [2 items]; Personal Protective Equipment [14 items]; Screening [4 items]; Cleaning and Disinfection of Patient Care Equipment [33 items]; and Hygiene and Sanitation [24 items]. The content validity index obtained for all dimensions was acceptable (> 80%). Experts statistically agreed on six of the eight dimensions. DISCUSSION/CONCLUSION: This study developed and validated a new instrument based on expert opinion, the time and motion guide, for the systematic assessment of costs relating to the human and material resources used in nosocomial infection prevention and control. This guide will prove useful to measure the intensity of the application of prevention and control measures taken before, during and after outbreak periods or during pandemics such as COVID-19.

Teah, M. K., et al. (2021). "Anaesthetic management in a patient requiring one lung ventilation during COVID-19 pandemic." BMJ Case Rep **14**(2).

Placement of a double-lumen tube to achieve one lung ventilation is an aerosol-generating procedure. Performing it on a patient with COVID-19 will put healthcare workers at high risk of contracting the disease. We herein report a case of its use in a patient with traumatic diaphragmatic rupture, who was also suspected to have COVID-19. This article aims to highlight the issues, it presented and ways to address them as well as the perioperative impact of personal protective equipment.

Team, V., et al. (2020). "Pressure Injury Prevention in COVID-19 Patients With Acute Respiratory Distress Syndrome." Front Med (Lausanne) **7**: 558696.

Coronavirus disease 2019 (COVID-19), which is caused by severe acute respiratory syndrome coronavirus 2 (SARS-CoV-2), was identified in China in December 2019 and became a pandemic in a short period of time. While most infected people might have mild symptoms, older people and people with chronic illnesses may develop acute respiratory distress syndrome (ARDS). Patients with ARDS with worsening hypoxemia require prone positioning to improve the respiratory mechanics and oxygenation. Intubated patients may stay in a prone position up to 12-16 h, increasing the risk of pressure injury (PI). Frequent skin inspections and PI risk assessment in COVID-19 patients will be challenging due to hospital infection control measures aimed to reduce the risk for health professionals. In this perspective article, we summarize the best practice recommendations for prevention of PI in SARS-CoV-2-infected ARDS patients in prone positioning. Prior to positioning patients in prone position, the main recommendations are to (1) conduct a skin assessment, (2) use pressure redistribution devices, (3) select an appropriate mattress or an overlay, (4) ensure that the endotracheal tube securing device is removed and the endotracheal tube is secured with tapes, (5) use a liquid film-forming protective dressing, and (6) lubricate the eyes and tape them closed. Once a patient is in prone position, it is recommended to (1) use the swimmer's position, (2) reposition the patient every 2 h, and (3) keep the skin clean. When the patient is repositioned to supine position, healthcare professionals are advised to (1) assess the pressure points and (2) promote early mobilization.

Tee, C. A., et al. (2020). "Psychological State and Associated Factors During the 2019 Coronavirus Disease (COVID-19) Pandemic Among Filipinos with Rheumatoid Arthritis or Systemic Lupus Erythematosus." Open Access Rheumatol **12**: 215-222.

BACKGROUND: Patients with systemic lupus erythematosus (SLE) and rheumatoid arthritis (RA) are perceived to be more vulnerable to worse COVID-19 infection outcome. Furthermore, severe shortage in hydroxychloroquine supply was experienced. OBJECTIVE: We presented the psychological responses of Filipino SLE and RA patients to the COVID-19 pandemic and shortage of hydroxychloroquine supply. METHODS: A total of 512 completed online surveys from SLE and RA patients were gathered from May 19 to 26, 2020. The online survey collected data on socio-demographics, health status, contact history, health service utilization, use of hydroxychloroquine, COVID-19 knowledge and concerns, precautionary measures, information needs, the validated Impact of Events Scale-Revised (IES-R) and Depression, Anxiety and Stress Scales (DASS-21) ratings. RESULTS: The psychological impact of COVID-19 outbreak was at least moderate in 20%. The mean IES-R score was higher among SLE (22.34, SD=14.39) than RA (18.85, SD=13.24) patients. Stress, anxiety and depression were moderate to severe in 12.3%, 38.7%, and 27.7% of respondents. The mean stress subscale score was 10.11 (SD=7.95), mean anxiety subscale score was 6.79 (SD=6.57) and mean depression subscale score was 9.03 (SD=8.77). The risk factors for adverse mental health during the COVID-19 pandemic include the presence of comorbidity of hypertension and asthma; being a healthcare worker; and presence of specific symptoms of myalgia, cough, breathing difficulty, dizziness and sore throat. The protective factors for mental health during the pandemic include satisfaction with available health information and wearing of face masks. CONCLUSION: In the third month of the pandemic in the Philippines, 20% of the respondents with lupus and RA experienced moderate to severe psychological impact. There was moderate to severe anxiety in 38.7% and moderate to severe depression in 27%. Identification of factors that affect mental health in lupus and RA is useful in implementation of effective psychological support strategies.

Teixeira, C. F. S., et al. (2020). "The health of healthcare professionals coping with the Covid-19 pandemic." Cien Saude Colet **25**(9): 3465-3474.

This work aims to systematize a set of scientific evidence presented in international papers that identify the main problems affecting health professionals directly involved in coping with the COVID-19 pandemic and point out actions and strategies for the protection and healthcare of these professionals. The risk of infection is the main issue and has led to absence from work, illness, death, and intense psychological distress, expressed in generalized anxiety and sleep disorders, fear of becoming ill and infecting colleagues and relatives. In the Brazilian reality, this work revives the analysis of the chronic problems affecting health workers, resulting from the underfinancing of the Brazilian Unified Health System (SUS), the sector's spending freeze, the deterioration of services and workforce's insecurity, and points out the acute challenges of work management and staff training, given the expanded hospital bed infrastructure and reorganization of the work process in primary care to face the pandemic, emphasizing the necessary measures for the protection and promotion of the physical and mental health of health professionals and workers.

Tekalegn, Y., et al. (2020). "Correct Use of Facemask Among Health Professionals in the Context of Coronavirus Disease (COVID-19)." Risk Manag Healthc Policy **13**: 3013-3019.

PURPOSE: Coronavirus disease (COVID-19) mainly spreads through respiratory droplets and close contacts. Wearing a facemask and other personal protective equipment (PPE) is essential in preventing the spread of COVID-19. However, the use of PPE alone does not provide a sufficient level of protection, and correct use and disposal are required. Hence, this study aimed to assess health professionals' practice regarding proper use of facemask in the perspectives of COVID-19 prevention. METHODS: A web-based online survey was conducted from June 3, 2020, to August 11, 2020, to assess health professionals' practice regarding correct use of facemask. The survey tool was prepared in Google form and distributed to the health professionals through their emails and social media pages. Data were analyzed using STATA version 14. A descriptive result was reported using frequency tables and bar charts. Factors associated with correct use of facemask were assessed using binary logistic regression model. RESULTS: A total of 368 health professionals have participated in this study. All of the participants' work involves direct contact with patients and 98 (26.6%) of them work in direct contact with COVID-19 patients daily. The level of overall correct use of facemask was 10.1% (95% CI: 7.4-13.6). Two hundred fifty-five (69.3%) do not perform hand hygiene before wearing a facemask and 238 (64.7%) do not perform hand hygiene after removing the facemask. Three hundred twenty-three (87.8%) of the study participants reuse disposable facemasks. The odds of practicing correct use of facemask were more than two times higher among health professionals who received training related to personal protective equipment utilization (AOR= 2.2, 95% CI: 1.1-4.5) compared to their counterparts. CONCLUSION: This study revealed that health professionals' practice regarding the correct use of facemask in the context of COVID-19 prevention is very low. Receiving training related to proper utilization of personal protective equipment was found to favor the correct use of facemask. In this regard, health authorities should provide training to enable the rational and correct use of facemask among healthcare workers.

Temkin, E. (2021). "Extremely low prevalence of asymptomatic COVID-19 among healthcare workers caring for COVID-19 patients in Israeli hospitals: a cross-sectional study." Clin Microbiol Infect **27**(1): 130.e131-130.e134.

OBJECTIVES: We aimed to compare the prevalence of asymptomatic coronavirus disease 19 (COVID-19) among clinical staff in designated COVID-19 units versus that among staff in similar units with no known or suspected COVID-19 patients. METHODS: We conducted a cross-sectional survey of healthcare workers (HCWs) in eight Israeli general hospitals. The survey involved a questionnaire and a PCR test for severe acute respiratory syndrome coronavirus 2 (SARS-CoV-2). We surveyed HCWs in COVID-19 units and comparison units (internal medicine and cardiology) between 30th April and 7th May 2020. RESULTS: There were 522 participants: 291 from COVID-19 units and 231 from comparison units. Only one participant (0.2%, 95%CI: 0.005-1.1%)-an asymptomatic nurse on a COVID-19 unit-tested positive for SARS-CoV-2. In participating COVID-19 units there were two symptomatic HCWs with confirmed COVID-19 in the 2 weeks before the survey; both were infected by contact with a co-worker outside of the COVID-19 unit. CONCLUSIONS: The low prevalence of asymptomatic COVID-19 among HCWs, coupled with an absence of symptomatic COVID-19 acquired during patient care, suggest that Israel's national guidelines for personal protective equipment, which are consistent with those of the World Health Organization, adequately protect HCWs.

Temsah, M. H., et al. (2020). "Knowledge, attitudes and practices of healthcare workers during the early COVID-19 pandemic in a main, academic tertiary care centre in Saudi Arabia." Epidemiol Infect **148**: e203.

As the Middle East respiratory syndrome coronavirus (MERS-CoV) continues to occur in small outbreaks in Saudi Arabia, we aimed to assess the knowledge, attitudes and intended practices of healthcare workers (HCWs) during the early stage of the COVID-19 pandemic and compare worry levels with previous findings during the MERS-CoV outbreak in 2015. We sent an adapted version of our previously published MERS-CoV questionnaire to the same cohort of HCWs at a tertiary hospital in Saudi Arabia. About 40% of our sample had previous experience with confirmed or suspected MERS-CoV patients, and those had a significantly higher knowledge score (13.16 ± 2.02 vs. 12.58 ± 2.27, P = 0.002) and higher adherence to protective hygienic practices (2.95 ± 0.80 vs. 2.74 ± 0.92, P = 0.003). The knowledge scores on COVID-19 were higher in the current cohort than the previous MERS-CoV outbreak cohort (68% vs. 79.7%, P < 0.001). HCWs from the current cohort who felt greater anxiety from COVID-19 compared to MERS-CoV were less likely to have been exposed to MERS-CoV infected/suspected cases (odds ratio (OR) = 0.646, P = 0.042) and were less likely to have attended the hospital awareness campaign on COVID-19 (OR = 0.654, P = 0.035). We concluded that previous experience with MERS-CoV was associated with increased knowledge and adherence to protective hygienic practices, and reduction of anxiety towards COVID-19.

Theoharides, T. C. and P. Conti (2020). "Dexamethasone for COVID-19? Not so fast." J Biol Regul Homeost Agents **34**(3): 1241-1243.

Recent announcements indicated, without sharing any distinct published set of results, that the corticosteroid dexamethasone may reduce mortality of severe COVID-19 patients only. The recent Coronavirus [severe acute respiratory syndrome (SARS)-CoV-2]-associated multiorgan disease, called COVID-19, has high morbidity and mortality due to autoimmune destruction of the lungs stemming from the release of a storm of pro-inflammatory cytokines. Defense against this Corona virus requires activated T cells and specific antibodies. Instead, cytokines are responsible for the serious sequelae of COVID-19 that damage the lungs. Dexamethasone is a synthetic corticosteroid approved by the FDA 1958 as a broad-spectrum immunosuppressor and it is about 30 times as active and with longer duration of action (2-3 days) than cortisone. Dexamethasone would limit the production of and damaging effect of the cytokines, but will also inhibit the protective function of T cells and block B cells from making antibodies, potentially leading to increased plasma viral load that will persist after a patient survives SARS. Moreover, dexamethasone would block macrophages from clearing secondary, nosocomial, infections. Hence, dexamethasone may be useful for the short-term in severe, intubated, COVID-19 patients, but could be outright dangerous during recovery since the virus will not only persist, but the body will be prevented from generating protective antibodies. Instead, a pulse of intravenous dexamethasone may be followed by administration of nebulized triamcinolone (6 times as active as cortisone) to concentrate in the lungs only. These corticosteroids could be given together with the natural flavonoid luteolin because of its antiviral and anti-inflammatory properties, especially its ability to inhibit mast cells, which are the main source of cytokines in the lungs. At the end, we should remember that "The good physician treats the disease; the great physician treats the patient who has the disease" [Sir William Osler's (1849-1919)].

Theuerkauff, O. and C. Hanak (2020). "Tobacco Use Cessation in Three Patients Suffering from Psychotic Disorders: The Impact of the COVID-19 Pandemic." Psychiatr Danub **32**(Suppl 1): 21-23.

BACKGROUND: Tobacco use is one of the major causes of morbidity and mortality amoung patients suffering from psychotic disorders. The association between severe COVID-19 and tobacco use is still debated. The aim of this paper is to enhance the importance of providing up to date informations about nicotine and tobacco use in connection with the SARS-CoV-2-related conditions. METHODS: We present 3 cases of sudden tobacco use cessation in 3 long-term heavy smokers receiving mental health care for chronic psychotic disorders. Fear of severe form of COVID-19 was their principal motivation. Nicotine replacement therapy and quitline counseling were provided and no major withdrawal symptoms were declared. RESULTS: As mass media later wrongly presented tobacco use as a protective factor regarding COVID-19, all three patients resumed smoking. Rigorous counseling took advantage of psychotic symptoms to correct false informations and finally promote tobacco use cessation. CONCLUSIONS: The COVID-19 pandemic might paradoxically represent a great motivational factor to quit smoking, espacially when considering patients suffering from severe mental health disorders. Beyond the terrible suffering it causes, we illustrate with a case serie that this opportunity must be exploited by mental health professionals to improve quality and life expectancy of their patients.

Thomas, J. P., et al. (2020). "Evaluating the national PPE guidance for NHS healthcare workers during the COVID-19 pandemic." Clin Med (Lond) **20**(3): 242-247.

Tragically, many of the infections and deaths recorded in the global coronavirus disease 2019 (COVID-19) pandemic have occurred in healthcare workers. Some have attributed this to inadequate provision of personal protective equipment (PPE). In the UK, several organisations have voiced their concerns that the national PPE guidance issued by Public Health England is inadequate. Despite recent revisions to these guidelines, concerns remain that they offer insufficient protection to frontline NHS healthcare workers. In this report, we evaluate whether these concerns are merited, through critical appraisal of the available evidence, review of international PPE guidance, and consideration of the ethical implications.

Thomas-Rüddel, D., et al. (2020). "Coronavirus disease 2019 (COVID-19): update for anesthesiologists and intensivists March 2020." Anaesthesist: 1-10.

The current outbreak of coronavirus disease (COVID-19) has reached Germany. The majority of people infected present with mild disease, but there are severe cases that need intensive care. Unlike other acute infectious diseases progressing to sepsis, the severe courses of COVID19 seemingly show prolonged progression from onset of first symptoms to life-threatening deterioration of (primarily) lung function. Diagnosis relies on PCR using specimens from the respiratory tract. Severe ARDS reflects the hallmark of a critical course of the disease. Preventing nosocomial infections (primarily by correct use of personal protective equipment) and maintenance of hospitals' operational capability are of utmost importance. Departments of Anaesthesia, Intensive Care and emergency medicine will envisage major challenges.

Publisher: Abstract available from the publisher.

ger

Thomas-Rüddel, D., et al. (2020). "[Coronavirus disease 2019 (COVID-19): update for anesthesiologists and intensivists March 2020]." Anaesthesist **69**(4): 225-235.

The current outbreak of coronavirus disease (COVID-19) has reached Germany. The majority of people infected present with mild disease, but there are severe cases that need intensive care. Unlike other acute infectious diseases progressing to sepsis, the severe courses of COVID19 seemingly show prolonged progression from onset of first symptoms to life-threatening deterioration of (primarily) lung function. Diagnosis relies on PCR using specimens from the respiratory tract. Severe ARDS reflects the hallmark of a critical course of the disease. Preventing nosocomial infections (primarily by correct use of personal protective equipment) and maintenance of hospitals' operational capability are of utmost importance. Departments of Anaesthesia, Intensive Care and emergency medicine will envisage major challenges.

Thome, J., et al. (2020). "The impact of the COVID-19 outbreak on the medico-legal and human rights of psychiatric patients." Eur Psychiatry **63**(1): e50.

The COVID-19 pandemic has raised significant concerns for population mental health and the effective provision of mental health services in the light of increased demands and barriers to service delivery [1]. Particular attention is being directed toward the possible neuropsychiatric sequelae of both COVID-19 and of the stringent societal mitigation steps deployed by national governments, concerns that are informed by historical increases in the incidence of psychotic disorders following influenza pandemics [2]. However, so far there has been scant attention paid to other important areas of psychiatry during COVID-19, including medico-legal aspects and human rights. In this paper, we discuss the legal implications for psychiatry of the COVID-19 pandemic and report a novel situation in which psychiatric patients may experience diminution of their statutory protections. We believe that this represents a paradigm shift in psychiatric care and that the consideration of the fundamental rights of psychiatric patients as "less important" than infection control measures compel mental health professionals to "advocate for … patients and their caregivers" in this time of crisis [1].

Tian, C., et al. (2021). "Risk factors and protective measures for healthcare worker infection during highly infectious viral respiratory epidemics: a systematic review and meta-analysis." Infect Control Hosp Epidemiol: 1-102.

OBJECTIVE: To investigate risk factors for HCW infection in viral respiratory pandemics (SARS-CoV-2, MERS, SARS CoV-1, influenza A H1N1, influenza H5N1) and improve understanding of HCW risk management amidst the COVID-19 pandemic. DESIGN: Systematic review and meta-analysis. METHODS: MEDLINE, EMBASE, CINAHL, and Cochrane CENTRAL databases were searched from conception until July 2020 for studies comparing infected HCWs (cases) and non-infected HCWs (controls) and risk factors for infection. Outcomes included HCW types, infection prevention practices, and medical procedures. Pooled effect estimates with pathogen-specific stratified meta-analysis and inverse variance meta-regression analysis were completed. GRADE framework was used to rate certainty of evidence. PROSPERO (CRD42020176232) 6 April 2020. RESULTS: Fifty-four comparative studies were included (n=191,004 HCWs). Compared to non-frontline HCWs, frontline HCWs were at increased infection risk (OR 1.66 95%CI 1.24 to 2.22) and greater for HCWs involved in endotracheal intubations (risk difference [95%CI]: 35.2% [21.4 to 47.9]). Use of gloves, gown, surgical mask, N95 respirator, face protection, and infection training were each strongly protective against infection. Meta-regression showed reduced infection risk in frontline HCWs working in facilities with infection designated wards (OR -1.04, 95%CI -1.53 to -0.33, p=0.004) and performing aerosol-generating medical procedures in designated centres (OR -1.30 95%CI -2.52 to -0.08; p=0.037). CONCLUSIONS: During highly infectious respiratory pandemics, widely available protective measures such as use of gloves, gowns, and face masks are strongly protective against infection and should be instituted, preferably in dedicated settings, to protect frontline HCW during waves of respiratory virus pandemics.

Tian, Q., et al. (2020). "Endoscopic mask innovation and protective measures changes during the coronavirus disease-2019 pandemic: Experience from a Chinese hepato-biliary-pancreatic unit." Dig Endosc **32**(7): 1105-1110.

Endoscopy is widely used as a clinical diagnosis and treatment method for certain hepatobiliary and pancreatic diseases. However, due to the distinctive epidemiological characteristics of severe acute respiratory syndrome coronavirus 2, the virus causing coronavirus disease-2019 (COVID-19), healthcare providers are exposed to the patient's respiratory and gastrointestinal fluids, rendering endoscopy a high risk for transmitting a nosocomial infection. This article introduces preventive measures for endoscopic treatment enacted in our medical center during COVID-19, including the adjustment of indications, the application of endoscope protective equipment, the design and application of endoscopic masks and splash-proof films, and novel recommendations for bedside endoscope pre-sterilization.

Tian, T., et al. (2020). "Mental health burden of frontline health professionals treating imported patients with COVID-19 in China during the pandemic." Psychol Med: 1-2.

Tian, Y., et al. (2021). "The Status of Occupational Protection During COVID-19 Pandemic: Knowledge, Attitudes, and Practice of Healthcare Workers in Endoscopy Units, China." Front Public Health **9**: 632608.

Background: SARS-CoV-2 spreads rapidly around the world, and some patients present gastrointestinal symptoms. The existence of the virus in the gastrointestinal tract makes digestive endoscopy a high-risk operation, which associated with an increased risk of infection rate in healthcare workers. This study aimed at exploring current knowledge, practice and attitudes of healthcare workers in endoscopy units in China regarding the status of occupational protection during COVID-19 pandemic. Methods: A cross-sectional study of a national online survey involving 717 healthcare workers in endoscopy units from 94 medical structures in 24 provinces and municipalities around China was conducted online via a questionnaire platform called Wenjuanxing (wjx.cn). The data were analyzed using correlation approaches, Kruskal-Wallis test for independent samples, and linear regression models. Results: Most Chinese healthcare workers in endoscopy units had a good knowledge of COVID-19 (median: 10; range: 7-12), showed a strikingly positive attitude (median: 65; range: 39-65), and carried out good practice (median: 47; range: 14-50) in strengthening the protection, disinfection and management of COVID-19. In terms of attitudes, female staff was more concerned about protection against COVID-19 than male staff (KW = 8.146, P = 0.004). Nurses performed better in both attitude (KW = 2.600, P = 0.009) and practice (KW = 6.358, P < 0.001) than endoscopic physicians when carrying out personal protection, patient care and environmental disinfection against SARS-CoV-2 infection. More positive attitudes in protection were related to better protective behavior in endoscopic daily medical work (r = 0.312; P < 0.001). Conclusion: The findings of this study suggest that Chinese endoscopy healthcare workers have an excellent mastery of knowledge about COVID-19, which is transformed into positive beliefs and attitudes, contributing to good practice during daily endoscopic procedures. Medical staff may benefit from further education. With the gradual normalization amid the ongoing COVID-19 pandemic, protection and management in endoscopy units may be changed accordingly.

Tian, Z., et al. (2020). "Personal protective equipment (PPE) and infection among healthcare workers - What is the evidence?" Int J Clin Pract **74**(11): e13617.

BACKGROUND: The worldwide outbreak of coronavirus disease-19 (COVID-19) has already put healthcare workers (HCWs) at a high risk of infection. The question of how to give HCWs the best protection against infection is a priority. METHODS: We searched systematic reviews and original studies in Medline (via Ovid) and Chinese Wan Fang digital database from inception to May, 2020, using terms 'coronavirus', 'health personnel', and 'personal protective equipment' to find evidence about the use of full-body PPEs and other PPEs by HCW exposed highly infectious diseases. RESULTS: Covering more of the body could provide better protection for HCWs. Of importance, it is not just the provision of PPE but the skills in donning and doffing of PPE that are important, this being a key time for potential transmission of pathogen to the HCW and in due time from them to others. In relation to face masks, the evidence indicates that a higher-level specification of face masks and respirators (such as N95) seems to be essential to protect HCWs from coronavirus infection. In community setting, the use of masks in the case of well individuals could be beneficial. Evidence specifically around PPE and protection from the COVID-19 virus is limited. CONCLUSION: Covering more of the body, and a higher-level specification of masks and respirators could provide better protection for HCWs. Community mask usecould be beneficial. High quality studies still need to examine the protection of PPE against COVID-19.

Tiri, B., et al. (2020). "Antimicrobial Stewardship Program, COVID-19, and Infection Control: Spread of Carbapenem-Resistant Klebsiella Pneumoniae Colonization in ICU COVID-19 Patients. What Did Not Work?" J Clin Med **9**(9).

The Italian burden of disease associated with infections due to antibiotic-resistant bacteria has been very high, largely attributed to Carbapenem-Resistant Klebsiella pneumoniae (CR-Kp). The implementation of infection control measures and antimicrobial stewardship programs (ASP) has been shown to reduce healthcare-related infections caused by multidrug resistance (MDR) germs. Since 2016, in our teaching hospital of Terni, an ASP has been implemented in an intensive care unit (ICU) setting, with the "daily-ICU round strategy" and particular attention to infection control measures. We performed active surveillance for search patients colonized by Carbapenem-Resistant Enterobacteriaceae (CRE). In March 2020, coronavirus disease 2019 (COVID-19) arrived and the same ICU was reserved only for COVID-19 patients. In our retrospective observational study, we analyzed the bimonthly incidence of CRE colonization patients and the incidence of CRE acquisition in our ICU during the period of January 2019 to June 2020. In consideration of the great attention and training of all staff on infection control measures in the COVID-19 era, we would have expected a clear reduction in CRE acquisition, but this did not happen. In fact, the incidence of CRE acquisition went from 6.7% in 2019 to 50% in March-April 2020. We noted that 67% of patients that had been changed in posture with prone position were colonized by CRE, while only 37% of patients that had not been changed in posture were colonized by CRE. In our opinion, the high intensity of care, the prone position requiring 4-5 healthcare workers (HCWs), equipped with personal protective equipment (PPE) in a high risk area, with extended and prolonged contact with the patient, and the presence of 32 new HCWs from other departments and without work experience in the ICU setting, contributed to the spread of CR-Kp in our ICU, determining an increase in CRE acquisition colonization.

Tirupakuzhi Vijayaraghavan, B. K., et al. (2020). "Hydroxychloroquine plus personal protective equipment versus standard personal protective equipment alone for the prevention of COVID-19 infections among frontline healthcare workers: the HydrOxychloroquine Prophylaxis Evaluation(HOPE) trial: A structured summary of a study protocol for a randomized controlled trial." Trials **21**(1): 754.

OBJECTIVES: To evaluate the effect of the combination of hydroxychloroquine (HCQ) and standard personal protective equipment (PPE) compared to the use of standard personal protective equipment alone on the proportion of laboratory confirmed COVID-19 infections among frontline healthcare workers(HCWs) in India TRIAL DESIGN: HOPE is an investigator initiated multi-centre open-label parallel group randomized controlled trial. PARTICIPANTS: All HCWs currently working in an environment with direct exposure to patients with confirmed COVID-19 infection are eligible to participate in the trial. The trial aims to be conducted across 20-30 centres (public and private hospitals) in India. HCWs who decline consent, who have a confirmed COVID-19 infection, those who are already on chloroquine/HCQ for any indication, or if pregnant or breast-feeding, or have known QT prolongation or are on medications that when taken with HCQ can prolong the QTc will be excluded. INTERVENTION AND COMPARATOR: The interventions to be compared in this trial are standard practice (use of recommended PPE) and HCQ plus standard practice. In the standard practice arm, HCWs will use recommended PPE as per institutional guidelines and based on their roles. They will be discouraged from taking HCQ to prevent contamination and contacted every week for the duration of the study to ascertain if they have taken any HCQ. Any such use will be reported as a protocol violation. In the intervention arm, HCWs will be administered 800mg of HCQ as a loading dose on the day of randomization (as two 400mg doses 12hrs apart) and subsequently continued on 400mg once a week for 12 weeks. This will be in addition to the use of recommended PPE as per institutional guidelines and based on their roles. HCWs will collect the drug once every week from designated research and pharmacy staff at site. A weekly phone reminder will be provided to participants in this arm to ensure compliance. An ECG will be performed between 4-6 weeks in this arm and if the QTc is prolonged (greater than 450milliseconds), the drug will be stopped. Follow-up will however continue. Participants in both arms will receive a weekly phone call for evaluation of the primary outcome, to monitor protocol compliance and development of any adverse events (in the HCQ group). MAIN OUTCOMES: Participants will be followed on a weekly basis. The primary outcome is the proportion of HCWs developing laboratory confirmed COVID-19 infection within 6 months of randomization. We will also evaluate a number of secondary outcomes, including hospitalization related to suspected/confirmed COVID-19 infection, intensive care unit or high-dependency unit admission due to suspected/confirmed COVID-19 infection, all-cause mortality, need for organ support ( non-invasive or invasive ventilation, vasopressors and renal replacement therapy), ICU and hospital length of stay, readmission, days off work and treatment-related adverse events. RANDOMISATION: Randomisation will be conducted through a password-protected, secure website using a central, computer-based randomisation program. Randomisation will be stratified by participating institutions and by the role of HCW - nursing, medical and other. Participants will be randomised 1:1 to either standard practice only or HCQ plus standard practice. Allocation concealment is maintained by central web-based randomisation BLINDING (MASKING): This is an unblinded study: study assigned treatment will be known to the research team and participant. Bias will be mitigated through an objective end point (laboratory confirmed COVID-19 infection). NUMBERS TO BE RANDOMISED (SAMPLE SIZE): A total of 6,950 HCWs will be enrolled (3475 to the intervention) and (3475 to the standard practice group) to detect a 25% relative reduction, or 2.5% absolute reduction, in the infection rate from an estimated baseline infection rate of 10%, with 80% statistical power using a two-sided test at 5% level of significance. Available data from China and Italy indicate that the rate of infection among frontline healthcare workers varies between 4% to 12%. We therefore assumed a baseline infection rate of 10% among HCWs. This sample size allows for a potential loss to follow-up rate of 10% and a potential non-compliance rate of 10% in both the treatment and control arms. TRIAL STATUS: HOPE protocol version 3.0 dated June 3(rd) 2020. Recruitment started on 29(th) June 2020 and currently 56 participants have been enrolled. Planned completion of enrolment is January 31(st) 2021. TRIAL REGISTRATION: Clinical Trials Registry of India: CTRI/2020/05/025067 (prospectively registered) Date of registration: 6(th) May 2020 FULL PROTOCOL: The full protocol is attached as an additional file, accessible from the Trials website (Additional file 1). In the interest of expedited dissemination of this material, the familiar formatting has been eliminated; this Letter serves as a summary of the key elements of the full protocol. The study protocol has been reported in accordance with the Standard Protocol Items: Recommendations for Clinical Interventional Trials (SPIRIT) guidelines (Additional file 2).

Tirupathi, R., et al. (2020). "Comprehensive review of mask utility and challenges during the COVID-19 pandemic." Infez Med **28**(suppl 1): 57-63.

Masks are widely discussed during the course of the ongoing COVID-19 pandemic. Most hospitals have implemented universal masking for their healthcare workers, and the Center for Disease Control currently advises even the general public to wear cloth masks when outdoors. The pertinent need for masks arises from plausible dissemination of the SARS-CoV-2 through close contacts, as well as the possibility of virus transmission from asymptomatic, pre-symptomatic, and mildly symptomatic individuals. Given current global shortages in personal protective equipment, the efficacy of various types of masks: N95 respirators, surgical masks, and cloth masks are researched. To accommodate limited supplies, techniques for extended use, reuse, and sterilization of masks are strategized. However, masks alone may not greatly slow down the COVID-19 pandemic unless they are coupled with adequate social distancing, diligent hand hygiene, and other proven preventive measures.

Tíscar-González, V., et al. (2020). "[Perceptions and experiences of 7-8 year old schoolchildren in the Basque Country during the COVID-19 health alert]." Gac Sanit.

OBJECTIVE: To explore the experiences of schoolchildren in an exceptional health alert situation in which strict confinement measures have been applied to the population. METHOD: Qualitative exploratory study through the analysis of illustrations carried out by schoolchildren aged 7 to 8. The analysis was carried out according to Rose's theoretical framework. Descriptive analysis of socio-demographic data was performed. RESULTS: Five categories emerged from the analysis of the content: 1) feelings of sadness and fear; 2) importance of the family nucleus, safety and protection; 3) sedentary living habits during confinement; 4) housing infrastructure and social inequalities (life through a window); and 5) social response, collective struggle and through health professionals. CONCLUSIONS: The COVID-19 pandemic situation and the strict confinement measures have had a significant impact on the school population, who had experienced the greatest restrictions. Household infrastructure has been shown to be a clear differentiator that deepens social inequalities and the impact that the situation has on school children. More studies are needed on the long-term psychosocial impact of this situation on their physical and mental health.

Publisher: Abstract available from the publisher.

spa

Tomita, Y., et al. (2020). "BCG vaccine may generate cross-reactive T cells against SARS-CoV-2: In silico analyses and a hypothesis." Vaccine **38**(41): 6352-6356.

The world is facing the rising emergency of SARS-CoV-2. The outbreak of COVID-19 has caused a global public health and economic crisis.Recent epidemiological studies have shown that a possible association of BCG vaccination program with decreased COVID-19-related risks, suggesting that BCG may provide protection against COVID-19. Non-specific protection against viral infections is considered as a main mechanism of BCG and clinical trials to determine whether BCG vaccine can protect healthcare workers from the COVID-19 are currently underway. We hypothesized that BCG may carry similar T cell epitopes with SARS-CoV-2 and evaluated the hypothesis by utilizing publicly available database and computer algorithms predicting human leukocyte antigen (HLA) class I-binding peptides. We foundthatBCG contains similar 9-amino acid sequences with SARS-CoV-2. These closely-related peptides had moderate to high binding affinity for multiple common HLA class I molecules, suggesting that cross-reactive T cells against SARS-CoV-2 could be generated by BCG vaccination.

Toomey, E. C., et al. (2021). "Extended use or reuse of single-use surgical masks and filtering face-piece respirators during the coronavirus disease 2019 (COVID-19) pandemic: A rapid systematic review." Infect Control Hosp Epidemiol **42**(1): 75-83.

BACKGROUND: Shortages of personal protective equipment during the coronavirus disease 2019 (COVID-19) pandemic have led to the extended use or reuse of single-use respirators and surgical masks by frontline healthcare workers. The evidence base underpinning such practices warrants examination. OBJECTIVES: To synthesize current guidance and systematic review evidence on extended use, reuse, or reprocessing of single-use surgical masks or filtering face-piece respirators. DATA SOURCES: We used the World Health Organization, the European Centre for Disease Prevention and Control, the US Centers for Disease Control and Prevention, and Public Health England websites to identify guidance. We used Medline, PubMed, Epistemonikos, Cochrane Database, and preprint servers for systematic reviews. METHODS: Two reviewers conducted screening and data extraction. The quality of included systematic reviews was appraised using AMSTAR-2. Findings were narratively synthesized. RESULTS: In total, 6 guidance documents were identified. Levels of detail and consistency across documents varied. They included 4 high-quality systematic reviews: 3 focused on reprocessing (decontamination) of N95 respirators and 1 focused on reprocessing of surgical masks. Vaporized hydrogen peroxide and ultraviolet germicidal irradiation were highlighted as the most promising reprocessing methods, but evidence on the relative efficacy and safety of different methods was limited. We found no well-established methods for reprocessing respirators at scale. CONCLUSIONS: Evidence on the impact of extended use and reuse of surgical masks and respirators is limited, and gaps and inconsistencies exist in current guidance. Where extended use or reuse is being practiced, healthcare organizations should ensure that policies and systems are in place to ensure these practices are carried out safely and in line with available guidance.

Torres, A. E., et al. (2020). "Ultraviolet-C and other methods of decontamination of filtering facepiece N-95 respirators during the COVID-19 pandemic." Photochem Photobiol Sci **19**(6): 746-751.

During global health emergencies such as the current COVID-19 pandemic, the decontamination of single-use personal protective equipment (PPE) becomes a necessary means to keep up with the growing demand from healthcare workers and patients alike. Many unverified methods are being considered, which can pose the risk of incomplete decontamination and lead to catastrophic results. Several factors come into play when determining the suitability of such methods including the quality of the decontamination technique, the targeted pathogen, cost, ease of installation and use, rate of sterilization, and the surface or material to be sterilized. The germicidal properties of ultraviolet-C are well known. This review will cover the most commonly described methods for the sterilization of N95 respirators, namely, ultraviolet germicidal irradiation, hydrogen peroxide vaporization, microwave-generated steaming, and dry heating. These techniques have been tested previously and have demonstrated efficacy in reducing or inactivating viral and bacterial pathogens, although testing against SARS-CoV-2 specifically has not been done. Moreover, it must be emphasized that proper disposal after a single use is still ideal under normal circumstances.

Torres, A. E., et al. (2020). "Ultraviolet-C and other methods of decontamination of filtering facepiece N-95 respirators during the COVID-19 pandemic." Photochem Photobiol Sci.

During global health emergencies such as the current COVID-19 pandemic, the decontamination of single-use personal protective equipment (PPE) becomes a necessary means to keep up with the growing demand from healthcare workers and patients alike. Many unverified methods are being considered, which can pose the risk of incomplete decontamination and lead to catastrophic results. Several factors come into play when determining the suitability of such methods including the quality of the decontamination technique, the targeted pathogen, cost, ease of installation and use, rate of sterilization, and the surface or material to be sterilized. The germicidal properties of ultraviolet-C are well known. This review will cover the most commonly described methods for the sterilization of N95 respirators, namely, ultraviolet germicidal irradiation, hydrogen peroxide vaporization, microwave-generated steaming, and dry heating. These techniques have been tested previously and have demonstrated efficacy in reducing or inactivating viral and bacterial pathogens, although testing against SARS-CoV-2 specifically has not been done. Moreover, it must be emphasized that proper disposal after a single use is still ideal under normal circumstances.

Tran, T. V., et al. (2020). "Impacts and interactions of COVID-19 response involvement, health-related behaviours, health literacy on anxiety, depression and health-related quality of life among healthcare workers: a cross-sectional study." BMJ Open **10**(12): e041394.

OBJECTIVES: We examined impacts and interactions of COVID-19 response involvement, health-related behaviours and health literacy (HL) on anxiety, depression, and health-related quality of life (HRQoL) among healthcare workers (HCWs). DESIGN: A cross-sectional study was conducted. Data were collected 6 April to 19 April 2020 using online-based, self-administered questionnaires. SETTING: 19 hospitals and health centres in Vietnam. PARTICIPANTS: 7 124 HCWs aged 21-60 years. RESULTS: The COVID-19 response-involved HCWs had higher anxiety likelihood (OR (95% CI)=4.41 (3.53 to 5.51)), higher depression likelihood (OR(95% CI)=3.31 (2.71 to 4.05)) and lower HRQoL score (coefficient, b(95% CI)=-2.14 (-2.89 to -1.38)), compared with uninvolved HCWs. Overall, HCWs who smoked or drank at unchanged/increased levels had higher likelihood of anxiety, depression and lower HRQoL scores; those with unchanged/healthier eating, unchanged/more physical activity and higher HL scores had lower likelihood of anxiety, depression and higher HRQoL scores. In comparison to uninvolved HCWs who smoked or drank at never/stopped/reduced levels, involved HCWs with unchanged/increased smoking or drinking had lower anxiety likelihood (OR(95% CI)=0.34 (0.14 to 0.83)) or (OR(95% CI)=0.26 (0.11 to 0.60)), and lower depression likelihood (OR(95% CI)=0.33 (0.15 to 0.74)) or (OR(95% CI)=0.24 (0.11 to 0.53)), respectively. In comparison with uninvolved HCWs who exercised at never/stopped/reduced levels, or with those in the lowest HL quartile, involved HCWs with unchanged/increased exercise or with one-quartile HL increment reported lower anxiety likelihood (OR(95% CI)=0.50 (0.31 to 0.81)) or (OR(95% CI)=0.57 (0.45 to 0.71)), lower depression likelihood (OR(95% CI)=0.40 (0.27 to 0.61)) or (OR(95% CI)=0.63 (0.52 to 0.76)), and higher HRQoL scores (b(95% CI)=2.08 (0.58 to 3.58)), or (b(95% CI)=1.10 (0.42 to 1.78)), respectively. CONCLUSIONS: Physical activity and higher HL were found to protect against anxiety and depression and were associated with higher HRQoL. Unexpectedly, smoking and drinking were also found to be coping behaviours. It is important to have strategic approaches that protect HCWs' mental health and HRQoL.

Traversari, L. and M. Camilli (2020). "[Efficacy of serological tests for COVID-19 in asymptomatic HD patients: the experience of an Italian hemodialysis unit]." G Ital Nefrol **37**(5).

We report the brief experience of the Nephrology Center located in a "no-COVID" Hospital in Massa Marittima. We describe the actions taken to prevent the transmission of the virus SARS-CoV-2 among hemodialysis patients and healthcare workers and the methods for diagnosing COVID-19, with particular attention to serological tests and nasopharyngeal swabs in asymptomatic subjects. The detection of IgM and IgG antibodies through the serological test performed on 34 patients, all negative for nasopharyngeal swabs, showed positivity in 41,18% of cases. These have been classified as false positives following repeated negative nasopharyngeal swabs, the evaluation of clinical and epidemiological history and of clinical manifestations and, finally, a second serological test performed after 18 days, which resulted negative for all patients. Interpreting serological tests is not easy; the strategies for diagnosis should include clinical and epidemiological history and clinical manifestations, as well as the results of confirmation tests and the evaluation over a precise observation period. Otherwise, there is a risk of considering as protected by antibodies subjects that are in fact false positives.

Trouillet-Assant, S., et al. (2020). "Assessment of serological techniques for screening patients for COVID-19 (COVID-SER): a prospective, multicentric study." BMJ Open **10**(11): e041268.

INTRODUCTION: The COVID-19 pandemic caused by SARS-CoV-2 threatens global public health, and there is an urgent public health need to assess acquired immunity to SARS-CoV-2. Serological tests might provide results that can be complementary to or confirm suspected COVID-19 cases and reveal previous infection. The performance of serological assays (sensitivity and specificity) has to be evaluated before their use in the general population. The neutralisation capacity of the produced antibodies also has to be evaluated. METHODS AND ANALYSIS: We set up a prospective, multicentric clinical study to evaluate the performance of serological kits among a population of healthcare workers presenting mild symptoms suggestive of SARS-CoV-2 infection. Four hundred symptomatic healthcare workers will be included in the COVID-SER study. The values obtained from a control cohort included during the prepandemic time will be used as reference. A workflow was set up to study serological response to SARS-CoV-2 infection and to evaluate antibody neutralisation capacity in patients with a confirmed SARS-CoV-2 infection. The sensitivity and specificity of the tests will be assessed using molecular detection of the virus as a reference. The measurement of IgM and IgG antibodies will be performed once per week for 6 consecutive weeks and then at 6, 12, 18, 24 and 36 months after the diagnosis. The kinetics of IgM and IgG will determine the optimal period to perform serological testing. The proportion of false negative PCR tests in symptomatic subjects will be determined on the basis of subsequent seroconversions. ETHICS AND DISSEMINATION: Ethical approval has been obtained from the national review board for biomedical research in April 2020 (Comité de Protection des Personnes Sud Méditerranée I, Marseille, France) (ID RCB 2020-A00932-37). Results will be disseminated through presentations at scientific meetings and publications in peer-reviewed journals. TRIAL REGISTRATION NUMBER: NCT04341142.

Tsang, Y., et al. (2020). "Meeting the challenges imposed by COVID-19: Guidance document by the ESTRO Radiation TherapisT Committee (RTTC)." Tech Innov Patient Support Radiat Oncol **15**: 6-10.

COVID-19 - a novel coronavirus was firstly reported in December 2019. In response to threats imposed by COVID-19, the European society for radiotherapy and oncology Radiation TherapisT Committee (ESTRO RTTC) prepared this document in conjunction with an infographic with four main domains: patient care, RTT workflow, remote working and RT practice. In the context of the current COVID-19 pandemic, RTTs should be empowered with appropriate guidance and personal protection equipment in order to provide a safe radiotherapy service by limiting potential viral exposure to patients, healthcare workers and general public.

Tsiga-Ahmed, F. I., et al. (2021). "COVID 19: Evaluating the Knowledge, Attitude and Preventive Practices of Healthcare Workers in Northern Nigeria." Int J MCH AIDS **10**(1): 88-97.

BACKGROUND: Although much is known about the rapidly spreading COVID-19 disease, a lot of knowledge is still evolving. The knowledge, attitude and practice (KAP) of healthcare workers (HCWs) towards COVID-19 remain key in protecting themselves and in fighting the "war" against the disease. This study assessed the KAP of HCWs in Kano, northern Nigeria. METHODOLOGY: A cross-section of different cadre of healthcare workers was recruited online via google forms. Using a link, the participants completed an adapted from a similar study, pre-tested questionnaire on KAP regarding COVID-19. Predictors of KAP were assessed using logistic regression modelling. RESULTS: Among the 651 HCWs invited to participate, 233 respondents responded giving a response rate of 35.8%. Of these, 195 (83.7%) had good knowledge, 183 (78.9%) had a positive attitude and 180 (77.6%) had good practice towards prevention of COVID-19. The odds of having good knowledge were significantly lower among Community Health Officers/Community Health Extension workers (aOR=0.2, 95% CI: 0.1-0.6;p<0.001) and other health workers compared to doctors. Positive attitude was predicted by good knowledge (aOR=4.8, 95% CI:1.7-010.2;p=0.003), being in the fifth decade of life (aOR=5.5, 95% CI: 1.1-29.3, p=0.04), female gender (aOR=3.0, 95% CI: 1.1-8.3;p=0.04), Christian faith (aOR=7.0, 95% CI: 1.3-40.4; p=0.03), and having a bachelors' or medical degree (aOR=4.6, 95% CI: 1.3-16.5).The only predictor of good practice was good knowledge on COVID-19 (aOR=7.8, 95% CI 2.8-12.4;p<0.001). CONCLUSION AND GLOBAL HEALTH IMPLICATIONS: Majority of the HCWs at the study site have good knowledge, attitude and practice regarding COVID-19. Continuous dissemination of information on prevention of spread of COVID-19 to all HCWs will strengthen the health workforce in the fight against it.

Tulchinsky, M. and S. Osmany (2020). "The American College of Nuclear Medicine Guidance on Operating Procedures for a Nuclear Medicine Facility During COVID-19 Pandemic." Clin Nucl Med.

The novel coronavirus 2 pandemic is causing widespread disruption in everyday life necessitating urgent and radical adaptations in operating procedures at Nuclear Medicine facilities. The potential for causing severe illness, COVID-19, calls for strict observance of preventive measures aimed to mitigate the spread of the virus. The threat of COVID-19 is particularly serious as there is no vaccine and no specific antiviral therapy. Further complications are introduced by shortages of personal protective equipment for healthcare workers who have direct contact with patients and effective testing to identify infected patients, raising the need for delaying some testing and therapies. Certain vulnerable segments of the general population have been identified (advanced age and certain comorbidities), which should heighten further their preventive efforts. Therefore, this guidance is intended to be operationalized depending on a facility's specific needs and local disease prevalence.

Tuli, I. P., et al. (2020). "Diagnostic and therapeutic endonasal rhinologic procedures generating aerosol during COVID-19 pandemic: a systematized review." Braz J Otorhinolaryngol.

INTRODUCTION: Most rhinologic procedures, particularly endoscopic sinonasal procedures, are liable to produce aerosols. The severe acute respiratory syndrome coronavirus 2 (SARS-Co V-2) transmits via respiratory droplets, but the degree of its spread through airborne routes by aerosol is unclear. OBJECTIVE: The aim of this article is to counsel rhinologists on how to modify their conventional practice during the COVID-19 pandemic by prioritising the need of procedures, identifying aerosol- generating procedures and using precise personal protection equipment for various endonasal procedures. METHODS: We did a review of articles indexed for MEDLINE on PubMed, ENT Cochrane, DOAJ and Web of Science databases using the keywords nasal endoscopy, SARS-CoV-2, COVID-19, aerosol generating medical procedures and rhinology to formulate guidelines for the safety of healthcare workers. RESULTS: The review included evidence from 28 articles from the otorhinolaryngology, surgery, infectious disease, head and neck surgery and cancer biology literature. We have provided recommendations and relevant information for rhinologists during the COVID-19 pandemic, based on the available studies and data, to warrant high-quality patient care and requisite levels of infection prevention during rhinology procedures. CONCLUSION: In rhinology, marked care is advised during nasal packing, electrocauterisation and use of high-speed rotating devices in potentially infected tissue as they are considerable aerosol- producing procedures. The choice of personal protective equipment is based on the risk of exposure and possible modes of aerosol generation.

Publisher: Abstract available from the publisher.

por

Tumram, N. K. (2020). "Personal protective equipment and personal cooling garments to reduce heat-related stress and injuries." Med Leg J **88**(1_suppl): 43-46.

Use of appropriate personal protective equipment is essential for healthcare workers when dealing with patients who have tested positive or are suspected of having Covid-19. Personal protective equipment is uncomfortable at best. In hot countries (like India) or in a hot place of work, its wearers are at a high risk of heat-related illnesses. Once in personal protective equipment a healthcare worker can remain in it for at least 6 h at a stretch. In summer when it is hot and humid, personal protective equipment can cause wearer dehydration, heat exhaustion or heat fatigue. In a severe form, this can result in heat stroke and a collapse while on duty. Preventive measures are needed to protect healthcare workers. This review aims to highlight the efficacy and applicability of personal cooling garments.

Turcato, G., et al. (2020). "The COVID-19 epidemic and reorganisation of triage, an observational study." Intern Emerg Med **15**(8): 1517-1524.

Recent studies have suggested different organisational strategies, modifying Emergency Departments (EDs) during the COVID-19 epidemic. However, real data on the practical application of these strategies are not yet available. The objective of this study is to evaluate the inclusion of pre-triage during the COVID-19 outbreak. In March 2020, the structure of the ED at Merano General Hospital (Italy) was modified, with the introduction of a pre-triage protocol to divide patients according to the risk of infection. The performance of pre-triage was evaluated for sensitivity, specificity and negative predictive value (NPV). From 4th to 31st March, 2,279 patients were successively evaluated at the pre-triage stage. Of these, 257 were discharged directly from pre-triage by triage out or home quarantine and none has subsequently been hospitalised. Of the 2022 patients admitted to ED, 182 were allocated to an infected area and 1840 to a clean area. The proportion of patients who tested COVID-19 positive was 5% and, of these, 91.1% were allocated to the infected area. The pre-triage protocol demonstrated sensitivity of 91.1%, specificity of 95.3% and NPV of 99.5%. In addition, none of the healthcare workers was infected during the study period. Pre-triage can be a useful tool that, if standardised and associated with a change in the structure of the ED, can limit the spread of infection within the ED, optimise ED resources and protect healthcare workers.

Tutuncu, E. E., et al. (2021). "Saliva samples for detection of SARS-CoV-2 in mildly symptomatic and asymptomatic patients." J Med Virol **93**(5): 2932-2937.

BACKGROUND: The ongoing coronavirus disease 2019 (Covid-19) pandemic has been rapidly spreading throughout the world with confirmed case numbers already exceeding 75 million. Although nasopharyngeal swabs are the most commonly utilized samples for based severe acute respiratory syndrome coronavirus 2 (SARS-CoV-2) RNA detection, collecting these specimens requires healthcare workers and necessitates the use of personal protective equipment as it presents a nosocomial transmission risk. We aimed to assess the diagnostic value of saliva samples in mildly symptomatic and asymptomatic patients with confirmed Covid-19. METHODS: We performed a cohort study to validate the use of saliva for SARS-CoV-2 detection in mildly symptomatic and asymptomatic patients with a confirmed diagnosis of Covid-19. Saliva samples of the patients were analyzed by reverse-transcriptase polymerase chain reaction (RT-PCR). RESULTS: In May 2020, 28 asymptomatic and 25 mildly symptomatic patients were enrolled in the study. The median age was 37 years (range 4-70). None of the patients had a fever on presentation. Among 53 patients with SARS-CoV-2 detected in the nasopharyngeal sample, the real-time RT-PCR was positive in the saliva specimens in 48 (90.56%) patients. The mean cycle threshold (CT) values for nasopharyngeal and saliva specimens (27.80 ± 3.44 and 30.64 ± 2.83, respectively) were significantly correlated between the two sample types (p = .016). The mean CT values of nasopharyngeal and saliva samples in mildly symptomatic and asymptomatic patients (27.18 ± 3.53 and 30.24 ± 3.29 vs. 28.36 ± 3.31 and 30.98 ± 2.39, respectively) were not significantly different (p = .236 and p = .733, respectively). CONCLUSIONS: Saliva specimens can be considered as a reliable and less resource-intensive alternative to nasopharyngeal specimens for screening asymptomatic SARS-CoV-2 infections.

Umer, F., et al. (2020). "Role of respirators in controlling the spread of novel coronavirus (COVID-19) amongst dental healthcare providers: a review." Int Endod J **53**(8): 1062-1067.

During the ongoing COVID-19 pandemic, healthcare professionals are at the forefront of managing the highly infectious coronavirus. As the most common route of transmission is via aerosols and droplet inhalation, it is critical for healthcare workers to have the correct personal protective equipment (PPE) including gowns, masks and goggles. Surgical masks are not effective in preventing the influenza and SARS, so they are unlikely to be able to resist contaminated aerosols from entering the respiratory system. Therefore, it is vital to use respirators which have been proven to offer better protection against droplets, aerosols and fluid penetration and which form a tight seal around the mouth and nose. Various types of respirators are used in healthcare settings, such as half-mask filtering facepiece respirators (FFRs) and powered air-purifying respirators (PAPRs). The most commonly used FFR is the N95 disposable respirator, which is tight fitting and has a 95% or above particle filtering efficiency for a median particle size of 0.3 µm. This review discusses respirators, their purpose, types, clinical efficiency and proper donning and doffing techniques.

Unoki, T., et al. (2020). "Personal Protective Equipment Use by Healthcare Workers in Intensive Care Unit During the COVID-19 Pandemic in Japan: Comparative Analysis With the PPE-SAFE Survey." Acute Med Surg **7**(1).

AIM: We investigated personal protective equipment (PPE) use and its shortage, training, and adverse events among healthcare workers (HCWs) in the intensive care unit (ICU) during the coronavirus disease (COVID-19) pandemic in Japan and compared the results with an international survey that used the same methodology. METHODS: This web-based survey was conducted from April 14 to May 6, 2020, in Japan and included HCWs directly involved in ICU management of COVID-19 patients. A survey invitation was emailed using the Japanese Society of Intensive Care Medicine's mailing list. RESULTS: We analyzed 460 valid responses from among 976 responses. The N95/FFP2 mask (77%) was most frequently used than in the international study, although half of our respondents reported reuse of single-use N95/FFP2 masks. The median duration (1 hour) of uninterrupted PPE use per shift was less than that in the international study. The commonest PPE-related adverse event was experiencing intense heat (75%). Logistic regression analysis revealed that being a nurse was independently associated with experiencing intense heat. CONCLUSION: PPE shortage and frequent mask reuse were prevalent during the COVID-19 pandemic in Japan. Intense heat is the most significant symptom, especially for nurses, even with short-duration PPE use. Strategies to protect HCWs from dehydration and intense heat stroke are needed.

Upadhyay, R., et al. (2020). "Healthcare professionals' knowledge, attitudes, and practices on coronavirus disease in Western Uttar Pradesh." J Educ Health Promot **9**: 359.

BACKGROUND AND OBJECTIVES: The ongoing coronavirus disease pandemic caused by a novel genetically-mutated strain of coronavirus has posed a tremendous challenge to healthcare professionals, worldwide. This study aimed to explore the healthcare professionals' knowledge, attitudes, and practices (KAP) related to coronavirus disease in Western Uttar Pradesh and provide data to improve awareness about this disease and remove the knowledge gap, if any, by disseminating the updated information to the corona warriors. MATERIALS AND METHODS: A cross-sectional survey was conducted in the form of a structured 28-item online questionnaire using SurveyMonkey software. The qualitative data were coded numerically, and the KAP score was calculated. Pearson Chi-square test was used to study the association, among variables. RESULTS: Of 509 respondents, 296 (58.19%) were males and 213 (41.81%) were females. The median age bracket of the sample was 20-40 years. By profession, 255 (50.10%) were doctors, 167 (32.74%) were medical students, 80 (15.78%) were other healthcare workers, and a minority 7 (1.38%) were the nursing staff. Educational status covered a range from secondary (2.01%) to senior secondary (17.51%) to bachelors and equivalent (29.18%) to masters and equivalent (51.31%). The average KAP scores were 54%, and the score was highest among doctors as compared to other healthcare professionals (P < 0.001). CONCLUSIONS: In light of these study findings, we conclude that healthcare professionals are aware of coronavirus disease. However, the overall KAP score regarding its clinical course, preventive strategies, and judicious use of personal protective equipment was less than expected. There is a need to address all misconceptions and empower the corona warriors with the right information through effective mass media communication and reinforcement through seminars and workshops in the medical institutes and hospitals. The healthcare professionals are a cornerstone to prevent the nosocomial and community spread of this pandemic.

Usher, K., et al. (2020). "Pandemic-related behaviours and psychological outcomes; A rapid literature review to explain COVID-19 behaviours." Int J Ment Health Nurs **29**(6): 1018-1034.

COVID-19 spread rapidly causing widescale loss of life and economic devastation. Efforts to contain it have resulted in measures such as closing of borders and restrictions around travel, social activities and attending places of worship. We conducted this rapid review to systematically examine, synthesize, and critically appraise the available evidence on the relationship between pandemic-related behaviours and psychological outcomes. The methods were compliant with the PRISMA guidelines. The review was preregistered with PROSPERO (Registration #: CRD42020181576). A literature search was conducted from January 2010 to April 2020 using ProQuest, Web of Science, PsycINFO, PsycARTICLES, Scopus, SAGE Journals, and CINAHL. Of 3844 articles identified, we included 11 quantitative articles in the final synthesis, representing data from 32, 049 individual respondents from eight countries. We identified three pandemics (COVID-19, MERS-CoV, Influenza A(H1N1) pdm09) as well as several psychological outcomes including anxiety, mental distress, post-traumatic stress disorder, and anger. We also identified several behaviours during pandemics and categorized them into protective, preparedness, and perverse behaviours. The review showed that even though there is limited evidence regarding pandemic-related behaviours and psychological outcomes, the current findings showed that the psychological outcomes significantly impacted on the adoption of the pandemic-related behaviours. Given the negative effects of psychological outcomes on behaviours, we recommend that mental health professionals should promote mental health support to people exhibiting psychological distress resulting from similar events in the future. Also, we recommend that future research should test the hypothesized effects of pandemics and psychological outcomes on behaviour change.

Uthayakumar, A. K., et al. (2021). "PPE-associated dermatoses: effect on work and wellbeing." Future Healthc J **8**(1): e67-e69.

The COVID-19 pandemic has led to more stringent infection control practice within hospitals, with the added requirement of personal protective equipment (PPE). Consequentially, the incidence of PPE-related dermatoses in healthcare workers is increasing. Analysis of staff members seen in a dermatology self-referral service during the first COVID-19 peak revealed a wide range of healthcare professionals being affected, with irritant hand dermatitis and acneiform eruptions being the commonest diagnoses. These PPE-related dermatoses had significant effects on staff members' work and wellbeing, which improved following assessment and treatment by dermatology. Given the change to our behaviour wearing PPE daily, which is likely to continue for the foreseeable future, the consequences will inevitably affect both healthcare workers and the general public, with these common dermatoses presenting in both primary and secondary care. It is therefore imperative to recognise both the physical and emotional impact that COVID-19 PPE has, and initiate prompt treatment to improve wellbeing.

Vagni, M., et al. (2020). "Coping With COVID-19: Emergency Stress, Secondary Trauma and Self-Efficacy in Healthcare and Emergency Workers in Italy." Front Psychol **11**: 566912.

Coping with the coronavirus disease (COVID-19) is a significant risk factor for the psychological distress of health workers. Hence, this study explores the relationship between coping strategies used by healthcare and emergency workers in Italy to manage the stress factors related to the COVID-19 emergency, which may result in the risk of developing secondary trauma. We study differences between healthcare (n = 121) and emergency workers (n = 89) in terms of their coping strategies, emergency stress, and secondary trauma, as well as the relationships of these differences to demographic variables and other stress factors (Instructions and Equipment). For this purpose, we collected data from participants through the following questionnaires online: Secondary Traumatic Stress Scale - Italian Version, The Coping Self-Efficacy Scale - Short Form, an original questionnaire on stressors, and the Emergency Stress Questionnaire (to assess organizational-relational, physical, decisional inefficacy, emotional, cognitive, and COVID-19 stress). We performed a t-test, correlational analysis, and hierarchical regression. The analyses reveal that compared with the emergency worker group, the health worker group has greater levels of emergency stress and arousal and is more willing to use problem-focused coping. Healthcare workers involved in the treatment of COVID-19 are exposed to a large degree of stress and could experience secondary trauma; hence, it is essential to plan prevention strategies for future pandemic situations. Moreover, individual efficacy in stopping negative emotions and thoughts could be a protective strategy against stress and secondary trauma.

van der Valk, J. P. M. and J. In 't Veen (2021). "SARS-Cov-2: The relevance and Prevention of Aerosol Transmission." J Occup Environ Med.

: Coronavirus Disease (COVID-19), caused by the Severe Acute Respiratory Syndrome Coronavirus 2 (SARS-Cov-2), has claimed many victims worldwide due to its high virulence and contagiousness.The person to person transmission of SARS-Cov-2 when in close contact is facilitated by respiratory droplets containing the virus particles, and by skin contact with contaminated surfaces. However, the large number of COVID-19 infections cannot be explained only by droplet deposition or contact contamination. It seems very plausible that aerosols are important in transmitting SARS-Cov-2. It has been demonstrated that SARS-CoV-2 remains viable in aerosols for hours, facilitating rapid distribution of the virus over great distances. Aerosols may, therefore, also be responsible for so-called super-spreader events (SSE's).Indirect evidence points to a correlation between ventilation and the transmission and spread of SARS-Cov-2, supporting ventilation as an important factor in preventing airborne transmission. Further actions to avoid transmission of COVID-19 include social distancing, hygiene measures and barrier measures, such as face-coverings. Professional masks offer better protection than cloth masks.These protection measures are especially relevant to healthcare workers, when performing endotracheal intubation, but the risk from non-invasive ventilation and nebulizing treatment seems to be moderate.

Van Elslande, J., et al. (2021). "Estimated half-life of SARS-CoV-2 anti-spike antibodies more than double the half-life of anti-nucleocapsid antibodies in healthcare workers." Clin Infect Dis.

van Roekel, H., et al. (2020). "Healthcare Workers Who Work With COVID-19 Patients Are More Physically Exhausted and Have More Sleep Problems." Front Psychol **11**: 625626.

In this survey study of 7,208 Dutch healthcare workers, we investigate whether healthcare workers dealing with COVID-19 patients experience lower general health, more physical and mental exhaustion and more sleep problems than other healthcare workers. Additionally, we study whether there are differences in well-being within the group of healthcare workers working with COVID-19 patients, based on personal and work characteristics. We find healthcare workers who are in direct contact with COVID-19 patients report more sleep problems and are more physically exhausted than those who are not in direct contact with COVID-19 patients. Mental exhaustion and general health do not significantly differ between healthcare workers who are in direct contact with COVID-19 patients and those who are not. Among healthcare workers in direct contact with COVID-19 patients, lower well-being on one or more indicators is reported by those who are female, living alone, without leadership role, or without sufficient protective equipment. Regarding age, physical exhaustion is more prevalent under healthcare workers older than 55 years, whereas mental exhaustion is more prevalent under healthcare workers younger than 36 years. These results stress the need of mental and physical support of healthcare workers during a pandemic, catered to the needs of healthcare workers themselves.

van Wezel, R. A. C., et al. (2020). "In hospital verification of non CE-marked respiratory protective devices to ensure safety of healthcare staff during the COVID-19 outbreak." J Hosp Infect **105**(3): 447-453.

AIM: To develop a protocol to ensure the quality of respiratory protective devices for healthcare workers nursing and treating patients with possible or confirmed COVID-19 in the Catharina hospital. BACKGROUND: Due to the COVID-19 outbreak a shortage of respirators is occurring worldwide; more specifically, CE-certified FFP2 respirators. This has resulted in an increased supply to hospitals of alternative respirators of uncertain quality. Nevertheless, the quality of the respirators used by our healthcare workers must be ensured. METHOD: A protocol and criteria based on applicable standards was developed to ensure the quality of respirators. The protocol has been implemented at the Catharina hospital and includes verification of the documents accompanying the respirator, visual inspection of the respirator and a test for total inward leak of particles into respirators. FINDINGS: 67% of the respirators brands and types received in the Catharina hospital did not meet quality criteria. CONCLUSION: With a simple verification protocol the quality of the respirators can be checked and guaranteed while there is a shortage of the CE approved respirators which are normally used. With this in-hospital protocol health care workers can be equipped with safe-to-use respirators.

Van Zundert, T., et al. (2020). "Revisiting safe airway management and patient care by anaesthetists during the COVID-19 pandemic." Br J Anaesth **125**(6): 863-867.

Vandyck-Sey, P., et al. (2020). "Incidental finding of COVID-19 infection amongst staff at a primary care facility in Ghana." Afr J Prim Health Care Fam Med **12**(1): e1-e4.

The COVID-19 pandemic has affected nearly every country worldwide and all African countries. The issue of healthcare workers (HCWs) contracting the disease is a growing concern in Ghana, because of the risk of spreading infections amongst themselves and to vulnerable patients in their care. This article illustrates how 14 staff at the Korle Bu Polyclinic/Family Medicine Department were incidentally found to be Covid-19 positive with most of them being asymptomatic. This observation led to a modification of the personal protective equipment (PPE) used by clinical staff when attending to patients. Furthermore, this finding suggests that a different criteria or guideline may be needed for testing of HCWs during a pandemic where a significant proportion of infected people are asymptomatic. We conclude that in the primary care setting HCWs must be ready to see all the following cases safely: routine patients, asymptomatic COVID-19 patients and suspected COVID-19 patients.

Varahachalam, S. P., et al. (2021). "Nanomedicine for the SARS-CoV-2: State-of-the-Art and Future Prospects." Int J Nanomedicine **16**: 539-560.

The newly emerged ribonucleic acid (RNA) enveloped human beta-coronavirus, severe acute respiratory syndrome coronavirus 2 (SARS-CoV-2) infection caused the COVID-19 pandemic, severely affects the respiratory system, and may lead to death. Lacking effective diagnostics and therapies made this pandemic challenging to manage since the SARS-CoV-2 transmits via human-to-human, enters via ACE2 and TMPSSR2 receptors, and damages organs rich in host cells, spreads via symptomatic carriers and is prominent in an immune-compromised population. New SARS-CoV-2 informatics (structure, strains, like-cycles, functional sites) motivated bio-pharma experts to investigate novel therapeutic agents that act to recognize, inhibit, and knockdown combinations of drugs, vaccines, and antibodies, have been optimized to manage COVID-19. However, successful targeted delivery of these agents to avoid off-targeting and unnecessary drug ingestion is very challenging. To overcome these obstacles, this mini-review projects nanomedicine technology, a pharmacologically relevant cargo of size within 10 to 200 nm, for site-specific delivery of a therapeutic agent to recognize and eradicate the SARS-CoV-2, and improving the human immune system. Such combinational therapy based on compartmentalization controls the delivery and releases of a drug optimized based on patient genomic profile and medical history. Nanotechnology could help combat COVID-19 via various methods such as avoiding viral contamination and spraying by developing personal protective equipment (PPE) to increase the protection of healthcare workers and produce effective antiviral disinfectants surface coatings capable of inactivating and preventing the virus from spreading. To quickly recognize the infection or immunological response, design highly accurate and sensitive nano-based sensors. Development of new drugs with improved activity, reduced toxicity, and sustained release to the lungs, as well as tissue targets; and development of nano-based immunizations to improve humoral and cellular immune responses. The desired and controlled features of suggested personalized therapeutics, nanomedicine, is a potential therapy to manage COVID-19 successfully. The state-of-the-art nanomedicine, challenges, and prospects of nanomedicine are carefully and critically discussed in this report, which may serve as a key platform for scholars to investigate the role of nanomedicine for higher efficacy to manage the COVID-19 pandemic.

Varghese, G. M., et al. (2020). "Clinical management of COVID-19." Indian J Med Res **151**(5): 401-410.

The novel coronavirus disease 2019 (COVID-19) with its early origin from Wuhan city in China has evolved into a global pandemic. Maximal precautionary measures and resources have been put forward by most nations in war footing to mitigate transmission and decrease fatality rates. This article was aimed to review the evidence on clinical management and to deal with the identification of high-risk groups, warning signs, appropriate investigations, proper sample collection for confirmation, general and specific treatment measures, strategies as well as infection control in the healthcare settings. Advanced age, cardiovascular disease, diabetes, hypertension and cancer have been found to be the risk factors for severe disease. Fever lasting for >five days with tachypnoea, tachycardia or hypotension are indications for urgent attention and hospitalization in a patient with suspected COVID-19. At present, reverse transcription-polymerase chain reaction (RT-PCR) from the upper respiratory tract samples is the diagnostic test of choice. While many drugs have shown in vitro activity against severe acute respiratory syndrome coronavirus 2 (SARS-CoV-2), there are insufficient clinical data to promote or dissuade their usage. Among the currently available drugs, hydroxychloroquine and lopinavir/ritonavir may be considered for patients with severe COVID-19 infection, awaiting further clinical trials. Stringent droplet and contact precautions will protect healthcare workers against most clinical exposures to COVID-19.

Vázquez-Nava, F., et al. (2020). "Risk factors of non-adherence to guidelines for the prevention of COVID-19 among young adults with asthma in a region with a high risk of a COVID-19 outbreak." J Asthma: 1-7.

OBJECTIVE: SARS-CoV-2 disease 2019 (COVID-19) is highly contagious and spreads rapidly. The application of preventive measures has proven to be the best strategy to minimize the number of patients and the dissemination of and deaths from COVID-19. The objective of this study was to determine the risk factors that limit the adherence of asthmatic patients to measures that prevent COVID-19 among residents of a region with a high risk of a COVID-19 outbreak. METHODS: Through a cross-sectional study, data from 2,372 participants aged 16-24 years were analyzed. To collect their information, a questionnaire was constructed using the Google Forms tool. Due to the prevention measures in place for COVID-19, the home quarantine of many people, and the lack of socialization, the questionnaire was distributed through email (Internet) and WhatsApp. A logistic regression analysis was performed to determine the relationship between the variables. RESULTS: The prevalence of asthma was 12.2%, and non-adherence to the guidelines for the prevention of COVID-19 was 53.1%. Approximately 30.8% of asthma patients did not comply with the basic prevention measures for COVID-19. The results of the logistic regression analysis showed that being male, active smoking, and believing that COVID-19 is not a more severe disease for people suffering from asthma than others were associated with non-adherence to the basic protection measures established in the guidelines for the prevention of COVID-19. CONCLUSIONS: It is important for health professionals to advise asthma patients to comply with the basic measures of protection against COVID-19 and timely use medications for asthma control.

Vega-Vega, O., et al. (2020). "[Prevention and control of SARS-CoV-2 (Covid-19) coronavirus infection in hemodialysis units.]." Salud Publica Mex **62**(3): 341-347.

The SARS-CoV-2 pandemic is of hich risk for patients on chronic hemodialysis due to their immunosuppressed state, advanced age, and the coexistence of significant comorbidities, in particular cardiovascular disease, diabetes mellitus, and others. Additionally, they constitute a closed conglomerate since they come to treatment regularly, spending hours in the treatment places, exposed to a possible acquisition of the infection. Finally, going to their treatment regularly prevents them from remaining in home isolation and with potential exposure. These recommendations summarize the interventions proposed by three international organizations and add some suggested by national experts, with the aim to early identify the patients and health personnel at risk and reducing the risk of infection.

Vela, J. M. (2020). "Repurposing Sigma-1 Receptor Ligands for COVID-19 Therapy?" Front Pharmacol **11**: 582310.

Outbreaks of emerging infections, such as COVID-19 pandemic especially, confront health professionals with the unique challenge of treating patients. With no time to discover new drugs, repurposing of approved drugs or in clinical development is likely the only solution. Replication of coronaviruses (CoVs) occurs in a modified membranous compartment derived from the endoplasmic reticulum (ER), causes host cell ER stress and activates pathways to facilitate adaptation of the host cell machinery to viral needs. Accordingly, modulation of ER remodeling and ER stress response might be pivotal in elucidating CoV-host interactions and provide a rationale for new therapeutic, host-based antiviral approaches. The sigma-1 receptor (Sig-1R) is a ligand-operated, ER membrane-bound chaperone that acts as an upstream modulator of ER stress and thus a candidate host protein for host-based repurposing approaches to treat COVID-19 patients. Sig-1R ligands are frequently identified in in vitro drug repurposing screens aiming to identify antiviral compounds against CoVs, including severe acute respiratory syndrome CoV-2 (SARS-CoV-2). Sig-1R regulates key mechanisms of the adaptive host cell stress response and takes part in early steps of viral replication. It is enriched in lipid rafts and detergent-resistant ER membranes, where it colocalizes with viral replicase proteins. Indeed, the non-structural SARS-CoV-2 protein Nsp6 interacts with Sig-1R. The activity of Sig-1R ligands against COVID-19 remains to be specifically assessed in clinical trials. This review provides a rationale for targeting Sig-1R as a host-based drug repurposing approach to treat COVID-19 patients. Evidence gained using Sig-1R ligands in unbiased in vitro antiviral drug screens and the potential mechanisms underlying the modulatory effect of Sig-1R on the host cell response are discussed. Targeting Sig-1R is not expected to reduce dramatically established viral replication, but it might interfere with early steps of virus-induced host cell reprogramming, aid to slow down the course of infection, prevent the aggravation of the disease and/or allow a time window to mature a protective immune response. Sig-1R-based medicines could provide benefit not only as early intervention, preventive but also as adjuvant therapy.

Vellone, V., et al. (2020). "How to Produce Cheap and Easy Custom-Made Sterilizable Filtering Facepiece 2/3 Masks for Healthcare Providers During Pandemic COVID-19 Emergency." J Craniofac Surg **31**(6): e640-e642.

On January 8, 2020, a novel coronavirus was officially announced as the causative pathogen of coronavirus disease (COVID-19) by the Chinese Center for Disease Control and Prevention.On February 26, COVID-19 has been recognized in 34 countries, with a total of 80,239 laboratory-confirmed patients and 2700 deaths.Protecting healthcare workers from infectious hazards is paramount to ensuring their safety in delivering health care.In addition, being able to protect healthcare workers, constituting the front-line response against high-threat respiratory pathogens, such as severe acute respiratory syndrome coronavirus 2, is important for reducing secondary transmission in healthcare-associated outbreaks.Authors present a simple, reliable, and cheap protocol to produce a custom-made sterilizable filtering facepiece 2/3 masks for healthcare providers during pandemic COVID-19 emergency.

Vemmou, E., et al. (2021). "Case Selection During the COVID-19 Pandemic: Who Should Go to the Cardiac Catheterization Laboratory?" Curr Treat Options Cardiovasc Med **23**(4): 27.

PURPOSE OF REVIEW: To summarize the best available evidence and recommendations regarding case selection for cardiac catheterization laboratory (CCL) during the coronavirus disease 2019 (COVID-19) pandemic with emphasis on ST segment elevation myocardial infarction (STEMI) management. RECENT FINDINGS: The restructuring of cardiovascular services to preserve hospital beds and personal protective equipment during the COVID-19 pandemic had a profound effect on healthcare delivery around the world with unintended consequences. In the United States, a significant 38% reduction in CCL activations for STEMI was noted in the early phase of the pandemic. Similarly, a 34% decline in utilization of invasive angiography, an 18% reduction in primary percutaneous coronary intervention (PPCI), and a 19% increase in door-to-balloon (D2B) times were also observed. These trends coincided with a significant increase in out-of-hospital cardiac arrests and late MI presentations. A shift to pharmacological reperfusion has been advocated in Asia, which resulted in increased morbidity and mortality. SUMMARY: COVID-19 has negatively affected many aspects of STEMI care, including timely access to mechanical reperfusion, which has resulted in increased morbidity and mortality. Balancing optimal STEMI care with the risk of infection to healthcare workers during the pandemic is challenging. Recommendations provided by consensus documents are a helpful guidance.

Verbeek, J. H., et al. (2020). "Personal protective equipment for preventing highly infectious diseases due to exposure to contaminated body fluids in healthcare staff." Cochrane Database Syst Rev **5**: Cd011621.

BACKGROUND: In epidemics of highly infectious diseases, such as Ebola, severe acute respiratory syndrome (SARS), or coronavirus (COVID-19), healthcare workers (HCW) are at much greater risk of infection than the general population, due to their contact with patients' contaminated body fluids. Personal protective equipment (PPE) can reduce the risk by covering exposed body parts. It is unclear which type of PPE protects best, what is the best way to put PPE on (i.e. donning) or to remove PPE (i.e. doffing), and how to train HCWs to use PPE as instructed. OBJECTIVES: To evaluate which type of full-body PPE and which method of donning or doffing PPE have the least risk of contamination or infection for HCW, and which training methods increase compliance with PPE protocols. SEARCH METHODS: We searched CENTRAL, MEDLINE, Embase and CINAHL to 20 March 2020. SELECTION CRITERIA: We included all controlled studies that evaluated the effect of full-body PPE used by HCW exposed to highly infectious diseases, on the risk of infection, contamination, or noncompliance with protocols. We also included studies that compared the effect of various ways of donning or doffing PPE, and the effects of training on the same outcomes. DATA COLLECTION AND ANALYSIS: Two review authors independently selected studies, extracted data and assessed the risk of bias in included trials. We conducted random-effects meta-analyses were appropriate. MAIN RESULTS: Earlier versions of this review were published in 2016 and 2019. In this update, we included 24 studies with 2278 participants, of which 14 were randomised controlled trials (RCT), one was a quasi-RCT and nine had a non-randomised design. Eight studies compared types of PPE. Six studies evaluated adapted PPE. Eight studies compared donning and doffing processes and three studies evaluated types of training. Eighteen studies used simulated exposure with fluorescent markers or harmless microbes. In simulation studies, median contamination rates were 25% for the intervention and 67% for the control groups. Evidence for all outcomes is of very low certainty unless otherwise stated because it is based on one or two studies, the indirectness of the evidence in simulation studies and because of risk of bias. Types of PPE The use of a powered, air-purifying respirator with coverall may protect against the risk of contamination better than a N95 mask and gown (risk ratio (RR) 0.27, 95% confidence interval (CI) 0.17 to 0.43) but was more difficult to don (non-compliance: RR 7.5, 95% CI 1.81 to 31.1). In one RCT (59 participants) coveralls were more difficult to doff than isolation gowns (very low-certainty evidence). Gowns may protect better against contamination than aprons (small patches: mean difference (MD) -10.28, 95% CI -14.77 to -5.79). PPE made of more breathable material may lead to a similar number of spots on the trunk (MD 1.60, 95% CI -0.15 to 3.35) compared to more water-repellent material but may have greater user satisfaction (MD -0.46, 95% CI -0.84 to -0.08, scale of 1 to 5). According to three studies that tested more recently introduced full-body PPE ensembles, there may be no difference in contamination. Modified PPE versus standard PPE The following modifications to PPE design may lead to less contamination compared to standard PPE: sealed gown and glove combination (RR 0.27, 95% CI 0.09 to 0.78), a better fitting gown around the neck, wrists and hands (RR 0.08, 95% CI 0.01 to 0.55), a better cover of the gown-wrist interface (RR 0.45, 95% CI 0.26 to 0.78, low-certainty evidence), added tabs to grab to facilitate doffing of masks (RR 0.33, 95% CI 0.14 to 0.80) or gloves (RR 0.22, 95% CI 0.15 to 0.31). Donning and doffing Using Centers for Disease Control and Prevention (CDC) recommendations for doffing may lead to less contamination compared to no guidance (small patches: MD -5.44, 95% CI -7.43 to -3.45). One-step removal of gloves and gown may lead to less bacterial contamination (RR 0.20, 95% CI 0.05 to 0.77) but not to less fluorescent contamination (RR 0.98, 95% CI 0.75 to 1.28) than separate removal. Double-gloving may lead to less viral or bacterial contamination compared to single gloving (RR 0.34, 95% CI 0.17 to 0.66) but not to less fluorescent contamination (RR 0.98, 95% CI 0.75 to 1.28). Additional spoken instruction may lead to fewer errors in doffing (MD -0.9, 95% CI -1.4 to -0.4) and to fewer contamination spots (MD -5, 95% CI -8.08 to -1.92). Extra sanitation of gloves before doffing with quaternary ammonium or bleach may decrease contamination, but not alcohol-based hand rub. Training The use of additional computer simulation may lead to fewer errors in doffing (MD -1.2, 95% CI -1.6 to -0.7). A video lecture on donning PPE may lead to better skills scores (MD 30.70, 95% CI 20.14 to 41.26) than a traditional lecture. Face-to-face instruction may reduce noncompliance with doffing guidance more (odds ratio 0.45, 95% CI 0.21 to 0.98) than providing folders or videos only. AUTHORS' CONCLUSIONS: We found low- to very low-certainty evidence that covering more parts of the body leads to better protection but usually comes at the cost of more difficult donning or doffing and less user comfort. More breathable types of PPE may lead to similar contamination but may have greater user satisfaction. Modifications to PPE design, such as tabs to grab, may decrease the risk of contamination. For donning and doffing procedures, following CDC doffing guidance, a one-step glove and gown removal, double-gloving, spoken instructions during doffing, and using glove disinfection may reduce contamination and increase compliance. Face-to-face training in PPE use may reduce errors more than folder-based training. We still need RCTs of training with long-term follow-up. We need simulation studies with more participants to find out which combinations of PPE and which doffing procedure protects best. Consensus on simulation of exposure and assessment of outcome is urgently needed. We also need more real-life evidence. Therefore, the use of PPE of HCW exposed to highly infectious diseases should be registered and the HCW should be prospectively followed for their risk of infection.

Verbeek, J. H., et al. (2020). "Personal protective equipment for preventing highly infectious diseases due to exposure to contaminated body fluids in healthcare staff." Cochrane Database Syst Rev **4**(4): Cd011621.

BACKGROUND: In epidemics of highly infectious diseases, such as Ebola, severe acute respiratory syndrome (SARS), or coronavirus (COVID-19), healthcare workers (HCW) are at much greater risk of infection than the general population, due to their contact with patients' contaminated body fluids. Personal protective equipment (PPE) can reduce the risk by covering exposed body parts. It is unclear which type of PPE protects best, what is the best way to put PPE on (i.e. donning) or to remove PPE (i.e. doffing), and how to train HCWs to use PPE as instructed. OBJECTIVES: To evaluate which type of full-body PPE and which method of donning or doffing PPE have the least risk of contamination or infection for HCW, and which training methods increase compliance with PPE protocols. SEARCH METHODS: We searched CENTRAL, MEDLINE, Embase and CINAHL to 20 March 2020. SELECTION CRITERIA: We included all controlled studies that evaluated the effect of full-body PPE used by HCW exposed to highly infectious diseases, on the risk of infection, contamination, or noncompliance with protocols. We also included studies that compared the effect of various ways of donning or doffing PPE, and the effects of training on the same outcomes. DATA COLLECTION AND ANALYSIS: Two review authors independently selected studies, extracted data and assessed the risk of bias in included trials. We conducted random-effects meta-analyses were appropriate. MAIN RESULTS: Earlier versions of this review were published in 2016 and 2019. In this update, we included 24 studies with 2278 participants, of which 14 were randomised controlled trials (RCT), one was a quasi-RCT and nine had a non-randomised design. Eight studies compared types of PPE. Six studies evaluated adapted PPE. Eight studies compared donning and doffing processes and three studies evaluated types of training. Eighteen studies used simulated exposure with fluorescent markers or harmless microbes. In simulation studies, median contamination rates were 25% for the intervention and 67% for the control groups. Evidence for all outcomes is of very low certainty unless otherwise stated because it is based on one or two studies, the indirectness of the evidence in simulation studies and because of risk of bias. Types of PPE The use of a powered, air-purifying respirator with coverall may protect against the risk of contamination better than a N95 mask and gown (risk ratio (RR) 0.27, 95% confidence interval (CI) 0.17 to 0.43) but was more difficult to don (non-compliance: RR 7.5, 95% CI 1.81 to 31.1). In one RCT (59 participants), people with a long gown had less contamination than those with a coverall, and coveralls were more difficult to doff (low-certainty evidence). Gowns may protect better against contamination than aprons (small patches: mean difference (MD) -10.28, 95% CI -14.77 to -5.79). PPE made of more breathable material may lead to a similar number of spots on the trunk (MD 1.60, 95% CI -0.15 to 3.35) compared to more water-repellent material but may have greater user satisfaction (MD -0.46, 95% CI -0.84 to -0.08, scale of 1 to 5). Modified PPE versus standard PPE The following modifications to PPE design may lead to less contamination compared to standard PPE: sealed gown and glove combination (RR 0.27, 95% CI 0.09 to 0.78), a better fitting gown around the neck, wrists and hands (RR 0.08, 95% CI 0.01 to 0.55), a better cover of the gown-wrist interface (RR 0.45, 95% CI 0.26 to 0.78, low-certainty evidence), added tabs to grab to facilitate doffing of masks (RR 0.33, 95% CI 0.14 to 0.80) or gloves (RR 0.22, 95% CI 0.15 to 0.31). Donning and doffing Using Centers for Disease Control and Prevention (CDC) recommendations for doffing may lead to less contamination compared to no guidance (small patches: MD -5.44, 95% CI -7.43 to -3.45). One-step removal of gloves and gown may lead to less bacterial contamination (RR 0.20, 95% CI 0.05 to 0.77) but not to less fluorescent contamination (RR 0.98, 95% CI 0.75 to 1.28) than separate removal. Double-gloving may lead to less viral or bacterial contamination compared to single gloving (RR 0.34, 95% CI 0.17 to 0.66) but not to less fluorescent contamination (RR 0.98, 95% CI 0.75 to 1.28). Additional spoken instruction may lead to fewer errors in doffing (MD -0.9, 95% CI -1.4 to -0.4) and to fewer contamination spots (MD -5, 95% CI -8.08 to -1.92). Extra sanitation of gloves before doffing with quaternary ammonium or bleach may decrease contamination, but not alcohol-based hand rub. Training The use of additional computer simulation may lead to fewer errors in doffing (MD -1.2, 95% CI -1.6 to -0.7). A video lecture on donning PPE may lead to better skills scores (MD 30.70, 95% CI 20.14 to 41.26) than a traditional lecture. Face-to-face instruction may reduce noncompliance with doffing guidance more (odds ratio 0.45, 95% CI 0.21 to 0.98) than providing folders or videos only. AUTHORS' CONCLUSIONS: We found low- to very low-certainty evidence that covering more parts of the body leads to better protection but usually comes at the cost of more difficult donning or doffing and less user comfort, and may therefore even lead to more contamination. More breathable types of PPE may lead to similar contamination but may have greater user satisfaction. Modifications to PPE design, such as tabs to grab, may decrease the risk of contamination. For donning and doffing procedures, following CDC doffing guidance, a one-step glove and gown removal, double-gloving, spoken instructions during doffing, and using glove disinfection may reduce contamination and increase compliance. Face-to-face training in PPE use may reduce errors more than folder-based training. We still need RCTs of training with long-term follow-up. We need simulation studies with more participants to find out which combinations of PPE and which doffing procedure protects best. Consensus on simulation of exposure and assessment of outcome is urgently needed. We also need more real-life evidence. Therefore, the use of PPE of HCW exposed to highly infectious diseases should be registered and the HCW should be prospectively followed for their risk of infection.

Verberk, J. D. M., et al. (2021). "Experiences and needs of persons living with a household member infected with SARS-CoV-2: A mixed method study." PLoS One **16**(3): e0249391.

BACKGROUND: Households are important sites for transmission of SARS-CoV-2 and preventive measures are recommended. This study aimed to 1) investigate the impact of living with a person infected with SARS-CoV-2; 2) understand how household members implemented infection control recommendations in their home; and 3) identify the information and support needs of household members. METHODS: For this observational mixed-methods study, households with a person with confirmed SARS-CoV-2 infection were recruited via drive-through testing sites of Municipal Health Services, healthcare worker screening or hospital emergency visits in the University Medical Centre Utrecht, the Netherlands and via primary care physicians, hospital emergency visits or preoperative screening in the University Hospital of Antwerp, Belgium. We recorded household characteristics, including characteristics of all household members, together with their views on prevention measures. In a subset of households one adult household member was asked to participate in an interview investigating their views on preventive measures. Survey data were analysed using descriptive statistics and interview data by rapid framework analysis. A triangulation protocol was used to integrate findings. RESULTS: Thirty-four households (120 household members) were included in the quantitative survey. Twenty-two households were invited to be interviewed, of which 18 completed an interview (response 81.8%). Survey data showed that almost all households implemented some preventive measures, the use of face masks being least frequently reported. Measures taken depended on what was physically possible, the perceived severity of illness of the index patient and to what extent household members were willing to limit social interaction. Respondents did not believe in the effectiveness of wearing face masks within the house, and from the interviews this was explained by media coverage of face masks, impracticality and the stigma associated with wearing masks. Interviewees reported that quarantine had a high emotional burden and wished to have more information about the exact duration of quarantine, their own COVID-19 status, symptoms and when to seek medical help. CONCLUSION: People were willing to implement prevention measures, however actual adherence depended on perceived severity of illness and the perceived risk of becoming infected. Homes are social environments and recommendations for infection prevention should account for this context. Incorporating our findings into policy making could provide households with more relevant and actionable advice.

Veritti, D., et al. (2020). "Infection control measures in ophthalmology during the COVID-19 outbreak: A narrative review from an early experience in Italy." Eur J Ophthalmol **30**(4): 621-628.

INTRODUCTION: The novel coronavirus (SARS-CoV-2) is infecting people and spreading easily from person-to-person. Cases have been detected in most countries worldwide. Italy is one of the most affected countries as of 30 March 2020. Public health response includes a rapid reorganization of the Italian National Healthcare System in order to reduce transmission of COVID-19 within hospitals and healthcare facilities, while optimizing the assistance to patients with severe COVID-19 complications. METHODS: We analysed the actions that were taken in three ophthalmology centres in northern Italy during the SARS-CoV-2 outbreak and how these measures affected patient's attendance. In addition, due to the rapidly evolving scenario, we reviewed the evidence available during the course of this pandemic. RESULTS: A full reorganization of ophthalmology services is mandatory according to current existing infection containment measures in order to continue dispensing urgent procedures without endangering the community with amplification of the diffusion chain. Ophthalmologists are considered at elevated risk of exposure when caring patients and vice versa, due to their close proximity during eye examination. High volumes of procedures typically generated by ophthalmologists with concurrent implications on the risk of infection are considered when re-assessing healthcare facilities reorganization. CONCLUSION: Containment measures in the event of pandemic due to infective agents should be well known by healthcare professionals and promptly applied in order to mitigate the risk of nosocomial transmission and outbreak.

Verma, N. K., et al. (2020). "Combination Therapy Using Inhalable GapmeR and Recombinant ACE2 for COVID-19." Front Mol Biosci **7**: 197.

Here we report our perspective on applying GapmeR technology in combination with recombinant angiotensin-converting enzyme 2 (ACE2) in the treatment of COVID-19 patients. GapmeR is a cell-permeating antisense single-stranded DNA molecule that can be designed to specifically target intracellular severe acute respiratory syndrome coronavirus 2 (SARS-CoV-2). Once internalized into host cells, such as lung alveolar cells, GapmeR molecules can bind to the viral RNA. This RNA/DNA hybrid will then be degraded by the RNase H enzyme abundantly present in the host cells. GapmeRs can be delivered to COVID-19 patients through inhalation or via nebulization. SARS-CoV-2-targeted GapmeR can also be given to frontline healthcare workers as a prophylactic protection. The recombinant ACE2 protein, the efficacy of which is being evaluated in clinical trials, will bind to the spike (S) glycoprotein of extracellular SARS-CoV-2 and potentially block viral infectivity. We propose that combining inhalable SARS-CoV-2-targeted GapmeRs with recombinant ACE2 could provide a viable and rapidly implementable more effective therapeutic approach for eradicating SARS-CoV-2 and save millions of lives.

Vermani, S., et al. (2020). "COVID-19 and the Radiology Department: What We Know So Far." SN Compr Clin Med: 1-7.

COVID-19 is a global healthcare pandemic that is now growing through nations across the world. The role of radiology is crucial, and a variety of guidelines have been published regarding the role of imaging. These aim to protect healthcare workers (HCWs) and the general public from exposure, while preserving critical radiology operations and conserving personal protective equipment (PPE) and other critical care resources during the COVID-19 pandemic. Fleischner Society published guidelines on indications of imaging various settings. These guidelines take into account resource availability, pre-test probability, degree of symptoms and risk factors, which is crucial for decision-making regarding need and indications of imaging. Mitigating steps and alternative approaches should be considered to provide the best care for patients while protecting all HCWs. Owing to overlap of COVID-19 imaging findings with other pathologies, standardized reporting acquires importance for risk assessment and effective communication of suspicious findings. RSNA followed by Dutch Radiological Society (NVvR) have published guidelines on standardized CT reporting for COVID-19, which show excellent inter-observer variability. Standardized reporting can provide guidance and confidence to radiologists as well as increased clarity to physicians through reduced reporting variability. The article discusses the published recommendations and aims to make radiologists aware of the protocols and guidelines that need to be followed in this ongoing public health crisis for effective patient care while protecting HCWs and conserving resources.

Vernez, D., et al. (2020). "Reusability of filtering facepiece respirators after decontamination through drying and germicidal UV irradiation." BMJ Glob Health **5**(10).

INTRODUCTION: During pandemics, such as the SARS-CoV-2, filtering facepiece respirators plays an essential role in protecting healthcare personnel. The recycling of respirators is possible in case of critical shortage, but it raises the question of the effectiveness of decontamination as well as the performance of the reused respirators. METHOD: Disposable respirators were subjected to ultraviolet germicidal irradiation (UVGI) treatment at single or successive doses of 60 mJ/cm(2) after a short drying cycle (30 min, 70°C). The germicidal efficacy of this treatment was tested by spiking respirators with two staphylococcal bacteriophages (vB_HSa_2002 and P66 phages). The respirator performance was investigated by the following parameters: particle penetration (NaCl aerosol, 10-300 nm), scanning electron microscopy (SEM), Fourier-transform infrared spectroscopy (FTIR), differential scanning calorimetry and mechanical tensile tests. RESULTS: No viable phage particles were recovered from any of the respirators after decontamination (log reduction in virus titre >3), and no reduction in chemical or physical properties (SEM, particle penetrations <5%-6%) were observed. Increasing the UVGI dose 10-fold led to chemical alterations of the respirator filtration media (FTIR) but did not affect the physical properties (particle penetration), which was unaltered even at 3000 mJ/cm(2) (50 cycles). When respirators had been used by healthcare workers and undergone decontamination, they had particle penetration significantly greater than never donned respirators. CONCLUSION: This decontamination procedure is an attractive method for respirators in case of shortages during a SARS pandemic. A successful implementation requires a careful design and particle penetration performance control tests over the successive reuse cycles.

Vest, N., et al. (2021). "Prison Population Reductions and COVID-19: A Latent Profile Analysis Synthesizing Recent Evidence From the Texas State Prison System." J Urban Health **98**(1): 53-58.

People in prison are particularly vulnerable to infectious disease due to close living conditions and the lack of protective equipment. As a result, public health professionals and prison administrators seek information to guide best practices and policy recommendations during the COVID-19 pandemic. Using latent profile analysis, we sought to characterize Texas prisons on levels of COVID-19 cases and deaths among incarcerated residents, and COVID-19 cases among prison staff. This observational study was a secondary data analysis of publicly available data from the Texas Department of Criminal Justice (TBDJ) collected from March 1, 2020, until July 24, 2020. This project was completed in collaboration with the COVID Prison Project. We identified relevant profiles from the data: a low-outbreak profile, a high-outbreak profile, and a high-death profile. Additionally, current prison population and level of employee staffing predicted membership in the high-outbreak and high-death profiles when compared with the low-outbreak profile. Housing persons at 85% of prison capacity was associated with lower risk of COVID-19 infection and death. Implementing this 85% standard as an absolute minimum should be prioritized at prisons across the USA.

Vicini, C., et al. (2020). "Overview of different modified full-face snorkelling masks for intraoperative protection." Acta Otorhinolaryngol Ital **40**(5): 317-324.

OBJECTIVE: The COVID-19 pandemic has caused significant impact on healthcare systems worldwide. The rate of infected healthcare workers is > 10% in Italy. Within this dramatic scenario, the development of new personal protective equipment (PPE) devices is mandatory. This study focuses on validation of modified full-face snorkel masks (MFFSM) as safe and protective equipment against SARS-CoV-2 infection during diagnostic and therapeutic procedures on the upper aerodigestive tract. METHODS: Five different MFFSM were tested during otolaryngological surgery and in anaesthesia procedures. Data were collected through an online survey to assess the feedback of operators. pO(2) and pCO(2) monitoring values during procedures were recorded in selected cases. RESULTS: All five MFFSM tested were easy to use and gave all operators a sound "feeling" of protection. All clinicians involved had common agreement regarding safety and the user-friendly format. CONCLUSIONS: In the future, specific development of different type of masks for protection in the operating room, intensive care units and/or office will be possible as a joint venture between clinicians and developers. Goals for clinicians include better definition of needs and priorities, while developers can devote their expertise to produce devices that meet medical requirements.

Villanueva, A. M. G., et al. (2020). "COVID-19 Screening for Healthcare Workers in a Tertiary Infectious Diseases Referral Hospital in Manila, the Philippines." Am J Trop Med Hyg **103**(3): 1211-1214.

COVID-19 is an emerging disease threatening the lives of patients and healthcare workers (HCWs) alike. In this article, we present initial results of COVID-19 screening performed among the hospital staff of an infectious diseases referral hospital in Manila, the Philippines. Of 324 HCWs tested, eight were positive; only one was exposed to COVID-19 patients, whereas seven others belonged to two different departments. Routine screening of hospital staff is invaluable for the safety of the HCWs and the patients in hospitals and should be performed on a regular basis. In monitoring HCWs, we protect one of our most valuable assets against COVID-19.

Vimercati, L., et al. (2021). "COVID-19 hospital outbreaks: Protecting healthcare workers to protect frail patients. An Italian observational cohort study." Int J Infect Dis **102**: 532-537.

OBJECTIVES: To determine the prevalence of SARS-CoV-2 infection among exposed healthcare workers (HCWs) after preventive protocol implementation. METHODS: A total of 5750 HCWs were included in the study. Those in contact with COVID-19 patients were allocated into a high-risk or a low-risk group based on contact type (PPE- or non-PPE-protected); high-risk workers underwent nasopharyngeal swab tests, while among low-risk workers, swab tests were carried out only for symptomatic workers (active surveillance). The prevalence was determined by real-time reverse transcriptase-polymerase chain reaction on nasopharyngeal samples. RESULTS: 3570 HCWs had contact with 1065 COVID-19 patients. Among them, 3494 were subjected to active surveillance (low-risk group); 2886 (82.60%) were subjected to a swab test; and 15 were positive (0.52%). Seventy-six HCWs (2.13% of exposed) were included in the high-risk group, and a swab test was mandatory for each participant. Overall, 66 (86.84% of high-risk) were negative, and 10 were positive (13.16%), resulting in a higher risk of infection than in the low-risk group [OR = 29.00; 95% CI:12.56-66.94; p < 0.0001]. CONCLUSION: To date, the SARS-CoV-2 infection prevalence is 0.70% among exposed HCWs and 0.435% among all HCWs working at the examined university hospital. The correct use of PPE and the early identification of symptomatic workers are essential factors to avoiding nosocomial clusters.

Vimercati, L., et al. (2020). "Prevention and protection measures of healthcare workers exposed to SARS-CoV-2 in a university hospital in Bari, Apulia, Southern Italy." J Hosp Infect **105**(3): 454-458.

Severe acute respiratory syndrome coronavirus-2 (SARS-CoV-2)-related infection has a major impact on public health, and healthcare workers (HCWs) are exposed to high biological risk. This paper describes the prevention procedures introduced at the University Hospital of Bari, Italy to reduce the risk to HCWs, consisting of enhanced preventive measures and activation of a report system to collect HCWs' contacts. Twenty-three confirmed cases of infection (0.4% of all HCWs) were reported in the 30-day observation period following implementation of the protocol. This shows that correct management of HCWs' contacts is essential to avoid nosocomial clusters.

Vimercati, L., et al. (2020). "The COVID-19 hotel for healthcare workers: an Italian best practice." J Hosp Infect **105**(3): 387-388.

Vindrola-Padros, C., et al. (2020). "Perceptions and experiences of healthcare workers during the COVID-19 pandemic in the UK." BMJ Open **10**(11): e040503.

OBJECTIVE: The COVID-19 pandemic has set unprecedented demand on the healthcare workforce around the world. The UK has been one of the most affected countries in Europe. The aim of this study was to explore the perceptions and experiences of healthcare workers (HCWs) in relation to COVID-19 and care delivery models implemented to deal with the pandemic in the UK. METHODS: The study was designed as a rapid appraisal combining: (1) a review of UK healthcare policies (n=35 policies), (2) mass media and social media analysis of front-line staff experiences and perceptions (n=101 newspaper articles, n=1 46 000 posts) and (3) in-depth (telephone) interviews with front-line staff (n=30 interviews). The findings from all streams were analysed using framework analysis. RESULTS: Limited personal protective equipment (PPE) and lack of routine testing created anxiety and distress and had a tangible impact on the workforce. When PPE was available, incorrect size and overheating complicated routine work. Lack of training for redeployed staff and the failure to consider the skills of redeployed staff for new areas were identified as problems. Positive aspects of daily work reported by HCWs included solidarity between colleagues, the establishment of well-being support structures and feeling valued by society. CONCLUSION: Our study highlighted the importance of taking into consideration the experiences and concerns of front-line staff during a pandemic. Staff working in the UK during the COVID-19 pandemic advocated clear and consistent guidelines, streamlined testing of HCWs, administration of PPE and acknowledgement of the effects of PPE on routine practice.

Virk, R. K., et al. (2021). "Impact of COVID-19 pandemic on functioning of cytopathology laboratory: Experience and perspective from an academic centre in New York." Cytopathology **32**(3): 304-311.

COVID-19 has extraordinarily impacted every facet of the health care facilities' operations. Various strategies and policies were implemented promptly to preserve resources, not only to provide medical care to the expected massive numbers of COVID-19 patients, but also to mitigate the contagion spread at the workplace to ensure safety of healthcare workers. All routine, non-essential medical services and procedures were ramped down and workers deemed non-essential were directed to work remotely from home to reduce the number of people at hospital premises and preserve much needed personal protective equipment that were in short supply at the outset of the pandemic. The laboratories did not remain unscathed and were under immense pressure to maintain workplace safety while being operational and provide best patient care with limited resources. In this paper, we share our experience and challenges that we faced in a cytopathology laboratory at a major academic centre in New York, USA during the peak of infection.

Viswanathan, M., et al. (2020). "Universal screening for SARS-CoV-2 infection: a rapid review." Cochrane Database Syst Rev **9**: Cd013718.

BACKGROUND: Coronavirus disease 2019 (COVID-19) is caused by the novel betacoronavirus, severe acute respiratory syndrome coronavirus-2 (SARS-CoV-2). Most people infected with SARS-CoV-2 have mild disease with unspecific symptoms, but about 5% become critically ill with respiratory failure, septic shock and multiple organ failure. An unknown proportion of infected individuals never experience COVID-19 symptoms although they are infectious, that is, they remain asymptomatic. Those who develop the disease, go through a presymptomatic period during which they are infectious. Universal screening for SARS-CoV-2 infections to detect individuals who are infected before they present clinically, could therefore be an important measure to contain the spread of the disease. OBJECTIVES: We conducted a rapid review to assess (1) the effectiveness of universal screening for SARS-CoV-2 infection compared with no screening and (2) the accuracy of universal screening in people who have not presented to clinical care for symptoms of COVID-19. SEARCH METHODS: An information specialist searched Ovid MEDLINE and the Centers for Disease Control (CDC) COVID-19 Research Articles Downloadable Database up to 26 May 2020. We searched Embase.com, the CENTRAL, and the Cochrane Covid-19 Study Register on 14 April 2020. We searched LitCovid to 4 April 2020. The World Health Organization (WHO) provided records from daily searches in Chinese databases and in PubMed up to 15 April 2020. We also searched three model repositories (Covid-Analytics, Models of Infectious Disease Agent Study [MIDAS], and Society for Medical Decision Making) on 8 April 2020. SELECTION CRITERIA: Trials, observational studies, or mathematical modelling studies assessing screening effectiveness or screening accuracy among general populations in which the prevalence of SARS-CoV2 is unknown. DATA COLLECTION AND ANALYSIS: After pilot testing review forms, one review author screened titles and abstracts. Two review authors independently screened the full text of studies and resolved any disagreements by discussion with a third review author. Abstracts excluded by a first review author were dually reviewed by a second review author prior to exclusion. One review author independently extracted data, which was checked by a second review author for completeness and accuracy. Two review authors independently rated the quality of included studies using the Quality Assessment of Diagnostic Accuracy Studies (QUADAS-2) tool for diagnostic accuracy studies and a modified form designed originally for economic evaluations for modelling studies. We resolved differences by consensus. We synthesized the evidence in narrative and tabular formats. We rated the certainty of evidence for days to outbreak, transmission, cases missed and detected, diagnostic accuracy (i.e. true positives, false positives, true negatives, false negatives) using the GRADE approach. MAIN RESULTS: We included 22 publications. Two modelling studies reported on effectiveness of universal screening. Twenty studies (17 cohort studies and 3 modelling studies) reported on screening test accuracy. Effectiveness of screening We included two modelling studies. One study suggests that symptom screening at travel hubs, such as airports, may slightly slow but not stop the importation of infected cases (assuming 10 or 100 infected travellers per week reduced the delay in a local outbreak to 8 days or 1 day, respectively). We assessed risk of bias as minor or no concerns, and certainty of evidence was low, downgraded for very serious indirectness. The second modelling study provides very low-certainty evidence that screening of healthcare workers in emergency departments using laboratory tests may reduce transmission to patients and other healthcare workers (assuming a transmission constant of 1.2 new infections per 10,000 people, weekly screening reduced infections by 5.1% within 30 days). The certainty of evidence was very low, downgraded for high risk of bias (major concerns) and indirectness. No modelling studies reported on harms of screening. Screening test accuracy All 17 cohort studies compared an index screening strategy to a reference reverse transcriptase polymerase chain reaction (RT-PCR) test. All but one study reported on the accuracy of single point-in-time screening and varied widely in prevalence of SARS-CoV-2, settings, and methods of measurement. We assessed the overall risk of bias as unclear in 16 out of 17 studies, mainly due to limited information on the index test and reference standard. We rated one study as being at high risk of bias due to the inclusion of two separate populations with likely different prevalences. For several screening strategies, the estimates of sensitivity came from small samples. For single point-in-time strategies, for symptom assessment, the sensitivity from 12 cohorts (524 people) ranged from 0.00 to 0.60 (very low-certainty evidence) and the specificity from 12 cohorts (16,165 people) ranged from 0.66 to 1.00 (low-certainty evidence). For screening using direct temperature measurement (3 cohorts, 822 people), international travel history (2 cohorts, 13,080 people), or exposure to known infected people (3 cohorts, 13,205 people) or suspected infected people (2 cohorts, 954 people), sensitivity ranged from 0.00 to 0.23 (very low- to low-certainty evidence) and specificity ranged from 0.90 to 1.00 (low- to moderate-certainty evidence). For symptom assessment plus direct temperature measurement (2 cohorts, 779 people), sensitivity ranged from 0.12 to 0.69 (very low-certainty evidence) and specificity from 0.90 to 1.00 (low-certainty evidence). For rapid PCR test (1 cohort, 21 people), sensitivity was 0.80 (95% confidence interval (CI) 0.44 to 0.96; very low-certainty evidence) and specificity was 0.73 (95% CI 0.39 to 0.94; very low-certainty evidence). One cohort (76 people) reported on repeated screening with symptom assessment and demonstrates a sensitivity of 0.44 (95% CI 0.29 to 0.59; very low-certainty evidence) and specificity of 0.62 (95% CI 0.42 to 0.79; low-certainty evidence). Three modelling studies evaluated the accuracy of screening at airports. The main outcomes measured were cases missed or detected by entry or exit screening, or both, at airports. One study suggests very low sensitivity at 0.30 (95% CI 0.1 to 0.53), missing 70% of infected travellers. Another study described an unrealistic scenario to achieve a 90% detection rate, requiring 0% asymptomatic infections. The final study provides very uncertain evidence due to low methodological quality. AUTHORS' CONCLUSIONS: The evidence base for the effectiveness of screening comes from two mathematical modelling studies and is limited by their assumptions. Low-certainty evidence suggests that screening at travel hubs may slightly slow the importation of infected cases. This review highlights the uncertainty and variation in accuracy of screening strategies. A high proportion of infected individuals may be missed and go on to infect others, and some healthy individuals may be falsely identified as positive, requiring confirmatory testing and potentially leading to the unnecessary isolation of these individuals. Further studies need to evaluate the utility of rapid laboratory tests, combined screening, and repeated screening. More research is also needed on reference standards with greater accuracy than RT-PCR. Given the poor sensitivity of existing approaches, our findings point to the need for greater emphasis on other ways that may prevent transmission such as face coverings, physical distancing, quarantine, and adequate personal protective equipment for frontline workers.

Vizheh, M., et al. (2020). "The mental health of healthcare workers in the COVID-19 pandemic: A systematic review." J Diabetes Metab Disord **19**(2): 1-12.

PURPOSE: The novel coronavirus 2019 (COVID-19) is widely spreading all over the world, causing mental health problems for most people. The medical staff is also under considerable psychological pressure. This study aimed to review all research carried out on the mental health status of health care workers (HCWs) to bring policymakers and managers' attention. METHODS: A literature search conducted through e-databases, including PubMed, EMBASE, Scopus, and Web of Science (WoS) from December 2019 up to April 12th 2020. All cross- sectional studies published in English which assessed the health workers' psychological well-being during the SARS-CoV-2 pandemic included. Study quality was analyzed using NHLBI Study Quality assessment tools. RESULTS: One hundred relevant articles were identified through systematic search; of which eleven studies were eligible for this review. Their quality score was acceptable. The lowest reported prevalence of anxiety, depression, and stress among HCWs was 24.1%, 12.1%, and 29.8%, respectively. In addition, the highest reported values for the aforementioned parameters were 67.55%, 55.89%, and 62.99%, respectively. Nurses, female workers, front-line health care workers, younger medical staff, and workers in areas with higher infection rates reported more severe degrees of all psychological symptoms than other health care workers. Moreover, vicarious traumatization in non-front-line nurses and the general public was higher than that of the front-line nurses. CONCLUSION: During SARS-CoV-2 outbreak, the health care workers face aggravated psychological pressure and even mental illness. It would be recommended to the policymakers and managers to adopt the supportive, encouragement & motivational, protective, and training & educational interventions, especially through information and communication platform.

Vlachoyiannopoulos, P., et al. (2020). "Anti-SARS-CoV-2 antibody detection in healthcare workers of two tertiary hospitals in Athens, Greece." Clin Immunol **221**: 108619.

Volo, T., et al. (2021). "Elective tracheostomy during COVID-19 outbreak: to whom, when, how? Early experience from Venice, Italy." Eur Arch Otorhinolaryngol **278**(3): 781-789.

PURPOSE: The need for prolonged invasive mechanical ventilation in COVID-19 patients is placing the otorhinolaryngologist in front of an increasing request for tracheostomy. Nowadays, there is uncertainty regarding the timing of tracheostomy, the prognosis of these patients and the safety of healthcare workers. The aim of this study is to evaluate the efficacy and safety of tracheostomy placement in patients with COVID-19. METHODS: A retrospective cohort study on 23 COVID 19 patients, to analyse the timing of tracheostomy, the risk factors associated with in-hospital death and the infection of the involved health care workers. Early tracheostomy was defined as ≤ 10 days and late ones > 10 days. RESULTS: The mortality rate of COVID-19 patients admitted to ICU that underwent tracheostomy was 18%. The overall mortality of patients admitted to ICU was 53%. The univariate analysis revealed that early tracheostomy, SOFA score > 6, and D-dimer level > 4 were significantly associated with a greater risk of death. At the multivariate analysis SOFA score > 6 and D-dimer level > 4 resulted as significant factors for a higher risk of death. No health care workers associated with tracheostomy are confirmed to be infected by SARS-CoV2. CONCLUSION: We suggest to wait at least 14 days to perform tracheostomy. In patients with SOFA score > 6 and D dimer > 4, tracheostomy should not be performed or should be postponed. Optimized procedures and enhanced personal protective equipment can make the tracheostomy safe and beneficial in COVID-19 patients.

von Freyburg, A., et al. (2020). "[COVID-19 cluster study at a teaching hospital]." MMW Fortschr Med **162**(9): 64-67.

On Mar 11th, 2020, the World Health Organization (WHO) stated in its Situation Report - 51 Coronavirus disease 2019 (COVID-19) as a pandemic. In early April 2020, a teaching hospital underwent shutdown and quarantine due to an outbreak of infection in accordance with Section 6 of the Infection Protection Act (index patient and 5 infected nursing staff). The complete staff (physicians, nurses and nonmedical personnel [NMP]) underwent COVID-19 testing within two phases: (1) between Apr 3rd and 5th, 2020 [n=1170], followed by (2) between Apr 8th and 9th, 2020 [n=953] with COVID-19 silent carrier positivity rates in accordance to testing phases of (1) n=19 (1.6%) and (2) n=25 (2.6%). The cumulative infection rate for NMP (1.6%), doctors (3.8%) and nurses (9.7%) was connected to type and extent of COVID-19 patient contact. Despite COVID-19 positivity of 34.8% (46 of 132 beds), a risk-free management of hospital operation is possible to a certain extent if hygiene regulations and strict patient selection are followed. However, a COVID-19-free clinic cannot be expected due to silent carriers.

von Rüden, U., et al. (2021). "[Demand-driven communication strategy of the Federal Centre for Health Education (BZgA) during the COVID-19 pandemic]." Bundesgesundheitsblatt Gesundheitsforschung Gesundheitsschutz **64**(3): 285-293.

With the declaration of a pandemic situation of national significance by the German Bundestag, the Federal Centre for Health Education (BZgA) is fulfilling its task of providing information for understanding the pandemic situation and implementing the necessary protective measures in a clear manner throughout Germany within the framework of the adapted National Pandemic Plan COVID-19. The BZgA targets its information according to the needs of specific groups and actively involves multipliers.In order to incorporate the perspectives of the population as well as those of prevention and health promotion professionals into the services developed by the BZgA, given the particularly initial low level of knowledge in the population, various methods and data sources were used: the COVID-19 Snapshot Monitoring (COSMO) population survey, the monitoring of citizens' enquiries via telephone and e‑mail to the BZgA, surveys of counselling professionals from nationwide telephone and online counselling centres, surveys of health professionals in early help (NZFH) and interdisciplinary practice expert hearings. Beyond providing pure information, practical and everyday offers should be developed and provided according to WHO guidelines and evidence-based criteria of effective communication in order to support the competences for a gradual adaptation to a "new normality".The paper describes the data-based and evidence-informed development process of communication content and offers, their dissemination via existing websites and channels for other topics as well as their integration into the new online platform www.zusammengegencorona.de . This demonstrates how demand-driven and target group-specific communication offers can be implemented beyond the classic and proven campaign appearance.

Voss, A., et al. (2020). "[Rational use of respiratory protective equipment: advice for health care professionals in time of COVID-19]." Ned Tijdschr Geneeskd **164**.

The current COVID-19 pandemic has led to a worldwide shortage of respiratory protective equipment. In order to offer maximum protection against infection for all healthcare workers, we need to optimise our use of the available equipment. This article provides practical advice on which type of mask is indicated in what specific situation, what requirements the mask should meet and how to optimise the local workflow, including the re-use of masks after decontamination.

Wachocka, M., et al. (2020). "Health Professionals of Prevention in Italy: The Value of Expertise During COVID-19 Pandemic." Front Public Health **8**: 575500.

There are 22 different degree courses related to the Healthcare Professions in the Italian university system, which are divided into four areas. "Healthcare Professions of Prevention" is the fourth area and it is fundamental for the National Health Service. In particular, in this pandemic emergency situation, the contribution of the Prevention Technicians in the Environment and Workplaces (PTEW) is essential in the field and workplace management. The "Core Competence" of the PTEW is to carry out, with professional autonomy, prevention, verification, and control activities in the field of hygiene and safety of living and working environments. In the hospitals, the indications provided by national and/or regional authorities are implemented through procedures on good hygiene practices developed by PTEW (e.g., hand hygiene, "respiratory tract hygiene," environmental hygiene, social distancing, and use of Personal Protective Equipment). One of the activities is the health surveillance on the field by population monitoring. The protocols foreseen for the "in-flow of workers" involve a wider control between social life and work. The PTEW will use a Check List divided into 3 macro phases: Entry, Activity Context, and Exit, defining each behavior of the work phases with a constant presence of verification of the procedures. The PTEW will be a Leader on the topics of education, training, and persuasion, considering a New Principle that "transforms the worker as active part in the application and diffusion of the safety measures".

Walker, G. and T. McCabe (2021). "Psychological defence mechanisms during the COVID-19 pandemic: A case series." Eur J Psychiatry **35**(1): 41-45.

BACKGROUND AND OBJECTIVES: COVID-19 has had a negative effect on mental health across the world's population. Healthcare workers in particular have experienced increased levels of psychological distress, depression and anxiety. Any perceived stress to an individual can provoke psychological defence mechanisms. Using psychoanalytic theory, a defence mechanism is described as an unconscious psychological strategy, with or without resulting behaviour, which aims to reduce or eliminate anxiety arising from unacceptable or potentially harmful stimuli. This paper aims to describe a range of psychological defence mechanisms encountered within colleagues in relation to the COVID-19 pandemic. METHODS: Using the methodology of a case series, specific defence mechanisms are explored with reference to further literature in the field. RESULTS: The author has encountered varying psychological defence mechanisms, both within himself and in other members of the multidisciplinary team. These have been illustrated in the attached clinical vignettes, relating to the specific psychological coping mechanisms of; denial, hypochondriasis, altruism, sublimation and humour. CONCLUSION: We encourage acknowledgement of psychological defence mechanisms and their implications on day to day practice. Whilst defence mechanisms can have a number of negative consequences as described in this article, they also have an important role, particularly in the case of mature defence mechanisms, as protective factors against psychological distress and symptom formation. Deeper understanding of the gold-standard hierarchical organisation of defence mechanisms could help increase utilisation of specific therapeutic interventions for enhancing changes from immature to mature defensive responses to stressful experiences as the COVID-19 pandemic progresses.

Wallace, D. W., et al. (2020). "An adapted emergency department triage algorithm for the COVID-19 pandemic." J Am Coll Emerg Physicians Open **1**(6): 1374-1379.

The novel coronavirus disease 2019 (COVID-19) pandemic, with its public health implications, high case fatality rate, and strain on hospital resources, will continue to challenge clinicians and researchers alike for months to come. Accurate triage of patients during the pandemic will assign patients to the appropriate level of care, provide the best care for the maximum number of patients, rationally limit personal protective equipment (PPE) usage, and mitigate nosocomial exposures. The authors describe an adapted COVID-19 pandemic triage algorithm for emergency departments (EDs) guided by the best available evidence and responses to prior pandemics, with recommendations for clinician PPE use for each level of encounter in the setting of an ongoing PPE shortage. Our algorithm adheres to Centers for Disease Control and Prevention guidelines and supports discharge of patients with mild symptoms coupled with explicit and strict return precautions and infection control education.

Wang, H., et al. (2020). "Healthcare workers' stress when caring for COVID-19 patients: An altruistic perspective." Nurs Ethics **27**(7): 1490-1500.

BACKGROUND: When the contagious COVID-19 spread worldwide, the frontline staff faced unprecedented excessive work pressure and expectations of all of the society. OBJECTIVE: The aim was to explore healthcare workers' stress and influencing factors when caring for COVID-19 patients from an altruistic perspective. METHODS: A cross-sectional, descriptive study was conducted in a tertiary hospital during the outbreak of COVID-19 between February and March 2020 in Wuhan, the capital city of Hubei province in China. Data were collected from 1208 healthcare workers. Descriptive statistics and multiple linear regression were used to analyze the data. ETHICAL CONSIDERATIONS: Research ethics approval (with the code of TJ-IRB20200379) was obtained from Tongji Hospital, Tongji Medical College of Huazhong University of Science and Technology. Written informed consent was also received from participants. RESULTS: Less than 60% of participants chose moderate or severe stress on all stressors, indicating a low stress level among healthcare workers. The main source of stress among frontline healthcare workers caring for COVID-19 patients came from the fear of being infected, the fear of family members being infected, and the discomfort caused by protective equipment. Frontline staff who were nurses, were married, and had worked more than 20 days suffered higher stress, whereas rescue staff showed lower stress. CONCLUSION: The healthcare workers caring for patients with COVID-19 had low stress level, although they still had the fear of being infected or uncomfortable feeling caused by personal protective equipment. A low stress level among healthcare workers indicated their professional devotion and altruism during COVID-19 epidemic. Medical institutions and the government should continue to strengthen infection prevention measures and provide more comprehensive care involving families of frontline healthcare workers, especially nurses and married staff. It will be a lesson to other countries that awaking healthcare workers' inside motivation and providing necessary support from government and society were significant.

Wang, J., et al. (2021). "The physical and mental health of the medical staff in Wuhan Huoshenshan Hospital during COVID-19 epidemic: A Structural Equation Modeling approach." Eur J Integr Med **44**: 101323.

INTRODUCTION: Early in the epidemic of coronavirus disease 2019, the Chinese government recruited a proportion of healthcare workers to support the designated hospital (Huoshenshan Hospital) in Wuhan, China. The majority of front-line medical staff suffered from adverse effects, but their real health status during COVID-19 epidemic was still unknown. The aim of the study was to explore the latent relationship of the physical and mental health of front-line medical staff during this special period. METHODS: A total of 115 military medical staff were recruited between February 17th and February 29th, 2020 and asked to complete questionnaires assessing socio-demographic and clinical characteristics, self-reported sleep status, fatigue, resilience and anxiety. RESULTS: 55 medical staff worked within Intensive Care and 60 worked in Non-intensive Care, the two groups were significantly different in reported general fatigue, physical fatigue and tenacity (P<0.05). Gender, duration working in Wuhan, current perceived stress level and health status were associated with significant differences in fatigue scores (P<0.05), the current perceived health status (P<0.05) and impacted on the resilience and anxiety of participants. The structural equation modeling analysis revealed resilience was negatively associated with fatigue (β=-0.52, P<0.01) and anxiety (β=-0.24, P<0.01), and fatigue had a direct association with the physical burden (β=0.65, P<0.01); Fatigue mediated the relationship between resilience and anxiety (β=-0.305, P=0.039) as well as resilience and physical burden (β=-0.276, P=0.02). CONCLUSION: During an explosive pandemic situation, motivating the effect of protective resilience and taking tailored interventions against fatigue are promising ways to protect the physical and mental health of the front-line medical staff.

Wang, J., et al. (2020). "Reasons for healthcare workers becoming infected with novel coronavirus disease 2019 (COVID-19) in China." J Hosp Infect **105**(1): 100-101.

Wang, N., et al. (2021). "Communication Chaos from Discrepancies in Personal Protective Equipment and Preoperative Guidelines." Laryngoscope **131**(3): E746-e754.

OBJECTIVES/HYPOTHESIS: To compare personal protective equipment (PPE) guidelines, specifically respirator use, among international public health agencies, academic hospitals, and otolaryngology-head and neck surgery (OHNS) departments in the United States for the care of coronavirus-19 (COVID-19) patients. STUDY DESIGN: Cross sectional survey. METHODS: Review of publicly available public health and academic hospitals guidelines along with review of communication among otolaryngology departments. RESULTS: Among 114 academic institutions affiliated with OHNS residencies, 20 (17.5%) institutions provided public access to some form of guidance on PPE and 73 (64%) provided information on screening or diagnostic testing. PPE guidelines were uniquely described based on several variables: location of care, COVID-19 status, involvement of aerosol generating or high-risk procedures, and physical distance from the patient. Six hospital guidelines were highlighted. Across these six institutions, there was agreement that N95 respirators were needed for high-risk patients undergoing high-risk procedures. Variations existed among institutions for scenarios with low-risk patients. Definitions of the low-risk patient and high-risk procedures were inconsistent among institutions. Three of the highlighted institutions had OHNS departments recommending higher level of airway protection than the institution. CONCLUSIONS: OHNS departments typically had more stringent PPE guidance than their institution. Discrepancies in communicating PPE use were frequent and provide inconsistent information on how healthcare workers should protect themselves in the COVID-19 pandemic. Identification of these inconsistencies serves as an opportunity to standardize communication and develop evidence-based guidelines. LEVEL OF EVIDENCE: V Laryngoscope, 131:E746-E754, 2021.

Wang, P., et al. (2021). "Spatiotemporal characteristics and factor analysis of SARS-CoV-2 infections among healthcare workers in Wuhan, China." J Hosp Infect **110**: 172-177.

BACKGROUND: Studying the spatiotemporal distribution of SARS-CoV-2 infections among healthcare workers (HCWs) can aid in protecting them from exposure. AIM: To describe the spatiotemporal distributions of SARS-CoV-2 infections among HCWs in Wuhan, China. METHODS: In this study, an open-source dataset of HCW diagnoses was provided. A geographical detector technique was then used to investigate the impacts of hospital level, type, distance from the infection source, and other external indicators of HCW infections. FINDINGS: The number of daily HCW infections over time in Wuhan followed a log-normal distribution, with its mean observed on January 23(rd), 2020, and a standard deviation of 10.8 days. The implementation of high-impact measures, such as the lockdown of the city, may have increased the probability of HCW infections in the short term, especially for those in the outer ring of Wuhan. The infection of HCWs in Wuhan exhibited clear spatial heterogeneity. The number of HCW infections was higher in the central city and lower in the outer city. CONCLUSION: HCW infections displayed significant spatial autocorrelation and dependence. Factor analysis revealed that hospital level and type had an even greater impact on HCW infections; third-class and general hospitals closer to infection sources were correlated with especially high risks of infection.

Wang, Y., et al. (2020). "Measures for preventing nosocomial infection with SARS-CoV-2 in hematology departments." Ann Hematol **99**(8): 1933-1938.

Wang, Y., et al. (2020). "Acute psychological effects of Coronavirus Disease 2019 outbreak among healthcare workers in China: a cross-sectional study." Transl Psychiatry **10**(1): 348.

To study the acute psychological effects of Coronavirus Disease 2019 (COVID-19) outbreak among healthcare workers (HCWs) in China, a cross-sectional survey was conducted among HCWs during the early period of COVID-19 outbreak. The acute psychological effects including symptoms of depression, anxiety, and post-traumatic stress disorder (PTSD) were assessed using the Patient Health Questionnaire-9 (PHQ-9), the Generalized Anxiety Disorder (GAD-7) questionnaire, and the Impact of Event Scale-Revised (IES-R). The prevalence of depression, anxiety, and PTSD was estimated at 15.0%, 27.1%, and 9.8%, respectively. Having an intermediate technical title, working at the frontline, receiving insufficient training for protection, and lacking confidence in protection measures were significantly associated with increased risk for depression and anxiety. Being a nurse, having an intermediate technical title, working at the frontline, and lacking confidence in protection measures were risk factors for PTSD. Meanwhile, not worrying about infection was a protective factor for developing depression, anxiety, and PTSD. Psychological interventions should be implemented among HCWs during the COVID-19 outbreak to reduce acute psychological effects and prevent long-term psychological comorbidities. Meanwhile, HCWs should be well trained and well protected before their frontline exposure.

Wang, Y. and Q. Yang (2021). "Post Abortion Care and Management After Induced Abortion During the COVID-19 Pandemic: A Chinese Expert Consensus." Adv Ther **38**(2): 1011-1023.

The rapid spread of novel coronavirus (COVID-19) has posed complex challenges to global public health. During this pandemic period, access to essential services including post-abortion care (PAC) has been disrupted. Along with the clinical management of the disease in women, protection of the healthcare workers and medical staff from nosocomial infection is important to ensure infection control. Thus, in order to implement the proper contraceptive measures and to reduce the rate of repeated abortion, the family planning group of minimally invasive gynecological branch of the Liaoning Medical Association organized a committee of experts to formulate guidance and suggestions to ensure the timely treatment and surgery of women opting for abortion, the implementation of PAC, implementation of safe contraceptive measures after surgery, and the protection of healthcare professionals and medical staff from infection. We believe these guidelines might be helpful for obstetrics and gynecology departments in China and globally, as well for women who wish to undergo abortion during these unprecedented times.

Wang, Y., et al. (2020). "Recommendations of protective measures for orthopedic surgeons during COVID-19 pandemic." Knee Surg Sports Traumatol Arthrosc **28**(7): 2027-2035.

PURPOSE: It was the primary purpose of the present systematic review to identify the optimal protection measures during COVID-19 pandemic and provide guidance of protective measures for orthopedic surgeons. The secondary purpose was to report the protection experience of an orthopedic trauma center in Wuhan, China during the pandemic. METHODS: A systematic search of the PubMed, Cochrane, Web of Science, Google Scholar was performed for studies about COVID-19, fracture, trauma, orthopedic, healthcare workers, protection, telemedicine. The appropriate protective measures for orthopedic surgeons and patients were reviewed (on-site first aid, emergency room, operating room, isolation wards, general ward, etc.) during the entire diagnosis and treatment process of traumatic patients. RESULTS: Eighteen studies were included, and most studies (13/18) emphasized that orthopedic surgeons should pay attention to prevent cross-infection. Only four studies have reported in detail how orthopedic surgeons should be protected during surgery in the operating room. No detailed studies on multidisciplinary cooperation, strict protection, protection training, indications of emergency surgery, first aid on-site and protection in orthopedic wards were found. CONCLUSION: Strict protection at every step in the patient pathway is important to reduce the risk of cross-infection. Lessons learnt from our experience provide some recommendations of protective measures during the entire diagnosis and treatment process of traumatic patients and help others to manage orthopedic patients with COVID-19, to reduce the risk of cross-infection between patients and to protect healthcare workers during work. LEVEL OF EVIDENCE: IV.

Wanigasooriya, K., et al. (2020). "Mental health symptoms in a cohort of hospital healthcare workers following the first peak of the COVID-19 pandemic in the UK." BJPsych Open **7**(1): e24.

BACKGROUND: The coronavirus disease 2019 (COVID-19) pandemic is likely to lead to a significant increase in mental health disorders among healthcare workers (HCW). AIMS: We evaluated the rates of anxiety, depressive and post-traumatic stress disorder (PTSD) symptoms in a population of HCW in the UK. METHOD: An electronic survey was conducted between the 5 June 2020 and 31 July 2020 of all hospital HCW in the West Midlands, UK using clinically validated questionnaires: the 4-item Patient Health Questionnaire(PHQ-4) and the Impact of Event Scale-Revised (IES-R). Univariate analyses and adjusted logistic regression analyses were performed to estimate the strengths in associations between 24 independent variables and anxiety, depressive or PTSD symptoms. RESULTS: There were 2638 eligible participants who completed the survey (female: 79.5%, median age: 42 years, interquartile range: 32-51). The rates of clinically significant symptoms of anxiety, depression and PTSD were 34.3%, 31.2% and 24.5%, respectively. In adjusted analysis a history of mental health conditions was associated with clinically significant symptoms of anxiety (odds ratio (OR) = 2.3, 95% CI 1.9-2.7, P < 0.001), depression (OR = 2.5, 95% CI 2.1-3.0, P < 0.001) and PTSD (OR = 2.1, 95% CI 1.7-2.5, P < 0.001). The availability of adequate personal protective equipment (PPE), well-being support and lower exposure to moral dilemmas at work demonstrated significant negative associations with these symptoms (P ≤ 0.001). CONCLUSIONS: We report higher rates of clinically significant mental health symptoms among hospital HCW following the initial COVID-19 pandemic peak in the UK. Those with a history of mental health conditions were most at risk. Adequate PPE availability, access to well-being support and reduced exposure to moral dilemmas may protect hospital HCW from mental health symptoms.

Wataganara, T., et al. (2020). "Minimizing cross transmission of SARS-CoV-2 in obstetric ultrasound during COVID-19 pandemic." J Perinat Med **48**(9): 931-942.

Severe acute respiratory syndrome coronavirus-2 (SARS-CoV-2)-associated infection (COVID-19) is affecting populations worldwide. This statement may serve as guidance for infection prevention and safe ultrasound practices during the COVID-19 pandemic. Ultrasound examination is a fundamental part of obstetric care, yet it is a potential vector for transmission of SARS-CoV-2. Decontamination methods should always be implemented for ultrasound equipment, especially in the presence of suspected or confirmed COVID-19 cases. There must be workflow policies to protect pregnant women and healthcare providers from nosocomial cross transmission of SARS-CoV-2. Cleaning and disinfecting of equipment must be in accordance with their potential of pathogen transmission. Consider using telemedicine and genetic technologies as an adjunctive of obstetric ultrasound to reduce patient crowding. Patient triage and education of healthcare providers of infection prevention are crucial to minimize cross contamination of SARS-CoV-2 during obstetric ultrasound.

Wax, R. S. and M. D. Christian (2020). "Practical recommendations for critical care and anesthesiology teams caring for novel coronavirus (2019-nCoV) patients." Can J Anaesth **67**(5): 568-576.

A global health emergency has been declared by the World Health Organization as the 2019-nCoV outbreak spreads across the world, with confirmed patients in Canada. Patients infected with 2019-nCoV are at risk for developing respiratory failure and requiring admission to critical care units. While providing optimal treatment for these patients, careful execution of infection control measures is necessary to prevent nosocomial transmission to other patients and to healthcare workers providing care. Although the exact mechanisms of transmission are currently unclear, human-to-human transmission can occur, and the risk of airborne spread during aerosol-generating medical procedures remains a concern in specific circumstances. This paper summarizes important considerations regarding patient screening, environmental controls, personal protective equipment, resuscitation measures (including intubation), and critical care unit operations planning as we prepare for the possibility of new imported cases or local outbreaks of 2019-nCoV. Although understanding of the 2019-nCoV virus is evolving, lessons learned from prior infectious disease challenges such as Severe Acute Respiratory Syndrome will hopefully improve our state of readiness regardless of the number of cases we eventually manage in Canada.

Wee, L. E., et al. (2020). "Experience of a Tertiary Hospital in Singapore with Management of a Dual Outbreak of COVID-19 and Dengue." Am J Trop Med Hyg **103**(5): 2005-2011.

During the COVID-19 pandemic, distinguishing dengue from cases of COVID-19 in endemic areas can be difficult. In a tertiary hospital contending with COVID-19 during a dengue epidemic, a triage strategy of routine COVID-19 testing for febrile patients with viral prodromes was used. All febrile patients with viral prodromes and no epidemiologic risk for COVID-19 were first admitted to a designated ward for COVID-19 testing, where enhanced personal protective equipment was used by healthcare workers until COVID-19 was ruled out. From January to May 2020, 11,086 admissions were screened for COVID-19; 868 cases of COVID-19 were diagnosed in our institution, along with 380 cases of dengue. Only 8.5% (943/11,086) of suspected COVID-19 cases were concurrently tested for dengue serology due to a compatible overlapping clinical syndrome, and dengue was established as an alternative diagnosis in 2% (207/10,218) of suspected COVID-19 cases that tested negative. There were eight COVID-19 cases with likely false-positive dengue serology and one probable COVID-19/dengue coinfection. From April to May 2020, 251 admissions presenting as viral prodromes with no respiratory symptoms were screened; of those, 15 cases had COVID-19, and 2/15 had false-positive dengue IgM. Epidemiology investigations showed no healthcare-associated transmission. In a dengue epidemic season coinciding with a COVID-19 pandemic, dengue was established as an alternative diagnosis in a minority of COVID-19 suspects, likely due to early availability of basic diagnostics. Routine screening of patients with viral prodromes during a dual outbreak of COVID-19 and dengue enabled containment of COVID-19 cases masquerading as dengue with false-positive IgM.

Wee, L. E., et al. (2020). "Containing COVID-19 in the Emergency Department: The Role of Improved Case Detection and Segregation of Suspect Cases." Acad Emerg Med **27**(5): 379-387.

OBJECTIVES: Patients with COVID-19 may present with respiratory syndromes indistinguishable from common viruses. This poses a challenge for early detection during triage in the emergency department (ED). Over a 3-month period, our ED aimed to minimize nosocomial transmission by using broader suspect case criteria for better detection and using appropriate personal protective equipment (PPE) for health care workers (HCWs). METHODS: All ED admissions with respiratory syndromes over a 3-month period were tested for COVID-19. The sensitivity and specificity of screening criteria in detecting COVID-19 were assessed. A risk-stratified approach was adopted for PPE usage in the ED, based on high-risk "fever areas" and lower-risk zones. When a case of COVID-19 was confirmed, surveillance was conducted for potentially exposed patients and HCWs. RESULTS: A total of 1,841 cases presenting with respiratory syndromes required admission over the study period. Among these, 70 cases of COVID-19 were subsequently confirmed. The majority (84.2%, 59/70) were detected at ED triage because they fulfilled suspect case criteria. Of these, 34 met the official screening criteria; an additional 25 were detected by the broader internal screening criteria. Over the 12-week period, the cumulative sensitivity of internal screening criteria was 84.3% (95% confidence interval [CI] = 73.6% to 91.9%), whereas the sensitivity of the official screening criteria was 48.6% (95% CI = 36.4% to 60.8%). Given the broadened internal criteria, the preexisting ED "fever area" was insufficient and had to be expanded. However, there were no cases of nosocomial transmission from intra-ED exposure, despite extensive surveillance. CONCLUSION: Frontline physicians need to be given leeway to decide on the disposition of cases based on clinical suspicion during an ongoing outbreak of COVID-19. If a broader criterion is used at ED triage, ED facilities and isolation facilities need to be readied to accommodate a surge of suspect cases. Usage of appropriate PPE is essential in minimizing nosocomial transmission.

Wee, L. E., et al. (2020). "Respiratory surveillance wards as a strategy to reduce nosocomial transmission of COVID-19 through early detection: The experience of a tertiary-care hospital in Singapore." Infect Control Hosp Epidemiol **41**(7): 820-825.

OBJECTIVES: Patients with COVID-19 may present with respiratory syndromes indistinguishable from those caused by common viruses. Early isolation and containment is challenging. Although screening all patients with respiratory symptoms for COVID-19 has been recommended, the practicality of such an effort has yet to be assessed. METHODS: Over a 6-week period during a SARS-CoV-2 outbreak, our institution introduced a "respiratory surveillance ward" (RSW) to segregate all patients with respiratory symptoms in designated areas, where appropriate personal protective equipment (PPE) could be utilized until SARS-CoV-2 testing was done. Patients could be transferred when SARS-CoV-2 tests were negative on 2 consecutive occasions, 24 hours apart. RESULTS: Over the study period, 1,178 patients were admitted to the RSWs. The mean length-of-stay (LOS) was 1.89 days (SD, 1.23). Among confirmed cases of pneumonia admitted to the RSW, 5 of 310 patients (1.61%) tested positive for SARS-CoV-2. This finding was comparable to the pickup rate from our isolation ward. In total, 126 HCWs were potentially exposed to these cases; however, only 3 (2.38%) required quarantine because most used appropriate PPE. In addition, 13 inpatients overlapped with the index cases during their stay in the RSW; of these 13 exposed inpatients, 1 patient subsequently developed COVID-19 after exposure. No patient-HCW transmission was detected despite intensive surveillance. CONCLUSIONS: Our institution successfully utilized the strategy of an RSW over a 6-week period to contain a cluster of COVID-19 cases and to prevent patient-HCW transmission. However, this method was resource-intensive in terms of testing and bed capacity.

Wee, L. E., et al. (2020). "Re: 'Personal protective equipment protecting healthcare workers in the Chinese epicenter of COVID-19' by Zhao et al." Clin Microbiol Infect **26**(12): 1719-1721.

Wee, L. E., et al. (2020). "Early Recognition of Coronavirus 2019 Disease (COVID-19) Infection in Surgical Inpatients: The Importance of a Risk-Stratified Approach for Early Testing and Isolation." Surg Infect (Larchmt) **21**(9): 760-765.

Background: In the ongoing coronavirus disease 2019 (COVID-19) pandemic, resuming provision of surgical services poses a challenge given that patients may have acute surgical pathologies with concurrent COVID-19 infection. We utilized a risk-stratified approach to allow for early recognition and isolation of potential COVID-19 infection in surgical patients, ensuring continuity of surgical services during a COVID-19 outbreak. Patients and Methods: Over a four-month period from January to April 2020, surgical patients admitted with concurrent respiratory symptom, infiltrates on chest imaging, or suspicious travel/epidemiologic history were placed in a dedicated ward in which they were tested for severe acute respiratory syndrome coronavirus-2 (SARS-CoV-2). If emergency operations were necessary prior to the exclusion of COVID-19, patients were managed as per suspected cases of COVID-19, with appropriate precautions and full personal protective equipment (PPE). Results: From January through April 2020, a total of 8,437 patients were admitted to our surgical department; 5.9% (498/8437) required peri-operative testing for SARS-CoV-2. Because testing was in-house with turnaround within 24 hours, only a small number of emergency operations (n = 10) were conducted for suspected COVID-19 cases prior to results; none tested positive. The testing yield was lower in surgical inpatients compared with medical inpatients (odds ratio [OR] = 0.20, 95% confidence interval [CI], 0.12-0.32, p < 0.001). Three operations were conducted in known COVID-19 cases; all healthcare workers (HCWs) used full PPE. A risk-stratified testing strategy picked up previously unsuspected COVID-19 in six cases; 66.7% (4/6) were asymptomatic at presentation. Although 48 HCWs were exposed to these six cases, delayed diagnosis was averted and no evidence of spread to patients or HCWs was detected. Conclusion: A risk-stratified approach allowed for early recognition, testing, and isolation of potential COVID-19 infection in surgical patients, ensuring continuity of surgical services.

Wee, L. E. I., et al. (2020). "Containing COVID-19 outside the isolation ward: The impact of an infection control bundle on environmental contamination and transmission in a cohorted general ward." Am J Infect Control **48**(9): 1056-1061.

BACKGROUND: During an ongoing outbreak of COVID-19, unsuspected cases may be housed outside of dedicated isolation wards. AIM: At a Singaporean tertiary hospital, individuals with clinical syndromes compatible with COVID-19 but no epidemiologic risk were placed in cohorted general wards for COVID-19 testing. To mitigate risk, an infection control bundle was implemented comprising infrastructural enhancements, improved personal protective equipment, and social distancing. We assessed the impact on environmental contamination and transmission. METHODS: Upon detection of a case of COVID-19 in the dedicated general ward, patients and health care workers (HCWs) contacts were identified. All patient and staff close-contacts were placed on 14-day phone surveillance and followed up for 28 days; symptomatic contacts were tested. Environmental samples were also obtained. FINDINGS: Over a 3-month period, 28 unsuspected cases of COVID-19 were contained in the dedicated general ward. In 5 of the 28 cases, sampling of the patient's environment yielded SARS-CoV-2; index cases who required supplemental oxygen had higher odds of environmental contamination (P = .01). A total of 253 staff close-contacts and 45 patient close-contacts were identified; only 3 HCWs (1.2%, 3/253) required quarantine. On 28-day follow-up, no patient-to-HCW transmission was documented; only 1 symptomatic patient close-contact tested positive. CONCLUSIONS: Our institution successfully implemented an intervention bundle to mitigate COVID-19 transmission in a multibedded cohorted general ward setting.

Wei, H., et al. (2021). "Controversies in airway management of COVID-19 patients: updated information and international expert consensus recommendations." Br J Anaesth **126**(2): 361-366.

Wei, J. T., et al. (2020). "Epidemiology of and Risk Factors for COVID-19 Infection among Health Care Workers: A Multi-Centre Comparative Study." Int J Environ Res Public Health **17**(19).

Healthcare workers (HCWs) worldwide are putting themselves at high risks of coronavirus disease 2019 (COVID-19) by treating a large number of patients while lacking protective equipment. We aim to provide a scientific basis for preventing and controlling the COVID-19 infection among HCWs. We used data on COVID-19 cases in the city of Wuhan to compare epidemiological characteristics between HCWs and non-HCWs and explored the risk factors for infection and deterioration among HCWs based on hospital settings. The attack rate (AR) of HCWs in the hospital can reach up to 11.9% in Wuhan. The time interval from symptom onset to diagnosis in HCWs and non-HCWs dropped rapidly over time. From mid-January, the median time interval of HCW cases was significantly shorter than in non-HCW cases. Cases of HCWs and non-HCWs both clustered in northwestern urban districts rather than in rural districts. HCWs working in county-level hospitals in high-risk areas were more vulnerable to COVID-19. HCW cases working in general, ophthalmology, and respiratory departments were prone to deteriorate compared with cases working in the infection department. The AR of COVID-19 in HCWs are higher than in non-HCWs. Multiple factors in hospital settings may play important roles in the transmission of COVID-19. Effective measures should be enhanced to prevent HCWs from COVID-19 infection.

Wei, L., et al. (2020). "Contamination of SARS-CoV-2 in patient surroundings and on personal protective equipment in a non-ICU isolation ward for COVID-19 patients with prolonged PCR positive status." Antimicrob Resist Infect Control **9**(1): 167.

OBJECTIVES: We performed an environmental sampling study to investigate the environmental contamination of SARS-CoV-2 by COVID-19 patients with prolonged PCR positive status of clinical samples. METHODS: We sampled the air from rooms for nine COVID-19 patients with illness or positive PCR > 30 days, before and after nasopharyngeal/oropharyngeal swabbing and before and after nebulization treatment. We also sampled patients' surroundings and healthcare workers' personal protection equipment (PPE) in a non-ICU ward. SARS-CoV-2 was detected by PCR. RESULTS: Eighty-eight samples were collected from high-touch surfaces and floors in patient rooms and toilets, with only the bedsheets of two patients and one toilet positive for SARS-CoV-2. All air samples (n = 34) were negative for SARS-CoV-2. Fifty-five samples collected from PPE were all negative. CONCLUSION: Contamination of near-patient surroundings was uncommon for COVID-19 patients with prolonged PCR positive status if environmental cleaning/disinfection were performed rigorously. Airborne transmission of SARS-CoV-2 was unlikely in these non-ICU settings.

Wesemann, C., et al. (2020). "3-D Printed Protective Equipment during COVID-19 Pandemic." Materials (Basel) **13**(8).

While the number of coronavirus cases from 2019 continues to grow, hospitals are reporting shortages of personal protective equipment (PPE) for frontline healthcare workers. Furthermore, PPE for the eyes and mouth, such as face shields, allow for additional protection when working with aerosols. 3-D printing enables the easy and rapid production of lightweight plastic frameworks based on open-source data. The practicality and clinical suitability of four face shields printed using a fused deposition modeling printer were examined. The weight, printing time, and required tools for assembly were evaluated. To assess the clinical suitability, each face shield was worn for one hour by 10 clinicians and rated using a visual analogue scale. The filament weight (21-42 g) and printing time (1:40-3:17 h) differed significantly between the four frames. Likewise, the fit, wearing comfort, space for additional PPE, and protection varied between the designs. For clinical suitability, a chosen design should allow sufficient space for goggles and N95 respirators as well as maximum coverage of the facial area. Consequently, two datasets are recommended. For the final selection of the ideal dataset to be used for printing, scalability and economic efficiency need to be carefully balanced with an acceptable degree of protection.

Whittle, J. S., et al. (2020). "Respiratory support for adult patients with COVID-19." J Am Coll Emerg Physicians Open **1**(2): 95-101.

The COVID-19 pandemic is creating unique strains on the healthcare system. While only a small percentage of patients require mechanical ventilation and ICU care, the enormous size of the populations affected means that these critical resources may become limited. A number of non-invasive options exist to avert mechanical ventilation and ICU admission. This is a clinical review of these options and their applicability in adult COVID-19 patients. Summary recommendations include: (1) Avoid nebulized therapies. Consider metered dose inhaler alternatives. (2) Provide supplemental oxygen following usual treatment principles for hypoxic respiratory failure. Maintain awareness of the aerosol-generating potential of all devices, including nasal cannulas, simple face masks, and venturi masks. Use non-rebreather masks when possible. Be attentive to aerosol generation and the use of personal protective equipment. (3) High flow nasal oxygen is preferred for patients with higher oxygen support requirements. Non-invasive positive pressure ventilation may be associated with higher risk of nosocomial transmission. If used, measures special precautions should be used reduce aerosol formation. (4) Early intubation/mechanical ventilation may be prudent for patients deemed likely to progress to critical illness, multi-organ failure, or acute respiratory distress syndrome (ARDS).

Whyler, N. C. A., et al. (2020). "Viral genomics to inform infection control response in occupational COVID-19 transmission." Clin Infect Dis.

Healthcare workers are at increased risk of occupational transmission of SARS-CoV-2. We report two instances of healthcare workers contracting SARS-CoV-2 despite no known breach of personal protective equipment. Additional specific equipment cleaning was initiated. Viral genomic sequencing supported this transmission hypothesis and our subsequent response.

Wichmann, D., et al. (2020). "[Intensive care back up for infectious disease disasters]." Med Klin Intensivmed Notfmed **115**(8): 641-648.

BACKGROUND: Outbreaks of infectious diseases pose particular challenges for hospitals and intensive care units. OBJECTIVES: Typical infectiological scenarios and their significance for modern intensive care medicine are presented. MATERIALS AND METHODS: Selected pathogens/infectious diseases that have significantly strained the resources of intensive care units are described. RESULTS: Intensive medical care is necessary in severe cases of many infectious diseases. In the context of epidemics/pandemics, many critically ill patients have to be admitted within a short time. Examples are the 2009 H1N1 influenza pandemic, the 2011 enterohemorrhagic Escherichia coli (EHEC) outbreak in northern Germany, the 2014/2015 Ebola fever outbreak and the 2020 coronavirus disease 19 (COVID-19) pandemic. Multidisciplinary teams, protocol development, adequate staffing, and training are required to achieve optimal treatment outcomes, including prevention of healthcare worker infections. CONCLUSIONS: Pandemics and epidemics are unique challenges for intensive care unit preparedness planning.

Widjaja, F. F., et al. (2020). "Health Issues Among Healthcare Workers During COVID-19 Pandemic: a Psychosomatic Approach." Acta Med Indones **52**(2): 172-176.

Healthcare workers as the front-liner to fight COVID-19 pandemic, need reassurance of their safety. The stress and the demands from the healthcare system can affect their health as well as the morality. Hence, psychosomatic approach should be used to keep their condition in good shape. The combination between biological, psychological, and social approaches may affect their vulnerability from SARS-CoV-2 infection. The biological aspect includes personal protective equipment, nutrition, and resting/sleeping time. The psychological distress is shown to affect the immune system; and mental relaxation and management of work shift may reduce the distress. There should be an effort from the stakeholders to keep the morality of the healthcare workers. The social aspect such as providing place to live, having supportive family members, and avoiding stigmatization also improve their psychological health. In conclusion, psychosomatic approach is important to keep the healthcare workers healthy.

Wilder, J. L., et al. (2021). "Pediatric Trainees as Parents: Perspectives from a Pandemic." Acad Pediatr.

OBJECTIVES: Resident parents are at the intersection of converging challenges during the coronavirus disease 2019 (COVID-19) pandemic, and their perspective has yet to be explored. This qualitative study aimed to identify themes related to the experience of pediatric resident parents during the COVID-19 pandemic. METHODS: We performed semi-structured interviews with pediatric residents who were also parents. Using thematic analysis, transcripts were analyzed iteratively until theoretical sufficiency was achieved. RESULTS: 12 residents were interviewed from 9 pediatric residency programs. Five major themes, representing conflicts or tensions experienced by participants, were defined by the data: (1) Duty as a doctor versus duty as a parent; (2) Balance of work obligations versus childcare challenges; (3) Appreciation of added support versus worry about peers' perceptions of accommodations; (4) Gratitude for the recognition of challenges faced by resident parents versus fear of reversion to the norm; and (5) Protective impact of parenthood versus challenges to mental health. CONCLUSIONS: Both parents and healthcare workers faced innumerable challenges during the COVID-19 pandemic, and this exploration of the experiences of pediatric resident parents provides a unique lens for examining the tensions that this group faced. Our results may allow residency programs to better support their pediatric resident parents during this crisis.

Wilkinson, L. S., et al. (2020). "Preparing healthcare facilities to operate safely and effectively during the COVID-19 pandemic: The missing piece in the puzzle." S Afr Med J **110**(9): 835-836.

The stated objective of the COVID-19 lockdown was to allow time to prepare healthcare facilities. Preparation must include administrative and environmental measures, which when combined with personal protective equipment, minimise the risk of the spread of infection to patients and healthcare workers (HCWs) in facilities, allowing HCWs to safely provide essential services during the pandemic and limit the indirect effects of COVID-19 caused by healthcare disruption. We present our model for facility preparation based on colour-coded zones, social distancing, hand hygiene, rapid triage and separate management of symptomatic patients, and attention to infection transmission prevention between HCWs in communal staff areas. This model specifically addresses the challenges in preparing a facility for COVID-19 in a low-resource setting and in rural areas. In addition, we include links to resources to allow workers in low-resource settings to prepare their facilities adequately.

Willer, B. L., et al. (2020). "The otolaryngologist's and anesthesiologist's collaborative role in a pandemic: A large quaternary pediatric center's experience with COVID-19 preparation and simulation." Int J Pediatr Otorhinolaryngol **136**: 110174.

There has been a rapid global spread of a novel coronavirus, the Severe Acute Respiratory Syndrome Coronavirus 2 (SARS-CoV-2), which originated in Wuhan China in late 2019. A serious threat of nosocomial spread exists and as such, there is a critical necessity for well-planned and rehearsed processes during the care of the COVID-19 positive and suspected patient to minimize transmission and risk to healthcare providers and other patients. Because of the aerosolization inherent in airway management, the pediatric otolaryngologist and anesthesiologist should be intimately familiar with strategies to mitigate the high-risk periods of viral contamination that are posed to the environment and healthcare personnel during tracheal intubation and extubation procedures. Since both the pediatric otolaryngologist and anesthesiologist are directly involved in emergency airway interventions, both specialties impact the safety of caring for COVID-19 patients and are a part of overall hospital pandemic preparedness. We describe our institutional approach to COVID-19 perioperative pandemic planning at a large quaternary pediatric hospital including operating room management and remote airway management. We outline our processes for the safe and effective care of these patients with emphasis on simulation and pathways necessary to protect healthcare workers and other personnel from exposure while still providing safe, effective, and rapid care.

Williams, D. L., et al. (2021). "A randomised crossover study to compare the user seal check and quantitative fit test between two types of duckbill N95 particulate respirator masks: The Halyard Fluidshield® N95 and the BSN Medical ProShield® N-95 particulate respirator masks." Anaesth Intensive Care: 310057x20974022.

N95 particulate respirator masks are currently recommended for all healthcare workers who care for patients with suspected or confirmed coronavirus disease (COVID-19) when performing aerosol-generating procedures. The protection provided by N95 particulate respirator masks is dependent on the filter's efficiency and seal quality. In this prospective randomised crossover study, we conducted the user seal check and the quantitative fit test on two readily available duckbill models of N95 masks, the Halyard Fluidshield® N95 (Halyard, Alpharetta, GA, USA) and the BSN Medical ProShield® N-95 (BSN Medical, Mount Waverley, Victoria) particulate respirator masks. We recruited a total of 96 anaesthetic staff, of whom 26% were of South-East Asian ethnicity. We found that both types of masks provided reasonably high fit test pass rates among our participants and there was no significant difference between the two brands (77% for the Fluidshield and 65% for the ProShield, P = 0.916). Ninety-two percent of the participants could find at least one well-fitted mask among these two types of masks. We also demonstrated that the user seal check had low accuracy and low concordance (kappa coefficient of 0.16 for the Fluidshield and 0.08 for the ProShield) when compared to the quantitative fit test, and hence was not a reliable method to test seal quality.

Williams, J. and C. Williams (2020). "Responsibility for vitamin D supplementation of elderly care home residents in England: falling through the gap between medicine and food." BMJ Nutr Prev Health **3**(2): 256-262.

INTRODUCTION: Daily vitamin D supplements are recommended for elderly care home residents; however, they are rarely given and vitamin D deficiency in care homes is widespread. This study aimed to understand the determinants of current practice and perceived responsibility for the vitamin D status of residents. METHODS: Thirteen semi-structured interviews were conducted with key informants in two areas of Southern England including care home managers, general practitioners (GPs) and public health professionals. Interviews were audio recorded and transcribed verbatim. RESULTS: Inductive thematic analysis identified four themes: framing of vitamin D supplements as medicines; professional and sector boundaries whereby GPs are perceived as responsible for the vitamin D status of residents and care home managers felt unable to administer over-the-counter vitamin tablets; low awareness of national guidance; and ethical and practical issues. This results in vitamin D supplements requiring prescription by medical professionals and few residents receiving them. CONCLUSION: The medical framing of vitamin D supplements in care homes is a practical barrier to implementation of longstanding nutrition guidelines. A paradigm shift is needed so that vitamin D is understood as a protective nutrient as well as a medicine, and a public health as well as a medical responsibility. Vitamin D is important for musculoskeletal health. Possible links with COVID-19 are still being investigated. The pandemic has drawn attention to conditions in care homes and there is an opportunity to revise current guidance on vitamin D supplementation which will have lasting benefit for this vulnerable group.

Winck, J. C. and N. Ambrosino (2020). "COVID-19 pandemic and non invasive respiratory management: Every Goliath needs a David. An evidence based evaluation of problems." Pulmonology **26**(4): 213-220.

BACKGROUND AND AIM: The war against Covid-19 is far from won. This narrative review attempts to describe some problems with the management of Covid-19 induced acute respiratory failure (ARF) by pulmonologists. METHODS: We searched the following databases: MEDLINE, EMBASE, Cochrane Central Register of Controlled Trials and reviewed the references of retrieved articles for additional studies. The search was limited to the terms: Covid-19 AND: acute respiratory distress syndrome (ARDS), SARS, MERS, non invasive ventilation (NIV), high flow nasal cannula (HFNC), pronation (PP), health care workers (HCW). RESULTS: Protection of Health care workers should be paramount, so full Personal Protective Equipment and Negative pressure rooms are warranted. HFNC alone or with PP could be offered for mild cases (PaO2/FiO2 between 200-300); NIV alone or with PP may work in moderate cases (PaO2/FiO2 between 100-200). Rotation and coupled (HFNC/NIV) strategy can be beneficial. A window of opportunity of 1-2h is advised. If PaO2/FIO2 significantly increases, Respiratory Rate decreases with a relatively low Exhaled Tidal Volume, the non-invasive strategy could be working and intubation delayed. CONCLUSION: Although there is a role for non-invasive respiratory therapies in the context of COVID-19 ARF, more research is still needed to define the balance of benefits and risks to patients and HCW. Indirectly, non invasive respiratory therapies may be of particular benefit in reducing the risks to healthcare workers by obviating the need for intubation, a potentially highly infectious procedure.

Wong, E. L., et al. (2021). "Compliance with Standard Precautions and Its Relationship with Views on Infection Control and Prevention Policy among Healthcare Workers during COVID-19 Pandemic." Int J Environ Res Public Health **18**(7).

Background: Standard precautions prevent the spread of infections in healthcare settings. Incompliance with infection control guidelines of healthcare workers (HCWs) may increase their risk of exposure to infectious disease, especially under pandemics. The purpose of this study was to assess the level of compliance with the infection prevention and control practices among HCWs in different healthcare settings and its relationship with their views on workplace infection control measures during the COVID-19 pandemic. Methods: Nurses in Hong Kong were invited to respond to a cross-sectional online survey, in which their views on workplace infection and prevention policy, compliance with standard precautions and self-reported health during pandemics were collected. Results: The respondents were dissatisfied with workplace infection and prevention policy in terms of comprehensiveness (62%), clarity (64%), timeliness (63%), and transparency (60%). For the protective behavior, the respondents did not fully comply with the standard precautions when they were involved in medical care. Their compliance was relatively low when having proper patient handling (54%) and performing invasive procedures (46%). A multivariate analysis model proved that the level of compliance of the standard precautions was positively associated with the satisfaction on infection control and prevention policy among high risk group (0.020; 95% CI: 0.005-0.036), while older respondents had higher level of compliance among the inpatient and outpatient groups (coefficient range: 0.065-0.076). The higher level of compliance was also significantly associated with working in designated team and having chronic condition of the respondents among high-risk and inpatient groups. Conclusions: Standard precautions are the most important elements to reduce cross-transmission among HCWs and patients while the satisfaction on infection control and prevention policy would increase the compliance among the high-risk group. An overall suboptimal compliance and poor views on the infection prevention and control guidelines is a warning signal to healthcare system especially during pandemics.

Wong, J., et al. (2020). "Preparing for a COVID-19 pandemic: a review of operating room outbreak response measures in a large tertiary hospital in Singapore." Can J Anaesth **67**(6): 732-745.

The coronavirus disease 2019 (COVID-19) outbreak has been designated a public health emergency of international concern. To prepare for a pandemic, hospitals need a strategy to manage their space, staff, and supplies so that optimum care is provided to patients. In addition, infection prevention measures need to be implemented to reduce in-hospital transmission. In the operating room, these preparations involve multiple stakeholders and can present a significant challenge. Here, we describe the outbreak response measures of the anesthetic department staffing the largest (1,700-bed) academic tertiary level acute care hospital in Singapore (Singapore General Hospital) and a smaller regional hospital (Sengkang General Hospital). These include engineering controls such as identification and preparation of an isolation operating room, administrative measures such as modification of workflow and processes, introduction of personal protective equipment for staff, and formulation of clinical guidelines for anesthetic management. Simulation was valuable in evaluating the feasibility of new operating room set-ups or workflow. We also discuss how the hierarchy of controls can be used as a framework to plan the necessary measures during each phase of a pandemic, and review the evidence for the measures taken. These containment measures are necessary to optimize the quality of care provided to COVID-19 patients and to reduce the risk of viral transmission to other patients or healthcare workers.

Wong, P. and W. Y. Lim (2020). "Aligning difficult airway guidelines with the anesthetic COVID-19 guidelines to develop a COVID-19 difficult airway strategy: a narrative review." J Anesth **34**(6): 924-943.

The coronavirus disease 2019 (COVID-19) pandemic is caused by a coronavirus that is transmitted primarily via aerosol, droplets or direct contact. This may place anesthetists at higher risk of infection due to their frequent involvement in aerosol-generating airway interventions. Many anesthethetic COVID-19 guidelines have emerged, whose underlying management principles include minimizing aerosol contamination and protecting healthcare workers. These guidelines originate from Australia and New Zealand, Canada, China, India, Italy, Korea, Singapore, the United States and the United Kingdom. Hospitalized COVID-19 patients may require airway interventions, and difficult tracheal intubation secondary to laryngeal edema has been reported. Pre-pandemic difficult airway guidelines include those from Canada, France, Germany, India, Japan, Scandinavia, the United States and the United Kingdom. These difficult airway guidelines require modifications in order to align with the principles of the anesthetic COVID-19 guidelines. In turn, most of the anesthetic COVID-19 guidelines do not, or only briefly, discuss an airway strategy after failed tracheal intubation. Our article identifies and compares pre-pandemic difficult airway guidelines with the recent anesthetic COVID-19 guidelines. We combine the principles from both sets of guidelines and explain the necessary modifications to the airway guidelines, to form a failed tracheal intubation airway strategy in the COVID-19 patient. Valuing, and a greater understanding of, these differences and modifications may lead to greater adherence to the new COVID-19 guidelines.

Wong, P., et al. (2020). "COVID-19 and cardiopulmonary resuscitation: an N95 respirator mask may not be adequate." Br J Anaesth **125**(3): e319-e322.

Wong, S. C., et al. (2020). "Infection control challenges in setting up community isolation and treatment facilities for patients with coronavirus disease 2019 (COVID-19): Implementation of directly observed environmental disinfection." Infect Control Hosp Epidemiol: 1-9.

BACKGROUND: Extensive environmental contamination by severe acute respiratory syndrome coronavirus 2 (SARS-CoV-2) has been reported in hospitals during the coronavirus disease 2019 (COVID-19) pandemic. We report our experience with the practice of directly observed environmental disinfection (DOED) in a community isolation facility (CIF) and a community treatment facility (CTF) in Hong Kong. METHODS: The CIF, with 250 single-room bungalows in a holiday camp, opened on July 24, 2020, to receive step-down patients from hospitals. The CTF, with 500 beds in open cubicles inside a convention hall, was activated on August 1, 2020, to admit newly diagnosed COVID-19 patients from the community. Healthcare workers (HCWs) and cleaning staff received infection control training to reinforce donning and doffing of personal protective equipment and to understand the practice of DOED, in which the cleaning staff observed patient and staff activities and then performed environmental disinfection immediately thereafter. Supervisors also observed cleaning staff to ensure the quality of work. In the CTF, air and environmental samples were collected on days 7, 14, 21, and 28 for SARS-CoV-2 detection by RT-PCR. Patient compliance with mask wearing was also recorded. RESULTS: Of 291 HCWs and 54 cleaning staff who managed 243 patients in the CIF and 674 patients in the CTF from July 24 to August 29, 2020, no one acquired COVID-19. All 24 air samples and 520 environmental samples collected in the patient area of the CTF were negative for SARS-CoV-2. Patient compliance with mask wearing was 100%. CONCLUSION: With appropriate infection control measures, zero environmental contamination and nosocomial transmission of SARS-CoV-2 to HCWs and cleaning staff was achieved.

Wood, R., et al. (2021). "Sharing a household with children and risk of COVID-19: a study of over 300 000 adults living in healthcare worker households in Scotland." Arch Dis Child.

OBJECTIVE: Children are relatively protected from COVID-19, due to a range of potential mechanisms. We investigated if contact with children also affords adults a degree of protection from COVID-19. DESIGN: Cohort study based on linked administrative data. SETTING: Scotland. STUDY POPULATION: All National Health Service Scotland healthcare workers and their household contacts as of March 2020. MAIN EXPOSURE: Number of young children (0-11 years) living in the participant's household. MAIN OUTCOMES: COVID-19 requiring hospitalisation, and any COVID-19 (any positive test for SARS-CoV-2) in adults aged ≥18 years between 1 March and 12 October 2020. RESULTS: 241 266, 41 198, 23 783 and 3850 adults shared a household with 0, 1, 2 and 3 or more young children, respectively. Over the study period, the risk of COVID-19 requiring hospitalisation was reduced progressively with increasing numbers of household children-fully adjusted HR (aHR) 0.93 per child (95% CI 0.79 to 1.10). The risk of any COVID-19 was similarly reduced, with the association being statistically significant (aHR per child 0.93; 95% CI 0.88 to 0.98). After schools reopened to all children in August 2020, no association was seen between exposure to young children and risk of any COVID-19 (aHR per child 1.03; 95% CI 0.92 to 1.14). CONCLUSION: Between March and October 2020, living with young children was associated with an attenuated risk of any COVID-19 and COVID-19 requiring hospitalisation among adults living in healthcare worker households. There was no evidence that living with young children increased adults' risk of COVID-19, including during the period after schools reopened.

Woon, Y. L., et al. (2021). "Serology surveillance of SARS-CoV-2 antibodies among healthcare workers in COVID-19 designated facilities in Malaysia." Lancet Reg Health West Pac **9**: 100123.

BACKGROUND: Asymptomatic severe acute respiratory syndrome coronavirus-2 (SARS-CoV-2) infections are well documented. Healthcare workers (HCW) are at increased risk of infection due to occupational exposure to infected patients. We aim to determine the prevalence of SARS-CoV-2 antibodies among HCW who did not come to medical attention. METHODS: We prospectively recruited 400 HCW from the National Public Health Laboratory and two COVID-19 designated public hospitals in Klang Valley, Malaysia between 13/4/2020 and 12/5/2020. Quota sampling was used to ensure representativeness of HCW involved in direct and indirect patient care. All participants answered a self-administered questionnaire and blood samples were taken to test for SARS-CoV-2 antibodies by surrogate virus neutralization test. FINDINGS: The study population comprised 154 (38.5%) nurses, 103 (25.8%) medical doctors, 47 (11.8%) laboratory technologists and others (23.9%). A majority (68.9%) reported exposure to SARS-CoV-2 in the past month within their respective workplaces. Adherence to personal protection equipment (PPE) guidelines and hand hygiene were good, ranging from 91-100% compliance. None (95% CI: 0, 0.0095) of the participants had SARS-CoV-2 antibodies detected, despite 182 (45.5%) reporting some symptoms one month prior to study recruitment. One hundred and fifteen (29%) of participants claimed to have had contact with known COVID-19 persons outside of their workplace. INTERPRETATION: Zero seroprevalence among HCW suggests a low incidence of undiagnosed COVID-19 infection in our healthcare setting during the first local wave of SARS-CoV-2 infection. The occupational risk of SARS-CoV-2 transmission within healthcare facilities can be prevented by adherence to infection control measures and appropriate use of PPE.

Wu, Y. C., et al. (2020). "The outbreak of COVID-19: An overview." J Chin Med Assoc **83**(3): 217-220.

In late December 2019, a previous unidentified coronavirus, currently named as the 2019 novel coronavirus#, emerged from Wuhan, China, and resulted in a formidable outbreak in many cities in China and expanded globally, including Thailand, Republic of Korea, Japan, United States, Philippines, Viet Nam, and our country (as of 2/6/2020 at least 25 countries). The disease is officially named as Coronavirus Disease-2019 (COVID-19, by WHO on February 11, 2020). It is also named as Severe Pneumonia with Novel Pathogens on January 15, 2019 by the Taiwan CDC, the Ministry of Health and is a notifiable communicable disease of the fifth category. COVID-19 is a potential zoonotic disease with low to moderate (estimated 2%-5%) mortality rate. Person-to-person transmission may occur through droplet or contact transmission and if there is a lack of stringent infection control or if no proper personal protective equipment available, it may jeopardize the first-line healthcare workers. Currently, there is no definite treatment for COVID-19 although some drugs are under investigation. To promptly identify patients and prevent further spreading, physicians should be aware of the travel or contact history of the patient with compatible symptoms.

Wundavalli, L., et al. (2020). "How to rapidly design and operationalise PPE donning and doffing areas for a COVID-19 care facility: quality improvement initiative." BMJ Open Qual **9**(3).

INTRODUCTION: Effective implementation of standard precautions specific to COVID-19 is a challenge for hospitals within the existing constraints of time and resources. AIM: To rapidly design and operationalise personal protective equipment (PPE) donning and doffing areas required for a COVID-19 care facility. METHODS: Literature review was done to identify all issues pertaining to donning and doffing in terms of Donabedian's structure, process and outcome. Training on donning and doffing was given to hospital staff. Donning and doffing mock drills were held. 5S was used as a tool to set up donning and doffing areas. Instances of donning and doffing were observed for protocol deviations and errors. Plan-do-study-act cycles were conducted every alternate day for 4 weeks. The initiative was reported using Standards for QUality Improvement Reporting Excellence (SQUIRE) guidelines. RESULTS: Best practices in donning and doffing were described. Our study recommends a minimum area of 16 m(2) each for donning and doffing rooms. Verbally assisted doffing was found most useful than visual prompts. DISCUSSION: Challenges included sustaining the structure and process of donning and doffing, varied supplies of PPE which altered sequencing of donning and/or doffing, and training non-healthcare workers such as plumbers, electricians and drivers who were required during emergencies in the facility. CONCLUSION: Our study used evidence-based literature and quality improvement (QI) tools to design and operationalise donning and doffing areas with focus on people, task and environment. Our QI will enable healthcare facilities to rapidly prototype donning and doffing areas in a systematic way.

Xiang, B., et al. (2020). "The impact of novel coronavirus SARS-CoV-2 among healthcare workers in hospitals: An aerial overview." Am J Infect Control **48**(8): 915-917.

The ongoing outbreak of COVID-19, caused by the novel coronavirus SARS-CoV-2, places healthcare workers at an increased risk of infection as they are in close contact with patients. In this article, we report an overview of cases of infected healthcare workers in China and Italy during the early periods of the COVID-19 epidemic. China's coronavirus response highlights the importance of implementing effective public health strategies. The authorities worldwide therefore, need to be extremely cautious when they implement stringent protective measures that safeguard healthcare workers in hospitals and counteract the threats created by the pandemic.

Xiang, Y. T., et al. (2020). "An overview of the expert consensus on the mental health treatment and services for major psychiatric disorders during COVID-19 outbreak: China's experiences." Int J Biol Sci **16**(13): 2265-2270.

The 2019 novel coronavirus disease (COVID-19) epidemic in China has presented substantial challenges to traditional forms of mental health service delivery. This review summarizes the expert consensus on the mental health treatment and services for severe psychiatric disorders during the COVID-19 outbreak developed by the Chinese Society of Psychiatry and other academic associations. The Expert Recommendations on Managing Patients with Mental Disorders during a Serious Infectious Disease Outbreak (COVID-19) outline the appropriate measures for psychiatric hospitals or psychiatric units in general hospitals, including the delivery of outpatient, inpatient, and community mental health services. The Expert Recommendations on Internet and Telehealth in Psychiatry during Major Public Health Crises (COVID-19) describe the assessment and treatment issues of internet-based mental health services during the COVID-19 outbreak. The expert consensus recommendations provide guidance for mental health professionals in managing psychiatric services during the COVID-19 outbreak in China. The experiences from China in addressing the challenges in the management of major psychiatric disorders may be useful and relevant to other countries who are combating the COVID-19 pandemic.

Xiang, Y. T., et al. (2020). "The COVID-19 outbreak and psychiatric hospitals in China: managing challenges through mental health service reform." Int J Biol Sci **16**(10): 1741-1744.

Recently, more than 300 Chinese patients with psychiatric disorders were diagnosed with the 2019 novel coronavirus disease (COVID-19). Possible reasons quoted in the report were the lack of caution regarding the COVID-19 outbreak in January and insufficient supplies of protective gear. We outlined major challenges for patients with psychiatric disorders and mental health professionals during the COVID-19 outbreak, and also discussed how to manage these challenges through further mental health service reform in China.

Xiao, X., et al. (2020). "Psychological impact of healthcare workers in China during COVID-19 pneumonia epidemic: A multi-center cross-sectional survey investigation." J Affect Disord **274**: 405-410.

BACKGROUND: Since the outbreak of 2019 new coronavirus (COVID-19) pneumonia, healthcare workers (HCW) have suffered psychological stress. The present study is to examine the prevalence of stress, anxiety and depression of HCW in China during the COVID-19 epidemic, and to determine the risk factors predicting psychological morbidities that can be used as psychological intervention targets. METHODS: A cross-sectional survey was conducted to investigate the psychological levels of HCW in multiple centers in China. The prevalence of stress, anxiety and depression were determined by using Perceived Stress Scale (PSS-14) and Hospital Anxiety / Depression scale (HAD). Psychology related factors were evaluated and correlation between job title and contact history was analyzed. RESULTS: We received 958 of effective responses, 73.6% of which were from Wuhan and 67.2% were female participants. 55.1% of respondents had psychological stress that is higher than that of HCW during SARS. 54.2% and 58% of participants had symptoms of anxiety and depression. Stress levels of HCW were different in job titles and years of work experience. Anxiety and depression levels were different between different gender, job titles, degrees of protective measures and levels of contact history. Gender, intermediate title, protective measures and contact history were the independent risk factors for anxiety. Protective measures and contact history were the independent risk factors for depression. CONCLUSIONS: The COVID-19 epidemic has induced stress levels for HCW, and high percentages of HCW have anxiety and depression. The situation of HCW is worrying and intervention service is urgent.

Xie, X. M., et al. (2021). "Workplace violence and its association with quality of life among mental health professionals in China during the COVID-19 pandemic." J Psychiatr Res **135**: 289-293.

OBJECTIVE: Mental health professionals are often exposed to workplace violence (WPV) in China. This study examined the prevalence of WPV and the associated factors and quality of life (QOL) among frontline mental health professionals during the COVID-19 pandemic. METHODS: This national survey was carried out between March 15 and March 20, 2020. WPV and QOL were assessed with standardized measures. RESULTS: A total of 10,516 participants were included. The prevalence of overall WPV was 18.5% (95% CI: 17.9%-19.3%), while verbal abuse/threats was 15.8% and physical violence was 8.4%. Multiple logistic regression analysis revealed that male gender (OR = 1.42, p < 0.01), higher educational level (OR = 1.40, p < 0.01), working in tertiary hospitals (OR = 1.33, p < 0.01), caring for COVID-19 patients (OR = 3.10, p < 0.01) and having more severe anxiety symptom (OR = 1.21, p < 0.01) were positively associated with WPV. In contrast, working in inpatient departments (OR = 0.74, p < 0.01), having longer work experience (OR = 0.99, p = 0.03), and being a junior nurse (OR = 0.73, p < 0.01) were negatively associated with WPV. After controlling for the covariates, mental health professionals who experienced WPV had a lower overall QOL compared to those without WPV (F((1, 10515)) = 68.28, p < 0.01). CONCLUSION: This study found that WPV was common among mental health professionals in China during the COVID-19 pandemic. Considering the negative impact of WPV on QOL and quality of patient care, appropriate measures to prevent WPV should be developed.

Xiong, Y., et al. (2020). "Clinical and CT characteristics of healthcare workers with COVID-19: A single-centered, retrospective study." Medicine (Baltimore) **99**(30): e21396.

A large number of healthcare workers have been infected with coronavirus disease-2019 (COVID-19). We aimed to investigate their clinical and chest computed tomography (CT) characteristics.The clinical, laboratory test and CT features of 43 medical and hospital staff with confirmed COVID-19 (MP group, 26-70 years old) were retrospectively analyzed, and compared to 43 non-medical related patients (non-MP group, 26-71 years old). Follow-up CT characteristics were analyzed to assess the disease progression in the period of hospitalization.At admission, the main complaints of the MP group, including fever (81.4%), fatigue (48.8%) and cough (41.9%), were similar to the non-MP group. The C-reactive protein, erythrocyte sedimentation rate, and lactate dehydrogenase levels were higher in the non-MP group than the MP group (17.5 ± 22.4 mg/L, 20.2 ± 23.4 mm/H and 219 ± 66U/L, respectively, P < .05). Ground-grass opacities, consolidation, interstitial thickening were common CT features of both groups. The severity of opacities on initial CT were less in the MP group (5.3 ± 3.9 scores) than in the non-MP group (9.1 ± 4.8 scores, P < .05). Before regular treatments, the sum score of the opacities showed weak to moderate correlations with duration, C-reactive protein, erythrocyte sedimentation rate and lactate dehydrogenase levels (R ranged from 0.341-0.651, P < .05). In the study time window, the duration from illness onset to when the most obvious pulmonary opacities were observed, according to CT findings, were similar in the MP group (13.3 ± 6.6 days) and the non-MP group (13.8 ± 5.1 days, P = .69). Mild to moderate anxiety and depression were observed in both groups.Despite greater knowledge of how to protect themselves than the general population, healthcare workers are also susceptible to COVID-19 infection. Occupational exposure is a very important factor. Healthcare workers have a higher vigilance about the infection in the early stage of the disease.

Xu, X., et al. (2020). "[Application and thinking of health standards related to medical care and health information in prevention and control of COVID-19]." Zhonghua Liu Xing Bing Xue Za Zhi **41**(11): 1765-1771.

Objective: To compare the technical elements of health standards for nosocomial infection control, health protection, health information, and health emergency and biosafety in the context of the prevention and control of COVID-19, and provide support for the further optimization of the epidemic prevention and control guidelines. Methods: Above mentioned health standards used in COVID-19 prevention and control were collected for a systematic comparison with "Guidelines for Prevention and Control of COVID-19 in Medical Institutions" (the 1(st) Edition) from the perspective of technical elements. Results: The application scope and technical elements of the current health standards basically meet the needs for the prevention and control of COVID-19 epidemic. Conclusions: The implementation of the current health standards can provide strong technical support for the prevention and control of COVID-19 epidemic. The experience obtained in the epidemic prevention and control can also contribute to the further revision and improvement of the health standards.

Xue, X., et al. (2020). "All Surfaces Are Not Equal in Contact Transmission of SARS-CoV-2." Matter **3**(5): 1433-1441.

The world faces a severe and acute public health emergency due to the ongoing coronavirus disease 2019 (COVID-19) global pandemic caused by the severe acute respiratory syndrome coronavirus 2 (SARS-CoV-2). Healthcare workers are in the front line of the COVID-19 outbreak response and are exposed to the risk of SARS-CoV-2 infection daily. Personal protective equipment (PPE) is their main defense against viral contamination; gloves, visors, face masks, and gown materials are designed to eliminate viral transfer from infected patients. Here, we review research investigating the stability of SARS-CoV-2 and similar viruses on surfaces and highlight opportunities for materials that can actively reduce SARS-CoV-2 surface contamination and associated transmission and improve PPE.

Yamamoto, H., et al. (2020). "A Strategy to Battle Coronavirus Disease 2019 in the Hospital: Identify and Use the Power of "Immune Survivors"." Jma j **3**(4): 303-306.

The outbreak of the severe acute respiratory syndrome coronavirus 2 (SARS-CoV-2) poses serious health and economic problems worldwide. One of the worst scenarios is the collapse of the medical care system due to nosocomial infections. SARS-CoV-2 quickly spreads in closed spaces, crowded areas, and close physical distances, which frequently occur in Japanese medical facilities. Although we are making efforts to avoid such situations, healthcare workers always face the risk of developing a SARS-CoV-2 infection in the workplace because of proximity. Thus, we need to battle SARS-CoV-2 using a unique strategy. We propose a novel strategy to eliminate SARS-CoV-2 infections: measurement of antibodies against SARS-CoV-2 and using the power of "immune survivors." We agree with using standard precautions and early isolation of patients with coronavirus disease 2019 (COVID-19) to block the spread of SARS-CoV-2 infection. However, we face difficulties carrying out these fundamental missions. Now, we focus on "immune survivors." If healthcare workers acquired the neutralizing antibody against SARS-CoV-2, they are considered "immune survivors" with a low risk of reinfection with SARS-CoV-2. These "immune survivors" can contribute to the care of patients with COVID-19 on the front line. Also, these "immune survivors" can function as an envelope by surrounding COVID-19 patients. As a result, "immune survivors" can eliminate the spread of SARS-CoV-2 in medical facilities as well as in society. We understand that the concept of "immune survivors" needs further discussion. No information is available on how long or the titer of neutralizing antibody required for protection from infection. We have just started to measure antibody levels against SARS-CoV-2 in healthcare workers in our hospital. This project will provide further information in the battle against the SARS-CoV-2 infection. (Clinical trial registration number: UMIN 000039997).

Yan, Y., et al. (2020). "Consensus of Chinese experts on protection of skin and mucous membrane barrier for health-care workers fighting against coronavirus disease 2019." Dermatol Ther **33**(4): e13310.

Health professions preventing and controlling Coronavirus Disease 2019 are prone to skin and mucous membrane injury, which may cause acute and chronic dermatitis, secondary infection and aggravation of underlying skin diseases. This is a consensus of Chinese experts on protective measures and advice on hand-cleaning- and medical-glove-related hand protection, mask- and goggles-related face protection, UV-related protection, eye protection, nasal and oral mucosa protection, outer ear, and hair protection. It is necessary to strictly follow standards of wearing protective equipment and specification of sterilizing and cleaning. Insufficient and excessive protection will have adverse effects on the skin and mucous membrane barrier. At the same time, using moisturizing products is highly recommended to achieve better protection.

Yang, H., et al. (2020). "Expert panel consensus statement on the applications and precaution strategies of bronchoscopy in patients with COVID-19." Endosc Ultrasound **9**(4): 211-219.

Severe acute respiratory syndrome coronavirus 2 (SARS-CoV-2) is a novel coronavirus with higher transmissibility compared with SARS coronavirus (SARS-CoV) and Middle East respiratory distress syndrome coronavirus. Coronavirus disease 2019 (COVID-19) caused by SARS-CoV-2 is an unprecedented global crisis that has not been experienced, which is still disrupting health systems, economies, and societies around the world by the rapid spread. Bronchoscopy plays an important role in diagnosis and therapy of pulmonary diseases, especially in patients with severe pulmonary infection, however, application of bronchoscopy in patients suspected or confirmed SARS-CoV-2 infection is extremely limited for the potential airborne transmission from aerosol generated during the procedure. This consensus statement was completed by expert panel of Interventional & Minimally Invasive Respiratory Committee of China Medical Education Association, and the issues were summarized as seven key topics to define the indications of bronchoscopy and matters needing attentions on the bronchoscopy procedures in patients with COVID-19, as well as the protective precaution strategies to avoid nosocomial SARS-CoV-2 infection.

Yang, M., et al. (2020). "Prevention and Control of COVID-19 Infection in a Chinese Mental Health Center." Front Med (Lausanne) **7**: 356.

Faced with the rapid spread of the novel coronavirus disease (COVID-19), a global public health threat, psychiatric hospitals are under huge pressure to prevent and control nosocomial infection. The current research analyzed the COVID-19 infection control practices in a regional mental health center in China and addressed how this type of medical institutions could enhance their ability to prevent and control hospital transmission of major respiratory diseases and general management of nosocomial infection risks. Firstly, hospital-related risks of COVID-19 were analyzed, and targeted prevention and control measures were then established. Pre- and post-intervention theoretical knowledge of nosocomial infection control, hand hygiene compliance and accuracy, use of personal protective equipment, and disinfection and sterilization effectiveness were evaluated and compared. All the indexes displayed significant improvements following the implementation of the prevention and control measures. Up to the submission of this paper, the mental health center had obtained no suspected or confirmed case of COVID-19 infection due to hospital transmission. The findings provide empirical evidence for the effectiveness of the COVID-19 preventive strategies and have important implications for integrated and characterized infection control in mental health centers during a major epidemic. The establishment of the transitional isolation ward and air fumigation using traditional Chinese medicine for patients and staff are preventive measures worthy of further discussion and dissemination.

Yanko, R., et al. (2020). "Inhalation Sedation During the COVID-19 Outbreak: An Expert Opinion." Isr Med Assoc J **22**(10): 599-601.

Severe acute respiratory syndrome coronavirus-2 (SARS-CoV-2), the pathogen that causes coronavirus disease-2019 (COVID-19), is thought to be transmitted via droplets and aerosols, and was detected in saliva of infected individuals. These droplets from the upper airway may infect the inhalation sedation mask and tubing. The authors determined the adequate measures needed to prevent the transmission of COVID-19 by nitrous-oxide (N2O) system during inhalation sedation in dentistry and provided evidence on mask and tubing sterilization. Additional measures to protect patients and healthcare workers from COVID-19 that may be transmitted by the inhalation sedation system are discussed. The authors recommend minimal use of a N2O system during inhalation sedation in dentistry. In case of need, the practitioners should have more than one scavenger kit and nasal masks for each N2O/O2 mixer. Biologic barriers should be mounted between the scavenger's tubing and the central evacuation system. Strict cleansing and sterilization should be performed for all parts of the N2O system. The use a disposable scavenger system and nasal mask should be considered as a viable option.

Yao, W., et al. (2020). "Emergency tracheal intubation in 202 patients with COVID-19 in Wuhan, China: lessons learnt and international expert recommendations." Br J Anaesth **125**(1): e28-e37.

Tracheal intubation in coronavirus disease 2019 (COVID-19) patients creates a risk to physiologically compromised patients and to attending healthcare providers. Clinical information on airway management and expert recommendations in these patients are urgently needed. By analysing a two-centre retrospective observational case series from Wuhan, China, a panel of international airway management experts discussed the results and formulated consensus recommendations for the management of tracheal intubation in COVID-19 patients. Of 202 COVID-19 patients undergoing emergency tracheal intubation, most were males (n=136; 67.3%) and aged 65 yr or more (n=128; 63.4%). Most patients (n=152; 75.2%) were hypoxaemic (Sao(2) <90%) before intubation. Personal protective equipment was worn by all intubating healthcare workers. Rapid sequence induction (RSI) or modified RSI was used with an intubation success rate of 89.1% on the first attempt and 100% overall. Hypoxaemia (Sao(2) <90%) was common during intubation (n=148; 73.3%). Hypotension (arterial pressure <90/60 mm Hg) occurred in 36 (17.8%) patients during and 45 (22.3%) after intubation with cardiac arrest in four (2.0%). Pneumothorax occurred in 12 (5.9%) patients and death within 24 h in 21 (10.4%). Up to 14 days post-procedure, there was no evidence of cross infection in the anaesthesiologists who intubated the COVID-19 patients. Based on clinical information and expert recommendation, we propose detailed planning, strategy, and methods for tracheal intubation in COVID-19 patients.

Yasmin, F., et al. (2021). "Exploring the impact of the COVID-19 pandemic on provision of cardiology services: a scoping review." Rev Cardiovasc Med **22**(1): 83-95.

The coronavirus disease-19 (COVID-19) pandemic has forced hospitals to prioritize COVID-19 patients, restrict resources, and cancel all non-urgent elective cardiac procedures. Clinical visits have only been facilitated for emergency purposes. Fewer patients have been admitted to the hospital for both ST-segment elevation myocardial infarctions (STEMI) and non-ST segment elevation myocardial infarctions (NSTEMI) and a profound decrease in heart failure services has been reported. A similar reduction in the patient presentation is seen for ischemic heart disease, decompensated heart failure, and endocarditis. Cardiovascular services, including catheterization, primary percutaneous coronary intervention (PPCI), cardiac investigations such as electrocardiograms (ECGs), exercise tolerance test (ETT), dobutamine stress test, computed tomography (CT) angiography, transesophageal echocardiography (TOE) have been reported to have declined and performed on a priority basis. The long-term implications of this decline have been discussed with major concerns of severe cardiac complications and vulnerabilities in cardiac patients. The pandemic has also had psychological impacts on patients causing them to avoid seeking medical help. This review discusses the effects of the COVID-19 pandemic on the provision of various cardiology services and aims to provide strategies to restore cardiovascular services including structural changes in the hospital to make up for the reduced staff personnel, the use of personal protective equipment in healthcare workers, and provides alternatives for high-risk cardiac imaging, cardiac interventions, and procedures. Implementation of the triage system, risk assessment scores, and telemedicine services in patients and their adaptation to the cardiovascular department have been discussed.

Yasukawa, K. and T. Minami (2020). "Point-of-Care Lung Ultrasound Findings in Patients with COVID-19 Pneumonia." Am J Trop Med Hyg **102**(6): 1198-1202.

Patients with novel coronavirus disease (COVID-19) typically present with bilateral multilobar ground-glass opacification with a peripheral distribution. The utility of point-of-care ultrasound has been suggested, but detailed descriptions of lung ultrasound findings are not available. We evaluated lung ultrasound findings in 10 patients admitted to the internal medicine ward with COVID-19. All of the patients had characteristic glass rockets with or without the Birolleau variant (white lung). Thick irregular pleural lines and confluent B lines were also present in all of the patients. Five of the 10 patients had small subpleural consolidations. Point-of-care lung ultrasound has multiple advantages, including lack of radiation exposure and repeatability. Also, lung ultrasound has been shown to be more sensitive than a chest radiograph in detecting alveolar-interstitial syndrome. The utilization of lung ultrasound may also reduce exposure of healthcare workers to severe acute respiratory syndrome-coronavirus-2 and may mitigate the shortage of personal protective equipment. Further studies are needed to evaluate the utility of lung ultrasound in the diagnosis and management of COVID-19.

Ye, Y., et al. (2021). "Point-of-care training program on COVID-19 infection prevention and control for pediatric healthcare workers: a multicenter, cross-sectional questionnaire survey in Shanghai, China." Transl Pediatr **10**(1): 44-53.

BACKGROUND: To evaluate the effectiveness of training on knowledge and practice of infection prevention and control (IPC) among pediatric health care workers (HCW) in Shanghai, China, in the context of COVID-19 pandemic. METHODS: An online training program was designed by the Shanghai Pediatric Clinical Quality Control Center (SPQCC) during the early phase of COVID-19 pandemic on disease knowledge and practice of IPC. Training took place in the 81 partner hospitals affiliated with SPQCC. A multicenter, cross-sectional questionnaire survey was designed with a 25-item self-administered questionnaire to evaluate the knowledge gained from the training. Stratified-random sampling was used to select HCW according to three professionals (i.e., pediatricians, nurses and administrators) within each partner hospital. Awareness and knowledge of COVID-19 and its related infection control and practice were assessed by comparing survey results between different types of hospitals, professionals and professional ranks. A higher survey score meant that the respondent was more prepared and knowledgeable about COVID-19 and its infection control measures. RESULTS: Completed questionnaires were returned from 1,062 subjects (385 pediatricians, 410 nurses, and 267 administrators), giving a response rate of 96.5%. Overall, awareness of clinical information related to COVID-19, importance of personal hygiene and isolation policy was high among the respondents. No statistical difference of scores on knowledge of COVID-19, IPC and relevant practice between the tertiary and peripheral hospitals. Among all respondents, middle-ranked health care personnel were most knowledgeable and achieved the highest score. CONCLUSIONS: Majority of pediatric HCW showed good recognition and practice in infection protection and control measures. The online training was able to achieve its aim to enhance knowledge and awareness and could have contributed to the zero infection rate among HCW caring for confirmed COVID-19 cases in Shanghai.

Yee, R., et al. (2020). "Saliva is a promising alternative specimen for the detection of SARS-CoV-2 in children and adults." medRxiv.

Testing efforts for SARS-CoV-2 have been burdened by the scarcity of testing materials and personal protective equipment for healthcare workers. The simple and painless process of saliva collection allows for widespread testing, but enthusiasm is hampered by variable performance compared to nasopharyngeal swab (NPS) samples. We prospectively collected paired NPS and saliva samples from a total of 300 unique adult and pediatric patients. SARS-CoV-2 RNA was detected in 32.2% (97/300) of the individuals using the TaqPath COVID-19 Combo Kit (Thermo Fisher). Performance of saliva and NPS were compared against the total number of positives regardless of specimen type. The overall concordance for saliva and NPS was 91.0% (273/300) and 94.7% (284/300), respectively. The positive percent agreement (PPA) for saliva and NPS was 81.4% (79/97) and 89.7% (87/97), respectively. Saliva detected 10 positive cases that were negative by NPS. In symptomatic and asymptomatic pediatric patients not previously diagnosed with COVID-19, the performances of saliva and NPS were comparable (PPA: 82.4% vs 85.3%). The overall PPA for adults were 83.3% and 90.7% for saliva and NPS, respectively, with saliva detecting 4 cases less than NPS. However, saliva performance in symptomatic adults was identical to NPS (PPA of 93.8%). With lower cost and self-collection capabilities, saliva can be an appropriate alternative sample choice to NPS for detection of SARS-CoV-2 in children and adults. SUMMARY: Saliva is an acceptable alternative specimen compared to nasopharyngeal swabs for detection of SARS-CoV-2. Specifically, saliva demonstrated comparable performance to nasopharyngeal swabs in symptomatic and asymptomatic pediatric patients and in symptomatic adults.

Yek, J. L. J., et al. (2020). "Perioperative considerations for COVID-19 patients: lessons learned from the pandemic -a case series." Korean J Anesthesiol **73**(6): 557-561.

BACKGROUND: As the coronavirus disease 2019 (COVID-19) pandemic spreads globally, hospitals are rushing to adapt their facilities, which were not designed to deal with infections adequately. Here, we present the management of a suspected COVID-19 patient. CASE: A 66-year-old man with a recent travel history, infective symptoms, and chest X-ray was presented to our hospital. Considering his septic condition, we decided to perform an emergency surgery. The patient was given supplemental oxygen through a face mask and transported to an operating theatre on a plastic-covered trolley. An experienced anesthetist performed rapid sequence intubation using a video laryngoscope. Due to the initial presentation of respiratory distress, the patient remained intubated after surgery to avoid re-intubation. Precautions against droplet, contact, and airborne infection were instituted. CONCLUSIONS: Our objective was to facilitate surgical management of patients with known or suspected COVID-19 while minimizing the risk of nosocomial transmission to healthcare workers and other patients.

Yi, J., et al. (2020). "A Key Factor for Psychosomatic Burden of Frontline Medical Staff: Occupational Pressure During the COVID-19 Pandemic in China." Front Psychiatry **11**: 590101.

The global outbreak of COVID-19 has severely affected the entire population, especially healthcare staff on the frontline, who bear heavy psychosomatic burdens. A cross-sectional study was conducted with 723 participants in China from April 26 to May 9, 2020. We evaluated the psychosomatic status, including depression, anxiety, quality of life, somatic symptoms, stress, sleep disturbances, and posttraumatic stress symptoms in different exposure groups. We explored the risk factors that affect psychosomatic burdens and analyzed the relationship between psychosomatic problems and medical occupations. We found that the psychosomatic burdens of medical staff were significantly greater than those of non-medical staff (p < 0.01) and were positively related with the number of COVID-19 patients they came in contact with. Occupational pressure was a key factor for healthcare staff's psychosomatic problems (p < 0.01 for quality of life, somatic symptoms, anxiety, depression, stress; p = 0.012 for sleep disturbances), and it had a strong canonical correlation (p < 0.01). Workload and time allocation (WTA), one of the subdimensional indicators of occupational pressure, was strongly correlated with psychosomatic indicators. We suggest that rationalization of WTA is a desirable approach for anti-epidemic medical employees to alleviate psychosomatic burdens. Public health interventions should be undertaken to reduce the occupational pressure on this special population, which is critical for mitigation. This study presents results regarding the psychosomatic burdens of the healthcare workforce related to occupational pressure and provides multilevel data with groups of different exposure risks for policymakers to protect medical personnel. These findings draw attention to the working environments of healthcare workers and provide applicable results for clinical practice.

Yin, X., et al. (2021). "Comparative efficacy of respiratory personal protective equipment against viral respiratory infectious diseases in healthcare workers: a network meta-analysis." Public Health **190**: 82-88.

OBJECTIVE: With the epidemic of coronavirus disease 2019 (COVID-19), the healthcare workers (HCWs) require proper respiratory personal protective equipment (rPPE) against viral respiratory infectious diseases (VRIDs). It is necessary to evaluate which type of mask and manner of wearing is the best suitable rPPE for preventing the VRID. STUDY DESIGN: A Bayesian network meta-analysis was performed to comprehensively analyze the protective efficacy of various rPPE. METHODS: This network meta-analysis protocol was registered in an international prospective register of systematic reviews (CRD42020179489). Electronic databases were searched for cluster randomized control trials (RCTs) of comparing the effectiveness of rPPE and wearing manner in preventing HCWs from VRID. The primary outcome was the incidence of laboratory-confirmed viral respiratory infection reported as an odds ratio (OR) with the associated 95% credibility interval (CrI). The secondary outcome was the incidence of clinical respiratory illness (CRI) reported as an OR with the associated 95% CrI. Surface under the cumulative ranking curve analysis (SUCRA) provided a ranking of each rPPE according to the primary outcome and the secondary outcome as data supplement. RESULTS: Six studies encompassing 12,265 HCWs were included. In terms of the incidence of laboratory-confirmed viral respiratory infection, the continuous wearing of N95 respirators (network OR, 0.48; 95% CrI: 0.27 to 0.86; SUCRA score, 85.4) showed more effective than the control group. However, in terms of reducing the incidence of CRI, there was no rPPE showing superior protective effectiveness. CONCLUSIONS: There are significant differences in preventive efficacy among current rPPE. Our result suggests that continuous wearing of N95 respirators on the whole shift can serve as the best preventive rPPE for HCWs from the VRID.

Yoshida, S., et al. (2020). "Real-world clinical data of endoscopy-based cancer detection during the emergency declaration for COVID-19 in Japan." World J Gastrointest Endosc **12**(10): 401-403.

The impact of the coronavirus disease 2019 (COVID-19) pandemic is widespread throughout the world, causing serious damage to healthcare systems. Therefore, we examined the significance of endoscopy based on the recommendation of Asian-Pacific Society for Digestive Endoscopy and Japan Gastroenterological Endoscopy Society during the COVID-19 pandemic by evaluating the details of gastrointestinal endoscopy performed during the declaration of emergency in Japan. We have continued performing gastrointestinal endoscopy at an outpatient clinic that specialized in endoscopic medical care in Tokyo, Japan. During the emergency declaration period, 544 patients underwent gastrointestinal endoscopy. As a control, we investigated 1327 patients who underwent gastrointestinal endoscopy during the same period in 2019. Although the total number of endoscopies during the emergency declaration was halved, the advanced cancer detection rate during the emergency declaration was significantly higher than that in 2019 (P = 0.04). Additionally, no COVID-19 infection was observed in healthcare workers, staff, or patients during this period. It is possible that an outpatient endoscopy units can contribute to the detection of advanced cancer, while the hospital in charge for patients with COVID-19 infection could not perform endoscopy during the declaration of emergency.

Youssef, F. A., et al. (2021). "Protected code blue: using in situ simulation to develop a protected code blue and modify staff training protocol-experience in a large community teaching hospital during the COVID-19 pandemic." BMJ Open Qual **10**(1).

The surge in clinical demand, shortage in personal protective equipment and high-exposure risk for healthcare workers during the COVID-19 pandemic has challenged hospital common practices and forced a reassessment of care delivery models. Code blue teams are highly specialised units that partake in life-saving situations that can jeopardise the safety of team members. There is a paucity of guidance in regards to proper infection control measures to protect the responders.This study describes a methodical approach to assessing vulnerabilities to transmission of SARS-CoV-2 within existing code blue practices, modalities to limit the number of code blue team responders and modifications to the protocol at a large community teaching hospital. The effort undertaken faced challenges due to the nature of the pandemic and the increased demand on healthcare workers. Quality improvement methods facilitated our protocol design and implementation. To this date, there has been no identified COVID-19 disease in any protected code blue (PCB) team members. We recommend that similar practices be considered and adopted widely and practised periodically.

Yu, G. Y., et al. (2020). "[Several suggestions of operation for colorectal cancer under the outbreak of corona virus disease 2019 in China]." Zhonghua Wei Chang Wai Ke Za Zhi **23**(3): 208-211.

Pneumonia caused by 2019-nCoV infection has been reported in Wuhan since December 2019, and spread rapidly across the country. The radical operation of colorectal cancer is semi-elective operation. Patients with colorectal cancer should receive operation as soon as possible after elective operation is resumed in each hospital. 2019-nCoV virus can be transmitted by asymptomatic infectors, and it has been confirmed to be transmitted by droplets and contact. However, fecal-oral transmission and aerosol transmission have not been excluded. Based on our experience with laparoscopic colorectal operation, we propose some surgery strategies for colorectal cancer patients under the corona virus disease 2019(COVID-19) situation: the screening process should be strictly carried out before surgery to reduce the risk of nosocomial infection in the later stage; laparoscopic-assisted surgery is recommended for radical surgery for patients with colorectal cancer; strict aerosol management must be made during the operation; natural orifice specimen extraction surgery and transanal total mesorectal excision are should be performed prudently; scientific and reasonable prophylactic stoma should be done; personnel protection in surgical ward and operation room must be strengthened.

Yu, N., et al. (2021). "SARS-CoV-2 not found in pressure injury exudates from COVID-19 patients." J Cosmet Dermatol **20**(2): 372-380.

BACKGROUND: The emergence of COVID-19 has become a global health emergency. The transmissibility of the disease is of great interest to healthcare workers and scientists alike. The primary route of transmission is via respiratory droplets, but viral RNA has also been found in feces and body fluids such as urine, serum, and semen. So far, there has been no report on whether SARS-CoV-2 is present in the exudates of cutaneous lesions. This study was designed to investigate whether SARS-CoV-2 can be found in the pressure injury exudates in patients with severe COVID-19 infections. METHODS: 46 critically ill COVID-19 patients who were admitted to the ICU of the Sino-French New City Branch of Tongji Hospital in Wuhan between February 4 and April 12 developed pressure injuries. 22 patients with pressure injuries had wound exudates. Wound and pharyngeal swabs of the 22 patients were collected and RT-PCRs were conducted to detect SARS-CoV-2 viral RNA. RESULTS: At the time of pressure injury, 5 patients still tested positive by pharyngeal swabs, the rest of the 17 patients tested negative. However, none of the wound exudate swabs from the participants tested positive for SARS-CoV-2 by RT-PCR. CONCLUSION: Our study suggests that it is rather unlikely that COVID-19 can be transmitted via pressure injury exudates, but we still recommend standardized personal protective equipment, face shield and an additional pair of gloves when treating pressure injuries.

Yuan, X. N., et al. (2020). "[Detection and evaluation of SARS-CoV-2 nucleic acid contamination in corona virus disease 19 ward surroundings and the surface of medical staff's protective equipment]." Beijing Da Xue Xue Bao Yi Xue Ban **52**(5): 803-808.

OBJECTIVE: To determine the environmental contamination degree of severe acute respiratory syndrome coronavirus 2 (SARS-CoV-2) in corona virus disease 2019 (COVID-19) wards, to offer gui-dance for the infection control and to improve safety practices for medical staff, by sampling and detecting SARS-CoV-2 nucleic acid from the air of hospital wards, the high-frequency contact surfaces in the contaminated area and the surfaces of medical staff's protective equipment in a COVID-19 designated hospital in Wuhan, China. METHODS: From March 11 to March 19, 2020, we collected air samples from the clean area, the buffer room and the contaminated area respectively in the COVID-19 wards using a portable bioaerosol concentrator WA-15. And sterile premoistened swabs were used to sample the high-frequency contacted surfaces in the contaminated area and the surfaces of medical staff's protective equipment including outermost gloves, tracheotomy operator's positive pressure respiratory protective hood and isolation clothing. The SARS-CoV-2 nucleic acid of the samples were detected by real-time fluorescence quantitative PCR. During the isolation medical observation period, those medical staff who worked in the COVID-19 wards were detected for SARS-CoV-2 nucleic acid with oropharyngeal swabs, IgM and IgG antibody in the sera, and chest CT scans to confirm the infection status of COVID-19. RESULTS: No SARS-CoV-2 nucleic acid was detected in the tested samples, including the 90 air samples from the COVID-19 wards including clean area, buffer room and contaminated area, the 38 high-frequency contact surfaces samples of the contaminated area and 16 surface samples of medical staff's protective equipment including outermost gloves and isolation clothing. Moreover, detection of SARS-CoV-2 nucleic acid by oropharyngeal swabs and IgM, IgG antibodies in the sera of all the health-care workers who participated in the treatment for COVID-19 were all negative. Besides, no chest CT scan images of medical staff exhibited COVID-19 lung presentations. CONCLUSION: Good ventilation conditions, strict disinfection of environmental facilities in hospital wards, guidance for correct habits in patients, and strict hand hygiene during medical staff are important to reduce the formation of viral aerosols, cut down the aerosol load, and avoid cross-infection in isolation wards. In the face of infectious diseases that were not fully mastered but ma-naged as class A, it is safe for medical personnel to be equipped at a high level.

Publisher: Abstract available from the publisher.

chi

Yuno, A., et al. (2020). "Successful management of a patient with active Cushing‍'‍s disease complicated with coronavirus disease 2019 (COVID-19) pneumonia." Endocr J.

We provide the details of the successful management of a patient with active Cushing's disease complicated with coronavirus disease 2019 (COVID-19) pneumonia. The patient was a 27-year-old Japanese female healthcare worker who was scheduled to undergo pituitary surgery for Cushing's disease. She had been in close contact with an undiagnosed patient infected with COVID-19 and then developed COVID-19 pneumonia. Despite a lack of known risk factors associated with severe COVID-19 infection, the patient's dyspnea worsened and her respiratory condition deteriorated, as indicated by the need for 7 L/min oxygen supply by mask to maintain her oxygen saturation at >90%. Medical treatment was initiated to control hypercortisolism by the 'block and replace' regimen using steroidogenesis inhibitors and hydrocortisone. The COVID-19 pneumonia improved with multi-modal treatment including antiviral therapy. One month later, after a negative severe acute respiratory syndrome coronavirus-2 (SARS-CoV-2) test result and with appropriate protection against virus transmission to medical staff in the operating room and daily medical care nurses, trans-sphenoidal surgery was performed by our highly experienced pituitary surgeon. One month after the surgery, the patient's basal ACTH and cortisol levels and urinary free cortisol were all under the detection limit. Surgical remission was expected. Since hypercortisolism due to active Cushing's disease may worsen a COVID-19 infection, multi-disciplinary management that includes appropriate and prompt treatment strategies is mandatory in such cases.

Zabarsky, T. F., et al. (2021). "What are the sources of exposure in healthcare personnel with coronavirus disease 2019 infection?" Am J Infect Control **49**(3): 392-395.

In our facility, 25% of personnel with coronavirus disease 2019 (COVID-19) had a higher-risk exposure to an infected patient or co-worker and 14% reported a higher-risk exposure in the community. All higher-risk exposures to infected patients occurred on non-COVID-19 units, often when there was a delay in diagnosis because COVID-19 was not initially suspected. Higher-risk exposures to co-workers with COVID-19 often involved lapses in compliance with masking in nonpatient care areas such as nursing stations and staff work or break rooms.

Zaçe, D., et al. (2021). "Interventions to address mental health issues in healthcare workers during infectious disease outbreaks: A systematic review." J Psychiatr Res **136**: 319-333.

Considering the importance of evidence on interventions to tackle mental health problems in healthcare workers (HCWs) during pandemics, we conducted a systematic review, aiming to identify and summarize the implemented interventions to deal with mental health issues of HCWs during infectious disease outbreaks and report their effectiveness. Web of Science, PubMed, Cochrane, Scopus, CINAHL and PsycInfo electronic databases were searched until October 2nd, 2020. Primary-data articles, describing any implemented interventions and their effectiveness were considered pertinent. Studies were screened according to the inclusion/exclusion criteria and subsequently data extraction was performed. Twenty-four articles, referring to SARS, Ebola, Influenza AH1N1 and COVID-19 were included. Interventions addressing mental health issues in HCWs during pandemics/epidemics were grouped into four categories: 1) informational support (training, guidelines, prevention programs), 2) instrumental support (personal protective equipment, protection protocols); 3) organizational support (manpower allocation, working hours, re-organization of facilities/structures, provision of rest areas); 4) emotional and psychological support (psychoeducation and training, mental health support team, peer-support and counselling, therapy, digital platforms and tele-support). These results might be helpful for researchers, stakeholders, and policymakers to develop evidence-based sustainable interventions and guidelines, aiming to prevent or reduce the immediate and long-term effect of pandemics on mental health status of HCWs.

Zaga, C. J., et al. (2020). "Speech-Language Pathology Guidance for Tracheostomy During the COVID-19 Pandemic: An International Multidisciplinary Perspective." Am J Speech Lang Pathol **29**(3): 1320-1334.

Purpose As the COVID-19 pandemic has unfolded, there has been growing recognition of risks to frontline health care workers. When caring for patients with tracheostomy, speech-language pathologists have significant exposure to mucosal surfaces, secretions, and aerosols that may harbor the SARS-CoV-2 virus. This tutorial provides guidance on practices for safely performing patient evaluation and procedures, thereby reducing risk of infection. Method Data were collated through review of literature, guidelines, and consensus statements relating to COVID-19 and similar high-consequent infections, with a focus on mitigating risk of transmission to health care workers. Particular emphasis was placed on speech-language pathologists, nurses, and other allied health professionals. A multinational interdisciplinary team then analyzed findings, arriving at recommendations through consensus via electronic communications and video conference. Results Reports of transmission of infection to health care workers in the current COVID-19 pandemic and previous outbreaks substantiate the need for safe practices. Many procedures routinely performed by speech-language pathologists have a significant risk of infection due to aerosol generation. COVID-19 testing can inform level of protective equipment, and meticulous hygiene can stem spread of nosocomial infection. Modifications to standard clinical practice in tracheostomy are often required. Personal protective equipment, including either powered air-purifying respirator or N95 mask, gloves, goggles, and gown, are needed when performing aerosol-generating procedures in patients with known or suspected COVID-19 infection. Conclusions Speech-language pathologists are often called on to assist in the care of patients with tracheostomy and known or suspected COVID-19 infection. Appropriate care of these patients is predicated on maintaining the health and safety of the health care team. Careful adherence to best practices can significantly reduce risk of infectious transmission.

Zaheer, R., et al. (2020). "Association of Personal Protective Equipment with De Novo Headaches in Frontline Healthcare Workers during COVID-19 Pandemic: A Cross-Sectional Study." Eur J Dent **14**(S 01): S79-s85.

OBJECTIVES:  This study aimed to determine the association of personal protective equipment (PPE) usage with new-onset headaches and exacerbation of pre-existing headache disorders among healthcare workers at the frontlines during coronavirus disease 2019 (COVID-19) pandemic. MATERIALS AND METHODS:  A descriptive cross-sectional survey was conducted across Pakistan in June-July 2020. The study was approved by Ethical Committee, Armed Forces Institute of Dentistry, Rawalpindi (IRB form no.905/Trg-ABP 1K2). A qualitative questionnaire was developed and was shared via different social networks. The questionnaire was closed when 241 responses were received. STATISTICAL ANALYSIS:  Descriptive analysis was performed on demographic data. Chi-squared analysis was performed between demographic data and PPE-usage patterns among participants with or without de novo headaches. Univariable and multivariable logistic regression models were used to compare variables with the development of new-onset headaches. Chi-squared test was also performed between demographic data and other factors that may be causing new-onset headaches. A p-value < 0.05 was considered significant. RESULTS:  A total of 241 healthcare workers participated, of which 68 participants (28.2%) reported de novo headaches since the start of the pandemic. Incidence of pre-existing headaches (odds ratio [OR] = 1.91; 95% confidence interval [CI]: 0.99-0.37; p = 0.049) was associated with new-onset headaches. Post hoc multivariable logistic regression analysis stated that incidence of pre-existing headaches (OR = 1.88; 95% CI: 0.94-3.78; p = 0.75) and age (OR = 2.21; 95% CI: 0.47-10.33; p = 0.36) was independently associated with new-onset PPE-induced headaches but was not statistically significant. Chi-squared analysis showed a statistically significant relationship between other factors (sleep deprivation, emotional stress, etc.) and department of activity, gender, and occupation (p < 0.05). CONCLUSION:  Healthcare workers with previous history of pre-existing headaches were found to be more susceptible to PPE-induced headaches during COVID-19 pandemic. However, age and the department where the healthcare workers performed may also be risk factors.

Zeng, P., et al. (2020). "Strategic management of pediatric intensive care unit in a tertiary children's hospital in southwest China during the COVID-19 pandemic." Transl Pediatr **9**(6): 849-862.

Since early December 2019, patients with unknown pneumonia have been found in Wuhan City, Hubei Province, China. The pathogen in these cases was quickly identified as a new type of coronavirus. The World Health Organization named it 2019 novel coronavirus (2019-nCoV), and the disease caused by the pathogen was called 2019 Coronavirus Disease (COVID-19), which was characterized by higher pathogenicity, transmission of human to human. So it has rapidly spread to more than 190 countries all over the world. With the sudden outbreak of COVID-19, preventing the spread of COVID-19 is the primary problem. Despite fewer children than adults have been affected by the COVID-19, Pediatric Intensive Care Unit (PICU), as a ward for critically ill patients, is also confronted with high risk of 2019-nCoV infection. It is necessary for PICU managers to thoroughly carry out scientific and effective department management and carefully execute of infection control measures to prevent the transmission. According to recommendations for the COVID-19 prevention and control, the relevant guidelines and the authors' work experience, this paper proposes and optimizes the strategic plan for the management of COVID-19 outbreak in PICU, and emphasizes that department managers should conduct comprehensive risk assessments, manage the pediatric patients and healthcare workers meticulously, strengthen the implementation of infection control measures, and use risk management and process control to effectively manage the department as well as to protect the safety of both the patients and the staff.

Zhang, B., et al. (2020). "2019 novel coronavirus disease epidemic: skin protection for healthcare workers must not be ignored." J Eur Acad Dermatol Venereol **34**(9): e434-e435.

Since first reported in 2019, pneumonia associated with 2019 novel coronavirus disease (COVID‐19) has rapidly developed into an outbreak across the world.(1) Number of the patients of all age groups has increased significantly.(2) In order to curb the spread of the epidemic, thousands of health care workers (HCWs) have joined the front line of the fight against this highly contagious disease.(3) When taking care of patients with COVID‐19 pneumonia, HCWs must first protect themselves by performing adequate hand hygiene and using protective equipment including medical mask, goggles/face shield, gown and gloves.(4) However, the wearing of these personal protective equipment (PPE) on a daily basis and the frequent use of hand disinfectants often cause skin problems which could reduce their enthusiasm for overloaded work and make them anxious at all stages of the pandemic.

Zhang, J., et al. (2020). "The relationship between resilience, anxiety and depression among patients with mild symptoms of COVID-19 in China: A cross-sectional study." J Clin Nurs **29**(21-22): 4020-4029.

AIMS AND OBJECTIVES: To explore the role of resilience in anxiety and depression and to clarify their relationships among patients with mild symptoms of coronavirus disease 2019 (COVID-19) in Wuhan, China. BACKGROUND: The outbreak of COVID-19 has negatively affected some individuals, but resilience plays a decisive role in the response of individuals under pressure and can help them deal with pressure more effectively. DESIGN: The cross-sectional descriptive correlational survey was reported in line with the STROBE guidelines. SUBJECT AND SETTING: In total, 296 patients from FangCang Hospital in Wuhan, Hubei, China, with mild symptoms of COVID-19 were recruited. METHODS: Participants were recruited through convenience sampling. The data collected included their demographic information, the Connor-Davidson Resilience Scale and Hospital Anxiety and Depression Scale. RESULTS: A small number of patients in this study had above threshold anxiety (subthreshold anxiety and major anxiety) and depression (subthreshold depression and major depression). The mean total resilience score of the participants was slightly below the normal level of ordinary Chinese adults. Resilience was inversely associated with and was a protective factor for both anxiety and depression in our samples. Risk factors for anxiety include being female and having colleagues with COVID-19, while a risk factor for depression was having family members with COVID-19. CONCLUSIONS: This study shows that after taking the general demographics into consideration, higher levels of resilience were associated with lower anxiety and depression among mild COVID-19 patients in Wuhan, China. RELEVANCE TO CLINICAL PRACTICE: Health professionals, especially clinical nurses, need to be aware of the psychological status of COVID-19 patients and promote resilience to improve their mental health.

Zhang, J. Y., et al. (2021). "How to Best Protect People With Diabetes From the Impact of SARS-CoV-2: Report of the International COVID-19 and Diabetes Summit." J Diabetes Sci Technol **15**(2): 478-514.

The coronavirus disease 2019 (COVID-19) pandemic caused by the severe acute respiratory syndrome coronavirus 2 (SARS-CoV-2) virus has rapidly involved the entire world and exposed the pressing need for collaboration between public health and other stakeholders from the clinical, scientific, regulatory, pharmaceutical, and medical device and technology communities. To discuss how to best protect people with diabetes from serious outcomes from COVID-19, Diabetes Technology Society, in collaboration with Sansum Diabetes Research Institute, hosted the "International COVID-19 and Diabetes Virtual Summit" on August 26-27, 2020. This unique, unprecedented real-time conference brought together physicians, scientists, government officials, regulatory experts, industry representatives, and people with diabetes from six continents to review and analyze relationships between COVID-19 and diabetes. Over 800 attendees logged in. The summit consisted of five sessions: (I) Keynotes, (II) Preparedness, (III) Response, (IV) Recovery, and (V) Surveillance; eight parts: (A) Background, (B) Resilience, (C) Outpatient Care, (D) Inpatient Care, (E) Resources, (F) High-Risk Groups, (G) Regulation, and (H) The Future; and 24 sections: (1) Historic Pandemics and Impact on Society, (2) Pathophysiology/Risk Factors for COVID-19, (3) Social Determinants of COVID-19, (4) Preparing for the Future, (5) Medications and Vaccines, (6) Psychology of Patients and Caregivers, (7) Outpatient Treatment of Diabetes Mellitus and Non-Pharmacologic Intervention, (8) Technology and Telehealth for Diabetes Outpatients, (9) Technology for Inpatients, (10) Management of Diabetes Inpatients with COVID-19, (11) Ethics, (12) Accuracy of Diagnostic Tests, (13) Children, (14) Pregnancy, (15) Economics of Care for COVID-19, (16) Role of Industry, (17) Protection of Healthcare Workers, (18) People with Diabetes, (19) International Responses to COVID-19, (20) Government Policy, (21) Regulation of Tests and Treatments, (22) Digital Health Technology, (23) Big Data Statistics, and 24) Patient Surveillance and Privacy. The two keynote speeches were entitled (1) COVID-19 and Diabetes-Meeting the Challenge and (2) Knowledge Gaps and Research Opportunities for Diabetes and COVID-19. While there was an emphasis on diabetes and its interactions with COVID-19, the panelists also discussed the COVID-19 pandemic in general. The meeting generated many novel ideas for collaboration between experts in medicine, science, government, and industry to develop new technologies and disease treatment paradigms to fight this global pandemic.

Zhang, K., et al. (2020). "Routine saliva testing for the identification of silent COVID-19 infections in healthcare workers." medRxiv.

Objective: Current COVID-19 guidelines recommend symptom-based screening and regular nasopharyngeal (NP) testing for healthcare personnel in high-risk settings. We sought to estimate case detection percentages with various routine NP and saliva testing frequencies. Design: Simulation modelling study. Methods: We constructed a sensitivity function based on the average infectiousness profile of symptomatic COVID-19 cases to determine the probability of being identified at the time of testing. This function was fitted to reported data on the percent positivity of symptomatic COVID-19 patients using NP testing. We then simulated a routine testing program with different NP and saliva testing frequencies to determine case detection percentages during the infectious period, as well as the pre-symptomatic stage. Results: Routine bi-weekly NP testing, once every two weeks, identified an average of 90.7% (SD: 0.18) of cases during the infectious period and 19.7% (SD: 0.98) during the pre-symptomatic stage. With a weekly NP testing frequency, the corresponding case detection percentages were 95.9% (SD: 0.18) and 32.9% (SD: 1.23), respectively. A 5-day saliva testing schedule had a similar case detection percentage as weekly NP testing during the infectious period, but identified about 10% more cases (mean: 42.5%; SD: 1.10) during the pre-symptomatic stage. Conclusion: Our findings highlight the utility of routine non-invasive saliva testing for frontline healthcare workers to protect vulnerable patient populations. A 5-day saliva testing schedule should be considered to help identify silent infections and prevent outbreaks in nursing homes and healthcare facilities.

Zhang, K., et al. (2021). "Routine saliva testing for the identification of silent coronavirus disease 2019 (COVID-19) in healthcare workers." Infect Control Hosp Epidemiol: 1-5.

OBJECTIVE: Current COVID-19 guidelines recommend symptom-based screening and regular nasopharyngeal (NP) testing for healthcare personnel in high-risk settings. We sought to estimate case detection percentages with various routine NP and saliva testing frequencies. DESIGN: Simulation modeling study. METHODS: We constructed a sensitivity function based on the average infectiousness profile of symptomatic coronavirus disease 2019 (COVID-19) cases to determine the probability of being identified at the time of testing. This function was fitted to reported data on the percent positivity of symptomatic COVID-19 patients using NP testing. We then simulated a routine testing program with different NP and saliva testing frequencies to determine case detection percentages during the infectious period, as well as the presymptomatic stage. RESULTS: Routine biweekly NP testing, once every 2 weeks, identified an average of 90.7% (SD, 0.18) of cases during the infectious period and 19.7% (SD, 0.98) during the presymptomatic stage. With a weekly NP testing frequency, the corresponding case detection percentages were 95.9% (SD, 0.18) and 32.9% (SD, 1.23), respectively. A 5-day saliva testing schedule had a similar case detection percentage as weekly NP testing during the infectious period, but identified ~10% more cases (mean, 42.5%; SD, 1.10) during the presymptomatic stage. CONCLUSION: Our findings highlight the utility of routine noninvasive saliva testing for frontline healthcare workers to protect vulnerable patient populations. A 5-day saliva testing schedule should be considered to help identify silent infections and prevent outbreaks in nursing homes and healthcare facilities.

Zhang, L., et al. (2021). "Relationship Between Acute Stress Responses and Quality of Life in Chinese Health Care Workers During the COVID-19 Outbreak." Front Psychol **12**: 599136.

This study aimed to determine the relationship between acute stress and quality of life and explore their influencing factors on health care workers. A descriptive cross-sectional study was conducted, and a sample of 525 health care workers was recruited from 15 hospitals through a convenient sampling method. Participants completed an online self-report questionnaire to assess their acute stress and quality of life. Descriptive and multiple linear regression statistics were used for this analysis. The results regarding acute stress responses varied significantly among the differences in marital status, physical activity, work status, perceived risk of contracting COVID-19, and the expected duration of the pandemic. Moreover, a younger age, lack of physical activity, being a front-line medical staff, and higher acute stress scores indicated a worse quality of life. Healthcare workers' acute stress was negatively correlated with their quality of life. Therefore, the authorities should pay special attention to health care workers' mental health and provide them with timely protection during the pandemic.

Zhang, L., et al. (2021). "Retrospective study on the effectiveness of a prevention strategy in a dental hospital during the COVID-19 pandemic." Clin Oral Investig: 1-8.

OBJECTIVES: To evaluate the effectiveness of a prevention strategy against the spread of SARS-CoV-2 infection among dental hospital staff over a 3-month period. MATERIALS AND METHODS: The effectiveness of the prevention strategy, which adopted healthcare staff protective measures, including patient triage and correct usage of personal preventive equipment, was evaluated by SARS-CoV-2 detection and serological testing. Patients who visited the Affiliated Stomatology Hospital, Zhejiang University School of Medicine, Hangzhou, China, between January 31 and March 1 (lockdown period) and March 2 and April 27 (reopening period) in 2020 and in the same period in 2019 were included in the study. Patients' diagnosis, age, gender, and several undergoing aerosol-generating procedures during the study period were collected from the hospital's database. Corresponding data were compared year-on-year (2020-on-2019). A total of 757 hospital staff underwent SARS-CoV-2 detection and serological testing on April 28-29, 2020. RESULTS: During the lockdown and reopening period in 2020, the number of hospital visits was dramatically decreased to about 6% and 58%, respectively, compared with the same period in 2019. However, emergency visits were increased 16- and 6.4-fold. A total of at least 6654 patients (13.58%) underwent aerosol-generating procedures during the study period in the year 2020. All hospital staff were negative according to SARS-CoV-2 detection and serological testing (IgG, IgM) data. CONCLUSION: During the study period in 2020, the total number of hospital visits dramatically reduced but emergency visits significantly increased. The prevention strategy implemented successfully prevented SARS-CoV-2 infection spread among healthcare workers in a dental hospital. CLINICAL RELEVANCE: The prevention strategy indicated patient triage and how to adopt preventive measures for controlling SARS-CoV-2 spread among healthcare workers. These data can be used as a reference for other sectors suffering from the COVID-19 pandemic.

Zhang, L. N., et al. (2020). "[Recommendations for the treatment of severe coronavirus disease 2019 based on critical care ultrasound]." Zhonghua Nei Ke Za Zhi **59**(9): 677-688.

Severe patients with coronaviras disease 2019 (COVID-19) are characterized by persistent lung damage, causing respiratory failure, secondary circulatory changes and multiple organ dysfunction after virus invasion. Because of its dynamic, real-time, non-invasive, repeatable and other advantages, critical ultrasonography can be widely used in the diagnosis, assessment and guidance of treatment for severe patients. Based on the recommendations of critical care experts from all over the country who fight against the epidemic in Wuhan, this article summarizes the guidelines for the treatment of COVID-19 based on critical ultrasonography, hoping to provide help for the treatment of severe patients. The recommendations mainly cover the following aspects: (1) lung ultrasound in patients with COVID-19 is mainly manifested by thickened and irregular pleural lines, different types of B-lines, shred signs, and other consolidation like dynamic air bronchogram; (2) Echocardiography may show right heart dysfunction, diffuse cardiac function enhancement, stress cardiomyopathy, diffuse cardiac depression and other multiple abnormalities; (3) Critical ultrasonography helps with initiating early treatment in the suspect patient, screening confirmed patients after intensive care unit admission, early assessment of sudden critical events, rapid grading assessment and treatment based on it; (4) Critical ultrasonography helps to quickly screen for the etiology of respiratory failure in patients with COVID-19, make oxygen therapeutic strategy, guide the implementation of lung protective ventilation, graded management and precise off-ventilator; (5) Critical ultrasonography is helpful for assessing the circulatory status of patients with COVID-19, finding chronic cardiopulmonary diseases and guiding extracorporeal membrane oxygenation management; (6) Critical ultrasonography contributes to the management of organs besides based on cardiopulmonary oxygen transport; (7) Critical ultrasonography can help to improve the success of operation; (8) Critical ultrasonography can help to improve the safety and quality of nursing; (9) When performing critical ultrasonography for patients with COVID-19, it needs to implement three-level protection standard, pay attention to disinfect the machine and strictly obey the rules from nosocomial infection. (10) Telemedicine and artificial intelligence centered on critical ultrasonography may help to improve the efficiency of treatment for the patients with COVID-19. In the face of the global spread of the epidemic, all we can do is to share experience, build a defense line, We hope this recommendations can help COVID-19 patients therapy.

Zhang, M., et al. (2020). "Masks or N95 Respirators During COVID-19 Pandemic-Which One Should I Wear?" J Oral Maxillofac Surg **78**(12): 2114-2127.

PURPOSE: Coronavirus Disease 2019 (COVID-19) has caused suffering and death around the world. Careful selection of facial protection is paramount for preventing virus spread among healthcare workers and preserving mask and N95 respirator supplies. METHODS: This paper is a comprehensive review of literature written in English and available on Pubmed comparing the risk of viral respiratory infections when wearing masks and N95 respirators. Current international oral and maxillofacial surgery guidelines for mask and N95 respirator use, patient COVID-19 disease status, aerosol producing procedures were also collected and incorporated into a workflow for selecting appropriate facial protection for oral and maxillofacial surgery procedures during the current pandemic. RESULTS: Most studies suggest N95 respirators and masks are equally protective against respiratory viruses. Some evidence favors N95 respirators, which are preferred for high-risk procedures when aerosol production is likely or when the COVID-19 status of a patient is positive or unknown. N95 respirators may also be used for multiple patients or reused depending on the type of procedure and condition of the respirator after each patient encounter. CONCLUSION: N95 respirators are preferred over masks against viral respiratory pathogens, especially during aerosol-generating procedures or when a patient's COVID-19 status is positive or unknown.

Zhang, M., et al. (2020). "Strategy of using personal protective equipment during aerosol generating medical procedures with COVID-19." J Clin Anesth **66**: 109911.

• Compared the effectiveness and cost-effectiveness between masks and respirators for the COVID-19 transmission. • Discussed the strategy to avoid severe nosocomial infection through aerosol-generating medical procedures. • Suggestions for healthcare workers to choose proper personal protective equipment.

Zhang, M., et al. (2020). "Knowledge, attitude, and practice regarding COVID-19 among healthcare workers in Henan, China." J Hosp Infect **105**(2): 183-187.

The study analysed healthcare workers' (HCWs) knowledge, practices, and attitudes regarding coronavirus disease 2019 (COVID-19). A cross-sectional survey was conducted from February 4(th) to February 8(th), 2020, involving a total of 1357 HCWs across 10 hospitals in Henan, China. Of those surveyed, 89% of HCWs had sufficient knowledge of COVID-19, more than 85% feared self-infection with the virus, and 89.7% followed correct practices regarding COVID-19. In addition to knowledge level, some risk factors including work experience and job category influenced HCWs' attitudes and practice concerning COVID-19. Measures must be taken to protect HCWs from risks linked to job category, work experience, working hours, educational attainment, and frontline HCWs.

Zhang, P. C., et al. (2020). "Optimization of community-led 3D printing for the production of protective face shields." 3D Print Med **6**(1): 35.

BACKGROUND: As the healthcare system faced an acute shortage of personal protective equipment (PPE) during the COVID-19 pandemic, the use of 3D printing technologies became an innovative method of increasing production capacity to meet this acute need. Due to the emergence of a large number of 3D printed face shield designs and community-led PPE printing initiatives, this case study examines the methods and design best optimized for community printers who may not have the resources or experience to conduct such a thorough analysis. CASE PRESENTATION: We present the optimization of the production of 3D printed face shields by community 3D printers, as part of an initiative aimed at producing PPE for healthcare workers. The face shield frames were manufactured using the 3DVerkstan design and were coupled with an acetate sheet to assemble a complete face shield. Rigorous quality assurance and decontamination protocols ensured community-printed PPE was satisfactory for healthcare use. CONCLUSION: Additive manufacturing is a promising method of producing adequate face shields for frontline health workers because of its versatility and quick up-start time. The optimization of stacking and sanitization protocols allowed 3D printing to feasibly supplement formal public health responses in the face of a global pandemic.

Zhang, S., et al. (2020). "Gastrointestinal endoscopy infection control strategy during COVID-19 pandemic: Experience from a tertiary medical center in China." Dig Endosc.

OBJECTIVES: Coronavirus disease 2019 (COVID-19) has spread globally and become a pandemic. The severe acute respiratory syndrome coronavirus 2 (SARS-CoV-2) not only infects the gastrointestinal (GI) tract and causes GI symptoms, but also increases nosocomial transmission risk during endoscopic procedures for aerosol generation. We hereby share our infection control strategies aiming to minimize COVID-19 transmission in the endoscopy center. METHODS: We established our infection control strategies based on the guidance of Chinese Society of Digestive Endoscopy and inputs from hospital infection control experts: admission control through the procedure and patient triage, environmental control to reduce possible virus exposure, proper usage of personal protective equipment (PPE), and scope disinfection and room decontamination. All endoscopic procedures accomplished during COVID-19 outbreak and progress of stepwise resumption of elective endoscopy procedures were retrospectively reviewed. RESULTS: Only urgent or semi-urgent procedures were performed during COVID-19 outbreak. After no local new-onset COVID-19 case in Beijing for four weeks, we reopened the endoscopy center for elective procedures and monitored the outbreak continuously while maintaining a sustainable endoscopy service. CONCLUSIONS: It is imperative that all endoscopy centers should establish standard infection control strategies in order to fight COVID-19 pandemic based on national guidance and academic society guidelines and tailor them to individual resources. These measures and setup can also be reserved for future pandemics.

Zhang, S. X., et al. (2020). "At the height of the storm: Healthcare staff's health conditions and job satisfaction and their associated predictors during the epidemic peak of COVID-19." Brain Behav Immun **87**: 144-146.

This study reports the physical health, mental health, anxiety, depression, distress, and job satisfaction of healthcare staff in Iran when the country faced its highest number of total active COVID-19 cases. In a sample of 304 healthcare staff (doctors, nurses, radiologists, technicians, etc.), we found a sizable portion reached the cutoff levels of disorders in anxiety (28.0%), depression (30.6%), and distress (20.1%). Age, gender, education, access to PPE (personal protective equipment), healthcare institutions (public vs. private), and individual status of COVID-19 infection each predicted some but not all the outcome variables of SF-12, PHQ-4, K6, and job satisfaction. The healthcare workers varied greatly in their access to PPE and in their status of COVID-19 infection: negative (69.7%), unsure (28.0%), and positive (2.3%). The predictors were also different from those identified in previous studies of healthcare staff during the COVID-19 crisis in China. This study helps to identify the healthcare staff in need to enable more targeted help as healthcare staff in many countries are facing peaks in their COVID-19 cases.

Zhang, W., et al. (2021). "Mechanism and prevention of facial pressure injuries: A novel emergent strategy supported by a multicenter controlled study in frontline healthcare professionals fighting COVID-19." Wound Repair Regen **29**(1): 45-52.

Numerous healthcare professionals fighting COVID-19 worldwide are suffering from the protective respirators related facial pressure injuries. This study explored the mechanism and prevention of such injuries and devised a novel emergent strategy, which was supported by a multicenter self-controlled study in 1161 frontline healthcare professionals. In this study, according to the anatomy of the face and the characteristics of facial pressure injuries, a respirator liner was designed using a polyurethane foam to redistribute the pressure across the face. A preclinical crossover trial was performed on eight participants to evaluate its efficacy. The strategy was then widely applied among 11  100 healthcare workers in seven frontline hospitals, and 1161 of them were sampled for a questionnaire investigation. The preclinical crossover trial showed that the novel strategy was very effective in preventing facial pressure injuries. The questionnaire investigation showed that pain score, wearing disturbance, and the incidence of pressure injury in the healthcare professionals were significantly correlated with wearing time (all ρ = 0.986). The new strategy significantly reduced the incidence of pressure injury from 84.7% to 11.1%, pain score IQR from 5 (2) to 1 (2), and wearing disturbance rate from 91.6% to 6.3%, and the results analyzed according to individual hospitals or different wearing time showed similar trends (all P < .0005). The protective respirators related facial pressure injuries can be effectively mitigated with this emergent strategy, which has also been applied in some European hospitals and can be popularized to help more healthcare professionals who are combating COVID-19 on the frontlines.

Zhang, X., et al. (2020). "The evidence of SARS-CoV-2 infection on ocular surface." Ocul Surf **18**(3): 360-362.

This is a cross-sectional study of patients who received a COVID-19 diagnosis between December 30, 2019 and February 7, 2020 at Tongji Hospital. A total of 102 patients (48 Male [47%] and 54 Female [53%]) with clinical symptoms, Rt, and chest Computed Tomography (CT) abnormalities were identified with a clinical diagnosis of COVID-19. Patients had a mean [SD] gestational age of 57.63 [14.90] years. Of a total of 102 patients identified, 72 patients (36 men [50%] and 36 women [50%]; mean [SD] age, 58.68 [14.81] years) were confirmed to have COVID-19 by laboratory diagnosis with a SARS-CoV-2 RT-PCR assay. Only two patients (2.78%) with conjunctivitis were identified from 72 patients with a laboratory confirmed COVID-19. Of those two patients, SARS-CoV-2 RNA fragments were found in ocular discharges by SARS-CoV-2 RT-PCR in only one patient. Our findings suspect the incidence of SARS-CoV-2 infection through the ocular surface is extremely low, while the nosocomial infection of SARS-CoV-2 through the eyes after occupational exposure is a potential route. To lower the SARS-CoV-2 nosocomial infection, all health care professionals should wear protective goggles. The inefficient diagnostic method and the sampling time lag may contribute to the lower positive rate of conjunctival swab samples of SARS-CoV-2.

Zhang, X., et al. (2020). "Safe and effective management of tracheostomy in COVID-19 patients." Head Neck **42**(7): 1374-1381.

BACKGROUND: An increasing number of COVID-19 patients worldwide will probably need tracheostomy in an emergency or at the recovering stage of COVID-19. We explored the safe and effective management of tracheostomy in COVID-19 patients, to benefit patients and protect health care workers at the same time. METHODS: We retrospectively analyzed 11 hospitalized COVID-19 patients undergoing tracheostomy. Clinical features of patients, ventilator withdrawal after tracheostomy, surgical complications, and nosocomial infection of the health care workers associated with the tracheostomy were analyzed. RESULTS: The tracheostomy of all the 11 cases (100%) was performed successfully, including percutaneous tracheostomy of 6 cases (54.5%) and conventional open tracheostomy of 5 cases (45.5%). No severe postoperative complications occurred, and no health care workers associated with the tracheostomy are confirmed to be infected by SARS-CoV-2. CONCLUSION: Comprehensive evaluation before tracheostomy, optimized procedures during tracheostomy, and special care after tracheostomy can make the tracheostomy safe and beneficial in COVID-19 patients.

Zhang, X., et al. (2021). "Psychological and occupational impact on healthcare workers and its associated factors during the COVID-19 outbreak in China." Int Arch Occup Environ Health: 1-13.

PURPOSE: China was affected by an outbreak of coronavirus disease 2019 (COVID-19) in 2019-2020. Research data are needed to develop evidence-driven strategies to reduce the adverse psychological and occupational impacts on healthcare workers (HCWs). METHODS: From March 1, 2020, to March 8, 2020, 946 HCWs in China completed a survey consist of sociodemographic data, precautionary measures against COVID-19, and concerns about COVID-19. Self-administered questionnaire were collected to assess psychological and occupational adverse outcomes of HCWs. Multivariable logistic regression analysis was performed to identify factors associated with the outcomes. RESULTS: A total of 55.0%, 56.0% and 48.3% of the HCWs experienced burnout, psychological distress and posttraumatic stress, respectively. A total of seven factors were independently associated with burnout: good health status (OR 0.51, 95% CI 0.36-0.71), fear of contagion (OR 1.31, 95% CI 1.003-1.79), avoiding contact with children (OR 1.40, 95% CI 1.03-1.91), enough staff support at the workplace (OR 0.59, 95% CI 0.38-0.92), having to work overtime (OR 1.37, 95% CI 1.03-1.83), maladaptive coping (OR 3.28, 95% CI 2.42-4.45) and adaptive coping (OR 0.47, 95% CI 0.35-0.62). A total of 11 factors were independently associated with high psychological distress: having one child (OR 0.54, 95% CI 0.38-0.77), good health status (OR 0.57, 95% CI 0.39-0.83), alcohol abuse (OR 1.51, 95% CI 1.02-2.25), thinking the epidemic would continue for quite a long time (OR 1.59, 95% CI 1.08-2.34), wearing extra-work clothes (OR 1.51, 95% CI 1.06-2.15),effective protective equipment (OR 0.45, 95% CI 0.22-0.90), enough staff support at the workplace (OR 0.55, 95% CI 0.34-0.89), unable to take care of families (OR 1.99, 95% CI 1.42-2.78), economic losses (OR 1.62, 95% CI 1.14-2.31), maladaptive coping (OR 6.88, 95% CI 4.75-9.97),and adaptive coping (OR 0.29, 95% CI 0.21-0.41). These factors were independently associated with posttraumatic stress: living with the elderly (OR 1.46, 95% CI 1.04-2.05), alcohol abuse (OR 1.41, 95% CI 1.002-1.98), working at a 3A hospital(OR 0.66, 95% CI 0.49-0.88), acquaintances confirmed COVID-19 (OR 2.14, 95% CI 1.20-3.84), fear of contagion (OR 1.87, 95% CI 1.40-2.50), believing they would survive if infected (OR 0.63, 95% CI 0.46-0.86), self-disinfected after arriving home (OR 1.43, 95% CI 1.01-2.02), interpersonal isolation (OR 1.65, 95% CI 1.21-2.26), unable to take care of families (OR 1.41, 95% CI 1.05-1.88) and maladaptive coping (OR 3.09, 95% CI 2.32-4.11). CONCLUSION: The variance in adverse outcomes was explained by the effect of various factors, which will help policymakers better prepare for subsequent potential outbreaks of COVID-19.

Zhang, Y. S. D., et al. (2021). "Public Health Messages About Face Masks Early in the COVID-19 Pandemic: Perceptions of and Impacts on Canadians." J Community Health: 1-10.

Early in the COVID-19 pandemic, Canadians faced much ambiguity in the public health messages around face mask use. As public health messaging plays a pivotal role in the provision of directives during a health crisis, this study examines Canadians' opinions on the early messaging they received regarding personal protection, especially around mask use, with the goal of identifying potential improvements to strengthen future health messaging. Nine online focus group interviews with 47 Canadians were conducted. These natural conversations focused on personal protective equipment (PPE) choices, mask-relevant public health information sources, and advice to Canadian authorities to improve public health messaging on mask use. Responses were imported into NVivo for thematic analysis. Four meta-themes of relevance were identified. Despite demonstrating trust in scientific evidence and public health authorities, the inconsistencies in public health messaging fostered confusion, and induced mistrust toward health professionals. Further, several information deficits were identified pertaining to the scientific efficacy, safe use, and disposal of masks. Rooted in loyalty to healthcare workers, these Canadians eschewed using medical grade masks during PPE shortages to ensure a sufficient supply for medical workers. The findings stress that consistency in public health messages should be prioritized, with necessary changes clearly justified and explained. More information should reach the public on the scientific benefits and proper use of masks. Public health recommendations should be evidence-based, simple, transparent, and realistic in the current circumstances to guide Canadians to make more informed personal protection choices in the rapidly evolving pandemic.

Zhao, C., et al. (2020). "Otolaryngology during COVID-19: Preventive care and precautionary measures." Am J Otolaryngol **41**(4): 102508.

Since the outbreak of novel coronavirus disease (COVID-19) in December 2019, it has spread to various regions and countries, forming a global pandemic. Reducing nosocomial infection is a new issue and challenge for all healthcare systems. Otolaryngology is a high-risk specialty as it close contact with upper respiratory tract mucous, secretions, droplets and aerosols during procedures and surgery. Therefore, infection prevention and control measures for this specialty are essential. Literatures on the epidemiology, clinical characteristics and infection control measures of COVID-19 were reviewed, practical knowledge from first-line otolaryngologists in China, the United States, and Brazil were reviewed and collated. It was recommended that otolaryngology professionals should improve screening in suspected patients with relevant nasal and pharyngeal symptoms and signs, suspend non-emergency consultations and examinations in clinics, and rearrange the working procedures in operating rooms. The guidelines of personal protective equipment for swab sampling, endoscopy and surgery were listed. Indications for tracheotomy during the pandemic should be carefully considered to avoid unnecessary airway opening and aerosol-generation; precautions during surgery to reduce the risk of exposure and infection were illustrated. This review aimed to provide recommendations for otolaryngologists to enhance personal protection against COVID-19 and reduce the risk of nosocomial infection.

Zhao, Y., et al. (2020). "COVID19: A Systematic Approach to Early Identification and Healthcare Worker Protection." Front Public Health **8**: 205.

The COVID-19 outbreak spread rapidly throughout the globe, with worldwide infections and deaths continuing to increase dramatically. To control disease spread and protect healthcare workers, accurate information is necessary. We searched PubMed and Google Scholar for studies published from December 2019 to March 31, 2020 with the terms "COVID-19," "2019-nCoV," "SARS-CoV-2," or "Novel Coronavirus Pneumonia." The main symptoms of COVID-19 are fever (83-98.6%), cough (59.4-82%), and fatigue (38.1-69.6%). However, only 43.8% of patients have fever early in the disease course, despite still being infectious. These patients may present to clinics lacking proper precautions, leading to nosocomial transmission, and infection of workers. Potential COVID-19 cases must be identified early to initiate proper triage and distinguish them quickly from similar infections. Early identification, accurate triage, and standardized personal protection protocols can reduce the risk of cross infection. Containing disease spread will require protecting healthcare workers.

Zhao, Y., et al. (2020). "Personal protective equipment protecting healthcare workers in the Chinese epicentre of COVID-19." Clin Microbiol Infect **26**(12): 1716-1718.

Zhao, Y. J., et al. (2021). "The prevalence of psychiatric comorbidities during the SARS and COVID-19 epidemics: a systematic review and meta-analysis of observational studies." J Affect Disord **287**: 145-157.

The coronavirus disease 2019 (COVID-19) and Severe Acute Respiratory Syndrome (SARS) are associated with various psychiatric comorbidities. This is a systematic review and meta-analysis comparing the prevalence of psychiatric comorbidities in all subpopulations during the SARS and COVID-19 epidemics. A systematic literature search was conducted in major international (PubMed, EMBASE, Web of Science, PsycINFO) and Chinese (China National Knowledge Internet [CNKI] and Wanfang) databases to identify studies reporting prevalence of psychiatric comorbidities in all subpopulations during the SARS and COVID-19 epidemics. Data analyses were conducted using the Comprehensive Meta-Analysis Version 2.0 (CMA V2.0). Eighty-two studies involving 96,100 participants were included. The overall prevalence of depressive symptoms (depression hereinafter), anxiety symptoms (anxiety hereinafter), stress, distress, insomnia symptoms, post-traumatic stress symptoms (PTSS) and poor mental health during the COVID-19 epidemic were 23.9% (95% CI: 18.4%-30.3%), 23.4% (95% CI: 19.9%-27.3%), 14.2% (95% CI: 8.4%-22.9%), 16.0% (95% CI: 8.4%-28.5%), 26.5% (95% CI: 19.1%-35.5%), 24.9% (95% CI: 11.0%-46.8%), and 19.9% (95% CI: 11.7%-31.9%), respectively. Prevalence of poor mental health was higher in general populations than in health professionals (29.0% vs. 11.6%; Q=10.99, p=0.001). The prevalence of depression, anxiety, PTSS and poor mental health were similar between SARS and COVID-19 epidemics (all p values>0.05). Psychiatric comorbidities were common in different subpopulations during both the SARS and COVID-19 epidemics. Considering the negative impact of psychiatric comorbidities on health and wellbeing, timely screening and appropriate interventions for psychiatric comorbidities should be conducted for subpopulations affected by such serious epidemics.

Zheng, C., et al. (2020). "Characteristics and transmission dynamics of COVID-19 in healthcare workers at a London teaching hospital." J Hosp Infect **106**(2): 325-329.

BACKGROUND: Healthcare worker (HCW)-associated coronavirus disease 2019 (COVID-19) is of global concern due to the potential for nosocomial spread and depletion of staff numbers. However, the literature on transmission routes and risk factors for COVID-19 in HCWs is limited. AIM: To examine the characteristics and transmission dynamics of severe acute respiratory syndrome coronavirus 2 (SARS-CoV-2) in HCWs in a university teaching hospital in London, UK. METHODS: Staff records and virology testing results were combined to identify staff sickness and COVID-19 rates from March to April 2020. Comparisons were made with staff professional groups, department of work, and ethnicity. FINDINGS: COVID-19 rates in our HCWs largely rose and declined in parallel with the number of community cases. White and non-White ethnic groups among our HCWs had similar rates of infection. Clinical staff had a higher rate of laboratory-confirmed COVID-19 than non-clinical staff, but total sickness rates were similar. Doctors had the highest rate of infection, but took the fewest sickness days. Critical care had lower rates than the emergency department (ED), but rates in the ED declined when all staff were advised to use personal protective equipment (PPE). CONCLUSION: Sustained transmission of SARS-CoV-2 among our hospital staff did not occur, beyond the community outbreak, even in the absence of strict infection control measures in non-clinical areas. Current PPE appears to be effective when used appropriately. Our findings emphasize the importance of testing both clinical and non-clinical staff groups during a pandemic.

Zheng, H., et al. (2020). "Perioperative management of patients with suspected or confirmed COVID-19: review and recommendations for perioperative management from a retrospective cohort study." Br J Anaesth **125**(6): 895-911.

BACKGROUND: Current guidelines for perioperative management of coronavirus disease 19 (COVID-19) are mainly based on extrapolated evidence or expert opinion. We aimed to systematically investigate how COVID-19 affects perioperative management and clinical outcomes, to develop evidence-based guidelines. METHODS: First, we conducted a rapid literature review in EMBASE, MEDLINE, PubMed, Scopus, and Web of Science (January 1 to July 1, 2020), using a predefined protocol. Second, we performed a retrospective cohort analysis of 166 women undergoing Caesarean section at Tongji Hospital, Wuhan during the COVID-19 pandemic. Demographic, imaging, laboratory, and clinical data were obtained from electronic medical records. RESULTS: The review identified 26 studies, mainly case reports/series. One large cohort reported greater mortality in elective surgery patients diagnosed after, rather than before surgery. Higher 30 day mortality was associated with emergency surgery, major surgery, poorer preoperative condition and surgery for malignancy. Regional anaesthesia was favoured in most studies and personal protective equipment (PPE) was generally used by healthcare workers (HCWs), but its use was poorly described for patients. In the retrospective cohort study, duration of surgery, oxygen therapy and hospital stay were longer in suspected or confirmed patients than negative patients, but there were no differences in neonatal outcomes. None of the 262 participating HCWs was infected with severe acute respiratory syndrome coronavirus 2 (SARS-CoV-2) when using level 3 PPE perioperatively. CONCLUSIONS: When COVID-19 is suspected, testing should be considered before non-urgent surgery. Until further evidence is available, HCWs should use level 3 PPE perioperatively for suspected or confirmed patients, but research is needed on its timing and specifications. Further research must examine longer-term outcomes. CLINICAL TRIAL REGISTRATION: CRD42020182891 (PROSPERO).

Zheng, H., et al. (2020). "Clinical experience with emergency endotracheal intubation in COVID-19 patients in the intensive care units: a single-centered, retrospective, descriptive study." Am J Transl Res **12**(10): 6655-6664.

Few studies have reported the implications of performing endotracheal intubation for critically ill COVID-19 patients admitted to intensive care units (ICUs). Therefore, this study aimed to summarize the outcomes of COVID-19 patients in the ICU following endotracheal intubation and provide a clinical reference for the high-risk procedure. From February 1 to February 18, 2020, we enrolled 59 critically ill COVID-19 patients who received emergency endotracheal intubation in the ICUs of Tongji Hospital. We recorded demographic information, laboratory parameters, comorbidities, changes in vital signs pre- and post-intubation, the airway grade, intubation success rate using three types of laryngoscopes, and the experience of intubators. Follow-up evaluations were performed for all proceduralists to monitor nosocomial infections. The majority of the patients requiring intubation were elderly and had at least one comorbidity. Of the patients, 86.4% developed hypoxia before intubation. The first and second attempts of successful endotracheal intubation with the Macintosh laryngoscope (70.0% and 83.3%), Airtraq videolaryngoscope (93.5% and 80%), and UE videolaryngoscope (88.9% and 100%) were performed. Notably, SpO(2) <93% and hypotension were observed 3 min after intubation in 32.2% and 39% patients, respectively. With the proper use of personal protective equipment (PPE), no nosocomial infections were observed among proceduralists. Full PPE increased the occurrence of fogging on goggles and myopia glasses. Overall, a higher success rate of intubation was achieved by senior intubators using a videolaryngoscope. Although inconvenient, appropriate ensembles of PPE could prevent nosocomial infections.

Zheng, K. I., et al. (2020). "COVID-19 Cross-Infection and Pressured Ulceration Among Healthcare Workers: Are We Really Protected by Respirators?" Front Med (Lausanne) **7**: 571493.

Zheng, L., et al. (2020). "Analysis of the Infection Status of Healthcare Workers in Wuhan During the COVID-19 Outbreak: A Cross-sectional Study." Clin Infect Dis **71**(16): 2109-2113.

BACKGROUND: Healthcare workers (HCWs) at the frontline are facing a substantial risk of infection during the coronavirus disease 2019 (COVID-19) outbreak. METHODS: We acquired information and data on general information on and infection and death status of HCWs in Wuhan during the COVID-19 outbreak and completed statistical analyses. RESULTS: We obtained the data on 2457 infected cases among HCWs in Wuhan, China. More than half of the infected individuals were nurses (52.06%), whereas 33.62% of infected cases were doctors and 14.33% of cases were medical staff. In particular, the case infection rate of nurses (2.22%) was remarkably higher than that of doctors (1.92%). Most infected cases among HCWs were female (72.28%). A majority of the infected HCWs (89.26%) came from general hospitals, followed by specialized hospitals (5.70%) and community hospitals (5.05%). The case infection rate of HCWs (2.10%) was dramatically higher than that of non-HCWs (0.43%). The case fatality rate of HCWs (0.69%) was significantly lower than that of non-HCWs (5.30%). CONCLUSIONS: The infection risk of HCWs is clearly higher than that of non-HCWs. HCWs play an essential role in fighting the pandemic. The analysis of the infection status of HCWs is essential to attract enough attention from the public, provide effective suggestions for government agencies, and improve protective measures for HCWs.

Zheng, R., et al. (2021). "Prevalence and associated factors of depression, anxiety, and stress among Hubei pediatric nurses during COVID-19 pandemic." Compr Psychiatry **104**: 152217.

BACKGROUND: The COVID-19 pandemic is putting healthcare workers across the world in an unprecedented situation. The purpose of this study was to evaluate the levels of depression, anxiety, and stress among Hubei pediatric nurses during the COVID-19 pandemic and to analyze the potential factors associated with them. MATERIALS AND METHODS: A self-designed online questionnaire survey, which consisted of the demographic and selected features, the occupational protection knowledge, attitudes, and practices of COVID-19, and the Chinese version of Depression, Anxiety, and Stress Scale, were used to assess the levels of depression, anxiety, and stress among Hubei pediatric nurses during COVID-19 pandemic. The logistic regression analyses were performed to analyze the potential factors associated with depression, anxiety, and stress. RESULTS: A total of 617 pediatric nurses were included in the survey. A considerable proportion of pediatric nurses reported symptoms of depression (95 [15.4%]), anxiety (201 [32.6%]), and stress (111 [18.0%]). Results of multivariable logistic regression analyses indicated that the good occupational protection practices (for depression: OR = 0.455, 95%CI: 0.281 to 0.739; for anxiety: OR = 0.597, 95%CI: 0.419 to 0.851; for stress: OR = 0.269, 95%CI: 0.166 to 0.438) and the personal protective equipment (PPE) meeting work requirements (for depression: OR = 0.438, 95%CI: 0.246 to 0.778; for anxiety: OR = 0.581, 95%CI: 0.352 to 0.959; for stress: OR = 0.504, 95%CI: 0.283 to 0.898) were independent protective factors against depression, anxiety, and stress, respectively. Yet, working in an isolation ward or fever clinic was an independent risk factor associated with depression, anxiety, and stress, respectively (for depression: OR = 1.809, 95%CI: 1.103 to 2.966; for anxiety: OR = 1.864, 95%CI: 1.221 to 2.846; for stress: OR = 2.974, 95%CI: 1.866 to 4.741). Having suspected or confirmed COVID-19 patients in the departments (OR = 1.554, 95%CI: 1.053 to 2.294) and coming in contact with the patient's bodily fluids or blood (OR = 1.469, 95%CI: 1.031 to 2.095) were independent risk factors for anxiety, while >3 times of training for COVID-19 related information was an independent protective factor for depression (OR = 0.592, 95%CI: 0.360 to 0.974). Moreover, >10 years of working was an independent risk factor for stress (OR = 1.678, 95%CI: 1.075 to 2.618). CONCLUSION: During the COVID-19 outbreak, a considerable proportion of Hubei pediatric nurses had psychological problems. The pediatric nurses endorsing the higher number of risk factors should be given special attention and necessary psychological intervention. Improving the levels of PPE so as to meet the work requirements and intensifying occupational protection practices might help safeguard pediatric nurses from depression, anxiety, and stress.

Zhong, T., et al. (2020). "Perioperative management for patients with coronavirus disease 2019." Zhong Nan Da Xue Xue Bao Yi Xue Ban **45**(5): 609-612.

The coronavirus disease 2019 (COVID-19) is a new infectious disease, which has a strong virus transmission power and complex transmission routes. This disease is prone to outbreak of cluster infection. It is difficult for medical workers to provide a better perioperative treatment for surgery patient with COVID-19 while avoiding hospital spread effectively. The perioperative management for such patients needs to fully consider the possible lung injury factors caused by anesthesia and surgery. It also needs to choose the suitable timing of the operation, carry out preoperative infection screening and evaluation, and implement lung protection strategies during and after the operation to avoid aggravating the lung injury. Meanwhile, it is necessary to pay more attention to infection prevention and control in order to avoid nosocomial infection.

Zhou, Q., et al. (2020). "Nosocomial infections among patients with COVID-19, SARS and MERS: a rapid review and meta-analysis." Ann Transl Med **8**(10): 629.

BACKGROUND: COVID-19, a disease caused by SARS-CoV-2 coronavirus, has now spread to most countries and regions of the world. As patients potentially infected by SARS-CoV-2 need to visit hospitals, the incidence of nosocomial infection can be expected to be high. Therefore, a comprehensive and objective understanding of nosocomial infection is needed to guide the prevention and control of the epidemic. METHODS: We searched major international and Chinese databases: Medicine, Web of Science, Embase, Cochrane, CBM (China Biology Medicine disc), CNKI (China National Knowledge Infrastructure) and Wanfang database for case series or case reports on nosocomial infections of COVID-19, SARS (severe acute respiratory syndromes) and MERS (Middle East respiratory syndrome) from their inception to March 31st, 2020. We conducted a meta-analysis of the proportion of nosocomial infection patients in the diagnosed patients, occupational distribution of nosocomial infection medical staff. RESULTS: We included 40 studies. Among the confirmed patients, the proportions of nosocomial infections with early outbreaks of COVID-19, SARS, and MERS were 44.0%, 36.0%, and 56.0%, respectively. Of the confirmed patients, the medical staff and other hospital-acquired infections accounted for 33.0% and 2.0% of COVID-19 cases, 37.0% and 24.0% of SARS cases, and 19.0% and 36.0% of MERS cases, respectively. Nurses and doctors were the most affected among the infected medical staff. The mean numbers of secondary cases caused by one index patient were 29.3 and 6.3 for SARS and MERS, respectively. CONCLUSIONS: The proportion of nosocomial infection in patients with COVID-19 was 44% in the early outbreak. Patients attending hospitals should take personal protection. Medical staff should be awareness of the disease to protect themselves and the patients.

Zhou, Y. and L. S. Cen (2020). "Managing acute appendicitis during the COVID-19 pandemic in Jiaxing, China." World J Clin Cases **8**(19): 4349-4359.

BACKGROUND: Coronavirus disease 19 (COVID-19) is a global pandemic and has had a profound impact on our routine surgical activities. Acute appendicitis is the most common abdominal emergency worldwide. Therefore, it is highly essential to assess the influence the pandemic has on acute appendicitis. AIM: To assess the efficacy of the management of acute appendicitis during the COVID-19 pandemic. METHODS: We retrospectively analyzed 90 patients who presented with acute appendicitis during the outbreak of COVID-19 in Jiaxing, China. Clinical data regarding appendectomies patients were also collected for the corresponding time frame from 2019. Preoperative management, intraoperative protective measures, and postoperative management were conducted. RESULTS: After screening, six patients were identified as unqualified due to fever and were then referred to the COVID-19 expert group. The results of the nucleic acid test were negative. Of the 76 patients enrolled in the simple group, nine patients received medication therapy, and all others underwent surgery. From this same group, 66 patients were diagnosed with suppurative appendicitis, and one patient was diagnosed with perforated appendicitis after surgery. There were 14 patients in the complex group, for which the postoperative diagnosis indicated perforated appendicitis. The proportion of men with perforated appendicitis was higher than that in 2019 (P < 0.05). The chief complaint duration for perforated appendicitis patients in 2020 was longer than that in 2019 (P < 0.05). The routine blood test showed that white blood cell counts and neutrophil ratios were higher in perforated appendicitis patients in 2020 than in 2019 (P < 0.05). The ratio of open appendectomies to the amount of mean blood loss during surgery was greater in 2020 than in 2019 (P < 0.05). Online consultation after discharge was selected in 59 cases (65.6%). No perioperative infection with COVID-19 or long-term postoperative complications were found. CONCLUSION: The management of acute appendicitis from Jiaxing effectively reduced the influence of the pandemic and minimized the risk of nosocomial infection.

Zhu, J., et al. (2021). "The ethics of refusing to care for patients during the coronavirus pandemic: A Chinese perspective." Nurs Inq **28**(1): e12380.

As a result of the coronavirus (COVID-19) pandemic, health professionals are faced with situations they have not previously encountered and are being forced to make difficult ethical decisions. As the first group to experience challenges of caring for patients with coronavirus, Chinese nurses endure heartbreak and face stressful moral dilemmas. In this opinion piece, we examine three related critical questions: Whether society has the right to require health professionals to risk their lives caring for patients; whether health professionals have the right to refuse to care for patients during the coronavirus pandemic; and what obligations there are to protect health professionals? Value of care, community expectations, legal obligations, professional and codes of practice may compel health professionals to put themselves at risks in emergency situations. The bioethical principles of autonomy, justice, beneficence and non-maleficence, as well as public health ethics, guide nurses to justify their decisions as to whether they are entitled to refuse to treat COVID-19 patients during the pandemic. We hope that the open discussion would support the international society in addressing similar ethical challenges in their respective situations during this public health crisis.

Zhu, Y., et al. (2020). "Chinese Society of Interventional Radiology Expert Consensus on the prevention and control of COVID-19 in interventional radiology procedures (first edition)." Quant Imaging Med Surg **10**(5): 1045-1057.

The COVID-19 pandemic seriously threatens the lives of the general public and poses momentous challenges to all medical workers, including those engaged in interventional radiology, who play an important role in the diagnosis and treatment of various diseases. To further standardize the prevention and control of nosocomial infections and ensure the safety of doctors and patients, the Chinese Society of Interventional Radiology (CSIR) organized multidisciplinary experts in the field of interventional radiology in China to prepare an "Expert Consensus" elaborating and summarizing the protective strategies and suggestions for medical workers in the field of interventional radiology when they engage in interventional diagnosis and treatment activities against the background of novel coronavirus infection control. The aim is to provide a reference for interventional procedures in hospitals and other medical institutions at all levels in China and worldwide. The key points include the following: (I) non-emergency interventional diagnosis and treatment should be suspended while work is ongoing to prevent and control the spread of COVID-19; (II) protective measures should be taken according to the appropriate level designated for COVID-19 infection prevention and control; (III) patients should take measures to protect themselves when they want to see a doctor, including accessing outpatient services online and other relevant channels of consultation.

Zolkefli, Y. (2020). "Be Honest: Individuals' Moral Responsibility within the COVID-19 Context." Malays J Med Sci **27**(6): 144-147.

We recognise that people lie to health professionals for several reasons. However, these incidents endanger the well-being of the professionals and bring us to the question of whether people have an exclusive moral duty to always profess the truth about their health and other facts, particularly in a pandemic crisis. This review argues that an honest patient is a key to undertaking their roles as health professionals and delivering the best services possible to meet the needs of the patient. Greater awareness and comprehension of the potential ramifications of dishonesty, not only helps establish the moral obligation, to tell the truth, particularly in a pandemic situation, but also translates into a better relationship with health professionals. It also enforces an ethical solidarity on every single of us to show tangible moral response to ensure that those most vulnerable to risks from the pandemic illness such as health professionals are protected as far as possible.

Zuliani, S., et al. (2020). "Organisational challenges, volumes of oncological activity and patients' perception during the severe acute respiratory syndrome coronavirus 2 epidemic." Eur J Cancer **135**: 159-169.

BACKGROUND: On February 23rd, the 1st case of severe acute respiratory syndrome coronavirus 2 (SARS-CoV-2) infection was diagnosed at the University Hospital Trust of Verona, Italy. On March 13th, the Oncology Section was converted into a 22-inpatient bed coronavirus disease (COVID) Unit, and we reshaped our organisation to face the SARS-CoV-2 epidemic, while maintaining oncological activities. METHODS: We tracked down (i) volumes of oncological activities (January 1st - March 31st, 2020 versus the same period of 2019), (ii) patients' and caregivers' perception and (iii) SARS-CoV-2 infection rate in oncology health professionals and SARS-CoV-2 infection-related hospital admissions of "active"' oncological patients. RESULTS: As compared with the same trimester in 2019, the overall reduction in total numbers of inpatient admissions, chemotherapy administrations and specialist visits in January-March 2020 was 8%, 6% and 3%, respectively; based on the weekly average of daily accesses, reduction in some of the oncological activities became statistically significant from week 11. The overall acceptance of adopted measures, as measured by targeted questionnaires administered to a sample of 241 outpatients, was high (>70%). Overall, 8 of 85 oncology health professionals tested positive for SARS-CoV-2 infection (all but one employed in the COVID Unit, no hospital admissions and no treatment required); among 471 patients admitted for SARS-CoV-2 infection, 7 had an "active"' oncological disease (2 died of infection-related complications). CONCLUSIONS: A slight, but statistically significant reduction in oncology activity was registered during the SARS-CoV-2 epidemic peak in Verona, Italy. Organisational and protective measures adopted appear to have contributed to keep infections in both oncological patients and health professionals to a minimum.

# List of papers in Web of Science for first screening

Abu-Zidan, Y., et al. (2021). "DESIGN OF A SMART PREFABRICATED SANITISING CHAMBER FOR COVID-19 USING COMPUTATIONAL FLUID DYNAMICS." Journal of Civil Engineering and Management **27**(2): 139-148.

The novel coronavirus (SARS-CoV-2) has spread at an unprecedented rate, resulting in a global pandemic (COVID-19) that has strained healthcare systems and claimed many lives. Front-line healthcare workers are among the most at risk of contracting and spreading the virus due to close contact with infected patients and settings of high viral loads. To provide these workers with an extra layer of protection, the authors propose a low-cost, prefabricated, and portable sanitising chamber that sprays individuals with sanitising fluid to disinfect clothing and external surfaces on their person. The study discusses computer-aided design of the chamber to improve uniformity of sanitiser deposition and reduce discomfort due to excessive moisture. Advanced computational fluid dynamics is used to simulate the dispersion and deposition of spray particle, and the resulting wetting pattern on the treated person is used to optimise the chamber design.

Ader, L. G. M., et al. (2022). mHealth Use in Healthcare Facilities: Raising Awareness in Data Protection, Privacy and Safety. 15th International Conference on Health Informatics (HEALTHINF) held as part of 15th International Joint Conference on Biomedical Engineering Systems and Technologies (BIOSTEC), Electr Network.

During the COVID-19 pandemic, many patients and healthcare professionals embraced the possibility of using available mobile devices and applications, exploring the opportunities to reduce the burden on strained services. However, despite strict surveillance under the European GDPR or Medical Device (MD) regulations, users are considered to be primarily responsible for verifying that their application of choice is approved and certified. We searched academic and grey literature and discuss some of the challenges related to the use of personal devices and mobile applications for health and medical purposes. Our position is that policies and technologies should be more considerate of users' behaviour, which includes use of non-medical software for medical purposes, and situations where users seem to choose usability over safety.

Agius, A. M., et al. (2020). "Experiences and Responses of Oral Health Care Professionals during the First Wave of the COVID-19 Pandemic in Malta." Community Dental Health **38**(4): 226-229.

Initial impetus for action: Maltese Oral Health Care Professionals (OHCP) experienced changes to clinical practice and redeployment during the COVID-19 pandemic. In the early stages, there were few data on the resultant changes to the provision of dental services or their impact on the wellbeing of dental professionals. Solutions Suggested: Two questionnaires were designed to explore different domains in OHCP working on the frontline of the COVID-19 pandemic as well as in other areas/sectors. The findings were intended to inform guidance documents and to better support the profession. Findings: Anxiety-provoking factors, challenges, and areas of concern of OHCP were identified and practical recommendations to support transitioning to the 'new normal' were presented. OHCP expressed anxiety about contracting COVID-19 from their workplace and passing infection to other family members, friends, or patients. As a result, OHCP expressed the need for better protective clothing, workplace ventilation, and air filtration systems. Future implications: Provision of adequate PPE for OHCP was a priority. New guidelines for dental practice were informed by the experiences of the particpating OHCP. University modules to enhance the resilience of OHCP were among the recommendations to support practitioners. Public health competencies illustrated: center dot Understanding the barriers to provision of oral care and health protection of the workforce and population during the COVID-19 pandemic. center dot Understanding the experiences and needs of oral health care professionals (OHCP) during the pandemic and learning from them.

Aguilar-Garduno, C. O., et al. (2021). "Surgical initial experience in patients with COVID-19." Cirugia Y Cirujanos **89**(2): 183-188.

Introduction: the COVID-19 pandemic has caused a reorganization of hospital and general surgery departments worldwide to assure the best medical and surgical treatment of patients with this disease and protection of the health-related personnel. Some of them will require surgical treatment either elective or urgent. Objective: report the initial experience in the management of patients with COVID-19 in a third level hospital. Material and Methods: a review of the surgical protocols, personal protection equipment used by the surgical teams, and results of the treatment of forty-two patients submitted to surgery. Results: During four months (April-July 2020) forty-two patients with suspicion or confirmed infection of SARS-CoV2 underwent surgical treatment. The most common surgery was tracheostomy in 16 patients (38%) followed by exploratory laparotomy in 8 patients (19%). The median postoperative stay was 17 days and the thirty-day postoperative mortality rate was 26%. Conclusions: reorganization of the general surgery department and the hospital, favors adequate management and treatment of patients with COVID-19 and protection to the health-related personnel. Due to the usual co-existence of comorbidities and pulmonary complications the postoperative mortality of these patients is high.

Ahmed, M. H. (2020). "Black and Minority Ethnic (BAME) Alliance Against COVID-19: One Step Forward." Journal of Racial and Ethnic Health Disparities **7**(5): 822-828.

During the COVID-19 pandemic, the data of Department of Health in United Kingdom (UK) showed an increase mortality and morbidity among the Black and Minority Ethnic (BAME) population. This high mortality can be due to social factors, genetic and immunological factors. Metabolic factors like high prevalence of diabetes, obesity, metabolic syndrome and hypertension were also found to contribute to the increased risk of COVID-19 infection in BAME population. In addition, a large number of BAME population are working in jobs that involve regular and daily contact with public, and this may increase risk of encountering COVID-19 infection. Therefore, future research should address all these factors and generate the correct health policy that will allow us to combat the danger of COVID-19. We recommend the establishment of BAME alliance against COVID-19 in order to improve occupational risks and hazards, adequate income protection, culturally and linguistically appropriate public health communications and decreasing barriers in accessing healthcare. The BAME alliance will initially focus on (i) development of central system to record data about COVID-19 patients from BAME population (ii) involvement of healthcare professionals and researchers from ethnic minorities and (iii) multidisciplinary and inter-institution collaboration; for example, civil engineers and architects need to think about house design and ventilation that decrease risk of COVID-19 especially in BAME populations.

Aikins, A. D., et al. (2021). "COVID-19, Chronic Conditions and Structural Poverty: A Social Psychological Assessment of the Needs of a Marginalized Community in Accra, Ghana." Journal of Social and Political Psychology **9**(2): 577-591.

In the African region COVID-19 infection and death rates are increasing (writing in May 2020), most deaths have occurred among individuals with chronic conditions, and poor communities face higher risks of infection and socio-economic insecurities. We assessed the psychosocial needs of a chronic illness support group in Accra, Ghana, within the context of their broader community. The community lives in structural poverty and has a complex burden of infectious and chronic non-communicable diseases (NCDs). Between March and May 2020, we conducted interviews, group discussions, and surveys, with members of the support group and their caregivers, frontline healthcare workers, and religious and community leaders. Data was analysed through the social psychology of participation framework. Community members understood COVID-19 as a new public health threat and drew on eclectic sources of information to make sense of this. Members of the support group had psychosocial and material needs: they were anxious about infection risk as well as money, food and access to NCD treatment. Some community members received government food packages during the lockdown period. This support ended after lockdown in April and while anti-poverty COVID policies have been unveiled they have yet to be implemented. We discuss the impact of these representational, relational and power dynamics on the community's access to COVID-19 and NCD support. We argue that strategies to address immediate and post-COVID needs of vulnerable communities have to focus on the politics and practicalities of implementing existing rights-based policies that intersect health, poverty reduction and social protection.

Akinleye, F. E., et al. (2020). "Towards universal health coverage: lessons learnt from the COVID-19 pandemic in Africa." Pan African Medical Journal **35**.

Access to quality and affordable health services is a significant element of social protection, specifically, through Universal Health Coverage (UHC). This current pandemic has shown that health systems and economies in several countries are not strong enough to tackle a massive health crisis. It has revealed limited and underfunded health services, inadequate and ineffective equipment, few and often unprotected health workers and a generally fragile healthcare system.

Al Demour, S., et al. (2021). "Knowledge, Practice, and Attitude Toward COVID-19 Among Physicians in Jordan and Palestine: Cross-Sectional Study." International Journal of General Medicine **14**: 77-87.

Background and Objectives: Coronavirus disease-2019 (COVID-19) is an emerging disease threatening the world with a rapid increase in cases and deaths since it was first identified in December 2019. Adequate knowledge, practice, and attitudes (KPA) toward COVID-19 among physicians at the frontline defense against the COVID-19 pandemic may enhance their ability to avoid the risk of self-infection, decrease mortality, and provide adequate medical care service in this pandemic. This study aimed to assess KPA toward COVID-19 among physicians in Jordan and Palestine. Methods: This is a cross-sectional study using an online survey conducted from 10 April to 26 April 2020 among Jordan and Palestine physicians. Invitations were sent to physician groups on Facebook and WhatsApp. This survey contains 36-items, divided into four main sections to assess the participants' socio-demographic characteristics, knowledge, practice, and attitude about COVID-19. Results: A total of 454 physicians participated in this study. The mean score of basic knowledge was 4.4 +/- 0.8 (range 2-4). There were significant differences between basic knowledge mean scores among physicians in different professional degrees and physicians in various health sectors (P=0.0315, P=0.0137, respectively). The mean scores of self-protection measures, were 6.1 +/- 1.1 (range 3-7) and measures if physician self-suspected of COVID-19 were 9.9 +/- 1.1 (range 5-11). The mean score of attitudes toward COVID-19 was 41.5 +/- 3.3 (range 21-45) and significantly related to the age and different experience years (P=0.0022, P=0.0077, respectively). Conclusion: As the global threat of COVID-19 continues to emerge, physicians from Jordan and Palestine showed adequate KPA toward COVID-19. There was a significant difference in knowledge level and attitude between physicians. Policymakers and physicians should keep continuous educational activities, training, and follow-up updates during this pandemic.

Alao, M. A., et al. (2020). "Assessment of Health Workers' Knowledge, Beliefs, Attitudes, and Use of Personal Protective Equipment for Prevention of COVID-19 Infection in Low-Resource Settings." Advances in Public Health **2020**.

Background.Severe acute respiratory syndrome coronavirus 2 (SARS-CoV-2) is a highly infectious disease with a potential for healthcare workers (HCWs) getting infected due to inadequate protection while attending to patients. Effective use of personal protective equipment (PPE) is key to mitigating the spread of SARS-CoV-2 infection in healthcare settings. Hence, there is a need to understand HCWs' use of PPE in resource-limited settings and how closely the currently recommended guidelines for PPE are followed. This study assessed the HCWs' knowledge about, attitudes towards, beliefs on, and use of PPE to prevent SARS-CoV-2 infection in a resource-limited setting.Methods. This cross-sectional study was conducted in April 2020 in Southwest and Northwest Nigeria. The selection of participants was performed via the snowball sampling technique using a 33-item, web-based, self-administered questionnaire via a social media network. We obtained relevant sociodemographic data and information on participants' occupations and knowledge about, attitudes towards, beliefs on, and use of PPE. We analysed the data using SPSS version 23.0 for Windows (IBM, Armonk, New York, USA). Apvalues Results. A total of 290 subjects responded to the questionnaire, and 18 (6.2%) were excluded because of incomplete data. The mean age of the respondents was 32.3 +/- 9.9 years. There were 116 males (42.6%). The majority of the respondents were medical doctors (114, 41.9%), followed by nurses and clinical students. Of the 272 respondents in this survey, only 70 (25.7%) had adequate knowledge about PPE. Of the respondents who presumed they had adequate knowledge about donning and doffing PPE, 94 (56%) were incorrect. The predictors of good knowledge were ages younger than 45 years (p=0.046) and practice location (p=0.009).Conclusion. This study showed that HCWs' knowledge about, attitudes towards, and beliefs on PPE and their PPE skill in practice in Nigeria were remarkably poor. There is an urgent need for nationwide practical training on PPE use to curtail the spread of SARS-CoV-2 infection among HCWs.

Alebouyeh, M., et al. (2021). "Re-positive PCR of SARS-CoV-2 in health care persons during COVID-19 pandemic." Cellular and Molecular Biology **67**(5): 138-143.

Reinfection rate with SARS-CoV-2 and degree of protection by the induced antibody after the first episode of the infection is not well known, so it makes a big dilemma for health care personnel (HCP) who work in the front line of combating SARS-CoV-2. In this study, we investigated the frequency of SARS-CoV-2 redetection among HCP after the initial onset of the infection in a children's hospital during one year. Out of 131 seropositive HCP, 13.7% of them were symptomatic and PCR positive during 74-360 days after first sampling. Analysis of demographic data of seropositive HCP showed a correlation between a higher number of family members, higher body mass index, and the existence of underlying diseases with SARS-CoV-2 redetection. In conclusion, reinfection is one of the important problems in the SARS-CoV-2 pandemic. Research on this topic can help us to find answers to questions for estimating the duration of human protection with produced immunity after the infection or vaccination.

Almazroa, A. (2021). "Sustainability of Ophthalmology Practice and Training During and Post the Pandemic of Coronavirus (COVID-19): A Review." Clinical Ophthalmology **15**: 2355-2365.

Purpose: To review and analyse the globally established ophthalmic practice protocols during the coronavirus disease (COVID-19). Methods: A literature review using search strategy was conducted to identify appropriate publications relevant to COVID-19 and ophthalmology practice and training. The safety and feasibility of the protocols were illustrated and discussed. Results: Challenges in different eye care settings at various international ophthalmology departments have identified and analysed to introduce solutions. Several clinical protocols were established and concerned for screening procedures, waiting area, clinical flow (ie, patients' registration, personal (patients and healthcare workers) protection), and equipment safety in the clinics and operation rooms. Discussion: In the review of this protocol, the strategic and operational missions of the Academic Medical Centers (AMCs) are demonstrated and discussed. This is in addition to the sustainability of the established protocols for cataract surgeries and glaucoma clinics and training during and after COVID-19. Conclusion: All the protocols have established for temporary circumstances, such as postponing elective appointments and surgeries as well as applying the technology for regular follow-ups (transmission of image, video, and face-to-face interactions via widely available applications). Only, one protocol was stronger for the sustainability. Accordingly, recommendations are suggested for clinical sustainability during and after COVID-19.

Alshahrani, S. M., et al. (2021). "Perspective of healthcare workers regarding self-protection during COVID-19 pandemic: A cross-sectional study from Saudi Arabia." International Journal of Advanced and Applied Sciences **8**(2): 101-105.

The aim of this study was to assess the perception of healthcare workers regarding self-protection during the COVID-19 pandemic in Saudi Arabia. The novel COVID-19 pandemic has completely changed the dynamic of governments, social lives, global economy, and health care systems priorities. Healthcare workers (HCWs) are one of the most group of people at risk of acquiring the infection, especially those who are taking care of COVID-19 patients. This cross-sectional survey-based study was conducted among HCWs during the period between May to July 2020 in Saudi Arabia. More than 70% of participating HCWs were able to access the personal safety policies and procedures in the workplace and COVID-19 treatment algorithm. In addition, the presence of an infection control team was also present in most of the institutes. The most common accessible personal protective equipment was hand gel sanitizer (89.9%), followed by disposable gloves (82.5%) and disposable masks (78.9%). More than 75% of participants reported that their institute has a special infection control team during the COVID-19 pandemic. Most of the respondents (similar to 75%) believe that their institute would take all necessary measurements to protect their personal integrity at work. Several precautionary measures were undertaken appropriately by the Saudi Government to overcome the COVID-19 immediate and futuristic consequences. Personal protective equipment and protective measures would be crucial for public health if implemented appropriately during highly spreading infections (e.g., COVID-19) to minimize the transmission and preserve health. (C) 2020 The Authors. Published by IASE.

Alvarez-Hernandez, L. R., et al. "COVID-19 Pandemic and Intimate Partner Violence: an Analysis of Help-Seeking Messages in the Spanish-Speaking Media." Journal of Family Violence.

The role of the Spanish-speaking media is crucial for how Latinx communities learn about seeking help when experiencing intimate partner violence (IPV). This study investigated the IPV help-seeking messages disseminated by the Spanish-speaking media in the U.S. during the COVID-19 pandemic. We engaged in an exploratory content analysis of videos from Univision's main website, the most-watched Spanish-speaking media network in the U.S. We searched for videos related to IPV help-seeking posted from March 19-April 21, 2020-including the weeks after the World Health Organization declared COVID-19 a global pandemic and the U.S. mandated a shelter-in-place. After assessing inclusion criteria, 29 videos were analyzed. Data were analyzed using basic content analysis to determine frequencies and inductive interpretive content analysis to code for help-seeking messages. We identified eight manifest messages related to seeking help when experiencing IPV in times of a crisis: (1) contact a professional resource; (2) contact law enforcement; (3) contact family, friends, and members of your community; (4) create a safety plan; (5) don't be afraid, be strong; (6) leave the situation; (7) protect yourself at home; and (8) services are available despite the pandemic. We found that the manifest messages alluded to three latent messages: (1) it is your responsibility to change your circumstances; (2) you are in danger and in need of protection; and (3) you are not alone. IPV and media professionals should ensure a structural understanding of IPV in their help-seeking messages and avoid perpetrating stigmatizing and reductionist messages.

Ammor, Y. M., et al. (2020). "Managing cancer patients during COVID-19 pandemic: a North African oncological center experience." Pan African Medical Journal **35**.

Introduction: since the first spread of the novel coronavirus (COVID-19) in Morocco in March 2020, the Moroccan Health System underwent an important pressure and remarkable efforts were spent to provide efficient reactions to this emergency. Public hospitals have set adapted strategies dedicated to overcoming the overload of COVID-19 patients, and our Oncology and Hematology Center (OHC) has implemented a flexible adapted strategy aiming to reduce the burden of COVID-19. We report our single-center experience on the detailed infection control measures undertaken to minimize virus transmission. Methods: we reviewed all patients treated at the OHC from March 2nd to April 20th, 2020 as measures were taken since the detection of the first COVID-19 case to ensure the protection of patients and healthcare providers, especially a screening zone for any patient entering the center. The patient's data were retrospectively collected and anonymized. Results: we notified a significant decrease in patients' admissions during the lockdown period at the different units of our center. The screening area received a total of 5267 patients during our study period, with an average of 105 patients per day. Interestingly, no healthcare professional was infected and only 8 patients showed symptoms of fever and cough, and all of them had a negative test for COVID-19 (RT-PCR). Thus, the OHC is considered as a COVID-19 free center with zero cases among patients and healthcare providers. Conclusion: by having a 0% rate of infection, the efficiency of our measures is proven, but efforts are still needed as we have to measure the impact of this pandemic in our cancer management.

Anagiotos, A. and G. Petrikkos (2021). "Otolaryngology in the COVID-19 pandemic era: the impact on our clinical practice." European Archives of Oto-Rhino-Laryngology **278**(3): 629-636.

Purpose To give an overview of the current knowledge about COVID-19 pandemic and its impact on otolaryngology clinical practice. Methods Recent findings about SARS-CoV-2 virus and the COVID-19 infection it causes are reviewed. In addition to international databases and in the absence of hard scientific data, literature search included reports published online from scientific societies and other institutions. Results The role of anosmia as a COVID-19-related symptom is presented. Further, considerations about steroid administration in ENT-related conditions are also discussed. Due to the close work with mucosa surfaces of the upper aerodigestive tract, otolaryngologists and surrounding staff are considered high risk for coronavirus transmission. Hence, staff protection measures for ENT examinations, surgeries and other procedures during COVID-19 pandemic are recommended. Conclusion Knowledge and evidence about the impact of COVID-19 infection on otolaryngology clinical practice are accumulating rapidly. Additionally to patient's management, safety of health care professionals should be a main goal right now by following strict safety guidelines.

Ananda-Rajah, M., et al. (2021). "Hearing the voices of Australian healthcare workers during the COVID-19 pandemic." Bmj Leader **5**(1): 31-35.

Background The statistics of healthcare worker (HCW) COVID-19 infections do not convey the lived experience of HCWs during the pandemic. This study explores the working conditions and issues faced by Australian HCWs. Methods Qualitative analysis of free-text responses from Australian HCWs from 3 August to 26 October 2020 from an open letter calling for better respiratory protection for HCWs, transparent reporting of HCW COVID-19 infections and diversity in national infection control policy development. The open letter was sent to an email list of 23 000 HCWs from a previous campaign and promoted on social media. Results Among 3587 HCWs who signed the open letter during the study period, 569 free-text responses were analysed. Doctors and nurses accounted for 58% and 33% of respondents, respectively. Most respondents came from Victoria (48%), New South Wales (20%), Queensland (12%) or Western Australia (11%). Dominant themes included concerns about: work health and safety standards; guidelines on respiratory protection including the omission of fit-testing of P2/N95 respirators; deficiencies in the availability, quality, appropriateness and training of personal protective equipment; and a command-and-control culture that enabled bullying in response to concerns about safety that culminated a loss of trust in leadership, self-reported COVID-19 infections in some respondents and moral injury. Conclusion Deficiencies in work health and safety, respiratory protection, personal protective equipment and workplace culture have resulted in a loss of psychological and physical safety at work associated with an occupational moral injury. The challenge for healthcare leaders is to repair trust by addressing HCW concerns and fast track solutions in collaboration with them.

Anna, V. and M. Melinda (2021). "Medically relevant aspects of severe acute respiratory syndrome coronavirus 2 (SARS-CoV-2) in animals." Orvosi Hetilap **162**(5): 163-170.

Since the discovery of the microscope, we have known that individuals living in close proximity to each other share not only their visible habitat, but also invisible microorganisms that have adapted to some degree to their hosts. However, as human habitat expands and, in parallel, animal territory declines, new animal species can come into dangerous proximity, which may result in consequential transmission of microbes and different microbial behaviour in the new host. Presumably, this phenomenon led to the development of the severe acute respiratory syndrome coronavirus 2 (SARS-CoV-2), which first appeared in humans in 2019 and infected millions over the course of a few months worldwide. All possible means must be used to combat the pandemic and prevent further epidemics, which will require cooperation between professionals in human medicine and veterinary medicine as well as in the ecological, evolutionary and environmental sciences, within the framework of the global "One Health". In a spirit of working together for common goals, the authors of this study, a veterinarian and a human physician, summarize the knowledge that may be relevant to both professions for SARS-CoV-2. The origin of the virus, its natural and artificial occurrence in different animal species, and experiences with animal coronaviruses that may contribute to the understanding of the functioning of SARS-CoV-2 and the development of protection against it are presented.

Anon, J. B., et al. (2020). "Patient-Worn Enhanced Protection Face Shield for Flexible Endoscopy." Otolaryngology-Head and Neck Surgery **163**(2): 280-283.

Objectives The primary objective of this study was to compare the protection afforded by a standard face shield design with a new enhanced design in a controlled setting. Methods This study was exempted from review by institutional review board waiver. A flexible fiberoptic endoscopy was placed through stellate openings in the standard face shield and the enhanced face shield. A series of simulated coughs were created with bursts of fluorescein dye through an atomizer tip placed within the test participant's mouth. Ultraviolet lighting illuminated the test area, and areas of dye splatter were noted. Results Fluorescein dye is easily aerosolized along the lateral inferior aspect of a standard shield with significant contamination of the surrounds. The enhanced face shield maintained a barrier to the aerosolized dye. Discussion Face shields, rather than face masks, should be considered a preferred alternative for the public and for health care professionals alike, as they address many of the personal protective equipment concerns especially during the COVID-19 pandemic. Otolaryngologists are at high risk from aerosol-generating procedures, such as flexible fiberoptic endoscopy, even when wearing personal protective equipment. Here we describe a uniquely designed face shield to be worn by the patient as another layer of protection for the environment and for medical personnel. Implications for Practice During the course of a flexible fiberoptic endoscopy, medical personnel are safely isolated from potential infectious particles with a newly designed face shield.

Apfelbeck, M., et al. (2021). "Does Pandemic Anxiety Affect Urology Health Care Workers?" Urologia Internationalis **105**(3-4): 192-198.

Purpose: To assess anxiety, stress level, and perception of safety during the coronavirus disease 2019 (COVID-19) pandemic in health care workers (HCWs) of one of Germany's largest urology university clinics. Methods: A cross-sectional study among urological HCWs was performed. HCWs were surveyed for anxiety about the pandemic, stress level and current workload, fear of coronavirus infection, current perception of safety at work, and attitude towards protective equipment and tests for severe acute respiratory syndrome coronavirus 2 (SARS-CoV-2). Results: Sixty-three HCWs filled in the questionnaire. Overall anxiety of infection with CO-VID-19 is at a median of 4.7 with no statistically significant difference between nurses and physicians (p = 0.0749). Safety at work reaches a median of 6 out of 10. In fact, the highest fear in 56.7% (31/63) of the personnel is to get infected by a colleague tested positive for SARS-CoV-2 despite wearing surgical face masks. A proportion of 55.7 and 74.6% highly favor swabs for SARS-CoV-2 on a regular basis in HCWs and patients, respectively (p = 0.0001). Workload in the urology department is clearly reduced during the pandemic (physicians 39.3% vs. nurses 32.2%, p = 0.0001) and 57.4% do not feel distress at all; only 27.9% express mental distress. Conclusion: During the pandemic, urology HCWs perceive lower burden by workload and deem themselves at low risk of infection. However, the greatest anxiety is related to infection by a SARS-CoV-2-positive colleague, despite reciprocal protection by surgical face masks. This highlights a relevant mental stress and uncertainty towards management of infected HCWs, calling for increased education and psychological support.

Arpaci, A. H. and B. Isik (2020). "Anesthesia Management in Patients with Covid-19." Cyprus Journal of Medical Sciences **5**(3): 267-273.

The coronavirus disease 2019 (COVID-19) pandemic that started in China in 2019 has led to millions of infection cases worldwide. As the number of people with COVID-19 has increased, the number of patients with health problems that require surgery during the pandemic also increased, and at the same time, anesthesia is needed for these patients. In patients infected with acute respiratory syndrome coronavirus 2, some features related to the clinical course of the disease should be known and considerations for the protection of healthcare workers and other patients due to increased contagiousness must be adopted. This review of the literature presents approaches in cases of patients diagnosed with COVID-19 and issues to be considered during anesthesia management.

Asmarawati, T. P., et al. (2020). "HEALTHCARE ASSOCIATED COVID-19 TRANSMISSION: STRATEGIES TO PREVENT." New Armenian Medical Journal **14**(4): 29-36.

Coronavirus disease 2019 (COVID-19) can cause another problem for healthcare facilities, which is nosocomial transmission. Clinicians performed nosocomial pneumonia prevention vigilantly during the COVID-19 pandemic. To date, the magnitude and risk factors for infection in healthcare environments are unknown in severe acute respiratory syndrome coronavirus 2 transmission. An overwhelming number of COVID-19 infections not only could paralyze the health system but could contribute, above all, to the occasional epidemic of hospital patients and the healthcare workers, along with elevated morbidity and mortality. The COVID-19 increasing case may limit the availability of occupancy rooms in hospitals, so doctors need tools to assess the likelihood of COVID-19 at the initial examination by triage based on epidemiological risks, routine investigations and bedside observation for safe isolation. The specific challenges of COVID-19 exist because this is a new disease with clinical, radiological, and laboratory features that can be variable We review nosocomial transmission of COVID-19. The data indicate that the prevalence of hospital-acquired COVID-19 infection varies. While reports regarding nosocomial transmission or healthcare-associated COVID-19 infection are still growing, several hospitals claim there were none or minimal nosocomial transmission. Infection prevention and control techniques were initially aimed at prompt patient identification, separation and monitoring in compliance with case descriptions. Engineering controls provide proper and efficient ventilation, likely augmented by filtration of pollutants and air disinfection, air recirculation protection, and overcrowding protection. Administrative assistance to reduce the risk of nosocomial severe acute respiratory syndrome coronavirus 2 is introduced with the reduction of choice healthcare services, decanting centers, deployment of isolation facilities, appropriate personal security facilities, coordination of the rapid molecular diagnostic laboratory network, constructive monitoring, and organization of the personnel forum and training. These principal strategies can suppress intra-hospital transmission of COVID-19 if strictly implemented during a pandemic era.

Assenza, G., et al. (2020). "Electroencephalography at the time of Covid-19 pandemic in Italy." Neurological Sciences **41**(8): 1999-2004.

Objective During the Covid-19 pandemic, government restrictions limited health care to urgent needs. Neurophysiology centers had to suddenly reschedule their activities, with a lack of specific recommendations about electroencephalography (EEG) execution. During the pandemic phase 1, we launched an online survey to understand the flaws and strengths of the EEG management in Italy at the time of Covid-19 pandemic. Methods A 45-item online survey (published from April 16 to 30, 2020), endorsed by the Italian Society of Clinical Neurophysiology (SINC), the Italian League Against epilepsy (LICE), and the Italian Association of Neurophysiology technologists (AITN), collected EEG management data (EEG's number and type, indications, personnel and patients safety, devices' sanification) during the Covid-19 pandemic. Results We received responses from 206 centers. The number of EEGs performed was reduced by 76 +/- 20%, and several types of specific EEG (video-EEG, ambulatory-EEG, LTM, polysomnography) were reduced at a minimum. Half of the centers performed inpatient EEGs only for urgencies. Repetitive seizures, encephalitis, and non-convulsive status epilepticus were the most common indications. Covid-19-positive patients received less EEG than negative ones (p < 0.0001). EEG requests came mainly not only from neurologists (n = 176) but also from general practitioners (n = 40), emergentists (n = 79), intensivists (n = 72), and other specialists (n = 53). Those centers which continued performing outpatient EEG examinations were instructed to perform the EEG after a Covid-19-related symptom screening for patients and using personal protective equipment (PPE) through all the procedure. Inpatient EEGs were performed using FFP2/FFP3 masks by neurophysiology technologists in only 50% of cases. Patients executed hyperventilation only for real clinical needs, but often (56%) with a mask. Conclusions Italian neurophysiology centers strongly adhered to government restrictions of lockdown. Some issues emerged, ranging from the evaluation of a proper indication for EEG, technical procedures of EEG recording, and protection of neurophysiology technicians.

Ayan, A. and F. S. Kirac (2020). "Guide for Nuclear Medicine Applications During the COVID-19 Outbreak." Molecular Imaging and Radionuclide Therapy **29**(2): 49-58.

A viral pneumonia rapidly spread from Wuhan, China to all countries in late 2019. In February 2020, WHO named as Coronavirus Disease 2019 (COVID-19) and declared the pandemic on March 11, 2020. To prevent the spread of COVID-19, Ministry of Health of Republic of Turkey and international institutions have published documents defining hygiene rules. After the lung computerized tomography (CT) findings which are important in the diagnosis of COVID-19 are described, protection measures against infection were defined in radiology departments. There is no publication involving protection measures for diagnostic and therapeutic procedures in nuclear medicine (NM) (appointment, patient acceptance, imaging and treatment procedures, disinfection etc). There are two reports on CT findings suggesting COVID-19 in F-18-fluorodeoxyglucose positron emission tomography/CT scan. These lung findings detected in hybrid images will be helpful in the early diagnosis of pulmonary involvement. Infected cases may be asymptomatic and can unintentionally disseminate the virus to surrounding people. This advisory guide has been prepared to avoid infection risk in NM clinics. During the COVID-19 outbreak, staff must use proper personal protective equipment and patients should be evaluated as the elective case according to clinical status. A questionnaire should be made for COVID-19. In cancer cases requiring urgent treatment, radionuclide treatment (RNT) should be planned according to the COVID-19 test result. If the result is negative, RNT can be applied; but if not or if the symptoms are present, RNT must be postponed. Following imaging procedures, scanners and room surfaces should be cleaned by personnel with proper disinfection training.

Azap, A. and F. S. Erdinc (2020). "Medical mask or N95 respirator: when and how to use?" Turkish Journal of Medical Sciences **50**: 633-637.

COVID-19 pandemic is now a global threat to human health reaching up to 2 million infected people all around the world. Since its first recognition in Wuhan, many topics were discussed intensively about COVID-19, both in the public and scientific community. Personal protective equipment, especially masks, has been among the hottest topics during this pandemic. Regardless of which mask is used, performing hand hygiene frequently with an alcohol-based hand rub or with soap and water if hands are dirty is the most effective preventive measure for COVID-19. The type of mask used when caring for COVID-19 patients will vary according to the setting, type of personnel/person, and activity. Although the main transmission route for COVID-19 is droplets, during aerosol generating procedures airborne transmission may occur. Keeping the distancing and medical masks and eye protection during close contact efficiently protects against respiratory diseases transmitted via droplets. Airborne precautions include goggles and respiratory protection with the use of an N95 or an equivalent mask respirator to prevent airborne transmission.

Azkur, A. K., et al. (2020). "Immune response to SARS-CoV-2 and mechanisms of immunopathological changes in COVID-19." Allergy **75**(7): 1564-1581.

As a zoonotic disease that has already spread globally to several million human beings and possibly to domestic and wild animals, eradication of coronavirus disease 2019 (COVID-19) appears practically impossible. There is a pressing need to improve our understanding of the immunology of this disease to contain the pandemic by developing vaccines and medicines for the prevention and treatment of patients. In this review, we aim to improve our understanding on the immune response and immunopathological changes in patients linked to deteriorating clinical conditions such as cytokine storm, acute respiratory distress syndrome, autopsy findings and changes in acute-phase reactants, and serum biochemistry in COVID-19. Similar to many other viral infections, asymptomatic disease is present in a significant but currently unknown fraction of the affected individuals. In the majority of the patients, a 1-week, self-limiting viral respiratory disease typically occurs, which ends with the development of neutralizing antiviral T cell and antibody immunity. The IgM-, IgA-, and IgG-type virus-specific antibodies levels are important measurements to predict population immunity against this disease and whether cross-reactivity with other coronaviruses is taking place. High viral load during the first infection and repeated exposure to virus especially in healthcare workers can be an important factor for severity of disease. It should be noted that many aspects of severe patients are unique to COVID-19 and are rarely observed in other respiratory viral infections, such as severe lymphopenia and eosinopenia, extensive pneumonia and lung tissue damage, a cytokine storm leading to acute respiratory distress syndrome, and multiorgan failure. Lymphopenia causes a defect in antiviral and immune regulatory immunity. At the same time, a cytokine storm starts with extensive activation of cytokine-secreting cells with innate and adaptive immune mechanisms both of which contribute to a poor prognosis. Elevated levels of acute-phase reactants and lymphopenia are early predictors of high disease severity. Prevention of development to severe disease, cytokine storm, acute respiratory distress syndrome, and novel approaches to prevent their development will be main routes for future research areas. As we learn to live amidst the virus, understanding the immunology of the disease can assist in containing the pandemic and in developing vaccines and medicines to prevent and treat individual patients.

Aznab, M. (2020). "Evaluation of COVID 19 infection in 279 cancer patients treated during a 90-day period in 2020 pandemic." International Journal of Clinical Oncology **25**(9): 1581-1586.

Background The aim of this study was investigation of COVID-19 disease and its outcome in cancer patients who needed treatment, in a 90-day period. Methods Cancer patient who required treatment, were evaluated for potential COVID-19 infection in a 90-day period, starting from beginning of this epidemic in Iran, January, to April 19, 2020. For treatment of solid tumor patients, if they did not have symptoms related to COVID-19, just chest X-ray was requested. If they showed COVID-19 related symptoms, high resolution CT scan of lungs was requested. For hematology cancer patients, PCR test for COVID-19 infection was requested as well. Protection measures were considered for personnel of oncology wards. Results 279 Patients were followed up in this 90-day period. No COVID-19 infection was observed in 92 cases of breast cancer, 14 cases of gastric cancer and 12 cases of pancreaticobiliary cancer. However, in 72 cases of colon cancer, 11 cases of lung cancer, 5 cases brain tumors and 12 cases ovarian cancer; 4 cases of COVID-19 were observed. In the hematology cancers group, which included 14 cases of Hodgkin's disease, 23 cases of lymphoproliferative disorder, 12 cases of acute leukemia and 12 cases of multiple myeloma; 3 cases of COVID-19 were observed. Conclusion Patients with cancer who need treatment can be treated by taking some measures. These measures include observing individual and collective protection principles in patients and health-care personnel, increasing patients' awareness particularly about self-care behavior, performing a COVID-19 test, and taking a chest X-ray, before the treatment starts

Ballard, D. H., et al. (2021). "Protection levels of N95-level respirator substitutes proposed during the COVID-19 pandemic: safety concerns and quantitative evaluation procedures." BMJ Open **11**(9).

Objective The COVID-19 pandemic has precipitated widespread shortages of filtering facepiece respirators (FFRs) and the creation and sharing of proposed substitutes (novel designs, repurposed materials) with limited testing against regulatory standards. We aimed to categorically test the efficacy and fit of potential N95 respirator substitutes using protocols that can be replicated in university laboratories. Setting Academic medical centre with occupational health-supervised fit testing along with laboratory studies. Participants Seven adult volunteers who passed quantitative fit testing for small-sized (n=2) and regular-sized (n=5) commercial N95 respirators. Methods Five open-source potential N95 respirator substitutes were evaluated and compared with commercial National Institute for Occupational Safety and Health (NIOSH)-approved N95 respirators as controls. Fit testing using the 7-minute standardised Occupational Safety and Health Administration fit test was performed. In addition, protocols that can be performed in university laboratories for materials testing (filtration efficiency, air resistance and fluid resistance) were developed to evaluate alternate filtration materials. Results Among five open-source, improvised substitutes evaluated in this study, only one (which included a commercial elastomeric mask and commercial HEPA filter) passed a standard quantitative fit test. The four alternative materials evaluated for filtration efficiency (67%-89%) failed to meet the 95% threshold at a face velocity (7.6 cm/s) equivalent to that of a NIOSH particle filtration test for the control N95 FFR. In addition, for all but one material, the small surface area of two 3D-printed substitutes resulted in air resistance that was above the maximum in the NIOSH standard. Conclusions Testing protocols such as those described here are essential to evaluate proposed improvised respiratory protection substitutes, and our testing platform could be replicated by teams with similar cross-disciplinary research capacity. Healthcare professionals should be cautious of claims associated with improvised respirators when suggested as FFR substitutes.

Ballatore, Z., et al. (2020). "Scientia Potentia Est: How the Italian World of Oncology Changes in the COVID-19 Pandemic." Jco Global Oncology **6**: 1017-1023.

PURPOSE After coronavirus disease 2019 (COVID-19) was declared a pandemic by the WHO, a response from the Italian Health System to react to an unprecedented condition became necessary and sudden. The COVID-19 pandemic has required oncologists to redefine clinical organization and patient management. The purpose of our study was to document the difficulties emerging during the SARS-CoV-2 pandemic in Italian oncology. METHODS We broadcasted an electronic survey to oncologic health care professionals. It consisted of 45 questions ranging from individual perception of pandemic management by hospital centers to physicians' and nurses' psychological distress and patient care. RESULTS A total of 383 oncology health workers participated in the survey. The majority were female (71.8%) and from central Italy (46.2%). Impressively, a total of 357 (93%) participants declared the oncologic department reorganized routine clinical activity, but only 40.5% were adequately trained about the required procedures; 20% of the survey respondents think they have not received adequate and timely protective devices. CONCLUSION Our survey demonstrated the flexibility of oncologic teams. However, the emergency response quality has been heterogeneous, and several drawbacks have emerged from the first analyses investigating how the world of oncology changes in the COVID-19 pandemic. Information, protection, testing, and training of health care professionals are key words that should be kept in mind to encourage recovery after this tragedy and to be ready to face a similar emergency in the future. (c) by American Society of Clinical Oncology

Barbieri, P. G. (2021). "Biological risk from SARS-CoV-2 and respiratory protection device for health workers during the first pandemic phase: a scenario that had to be avoided." Epidemiologia & Prevenzione **45**(1-2): 110-116.

In Italy, the SARS-CoV-2 pandemic showed the devastating consequences of a widespread biological risk not only for the general population, but also for healthcare workers that diagnose COVID-19 and treat patients. In the set of preventive measures taken to reduce this contagion, a fundamental role in personal protection is played by equipment suitable for preventing the contaminated air inhalation. Despite this severe biological risk during the first epidemic phase, some institutional operating protocols and recommendations have shown limitations and contradictions and, therefore, they must not be repeated. It was observed a widespread inadequate use of respiratory protections, such as surgical masks, which show a low efficacy for health workers; adequate respiratory protective devices are instead rarely used, while their use should have been guaranteed more extensively than it is recommended and noticed, according to the current work hygiene legislation.

Basavaraja, G. V., et al. (2021). "IAP Guideline on Practicing Safely During COVID-19 Era: Clinics and Small Establishments." Indian Pediatrics **58**(4): 383-390.

Justification The unprecedented COVID-19 pandemic has had a formidable impact on Indian health care. With no sight of its end as yet, various establishments including the smaller clinics and nursing homes are restarting full operations. Hence, there is the need for recommendations to allow safe practice ensuring the safety of both the heath care worker (HCW) and patients. Process Indian Academy of Pediatrics organized an online meeting of subject experts on 27 July, 2020. A committee was formed comprising of pediatricians, pediatric and neonatal intensivists, and hospital administrators. The committee held deliberations (online and via emails) and a final consensus was reached by November, 2020. Objectives To develop recommendations to provide a safe and practical healthcare facility at clinics and small establishments during COVID times. Recommendations The key recommendation to practise safely in this setting are enumerated. Firstly, organizing the out-patient department (OPD). Secondly, appropriate personal protective equipment (PPE) to provide protection to the individual. Thirdly, decontamination/disinfection of various common surfaces and equipment to prevent transmission of infection from fomites. Next, maintaining the heating ventilation and air conditioning (HVAC) to provide a stress-free, comfortable, and safe environment for patients and HCWs. Finally, steps to effectively manage COVID-19 exposures in a non-COVID-19 facility. All these measures will ensure safe practice during these unprecedent times in clinics and smaller establishments.

Bassan, S. (2020). "Data privacy considerations for telehealth consumers amid COVID-19." Journal of Law and the Biosciences **7**(1).

The COVID-19 emergency poses particularly high infection risks in a clinical setting, where patients and health care providers are placed in the same room. Due to these risks, patients are encouraged to avoid clinics and instead use Telemedicine for safer consultations and diagnoses. In March, the Office for Civil Rights (OCR) at the U.S. Department for Health and Human Services (HHS) issued a notice titled Notification of Enforcement Discretion for Telehealth Remote Communications During the COVID-19 Nationwide Public Health Emergency (the 'Notification'). The Notification relaxes the enforcement of privacy and security safeguards established by the Health Insurance Portability and Accountability Act (HIPAA) until further notice, in order to facilitate the transition to telehealth services for the broader purpose of promoting public health during the pandemic. Specifically, covered healthcare providers can use telehealth to provide all services that, in their professional judgment, they believe can be provided through telehealth. If providers make good faith efforts to provide the most timely and accessible care possible, they will not be subject to penalties for breaching the HIPAA Privacy, Security, and Breach Notification Rules. This paper examines the implications of the Notification on patients' health information privacy. It recommends that patients should undertake a careful reading of provider privacy policies to make sure their protected health information (PHI) is not at risk before switching to telehealth consultation. Acknowledging the limitations of patient self-protection from bad privacy practices when in need for medical treatment during pandemic, the paper proposes that consumers' data privacy should be protected through one of two alternative regulatory interventions: the FTC's authority under 5, or HIPAA's business associates agreements.

Behera, D., et al. (2020). "Protecting Indian health workforce during the COVID-19 pandemic." Journal of Family Medicine and Primary Care **9**(9): 4541-4546.

Rapidly growing rate of infection among health workers during the current COVID-19 pandemic, is posing a serious challenge to global health systems. Lately, India is also witnessing an intensifying COVID-19 disease burden and its impact on health workers. This paper aims to discuss the challenges to health worker protection in India and the possible ways forward. Given the inadequate and unequally distributed healthcare workforce, it is highly essential for the country to strategize prompt measures for ensuring occupational health and safety of its health workers. Information for this paper were gathered by searching PubMed and Google Scholar databases using "COVID-19", "Infection Control", "Health worker", "India" as search keywords in different combinations. In addition, websites of Government of India, relevant UN agencies and leading news agencies were also searched manually for related reports and publications. India must take timely measures in rapid manufacturing and procurement of essential personal protective equipment (PPE) to ensure adequate stockpiling to meet the rising demands. Comprehensive and repeated training with sharply focussed content including usage of PPE kits as well as active surveillance of adherence to recommended protocol are critical in protecting health workers especially the primary care physicians and frontline health staff from the deadly COVID-19 infection. The provision of psychological and financial support for health workers and their families is absolutely critical in building trust and dedicated work efforts by the health workforce for a continuous fight against the deadly disease.

Benzian, H. and R. Niederman (2020). "A Dental Response to the COVID-19 Pandemic-Safer Aerosol-Free Emergent (SAFER) Dentistry." Frontiers in Medicine **7**.

Dental services are significantly impacted by the COVID-19 pandemic. Almost all dental procedures carry a high infection risk for providers and patients due to the spread of aerosols. As a consequence, public health agencies and professional associations have issued guidelines for enhanced infection control and personal protection equipment and have also limited care to urgent or emergency services. However, there is no dental service concept for pandemic disaster preparedness or response that might be applied. Moreover, pathways to dental care provision in a post-pandemic future with persisting risks are needed. We proposeSafer Aerosol-Free Emergent Dentistry(SAFER Dentistry) as one approach to dental services during and emerging from the pandemic. The concept's starting point is the identification of the most common patient needs. The next step is to replace common treatments addressing the most frequent needs with alternative interventions involving a lower infection risk because they do not generate aerosols. SAFER Dentistry is innovative, avoids risk, and responds to the requirements of a pandemic and post-pandemic emergency where the risk of airborne disease transmission remains high. SAFER Dentistry thereby ensures continuity of dental services while protecting providers and patients from infectious pathogens. Moreover, SAFER Dentistry allows dental service providers to remain operational and generate income even under pandemic conditions. Potential implementation and policy options for SAFER Dentistry include universal availability without co-payments by patients and a uniform bundled payment scheme for providers to simplify budgeting, reimbursement, and administration during a pandemic. Adaptations and adjustments of the concept are possible and encouraged as long as the principle of avoiding aerosol-generating procedures is maintained.

Beskow, A. F., et al. (2020). "CoViD-19 Pandemic and Bariatric Surgery in Argentina." Obesity Surgery **30**(12): 5170-5176.

Argentina was able to anticipate public health interventions in order to flatten the contagion curve of CoViD-19. Eighty-three surgeons answered an online survey to assess the impact of the pandemic on bariatric surgery (BS) in Argentina. Most of them showed a high economic dependence on BS. Near 90% of health institutions were on phase 0 or I. While 90% still performed other laparoscopic surgeries, BS was suspended. In many surgeries for nonsuspected CoViD-19 patients, high personnel protection resources were applied. Ninety-five percent offered virtual consults. Most surgeons would not change usual algorithms or techniques. To restart BS a scientific society recommendation was expected, including patient selection criteria. The opinions gathered by this survey were taken into account to elaborate official recommendations for restarting elective BS.

Blumberg, T. J., et al. (2020). "Universal Screening for COVID-19 in Children Undergoing Orthopaedic Surgery: A Multicenter Report." Journal of Pediatric Orthopaedics **40**(10): E990-E993.

Background: The COVID-19 pandemic has substantially altered the typical process around performing surgery to ensure protection of health care workers, patients, and their families. One safety precaution has been the implementation of universal preoperative screening for severe acute respiratory syndrome coronavirus 2 (SARS-CoV-2). This study examines the results of universal screening on children undergoing orthopaedic surgery. Methods: This is a retrospective cohort study evaluating the incidence and symptomatology of COVID-19 in all patients presenting for orthopaedic surgery at 3 pediatric tertiary care children's hospitals during the COVID-19 pandemic (March to June 2020). All patients underwent universal screening with a nasopharyngeal swab to detect presence of SARS-CoV-2. Bivariate and multivariate logistic regression analysis was performed to identify risk factors for positive COVID-19 screening. Results: In total, 1198 patients underwent preoperative screening across all 3 institutions and 7 (0.58%) had detection of SARS-CoV-2. The majority of patients (1/7, 86%) were asymptomatic. Patients that tested positive were significantly more likely to be Hispanic (P=0.046) and had greater number of medical comorbidities (P=0.013), as scored on the American Society of Anesthesiologists (ASA) physical status score. A known COVID-19 positive contact was found to be a significant risk factor in the multivariate analysis (P=0.004). Conclusions: Early results of universal preoperative screening for COVID-19 demonstrates a low incidence and high rate of asymptomatic patients. Health care professionals, especially those at higher risk for the virus, should be aware of the challenges related to screening based solely on symptoms or travel history and consider universal screening for patients undergoing elective surgery.

Bonde, S. S., et al. (2021). "Risk & Hazards of COVID 19 Pandemic on Occupational Professionals." Journal of Pharmaceutical Research International **33**(35B): 79-85.

Coronavirus outbreak 2019 causes health-care employees an occupational safety concern. Several thousand health-care workers have been contaminated already. There are various reasons where, various occupational individual expose to the various types of the infections in the surrounding. The infections then have chances to spread in the community via these professionals. World Health Organization therefore has developed some of the Ideal precautionary measures to tackle or overcome this kind of the spreading of such infections. Therefore it is a concern to avoid intra-hospital spread of the communicable disease. Based on the Patient Safety Model Systems Technology Framework, approaches and interventions to protect healthcare workers in an emergency tertiary hospital are defined. In the fields of job activities, technology and equipment, environmental causes of job and organizational conditions. Faced with a possible pandemic, the concept of zero workplace infection remains an ambitious target that all health-care programs must aspire for. Various Technology and Equipment are therefore mandatory to provide to health care individuals, such as, Face mask, Hand gloves, face shield, Personal Protection Kit (PPE), hand sanitizers etc. Also awareness talks from the health care professionals are podcasted in different radio channels, news stations are continuously been published, Also the hospital waste management has primary role to dump out the hospital waste at given professional manner.

Bonenti, D. (2021). "The personality profile of the civil defense volunteer in the footsteps of a patron saint. Research on the Apostle Barnabas." Rivista Di Psicologia Dell Emergenza E Dell Assistenza Umanitaria(26): 52-58.

The author, former director of the social and health service of the Sondrio ASL (Local Health Authority) and first president of the association Psicologi per i Popoli - Lombardia, returned some time ago to his native village of Bondo in the prov-ince of Trento. During the long months of lockdown for Covid-19, he became inter-ested in researching the patron saint of his native village. We are talking about St Barnabas, Apostle, who is celebrated every year in the 11th June village festival, but whose historical and biographical features have been lost over time. Through painstaking research into the apostle Barnabas, the author captured the personal-ity traits that should characterise volunteers, including psychologists, in their com-plex and delicate role in emergency situations. Who, if not Barnabas the Apostle, the author wonders, could be the patron saint of Civil Protection volunteers? The work presented here has the merit of focusing attention also on the necessary processes of selection, training and employment of professional personnel called upon to work as volunteers in emergency situations.

Bordea, I. R., et al. (2021). "Impact of COVID-19 Pandemic on Healthcare Professionals and Oral Care Operational Services: A Systemic Review." Risk Management and Healthcare Policy **14**: 453-463.

The unprecedented climate of the COVID-19 pandemic has some restrictions on oral care operational services, which heavily impacted the delivery of aerosol generating procedures (AGPs). This led the health authorities to set up guidelines and policies that should be followed to minimize the virus spread and ensure safe and effective dental care delivery. This systemic review aimed to evaluate the current guidelines and strategies in providing safe dental services and ensuring efficacy of the current universal personal preventive and protective measures, as well the impact that this outbreak might have on practicing the dental profession in full scope. The review focus questions were as follows: are the current guidelines and measures in literature mitigated enough to ensure safe and effective oral care delivery to patients during the COVID-19 pandemic? Is it possible to highlight the essential and fundamental cross-infection control measures and policies? Twenty articles were chosen out of 180,248 after the inclusion and exclusion criteria were applied in the period between January 1st and August 1st 2020. Our results highlighted effective safety measures that can be implanted in protecting healthcare professionals and patients and ensuring optimal dental care delivery in a safe and healthy environment, taking into consideration the risk assessment and management of AGPs. In conclusion, informative updated standardized policies and protocols are required when more knowledge on the virus behavior keeps evolving. Moreover, there is a need for evidence-based protective measures to be modified on a regular basis to be followed, until a safe vaccine is produced.

Botti, S., et al. (2021). "Hematology Patient Protection During the COVID-19 Pandemic in Italy: A Nationwide Nursing Survey." Mediterranean Journal of Hematology and Infectious Diseases **13**.

Background: Italy has been one of the first European countries hit by the COVID-19 pandemic, with many patients dying from severe respiratory issues, especially frail subjects. Hematology patients are generally thought to be at high risk of developing severe COVID-19-associated complications. The aim of this work was to describe the infection control measures adopted in Italian hematology settings to protect patients and health-care professionals. Materials and Methods: On behalf of the Nursing Campus in Hematology Group, a nationwide nursing survey was conducted. Questionnaire items included general information, infection control measures, patient and health-care professional protection, information management, and participants' opinion on critical issues. Data have been analyzed by center location (Northern, Central, or Southern Italy) and by patient age (adult vs pediatric). Results: Forty-four Italian hematology centers participated, representing 52.4% of those invited. Patients underwent nasopharyngeal swabs (93.2%) generally the day before admission (43.2%), though less frequently in Southern centers (p = 0.0377). Visitor restrictions were implemented in all centers: 65.9% barred all visitors, while 25.0% allowed visitors only for patients with specific conditions, especially in Central Italy. Deficiency of personal protective equipment, including masks (45.5%) and gloves (22.7%), was reported, although the nurses' opinion was that the emergency was nevertheless well managed to protect patients and professionals. Almost all healthcare institutions (97.7%) provided recommendations on emergency management. No significant differences were found between adult and pediatric centers in terms of infection prevention and control. Discussion: Low variability in patient protection strategies was observed, meaning that national recommendations were effective. However, some critical issues emerged regarding the management of infected health-care professionals and their contacts.

Braude, D., et al. (2021). "Safety of air medical transport of patients with COVID-19 by personnel using routine personal protective equipment." Journal of the American College of Emergency Physicians Open **2**(2).

Objective Air medical transport of patients with known or suspected coronavirus disease 2019 (COVID-19) likely represents a high-risk exposure to crew members as aircraft cabins are quite small resulting in close personal contact. The actual risk to medical crew members is not known. Methods We conducted an institutional review board-exempt, retrospective study of air medical transport of patients with known or suspected COVID-19 by 8 programs in the Four Corners Region to determine the number of symptomatic COVID-19 among air medical crew members compared to total exposure time. All programs used similar routine personal protective equipment (PPE), including N-95 masks and eye protection. Total exposure time was considered from time of first patient contact until handoff at a receiving hospital. Results There were 616 air transports: 62% by fixed-wing and 38% by rotor-wing aircraft between March 15 and September 6, 2020. Among transported patients, 407 (66%) were confirmed COVID+ and 209 (34%) were under investigation. Patient contact time ranged from 38 to 432 minutes with an average of 140 minutes. The total exposure time for medical crew was 2924 hours; exposure time to confirmed COVID+ patients was 2008 hours. Only 30% of patients were intubated, and the remainder had no oxygen (8%), low-flow nasal cannula (42%), mask (11%), high-flow nasal cannula (4.5%), and continuous positive airway pressure or bilevel positive airway pressure (3.5%). Two flight crew members out of 108 developed COVID that was presumed related to work. Conclusions Air medical transport of patients with known or suspected COVID-19 using routine PPE is considered effective for protecting medical crew members, even when patients are not intubated. This has implications for health care personnel in any setting that involves care of patients with COVID-19 in similarly confined spaces.

Bressington, D. T., et al. (2020). "Association Between Depression, Health Beliefs, and Face Mask Use During the COVID-19 Pandemic." Frontiers in Psychiatry **11**.

The 2019 novel coronavirus (COVID-19) pandemic is associated with increases in psychiatric morbidity, including depression. It is unclear if people with depressive symptoms understand or apply COVID-19 information differently to the general population. Therefore, this study aimed to examine associations between depression, health beliefs, and face mask use during the COVID-19 pandemic among the general population in Hong Kong. This study gathered data from 11,072 Hong Kong adults via an online survey. Respondents self-reported their demographic characteristics, depressive symptoms (PHQ-9), face mask use, and health beliefs about COVID-19. Hierarchical logistic regression was used to identify independent variables associated with depression. The point-prevalence of probable depression was 46.5% (n = 5,150). Respondents reporting higher mask reuse (OR = 1.24, 95%CI 1.17-1.34), wearing masks for self-protection (OR = 1.03 95%CI 1.01-1.06), perceived high susceptibility (OR = 1.15, 95%CI 1.09-1.23), and high severity (OR = 1.33, 95%CI 1.28-1.37) were more likely to report depression. Depression was less likely in those with higher scores for cues to action (OR = 0.82, 95%CI 0.80-0.84), knowledge of COVID-19 (OR = 0.95, 95%CI 0.91-0.99), and self-efficacy to wear mask properly (OR = 0.90 95%CI 0.83-0.98). We identified a high point-prevalence of probable major depression and suicidal ideation during the COVID-19 outbreak in Hong Kong, but this should be viewed with caution due to the convenience sampling method employed. Future studies should recruit a representative probability sample in order to draw more reliable conclusions. The findings highlight that COVID-19 health information may be a protective factor of probable depression and suicidal ideation during the pandemic. Accurate and up-to-date health information should be disseminated to distressed and vulnerable subpopulations, perhaps using digital health technology, and social media platforms to prompt professional help-seeking behavior.

Brondani, M., et al. (2020). "A Pan-Canadian narrative review on the protocols for reopening dental services during the COVID-19 pandemic." BMC Oral Health **20**(1).

The current coronavirus disease 2019 (COVID-19) pandemic is impacting the way in which dental services are provided. The aim of this narrative review was twofold: to summarize key areas from the Canadian protocols available for the reopening and restructuring of dental services across the country and to critically review these protocols based on existing evidence. A narrative review of the existing Canadian protocols, written in English and French, was undertaken between April 15 and July 13, 2020. The protocols were obtained by searching through regulatory bodies and websites from professional organizations, and from personal contacts through academic institutions and policy leaders. The data extraction form focused only on protocols related to dentistry, and the information was compiled by a hired assistant. Content was categorized via group discussions with the research team on eight areas: office management and procedures, patient and staff screening, treatment procedures, office layout, risk reduction, personal protective equipment, supporting information, and length and readability. Thirteen protocols were identified and offered substantial variation in the level of details provided. All but two protocols specified proper donning/doffing of personal protective equipment, while all protocols recommended daily monitoring of COVID-19 related signs and symptoms in staff and patients. They varied in terms of recommended mask types, eye and face shield protection, and head coverings. While all protocols aimed at restructuring emergency dental services, their recommendations were often not based on the published evidence. This narrative review summarized key areas from 13 provincial and territorial protocols in Canada to help oral health care providers plan the reopening of their services. The information conveyed across all documents was clear, but variance highlights the need for a coordinated effort to develop an evidence-based document for dental practitioners.

Bukhari, N., et al. (2020). "Pharmacists at the frontline beating the COVID-19 pandemic." Journal of Pharmaceutical Policy and Practice **13**(1).

As the lockdowns are being observed all over the globe and the national level pharmacy professionals are performing frontline roles, this editorial highlights the role of pharmacists in the COVID - 19 pandemic. Pharmacists globally are providing services amidst pandemic, including TRIAGE services, seeing patients and reducing the patients' burden on health care facilities such as hospitals and GP practices. Pharmacists are also working to providing home deliveries, as well as dealing with the increasing number of patients coming through to pharmacies with the other ailments. Pharmacy associations have issued their guidelines and in this editorial, several global examples of pharmacists' role in the COVID 19 are being discussed. Pakistan is used as a country case study in this editorial. The editorial also elaborates how pharmacists in the UK and Pakistan have teamed up together to compile 10-steps protection guidelines for the pharmacy teams in Pakistan in English and Urdu language. This 10-point guidance educates community pharmacies for safety and standard operation as the number of patients in the country continues to rise. These guidelines are endorsed by the government and private bodies. These can be adopted and adapted by any country; keeping in view their laws and regulations.y

Campos, R. K., et al. (2020). "Decontamination of SARS-CoV-2 and Other RNA Viruses from N95 Level Meltblown Polypropylene Fabric Using Heat under Different Humidities." ACS Nano **14**(10): 14017-14025.

In March of 2020, the World Health Organization declared a pandemic of coronavirus disease 2019 (COVID-19), caused by the severe acute respiratory syndrome coronavirus 2 (SARS-CoV-2). The pandemic led to a shortage of N95-grade filtering facepiece respirators (FFRs), especially surgical-grade N95 FFRs for protection of healthcare professionals against airborne transmission of SARS-CoV-2. We and others have previously reported promising decontamination methods that may be applied to the recycling and reuse of FFRs. In this study we tested disinfection of three viruses, including SARS-CoV-2, dried on a piece of meltblown fabric, the principal component responsible for filtering of fine particles in N95-level FFRs, under a range of temperatures (60-95 degrees C) at ambient or 100% relative humidity (RH) in conjunction with filtration efficiency testing. We found that heat treatments of 75 degrees C for 30 min or 85 degrees C for 20 min at 100% RH resulted in efficient decontamination from the fabric of SARS-CoV-2, human coronavirus NL63 (HCoV-NL63), and another enveloped RNA virus, chikungunya virus vaccine strain 181/25 (CHIKV-181/25), without lowering the meltblown fabrics filtration efficiency.

Casaer, S., et al. (2020). "Organizing a safe operating room during a pandemic. What did we learn from COVID-19?" Acta Anaesthesiologica Belgica **71**(3): 111-121.

During the COVID-19 pandemic, multiple guidelines have been issued on hospital safety and protection measures to prevent transmission to healthcare workers and to other patients. The operating room is a high-risk environment where enhanced precautions are required. The guidelines differ and practical implementation between hospitals can also vary, according to interpretation and budget. Staff at risk may question if the local policies are sufficient and correct. This article provides an overview and theoretical background to the additional safety measures required in the operating room during a viral pandemic like the COVID-19 pandemic. This may serve as a touchstone and tool for anesthetists and OR managers.

Catton, H. (2021). "COVID-19: The future of nursing will determine the fate of our health services." International Nursing Review **68**(1): 9-11.

Preparing the world to manage future pandemics must take priority. It is clear that we were not prepared for the COVID-19 pandemic which continues to cause great suffering around the world. Nurses and other health professionals everywhere must be involved in health policy planning and implementation of public health measures to combat this and future pandemics. Such preparation needs community policy involvement at grassroots levels and needs to be collaboratively instigated at international levels. The death so far of over 2000 nurses is unacceptable in this pandemic, and we need to better protect and sustain the workforce. The International Council of Nurses has been instrumental in data gathering of nurses' experiences during COVID-19. Key points from analysis of this data have been included in Second Progress Report of the World Health Organization's Independent Panel for Pandemic Preparedness and Response. This paper summarises the key messages from this report, as well as the nursing shortage. The International Council of Nurses resounds the call for massive investment in nursing education, leadership and jobs, as well as protection for our nurses on the frontlines of the pandemic.

Cepeda, M., et al. (2020). "Strategies and recommendations for urolithiasis treatment and follow-up in COVID-19 pandemia." Archivos Espanoles De Urologia **73**(5): 438-446.

INTRODUCTION: The health crisis caused by COVID-19 pandemic has led to a restructuring of urological activity in order not to delay priority situations. An important port to prioritize within Urology is Urolithiasis. The objective of this article is to establish strategies and recommendations for the treatment and follow-up in COVID-19 pandemic in phases I, II and III, based on available scientific evidence and the consensus of a group of experts in these pathologies. MATERIAL AND METHODS: The document is based on the evidence available in the literature so far on SARSCoV-2 and the experience of the authors in the management of COVID-19 in their institutions. A narrative review of the literature was conducted, and a modified nominal group technique was used due to the extraordinary restrictions of assembly and mobility during the pandemic. RESULTS: Recommendations are made regarding the epidemiological evaluation of patients before surgery, the management of positive patients, the epidemiological measures for healthcare personnel, the management of renal colic, the type of anesthesia, endourological surgery, shockwave lithotripsy, hospitalization, clinical tests, out-patient service and priorities on the surgical waiting list. CONCLUSION: Treatment of Urolithiasis in COVID-19 pandemic calls for prioritization of patients, maximum efficiency in treatments, adequate protection of healthcare personnel, and the implementation of telemedicine as a measure to reduce patient attendance to the hospital.

Cernigliaro, M., et al. (2021). "Observational study on healthcare workers protection in the angiographic suite during the SARS-CoV-2 pandemic: before and during vax era." Journal of Public Health Research **10**(4).

Background: Since the first case of Coronavirus Disease 2019 (COVID-19) in Italy, all the hospital facilities had to reform their daily activities. Amidst them, the Interventional Radiology Department in the "Azienda Ospedaliera Universitaria" of Novara (Italy) had to create a dedicated protocol for the patent's management during the pandemic. Design and Methods: The time interval between February 2020 and March 2021 was divided into three different periods and we reported the evolution of our safety protocol, the changes in our daily activities and the rates of SARS-CoV-2 infection among the healthcare workers (HCW) of the Angiographic Suite. Personnel who had positive partners/family members or who had established close contacts of another nature outside the workplace were excluded from the study, in order to reduce any bias. Results: A total of 35 HCWs served in 355 patient procedures on SARS-CoV-2 positive patients from February 2020 to March 2021. During the year there was a reduction in the morbidity rate first from 7.9% to 1.4% and then currently reaching 0%. Conclusions: Dedicated routes, elevators, establishing filter areas and a clear demarcation between clean and contaminated areas, dressing and undressing procedures, cleaning procedures and the obligation to always wear a surgical mask during the working shift are essential to prevent in-hospital infection. The vaccines' arrival seems to further reduce the risk for healthcare workers, but it is still necessary to take docile precautions in view of the new mutations of the virus.

Cesario, C. D., et al. (2021). "Handmade face mask can be sterilized without compromising their efficacy." Revista De Epidemiologia E Controle De Infeccao **11**(3): 1-11.

Background and Objectives: COVID-19 caused by SARS-CoV-2 is transmitted by contact, droplets and aerosols. The World Health Organization recommends the mandatory use of surgical masks for health professionals and encourages countries to adhere to their use, en masse, in order to minimize the transmission of the virus. Since the lack of this personal protective equipment is a cause for concern, the objective of this study was to evaluate the characteristics of facial masks regarding (i) the mechanical properties of elongation, rupture stress and resistance to air passage and (ii) the morphometric properties of thickness, fiber diameter and pore distribution after autoclave sterilization. Methods: The masks were made in TNT Spunbonded (100% polypropylene, 60 g/m(2)), autoclaved at 70 degrees C for 5 minutes and stored in plastic films at room temperature. Then, the mechanical properties were determined in an Instron universal testing machine and Gurley type automatic densimeter and the morphometric properties in a semi-automatic thickness gauge and scanning electron microscope. Results: It was observed that a cycle of sterilization of facial masks, by moist heat under pressure, increased the blanket's stiffness, did not produce physical damage and did not reduce the blanket's barrier capacity. Conclusion: The sterilization of face masks made of TNT can be considered a strategy to increase safety in their production and use.

Chang, C. T., et al. (2021). "Filtering Facepiece Respirators for Healthcare Workers Protection in the Time of COVID-19 Pandemic." Malaysian Journal of Medical Sciences **28**(3): 151-154.

Protecting healthcare workers (HCWs) who are in the frontline during the time of the COVID-19 pandemic is paramount. The filtering facepiece respirators (FFRs) or N95 respirator is one of the personal protective equipment (PPE) used to protect HCWs exposed to airborne pathogens in clinical practice or when performing aerosol generating procedures. The FFRs should be able to serve the intended purpose without causing additional health and safety hazards for the HCWs. The following commentary will provide some basic information on selecting correct FFRs and conducting fitness test.

Chang, T. C., et al. (2020). "The First 100 Days: Establishment and Effectiveness of Campus Protection Measures at a College during the COVID-19 Pandemic." Healthcare **8**(3).

To prevent transmission of the coronavirus, we established the campus protection measures for coronavirus disease 2019 (COVID-19) (CPMCV-19) and analyzed the effectiveness and cost in practice. This project was set in Taiwan. We organized an anti-epidemic task force team from multidisciplinary co-workers to establish the CPMCV-19. The essential components were as follows: no close contact communication, sterilization, temperature control, social distancing, activity restrictions, personal hygiene control, and situational awareness. During 100 days of operation, the mean time spent for frontal temperature measuring was 2.7 +/- 0.3 s per person. The mean on-duty time for individual personnel to control the gate and measure temperature was 3.5 h per day. In total, 31 persons with loss of taste/smell or fever were detected on campus and sent to hospital for screening within 1 h. A total of 6 persons were instructed to observe self-health management due to possible contact or travel history, and none were diagnosed with COVID-19 infection. A total budget of USD 27,100 was used for CMPCV-19 in this period. The established campus protection measures for COVID-19 were practical and might be effective. They can be used as reference for schools in a pandemic, such as COVID-19.

Chau, J. P. C., et al. (2021). "Nurses' experiences of caring for people with COVID-19 in Hong Kong: a qualitative enquiry." BMJ Open **11**(8).

Objectives Nurses are the largest group of healthcare workers on the front line of efforts to control the COVID-19 pandemic. An understanding of their nursing experiences, the challenges they encountered and the strategies they used to address them may inform efforts to better prepare and support nurses and public health measures when facing a resurgence of COVID-19 or new pandemics. This study aimed to explore the experiences of nurses caring for people with suspected or diagnosed COVID-19 in Hong Kong. Design A qualitative study was conducted using individual, semistructured interviews. All interviews were audio-recorded and transcribed verbatim for thematic analysis. Setting Participants were recruited from acute hospitals and a public health department in Hong Kong from June 2020 to August 2020. Participants A purposive sample of registered nurses (N=39) caring for people with COVID-19 in Hong Kong were recruited. Results Two-thirds of the nurses had a master's degree and over a third had 6-10 years of nursing experience. Around 40% of the nurses cared for people with COVID-19 in isolation wards and a quarter performed COVID-19-related work for 31-40 hours/week. Most (90%) had training in COVID-19 and three-quarters had experience of working in infection control teams. Six key themes emerged: confronting resource shortages; changes in usual nursing responsibilities and care modes; maintaining physical and mental health; need for effective and timely responses from relevant local authorities; role of the community in public health protection and management; and advanced pandemic preparedness. Conclusions Our study found that nurses possessed resilience, self-care and adaptability when confronting resource shortages, changing nursing protocols, and physical and mental health threats during the COVID-19 pandemic. However, coordinated support from the clinical environment, local authorities and community, and advanced preparedness would likely improve nursing responses to future pandemics.

Chukwuorji, J. C. and S. K. Iorfa (2020). "Commentary on the Coronavirus Pandemic: Nigeria." Psychological Trauma-Theory Research Practice and Policy **12**: S188-S190.

About a week after the confirmation of Nigeria's index case of COVID-19 on February 27, 2020, the Nigerian federal government set up a 12-member Presidential Task Force for the Control of the Coronavirus. The country's borders were closed on March 23. and the lockdown of cities was also implemented. The unanticipated disruption of scholarly or professional advancement for the 94% of university students who are not currently learning may increase the burden of mental illness among these students and predispose them to social vices. Two suicide deaths occurred during the lockdown. Poverty, lack of trust in the government, ignorance, denial and misplaced religious zealotry negatively impact on the behavior of Nigerians. Fear-induced behavioral changes such as consuming lemon, ginger, garlic, local herbs, and other substances for protection have also become rampant. Loss of income due to the lockdown and the accompanying destitution can also be a pathfinder for numerous other sicknesses and deaths. For the benefit of enhanced prevention and control of infection, only government-funded hospitals and a few designated privately owned hospitals have been permitted to provide continued services at this time. The number of shifts in these hospitals were adjusted such that there are fewer health care professionals in the hospital at a given time. This strategy has inhibited treatment and care for certain groups of people who are not COVID-19 patients. Efforts are being made to develop telehealth services, but most rural residents may not benefit from them.

Ciniselli, C. M., et al. (2021). "SARS-CoV-2 Serology Monitoring of a Cancer Center Staff in the Pandemic Most Infected Italian Region." Cancers **13**(5).

Simple Summary Since the beginning of the COVID-19 outbreak, Cancer Centers adopted specific procedures to protect patients as well as to monitor the possible spread of SARS-CoV-2 among healthcare personnel. In April 2020, we implemented a prospective longitudinal study aimed at monitoring the serological response to SARS-Cov-2 in the healthcare personnel of a Comprehensive Cancer Center identified by the Lombardy region (the Italian region most affected by the COVID-19 pandemic) as one of the three oncologic hubs where the Regional Health Authorities referred all the cancer patients in the region. We identified a fraction of healthcare personnel with long-term anti-SARS-CoV-2 antibody response, though negative for viral RNA, who could safely approach fragile cancer patients. Since the beginning of the COVID-19 outbreak, Cancer Centers adopted specific procedures both to protect patients and to monitor the possible spread of SARS-CoV-2 among healthcare personnel (HCP). In April 2020 at Fondazione IRCCS Istituto Nazionale dei Tumori, Milano, one of the three oncologic hubs in Lombardy where the Health Regional Authorities referred all the cancer patients of the region, we implemented a prospective longitudinal study aimed at monitoring the serological response to SARS-Cov-2 in HCP. One hundred and ten HCP answered a questionnaire and were screened by nasopharyngeal swabs as well as for IgM/IgG levels; seropositive HCPs were further screened every 40-45 days using SARS-CoV-2-specific serology. We identified a fraction of HCP with long-term anti-SARS-CoV-2 antibody responses, though negative for viral RNA, and thus probably able to safely approach fragile cancer patients. Monitoring asymptomatic HCP might provide useful information to organize the healthcare service in a Cancer Center, while waiting for the effectiveness of the active immunization by SARS-CoV-2 vaccines, which will provide protection from infection.

Cocarta, D. M., et al. (2021). "Indoor Air Pollution with Fine Particles and Implications for Workers' Health in Dental Offices: A Brief Review." Sustainability **13**(2).

(1) Background: Indoor air pollution can affect the well-being and health of humans. Sources of indoor pollution with particulate matter (PM) are outdoor particles and indoor causes, such as construction materials, the use of cleaning products, air fresheners, heating, cooking, and smoking activities. In 2017, according to the Global Burden of Disease study, 1.6 million people died prematurely because of indoor air pollution. The health effects of outdoor exposure to PM have been the subject of both research and regulatory action, and indoor exposure to fine particles is gaining more and more attention as a potential source of adverse health effects. Moreover, in critical situations such as the current pandemic crisis, to protect the health of the population, patients, and staff in all areas of society (particularly in indoor environments, where there are vulnerable groups, such as people who have pre-existing lung conditions, patients, elderly people, and healthcare professionals such as dental practitioners), there is an urgent need to improve long- and short-term health. Exposure to aerosols and splatter contaminated with bacteria, viruses, and blood produced during dental procedures performed on patients rarely leads to the transmission of infectious agents between patients and dental health care staff if infection prevention procedures are strictly followed. On the other hand, in the current circumstances of the pandemic crisis, dental practitioners could have an occupational risk of acquiring coronavirus disease as they may treat asymptomatic and minimally symptomatic patients. Consequently, an increased risk of SARS-CoV-2 infection could occur in dental offices, both for staff that provide dental healthcare and for other patients, considering that many dental procedures produce droplets and dental aerosols, which carry an infectious virus such as SARS-CoV-2. (2) Types of studies reviewed and applied methodology: The current work provides a critical review and evaluation, as well as perspectives concerning previous studies on health risks of indoor exposure to PM in dental offices. The authors reviewed representative dental medicine literature focused on sources of indoor PM10 and PM2.5 (particles for which the aerodynamic diameter size is respectively less than 10 and 2.5 mu m) in indoor spaces (paying specific attention to dental offices) and their characteristics and toxicological effects in indoor microenvironments. The authors also reviewed representative studies on relations between the indoor air quality and harmful effects, as well as studies on possible indoor viral infections acquired through airborne and droplet transmission. The method employed for the research illustrated in the current paper involved a desk study of documents and records relating to occupational health problems among dental health care providers. In this way, it obtained background information on both the main potential hazards in dentistry and infection risks from aerosol transmission within dental offices. Reviewing this kind of information, especially that relating to bioaerosols, is critical for minimizing the risk to dental staff and patients, particularly when new recommendations for COVID-19 risk reduction for the dental health professional community and patients attending dental clinics are strongly needed. (3) Results: The investigated studies and reports obtained from the medical literature showed that, even if there are a wide number of studies on indoor human exposure to fine particles and health effects, more deep research and specific studies on indoor air pollution with fine particles and implications for workers' health in dental offices are needed. As dental practices are at a higher risk for hazardous indoor air because of exposure to chemicals and microbes, the occupational exposures and diseases must be addressed, with special attention being paid to the dental staff. The literature also documents that exposure to fine particles in dental offices can be minimized by putting prevention into practice (personal protection barriers such as masks, gloves, and safety eyeglasses) and also keeping indoor air clean (e.g., high-volume evacuation, the use of an air-room-cleaning system with high-efficiency particulate filters, and regularly maintaining the air-conditioning and ventilation systems). These kinds of considerations are extremely important as the impact of indoor pollution on human health is no longer an individual issue, with its connections representing a future part of sustainability which is currently being redefined. These kinds of considerations are extremely important, and the authors believe that a better situation in dentistry needs to be developed, with researchers in materials and dental health trying to understand and explain the impact of indoor pollution on human health.

Colindres, C., et al. (2021). "Migrant Agricultural Workers' Health, Safety and Access to Protections: A Descriptive Survey Identifying Structural Gaps and Vulnerabilities in the Interior of British Columbia, Canada." International Journal of Environmental Research and Public Health **18**(7).

In this paper, we provide descriptive data that characterize the health, safety, and social care environment of migrant agricultural workers in British Columbia, Canada. Through the administration of surveys (n = 179), we gathered information in three domains: (1) living and working conditions; (2) barriers to rights, health, safety and advocacy/reporting; (3) accessibility of services. Our study confirms what predominantly qualitative studies and Ontario-based survey data indicate in terms of health, legal, and social barriers to care and protection for this population. Our findings also highlight the prevalence of communication barriers and the limited degree of confidence in government authorities and contact with support organizations this population faces. Notably, survey respondents expressed a strong intention to report concerns/issues to authorities while simultaneously reporting that they lacked the knowledge to initiate such complaints. These findings call into question government responses that task the agricultural industry with addressing access and service gaps that may be more effectively addressed by government agencies and service providers. In order to improve supports and protections for migrant agricultural workers, policies and practices should be implemented that: (1) empower workers to independently access health, social, and legal protections and limit workers' dependence on their employers when help-seeking; (2) provide avenues for increased proactive inspections, anonymous reporting, alternative housing/employment and meaningful 2-way communication with regulators so that the burden of reporting is lessened for this workforce; (3) systematically address breaches in privacy, translation, and adequate workplace injury assessments in the healthcare system. Ultimately, the COVID-19 context has put into sharper focus the complex gaps in health, social and legal services and protections for migrant agricultural workers. The close chronology of our data collection with this event can help us understand the factors that have resulted in so much tragedy among this workforce.

Conlon, C., et al. (2021). "The impact of the COVID-19 pandemic on child health and the provision of Care in Paediatric Emergency Departments: a qualitative study of frontline emergency care staff." Bmc Health Services Research **21**(1).

BackgroundThe COVID-19 pandemic and subsequent public health guidance to reduce the spread of the disease have wide-reaching implications for children's health and wellbeing. Furthermore, paediatric emergency departments (EDs) have rapidly adapted provision of care in response to the pandemic. This qualitative study utilized insight from multidisciplinary frontline staff to understand 1) the changes in paediatric emergency healthcare utilization during COVID-19 2) the experiences of working within the restructured health system.MethodsFifteen semi-structured interviews were conducted with frontline staff working in two paediatric EDs and two mixed adult and children EDs. Participants included emergency medicine clinicians (n=5), nursing managerial staff (n=6), social workers (n=2) and nursing staff (n=2). Thematic Analysis (TA) was applied to the data to identify key themes.ResultsThe pandemic and public health restrictions have had an adverse impact on children's health and psychosocial wellbeing, compounded by difficulty in accessing primary and community services. The impact may have been more acute for children with disabilities and chronic health conditions and has raised child protection issues for vulnerable children. EDs have shown innovation and agility in the structural and operational changes they have implemented to continue to deliver care to children, however resource limitations and other challenges must be addressed to ensure high quality care delivery and protect the wellbeing of those tasked with delivering this care.ConclusionsThe spread of COVID-19 and subsequent policies to address the pandemic has had wide-reaching implications for children's health and wellbeing. The interruption to health and social care services is manifesting in myriad ways in the ED, such as a rise in psychosocial presentations. As the pandemic continues to progress, policy makers and service providers must ensure the continued provision of essential health and social services, including targeted responses for those with existing conditions.

Coyer, F., et al. (2020). "Maintaining skin health and integrity for staff wearing personal protective equipment for prolonged periods: a practical tip sheet." Wound Practice and Research **28**(2): 75-83.

Patient skin injuries associated with medical or therapeutic devices are increasingly reported in the literature. With the coronavirus (COVID-19) pandemic, healthcare staff are wearing personal protective equipment (PPE) for prolonged periods. Anecdotally, cases of healthcare workers' self report of injuries to their face, particularly the bridge of the nose, upper cheek, forehead and above the ears, as a result of extended use of eye protection and masks is increasing. Protecting the skin of frontline healthcare staff is as important as protecting patients' skin. The tip sheet presented in this paper provides staff with a guide in the form of written and visual assistance in order to reduce the risk of device-related pressure injuries (DRPIs) in healthcare staff caused by PPE.

Cui, S. S., et al. (2021). "Impact of COVID-19 on Anxiety, Stress, and Coping Styles in Nurses in Emergency Departments and Fever Clinics: A Cross-Sectional Survey." Risk Management and Healthcare Policy **14**: 585-594.

Background: During the Coronavirus Disease 2019 (COVID-19) pandemic, emergency departments and fever clinics nurses acted as gatekeepers to the health care system. To manage the psychological problems that these nurses experience, we should develop appropriate training and intervention programs. Objective: To identify the impact of COVID-19 on the psychology of Chinese nurses in emergency departments and fever clinics and to identify associated factors. Methods: This online cross-sectional study recruited participants through snowball sampling between 13 February and 20 February 2020. Nurses self-administered the online questionnaires, including a general information questionnaire, the Self-Rating Anxiety Scale, the Perceived Stress Scale-14, and the Simplified Coping Style Questionnaire. Results: We obtained 481 responses, of which 453 were valid, an effective response rate of 94.18%. Participants who had the following characteristics had more mental health problems: female gender, fear of infection among family members, regretting being a nurse, less rest time, more night shifts, having children, lack of confidence in fighting transmission, not having emergency protection training, and negative professional attitude. Conclusion: Effective measures are necessary to preserve mental health of nurses in emergency departments and fever clinics. These include strengthening protective training, reducing night shifts, ensuring adequate rest time, and timely updating the latest pandemic situation.

Cui, S. S., et al. (2020). "Experiences and Psychological Adjustments of Nurses Who Voluntarily Supported COVID-19 Patients in Hubei Province, China." Psychology Research and Behavior Management **13**: 1135-1145.

Background: The COVID-19 pandemic poses a major threat to global public health and economic development. Moreover, it has put considerable psychological pressure on nurses, who have played a vital role in the prevention and control of the epidemic. Objective: This qualitative study aimed at exploring the experiences and psychological adjustments of nurses who voluntarily traveled to Hubei Province in China to provide support during the COVID-19 epidemic. Methods: We conducted semi-structured, face-to-face interviews with twelve nurses recruited from three hospitals in Jiangsu Province and performed qualitative content analysis of the interview data. Results: The following themes emerged from the analysis: (1) motivations for supporting the hardest-hit areas (professional commitment, family support, and media propaganda); (2) challenges faced during the support missions (heavy workloads, changes in working patterns, communication barriers, and barriers associated with wearing personal protective equipment); (3) psychological experiences (a sense of uncertainty, fear of infection, loneliness, stressful events, and sleep disorders); (4) psychological adjustments (adequate training and personal protective equipment, positive responses to stress, and social support); and (5) personal and professional growth (a strong professional identity, a positive work attitude, a perception of expanded possibilities, realization of the value of learning, and cherishing life). Conclusions and Recommendations: Policy makers and nursing managers should implement effective measures for supporting nurses. They include ensuring adequate workforce preparedness for nurses, strengthening protection training, including professional psychologists in support teams, encouraging nurses to apply self-regulation methods, such as exercising and listening to music, and seeking social support to promote mental health.

Dabanch, J., et al. (2021). "Consolidated recommendations of the Advisory Committee on Vaccines and Immunization Strategies- on prioritizing COVID-19 vaccination." Revista Chilena De Infectologia **38**(2): 185-188.

COVID-19 is a global public health issue due to its epidemic nature that, to date, lacks pharmacological treatment. However, some COVID-19 vaccines have been authorized for emergency use, although the duration of their protection, their ability to interrupt viral transmission, and their efficacy against emerging variants of SARS-CoV-2 are being studied. Chile's SARS-CoV-2 vaccination campaign required design and planning, like any other campaign. This process included the prioritization of risk groups for vaccination given the limited supply of COVID-19 vaccines globally. Throughout 2020, CAVEI issued recommendations on the prioritization of population groups to be vaccinated against SARS-CoV-2 in response to different needs and in accordance with available evidence. These recommendations are consolidated in Table I in this report. In summary, it was recommended that healthcare workers, people in long-term residences and essential State personnel be vaccinated in phase I . In phase 2, persons over 65 years of age and people with comorbidities. In phase 3, essential tasks workers and, lastly, the general population.

Dabas, V., et al. (2020). "Management of Orthopaedic Accidental Emergencies Amidst COVID-19 Pandemic: Our Experience in Preparing to Live with Corona." Indian Journal of Orthopaedics **54**(SUPPL 2): 380-385.

Introduction With increasing prevalence of coronavirus cases (including among health care providers), the current advice for orthopaedic surgeons is to favor non-operative management of most injuries and reduce face-to-face follow-up. We present our experience in managing the patients at Government-run non-COVID-19 trauma center in Delhi in an algorithmic form. Our standard operating protocols were mainly based on recommendations of Indian Orthopaedic Association and targeted to provide healthcare at a minimum risk to the treating team as well as other patients admitted to the hospital. Methodology We describe the inflow, in-hospital management and outflow of patients at our facility during the lockdown period and in the following unlock period (from 23 March to 8 July 2020). Those patients who had absolute indications for surgery were offered surgery, while conservative treatment was more favored in those with relative indications. We also highlight the changes incorporated in OT settings as well as in rehabilitative and follow-up period. Results Following the described protocol helped us maintain a balance between the safety of patients and our front line workers which was evident by very low COVID-19-positive rate in admitted patients (4.22%) and health care providers (16.67%) in the above-mentioned time period. Conclusions We need to be prepared to cohabitate with this deadly Novel Coronavirus and adapt our surgical practices according to the need of the hour by minimizing surgical indications and strengthening the training in conservative principles.

Dar-Odeh, N., et al. (2020). "COVID-19: Present and Future Challenges for Dental Practice." International Journal of Environmental Research and Public Health **17**(9).

COVID-19 was declared a pandemic by the World Health Organization, with a high fatality rate that may reach 8%. The disease is caused by SARS-CoV-2 which is one of the coronaviruses. Realizing the severity of outcomes associated with this disease and its high rate of transmission, dentists were instructed by regulatory authorities, such as the American Dental Association, to stop providing treatment to dental patients except those who have emergency complaints. This was mainly for protection of dental healthcare personnel, their families, contacts, and their patients from the transmission of virus, and also to preserve the much-needed supplies of personal protective equipment (PPE). Dentists at all times should competently follow cross-infection control protocols, but particularly during this critical time, they should do their best to decide on the emergency cases that are indicated for dental treatment. Dentists should also be updated on how this pandemic is related to their profession in order to be well oriented and prepared. This overview will address several issues concerned with the COVID-19 pandemic that directly relate to dental practice in terms of prevention, treatment, and orofacial clinical manifestations.

Das, S., et al. (2021). "Death and Mourning Process in Frontline Health Care Professionals and Their Families During COVID-19." Frontiers in Psychiatry **12**.

The COVID-19 epidemic has mushroomed globally, disrupting the existence of millions. Under this current pandemic situation, the frontline health care professionals are looped in the clutch of the virus and are relatively more exposed to the patients infected with the disease. In this precarious situation, the frontline health care professionals have contributed their best to provide utmost care to the patients infected with the ailment. The direct involvement of these professionals, however, has taken a toll on their physical health as well as on their mental well-being. Several studies conducted recently have reported that frontline health care workers engaged in direct diagnosis, treatment, and care of patients with COVID-19 are associated with a higher risk of symptoms of depression, post-traumatic stress disorder and other mental health issues. Lack of personal protection equipment, unreasonable amounts of work, improper medicines, fear of contracting the disease, and lack of skilled training have interposed the frontline health care workers with unimaginable stress. Due to the widespread outbreak, the death count of the frontline health care professionals has also surged. However, studies exploring the physical and mental welfare of the frontline health care professionals and their families are very few and far behind. To address this aperture, the present paper attempts to highlight the psychological and physical impact of the COVID-19 pandemic on the frontline health care professions and to understand the impact of the death of these frontline health care professionals on the psychological well-being, mourning process, and complicated grief among the family members of healthcare professionals. The paper also presents some recommendations for providing psychological support to healthcare professionals and their bereaved families.

Dascalu, A. M., et al. (2020). "IMPACT OF THE COVID-19 PANDEMIC UPON THE OPHTHALMOLOGICAL CLINICAL PRACTICE." Romanian Journal of Legal Medicine **28**(1): 96-100.

Introduction. With more than 1,000,000 infections around the world, SARS-Cov 2 is responsible for the most severe healthcare crisis in the last 100 years. Aim. The paper analyzes the impact of COVID 19 pandemic upon the current ophthalmological clinical activity. Materials and Method. A comparative analyses was carried regarding the clinical activity in March 2019 and March 2020: the number of one day hospitalizations, of continuous hospitalizations, the percentage of emergencies, the number of surgeries, bed turn over, mean hospital stay. The protocols for the health care personnel protection and decontamination were analyzed. Results. During March 2019, 381 patients were treated in the day hospitalization and 297 patients in the continuous hospitalization regime, with an average length of hospitalization of 3.2 days, and a bed turnover of 6.05. Of these, 76.43% underwent ophthalmic surgery and 14.1% represented ophthalmological emergencies. 1 he comparative analysis shows that in March 2020, the number of one day hospitalizations decreased by 87.4%, and that of the hospitalizations continued with 89.2%. Of the patients admitted, 46.8% were in emergency. Clinical ophthalmological practice has been deeply changed in the context of the COVID 19 pandemic in order to prevent contamination both for patients and medical personnel. Conclusions. The ocular manifestations associated to SARS-Cov 2 infection are self-limited, but the possibility of infection due to the presence of the virus at the level of the tear secretion is an aspect that we must consider in the ophthalmological clinical practice.

Dawson, M. A. (2021). "Black Nurses Collaborative Approach to Addressing COVID-19 in Black Communities." Journal of Racial and Ethnic Health Disparities **8**(3): 790-793.

As with other national disasters, epidemics, and pandemics, the novel coronavirus SARS-CoV-2 (COVID-19) pandemic has highlighted health disparities in Black communities in the USA. Healthcare providers, community activists, politicians, members of faith-based organizations, professional athletes, and Black families are asking crucial questions about why Black and Brown people are disproportionately infected by, and dying from, the COVID-19. Evidence in healthcare and social sciences literature demonstrates that historically, systemic racism and injustices play a large role in the health and well-being of Blacks living in the USA. For decades, the National Black Nurses Association has been on the forefront, engaging our people using a collaborative community-based practice model. The healthcare goal in the USA should center on health protection, promotion, and prevention, moving toward a wellness model and away from treatment of illnesses that contribute to healthcare waste. Finally, awareness of social determinants of health has taken center stage, demonstrating how laws, policies, and practice affect health outcomes and the well-being of Black and Brown communities. In order to address social determinants of health and healthcare inequity, the National Black Nurses Association has called for an increase in the number of Black registered nurses and licensed vocational and practical nurses. The healthcare goal in the USA should center on health protection, promotion, and preventions moving toward a wellness model and away from treatment of illnesses that contributes to healthcare waste.

de Leeuw, R. A., et al. (2020). "COVID-19 and Laparoscopic Surgery: Scoping Review of Current Literature and Local Expertise." Jmir Public Health and Surveillance **6**(2).

Background: The current coronavirus disease (COVID-19) pandemic is holding the world in its grip. Epidemiologists have shown that the mortality risks are higher when the health care system is subjected to pressure from COVID-19. It is therefore of great importance to maintain the health of health care providers and prevent contamination. An important group who will be required to treat patients with COVID-19 are health care providers during semiacute surgery. There are concerns that laparoscopic surgery increases the risk of contamination more than open surgery; therefore, balancing the safety of health care providers with the benefit of laparoscopic surgery for the patient is vital. Objective: We aimed to provide an overview of potential contamination routes and possible risks for health care providers; we also aimed to propose research questions based on current literature and expert opinions about performing laparoscopic surgery on patients with COVID-19. Methods: We performed a scoping review, adding five additional questions concerning possible contaminating routes. A systematic search was performed on the PubMed, CINAHL, and Embase databases, adding results from gray literature as well. The search not only included COVID-19 but was extended to virus contamination in general. We excluded society and professional association statements about COVID-19 if they did not add new insights to the available literature. Results: The initial search provided 2007 records, after which 267 full-text papers were considered. Finally, we used 84 papers, of which 14 discussed severe acute respiratory syndrome coronavirus 2 (SARS-CoV-2). Eight papers discussed the added value of performing intubation in a low-pressure operating room, mainly based on the SARS outbreak experience in 2003. Thirteen papers elaborated on the risks of intubation for health care providers and SARS-CoV-2, and 19 papers discussed this situation with other viruses. They conclude that there is significant evidence that intubation and extubation is a high-risk aerosol-producing procedure. No papers were found on the risk of SARS-CoV-2 and surgical smoke, although 25 papers did provide conflicting evidence on the infection risk of human papillomavirus, hepatitis B, polio, and rabies. No papers were found discussing tissue extraction or the deflation risk of the pneumoperitoneum after laparoscopic surgery. Conclusions: There seems to be consensus in the literature that intubation and extubation are high-risk procedures for health care providers and that maximum protective equipment is needed. On the other hand, minimal evidence is available of the actual risk of contamination of health care providers during laparoscopy itself, nor of operating room pressure, surgical smoke, tissue extraction, or CO2 deflation. However, new studies are being published daily from current experiences, and society statements are continuously updated. There seems to be no reason to abandon laparoscopic surgery in favor of open surgery. However, the risks should not be underestimated, surgery should be performed on patients with COVID-19 only when necessary, and health care providers should use logic and common sense to protect themselves and others by performing surgery in a safe and protected environment.

de Miranda, R. R., et al. (2020). "Head and neck cancer therapy-related oral manifestation management in the COVID-19 pandemic: a critical review." Brazilian Oral Research **34**.

With the onset of the new coronavirus disease (COVID-19) pandemic, the dental treatment of patients at risk of infection has become quite challenging. In view of this, patients with head and neck cancer may present with oral complications due to anticancer therapy, making dental assistance necessary. Thus, the objective of the study was to review the literature and critically discuss important concerns about the treatment of patients with head and neck cancer during the COVID-19 pandemic. Because dental professionals are in close contact with the main viral transmission routes, this study presents recommendations for management and protection during clinical dental care. The main characteristics and transmission routes of COVID-19 are also discussed. Dental professionals should control pain and the side effects of antineoplastic treatment and use preventive measures for infection control. During this pandemic, patients with head and neck cancer should not undergo elective procedures, even if they do not have symptoms or a history of COVID-19; therefore, in asymptomatic or painless cases, only preventive actions are recommended. In symptomatic or painful cases, precautions for safe interventional treatments must be implemented by following the hygiene measures recommended by health agencies and using personal protective equipment. During health crises, new protocols emerge for cancer treatment, and professionals must act with greater attention toward biosafety and updated knowledge. It is important to offer adequate individualized treatment based on the recommendations of preventative and interventional treatments so that patients can face this difficult period with optimized quality of life.

de Perio, M. A., et al. (2020). "Strategies for Optimizing the Supply of N95 Filtering Facepiece Respirators During the Coronavirus Disease 2019 (COVID-19) Pandemic." Disaster Medicine and Public Health Preparedness **14**(5): 658-669.

N95 respirators are personal protective equipment most often used to control exposures to infections transmitted via the airborne route. Supplies of N95 respirators can become depleted during pandemics or when otherwise in high demand. In this paper, we offer strategies for optimizing supplies of N95 respirators in health care settings while maximizing the level of protection offered to health care personnel when there is limited supply in the United States during the 2019 coronavirus disease pandemic. The strategies are intended for use by professionals who manage respiratory protection programs, occupational health services, and infection prevention programs in health care facilities to protect health care personnel from job-related risks of exposure to infectious respiratory illnesses. Consultation with federal, state, and local public health officials is also important. We use the framework of surge capacity and the occupational health and safety hierarchy of controls approach to discuss specific engineering control, administrative control, and personal protective equipment measures that may help in optimizing N95 respirator supplies.

Delfani, F. and M. Shoghi (2021). "Burn care strategy in the covid-19 pandemic: a narrative review study." International Journal of Burns and Trauma **11**(4): 289-295.

Introduction: The COVID-19 pandemic has changed the world and intensified health management in many parts of the world. Sensitivity in burn patients' care and prevalence of COVID-19 have a major impact on this group's healthcare system and infection control strategies. The purpose of this study is to review burn care strategies during the COVID-19 outbreak. Materials and methods: The present study is a narrative review study. In writing this paper, to access information, the keywords of Coronavirus, COVID-19, burn, strategy, care, and SARS were used. Due to the emergence of COVID disease, searches in Science Direct, PubMed, Scopus, Web of Science, Google Scholar, SID, Magiran, Springer, and Elsevier databases were performed without time limit. Research articles were purposefully selected and approved to be investigated based on the criteria for entering the research. A total of 14 articles had a full text related to the research scope. The researchers used qualitative content analysis to analyze the data. Data analysis was conducted as a comprehensive review of articles, qualitative conclusions, and results. Results: In this study, 14 articles related to burn care strategies in the COVID-19 epidemic were selected. Their results were reviewed in two areas of staff care strategies and burn patients during the COVID-19 outbreak. Findings indicate that appropriate prevention and protection strategies and measures should be followed to prevent the transmission of the COVID-19 virus to burn care professionals. In addition, burn patient care strategies were proposed in two levels of minor and moderate to severe burns, so that patients with burns receive adequate burn care during the COVID-19 outbreak. Conclusion: Implementation of staff and burn patient care strategies during the COVID-19 pandemic is essential to treat burn patients and prevent the spread of the COVID virus.

Deora, H., et al. (2020). "The Aftermath of COVID-19 Lockdown- Why and How Should We Be Ready?" Neurology India **68**(4): 774-791.

Introduction: Severe acute respiratory syndrome, coronavirus 2 (SARS-COV 2) has inexplicably and irreversibly changed the way of neurosurgery practice. There has been a substantial reduction in neurosurgical operations during the period of lockdown. The lockdown might be the most effective measure to curtail viral transmission. Once we return to the normalization of the lifestyle, there will be a backlog of unoperated pending cases along with the possibility of further spread of the coronavirus. Methods: We reviewed the available literature and protocols for neurosurgical practice in different geographic locations. We drafted a consensus statement based on the literature and protocols suggested by the World Health Organization (WHO) and various professional societies to prevent the spread of SARS-COV2 while streamlining the neurosurgical practice. Results: The consensus statement suggests the patient triage, workflow, resource distribution, and operational efficacy for care providers at different stages of management. The priority is set at personal protection while ensuring patients' safety, timely management, and capacity building. We performed a detailed subsection analysis for the management of trauma and set up for COVID-free hospitals for simultaneous management of routine neurosurgical indications. In this time of medicolegal upheaval, special consent from the patients should be taken in view of the chances of delay in management and the added risk of corona infection. The consensus statements are applicable to neurosurgical setups of all capacities. Conclusion: Along with the glaring problem of infection, there is another threat of neurosurgery emergency building up. This wave may overwhelm the already stretched systems to the hilt. We need to flatten this curve while avoiding contagion. These measures may guide neurosurgery practitioners to effectively manage patients ensuring the safety of caregivers and care seekers both.

Dey, A., et al. (2021). "COVID-19 pandemic lockdown-induced altered sleep/wake circadian rhythm, health complaints and stress among traffic police personnel in India." Chronobiology International **38**(1): 140-148.

COVID-19 is a highly contagious disease caused by Severe Acute Respiratory Syndrome coronavirus-2, capable of high human transmission. To protect against the COVID-19 pandemic, the World Health Organization proposed intervening guidance that included movement restrictions, isolation, restriction of national and international travel or movement, and full or partial closure of organizations and institutions. Police personnel play a vital role in safeguarding the spread of COVID-19; thus, potentially causing severe stress due to increased consignment and direct exposure to infection during duty. The study aimed to determine the effect of the added stress of the COVID-19 pandemic and population lockdown on the sleep/wake 24 h rhythm of traffic police. A concise online questionnaire survey was conducted among the traffic police personnel of India. Collected data were analyzed statistically. Disorientation of working schedule, fear of being vulnerable to disease, pressure of maintaining law, and orders during lockdown increased stress level. The survey identified discontinuation of sleep, shift of mid-sleep time, increase in depression, plus stress and anxiety among traffic police personnel that affected their chronobiological milieu. Proper awareness of adequate health and safety measures, use of personnel protection equipment, regular conversation with family members, and meditation can help reduce stress during this pandemic situation.

Dockery, D. M., et al. (2020). "THE OCULAR MANIFESTATIONS AND TRANSMISSION OF COVID-19: RECOMMENDATIONS FOR PREVENTION." Journal of Emergency Medicine **59**(1): 137-140.

Background: Coronavirus disease-2019 (COVID-19), caused by a novel coronavirus termed severe acute respiratory syndrome coronavirus-2 (SARS-CoV-2), has been linked to ocular signs and symptoms in several case reports. Research has demonstrated that SARS-CoV-2 is spread primarily through close contact via respiratory droplets, but there is the possibility for ocular transmission, with the conjunctiva as a conduit as well as a source of infection. Discussion: Ocular manifestations of SARS-CoV-2 include follicular conjunctivitis, and have been repeatedly noted as an initial or subsequent symptom of COVID-19-positive patients. Particularly in patients with ocular manifestations, there is evidence that the virus may present in tears, based on the detection of SARS-CoV-2 in conjunctival swab samples via reverse transcription polymerase chain reaction. The virus may therefore be transmittable from the ocular surface to a new host via contact with the ocular mucosa, tears, or subsequent fomites. Conclusions: All health care professionals should ask patients about ocular symptoms consistent with SARS-CoV-2, and use eye protection such as goggles or face shields as part of the standard personal protective equipment for high-risk patients in addition to wearing of masks by both the patient and provider, and should consider tears to be potentially infectious. (C) 2020 Elsevier Inc. All rights reserved.

Dominelli, L. (2021). "A green social work perspective on social work during the time of COVID-19." International Journal of Social Welfare **30**(1): 7-16.

COVID-19 has challenged social workers to engage with health pandemics and provide essential services in conditions of uncertainty and high risk. They have safeguarded children, older adults and diverse adults in 'at risk' groups under tough conditions mediated by digital technologies, adhered to government injunctions, maintained social and physical distancing under lockdown and worked from home remotely. Social workers and social care workers have risen to the challenges, providing services with inadequate personal protective equipment and limited supervision and support. This article highlights the degraded physical environments, socio-economic and political contexts that intensify precariousness and constraints that neoliberalism imposed on professional capacity before and during this health pandemic. It provides guidelines to protect practitioners and service users. It concludes that practitioners ought to understand zoonotic diseases, environmental concerns, acquire disaster expertise and training, widen their practice portfolio and value their contributions to this pandemic. Key Practitioner Message: center dot Develop technological skills and innovate to support stressed individuals, safeguard children, adolescents and elders and deal with poverty and unemployment; center dot Use digital technologies involving peers to explore tricky situations, examine ethical dilemmas through scenario building exercises, and tips for self-care; center dot Contribute to environmental protections that prevent the spread of zoonotic diseases like COVID-19; center dot Seek supervision and support for disaster-based training from your line manager.

Drazkiewicz, E. (2020). "Scientific Intimacy The Changing Relationship with Medical Data at the Time of COVID-19 pandemic." Anthropology in Action-Journal for Applied Anthropology in Policy and Practice **27**(3): 71-77.

As the coronavirus started to spread in Ireland, the epidemiological data became the most sought-after information in the country. This article will examine the ways in which COVID-19 redefined the intimacies of the relationships that health professionals and the members of the public have with medical data. It will focus on Irish examples and explore how the context of the pandemic turned numbers from abstract cognitive tools into important and affective tenets of social lives that dictated the moral values and conditions of sociality. It will examine the role of enumeration and metrics in mediating new forms of intimacy with state and society.

Driscoll, J., et al. (2020). "Multi-agency safeguarding arrangements: overcoming the challenges of Covid-19 measures." Journal of Childrens Services **15**(4): 267-274.

Purpose Information sharing and joint working between agencies undertaking direct work with children have long been recognised as fundamental to robust and effective safeguarding and child protection arrangements. The public health response to Covid-19 disrupted those arrangements abruptly. This study aims to identify some of the innovative practices that have been implemented and how responses might inform planning for multi-agency working in the future. Design/methodology/approach This study presents reflections on preliminary fieldwork from a study of how agencies in London are responding to the challenges for multi-agency safeguarding arrangements created by the Covid-19 measures. It draws on the experience of expert practitioners in the research team as well as interviews with 17 senior professionals from local authorities, safeguarding partnerships and health. Findings The study participants endorsed known concerns around increased risks to children and raised new concerns about particular groups of children that under normal circumstances would not have been at risk. They identified some unexpected benefits derived from new arrangements, especially in relation to engagement with remote working. Originality/value Early insights are offered into promising initiatives to preserve strong multi-agency arrangements in crises and strengthen the resilience of the child protection system.

Dryzun, J. (2020). "An Exceptional Condition in the Time of COVID-19: Health professionals and Workers Cannot Isolate Themselves." Acta Psiquiatrica Y Psicologica De America Latina **66**(1): 70-74.

In these exceptional times caring for the caregivers represents a priority as important as implementing the measures required for the Covid19 pandemic. The world has stopped and it appears an uncertain perspective of how and what it will be like to inhabit it again. In this dramatic situation, the community of health workers is in full activity while undergoing an unprecedented physical and mental demand. Compliance with social isolation is what keeps the community safe in general, but health workers remain in the trench. Their specific activity and work, which prevent them from isolating themserlves, imply maximum exposure increasing both their infectious vulnerability and emotional health. Close and continuous contact with the effects of the pandemic in the health area agressively exposes them to the emotional drama that they must attend and cannot avoid. Vulnerability and protection factors are described, both individually and from the community health network.

El Kassas, M., et al. (2020). "Emergency endoscopic variceal band ligation in a COVID-19 patient presented with hematemesis while on mechanical ventilation." Digestive Endoscopy **32**(5): 812-815.

COVID-19, caused by severe acute respiratory syndrome coronavirus (SARS-CoV-2), is now a global pandemic with serious health consequences. Currently, many strict control measures are applied in health care settings, including endoscopy units, in order to limit virus spread. Several recommendations called to limit endoscopic procedures to emergent endoscopies; however, several uncertainties still exist concerning patient safety, protective measures, and infection control methods in emergency endoscopic settings. In this case report, we present a case of successful endoscopic band ligation for bleeding esophageal varices in man with COVID-19 disease who presented with an acute attack of hematemesis while on mechanical ventilation (MV). Esophago-gastroduodenoscopy was performed in the ICU room after preparing the setting, and revealed large, risky esophageal varices. Endoscopic band ligation was done with successful control of bleeding. Third-level measures of medical protection were applied for the participating medical personnel, and patient monitoring was maintained all through the procedure. After the procedure, the bleeding stopped, and the patient was vitally stable and conscious. We conclude that emergency endoscopic interventions could be performed safely with appropriate arrangements in patients with confirmed COVID-19 on MV.

Esmizadeh, E., et al. (2021). "Can Medical-Grade Gloves Provide Protection after Repeated Disinfection?" Acs Applied Polymer Materials **3**(1): 445-454.

The advent of the COVID-19 pandemic has generated an increased consumption of personal protective equipment (PPE), including gloves and masks, by healthcare workers and by the general public at a global scale. This has generated substantial shortage of these single-use and disposable PPEs that will end up as a landfill waste. Extending the life cycle of PPEs, such as gloves, by disinfecting treatments could help mitigate these concerns. However, the effect of various disinfection treatments on the functionality of gloves is unknown. In this study, six commonly used viral disinfection treatment methods (i.e., ultraviolet (UV) radiation, dry heat, steam, alcohol, chlorine compounds, and quaternary ammonium compounds) were evaluated for their effect on the performance attributes of two commonly used medical-grade gloves and nitrile and vinyl (latex) gloves. The barrier properties of both gloves against water and ethanol vapor flux were not affected up to 10 cycles of disinfection cycles. However, the increase in the disinfection cycle from 10 to 20 slightly reduced their barrier properties with minor variation in the type of disinfection and glove type. Infrared spectroscopy and microscopy investigations confirmed that both types of gloves could withstand up to 20 cycles of disinfection treatments with no observable change in the chemical structure and surface morphology of the disinfected surfaces, respectively. Lastly, tear property testing of the gloves indicated little to no change from the baseline after 20 cycles of treatment in both the nitrile and vinyl-based gloves. Overall, this study indicated that alcohol, UV, and heat treatment could be acceptable disinfection methods that allow the reuse of gloves up to 20 cycles. Such repeated disinfection of gloves not only reduces the strain on the supply of gloves but also decreases the postconsumer landfilled waste and environmental footprint of gloves.

Esposito, C., et al. (2020). "Letter to the Editor: Robot-Assisted and Minimally Invasive Pediatric Surgery and Urology During the COVID-19 Pandemic: A Short Literature Review." Journal of Laparoendoscopic & Advanced Surgical Techniques **30**(8): 915-918.

The COVID-19 outbreak has dramatically impacted our activities of pediatric surgeons and urologists over the past 3 months, especially in the field of minimally invasive surgery (MIS) and robotics. Analyzing the available literature, there is very scarce evidence regarding the use of MIS and robotics for treatment of pediatric surgical and urological pathologies during this pandemic. However, we found some useful information that we would like to share with other pediatric surgeons and urologists through this journal. Based upon the available data, we believe that surgery should only be performed in pediatric patients with emergent/urgent and oncological indications until resolution of the COVID-19 outbreak. Robotics and MIS may be safely performed in such selected children by adopting specific technical precautions such as prevention of aerosol dispersion using filters/suction or adapted systems and appropriate use of electrocautery and other sealing devices for reduction of surgical smoke, as reported in our recent experience. Another key point to manage this pandemic emergency is that all hospitals should provide health care professionals with adequate individual protections and perform universal screening in all patients undergoing surgery. Considering that this pandemic is a rapidly evolving situation with new information available daily, these data resulting from the analysis of literature focused on pediatric robotics and MIS may be further revised and updated.

Fan, T., et al. (2021). "The Impact of COVID-19 on Otolaryngology Community Practice in Massachusetts." Otolaryngology-Head and Neck Surgery **165**(3): 424-430.

Objectives Coronavirus disease 2019 (COVID-19) significantly affected many health care specialties, including otolaryngology. In response to governmental policy changes, many hospitals and private practices in Massachusetts canceled or postponed nonurgent office visits and elective surgeries. The objective of this study was to quantify the impact of COVID-19 on the provision and practice trends of otolaryngology services for 10 private practices in Massachusetts. Study Design Retrospective review. Setting Multipractice study for community practices in Massachusetts. Methods Electronic billing records from 10 private otolaryngology practices in Massachusetts were obtained for the first 4 months of 2019 and 2020. Questionnaires from these otolaryngology practices were collected to assess financial and staffing impact of COVID-19. Results The local onset of the COVID-19 pandemic had a significant decrease of 63% of visits in comparison to equivalent weeks in 2019. Virtual visits overtook in-person visits over time. A greater decline in operating room (OR) procedures than for office procedures was recorded. Ninety percent of practices reduced working hours, and 80% furloughed personnel. Seventy percent of practices applied for the Paycheck Protection Program (PPP). Conclusion COVID-19 has had a multifaceted impact on private otolaryngology practices in Massachusetts. A significant decline in provision of otolaryngology services aligned with the Massachusetts government's public health policy changes. The combination of limited personnel and personal protective equipment, as well as suspension of nonessential office visits and surgeries, led to decrease in total office visits and even higher decrease in OR procedures.

Farhat, A. S., et al. (2021). "Does the Use of Personal Protection Equipment for the Medical Staff Working with Patients with Coronavirus Disease 2019 Need to be Revised?" Iranian Journal of Neonatology **12**(2): 102-104.

Background: Personal protective equipment (PPE) is one of the protective equipment that health workers can use to protect themselves against coronavirus disease 2019 (COVID-19); however, it is accompanied by some problems for medical staff. Since severe acute respiratory syndrome coronavirus 2 (SARS-CoV-2) is not transmitted through healthy skin, the hypothesis was examined as to whether the cotton gown could be used instead of PPE. Methods: The medical staff of the Neonatal Care Unit took care of 51 patients with 1180 shifts. During the first and a half months, the medical personnel used a special PPE package for providing care of patients, which included an airimpermeable plastic gown with a head-to-toe cover, an N95 mask, gloves, and a face shield. However, from the beginning of the epidemic, doctors used cotton surgical gowns instead of the ones in the package. Following that, the nursing team gradually replaced the air-impermeable plastic gowns with cotton surgical gowns, and finally, all the staff used the cotton gowns. Results: After three months, all medical staff was evaluated for clinical signs of COVID-19, such as fever, cough, nausea, and headache. During these three months, no symptoms or absence due to illness were observed in the staff. It should be mentioned that the antibody titer was not evaluated at the end of the study. Conclusion: A retrospective review of the staff showed that the cotton surgical gown protected the staff against COVID-19. Accordingly, the cotton surgical gown can be used in medical centers to replace PPE sets containing an air-impermeable plastic gown with a head-to-toe cover. The result of this study can improve some of the problems of the medical staff.

Fernandez-Simo, D., et al. (2020). "Incidence of the State of Alarm on School Inequality of Adolescents within the Protection System in Galicia." Revista Internacional De Educacion Para La Justicia Social **9**(3): 141-156.

Students with administrative protection records are the protagonists of most of the academic itineraries of school exclusion. The declaration of the state of alarm, motivated by the health alert of Covid-19, leads to a change in the school dynamics of the students. This research aims to analyze which aspects of educational planning, as well as the collaboration between teachers and professionals of the specific protection resources of Galicia during the state of alarm, have an impact on the inclusion or exclusion of students in social difficulty. Eleven longitudinal follow-ups are carried out with 11 adolescents, five boys and six girls. The study is based on the grounded theory with the intention of triangulating the discourses derived from the interviews with students and instrumental case studies involving 14 professionals. The sample of educational teams consists of 11 female educators and 3 male educators. The results highlight that the dynamics of school inequality have increased during the period of the state of alarm, observing that the contact between students and teachers is reduced, which leads to deficits in the students' homework; and that the collaboration between the professionals in the protection system and the school is limited to timely exchanges of information. These results underline that, during the state of alarm motivated by the current health crisis, the school and education policies in Galicia neglect the situation of greater social difficulty of the students under protection.

Ferorelli, D., et al. (2020). "Medical Legal Aspects of Telemedicine in Italy: Application Fields, Professional Liability and Focus on Care Services During the COVID-19 Health Emergency." Journal of Primary Care and Community Health **11**.

Telemedicine services can be classified into the macro-categories of specialist Telemedicine, Tele-health and Tele-assistance. From a regulatory perspective, in Italy, the first provision dedicated to the implementation of Telemedicine services is represented by the Agreement between the Government and the Regions on the document bearing "Telemedicine-National guidelines," approved by the General Assembly of the Superior Health Council in the session of 10th July 2012 and by the State Regions Conference in the session of 20th February 2014. Scientifically, several studies in the literature state that information and communication technologies have great potential to reduce the costs of health care services in terms of planning and making appropriate decisions that provide timely tools to patients. Another clear benefit is the equity of access to health care. The evolution of telemedicine poses a series of legal problems ranging from the profiles on the subject of authorization and accreditation to those concerning the protection of patient confidentiality, the definition and solution of which, in the absence of specific regulatory provisions, is mainly left to the assessment of compatibility of the practices adopted so far, with the general regulatory framework. In terms of professional liability, it is necessary to first clarify that the telemedicine service is comparable to any diagnostic-therapeutic health service considering that the telemedicine service does not replace the traditional health service, but integrates the latter to improve its effectiveness, efficiency and appropriateness.

Fletcher, J. J., et al. (2022). "Healthcare-acquired coronavirus disease 2019 (COVID-19) is less symptomatic than community-acquired disease among healthcare workers." Infection Control and Hospital Epidemiology **43**(4): 490-496.

Objective: We hypothesized that healthcare workers (HCWs) with high-risk exposures outside the healthcare system would have less asymptomatic coronavirus 2019 (COVID-19) disease and more symptoms than those without such exposures. Design: A longitudinal point prevalence study was conducted during August 17-September 4, 2020 (period 1) and during December 2-23, 2020 (period 2). Setting: Community based teaching health system. Participants: All HCWs were invited to participate. Among HCWs who acquired COVID-19, logistic regression models were used to evaluate the adjusted odds of asymptomatic disease using high-risk exposure outside the healthcare system as the explanatory variable. The number of symptoms between exposure groups was evaluated with the Wilcoxon rank-sum test. The risk of seropositivity among all HCS by work exposure was evaluated during both periods. Interventions: Survey and serological testing. Result: Seroprevalence increased from 1.9% (95% confidence interval [CI], 1.2%-2.6%) to 13.7% (95% CI, 11.9%-15.5%) during the study. Only during period 2 did HCWs with the highest work exposure (versus low exposure) have an increased risk of seropositivity (risk difference [RD], 7%; 95% CI, 1%-13%). Participants who had a high-risk exposure outside of work (compared to those without) had a decreased probability of asymptomatic disease (odds ratio [OR], 0.38; 95% CI, 0.16-0.86) and demonstrated more symptoms (median 3 [IQR, 2-6] vs 1 [IQR, 0-4]; P = .001). Conclusions: Healthcare-acquired COVID-19 increases the probability of asymptomatic or mild COVID-19 disease compared to community-acquired disease. This finding suggests that infection prevention strategies (including masks and eye protection) may be mitigating inoculum and supports the variolation theory in COVID-19.

Frieling, T. (2020). "Consequences of the COVID-19/SARS-CoV-2 pandemic for gastroenterology in Germany." Gastroenterologe **15**(6): 498-505.

The recommendations of the German Society of Gastroenterology, Digestive and Metabolic Diseases (DGVS) on coronavirus disease 2019 (COVID-19) in gastroenterological patients are accessible on the homepage of the Society under information at . Recommendations for protection against severe acute respiratory syndrome coronavirus 2 (SARS-CoV-2) have been published by the European Society of Gastrointestinal Endoscopy (ESGE) and the European Society of Gastroenterology and Endoscopy Nurses and Associates (ESGENA). Separate endoscopy rooms for high-risk patients and negative pressure rooms should be included in the planning of future endoscopy unit projects. It is unclear whether a continuing prioritization of gastroenterological services after the first wave of COVID-19 is necessary in the future. The report of the Scientific Institute of the AOK health insurance company (WldO) and surveys of the Working Group of Clinical Gastroenterologists (ALGK) and the Professional Association of Private Gastroenterologists in Germany (bng) demonstrated that the coronavirus lockdown led to a significant reduction of gastrointestinal diseases and gastroenterological services including screening colonoscopy. It is likely that the reduction of the overcapacity of the German healthcare system will also be continued by reduction of inpatient services after the corona pandemic has been overcome. It is essential to keep the medical profession attractive for young colleagues. The high rates of burnout and suicide and the difficult compatibility with the requirements of a life balance are alarm signals that have to be solved.

Gandolfi, M. G., et al. (2020). "Coronavirus contamination in dental clinics. New systems and operating devices." Dental Cadmos **88**(6): 368-378.

OBJECTIVES Sanitary emergency of SARS-CoV-2 is radically changing all the procedures in dental clinics. A great number of health-care workers resulted affected by Coronavirus. Dentists work closely face-to-face with the patients and this condition greatly increases the risk for Coronavirus infection. Normal dental procedures create a tremendous hazard and play a number of risks for contaminations with patient's saliva and exhaled particles. All patients may be asymptomatic but Coronavirus-positive and may represent a great risk for virus diffusion in the dental room and among the personnel and/or other patients. The principal problem in dental office is constituted by the presence of aerosols and spray droplets created by high-speed water-cooled handpiece, ultrasonic instrument, ultrasonic scalers and dental air-water spray guns. The second important problem is connected with the long face-to-face of the operators (dentist and assistants) with patients. The airborne fluctuating particles - composed by saliva, mucus, blood, dentin-enamel debris, smear layer, fragments of restorations - can remain floating for a long time in the aerosol tide and are diffused by air vortexes created by high-speed hand-piece. MATERIALS AND METHODS The study identified different type of droplets and airborne produced by dental-unit chair (DUC) instruments. These droplets and airborne particles are produced by DUC instruments and may be immediately infected by patient mucus and saliva - and in theory plaque and blood - and diffused around as fluctuating particles. These particles present a diameter from 200 to 2 microns and may remain in air for long time (minutes and hours) and settle in all the surface around DUC. They usually present a ballistic trajectory and may be deviated by turbulences and operator movements. Operator masks, glasses, gown etc. are all exposed surfaces that may be fast contaminated. Also boxes, floor and devices in the dental room may be covered by a layer of droplets. Several new devices and new operative techniques were recently developed to try to reduce the spray diffusion and to limit the risk for operator contamination. A new designed device is constituted by a double rubber-dam arch with a sliding suction pipe able to uptake spray produced in the mouth and by the nose. The device and other types of lip oral cannulas consistently reduce the spray diffusion. Other additional innovative procedures for operators are represented by the use of specific waterproof sprayhood to prevent any droplets contact with masks (FFP2/FFP3) and eyes and by Tynek suites to cover all operator's body. Finally, all DUC and room disinfection procedures must be evaluated to prevent (and to remove) droplets and particles deposits from the surfaces. Additional tailored procedures must be standardized, such as the use of FFP2/3, mounthrinses, disinfection of DUC circuits etc. CONCLUSIONS There is the need for all dental offices and dental Clinics to rapidly modify all the clinical procedures in attempt to reduce airborne and spray droplets.The close contact of the operators with patient's nose and mouth and the potential presence of virus into mucus and saliva create a tremendous hazard due to the production of DUC spray. Again, there is a need for Universities, Dental Clinics and Hospitals to protect students, workers and patients with new tailored operative procedures. CLINICAL SIGNIFICANCE In this position paper, it is support the concept that we need major and rapid reorganization of dental office procedures. The main concept is to restrict the generation of dental spray and use adequate procedures to prevent diffusion in dental offices. New tailored operative procedures are proposed after the evaluation of high risks for airborne and dental spray droplets production and contamination dynamic routes. The use of powerful novel suction cannula designed for fast spray/saliva droplets aspiration and the use of innovative sprayhoods and body suites for dental-care worker protections are important procedures to prevent virus transmission in dental office. Additional procedures may be standardized to remove droplets from all room surface.

Garg, R., et al. (2020). "Strictly Adhering to Infection Control Practices is the Key to Safe Surgical Procedures Amidst the COVID-19 Crisis." Journal of Pure and Applied Microbiology **14**(2): 1099-1105.

Currently, the world is facing a challenge to curb the outbreak of the highly contagious COVID-19, which has significantly impacted many lives and is affecting the practice of healthcare professionals as well. During this containment phase, an influx of COVID-19 patients requiring surgical interventions is expected. To combat the situation, every health care staff must implement and adhere to various infection prevention and control measures. In this article, we have addressed the importance of basic infection prevention and control practices required for handling COVID-19 cases, especially, those requiring acute surgical care. On one hand, certain non-emergency procedures need to be delayed, on the other hand, all surgeries during this period need to be proactively performed with appropriate infection control precautions regardless of the COVID-19 status of the patient. Prudent decisions and a conservative approach while making the best choices regarding surgeries will not only ensure personal protection but will also preserve important resources, manpower, hospital beds, and equipment for repurposing during this urgent crisis.

Garofalo, P., et al. (2020). "Recommendations of the ECOCARDIOGRAPHY and CARDIAC IMAGES Committee for the protection of patients and health personnel during the COVID-19 pandemic." Revista De La Federacion Argentina De Cardiologia **49**(1): 43-45.

Garzon, M. M. (2020). "Managing Suspected COVID-19 Patients in Surgical Settings: An Integrative Review." Revista Cuidarte **11**(2).

Introduction: The need for surgery in the context of the current COVID-19 pandemic may be a risk for health professionals. For urgent diseases requiring surgical interventions, appropriate decisions should be made according to international recommendations on management of the SARS-CoV2 virus. The study aims to describe and analyze the different recommendations for managing suspected COVID-19 patients in surgical settings. Materials and Methods: An integrative descriptive literature review was conducted on articles published between November 2019 and April 2020 in PubMed, Medline, Science-Direct, Google Scholar, and Scopus databases. Results: 12 studies related to measures and recommendations for the management of suspected COVID-19 patients met the inclusion criteria and were selected. During preoperative care, aspects such as the preparation of the operating room, the use of personal protection equipment, and patient transfer to the operating room were highlighted. During intraoperative care, measures for anesthesia, airway management, cleaning and disinfection of operating rooms, and specific recommendations for postoperative care are highlighted. Discussion: Personal protective equipment and techniques to minimize the risk of cross-infection are reviewed. Conclusions: The surgical team should maintain standard measures for the management of surgical suspected COVID-19 patients to prevent the spread and possible infection with SARS-CoV-2 virus and ensure safe surgical care.

Gebreheat, G. and H. Teame (2021). "Ethical Challenges of Nurses in COVID-19 Pandemic: Integrative Review." Journal of Multidisciplinary Healthcare **14**: 1029-1035.

Currently, nurses are in the middle of the battle against COVID-19. The pandemic situation has put these professionals against various ethical challenges. Therefore, this review aims to identify the main ethical challenges faced by nurses during COVID-19 pandemic. This integrative review was guided by the Preferred Reporting Items of Systematic Reviews and Meta-Analysis Protocols. All English version studies that reported ethical challenges of nurses during the COVID-19 pandemic, from November 9, 2019, to November 9, 2020, were eligible for the review. The electronic databases used were PubMed, Google Scholar, JURN, Cochrane Library E-Journals, MEDLINE, Academic Search Complete, CINAHL. Accordingly, 8 articles were included for further analysis and synthesis. The ethical challenges of nurses were categorized into three thematic areas: nurses' safety, role and moral distress, resource allocation, and client-nurse relationship. Thus, the lack of full protection of nurses across the health industry has raised ethical questions such as the extent of their duty, scarce resources, and the failure of personal protective equipment. In connection, a significant number of nurses were also facing moral distress because of prolonged pressure to maintain the resources needed to provide safe and high-quality nursing care. Furthermore, nurses were challenged to restrict many COVID-19 patients from having end-of-life communication with their families. Overall, nurses are still facing various ethical challenges across the globe. Therefore, it is important to mobilize resources and invest in nurses to bring long-lasting solutions.

Gerall, C., et al. (2020). "Allocation of resources and development of guidelines for extracorporeal membrane oxygenation (ECMO): Experience from a pediatric center in the epicenter of the COVID-19 pandemic." Journal of Pediatric Surgery **55**(12): 2548-2554.

The rapid spread of coronavirus disease 2019 (COVID-19) has exceeded the standard capacity of many hospital systems and led to an unprecedented scarcity of resources, including the already limited resource of extracorporeal membrane oxygenation (ECMO). With the large amount of critically ill patients and the highly contagious nature of the virus, significant consideration of ECMO candidacy is crucial for both appropriate allocation of resources as well as ensuring protection of health care personnel. As a leading pediatric ECMO program in the epicenter of the pandemic, we established new protocols and guidelines in order to continue caring for our pediatric patients while accepting adult patients to lessen the burden of our hospital system which was above capacity. This article describes our changes in consultation, cannulation, and daily care of COVID-19 positive patients requiring ECMO as well as discusses strategies for ensuring safety of our ECMO healthcare personnel and optimal allocation of resources. Level of Evidence: Level V. (c) 2020 Elsevier Inc. All rights reserved.

Gherlone, E., et al. (2021). "Dentistry and COVID-19 pandemic: operative indications post-lockdown." New Microbiologica **44**(1): 1-11.

A new Coronavirus, the seventh member of the Coronaviridae family, identified as SARS-CoV-2, spread in late December 2019 in the territory of Wuhan in China. CoV-2019 can be transmitted directly from person to person by respiratory drops, direct contact and contaminated material. Furthermore, 2019-nCov penetrates cells similarly to the SARS coronavirus, i.e., through the ACE2 receptor. This may promote human-to-human transmission. Patients and dental professionals are exposed daily to pathogenic microorganisms, including viruses and bacteria, which infect the oral cavity and respiratory tract. Dental procedures carry the risk of 2019-nCoV infection due to their specificity. Direct transmission regards the distance between operator and patient, exposure to saliva, blood and other body fluids, the use of sharp instruments and "droplet-generating" rotating instruments, contact with the conjunctival mucosa, and finally the contact with droplets produced by coughing and nasal secretion of an infected individual without mask at short distance, increasing the biological risk for the operator. In light of the pandemic linked to COVID-19, although there is no clear scientific evidence in the literature, it is necessary to identify protections with regard to clothing, operating protocols, disinfection of environments and management of waiting rooms and front offices. This paper is a basis for operative indications for dentists and other health care professionals in phase 2 post lock-down for both private and public structures.

Gibbs, J. L. and M. W. Nonnenmann (2020). "OSHA Voluntary Respirator Use: Challenges incurred with use of N95 filtering facepiece respirators during the COVID-19 pandemic." Toxicology and Industrial Health **36**(9): 681-688.

This article discusses several lessons learned in dealing with the interpretation of the Occupational Health and Safety Administration (OSHA) Voluntary Use provision of the Respiratory Health Standard at health-care facilities during the COVID-19 pandemic in the United States. This includes (but is not limited to) (a) confusion about OSHA policy and procedures when health-care workers brought outside personal protective equipment (PPE; N95 filtering facepiece respirators) into the workplace; (b) challenges in adhering to guidelines stated in Appendix D of the Respiratory Protection Standard; (c) difficulty in achieving respirator fit testing for workers; and (d) vague or inconsistent determination of "non-hazardous" environments (concerning COVID-laden droplets and aerosols). The purpose was to identify gaps in knowledge to help policy makers, enforcement personnel, safety managers, and health-care workers in the United States prepare for similar future events involving PPE shortages.

Gil-Navarro, M. V. and R. Luque-Marquez (2020). "Hospital Pharmacy in the multidisciplinary team of COVID inpatient units." Farmacia Hospitalaria **44**: 40-42.

Since the implementation of the Antimicrobial Stewardship Programs, hospital pharmacy specialists have collaborated with infectious disease specialists on a regular basis in most hospitals in Spain. Cooperation between these professionals ensures the integrated management of patients with infectious diseases and the appropriate use of antimicrobials in hospitals. The COVID-19 pandemic forced hospital pharmacists to abruptly suspend all their structured activities and concentrate on the health crisis. Real-time information sharing between different medicine specialties is an effective strategy to generate and maintain treatment protocols adapted to each center, with continuous evidence-based modifications as new publications appear. Hospital pharmacies had to reorganize their activities to respond to the pandemic. On the one side were patients with COVID-19, and on the other were routine hospital pharmacy tasks, with the added difficulty of adapting to individual protection measures. New communication and collaboration strategies were adopted. Protocols were established for the management of COVID-19 patients, with continuous changes; special medications had to be prepared and distributed; circuits were designed for the home- or institution-based care of patients; internal circuits were created to minimize the movements of hospital staff and professionals caring for COVID-19 patients. The most effective antiviral drug and anti inflammatory therapy remains elusive. In this scenario, hospital pharmacists emerge as a key player, as they have a deep understanding of the mechanisms of action of drugs and potential interactions. In a setting where experimental drugs preferably tested in clinical trials are being used, the role of hospital pharmacists in interdisciplinary teams has become essential for the optimization of clinical outcomes.

Gonzalez-Perez, M., et al. (2021). "The BCG Vaccine for COVID-19: First Verdict and Future Directions." Frontiers in Immunology **12**.

Despite of the rapid development of the vaccines against the severe acute respiratory syndrome coronavirus 2 (SARS-CoV-2), it will take several months to have enough doses and the proper infrastructure to vaccinate a good proportion of the world population. In this interim, the accessibility to the Bacille Calmette-Guerin (BCG) may mitigate the pandemic impact in some countries and the BCG vaccine offers significant advantages and flexibility in the way clinical vaccines are administered. BCG vaccination is a highly cost-effective intervention against tuberculosis (TB) and many low-and lower-middle-income countries would likely have the infrastructure, and health care personnel sufficiently familiar with the conventional TB vaccine to mount full-scale efforts to administer novel BCG-based vaccine for COVID-19. This suggests the potential for BCG to overcome future barriers to vaccine roll-out in the countries where health systems are fragile and where the effects of this new coronavirus could be catastrophic. Many studies have reported cross-protective effects of the BCG vaccine toward non-tuberculosis related diseases. Mechanistically, this cross-protective effect of the BCG vaccine can be explained, in part, by trained immunity, a recently discovered program of innate immune memory, which is characterized by non-permanent epigenetic reprogramming of macrophages that leads to increased inflammatory cytokine production and consequently potent immune responses. In this review, we summarize recent work highlighting the potential use of BCG for the treatment respiratory infectious diseases and ongoing SARS-CoV-2 clinical trials. In situations where no other specific prophylactic tools are available, the BCG vaccine could be used as a potential adjuvant, to decrease sickness of SARS-CoV-2 infection and/or to mitigate the effects of concurrent respiratory infections.

Gralnek, I. M., et al. (2020). "ESGE and ESGENA Position Statement on gastrointestinal endoscopy and the COVID-19 pandemic." Endoscopy **52**(6): 483-490.

We are currently living in the throes of the COVID-19 pandemic that imposes a significant stress on health care providers and facilities. Europe is severely affected with an exponential increase in incident infections and deaths. The clinical manifestations of COVID-19 can be subtle, encompassing a broad spectrum from asymptomatic mild disease to severe respiratory illness. Health care professionals in endoscopy units are at increased risk of infection from COVID-19. Infection prevention and control has been shown to be dramatically effective in assuring the safety of both health care professionals and patients. The European Society of Gastrointestinal Endoscopy ( www.esge.com ) and the European Society of Gastroenterology and Endoscopy Nurses and Associates ( www.esgena.org ) are joining forces to provide guidance during this pandemic to help assure the highest level of endoscopy care and protection against COVID-19 for both patients and endoscopy unit personnel. This guidance is based upon the best available evidence regarding assessment of risk during the current status of the pandemic and a consensus on which procedures to perform and the priorities on resumption. We appreciate the gaps in knowledge and evidence, especially on the proper strategy(ies) for the resumption of normal endoscopy practice during the upcoming phases and end of the pandemic and therefore a list of potential research questions is presented. New evidence may result in an updated statement.

Granados-Montiel, J., et al. (2021). "New prophylaxis regimen for SARS-CoV-2 infection in health professionals with low doses of hydroxychloroquine and bromhexine: a randomised, double-blind placebo clinical trial (ELEVATE Trial)." BMJ Open **11**(8).

Introduction SARS-CoV-2 infection in Mexico has caused similar to 2.7 million confirmed cases; around 20%-25% of health workers will be infected by the virus at their workplace, with approximately 4.4% of mortality. High infectivity of SARS-CoV-2 is related with cell entry mechanism, through the ACE receptor. SARS-CoV-2 requires transmembrane protease serine 2 to cleave its spike glycoprotein and ensure fusion of host cell and virus membrane. We propose studying prophylactic treatment with hydroxychloroquine (HCQ) and bromhexine (BHH), which have been shown to be effective in preventing SARS-CoV-2 infection progression when administered in early stages. The aim of this study is to assess the efficacy of HCQ and BHH as prophylactic treatments for SARS-CoV-2 infection in healthy health workers exposed to the virus. Methods and analysis Double-blind randomised clinical trial, with parallel allocation at a 1:1 ratio with placebo, of low doses of HCQ plus BHH, for 60 days. Study groups will be defined as follows: (1) HCQ 200 mg/day+BHH 8 mg/8 hours versus (2) HCQ placebo plus BHH placebo. Primary endpoint will be efficacy of both interventions for the prevention of SARS-CoV-2 infection, determined by the risk ratio of infected personnel and the absolute risk. At least a 16% reduction in absolute risk is expected between the intervention and placebo groups; a minimum of 20% infection is expected in the placebo group. The sample size calculation estimated a total of 214 patients assigned: two groups of 107 participants each. Ethics and dissemination This protocol has been approved by the local Medical Ethics Committee (National Institute of Rehabilitation 'Luis Guillermo Ibarra Ibarra', approval number INRLGII/25/20) and by the Federal Commission for Protection against Sanitary Risks (COFEPRIS, approval number 203 300 410A0058/2020). The results of the study will be submitted for publication in peer-reviewed journals and disseminated through conferences.

Gribble, K., et al. (2020). "Mistakes from the HIV pandemic should inform the COVID-19 response for maternal and newborn care." International Breastfeeding Journal **15**(1).

Background In an effort to prevent infants being infected with SARS-CoV-2, some governments, professional organisations, and health facilities are instituting policies that isolate newborns from their mothers and otherwise prevent or impede breastfeeding. Weighing of risks is necessary in policy development Such policies are risky as was shown in the early response to the HIV pandemic where efforts to prevent mother to child transmission by replacing breastfeeding with infant formula feeding ultimately resulted in more infant deaths. In the COVID-19 pandemic, the risk of maternal SARS-CoV-2 transmission needs to be weighed against the protection skin-to-skin contact, maternal proximity, and breastfeeding affords infants. Conclusion Policy makers and practitioners need to learn from the mistakes of the HIV pandemic and not undermine breastfeeding in the COVID-19 pandemic. It is clear that in order to maximise infant health and wellbeing, COVID-19 policies should support skin-to-skin contact, maternal proximity, and breastfeeding.

Griffin, M., et al. (2021). "Preparing for COVID-19 exit strategies." Annals of Medicine and Surgery **61**: 88-92.

The COVID-19 pandemic has affected 20 million people worldwide with over 732,000 deaths and trillions of dollars of lost economic productivity. It has put many countries into lockdown to contain the virus and save lives. As COVID-19 cases in some countries start to plateau and societies work hard to 'flatten the curve', leaders are being asked to formulate plans for safe and staged 'exit strategies' to reopen society. Each country will decide on their own exit strategy but many plans are considering similar vital healthcare principles including the maintenance of social distancing to prevent ongoing community transmission, testing capacity, protection of the healthcare systems and the health of their care workers. This review aims to provide an overview of essential factors that plans for exit strategy should consider and their effect on the societies' social and healthcare life.

Grote, U., et al. (2021). "Measures to cope with the COVID-19 pandemic in Germany: nonpharmaceutical and pharmaceutical interventions." Bundesgesundheitsblatt-Gesundheitsforschung-Gesundheitsschutz **64**(4): 435-445.

When the emerging novel SARS-CoV-2 virus first appeared in December 2019, neither specific therapeutic options nor vaccinations were available. The role of nonpharmaceutical interventions (NPIs) became of central importance. At the Robert Koch Institute, a multilayer strategy consisting of population-based and individual preventive measures to control the pandemic was developed, which built upon existing influenza pandemic plans as well as generic plans. This paper explains the recommended NPIs and illustrates the pharmaceutical approaches developed in parallel. Among others, general contact bans, providing material for infection prevention and control, ban of events, closing educational institutions, and restricting travel are counted among population-based measures. Additional individual preventive measures are necessary, e.g., keeping a minimum distance, reducing contacts, and wearing a mouth-nose covering as well as quarantine and isolation. Measures within the health system are based on recommendations of the Commission on Hospital Hygiene and Infection Protection (Kommission fur Krankenhaushygiene und Infektionspravention (KRINKO)) and specified and implemented by professional societies. Since November 2020, an antiviral therapy with remdesivir and treatment with the glucocorticoid dexamethasone have been available as pharmaceutical interventions. Monoclonal antibodies are at this time not approved. Therapeutic anticoagulation is recommended. Recommendations are constantly adapted to the increasing knowledge on the pathogen and its means of transmission. A challenge is to strengthen the trust of the population. Many measures have to be applied on an individual basis in order to work together.

Guadalupe, S., et al. (2021). "Resilient Coping and Burnout in Healthcare Social Workers during the First Wave of the COVID-19 Pandemic in Portugal and Spain." Mundo Da Saude **45**(1): 517-532.

Healthcare social workers faced a demanding professional challenge during the COVID-19 pandemic, which had multidimensional repercussions. Studies with health professionals concerning Burnout and resilience strategies have been considered relevant and gained special attention for their position in the response to the pandemic. Here we highlight the risks of Burnout and the potential protection of coping strategies in a profession that mediates the social dimension of health and disease. We aimed to understand their impact on social workers who worked in the health field in Portugal and Spain during the first wave, analyzing the levels of resilience and burnout. 336 social workers from Portugal (n = 252) and Spain (n = 84) participated. Despite the high perception of multidimensional stressors (at a personal, relational, professional, and organizational level), burnout risks were moderate to low. The predisposition to burnout due to depersonalization was residual (1.8%), but that due to low personal accomplishment (18.8%) and emotional exhaustion (21.4%) were higher; however, the majority did not show any risk of Burnout. The levels of resilience demonstrated facing the COVID-19 Pandemic were low (47%) or moderate (29.8%), with 23.2% at high levels. The aggregation of Burnout and coping strategies into three risk profiles reveals differences in the perception of fear of implications in working with patients, the impact felt at work, and the support felt at the health unit. Support and resilience strategies that favor the psychosocial and professional well-being of healthcare social workers must be promoted, especially in emergencies.

Guclu, D. and E. N. Unlu (2020). "Duzce University Faculty of Medicine Radiology Department in the COVID-19 Pandemic Process." Konuralp Tip Dergisi **12**: 378-380.

Radiology plays an important role in the COVID-19 pandemic outbreak. Besides its role in the diagnosis and follow-up of COVID-19, it undertakes important tasks both in the protection of healthcare workers and in avoiding a potential cause of spread. The aim of the present study is to share our experience and approach during the pandemic and to contribute to the management of other probable pandemic processes.

Guevara-Hoyer, K., et al. (2021). "Serological Tests in the Detection of SARS-CoV-2 Antibodies." Diagnostics **11**(4).

Early detection of SARS-CoV-2 is essential for a timely update of health policies and allocation of resources. Particularly, serological testing may allow individuals with low-risk of being contagious of SARS-CoV-2 to return to daily activities. Both private and academic initiatives have sought to develop serological assays to detect anti-SARS-CoV-2 antibodies. Herein, we compared five different assays in active healthcare personnel exposed to SARS-CoV-2 in a large center in Madrid, Spain, in a retrospective study. Median time lapse between polymerase chain-reaction (PCR) and serological testing was 11 days (7-21). All tests assessed IgM/IgG titers except for Euroimmun (IgA/IgG) and The Binding-Site (IgA/IgM/IgG). The highest concordance rate was observed between Dia.Pro and Euroimmun (75.76%), while it was lowest between The Binding-Site and Euroimmun (44.55%). The Binding-Site assay showed the highest concordance (85.52%) with PCR results. Considering PCR results as reference, Dia.Pro was the most sensitive test, although The Binding-Site assay exhibited the highest area under the curve (AUC; 0.85). OrientGene and MAGLUMI tests were performed in a smaller cohort with confirmed infection and thus were not adequate to estimate sensitivity and specificity. The Binding-Site assay presented the best joint sensitivity and specificity among all the tests analyzed in our cohort. Likewise, this serological assay presents a greater repertoire of antibodies and antigen-regions tested, which is why each individual's humoral immunity is more accurately reflected. The better the immunity test, the most adequate the health strategy to take in terms of organization of consultations, surgery, and treatments in vulnerable patients. The three antibody classes (IgG/IgM/IgA) were determined jointly, which translates to an economic impact on healthcare. While their role in the protection status remains elusive, serological tests add a valuable tool in the early management of SARS-CoV-2 after known exposition.

Habchi, K., et al. (2020). "COVID-19 Crisis: International Transfers of Critical Ill Patients, a Solution for Border Counties." Annales Francaises De Medecine D Urgence **10**(4-5): 298-305.

The COVID-19 health crisis has imposed an unprecedented adaptability of our health system. Intensive care units and emergency departments had to innovate and develop new strategies to guarantee quality care for patients needing intensive care. The "Grand-Est" region and particularly the counties of Moselle, Bas-Rhin and Haut-Rhin, were strongly impacted. Among the 349 patients transferred out of region, 164 were transferred to European countries (Austria, Germany, Luxembourg, and Switzerland) between March 14 and April 4, 2020. These international transfers, organized by the regional health agency and the emergency medical call centers, mainly use flying ambulances belonging to hospitals, army, and civil protection. The support of these patients was provided by a specialized physiciannurse team. The choice of this strategy made it possible to avoid saturation of intensive care units and to make a choice between patients admitted or not in intensive care. Adaptability of health care providers and an organization as close as possible to the field allowed these transfers to be carried out under good conditions. This episode demonstrates the importance of leaving the management of health crisis to health professionals. This article presents the organization set up to prepare and carry out these international transfers.

Hamedani, S. and N. Farshidfar (2020). "The practice of oral and maxillofacial radiology during COVID-19 outbreak." Oral Radiology **36**(4): 400-403.

The current coronavirus disease 2019 (COVID-19) outbreak has brought substantial challenges to the world health system, including the practice of dental and maxillofacial radiology (DMFR). DMFR will carry on an imperative role in healthcare during this crisis. This rapid communication has collected and evaluated all the best current evidence and published guidelines as well as professional recommendations to help maxillofacial radiologists and dental practitioners for safer radiological and imaging examinations on healthy, suspected, or confirmed COVID-19 patients during outbreak. Some strategies have been depicted including procedural indications, infection control, and correct employment of personal protection equipment along with evoking the proper practice environment during and after the COVID-19 outbreak.

Hao, P., et al. (2020). "Xi Jin, China CDC's Chief Expert of Maternal and Child Health." China Cdc Weekly **2**(28): 534-535.

Editorial Our new column Profiles is a new series that aims to introduce China CDC's chief experts, major decisionmakers, and key contributors that ensure the high standard of operation of China CDC. These articles are meant to present high-achieving individuals of China CDC and will describe the background, achievements, and paths to success of the eponymous individuals. To start, Profiles will first cover individuals from China CDC's Chief Expert System. To strengthen the establishment of a team of public health talent, China CDC expanded the career development capacity for its most well-known experts under Director-General George F. Gao's directive: "To further perfect the system of chief experts and continuously expand the influence of chief experts". In December 2019, China CDC selected the nominations of chief experts based on the needs of key areas of public health and the academic level and professional influence of the experts. After domestic peer review and comprehensive evaluation by China CDC's academic committee, 13 individuals (in no particular order) were appointed to lead 13 major public health fields: 1) Guizhen Wu, Chief Expert of Biosafety; 2) Yiming Shao, Chief Expert of AIDS; 3) Weiping Wu, Chief Expert of Parasitic Diseases; 4) Huaqing Wang, Chief Expert of Immunization Planning; 5) Zunyou Wu, Chief Expert of Epidemiology; 6) Linhong Wang, Chief Expert of Chronic Diseases; 7) Xu Su, Chief Expert of Radiation Protection; 8) Wenhua Zhao, Chief Expert of Nutrition; 9) Tao Li, Chief Expert of Occupational Health; 10) Liubo Zhang, Chief Expert of Disinfection; 11) Jiaqi Ma, Chief Expert of Health Informatics; 12) Xi Jin, Chief Expert of Maternal and Child Health; and 13) Qiyong Liu, Chief Expert of Vector Biology. In response to the prevention and control of the coronavirus disease 2019 (COVID-19) pandemic, the chief experts were dispatched to the front lines and highly-affected areas to supervise the response, tasked with leading scientific research to shape the response and the preparation of technical documents for prevention and control, and were responsible for promptly supporting government decisionmaking and responding to social concerns as the scientific authorities. The voices of these chief experts has played an important role in the effective management of the epidemic in their respective professional fields. China CDC plans to dynamically adjust the fields represented by the chief experts based on the country's major public health needs by enriching the chief expert's system and guiding the chief experts to play the role of "public opinion leaders" in academic communication and health communication at home and abroad. Our first publication in Profiles, therefore, is China CDC's Chief Expert of Maternal and Child Health, Jin Xi, who has over 30 years of experience in developing and managing maternal and child healthcare (MCH) institutions. With the advent of the 31st World Population Day on July 11, 2020, the public health community needs to prioritize MCH as a fundamental basis for the improvement of health globally.

Hasmi, A. H., et al. (2020). "The craniotomy box: an innovative method of containing hazardous aerosols generated during skull saw use in autopsy on a COVID-19 body." Forensic Science Medicine and Pathology **16**(3): 477-480.

During a disease pandemic, there is still a requirement to perform postmortem examinations within the context of legal considerations. The management of the dead from COVID-19 should not impede the medicolegal investigation of the death where required by the authorities and legislation but additional health and safety precautions should be adopted for the necessary postmortem procedures. The authors have therefore used the craniotomy box in an innovative way to enable a safe alternative for skull and brain removal procedures on suspected or confirmed COVID-19 bodies. The craniotomy box technique was tested on a confirmed COVID-19 positive body where a full postmortem examination was performed by a team of highly trained personnel in a negative pressure Biosafety Level 3 (BSL-3) autopsy suite in the National Institute of Forensic Medicine (IPFN) Malaysia. This craniotomy box is a custom-made transparent plastic box with five walls but without a floor. Two circular holes were made in one wall for the placement of arms in order to perform the skull opening procedure. A swab to detect the presence of the SARS-CoV-2 virus was taken from the interior surface of the craniotomy box after the procedure. The result from the test using real-time reverse transcriptase polymerase chain reaction (rRT-PCR) proved that an additional barrier provided respiratory protection by containing the aerosols generated from the skull opening procedure. This innovation ensures procedures performed inside this craniotomy box are safe for postmortem personnel performing high risk autopsies during pandemics.

Herzog, M., et al. (2021). "The first year of the SARS-CoV-2 pandemic-impact on otorhinolaryngology." Hno **69**(8): 615-622.

Background The first year of the severe acute respiratory syndrome coronavirus 2 (SARS-CoV-2) pandemic has already affected our public health care system to an enormous extent and will continue to do so in the future. Otorhinolaryngologists (ORLs) are suspected to be at high risk of infection, due to the high viral load in the mucosa of the upper airways. The current review evaluates the impact of the pandemic on ORLs' activities and assesses the risk infection. Methods A selective literature research was conducted using relevant English and German terms for ORL, SARS-CoV-2, risk, and infection at PubMed, medRxiv, and bioRxiv, as well as in the Deutsches arzteblatt and on the websites of the Robert Koch Institute and the Johns Hopkins University. Results Protection recommendations for ORL include general hygiene measures and wearing KN95 masks for routine professional activities. When in contact with coronavirus disease 2019 (COVID-19) patients, it is recommended to extend the personal protective equipment by eye protection, gloves, cap, and gown. International otorhinolaryngology societies have released guidelines for procedures (e.g., tracheostomy, sinus surgery), propagating personal protection for the surgical team and reduction of aerosols. Testing for SARS-CoV-2 in patients and medical staff can contribute to reducing the risk of infection. Vaccination would provide some additional protection for ORLs and other health care professionals with increased exposure to aerosols. There is increasing evidence that ORLs are at a high risk of contracting SARS-CoV-2. Conclusion Consequent personal protection, frequent testing of patients and health care professionals, and the promised SARS-CoV-2 vaccinations may provide adequate protection for highly exposed persons.

Hou, F. F., et al. (2020). "Personnel protection strategy for healthcare workers in Wuhan during the COVID-19 epidemic." Precision Clinical Medicine **3**(3): 169-174.

Objective: To identify the effectiveness of a personnel protection strategy in protection of healthcare workers from SARS-CoV-2 infection. Design: During the COVID-19 pandemic, 943 healthcare staff sent from Guangzhou to Wuhan to care for patients with suspected/confirmed COVID-19 received infection precaution training before their mission and were equipped with Level 2/3 personal protective equipment (PPE), in accordance with guidelines from the National Health Commission of China. We conducted a serological survey on the cumulative attack rate of SARS-CoV-2 among the healthcare workers sent to Wuhan and compared the seropositive rate to that in local healthcare workers from Wuhan and Jingzhou. Results: Serial tests for SARS-CoV-2 RNA and tests for SARS-CoV-2 immunoglobulin M and G after the 6-8 week mission revealed a zero cumulative attack rate. Among the local healthcare workers in Wuhan and Jingzhou of Hubei Province, 2.5% (113 out of 4495) and 0.32% (10 out of 3091) had RT-PCR confirmed COVID-19, respectively. The seropositivity for SARS-CoV-2 antibodies (IgG, IgM, or both IgG/IgM positive) was 3.4% (53 out of 1571) in local healthcare workers from Wuhan with Level 2/3 PPE working in isolation areas and 5.4% (126 out of 2336) in healthcare staff with Level 1 PPE working in non-isolation medical areas, respectively. Conclusions and relevance: Our study confirmed that adequate training/PPE can protect medical personnel against SARS-CoV-2.

Hou, H., et al. (2021). "Factors Associated with Turnover Intention Among Healthcare Workers During the Coronavirus Disease 2019 (COVID-19) Pandemic in China." Risk Management and Healthcare Policy **14**: 4953-4965.

Purpose: This study assessed the prevalence of turnover intention and explored associated factors on turnover intention among healthcare workers during the COVID-19 pandemic in China. Methods: An institutional-based cross-sectional study was conducted from July to February 13th to 20th, 2020, in 31 provinces of mainland China. A total of 1403 healthcare workers were recruited. Hierarchical logistic regressions were used to identify potential factors associated with turnover intention among Chinese health care workers. Results: The prevalence of turnover intention among healthcare workers was 10.1% during the COVID-19 pandemic in China. Results of hierarchical regression revealed that working in Grade II hospital (OR = 1.78), technician (OR = 0.30), daily working hours over 12 h (OR = 2.92), frequency of mask replacement between 4 and 8 h (OR = 3.51), refuse volunteer to frontline (OR = 1.68), patient-physician relation unchanged (OR = 1.73), depression (OR = 2.21) and lower social support (OR = 1.75) were significantly associated with the risk of turnover intention. Additionally, healthcare worker's psychosocial syndemic (OR = 6.13) was positively associated with turnover intention. Conclusion: Turnover intention is relatively prevalent among healthcare workers during the COVID-19 pandemic in China, and the factors contributing to turnover intention were complex and varied. Early screening of high-risk groups for turnover intention among healthcare workers and more psychosocial health care and physical protection are needed during the COVID-19 pandemic in China.

Hrevtsova, R. Y. (2020). "INSTITUTIONAL AND LEGAL ASPECTS OF HEALTH CARE IN TIMES OF COVID-19: LEARNING FROM THE UKRAINIAN EXPERIENCE." Medicine and Law **39**(2): 333-350.

The paper discusses the changes in the Ukrainian institutional and legal landscape that have become critical with regard to the novel coronavirus disease (COVID-19) outbreak. Ukraine has faced the pandemic during the reforms of its healthcare system and others. Some of the institutional changes, aimed at resolving the burning issues and fulfilling political commitments (such as liquidation of the notorious health system institution with a branched structure, still responsible for the epidemiological safety, financing infectious disease units for the actually provided services) have shown underperformance, particularly in light of healthcare emergency preparedness. Some other changes (including introduction of medical self-governance) require boosting. The coronavirus crisis should serve as an impulse to changing the attitudes of the state and society towards healthcare workers who currently have to reclaim their rights, especially the rights to safe working conditions and proper remuneration and ensuring due protection and respect. Special attention shall be paid to legal substantiation of the development of innovations. This primarily relates to the use of telemedicine tools which may be essential for ensuring access to healthcare, both in everyday life and emergencies.

Hsieh, T. Y., et al. (2020). "A Guide to Facial Trauma Triage and Precautions in the COVID-19 Pandemic." Facial Plastic Surgery & Aesthetic Medicine **22**(3): 164-169.

COVID-19 is an emerging viral illness that has rapidly transmitted throughout the world. Its impact on society and the health care system has compelled hospitals to quickly adapt and innovate as new information about the disease is uncovered. During this pandemic, essential medical and surgical services must be carried out while minimizing the risk of disease transmission to health care workers. There is an elevated risk of COVID-19 viral transmission to health care workers during surgical procedures of the head and neck due to potential aerosolization of viral particles from the oral cavity/naso-oropharynx mucosa. Thus, patients with facial fractures pose unique challenges to the variety of injuries and special considerations, including triaging injuries and protective measures against infection. The proximity to the oral cavity/naso-oropharyngeal mucosa, and potential for aerosolization of secretions containing viral particles during surgical procedures make most patients undergoing operative interventions for facial fractures high risk for COVID-19 transmission. Our proposed algorithm aims to balance patient care with patient/medical personnel protection as well as judicious health care utilization. It stratifies facial trauma procedures by urgency and assigns a recommended level of personal protective equipment, extreme or enhanced, incorporating current best practices and existing data on viral transmission. As this pandemic continues to evolve and more information is obtained, the protocol can be further refined and individualized to each institution.

Huang, X. L., et al. (2021). "Novel combined shield design for eye and face protection from COVID-19." Advances in Manufacturing **9**(1): 130-135.

The World Health Organization emphasized the importance of goggles and face shields for protection of medical personnel at the outbreak of the COVID-19 pandemic. Unsurprisingly, almost all countries suffered from a critical supply shortage of goggles and face shields, as well as many other types of personal protective equipment (PPE), for a long period, owing to the lack of key medical material supplies and the inefficiency of existing fabrication methods arising from the need to avoid crowds during the outbreak of COVID-19. In this paper, we propose a novel combined shield design for eye and face protection that can be rapidly fabricated using three-dimensional printing technology. The designed prototype eye-face shield is accessible to the general public, offering more possibilities for yield improvement in PPE during emergent infectious disease events such as COVID-19.

Hubert, K., et al. (2020). "Acute coronary syndromes and COVID-19 pandemic." Journal Fur Kardiologie **27**(5): 139-145.

The COVID-19 pandemic poses a threat to patients with acute coronary syndromes (ACS) and interventional cardiologists as well as other healthcare workers. The number of COVID-19 positive or suspected positive patients requiring hospital admission has overwhelmed many health systems and negatively affected standard of care for ACS patients in these countries. This manuscript aims to assist cardiologists in the invasive management of acute coronary syndrome (ACS) patients in the context of the COVID-19 pandemic. Modified diagnostic and treatment algorithms, which have been developed in countries heavily beaten by this unpredictable challenge, are discussed as are various clinical scenarios and management algorithms for patients with a confirmed or suspected COVID-19 infection presenting with ST segment elevation myocardial infarction (STEMI) or non-ST elevation ACS (NSTE-ACS). Further described topics include the need for re-organization of pre-hospital (STEMI networks) and in-hospital structures (emergency rooms and cardiac units), with examples coming from multiple European countries. Finally, this manuscript aims to help re-organizing of catheterization laboratory structures and personnel and to discuss measures for protection of healthcare providers involved with invasive procedures.

Hussain, R., et al. (2021). "Drive-thru pharmacy services: A way forward to combat COVID-19 pandemic." Research in Social & Administrative Pharmacy **17**(1): 1920-1924.

Countries around the globe have responded to pandemic preparedness and developed strategies to cope with the COVID-19 crisis. In this context, the role of healthcare professionals is of paramount importance. Pharmacists are playing a vital role in dealing, preparedness, prevention, protection, promoting access to medicines and to improve health outcomes during this crisis. In this context, "Drive-thru" pharmacy services improve access to medicines while ensuring the preventive measures suggested by the World Health Organization. This commentary provides an overview of opportunities and challenges related to the implementation of "drive-thru pharmacy services" and their role in improving public health during this crisis.

Insua, C., et al. (2020). "Seroprevalence of SARS-CoV-2 antibodies among physicians from a children's hospital." Archivos Argentinos De Pediatria **118**(6): 381-385.

Introduction. In just a few months, coronavirus disease 2019 (COVID-19) has become a pandemic, causing millions of cases and hundreds of thousands of deaths. Due to its high infectiousness, the health care staff is even more exposed. Nevertheless, there is still very limited information about it, especially among pediatricians. Objective. To assess the seroprevalence of SARS-CoV-2 antibodies among physicians from a children's hospital 3 months after the first case in Argentina. To explore if certain demographic, occupational, and epidemiological characteristics are risk factors for SARS-CoV-2 seropositivity. Methods. Cross-sectional study of physicians randomly selected from the list of workers of a children's hospital. All of them were screened for SARS-CoV-2 antibodies by chemiluminescence (immunoglobulins G/M [IgG/IgM]) and their epidemiological history was collected (age, place of residence, professional category, contact with infected people)in order to explore the association between predictors and seropositivity. Results. A total of 116 physicians were included; their average age was 45.6 +/- 13.3 years. Of them, 62.9% were staff physicians, and the rest, residents; 42.2 % worked in areas designated for direct care of COVID-19 patients. Four (35 %)referred contact with infected people without the appropriate protection, and 6 (5.2 %), having traveled to risk areas. SARS-CoV-2 antibodies (IgG) were only detected in one of the professionals. Because of this, it was impossible to assess the association between potential predictors and the presence of SARS-CoV-2 antibodies. Conclusion. It was observed that only 0.9 % of the physicians from a children's hospital had SARS-CoV-2 antibodies.

Iorga, M., et al. (2021). "How the SARS-CoV-2 Pandemic Period Influenced the Health Status and Determined Changes in Professional Practice among Obstetrics and Gynecology Doctors in Romania." Medicina-Lithuania **57**(4).

Background and Objectives: The beginning of the SARS-Cov-2 pandemic period has had a strong impact on patients' life, but also on doctors. The main goal of this research is to identify the difficulties related to the professional activity and personal life of obstetrics and gynecology doctors. Material and Methods: In total, 94 physicians from a single university center answered to an online questionnaire. Socio-demographic, health, family, and job-related data were collected. Data were processed using SPSS (v.25). Results: 7.4% of the doctors were confirmed infected with SARS-Cov-2 during the first 6 months of the pandemic, and 48.94% treated infected patients. Due to the large number of patients, 10.64% of the doctors have had no days-off during the last 6 months, and 22.34% of them have had new medical problems that led them to see a specialist. Seventeen to nineteen percent mentioned an increasing number of working hours and shifts per month due to the pandemic period, more than 10% used pills to cope with work-stress, and 25% of them had sleep disorders along with appetite loss. Extra-protection rules and negative consequences of wearing special equipment were identified: thermal discomfort that caused decreasing resistance and concentration during the surgery (52%), reduced mobility and accuracy of surgical or medical gestures (40%), and intraoperative visibility (47%). Doctors who were working with confirmed pregnant women preferred caesarean section. Conclusions: Working under the stress of an infection with SARS-Cov-2 is causing a lot of pressure and determines changes in personal, familial, social, and professional life. Understanding the challenges that ob-gyn doctors are facing will help institutions to better provide support.

Iqbal, M. R. and A. Chaudhuri (2020). "COVID-19: Results of a national survey of United Kingdom healthcare professionals' perceptions of current management strategy - A cross-sectional questionnaire study." International Journal of Surgery **79**: 156-161.

Objective: COVID-19 has caused a global healthcare crisis with increasing number of people getting infected and dying each day. Different countries have tried to control its spread by applying the basic principles of social distancing and testing. Healthcare professionals have been the frontline workers globally with different opinions regarding the preparation and management of this pandemic. We aim to get the opinion of healthcare professionals in United Kingdom regarding their perceptions of preparedness in their workplace and general views of current pandemic management strategy. Method: A questionnaire survey, drafted using Google Forms, was distributed among healthcare professionals working in the National Health Service (NHS) across the United Kingdom. The study was kept open for the first 2 weeks of April 2020. Results: A total of 1007 responses were obtained with majority of the responses from England (n = 850, 84.40%). There were 670 (66.53%) responses from doctors and 204 (20.26%) from nurses. Most of the respondents (95.23%) had direct patient contact in day to day activity. Only one third of the respondents agreed that they felt supported at their trust and half of the respondents reported that adequate training was provided to the frontline staff. Two-thirds of the respondents were of the view that there was not enough Personal Protective Equipment available while 80% thought that this pandemic has improved their hand washing practice. Most of the respondents were in the favour of an earlier lockdown (90%) and testing all the NHS frontline staff (94%). Conclusion: Despite current efforts, it would seem this is not translating to a sense of security amongst the UK NHS workforce in terms of how they feel trained and protected. It is vital that healthcare professionals have adequate support and protection at their workplace and that these aspects be actively monitored.

Izquierdo-Dominguez, A., et al. (2021). "Management of Allergic Diseases During COVID-19 Outbreak." Current Allergy and Asthma Reports **21**(2).

Purpose of ReviewThe coronavirus disease 2019 (COVID-19) has challenged healthcare system capacities and safety for health care workers, reshaping doctor-patient interaction favoring e-Health or telemedicine. The pandemic situation may make difficult to prioritize patients with allergies diseases (AD), face-to-face evaluation, and moreover concern about the possible COVID-19 diagnosis, since COVID-19 shared many symptoms in common with AD. Being COVID-19 a novel disease, everyone is susceptible; there are some advances on vaccine and specific treatment. We evaluate existing literature on allergic diseases (AD): allergic rhinitis, asthma, food allergy, drug allergy, and skin allergy, and potential underlying mechanisms for any interrelationship between AD and COVID-19.Recent FindingsThere is inconclusive and controversial evidence of the association between AD and the risk of adverse clinical outcomes of COVID-19. AD patients should minimize hospital and face-to-face visits, and those who have used biologics and allergen immunotherapy should continue the treatment. It is essential to wear personal protective equipment for the protection of health care workers.SummarySocial distancing, rational use of facemasks, eye protection, and hand disinfection for health care workers and patients deserve further attention and promotion. Teleconsultation during COVID-19 times for AD patients is very encouraging and telemedicine platform can provide a reliable service in patient care.

Jain, S., et al. (2022). "Strategy for repurposing of disposed PPE kits by production of biofuel: Pressing priority amidst COVID-19 pandemic." Biofuels-Uk **13**(5): 545-549.

COVID-19, a disease caused by severe acute respiratory syndrome-corona virus-2 (SARS-CoV-2) has taken the lives of millions of people across the globe. Researchers are working on the development of different aids to cure and prevent the disease. Personal protection equipment (PPE) has become an essential prerequisite for healthcare workers. PPE kits are reported to provide sufficient protection against pathogens but their disposal can be devastating to environment. The proper disposal of PPE is being taken care of by the national authorities according to the guidelines provided by WHO.The plethora of disposed PPE kits will further increase the burden of polymer on our earth.This review presents a strategy to dispose the PPE kits by their conversion to alternative fuel.

Jamieson, D. J., et al. (2020). "Obstetricians on the Coronavirus Disease 2019 (COVID-19) Front Lines and the Confusing World of Personal Protective Equipment." Obstetrics and Gynecology **135**(6): 1257-1263.

As health care systems struggle to maintain adequate supplies of personal protective equipment, there is confusion and anxiety among obstetricians and others about how to best protect themselves, their coworkers, and their patients. Although use of personal protective equipment is a critical strategy to protect health care personnel from coronavirus disease 2019 (COVID-19), other strategies also need to be implemented on labor and delivery units to reduce the risk of health care-associated transmission, including screening of all pregnant women who present for care (case identification), placing a mask on and rapidly isolating ill pregnant women, and minimizing the number of personnel who enter the room of an ill patient (physical distancing). Although the mechanism of transmission of COVID-19 is not known with certainty, current evidence suggests that COVID-19 is transmitted primarily through respiratory droplets. Therefore, strict adherence to hand hygiene and consistent use of recommended personal protective equipment are cornerstones for reducing transmission. In addition, it is critical that health care professionals receive training on and practice correct donning (putting on) and doffing (removing) of personal protective equipment and avoid touching their faces as well as their facial protection to minimize self-contamination.

Janik, E., et al. (2021). "SARS-CoV-2: Outline, Prevention, and Decontamination." Pathogens **10**(2).

The new coronavirus began to spread around the world in late 2019. Initially, it was found only in China, but in the following days there were reported cases of infections in other countries. Subsequently, based on taxonomy, phylogeny, and accepted practice, the virus was officially designated as severe acute respiratory syndrome coronavirus 2 (SARS-CoV-2). As a result of the rapid spread of SARS-CoV-2 in different countries around the world, on March 11, 2020, the World Health Organization (WHO) announced a status change in the disease caused by this coronavirus-from an epidemic to a pandemic disease. Although the world is taking unprecedented efforts to control the spread of SARS-CoV-2, the number of confirmed cases is rising. Therefore, effective preventive measures are needed in order to limit the spread of illness. The prevention measures are mainly based on information on the virus transmission routes, its environmental stability, and persistence on commonly touched surfaces. Social distancing, mask usage, and good hygiene practice are the most important recommendations for general public. Healthcare professionals who are directly involved in SARS-CoV-2 patients care are more exposed to virus infection and additional protection measures are necessary, including protective suits, aprons, face shields, goggles, and gloves. Due to the stability of SARS-CoV-2 on different surfaces, such as glass, paper, or wood, proper disinfection is crucial. Several studies have shown that despite the virus's stability, it is sensitive to various disinfectants, such as ethanol, isopropanol, sodium hypochlorite, or hydrogen peroxide. These findings underline the importance of having comprehensive knowledge about SARS-CoV-2 and multidirectional strategies in order to limit the spread of the virus. This review is a summary of the most important information about SARS-CoV-2, such as its stability on different surfaces, protection strategies, and decontamination options.

Jastrzebska, A. M. and A. S. Vasilchenko (2021). "Smart and Sustainable Nanotechnological Solutions in a Battle against COVID-19 and Beyond: A Critical Review." Acs Sustainable Chemistry & Engineering **9**(2): 601-622.

The variety of available biocidal features make nanomaterials promising for fighting infections. To effectively battle COVID-19, categorized as a pandemic by the World Health Organization (WHO), materials scientists and biotechnologists need to combine their knowledge to develop efficient antiviral nanomaterials. By design, nanostructured materials (spherical, twodimensional, hybrid) can express a diverse bioactivity and unique combination of specific, nonspecific, and mixed mechanisms of antiviral action. It can be related to the material's specific features and their multiple functionalization strategies. This is a complex guiding approach in which an interaction target is constantly moving and quickly changing. On the other hand, in such a rush, sustainability may be put aside. Therefore, to elucidate the most promising nanotechnological solutions, we critically review available data within the frame of severe acute respiratory syndrome coronavirus 2 (SARS-CoV-2) and other types of viruses. We highlight solutions that are, or could be, more sustainable and less toxic. In this regard, reduction of the number of synthetic routes, organic solvents, byproducts, and residues is highly recommended. Such efficient, green solutions may be further used for the prevention of virion-host interactions, treatment of the already developed infection, reducing inflammation, and finally, protecting healthcare professionals with masks, fabrics, equipment, and in other associated areas. Further translation into the market needs putting on the fast track with respect to principles of green chemistry, feasibility, safety, and the environment.

Jorge, C. (2021). "Landscape intervention in the Hospital for Covid-19 pandemic in Madrid." Landscape Architecture and Art **18**(18): 39-48.

From Design with Nature of Ian L. McHarg to The Origin of Eukaryotic Cells of Lynn Margulis, the role of the microorganisms in the cycle of life, health, and disease, and in climate change constitutes the life support system of the biosphere. The micro-parklands of the Emergency Hospital in Madrid create a natural system of prefabricated elements following the aim of rapid implementation, isolation, and protection taking as reference the simple integrated system of living microorganisms. These micro-enclosures provide circular areas where patients, visitors, or healthcare professionals can meet in secret places surrounded by trees and shrubs or long green islands where they can walk around. The landscape architecture project has a surface of 7,434 sqm and occupies a plot of 69,791 sqm located in the Hortaleza district of Madrid. Belonging to the previous City of Justice project on the north side of the Institute of Legal Medicine, the plot, which is trapezoidal with a drop of 4,5 meters, is destined for a public hospital constructed in four months during the Covid-19 pandemic. Due to dry climatic conditions, adapted species with low water demand have been selected reducing the risk of allergies or respiratory problems. The topography has been modified to conserve rainwater and direct it to green areas that act as sponges that reduce runoff, store water, remove sediment and pollutants and release it into other ecosystems. There is no separation between soft scape (planting) and hard scape (soil) in the intervention, both are soft and porous and have macro and micro living beings. Following the references of these books, creativity and destruction as real phenomena both have attributes such as fitness and unfitness in the evolutionary way or health and disease. The vital system of living organisms (creativity) and viruses (destruction) has guided the design and distribution of these external areas that intend to prevent infections in the open air, as part of the mutation and adaptation process.

Junqueira-Kipnis, A. P., et al. (2020). "BCG revaccination of health workers in Brazil to improve innate immune responses against COVID-19: A structured summary of a study protocol for a randomised controlled trial." Trials **21**(1).

Objectives: The BCG vaccine, widely used in Brazil in new-borns, induces adjuvant protection for several diseases, including childhood virus infections. BCG activates monocytes and innate memory NK cells which are crucial for the antiviral immune response. Therefore, strategies to prevent COVID-19 in health workers (HW) should be carried out to prevent them becoming unwell so that they can continue to work during the pandemic. The hypothesis is that BCG will improve the innate immune response and prevent symptomatic infection or COVID-19 severity.The primary objective is to verify the effectiveness and safety of the BCG vaccine to prevent or reduce incidence of severe acute respiratory syndrome coronavirus-2 (SARS-CoV-2) infection in the city of Goiania (Brazil) among HW previously vaccinated with BCG and also its severity and mortality during the pandemic of the disease.Secondary objectives are to estimate the incidence of COVID-19 among these professionals and the innate immune response elicited to BCG. Trial design: This a phase II trial for repositioning BCG as a preventive strategy against COVID-19.The trial is an open-label, parallel-group randomised clinical trial, comparing HW vaccinated with BCG and HW not vaccinated. Participants: The trial will recruit 800 HW of Goiania - Goias, Brazil to reach a total of 400 HW included after comorbidities questioning and laboratorial evaluation. Eligibility criteria: Any HW presenting BCG vaccination scar with direct contact with suspected COVID-19 patients for at least 8 hours per week, whether in hospital beds, ICU, or in transportation or admission (nurses, doctors, physiotherapists, nutritionists, receptionists, etc.) who have negative IgM and IgG COVID-19 test.Participants with any of the following characteristics will be excluded:- - Have had in the last fifteen days any signs or symptoms of virus infection, including COVID-19; - Have had fever in the last fifteen days; - Have been vaccinated fifteen days before the inclusion; - Have a history or confirmation of any immunosuppressive disease such as HIV, presented solid tumour in the last two years or autoimmune diseases; - Are under preventive medication with antibiotics, steroid anti-inflammatories, or chemotherapy; - Have less than 500 neutrophils per mL of blood; - Have previously been diagnosed with tuberculosis; - Are breastfeeding or pregnant; - Are younger than 18 years old; - Are participating as an investigator in this clinical trial. Intervention and comparator: HW will be randomized into the BCG vaccinated group or the BCG unvaccinated control group. The BCG vaccinated group will receive in the right arm, intradermally, a one off dose of 0.1 mL corresponding to approximately 2 x10(5) to 8 x10(5) CFU of live, freeze-dried, attenuated BCG Moscow 361-I, Bacillus Calmette Guerin vaccine (Serum Institute of India PVT. LTD.). The unvaccinated control group will not be vaccinated. The HW allocated in both groups will be followed up at specific times points until 180 days post inclusion. The vaccinated and control groups will be compared according to COVID-19 related outcomes. Main outcomesThe primary outcomes are the incidence coefficient of infection by SARS-CoV-2 determined by RT-PCR of naso-oropharyngeal swab specimen or rapid lateral flow IgG and IgM test, and presence of general COVID-19 symptoms, disease severity and admission to hospital during the 180 days of follow up.The secondary outcome is the innate immune response elicited 15-20 days after vaccination. Randomisation: The vaccine vial contains approximately 10 doses. In order to optimize the vaccine use, the randomisation was performed in blocks of 20 participants using the platform randomization.com [http://www.jerrydallal.com/random/permute.htm].The randomization was prepared before any HW inclusion. The results were printed and inserted in sealed envelopes that were numbered with BCG-001 to BCG-400. The printed results as well the envelopes had the same numbers. At the time of the randomisation, each participant that meets the inclusion criteria will receive a consecutive participant number [BCG-001-BCG-400]. The sealed envelope with the assigned number, blinded to the researchers, will be opened in front of the participant and the arm allocation will be known. Blinding (masking): There is no masking for the participants or for the healthcare providers.The study will be blinded to the laboratory researchers and to those who will be evaluating the outcomes and performing the statistical analyses. In this case, only the participant identification number will be available. Numbers to be randomised (sample size): Four hundred heath workers will be randomised in two groups. Two hundred participants will be vaccinated, and 200 participants will not be vaccinated. Trial Status: The protocol approved by the Brazilian Ethical Committee is the seventh version, number CAAE: 31783720.0.0000.5078. The trial has been recruiting since September 20(th), 2020. The clinical trial protocol was registered on August 5(th), 2020. It is estimated that recruitment will finish by March 2021. Full protocol: The full protocol is attached as an additional file, accessible from the Trials website (Additional file 1). In the interest in expediting dissemination of this material, the familiar formatting has been eliminated; this Letter serves as a summary of the key elements of the full protocol.

Kaddu-Mulindwa, D., et al. (2021). "Protection strategy against outbreak of COVID-19 at a tertiary hematology-oncology: strengths and pitfalls." Infectious Agents and Cancer **16**(1).

Due to the worldwide COVID-19 outbreak it is mandatory for health care workers to develop containment strategies. Recently published data showed, that cancer patients might have a higher risk for severe course of the disease. We therefore developed a strategy of screening and containment for SARS-CoV-2 for hospitalized cancer patients. Our approach includes a temporary isolation in a so-called floating zone and testing strategy for screening of asymptomatic individuals by pooling of samples before RT-PCR amplification. Patients as far as health care professionals got tested twice a week. Nurses and physicians entered the floating zone with full body protection. Within 8 weeks we tested 418 individuals (professionals and patients) in total. Only 2 patients had COVID-19 without documented further transmission of SARS-CoV-2. We therefore think that our strategy might be a useful approach to protect inpatients with cancer at high risk for SARS-CoV-2 infection during this ongoing pandemic.

Kaushal, D., et al. "Endoscopy in Otorhinolaryngology During Corona Outbreak: A Proposal for Safe Practice." Indian Journal of Otolaryngology and Head & Neck Surgery.

Coronavirus disease 2019 (COVID-19) is a type of viral pneumonia that has paralysed the entire world both in terms of health and economy. It has been recently declared as a global pandemic. All the health care professionals must be aware of the disease entity and take precautionary measures to control its transmission from person to person, particularly in hospital settings. In this article, we propose essential steps that can be implemented at the departmental and institutional levels to do endoscopic diagnostic procedures effectively during COVID-19 outbreak and to break the transmission chain.

Kavanagh, K. T., et al. (2020). "COVID-19: through the eyes through the front line, an international perspective." Antimicrobial Resistance and Infection Control **9**(1).

COVID-19 is continuing to ravage the globe. In many Western Countries, the populous has not embraced public health advice which has resulted in a resurgence of the COVID-19 virus. In the United States, there is an absence of a coordinated Federal response. Instead, frontline workers and average citizens are having to cope with extensive mixed messaging regarding mask usage and social distancing from the highest levels of government. This has resulted in the United States not being able to achieve a low level of infection since the pandemic began. In addition, many citizens hold a profound belief that individual freedoms must be preserved, even at the expense of public health; and view the wearing of masks as renouncing this right. These engrained political beliefs can be traced back to the late 1800s. The response of the United States has also been hampered by a highly cost-efficient healthcare system, which does not provide universal care and has a just-in-time supply chain, with far too few supplies in reserve. This efficiency prevented a rapid scaling up of the healthcare response, which resulted in severe deficiencies in available personal protective equipment (PPE) and healthcare staff. To compound issues many healthcare staff are not provided an economic or healthcare safety net. Other frontline workers, such as those who work in transportation and food services, are working under even greater adversities. Many of these workers are from diverse backgrounds, who, along with their families, are at even greater risk for COVID-19. This vulnerable population of frontline workers are faced with a choice of going to work with inadequate PPE or placing food on their families' table. In the United States, official recommendations seem to be ever changing, based more upon supply and test availability, than on science. We must rely on science and learn from the lessons of past pandemics or we will relive, even to a greater degree, the deaths and devastations experienced by our ancestors over 100 years ago.

Kayipmaz, A. E. and A. Demircan (2021). "Pandemic hospitals and reorganizing emergency departments." Turkish Journal of Medical Sciences **51**: 3221-3228.

Emergency departments have always been the first point of contact for hospitals in many situations, including man-made and natural disasters. The first places where patients with symptoms of COVID-19 were met in health institutions were also emergency departments. Emergency departments play an important role in diagnosing the disease and isolating patients (by hospitalization if necessary). The process, which starts with the triage of outpatients admitted to the emergency department and brought by ambulance, continues as isolation of the patients in appropriate areas including physical evaluation, management of laboratory and scanning processes and, if necessary, providing cardiopulmonary resuscitation with airway support. Afterwards, patients can be treated as an outpatient, or hospitalized, or treated at the intensive care unit in line with their preliminary diagnosis and clinical conditions. Disruptions that may occur in one or more of these stages can lead to crowds and lengthy queues in the emergency department by prolonging the follow-up period of the patients. One of the strengths of Turkey at this point is that emergency departments are accustomed to the heavy patient load. The experiences gained from these conditions have facilitated the organization of pre-hospital emergency medical services, pandemic hospitals, and their emergency departments. In this organization, the main goal should be to provide uninterrupted and high-quality patient care through personnel training, personal protection measures, and the creation of physical conditions. Turkey's emergency departments are accustomed to managing the intensive patient flow, as they work at full capacity during normal times. Thanks to the experiences of emergency healthcare workers, health service was provided without any patient being turned away from the door of the emergency departments during the COVID-19 pandemic. In this review, we aimed to present the organization of pandemic hospitals and emergency departments during the COVID-19 pandemic. We made a schematic representation of the architectural areas through the emergency department of Ankara City Hospital, which has a bed capacity of 4200 with 256 beds in emergency department.

Kelly, A. M. (2020). "COVID-19 and medical litigation: More than just the obvious." Emergency Medicine Australasia **32**(4): 703-705.

COVID-19 has massively changed the health landscape around the world. Wide-ranging changes to healthcare delivery have occurred, especially in hospitals and EDs. Health services have made local decisions about care pathways, in some cases deviating from what would, until recently, have been considered widely accepted care. These changes bring with them new medicolegal risk for clinicians. In Australia, civil liability Acts provide protection for professionals when the criterion of having undertaken 'competent' practice that would be 'widely accepted' 'in the circumstances' is met. There is doubt how courts, and the medical experts who advise them, will evaluate clinical care provided during the pandemic when health services have developed local care pathways and there is no nationally accepted standard.

Kempfle, J. S., et al. (2021). "Effect of Powered Air-Purifying Respirators on Speech Recognition Among Health Care Workers." Otolaryngology-Head and Neck Surgery **164**(1): 87-90.

Powered air-purifying respirators (PAPRs) are used as personalized protective equipment for health care personnel. PAPRs offer health care workers added protection when dealing with patients who have high-risk infectious disease such as COVID-19. Unfortunately, PAPRs can produce notable levels of background noise. We hypothesize that PAPR use may be associated with increased hearing thresholds and impaired word discrimination and may ultimately have a negative impact on effective communication. Herein, we (1) determined sound levels generated by PAPRs and (2) measured hearing thresholds and word discrimination with and without operational PAPRs. All participants had normal hearing. When the PAPR was operational, mean +/- SD thresholds increased from 4.5 +/- 3.6 to 38.6 +/- 5.6 dB HL (P< .001). Word discrimination dropped from 100% in all participants in quiet to a mean 48% +/- 14% with operational PAPR (P< .001). Thus, we find that use of PAPR hoods results in hearing impairment comparable to moderate to severe hearing loss, and we suspect that users will experience communication difficulties as a result. Level of Evidence.Prospective study.

Khanji, M. Y., et al. (2021). "Early and Mid-Term Implications of the COVID-19 Pandemic on the Physical, Behavioral and Mental Health of Healthcare Professionals: The CoPE-HCP Study Protocol." Frontiers in Psychology **12**.

Introduction: The COVID-19 pandemic has led to unprecedented strain to healthcare systems worldwide and posed unique challenges to the healthcare professionals (HCPs) and the general public. Objectives: The aim of this study is to evaluate the impact of COVID-19 on the mental health, behavioral, and physical wellbeing of HCPs in the early and mid-term periods of the pandemic in comparison to non-HCPs. Thus, facilitating and guiding optimum planning and delivery of support to HCPs. Methods and Analysis: An observational cross-sectional survey and cohort study aiming to enroll over 1050 participants (minimum, 800 HCPs and 250 controls). Study questionnaires will be completed at baseline and after 6-weeks and 4-months. Recruitment initiated July 2020. The study was designed in London, United Kingdom, but open to participants worldwide. Baseline: Questionnaires comprising of validated self-administered screening tools for depression, anxiety, sleep-related issues, wellbeing, and burnout. The questionnaires also explore changes in behavior and physical wellbeing of the participants. In addition, associations of these mental health and behavioral factors with work-related factors and support will be explored. Six-weeks and 4-months follow-up: Follow-up questionnaires will assess change in symptoms of anxiety and depression, sleep disorders, use of alcohol and other substances, behavioral or interpersonal relationship changes. Physical wellbeing will be assessed through the presence of suspected or confirmed COVID-19 infection and absence from work. We will also evaluate the impact of variable provision of personal protection equipment (supply and training), extended working hours, and concern for the wellbeing of family members, anxiety levels, and evidence of burnout. Statistical Considerations: The study has 80% power to detect a 10% difference of combined depression and/or anxiety symptoms between the groups using two-sided type 1 error at 0.05 at baseline. Assuming that only 50% of these HCPs agree to be a part of a cohort survey, we will have 80% power to detect around 12% difference in the two groups in reported physical symptoms (20% vs. 32.3%), or prevalence of depression and/or anxiety at the end of the study. Ethics: The study was approved by the Cambridge East, Research Ethics Committee (20/EE/0166).

Khanna, R. C., et al. (2021). "Response of L V Prasad Eye Institute to COVID-19 outbreak in India: experience at its tertiary eye care centre and adoption to its Eye Health Pyramid." International Journal of Ophthalmology **14**(1): 1-9.

AIM: To summarize the experience of response to COVID-19 outbreak at a tertiary eye care institute and its network of health facilities in India. METHODS: Our responses are based on the principles of social distancing, hand hygiene, respiratory etiquettes, surface disinfection protocol, and rational use of appropriate personal protective equipment (PPE). We describe our response in terms of administrative controls, clinical protocols, staff protection, environmental controls, and social distancing measures. We also discuss our communication strategies and monitoring systems, to ensure compliance to protocols. RESULTS: Administrative control is mainly related to formation of task force and its functions. Clinical protocols are related to patient triaging methods and clinical examination guidelines in Outpatient, Inpatient and Operating Room. Staff protection is focused on training staff on the protocols to be followed in hospital as well as at home, and use of PPE. Environmental protocol is focused on cleaning and disinfectant methods to be used in the hospital. In addition, there are systems for communication as well as monitoring compliance to protocols. CONCLUSION: We hope that these protocols and our experience would help the ophthalmic community globally and serve as a guide to protect ophthalmologists and ophthalmic care personnel, and their patients across the world.

Kindgen-Milles, D., et al. (2021). "Prevalence of SARS-COV-2 positivity in 516 German intensive care and emergency physicians studied by seroprevalence of antibodies National Covid Survey Germany (NAT-COV-SURV)." PLoS One **16**(4).

Healthcare personnel are at risk to aquire the corona virus disease 2019 (COVID-19) caused by the severe acute respiratory syndrome coronavirus 2 (SARS-CoV-2). We evaluated the prevalence of SARS-CoV-2 antibodies and positive nasopharyngeal reverse transcriptase polymerase-chain reaction (RT-PCR) tests in German intensive care and emergency physicians. Physicians attending intensive care and emergency medicine training courses between June 16(th) and July 2(nd) 2020 answered a questionnaire and were screened for SARS-CoV-2 antibodies via automated electrochemiluminiscence immunoassay. We recruited 516 physicans from all parts of Germany, 445/516 (86%) worked in high risk areas, and 379/516 (73%) had treated patients with COVID-19. The overall positive rate was 18/516 (3.5%), 16/18 (89%) had antibodies against SARS-COV-2, another 2 reported previous positive RT-PCR results although antibody testing was negative. Of those positive, 7/18 (39%) were unaware of their infection. A stay abroad was stated by 173/498 (35%), mostly in Europe. 87/516 (17%) reported a febrile respiratory infection after January 1(st) 2020 which was related to SARS-CoV-2 in 4/87 (4.6%). Contact to COVID-19 positive relatives at home was stated by 22/502 (4.4%). This was the only significant risk factor for Covid-19 infection (Fisher ' s exact test, p = 0.0005). N95 masks and eye protection devices were available for 87% and 73%, respectively. A total of 254/502 (51%) had been vaccinated against seasonal influenza. The overall SARS-CoV-2 infection rate of german physicians from intensive care and emergency medicine was low compared to reports from other countries and settings. This finding may be explained by the fact that the German health care system was not overwhelmed by the first wave of the SARS-CoV-2 pandemic.

Kipp, C. and K. Kretz (2021). "Masks, Art, Protection, and Politics. July 15, 2020." Journal of Modern Craft **14**(1): 19-24.

Since April 2020, the U.S. Center for Disease Control has asked for people to wear face masks in public to curb the spread of COVID-19. This shift has given rise to a mass movement of cloth mask making. Artists have leapt into action to produce masks for healthcare and frontline workers or as an alternative economy for lost income, while others customize masks to communicate identity, beliefs, or concerns. Mask making has also raised questions about the future of fashion with regards to health and sustainability. Through a series of conversations, experts from the arts, fashion, sustainability, and medical fields were invited to talk about the variety of issues, and questions this moment of mask making and wearing have produced. In May 2019, Kate Kretz skyrocketed into the international media when she was banned from Facebook for her now infamous MAGA Hat series.(1) (Figures 1 and 2) Kretz's artwork draws direct parallels between known symbols of fascism, oppression, and racism, and former President Trump's branding, specifically his campaign slogan "Make America Great Again." Using counterfeit MAGA hats, Kretz deconstructs and reconfigures this widely popularized consumer merchandise to "function as corrective physical manifestations of the truth."(2) In her most recent addition to the series, Kretz has created Social Murder, a critique of the Trump administrations management of the COVID-19 pandemic.

Klimek, L., et al. (2020). "A new form of irritant rhinitis to filtering facepiece particle (FFP) masks (FFP2/N95/KN95 respirators) during COVID-19 pandemic." World Allergy Organization Journal **13**(10).

Filtering facepiece particle (FFP) masks are important items of personal protective equipment in fighting COVID-19 pandemic. They shall protect the wearer of the mask from particles, droplets, and aerosols, but they also can prevent the spread of aerosol-transmitted viruses if the wearer becomes infected. Most often, FFP respirators consist of multiple layers of non-woven fabric made from polypropylene. Worldwide, FFP respirators are subject to various regulatory standards that specify physical properties and performance characteristics. During the SARS-CoV-2 pandemic, health authorities have temporarily repealed standards for respirators. We report on 46 patients that presented with rhinitis-like symptoms strongly associated to the use of FFP masks. Some of them were obliged to use FFP masks in their work environment. Nasal endoscopy showed edemata of the nasal mucosa that significantly decreased after a period of non-use of FFP masks. Subjectively reported symptom levels decreased after cessation of FFP use for 3 or more days. The presence of polypropylene fibres isolated from nasal rinsing solution was significantly associated with the use of FFP masks in our patients. Material safety and performance deregulation of FFP masks can pose a health risk. Thus, especially health care professionals and other individuals with occupational need for FFP masks should be aware of possible hazards that come with COVID-19 pandemic protection measures.

Kotfis, K., et al. (2020). "COVID-19: ICU delirium management during SARS-CoV-2 pandemic." Critical Care **24**(1).

The novel coronavirus, SARS-CoV-2-causing Coronavirus Disease 19 (COVID-19), emerged as a public health threat in December 2019 and was declared a pandemic by the World Health Organization in March 2020. Delirium, a dangerous untoward prognostic development, serves as a barometer of systemic injury in critical illness. The early reports of 25% encephalopathy from China are likely a gross underestimation, which we know occurs whenever delirium is not monitored with a valid tool. Indeed, patients with COVID-19 are at accelerated risk for delirium due to at least seven factors including (1) direct central nervous system (CNS) invasion, (2) induction of CNS inflammatory mediators, (3) secondary effect of other organ system failure, (4) effect of sedative strategies, (5) prolonged mechanical ventilation time, (6) immobilization, and (7) other needed but unfortunate environmental factors including social isolation and quarantine without family. Given early insights into the pathobiology of the virus, as well as the emerging interventions utilized to treat the critically ill patients, delirium prevention and management will prove exceedingly challenging, especially in the intensive care unit (ICU). The main focus during the COVID-19 pandemic lies within organizational issues, i.e., lack of ventilators, shortage of personal protection equipment, resource allocation, prioritization of limited mechanical ventilation options, and end-of-life care. However, the standard of care for ICU patients, including delirium management, must remain the highest quality possible with an eye towards long-term survival and minimization of issues related to post-intensive care syndrome (PICS). This article discusses how ICU professionals (e.g., physicians, nurses, physiotherapists, pharmacologists) can use our knowledge and resources to limit the burden of delirium on patients by reducing modifiable risk factors despite the imposed heavy workload and difficult clinical challenges posed by the pandemic.

Kowalski, L. P., et al. (2020). "COVID-19 pandemic: Effects and evidence-based recommendations for otolaryngology and head and neck surgery practice." Head and Neck-Journal for the Sciences and Specialties of the Head and Neck **42**(6): 1259-1267.

The 2019 novel coronavirus disease (COVID-19) is a highly contagious zoonosis produced by SARS-CoV-2 that is spread human-to-human by respiratory secretions. It was declared by the WHO as a public health emergency. The most susceptible populations, needing mechanical ventilation, are the elderly and people with associated comorbidities. There is an important risk of contagion for anesthetists, dentists, head and neck surgeons, maxillofacial surgeons, ophthalmologists, and otolaryngologists. Health workers represent between 3.8% and 20% of the infected population; some 15% will develop severe complaints and among them, many will lose their lives. A large number of patients do not have overt signs and symptoms (fever/respiratory), yet pose a real risk to surgeons (who should know this fact and must therefore apply respiratory protective strategies for all patients they encounter). All interventions that have the potential to aerosolize aerodigestive secretions should be avoided or used only when mandatory. Health workers who are: pregnant, over 55 to 65 years of age, with a history of chronic diseases (uncontrolled hypertension, diabetes mellitus, chronic obstructive pulmonary diseases, and all clinical scenarios where immunosuppression is feasible, including that induced to treat chronic inflammatory conditions and organ transplants) should avoid the clinical attention of a potentially infected patient. Health care facilities should prioritize urgent and emergency visits and procedures until the present condition stabilizes; truly elective care should cease and discussed on a case-by-case basis for patients with cancer. For those who are working with COVID-19 infected patients' isolation is compulsory in the following settings: (a) unprotected close contact with COVID-19 pneumonia patients; (b) onset of fever, cough, shortness of breath, and other symptoms (gastrointestinal complaints, anosmia, and dysgeusia have been reported in a minority of cases). For any care or intervention in the upper aerodigestive tract region, irrespective of the setting and a confirmed diagnosis (eg, rhinoscopy or flexible laryngoscopy in the outpatient setting and tracheostomy or rigid endoscopy under anesthesia), it is strongly recommended that all health care personnel wear personal protective equipment such as N95, gown, cap, eye protection, and gloves. The procedures described are essential in trying to maintain safety of health care workers during COVID-19 pandemic. In particular, otolaryngologists, head and neck, and maxillofacial surgeons are per se exposed to the greatest risk of infection while caring for COVID-19 positive subjects, and their protection should be considered a priority in the present circumstances.

Kristoffersen, E. S., et al. (2021). "Experiences, distress and burden among neurologists in Norway during the COVID-19 pandemic." PLoS One **16**(2).

Background The ongoing COVID-19 pandemic has caused rapid changes in the healthcare system. Workforce reorganization, reduced standard of care and a lack of personal protection equipment (PPE) for health care workers were among the concerns raised in the first wave of the pandemic. Our aim was to explore the experiences, distress and burden among Norwegian neurologists during the first weeks of the pandemic. Methods Hospital-based neurologists in Norway (n = 400) were invited to a web-based survey in April 2020. The study focused on patient management, organizational changes and personal stress during the first weeks of the pandemic lockdown. Work-home interface stress was assessed by the Cooper Job Stress Questionnaire. Results In total, 135 neurologists participated. Seventy-three% experienced a change in their personal work situation, and 67% examined patients with suspected COVID-19 infection and neurological disease. Changed access to resources, and the perception that medical follow-up was unsatisfactory, were associated with a high degree of burden and stress. Neurologists were also worried about the potential lack of PPE and the fear of spreading SARS CoV-2 to close family members. The mean score of work-home interface stress was 2.8 with no significant differences between gender or specialist status. Reduced standard of care was reported for all neurological conditions, and in particular for non-emergency treatments. Conclusion The vast majority of neurologists in Norway experienced a change in their personal work situation during the first phase of the pandemic. The fear of becoming infected and ill was not a major contributor to burden and stress.

Krusche, M., et al. (2020). "Telemedicine in rheumatology." Zeitschrift Fur Rheumatologie **79**(9): 883-892.

Due to the outbreak of the COVID-19 pandemic, in recent months we have experienced deep changes in our daily and professional lives. In the context of pandemic containment, routine rheumatological procedures have changed in many areas. To guarantee sufficient protection against the infection for patients and staff, telemedicine (especially telephone and video consultation) has increasingly been used. Due to the Digital Healthcare Act (DiGAV), whereby patients will have a legal claim to specific digital health applications in Germany, medical apps and wearables will offer new opportunities for telemedical monitoring. This article provides an overview of telemedical care options in the field of rheumatology. Furthermore, opportunities and limitations of telemedicine in rheumatology are reviewed.

Kumur, H. and J. S. Malik (2021). "Impact of Safety Measures in Endoscopic Procedures in Health Care Professionals during Corona Virus Pandemic." Journal of Pharmaceutical Research International **33**(47A): 786-793.

Background and Aims: During the Corona Virus Disease-19 outbreak, it is believed that ten percent of all health care professionals (HCPs) were affected. HCPs' safety measures have changed because of aerosol-generating techniques. As a result, we looked at the effectiveness of endoscopic safety measures and prevalence among HCPs who operate in an endoscopy unit. Methods: Techniques such as polymerase chain reaction (PCR) and immunoglobulin G (IgG) serum-assay were used to assess the COVID-19 status of 117 healthcare workers (HCWs). It has been shown that the existence of the COVID-19 is related to demographic variables, work profile, location of employment, & medical history in research. Results: 38 HCWs tested positive for COVID-19 using either a PCR assay (23.93%) or simply an IgG testing (32.48 percent) (8.55%). COVID-19 incidence was significantly greater (P = 0.003) among endoscopy technicians when compared to doctors (68.75% (20.69%). Those who worked in critical care units were shown to be more susceptible to COVID-19 (42.86%). One hundred sixty-six healthcare workers who received enough hydroxychloroquine prophylaxis were tested for COVID-19, and none were positive. Everyone who had been exposed to COVID-19 recovered. There was also a substantial decrease in the number of "man-days" worked. Conclusion: We discovered a statistically significant COVID-19 risk among healthcare workers in the Gastroenterology department, with endoscopy technologists having the highest risk. More stringent triaging & pre-testing of patients and healthcare workers may help to decrease the risk of COVID-19 infection. Additional multicenter studies are needed to further understand the risk and its related variables.

Lakshmi, K., et al. (2021). "Knowledge, awareness and outlook on the COVID-19 disease among the dental professionals in Tamil Nadu." Journal of Indian Academy of Oral Medicine and Radiology **33**(1): 40-46.

Aim: The aim of this study was to analyze the knowledge, level of awareness, and the attitude of the dental professionals towards the pandemic disease (COVID-19). Materials and Methods: This questionnaire-based survey was conducted among 295 dental professionals residing in Tamil Nadu through an online portal. The questionnaire consisted of four sections that addressed the demographic data, knowledge, awareness, and outlook of the COVID-19 disease by dental professionals. All the received responses were tabulated and the results were represented graphically. Result: The result of the study showed significant awareness among dental professionals towards COVID-19. The dental professionals needed more attention towards the precautions to be followed during this pandemic as personal protection and a safe environment are essential for a secure practice. Conclusion: This study emphasizes the role of oral health professionals in the prevention of the transmission of coronavirus among the public along with the management of dental emergencies with appropriate personal protective measures. This study also enforced the need to enrich the knowledge about infection, transmission, prevention, and control towards COVID-19. This may act as a source of information for the future pandemic crisis.

Lammers, M. J. W., et al. (2020). "Guidance for otolaryngology health care workers performing aerosol generating medical procedures during the COVID-19 pandemic." Journal of Otolaryngology-Head & Neck Surgery **49**(1).

Background Severe acute respiratory syndrome coronavirus 2 (SARS-CoV-2), the virus responsible for Coronavirus disease 2019 (COVID-19) has a predilection for infecting the mucosa of the upper and lower airways. Otolaryngologists and supporting health care workers (HCWs) are particularly at high risk of becoming infected while treating patients as many in-office procedures and surgeries are Aerosol Generating Medical Procedures (AGMP). Based on a review of the literature and various guidelines, recommendations are made to mitigate the risk to health care workers of becoming infected with SARS-CoV-2 while providing clinical care. Recommendations During the COVID-19 pandemic all elective and non-time sensitive Otolaryngology procedures should be deferred to mitigate the risk of transmission of infection to HCWs. For non-AGMPs in all patients, even COVID-19 positive patients Level 1 PPE (surgical mask, gown, gloves and face shield or goggles) is sufficient. If local prevalence is favourable and patients are asymptomatic and test negative for SARS-CoV-2, Level 1 PPE can be used during short duration AGMPs, with limited risk of infected aerosol spread. For AGMPs in patients who test positive for SARS-CoV-2 a minimum of Level 2 PPE, with adequate protection of mucosal surfaces, is recommended (N95/FFP2 respirator, gown, double gloves, goggles or face shield and head cover). For long duration AGMPs that are deemed high-risk in COVID-19 positive patients, Level 3 PPE can provide a higher level of protection and be more comfortable during long duration surgeries if surgical hoods or PAPRs are used. It is recommended that these procedures are performed in negative pressure rooms, if available. It is essential to follow strict donning and doffing protocols to minimize the risk of contamination. Conclusions By following strict infection prevention recommendations, the risk of HCWs becoming infected with SARS-CoV-2 while treating patients can be minimized. As the COVID-19 pandemic evolves rapidly, these recommendations should serve as guidance and need to be interpreted based on local factors and availability of healthcare resources.

Lan, X. L., et al. (2021). "Expert consensus on the safety prevention and control of nuclear medicine diagnosis and treatment during the outbreak of COVID-19 (1st edition): (translated from Chinese version)." European Journal of Nuclear Medicine and Molecular Imaging **48**(4): 1134-1143.

In December 2019, an infectious disease caused by a new type of coronavirus infection was prevalent in Wuhan and across the country. On January 20, 2020, the National Health Commission of the People's Republic of China issued No.1 Announcement, which incorporated the novel coronavirus pneumonia into the Class B infectious disease according to the Law on Prevention and Control of Infectious Disease, but the disease should be adopted in the management of Class A infectious disease. In order to effectively control the source of infection, cut off the transmission route, protect the susceptible population, ensure the medical quality and medical safety, perform epidemic prevention and control, and comprehensively guarantee the life safety and physical health of medical staff, patients, and family members, it is very important to organize and carry out nuclear medicine diagnosis and treatment scientifically and safely. According to the national prevention and control policy, Chinese Society of Nuclear Medicine and editorial board of the Chinese Journal of Nuclear Medicine and Molecular Imaging organized professionals to formulate the expert consensus on the safety prevention process of nuclear medicine imaging and nuclide therapy during the period of new coronavirus infection.

Lauritano, D., et al. (2020). "Environmental Disinfection Strategies to Prevent Indirect Transmission of SARS-CoV2 in Healthcare Settings." Applied Sciences-Basel **10**(18).

(1) Introduction: The novel respiratory syndrome coronavirus 2 (SARS-CoV-2), also called coronavirus disease 2019 (COVID-19), is rapidly spreading in many countries and represents a public health emergency of international concern. The SARS-CoV-2 transmission mainly occurs from person-to-person via respiratory droplets (direct transmission route), leading to the onset of mild or severe symptoms or even causing death. Since COVID-19 is able to survive also on inanimate surfaces for extended periods, constituting an indirect transmission route, healthcare settings contaminated surfaces should be submitted to specific disinfection protocols. Our review aimed to investigate the existing disinfection measures of healthcare settings surfaces, preventing the nosocomial transmission of SARS-CoV-2. (2) Materials and Methods: We conducted electronic research on PubMed, Scopus, Science Direct, and Cochrane Library, and 120 items were screened for eligibility. Only 11 articles were included in the review and selected for data extraction. (3) Results: All the included studies proposed the use of ethanol at different concentrations (70% or 75%) as a biocidal agent against SARS-CoV-2, which has the capacity to reduce the viral activity by 3 log(10) or more after 1 min of exposure. Other disinfection protocols involved the use of chlorine-containing disinfectant, 0.1% and 0.5% sodium hypochlorite, quaternary ammonium in combination with 75% ethanol, isopropyl alcohol 70%, glutardialdehyde 2%, ultraviolet light (UV-C) technology, and many others. Two studies suggested to use the Environmental Protection Agency (EPA)-registered disinfectants, while one article chooses to follow the WST-512-2016 Guidance of Environmental and Surfaces Cleaning, Disinfection and Infection Control in Hospitals. (4) Conclusion: Different surface disinfection methods proved to reduce the viral activity of SARS-CoV-2, preventing its indirect nosocomial transmission. However, more specific cleaning measures, ad hoc for the different settings of the healthcare sector, need to be formulated.

Lazaro-Perez, C., et al. (2021). "Communication and Cultural Approach to the Situation of Health Professionals in the Face of the COVID-19 Pandemic." Revista Latina De Comunicacion Social **79**: 357-380.

Introduction. The beginning of the third decade of the 21st century will be remembered for the COVID-19 pandemic and the health crisis it has produced, altering different systems such as the cultural, political, economic, media and communication systems. From the anthropology of health, the interconnections between the health system, communication, and culture can be observed, visible and indivisible by the many edges that coexist and conform to a field of own analysis. Methodology: This empirical research has been developed from a qualitative perspective through 40 anonymous semi-structured interviews in Spain during the height of the health crisis - in the state of alarm - and investigates how this pandemic affected health professionals during the first wave of the pandemic. They were the fundamental barrier to deal with the SARS-CoV-2 coronavirus, and they carried out their work in highly precarious conditions, with hardly any personal protection equipment, sufficient human resources, and the essential infrastructures to care for patients. Results/Discussion: The study focuses on three dimensions: (a) the cultural aspects that impregnate the professional activity of the health workers, (b) the emotional aspects in the development of the same about the processes of mourning and death from its symbolic components, and (c) the perceptions it has of the health management carried out by the public response. Conclusions: The results describe the extreme situation these professionals in a health crisis without precedent in decades, reflecting a new anthropological and sociological scenario.

Lazaro-Perez, C., et al. (2020). "COVID-19 Pandemic and Death Anxiety in Security Forces in Spain." International Journal of Environmental Research and Public Health **17**(21).

The pandemic caused by the SARS-CoV-2 coronavirus, which produces COVID-19 disease, has revealed to political and social circles a series of needs that have not yet been met. The workers of the State Security Forces and the Armed Forces have done an extraordinary job to try to alleviate the effects that the pandemic has had on the population and to return stability to the citizenry as much as possible. In this context, the following investigation is developed based on two objectives: (PO1) to know the level of anxiety in the face of death in these professionals; (PO2) to determine the predictive variables in the above-mentioned phenomenon. Professionals from all over Spain have participated in the study (n = 2079). From a quantitative perspective, a questionnaire was developed from the Collet-Lester death anxiety scale. The results show a total level of 69.2% in the scale, as well as some higher levels about the fear of death of others (82.1%) and the fear of the process of dying of others (78.2%). On the other hand, from the binary logistic regressions, four variables are evidenced that condition the risk of suffering death anxiety: (a) certainty of needing psychological treatment in the future; (b) absence of Individual Protection Equipment (PPE); (c) high levels of Emotional Exhaustion; (d) high levels of depersonalization-these last two come from the Maslach and Jackson Burnout scale. These data show a need for training and intervention in the emotional and psychological demands of the professionals of the Armed Forces and State Security Forces, as well as the obligation to develop a continuous dialogue with the institutions they represent to foster the feeling of belonging to them. It is essential, regardless of the serious consequences that the virus has caused, to understand the psychosocial and emotional demands of enforcement agents and to improve their occupational health.

Levine, D. T., et al. (2020). "Child safety, protection, and safeguarding in the time of COVID-19 in Great Britain: Proposing a conceptual framework." Child Abuse & Neglect **110**.

Background: Great Britain has the highest coronavirus death rate in Europe. While the pandemic clearly poses a risk to the lives and wellbeing of vulnerable groups, necessary public health measures taken to delay or limit the spread of the virus have led to distinctive challenges for prevention, family support, court processes, placement and alternative care. The pandemic has also come about at a time when statutory changes to partnerships have led to a reduction in the importance of educational professional representation in the new formulation in England and Wales. Objectives: In this discussion paper, we propose a novel and pragmatic conceptual framework during this challenging time. Participants: We consulted with 8 education professionals and 4 field-based student social workers. Setting: Bodies responsible for safeguarding have been working quickly to develop new approaches to fulfilling their responsibilities, for example through online home visits and case conferences. However, some communities have been highlighted as experiencing particular challenges because of the pandemic and its impacts. Protection of vulnerable children is increasingly dependent on individualised - and often pathologising - practice with a lack of emphasis on the importance of the social. Holistic consideration of the child is side-lined. Results: Our framework comprises two phases: pandemic and aspirational. Conclusion: The framework illuminates the importance of interconnected sectors and multiagency working, the need for resilient and adaptable support systems, and the need to promote the importance of children's rights and voices to be heard above the noise of the pandemic.

Levy, T., et al. (2021). "Management of Cancer Patients by ARS Brittany during the COVID-19 Pandemic." Oncologie **23**(1): 3-38.

Introduction: For several centuries, scientists such as Avicenna have been trying to understand the processes involved in the spread of epidemics. Isolation instructions appeared during the black plague. Magistrates and crisis managers manage the protection of all populations. Nowadays, regional health agencies regulate the provision of health care even in times of health crisis. Methods: The creation of the COVID and Cancer regional committee organized and run by the ARS and the Oncobretagne regional cancer network enables exchanges between oncology health professionals. This committee relays information to national representatives: Ministry, INCa (National Cancer Institute) who adjust their recommendations to cancer patients. In addition, the players are alerted to the dysfunctions caused by the COVID pandemic and can improve their actions. Moreover, the action of the Brittany regional health agency immediately follows the instructions of the ministry and adapts them to the regional context. Results. The impact of the COVID pandemic in Brittany had little effect on chemotherapy, radiotherapy and oncological surgery activities in 2020 compared to 2019. Only oncological digestive surgery on all these organs shows a clear decrease in activity, it will require follow-up in the future. Surgical activity in otorhinolaryngology is also experiencing a decline to monitor. Maintaining the organization of RCPs (Multidisciplinary consultation meetings), regional RCPs and RCPs of recourse managed by the regional oncology pole does not present any major difficulty apart from logistical adjustments. The inclusions in therapeutic trials have decreased due to the promoters' decisions but with no clear interruption. The decisions collaboratively taken by ARS Brittany and the regional cancer center allow the maintenance of therapeutic trials in Brittany and in the inter-region. The feedback from the experience carried out in Brittany continues to support the decisions taken by ARS (Regional Health Agency) Brittany to facilitate the care of cancer patients during the COVID pandemic period. Conclusion: The Brittany Regional Health Agency in collaboration with the Oncobretagne regional cancer network and the regional cancer center are succeeding in limiting the impact of the COVID pandemic for cancer patients. The creation of the COVID and Cancer committee is one of the organizations initiated by the Brittany Regional Health Agency and oncobretagne, which enables the day-to-day management of regional actions on the cancer theme. Few references exist to allow us a precise comparison with other international healthcare systems in the context of the COVID and Cancer action.

Li, G. W., et al. (2020). "Precautions in dentistry against the outbreak of corona virus disease 2019." Journal of Infection and Public Health **13**(12): 1805-1810.

The outbreak of Coronavirus Disease 2019 (COVID-19) has become a severe global acute respiratory pandemic around the world in just a few months with an increasing number of infections and deaths. COVID-19 is a highly contagious and fatal disease. Almost everyone in the population is susceptible, and the incubation period is 1-14 days, mostly 3-7 days. The clinical symptoms of the COVID-19 are fever, dry cough and fatigue. Some patients are accompanied by symptoms such as nasal congestion, runny nose, sore throat, myalgia and diarrhea. Severe patients could even develop acute respiratory distress syndrome, septic shocks, metabolic acidosis and multifunctional organ failure, etc. Due to the relatively closed environment of dental clinics and the unique nature of dental procedures, both dental personnel and patients are easy to get infection through currently known respiratory droplet transmission, aerosol transmission, close contact transmission and other ways, inducing mutual cross-infection. Dental practitioners are facing unprecedented challenges due to the high risk of exposure to droplets and aerosols from saliva and other body fluids during dental procedures. Based on our experience and relevant research, this article introduces the basic knowledge about COVID-19 and the corresponding protective measures for dental practitioners, includes the risk of infection during dental procedures, the precautions related to the patients, infection control measures during dental treatment in clinics, protection measures at different levels for dental practitioners, and emergency dental treatment for confirmed COVID-19. It is the responsibility of every dental practitioner to fully understand the characteristics of the new coronavirus and strictly implement the most appropriate protective measures to reduce and control the risk of cross infection in dental procedures. (C) 2020 Published by Elsevier Ltd on behalf of King Saud Bin Abdulaziz University for Health Sciences.

Li, Y. T. and J. Ng "Moral Dilemma of Striking: A Medical Worker's Response to Job Duty, Public Health Protection and the Politicization of Strikes." Work Employment and Society.

The emergence of COVID-19 has led many countries to take strong border control measures. In Hong Kong, in reaction to government reluctance to close the border, more than 9000 medical workers went on strike. The strike lasted for five days only, yet it provoked a moral dilemma for healthcare occupations - when workers strike, citizens' medical needs may be sacrificed. This article presents Jenna, a medical worker who went on strike, and her evaluation of the moral dilemma. Her account shows the ways in which different narratives shape power and politics and lend legitimacy to striking. Her example reveals the contested framing of professionalism - the struggle between job duties, workplace safety and a commitment to the public interest (public health). This contribution highlights how the moral dilemma of medical strikes can be resolved, and how the politicization of strikes can be legitimized by medical workers.

Lin, P. T., et al. (2020). "Reducing the consumption of personal protective equipment by setting up a multifunctional sampling station in the emergency department to screen for COVID-19 infection in Taiwan." Environmental Health and Preventive Medicine **25**(1).

In Taiwan, high-risk patients have been identified and tested for preventing community spread of COVID-19. Most sample collection was performed in emergency departments (EDs). Traditional sample collection requires substantial personal protective equipment (PPE), healthcare professionals, sanitation workers, and isolation space. To solve this problem, we established a multifunctional sample collection station (MSCS) for COVID-19 testing in front of our ED. The station is composed of a thick and clear acrylic board (2 cm), which completely separates the patient and medical personnel. Three pairs of gloves (length, 45 cm) are attached and fixed on the outside wall of the MSCS. The gloves are used to conduct sampling of throat/nasal swabs, sputum, and blood from patients. The gap between the board and the building is only 0.2 cm (sealed with silicone sealant). ED personnel communicate with patients using a small two-way broadcast system. Medical waste is put in specific trashcans installed in the table outside the MSCS. With full physical protection, the personnel conducting the sampling procedure need to wear only their N95 mask and gloves. After we activated the station, our PPE, sampling time, and sanitization resources were considerably conserved during the 4-week observation period. The MSCS obviously saved time and PPE. It elevated the efficiency and capacity of the ED for handling potential community infections of COVID-19.

Lin, Y. Y., et al. (2021). "COVID-19 Pandemic Is Associated with an Adverse Impact on Burnout and Mood Disorder in Healthcare Professionals." International Journal of Environmental Research and Public Health **18**(7).

The coronavirus disease 2019 (COVID-19) pandemic results in a profound physical and mental burden on healthcare professionals. This study aims to evaluate burnout status and mood disorder of healthcare workers during this period. An online questionnaire was voluntarily answered by eligible adult employees in a COVID-19 specialized medical center. The major analysis included the burnout status and mood disorder. Factors related to more severe mood disorder were also identified. A total of 2029 participants completed the questionnaire. There were 901 (44.4%) and 923 (45.5%) participants with moderate to severe personal and work-related burnout status, respectively. Nurses working in the emergency room (ER), intensive care unit (ICU)/isolation wards, and general wards, as well as those with patient contact, had significantly higher scores for personal burnout, work-related burnout, and mood disorder. This investigation identified 271 participants (13.35%) with moderate to severe mood disorder linked to higher personal/work-related burnout scores and a more advanced burnout status. Univariate analysis revealed that nurses working in the ER and ICU/isolation wards were associated with moderate to severe mood disorder risk factors. Multivariate analysis demonstrated that working in the ER (OR, 2.81; 95% CI, 1.14-6.90) was the only independent risk factor. More rest, perquisites, and an adequate supply of personal protection equipment were the most desired assistance from the hospital. Compared with the non-pandemic period (2019), employees working during the COVID-19 pandemic (2020) have higher burnout scores and percentages of severe burnout. In conclusion, this study suggests that the COVID-19 pandemic has had an adverse impact on healthcare professionals. Adequate measures should be adopted as early as possible to support the healthcare system.

Liu, H., et al. (2021). "Experience of comprehensive interventions in reducing occupational exposure to COVID-19." Journal of Infection and Public Health **14**(2): 201-205.

Importance: The infection of medical personnel with COVID-19 was a disaster for both patients and doctors. However, some effective measures can prevent medical staff from becoming infected. This article introduces those measures and thus provides a reference for other hospitals. Objective: In order to reduce the risk of occupational exposure and of the infection of medical staff, this article analyzed the factors, causes and experience of medical personnel on their occupational exposure to COVID-19. Some effective and targeted intervention measures can be implemented in order to avoid the occupational exposure of medical staff to COVID-19. Evidence review: In this single-center case series involving 196 medical personnel, occupational exposure to COVID-19 was present. Nursing staff accounted for 67.35% of those cases. The relationships with an exposure source were found to be as follows: doctors and patients (87.24%), colleagues (10.20%), and roommates (2.55%). Occupational exposure was found to be present in the clinical department, radiology department, central sterile supply department, as well as in the outpatient clinics and operating rooms. The non-surgical departments accounted for 72.96% and direct contact accounted for 84.69% while failure to wear surgical masks (84.18%) and operating on the patient without wearing goggles/face shield (8.16%) were the main causes of occupational exposure. The occurrence of occupational exposure to COVID-19 declined to 0.19% after an extensive and comprehensive intervention program. Conclusions and relevance: Some effective measures such as hand hygiene, wearing surgical masks in and around the hospital, reasonable use of goggles/face screens, raising awareness of protective measures, minimizing the number of elective operations, strengthening training as well as many other control measures were instrumental in reducing occupational exposure. For any medical institution there is room for improvement in terms of personal protection to reduce occupational exposure. (C) 2020 The Author(s). Published by Elsevier Ltd on behalf of King Saud Bin Abdulaziz University for Health Sciences.

Liu, L., et al. (2021). "Real-time monitoring and controlling strategies of indoor environment in the frontline hospitals during COVID-19 pandemic." Chinese Science Bulletin-Chinese **66**(4-5): 424-432.

Many emergent measures on mitigating and controlling the COVID-19 pandemic have been unprecedented in China, one of which is the urgent construction of isolation wards in Huoshenshan Hospital and Leishenshan Hospital, as well as in sixteen Fangcang shelter hospitals. These hospitals, built or reconstructed within a remarkably short period, have treated a large number of COVID-19 patients in time and successfully mitigated disease spread in population. One of the many challenges during the hospitals' functioning period is the precaution of potential airborne transmission. Airborne transmission could occur during medical procedures including tracheotomy, intubation, extubation, etc., and several surveillance studies have detected SARS-CoV-2 virus in air by polymerase chain reaction (PCR), but direct methods on monitoring airborne SARS-CoV-2 in real-time have not been developed so far. Though it is yet to know whether these airborne viruses are viable and able to cause person-to-person infection, airborne precautions such as negative-pressure wards and N95 respirators have already been recommended and implemented in China. In order to protect the healthcare workers (HCWs) and reduce the demand of high-level protection gear, this study proposed an indirect method to identify and alert the airborne infection risk. We deployed numerous sensors to monitor CO2 concentration, PM2.5 mass concentration, air temperature and relative humidity, and illumination at ICUs (intensive care units), general wards, nurse stations, buffer rooms, etc. in real-time. Online CO2 concentration reflects the dilution of occupants' exhalation substances by the ventilation and air distribution system. High CO2 concentrations indicate either the fresh air supply fails or the room is overcrowded at the moment. The readings of CO2 concentration across adjacent zones with known functions can also help identify whether the airflow direction is consistent with the designed requirement. PM2.5 mass concentration indicates the air cleanness of the measured zones, which could be crucial for patient recovery and reduction of symptom complication. Air temperature and relative humidity are key parameters that influence the evaporation of expiratory droplets and the thermal comfort of patients, while illumination indicates the intake of ultraviolet from outdoor. The sensors could keep quiet during the sampling process, so they would not disturb the HCWs and patients. Moreover, they are integrated with 3G SIM cards and powered just by a DC 5V/3A power adapter with a USB Type-C line, so they could be easily installed at target locations, and adapt for multi-scene, multi-demand, and long-term online and offline monitoring. When integrated with online meta-analysis and computer fluid dynamics (CFD) simulation, this method can send alarms to the HCWs about the locations where the ventilation against potential pathogen-laden aerosols fails. This study introduces the principle of the method in detail as well as its application at Huoshenshan Hospital, Leishenshan Hospital and Jiangxia Dahuashan Fangcang shelter hospital, etc., in Wuhan, China, during February and March of 2020. As the society gradually steps into post-pandemic phase, the proposed method could help optimize the ventilation and filtration strategies in public buildings and transportations.

Liu, X. H., et al. (2020). "COVID-19 Outbreak Can Change the Job Burnout in Health Care Professionals." Frontiers in Psychiatry **11**.

Background: The outbreak of COVID-19 in China was a sudden bio-disaster, which may bring a negative impact on the job burnout of health care professionals (HCPs). Objective: We aim to find out the association factors, especially those closely related to this outbreak, of job burnout in Chinese HCPs. Method: The cross-sectional survey about HCPs' job burnout based on a network platform was conducted in high and low infection regions during the COVID-19 outbreak in China. The demographic characteristics, medical-work-related factors, risk of getting infected due to occupational exposure, and family factors were collected by the self-reported questionnaire. The Chinese version of the Maslach Burnout Inventory (CMBI) and the Trait Coping Style Questionnaire (TCSQ) were employed in this study to evaluate the job burnout and coping style, respectively. Furthermore, statistical analysis was done to find out the associated factors of job burnout. Results: We collected 880 complete questionnaires from doctors and nurses from February 9, 2020 to February 11, 2020. In this study, the positive rates of three dimensions of burnout (emotional exhaustion, depersonalization, and reduced personal accomplishment) and overall burnout were 9.09, 50.57, 56.59, and 73.98%, respectively. After the statistical analysis, we found that several factors can independently affect the dimensions. Working in the high infection region and negative coping styles can affect all three dimensions at once. More night shift quantity and having symptoms could increase emotional exhaustion and depersonalization, while higher work intensity and senior title could increase emotional exhaustion and reduce personal accomplishment, respectively. Conclusion: The rate of moderate and severe burnout had increased due to the outbreak. More attention should be paid to burnout in HCPs, especially those with negative coping. There were some potential ways to reduce burnout, such as reducing their workload and providing better protection from the virus.

Loibner, M., et al. (2021). "Biosafety Requirements for Autopsies of Patients with COVID-19: Example of a BSL-3 Autopsy Facility Designed for Highly Pathogenic Agents." Pathobiology **88**(1): 37-45.

Information obtained from autopsies of patients infected with high-risk pathogens is an important pillar in managing a proper response to pandemics, particular in the early phase. This is due to the fact that autopsy allows efficient evaluation of comorbidities for risk assessment, as well as identification of key pathophysiological and molecular mechanisms in organs driving the severity of disease which might be important targets for therapeutic interventions. In the case of patients who have died of infection with unknown pathogens, isolation and culture of pathogens from the affected organs is another important opportunity for a proper response to (re)emerging infectious diseases. However, the situation of COVID-19 demonstrated that there were concerns about performing autopsies because of biosafety risks. In this review we compare requirements for biosafety level 3 (BSL-3) laboratories from the European Commission and the World Health Organization and summarize specific recommendations for postmortem analysis of COVID-19-deceased patients from the Centers for Disease Control and Prevention. Furthermore, we describe in detail a BSL-3 facility with enhanced protection of personnel and an environment that has been designed for performing autopsies, biobanking of collected tissue specimens, and culture of pathogens in cases of high-risk pathogen infections and report on the experience obtained in operating this facility in the context of COVID-19.

Loth, A. G., et al. (2021). "Aerosol Exposure During Surgical Tracheotomy in SARS-CoV-2 Positive Patients." Shock **55**(4): 472-478.

Introduction: Since December 2019, the novel coronavirus SARS-CoV-2 has been spreading worldwide. Since the main route of infection with SARS-CoV-2 is probably via contact with virus-containing droplets of the exhaled air, any method of securing the airway is of extremely high risk for the health care professionals involved. We evaluated the aerosol exposure to the interventional team during a tracheotomy in a semiquantitative fashion. In addition, we present novel protective measures. Patients and Methods: To visualize the air movements occurring during a tracheotomy, we used a breathing simulator filled with artificial fog. Normal breathing and coughing were simulated under surgery. The speed of aerosol propagation and particle density in the direct visual field of the surgeon were evaluated. Results: Laminar air flow (LAF) in the OR reduced significantly the aerosol exposure during tracheostomy. Only 4.8 +/- 3.4% of the aerosol was in contact with the surgeon. Without LAF, however, the aerosol density in the inspiratory area of the surgeon is 10 times higher (47.9 +/- 10.8%, P < 0.01). Coughing through the opened trachea exposed the surgeon within 400 ms with 76.0 +/- 8.0% of the aerosol-independent of the function of the LAF. Only when a blocked tube was inserted into the airway, no aerosol leakage could be detected. Discussion: Coughing and expiration during a surgical tracheotomy expose the surgical team considerably to airway aerosols. This is potentially associated with an increased risk for employees being infected by airborne-transmitted pathogens. Laminar airflow in an operating room leads to a significant reduction in the aerosol exposure of the surgeon and is therefore preferable to a bedside tracheotomy in terms of infection prevention. Ideal protection of medical staff is achieved when the procedure is performed under endotracheal intubation and muscle relaxation.

Ma, X. Y., et al. (2020). "Emergency and essential surgical healthcare services during COVID-19 in low- and middle-income countries: A perspective." International Journal of Surgery **79**: 43-46.

The COVID-19 pandemic resulted in significant changes in health care systems worldwide, with low- and middle-income countries (LMIC) sustaining important repercussions. Specifically, alongside cancellation and postponements of non-essential surgical services, emergency and essential surgical care delivery may become affected due to the shift of human and material resources towards fighting the pandemic. For surgeries that do get carried through, new difficulties arise in protecting surgical personnel from contracting SARS-CoV-2. This scarcity in LMIC surgical ecosystems may result in higher morbidity and mortality, in addition to the COVID-19 toll. This paper aims to explore the potential consequences of COVID-19 on the emergency and essential surgical care in LMICs, to offer recommendations to mitigate damages and to reflect on preparedness for future crises. Reducing the devastating consequences of the COVID-19 pandemic on LMIC emergency and essential surgical services can be achieved through empowering communities with accurate information and knowledge on prevention, optimizing surgical material resources, providing quality training of health care personnel to treat SARS-CoV-2, and ensuring adequate personal protection equipment for workers on the frontline. While LMIC health systems are under larger strain, the experience from previous outbreaks may aid in order to innovate and adapt to the current pandemic. Protecting LMIC surgical ecosystems will be a pivotal process in ensuring that previous health system strengthening efforts are preserved, comprehensive care for populations worldwide are ensured, and to allow for future developments beyond the pandemic.

Maal-Bared, R., et al. (2021). "Implications of SARS-CoV-2 on current and future operation and management of wastewater systems." Water Environment Research **93**(4): 502-515.

While researchers have acknowledged the potential role of environmental scientists, engineers, and industrial hygienists during this pandemic, the role of the water utility professional is often overlooked. The wastewater sector is critical to public health protection and employs collection and treatment system workers who perform tasks with high potential for exposures to biological agents. While various technical guidances and reports have initially provided direction to the water sector, the rapidly growing body of research publications necessitates the constant review of these papers and data synthesis. This paper presents the latest findings and highlights their implications from a water and wastewater utility operation and management perspective. Practitioner points Extrapolation from SARS-CoV-1 and MERS-CoV, as well as other surrogates, has helped predicting SARS-CoV-2 behavior and risk management. Data from treated wastewater effluent suggest that current processes are sufficient for SARS-CoV-2 control. Scientific evidence supports the possibility of fecal-oral transmission for SARS-CoV-2. Limited evidence supports the potential survival of infective SARS-CoV-2 on surfaces and in aerosols and the efficacy of control measures at reducing transmission. Protective practices and PPE can protect workers from SARS-CoV-2 and other pathogens found in wastewater.

Maal-Bared, R. and J. Loudon (2020). "Strategies for managing N95 mask shortages at water resource recovery facilities during pandemics: a review." Water Science and Technology **82**(12): 2798-2812.

As the numbers of COVID-19 cases grew globally, the severe shortages of health care respiratory protective equipment impacted the ability of water resource recovery facilities (WRRFs) to acquire N95 masks for worker protection. While the Occupational Safety and Health Administration (OSHA) encourages WRRFs to conduct job safety assessments to mitigate risks from bioaerosols, it does not provide clear guidance on respiratory protection requirements, leaving the use of N95 masks across the industry non-standardized and difficult to justify. Strategies need to be developed to cope with shortages during pandemics, and these should take into consideration a WRRF's size and disinfection equipment available. Our objective is to provide an overview of respiratory protection-related practices recommended for health care professionals that apply to WRRFs (e.g., elimination, substitution, extended use, reuse, disinfection). Reviewed N95 mask disinfection strategies included using hydrogen peroxide, autoclaving, moist heat, dry heat, ultraviolet germicidal irradiation (UVGI), ethylene oxide, chlorine and ethanol. Of these, dry heat, autoclaving and UVGI present the most promise for WRRFs, with UVGI being limited to larger utilities. We recommend that WRRFs work closely with disinfection technology manufacturers, mask providers, health and safety staff and inspectors to develop suitable programs to cope with N95 mask shortages during pandemics.

Maglitto, F., et al. (2021). "Virtual Surgical Planning and the "In-House" Rapid Prototyping Technique in Maxillofacial Surgery: The Current Situation and Future Perspectives." Applied Sciences-Basel **11**(3).

Background: The first applications of computer-aided design/computer-aided manufacturing (CAD/CAM) in maxillofacial surgery date back to the 1980s. Since then, virtual surgical planning (VSP) has undergone significant development and is now routinely used in daily practice. Indeed, in an extraordinary period, such as that of the current COVID-19 pandemic, it offers a valuable tool in relation to the protection of healthcare workers. In this paper we provide a comprehensive summary of the clinical applications reported in the literature and review our experience using an in-house rapid prototyping technique in the field of maxillofacial surgery. methods: Our research was focused on reconstructive surgery, traumatology (especially in relation to orbital floor and zygomatic arch fractures), and COVID-19 masks. The first step was a radiographic study. Next, computed tomography (CT) scans were segmented in order to obtain a three-dimensional (3D) model. Finally, in the editing phase, through the use of specific software, a customized device for each patient was designed and printed. results: Four reconstructive procedures were performed with a perfect fitting of the surgical device produced by means of VSP. In nine orbital floor fracture cases a good overlapping of the mesh on the orbital floor was obtained. In sixteen zygomatic arch cases the post-operative CT scan showed an excellent fitting of the device and a correct fracture reduction. Regarding the COVID-19 period, six masks and shields produced proved to provide effective protection. conclusions: The timescale and costs required for the production of our "home-made" virtual design are low, which makes this method applicable to a large number of cases, for both ordinary and extraordinary activities.

Maillard, J. Y., et al. (2020). "Reducing antibiotic prescribing and addressing the global problem of antibiotic resistance by targeted hygiene in the home and everyday life settings: A position paper." American Journal of Infection Control **48**(9): 1090-1099.

Antimicrobial resistance (AMR) continues to threaten global health. Although global and national AMR action plans are in place, infection prevention and control is primarily discussed in the context of health care facilities with home and everyday life settings barely addressed. As seen with the recent global SARS-CoV-2 pandemic, everyday hygiene measures can play an important role in containing the threat from infectious microorganisms. This position paper has been developed following a meeting of global experts in London, 2019. It presents evidence that home and community settings are important for infection transmission and also the acquisition and spread of AMR. It also demonstrates that the targeted hygiene approach offers a framework for maximizing protection against colonization and infections, thereby reducing antibiotic prescribing and minimizing selection pressure for the development of antibiotic resistance. If combined with the provision of clean water and sanitation, targeted hygiene can reduce the circulation of resistant bacteria in homes and communities, regardless of a country's Human Development Index (overall social and economic development). Achieving a reduction of AMR strains in health care settings requires a mirrored reduction in the community. The authors call upon national and international policy makers, health agencies, and health care professionals to further recognize the importance of targeted hygiene in the home and everyday life settings for preventing and controlling infection, in a unified quest to tackle AMR. (C) 2020 Association for Professionals in Infection Control and Epidemiology, Inc. Published by Elsevier Inc.

Majernik, M., et al. (2021). "Environmental Waste Management of Disposable Surgical Coverage." Polish Journal of Environmental Studies **30**(6): 5163-5174.

The sustainable direction in the field of patient and medical staff health safety is the use of disposable medical devices, including disposable surgical masks, as recommended by the actual European standard. Disposable surgical coverage is the result of cooperation between medical professional workplaces worldwide and professionals in the area of nonwovens and lamination of material. Various variants were developed, tested and then rejected and then finally reach the material and variant composition. The use of disposable medical devices in healthcare is now a common practice, but also an environmental problem on a global scale. Despite the widespread adoption of this best practice, many healthcare facilities and patients still register resistance and scepticism against this form, actually also in connection with protection against COVID-19. The promoted opinion also by research-confirmed opinion is that the disposable form significantly reduces the amount of regulated medical waste that is generated during surgical procedures, subsequent sterilization and in the framework of protection against COVID-19. At the same time, logistical modeling of the best practice of environmental management in waste management remains a problem for the future.

Malekpour, F., et al. (2021). "Prevention measures and risk factors for COVID-19 in Iranian workplaces." Work-a Journal of Prevention Assessment & Rehabilitation **69**(2): 327-330.

BACKGROUND: Exposure to the coronavirus (COVID-19) in industrial workplaces has caused concerns all over the world. Implementation of prevention measures is the best solution for protection of workers' health. OBJECTIVE: The present study investigated prevention measures and risk factors for the COVID-19 infection in Iranian small, medium, and large industries. METHODS: This study was conducted on 349 occupational health, safety, and environment professionals working in different industries. Data was collected using a checklist of prevention measures for COVID-19 and a questionnaire of risk factors for the infection. RESULTS: The results demonstrated that 32.60% of industries reported positive cases of COVID-19. The mean of prevention measures in all the dimensions, except use of personal protective equipment (PPE), was in favorable status. Among prevention measures against COVID-19, the dimensions of education, engineering controls, use of PPE, use of hand washing soap and disinfectant, and physical distance were not significantly different among small, medium, and large industries. However, administrative controls were statistically lower in small and medium industries compared to the large industries. Moreover, screening was statistically lower in small industries than in large industries. The mean risk factors of ventilation, use of common tools and equipment, and physical distance as well as total risk factors were not statistically different among different industries. CONCLUSION: Workers are at risk of COVID-19 infection. The risk factors of ventilation, sharing tools and equipment, and physical distance are critical in classification of risk of infection with COVID-19 in industrial settings.

Mani, A., et al. (2021). "COVID-19 and cardiac health: A review." Journal of the Practice of Cardiovascular Sciences **7**(1): 24-30.

The ongoing novel coronavirus pandemic has caused a serious impact on patients suffering from cardiovascular disorders as they are predisposed to COVID infection as well as to exacerbation of their preexisting conditions which can prove to be fatal. Novel coronavirus disease-2019 (nCOVID-19) has a varied effect on the cardiac system ranging from myocardial injury to thromboembolic complications. A significant proportion of patients are noted to have comorbidities. Human angiotensin-converting enzyme 2 (ACE 2) receptor is considered the target of the severe acute respiratory syndrome coronavirus 2 (SARS-Cov-2). SARS-Cov-2 leads to imbalance in RAAS activity which is responsible for target organ damage. Recombinant ACE 2 has been shown to restore ACE 2 levels in explanted hearts and restore balanced RAAS activity. nCOVID-19 can have varied cardiac manifestations ranging from acute coronary syndrome to heart failure, arrhythmias, pulmonary thromboembolism, and pericarditis. The current nCOVID-19 pandemic has led to changes in management strategies for cardiovascular diseases. Patients with ST-elevation myocardial infarction can be fibrinolysed when a primary percutaneous coronary intervention facility with adequate protection is not available. Angiotensin-converting enzyme inhibitor/angiotensin receptor blocker needs to be continued in patients with hypertension. Continuity of care for patients with chronic cardiovascular illness needs to be maintained. Management of cardiovascular emergencies needs to be done in a way which ensures the safety of health-care professionals and prevents infection transmission. Strict prevention of infection and health control measures will help to prevent spread of infection and reduce disease incidence.

Mantelakis, A., et al. (2021). "Availability of Personal Protective Equipment in NHS Hospitals During COVID-19: A National Survey." Annals of Work Exposures and Health **65**(1): 136-140.

Objectives: The continuous supply of personal protective equipment (PPE) in the National Health Service (NHS) is paramount to reduce transmission of COVID-19 to patients, public, and staff. Methods: A 16-question survey was created to assess the availability and personal thoughts of healthcare professionals regarding PPE supply in England. The survey was distributed via social media (Facebook (c) and Twitter (c)) to UK COVID-19 healthcare professional groups, with responses collected over 3 weeks in March 2020 during the beginning of the pandemic. Results: A total of 121 responses from physicians in 35 different hospitals were collected (105 inpatient wards, 16 from intensive care units). In inpatient wards, eye and face protection were unavailable to 19% (20/105). Fluid-resistant surgical masks were available to 97% (102/105) whereas filtering facepiece class 3 (FFP3) respirator for 53% (56/105) of respondents. Gloves were accessible for all respondents ( 100%). Body protection was available primarily as a plastic apron 84% (88/105). All of respondents working in intensive care had access to full-body PPE, except FFP3 respirators (available in 88%, 14/16). PPE is 'Always' available for 30% (36/121) of all respondents. There was a statistically significant difference between London and non-London respondents that 'Always' had PPE available (44 versus 19%, P = 0.003). Conclusions: Our survey demonstrated an overall lack of PPE volume supply in the UK hospitals during March 2020, demonstrating a lack of preparedness for a pandemic. PPE was more readily available in London which was the epicentre of the outbreak. Eye and full body protection are in most lack of supply.

Maria, G. A., et al. (2020). "Urologic surgery and invasive procedures during coronavirus pandemic: Retrospective comparison of risk infection in a referral Covid hospital and in a free-Covid hospital." Urologia Journal **87**(4): 163-166.

Introduction: On 21 February 2020, 'Luigi Sacco' Hospital was identified as a Covid-19 referral Hospital in Lombardy. The Department of Urology of our Healthcare Institution consists of two Urology Units, one at 'L.Sacco' Hospital (hereinafter referred to as Covid-19 hospital) and one at 'Fatebenefratelli' Hospital (hereinafter referred to as Covid-19-free hospital). The Healthcare System's Administration communicated to all personnel the implementation of a planned 'biological risk protocol' at the Covid-19 hospital, while the Covid-19-free hospital followed regular government recommendations. We evaluated the risk of Sars-Cov-2 infection in the patients treated for surgical or invasive urologic procedures during the epidemic in the two different hospitals. Materials and methods: At the Covid-19 hospital, 12 patients underwent surgery and 51 patients invasive diagnostic procedures between 22 February and 8 March 2020; in the same period, at the Covid-19-free hospital, 21 patients underwent surgery and 56 patients invasive diagnostic procedures. We compared the incidence of Sars-Cov-2 infection among the patients who were accessed in the two Urology Units in the period of different restrictive measures after the outbreak of the epidemic. Results: We registered no cases of Sars-Cov-2 infection in the patients hospitalized in that period in Covid-19 hospital, despite three cases of swab positivity in the Covid-19-free hospital. Conclusion: The early implementation of extraordinary measures to restrict the spread of the virus offers a good protection also in a Covid-19 referral Hospital. The adoption of safety measures may be considered even after the end of the pandemic in all the health systems.

Martinez-Martin, N., et al. (2020). "Ethics of Digital Mental Health During COVID-19: Crisis and Opportunities." Jmir Mental Health **7**(12).

Social distancing measures due to the COVID-19 pandemic have accelerated the adoption and implementation of digital mental health tools. Psychiatry and therapy sessions are being conducted via videoconferencing platforms, and the use of digital mental health tools for monitoring and treatment has grown. This rapid shift to telehealth during the pandemic has given added urgency to the ethical challenges presented by digital mental health tools. Regulatory standards have been relaxed to allow this shift to socially distanced mental health care. It is imperative to ensure that the implementation of digital mental health tools, especially in the context of this crisis, is guided by ethical principles and abides by professional codes of conduct. This paper examines key areas for an ethical path forward in this digital mental health revolution: privacy and data protection, safety and accountability, and access and fairness.

Martinkevich, P., et al. (2020). "Physical child abuse demands increased awareness during health and socioeconomic crises like COVID-19 A review and education material." Acta Orthopaedica **91**(5): 527-533.

Background and purpose - Physical abuse of children, i.e., nonaccidental injury (NAI) including abusive head trauma (AHT) is experienced by up to 20% of children; however, only 0.1% are diagnosed. Healthcare professionals issue less than 20% of all reports suspecting NAI to the responsible authorities. Insufficient knowledge concerning NAI may partly explain this low percentage. The risk of NAI is heightened during health and socioeconomic crises such as COVID-19 and thus demands increased awareness. This review provides an overview and educational material on NAI and its clinical presentation. Methods - We combined a literature review with expert opinions of the senior authors into an educational paper aiming to help clinicians to recognize NAI and act appropriately by referral to multidisciplinary child protection teams and local authorities. Results - Despite the increased risk of NAI during the current COVID-19 crisis, the number of reports suspecting NAI decreased by 42% during the lockdown of the Danish society. Healthcare professionals filed only 17% of all reports of suspected child abuse in 2016. Interpretation - The key to recognizing and suspecting NAI upon clinical presentation is to be aware of inconsistencies in the medical history and suspicious findings on physical and paraclinical examination. During health and socioeconomic crises the incidence of NAI is likely to peak. Recognition of NAI, adequate handling by referral to child protection teams, and reporting to local authorities are of paramount importance to prevent mortality and physical and mental morbidity.

Mathew, E., et al. (2021). "Antimicrobial 3D Printed Objects in the Fight Against Pandemics." 3d Printing and Additive Manufacturing **8**(1): 79-86.

The uncharted nature of the COVID-19 pandemic has caused uncertainty globally, resulting in many health care professionals and key-workers being left with supply shortages in medical consumables and personal protective equipment, exacerbated by supply line issues and in some cases delays resulting from governmental policies. 3D printing (3DP) has played an important role in providing essential items to hospitals and the wider communities, such as visors, face masks, and ventilator components. This short-review article covers the potential of antimicrobial materials in the manufacturing of 3DP essential products, as an approach for added protection against pandemics.

Matuschek, C., et al. (2020). "Measures of infection prevention and incidence of SARS-CoV-2 infections in cancer patients undergoing radiotherapy in Germany, Austria and Switzerland." Strahlentherapie Und Onkologie **196**(12): 1068-1079.

Purpose COVID-19 infection has manifested as a major threat to both patients and healthcare providers around the world. Radiation oncology institutions (ROI) deliver a major component of cancer treatment, with protocols that might span over several weeks, with the result of increasing susceptibility to COVID-19 infection and presenting with a more severe clinical course when compared with the general population. The aim of this manuscript is to investigate the impact of ROI protocols and performance on daily practice in the high-risk cancer patients during this pandemic. Methods We addressed the incidence of positive COVID-19 cases in both patients and health care workers (HCW), in addition to the protective measures adopted in ROIs in Germany, Austria and Switzerland using a specific questionnaire. Results The results of the questionnaire showed that a noteworthy number of ROIs were able to complete treatment in SARS-CoV-2 positive cancer patients, with only a short interruption. The ROIs reported a significant decrease in patient volume that was not impacted by the circumambient disease incidence, the type of ROI or the occurrence of positive cases. Of the ROIs 16.5% also reported infected HCWs. About half of the ROIs (50.5%) adopted a screening program for patients whereas only 23.3% also screened their HCWs. The range of protective measures included the creation of working groups, instituting home office work and protection with face masks. Regarding the therapeutic options offered, curative procedures were performed with either unchanged or moderately decreased schedules, whereas palliative or benign radiotherapy procedures were more often shortened. Most ROIs postponed or cancelled radiation treatment for benign indications (88.1%). The occurrence of SARS-CoV-2 infections did not affect the treatment options for curative procedures. Non-university-based ROIs seemed to be more willing to change their treatment options for curative and palliative cases than university-based ROIs. Conclusion Most ROIs reported a deep impact of SARS-CoV-2 infections on their work routine. Modification and prioritization of treatment regimens and the application of protective measures preserved a well-functioning radiation oncology service and patient care.

Mavioglu, H. L., et al. (2020). "Perioperative planning for cardiovascular operations in the COVID-19 pandemic." Turk Gogus Kalp Damar Cerrahisi Dergisi-Turkish Journal of Thoracic and Cardiovascular Surgery **28**(2): 236-243.

Coronavirus disease-2019 (COVID-19) is a serious health concern which alert all healthcare professionals worldwide. There are two main issues caused by this pandemic regarding for the healthcare system. First, it is a necessity to postpone many elective procedures/surgeries to reduce the burden of the healthcare system which may be confronted with strain by the increased speed of transmission. Therefore, we aimed to develop an algorithm called as Level of Priority to identify the priority and deferrability of cardiovascular diseases requiring surgery. Second, it is essential to perform surgery and intensive care unit follow-up in a setting where the infection prevention measures are followed for primary patients requiring emergency cardiovascular surgery. In this article, we present necessary precautions to be exercised to provide the patient safety and the highest level of protection for the surgical team.

Maza-Solano, J. M., et al. (2020). "Strategies for the practice of otolaryngology and head and neck surgery during the monitoring phase of COVID-19." Acta Otorrinolaringologica Espanola **71**(6): 367-378.

The appearance of a new coronavirus disease called COVID-19 at the end of 2019 and its pandemic expansion in the world has changed the usual practice of the specialty of Otorhinolaryngology (ENT). After a phase of exponential growth of infections, it has been possible to enter a phase of control of the spread of the disease in which the possibility of infection persists, and the appearance of new cases is considered acceptable by the health system. The aim of this document is to review the available evidence and propose strategies and recommendations for the medical-surgical practice of otorhinolaryngology and head and neck surgery, which allow establishing the usual activity, adapting the safety and efficacy standards to the current situation. Therefore, it is required to identify and classify patients according to criteria of infectious-immunological status, and to establish recommendations for protection in consultations, hospitalization and the operating room, which avoid the transmission of the disease to other users and healthcare personnel, in the specific context of the development of our specialty. This document is the result of the collaboration of all the scientific commissions and the SEORLCCC COVID-19 committee. (C) 2020 Sociedad Espanola de Otorrinolaringologia y Cirugia de Cabeza y Cuello. Published by Elsevier Espana, S.L.U. All rights reserved.

McDonald, T. (2020). "Getting the COVID-19 pandemic into perspective: a nursing imperative." International Nursing Review **67**(3): 305-317.

As we approach the seventh month of the latest pandemic to wreak havoc and death across all societies, it beggars belief that many world and national leaders have acted surprised at the speed and destructive efficiency of COVID-19. Nurses are not surprised by the level of misery and devastation that infectious diseases can cause. We have seen it all before. In every outbreak through history, nurses have responded to the dangers that direct engagement with such crises entails. The inherent risks nurses face in controlling potentially fatal pathogens are compounded by flaccid public policy and incompetent management, resulting in poorly resourced preparedness for the epidemics and pandemics that routinely punctuate our history. That nurses are repeatedly placed in such precarious work environments is an indictment on all involved. Over time, nurses responding to populations in crisis have learned not to rely too heavily on those occupying leadership positions for front-line resources and protection or even early warnings or open disclosure about contagion risk, case numbers and outcomes. Once the danger has passed, the usual public expressions of gratitude for nurses who put themselves in harm's way to help victims of these catastrophes seem to fade, amid hasty efforts to consolidate policies, processes and hierarchies back to the same settings that allowed the current crisis to flourish. So far in this pandemic, an unconfirmed number of nurses and other health workers have either died usually as a result of their involvement with infectious patients or become infected themselves. Data collection on this is haphazard at best, with nurse deaths absorbed within the aggregated morbidity data reported on during pandemics. This is despite nurses being the prominent professional workforce providing crucial front-line interventions to protect public health. It is time nurses involved themselves with the overdue reformation of public health policies, information management and health systems that would seek to return nurses to precarious work environments and trivialize foreseeable risks to us, our communities and patients.

Meister, K. D., et al. (2021). "Multidisciplinary Safety Recommendations After Tracheostomy During COVID-19 Pandemic: State of the Art Review." Otolaryngology-Head and Neck Surgery **164**(5): 984-1000.

Objective In the chronic phase of the COVID-19 pandemic, questions have arisen regarding the care of patients with a tracheostomy and downstream management. This review addresses gaps in the literature regarding posttracheostomy care, emphasizing safety of multidisciplinary teams, coordinating complex care needs, and identifying and managing late complications of prolonged intubation and tracheostomy. Data Sources PubMed, Cochrane Library, Scopus, Google Scholar, institutional guidance documents. Review Methods Literature through June 2020 on the care of patients with a tracheostomy was reviewed, including consensus statements, clinical practice guidelines, institutional guidance, and scientific literature on COVID-19 and SARS-CoV-2 virology and immunology. Where data were lacking, expert opinions were aggregated and adjudicated to arrive at consensus recommendations. Conclusions Best practices in caring for patients after a tracheostomy during the COVID-19 pandemic are multifaceted, encompassing precautions during aerosol-generating procedures; minimizing exposure risks to health care workers, caregivers, and patients; ensuring safe, timely tracheostomy care; and identifying and managing laryngotracheal injury, such as vocal fold injury, posterior glottic stenosis, and subglottic stenosis that may affect speech, swallowing, and airway protection. We present recommended approaches to tracheostomy care, outlining modifications to conventional algorithms, raising vigilance for heightened risks of bleeding or other complications, and offering recommendations for personal protective equipment, equipment, care protocols, and personnel. Implications for Practice Treatment of patients with a tracheostomy in the COVID-19 pandemic requires foresight and may rival procedural considerations in tracheostomy in their complexity. By considering patient-specific factors, mitigating transmission risks, optimizing the clinical environment, and detecting late manifestations of severe COVID-19, clinicians can ensure due vigilance and quality care.

Melidis, C. and M. Vantsos (2020). "Ethical and practical considerations on cancer recommendations during COVID-19 pandemic." Molecular and Clinical Oncology **13**(3).

National and international authorities and societies have recently published important cancer treatment recommendations in order to propose extra measures that should be taken during the COVID-19 pandemic, such as prioritisation of intend-to-cure treatments and younger patients, omission of non-urgent cases, and reduction of personnel present. These measures raise important ethical considerations, since they prioritise protection of Health Systems and Professionals without seemingly taking cancer patient feelings of stress into consideration. This could lead to an erosion of the physician-patient relationship, which is considered the core element of medical ethics. Moreover, they raise practical concerns about the continuous education of Health Professionals, the status of reference centres and the evaluation of the hitherto cancer treatments.

Menon, A., et al. (2020). "Medical Students Are Not Essential Workers: Examining Institutional Responsibility During the COVID-19 Pandemic." Academic Medicine **95**(8): 1149-1151.

In light of the evolving COVID-19 pandemic, the Association of American Medical Colleges (AAMC) and Liaison Committee on Medical Education (LCME) released a joint statement in March 2020 recommending an immediate suspension of medical student participation in direct patient contact. As graduating medical students who will soon begin residency, the authors fully support this recommendation. Though paid health care workers, like residents, nurses, and environmental services staff, are essential to the management of COVID-19 patients, medical students are not. Students' continued involvement in direct patient care will contribute to SARS-CoV-2 exposures and transmissions and will waste already limited personal protective equipment. By decreasing nonessential personnel in health care settings, including medical students, medical schools will contribute to national and global efforts to "flatten the curve." The authors also assert that medical schools are responsible for ensuring medical student safety. Without the protections provided to paid health care workers, students are uniquely disadvantaged within the medical hierarchy; these inequalities must be addressed before medical students are safely reintegrated into clinical roles. Although graduating medical students and institutional leadership may worry that suspending clinical rotations might prevent students from completing graduation requirements, the authors argue the ethical obligation to "flatten the curve" supersedes usual teaching responsibilities. Therefore, the authors request further guidance from the LCME and AAMC regarding curricular exemptions/alternatives and adjusted graduation timelines. The pool of graduating medical students affected by this pause in direct patient contact represents a powerful reserve, which may soon need to be used as the COVID-19 pandemic continues to challenge the U.S. health care infrastructure.

Mergen, V., et al. (2020). "COVID-19 Imaging in a Tertiary Care Setting - The Zurich Approach." Eurasian Journal of Medicine and Oncology **4**(3): 260-263.

The COVID-19 pandemic not only poses an existential threat to the entire society, but also poses a major challenge for hospitals, which cannot be met without interdisciplinary teamwork. For radiological institutions, trade-offs need to be made between radiological imaging capacity required for timely diagnosis of COVID-19, available resources for non-COVID-19-related diseases as well as the risk of infection to healthcare co-workers such as technicians and radiologists. In this study, we describe the scheme implemented at the University Hospital Zurich (Switzerland). The essence of the Zurich approach is early triaging of patients into four categories with either (i) proven COVID-19, (ii) high-risk of COVID-19, (iii) low risk of COVID-19, and (iv) no suspicion of COVID-19, with subsequent procedures including separation of patient pathways taking into account the availability of imaging modalities and protection measures for exposed healthcare workers. With the imposed measures, we were able to avoid any infections to healthcare workers at our hospital over a 3 months period at the height of the COVID-19 crisis in spring 2020. We hope to provide helpful insights for radiological institutions preparing for or coping with the COVID-19 pandemic with the following description.

Meyer, J. C., et al. (2021). "Vaccination against influenza saves lives - a 2021 update." Sa Pharmaceutical Journal **88**(2): 13-18.

Influenza is a highly contagious, acute respiratory viral infection responsible for annual epidemics causing severe morbidity and mortality. Vaccination remains the most effective, live-saving preventative strategy, especially amongst populations at high risk of developing influenzarelated complications. The inactivated trivalent seasonal influenza vaccine contains two World Health Organization (WHO)-recommended influenza A and one influenza B strains, while the quadrivalent vaccine contains an additional B strain, providing broader protection against co-circulating influenza B lineages. Healthcare workers are instrumental in ensuring effective communication about the benefits of influenza vaccination to achieve optimal seasonal influenza vaccination coverage. This article provides an updated overview of the influenza vaccines with special consideration of the current COVID-19 pandemic.

Milionis, C., et al. (2021). "Maintaining fair and efficient health care during the COVID-19 pandemic: Theoretical context and practical implications." International Journal of Care Coordination **24**(1): 5-9.

Coronavirus disease 2019 is a respiratory infection that has evolved to a pandemic with an enormous burden both on human life and health care. States throughout the world have pursued strategies to restrict the transmission of the virus in the community. Health systems have a crucial dual role as they are at the frontline of the fight against the pathogen and at the same time they must continue to offer emergency and routine health services. The provision of health care in the context of the COVID-19 pandemic finds certain barriers. The simultaneous protection of both universal health coverage and health care efficiency is a difficult task due to conflicting challenges of these two goals. Key actions need to be decided and implemented in the fields of health policy, operation of health services, and clinical interaction between health personnel and patients, so that health care continues to perform its mission in a sustainable manner. As the scientific community prepares for the widespread production and application of effective protective and therapeutic agents against COVID-19, it is vital for the general population to remain safe and for the health systems to survive. Allocation of resources and priority setting need to be applied fairly and efficiently for the achievement of the maximum benefit.

Mittal, N. and N. Singh (2020). "Measures in Controlling of Contamination by Covid-19 in Dental Office." Bioscience Biotechnology Research Communications **13**(10): 53-55.

On 31 December 2019, the World Health Organization (WHO) was informed of cases of pneumonia by unknown microbial aetiology. It was associated with Wuhan City, Hubei Province, China. Later, WHO declared that a novel corona virus had been recognized in samples taken from these patients and it spread rapidly around the world was called Corona Virus disease 2019 (COVID-19). On 11 March 2020, WHO declared it as a pandemic. Clinical trials and investigations are ongoing to learn more about the virus and its management. In oral healthcare, aerosols are generated during dental treatment and COVID-19 spreads by respiratory droplets and also by aerosols. Airborne contamination should be controlled by triage, engineering, and workplace controls followed by personal protection equipment (PPE) which are necessary for oral health care workers.

Modi, T., et al. (2020). "COVID-19: How the Radiology Department Should Combat this Global Pandemic." Journal of Clinical and Diagnostic Research **14**(9).

The impact of the global pandemic due to novel Coronavirus Disease (nCOVID-19) has been braced by all medical subspecialties, including the radiology department. As Computerised Tomography (CT) of the chest as well as radiographs gain more and more importance in diagnosing, following up and prognosticating this respiratory infection, it becomes essential to have set protocols in place regarding imaging, disinfection and departmental workflow to ensure smooth functioning and protection of patients and health care workers. We have formulated the "AMMO" protocol as a guide to smooth functioning of the radiology department, which enumerates the steps to be taken for organisation of patient workload, organising equipment, disinfection protocols, maintaining the health and safety of radiology healthcare workers along with timely and accurate reporting. By combining the established guidelines and the knowledge gained from our experience at a dedicated COVID-19 hospital, this article aims to provide a reference in the management of radiology departments during this pandemic.

Moey, P. K. S., et al. (2021). "What are the measures taken to prevent COVID-19 infection among healthcare workers? A retrospective study in a cluster of primary care clinics in Singapore." BMJ Open **11**(6).

Objective To examine factors contributing to the low COVID-19 infectivity rate among healthcare workers in SingHealth Polyclinics (SHP), Singapore, from February to July 2020. Design Retrospective description, analysis and discussion of the factors and their contribution. Setting Single-institution study. Methods We describe and discuss the healthcare policies, infrastructure, people and processes contributing to the low COVID-19 infectivity rate in SHP. There were 1212 full-time and 198 contract staff. Of these, 171 SHP employees also supported the work in dormitories, isolation and community care facilities. During the review period, healthcare workers (HCWs) in SHP managed about 867 076 patient attendances, including 63 503 for upper respiratory tract infections, across its cluster of eight polyclinics. 29 642 swabs for COVID-19 were performed in SHP, with 126 positive results. 395 swabs were carried out in the dormitories and 59 were positive. Despite the high exposure, only two SHP staff were infected with COVID-19. Both have recovered well. Results Provision of adequate personal protection equipment, zonal segregation of high-risk patients, reduction in physical patient visits, effective staff communication, implementation of self-declared temperature monitoring and the maintenance of sustainable workload and work hours of HCWs contributed to the mitigation of COVID-19 infection risk among our staff. Conclusions Until the widespread uptake of safe and effective vaccines against COVID-19, these measures are important in protecting HCWs. They are also important when managing future pandemics of similar nature to COVID-19.

Mojtahedzadeh, N., et al. (2021). "Job Demands, Resources and Strains of Outpatient Caregivers during the COVID-19 Pandemic in Germany: A Qualitative Study." International Journal of Environmental Research and Public Health **18**(7).

The COVID-19 pandemic has affected health professionals in a special way, as they are responsible for the care of vulnerable groups. Little is known about how outpatient caregivers perceive their working conditions during the pandemic in Germany and about the difficulties they face. The aims of this study were (1) to examine specific job demands of outpatient caregivers in regard to the COVID-19 pandemic, (2) to illuminate their job resources they can rely on and (3) to identify potential strain reactions they experience. Fifteen semi-structured telephone interviews were conducted with outpatient caregivers working in Northern Germany in the period May-June 2020. Interviews were analyzed by using qualitative content analysis. Outpatient caregivers experienced daily mask obligation, lack of personal protection equipment (PPE) and stricter hygiene regulations as demanding during the pandemic. They also described a higher workload and emotional demands such as fear of infection or infecting others. They perceived team spirit and communication as important work-related resources. Depressive symptoms and feelings of stress were described as strain reactions. Outpatient care services need to be better prepared for sudden pandemic situations and provide their employees with sufficient PPE and education to reduce pandemic-related job demands leading to negative strain reactions.

Moreno, M. V. M., et al. (2020). "Dentistry in the COVID-19 environment. Adaptation of the dental health units in Madrid's public primary health centres." Revista Espanola De Salud Publica **94**.

The COVID-19 respiratory manifestations go from mild symptoms similar to those of a common cold to severe pneumonia with respiratory distress syndrome, septic shock and multiorgan failure. The disease is caused by the SARS-CoV-2 virus, whose transmission mechanism most relevant to dentistry is through respiratory droplets and possibly also aerosols, as well as direct contact with mucous membranes of the nose, mouth or eye via contaminated hands or objects. The professionals of the dental health units have a high risk exposure since they work at short distances (less than 1 m from the head of the patient) in the oral cavity, where a maximal expression of possible cell receptors for the virus has been reported. Also, most procedures in the dental cabinet imply aerosol generation. Cross-infection is possible during dental care, not only with diagnosed COVID-19-positive patients but also with patients who remain undetected due to asymptomatic or presymptomatic disease. For all these reasons, dental care in the primary health setting has had to change in order to adapt to the pandemic. The changes affect both the appointment scheduling and the care itself and imply the establishment of general and specific barrier protections as well as measures related to ventilation, cleaning, disinfection and sterilization, reinforced with additional infection prevention and control measures. This article summarizes the available scientific evidence about this adaptation.

Mortada, E., et al. (2021). "Preventive Behaviors Towards Covid-19 Pandemic Among Healthcare Providers in Saudi Arabia Using the Protection Motivation Theory." Risk Management and Healthcare Policy **14**: 685-694.

Purpose: The main objectives of the study are firstly to measure the COVID-19 preventive health behaviors related among health care providers (HCPs), then to identify the determinants of such behavior using the protection motivation theory (PMT). Patients and Methods: An online cross-sectional survey, containing closed-ended questions, was distributed among healthcare professionals including physicians, pharmacists, technicians, and nurses. It consisted of questions assessing socio-demographic and occupational characteristics, in addition to questions from the modified PMT that has been tailored for the COVID-19 pandemic through five sub-constructs: perceived severity and perceived vulnerability, response efficacy, self-efficacy, response costs, and behavioral intention. Results: A total of 385 HCPs have participated in the study with a mean age of 40.08 +/- 8.2 years; the majority was Saudi, married, and having children. There was a significant association between intention to comply with COVID-19 preventive behavior and being females, nurses, having training in Infection prevention and control (IPC) measures, and availability of personal protective equipment (PPE) during work time (p=0.01). Other demographic variables, working experience, the status of being in the workforce during the pandemic COVID-19 in Saudi Arabia did not have a significant effect on the intention of HCPs to comply with COVID-19 preventive behavior. The vast majority 85.7% of HCPs answered "always" regarding the behavioral intention of HCPs to comply with COVID-19 preventive behavior. There was a significant positive correlation between COVID-19 behavioral intention and other constructs of PMT model, including perceived severity (r=0.272) perceived vulnerability (r= 0.248), self-efficacy (r=0.218), response-efficacy (r=0.167), and response-cost (r= 0.13). Gender, availability of PPE, and self-efficacy had a significant prediction of COVID-19 behavioral intentions (P < 0.05) with a predicted increase of 0.56, 0.37, and 0.12, respectively, in the mean of the intention score. Self-efficacy was the highest significant predictor of the behavior (p=0.008). Conclusion: Females' gender, nurses, having training in IPC measures, and availability of PPE during work time have a significant association with intention to comply with COVID-19 preventive behavior. The present study coping appraisal particularly self-efficacy predicted the COVID-19 pandemic protection motivation and preventive behavior more than threat appraisal. Therefore, future training programs must consider the level of self-efficacy of HCPS, and increase their knowledge regarding the effectiveness of recommendation strategies to perform protective measures against the COVID-19.

Mueller, S. K., et al. (2021). "Considerations for Continuing Semielective and Emergency Otolaryngological Procedures During the COVID-19 Pandemic." Ent-Ear Nose & Throat Journal **100**(1): 19-25.

Introduction: During the COVID-19 pandemic, worldwide over 600,000 human beings died due to the cause of the disease. In order to deescalate the transmission rate and to avoid crush loading the countries medical health systems social distancing, face masks, and lockdowns have been considered essential by the majority of governments. Whereas some countries have highly reduced or completely stopped otorhinolaryngological procedures, other countries have continued selected surgeries. The objective of this study was to analyze procedures and outcomes of continuing semielective and emergency surgeries during the COVID-19 pandemic. Methods: Retrospective analysis of n = 750 patients who received semi-elective or emergency surgery between March 26 and June 16, 2020, in the Otolaryngology Department of the Friedrich-Alexander-University of Erlangen-Nurnberg. All patients were screened for COVID symptoms and swabbed for SARS-CoV-2 prior to surgery. Results: Of the n = 750 patients, n = 699 patients received semielective surgery and n = 51 emergency surgery. For 27 patients, the swab result could not be awaited due to a life-threatening condition. In these cases, surgery was performed in full protective equipment. No patient was tested positive during or after the surgery (follow-up 45 to 127 days). No member of the medical personnel showed symptoms or was tested positive after contact with patients. Due to the continuation of surgeries, patients' lives were saved and improvement of long-term quality-of-life and outcomes is anticipated. Conclusions: Continuing selected otorhinolaryngological surgeries is crucial for patients' health, survival, and long-time quality of life, yet, the protection of the medical personnel has to be granted.

Munoz-Moreno, R., et al. (2020). "COVID-19 and social services in Spain." PLoS One **15**(11).

During the state of alarm declared in Spain by COVID-19 due to the pandemic, the country's authorities declared Social Services and their workers to be essential, considering that the activity of these professionals with the vulnerable population was crucial and that services should continue to be provided to guarantee the well-being of users in this exceptionally serious situation. This article analyzes the impact that the COVID-19 and the state of alarm has had on Spanish social service professionals. An ad hoc questionnaire was used, administered on-line, individually, voluntarily and anonymously to 560 professionals working in social services, both in the public and private sectors, based throughout Spain. This questionnaire has five different parts: socio-demographic profiling, impact that the health crisis has had on the practice of professional functions, degree of knowledge of the measures imposed to guarantee the protection and safety of professionals and users, impact that it has had on the professional and personal development of social services professionals and, the fifth and last part, degree of adaptation of the measures aimed at the care of the vulnerable population. These results are discussed based on the situation in which professionals working in this sector find themselves in the face of the changes they are experiencing in the development of their work, and we are able to determine the profile of the workers who have felt most affected by the situation, with the consequent and foreseeable mental and emotional affectation that this implies. These professionals tend to value more negatively the set of measures developed to mitigate the impact of COVID-19 on Spanish social services.

Nasrabadi, A. N., et al. (2021). "Challenges with healthcare workers' protection during the COVID-19 in Iran." British Journal of Oral & Maxillofacial Surgery **59**(3): E114-E117.

During the COVID-19 pandemic, healthcare workers stand in the frontline of the fight against the disease. This study aimed to assess the challenges with personal protection during the pandemic in Tehran, Iran. Seven-hundred healthcare workers were surveyed between April and May 2020, and the study was performed in surgical care wards. Questionnaires, which were sent to 1000 workers via online platforms, social networks, and emails to different associations, were used to assess the use of personal protective equipment (PPE) and the reasons for not using it. A total of 700 replied. Face masks and face shields had the highest and lowest use, respectively. Equipment shortage, time constraint, low supervision, and lack of information were the reasons for personnel not using PPE. To reduce the burden of COVID-19 and accelerate the process of patient care, it is important to protect and maintain the well-being of staff. Organised national and international arrangements to protect them are critical. (c) 2020 The British Association of Oral and Maxillofacial Surgeons. Published by Elsevier Ltd. All rights reserved.

Nazarious, M. I., et al. (2020). "Pressure Optimized PowEred Respirator (PROPER): A miniaturized wearable cleanroom and biosafety system for aerially transmitted viral infections such as COVID-19." Hardwarex **8**.

The supply of Personal Protective Equipment (PPE) in hospitals to keep the Health Care Professionals (HCP) safe taking care of patients may be limited, especially during the outbreak of a new disease. In particular, the face and body protective equipment is critical to prevent the wearer from exposure to pathogenic biological airborne particulates. This situation has been now observed worldwide during the onset of the COVID-19 pandemic. As concern over shortages of PPE at hospitals grows, we share with the public and makers' community the Pressure Optimized PowEred Respirator (PROPER) equipment, made out of COTS components. It is functionally equivalent to a Powered Air Purifying Respirator (PAPR). PROPER, a hood-based system which uses open source and easily accessible components is low-cost, relatively passive in terms of energy consumption and mechanisms, and easy and fast to 3D print, build and assemble. We have adapted our experience on building clean room environments and qualifying the bioburden of space instruments to this solution, which is in essence a miniaturized, personal, wearable cleanroom. PROPER would be able to offer better protection than an N95 respirator mask, mainly because it is insensitive to seal fit and it shields the eyes as well. The PROPER SMS fabric is designed for single-use and not intended for reuse, as they may start to tear and fail but the rest of the parts can be disinfected and reused. We provide a set of guidelines to build a low-cost 3D printed solution for an effective PAPR system and describe the procedures to validate it to comply with the biosafety level 3 requirements. We have validated the prototype of PROPER unit for air flow, ISO class cleanliness level, oxygen and carbon-dioxide gas concentrations during exhalation, and present here these results for illustration. We demonstrate that the area inside the hood is more than 200 times cleaner than the external ambient without the operator and more than 175 times with the operator and in an aerosol exposed environment. We also include the procedure to clean and disinfect the equipment for reuse. PROPER may be a useful addition to provide protection to HCPs against the SARS-CoV-2 virus or other potential future viral diseases that are transmitted aerially. (C) 2020 The Authors. Published by Elsevier Ltd.

Ng, B. (2020). "Solutions to prevent and address physician burnout during the pandemic in Mexico." Indian Journal of Psychiatry **62**(9): 467-469.

Background: The COVID-19 pandemic has tested the level of preparedness and readiness of governments globally. The demand for services exceeding the capacity of the health systems in both developed and developing countries has been the rule rather than the exception. Physicians and the rest of the health-care personnel have been put through unprecedented levels of demand, within a field of uncertainty, from an evolving and insufficient understanding of the pathophysiology of the viral process, the unclear benefit of face coverings used by the general public, numerous pharmacological candidates, insufficient personal protection equipment, and the highly expected vaccine. Aims and Objectives: Design a program to address the emotional and psychiatric needs of COVID-19 first response Healthcare personnel in Mexico. Materials: in march 2020, the Mexican Psychiatric Association was invited to be part of the Workgroup for the fortification of Mental Health during Disasters of the Ministry of Health in Mexico. The charge was to develop a program to address the needs and prevent burn out in physicians and the rest of healthcare personal. The details of how this program was planned, implemented, and launched will be presented. Results: The program was launched in two phases. Phase A through a chat with text messaging capability was launched on 25 April, 2020. B through telepsychiatric video calls, was launched on 15 June, 2020. Phase A had a very limited demand. Phase B also had a very limited demand until the month 5 September, 2020. Conclusions: from the time of program launch through the first four months, the demand was very low, what may be explained due to "normalization" of stress and/or stigma among healthcare professionals. Our personnel deserve the utmost support from their society.

Ng, W. T. (2021). "COVID-19: Protection of Workers at the Workplace in Singapore." Safety and Health at Work **12**(1): 133-135.

The COVID-19 pandemic has resulted in movement restrictions being instituted globally and the cessation of work at many workplaces. However, during this period, essential services such as healthcare, law enforcement, and critical production and supply chain operations have been required to continue to function. In Singapore, measures were put in place to protect the workers from infection at the workplace, as well as to preserve the operational capability of the essential service in a COVID-19 pandemic environment. This paper critically analyses the measures that were implemented and discusses the extension to broader general industry. (C) 2020 Occupational Safety and Health Research Institute, Published by Elsevier Korea LLC.

Nienhaus, A. and R. Hod (2020). "COVID-19 among Health Workers in Germany and Malaysia." International Journal of Environmental Research and Public Health **17**(13).

We report on the suspected case reports filed for SARS-CoV-2 infections and COVID-19 illnesses among health and social welfare workers in Germany. In addition, we report about COVID-19 in health workers in Malaysia. Claims for occupational diseases caused by SARS-CoV-2 are recorded separately in a database of the Statutory Accident Insurance and Prevention in the Health and Welfare Services (BGW). This database is analyzed according to its content as of May 22, 2020. In addition, the notifiable cases of SARS-CoV-2 infections from personnel in medical institutions (e.g., clinics and doctor's office) and social welfare institutions (e.g., nursing homes, shelters and refugee camps) following the German Infection Protection Act are analyzed. The report from Malaysia is based on personal experience and publications of the government. In Germany at present, 4398 suspected case reports for the diagnosis of SARS-CoV-2 infections among health and social workers have been filed. This figure is four times the number of all reported infections normally received per year. The majority of claims, regardless of being a confirmed infection, concerned nurses (n = 6927, 63.9%). The mortality rate for workers infected with SARS-CoV-2 is 0.2% to 0.5%. Doctors are affected by severe illness more frequently than other occupational groups (8.1% vs. 4.1%). In Malaysia, work-related infection of health workers (HW) occurred mainly when COVID-19 was not suspected in patients and no adequate personal protective equipment (PPE) was worn. Although knowledge on the spread of SARS-CoV-2 infections among workers remains limited, the impact appears to be substantial. This is supported by the mortality rate among infected workers. Occupational health check-ups carried out at the present time should be systematically analyzed in order to gain more information on the epidemiology of COVID-19 among HW. Since the supply and use of PPE improved, the infection risk of HW in Malaysia seems to have decreased.

Nishimura, T., et al. (2021). "SARS-CoV-2 Infection among Medical Institution Faculty and Healthcare Workers in Tokyo, Japan." Internal Medicine **60**(16): 2569-2575.

Objective To consider effective measures against severe acute respiratory syndrome coronavirus 2 (SARS-CoV-2) infection in medical institutions, this study estimated the SARS-CoV-2 infection rate among health-care workers (HCWs) in Tokyo, Japan, and determined the specific findings for mild coronavirus disease 2019 (COVID-19) cases. Methods This study analyzed the results of serologic tests to detect immunoglobulin G antibodies against SARS-CoV-2 and evaluated the demographic and clinical characteristics of the faculty and HCWs at a Tokyo medical institution in August 2020. The demographic and clinical characteristics of participants with antibody-positive results were compared to those of participants with antibody-negative results. Materials This study recruited 2,341 faculty and HCWs at a Tokyo medical institution, 21 of whom had a COVID-19 history. Results Of the 2,320 participants without a COVID-19 history, 20 (0.862%) had positive serologic test results. A fever and dysgeusia or dysosmia occurred with greater frequency among the participants with positive test results than in those with negative results [odds ratio (OR), 5.475; 95% confidence interval (CI), 1.960-15.293 and OR, 24.158; 95% CI, 2.693-216.720, respectively]. No significant difference was observed in the positivity rate between HCWs providing medical care for COVID-19 patients using adequate protection and other HCWs (OR, 2.514; 95% CI, 0.959-6.588). Conclusion To reduce the risk of COVID-19 spread in medical institutions, faculty and HCWs should follow standard and necessary transmission-based precautions, and those with a fever and dysgeusia or dysosmia should excuse themselves from work as soon as possible.

Nyabi, O., et al. (2021). "Diagnostic Value of IgM and IgG Detection in COVID-19 Diagnosis by the Mobile Laboratory B-LiFE: A Massive Testing Strategy in the Piedmont Region." International Journal of Environmental Research and Public Health **18**(7).

Coronavirus disease 2019 (COVID-19) is an acute infectious disease caused by the novel coronavirus (SARS-CoV-2) identified in 2019. The COVID-19 outbreak continues to have devastating consequences for human lives and the global economy. The B-LiFe mobile laboratory in Piedmont, Italy, was deployed for the surveillance of COVID-19 cases by large-scale testing of first responders. The objective was to assess the seroconversion among the regional civil protection (CP), police, health care professionals, and volunteers. The secondary objective was to detect asymptomatic individuals within this cohort in the light of age, sex, and residence. In this paper, we report the results of serological testing performed by the B-LiFe mobile laboratory deployed from 10 June to 23 July 2020. The tests included whole blood finger-prick and serum sampling for detection of SARS-CoV-2 spike receptor-binding domain (S-RBD) antibodies. The prevalence of SARS-CoV-2 antibodies was approximately 5% (294/6013). The results of the finger-prick tests and serum sample analyses showed moderate agreement (kappa = 0.77). Furthermore, the detection rates of serum antibodies to the SARS-CoV-2 nucleocapsid protein (NP) and S-RBD among the seroconverted individuals were positively correlated (kappa = 0.60), at least at the IgG level. Seroprevalence studies based on serological testing for the S-RBD protein or SARS-CoV-2 NP antibodies are not sufficient for diagnosis but might help in screening the population to be vaccinated and in determining the duration of seroconversion.

Oborn, E., et al. (2021). "Institutional logics and innovation in times of crisis: Telemedicine as digital 'PPE'." Information and Organization **31**(1).

How do crises shape digital innovation? In this paper we examine the rapid adoption of digital telemedicine technologies in an Israeli hospital with a focus on the role of the institutional logics held by the stakeholders responding to emerging events. With the onset of COVID-19, the need for social distancing and minimal physical contact challenged and interrupted hospital practices. In response, remote audio-visual functionality of digital technologies were appropriated in different ways, as stakeholders ? state actors, managers, health professionals, and family members ? sought to improvise and enhance the protection of persons concerned. We show how emerging practices were guided by the dominant institutional logics of stakeholders responding to the crisis. Acting for many as a digital form of ?personal protective equipment? (PPE), the technologies enabled diverse action possibilities to become manifest in practices. We add to understanding the role of institutional logics in directing the attention of stakeholders to shape digital innovation in times of crisis.

O'Connell, D. A., et al. (2020). "Recommendations from the Canadian Association of Head and Neck Surgical Oncology for the Management of Head and Neck Cancers during the COVID-19 pandemic." Journal of Otolaryngology-Head & Neck Surgery **49**(1).

Introduction The SARS-CoV-2 virus (COVID19) pandemic has placed extreme pressures on the Canadian Healthcare system. Many health care regions in Canada have cancelled or limited surgical and non-surgical interventions on patients to preserve healthcare resources for a predicted increase in COVID19 related hospital admissions. Also reduced health interventions may limit the risk of possible transmission of COVID19 to other patients and health care workers during this pandemic. The majority of institutions in Canada have developed their own operational mandates regarding access to surgical resources for patients suffering from Head and Neck Cancers during this pandemic. There is a large degree of individual practitioner judgement in deciding access to care as well as resource allocation during these challenging times. The Canadian Association of Head and Neck Surgical Oncology (CAHNSO) convened a task force to develop a set of guidelines based on the best current available evidence to help Head and Neck Surgical Oncologists and all practitioners involved in the care of these patients to help guide individual practice decisions. Main body The majority of head and neck surgical oncology from initial diagnosis and work up to surgical treatment and then follow-up involves aerosol generating medical procedures (AGMPs) which inherently put head and neck surgeons and practitioners at high risk for transmission of COVID19. The aggressive nature of the majority of head and neck cancer negates the ability for deferring surgical treatment for a prolonged period of time. The included guidelines provide recommendations for resource allocation for patients, use of personal protective equipment for practitioners as well as recommendations for modification of practice during the current pandemic. Conclusion Enhanced triaging should be used to identify patients with aggressive malignancies. These patients should be prioritized to reduce risk of significant disease progression in the reduced resource environment of COVID19 era. Enhanced triaging including aggressive pre-treatment COVID19 testing should be used to identify patients with high risk of COVID19 transmission. Enhanced personal protective equipment (PPE) including N95 masks and full eye protection should be used for any AGMPs performed even in asymptomatic patients. Enhanced PPE including full eye protection, N95 masks and/or powered air purifying respirators (PAPRs) should be used for any AGMPs in symptomatic or presumptive positive COVID 19 patients.

Olszewski, T., et al. (2020). "COVID-19 outbreak and endoscopy: Considerations in patients encountered in a foregut surgery practice." World Journal of Gastrointestinal Surgery **12**(5).

Severe acute respiratory syndrome coronavirus has become a critical challenge to global health. Since the arrival of coronavirus disease 2019 in the United States, several government agencies and professional societies have issued guidelines to healthcare systems and medical providers. Endoscopy is a substantial portion of the practice of many general surgeons in the United States. With upper endoscopy, manipulation of the upper aerodigestive tract can turn the droplets to an aerosolized form and increase the likelihood of transmission and therefore is considered a high-risk procedure. In this article we review some aspects of the coronavirus disease 2019 outbreak that are relevant to practice of surgical endoscopy. The emphasis of this communication is on the mode of transmission, previous experiences during other coronavirus outbreaks and society guidelines. We then highlight the changes that we have made to our practice to incorporate these factors to improve the safety of patients, health care providers, and community as a whole.

Oygar, P. D., et al. (2020). "Education of Healthcare Personnel Working with Pediatric Patients During COVID-19 Pandemic within the Framework of Infection Control." Journal of Pediatric Infection **14**(4): 195-200.

Objective: In the early stages of any epidemic caused by new emerging pathogens healthcare personnel is subject to a great risk. Pandemic caused by SARS-CoV-2, proved to be no exception. Many healthcare workers died in the early stages of pandemic due to inadequate precautions and insufficient protection. It is essential to protect and maintain the safety of healthcare personnel for the confinement of pandemic as well as continuity of qualified healthcare services which is already under strain. Educating healthcare personnel on appropiate use of personal protective equipment (PPE) is as essential as procuring them. Material and Methods: A survey is conducted on 4927 healthcare personnel working solely with pediatric patients from 32 different centers. Education given on PPE usage were questioned and analyzed depending on age, sex, occupation and region. Results: Among four thousand nine hundred twelve healthcare personnel from 32 different centers 91% (n=4457) received education on PPE usage. Of those who received education only 36% was given both theoretical and applied education. Although there was no differences among different occupation groups, receiving education depended on regions. Conclusion: It is essential to educate healthcare personnel appropiately nationwidely for the continuity of qualified healthcare services during the pandemic.

Ozkara, A., et al. (2020). "Effective implementation of unprecedented measures for the protection from COVID-19 syndrome." Bezmialem Science **8**: 63-66.

Despite 82 million populations, Turkey is one of the countries with the lowest mortality rates in the world as a result of successful crisis management and public compliance. Turkey's public health response to the COVID-19 pandemic has been rapid and continually evolving as described here. In this short communication we offer insight into the preparedness and response by Turkey of this continued global health threat posed by COVID-19. Turkey implemented multiple containment strategies prior to the first reported case within its borders- to reduce the burden and deadly risk of the virus. In the absence of a specific vaccine, governments, health care professionals and communities in general are continually working together to reduce exposure, infection, clinical severity and community transmission of COVID-19.

Ozturk, C. N., et al. (2020). "Plastic Surgery and the COVID-19 Pandemic A Review of Clinical Guidelines." Annals of Plastic Surgery **85**(2S): S155-S160.

Background: A novel coronavirus disease (COVID-19) was first reported in December 2019 in China and was soon declared a pandemic by the World Health Organization. Many elective and nonessential surgeries were postponed worldwide in an effort to minimize spread of disease, as well as to conserve resources. Our goal with this article is to review current practice guidelines in setting of the COVID-19 pandemic, based on available data and literature. Methods: Websites pertaining to surgical and medical societies, and government agencies were reviewed, along with recently published literature to identify recommendations related to COVID-19 and plastic surgery procedures. Results: Clinical practice modifications are recommended during the pandemic in outpatient and perioperative settings. Use of personal protective equipment is critical for aerosol-generating procedures, such as surgery in the head and neck area. Care for trauma and malignancy should continue during the pandemic; however, definitive reconstruction could be delayed for select cases. Specific recommendations were made for surgical treatment of cancer, trauma, and semiurgent reconstructive procedures based on available data and literature. Conclusions: The risk and benefit of each reconstructive procedure should be carefully analyzed in relation to necessary patient care, minimized COVID-19 spread, protection of health care personnel, and utilization of resources. Recommendations in this article should be taken in the context of each institute's resources and prevalance of COVID-19 in the region. It should be emphasized that the guidelines provided are a snapshot of current practices and are subject to change as the pandemic continues to evolve.

Pagano, A. M., et al. (2020). "COVID-19 Risk Management and Screening in the Penitentiary Facilities of the Salerno Province in Southern Italy." International Journal of Environmental Research and Public Health **17**(21).

(1) Background: The emergency linked to the spread of COVID-19 in Italy has led to inevitable consequences on the penitentiary system. The risks of this emergency in prisons is mainly related to the problem of persistent overcrowding that makes social distancing difficult and the isolation of any contagion hard to arrange. The Department of Protection for Adults and Minors of the ASL Salerno Criminal Area has taken steps in order to perform screening operations and minimize the risks for prisoners and operators. (2) Methods: We conducted a two-phase observational study. In the first phase, we offered and then executed serum COVID-19 screening to all the convicted inmates. For those who had a doubtful or positive result, a swab was executed in the shortest time possible. In the second phase, a pharyngeal swab was offered and executed to all the police officers, the penitentiary administrative staff and the medical personnel working in the prison. (3) Results: In the first phase, we executed 485 COVID-19 blood tests on prisoners, 3 (0.61%) of which were positive. The three positive inmates underwent nasopharyngeal swabbing, which ultimately were negative. After that, we executed 276 nasopharyngeal swabs on the prison personnel, penitentiary administrative staff and medical personnel-all were negative. (4) Conclusion: All tests (blood tests and swabs) that were carried out on the prisoners and on the staff were negative for COVID-19. We believe that all prisons in Italy and in the world should take action to ensure preventive and control measures in order to safeguard the health of the prison population and of all the people who work there.

Pajor, P. (2021). "Psychiatry in the time of the pandemic. Is COVID-19 changing the discipline?" Postepy Psychiatrii I Neurologii **30**(2): 113-121.

Purpose: COVID-19 is an infectious disease caused by the newly discovered coronavirus SARS-CoV-2. The research conducted to date has focused mainly on the somatic complications of the disease, while only a few studies look at the potential direct mental health effects of the virus and its neurotropic potential. Views: The COVID-19 pandemic is also a psychologically significant stressor, affecting not only every aspect of an individual's life, but also economic and social sectors around the world. The deterioration of mental health can be caused not only by the fear of illness, death, or job loss, and uncertainty about the future, but also social isolation resulting from the loss of structured educational activities, professional activities and sanitary regime. We are slowly beginning to see the progressing effect of the current pandemic - not only the increasing symptoms of panic, anxiety and depression, but also somatization disorders, disadaptive syndromes, chronic fatigue syndrome, psychoactive substance abuse, and possibly also fixed post-pandemic personality disorders. Nosophobia, when it comes to the pandemic, takes the form of "coronaphobia'. Conclusions: The protracted duration of the pandemic poses a threat to mental health. It causes a growing failure of psychological mechanisms for coping with the current situation. The study attempts to identify especially mentally vulnerable social groups, including the impact on health protection professionals from the perspective of a physician specializing in psychiatry. Disturbing social phenomena, highlighted by the pandemic, were identified, and practical aspects of the pandemic discussed - the new tasks that psychiatrists and psychologists face.

Palatnik, A. and J. J. McIntosh (2020). "Protecting Labor and Delivery Personnel from COVID-19 during the Second Stage of Labor." American Journal of Perinatology **37**(08): 854-856.

The novel coronavirus disease 2019 (COVID-19) is spreading fast and is affecting the clinical workers at much higher risk than the general population. Little is known about COVID-19 effect on pregnant women; however, the emerging evidence suggests they may be at high risk of asymptomatic disease. In light of projected shortage of personal protective equipment (PPE), there is an aggressive attempt at conservation. In obstetrics, the guidelines on PPE use are controversial and differ among hospitals, globally, as well as nationally. The centers for disease control and prevention (CDC) recommend using N95 respirators, which are respirators that offer a higher level of protection instead of a facemask for when performing or present for an aerosol-generating procedures (AGP). However, the second stage of labor is not considered an AGP. The second stage of labor can last up to 4 hours. During that time, labor and delivery personnel is in close contact to patients, who are exerting extreme effort during and frequently blow out their breath, cough, shout, and vomit, all of which put the health care team at risk, considering that COVID-19 transmission occurs through aerosol generated by coughing and sneezing. The CDC and the American College of Obstetricians and Gynecologists (ACOG) do not provide clarification on the use of N95 during the second stage. We recommend that labor and delivery personnel have the utmost caution and be granted the protection they need to protect themselves and other patients. This includes providing labor and delivery personnel full PPE including N95 for the second stage of labor. This is critical to ensure the adequate protection for health care workers and to prevent spread to other health care workers and patients.

Pan, L. C., et al. (2021). "Social protection and therapeutic-occupational experiences: life during the Covid-19 pandemic." Interface-Comunicacao Saude Educacao **25**.

This text elaborates some reflections regarding the responses that have been undertaken by the sectors: health, social security and social assistance, which make up social security in Brazil. It assumes its centrality, to face the pandemic caused by SARS-CoV-2. Considering this context and assuming a professional action for social participation with autonomy, we share experiences in Social Occupational Therapy with young people who live in urban peripheries, certainly poor for the market/consumption, but rich in life, in the Covid-19 pandemic. The aim was to produce care that is consistent with social protection and is directed, in defense of the non-negotiable value of each life and of its pulse, towards the promotion of an emancipatory circulation, an issue that existed prior to the pandemic, albeit now aggravated, and always present among this group of young people.

Parlin, A. F., et al. (2020). "A laboratory-based study examining the properties of silk fabric to evaluate its potential as a protective barrier for personal protective equipment and as a functional material for face coverings during the COVID-19 pandemic." PLoS One **15**(9).

The worldwide shortage of single-use N95 respirators and surgical masks due to the COVID-19 pandemic has forced many health care personnel to use their existing equipment for as long as possible. In many cases, workers cover respirators with available masks in an attempt to extend their effectiveness against the virus. Due to low mask supplies, many people instead are using face coverings improvised from common fabrics. Our goal was to determine what fabrics would be most effective in both practices. Under laboratory conditions, we examined the hydrophobicity of fabrics (cotton, polyester, silk), as measured by their resistance to the penetration of small and aerosolized water droplets, an important transmission avenue for the virus causing COVID-19. We also examined the breathability of these fabrics and their ability to maintain hydrophobicity despite undergoing repeated cleaning. Laboratory-based tests were conducted when fabrics were fashioned as an overlaying barrier for respirators and when constructed as face coverings. When used as material in these two situations, silk was more effective at impeding the penetration and absorption of droplets due to its greater hydrophobicity relative to other tested fabrics. We found that silk face coverings repelled droplets in spray tests as well as disposable single-use surgical masks, and silk face coverings have the added advantage over masks such that they can be sterilized for immediate reuse. We show that silk is a hydrophobic barrier to droplets, can be more breathable than other fabrics that trap humidity, and are re-useable via cleaning. We suggest that silk can serve as an effective material for making hydrophobic barriers that protect respirators, and silk can now be tested under clinical conditions to verify its efficacy for this function. Although respirators are still the most appropriate form of protection, silk face coverings possess properties that make them capable of repelling droplets.

Parry, A. H., et al. (2021). "Management of a diagnostic radiology department amid Coronavirus disease-19 (COVID-19) pandemic." Indian Journal of Radiology and Imaging **31**(5): S192-S195.

Severe acute respiratory syndrome coronavirus-2 (SAR-CoV-2) is a highly contagious infectious disease and spreads through aerosols and fomites. Health care personnel who are at the forefront of the fight against coronavirus disease-19 (COVID-19) pandemic are also at greater risk of contracting the infection. Mixing of uninfected people with infected people is potentially hazardous, especially in a radiology department. Implementation of meticulous operational changes, curtailment of nonurgent radiological work, rationalization of staff, equipment disinfection, use of personal protection equipment, and psychological support are needed to combat COVID-19 or any such infectious disease outbreak. This technical note will familiarize radiology workers with infectious disease outbreak-response to be adopted to ensure the safety of staff and patients.

Parush, A., et al. (2020). "Human Factor Considerations in Using Personal Protective Equipment in the COVID-19 Pandemic Context: Binational Survey Study." Journal of Medical Internet Research **22**(6).

Background: Full level 1 personal protective equipment (PPE) is used in various domains and contexts. Prior research has shown influences of such equipment on performance, comfort, and contamination levels. The coronavirus disease (COVID-19) pandemic forced a pervasive requirement of PPE, with little preparation, rushed deployment, inadequate time for training, and massive use by personnel who are inexperienced or not qualified in its effective use. Objective: This study aims to examine the key human factors (physical and ergonomic, perceptual and cognitive) that influence the use of level 1 PPE when attending to patients with suspected or confirmed COVID-19. Methods: The research approach consisted of a short survey disseminated to health care professionals in two countries, Israel and Portugal, with similar demographics and health care systems. The survey included 10 items with a 5-point Likert scale regarding the key human factors involved in level 1 PPE, as identified in prior research. Results: A total of 722 respondents from Israel and 301 respondents from Portugal were included in the analysis. All the respondents reported using level 1 PPE with patients with COVID-19 in the range of several hours daily to several hours weekly. The Cronbach alpha was .73 for Israel and .75 for Portugal. Responses showed high levels of difficulty, with medians of 4 for items related to discomfort (n=539/688, 78% in Israel; n=328/377, 87% in Portugal), hearing (n=236/370, 64% in Portugal; n=321/642, 50% in Israel), seeing (n=697/763, 89% in Israel; n=317/376, 84% in Portugal), and doffing (n=290/374, 77% in Portugal; n=315/713, 44% in Israel). A factor analysis showed a set of strongly related variables consisting of hearing, understanding speech, and understanding the situation. This suggests that degradation in communication was strongly associated with degradation in situational awareness. A subsequent mediation analysis showed a direct effect of PPE discomfort on situational awareness (P<.001); this was also influenced (mediated) by difficulties in communicating, namely in hearing and understanding speech. Conclusions: In 2020, the COVID-19 pandemic is paving the way for updating PPE design. The use of already deployed technology affords ample opportunities to improve, adapt, and overcome caveats. The findings here suggest that the use of level 1 PPE with patients with COVID-19 has perceptual and cognitive effects, in addition to physical and ergonomic influences. Efforts should be taken to mitigate the harmful effects of such influences, both regarding the performance of medical actions and the risk of contamination to health care workers. Such efforts involve the design of PPE; the introduction of technologies to enhance vision, hearing, and communicating during the use of PPE; and training staff in using the equipment and in effective communication and teamwork protocols.

Passarelli, P. C., et al. (2020). "The impact of the COVID-19 infection in dentistry." Experimental Biology and Medicine **245**(11): 940-944.

The novel Coronavirus pandemic defines a new risk for all dental practitioners, hygienists, and dental assistants. As an increasing number of dentists are now developing this disease, we wanted to provide some measures to manage this risk in the dental practice, by undergoing a review of the current literature. This minireview searches the literature for articles that both defined the infection risk in the dental practice and provided evidence on the efficacy of some procedures on reducing the infection risk. Several articles have already pointed out some necessary measures: fewer patients have to be admitted to the practice, a short triage should be carried out, and the appropriate measures of protection have to be used. On the basis of the literature collected, a short questionnaire and a flowchart is proposed to define the risk that each patient carries, and to appropriately adapt each procedure based on the patient's risk. The literature is still limited on this subject, but on the basis of what is available, dental practices have to adapt to the situation in order to protect dental health professionals. Impact statement Dentists have always been taught how to protect themselves and their patients from potential blood-borne pathogens, but the Coronavirus pandemic has brought a new unprecedented challenge to the world of dentistry; we therefore reviewed the literature to provide suggestions on how to accordingly change dental practice prevention.

Pedraja, J., et al. (2020). "Role of 3D printing for the protection of surgical and critical care professionals in the COVID-19 pandemic." Revista Espanola De Anestesiologia Y Reanimacion **67**(8): 417-424.

Background and objective: There is a shortage of supplies for the protection of professionals during the COVID-19 pandemic. 3D printing offers the possibility to compensate for the production of some of the equipment needed. The objective is to describe the role of 3D printing in a health service during the COVID-19 pandemic, with an emphasis on the process to develop a final product ready to be implemented in the clinical environment. Methods: A working group was formed between the healthcare administration, clinicians and other public and private institutions in Cantabria, Spain coordinated by the Valdecilla Virtual Hospital. The process included receiving the printing proposals, learning about the printing resources in the region, selecting the devices, creating a team for each project, prototyping, evaluation and redesign, manufacturing, assembly and distribution. Results: The following supplies are produced: 1) devices that help protect providers: face protection screens (2,400 units), personalized accessories for photophores (20 units) and ear protection forks for face-masks (1,200 units); 2) products related to the ventilation of infected patients: connectors for non-invasive ventilation systems; and 3) oral and nasopharyngeal swabs (7,500 units) for the identification of coronavirus carriers with the aim of designing action protocols in clinical areas. Conclusions: 3D printing is a valid resource for the production of protective material for professionals whose supply is reduced during a pandemic. (C) 2020 Sociedad Espanola de Anestesiologia, Reanimacion y Terapeutica del Dolor. Published by Elsevier Espana, S.L.U. All rights reserved.

Pedreno, M. H., et al. (2021). "Social vulnerability and health risk. Analysis in agricultural contexts with cultural diversity." Acciones E Investigaciones Sociales(42): 13-42.

Although there is a significant body of research associating levels of contagion of Covid-19 with certain social conditions, these have not been widely examined in agricultural contexts. In view of this gap, the aim of this study is to take a deep dive into the social factors impacting on high incidence rates of the pandemic among farm labourers. This situation is characterised by the convergence of significant numbers of immigrant residents, as is the case of Totana (a district in the region of Murcia). Using an action research methodology, secondary sources of information (study reviews and statistical analyses of databases) are complemented with three primary sources applied to various social agents: a) a questionnaire, administered to fruit and vegetable company workers; b) an in-depth interview with representatives from vulnerable households; and c) a discussion group with professionals from various areas of intervention involved in tackling emergencies in a health and social care setting. The results allow the town's social conditions to be linked to the lowest levels of social protection against the pandemic, and hence prevention. While the population surveyed manifests a medium or high level of knowledge of the risks concerning the pandemic, in more vulnerable groups such knowledge is severely limited on account of the deficient social, work and living conditions. Professionals point out that the pandemic has highlighted pre-existing social risks linked to the significant attraction the region has for seasonal farmworkers. The study verifies the fact that the precariousness of work and living conditions of a large part of the local population has influenced their greater rates of contagion, this being most prevalent in foreign residents, who have poorer family and/or educational resources.

Pei, C. X., et al. (2020). "Alternative Face Masks Made of Common Materials for General Public: Fractional Filtration Efficiency and Breathability Perspective." Aerosol and Air Quality Research **20**(12): 2581-2591.

As COVID-19 pandemic has caused more than 24 million confirmed cases globally (as of August 28th, 2020), it is critical to slow down the spreading of SARS-CoV-2 to protect the healthcare system from overload. Wearing a respirator or a mask has been proven as an effective method to protect both the wearer and others, but commercially available respirators and masks should be reserved for healthcare workers under a currently desperate shortage. The use of alternative materials becomes an option for the general public to make the do-it-yourself (DIY) masks, with their efficacy seldom reported. In this study, we tested commercial respirators and masks, furnace filters, vacuum cleaner filters, and common household materials. We evaluated the materials' fractional filtration efficiency and breathing resistance, which are primary factors affecting respiratory protection. To compare the efficiency-resistance tradeoff, the figure of merit of each tested common material was also calculated. Filter media with electrostatic charges (electret) is recommended due to its high efficiency with low flow resistance; multiple-layer household fabrics and sterilization wraps are acceptable materials; a coffee filter is inadvisable due to its low efficiency. The outcome of this study can not only offer guidance for the general public under the current pandemic but also suggest the appropriate alternative respiratory protection materials under heavy air pollution episodes.

Pereira, L. J., et al. (2021). "Streamlining the dental care during COVID-19 pandemic: updated clinical recommendations and infection control management framework." Brazilian Oral Research **35**.

Several guidelines for dental practice have been published by dental associations and regulatory boards since the beginning of the pandemic. Initially, all non-emergency dental treatment were suspended. Healthcare personnel around the world are gradually expanding back to elective procedures. International updated recommendations alert that professionals must maintain regular observation of local health department reports, ensure personal protective equipment, and screen all patients for COVID-19 signs and symptoms. Telehealth strategies, patient screening, rescheduling when presenting COVID-19 symptoms and/or history of contact with infected people and hand/environment hygiene practices are reinforced. Appropriate cleaning and surface disinfection are mandatory. The dental staff must be trained to use appropriate Personal Protective Equipment (PPE), following a risk assessment and standard precautions: gloves; fluid resistant disposable gown, eye protection (face shield or goggles) and a medical mask. A fit tested N95 or KN95 respirator (or higher) is recommended when aerosol generating procedures are performed. Only essential accompanying persons should attend to dental appointment with the patient and must wear a cloth face covering or facemask. Social distancing and mask wearing by all staff are necessary in all areas of the office. Dental health care providers should keep up to date to the current guidance of clinical practice during the pandemic.

Perrone, G., et al. (2021). "Operating Room Setup: How to Improve Health Care Professionals Safety During Pandemic COVID-19-A Quality Improvement Study." Journal of Laparoendoscopic & Advanced Surgical Techniques **31**(1): 85-89.

Background:COVID-19 is a terrific pandemic and a potential risk for every health care professional (HCP), especially during emergency conditions where the right timing is essential for the correct treatment. During surgery the correct setting of operative room (OR) is mandatory to reduce the risk of contamination. Personal protection equipment (PPE), specific devices, and planned OR setting are essential during surgery in pandemic COVID-19. Methods:Medline, PubMed, Scientific societies recommendations, and guidelines were consulted to identify articles reporting the setup of OR during pandemic COVID-19. Results:OR must have a high-efficiency particulate air (HEPA) filter with negative pressure and a high air exchange cycle rate. Every supply kit should be packed and placed in the OR before patient arrival. A detailed checklist of equipment and devices is necessary. Personal PPE at the highest level should be provided to every HCP (Association of the Advancement of Medical Instrumentation [AAMI]-Level-III surgical gowns; double latex-free gloves with Acceptable Quality Level <1.0; FFP3 or powered air-purifying respirator masks with face shield). Anesthesia should be performed with a rapid sequence intubation. During surgery energy devices should be settled to the lower level in combination with a smoke evacuation switch pen with disposable smoke evacuation HEPA filter to minimize surgical smoke spread. During laparoscopy low pneumoperitoneum pressures and aspiration systems must be provided. Conclusions:Emergency surgery during pandemic COVID-19 increases the risk for every HCP in the OR. A theoretical risk of transmission from the surgical field exists. It is mandatory the adoption of strong strategies to reduce the risk of contamination in the OR.

Perrone, S. V., et al. (2020). "Heroes, vectors or victims Health professionals require all the necessary resources to fight the COVID-19 pandemic." Insuficiencia Cardiaca **15**(2): 52-62.

The involvement of SARS-CoV-2 infection in health personnel constitutes a serious problem for controlling the effects of the pandemic. The contagion of health personnel constitutes not only a risk for the professionals who are infected, but also for their work, family and social environment, becoming a "vector of trust" during the incubation period of the disease. The ignorance of an exact viral contagion period and the high possibility that totally asymptomatic or oligosymptomatic patients are transmitters of the virus constitutes a serious problem and is one of the factors of the fast and increasing number of infections. The advance of the pandemic, its rapid acceleration and easy spread, the access of health personnel to the adequate protection equipment for the management of patients who are carriers or at risk of being carriers of the SARS-CoV-2 virus is a growing concern. In many countries, health workers have priority in accessing the scarce personal protective equipment (PPE), but frequently they are obliged to assist patients, possibly in periods of infection without clinical manifestations. Becoming vectors of pathology is another cause of concern for health workers. The fact that these workers often have to work in various institutions further increases the risk of transmission and, due to the contagion, they are forced to complete isolation, leaving their workplaces uncovered, forced to hire new personnel, often without adequate training or unaware of the work methodology in that place with the consequent increase in errors in the proper management of patients. It is vital that the authorities do not see health workers as mere pawns to be deployed on a battlefield without the adequate weapons to combat the pandemic, but must prioritize the indispensable resources in addition to the economic one and provide, in addition to the measures of personal protection for him and his patients, but also of the resources, provision of adequate rest, support for him and his family and even psychological and legal support that allow him to carry out this battle with the least amount of victims. By the way, one can acquire the protection elements on the market perhaps from one day to the next, but they will not be able to train trained health personnel in such a short time. The following presentation tries to provide authorities and health professionals with a vision of the forms of protection that are absolutely necessary, even after the patient's death, to avoid becoming vectors of the disease and continue working as the heroes of this disease. pandemic. It is absolutely necessary to understand that protecting them protects their environment, other patients, their family and finally the community, thus avoiding the vicious circle that maintains the pandemic.

Petsonk, E. L. and P. Harber (2020). "Respiratory protection for health care workers: A 2020 COVID-19 perspective." American Journal of Industrial Medicine **63**(8): 655-658.

As the US health care system began to respond to the coronavirus disease-2019 pandemic, demand for respiratory personal protective equipment (PPE) increased precipitously, as did the number of users. This commentary discusses ensuing deviations from accepted respiratory PPE program practices, which potentially increased risk to health care workers. Such lapses included omitting user training and fit testing, provision of unapproved devices, and application of devices in settings and ways for which they were not intended. The temporary compromise of professionally accepted standards due to exigencies must not become the new normal. Rather, the current attention to PPE should be leveraged to enhance practice, motivate vital research, and strengthen professional, governmental, and institutional capabilities to control health care worker exposures to infectious hazards.

Pieter, J., et al. (2021). "Adaptation of infection protection measures in public services due to the COVID-19 pandemic Recommendations for technical, organisational and personal protective measures to reduce the spread of infection from SARS-CoV-2." Zentralblatt Fur Arbeitsmedizin Arbeitsschutz Und Ergonomie **71**(2): 56-62.

The COVID-19 pandemic is changing the requirements for occupational health and safety in the workplace. The aim of the measures implemented nationwide is to reduce the spread of the virus. This applies to all areas of life, including the workplace. The "public service" includes many system-relevant occupational groups: police, fire brigade, judiciary, municipal cleaning, waterworks and administrative bodies at local and state levels. There is a broad diversity of activities in communal and federal state enterprises, especially with regard to internal organisation as well as personnel and spatial conditions. Direct interactions with people are often necessary. Maintaining the functionality of these structures is essential for public life. Education institutions and care facilities for children, people with handicaps and vulnerable groups, institutions for nursing and geriatric care as well as public transport are not covered in this statement due to their special work structures. These recommendations address the professionally accountable in the respective public service institutions. It deals with infection control measures at work in the public service, derived from the risk assessments of various settings. The measures presented can be implemented as part of an operational concept for temporary infection control measures. The aim is to provide general guidance on infection protection with respect to work situations in order to slow down the dissemination of severe acute respiratory syndrome coronavirus 2 (SARS-CoV-2), to protect at-risk groups, and ensure the functioning of the aforementioned structures.

Pietrzak, P. and W. Hanke (2021). "COVID-19 AND DENTISTRY-SAFETY ISSUES REGARDING DOCTOR AND PATIENT SITUATION IN TIME OF VACCINE AVAILABILITY." Medycyna Pracy **72**(6): 729-737.

Since one of the main mode of the COVID-19 transmission is through close contact with the infected person (<1.5 m distance) and body fluids (saliva, blood, aerosol particles), the dentists have become one of the groups being at most risk of being infected. The aim of this article is to present, based on the research search (publications from the last year 2020 and from the beginning of the current year 2021), the general overview of the situation during the COVID-19 pandemic with regard to patients and dentist as well as supporting personal protection. Authors identified 7 questions related to this topic. They address among others such issues as identification of the COVID-19 patients coming to the dental practice; protection of both the dentist and the patient from infection and a need for guidelines on the scope of emergency dental services during the pandemic. Opinions on the subject, expressed by the experts in the field, including general guidelines were also taken into consideration. In general, based on the literature overview, the COVID-19 pandemic has completely transformed the healthcare system, including dentistry. The identification of the COVID-19 patients coming to the dental practice has become a necessity (including taking epidemiological survey, temperature measurement). Currently, since "the group zero" in significant part has completed the full vaccination process, the health personnel (including dentists) might feel safer. The efficacy of the vaccines is high and provides a certain sense of security.

Pinchi, V., et al. (2020). "Responsibility in the time of the COVID-19 pandemic." Dental Cadmos **88**(7): 448-461.

OBJECTIVES The COVID-19 pandemic had severe consequences on the health system, involving dental structures and professionals. VI the first phase of the emergency, the delivery of dental care was limited to emergencies. During the following phases. the clinical activity will be extended to the treatments suspended due to the COVID-19 emergency and to therapies that cannot be postponed without risk for oral health of patients. DISCUSSION The work of dental professionals takes place close to the patient's mouth, without protection, in a situation that involves aerosol generation. At present, there is no procedure able to eliminate the cross-infection risk, neither for patients nor for dental staff. Therefore the dentist can be claimed for negligence in the case of SARSCoV-2 infection of the patient, if this one assumes to become infected during the dental care session. In this case, the clinical recommendations and good clinical practices issued by institutions or scientific societies, according to Law 24/2017 (so-called Gelli-Bianco law), are of great importance. There is a discussion about the possibility that COVID-19 emergency could lead, at least in certain circumstances, to the mitigation of liability of professionals and healthcare structures. This could be true both in the case of claims for compensation by a patient who complain cross-infection and by the worker who takes action against the employer. There are many interpretations based on the availability of valid and evidence-based preventive measures available at the moment in which COVID-19 pandemic occurred. On the other hand. the same interpretations take into account the reason why such possible precautions have not been respected. The possible offense could only occur if valid measures were available, and the healthcare professional did not respect them without a valid reason. Talking about the responsibility towards the patient, a central role is played by the possibility by the healthcare professional to demonstrate the validity of treatment indications (in that pandemic/ epidemic phase). A careful balancing of the risks provided by the different treatment options must also be performed. The information to the patient for COVID-19 has the dual purpose: to obtain the highest compliance with the preventive measures (triage, behavior in the studio etc.) and to make him aware of the risk of developing severe forms of COVID-19. SARS-CoV-2 cross-infection during work represents an increase in the biological risk typical of dental structures. For this reason, actions are required about the Risk Assessment Document (DVR) and in terms of health surveillance of workers by the occupational physician. Within dental structures, workers exposed to specific or general aggravated risk can be identified. From this situation can emerge the work accident with consequent compensation for the SARS-CoV-2 contagion by INAIL. The civil or criminal liability of the employer is based on different assumptions and requires proof of the tort or the crime. CONCLUSIONS Dental work is carried out close to the patient mouth without airway protection and a significant amount of aerosol can be produced. The pandemic created new profiles of professional responsibility both towards the patient and towards the worker. CLINICAL SIGNIFICANCE Knowledge of the procedures for the prevention of contagion as well as of the new profiles of responsibility towards workers and patients leads the professional to a more aware and careful professional practice. Govemment and scientific societies play a central role, providing indications aimed at risk prevention. These must be systematically calibrated and reviewed as the pandemic scenario changes considering the medical/ legal value they have.

Pisu, A. (2020). "RIGHT TO HEALTH AND MEDICAL LIABILITY IN THE COVID-19 EMERGENCY." Biolaw Journal-Rivista Di Biodiritto(1): 399-414.

The CoViD-19 pandemic has promptly brought together the social and individual aspects of the right to health, highlighting some issues that are typical of the healthcare service: nosocomial contagion risk and cure decision against the backdrop of disequilibrium between health needs and available resources. The essay tackles these matters in light of the civil liability system outlined within the law n. 24/2017, putting forward some integrative proposals of the protection framework.

Polyak, G. and K. Nagy (2021). "Regulating health communication in the post-truth era." Intersections-East European Journal of Society and Politics **7**(3): 120-138.

As a global epidemic of the social media age, COVID-19 has also resulted in an 'infodemic', which means the uncontrolled spreading of false information about the health situation. Spreading of health information is a special intersection point of the freedom of speech, freedom of science, and the fundamental right to life and health. The paper analyses the European and Hungarian legal framework for health communication from multiple perspectives. Regulatory challenges and solutions differ for professional health communication, commercial communication and health communication by laypeople. As with all forms of misinformation, private regulations of platform operators have a significant regulatory role to play in relation to health disinformation. As a result of the analysis, the paper provides a detailed regulatory map that also covers private regulation solutions and explores the factors that need to be considered when designing a comprehensive future regulation.

Poonuraparampil, J. A., et al. (2021). "Revisiting electronic systems and gadgets for patient management in COVID-19 Intensive Care Units." Anaesthesia Pain & Intensive Care **25**(1): 5-7.

Monitoring and highly sophisticated care of the Covid-19 pandemic patients has taught us many new lessons. The ever-changing management strategies and the therapies for the disease containment is one aspect of the wide spectrum of innovations and refinements, which were the result of global effort by the scientists and the healthcare workers; the self-protection of front-line workers soon became immensely important to save them from contracting the disease and thus prevent rapid reduction in their numbers. Personal protective measures were vehemently enforced and means and measures to reduce (if not eliminate) direct contact with the known as well as yet unknown Covid positive patients were advocated. Different countries adopted different measures according to their customs and resources, but all with the same purpose. This paper is written to highlight the same and present our approach at our center.

Provenzano, B. C., et al. (2021). "The impact of healthcare-associated infections on COVID-19 mortality: a cohort study from a Brazilian public hospital." Revista Da Associacao Medica Brasileira **67**(7): 997-1002.

OBJECTIVE: This study aims to analyze the risk factors for in-hospital mortality in a cohort of patients admitted to a newly adapted intensive care unit in a public hospital in Rio de Janeiro. METHODS: This was an observational, retrospective, and descriptive study. Data were obtained from electronic medical records. Coronavirus disease 2019 (COVID-19) was diagnosed by detecting viral ribonucleic acid using reverse transcription polymerase chain reaction. Factors associated with the risk/protection from death were determined using the odds ratio and adjusted odds ratio. RESULTS: Fifty-one patients were admitted to the hospital. The median age of the patients was 63 years, 60% were male patients, and 54% were white patients. Sixty-seven percent of the patients were diagnosed with COVID-19. Sepsis at admission increased the chance of in-hospital death by 21 times (adjusted odds ratio=21.06 [0.79-555.2]; p=0.06). The strongest risk factor for death was the development of septic shock during hospitalization (adjusted odds ratio=98.56 [2.75-352.5]; p=0.01), and one in four patients had multidrug-resistant bacteria. Mechanical ventilation, vasopressors, neuromuscular blockers, and sedatives were also the risk factors for in-hospital mortality. The in-hospital mortality rate was 41%, and the mortality rate of patients on mechanical ventilation was 60%. The diagnosis of COVID-19 was not statistically related to the adverse outcomes. CONCLUSIONS: In this cohort, the strongest risk factor for in-hospital death was the development of nosocomial septic shock. Healthcare-associated infections have a significant impact on mortality rates. Therefore, to have a better outcome, it is important to consider not only the availability of beds but also the way healthcare is delivered.

Quadros, C. A., et al. (2020). "Preclinical validation of occupational and environmental safety of an isolation system for noninvasive ventilation in COVID-19 and other aerosol-transmitted infections." Expert Review of Medical Devices **17**(11): 1211-1220.

Background: The current SARS-CoV-2 pandemic has provoked the collapse of some health systems due to insufficient intensive care unit capacity. The use of continuous positive airway pressure (CPAP) and high-flow nasal oxygen (HFNO) therapies has been limited in consideration of the risk of occupational infection in health-care professionals. Aims: In preclinical experimental simulations, evaluate occupational and environmental safety of the newly developed isolation system for aerosol-transmitted infections (ISATI). Method: Simulations were conducted to test ISATI's capability to isolate aerosolized molecular (caffeine), and biological (SARS-CoV-2 synthetic RNA) markers. Caffeine deposition was analyzed on nitrocellulose sensor discs by proton nuclear magnetic resonance spectroscopy. Synthetic SARS-CoV-2 detection was performed by reverse transcription-polymerase chain reaction. Results: ISATI demonstrated efficacy in isolating molecular and biological markers within the enclosed environment in simulated conditions of CPAP, HFNO and mechanical ventilation therapy. Neither the molecular marker nor substantial amounts of synthetic SARS-CoV-2 RNA were detected in the surrounding environment, outside ISATI, indicating appropriate occupational safety for health-care professionals. Conclusion: Aerosolized markers were successfully contained within ISATI in all experimental simulations, offering occupational and environmental protection against the dissemination of aerosolized microparticles under CPAP or HFNO therapy conditions, which are indicated for patients with acute respiratory infections.

Radulesco, T., et al. (2021). "Sinus and anterior skull base surgery during the COVID-19 pandemic: systematic review, synthesis and YO-IFOS position." European Archives of Oto-Rhino-Laryngology **278**(6): 1733-1742.

Purpose The COVID-19 pandemic has caused significant confusion about healthcare providers' and patients' pandemic-specific risks related to surgery. The aim of this systematic review is to summarize recommendations for sinus and anterior skull base surgery during the COVID-19 pandemic. Methods PubMed/MEDLINE, Google Scholar, Scopus and Embase were searched by two independent otolaryngologists from the Young Otolaryngologists of IFOS (YO-IFOS) for studies dealing with sinus and skull base surgery during COVID-19 pandemic. The review also included unpublished guidelines edited by Otolaryngology-Head and Neck Surgery or Neurosurgery societies. Perioperative factors were investigated including surgical indications, preoperative testing of patients, practical management in operating rooms, technical aspects of surgery and postoperative management. The literature review was performed according to PRISMA guidelines. The criteria for considering studies or guidelines for the review were based on the population, intervention, comparison, outcome, timing and setting (PICOTS) framework. Results 15 International publications met inclusion criteria. Five references were guidelines from national societies. All guidelines recommended postponing elective surgeries. An algorithm is proposed that classifies endonasal surgical procedures into three groups based on the risk of postponing surgery. Patients' COVID-19 status should be preoperatively assessed. Highest level of personal protective equipment (PPE) is recommended, and the use of high-speed powered devices should be avoided. Face-to-face postoperative visits must be limited. Conclusions Sinus and skull base surgeries are high-risk procedures due to potential aerosolization of SARS-CoV-2 virus. Protection of health care workers by decreasing exposure and optimizing the use of PPE is essential with sinus and anterior skull base surgery.

Rahi, S., et al. (2021). "Factors influencing the adoption of telemedicine health services during COVID-19 pandemic crisis: an integrative research model." Enterprise Information Systems **15**(6): 769-793.

This study aims to investigate patient's behaviour towards the adoption of telemedicine health services with the integration of three well-Known theories, namely, unified theory of acceptance and use of technology (UTAUT), the protection motivation theory (PMT) and DeLone & McLean information success model. The results of structural equation modeling indicate that altogether UTAUT, PMT and DeLone & McLean information success model explained 80.4% variance in patient attitude towards adoption of telemedicine health services. Findings suggest that health-care professional can boost patient attitude towards adoption of telemedicine health services by improving factors such as service quality, computer self-efficacy and performance expectancy.

Ramadan, M., et al. (2021). "Beyond knowledge: Evaluating the practices and precautionary measures towards COVID-19 amongst medical doctors in Jordan." International Journal of Clinical Practice **75**(6).

Background Health Care Workers (HCWs), including medical doctors, played a pivotal role as a first-line defence against the COVID-19 pandemic. Because of high exposure, HCWs are at an increased risk of contracting the disease. Aims This study aims to assess the level of precautionary measures, both at home and the workplace, amongst medical doctors who were on duty during the national lockdown in Jordan. Methods A cross-sectional study was conducted between March 23 and May 1, 2020, utilising a self-administered web-based questionnaire to examine a sample of medical doctors (n = 270) working at different healthcare institutions in Jordan. Likert scale was used to code the data and generate means and percentages. Results The most practiced on-duty precautionary measures were cleaning hands with water and disinfectant for more than 20 seconds (47.4%), followed by proper hygiene before and during meals (38.9%). The most practiced off-duty measures were taking off clothes before entering the residential place (65.9%) and prohibiting visitors (58.1%). Overall, the mean work protection percentage score was 73.8% (range: 28%-100%), while the mean home safety percentage score was 71.3% (range: 25%-100%). Work protection score was positively correlated with the home safety score. Female doctors were found to be more precautious at home than males. Doctors with chronic illness(es) were found to be less precautious than their healthier counterparts. Participants who isolated themselves expressed the highest level of home safety practice. Doctors who reported to smoke were found more precautious at home and doctors who preferred to work during lockdowns were more precautious at the workplace. Conclusion The level of precautionary behaviour of medical doctors in Jordan was not optimal. More attention and efforts are needed to enhance the adherence of doctors to precautionary guidance. Strengthening the role of infectious disease and infection control units within healthcare settings remains a necessity.

Ramanathan, M., et al. (2022). "COVID-19 and Cleft and Craniofacial Surgery in Indian Scenario." Journal of Maxillofacial & Oral Surgery **21**(2): 460-465.

The coronavirus disease (COVID-19) had created the new normal approach towards the management of all maxillofacial problems as it is highly contagious and causing a threat to the health care professionals. The surgical management of patients with cleft and craniofacial deformities has caused lots of anxiety among patients and doctors in the recent COVID era as some essential treatment will be required for cleft babies from day one. Safety and protection for cleft children and parents must be the priority while dealing with this non-emergency disease. This article will highlight the important steps of management of the cleft and craniofacial cases during this pandemic by adhering to the protocols. It also throws light towards the strategies in revoking the cleft surgical management at least till this infection subsides.

Rasmussen, A. and K. Dambrino (2021). "Is There an Ethical Mandate to Practice Without Proper Personal Protective Equipment?" Journal of Hospice & Palliative Nursing **23**(2): 114-119.

In the spring of 2020, a novel virus known as COVID-19 (coronavirus disease 2019) was introduced to the human population, and the world faced a global pandemic with far-reaching consequences. One of the most difficult challenges that nurses faced in the midst of the crisis was the lack of proper personal protective equipment (PPE). The lack of PPE left health care professionals with a complicated ethical dilemma: Is there an ethical duty to care for patients in the absence of proper PPE? This article seeks to help the individual nurse (a) understand the ethical dilemma and the tensions that it brings, (b) look to the literature for guidance, and (c) understand how individuals can apply these ethical principles. After careful analysis, the recommendation is for the individual nurse to make a thorough assessment of their personal situation. This will include consideration for their family, community, financial responsibilities, legal protections, organizational policies, and personal health risk. Finally, this article serves as a call to organizations and professional leaders to increase their support of frontline health care workers and provide the individual nurse with the information they need in order to make sound decisions.

Ren, Y. F., et al. (2020). "Dental Care and Oral Health under the Clouds of COVID-19." Jdr Clinical & Translational Research **5**(3): 202-210.

Coronavirus disease 2019 (COVID-19), caused by the severe acute respiratory syndrome coronavirus 2 (SARS-CoV-2), has caused much anxiety and confusion in the community and affected the delivery of vital health care services, including dental care. We reviewed current evidence related to the impact of SARS-CoV-2/COVID-19 on dental care and oral health with the aim to help dental professionals better understand the risks of disease transmission in dental settings, strengthen protection against nosocomial infections, and identify areas of COVID-19-related oral health research. When compared with other recent pandemics, COVID-19 is less severe but spreads more easily, causing a significantly higher number of deaths worldwide. Protection of dental patients and staff during COVID-19 is challenging due to the existence of patients who are infectious yet asymptomatic. Dental professionals are ill prepared for the pandemic, as they are not routinely fitted for the N95 respirators now required for preventing contagion during dental treatments. Biological and clinical evidence supports that oral mucosa is an initial site of entry for SARS-CoV-2 and that oral symptoms, including loss of taste/smell and dry mouth, might be early symptoms of COVID-19, presenting before fever, dry cough, fatigue, shortness breath, and other typical symptoms. Oral health researchers may play a more active role in early identification and diagnosis of the disease through deciphering the mechanisms of dry mouth and loss of taste in patients with COVID-19. Rapid testing for infectious diseases in dental offices via saliva samples may be valuable in the early identification of infected patients and in disease progress assessment. Knowledge Transfer Statement: This commentary provides a timely evidence-based overview on the impact of COVID-19 on dental care and oral health and identifies gaps in protection of patients and staff in dental settings. Oral symptoms are prominent before fever and cough occur. Dental professionals may play an important role in early identification and diagnosis of patients with COVID-19.

Resnicow, K., et al. (2021). "Novel Predictors of COVID-19 Protective Behaviors Among US Adults: Cross-sectional Survey." Journal of Medical Internet Research **23**(4).

Background: A central component of the public health strategy to control the COVID-19 pandemic involves encouraging mask wearing and social distancing to protect individuals from acquiring and transmitting the virus. Objective: This study aims to understand the psychological factors that drive adoption or rejection of these protective behaviors, which can inform public health interventions to control the pandemic. Methods: We conducted an online survey of a representative sample of 1074 US adults and assessed three novel potential predictors of COVID-19 behaviors: trait reactance, COVID-19 conspiracy beliefs, and COVID-19 apocalypse beliefs. Key outcomes (dependent variables) included an index of COVID-19 protective behaviors, the number of trips taken from the home, and COVID-19 knowledge. Results: In bivariate analyses, all three predictors were significantly correlated in the hypothesized direction with the three COVID-19 outcomes. Specifically, each predictor was negatively (P<.01) correlated with the COVID-19 protective behaviors index and COVID-19 knowledge score, and positively correlated with trips taken from home per week (more of which was considered higher risk). COVID-19 protective behaviors and COVID-19 knowledge were significantly lower in the top median compared to the bottom median for all three predictors. In general, these findings remained significant after adjusting for all novel predictors plus age, gender, income, education, race, political party, and religiosity. Self-identified Republicans (vs other political affiliations) reported the highest values for each of the novel predictors. Conclusions: This study can inform the development of health communication interventions to encourage the adoption of COVID-19 protective behaviors. Interestingly, we found that higher scores of all three novel predictors were associated with lower COVID-19 knowledge, suggesting that lack of an accurate understanding of the virus may be driving some of these attitudes; although, it is also possible that these attributes may interfere with one's willingness or ability to seek and absorb accurate health information. These individuals may be particularly immune to accepting new information and yielding their beliefs. Health communication professionals may apply lessons learned from countering similar beliefs around climate change and vaccine hesitancy. Messages designed for individuals prone to reactance may be more effective if they minimize controlling language and emphasize the individual's independence in adopting these behavioral recommendations. Messaging for those who possess conspiracy beliefs should similarly not assume that providing evidence contrary to these beliefs will alone alter behavior. Other communication techniques such as rolling with resistance, a strategy used in motivational interviewing, may be helpful. Messaging for those with apocalyptic beliefs may require using religious leaders as the message source and using scripture that would support the adoption of COVID-19 protection behaviors.

Reynolds, C. W., et al. (2021). "Challenges and Effects of the COVID-19 Pandemic on Asylum Seeker Health at the US-Mexico Border." Health Equity **5**(1): 169-180.

Purpose: The coronavirus disease 2019 (COVID-19) pandemic presents health care challenges to asylum seekers living in congregate encampments, including those along the U.S.-Mexico border. It is necessary to understand the impact of the pandemic among this population to address health care needs, reduce transmission, and diminish COVID-19-related morbidity. Methods: Thirty interviews were conducted with asylum seekers and health care professionals in a temporary camp in Matamoros, Mexico to determine challenges, perceptions, and effects of the COVID-19 pandemic. Interviews were coded in NVivo12 by using a team-based approach. Results: The pandemic caused significant mental health burdens but no perceived adverse physical effects, with the U.S. border closure and health care access barriers as more pressing concerns. Participants reported access to information about COVID-19 but had varied levels of knowledge and adherence to disease reduction strategies due to camp conditions. Most participants believed that they had special protection from COVID-19, including strong immune systems or from God. The nongovernmental organizations providing health care and sanitation faced multiple challenges to implement new policies to manage COVID-19. The institution of required temperature checks and quarantine of COVID-19 positive patients led to distrust, decreased seeking of health care services among asylum seekers, and possible underreporting of COVID-19 cases. Conclusion: Our findings among asylum seekers in a Matamoros camp highlight the challenges to implementing disease reduction policies in low-resource congregate camps. Policies to address disease outbreaks focusing on the social determinants of health, health care access barriers, and community engagement may be more acceptable to asylum seekers, suggesting the need for effective strategies to provide prevention information that complements such measures.

Richterman, A., et al. (2020). "Hospital-Acquired SARS-CoV-2 Infection Lessons for Public Health." Jama-Journal of the American Medical Association **324**(21): 2155-2156.

This Viewpoint reviews the rare incidence of nosocomial SARS-CoV-2 infection and discusses ways it can be minimized, including use of surgical masks, proper ventilation, physical distancing, eye protection, regular testing, and the availability of sick leave for health care workers.

Rim, K. T. (2021). "COVID-19 pandemic and the protection of workers' health from disinfectant chemicals." Toxicology and Environmental Health Sciences **13**(1): 1-7.

Objective and methods This study examines the protection of workers from the "COVID-19" pandemic, especially healthcare workers, disinfection workers, and future prospects in occupational health. To summarize this concept, I searched the major websites using the key terms "COVID-19," "coronavirus," "pandemic," "workers' health," and "quarantine" or "disinfection." Results and conclusion The use of disinfectants is recognized to be effective in preventing viruses, but this is also increasing the need to solve problems, such as the side effects caused by the increase in disinfectant use. This paper presents the precautions for safer use of chemicals handled by workers during quarantine and disinfection and proposes policy suggestions. The COVID-19 pandemic will have long-lasting effects on workers. Syndromic methods for monitoring illness outside of healthcare settings can be useful adjuncts to conventional disease reporting. From these results, it is important that the precautions for chemicals' safety handled by workers during quarantine and disinfection as well as policy suggestions for quarantine and disinfection workers are very important to protect workers.

Riutord-Sbert, P., et al. (2020). "An innovative, reusable and sustainable face-seal device to improve protection efficacy of surgical masks against COVID-19." Medicina Balear **35**(4): 74-77.

The outbreak of the Severe Acute Respiratory Syndrome Coronavirus 2 (SARS-CoV-2) pandemic has had a direct impact on the global health system, causing an alarming shortage of Personal Protective Equipment (PPE). Recent studies have shown that a significant number of healthcare professionals have been contaminated by the COVID-19 at their workplace due to the lack of appropriate PPE. Consequently, the PEE requirements have changed, making the use of filtering face-piece respirators (FFR) N95 and NK95 (FFP2 or FFP3, respectively) mandatory in place of the surgical masks previously used by healthcare professionals. Applying individualized face-seal devices in surgical masks, such as a thermoplastic resin ring, may significantly avoid inhalation of unfiltered air. Besides reducing leakage around the mask, which could convert surgical masks into PPE dual masks due to the high percentage of face-seal, it would allow a bidirectional protection for both healthcare professionals and patients, thus becoming a medical device. The polylactic acid (corn starch) thermoplastic resin ring is the device proposed here to be used in order to decrease leakage of potentially contaminated air. The use of poly lactic acid is of particular interest due to the fact that is a material appropriate for sanitary use, reusable and biodegradable. Therefore, healthcare professionals and organizations can maintain clinical activity in a cost-efficient manner whilst improving clinical safety.

Rivas, M. N., et al. (2021). "Rationale for Randomized Clinical Trials Investigating the Potential of BCG Vaccination in Preventing COVID-19 Infection." Bladder Cancer **7**(2): 121-131.

Despite the implementation of mitigation measures, Coronavirus disease 2019 (COVID-19), caused by severe acute respiratory syndrome coronavirus 2 (SARS-CoV-2), is still spreading worldwide, and has caused more than 1 million deaths so far. Although recent reports indicate that three vaccine candidates are effective against SARS-CoV-2, more time is needed to generate enough doses for the general population. Meanwhile, frontline healthcare workers are at high risk of SARS-CoV-2 exposure. To avoid collapse of the medical care system, there is a need to develop novel approaches to limit SARS-CoV-2 spread. Through a process called trained immunity, the Bacillus Calmette-Guerin (BCG) vaccine boosts the action of innate immune cells, resulting in a nonspecific reduction in the incidence of viral infections. Due to this immunomodulatory action, the BCG vaccine is currently used as a therapeutic in bladder cancer. Data collected from epidemiological and observational studies indicate that BCG vaccination might provide protection against COVID-19. While these observations do not provide evidence of causality and are limited by cofounding and intrinsic biases, it is crucial to explore the hypothesis that BCG vaccination may provide a nonspecific innate immune boost and therefore protect against COVID-19 in randomized controlled clinical trials, particularly for people at higher risk of developing COVID-19, such as frontline healthcare workers.

Robles-Perez, E., et al. (2021). "Infection and death by COVID-19 in a cohort of healthcare workers in Mexico." Scandinavian Journal of Work Environment & Health **47**(5): 349-355.

Objective This study aimed to estimate the risk of SiRS-Cov2 infection and severe COVID-19 among healthcare workers from a major social security system. Methods This study actively followed a cohort of social security workers from March to December 2020 to determine the number of laboratory-confirmed symptomatic cases, asymptomatic associated contacts and COVID-19-associated hospitalizations and deaths. Workers were classified into those providing direct care to infected patients (COVID teams), other active healthcare workers (OAHCW), and workers under home protection (HPW). The number of cases and rates were also estimated by job category. Results Among a total of 542 381 workers, 41 461 were granted stay-at-home protection due to advanced age or comorbidities. Among the 500 920 total active workers, 85 477 and 283 884 were classified into COVID teams and OAHCW, respectively. Infection rates for COVID teams, OAHCW, and HPW were 20.1% [95% confidence interval (CI) 19.8-20.4], 13.7% (95% CI 13.5-13.8), and 12.2% (95% CI 11.8-12.5), respectively. The risk of hospitalization was higher among HPW. COVID teams had lower mortality rate per 10 000 workers compared to HPW (5.0, 95% CI 4.0-7.0 versus 18.1, 95% CI 14.0-23.0). Compared to administrative workers, ambulance personnel (RR 1.20; 954/0 CI 1.09-1.32), social workers (RR 1.16; 95% CI 1.08-1.24), patient transporters (RR 1.15; 95% CI 1.09-1.22) and nurses (RR 1.13; 95% CI 1.10-1.15) had a higher risk of infection after adjusting for age and gender. Crude differences in mortality rates were observed according to job category, which could be explained by differences in age, sex, and comorbidity distribution. Diabetes, obesity, hypertension, hemolytic anemia, and HIV were associated with increased fatality rates. Conclusions COVID team workers had higher infection rates compared to the total population of active workers and HPW. Doctors had lower risk of infection than respiratory therapists, nurses, and patient transporters, among whom interventions should be reconsidered to reduce risks. The presence of comorbidities, such as diabetes, obesity, arterial hypertension, hemolytic anemia, and HIV, increased the likelihood of complications caused by COVID-19, culminating in a poor prognosis.

Rodrigues, K. F., et al. (2020). "Disaster preparedness and response in Brazil in the face of the COVID-19 pandemic." Revista De Administracao Publica **54**(4): 614-634.

This article aims to understand how the National System of Protection and Civil Defense functions in response to COVID-19, with emphasis on the work of the Ministry of Health, which is the body responsible for tackling health threats. Three specific objectives were used: the first characterizes COVID-19 as a public health event that can represent a disaster; the second situates the concepts of preparedness and response in the disaster governance literature; the third identifies the jurisprudence and the functioning of disaster management in Brazil. The findings show that, despite the political decision-making tensions, the Brazilian professional bureaucracy managed to guarantee the activation of the disaster governance system related to the preparation and response phases. However, its activation was not enough to allay the crisis. The severity of the pandemic exposed flaws in the phases of disaster prevention and mitigation, as well as the lack of coordinated government response.

Rotila, V. (2021). "Physicians Professional Immunity in the COVID-19 Pandemic. Problems and Solutions." Postmodern Openings **12**(1): 356-392.

In this article, by professional immunity we refer to limitation of liability to the specific pandemic context, respectively to the concrete possibilities of diagnosis, treatment, care and too few degrees of professional freedom still available. Our thesis is that, regardless of the form of transposition into practice, a form of health professionals protection, such as professional immunity is necessary. The need for specific measures for professional immunity is not identical in each state, depending on the specifics of the legal system, social values, measures characteristics taken to combat the pandemic, the degree of transparency of each healthcare system. However, this need doesn't start from "no measure", the appropriate minimum level of protection seemingly to constitute a patient compensation no-fault model, plus the characteristics of the professional immunity, designed to adapt liability to specific conditions. In the absence of professional immunity there is a risk of transferring the cost of ignorance, visible, in the inadequate expectations of medical science, on healthcare professionals, the limits of scientific knowledge risking to be reinterpreted as medical fault. The COVID-19 pandemic has somehow forced an additional step towards broadening discussions on universal human rights, being forced to consider ways of putting them into practice in the context of limited resources. Assuming that the increase in social complexity increases the risks of such events, we approach these problems from the perspective of creating rapid mechanisms to adapt to such changes, the lessons learned now being applied in the future to other similar cases.

Safradin, B., et al. (2021). "Fundamental Social Rights Protection and Covid-19 in the EU: Constraints & Possibilities." Utrecht Law Review **17**(3): 103-117.

The Covid-19 pandemic has had major socio-economic consequences, particularly for critical workers such as healthcare workers, seasonal workers and platform workers in their social rights enjoyment. This article analyses how EU law could protect social rights in times of Covid-19, with a specific focus on the possibilities and limits of the EU Charter in times of crisis for these certain categories of EU workers. The potential of EU legal instruments to protect vulnerable workers' social rights is limited both by the limited legislative competences in the social policy field and the limited scope of application of the EU Charter. Furthermore, social rights enshrined in the Charter are often formulated as principles, which means they cannot be invoked directly in court, but need to be elaborated in legislation. Nevertheless, the EU could further strengthen the potential of social rights in the EU legal order through harmonisation of social standards in two ways. First, by harmonisation of social rights using the legal bases in the Treaty. Secondly, by implementing the European Pillar of Social Rights effectively and by improving application of the EU Charter at national level, both by clarifying and broadening horizontal direct effect as by increasing application of the EU Charter by national policymakers and the judiciary and raising awareness.

Sahashi, Y., et al. (2021). "Worries and concerns among healthcare workers during the coronavirus 2019 pandemic: A web-based cross-sectional survey." Humanities & Social Sciences Communications **8**(1).

Healthcare workers (HCWs) treating and caring for patients with emerging infectious diseases often experience psychological distress. However, the psychological impact and behavior change of the coronavirus disease 2019 (COVID-19) pandemic among HCWs are still unknown. This study aimed to investigate the worries and concerns of HCWs regarding the COVID-19 pandemic. In this cross-sectional survey, a web-based questionnaire was distributed among HCWs working in hospitals or clinics across Japanese medical facilities from April 20 to May 1, 2020. The questionnaire comprised items on demographics, worries and concerns, perceptions regarding the sufficiency of information, and behavioral changes pertaining to the COVID-19 pandemic. A total of 4386 HCWs completed the survey; 1648 (64.7%) were aged 30-39 years, 2379 (54.2%) were male, and 782 (18.1%) were frontline HCWs, directly caring for patients with COVID-19 on a daily basis. 3500 HCWs (79.8%) indicated that they were seriously worried about the pandemic. The most frequent concern was the consequence of becoming infected on their family, work, and society (87.4%). Additionally, the majority (55.5%) had restricted social contact and almost all HCWs endorsed a shortage in personal protective equipment (median, 8/9 (interquartile range; 7-9) on a Likert scale). There was no significant difference in the degree of worry between frontline and non-frontline HCWs (8/9 (7-9) vs. 8/9 (7-9), p = 0.25). Frontline HCWs, compared to non-frontline HCWs, were more likely to have the need to avoid contact with families and friends (24.8% vs. 17.8%, p < 0.001) and indicated that they cannot evade their professional duty during the COVID-19 pandemic (9/9 (7-9) vs. 8/9 (6-9), p < 0.001). Further, the extremely low proportion of frontline HCWs reported that they would take a leave of absence to avoid infection (1.2%). In conclusions, both frontline and non-frontline HCWs expressed comparable concerns regarding the COVID-19 pandemic. Because HCWs, especially frontline HCWs, reported that they cannot be obliged to do avoid their duty, effective mental health protection strategies should be developed and implemented for HCWs.

Saifuddin, A., et al. (2021). "COVID Arm After Moderna Booster in Healthcare Worker: A Case Report." Acta Medica Indonesiana **53**(3): 326-330.

SARS CoV-2 virus has infected more than 200 million people worldwide and more than 4.4 million in Indonesia. The vaccination program has become one of the solutions launched by many countries globally, including Indonesia, to reduce the transmission rate of COVID-19. Various vaccination platforms are produced, such as inactivated, viral vector, mRNA, and protein subunit. The vaccination booster program with mRNA platform (Moderna) was launched by the Indonesian government to give better protection for health care workers, particularly from delta variant. In this case report, we discuss one of the typical side effects of Moderna vaccine, which is referred to as the COVID arm.

Sakpal, O. S., et al. (2020). "A Study to Evaluate the Knowledge and Awareness amongst Dental Professionals about COVID-19 Outbreak - A Survey." Journal of Evolution of Medical and Dental Sciences-Jemds **9**(43): 3186-3191.

BACKGROUND Across the globe, there have been reports which have shown that all the dental professionals and the field of dentistry to be more vulnerable to the coronavirus infection. Dentists in general are comparatively more prone to infections as they are more exposed to aerosols and droplets emerging out of the patient's oral cavity. This scare of coronavirus has unmasked the crevices in the integration of the dental health care professionals in to the public health care sector of various countries. It has significantly sidelined the dental treatment approach towards the patients and for the dental team as well. At this point, it is very difficult to project and calculate the exact number of cases of Covid-19 carriers or infected who are being treated by dentists. Such a preliminary survey will give us an idea about the precautionary measures that can be taken or being taken in dental practice. The aim of the study was to assess the knowledge, attitude and practice amongst dental professionals of different specialties about the Covid-19 and precautionary measures at different levels. METHODS An online questionnaire based survey was carried out to assess the knowledge, attitude and practice amongst the dental professionals of different specialties about recent Covid 19 pandemic all over the world. A total of 250 participants were sent a survey questionnaire via Google Survey form. The responses were accepted in a 2 week time frame. The survey majorly focused on the clinical practice awareness and precautions taken by the dental professionals at their university / institute / college / private clinics while treating their patients based on which their knowledge and awareness were assessed. Demographic characteristics of study participants and their knowledge, awareness and practice related to Covid-19 were presented using descriptive statistics (mean and frequency distribution). RESULTS Two hundred and forty-four (97.6 %) out of 250 participants surveyed responded to the questionnaire. Overall awareness amongst dental professionals was seen to be adequate with maximum correct responses received from the Teaching faculty of different institutes. Regarding the precaution level taken at the respective Institute / College / University the average score calculated on the base of responses was 6.92. CONCLUSIONS The survey concluded that there is a dire need for regular educational interventions regarding the spread of the diseases and infection control at definite intervals. All dental professionals must be well aware of the protection kits available and their appropriate use during patient diagnosis and treatment planning.

Saleem, M., et al. (2020). "Operating Facilities:- Suggested Guidelines to Cope COVID-19 in Third World Countries." Annals of King Edward Medical University Lahore Pakistan **26**: 115-119.

The whole world is passing through an unprecedented time during the pandemic of COVID-19. COVID-19 has affected all of medical practices in relation to patient management, availability of resources, logistics, safety and protection of healthcare workers and off course emergency management of healthcare facilities. Although surgeons are not frontline workers but provision of emergency and urgent surgeries without knowing status of patient in relation to COVID-19 put the surgical team at risk. Moreover our operative healthcare settings are not designed to deal with paradigm of COVID 19. We are living in a 3rd world country. Our resources are scarce and our theater settings are not up to mark. This article deals with suggested changes in already existing theater facilities or creating new facilities to come up with new requirement of COVID-19.

San-Juan, D., et al. (2020). "Guidance for clinical neurophysiology examination throughout the COVID-19 pandemic. Latin American chapter of the IFCN task force - COVID-19." Clinical Neurophysiology **131**(7): 1589-1598.

On 31st December 2019, China notified the World Health Organization of an outbreak of atypical pneumonia from patients at a local seafood market in Wuhan, Hubei, China, responsible for a new coronavirus called Severe Acute Respiratory Syndrome Coronavirus 2 (SARS-CoV-2) that caused COVID-19 disease, which spread rapidly around the world. WHO declared a state of pandemic (11th March, 2020), which has caused more than 1 million infected and more than 110,000 deaths; it was observed that up to 29% of those infected were health care personnel. The main route of transmission of SARS-CoV2 is through respiratory secretions and direct contact with contaminated surfaces and material. The pandemic induced an international saturation of health care services and a rupture in the supply chain of protective equipment for healthcare personnel, which poses a high occupational risk to all. Based on the different healthcare systems, human resources, infrastructure and medical emergencies that will warrant the conduct of clinical neurophysiology studies and the lack of a guide for the management of the situation, it was decided by an expert task force of the Latin American Chapter of the International Federation of Clinical Neurophysiology to carry out these guidelines for the protection of patient and healthcare professionals conducting clinical neurophysiological studies. (C) 2020 International Federation of Clinical Neurophysiology. Published by Elsevier B.V. All rights reserved.

Sansone, E., et al. (2021). "Effectiveness of BNT162b2 vaccine against SARS-CoV-2 among healthcare workers." Medicina Del Lavoro **112**(3): 250-255.

Background: The present study was aimed at evaluating the effectiveness of BNT162b2 among HCWs of a university hospital while a recrudescence of pandemics was hitting the province, with a high rate of the B.1.1.7 variant. Methods: The study was performed in the context of health surveillance at the workplaces. We monitored the SARS-CoV-2 infection and COVID-19 symptoms among HCWs classified by having received the entire vaccine schedule or not; the latter further classified in not vaccinated workers and workers who had received the first shot more than 14 days earlier. The SARS-CoV-2 infection was diagnosed by conventional RT-PCR on rhino-pharyngeal swabs, followed by gene sequencing in positive vaccinated HCWs. The cumulative incidence of infections in the period was normalised to 100,000 people. Results: At the end of the observation period, HCWs that had completed the full schedule were at lower infection risk than both unvaccinated HCWs and the workforce who had not yet gained the complete theoretical protection from the vaccine (by 2.4-folds). Overall, ninety-two SARS-CoV-2 infections were observed among HCWs, mostly among not protected workers (52 cases) but none of them showed symptoms requiring hospitalisation. Conclusions: The vaccination campaign effectively reduced the appearance of symptoms and the incidence of infections among vaccinated HCWs. Among vaccinated HCWs, gene sequencing was possible in five cases only, 4 B.1.1.7 and 1 B1.525 variants. The high rate of unsuccessful gene sequencing observed among infected vaccinated workers could be explained by a low viral burden. Vaccination for COVID-19 should be mandatory in occupational settings with a high infective risk.

Sava, A., et al. (2020). "Protection measures against SARS-CoV-2 infection for cytopathology and histopathology laboratories personnel: practical recommendations." Romanian Journal of Morphology and Embryology **61**(4): 1067-1076.

In the severe acute respiratory syndrome coronavirus 2 (SARS-CoV-2) pandemic, healthcare workers are at high risk to be infected with this new coronavirus, particularly when they handle not only patients, but also their body fluids. In Romania, even though the protective measures to be used by medical staff in emergency departments, clinical departments, radiology departments, clinical laboratories and morgues services are well known, there is little information about the protection of medical staff in the laboratories of cytopathology and histopathology. In this article, we will discuss the transmission routes of the new coronavirus, the surfaces it could contaminate in a hospital, as well as the modalities of its inactivation. We will present some guidelines for preparing the pathology departments to face the pandemic situation like the present one. Also, we will point out some possible recommendations/suggestions for protective measures to be taken by laboratory staff during the cytological and histopathological procedures when they manipulate body fluids or surgical samples of patients with suspected or confirmed coronavirus disease 2019 (COVID-19). Laboratory personnel should be aware that any body fluid or surgical specimen that arrives in the laboratory may contain SARS-CoV-2 and, as such, they should act after new working procedures. We recommend restraint from performing extemporaneous examination (smear and frozen section) and cytopathological examination in laboratories that do not have adequate condition for handling and processing Hazard Group 3 (HG3) pathogens, as SARS-CoV-2. Also, laboratory personnel should pay attention to instruments, technical equipment, or environmental surfaces as these also can be contaminated with the new coronavirus.

Sbert, P. R., et al. (2020). "Survey on the situation of professionals of oral health in the Balearic Islands against COVID-19 in time of confinement population and start of de-escalation." Medicina Balear **35**(4): 49-61.

Introduction: The ADEMA-UIB University School of Dentistry, faced with the situation caused by COVID-19, has carried out a survey for dentists and stomatologists, superior technicians in oral hygiene and superior technicians in dental prosthesis of the Balearic Islands, in order to know various important aspects of their work and personal situation in relation to COVID-19, as well as the measures adopted and the functionality of the Dental Clinics and the Dental Prosthesis Laboratories, during the confinement of the population and the start of de-escalation. The aim of the study is to know the changes produced in the oral care of the population during this stage of the pandemic. Material and method: We carried out an online self-filling survey, with free access to the 1,482 professionals involved in oral care of the population of the Balearic Islands. The data has been collected between days 1 and 30 of June 2020, through a form served through the Google Forms Gsuit application. This study is quantitative, descriptive and anonymous. Results: The responses received by the 148 professionals who filled in the form, already reported specific percentage trends. After comparing their sociodemographic aspects, individual protection equipment, infections and operations of dental clinics and prosthetic laboratories, significant differences between them were observed. Conclusions: The surveyed population presented a greater exposure to the risk of contracting the viral infection than in the general population. Around 30% of those surveyed have maintained their usual activity and around 15% have declared that they have closed their center. Furthermore, the most frequent masks used by them were surgical masks and FFP2, although among the higher technicians in dental prosthesis the use of surgical masks was more frequent. Discussion: In reference to the data observed in the surveys carried out at a national level, it is worth highlighting some differences between the results from the Balearic Islands surveys and the national ones. While the Balearic Islands had a percentage of 83% in relation to the participation of the urban environment, the national surveys reported a percentage of 58%. Moreover, the use of waterproof suits resulted in 78,7% in the Balearic Islands compared to 15% at a national level and around 11,5% of the participants had difficulties to obtain PPEs in the Islands compared to 80% at a national level. Finally, the number of tests for the diagnosis of COVID-19 which have been carried out on the surveyed professionals reached 43.9% in the Balearic Islands compared to just 1% of professionals nationwide, which justifies the underdiagnoses of COVID-19 among the country.

Schaffer, S. and P. O'Neill (2021). "Analysis and recommendations regarding surgeons' liabilities during an acute health crisis." Legal Medicine **51**.

The SARS-CoV-2 pandemic has highlighted discrepancies between surgeons' professional duties and legal protections when acting outside their specialities during the pandemic. These discrepancies between legal and professional standards leave surgeons and the NHS vulnerable to litigation. In the following article, we explore the liabilities that have arisen for surgeons during this period in the United Kingdom and Canada. We recommend, upon review of the literature, that a two-pronged approach be taken to address these discrepancies; (a) a change in policy at the national level to accurately reflect the constraints and demands placed upon the profession in this acute health crisis and (b) the provision of clearer, more stringent legal protection. In the interim, we suggest that individual surgeons utilise a decision-making framework where they consider their personal and professional obligations in regard to resource stewardship, innovation in practice, patient-specific contexts, and patient advocacy while acting outside of their speciality.

Schroeder, R. A. (2022). "Adaptation or Revolution: Telemental Health and Advanced Practice Psychiatric Nursing During COVID-19." Journal of the American Psychiatric Nurses Association **28**(3): 241-248.

OBJECTIVE: This article will describe the current COVID-19 crisis and the evolving mental health concerns associated with it, discuss how mental health practice has changed, and ways in which psychiatric mental health nurse practitioners (PMHNPs) can adapt and prepare for the future. METHOD: A search of current literature on the COVID-19 crisis, and topics relevant to the mental health components associated with the pandemic are reviewed. Telemental health (TMH) and PMHNP practice are discussed as they relate to the unfolding picture of the viral pandemic. RESULTS: The COVID-19 crisis is having far-reaching implications for mental health treatment and in particular for PMHNPs in practice settings. There have been widespread consequences of the containment measures used for the protection and mitigation of the disease. One such result has been the inability of patients to have face-to-face contact with their providers. The role of TMH has become increasingly important as an adaptation in professional practice. CONCLUSION: Technology has rapidly transformed traditional practice due to the COVID-19 crisis and there is strong evidence that it is well accepted by patients and providers. It is incumbent on PMHNPs to embrace TMH and become educated on best practices and TMH services.

Schultz, P., et al. (2020). "French consensus regarding precautions during tracheostomy and post-tracheostomy care in the context of COVID-19 pandemic." European Annals of Otorhinolaryngology-Head and Neck Diseases **137**(3): 167-169.

Tracheostomy post-tracheostomy care are regarded as at high risk for contamination of health care professionals with the new coronavirus (SARS-CoV-2). Considering the rapid spread of the infection, all patients in France must be considered as potentially infected by the virus. Nevertheless, patients without clinical or radiological (CT scan) markers of COVID-19, and with negative nasopharyngeal sample within 24 h of surgery, are at low risk of being infected. Instructions for personal protection include specific wound dressings and decontamination of all material used. The operating room should be ventilated after each tracheostomy and the pressure of the room should be neutral or negative. Percutaneous tracheostomy is to be preferred over surgical cervicotomy in order to reduce aerosolization and to avoid moving patients from the intensive care unit to the operating room. Ventilation must be optimized during the procedure, to limit patient oxygen desaturation. Drug assisted neuromuscular blockage is advised to reduce coughing during tracheostomy tube insertion. An experienced team is mandatory to secure and accelerate the procedure as well as to reduce risk of contamination. (C) 2020 Published by Elsevier Masson SAS.

Scopetti, M., et al. (2021). "Expanding frontiers of risk management: care safety in nursing home during COVID-19 pandemic." International Journal for Quality in Health Care **33**(1).

Background: Nursing homes provide long-term care and have residential-oriented hospitalizations characterized by medical, nursing and social-care treatments for a typically geriatric population. In the current emergency phase, the problem of infections in residential structures for the elderly is taking on considerable importance in relation to the significant prevalence rates of coronavirus disease 2019 (COVID-19). Safety improvement strategies: Prevention and control measures for severe acute respiratory syndrome coronavirus 2 infection in nursing homes should be planned before a possible outbreak of COVID-19 occurs and should be intensified during any exacerbation of the same. Each facility should identify a properly trained contact person-also external-for the prevention and control of infections, who can refer to a multidisciplinary support committee and who is in close contact with the local health authorities. The contact person should collaborate with professionals in order to prepare a prevention and intervention plan that considers national provisions and scientific evidence, the requirements for reporting patients with symptoms compatible with COVID-19 and the indications for the management of suspected, probable or confirmed cases of COVID-19. Discussion: Adequate risk management in residential structures implies the establishment of a coordination committee with dedicated staff, the implementation of a surveillance program for the rapid recognition of the outbreaks, the identification of suitable premises and equipment, the application of universal precautions, the adaptation of care plans to reduce the possibility of contagion among residents and the protection of operators and staff training initiatives.

Senyuta, I., et al. (2021). "PERSONAL DATA PROTECTION IN TERMS OF COVID-19: EXPERIENCE OF UKRAINE." Medicine and Law **40**(2): 147-163.

In terms of the novel coronavirus disease, the issues of human rights, namely those of healthcare provider and the patient, are of great interest in different states. Considering the existence of the legislative controversies and loopholes in the said area in Ukraine, the objective of the article aims to outline the procedure of personal data processing in the conditions of COVID-19, according to the legislation of Ukraine, to identify problematic issues and search for ways to solve them, to analyze the experience of foreign countries to clarify best state practices and to cover the right to privacy about the state of health, ascertaining whether there are any changes regarding the performance of the professional duty to maintain medical secrecy in conditions of the pandemic. The article outlines foreign practices on restricting human rights to privacy and reveals a balance with public health interests, according to the European Court of Human Rights jurisprudence. The current issues of the exercise of the right to medical secrecy about the state of health have been determined. The importance of the case law of the European Court of Human Rights is due to the fact that the courts in Ukraine apply the Convention for the Protection of Human Rights and Fundamental Freedoms and the case law of the European Court of Human Rights as a source of law. The effectiveness of the rights provided for in this Convention is impossible without the use of the interpretation of the Convention provided by the European Court of Human Rights in its cases. This Court establishes a coordinate system for lawmaking and law enforcement. The relevance of the outlined issues is in their sensibility and vulnerability, provided that they deal mainly with human right to privacy, finding the right balance is particularly difficult. It has been substantinated that any interference in the said right shall be temporary, legitimate and justified in a democratic society.

Sharma, A., et al. (2020). "Initial experiences of US neurologists in practice during the COVID-19 pandemic via survey." Neurology **95**(5): 215-220.

Objective To test the hypothesis that US neurologists were experiencing significant challenges with lack of personal protective equipment (PPE), rapid changes in practice, and varying institutional protocols, we conducted this survey study. The current coronavirus disease of 2019 (COVID-19) pandemic has caused widespread disease and death. Rapid increases in patient volumes have exposed weaknesses in health care systems and challenged our ability to provide optimal patient care and adequate safety measures to health care workers (HCWs). Methods A 36-item survey was distributed to neurologists around the United States through various media platforms. Results Over a 1-week period, 567 responses were received. Of these, 56% practiced in academia. A total of 87% had access to PPE, with 45% being asked to reuse PPE due to shortages. The pandemic caused rapid changes in practice, most notably a shift toward providing care by teleneurology, although a third experienced challenges in transitioning to this model. Wide variations were noted both in testing and in the guidance provided for the exposed, sick, or vulnerable HCWs. Notably, 59% of respondents felt that their practices were doing what they could, although 56% did not feel safe taking care of patients. Conclusions Results from our survey demonstrate significant variability in preparedness and responsiveness to the COVID-19 pandemic in neurology, affected by region, health care setting, and practice model. Practice guidelines from professional societies and other national entities are needed to improve protection for physicians and their patients, promote recommended practice changes during a pandemic, and optimize future preparedness for public health emergencies.

Sharma, H. B., et al. (2020). "Challenges, opportunities, and innovations for effective solid waste management during and post COVID-19 pandemic." Resources Conservation and Recycling **162**.

The crisis brought upon by the COVID-19 pandemic has altered global waste generation dynamics and therefore has necessitated special attention. The unexpected fluctuations in waste composition and quantity also require a dynamic response from policymakers. This study highlights the challenges faced by the solid waste management sector during the pandemic and the underlying opportunities to fill existing loopholes in the system. The study presents specific cases for biomedical waste, plastic waste, and food waste management - all of which have been a major cause of concern during this crisis. Further, without active citizen participation and cooperation, commingled virus-laden biomedical waste with the regular solid waste stream pose significant negative health and safety issues to sanitation workers. Single-use plastic usage is set to bounce back due to growing concerns of hygiene, particularly from products used for personal protection and healthcare purposes. It is expected that household food waste generation may reduce due to increased conscious buying of more non-perishable items during lockdown and due to concerns of food shortage. However, there is a chance of increase in food waste from the broken supply chains such as food items getting stuck on road due to restriction in vehicle movements, lack of workers in the warehouse for handling the food products, etc. The study also stresses the need for building localized resilient supply chains to counter such situations during future pandemics. While offering innovative solutions to existing waste management challenges, the study also suggests some key recommendations to the policymakers to help handle probable future pandemics if any holistically.

Sharma, K., et al. (2020). "The role of Yoga in working from home during the COVID-19 global lockdown." Work-a Journal of Prevention Assessment & Rehabilitation **66**(4): 731-737.

BACKGROUND: The COVID-19 pandemic has become a major cause of stress and anxiety worldwide. Due to the global lockdown, work, employment, businesses and the economic climate have been severely affected. It has generated stress among people from all sections of society, especially to workers who have been assigned to cater to healthcare service or those constrained to secure daily essential items. It is widely perceived that elderly or those affected by diabetes, hypertension and other cardiovascular diseases are prone to COVID-19. As per an ongoing survey, the initial data shows that the above mentioned anxiety and stress cause insomnia, and has the considerable potential to weaken the immune system, the sole protection against the virus. OBJECTIVE: This study focuses on the need of Yoga practice at work places and at home during the global lockdown due to the COVID-19 pandemic. METHODS: Literature was searched using PubMed and Google Scholar for COVID-19-related stress and anxiety at work and society due to the worldwide lockdown. The predisposing comorbidities, viral mechanism of action and treatment regimen were also searched. Yoga-based intervention studies and online programs were also searched. RESULTS: As the lockdown cannot last forever and workplaces will have to be functional soon, there is an increased possibility of recurrent infection. Therefore, Yoga can provide the necessary tool for risk reduction, amelioration of stress and anxiety and strengthening of the immune function. The online platforms provide a good media for Yoga training at work places and homes. CONCLUSION: Due to social distancing norms, the availability of Yoga trainers has become restricted. Yoga practice is actively sought to achieve reduced anxiety and stress so that improved sleep may positively impact immunity. As a consequence, there is a spurt in social media, catering to daily online Yoga sessions which apparently prove useful in providing accessible means to achieve mental as well as physical well-being.

Sharma, T., et al. (2021). "Enabling User-centered Privacy Controls for Mobile Applications: COVID-19 Perspective." Acm Transactions on Internet Technology **21**(1).

Mobile apps have transformed many aspects of clinical practice and are becoming a commonplace in health-care settings. The recent COVID-19 pandemic has provided the opportunity for such apps to play an important role in reducing the spread of the virus. Several types of COVID-19 apps have enabled healthcare professionals and governments to communicate with the public regarding the pandemic spread, coronavirus awareness, and self-quarantine measures. While these apps provide immense benefits for the containment of the spread, privacy and security of these digital tracing apps are at the center of public debate. To address this gap, we conducted an online survey of a midwestern region in the United State to assess people's attitudes toward such apps and to examine their privacy and security concerns and preferences. Survey results from 1,550 participants indicate that privacy/security protections and trust play a vital role in people's adoption of such apps. Furthermore, results reflect users' preferences wanting to have control over their personal information and transparency on how their data is handled. In addition, personal data protection priorities selected by the participants were surprising and yet revealing of the disconnect between technologists and users. In this article, we present our detailed survey results as well as design guidelines for app developers to develop innovative human-centered technologies that are not only functional but also respectful of social norms and protections of civil liberties. Our study examines users' preferences for COVID-19 apps and integrates important factors of trust, willingness, and preferences in the context of app development. Through our research findings, we suggest mechanisms for designing inclusive apps' privacy and security measures that can be put into practice for healthcare-related apps, so that timely adoption is made possible.

Shen, J., et al. (2020). "Prevention and control of COVID-19 in public transportation: Experience from China." Environmental Pollution **266**.

Due to continuous spread of coronavirus disease 2019 (COVID-19) worldwide, long-term effective prevention and control measures should be adopted for public transport facilities, as they are increasing in popularity and serve as the principal modes for travel of many people. The human infection risk could be extremely high due to length of exposure time window, transmission routes and structural characteristics during travel or work. This can result in the rapid spread of the infection. Based on the transmission characteristics of Severe Acute Respiratory Syndrome Coronavirus 2 (SARS-CoV-2) and the nature of public transport sites, we identified comprehensive countermeasures toward the prevention and control of COVID-19, including the strengthening of personnel management, personal protection, environmental cleaning and disinfection, and health education. Multi-pronged strategies can enhance safety of public transportation. The prevention and control of the disease during the use of public transportation will be particularly important when all countries in the world resume production. The aim of this study is to introduce experience of the prevention and control measures for public transportation in China to promote the global response to COVID-19. (C) 2020 Elsevier Ltd. All rights reserved.

Shields, A. D., et al. "Maintaining access to maternal fetal medicine care by telemedicine during a global pandemic." Journal of Telemedicine and Telecare.

Objective This study aims to compare a conventional medical treatment model with a telehealth platform for Maternal Fetal Medicine (MFM) outpatient care during the global novel coronavirus pandemic. Methods In this study, we described the process of converting our MFM clinic from a conventional medical treatment model to a telemedicine platform. We compared clinical productivity between the two models. Outcomes were analysed using standard statistical tests. Results We suffered three symptomatic COVID-19 infections among our clinical providers and staff prior to the conversion, compared with none after the conversion. We had a significant decrease in patient visits following the conversion (53.35 visits per day versus 40.3 visits per day,p < 0.0001). However, our average daily patient visits per full-time equivalent (FTE) were only marginally reduced (11.1 visit per FTE versus 7.6 visits per FTE,p < 0.0001), resulting in a relative decrease in adjusted work relative value units (6987 versus 5440). There was an increase in more basic follow-up ultrasound procedures, complexity (current procedural technology [CPT] code 76816 (10.7% versus 19.5%, relative risk [RR] 1.81, 95% CI 1.60-2.05,p < 0.0001)) over comprehensive follow-up ultrasound procedures, CPT code 76805 (17.2% versus 7.8%, RR 0.46, 95% CI 0.39-0.53,p < 0.0001) after conversion. Despite similar proportions of new consults, there was an increase in the proportion of follow-up visits and medical decision-making complexity evaluation and management CPT codes (e.g. 99214/99215) after the conversion (17.2% versus 24.6%, RR 1.43, 95% CI 1.26-163, p < 0.0001). There were no differences between amniocentesis procedures performed between the two time periods (0.3% versus 0.2%,p = 0.5805). Conclusion The rapid conversion of an MFM platform from convention medical treatment to telemedicine platform in response to the novel coronavirus pandemic resulted in protection of healthcare personnel and MFM patients, with only a modest decrease in clinical productivity during the initial roll-out. Due to the ongoing threat from the novel coronavirus-19, an MFM telemedicine platform is a practicable and innovative solution and merits the continued support of CMS and health care administrators.

Silva, A., et al. (2021). "Limiting factors of face shield use for health professionals during the COVID-19 pandemic." Work-a Journal of Prevention Assessment & Rehabilitation **70**(2): 355-363.

BACKGROUND: Coronavirus disease (COVID-19) is caused by SARS-COV2, a highly transmissible and pathogenic viral infection, and was identified in December 2019 in Wuhan, China. Three months later, it became a severe pandemic. OBJECTIVE: To identify scientific evidence on the use of face shields by health professionals during the COVID-19 pandemic period. METHOD: An integrative literature review of articles obtained from PubMed, Cumulative Index to Nursing and Allied Health Literature (CINAHL), and Web of Science databases was undertaken. For the search, controlled, non-controlled descriptors and specific keywords: "face shield," "fluid resistance procedure," "respiratory infections," "healthcare workers," "COVID-19," "aerosols," and "personal protection infection" were used. RESULTS: The sample comprised seven studies. The available evidence has shown that face shields do not have a defined standard for their production-their effectiveness depends on the quality of the visor, structure, and fixation system. They must be used as adjuvants to other personal protective equipment (PPE), and their isolated use is not recommended due to the fragilities of peripheral face sealing, especially during the COVID-19 pandemic period. CONCLUSION: Due to the shortage of this equipment, domestic face shields can be indicated if they meet production requirements, based on scientific evidence for their efficient use.

Sinonquel, P., et al. (2020). "COVID-19 and gastrointestinal endoscopy: What should be taken into account?" Digestive Endoscopy **32**(5): 723-731.

On March 11, 2020 the World Health Organization declared COVID-19 pandemic, leading to a subsequent impact on the entire world and health care system. Since the causing Severe Acute Respiratory Syndrome Coronavirus 2 houses in the aerodigestive tract, activities in the gastrointestinal outpatient clinic and endoscopy unit should be limited to emergencies only. Health care professionals are faced with the need to perform endoscopic or endoluminal emergency procedures in patients with a confirmed positive or unknown COVID-19 status. With this report, we aim to provide recommendations and practical relevant information for gastroenterologists based on the limited amount of available data and local experience, to guarantee a high-quality patient care and adequate infection prevention in the gastroenterology clinic.

Smith, P. M., et al. (2021). "The Association between the Perceived Adequacy of Workplace Infection Control Procedures and Personal Protective Equipment with Mental Health Symptoms: A Cross-sectional Survey of Canadian Health-care Workers during the COVID-19 Pandemic: L'association entre le caractere adequat percu des procedures de controle des infections au travail et de l'equipement de protection personnel pour les symptomes de sante mentale. Un sondage transversal des travailleurs de la sante canadiens durant la pandemie COVID-19." Canadian Journal of Psychiatry-Revue Canadienne De Psychiatrie **66**(1): 17-24.

Objectives: To examine the relationship between perceived adequacy of personal protective equipment (PPE) and workplace-based infection control procedures (ICP) and mental health symptoms among a sample of health-care workers in Canada within the context of the current COVID-19 pandemic. Methods: A convenience-based internet survey of health-care workers in Canada was facilitated through various labor organizations between April 7 and May 13, 2020. A total of 7,298 respondents started the survey, of which 5,988 reported information on the main exposures and outcomes. Anxiety symptoms were assessed using the Generalized Anxiety Disorder (GAD-2) screener, and depression symptoms using the Patient Health Questionnaire (PHQ-2) screener. We assessed the perceived need and adequacy of 8 types of PPE and 10 different ICP. Regression analyses examined the proportion of GAD-2 and PHQ-2 scores of 3 and higher across levels of PPE and ICP, adjusted for a range of demographic, occupation, workplace, and COVID-19-specific measures. Results: A total of 54.8% (95% confidence interval [CI], 53.5% to 56.1%) of the sample had GAD-2 scores of 3 and higher, and 42.3% (95% CI, 41.0% to 43.6%) of the sample had PHQ-2 scores of 3 and higher. Absolute differences of 18% (95% CI, 12% to 23%) and 17% (95% CI, 12% to 22%) were observed in the prevalence of GAD-2 scores of 3 and higher between workers whose perceived PPE needs and ICP needs were met compared to those who needs were not met. Differences of between 11% (95% CI, 6% to 17%) and 19% (95% CI, 14% to 24%) were observed in PHQ-2 scores of 3 and higher across these same PPE and ICP categories. Conclusions: Our results suggest strengthening employer-based infection control strategies likely has important implications for the mental health symptoms among health-care workers in Canada.

Spoelder, E. J., et al. (2021). "Helicopter transport of critical care COVID-19 patients in the Netherlands: protection against COVID-19 exposure-a challenge to critical care retrieval personnel in a novel operation." Scandinavian Journal of Trauma Resuscitation & Emergency Medicine **29**(1).

Background During the Coronavirus Disease 2019 (COVID-19) outbreak in the Netherlands, the demand for intensive care beds exceeded availability within days. Initially, patients were redistributed regionally by ground transport. When transport over longer distances became necessary, we initiated a new Helicopter Emergency Medical Service (HEMS) operation. We hypothesize that the transport of contagious COVID-19 patients is feasible and safe for patients and HEMS personnel. Methods In this retrospective, single-centre observational study, flight and monitor data were used to calculate the exposure time of the retrieval team to COVID-19 patients. All the crew members (n = 18) were instructed on the proper use of personal protective equipment (PPE), dressing and undressing routine using buddy check supervision and cleaning procedures. All the team members were monitored for possible COVID-19 symptoms, as advised by our National Institute for Health and Environment. One month after completing the aeromedical transport all crew members were asked to donate a blood sample which was examined for the presence of IgG antibodies to SARS-CoV-2. Results From March 24 to May 25, 2020 the HEMS team transported 67 ventilated critical care COVID-19 patients. The exposure time was 7451 min (124 h and 11 min). One HEMS member reported pneumonia 6 weeks before the start of the patient transport. He tested positive for IgG SARS-CoV-2 by serology testing. We speculate that he was infected before the start of the operation; irrefutable evidence is lacking to support this claim because we did not perform serology testing before this operation started. Conclusion Occupational COVID-19 exposure during helicopter transport of ventilated critical care COVID-19 patients can be performed safely when proper PPE is applied.

Spoletini, G., et al. (2020). "Transplantation during the COVID-19 pandemic: nothing noble is accomplished without danger." Bmc Gastroenterology **20**(1).

The global health crisis due to the fast spread of coronavirus disease (COVID-19) has caused major disruption in all aspects of healthcare. Transplantation is one of the most affected sectors, as it relies on a variety of services that have been drastically occupied to treat patients affected by COVID-19. With this report from two transplant centers in Italy, we aim to reflect on resource organization, organ allocation, virus testing and transplant service provision during the course of the pandemic and to provide actionable information highlighting advantages and drawbacks. To what extent can we preserve the noble purpose of transplantation in times of increased danger? Strategies to minimize risk exposure to the transplant population and health- workers include systematic virus screening, protection devices, social distancing and reduction of patients visits to the transplant center. While resources for the transplant activity are inevitably reduced, new dilemmas arise to the transplant community: further optimization of time constraints during organ retrievals and implantation, less organs and blood products donated, limited space in the intensive care unit and the duty to maintain safety and outcomes.

Stuby, L., et al. (2021). "Impact of Face-to-Face Teaching in Addition to Electronic Learning on Personal Protective Equipment Doffing Proficiency in Student Paramedics: Protocol for a Randomized Controlled Trial." Jmir Research Protocols **10**(4).

Background: The COVID-19 pandemic has brought attention to the importance of correctly using personal protective equipment (PPE). Doffing is a critical phase that increases the risk of contamination of health care workers. Although a gamified electronic learning (e-learning) module has been shown to increase the adequate choice of PPE among prehospital personnel, it failed to enhance knowledge regarding donning and doffing sequences. Adding other training modalities such as face-to-face training to these e-learning tools is therefore necessary to increase prehospital staff proficiency and thus help reduce the risk of contamination. Objective: The aim of this study is to assess the impact of the Peyton 4-step approach in addition to a gamified e-learning module for teaching the PPE doffing sequence to first-year paramedic students. Methods: Participants will first follow a gamified e-learning module before being randomized into one of two groups. In the control group, participants will be asked to perform a PPE doffing sequence, which will be video-recorded to allow for subsequent assessment. In the experimental group, participants will first undergo face-to-face training performed by third-year students using the Peyton 4-step approach before performing the doffing sequence themselves, which will also be video-recorded. All participants will then be asked to reconstruct the doffing sequence on an online platform. The recorded sequences will be assessed independently by two investigators: a prehospital emergency medicine expert and an infection prevention and control specialist. The assessors will be blinded to group allocation. Four to eight weeks after this first intervention, all participants will be asked to record the doffing sequence once again for a subsequent skill retention assessment and to reconstruct the sequence on the same online platform to assess knowledge retention. Finally, participants belonging to the control group will follow face-to-face training. Results: The study protocol has been presented to the regional ethics committee (Req-2020-01340), which issued a declaration of no objection as such projects do not fall within the scope of the Swiss federal law on human research. Study sessions were performed in January and February 2021 in Geneva, and will be performed in April and June 2021 in Bern. Conclusions: This study should help to determine whether face-to-face training using the Peyton 4-step approach improves the application and knowledge retention of a complex procedure when combined with an e-learning module.

Subert, M. (2020). "A gender-sensitive approach to US crisis communication for COVID-19 and beyond." Journal of Contingencies and Crisis Management **28**(4): 487-489.

Examining the COVID-19 pandemic communication from a gender perspective is ethically critical and socially timely. Combined with class, it helps us to identify how effective communication has been during this pandemic. This Forum contribution takes a gender-sensitive approach with the premise that all public health crises are inherently gendered phenomena. Two global initiatives are crucial for our purposes: The United Nations Security Council Resolution 1325 (UNSCR 1325) and the Women, Peace and Security (WPS) agenda. Based on the four strategic pillars (prevention, participation, protection, and relief and recovery) we ask the following: "Who (a) is prevented from harm by the COVID-19 recovery efforts, who (b) is participating in, who (c) receives protection from and who (d) will receive assistance through the COVID-19 recovery efforts?". We conclude that the COVID-19 crisis has had its worst effects on the poorest and most vulnerable. During the pandemic, there was a strong military-style communication limited to government and health professionals that lacked empathy. The gender-sensitive perspective reflected by UNSCR 1325 and the WPS requires us to give up military masculinity in communication, include more empathy and dialog (mutuality and cooperation), and extend women's participation to reduce the damaging effects of any given crisis on all people.

Suppan, L., et al. (2020). "Effect of an E-Learning Module on Personal Protective Equipment Proficiency Among Prehospital Personnel: Web-Based Randomized Controlled Trial." Journal of Medical Internet Research **22**(8).

Background: To avoid misuse of personal protective equipment (PPE), ensure health care workers' safety, and avoid shortages, effective communication of up-to-date infection control guidelines is essential. As prehospital teams are particularly at risk of contamination given their challenging work environment, a specific gamified electronic learning (e-learning) module targeting this audience might provide significant advantages as it requires neither the presence of learners nor the repetitive use of equipment for demonstration. Objective: The aim of this study was to evaluate whether a gamified e-learning module could improve the rate of adequate PPE choice by prehospital personnel in the context of the coronavirus disease (COVID-19) pandemic. Methods: This was an individual-level randomized, controlled, quadruple-blind (investigators, participants, outcome assessors, and data analysts) closed web-based trial. All emergency prehospital personnel working in Geneva, Switzerland, were eligible for inclusion, and were invited to participate by email in April 2020. Participants were informed that the study aim was to assess their knowledge regarding PPE, and that they would be presented with both the guidelines and the e-learning module, though they were unaware that there were two different study paths. All participants first answered a preintervention quiz designed to establish their profile and baseline knowledge. The control group then accessed the guidelines before answering a second set of questions, and were then granted access to the e-leaming module. The e-learning group was shown the e-learning module right after the guidelines and before answering the second set of questions. Results: Of the 291 randomized participants, 176 (60.5%) completed the trial. There was no significant difference in baseline knowledge between groups. Though the baseline proportion of adequate PPE choice was high (75%, IQR 50%-75%), participants' description of the donning sequence was in most cases incorrect. After either intervention, adequate choice of PPE increased significantly in both groups (P<.001). Though the median of the difference in the proportion of correct answers was slightly higher in the e-learning group (17%, IQR 8%-33% versus 8%, IQR 8%-33%), the difference was not statistically significant (P=.27). Confidence in the ability to use PPE was maintained in the e-learning group (P=.27) but significantly decreased in the control group (P=.04). Conclusions: Among prehospital personnel with an already relatively high knowledge of and experience with PPE use, both web-based study paths increased the rate of adequate choice of PPE. There was no major added value of the gamified e-learning module apart from preserving participants' confidence in their ability to correctly use PPE.

Sureka, B., et al. (2021). "Suraksha Chakra: A new scoring system to protect the healthcare workers from COVID." Journal of Family Medicine and Primary Care **10**(4): 1512-1514.

Doctors, nurses, house cleaning staff and hospital ward attendants are at increased risk of acquiring coronavirus disease 2019 (COVID-19) if there is a breach in the personal protection equipment. A new simple, easy to implement scoring system has been developed by our Suraksha Chakra team which can be used by the policy makers and hospital administrators. The scoring system is not to discourage anyone but constantly reinforce the healthcare workers for safe practices during patient care.

Swart, R., et al. (2021). "The influence of a Perspex intubation box on time to intubation: a simulation-based randomised crossover study." Southern African Journal of Anaesthesia and Analgesia **27**(1): 24-28.

Background: Standard personal protective equipment guidelines are insufficient to prevent contamination of healthcare workers with droplet spread during the COVID-19 crisis. The added challenge of adequate aerosol protection has led to the development of an initial prototype intubation box. The primary objective was to determine the impact of an intubation box on the mean time to completion of intubation in a simulated airway. Secondary objectives included the best laryngoscopic view, the effect of intubator seniority and mode of laryngoscopy on intubation. Methods: This was a randomised crossover study of the influence of an intubation box on mean time to completion of intubation of an airway management part-task trainer. Senior anaesthesiology staff were assigned to two groups and recordings of their attempts at intubation were analysed by two independent observers. Results: The intubation box led to a significantly longer mean time to completion of intubation of 7.6 seconds (95% CI 3.1; 12.2; p = 0.001) with direct laryngoscopy and 9.2 seconds (95% CI 3.8; 14.7; p = 0.001) with videolaryngoscopy. It did not influence best glottic view. Conclusion: We found that the use of an intubation box significantly prolonged the time to completion of intubation, but the clinical significance of the effect size is uncertain.

Swiatkowska, B., et al. (2021). "HEALTH PROTECTION OF EMPLOYEES AGAINST SARS-COV-2 CORONAVIRUS INFECTION CAUSING THE COVID-19 DISEASE - THE CURRENT STATE OF KNOWLEDGE AND RECOMMENDATIONS." Medycyna Pracy **72**(1): 69-87.

The COVID-19 pandemic, despite the restrictions and preventive measures applied, has rapidly spread and reached Poland. The adaptation to the dynamically changing epidemiological situation requires a prompt implementation of effective preventive measures. The aim of the publication is to provide current knowledge to all persons involved in the preventive care system, i.e., employees, employers and professionals of occupational medicine, about the epidemiological situation related to SARS-CoV-2, as well as recommendations and possible solutions. In order to analyze these issues, a review of literature was conducted based on medical research databases: PubMed, SCOPUS, and the Web of Science Core Collection. The literature was supplemented with studies found on websites of the Ministry of Health and the World Health Organization. Data on the cases of and deaths due to COVID-19 come from reports provided by the Ministry of Health, data published on the websites of the European Center for Disease Prevention and Control, and ourworldindata.org. By the time of submitting the publication, 34 154 cases and 1444 deaths due to coronavirus had been recorded in Poland. Data from published studies suggest that the virus is mainly transmitted via droplets or through contact with contaminated objects and surfaces. Therefore, in the absence of an effective vaccine, preventive actions are based mainly on strategies that minimize the risk of pathogen transmission. In addition to discussing the current epidemiological situation, diagnostic procedures, risk groups and COVID-19 characteristics, the paper presents recommendations and proposed solutions for employers and employees regarding the prevention of SARS-CoV-2, along with currently applicable laws and recommendations on employee prophylactic examinations during the pandemic. Subsequently, COVID-19 was discussed in the aspect of an occupational disease and other health threats related to the pandemics. The epidemiological situation regarding coronavirus indicates the need to take immediate and effective actions to minimize infection transmission among employees, and to develop procedures for a quick and effective ability to locate the COVID-19 outbreaks in workplaces.

Talat, N., et al. (2020). "Psychosocial Effects of COVID-19 on Health Care Workers: A Cross Sectional Study from Tertiary Level Pediatric Hospital." Annals of King Edward Medical University Lahore Pakistan **26**: 170-175.

Objective: To identify the Psychosocial effects of COVID-19 pandemic on health care workers of a tertiary level pediatric hospital. Methodology: This is a cross-sectional study done at The Children's Hospital and the Institute of Child Health, Lahore from 1st April to 15th April 2020. A questionnaire was circulated among health care workers of the hospital and responses were analyzed with SPSS V. 23. Results: The questionnaire was filled by 989 hospital employees but 914 forms with complete were analyzed. Most participants (75%) were younger than 40 years of age. The male to female ratio was 1:1.5. There was almost equal participation of married and unmarried medical personnel (52% vs 48%). Regarding concerns about personal and family health, 544 (59.5%) were deeply concerned about their health, but the concern was far greater about their families (672, 73.5%). The fear of going home was expressed by 629 (69%) participants. Regarding employee protection using personal protective equipment (PPE), 680 (74.4%) hospital workers were dissatisfied. Wearing a protective suit and gloves were the precautions frequently cited as most bothersome (38.8%). Over 65% of workers felt anxiety while dealing with febrile patients. Over 60% of health care workers cited religion as their main source of coping with psychological impact. Conclusions: Health care workers experience a great deal of psychological implications of the COVID-19 pandemic related to the uncertainty of the disease behavior, associated comorbidities and risk factors in the health care workers, and healthcare-related local policies.

Talukdar, D., et al. (2021). "Strategic Role and Challenges of Community Pharmacists in SARS-CoV-2 Outbreak." Journal of Research in Pharmacy Practice **10**(1): 1-9.

This study highlights the importance of community pharmacists' strategic role in hindering the progression of the SARS-CoV-2 virus in the community setting and innovative measures to protect themselves. This article focuses on the features, control, and prevention of COVID-19 and social awareness measures of e pandemic. The means employed by the community pharmacist to safeguard his health while providing pharmaceutical services during COVID-19 is compiled and presented to benefit health-care professionals around the world. As per the US Center for Disease Control and Prevention, community pharmacists play a crucial role in providing essential drugs to patients without knowing their current COVID-19 status. They also work in conditions that make them susceptible to COVID-19 exposure. Despite the availability of guidelines, community pharmacists need to be trained in personal protective equipment for efficient protection and prevention of spread. Community pharmacists are essential frontline warriors against transmission of the SARS-CoV-2 virus in the community and act as frontline workers to educate the public on COVID-19. They are at high risk and need to observe necessary precautions to mitigate the spread of the virus.

Tarakji, B., et al. (2020). "COVID-19 Guidelines to Protect Healthcare Workers at Hospitals and Dental Professionals at Dental Office." Ethiopian Journal of Health Sciences **30**(6): 1037-1042.

BACKGROUND. Coronavirus disease 2019 is an infectious disease caused by severe acute respiratory syndrome coronavirus 2. This study aimed to address the preventive procedures to protect healthcare workers at hospital to avoid COVID-19, and infection control procedures to protect dental professionals in dental office. METHODS: We conducted a search of published articles from PubMed, google scholar databases using key words such as COVID-19, healthcare worker, infection control, and dental practice. Relevant articles were identified and reviewed. Most published papers were clinical reports and case studies. We have selected some of the current published papers written in English in 2020. RESULTS: Infection control procedures to protect health workers at hospitals, and dental professionals at dental office were summurised and presented. Infection control procedures for healthcare workers at hospitals include Personal protective equipment, Korea filter (KF)94 respirator, goggles, face protector, disposable waterproof long-arm gown, and gloves, and others. Extra-protection procedures should be taken with old and vulnerable healthcare workers. Dental professionals should evaluate patients in advance before starting dental treatment. Aerosols generating procedures should be avoided and personal protective equipment should be used. Dental treatment should be restricted to emergency cases only. CONCLUSION: Old medical staff should be in safer distance to avoid infection, but young physicians and nurses should work at frontline as their immunity is better than their colleagues at old age. Screening patients and measurement of the body temperature are essential measures before dental treatment.

Thapa, B. (2021). "Role of Eyes and Eyes Protection Amidst SARS-CoV-2 Infection." Journal of Nepal Medical Association **59**(233): 108-110.

Severe Acute Respiratory Syndrome Coronavirus 2 pandemic has infected millions of people. The conjunctival epithelium is easily exposed to infectious droplets and body fluids making eyes a potential route and reservoir of the infection. The CD147 and ACE2 receptor has been demonstrated in ocular surface cells, which implies that these cells may facilitate as a portal of entry for transmission of Severe Acute Respiratory Syndrome Coronavirus 2. Despite low viral load in tears and conjunctival swab, the negative RT-PCR results cannot exclude the possibility of the presence of Severe Acute Respiratory Syndrome Coronavirus 2 in ocular secretions. Pathogens might be transported by constant tear flow through the lacrimal duct system to the respiratory tract causing infection. Eyes are unlikely to be the main transmission route, however, their role in the transmission of Severe Acute Respiratory Syndrome Coronavirus 2 cannot be overlooked. Therefore, proper eye protection should be instituted while attending Severe Acute Respiratory Syndrome Coronavirus 2 positive individuals, especially by health professionals.

Thor, J., et al. (2020). "Roadmap Out of COVID-19." Malaysian Orthopaedic Journal **14**(3): 4-9.

The recent coronavirus disease (COVID-19) was declared as a public health emergency by the World Health Organisation on 30th January 2020, and has now affected more than 100 countries. Healthcare institutions and governments worldwide have raced to contain the disease, albeit to varying degrees of success. Containment strategies adopted range from complete lockdowns to remaining open with public advisories regarding social distancing. However, general principles adopted by most countries remain the same, mainly to avoid gatherings in large numbers and limit social interactions to curb the spread of disease. In Singapore, this disease had a very different progression. The first wave of the disease started with the confirmation of the first COVID-19 positive patient in Singapore on 23rd January 2020. Initially, the daily number of confirmed cases were low and manageable. With a rise in unlinked cases, the Disease Outbreak Response System Condition (DORSCON) status was raised from yellow to orange. New cluster outbreaks in foreign worker dormitories led to the rampant spread of disease, with daily spikes of COVID-19 cases. As of 7th June 2020, we have a total of 37,910 confirmed cases of COVID-19 infections, the highest in Southeast Asia, 12,999 active cases and a manageable mortality count of 25 deaths. This details our unique method for dealing with a pandemic, including a brief demographic of trauma patients during this period. We were able to conserve sufficient resources to ensure that our essential services can still continue. Moving on, we have to ensure the continued protection of our population, especially the vulnerable groups such as the elderly and the immunocompromised, as we reopen.

Tsilingiris, D., et al. (2020). "Providing evidence on the ongoing health care workers' mask debate." Internal and Emergency Medicine **15**(5): 773-777.

The scarcity of facemasks, particularly N95 respirators, combined with the lack of solid data to address the suitability of each mask type for adequate health care worker (HCW) protection have caused turmoil among HCWs. Current recommendations suggest mask usage solely during HCW contact with Covid-19 patients, namely plain medical mask for low-risk contacts and N95 for aerosol generating procedures. The distinction regarding the escalation of mask complexity depending on contact type is nevertheless based on plausible theoretical assumptions rather than hard evidence of a clear benefit. Conversely, we suggest that at least a plain mask should be used during all HCWs' contacts in healthcare facilities which constitute a highly probable but often overlooked means of SARS-CoV-2 transmission among HCWs.

Turktemiz, H., et al. (2021). "Assessment of healthcare professionals' perceptions and attitudes towards the COVID-19 pandemic in Turkey." Work-a Journal of Prevention Assessment & Rehabilitation **69**(4): 1163-1170.

BACKGROUND: Determining the healthcare professionals' perceptions and attitudes towards the COVID-19 pandemic will contribute to managing and struggling their efforts against COVID-19. OBJECTIVE: This study aimed to determine the perceptions and attitudes of healthcare professionals who are at the forefront in the fight against this pandemic. METHODS: In the study, the survey technique used as the data collection method and 216 participants agreed to participate. The questionnaire consisted of three sections. Reliability analysis and descriptive statistical methods used to analyze the data obtained using the SPSS package program. RESULTS: According to the study results, 80.4% of the participants perceive the probability of being infected with the COVID-19 to be very high, and 64% of them consider that the conditions are very favorable for them to be infected. More than half of them are afraid of being infected and believe that if they contract COVID-19, adverse effects may continue for a long time. Furthermore, almost all participants (96.20%) consider personal protective equipment beneficial and will protect them from the COVID-19 as a wise preventive measure. However, 82.30% of the participants stated that using personal protective equipment is uncomfortable, and 76.60% said it is challenging to take care of patients while using personal protective equipment. CONCLUSIONS: The current study results show that healthcare professionals are aware of how serious COVID-19 is, understand the importance of protective equipment in protection from COVID-19, and are seriously afraid of COVID-19. Given that the fears of healthcare professionals may have adverse effects on them, it is recommended that healthcare administrators take measures to comfort healthcare workers and ensure that they maintain their positive attitude towards COVID-19.

Unger, J. P. (2021). "Comparison of COVID-19 Health Risks With Other Viral Occupational Hazards." International Journal of Health Services **51**(1): 37-49.

The European Commission periodically classifies viruses on their occupational hazards to define the level of protection that workers are entitled to claim. Viruses belonging to Groups 3 and 4 can cause severe human disease and hazard to workers, as well as a spreading risk to the community. However, there is no effective prophylaxis or treatment available for Group 4 viruses. European trade unions and the Commission are negotiating the classification of the COVID-19 virus along these 2 categories. This article weighs the reasons to classify it in Group 3 or 4 while comparing its risks to those of the most significant viruses classified in these 2 categories. COVID-19 characteristics justify its classification in Group 4. Contaminated workers in contact with the public play an important role in disseminating the virus. In hospitals and nursing homes, they increase the overall case fatality rate. By strongly protecting these workers and professionals, the European Union would not only improve health in work environments, but also activate a mechanism key to reducing the COVID-19 burden in the general population. Admittedly, the availability of a new vaccine or treatment would change this conclusion, which was reached in the middle of the first pandemic.

Unuvar, G. K., et al. (2021). "Current infection prevention and control strategies of COVID-19 in hospitals." Turkish Journal of Medical Sciences **51**: 3215-3220.

Background/aim: Coronavirus disease 2019 (COVID-19), caused by the severe acute respiratory syndrome coronavirus-2 (SARS-CoV-2), has been appeared first in China since December 2019. Transmission of SARS-CoV-2 occurs primarily with droplets through coughing and sneezing and also occurs through inhalation of aerosolized secretions, which travel, remain suspended in the air longer. Materials and methods: Since early stages of the outbreak, COVID-19 cases have been described in healthcare workers (HCWs). However, in the early stages, the disease may be asymptomatic. This may lead to incorrect diagnosis or delayed diagnosis and may lead to the nosocomial spread of the virus. One of the most important causes of transmission among HCWs is being exposed to an aerosolized virus in a closed environment for a long time. It is possible to prevent and control the spread of COVID-19 in hospitals with outpatient treatment and triage. Results: Infection control measures, including wearing surgical masks, hand hygiene, and social distance are considered essential in preventing human-to-human transmissions of SARS-CoV-2. Immediate response and practices of infection control measures are critical for saving lives during an epidemic inside and outside the hospital. Conclusion: Analyzing current knowledge about the features of SARS-CoV-2 infection, screening, personal protection protocols, triage and psychological support practices for healthcare professionals can be promising in terms of controlling the infection.

Uzman, S. and T. A. Ayazoglu (2021). "SARS-CoV-2 Infection in Intensive Care Unit Healthcare Workers in Turkey: A Tertiary Center Cohort Study." Haseki Tip Bulteni-Medical Bulletin of Haseki **59**(2): 103-107.

Aim: Intensive care unit (ICU) workers have a greater risk of being infected due to procedures requiring intensive contact with the coronavirus disease-2019 (COVID-19) patient. We aimed to explain how we applied the protection of healthcare workers against the contagion of COVID-19 infection in the ICU of a university hospital. Methods: The study was performed in ICUs of the Gortepe Training Research Hospital with 650 beds which was affiliated to Medeniyet University in Istanbul, Turkey. We recorded the number of COVID-19 patients. We also recorded ICU workers (doctor, nurse, and others), and their working layouts. The frequency of the contagion of severe acute respiratory syndrome coronavirus-2 (SARS-CoV-2) to ICU workers was investigated. We also evaluated precautions for the prevention and evaluated the arrangement of ICUs for COVID-19 patients. Results: Between March 17th 2020 and May 26th 2020 totaly, a total of 120 hospitalized COVID-19 patients were treated in ICUs. Laboratory confirmed COVID-19 was detected in only one health-care worker in ICUs. Conclusion: Our experience showed that the correct and proper use of personal protective equipment according to the protocols are effective in protecting ICU workers against the contagion of SARS-CoV-2.

Vega, M. L., et al. (2020). "Preliminary results of a non-invasive ventilatory support unit in SARS-COVID-2." Medicina-Buenos Aires **80**: 1-8.

The use of non-invasive respiratory support in the context of the COVID-19 pandemic is controversial. The aim of this observational study was to show the experience of the first month since the creation of a Non-invasive Ventilatory Support Unit (NIVSU) at Hospital Fernandez. We describe the creation of the NIVSU, the health professional-patient ratio. the type of room, the modified personal protection equipment; diagnostic. monitoring and ventilatory support equipment for treatment. as well as the inclusion criteria and the treatment algorithm. Twenty five (63%) of patients were referred from the Internal Medicine Ward, 10 (25%)) from Shock Room, and 5 (13%) from Emergency Ward. National Early Warning Score. Acute Physiology and Chronic Health Disease Classification System II and Sequential Organ Failure Assessment, were calculated on admission. with a median of 12, 8, and 2 points, respectively. The Lung Ultrasonography Score was taken to quantify lung ultrasound findings. All patients were admitted with a reservoir mask, 80% inspired O-2 fraction was estimated for the calculation of arterial O-2 pressure/ inspired O-2 fraction ratio (Pa/FiO(2)) at admission. The median of time elapsed from the onset of symptoms referred by the patient to UNIT admission was 13 days. The development of NIVSU prevented a large proportion of patients from being transferred to Intensive Care Unit (ICU) and it could be beneficial in preserving ICUs capacity. These early results suggest that non-invasive treatment may be beneficial for the treatment of severe acute respiratory failure by COVID-19.

Venketasubramanian, N., et al. (2021). "Stroke Care during the COVID-19 Pandemic: International Expert Panel Review." Cerebrovascular Diseases **50**(3): 245-261.

Background: Coronavirus disease 2019 (COVID-19) has placed a tremendous strain on healthcare services. This study, prepared by a large international panel of stroke experts, assesses the rapidly growing research and personal experience with COVID-19 stroke and offers recommendations for stroke management in this challenging new setting: modifications needed for prehospital emergency rescue and hyperacute care; inpatient intensive or stroke units; posthospitalization rehabilitation; follow-up including at-risk family and community; and multispecialty departmental developments in the allied professions. Summary: The severe acute respiratory syndrome coronavirus 2 uses spike proteins binding to tissue angiotensin-converting enzyme (ACE)-2 receptors, most often through the respiratory system by virus inhalation and thence to other susceptible organ systems, leading to COVID-19. Clinicians facing the many etiologies for stroke have been sobered by the unusual incidence of combined etiologies and presentations, prominent among them are vasculitis, cardiomyopathy, hypercoagulable state, and endothelial dysfunction. International standards of acute stroke management remain in force, but COVID-19 adds the burdens of personal protections for the patient, rescue, and hospital staff and for some even into the postdischarge phase. For pending COVID-19 determination and also for those shown to be COVID-19 affected, strict infection control is needed at all times to reduce spread of infection and to protect healthcare staff, using the wealth of well-described methods. For COVID-19 patients with stroke, thrombolysis and thrombectomy should be continued, and the usual early management of hypertension applies, save that recent work suggests continuing ACE inhibitors and ARBs. Prothrombotic states, some acute and severe, encourage prophylactic LMWH unless bleeding risk is high. COVID-19-related cardiomyopathy adds risk of cardioembolic stroke, where heparin or warfarin may be preferable, with experience accumulating with DOACs. As ever, arteritis can prove a difficult diagnosis, especially if not obvious on the acute angiogram done for clot extraction. This field is under rapid development and may generate management recommendations which are as yet unsettled, even undiscovered. Beyond the acute management phase, COVID-19-related stroke also forces rehabilitation services to use protective precautions. As with all stroke patients, health workers should be aware of symptoms of depression, anxiety, insomnia, and/or distress developing in their patients and caregivers. Postdischarge outpatient care currently includes continued secondary prevention measures. Although hoping a COVID-19 stroke patient can be considered cured of the virus, those concerned for contact safety can take comfort in the increasing use of telemedicine, which is itself a growing source of patient-physician contacts. Many online resources are available to patients and physicians. Like prior challenges, stroke care teams will also overcome this one. Key Messages: Evidence-based stroke management should continue to be provided throughout the patient care journey, while strict infection control measures are enforced.

Vergara, J., et al. (2021). "Swallowing and Communication Management of Tracheostomy and Laryngectomy in the Context of COVID-19 A Review." Jama Otolaryngology-Head & Neck Surgery **147**(1): 85-90.

This review synthesizes the literature regarding tracheostomy and laryngectomy management in the context of the COVID-19 pandemic. Importance The care of patients with a surgically modified airway, such as tracheostomy or laryngectomy, represents a challenge for speech-language pathologists (SLPs) in the context of the coronavirus disease 2019 (COVID-19) pandemic. The objective was to review available publications and practice guidelines on management of tracheostomy and laryngectomy in the context of COVID-19. This study performed a review and synthesis of information available in the PubMed database and from national SLP organizations across 6 countries. Observations From the search, 22 publications on tracheostomy and 3 referring to laryngectomy were identified. After analysis of titles and abstracts followed by full-text review, 4 publications were identified as presenting guidelines for specific approaches to tracheostomy and were selected; all 3 publications on laryngectomy were selected. The main guidelines on tracheostomy described considerations during management (eg, cuff manipulation, suctioning, valve placement) owing to the increased risk of aerosol generation and transmission during swallowing and communication interventions in this population. Regarding laryngectomy, the guidelines focused on the care and protection of both the professional and the patient, offering recommendations on the management of adverse events and leakage of the tracheoesophageal prosthesis. Conclusions and Relevance Frequent guideline updates for SLPs are necessary to inform best practice and ensure patient and health care worker protection and safety while providing high-quality care and rehabilitation.

Verma, A., et al. (2020). "Caring for Dialysis Patients in a Time of COVID-19." Kidney Medicine **2**(6): 787-792.

Coronavirus disease 2019 (COVID-19), caused by severe acute respiratory syndrome coronavirus 2 (SARS-CoV-2), is a pandemic and a public health emergency. The overwhelming increase in the number of cases has brought significant challenges to health care systems worldwide. Patients with end-stage kidney disease are highly vulnerable with the multiple comorbid conditions that make them susceptible to adverse outcomes with COVID-19. More than 2 million people worldwide receive maintenance hemodialysis at outpatient centers. Effectively preventing the spread of infection among hemodialysis centers, health care personnel, and patients is essential to ensure the continued delivery of dialysis to patients with end-stage kidney disease. This article discusses dialysis patients' care during COVID-19, addressing measures for patient and health care personnel protection and care of dialysis patients with suspected or confirmed COVID-19. (C) 2020 The Authors. Published by Elsevier Inc. on behalf of the National Kidney Foundation, Inc.

Virani, R., et al. (2021). "Assessing the Practice Protocol of Dental Practitioners during COVID-19 Pandemic: A Questionnaire Study." Advances in Human Biology **11**(1): 113-117.

Background: The current COVID-19 pandemic has become a serious issue for the health-care professionals including dental practitioners. Materials and Methods: A total of 100 subjects were selected for the study. An online questionnaire using Google Forms was used to collect the data regarding the dentist's knowledge, perceptions and infection control in dental clinics and if they were affected by COVID-19. Data were analysed using software. Results: Out of 100 participants, 61% of them were male with a mean age of 35.21 years. More than 50% of practitioners took 10-20 min for completion of their procedure. 66% used all the personal protective equipment (PPE) with 64% of them using an N95 mask and around 90% used the PPE kit. Amongst the study group, it was found that 3% were affected by COVID-19, who were between 27 and 33 years. All of them used PPE kit with 67% using N95 mask and 33% used half respirator. 67% amongst them used high-speed handpiece and performed aerosol-producing procedures in a well-ventilated atmosphere with air conditioners. Conclusion: The findings of the study showed that the maximum number of practitioners did follow the clinical protection protocol and when all protocol was followed, the number of dental practitioners affected by COVID-19 was not statistically significant.

von Hillebrandt-Andrade, C., et al. (2021). "Co-Designing a safe ocean in the Western Tropical Atlantic within the framework of the UN Decade of Ocean Science for Sustainable Development." Ocean and Coastal Research **69**.

The frequency and intensity of ocean related hazards and its overlapping and cascading effects are escalating with devastating and unsustainable impact on life and livelihoods in the Western Tropical Atlantic. From the Bahamas to Brazil, from Mexico and Central America to the Lesser Antilles, coastal and maritime communities have been impacted by hurricanes, storm surges, earthquakes, sargassum and oil spills along with other climate, weather and human induced events. In addition to the coastal impacts, ocean hazards also have affected the safe and timely delivery of goods and services in a region that is extremely dependent on maritime transport. COVID-19 has compounded even more the situation of millions of people in the region and put extreme pressure on government officials, including disaster management and health professionals as well as the maritime industry. Climate change will only exacerbate the situation. The UN Decade of Ocean Science for Sustainable Development (2021-2030) presents itself as a once-in-a-lifetime opportunity to stop the cycle of disasters with "A Safe Ocean" as one of its seven major societal outcomes. The Western Tropical Atlantic Safe Ocean Working Group was established to co-design and co-deliver with local, national and regional partners transformative strategies that will facilitate and inspire actions to reduce and mitigate the ocean related threats while creating resilient and safer coastal communities and maritime activities. At the core is an Integrated Multi Hazard Ocean Data and Forecast System. Its services would be linked to education, outreach, readiness and communication that empowers and recognizes national and local policy decisions and includes individual response for the protection of life and livelihoods. This effort should support relevant science and strengthen capacity leaving no one or country behind.

Walcher, S. (2020). "Corona Crisis and Addiction Medicine: A Field Report." Sucht-Zeitschrift Fur Wissenschaft Und Praxis **66**(5): 286-290.

Initial situation: The threat imposed by Covid-19 has a significant impact on the world - especially that of substance-users. Drugs suddenly became poorly available, the access to substitution-therapy, addiction-help and addiction-medicine underwent restrictions. Close living-conditions, lockdown and poor health-conditions of substance-users led to serious concerns within patients, counselors and doctors. Measures: After briefly hesitating decisive action followed and self- and third party protection was installed - where scarce material-supply allowed it. Professional institutions improved political and medical framework, infection protection, take-home-prescriptions, test-capacities and virtual consultations as well as team conferences were expanded - and so far bigger infection-clusters could be avoided in this group of patients. Conclusions: Some of the current changes will remain - and will have an ongoing impact on our living together. We gained a lot of experience in crisis-management - and we improved the trustful relation to our patients. Good addiction-work is relationship-work: this crisis has just confirmed that!

Wang, C., et al. (2021). "Emergency Management in a Dental Clinic During the Coronavirus Disease 2019 (COVID-19) Epidemic in Beijing." International Dental Journal **71**(1): 32-39.

Background: In mid-March 2020, the World Health Organization declared that COVID-19 was to be characterised as a pandemic. The purpose of this article is to recommend emergency management procedures for dental clinics during this public health emergency. Materials and Methods: We have implemented a series of emergency management measures to prevent cross-infection in our dental clinic during the COVID-19 pandemic, including personnel scheduling, division of the clinic into functional areas, limitation or delay of non-emergency patients, staff protection and infection controls, clinical environmental disinfection, and the use of online consultation services, among others. Results: Due to public health policy and dental emergency management, the number of dental visitors to our clinic dropped sharply, and no COVID-19 suspected cases or high-risk patients received treatment. There have been no reports of infection of dental staff or patients during dental treatment in China to date. Conclusion: These public health policies and dental emergency management measures were effective in controlling cross-infection of COVID-19 in the dental clinic. Practical Implications: We share control measures for COVID-19, and hope that they will be helpful for dental professionals worldwide to continue to provide dental care in a safe and orderly manner. (C) 2021 The Authors. Published by Elsevier Inc on behalf of FDI World Dental Federation.

Wang, L. X., et al. (2021). "The challenge and management of nosocomial infection control during the "post-outbreak" period of SARS-CoV-2." Chinese Science Bulletin-Chinese **66**(4-5): 439-452.

The novel coronavirus SARS-CoV-2 is causing a pandemic worldwide. Several months after the initial outbreak, with the help of tight isolation policy, SARS-CoV-2 transmission in the general population is now under control in China. The number of new cases dramatically decreases compared to the outbreak period. Thus, we name this phase the "post-outbreak" period when China has lived through the pandemic phase while many other countries are still facing fast growing cases. To keep the number of new cases at low level under the consistent pressure of imported cases from other countries and prevent a second outbreak in China are tremendous challenges to all workers in the health care system. Currently available studies of the basic reproduction number (R-0) of SARS-CoV-2 demonstrate that the new virus has a higher transmission capacity than other coronaviruses such as SARS-CoV and MERS-CoV. Besides, longitudinal serological studies illuminate that protective antibodies to SARS-CoV-2 may turn negative at early convalescent phase in certain patients, indicating a relatively short protection time. Recent prediction models of the transmission dynamics suggested a risk of recurrent outbreaks of SARS-CoV-2, even in seasonal or perennial pattern. When autumn and winter are approaching, the number of patients infected with SARS-CoV-2 will be likely to increase if appropriate measures are not taken, which will further challenge the medical system. It has been shown that during the outbreak period, the infection rate of SARS-CoV-2 among medical workers is relatively high. According to the serum specific antibody test, about half of the front-line medical staffs have been infected in some countries. Some cluster cases of SARS-CoV-2 infection in hospital settings have been reported. This indicates that the risk of virus transmission in the hospital environment is high and more intensive infection control is needed. In this article, we particularly focus on several high-risk areas, such as respiratory clinics, eye clinics and obstetrical clinics. We summarize their unique clinical practices and possible risks of virus transmission, and propose some suggestions for the protection of health care workers in daily work during the "postoutbreak" period based on guidelines proposed by international groups and specialists of the fields. The review also discusses some approaches that hospitals can take during the "post-outbreak" period to early detect infected patients and cut nosocomial transmission. We focus on the topics including the prevention of the virus spreading via aerosol between patients and doctors, innovation of fast and reliable diagnostic methods to effectively identify SARS-CoV-2 carriers from large number of suspected patients, implementation of effective remote doctor consultation and establishment of a patient stratification system to reduce cross-infection, etc. This article aims to highlight the awareness of SARS-CoV-2 virus transmission in hospitals and clinics during the "post-outbreak" phase and propose crucial aspects for the research of nosocomial infection control.

Wang, S. X., et al. (2020). "A New System for Surveillance and Digital Contact Tracing for COVID-19: Spatiotemporal Reporting Over Network and GPS." Jmir Mhealth and Uhealth **8**(6).

The current pandemic of the coronavirus disease (COVID-19) has highlighted the importance of rapid control of the transmission of infectious diseases. This is particularly important for COVID-19, where many individuals are asymptomatic or have only mild symptoms but can still spread the disease. Current systems for controlling transmission rely on patients to report their symptoms to medical professionals and be able to recall and trace all their contacts from the previous few days. This is unrealistic in the modern world. However, existing smartphone-based GPS and social media technology may provide a suitable alternative. We, therefore, developed a mini-program within the app WeChat. This analyzes data from all users and traces close contacts of all patients. This permits early tracing and quarantine of potential sources of infection. Data from the mini-program can also be merged with other data to predict epidemic trends, calculate individual and population risks, and provide recommendations for individual and population protection action. It may also improve our understanding of how the disease spreads. However, there are a number of unresolved questions about the use of smartphone data for health surveillance, including how to protect individual privacy and provide safeguards against data breaches.

Wang, Y. C., et al. (2021). "A Semi-Quantitative Risk Assessment and Management Strategies on COVID-19 Infection to Outpatient Health Care Workers in the Post-Pandemic Period." Risk Management and Healthcare Policy **14**: 815-825.

Background: In the pandemic of COVID-19, due to asymptomatic patients and high personnel fluidity in outpatient clinics, health care workers (HCWs) in outpatients were facing severe threat from infection. There is an urgent need for a risk assessment to recognize and prevent infection risks. Purpose: To establish a semi-quantitative risk assessment model on COVID-19 infections for HCWs in outpatient departments, and apply it to practices. Further to provide infection risk management strategies to reduce infection threats in the post-pandemic of COVID-19. Methods: We used the method of Brainstorm, Literature study and Analytic Hierarchy Process (AHP) for risk factors selection and model construction, we also created corresponding indicators for each risk factors, in order to collect data in assessment practice. Results: Eighteen risk factors were recognized and selected for model construction, by scatter plot, these risk factors had been classified into four parts, spanned the scopes of diagnosis and treatment, environment, personal protection and emergency handling, with specific management suggestions provided. In the practice, outpatient clinics were divided into three risk levels, 5 clinics in high risk level, 9 in medium risk level and 11 in low risk level. Conclusion: A proper comprehensive risk assessment model for COVID-19 infections has been successfully established. With the model, the ability to COVID-19 prevention in outpatients can be easily evaluated. The strategies on disinfection, surveillance and personal protection were also valuable references in the post-pandemic of COVID-19.

Wang, Z. Y., et al. (2020). "Impact of Shenfu injection on a composite of organ dysfunction development in critically ill patients with coronavirus disease 2019 (COVID-19): A structured summary of a study protocol for a randomized controlled trial." Trials **21**(1).

Objectives: This study aims to determine the protection provided by Shenfu injection (a traditional Chinese medicine) against development of organ dysfunction in critically ill patients with coronavirus disease 2019 (COVID-19). Trial design: This study is a multicenter, randomized, controlled, open-label, two-arm ratio 1:1, parallel group clinical trial. Participants: The patients, who are aged from 18 to 75 years old, with a confirmed or suspected diagnosis of severe or critical COVID-19, will be consecutively recruited in the study, according to the guideline on diagnosis and treatment of COVID-19 (the 7(th)version) issued by National Health Commission of the People's Republic of China. Exclusion criteria include pregnant and breastfeeding women, atopy or allergies to Shenfu Injection (SFI), severe underlying disease (malignant tumor with multiple metastases, uncontrolled hemopathy, cachexia, severe malnutrition, HIV), active bleeding, obstructive pneumonia caused by lung tumor, severe pulmonary interstitial fibrosis, alveolar proteinosis and allergic alveolitis, continuous use of immunosuppressive drugs in last 6 months, organ transplantation, expected death within 48 hours, the patients considered unsuitable for this study by researchers. The study is conducted in 11 ICUs of designated hospitals for COVID-19, located in 5 cities of China. Intervention and comparator: The enrolled patients will randomly receive 100 ml SFI (study group) or identical volume of saline (control group) twice a day for seven consecutive days. Patients in the both groups will be given usual care and the necessary supportive therapies as recommended by the latest edition of the management guidelines for COVID-19 (the 7(th)version so far). Main outcomes: The primary endpoint is a composite of newly developed or exacerbated organ dysfunction. This is defined as an increase in the sequential organ failure assessment (SOFA) score of two or more, indicating sepsis and involvement of at least one organ. The SOFA score will be measured for the 14 days after enrolment from the baseline (the score at randomization). The secondary endpoints are shown below: SOFA score in total Pneumonia severity index score Dosage of vasoactive drugs Ventilation free days within 28 days Length of stay in intensive care unit Total hospital costs to treat the patient 28-day mortality The incidence of adverse drug events related to SFI Randomisation: The block randomization codes were generated by SAS V.9.1 for allocation of participants in this study. The ratio of random distribution is 1:1. The sealed envelope method is used for allocation concealment. Blinding (masking): The patients and statistical personnel analyzing study data are both blinded. The blinding of group assignment is not adopted for the medical staff. Numbers to be randomised (sample size): This study is expected to recruit 300 patients with COVID-19, (150 in each group). Trial Status: Protocol version 2.0, February 15, 2020. Patient recruitment started on February 25, and will end on August 31, 2020. Trial registration: Chinese Clinical Trial Registry: ChiCTR2000030043. Registered February 21, 2020, http://www.chictr.org. cn/showprojen.aspx?proj=49866 Full protocol: The full protocol is attached as an additional file, accessible from theTrialswebsite (Additional file1). In the interest in expediting dissemination of this material, the familiar formatting has been eliminated; this letter serves as a summary of the key elements of the full protocol.

Watterson, A. (2020). "COVID-19 in the UK and Occupational Health and Safety: Predictable not Inevitable Failures by Government, and Trade Union and Nongovernmental Organization Responses." New Solutions-a Journal of Environmental and Occupational Health Policy **30**(2): 86-94.

This commentary examines the occupational health and safety issues faced by the UK workers in the COVID-19 pandemic, against the background of government cuts in health care and in occupational health and safety budgets, and a deregulatory climate. The UK government has been obsessed, blinkered, and distracted by the desire to leave the European Union (Brexit). The state of knowledge about the virus, especially from international agencies that identified pandemic threats and strategies to combat it, is outlined. UK politicians, government bodies, medical and scientific advisors, and employers periodically ignored or abused that knowledge. Regulatory and ministerial inaction and errors on the workplace virus risks emerged. In contrast, several trade unions, health professional bodies, and nongovernmental organizations identified COVID-19 threats from poor personal protection equipment, working practices, and knowledge gaps and offered solutions for health care workers, social care workers, production workers, and service workers in "essential" occupations.

Welfare-Wilson, A., et al. (2021). "COVID-19 and how the wearing of face coverings can affect those with an experience of trauma." Journal of Psychiatric and Mental Health Nursing **28**(5): 777-782.

Accessible summary What is known on the subject? With the ongoing and possible evolving use of face coverings as a public health protection measure against the transmission of COVID-19, this is likely to be an ongoing challenge for those who find their use challenging. The wearing of face coverings following trauma is likely to be of ongoing relevance, making this an area that would benefit from further research. What this paper adds to existing knowledge? The authors present their personal and professional experiences as a means of highlighting the difficulties that can be faced as a result of the use of face coverings. The window of tolerance helps to understand the difficulties that can be caused by wearing face coverings and provides a visual means of conceptualizing the cognitive, behavioural, physiological and emotional reactions that can occur as a result of their use. What are the implications for practice? This paper provides an awareness of the link between trauma and the wearing of face coverings, and how their use could be re-traumatizing for those accessing services. This topic is relevant across all sectors where it is only just beginning to be acknowledged that for many, particularly those with experiences of interpersonal trauma, difficulties can arise due to the use of face coverings. The sharing of grounding techniques and an introduction to the window of tolerance provides a means of collaboratively developing skills and developing a shared understanding of the difficulties associated with the use of face coverings.

Wichterich, C. (2021). "Protection and Protest by "Voluntary" Community Health Workers: COVID-19 Authoritarianism in India." Historical Social Research-Historische Sozialforschung **46**(4): 163-188.

During the COVID-19 crisis, the Indian state assigned an outstanding role in the containment of the pandemic to ASHAs, "voluntary" community healthcare workers. Local women were recruited as "honorary" workers receiving only a small honorarium or piece rate "incentives." The feminisation, casualisation, and informalisation of work in state-funded social welfare schemes are examples of care extractivism. ASHAs had to spearhead awareness-raising, identify infections, and organise vaccinations in rural areas, often without proper protective equipment and always without fair payment. Despite such care extractivism, these caregivers were celebrated by the middle classes as frontline fighters of the nation amidst a masculinist discourse of "war," "warriors," "heroes," and "sacrifice." Yet, at the height of the crisis in 2020 when thousands of ASHAs contracted COVID-19, they went on strike. Having gained confidence in earlier struggles, they complained about extreme exhaustion, increased vulnerability, and the depletion of caring capacities. Their efforts reflect a feminisation of labour struggles that focuses on care work with an emphasis on both the care-recognition-gap and the care-pay-gap.

Wise, P. H., et al. (2021). "The political and security dimensions of the humanitarian health response to violent conflict." Lancet **397**(10273): 511-521.

The nature of armed conflict throughout the world is intensely dynamic. Consequently, the protection of noncombatants and the provision of humanitarian services must continually adapt to this changing conflict environment. Complex political affiliations, the systematic use of explosive weapons and sexual violence, and the use of new communication technology, including social media, have created new challenges for humanitarian actors in negotiating access to affected populations and security for their own personnel. The nature of combatants has also evolved as armed, non-state actors might have varying motivations, use different forms of violence, and engage in a variety of criminal activities to generate requisite funds. New health threats, such as the COVID-19 pandemic, and new capabilities, such as modern trauma care, have also created new challenges and opportunities for humanitarian health provision. In response, humanitarian policies and practices must develop negotiation and safety capabilities, informed by political and security realities on the ground, and guidance from affected communities. More fundamentally, humanitarian policies will need to confront a changing geopolitical environment, in which traditional humanitarian norms and protections might encounter wavering support in the years to come.

Wong, P. W., et al. (2021). "The Resilience of Social Service Providers and Families of Children With Autism or Development Delays During the COVID-19 Pandemic-A Community Case Study in Hong Kong." Frontiers in Psychiatry **11**.

Background: Hong Kong is one of the earliest cities to have hampered by the COVID-19. When preventive public health measures are enforced, specific groups, who have already been facing inequality before the outbreak, are likely to become more overlooked and vulnerable. Aim: This community case study aims to describe the additional needs of families of children with autism spectrum disorder and other developmental issues, as well as unexpected difficulties and challenges social service professionals encountered when delivering service and their solutions toward these challenges. Methods: A focus group with 10 professionals providing the Caregiver Skills Training Program was conducted. Results: Poor families of vulnerable children were found to be challenged, more than average, in finding daily necessities during the initial stage of the outbreak. Most vulnerable children displayed additional problematic behaviors and emotional problems during the quarantine. The social service professionals addressed the family needs by providing tangible resources and offering online training, workshops, and programs to meet their needs. Several important lessons were learned. First, technology know-how on conducting online training, workshop, and program could be a challenge to some social service professionals and the parents. Second, the professionals reported that they made huge efforts to produce guidelines in protecting services users' privacy, to equip themselves with necessary skills in executing privacy-protection measures, and to keep exploring for safer alternatives. Third, providing tele-services in online mode represented a different interaction pattern between social service professionals and service users, especially in the recruitment processes and group dynamics. Conclusion: In comparison with other cities, Hong Kong has responded to the COVID-19 efficiently and effectively based on the citizen's strict adherence to behavioral advice and the innovative altruistic efforts from the multi-sectors in the community.

Wu, X. Y., et al. (2020). "Contingency Nursing Management in Designated Hospitals During COVID-19 Outbreak." Annals of Global Health **86**(1).

Background: In December 2019, early cases of COVID-19 were identified in Wuhan, China. By late January 2020, it was evident that COVID-19 was rapidly spreading and represented a national health emergency. In order to contain the spread of COVID-19, China adopted a centralized treatment plan by appointing designated hospitals in each region. Shantou Central Hospital is a Grade A Class A general hospital in Guangdong Province. It was appointed as a provincial COVID-19 designated treatment hospital on January 21, 2020, to provide all COVID-19-related treatments for the city of Shantou. The nursing department at Shantou Central Hospital is fully responsible for hospital nursing administration, nursing human resource management, nursing quality management, and all nursing tasks related to hospital medical care, nursing, teaching, scientific research, preventive healthcare, and so on. Objective: To summarize the role of nursing management in transforming a general hospital into a-designated hospital for treatment of COVID-19 patients. Methods: We undertook a series of nursing management measures in the strategic phase and the-implementation phase. Findings: Through a series of nursing management measures, all COVID-19 patients admitted to our-hospital were cured and discharged. All non-COVID-19 patients and staff hospitalized during the same period were not infected with the virus. During this period, our hospital completed 7,466 operations. Hence, our nursing management measures were effective. Conclusions: Our efficient nursing management system, first of all, effectively mobilized all available manpower; secondly, up-skilled and trained personnel within a very short period of time; thirdly, provided reliable logistical support for front-line protection equipments; and finally, motivated nurses during this very difficult time to make a significant positive contribution to the fight against COVID-19 pandemic.

Yang, H. S., et al. (2020). "Routine Laboratory Blood Tests Predict SARS-CoV-2 Infection Using Machine Learning." Clinical Chemistry **66**(11): 1396-1404.

BACKGROUND: Accurate diagnostic strategies to identify SARS-CoV-2 positive individuals rapidly for management of patient care and protection of health care personnel are urgently needed. The predominant diagnostic test is viral RNA detection by RT-PCR from nasopharyngeal swabs specimens, however the results are not promptly obtainable in all patient care locations. Routine laboratory testing, in contrast, is readily available with a turn-around time (TAT) usually within 1-2 hours. METHOD: We developed a machine learning model incorporating patient demographic features (age, sex, race) with 27 routine laboratory tests to predict an individual's SARS-CoV-2 infection status. Laboratory testing results obtained within 2 days before the release of SARS-CoV-2 RT-PCR result were used to train a gradient boosting decision tree (GBDT) model from 3,356 SARS-CoV-2 RT-PCR tested patients (1,402 positive and 1,954 negative) evaluated at a metropolitan hospital. RESULTS: The model achieved an area under the receiver operating characteristic curve (AUC) of 0.854 (95% CI: 0.829-0.878). Application of this model to an independent patient dataset from a separate hospital resulted in a comparable AUC (0.838), validating the generalization of its use. Moreover, our model predicted initial SARS-CoV-2 RT-PCR positivity in 66% individuals whose RT-PCR result changed from negative to positive within 2 days. CONCLUSION: This model employing routine laboratory test results offers opportunities for early and rapid identification of high-risk SARS-CoV-2 infected patients before their RT-PCR results are available. It may play an important role in assisting the identification of SARS-CoV-2 infected patients in areas where RT-PCR testing is not accessible due to financial or supply constraints.

Yapici, G., et al. (2021). "Assessment of COVID-19 Risky Contact of Healthcare Workers in an University Hospital." Mikrobiyoloji Bulteni **55**(2): 161-179.

Healthcare workers are the group with the highest risk of COVID-19 transmission. The illness of healthcare workers poses a risk to patients admitted to the hospital, colleagues and households besides their own health. In this study, it was aimed to determine the risk assessment and the factors associated with risk status of an university hospital healthcare workers after risky COVID-19 contact. The data of the descriptive study were obtained from 773 follow-up records of 555 healthcare workers who applied with COVID-19 risky contact between 06.04-10.05.2020. Employees who were positive for RT-PCR evaluated as "patients" and others as "risky contact". Risk assessment was classified as no risk, low, medium and high risk contact according to the "Algorithm of Assessment of Health Workers with COVID-19 Contact" of the Ministry of Health. The relationship between the risk levels of the participants and their demographic and workplace characteristics and their usage of personal protection were evaluated. Mean, standard deviation, percentage, chi-square and ANOVA tests were used in the analysis of the data. The average age of the healthcare workers was determined as 34.4 +/- 7.6 years. It was determined that 56.2% of those who had contact were female, 62.9% were married and 17.5% had an additional disease. It was determined that 45.6% of the risky contacts were nurses, 18.4% were supportive personnel and 16.9% were doctors. While 46.5% of the contacts were found as medium, 28.0% low, 17.1% high risk and 8.4% risk free. 38.2% of risky contacts occurred while working in internal/surgical clinics. While 66.0% of the employees had risky contact during patient care and treatment, 25% had risky contact with colleagues in social settings. High-risk contact was higher in social relations between employees. The source of the contact was a colleague in 73.2% of the employees. The average age of high-risk employees was smaller than those of low-risk. While 54.5% of the employees wore surgical masks during contact, 67.8% of the patients did not have a mask. Of 555 employees followed, 37 (6.7%) were diagnosed as COVID-19; 48.6% of the patients were nurses and 18.9% were doctors. It was determined that 48.6% of the healthcare workers were working in the COVID-19 service, outpatient clinic or intensive care unit at the time of diagnosis. The source of the infection was thought to be a colleague in 51.6% of the patients. COVID-19 was more common in nurses and doctors. It was determined that risky contact also occurred in services other than the units where COVID-19 patients were treated and risky contact often took place while providing healthcare to the patients and during social relations between the employees. Unprotected contact of the employees with each other in the workplace was identified as an important risk source. Inadequate use of personal protective equipment by healthcare workers led to an increase in medium and high risk contacts. The use of masks by patients and their relatives during the health service delivery and the proper use of personal protective equipment by healthcare professionals will reduce the risk. With the physical improvement of the rest areas of the employees and the arrangement of the breaks, the risky contact between colleagues in the social areas will be reduced.

Yasmin, R., et al. (2020). "Knowledge and practice of infection prevention and control among healthcare workers: a COVID-19 pandemic experience." Anaesthesia Pain & Intensive Care **24**(6): 596-602.

Background: Coronavirus disease (COVID-19) is a global outbreak caused by novel SARS-CoV-2 (severe acute respiratory syndrome coronavirus 2). Study aims to evaluate knowledge and practices of healthcare workers (HCW) regarding infection prevention and control of corona virus disease Material and methods: A cross sectional validation study was conducted. Study was conducted at department of Medicine, Pakistan Ordinance Factory Hospital, Wah Cantt. Study duration was 2 months (May 2020-June2020)A sample size of 217 HCW was calculated using WHO calculator. HCW were selected through non probability sampling (consecutive). Ethical permission was taken and research consent was signed by each participant. SPSS version 24 was used for analysis purpose. Post stratification fissure exact and chi-square test was applied. We found p value =0.05 as statistically significant. Results: Total 217 HCW were included in our study. Out of them, 85 (39.2%) were male and 132 (60.8%) were female in our study. Mean age of HCW was 34.5 +/- 2.4SD. In knowledge assessment majority of HCW had fair knowledge (46.5%), followed by good (30%) and poor knowledge (23.5%) regarding corona virus associated infection control and prevention. In practice assessment, among all those, 38.2% HCW showed better practices while 61.8% showed poor practices. Knowledge is significantly associated with virus transmission mode (p = 0.00), close contact (p = 0.000), protection from COVID-19 in triage area (p = 0.000), wearing PPE (p = 0.000) etc. Conclusion: Corona virus disease is a major challenge in resource limited countries. Healthcare workers showed relatively improved knowledge and practices regarding infection prevention and control in this COVID-19 Pandemic.

Yavorovsky, A. P., et al. (2020). "Problems of safety, occupational hygiene and control over infections in fighting with occupational diseases of healthcare workers with COVID-19 in treatment facilities of Ukraine." Medical Perspectives-Medicni Perspektivi **25**(4): 159-165.

The objective of the publication was to assess the safety of treatment facilities, occupational health and infection control in Kiev, Zhytomyr and Zhytomyr region to enhance risk management of SARS-CoV-2 infection of healthcare workers and reduce occupational illness and mortality for COVID-19. Bibliosemantic, hygienic, questionnaire, statistical methods and methods of comparative and system analysis have been used. The work of doctors involved in overcoming COVID-19 pandemic is classified as dangerous (extreme). In addition to the SARS-CoV-2 virus, the working conditions of medical workers are influenced by physical, chemical factors and high physical and neuro-emotional stress. Among medical workers of the Zhytomyr region who were diagnosed with an acute occupational disease COVID-19, nurses prevailed (38.57%). Junior nurses (26.1%) - the second COVID-19 incidence, paramedics (5.31%) occupy the third place. The doctors' incidence was ranked in the following sequence: doctors of GPFM - 4.85%, surgeons - 4.16%, anesthesiologists - 2.54%, infectious disease doctors - 2.08%, radiologists - 1.85%. This distribution of medical professions is observed for all Ukraine regions. Chance of becoming infected with SARS CoV 2 for healthcare workers in October was by 3.8 times higher than the general population. Risk of dying from COVID-19 in healthcare workers is greater by 1.5 times than the general population. The high level of occupational morbidity of COVID-19 in Ukrainian medical personnel is determined by personal negligence, incomplete staffing of TF with medical workers, of medical workers with PPE; dis-use of PPE if available, absence or poor-quality instruction on labor protection; shortage of epidemiologists, hygienists and occupational pathologists.

Yee, K., et al. (2021). "Stressors and coping strategies of migrant workers diagnosed with COVID-19 in Singapore: a qualitative study." BMJ Open **11**(3).

Introduction The health, psychological and socioeconomic vulnerabilities of low-wage migrant workers have been magnified in the COVID-19 pandemic, especially in high-income receiving countries such as Singapore. We aimed to understand migrant worker concerns and coping strategies during the COVID-19 pandemic to address these during the crisis and inform on comprehensive support needed after the crisis. Methods In-depth semi-structured interviews were carried out with migrant workers diagnosed with COVID-19. The participants were recruited from a COVID-19 mass quarantine facility in Singapore through a purposive sampling approach. Interviews were transcribed verbatim and thematic analysis performed to derive themes in their collective experience during the crisis. Results Three theme categories were derived from 27 interviews: migrant worker concerns during COVID-19, coping during COVID-19 and priorities after COVID-19. Major stressors in the crisis included the inability to continue providing for their families when work is disrupted, their susceptibility to infection in crowded dormitories, the shock of receiving the COVID-19 diagnosis while asymptomatic, as well as the isolating conditions of the quarantine environment. The workers coped by keeping in contact with their families, accessing healthcare, keeping updated with the news and continuing to practise their faith and religion. They looked forward to a return to normalcy after the crisis with keeping healthy and having access to healthcare as new priorities. Conclusion We identified coping strategies employed by the workers in quarantine, many of which were made possible through the considered design of care and service delivery in mass quarantine facilities in Singapore. These can be adopted in the set-up of other mass quarantine facilities around the world to support the health and mental well-being of those quarantined. Our findings highlight the importance of targeted policy intervention for migrant workers, in areas such as housing and working environments, equitable access to healthcare, and social protection during and after this crisis.

Yigzaw, T., et al. (2021). "Collaborative Engagement of Health Professional Associations in Ethiopia's COVID-19 Response: Achievements and Lessons Learned." Ethiopian Journal of Health Development **35**(4).

Introduction: Effective and speedy response to a public health crisis such as the covid-19 pandemic requires collaboration. Therefore, Ethiopian health professional associations formed a scientific advisory council in March 2020 to provide collaborative technical assistance to the Ministry of Health (MOH) in the national COVID-19 pandemic response. In this paper, we reflect on achievements and lessons learned in the collaborative partnership between health professional associations and the MOH in the COVID-19 response. Achievements and challenges: The scientific advisory council created a platform for coordinated communication between professional associations and the MOH, which provided technical assistance on preventive and control measures and established a web-based information repository to share credible and locally relevant COVID -19 resources to health workers. Professional associations also cooperatively provided COVID -19 training to health workers, engaged the media for public health education and advocacy, and called for better protection and support of health workers. However, the lack of institutional arrangement for collaboration, delay in implementation of recommendations, decrease in engagement of scientific advisory council members over time, and shortage of funding was observed as challenges in the collaborative engagement of professional associations. Conclusion and recommendation: The COVID-19 pandemic created unprecedented opportunities for a collaborative partnership between professional associations and the MOH. We believe this partnership contributed to reducing the spread of COVID-19 and saved many lives. Going forward, the partnership should be formalized to ensure effective and sustainable relationships and this partnership should be upgraded to engage professional associations in co-creating, implementing, and monitoring health policies and programs.

Yuryeva, L. M. (2020). "Mental health care of medical workers during COVID-19 pandemic." Medicni Perspektivi **25**(4): 12-21.

During the pandemic caused by the SARS-CoV-2 virus, healthcare workers are at the forefront of the battle undergoing not only significant physical but also emotional stress. At the same time medical workers are considered to be at high risk for the incidence of COVID-19. Staying in a state of constant emotional stress over time leads to the emergence of both mental and somatic disorders. The purpose of this article is to conduct a literature review on the principles of consistency regarding the mental health of staff members of medical institutions providing care to patients with COVID-19. A systematic literature search has been carried out, as a result of which 32 articles with reports of mental and behavioral disorders during the pandemic have been selected for analysis. The main group of disorders are disorders of the anxious-depressive spectrum. According to various sources, from a quarter to a third of medical workers have clinically significant anxiety, about a third - depression. Significant prevalence of sleep disorders in individuals providing medical care to patients with COVID-19 has also been indicated. In the long term, the expectation of an increase in the level ofpost-traumatic stress disorder has been indicated. The article also provides modern views on the socio-psychological effects of epidemics and pandemics. Excessive attention, especially on social media devoted to the problem of COVID-19 significantly complicates the fight against the real problem of overcoming the pandemic. It has been proven that over concentration of attention on problems associated with COVID-19 is a factor of the increased risk of developing generalized anxiety disorder. Thus, the protection of mental health and the socio-psychological support of medical workers are some of the important directions in the fight against the coronavirus pandemic. The management of psychological crises during pandemics should be based on psycho-hygienic and psycho-preventive measures both at the level of the individual and society as a whole.

Zaidi, S. T. R. and S. S. Hasan (2021). "Personal protective practices and pharmacy services delivery by community pharmacists during COVID-19 pandemic: Results from a national survey." Research in Social & Administrative Pharmacy **17**(1): 1832-1837.

Introduction: Coronavirus disease (COVID19) has adversely affected the delivery of various health services. Little is known about the impact of COVID19 on pharmacy services across the United Kingdom (UK). We surveyed community pharmacists across the UK to understand their protective practices, professional and general wellbeing, and the delivery of pharmacy services during the COVID19 pandemic. Methods: Community pharmacists were invited to participate in a cross-sectional survey via the nationwide weekly newsletter of Pharmaceutical Services Negotiating Committee and the local pharmaceutical committees during the second week of May 2020. The survey remained open for 4 weeks. Results: A total of 206 pharmacists responded to the survey with representations from England, Northern Ireland, Scotland, and Wales. The majority of pharmacists (>75% or above) reported an increase in customer traffic to their pharmacy and were asking relevant questions from patients with flu-like symptoms before signposting them to the appropriate care. Most pharmacists (>85%) were maintaining a safe distance of 2 m from customers and 72% were wearing an N95 protective mask and 28% were using protective gloves and apron in addition to safe distancing and protective masks. Ninety-nine percent of pharmacists reported drug shortages with 38% and 26% reported significant drug shortages and critical drug shortages causing disruptions beyond over the counter medicines. Eighty-nine percent pharmacists reported inappropriate behaviour from patients or carers with 31% and 16% reported it to be a regular or frequent problem, respectively. Fifty-three% of pharmacists were willing to offer their assistance for mass testing of COVID19 antibodies if adequate training and reimbursement are provided. Conclusion: Community pharmacists in the UK are facing considerable challenges in terms of personal protection and the delivery of pharmacy services. Inappropriate behaviour from patients and carers towards community pharmacists require urgent attention to ensure a safe working place for all community pharmacists.

Zangrillo, A., et al. (2020). "Fast reshaping of intensive care unit facilities in a large metropolitan hospital in Milan, Italy: facing the COVID-19 pandemic emergency." Critical Care and Resuscitation **22**(2): 91-94.

At the end of 2019, the severe acute respiratory syndrome coronavirus 2 (SARS-CoV-2) outbreak spread from China all around the world, causing thousands of deaths. In Italy, the hardest hit region was Lombardy, with the first reported case on 20 February 2020. San Raffaele Scientific Institute - a large tertiary hospital and research centre in Milan, Italy - was immediately involved in the management of the public health emergency. Since the beginning of the outbreak, the elective surgical activity of the hospital was rapidly reduced and large areas of the hospital were simultaneously reorganised to admit and assist patients with coronavirus disease 2019 (COVID-19). In addition, the hospital became the regional referral hub for cardiovascular emergencies in order to keep ensuring a high level of health care to non-COVID-19 patients in northern Italy. In a few days, a COVID-19 emergency department was created, improving the general ward capacity to a total number of 279 beds dedicated to patients with COVID-19. Moreover, the number of intensive care unit (ICU) beds was increased from 28 to 72 (54 of them dedicated to patients with COVID-19, and 18 to cardiology and cardiac surgery hub emergencies), both converting pre-existing areas and creating new high technology spaces. All the involved health care personnel were rapidly trained to use personal protection equipment and to manage this particular category of patients both in general wards and ICUs. Furthermore, besides clinical activities, continuously important research projects were carried out in order to find new strategies and more effective therapies to better face an unprecedented health emergency in Italy.

Zengin, H., et al. (2020). "Structural Properties and Performance Test Standards of Surgical Masks with Respirators Used in Healthcare Settings." Flora Infeksiyon Hastaliklari Ve Klinik Mikrobiyoloji Dergisi **25**(3): 292-300.

Healthcare workers may be exposed to various aerosols formed during patient care and treatment. Transmission of bacterial or viral infections to the healthcare staff by this route can be life threatening. Worldwide, many national and international occupational health and safety organizations consider medical masks and respirators as an important element of protecting healthcare workers against exposure to hazardous aerosol particles. With the recent COVID-19 pandemic, the features and standards of masks that should be used in different settings have become an important topic of discussion. The masks produced in compliance with standards are widely used against harmful aerosols and protect the user when properly installed. These medical and respiratory masks are among the important personal protection equipment widely used in healthcare to prevent transmission of bacterial and viral infections as well as COVID-19. Here, it was aimed to review the structural features and performance test standards of the masks used during medical procedures.

Zhang, L., et al. (2021). "Burnout in nurses during the COVID-19 pandemic in China: New challenges for public health." Bioscience Trends **15**(2): 129-131.

During the COVID-19 pandemic, frontline nurses have faced extraordinary personal and professional challenges. These challenges have had mental health consequences, and concerning reports of burnout have emerged globally. We conducted a cross-sectional survey at a designated COVID-19 hospital in Shanghai at the peak of the pandemic, i.e. about 2 months after the onset of the outbreak from February to April 2020. Findings revealed burnout in 6.85% of nurses. Of 336 respondents, 87 (25.89%) had a high level of emotional exhaustion, 61 (18.15%) had a high level of depersonalization, and 100 (29.76%) had a low level of personal accomplishment. Burnout can be prevented by offering more support from families and supervisors, paying attention to health monitoring and personal protection, and creating a rational human resource allocation and shift management system. Specific training on infection control and self-protection, mental health guidance, and stress coping techniques must be implemented. As the current health crisis ultimately abates, moving the focus from mental health issues to public health issues, more attention and support at the national and organizational levels are needed to reduce occupational discrimination, nurse autonomy and status need to be promoted, and public health emergency teams need to be created. A positive and fair working environment is essential to effective healthcare delivery.

Zhao, Y. J., et al. (2020). "Radiology department strategies to protect radiologic technologists against COVID19: Experience from Wuhan." European Journal of Radiology **127**.

The outbreak of Coronavirus Disease 2019 (COVID-19) is a huge threat to global public health security. In the absence of specific antiviral medicines to prevent or treat COVID-19, it is essential to detect the infected patients at an early stage and immediately isolate them from the healthy population. In view of the advantages of sensitivity and high spatial resolution, CT imaging has played an important role in screening and diagnosing of COVID-19 in China. The radiologic technologists performing CT scans for the infected patients become high-risk medical care personnel. It is critical for the radiology department to ensure the personal safety of radiologic technologists and avoid cross-infection. In this review article, we describe the systematic strategies to combat COVID-19 from the radiology department in Tongji hospital in Wuhan, P.R. China, including personnel arrangements, environmental modification, protection levels and configurations, radiological imaging (CT and radiography), and disinfection methods. It can provide guidance to other radiology departments faced with COVID-19 to reduce infection risk for radiologic technologists.
